# Construction and validation of a three-microRNA signature as prognostic biomarker in patients with hepatocellular carcinoma

**DOI:** 10.7150/ijms.49126

**Published:** 2021-01-01

**Authors:** Xi Zhang, Li Ma, Li Zhai, Dong Chen, Yong Li, Zhongjun Shang, Zongmei Zhang, Yanzhang Gao, Wei Yang, Yixun Li, Yuqing Pan

**Affiliations:** 1 Department of Clinical Laboratory, Yunnan Cancer Hospital, The Third Affiliated Hospital of Kunming Medical University, Kunming, Yunnan, P.R. China; 2 Department of Ultrasound, Yunnan Cancer Hospital, The Third Affiliated Hospital of Kunming Medical University, Kunming, Yunnan, P.R. China; 3 Department of Abdominal Surgery, Yunnan Cancer Hospital, The Third Affiliated Hospital of Kunming Medical University, Kunming, Yunnan, P.R. China; 4 Department of Hospital Affairs, Yunnan Cancer Hospital, The Third Affiliated Hospital of Kunming Medical University, Kunming, Yunnan, P.R. China; 5 Department of Pathology, Yunnan Cancer Hospital, The Third Affiliated Hospital of Kunming Medical University, Kunming, Yunnan, P.R. China; 6 Department of Clinical Laboratory, The First Affiliated Hospital of Kunming Medical University, Yunnan Institute of Experimental Diagnosis, Yunnan Key Laboratory of Laboratory Medicine, Kunming, Yunnan, P.R. China

**Keywords:** hepatocellular carcinoma, TCGA, miRNA, prognosis

## Abstract

Hepatocellular carcinoma (HCC), a common type of primary liver cancer, is one of the most aggressive malignant tumors worldwide. Although overall survival (OS) rates for HCC has significantly improved in recent years, however, the exact predictive value of microRNA (miRNA) for the prognosis of HCC has not yet been recognized. Here, we aimed to identify potential prognostic miRNAs involved in HCC by bioinformatics analysis and validated expression levels through quantitative polymerase chain reaction (qPCR) and GEO database. The RNA expression profiles and corresponding clinical information of HCC were available from The Cancer Genome Atlas (TCGA) datasets. Differentially expression and standardization analysis of miRNAs, Kaplan-Meier curve and time dependent ROC curve were performed by using R tools. Differentially expressed miRNAs (DEmiRNAs) and clinical parameters involved in the OS of HCC were confirmed by Cox regression models. And functional enrichment analysis was used to establish functions of the targeted genes of DEmiRNAs. A total of 300 DEmiRNAs were significantly related with HCC, of which 40 were down-regulated and 260 were up-regulated. A total of 344 patients with DEmiRNAs, status, overall survival (OS) time were randomized into training group (172) and test group (172). Multivariate Cox regression analyses revealed that 3 miRNA (hsa-miR-139-3p, hsa-miR-760, hsa-miR-7-5p) had independent prognostic significance for the OS of HCC in both training and test group. Moreover, according to Kaplan Meier analysis, the OS of HCC patients with high-risk score was shorter in validation and entire series. The time dependent ROC curve demonstrated high accuracy of the signature for OS. Besides, target genes of three miRNAs were analyzed by functional enrichment analysis and 20 genes associated with OS were verified by using Kaplan-Meier method. Compared with normal and benign group, the relative expression level of hsa-miR-139-3p was significantly decreased, while hsa-miR-7-5p and hsa-miR-760 were distinctly increased in the plasma of HCC patients. The same results were observed in the independent cohort. Collectively, our research suggested that three-miRNA signature could serve as an independent prognostic indicator for HCC patients.

## Introduction

Hepatocellular carcinoma (HCC) is the most prevalent histological type of liver cancer, ranking the third cause of cancer-related death 1, 2. With the characteristics of strong invasion and high mortality, it is still the fifth leading cause of death in men and seventh in female in the United States 3. As a result of remarkable progress in therapy along with improved supportive care, the long-term survival rate of HCC has been greatly improved in some western countries 4. However, due to a high prevalence of hepatitis B virus infection and lifestyle factors, together with lacking awareness about cancer early detection, the mortality rate is rising and the treatment effect is still not optimistic for some developing countries 5. On the other hand, liver transplantation is considered as the most effective method for the treatment of liver tumors and potential liver diseases in recent years. Nevertheless, limited by surgical indications and shortage of living donors, liver transplantation has limited contribution to reduce the overall mortality of liver cancer 6. Fortunately, sorafenib is regarded as a targeted chemotherapy drug for patients with advanced liver cancer, but not all patients can bear the cost, and its long-term drug effect is still limited 7. Therefore, it is vital to discover biomarkers for the early diagnosis and drug therapy target for HCC.

At present, it is generally believed that miRNA is an extremely conservative noncoding RNA of ~20 nucleotides in eukaryotes 8. A miRNA-induced silencing complex is formed by combining with mRNA, which results transcription inhibition and targeted mRNA degradation 9, 10. Several lines of evidence have shown that miRNAs are closely related to cell differentiation 11, apoptosis 12, proliferation 13, invasion 14, and migration 15 of HCC cells. Moreover, as a novel prognosis marker, some miRNAs are involved in the development of HCC 16, 17. On the other hand, several studies have proposed prognostic models on some solid tumors such as bladder cancer 18, uveal melanomas 19, and glioblastoma 20, which depend on molecular profiles and bioinformatics analysis. Thus, it can be seen that researchers have high expectations for miRNAs as prospective biomarkers in the diagnosis, prognosis and targeted therapy of different tumors. And, it is worth mentioning that although the clinical prediction value of miRNA has been well known, the consistency of the results is not satisfactory.

The etiology of HCC remains unclear so far. It is important to understand the molecular mechanisms underlying the condition of miRNA involved in tumor stages and mutational loads. Therefore, in this academic work, we downloaded RNA expression profiles of HCC patients based on the TCGA database and identified the DEmiRNAs, then we furtherly calculated a risk score by using the prognosis-associated DEmiRNAs to predict the clinical outcome of patients with HCC. Notably, meaningful target genes of miRNAs involved in gene ontology (GO) functional annotation analysis and Kyoto Encyclopedia of Gene and Genome (KEGG) pathway enrichment analysis were visualized by using R software packages, then PPI network in HCC were constructed by using bioinformatics analysis, which could contribute to develop more effective treatments. Besides, the expression levels of three miRNAs were detected so as to validate the signature in vitro. Compared with previous studies that preferred the single miRNA strategy, we performed a novel predictor by integrating multiple miRNAs and clinical data, which was expected to provide more effective information for miRNA to participate in the occurrence and development of HCC. Furthermore, the novel miRNA signature showed an important clinical practical value in survival prediction of HCC patients, indicating that it could be used as a potential therapeutic target and even guide the clinical treatment of HCC.

## Materials and methods

### Patient's information and database

The mRNA expression profile [Case (371): Primary Site (Liver and intrahepatic bile ducts), Program (TCGA), Project (TCGA-LIHC), Disease Type (Adenomas and adenocarcinomas); Files (530): Data Category (Transcriptome profiling), Workflow Type(HTSeq - Counts), Data Type (Gene Expression Quantification)], miRNA expression information [Case (373): Primary Site (liver and intrahepatic bile ducts), Program (TCGA),Project (TCGA-LIHC), Disease Type (Adenomas and adenocarcinomas); Files(425): Data Category (Transcriptome profiling), Data Type (Isoform Expression Quantification), Workflow Type(BCGSC miRNA Profiling)]and clinicopathological data[Case (377): Primary Site (liver and intrahepatic bile ducts); Files(423): Data Category: Clinical, Data Format: BCR XML] (Table 1) were obtained from TCGA (https://portal.gdc.cancer.gov/) database. And, the mRNA expression profile included 374 tumor samples and 50 non-tumor controls while miRNA expression profile contained 375 tumor samples and 50 normal controls. Subsequently, we downloaded all mature miRNA sequences (Fasta format, mature.fa) from the miRbase (http://www.mirbase.org/), then expression profile for each mature miRNA of HCC were obtained by Perl language through combining two sets of data. Independent validation cohort was obtained from GEO (http://www.ncbi.nlm.nih.gov/geo) database. The key words of 'hepatocellular carcinoma' and 'miRNA' were performed to screen out relevant dataset. And then, GSE63046 based on GPL11154 platform was selected. The gene expression profile includes 24 HCC patients and 24 unaffected tissues adjacent to tumors. Besides, we selected frozen plasma from 31 patients who undergone the hepatectomy and were initially diagnosed with HCC based on pathological biopsy at the Department of Abdominal Surgery of Yunnan Cancer Hospital from January 2020 to May 2020 as the HCC group for validation. No chemoradiotherapy or immunotherapy was received before the operation, and no other malignant tumor was observed. On the other hand, a total of 40 healthy physical examination patients and 18 patients with cirrhosis were enrolled as the normal group and benign group respectively. This study was approved by the ethics committee of Yunnan Cancer Hospital, and the enrolled patients have completed the informed consent.

### Differentially expressed mRNAs (DEmRNAs), DEmiRNAs and combine them with survival data

**Figure 1:**
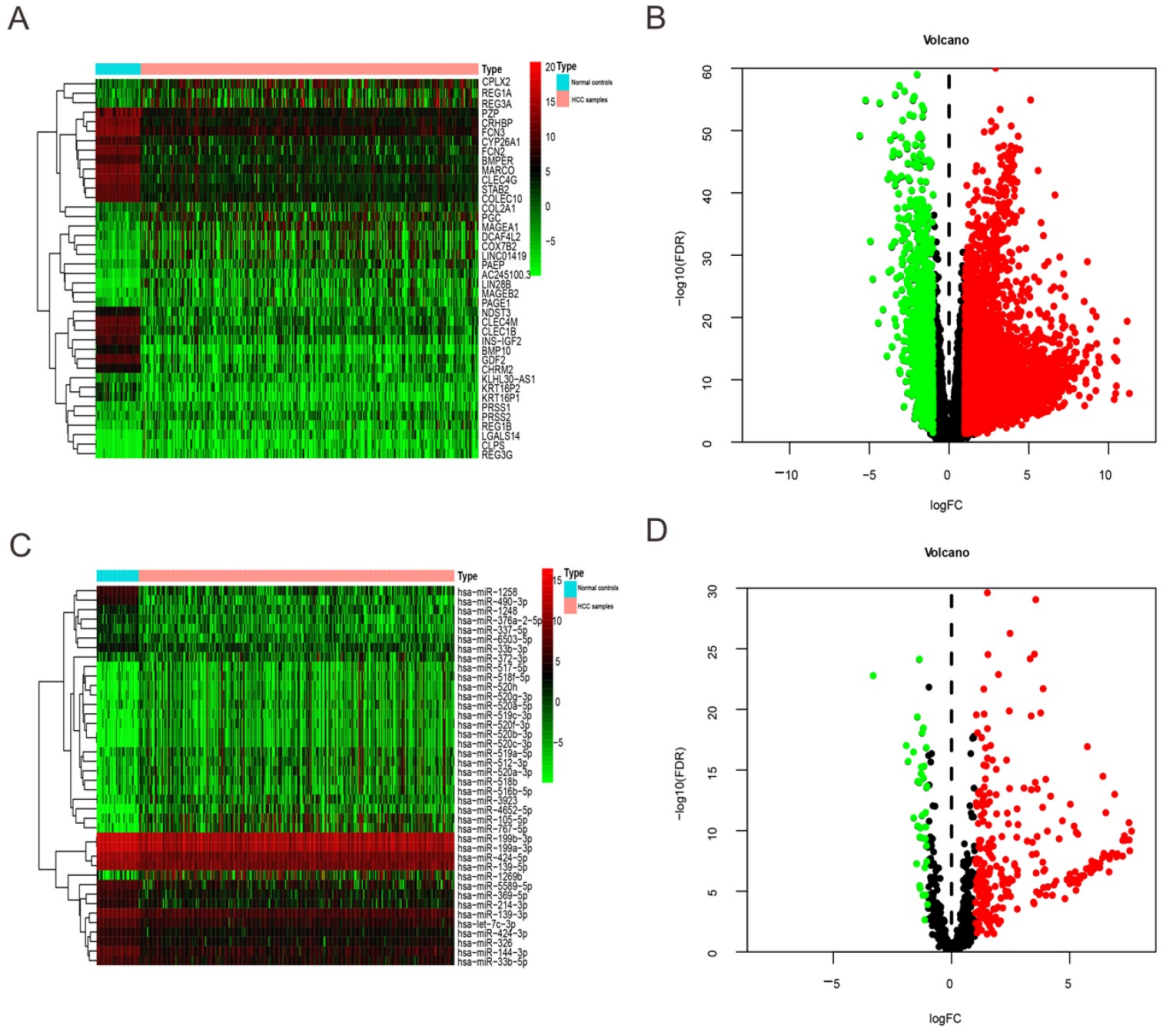
** Heatmap and volcano plots of DEmRNAs and DEmiRNAs between HCC patients and normal controls.** (A) and (C) Heatmap of top 40 DEmRNAs and DEmiRNAs, mRNA and miRNA that up-regulated are in red. mRNA and miRNA that down-regulated are in green. mRNA and miRNA that without any significant difference are in black. (B) and (D) Volcano plots of DEmRNAs and DEmiRNAs, Red dot represents the up-regulated mRNA and miRNA, green dot represents the down-regulated mRNA and miRNA, and black dot is the mRNA or miRNA without significant difference. The differences are set as FDR <0.05 and |log_2_FC| > 1.

DEmRNAs, DEmiRNAs between HCC patients and normal controls were detected and normalized via edgeR package in R tools on the basis of false discovery rate (FDR) < 0.05 and |log_2_ fold change (FC)| > 1. For the difference results, ggplot package was used to draw volcanic maps, the expression data of all the DEmRNAs, DEmiRNAs with larger fold change were mapped by pheatmap package (Figure 1). We filtered out the unqualified cases (29) with survival time less than 30 days and then combined remaining cases (348) with expression datasets of DEmRNAs, DEmiRNAs respectively. Because clinicopathological data actually included other multi-group data, such as single nucleotide polymorphism (SNP), copy number variants (CNV), the final qualified cases were 344. Besides, the right censoring percentage was 64.8% (223/344).

### Grouping of samples, construction and validation of prognostic model

In this study, we used the training group to determine the model parameters, and took advantage of the discriminant effect on the test group to estimate the generalization ability of the model in practical application. And then, training group and test group, which included profiles of patient's DEmiRNAs and whole survival information were randomly divided by “caret” package for R tools. In training group, DEmiRNAs associated with survival were screened out by using the univariate and multivariate Cox analysis. In order to construct a prognostic model, we set miRNAs with *P* value< 0.01 as a criterion to minimize the number of miRNAs with similar expression. Then, coefficients and prognostic DEmiRNAs were acquired form multivariate Cox regression analysis. For each patient, risk score was obtained by multiplying the coefficient and the expression level of prognosis‑associated DEmiRNAs. Finally, high/low-risk groups were divided depending on median value grouping of risk score, which could measure the prognostic risk for each HCC patient. In addition, based on the survivalROC package in R software, the performance of prognostic miRNA signature was validated using the area under curve (AUC) of 3-year dependent ROC 21.

### Independent prognostic miRNA signature including other clinical variables

Based on our initial works, we selected 172 DEmiRNAs and clinicopathological parameters in training group which related with HCC. OS was defined as the time from diagnosis to death. Univariate and multivariate survival analyses were performed by using the Cox regression model to test independent significance. Kaplan-Meier method and corresponding log rank test were used for survival analyses on categorical variables. In addition, the predictive accuracy of this risk model including risk score and clinical characteristics was also verified by time-dependent ROC curves.

### miRNA-target genes and their potential functions

In order to predict target genes of miRNA signature, datasets of targetScan (http://www.targetscan.org/), miRDB (http://www.mirdb.org/), and miRTarBase (http://mirtarbase.mbc.nctu.edu.tw/) were downloaded and the potential miRNA-target genes which covered at least two datasets were evaluated by Perl language and then visualized by Venn diagram. Next, we illustrated the relationship between miRNA and their target genes by utilizing Cytoscape 3.7.2. Besides, in order to elucidate the function of target genes involved in the progression and development of HCC, we intersected these target genes with differentially expressed genes in HCC and then visualized the obtained intersection genes by GO enrichment analysis and KEGG signaling pathway analysis based on clusterProfiler package and theorg.Hs.eg.db package in R tools. In addition, cut-off criteria were set as the q value < 0.05 and *P* adjust < 0.05.

### Identification of hub gene and survival related gene

STRING (https://string-db.org/) is a free software of protein-protein interactions, which is used to filter and evaluate functional genomics data, and provides a more intuitive platform to annotate the structure, function and evolution of proteins 22. The protein-protein interaction (PPI) network of target genes was constructed by STRING and then visualized by Cytoscape software (http://www.cytoscape.org/) 23. And, we used CytoHubba plug-in of Cytoscape to calculate the degree of each protein node and obtain the top hub genes. Meanwhile, the intersection genes that related with the OS of HCC patients were checked by using the Kaplan-Meier method and log rank test < 0.05.

### Validation of expression levels of miRNAs using qPCR and independent cohort

To study on the expression levels of significant miRNAs in the prognostic model of the HCC patients. Total RNA was extracted from plasma samples using miRNeasy Micro Kit (catalog No.: 217084, Qiagen, Hilden, Germany). According to the protocol, cDNA synthesis and qPCR reaction were performed to quantify the expression level of three miRNAs through the use of MicroRNA First-Strand Synthesis kit (Code No.: 638315, Takara Biotechnology Co., Ltd., Dalian, China) and 2×SYBR Green master mix kit (Code No.: SR1110, Applied Biosystems, CA, USA). U6 small nuclear RNA was taken as the internal reference. Each experiment was repeated three times on the Applied Biosystems V7ii system, and the relative quantitative of miRNA expression was carried out by 2^-ΔΔCt^ method. Besides, DEmiRNAs between HCC patients and unaffected tissues adjacent to tumors in the GSE63046 were identified via Limma R based on R tools, cutoff criteria that FDR < 0.05 and |log_2_FC| > 1 were considered as DEmiRNAs. Also, we simultaneously compared and revalidated significant expression change in TCGA and GEO repository.

### Statistical analysis

All statistical analyses were performed with R software (vision 3.6.0, www.r-project.org), and a value of *P* < 0.05 was regarded as statistically significant. Cox regression model was used for univariate and multivariate hazards analysis to test the independent significance of OS in HCC patients. Kaplan-Meier method and log rank test were used to evaluate the OS of the high- and low-risk patients. Data were presented as hazard ratios (HR) and 95% confidence intervals (95%CI). Mann-Whitney U test was used for comparison between two groups.

## Results

### Identification of DEmiRNAs and DEmRNAs between HCC patients and normal controls

We extracted expression level of DEmRNAs and DEmiRNAs from TCGA database and then obtained 6219 DEmRNAs (Table S1) and 300 DEmiRNA (Table S2) correlated with HCC base on FDR < 0.05 and |log_2_FC| > 1. Among them, 1349 DEmRNAs were down-regulated and 4870 were up-regulated (Figure 1A-B). While 40 DEmiRNA were down-regulated and 260 were up-regulated (Figure 1C-D).

### Construction of the DEmiRNAs-based prognostic signature

**Figure 2:**
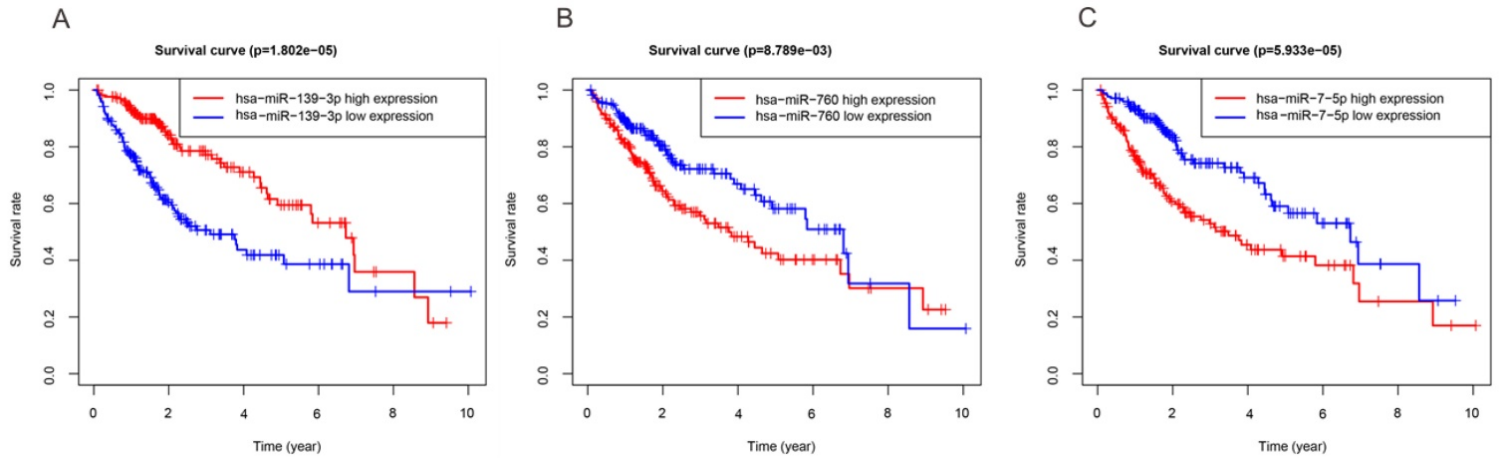
** Three miRNAs associated with OS of HCC patients.** High and low expression groups were subdivided according to the median expression of each miRNA. (A) hsa-miR-139-3p. (B) hsa-miR-760. (C) hsa-miR-7-5p.

In order to investigate the DEmiRNAs associated with OS of HCC, we randomly divided the entire datasets (N = 344) with DEmiRNAs profiles into training group (N =172) (Table S3) and test group (N = 172) (Table S4). Then, we performed COX proportional hazards model which took survival outcome and survival time as dependent variables and can simultaneously analyze the influence of many factors on survival time of HCC patients. The univariate Cox regression analysis revealed that DEmiRNAs including hsa-miR-139-3p, hsa-miR-139-5p, hsa-miR-101-3p, hsa-miR-3677-3p, hsa-miR-760, hsa-miR-3189-3p, and hsa-miR-7-5p were significantly associated with OS in the training group (Table 2). Thereafter, we implemented stepwise multivariate Cox regression analysis to eliminate redundant variables which were not relevant with the OS of HCC patients. Finally, a total of three miRNAs including hsa-miR-139-3p, hsa-miR-760, hsa-miR-7-5p were finally screened out (Table 2). In addition, Kaplan-Meier method demonstrated that three miRNAs were associated with patients' overall survival (Figure 2). And then, a DEmiRNAs prognostic model for prediction and diagnosis in the whole HCC cohort was established by using coefficients and corresponding expression values of each risk differentially expressed miRNA. In our current work, risk score = (0.306×expression value of hsa-miR-7-5p) + (0.257×expression value of hsa-miR-760) - (0.259×expression value of hsa-miR-139-3p).

### Validation of the three-miRNA signature in the training group, test group, and entire set

**Figure 3:**
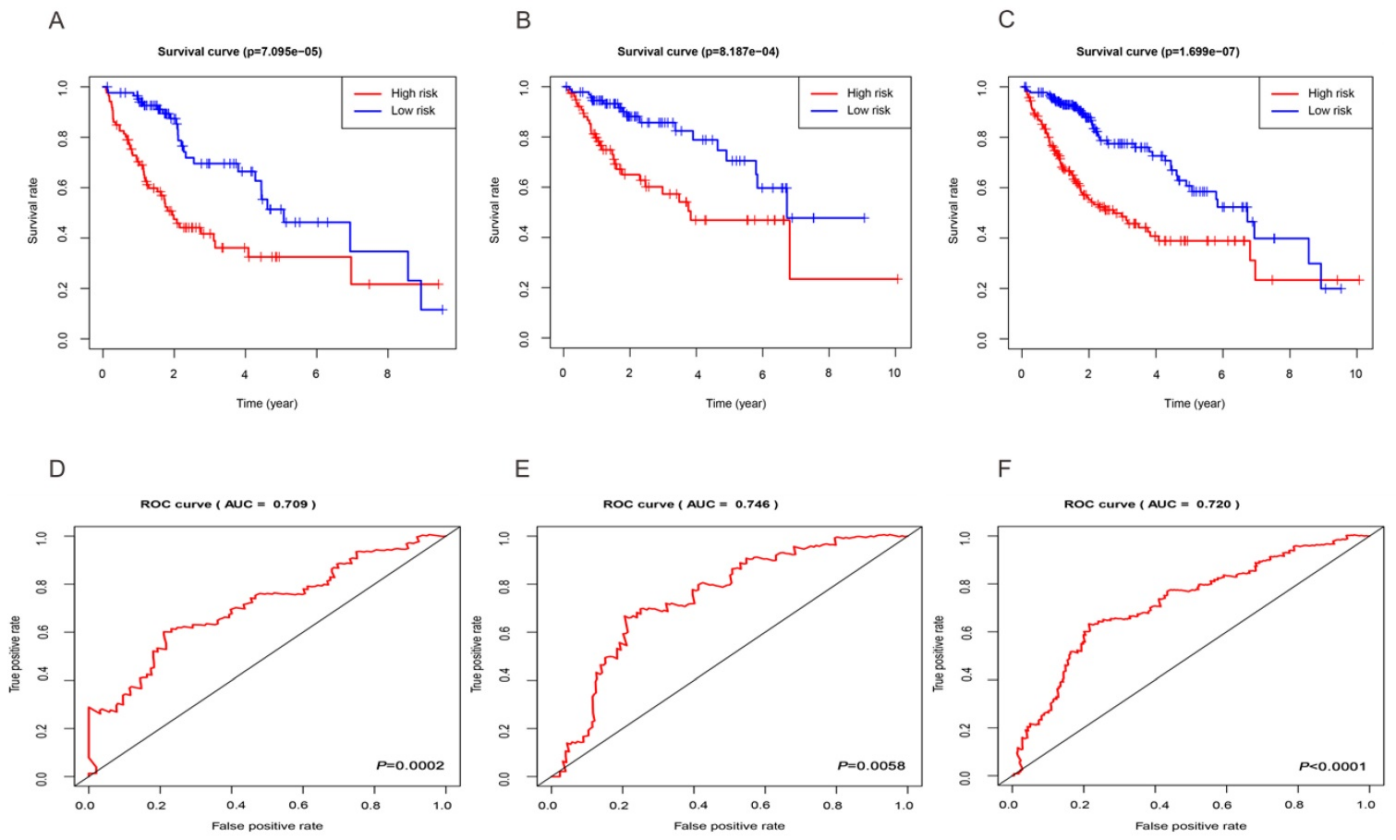
** Evaluation and validation of the three-miRNA signature.** Kaplan-Meier curves of the training group (A), test group (B), entire set (C); The AUC of the 3-year dependent ROC curve in the training group (D), test group (E), entire set (F).

Based on the median risk score, we subdivided all HCC datasets into two groups with high and low risk. As demonstrated by the Kaplan-Meier survival and log-rank test, HCC patients with low-risk score had good outcomes compared with high-risk patients in the training group (Figure 3A), test group (Figure 3B), and entire set (Figure 3C). In addition, the results showed that the 3-year rates of the patients with high risk (38.9%) were significantly lower than those of patients with low risk (69.6%) in the training group. The test group showed that the 3-year OS rates for patients with high risk (57.3%) were significantly shorter than patients with low risk (82.4%). The entire set demonstrated that the 3-year OS rates for patients with high and low risk group were 48.5 and 76.0%, respectively. Besides, the AUC of the 3-year dependent ROC curve in the training group was 0.709 (Figure 3D), whereas the AUC for the test group was 0.746 (Figure 3E), and for all HCC cohorts was 0.720 (Figure 3F), which demonstrated a good performance of DEmiRNAs involved signature to predict the survival risk of HCC patients.

### The three-miRNA signature is an independent prognostic factor considering other clinical factors

**Figure 4:**
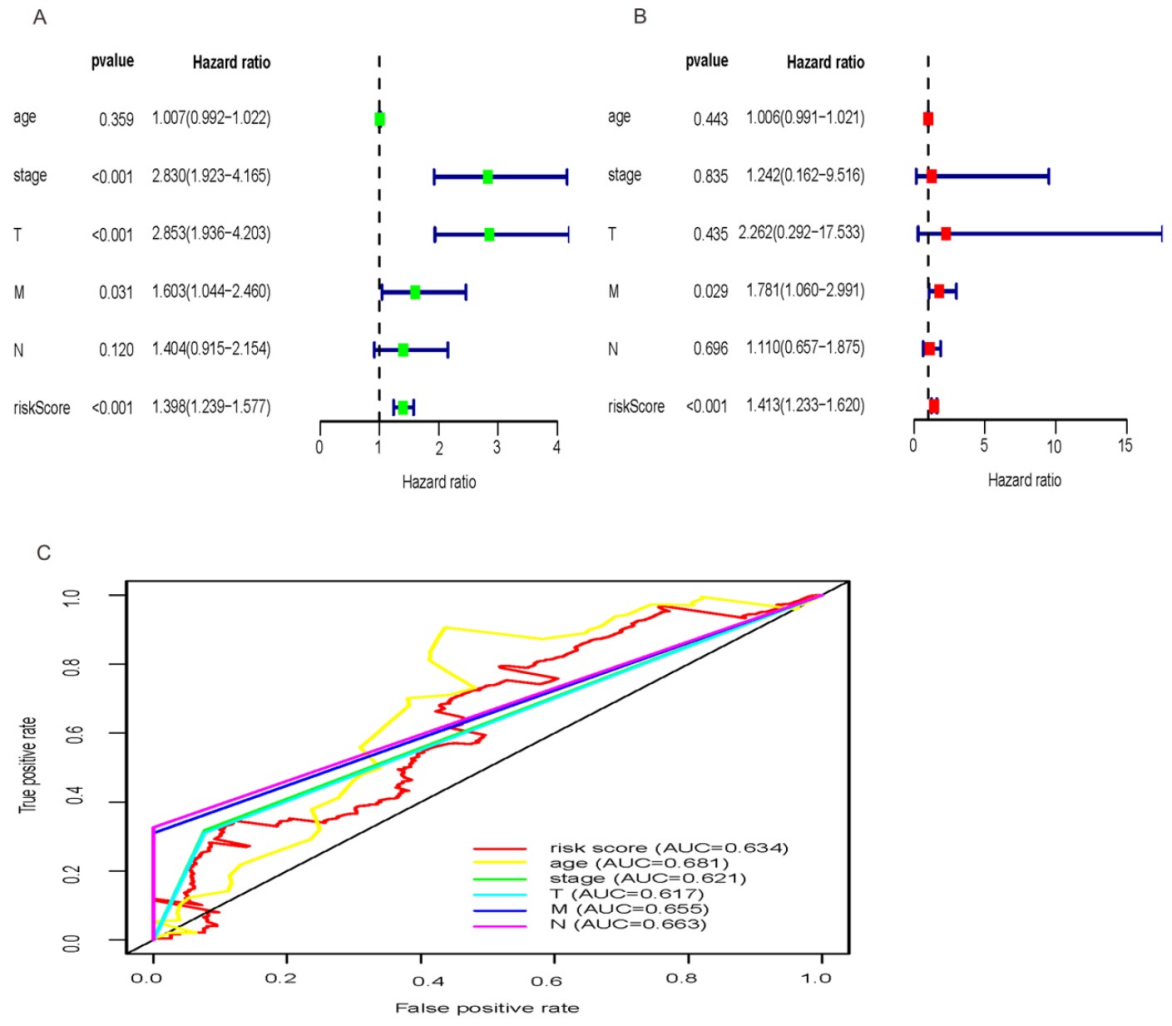
** Univariate and multivariate Cox regression hazards model for predictors of OS with HCC and prediction accuracy of risk score and clinical features.** Univariate Cox (A) and multivariate Cox (B) regression analyses of risk score and clinicopathological parameters with prognostic potential. The dot represents Hazard ratio, and transverse line represents 95% Confidence Interval. (C) ROC curve for predicting OS in HCC patients by the risk score and clinicopathological parameters.

Exploring whether the signatures of the three miRNAs were independent with OS considering other clinical characters such as TNM stage, age and clinical stage, univariate Cox regression analysis demonstrated that the three-miRNA signature was strongly related with OS of HCC patients (Figure 4A; HR = 1.398, 95% CI = 1.239-1.577, *P*<0.001). While, multivariate Cox regression analysis were applied to designate that the three- miRNA signature remained an independent prognostic factor (Figure 4B; HR = 1.413, 95% CI = 1.233-1.620, *P*<0.001). And, distant metastasis was also considered as an independent prognostic factor (Figure 4B; HR = 1.781, 95% CI = 1.060-2.991, *P* = 0.029). In addition, the predictive ability of the signature including risk score and clinical factors was validated by the Roc curves (Figure 4C). The results showed that the AUC of risk score (0.634), age (0.681), clinical stage (0.621), T stage (0.617), distant metastasis (0.655), lymph-node status (0.663) were all greater than 0.5, indicating a superior predictive power. Besides, we performed the inbuilt “cox.zph” in R language to validate the proportional hazard assumption of cox regression model. The results demonstrated that *P* value of the risk score (0.27) was greater than 0.05, indicating that the variable met the proportional hazard assumption. In addition, the *P* value of the overall test was 0.12, indicating that the validity of the risk score depends on the validity of this assumption (Table S7).

### Prediction of target genes for the three miRNAs

**Figure 5:**
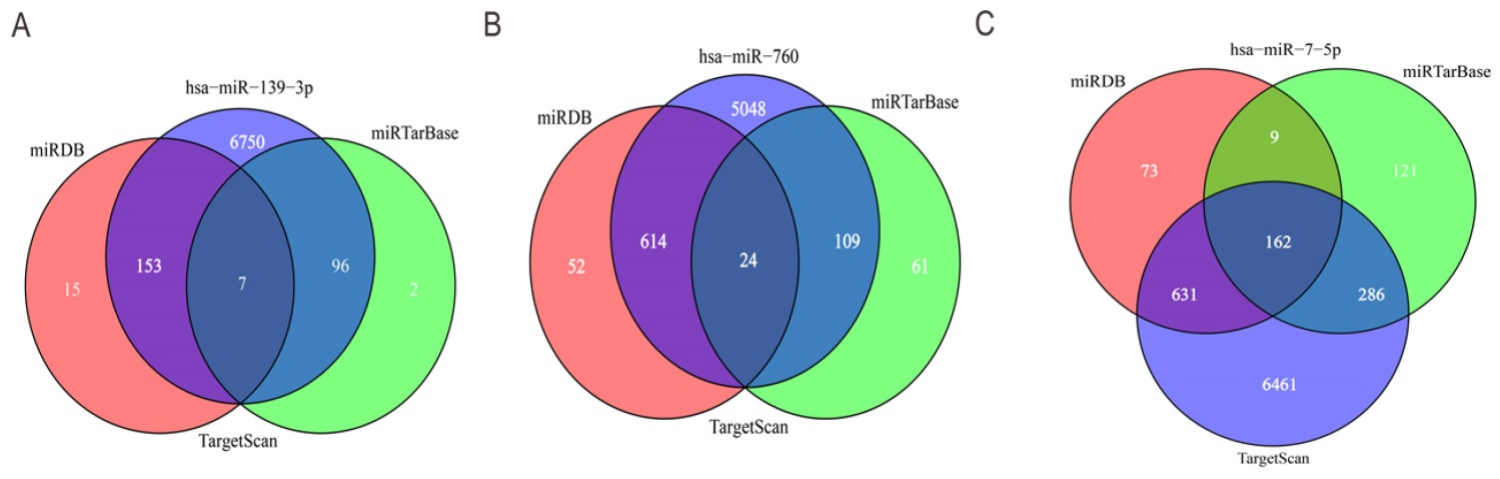
** Venn diagram of the intersection of three miRNA target genes.** (A) hsa-miR-139-3p, (B) hsa-miR-760, (C) hsa-miR-7-5p.

**Figure 6:**
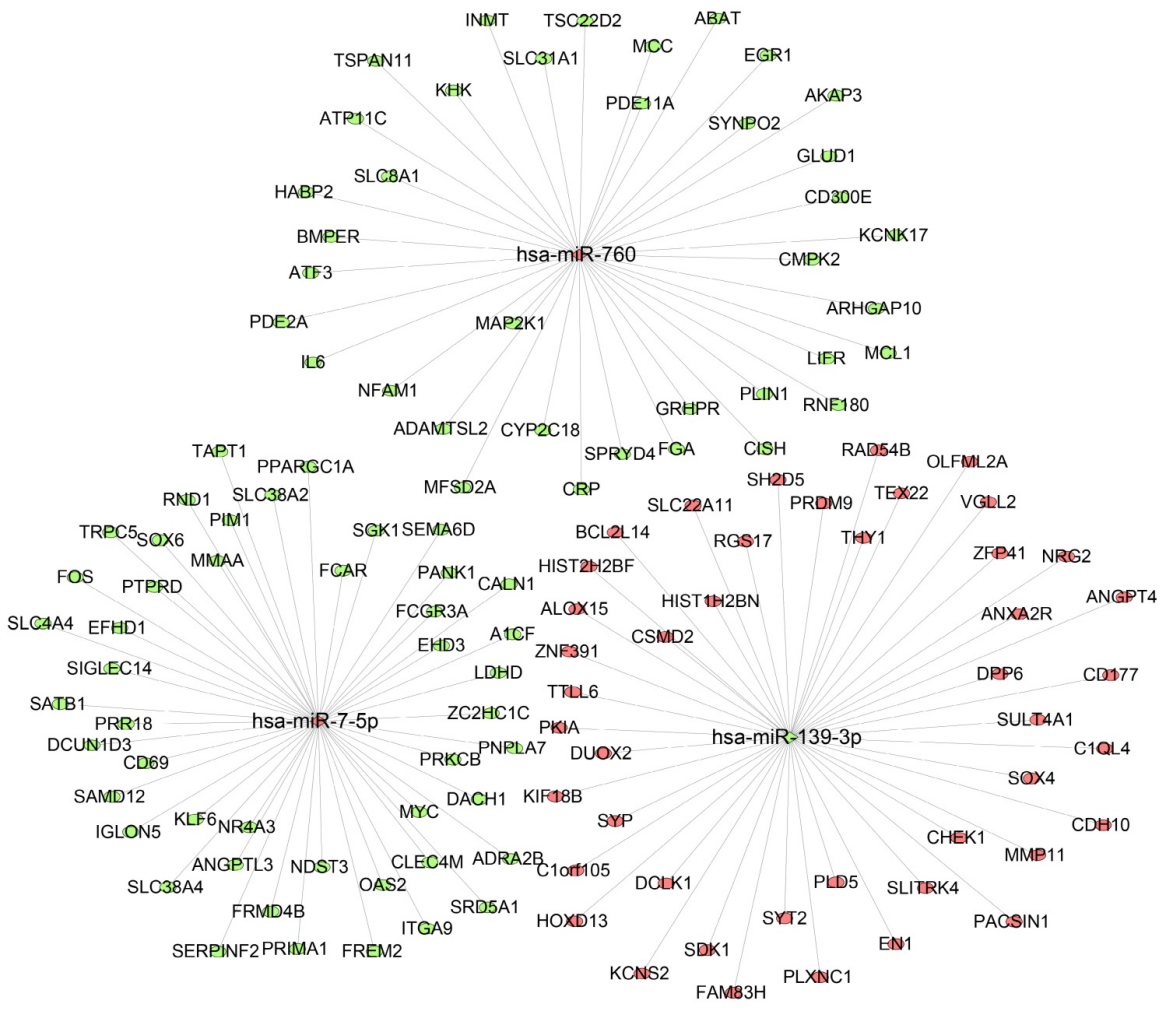
** Interaction networks of the three miRNAs and their target genes.** The ellipse represents target gene, rhombus represents miRNA, and the link in black indicates a miRNA-target gene relationship. Red means upregulated; green means downregulated.

To further predict the targets genes of hsa-miR-139-3p, hsa-miR-760, hsa-miR-7-5p, the online target prediction analysis tools including TargetScan, miRDB and miRTarBase were utilized and visualized by Venn diagram (Figure 5) and network map of miRNA target genes (Figure 6). The results showed that 162, 7, 24 overlapping genes were identified for hsa-miR-7-5p, hsa-miR-139-3p, and hsa-miR-760. Moreover, a total of 128 target genes were screened out for the three miRNAs, of which 84 were downregulated and 44 were upregulate, respectively.

### Gene ontology and KEGG pathway analysis for target genes in HCC

**Figure 7:**
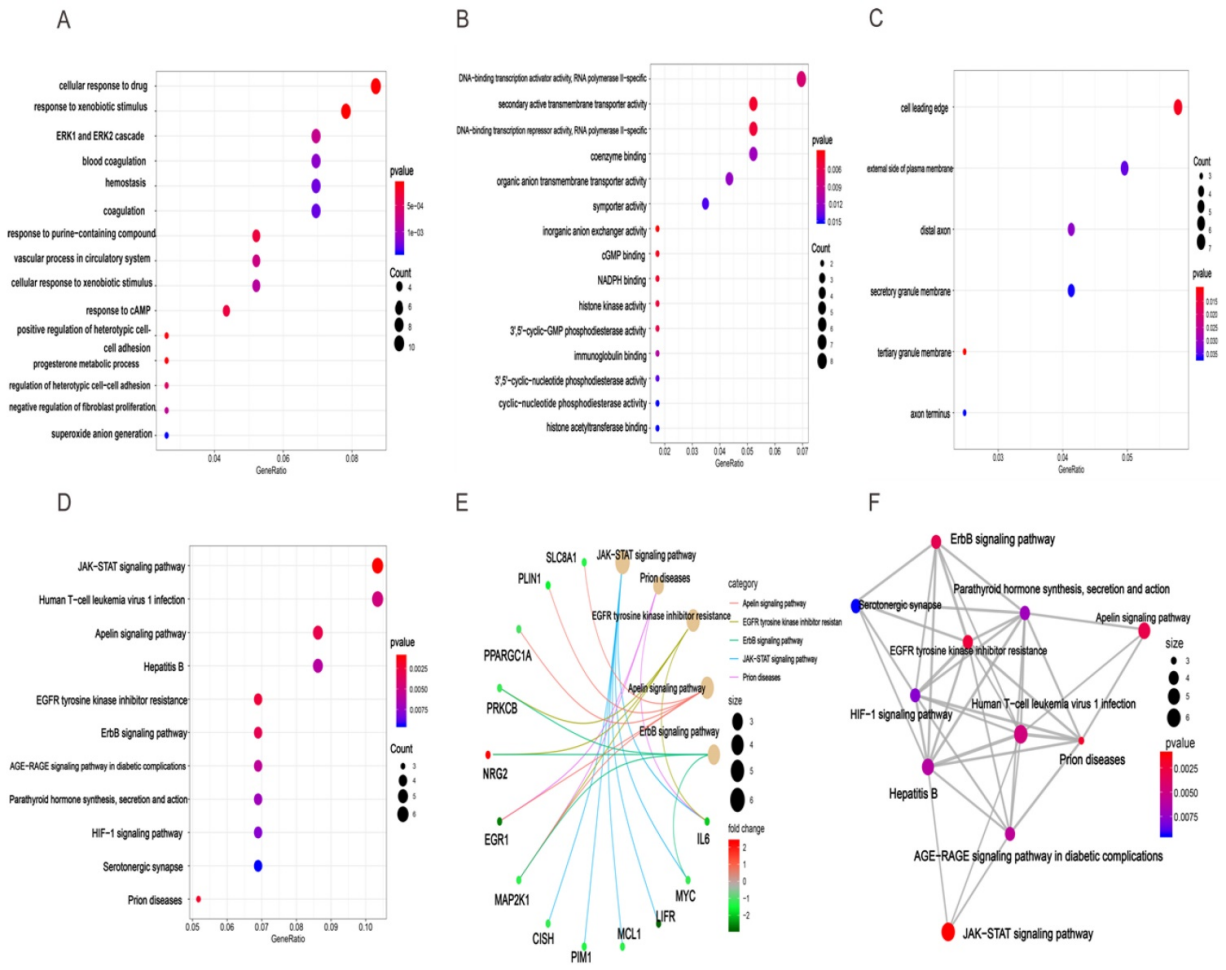
** GO enrichment and KEGG pathway enrichment analysis of target genes associated with HCC.** BP(A), MF(B), and CC(C) involved in molecular characteristics of target genes, (D)Top 11 signaling pathways of KEGG, (E) pathway-gene network, (F) pathway-pathway network.

Based clusterProfiler package in R tools, we acquired GO annotations and KEGG pathway enrichment of target genes involved in HCC. Results of GO analysis indicated that there were 381 GO annotations associated with the development of HCC and the top 15 terms of biological process (BP), cellular component (CC), and molecular function (MF) were displayed in dot plot (Figures 7A-C). Among these three categories, target genes were mainly enriched in BP terms and mostly include cellular response to drug, response to xenobiotic stimulus, and ERK1 and ERK2 cascade. For CC section, analysis showed that the tops three terms were cell leading edge, external side of plasma membrane, and distal axon. In the MF section, DNA-binding transcription activator activity, secondary active transmembrane transporter activity, and DNA-binding transcription repressor activity were top three terms enriched by target genes (Table S5). In addition, the results of KEGG analysis demonstrated that 11 HCC related pathways were mainly enriched in JAK-STAT signaling pathway, human T-cell leukemia virus 1 infection, apelin signaling pathway, and Hepatitis B (Figures 7D, Table S6). Besides, we also provided “pathway-pathway network” and “pathway-gene network” to illustrate the relation between KEGG pathway and target genes (Figures 7E-F).

### Protein-protein interaction network (PPI) construction, hub gene identification and survival analysis of target genes

**Figure 8:**
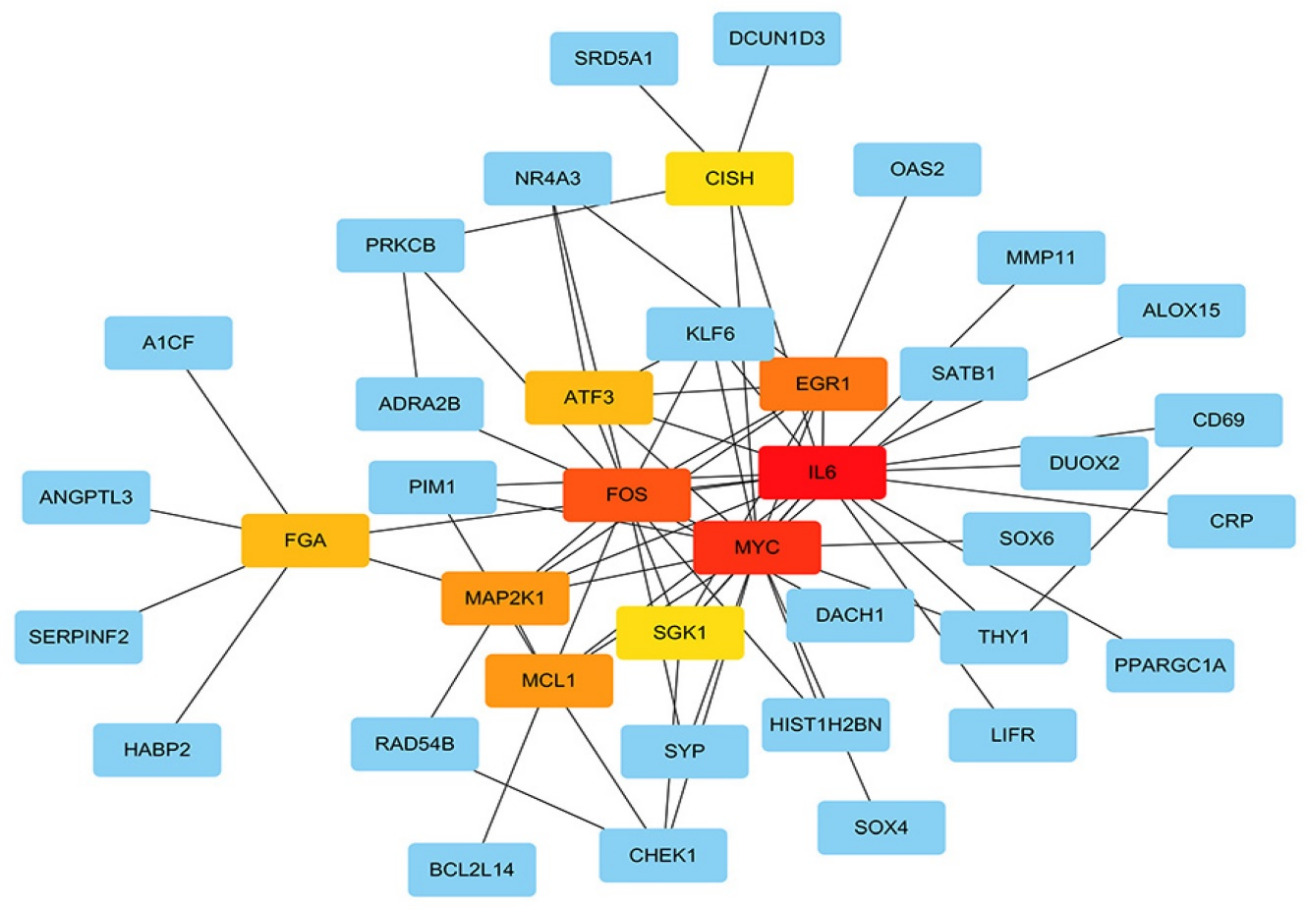
** Hub genes analyzed by cytoHubba in Cytoscape software.** The darker color represents the bigger degree.

**Figure 9:**
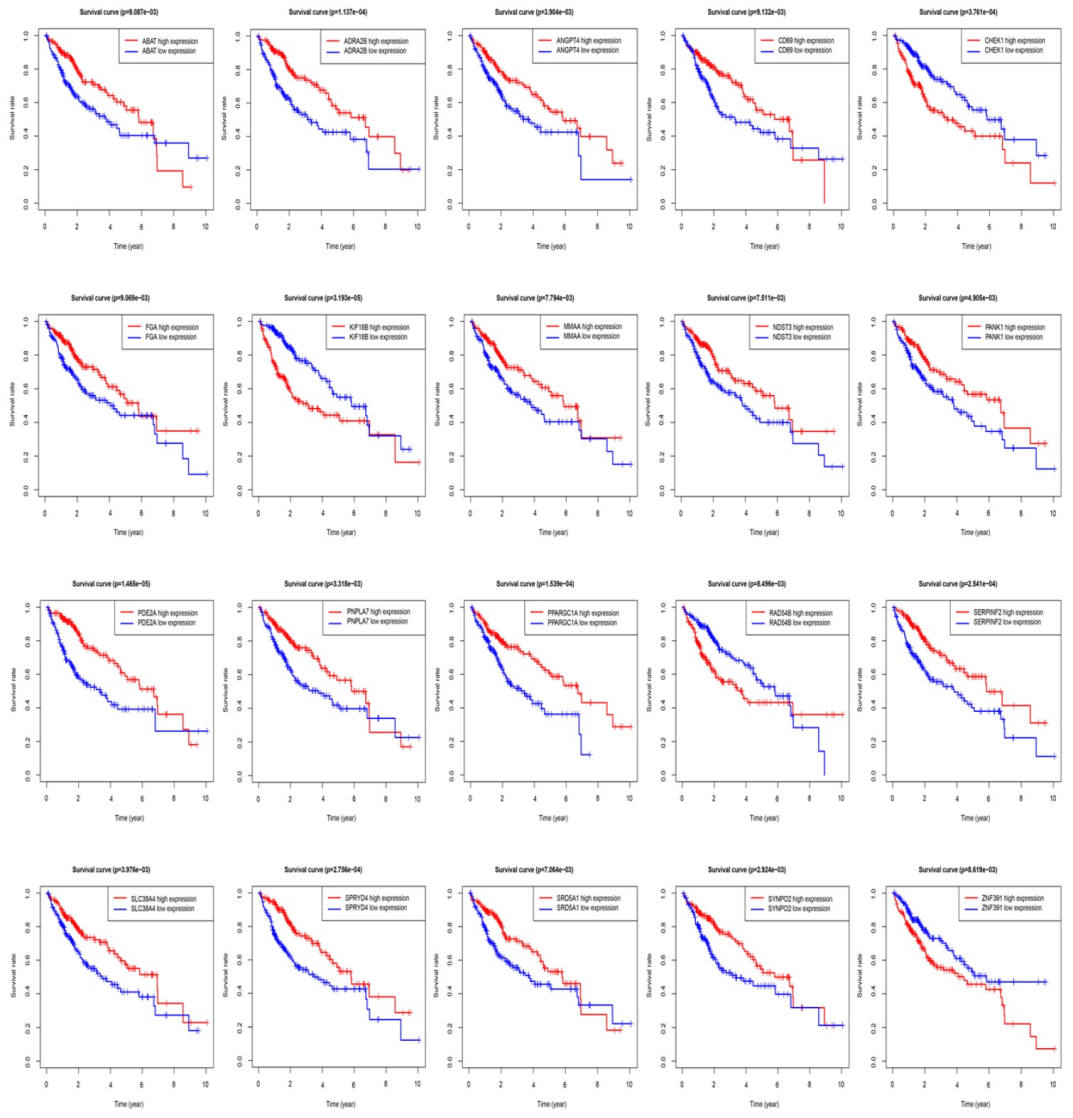
Overall survival (OS) of target genes in HCC patients.

The STRING protein interaction database was used to construct a PPI network containing 128 target genes. A total of 128 nodes and 97 edges were involved in PPI network. The connectivity degree was used as the truncation standard to calculate the degree of each protein node, and the top 10 hub genes (IL6, MYC, FOS, EGR1, MCL1, MAP2K1, ATF3, FGA, SGK1, CISH) were screened out by plug-in cytoHubba of Cytoscape (Figure 8 and Table 3). In addition, a total of 20 target genes including ABAT, ADRA2B, ANGPT4, CD69, CHEK1, FGA, KIF18B, MMAA, NDST3, PANK1, PDE2A, PNPLA7, PPARGC1A, RAD54B, SERPINF2, SLC38A4, SPRYD4, SRD5A1, SYNPO2, ZNF391 were significantly related with OS of HCC patient using Kaplan-Meier method (Figure 9).

### Expression levels of three miRNAs

**Figure 10:**
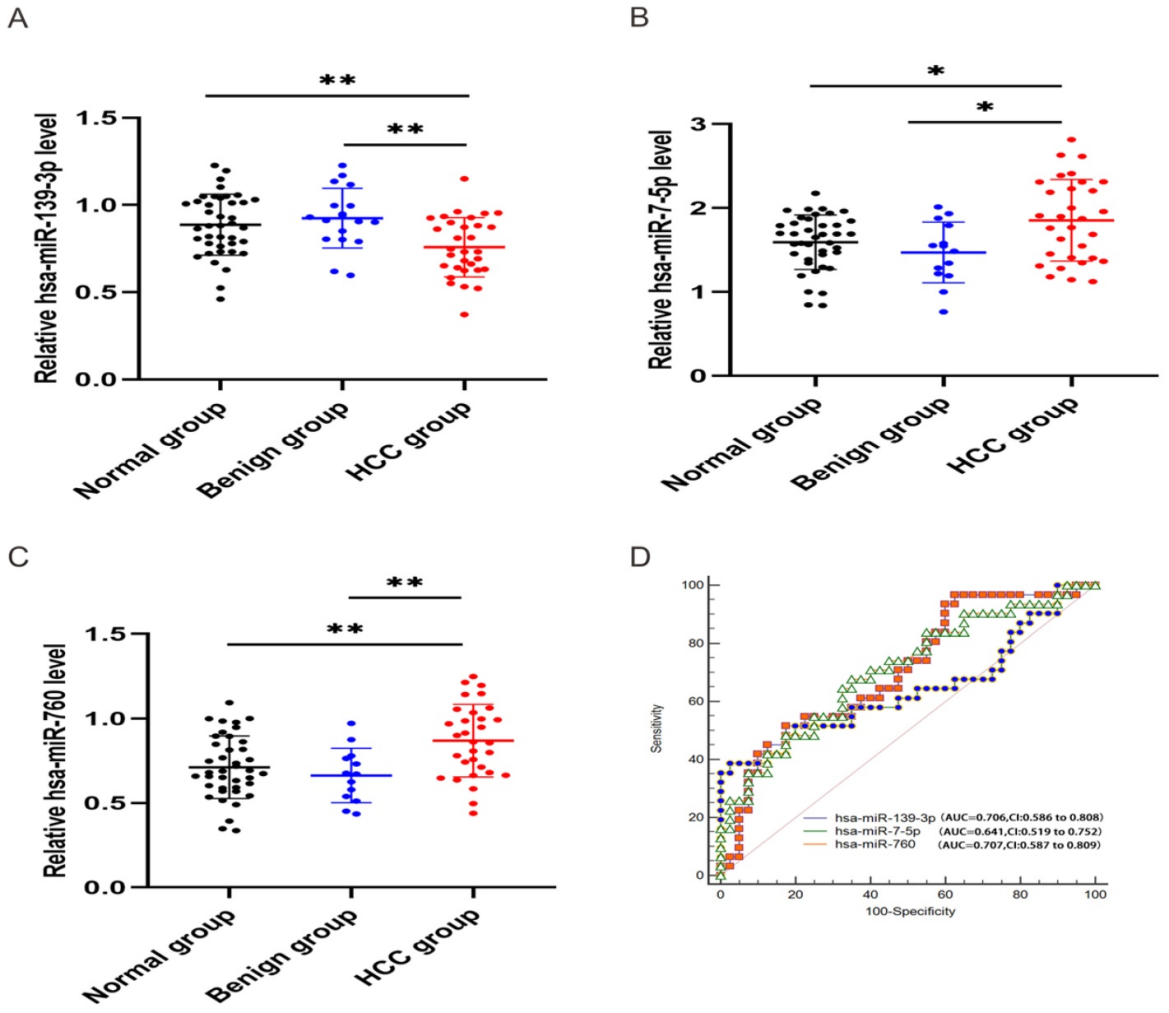
** Relative expression level of hsa-miR-139-3p, hsa-miR-760, hsa-miR-7-5p.** qPCR analysis showed that hsa-miR-139-3p was dramatically down-regulated (A), while hsa-miR-7-5p (B) and hsa-miR-760 (C) in HCC group were significantly up-regulated compared with benign and normal group. Besides, ROC curve was used to predict diagnostic efficiency of three miRNAs between HCC patients and normal controls (D). *P <0.05 (vs. normal or benign control), **P<0.01 (vs. normal or benign control)

**Figure 11:**
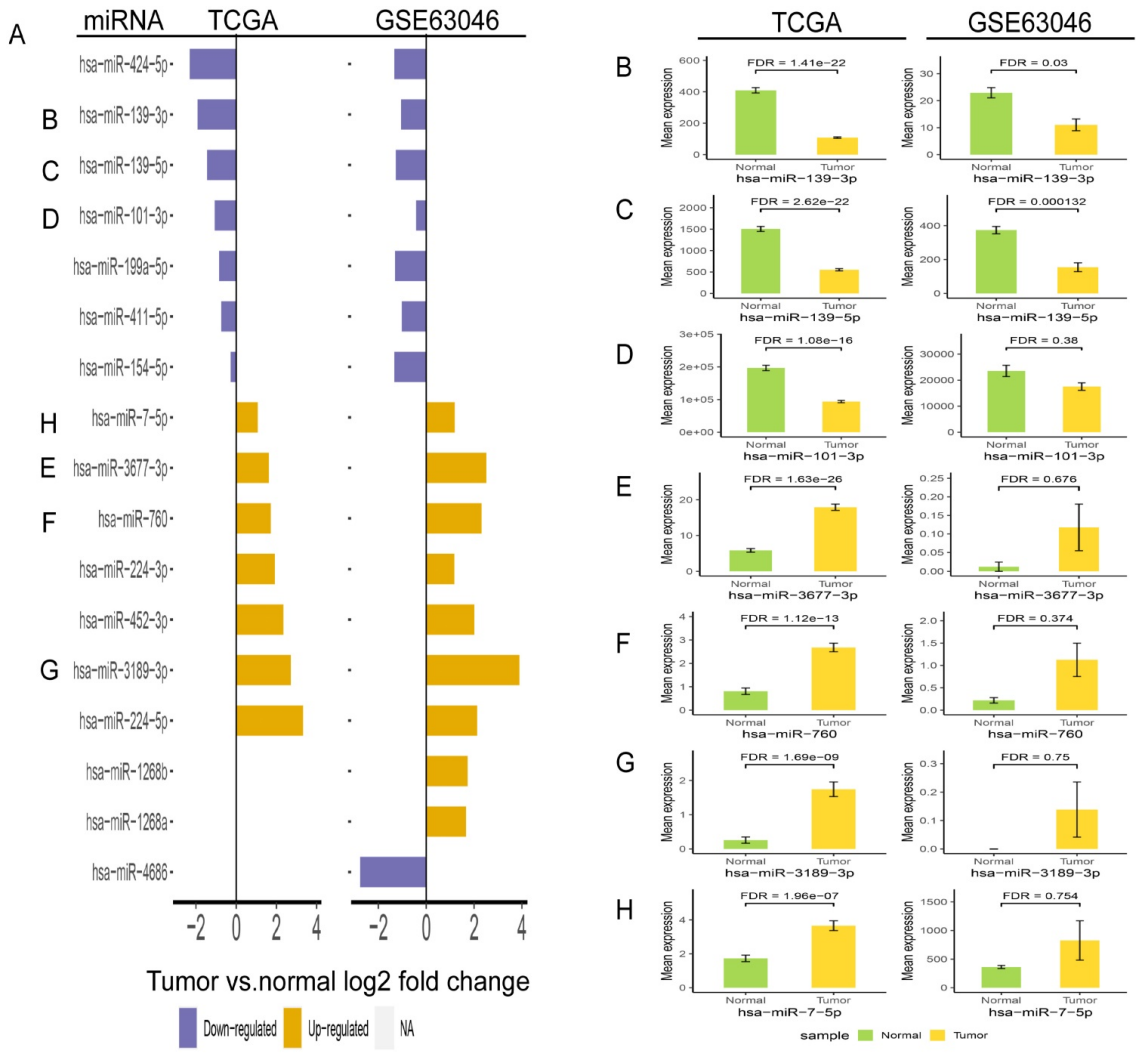
Validation of fold change (A) and expression change (B-H) for seven miRNAs in TCGA and GSE63046 database.

In order to validate the three miRNAs signature in vitro, we performed qPCR to detect the expression levels of hsa-miR-139-3p, hsa-miR-760, hsa-miR-7-5p in plasma of HCC patients. Compared with benign and normal patients, the levels of hsa-miR-139-3p were generally decreased (Figure 10A), while the levels of hsa-miR-7-5p and hsa-miR-760 were generally increased (Figure 10B-C). In addition, we performed the Roc curves to predict diagnostic efficiency of three miRNAs between HCC patients and normal control (Figure 10D). The results demonstrated that the AUC of hsa-miR-139-3p (0.706), hsa-miR-760 (0.707), hsa-miR-7-5p (0.641) also showed a superior diagnostic power. Besides, fold change of seven miRNAs (hsa-miR-139-3p, hsa-miR-139-5p, hsa-miR-101-3p, hsa-miR-3677-3p, hsa-miR-760, hsa-miR-3189-3p, and hsa-miR-7-5p) were significantly changed in both TCGA and GEO database (Figure 11A). Undoubtedly, the expression levels of hsa-miR-139-3p and hsa-miR-139-5p were dramatically up-regulated in GSE63046(Figure 11B-C). The remaining miRNAs expression changes were shown in Figure 11D-H.

## Discussion

With the popularization of serum marker detection and imaging examination technology, the diagnostic rate of early HCC has been significantly improved in recent years. However, the overall prognosis is still disappointing which is closely related to the high aggressiveness of tumor cells 24, 25. Therefore, reproducible biomarkers are needed to predict the survival of patients with HCC at clinical settings. Based on bioinformatic studies, extensive evidence has been theoretically investigated to show that abundant target genes were regulated by miRNAs, which virtually involves in every process of malignant tumors pathway regulation, therapeutic strategy, and prognosis^26^-^29^. In particular, in the process of tumorigenesis and development, a large number of miRNAs play different roles as prognostic biomarkers, including lung cancer 30, breast cancer 31, colorectal cancer 32, prostate cancer 33, gastric cancer 34, and pancreatic cancer 35. Indeed, previous studies have demonstrated that multiple miRNA signatures were much more accurate as prognosis factors compared with single miRNA. And, prior to our study, there were already numerous HCC prognostic markers based on multiple miRNA signatures. It was shown that the prognostic signature consisting of five miRNAs remarkably correlated with OS of HCC patients. Especially, miR-424, miR-326 and miR-511 could be used as biomarkers of HCC 36. Another research demonstrated a group of seven-miRNA (miR-187, miR-9, miR-490, miR-1258, miR-3144, miR-551a, miR-665) that also revealed a good predictive performance of OS for HCC patients 37. Besides, cross-validation were performed to verify miRNA prognostic power based on TCGA database and GEO repositories 38. While, the biggest highlight of our research is the emphasis on research methods, especially sample groupings and mature miRNAs were used to validate the accuracy of the model. And it was also worth noting that the expression levels of miRNAs were validated by independent cohort and external experimental study.

In this current study, we downloaded and merged related RNA expression profile and clinical information of HCC from TCGA database. Then, edgeR package for R tools was used to identify DEmiRNAs and DEmRNAs in HCC patients. Afterwards we divided all the patients into groups, which led to training group and test group. And, through Cox proportional hazards analyses, a DEmiRNA related signature model was constructed in train group. The stepwise multivariate Cox regression showed that hsa-miR-139-3p, hsa-miR-760, hsa-miR-7-5p have independent prognostic significance and the three miRNAs were significantly associated with OS of HCC patients. Meanwhile, three-miRNA signature was evaluated and validated in the training group, test group, and entire set based on the median risk score. Kaplan Meier survival and log-rank test demonstrated that patients with high risk score had worse outcomes compared with low risk patients. What's more, the results of ROC curve showed that the predict power of three-miRNA signature for OS in three groups. In addition, considering other clinical factors, the three-miRNA signature is still an independent prognostic factor for HCC patients. What's more, we also verified the expression levels of three miRNAs in GEO database and clinical specimens, which provided data support for subsequent studies.

Due to the application of tumor Big data analyzed by computational methods, many researches attempted to identify potential miRNA signature that involved in the pathogenesis of cancer, comprehensively analyze the complexity of tumor and find out targeted tumor treatment schemes 20, 39-41. In fact, three miRNAs have been reported to be involved in the development of various tumors. It has been found that the process of cell proliferation, invasion and metastasis can be suppressed by up-regulating the expression of miRNA-139-3p in glioma cells 42. And, ELAV Like RNA Binding Protein 1 (ELAVL1) was inversely regulated by miRNA-139-3p, which played an important role in the cell proliferation, invasiveness and migration of ovarian cancer cell lines 43. More interestingly, the expression level of miRNA-139-3p in HCC tissues was apparently lower than that in normal control 44, and was positively correlated with better prognosis 45.

Previous studies reported that epithelial-mesenchymal transition (EMT) and phosphatidylinositol 3-kinase/ protein kinase B (PI3K/AKT) signal pathway was strictly regulated by up-regulation of miR-760 in colorectal carcinoma (CRC) 46. As an independent prognostic indicator, overexpression of miR-760 can significantly inhibit proliferation of esophageal squamous cell carcinoma by regulating fat metabolism via c-Myc expression 47. Moreover, the miR-760/MOV10/ITGB1 pathway was important for the sensitivity of cells to the chemotherapy drugs in pancreatic cancer 48. Similarly, miR-760 can effectively enhance the function of doxorubicin in HCC by modulating Notch1/Hes1-PTEN/Akt signal pathway 49. In addition, dysregulation of miR-7-5p was detected in gastric cancer stem cells, which was caused by increased DNA methylation 50. Notably, inhibition of miR-7-5p can directly induce apoptosis via targeting P21-activated kinase 2 (PAK2) in non-small-cell lung cancer 51. It was also demonstrated that miR-7-5p/YAP1 axis exerted a big role in progression of CRC 52.

Then, target genes of the three miRNAs were predicted by online target analysis database and later visualized by Venn diagram. In our study, we screened out 131 target genes of the three miRNAs. Next, GO and KEGG were used to analyze the function and pathway enrichment of target genes, then PPI was constructed using Cytoscape software and the top 10 hub genes were screened out by plug-in cytoHubba. Our results revealed that GO annotations of the target genes were mainly related with cellular response to drug, xenobiotic stimulus, ERK1 and ERK2 cascade, cell leading edge, external side of plasma membrane, distal axon, DNA-binding transcription activator activity, secondary active transmembrane transporter activity, and DNA-binding transcription repressor activity. KEGG analysis of target genes mainly enriched in JAK-STAT signaling pathway, human T-cell leukemia virus 1 infection, apelin signaling pathway, and hepatitis B. Aberrant signaling pathways were crucial for the tumorigenesis and increasing evidence indicated that JAK-STAT pathway was involved in the development of HCC 53. Human T-cell leukemia viruses affected the clearance of hepatitis C virus (HCV) and then accelerated the development of HCC 54. And, Apelin seems to participate the hepatic remodeling and carcinogensis in HCC patients 55. Next, 10 hub genes of the miRNA signature were screened out by plug-in cytoHubba of Cytoscape. Furthermore, we also identified 20 genes that definitely associated with OS of HCC. Surprisingly, FGA (fibrinogen alpha chain) was not only the hub gene of PPI network, but also related to the prognosis of HCC patients. Previous studies demonstrated that alpha subunit of the coagulation factor fibrinogen encoded by FGA can involve in the progression of several disorders, including dysfibrinogenemia, hypofibrinogenemia, afibrinogenemia and renal amyloidosis. Especially, FGA served as a new biomarker for HCV-infected alcoholic patients with cirrhosis 56.

In fact, the proposed prognostic model in our study was estimated using the normalized data and the normalization process somewhat specific for the features of the current data. There will be some practical difficulties for one to use the model for a new patient based on his/her expression data, as the data should go through one or few layers of data normalization. For this, when the predictive ability of the prediction model in the new queue is not as good as that in our modeling queue, the first consideration is to use the new data to adjust the model rather than to establish a new model. In this way, the proposed prediction model can provide an objective prediction of the risk of outcome events and serve as a supplement to clinicians' judgment and clinical guidelines.

However, there are still some limitations in our research. Firstly, due to the insufficient plasma samples enrolled in our validation, we still need to further expand the sample size for verification in the next phase. Secondly, we measured the levels of three miRNAs in vitro, but it is still unclear whether miRNAs were produced by the cancer itself or by the host's own response to the cancer. Therefore, further research is required to study the function of three miRNAs.

## Conclusion

In summary, we constructed the DEmiRNAs related prognosis model that could predict the OS of HCC patients. What's more, the three miRNAs could be used as prognostic biomarkers and molecular target in HCC. Nevertheless, a large independent cohort is needed to validate the signature before it is applied to clinical practice.

## Supplementary Material

Supplementary tables.

## Figures and Tables

**Table 1:** Clinical characteristics of HCC patients.

Covariates	Case	Percentage (%)
**Age(years)**		
<60	172	45.6
≥ 60	204	54.1
Unknown	1	0.3
**Gender**		
Female	122	32.4
Male	255	67.6
**Race**		
White	187	49.6
Asian	161	42.7
Black or African American	17	4.5
American Indian or Alaska native	2	0.5
Not reported	10	2.7
**Lymph node status**		
N0	257	68.1
N1	4	1.1
NX	115	30.5
Unknown	1	0.3
**Stage**		
I	175	46.4
II	87	23.1
III	86	22.8
IV	5	1.3
Unknown	24	6.4
**T stage**		
T1	185	49.1
T2	95	25.2
T3	81	21.5
T4	13	3.4
TX	1	0.3
Unknown	2	0.5
**Metastasis**		
M0	272	72.1
M1	4	1.1
MX	101	26.8

**Table 2:** DEmiRNAs involved in prognosis associated model of HCC

ID	Univariate Cox regression					Multivariate Cox regression				
	HR	HR.95L	HR.95H	P value		Co ef	HR	HR.95L	HR.95H	P value
hsa-miR-139-3p	0.720	0.602	0.859	0.000		-0.259	0.772	0.642	0.928	0.006
hsa-miR-139-5p	0.677	0.564	0.812	0.000						
hsa-miR-101-3p	0.697	0.535	0.906	0.007						
hsa-miR-3677-3p	1.370	1.094	1.716	0.006						
hsa-miR-760	1.375	1.119	1.690	0.002		0.257	1.293	1.050	1.592	0.015
hsa-miR-3189-3p	1.311	1.082	1.589	0.006						
hsa-miR-7-5p	1.424	1.167	1.736	0.000		0.306	1.359	1.112	1.660	0.003

**Table 3:** Top 10 hub genes with higher degree of connectivity

node_ name	MCC	DMNC	MNC	Degree	EPC	Bottle Neck	Ec Centricity	Closeness	Radiality	Betweenness	Stress	Clustering Coefficient
IL6	140	0.306	13	19	15.705	35	0.134	32.850	6.493	1155.228	2040	0.140
MYC	141	0.292	14	17	15.589	39	0.161	31.783	6.508	920.295	1760	0.191
FOS	133	0.373	11	14	15.246	15	0.161	30.116	6.447	739.328	1512	0.252
EGR1	79	0.512	7	8	13.839	3	0.134	26.016	6.189	226.785	432	0.500
MCL1	33	0.427	6	7	13.062	2	0.134	25.266	6.143	124.052	264	0.428
MAP2K1	51	0.475	6	7	13.498	9	0.134	26.350	6.265	274.566	762	0.476
ATF3	54	0.523	6	6	12.854	2	0.134	24.683	6.128	17.252	70	0.733
FGA	6	0.307	2	6	8.921	7	0.115	23.492	5.915	590.000	1322	0.066
SGK1	26	0.453	5	5	12.295	2	0.134	24.350	6.128	14.833	52	0.700
CISH	5	0.307	2	5	9.519	5	0.134	24.100	6.098	245.933	398	0.100
