# Supplementary material for: Construction and validation of a three-microRNA signature as prognostic biomarker in patients with hepatocellular carcinoma
Source: Int J Med Sci. 2021 Jan 1;18(4):984–99. doi: 10.7150/ijms.49126 (PMC7807177; doi:10.7150/ijms.49126)
Supplement: Supplementary file 1 — Supplementary tables. [file ijmsv18p0984s1.pdf]

Table S1. Differentially expressed mRNAs between hepatocellular carcinoma samples and norm

id	logFC	logCPM	PValue	FDR
ADAMTS13	-2.833207344	3.334405025	1.15E-129	2.56E-125
STAB2	-4.870650299	2.305374593	1.47E-113	1.64E-109
OIT3	-3.262127723	4.410218665	3.44E-113	2.57E-109
KLHL30-AS1	-7.265158998	-0.701741025	2.55E-106	1.42E-102
CFP	-3.418713806	3.044824775	3.10E-95	1.39E-91
ECM1	-3.086527178	3.816488518	1.45E-94	5.41E-91
BMPER	-4.526397199	0.973575663	3.92E-91	1.25E-87
CCL23	-2.952799406	-0.954369445	3.10E-90	8.68E-87
ANGPTL6	-2.982287311	2.453902659	9.27E-89	2.30E-85
CSRNP1	-2.250080147	5.294886342	4.77E-86	1.07E-82
CLEC4G	-5.413207123	3.406424078	3.15E-83	6.41E-80
CRHBP	-4.491933308	3.677574701	5.54E-79	1.03E-75
RCAN1	-2.402435306	6.072760912	9.34E-77	1.61E-73
RND3	-2.504751072	5.552944615	5.25E-76	8.38E-73
PTH1R	-3.256419562	2.508696952	1.20E-75	1.79E-72
MARCO	-4.874420498	3.961913955	3.75E-75	5.25E-72
VIPR1	-3.480629262	2.677165122	1.14E-74	1.50E-71
NTF3	-3.514934593	0.280446008	2.78E-73	3.46E-70
FCN3	-4.111636594	4.706777862	4.61E-73	5.43E-70
FCN2	-4.89769589	3.248707139	1.23E-72	1.38E-69
MAP2K1	-1.270179745	5.319579407	8.05E-71	8.58E-68
GABRD	4.505903141	1.732527598	6.85E-70	6.96E-67
LRAT	-2.909145621	1.506527597	3.21E-68	3.12E-65
ZFP36	-2.162319752	7.231484262	1.03E-66	9.56E-64
COLEC10	-3.960302226	2.359672061	1.38E-66	1.23E-63
NDST3	-4.327716977	-1.757923699	1.57E-65	1.35E-62
DBH	-3.207724149	3.169431722	1.76E-65	1.46E-62
LIFR	-2.833569571	4.128856592	6.18E-65	4.94E-62
CETP	-2.823607461	3.204268457	1.12E-64	8.63E-62
PLVAP	2.914687883	6.459530208	1.22E-63	9.12E-61
TRIB1	-2.00351332	6.860251547	1.62E-62	1.17E-59
FOS	-3.10958211	6.900245909	9.37E-61	6.55E-58
AADAT	-2.794140341	3.584448019	7.91E-60	5.36E-57
COL6A6	-3.365140044	-1.178008819	3.08E-59	2.03E-56
EHD3	-2.168964033	2.822626494	6.31E-59	4.03E-56
EBF2	5.112343013	0.029766046	2.08E-58	1.29E-55
CPEB3	-2.259541094	3.866783087	2.30E-58	1.39E-55
CLEC1B	-5.231473106	1.599480629	2.65E-58	1.56E-55
PZP	-4.339437092	4.333728761	7.91E-58	4.54E-55
C21orf91	-1.693495338	2.808246857	2.08E-57	1.16E-54
UBE2T	3.202818076	2.893945291	7.48E-57	4.08E-54
CDC37L1	-1.584928145	4.902992692	8.70E-57	4.63E-54
PHLDA1	-2.465253997	6.526229987	6.05E-56	3.15E-53
ADRA2B	-2.23560979	1.335785851	1.00E-55	5.11E-53
C1RL	-1.653099269	7.164358681	1.23E-55	6.10E-53
DNASE1L3	-2.911524873	4.754898205	5.31E-55	2.59E-52
SLC26A6	2.642070157	4.338731715	6.94E-55	3.30E-52
CDKN3	3.901529846	2.884650843	3.96E-54	1.85E-51
ARHGEF39	2.934250836	2.126142692	5.90E-54	2.69E-51
MXD3	2.672113318	3.115045806	2.95E-53	1.32E-50
CXorf36	2.207948308	3.096506985	3.75E-53	1.64E-50
TUBE1	-1.813860819	3.518498971	1.22E-52	5.25E-50
GPR182	-3.397934343	0.511901649	1.43E-52	6.03E-50
CDC25C	4.328877226	1.973177273	1.92E-52	7.87E-50
CLEC4M	-5.596890211	2.119280736	1.93E-52	7.87E-50
PLSCR4	-1.929454379	4.369865263	2.92E-52	1.17E-49

LYVE1	-2.996162952	3.076923699	3.32E-52	1.30E-49
ZFP1	-1.70846279	2.968239752	4.17E-52	1.61E-49
PVALB	-3.663113767	-1.588606444	9.78E-52	3.71E-49
CXCL12	-2.69515322	6.038771171	4.61E-51	1.72E-48
EGR1	-2.602009865	7.268588566	5.26E-51	1.93E-48
EPHA2	-2.10199353	4.87201391	6.42E-51	2.32E-48
CDCA5	3.430879422	3.402449364	7.25E-51	2.57E-48
ETS2	-1.692222246	7.616584129	8.12E-51	2.84E-48
CENPF	3.890427776	4.309616199	9.39E-51	3.23E-48
HIGD1B	3.103577771	-0.007476405	1.71E-50	5.79E-48
NUF2	4.196180337	2.45023513	1.96E-50	6.54E-48
DACH1	-2.623416035	-0.384173694	2.59E-50	8.54E-48
TROAP	4.20571057	2.933063558	3.66E-50	1.19E-47
SKA1	4.444214903	2.138499191	3.78E-50	1.21E-47
KIFC1	3.763455194	3.674524847	7.34E-50	2.31E-47
KCNN2	-3.470205405	1.942981122	7.76E-50	2.41E-47
ACADS	-1.788870377	6.74431194	2.07E-49	6.34E-47
BCO2	-3.126095784	3.462702156	2.20E-49	6.64E-47
FBXO43	4.002297253	0.272910594	2.23E-49	6.64E-47
SPC24	3.469306426	3.279104056	3.52E-49	1.04E-46
DUSP6	-1.752497973	6.183282233	4.01E-49	1.16E-46
HJURP	3.886927713	2.751061611	5.15E-49	1.48E-46
TMEM26	-2.204641262	-0.119010578	5.41E-49	1.53E-46
CCBE1	-3.539055542	1.006994101	5.71E-49	1.60E-46
PRC1	3.227716644	3.951464961	8.55E-49	2.36E-46
KIF4A	3.911121104	3.220019859	1.60E-48	4.36E-46
MELK	3.790950691	2.543055761	1.64E-48	4.41E-46
CCNB2	3.722578322	3.083256723	1.70E-48	4.52E-46
MCL1	-1.265088077	8.351082955	2.48E-48	6.52E-46
SPRY2	-1.545435152	3.665805734	3.44E-48	8.95E-46
NEK2	3.935290721	2.762918243	4.25E-48	1.09E-45
CD4	-1.96638461	5.804357359	4.38E-48	1.11E-45
NRBF2	-1.113897466	4.494600528	6.54E-48	1.65E-45
LCAT	-2.577900383	6.170762118	8.53E-48	2.12E-45
CCNB1	3.268903819	4.18371021	1.01E-47	2.49E-45
NDC80	3.307570204	2.71548371	1.11E-47	2.69E-45
TCIM	-2.266309163	5.560928457	1.17E-47	2.82E-45
BIRC5	4.083148061	4.350888839	1.76E-47	4.18E-45
TOMM40L	1.725435431	4.884700557	2.09E-47	4.91E-45
DCUN1D3	-1.197761778	3.232282457	2.66E-47	6.19E-45
IL1RAP	-2.039431526	5.251851564	3.33E-47	7.67E-45
COL15A1	3.792555351	4.351255537	3.97E-47	9.05E-45
NCAPG	3.799833307	3.028462493	4.20E-47	9.43E-45
TBXA2R	-1.814139536	1.870404732	4.22E-47	9.43E-45
EXO1	3.597602151	1.997866891	5.02E-47	1.11E-44
KIF2C	3.927320027	3.068040898	1.09E-46	2.38E-44
SFTA1P	5.580914249	-1.812840263	1.24E-46	2.69E-44
CD34	2.092795004	5.220298078	1.26E-46	2.71E-44
FAM189B	1.671325648	4.686305726	1.84E-46	3.92E-44
ASPM	3.724380901	4.052493359	2.24E-46	4.72E-44
BUB1	3.500437108	2.742916192	5.41E-46	1.13E-43
CYP2C19	-3.694587771	1.327288498	5.48E-46	1.14E-43
PTTG1	3.719029618	3.836533948	9.07E-46	1.86E-43
S100A12	-2.975110641	-1.906699315	1.29E-45	2.63E-43
CDK1	3.486034406	3.65302642	1.38E-45	2.79E-43
TIMD4	-3.481505781	0.660887847	1.90E-45	3.80E-43
EGR2	-2.566513992	2.034900708	2.27E-45	4.49E-43
KIF18B	4.029888876	2.396541228	2.95E-45	5.80E-43

CYP26A1	-3.836614271	2.376722565	3.56E-45	6.89E-43
DLGAP5	3.699291626	2.335593351	3.57E-45	6.89E-43
FOSB	-3.248962452	5.300903778	3.83E-45	7.33E-43
KIF20A	3.711121242	3.101045683	4.11E-45	7.79E-43
CDCA3	3.286763701	2.670586024	4.72E-45	8.88E-43
CENPA	4.078243622	1.417957595	5.10E-45	9.50E-43
GHR	-2.470380115	6.204018834	5.33E-45	9.85E-43
SGMS2	-1.780408957	3.977123541	8.08E-45	1.48E-42
EZH2	2.404516632	3.361885861	8.38E-45	1.52E-42
RACGAP1	2.421285691	3.664460337	9.50E-45	1.71E-42
PNRC1	-1.45122583	6.640258987	1.03E-44	1.84E-42
CDC20	4.262950453	3.97822584	1.05E-44	1.86E-42
TOP2A	3.913729191	5.187779348	1.76E-44	3.10E-42
VPS45	1.118735997	4.651054362	3.80E-44	6.62E-42
IL1RL1	-3.380638779	0.731543049	3.82E-44	6.62E-42
CDCA8	3.16802918	2.830748877	4.51E-44	7.76E-42
PKMYT1	3.155220961	2.368622177	7.90E-44	1.35E-41
SKA3	3.539708316	1.854395224	8.31E-44	1.41E-41
CENPL	2.374927475	1.910606015	1.70E-43	2.86E-41
UBE2C	4.10218537	3.684436894	1.90E-43	3.17E-41
ETFDH	-1.672283823	5.922119093	1.91E-43	3.17E-41
CDH13	2.340914464	3.004873796	2.68E-43	4.40E-41
MSTO1	1.692095803	3.494776398	2.81E-43	4.58E-41
MRO	-2.340444406	1.529767015	2.92E-43	4.74E-41
FOXM1	3.612486641	3.835480327	3.18E-43	5.12E-41
AC092171.2	2.596432091	3.018486498	3.69E-43	5.90E-41
HMMR	3.237924186	2.894353193	5.88E-43	9.33E-41
TRAIP	2.802739882	1.696199647	6.05E-43	9.54E-41
GPM6A	-3.158324873	1.22592119	9.53E-43	1.49E-40
EME1	3.228459906	1.020553577	1.09E-42	1.69E-40
CHIC2	-1.001041785	2.926909021	1.47E-42	2.27E-40
ZIC2	6.646477117	2.271570847	1.50E-42	2.29E-40
PLK1	3.542849123	3.261025635	1.81E-42	2.76E-40
SPC25	3.128184847	1.436469893	1.92E-42	2.90E-40
AL391845.2	4.093942997	-2.291080989	2.54E-42	3.81E-40
CEP131	1.798712232	3.647507439	2.84E-42	4.23E-40
RNF152	-1.799113613	5.192862629	9.09E-42	1.35E-39
FLVCR1	2.199884371	3.679271456	9.73E-42	1.43E-39
C5orf34	2.391828888	0.469468302	1.02E-41	1.49E-39
SPATS2	1.571823604	3.851120567	1.05E-41	1.52E-39
PLPP3	-1.666360276	6.764789243	1.11E-41	1.60E-39
KDM8	-2.423715572	4.727775284	1.18E-41	1.69E-39
AC008556.1	-2.023342731	-0.693758158	1.21E-41	1.72E-39
AURKA	2.685523947	4.00485179	1.55E-41	2.20E-39
TTK	3.774642984	2.126555887	2.18E-41	3.06E-39
LILRA2	-1.973237195	0.280182971	2.34E-41	3.27E-39
SIGLEC11	-2.353951186	-0.025975551	2.91E-41	4.05E-39
E2F1	3.673389864	4.151997679	3.23E-41	4.46E-39
TRIP13	3.42303206	2.220491707	3.56E-41	4.89E-39
NAAA	-1.644548356	4.37571523	3.72E-41	5.07E-39
RRM2	3.07827395	4.59933559	4.94E-41	6.70E-39
TMEM120B	1.247105125	3.097097058	5.83E-41	7.86E-39
ANLN	3.644674436	3.314285097	7.04E-41	9.43E-39
LILRA5	-2.168482207	0.615041902	7.95E-41	1.06E-38
KIF19	-2.420974263	-0.883144607	1.02E-40	1.36E-38
CDT1	3.402024147	3.417394398	1.34E-40	1.77E-38
DTL	3.261776077	3.233781092	1.74E-40	2.27E-38
GTSE1	3.532104115	2.129075582	1.76E-40	2.29E-38

SIGLEC7	-2.049080275	0.527183715	1.77E-40	2.30E-38
MAP2K3	-1.273657703	5.892191331	1.87E-40	2.41E-38
C21orf62	-2.761158989	-2.737507742	2.05E-40	2.62E-38
CENPE	3.15180079	2.163073015	2.14E-40	2.72E-38
FRMD4B	-1.36124619	4.253059152	2.44E-40	3.08E-38
AURKB	3.553329822	2.828915208	2.56E-40	3.22E-38
MT2P1	-3.24485258	1.034749418	2.78E-40	3.47E-38
PLK3	-1.618232442	2.976687675	3.30E-40	4.11E-38
GSTZ1	-2.029403457	5.658842136	3.46E-40	4.28E-38
MKI67	3.359866257	4.723067222	4.40E-40	5.40E-38
LRRC4	-1.903800256	-0.287900518	5.15E-40	6.29E-38
TICRR	3.646089317	1.146884417	6.03E-40	7.29E-38
BUB1B	3.515602218	2.388134904	6.03E-40	7.29E-38
ZBTB21	-1.43554486	3.756862392	6.07E-40	7.30E-38
MYBL2	4.537449027	4.233700634	6.83E-40	8.18E-38
CDC6	3.203270181	3.478980096	7.61E-40	9.05E-38
LRRC14	1.497408677	4.623174493	9.87E-40	1.17E-37
SERTAD1	-1.576272993	4.100633372	1.06E-39	1.25E-37
SLC38A2	-1.631465862	7.894085372	1.11E-39	1.30E-37
IQGAP3	3.408138251	3.879209751	1.64E-39	1.92E-37
CD302	-1.688208647	4.8367851	1.75E-39	2.03E-37
CENPM	3.522665504	2.543379746	2.12E-39	2.44E-37
APLN	3.90345323	2.857253535	2.53E-39	2.90E-37
MND1	2.918969326	1.1567664	3.45E-39	3.94E-37
CAPN10-DT	2.324225495	0.072703297	3.52E-39	3.98E-37
ERLIN1	-1.292235277	6.127518307	4.39E-39	4.93E-37
ESM1	4.212404298	3.229090118	5.31E-39	5.94E-37
AC007906.2	-2.516928447	-0.941951837	5.62E-39	6.25E-37
CELSR3	3.492853434	2.557649963	5.70E-39	6.31E-37
CYR61	-2.009858989	5.934157091	6.48E-39	7.13E-37
SLC39A14	-1.649719722	8.469564161	6.50E-39	7.13E-37
SPDL1	1.808074984	2.506377624	9.59E-39	1.05E-36
RASGEF1B	-1.846377257	4.385809655	9.94E-39	1.08E-36
NCAPH	2.971508428	2.291962476	1.08E-38	1.16E-36
ADRA1A	-3.121654017	3.344723745	1.16E-38	1.24E-36
CDKN2A	4.032562376	3.764997736	1.41E-38	1.51E-36
PARPBP	2.716478425	1.522373127	1.43E-38	1.52E-36
ADGRG7	-2.68074622	2.423419496	1.70E-38	1.80E-36
CDC45	3.068149753	2.361189612	1.81E-38	1.91E-36
NOX4	2.825702746	0.298061527	1.94E-38	2.03E-36
LRRC55	-2.775485962	-0.949693641	1.97E-38	2.06E-36
ZC2HC1C	-1.416607241	1.985250871	2.08E-38	2.16E-36
FOXO1	-1.712197888	4.989559781	2.61E-38	2.70E-36
C1R	-1.782579433	10.34940988	2.98E-38	3.07E-36
CENPI	3.091751036	0.858345143	4.15E-38	4.26E-36
THY1	2.53572187	5.418816626	5.00E-38	5.11E-36
NEIL3	4.122296446	0.692649064	5.74E-38	5.83E-36
THBS4	5.771614626	4.10871723	6.10E-38	6.17E-36
MYD88	-1.043442216	5.528660324	6.78E-38	6.83E-36
MPC1	-1.540706654	6.392926294	7.13E-38	7.16E-36
DEPDC1	3.762327638	2.112302471	7.30E-38	7.29E-36
TBC1D16	1.703701409	5.287227345	7.36E-38	7.31E-36
SOCS3	-2.332909981	5.533623258	8.63E-38	8.54E-36
IGFBP3	-1.99656871	7.731067634	8.86E-38	8.74E-36
PANK1	-1.577153299	5.667277703	9.85E-38	9.67E-36
IL1RN	-2.149000338	6.048257843	1.01E-37	9.90E-36
TARBP1	1.794645449	4.734330055	1.13E-37	1.10E-35
PYGO2	1.214451335	5.519285322	1.29E-37	1.25E-35

CKAP2L	3.195682406	2.035249198	1.38E-37	1.33E-35
KIF23	3.297405629	2.449134911	1.56E-37	1.50E-35
LILRB5	-1.94571476	2.828138295	1.84E-37	1.76E-35
KIF14	3.104393206	1.959205903	1.85E-37	1.76E-35
TTC13	1.405753242	3.756515271	2.07E-37	1.96E-35
ERCC6L	3.184322599	0.410626112	2.12E-37	2.01E-35
TCF19	2.785270507	3.947651192	2.24E-37	2.10E-35
JRK	1.754733319	2.918144579	2.40E-37	2.25E-35
DSCC1	2.063267864	1.741907694	2.67E-37	2.49E-35
ZNF692	1.682206122	4.175412721	3.20E-37	2.96E-35
AC132192.2	2.39022501	-0.092531594	3.23E-37	2.98E-35
PBK	3.398155712	2.329374404	3.41E-37	3.13E-35
IGFALS	-3.195526355	5.589512723	3.44E-37	3.14E-35
MT1XP1	-3.10776891	-0.77682528	4.13E-37	3.76E-35
IER2	-1.3450004	6.059528732	4.60E-37	4.16E-35
SGO1	3.047773774	1.351579413	5.34E-37	4.82E-35
SOCS2	-2.179850483	4.447551088	6.08E-37	5.46E-35
APOF	-2.8720168	6.772005714	6.90E-37	6.18E-35
KLHL15	-1.351799272	3.620385931	7.09E-37	6.32E-35
KIF18A	3.341573296	1.180179653	8.92E-37	7.92E-35
TONSL	1.924072183	3.765286997	1.36E-36	1.20E-34
MS4A6A	-1.831008537	4.564168599	1.66E-36	1.46E-34
ASF1B	2.977289977	2.983303588	2.09E-36	1.83E-34
DIRAS3	-2.84601366	1.03852694	2.80E-36	2.45E-34
GMNN	2.187673837	5.072569252	2.92E-36	2.54E-34
ID2	-1.456356264	7.33272645	2.93E-36	2.54E-34
ALDH2	-1.674502183	9.779518157	3.20E-36	2.77E-34
AC004477.1	2.765856846	-0.853993301	3.26E-36	2.80E-34
LRRN3	-2.604735049	-1.193476534	3.46E-36	2.96E-34
ITGA9	-1.848708896	3.715722221	5.74E-36	4.90E-34
N4BP2L1	-1.448102897	4.66407139	5.88E-36	5.00E-34
GADD45B	-1.945354867	7.236468921	5.90E-36	5.00E-34
UCK2	1.643730532	4.773020752	6.33E-36	5.34E-34
GPC3	5.923086944	9.190017452	9.90E-36	8.33E-34
KAT2A	1.485961729	5.569488446	1.33E-35	1.11E-33
CCNF	2.180701617	2.860595901	1.42E-35	1.19E-33
ZFP41	1.923813481	3.17420154	1.79E-35	1.49E-33
ACADSB	-1.752197885	7.977199966	1.79E-35	1.49E-33
CD163	-1.995811399	5.049792217	1.87E-35	1.54E-33
FANCI	2.267942568	3.428349795	2.38E-35	1.96E-33
MTFR2	2.999241571	0.450123663	2.53E-35	2.07E-33
FPR2	-2.786272122	-1.416331021	2.70E-35	2.21E-33
E2F8	3.662634387	1.537416746	2.87E-35	2.33E-33
ORC1	2.913135139	1.745071046	3.03E-35	2.46E-33
NDRG2	-1.500175714	7.646350147	3.24E-35	2.61E-33
CCNA2	3.507763789	3.488607344	3.46E-35	2.78E-33
ANKRD52	1.426309336	4.778314201	4.32E-35	3.47E-33
OR51E1	3.012692039	-0.959363709	4.65E-35	3.71E-33
SOCS6	-1.071410012	4.794980083	4.66E-35	3.71E-33
RECQL4	2.667813205	4.182892091	5.01E-35	3.96E-33
SHCBP1	3.318245689	1.750416291	5.01E-35	3.96E-33
TEDC2	2.765751046	2.064848054	5.56E-35	4.38E-33
CDKN2B-AS1	3.5787177	-0.487369081	5.58E-35	4.38E-33
CCDC71L	-1.765672139	4.725484141	5.85E-35	4.58E-33
NXF3	-2.952296313	-1.70664579	6.33E-35	4.94E-33
RASD2	2.62848314	0.338841818	6.53E-35	5.07E-33
SLC35D1	-1.356411251	6.246536313	6.70E-35	5.19E-33
DDX11-AS1	2.786618572	-1.23352923	6.82E-35	5.26E-33

ARHGAP11A	2.543160339	2.793979067	6.86E-35	5.28E-33
PXDC1	-1.337875979	6.770272209	7.86E-35	6.03E-33
ZWINT	2.46189527	3.851004249	8.32E-35	6.36E-33
CCL14	-1.890014499	1.801242566	8.77E-35	6.68E-33
KIF15	3.358356865	1.6437308	9.22E-35	6.99E-33
XRCC2	2.892989914	1.065032173	9.30E-35	7.03E-33
BMP10	-4.929830589	-1.97289127	9.97E-35	7.51E-33
LINC01093	-3.39504491	2.916090518	1.09E-34	8.17E-33
STIL	2.384359636	1.939603425	1.31E-34	9.78E-33
ASS1P2	-3.440355338	-0.564375575	1.54E-34	1.15E-32
C21orf58	2.279061336	1.77298165	1.93E-34	1.43E-32
OLFML2B	3.161287378	3.108665788	2.36E-34	1.75E-32
CENPW	2.575095447	2.380395015	2.60E-34	1.91E-32
INMT	-2.484148596	3.611993241	2.63E-34	1.93E-32
ZFP36L1	-1.107838502	7.792708559	2.78E-34	2.04E-32
XRCC3	1.631402584	2.706546377	2.80E-34	2.04E-32
ACAA1	-1.534208056	7.765507669	2.96E-34	2.15E-32
MT-ATP6	-1.405559769	12.38635673	3.34E-34	2.42E-32
TNFRSF4	2.318766142	1.642037676	3.61E-34	2.61E-32
RFC4	1.730937752	3.738236344	3.88E-34	2.79E-32
SERPINB9	-1.604977628	3.624434587	4.15E-34	2.98E-32
ORC6	2.843257744	1.460186018	4.29E-34	3.06E-32
RNASEH2A	1.870093048	4.122494239	4.59E-34	3.27E-32
AGL	-1.496336384	5.228215308	5.47E-34	3.88E-32
SGO2	2.386058307	1.882552836	6.36E-34	4.51E-32
STMN1	2.109406321	6.037641045	7.17E-34	5.06E-32
CXCL14	-3.59864496	3.038782779	7.41E-34	5.22E-32
EHMT2	1.312882554	5.529153641	8.12E-34	5.70E-32
ZADH2	-1.054639332	4.555126409	8.55E-34	5.98E-32
USP21	1.164067827	3.767443001	8.69E-34	6.06E-32
CYP2C8	-2.678227447	8.759419192	9.32E-34	6.47E-32
E2F2	2.983501569	1.220639119	1.05E-33	7.29E-32
HAGLROS	4.815882957	-2.142635193	1.19E-33	8.24E-32
AF165147.1	-2.436384524	-1.914513082	1.26E-33	8.65E-32
ABCC10	1.358024133	3.602995249	1.40E-33	9.64E-32
BCKDHB	-1.510453501	4.991003382	1.42E-33	9.72E-32
WDR62	2.541659567	2.552200928	1.46E-33	9.92E-32
ZNF330	-1.012322508	4.422000942	1.46E-33	9.92E-32
CBFA2T3	-1.971248924	1.908583896	1.47E-33	9.95E-32
ZKSCAN2-DT	1.946032292	-0.01948757	1.92E-33	1.29E-31
FATE1	4.394118865	-1.259304399	1.92E-33	1.29E-31
GNE	-1.567875675	6.394413564	2.04E-33	1.37E-31
SMG5	1.338332328	6.917418078	2.58E-33	1.73E-31
DHODH	-1.775170852	5.935098225	3.05E-33	2.04E-31
MTHFD2L	-1.413669652	3.030855582	3.12E-33	2.07E-31
GINS1	2.728122389	2.897911258	3.90E-33	2.59E-31
POLQ	2.986329968	1.281303628	5.25E-33	3.47E-31
FXN	-1.197865996	3.66527936	5.35E-33	3.53E-31
MDK	3.798011257	7.152482736	6.03E-33	3.96E-31
JUN	-1.553173437	7.297780018	6.67E-33	4.36E-31
LDLR	-1.508544947	6.708087357	7.95E-33	5.18E-31
FENDRR	-2.791304915	-1.706755631	8.11E-33	5.27E-31
AC134043.2	3.932154798	-1.323846209	8.86E-33	5.73E-31
NCOR1	-1.143034388	5.797555564	9.28E-33	5.98E-31
FAHD2A	-1.154836256	5.002704377	9.73E-33	6.26E-31
NR4A1	-2.164417576	4.986941066	1.15E-32	7.39E-31
RASL12	2.215611013	1.595518588	1.21E-32	7.74E-31
FEN1	1.623539054	4.654987429	1.23E-32	7.85E-31

OLFML2A	2.400266291	3.189914981	1.33E-32	8.44E-31
KMT5C	1.490977315	2.906764085	1.42E-32	9.00E-31
LILRA1	-1.735687116	-0.49649095	1.42E-32	9.00E-31
AP5Z1	1.115615207	5.025178593	1.49E-32	9.36E-31
AC010719.1	2.829187521	0.380467232	1.50E-32	9.42E-31
P2RY13	-1.935456177	1.122933597	1.52E-32	9.53E-31
TBC1D31	1.383433632	2.388162246	1.54E-32	9.60E-31
NFIL3	-1.428051738	5.586983447	1.66E-32	1.03E-30
JUNB	-1.554062947	6.744910315	1.84E-32	1.15E-30
NUSAP1	2.263651832	4.252972276	2.51E-32	1.56E-30
HDAC11	1.74473613	4.342518727	2.75E-32	1.70E-30
AC138356.1	-2.301312491	-0.359527519	3.32E-32	2.05E-30
HOXA13	6.937204854	2.470393595	3.40E-32	2.09E-30
FAM83D	2.399008353	3.384503576	3.60E-32	2.21E-30
RAD54L	3.352040383	1.0671116	3.98E-32	2.43E-30
AC092119.2	2.402662006	-1.136196927	4.11E-32	2.51E-30
HIST2H2BC	2.133456674	2.327370785	4.67E-32	2.84E-30
CENPH	1.851118716	1.915795325	4.72E-32	2.86E-30
CDCA2	3.180552239	1.053990968	4.98E-32	3.00E-30
UHRF1	3.232967589	2.386845889	4.99E-32	3.00E-30
PLXDC1	2.97211641	2.720433524	5.64E-32	3.38E-30
CDKN2C	2.30581737	3.953270064	6.00E-32	3.59E-30
ANKRD55	-2.258920241	-0.387257192	6.03E-32	3.60E-30
SCAMP3	1.270561352	6.542349544	6.70E-32	3.99E-30
COX4I2	2.845311189	0.904677101	6.92E-32	4.10E-30
CEP250	1.084144664	4.42762511	6.93E-32	4.10E-30
RNF125	-1.879660019	3.606281738	7.01E-32	4.14E-30
NAT2	-2.944171574	3.5478492	7.72E-32	4.55E-30
PCLAF	2.464813962	2.95735616	7.88E-32	4.63E-30
TMEM206	1.32821613	1.930606785	9.31E-32	5.44E-30
PTGS2	-2.460660848	0.50749681	9.93E-32	5.78E-30
CENPK	2.639359395	1.201804346	1.13E-31	6.58E-30
P2RY12	-2.417567448	-1.454995733	1.14E-31	6.63E-30
NR2C2AP	1.318925484	3.871472486	1.21E-31	7.00E-30
ACAA2	-1.589878561	8.351972217	1.55E-31	8.93E-30
SLCO1C1	3.942202759	-1.821796758	1.65E-31	9.51E-30
ATF3	-1.750028706	5.47528364	1.67E-31	9.59E-30
HPS5	-1.524173701	5.043252251	1.82E-31	1.04E-29
EGR3	-2.147016417	0.675923716	1.98E-31	1.13E-29
HAGLR	6.211087784	2.403614355	2.04E-31	1.16E-29
TERT	8.677196916	2.464243848	2.13E-31	1.21E-29
PIF1	2.737265427	1.272561389	2.20E-31	1.25E-29
KIAA1841	1.543561428	1.845836697	2.33E-31	1.32E-29
LINC00907	-2.893953156	-1.309924142	2.38E-31	1.34E-29
CEP55	3.046907861	1.765824894	2.64E-31	1.49E-29
FAM72D	3.538574261	-1.412730763	2.69E-31	1.51E-29
SYNE1	-1.307546409	4.552642203	3.23E-31	1.81E-29
SEMA5B	2.297139757	1.961257774	4.04E-31	2.25E-29
MTBP	1.859581396	1.158117977	4.46E-31	2.48E-29
DNAJC25	-1.252424607	4.371505903	5.42E-31	3.01E-29
DBF4B	1.558200046	2.003857242	6.04E-31	3.34E-29
RIPK4	-1.384649355	4.562949329	6.10E-31	3.36E-29
KCTD9P4	-2.928040496	-2.657125445	6.10E-31	3.36E-29
FERMT2	-1.078692569	5.743424446	6.87E-31	3.78E-29
ACSM3	-2.03882602	4.991843993	6.93E-31	3.80E-29
PTP4A2P2	2.818364215	-2.637626566	6.94E-31	3.80E-29
KHDC4	1.299366631	4.519222628	6.97E-31	3.80E-29
E2F7	3.484654721	0.74600369	7.30E-31	3.98E-29

PPOX	1.15500702	4.055619626	7.69E-31	4.18E-29
TACC3	2.10596896	4.579944535	8.20E-31	4.44E-29
USP49	1.269855149	1.938355108	8.48E-31	4.58E-29
SEMA3F	1.27207077	4.195340573	8.64E-31	4.66E-29
AL359715.3	-1.551457381	0.24907575	9.07E-31	4.88E-29
RDM1	3.424687445	-0.361943005	9.12E-31	4.89E-29
NMRAL2P	5.991208536	2.277794588	9.45E-31	5.06E-29
SPRYD4	-1.31686107	5.914215743	9.99E-31	5.32E-29
ACAT1	-1.399133777	8.276099948	1.01E-30	5.38E-29
MMAA	-1.181607931	4.176530055	1.10E-30	5.81E-29
MBNL2	-1.374821087	5.766586745	1.10E-30	5.82E-29
FANCD2	2.205647689	2.683451438	1.14E-30	6.03E-29
AL606489.1	3.076935989	-1.54391163	1.18E-30	6.23E-29
MESP2	3.359132286	0.214808969	1.19E-30	6.24E-29
OTX1	4.363341241	0.191845979	1.25E-30	6.53E-29
IVD	-1.214853319	7.383613731	1.27E-30	6.64E-29
LARP1B	-1.102185188	4.734590916	1.29E-30	6.75E-29
MCM10	3.032036263	1.699862372	1.39E-30	7.24E-29
PEA15	1.167783941	6.651726068	1.56E-30	8.12E-29
TK1	2.326590173	4.919286382	1.63E-30	8.43E-29
MCM2	2.392992551	4.813353308	1.64E-30	8.49E-29
EPHB1	-2.061646804	0.046912319	1.84E-30	9.47E-29
ECT2	2.397862478	3.458413293	1.89E-30	9.71E-29
C1orf35	1.298818541	4.087829324	1.92E-30	9.87E-29
GJC1	2.350237592	1.990541988	2.02E-30	1.03E-28
MSH2	1.292211335	3.842356454	2.23E-30	1.14E-28
DENND4B	1.04974288	5.029267707	2.26E-30	1.15E-28
NPY1R	-2.393552916	1.840758114	2.63E-30	1.34E-28
DIAPH3	3.687524988	1.878187771	3.41E-30	1.73E-28
MAD2L1	2.169689247	2.80273169	3.73E-30	1.89E-28
MMP11	3.776793493	3.938812741	3.87E-30	1.95E-28
SCX	3.236499478	-0.108994271	4.47E-30	2.25E-28
CAP2	2.285302338	4.729029072	4.74E-30	2.38E-28
CHAF1A	1.461349795	3.887962779	4.97E-30	2.49E-28
PGM1	-1.245074743	7.45806952	5.11E-30	2.55E-28
HAMP	-3.615381632	6.989144009	5.14E-30	2.56E-28
ZNF517	1.72518026	3.794098931	5.32E-30	2.64E-28
NDUFA4L2	2.849782012	4.558946405	6.04E-30	3.00E-28
4-Mar	3.649548373	-2.290102143	6.54E-30	3.23E-28
MSRA	-1.393721208	4.898427607	6.54E-30	3.23E-28
MUT	-1.332490413	6.890556229	6.84E-30	3.37E-28
CYP2B6	-2.626474612	7.44782409	7.23E-30	3.56E-28
MCM6	1.738102391	4.729678527	7.40E-30	3.63E-28
RAD51	2.320847702	1.496064537	7.46E-30	3.65E-28
CHTF18	1.700452145	3.167118068	7.82E-30	3.81E-28
KIF11	2.201384545	2.826904768	7.82E-30	3.81E-28
PIGC	1.06092064	4.819086978	7.86E-30	3.82E-28
SDC4	-1.287025213	8.690513995	8.28E-30	4.02E-28
CDH24	2.181393864	1.539126655	8.84E-30	4.28E-28
ANTXR2	-1.26242288	4.907048995	9.13E-30	4.41E-28
IQCC	1.477067717	0.889612858	9.25E-30	4.46E-28
DHX34	1.145250694	4.384013611	1.04E-29	5.00E-28
KLF10	-1.446993983	5.753837381	1.08E-29	5.18E-28
MAT1A	-1.808594934	9.961287836	1.10E-29	5.27E-28
ALDH6A1	-1.744896512	7.910229895	1.12E-29	5.34E-28
SNHG1	1.584892113	4.167391133	1.15E-29	5.49E-28
CHRM2	-3.781975935	-1.659556974	1.16E-29	5.53E-28
TRIM11	1.119071395	4.219593851	1.19E-29	5.66E-28

PBLD	-1.761552993	6.738951733	1.20E-29	5.67E-28
AC005332.5	1.598902648	1.990807773	1.37E-29	6.46E-28
CBFA2T2	1.188396429	3.993565719	1.42E-29	6.72E-28
LPA	-2.340531195	5.13468301	1.44E-29	6.78E-28
ACSL1	-1.825483494	9.282509424	1.52E-29	7.11E-28
CSPG4	2.115277721	3.31835508	1.56E-29	7.30E-28
RUSC1	1.376234529	4.242903051	1.73E-29	8.10E-28
TMEM79	1.092901933	2.503112853	1.83E-29	8.55E-28
AL162431.2	2.360184934	-0.556422777	1.94E-29	9.01E-28
CAT	-1.425216055	8.579994649	2.01E-29	9.34E-28
CYP4A11	-2.239439019	8.690138961	2.06E-29	9.52E-28
SERPINE1	-2.218331342	7.51788261	2.07E-29	9.58E-28
C17orf82	2.96079973	-0.821836088	2.14E-29	9.88E-28
CLTRN	-2.45922944	2.795627021	2.22E-29	1.02E-27
HOTTIP	7.195629067	0.397647083	2.32E-29	1.06E-27
NAT1	-1.295973342	2.291120039	2.50E-29	1.14E-27
AL353708.3	2.447681179	-1.450912162	2.62E-29	1.20E-27
TIGD5	1.582048753	3.567896489	2.64E-29	1.20E-27
FANCG	1.464136939	3.135876921	2.70E-29	1.22E-27
TRIM45	2.274292146	1.780074624	3.14E-29	1.42E-27
PDLIM5	-1.082749185	6.360045129	3.27E-29	1.47E-27
PRIM2	1.166183349	2.824334844	3.54E-29	1.59E-27
NR4A3	-2.217160034	2.19911881	3.58E-29	1.61E-27
MCM3	1.53691446	5.918883416	4.52E-29	2.03E-27
HASPIN	2.716052245	-0.471983251	4.98E-29	2.23E-27
AC092171.4	2.496250975	-1.852697399	5.00E-29	2.23E-27
IRF8	-1.574263731	4.112395407	5.14E-29	2.29E-27
PITPNM3	-2.532336034	0.734138791	5.40E-29	2.40E-27
CD5L	-3.035829803	4.380442327	5.56E-29	2.47E-27
CAPN11	2.076562762	-0.811999771	5.75E-29	2.55E-27
TAPT1	-1.024899793	5.025582939	5.76E-29	2.55E-27
DDX12P	2.017429529	0.383149333	6.02E-29	2.65E-27
SOCS7	1.516527763	2.989441206	6.71E-29	2.94E-27
UBAP2L	1.001863222	6.543734189	6.84E-29	2.99E-27
CHAF1B	2.573559872	2.443693408	6.93E-29	3.02E-27
SCIMP	-1.672395558	1.390835115	7.21E-29	3.14E-27
MLYCD	-1.265494691	4.835919939	7.31E-29	3.17E-27
GNPNAT1	-1.0907947	5.343085215	8.13E-29	3.52E-27
DDX39A	1.457722259	5.596522456	8.34E-29	3.60E-27
TGM3	5.350216022	4.124414147	8.48E-29	3.66E-27
KLRF1	-1.80704542	-1.295289376	1.00E-28	4.30E-27
GNA14	-1.774675897	1.233206192	1.01E-28	4.31E-27
SOWAHC	-1.173738073	5.299602453	1.06E-28	4.52E-27
MT-ND4	-1.296298794	13.74235466	1.09E-28	4.65E-27
MAPT	2.990782888	2.723504866	1.12E-28	4.78E-27
RFX8	2.735683171	-1.253663795	1.25E-28	5.30E-27
CENPU	2.190429889	3.063420128	1.33E-28	5.63E-27
LINC01089	1.677509981	2.092828614	1.36E-28	5.76E-27
CNDP1	-3.274317985	3.934041304	1.37E-28	5.79E-27
MSTO2P	1.962143048	0.446721476	1.44E-28	6.08E-27
CPED1	-2.015230152	3.966661567	1.46E-28	6.13E-27
PRR11	2.493029005	2.369145189	1.50E-28	6.28E-27
DBT	-1.10384465	5.114350653	1.65E-28	6.91E-27
CAMK4	-1.727822425	0.496742537	1.66E-28	6.94E-27
MASP1	-1.60291241	6.946312904	1.67E-28	6.95E-27
ADCY6	1.172163998	4.726836831	1.76E-28	7.31E-27
GDF2	-4.785652483	1.496209684	2.07E-28	8.56E-27
SIX1	4.841789383	0.045215884	2.15E-28	8.87E-27

CAD	1.272153659	4.843325393	2.27E-28	9.37E-27
SCGB3A1	-2.195086531	-0.957017619	2.30E-28	9.49E-27
FITM1	-2.075781878	1.351384656	2.32E-28	9.54E-27
CMTM6	-1.034721505	6.337216693	2.38E-28	9.77E-27
CTBS	-1.063436679	4.616362459	2.62E-28	1.07E-26
GBA	1.369444218	6.136778436	2.72E-28	1.11E-26
MMRN1	-1.959030594	1.815626699	2.82E-28	1.15E-26
SREBF2-AS1	1.597813349	1.202416969	3.27E-28	1.33E-26
COL25A1	-2.647442643	-1.220217452	3.35E-28	1.36E-26
AL121906.2	2.810007446	-1.211850284	4.22E-28	1.71E-26
B4GALNT1	4.209876814	2.73093338	4.23E-28	1.71E-26
SPSB2	1.497670063	3.105741976	4.25E-28	1.72E-26
NXPH4	5.630668269	2.367137103	4.25E-28	1.72E-26
GNAZ	3.621037023	3.337767611	4.27E-28	1.72E-26
CCHCR1	1.408220489	4.774340009	4.30E-28	1.73E-26
LINC01311	2.141284716	-0.791388365	4.69E-28	1.89E-26
ZNF251	1.253517039	3.48355724	4.80E-28	1.92E-26
PLIN2	-1.664653141	8.533936223	4.90E-28	1.96E-26
ALDH8A1	-1.914427229	6.627365732	5.39E-28	2.15E-26
HELLS	2.433744897	2.7088137	6.19E-28	2.47E-26
ALB	-1.833471096	15.92169367	6.34E-28	2.52E-26
CCT3	1.163480202	8.267092326	7.78E-28	3.09E-26
NSMCE2	1.429661763	4.30182289	7.96E-28	3.15E-26
FAM13A	-1.877650345	4.466328039	7.97E-28	3.15E-26
DUSP1	-1.673787388	7.95713914	8.18E-28	3.24E-26
STEAP3	-1.714314304	6.957479018	8.47E-28	3.34E-26
PLK4	2.133760411	1.252838148	8.88E-28	3.50E-26
RTL10	1.119709558	3.642489595	9.15E-28	3.60E-26
RAD54B	1.621920026	0.624688368	9.24E-28	3.63E-26
HMGCL	-1.293444287	7.342020113	9.38E-28	3.67E-26
KDM6B	-1.036484918	4.551222124	9.45E-28	3.70E-26
WDR76	2.237552552	2.487882591	9.52E-28	3.72E-26
VPS72	1.064724688	5.121766771	1.08E-27	4.23E-26
WDHD1	1.996527392	1.890690238	1.09E-27	4.24E-26
BCAN	4.581980481	1.50631714	1.10E-27	4.26E-26
FAAP24	1.444174569	1.141859394	1.12E-27	4.33E-26
AL355488.1	1.923673157	1.139296668	1.17E-27	4.53E-26
CYP4V2	-1.434814852	6.593827268	1.18E-27	4.54E-26
SAPCD1	2.802350595	-1.220517671	1.18E-27	4.54E-26
ZCCHC24	-1.095954166	5.254592488	1.19E-27	4.58E-26
DLC1	-1.265059409	5.064262414	1.29E-27	4.96E-26
EPB41L4B	-1.570681775	5.795711432	1.39E-27	5.32E-26
CUL7	1.154561444	5.089548491	1.57E-27	6.00E-26
MYLK-AS1	2.144483802	-0.753479632	1.61E-27	6.18E-26
IL6ST	-1.006779986	7.675131356	1.65E-27	6.30E-26
AC026369.3	-2.607910309	-2.835450658	1.66E-27	6.32E-26
COG2	1.012973051	4.4236336	1.75E-27	6.65E-26
NAT9	1.218483128	4.600624489	1.86E-27	7.05E-26
PLPBP	-1.088684044	5.483962271	1.98E-27	7.48E-26
TP73	3.424680839	1.48755946	1.98E-27	7.48E-26
GPRIN1	2.59553947	1.793654811	2.16E-27	8.16E-26
SLC9B2	-1.43881078	4.708638282	2.25E-27	8.50E-26
TENT5A	-1.419280102	4.78993585	2.32E-27	8.75E-26
TRPC6	2.084331318	-0.326015907	2.34E-27	8.81E-26
DEPDC1B	3.200245596	2.207596653	2.36E-27	8.86E-26
SCNM1	1.379086988	4.519645338	2.42E-27	9.06E-26
EFNA4	1.549157619	3.125804075	2.56E-27	9.57E-26
ESR1	-2.476653657	3.581503391	2.59E-27	9.68E-26

KCTD7	1.309256819	2.475901272	2.60E-27	9.69E-26
ACBD6	1.079017516	4.759808888	2.74E-27	1.02E-25
COL9A1	3.793055709	-0.839827349	3.12E-27	1.16E-25
AQP3	-1.555160377	5.978228296	3.38E-27	1.25E-25
AJM1	1.871852965	2.185237962	3.44E-27	1.27E-25
PPP1R3B	-1.661912359	6.536686339	3.63E-27	1.34E-25
CDC7	2.058151959	1.884865224	3.72E-27	1.37E-25
ZIC5	6.578038686	0.843699666	3.76E-27	1.38E-25
GLI4	1.551385428	3.71168847	3.77E-27	1.38E-25
ARL5B	-1.046712089	5.488802031	3.83E-27	1.40E-25
AC016773.1	2.526241184	-1.333779856	3.91E-27	1.43E-25
AL357055.3	-1.48048433	-1.508308437	4.48E-27	1.64E-25
TSC22D2	-1.000734239	4.59241176	4.75E-27	1.73E-25
OIP5	2.239647101	0.898763249	4.89E-27	1.78E-25
RHOB	-1.468233128	9.123699717	4.91E-27	1.78E-25
RBMS3	-1.534260967	1.980241027	5.23E-27	1.90E-25
MAFG-DT	2.921125512	1.155366809	5.27E-27	1.91E-25
HCG25	1.722278164	-0.934453117	5.39E-27	1.95E-25
BLM	2.450063338	1.538847387	5.53E-27	2.00E-25
TIGD1	1.785513814	1.403909225	5.54E-27	2.00E-25
CLK2	1.037685945	4.76535222	5.65E-27	2.03E-25
MYO19	1.205944418	4.640458839	5.96E-27	2.14E-25
RAD51AP1	2.274676366	1.769739032	5.97E-27	2.14E-25
AC102953.2	2.049527057	0.34333244	6.14E-27	2.20E-25
IGF2BP1	6.982597853	3.657065272	6.16E-27	2.20E-25
PAMR1	-1.953923056	2.116563736	6.49E-27	2.32E-25
CBX2	2.690237296	2.169440744	6.95E-27	2.47E-25
NSUN5P1	1.548423361	2.752326958	7.55E-27	2.68E-25
TLR4	-1.48539071	2.994531547	7.57E-27	2.68E-25
NELFE	1.345728901	5.970124223	7.87E-27	2.78E-25
KLF11	-1.227232211	4.947977924	8.17E-27	2.88E-25
NSUN5P2	1.651940481	-0.038338147	8.26E-27	2.91E-25
POLD1	1.271708693	4.209719649	8.31E-27	2.92E-25
DUSP9	5.004351893	4.498267789	8.50E-27	2.98E-25
TMEM201	1.304723867	3.199756865	8.84E-27	3.09E-25
BDH2	-1.317906143	4.61408959	9.91E-27	3.46E-25
MT2A	-2.461577884	9.569222447	1.14E-26	3.96E-25
FBP1	-2.032292995	8.349199418	1.20E-26	4.18E-25
DSN1	1.264421497	3.91571325	1.24E-26	4.32E-25
LINC02475	6.398029811	-0.839951118	1.27E-26	4.39E-25
AL137060.1	2.643356763	-2.271400042	1.33E-26	4.59E-25
HOXD9	4.582915391	0.778759019	1.35E-26	4.67E-25
NUDT10	-2.966765654	-2.084874988	1.36E-26	4.70E-25
TAZ	1.051831327	4.499473061	1.43E-26	4.93E-25
NSUN6	-1.131956838	4.531733479	1.43E-26	4.94E-25
NUDT17	1.819765799	0.713572176	1.45E-26	5.00E-25
PIGU	1.113736967	4.586560671	1.60E-26	5.49E-25
ZNF219	1.279433769	4.615113734	1.70E-26	5.81E-25
KLF6	-1.340089574	7.055440718	1.71E-26	5.84E-25
UBE2S	1.953611602	3.784939639	1.83E-26	6.25E-25
AC124944.3	2.146288335	-1.070498823	1.86E-26	6.34E-25
CD160	-1.683651254	-0.685329493	2.22E-26	7.55E-25
KIAA1522	1.501748716	5.997414228	2.35E-26	7.96E-25
HIVEP1	-1.155305999	4.03062847	2.42E-26	8.19E-25
CXCR2	-2.277554498	-1.160048286	2.63E-26	8.89E-25
KBTBD11	-2.343790731	2.864937714	2.88E-26	9.72E-25
ADK	-1.120759125	5.617219623	3.01E-26	1.02E-24
PRAM1	-1.552626115	0.322216844	3.11E-26	1.05E-24

AL359510.2	3.089750585	-2.095597882	3.13E-26	1.05E-24
ST8SIA6	-2.442337282	-2.152248169	3.16E-26	1.06E-24
FAM111B	2.608976944	2.674371628	3.26E-26	1.09E-24
SNORD104	2.729652254	0.956777862	3.52E-26	1.18E-24
AC055713.1	1.328855362	0.768702231	3.54E-26	1.19E-24
AC145207.9	2.514669953	-1.509399181	3.83E-26	1.28E-24
ABCC5	1.081574224	3.775272542	4.01E-26	1.34E-24
FAM57B	3.89942742	-1.907993529	4.21E-26	1.40E-24
PLAC8	-2.3190054	1.562623989	4.27E-26	1.42E-24
SLC31A1	-1.046528358	6.757354069	4.46E-26	1.48E-24
TMEM220	-1.493612812	5.41773326	4.73E-26	1.57E-24
AL023803.2	3.013251067	-1.606462272	4.87E-26	1.61E-24
RUSC1-AS1	1.826959059	2.242074553	5.06E-26	1.67E-24
AC006277.1	1.994593095	-1.475369157	5.27E-26	1.74E-24
ASS1P1	-1.867587546	0.363304744	5.51E-26	1.81E-24
VMO1	-1.425251448	1.64251352	5.66E-26	1.86E-24
EPS8L3	4.919206572	3.930463394	5.94E-26	1.95E-24
CRNDE	3.374615908	1.570514602	6.31E-26	2.07E-24
VSIG4	-1.916324115	3.329103581	6.59E-26	2.15E-24
NOCT	-1.775256334	2.75180333	7.02E-26	2.29E-24
POLA2	1.278627672	3.607903852	7.31E-26	2.38E-24
HHIP	-3.317505243	2.011783323	7.72E-26	2.50E-24
KIFC2	1.947383806	3.422683163	8.14E-26	2.63E-24
CBR4	-1.176620971	4.923788483	8.30E-26	2.68E-24
MSH5	2.246450024	0.923592207	8.36E-26	2.70E-24
TJP2	-1.04872092	5.921016767	8.46E-26	2.73E-24
PDE7B	-1.683748067	1.665605415	8.60E-26	2.77E-24
GABRQ	4.478637636	-1.462604942	8.91E-26	2.86E-24
ROBO1	2.905092146	5.872175144	9.19E-26	2.95E-24
PALM2	-1.804749228	0.298853131	9.97E-26	3.20E-24
RHNO1	1.147698531	3.355495216	1.02E-25	3.26E-24
MTATP6P1	-1.369389293	9.422161088	1.02E-25	3.26E-24
TMEM106C	1.484590682	5.839942628	1.07E-25	3.42E-24
EDNRB	-1.438046254	4.514721158	1.08E-25	3.44E-24
LHX4	2.5190577	-0.315158931	1.09E-25	3.48E-24
NECAB3	1.472202573	5.280610362	1.13E-25	3.60E-24
BHLHE40	-1.389790108	7.237150398	1.17E-25	3.73E-24
ID1	-1.836401556	4.905075275	1.19E-25	3.78E-24
JDP2	-1.200642175	3.408173986	1.25E-25	3.94E-24
SF3B4	1.053280792	6.089209505	1.31E-25	4.13E-24
AC091057.1	2.122397342	0.680289147	1.32E-25	4.16E-24
RXFP1	-1.789035544	-1.07203856	1.35E-25	4.24E-24
AC010973.2	1.806217659	-0.606748772	1.36E-25	4.27E-24
ACAD11	-1.71289835	1.138541979	1.53E-25	4.81E-24
CCNE1	4.007663806	2.739309518	1.54E-25	4.81E-24
LINC02163	7.276802801	-1.008934399	1.54E-25	4.81E-24
C20orf204	5.068232091	2.390937092	1.66E-25	5.19E-24
ECHS1	-1.22041733	9.138562483	1.74E-25	5.42E-24
LENG8-AS1	1.506991195	-0.145393653	1.79E-25	5.58E-24
HAUS5	1.005653418	3.606135686	1.83E-25	5.70E-24
MCC	-1.738482654	3.985197487	1.84E-25	5.72E-24
TCOF1	1.053253817	5.091285987	1.85E-25	5.75E-24
FBXL18	1.361738513	1.596669208	1.94E-25	6.01E-24
ZNF252P-AS1	2.120791376	-1.582742482	2.03E-25	6.30E-24
CYP4A22	-2.169031141	6.792292511	2.53E-25	7.82E-24
CD300E	-1.715023948	0.076680103	2.62E-25	8.07E-24
NT5DC2	2.636809691	4.463467384	2.62E-25	8.07E-24
CHEK1	1.65581539	2.767899536	2.76E-25	8.49E-24

CTHRC1	3.977280438	2.701168238	2.79E-25	8.56E-24
DUXAP10	3.915105882	-1.625760313	2.86E-25	8.79E-24
SLC22A10	-2.316187443	4.822793219	2.88E-25	8.82E-24
ASPA	-1.926494531	1.901519416	2.88E-25	8.83E-24
AL133367.1	2.130826923	-0.485512057	3.06E-25	9.35E-24
SCRIB	1.309698636	6.413755105	3.07E-25	9.37E-24
PTPN3	-1.021812195	5.460612791	3.29E-25	1.00E-23
NUDT1	1.794067132	3.175359877	3.37E-25	1.02E-23
NOL12	1.14917254	1.754789283	3.37E-25	1.02E-23
PTGES2-AS1	3.451156335	-2.313543178	3.40E-25	1.03E-23
MAST2	1.057607899	4.675202814	3.41E-25	1.03E-23
C17orf53	1.918934335	1.41578469	3.56E-25	1.08E-23
ASS1	-1.761020256	9.976902604	4.75E-25	1.43E-23
PROS1	-1.219164169	7.550943317	4.90E-25	1.48E-23
ASS1P11	-2.747291388	-1.773334272	5.07E-25	1.52E-23
CXCR1	-2.269997092	-1.113892422	5.42E-25	1.63E-23
RNFT2	2.683140954	0.762969626	5.44E-25	1.63E-23
FOXK1	1.217687391	4.15864955	5.99E-25	1.80E-23
DUXAP9	3.707845601	-1.713685552	6.11E-25	1.83E-23
EMC3-AS1	2.205474564	-0.148615316	6.16E-25	1.84E-23
SPSB4	-2.681821428	-2.266864541	6.23E-25	1.86E-23
DNAH14	1.265629179	2.558757068	7.46E-25	2.22E-23
AHSA2P	1.35621216	4.205246318	7.53E-25	2.24E-23
TEX19	4.658520825	-2.075475118	8.01E-25	2.37E-23
ETFRF1	-1.03249085	4.665306849	8.24E-25	2.44E-23
C8orf33	1.270044236	5.567110061	8.52E-25	2.52E-23
CPT2	-1.191352315	5.980389988	9.14E-25	2.70E-23
RMDN2	-1.150538627	3.322943794	9.61E-25	2.82E-23
KNTC1	1.928989012	3.417820938	1.01E-24	2.96E-23
SMPD3	-1.93774364	1.38408042	1.07E-24	3.14E-23
AC107375.1	1.492767524	0.448701239	1.08E-24	3.16E-23
TRIM71	8.471907651	2.823535489	1.10E-24	3.20E-23
MCM4	1.610093434	5.34470181	1.13E-24	3.28E-23
WDR66	-1.603369392	0.073322821	1.22E-24	3.54E-23
EPHX2	-1.518667615	7.019925749	1.34E-24	3.89E-23
LY6H	4.253552758	-0.798866345	1.34E-24	3.89E-23
LOXL2	1.62609723	3.696876972	1.38E-24	4.01E-23
FOXS1	2.827667297	1.072447003	1.39E-24	4.01E-23
TSLP	-2.349602605	1.335812863	1.43E-24	4.12E-23
SARDH	-1.489012882	6.83891499	1.46E-24	4.21E-23
TRAF2	1.154698691	4.573018277	1.49E-24	4.31E-23
SIGLEC16	-1.670212812	-0.753004506	1.60E-24	4.60E-23
DNAJC6	2.582650314	2.071433929	1.66E-24	4.76E-23
LAMA4	1.516172064	4.449196481	1.90E-24	5.45E-23
GLYATL1	-2.05535975	6.736745207	1.92E-24	5.48E-23
PIM1	-1.387295878	5.375665695	2.36E-24	6.76E-23
AC129507.1	-1.793404706	-1.526697212	2.42E-24	6.90E-23
DTYMK	1.307522664	4.531407929	2.48E-24	7.08E-23
SPATC1L	2.890862386	2.273460251	2.49E-24	7.10E-23
AC018809.1	2.516418136	-2.334367646	2.54E-24	7.22E-23
TPX2	2.106956461	4.640439804	2.60E-24	7.37E-23
HOXD8	3.079527648	-0.130621203	2.60E-24	7.38E-23
AMHR2	-2.990215633	-2.096013196	2.67E-24	7.57E-23
TPPP2	-2.127596767	0.873557058	2.73E-24	7.71E-23
CYP39A1	-2.434561264	4.439727784	2.79E-24	7.88E-23
ZNF775	1.661786454	3.974429469	2.91E-24	8.22E-23
NOTCH3	2.049126731	5.291662312	3.09E-24	8.71E-23
KPNA2	1.422211539	5.6524885	3.25E-24	9.14E-23

RCL1	-1.4606037	5.166012086	3.46E-24	9.73E-23
AC010280.2	-2.576088962	-0.688839915	3.48E-24	9.77E-23
MT1F	-2.928239439	5.918781953	3.75E-24	1.05E-22
SMG9	1.16072013	4.256861755	3.95E-24	1.11E-22
ZNF696	1.203065349	2.749377092	4.52E-24	1.26E-22
IRAK1	1.298561989	6.694446933	4.63E-24	1.29E-22
ASB16	2.07320943	-0.300097086	4.73E-24	1.32E-22
MT-CYB	-1.236345967	12.94977543	4.74E-24	1.32E-22
CCDC34	1.698114091	3.32158089	4.79E-24	1.33E-22
BACE1-AS	1.251895763	0.883281928	4.93E-24	1.37E-22
TSSK6	1.395469876	0.805303351	4.97E-24	1.38E-22
SLC41A2	-1.371505514	5.678917048	5.27E-24	1.46E-22
ADAM15	1.317453	6.190673077	5.53E-24	1.53E-22
ERFE	2.644292393	0.370524015	5.56E-24	1.53E-22
AC110285.6	3.571049281	-1.864205341	5.78E-24	1.59E-22
CCDC25	-1.003051909	4.974681465	5.89E-24	1.62E-22
C8A	-1.832836899	8.052133706	6.09E-24	1.67E-22
KLKB1	-1.630509139	6.667385575	6.12E-24	1.68E-22
LIN9	1.555024763	1.869184361	6.16E-24	1.69E-22
MCM7	1.468490965	6.212911876	6.34E-24	1.74E-22
GRAMD1C	-1.587304775	3.424624369	6.48E-24	1.77E-22
ELMSAN1	-1.021431185	4.053330105	6.62E-24	1.81E-22
FLAD1	1.01487061	5.584949306	6.99E-24	1.91E-22
AC004080.2	6.566462756	-1.863049175	6.99E-24	1.91E-22
COA6	1.268801623	4.88435602	7.44E-24	2.03E-22
CKS1B	1.22905065	4.427746361	7.94E-24	2.16E-22
TMC7	2.156272788	1.015099749	7.99E-24	2.17E-22
ZBTB12	1.896960617	1.483878109	8.91E-24	2.41E-22
ARHGAP33	1.553675586	2.356528136	8.94E-24	2.42E-22
TTC36	-2.773994409	4.523857208	9.22E-24	2.49E-22
PDLIM1P4	-1.669173784	-1.766657137	9.33E-24	2.52E-22
PDE2A	-1.499535143	3.248863892	9.52E-24	2.57E-22
WEE1	-1.173326602	5.035054017	9.78E-24	2.63E-22
MS4A7	-1.496961814	3.915405561	1.01E-23	2.73E-22
MT-ND1	-1.166441907	12.28197047	1.08E-23	2.91E-22
FANCE	1.659039715	1.730287945	1.09E-23	2.93E-22
YEATS2	1.043826853	3.793898169	1.12E-23	2.99E-22
EPM2A	-1.019726701	2.357902253	1.14E-23	3.05E-22
CFI	-1.372669803	8.659528387	1.15E-23	3.08E-22
MYO1B	-1.153341853	7.879101513	1.30E-23	3.45E-22
MRPL53	1.00040122	1.392913033	1.30E-23	3.45E-22
GCH1	-1.522449449	5.417816443	1.30E-23	3.46E-22
TRIM65	1.044793176	3.696787014	1.34E-23	3.56E-22
FAM72C	3.108228984	-1.886590206	1.38E-23	3.67E-22
HSF4	1.79460024	3.118134278	1.41E-23	3.72E-22
OSBPL3	1.762821953	2.879667391	1.44E-23	3.79E-22
CLSPN	2.797071052	0.702797835	1.47E-23	3.88E-22
NAA40	1.084598869	3.333936845	1.52E-23	3.99E-22
CIP2A	2.171276679	1.510460437	1.52E-23	4.00E-22
ORM1	-1.846692247	11.5420411	1.57E-23	4.11E-22
LIG1	1.130702336	4.707032161	1.77E-23	4.65E-22
SLC7A11	4.308068505	2.750053947	1.81E-23	4.74E-22
SNRPE	1.18017192	5.629760398	1.82E-23	4.77E-22
CDCA4	1.397009846	2.50028206	1.98E-23	5.16E-22
ADGRE1	-2.067124819	-0.060359749	2.04E-23	5.31E-22
MCM5	1.400433803	5.660686424	2.12E-23	5.52E-22
MAN1A1	-1.177768912	7.282279905	2.15E-23	5.60E-22
C11orf54	-1.158741946	6.537044624	2.17E-23	5.66E-22

KIF24	1.892729978	1.034261361	2.26E-23	5.88E-22
MYOM2	-2.061711642	1.062140301	2.35E-23	6.09E-22
PNPLA7	-1.386373048	3.504332982	2.44E-23	6.33E-22
INS-IGF2	-4.13908567	0.689986819	2.55E-23	6.58E-22
MIR4435-2HG	1.836756105	3.841015908	2.67E-23	6.87E-22
C6	-1.961832085	8.298809025	2.69E-23	6.92E-22
AC024361.1	2.199779559	-1.516661386	2.71E-23	6.95E-22
ENAH	1.556193598	5.999386148	2.73E-23	6.99E-22
STARD5	-1.503221229	2.460945424	3.03E-23	7.74E-22
AC109322.1	2.042738351	0.794064274	3.07E-23	7.81E-22
HOXA10	5.612149318	1.187140691	3.19E-23	8.10E-22
RAB24	1.06122772	2.970795184	3.22E-23	8.17E-22
ADAMTS1	-1.572223433	4.80629299	3.28E-23	8.30E-22
SUCLG2	-1.063376338	7.06376415	3.28E-23	8.30E-22
RFX5	1.163196189	4.907064197	3.41E-23	8.63E-22
TCF3	1.036522309	4.894468079	3.44E-23	8.67E-22
GAS5	1.878429077	6.689558176	3.54E-23	8.92E-22
BACH2	-1.725564953	1.618117159	3.60E-23	9.08E-22
ABAT	-1.600342495	8.305677041	3.62E-23	9.10E-22
SSR2	1.193331525	7.903776271	3.66E-23	9.19E-22
SUCO	1.120167704	5.001618047	3.87E-23	9.72E-22
KRTCAP2	1.163730467	4.798514015	3.94E-23	9.89E-22
C1S	-1.337847181	11.00572918	3.99E-23	9.99E-22
COL4A1	1.77871274	7.516188102	4.07E-23	1.02E-21
IQCE	1.152503372	3.694202629	4.21E-23	1.05E-21
MAG	-2.894757482	-2.537165853	4.29E-23	1.07E-21
LINC02202	2.666280381	-2.060723443	4.43E-23	1.10E-21
MAGEA1	9.011943288	2.880740365	4.44E-23	1.11E-21
RAB11FIP4	1.828374486	5.03570902	4.60E-23	1.14E-21
NCAPD2	1.419673517	4.654774087	4.97E-23	1.24E-21
FBF1	1.430043634	1.366421915	5.13E-23	1.27E-21
FSTL4	4.512409362	1.138571596	5.32E-23	1.32E-21
MAP3K9	1.31199841	2.419691669	5.47E-23	1.35E-21
ACLY	1.049699925	6.402253693	5.78E-23	1.43E-21
ATAD5	1.651355325	1.42536694	5.81E-23	1.43E-21
C11orf96	-1.912862189	4.317833772	6.07E-23	1.50E-21
SFRP1	-2.45295477	0.996120019	6.20E-23	1.53E-21
CNIH4	1.083128772	5.009803842	6.31E-23	1.55E-21
TCF15	2.288352046	-0.644394445	6.35E-23	1.56E-21
LINC00261	-1.372227352	7.153136703	6.48E-23	1.59E-21
ZNF605	1.116321745	3.057861694	6.87E-23	1.68E-21
CFL2	-1.054021738	6.12883768	6.97E-23	1.70E-21
PMFBP1	2.737559529	-0.125101765	7.10E-23	1.73E-21
DSE	-1.42188882	2.852162547	7.27E-23	1.77E-21
SNRPB	1.145675469	6.960526322	7.27E-23	1.77E-21
ZKSCAN3	1.425231846	2.750967183	7.61E-23	1.85E-21
RASGRF2	1.881120585	2.503164992	7.85E-23	1.91E-21
GPR19	2.495416629	-1.120376676	7.86E-23	1.91E-21
AXL	-1.374547259	3.587220898	8.03E-23	1.94E-21
AL136162.1	2.166714411	-1.476175926	8.11E-23	1.96E-21
G6PD	2.597462491	5.209058935	8.30E-23	2.00E-21
PTP4A3	2.549575383	4.599644955	8.62E-23	2.08E-21
MT1X	-2.546957906	8.11723447	9.19E-23	2.21E-21
EBF1	2.141230885	1.73147693	9.91E-23	2.38E-21
HSF2BP	2.100956619	-1.427350193	1.02E-22	2.45E-21
GPSM2	1.60162088	2.147529037	1.06E-22	2.53E-21
ST8SIA6-AS1	5.704032519	1.795576114	1.12E-22	2.68E-21
FAM149A	-1.362255105	4.101504037	1.19E-22	2.85E-21

PMS2P3	1.092399346	0.481122473	1.22E-22	2.92E-21
NGFR	-2.041448195	3.696371984	1.23E-22	2.95E-21
PSMC3IP	1.609826191	0.702087048	1.25E-22	2.99E-21
SIX2	6.148112513	-0.019683657	1.26E-22	3.00E-21
CCL3	-1.628335081	2.053244056	1.27E-22	3.02E-21
FANCB	2.233324498	-1.245926125	1.27E-22	3.02E-21
FBXL19	1.067559294	2.904932409	1.28E-22	3.05E-21
PADI4	-2.23536776	-2.676238798	1.29E-22	3.07E-21
ARHGEF11	1.144751164	5.55017184	1.31E-22	3.11E-21
GPAA1	1.40480254	7.679356171	1.35E-22	3.20E-21
TRIM59	1.934340776	0.905043044	1.36E-22	3.21E-21
TSEN54	1.034177078	4.576531464	1.36E-22	3.23E-21
MSH5-SAPCD1	1.785088526	-0.345136406	1.41E-22	3.33E-21
SCP2	-1.373777685	8.730020804	1.43E-22	3.37E-21
HBB	-2.092745197	4.486595717	1.43E-22	3.38E-21
AC092821.1	2.429020167	-1.515590294	1.46E-22	3.43E-21
AC116049.2	5.045701981	-1.783846316	1.63E-22	3.82E-21
FEZ1	-1.471722	2.205868572	1.63E-22	3.83E-21
FPR1	-1.94572151	1.47800366	1.64E-22	3.85E-21
SIX4	3.360270038	0.211324512	1.68E-22	3.94E-21
TMEM145	3.926995239	-0.05401488	1.72E-22	4.02E-21
SP6	3.113500868	0.301803337	1.91E-22	4.47E-21
ATAD2	1.702407493	5.373428637	2.03E-22	4.74E-21
GBAP1	1.766287314	0.67956201	2.04E-22	4.75E-21
MTX1P1	1.961222287	0.980841294	2.06E-22	4.80E-21
TOB2P1	2.505047131	-0.766071282	2.09E-22	4.87E-21
H2AFZ	1.163347547	6.414473346	2.09E-22	4.87E-21
SEC24B	-1.052917864	5.200866797	2.13E-22	4.96E-21
ZC3H3	1.120839265	4.957806368	2.21E-22	5.13E-21
DNAJC9-AS1	1.487625415	-1.526354244	2.39E-22	5.53E-21
HOGA1	-1.808900316	4.586413164	2.46E-22	5.69E-21
MRAP2	4.168654199	1.867908749	2.52E-22	5.81E-21
KLC2	1.134616268	3.631053922	2.53E-22	5.84E-21
PLGLB2	-1.902574159	1.864931768	2.57E-22	5.93E-21
YPEL2	-1.049818824	4.659669277	2.61E-22	6.01E-21
GAREM1	-1.331043674	3.505093627	2.70E-22	6.21E-21
AC110995.1	-1.649127374	-1.883958726	2.71E-22	6.23E-21
NPEPL1	1.175086841	3.109958769	2.75E-22	6.31E-21
MAPK8IP2	3.258083061	3.13842971	2.78E-22	6.36E-21
AKR1C3	1.592597136	8.332745837	2.89E-22	6.60E-21
GBA3	-2.273825676	5.290839537	2.95E-22	6.72E-21
ACADM	-1.275231564	6.749979306	2.97E-22	6.75E-21
RAB3B	3.769970304	3.551150529	3.02E-22	6.87E-21
AC011294.1	4.430104622	-0.159878512	3.07E-22	6.96E-21
SOGA1	1.489162488	3.894091528	3.08E-22	6.99E-21
TESMIN	2.295220469	1.159062041	3.17E-22	7.17E-21
DPF3	-1.792475682	1.005062015	3.36E-22	7.59E-21
DCN	-2.337030988	6.537267959	3.45E-22	7.79E-21
AC100810.1	-1.085035471	2.75849531	3.46E-22	7.80E-21
COCH	4.323397524	2.230081672	3.47E-22	7.82E-21
B4GALT1-AS1	-1.391200421	0.562746931	3.48E-22	7.82E-21
NDOR1	1.009015711	2.877464295	3.52E-22	7.92E-21
KRT8P12	1.237152807	2.129255888	3.54E-22	7.95E-21
CBX8	1.203377091	3.31718128	3.61E-22	8.09E-21
CPLX2	9.227349359	5.78958935	3.73E-22	8.35E-21
PLOD3	1.022532031	6.851139434	4.08E-22	9.11E-21
AL451050.2	2.30099363	-2.199337644	4.16E-22	9.28E-21
BMS1P8	6.026233318	1.688098623	4.29E-22	9.56E-21

NAMPT	-1.516057663	7.432807538	4.29E-22	9.56E-21
ARHGAP10	-1.492707825	2.678580943	4.41E-22	9.80E-21
CENPO	1.555914991	2.491597205	4.41E-22	9.81E-21
MYO18B	7.101782627	1.663458302	4.46E-22	9.91E-21
CR1	-2.082175178	0.226748466	4.71E-22	1.04E-20
DNMT3A	1.296584459	4.420506034	4.78E-22	1.06E-20
EGFL6	3.986397733	-1.55320407	4.89E-22	1.08E-20
PLPPR2	1.17381061	4.648484406	4.99E-22	1.10E-20
ZNF789	1.038454859	2.294716808	5.00E-22	1.10E-20
ACYP1	1.118281632	1.231447885	5.35E-22	1.18E-20
CD244	-1.688717416	0.213166736	5.45E-22	1.20E-20
NR4A2	-1.899477562	3.385281852	5.62E-22	1.23E-20
CCR1	-1.519155594	2.332847938	5.72E-22	1.25E-20
AC080080.1	2.027943405	-2.024241108	5.77E-22	1.26E-20
CACYBP	1.047153805	5.588638256	5.97E-22	1.30E-20
AL451074.2	2.002588991	-1.792397334	6.04E-22	1.32E-20
IL3RA	1.263646218	2.548331527	6.08E-22	1.32E-20
TMEM56	-1.376659706	6.851087217	6.54E-22	1.42E-20
PXDNL	2.865292147	-0.752424754	6.64E-22	1.44E-20
CD200	1.87219607	1.498320459	6.79E-22	1.47E-20
AL513320.1	2.099943579	-2.331816518	7.19E-22	1.55E-20
C8orf44	1.217425808	0.969193436	8.66E-22	1.87E-20
MESP1	1.971166893	1.329390787	9.20E-22	1.98E-20
OTUB2	1.568221948	1.132720435	9.88E-22	2.12E-20
SLC7A6	1.829822676	1.778210411	1.02E-21	2.18E-20
MT1P3	-3.401800483	-2.526609563	1.06E-21	2.27E-20
GCDH	-1.302329393	6.16894993	1.07E-21	2.29E-20
MTHFD1L	1.503784067	3.357772094	1.10E-21	2.35E-20
RPS6KL1	2.017412386	2.241495628	1.10E-21	2.36E-20
SUZ12P1	1.200698711	1.562332518	1.17E-21	2.49E-20
NMB	2.148816268	2.027022998	1.20E-21	2.56E-20
AC116025.2	3.155291369	-2.76249467	1.22E-21	2.59E-20
CCDC28B	2.35388846	2.314003923	1.22E-21	2.60E-20
SLC38A6	1.173530372	2.954717994	1.24E-21	2.63E-20
FAM222A	1.734630273	3.673719883	1.27E-21	2.69E-20
NPM1P25	-1.342140861	0.587748329	1.29E-21	2.72E-20
TCF21	-1.73976852	0.073492001	1.34E-21	2.84E-20
GABRE	2.841046362	4.033070007	1.41E-21	2.99E-20
HHIPL2	6.397986188	2.066107587	1.44E-21	3.04E-20
ARL16	1.169963318	4.201800599	1.46E-21	3.08E-20
AC105137.2	2.073305029	-2.441608373	1.50E-21	3.15E-20
CRYBG2	2.554128566	1.547346983	1.50E-21	3.16E-20
TUBG1	1.075995413	4.982285358	1.58E-21	3.31E-20
ACVR1C	-1.667580413	0.788641266	1.58E-21	3.31E-20
RTKN2	2.857542301	0.544481436	1.63E-21	3.41E-20
GARS-DT	1.104882592	3.288645734	1.63E-21	3.41E-20
CLEC12A	-1.692047367	-0.096153622	1.72E-21	3.59E-20
ISX	7.091824978	2.643726193	1.73E-21	3.60E-20
THEM5	1.752905612	1.598054082	1.74E-21	3.62E-20
AL139089.1	1.687399387	-1.392354619	1.74E-21	3.63E-20
FOXD2-AS1	2.093304997	1.663262063	1.76E-21	3.66E-20
COLCA2	2.755313242	1.279014967	1.92E-21	3.99E-20
AC011921.1	2.270707756	-2.745383117	2.04E-21	4.24E-20
PAQR4	2.001297329	2.624322854	2.08E-21	4.30E-20
ANKS3	1.056398681	3.297303291	2.08E-21	4.31E-20
LINC01419	11.16861331	3.47921708	2.11E-21	4.36E-20
ALG1L	3.36059268	2.551629168	2.12E-21	4.38E-20
N4BP3	1.987358097	0.776085874	2.14E-21	4.41E-20

U73166.1	2.167931016	-1.163831987	2.15E-21	4.42E-20
MAN1C1	-1.696091311	4.420544667	2.18E-21	4.48E-20
C8B	-1.566875574	8.477884318	2.20E-21	4.53E-20
AGTR1	-1.452145103	5.287492515	2.32E-21	4.76E-20
IQCD	2.686179963	0.325653288	2.56E-21	5.24E-20
LRG1	-1.667093146	9.323989066	2.56E-21	5.24E-20
GOLGA8B	1.624934143	4.052650046	2.58E-21	5.28E-20
SLC25A47	-2.65341485	7.416696125	2.70E-21	5.49E-20
EDIL3	2.774372368	2.545223307	2.79E-21	5.68E-20
CASTOR3	1.552336266	2.648110988	2.82E-21	5.74E-20
AC084033.3	1.637272398	1.078027924	2.94E-21	5.98E-20
GNAO1	-2.248139572	3.53791965	2.97E-21	6.03E-20
MROH1	1.310385803	5.158782675	2.99E-21	6.05E-20
SLC7A2	-1.709349743	7.712940731	3.14E-21	6.34E-20
RETREG1	-2.272490971	4.355370185	3.16E-21	6.38E-20
DLG5-AS1	2.329067966	-0.697709144	3.26E-21	6.59E-20
TTC39A	3.141834031	3.332957341	3.61E-21	7.27E-20
APOL6	-1.120872001	6.749646038	3.75E-21	7.55E-20
PYGB	1.410359587	6.095872585	3.89E-21	7.82E-20
SAPCD2	2.740694847	1.454367374	4.16E-21	8.36E-20
TRIM16	2.965125336	3.922933051	4.17E-21	8.38E-20
SERPINB8	-1.477795897	3.449202072	4.26E-21	8.53E-20
CCNE2	2.109393935	0.964744409	4.31E-21	8.62E-20
FOXD2	2.093331302	1.150604357	4.32E-21	8.64E-20
TMEM220-AS1	-1.4071738	2.389594094	4.44E-21	8.88E-20
HPX	-1.740477902	11.40721629	4.50E-21	8.99E-20
AL163636.2	-1.361015323	0.242876811	4.58E-21	9.14E-20
HOXD3	3.516846797	-2.931420622	4.70E-21	9.36E-20
AL136116.3	-1.186123466	1.780288564	4.87E-21	9.68E-20
AL731569.1	1.467001708	-0.611276697	4.97E-21	9.88E-20
ZBED8	1.238364498	1.391784435	4.99E-21	9.90E-20
KRT16P2	-4.441685263	-1.271104133	5.03E-21	9.98E-20
ITPKA	2.565359737	2.890993766	5.04E-21	9.99E-20
LINC01138	1.545773854	1.776446898	5.05E-21	9.99E-20
AL365181.3	3.766552821	3.506452155	5.11E-21	1.01E-19
GINS3	1.417844186	1.665242797	5.11E-21	1.01E-19
AC010201.2	2.078996311	-1.468563733	5.23E-21	1.03E-19
HMGB2	1.378975603	5.290204746	5.25E-21	1.04E-19
LINC01703	2.354385162	-0.438459878	5.30E-21	1.05E-19
LINC00685	2.112557474	-1.559677532	5.52E-21	1.09E-19
LY6E	-2.021265743	7.411945673	5.58E-21	1.10E-19
PLG	-1.707716666	10.49521084	5.69E-21	1.12E-19
PABPC1L	1.699674553	4.245449449	5.69E-21	1.12E-19
CDC25A	2.128846655	1.849111571	6.20E-21	1.22E-19
MAFG	1.296552627	4.149340848	6.20E-21	1.22E-19
TMEM262	1.149086844	-0.554486811	6.57E-21	1.28E-19
CCDC137	1.004053418	4.29306204	6.58E-21	1.29E-19
LEF1	3.005263127	2.575862274	6.62E-21	1.29E-19
IL1B	-1.820399781	0.507752676	6.70E-21	1.31E-19
EME2	1.202799251	2.652540795	6.98E-21	1.36E-19
AL132780.5	1.82336869	-2.490682809	7.09E-21	1.38E-19
SERPINA11	-1.991763497	7.573098348	7.17E-21	1.39E-19
CSPG5	2.513829657	0.39539768	7.21E-21	1.40E-19
ALKBH6	1.208723581	1.313724081	7.43E-21	1.44E-19
PHF19	1.423187576	3.39023848	7.47E-21	1.44E-19
HID1-AS1	-1.592080917	-2.825876841	7.52E-21	1.45E-19
SIGLEC9	-1.413994274	0.866002978	7.55E-21	1.46E-19
LAMC1	1.373833041	6.954678145	7.65E-21	1.47E-19

RNF139-AS1	1.464551021	-0.068512812	8.01E-21	1.54E-19
PEMT	-1.337293737	5.977355689	8.34E-21	1.60E-19
SMIM14	-1.08404297	7.536923819	8.42E-21	1.62E-19
MT1E	-2.595223842	7.619156679	8.62E-21	1.66E-19
ALDH1B1	-1.338603934	7.147279127	8.64E-21	1.66E-19
AC004816.1	2.02864179	-0.734724124	8.72E-21	1.67E-19
CENPJ	1.257931959	2.191099964	8.91E-21	1.71E-19
AFM	-1.923905049	7.531234869	8.96E-21	1.72E-19
LINC00853	2.404788052	0.35128935	9.16E-21	1.75E-19
DUXAP8	3.106639181	-0.475166982	9.38E-21	1.79E-19
PVT1	2.338434261	2.070792969	9.71E-21	1.85E-19
SNHG20	1.169283298	1.747632078	1.04E-20	1.98E-19
NETO2	2.260353505	1.421901855	1.07E-20	2.04E-19
STX1A	1.549293208	1.00184026	1.11E-20	2.10E-19
CCDC18-AS1	1.370419616	2.002582162	1.11E-20	2.11E-19
B3GNTL1	1.463485877	2.390864446	1.15E-20	2.19E-19
LINC00355	7.270984997	-1.027324258	1.16E-20	2.19E-19
JCAD	1.75471065	4.281091651	1.19E-20	2.26E-19
NR3C2	-1.364429263	2.916350769	1.20E-20	2.27E-19
AMD1P4	-2.623242213	-0.469139385	1.21E-20	2.28E-19
ARHGAP39	1.535899373	1.901353393	1.21E-20	2.29E-19
F8	-1.298181562	3.796510729	1.22E-20	2.29E-19
AC093535.2	-1.084253772	0.280044782	1.22E-20	2.30E-19
IL18R1	-1.489389538	2.187527096	1.22E-20	2.30E-19
IDNK	-1.182198794	3.956356873	1.24E-20	2.34E-19
ASIC3	1.98643078	0.466586809	1.24E-20	2.34E-19
SMUG1P1	-3.32975208	-0.338697828	1.26E-20	2.36E-19
PODXL	1.589945692	5.080159669	1.29E-20	2.42E-19
DGKI	2.292124431	-1.002336369	1.30E-20	2.43E-19
TLCD1	1.610596958	3.495004992	1.32E-20	2.47E-19
PON3	-1.506519313	7.264909213	1.32E-20	2.47E-19
GINS2	1.770924917	2.872834987	1.42E-20	2.65E-19
PPP2R3B	1.041897726	3.049581693	1.43E-20	2.66E-19
STRIP2	2.634243077	1.00505779	1.43E-20	2.66E-19
HP	-2.029337452	13.29987408	1.43E-20	2.67E-19
ST3GAL6	-1.392244583	4.757166849	1.46E-20	2.71E-19
SH3PXD2B	1.30065766	3.574770727	1.50E-20	2.78E-19
ZNF767P	1.023961326	2.045270313	1.52E-20	2.82E-19
NSD2	1.1167698	4.882384523	1.54E-20	2.85E-19
POLE2	1.613642955	1.977267327	1.59E-20	2.93E-19
AC007842.1	2.115370782	-0.846907507	1.62E-20	2.99E-19
SAC3D1	1.270879921	3.815299608	1.62E-20	2.99E-19
TMPO-AS1	1.637395919	1.668932592	1.63E-20	3.01E-19
SCN4A	2.133244073	0.712381551	1.65E-20	3.03E-19
DUSP5	-1.581223421	4.407504725	1.68E-20	3.09E-19
SERPING1	-1.227273804	11.29079699	1.68E-20	3.10E-19
NPSR1-AS1	6.383854959	-0.019723047	1.78E-20	3.27E-19
HSPB1	1.632968073	8.90079641	2.09E-20	3.83E-19
AP001469.3	1.955651256	-0.263078241	2.15E-20	3.94E-19
DDIAS	1.567427512	0.952355068	2.23E-20	4.08E-19
ATP11C	-1.287466936	4.769594226	2.33E-20	4.25E-19
LYPD1	4.088008763	2.570705807	2.37E-20	4.32E-19
H2AFX	1.44048009	4.567581445	2.42E-20	4.41E-19
PDGFA	1.957290811	4.389079121	2.48E-20	4.52E-19
PIMREG	3.118287836	0.544065567	2.55E-20	4.63E-19
IGF2BP3	4.764523192	1.648392592	2.65E-20	4.81E-19
GPR83	-1.829499496	-2.505308477	2.68E-20	4.86E-19
RNF157	2.559583673	3.901825225	2.70E-20	4.91E-19

KCNMB2-AS1	4.668956804	0.024695903	2.71E-20	4.91E-19
LINC02476	7.748747119	-0.180930023	2.71E-20	4.91E-19
AC099850.3	2.633316734	-0.946942189	2.77E-20	5.01E-19
AC245100.6	7.727889662	0.130283198	2.79E-20	5.04E-19
EFNB3	-1.926536978	-0.134092304	2.83E-20	5.11E-19
LINC00205	1.551907536	0.636340626	2.91E-20	5.25E-19
AFAP1-AS1	7.132416747	2.687150821	2.92E-20	5.28E-19
PCK1	-2.205957023	9.934917829	2.93E-20	5.28E-19
STAM-AS1	2.217193607	-2.40294119	2.94E-20	5.30E-19
HMGA1	1.699727879	6.158983309	2.98E-20	5.37E-19
AC023908.3	2.045655933	-2.212669241	3.15E-20	5.65E-19
AC026362.1	1.424061919	-1.693356482	3.31E-20	5.93E-19
AC009831.1	-1.063167848	-0.747127927	3.33E-20	5.97E-19
SLC50A1	1.222515672	6.15517624	3.46E-20	6.19E-19
JAG2	1.673341934	3.374043445	3.50E-20	6.27E-19
TLDC2	2.336208158	1.248985236	3.55E-20	6.34E-19
TDRKH-AS1	1.776559685	-1.572344835	3.55E-20	6.34E-19
AC132872.2	2.507160484	-2.529057418	3.60E-20	6.42E-19
HGF	-2.091713136	3.493012852	3.67E-20	6.55E-19
CENPP	1.095677576	1.331977968	3.80E-20	6.77E-19
SLC8A1	-1.339020044	1.685788704	3.92E-20	6.97E-19
TYMS	1.783278486	4.101028641	4.06E-20	7.22E-19
BAIAP2L2	2.980712438	3.497235108	4.07E-20	7.23E-19
DSTNP2	1.118374621	1.447762901	4.33E-20	7.69E-19
ITGA6	1.420696753	5.827211685	4.35E-20	7.71E-19
MELTF-AS1	2.326392015	-0.213633505	4.37E-20	7.73E-19
FAM83H	1.451619465	6.490451541	4.43E-20	7.84E-19
RBP7	1.994484205	3.329397002	4.52E-20	7.98E-19
ADH4	-2.37750868	9.744922181	4.57E-20	8.07E-19
NAPSB	-1.641678057	2.117015851	4.62E-20	8.15E-19
C4orf46	1.165371581	1.669618995	4.65E-20	8.18E-19
LEF1-AS1	3.74841398	-2.591194867	4.68E-20	8.24E-19
GPHN	-1.215051073	4.831291847	4.73E-20	8.32E-19
LINC02241	8.855924683	0.545682453	4.78E-20	8.40E-19
PHLDB2	-1.013102815	4.870618706	5.02E-20	8.81E-19
NEU1	1.155946326	6.770459569	5.09E-20	8.91E-19
RIPOR3	-2.046351144	1.829044758	5.14E-20	8.99E-19
CRIP3	2.127114442	2.747826339	5.26E-20	9.20E-19
AL590666.2	3.244140172	0.638720452	5.36E-20	9.38E-19
PITX1	5.629102108	2.151723644	6.06E-20	1.06E-18
BRSK1	1.765921069	1.157248873	6.08E-20	1.06E-18
CABYR	4.254313101	2.564001941	6.54E-20	1.14E-18
GUSBP11	1.819809169	0.120142947	6.68E-20	1.16E-18
CSPG4P10	1.759713418	-0.178577881	6.86E-20	1.19E-18
AC139100.2	2.058559004	-0.835056422	6.92E-20	1.20E-18
COL4A2	1.63603553	7.685645598	7.05E-20	1.22E-18
BICDL1	2.256566892	3.453193889	7.15E-20	1.24E-18
SH2D1B	-1.449326611	-1.371873747	7.16E-20	1.24E-18
NKX3-1	-1.553560042	1.156941791	7.21E-20	1.24E-18
WHRN	1.489778516	2.861030875	7.55E-20	1.30E-18
AC010864.1	1.619097778	-1.980477277	7.64E-20	1.32E-18
LINC01532	5.596733618	-2.037718808	7.81E-20	1.34E-18
ACOT2	-1.088811427	5.049293631	8.09E-20	1.39E-18
TFPI2	-2.016077	1.503588518	8.37E-20	1.44E-18
DMGDH	-1.602717393	6.648307678	8.47E-20	1.45E-18
EFNA3	2.097297065	1.447074865	8.61E-20	1.48E-18
GYS2	-2.168128334	5.815639553	8.78E-20	1.50E-18
MT-CO1	-1.080807731	14.08304988	8.86E-20	1.52E-18

AC079174.2	1.764334511	-1.079285503	8.93E-20	1.53E-18
DMD	-1.288002028	5.118110411	9.06E-20	1.55E-18
LMNB2	1.297529833	4.52735579	9.12E-20	1.56E-18
STXBP6	1.677796801	3.420844385	9.26E-20	1.58E-18
CITED2	-1.142139277	5.179769338	9.30E-20	1.58E-18
HS3ST3B1	-1.532063881	5.090831489	9.41E-20	1.60E-18
P4HA2	1.435519603	4.937665146	9.70E-20	1.65E-18
CREG2	3.130162633	-1.942404479	9.93E-20	1.69E-18
FAM72A	1.850170173	-0.999733658	9.97E-20	1.69E-18
AL161740.1	-1.621621223	-1.232696837	1.06E-19	1.81E-18
AC011498.7	1.603896103	-0.964597448	1.07E-19	1.81E-18
NQO1	4.786567683	7.288152964	1.08E-19	1.82E-18
ITLN1	-2.690737539	-0.228980279	1.09E-19	1.84E-18
VKORC1	-1.041878391	6.049693884	1.09E-19	1.85E-18
AC009690.2	1.314912216	-0.151593255	1.10E-19	1.85E-18
CEP152	1.38087451	1.209567904	1.13E-19	1.90E-18
PRRG3	2.358221476	-1.703589728	1.13E-19	1.90E-18
PSPH	1.449870381	4.323098402	1.19E-19	2.00E-18
ZGRF1	1.371671393	0.959609293	1.22E-19	2.04E-18
UGP2	-1.130317734	8.169098282	1.24E-19	2.07E-18
AL158212.3	1.506851891	0.366397758	1.27E-19	2.13E-18
NIPAL1	-1.673836464	3.017051005	1.29E-19	2.16E-18
FEZF1-AS1	5.565695092	0.03001663	1.41E-19	2.34E-18
IGSF3	2.541067746	3.660318502	1.46E-19	2.43E-18
TEX22	2.132665528	-1.246948875	1.50E-19	2.50E-18
KMO	-1.816958673	4.714565845	1.51E-19	2.51E-18
PCNA	1.058586547	5.860635861	1.56E-19	2.59E-18
BCL9	1.341879982	4.145683411	1.58E-19	2.62E-18
CYTOR	1.965224166	3.587206048	1.62E-19	2.69E-18
AL445524.1	2.206595635	3.541114631	1.70E-19	2.82E-18
SNHG4	2.385862767	-0.556305594	1.76E-19	2.92E-18
3-Sep	3.107657288	0.3973147	1.81E-19	3.00E-18
TBX4	7.891595443	1.222269206	1.84E-19	3.05E-18
CCDC163	1.428954862	0.991960986	1.86E-19	3.06E-18
TPM2	1.8878869	5.305338343	1.96E-19	3.23E-18
LINC01537	-1.512315383	-1.701759186	1.96E-19	3.23E-18
MFAP3L	-1.878834069	4.519578187	1.99E-19	3.28E-18
AC127496.7	1.690886596	-1.160925977	2.00E-19	3.28E-18
SLC52A2	1.393412244	4.676116713	2.00E-19	3.28E-18
SNHG7	1.555415428	4.57523651	2.04E-19	3.34E-18
HOXD4	4.856158127	-2.490698844	2.05E-19	3.35E-18
PSMD4	1.07674386	7.859499022	2.12E-19	3.46E-18
PRKAR2A-AS1	1.574696214	0.332003774	2.12E-19	3.46E-18
HAGHL	2.803808741	1.027059153	2.20E-19	3.58E-18
RIBC2	2.942297051	0.151184502	2.22E-19	3.62E-18
MT-ND3	-1.165212696	11.02453258	2.24E-19	3.65E-18
SNX25P1	2.102356287	-1.213848544	2.28E-19	3.70E-18
ZSCAN16	1.121238247	1.723262866	2.35E-19	3.81E-18
HOXA11-AS	5.682790707	-2.061350698	2.37E-19	3.84E-18
AC016550.2	5.572564746	-2.693297162	2.41E-19	3.90E-18
IDUA	1.225670178	3.901195503	2.50E-19	4.03E-18
GRHPR	-1.1917697	8.400869504	2.51E-19	4.04E-18
C5orf46	3.755588804	-0.77882667	2.61E-19	4.21E-18
PLGLA	-2.234864412	1.5196749	2.62E-19	4.22E-18
FAM180A	-2.474729184	1.269361635	2.63E-19	4.24E-18
SLC26A2	1.212055394	3.010100147	2.72E-19	4.37E-18
DNMT3B	1.689271091	1.344884748	2.95E-19	4.73E-18
RNY4P10	1.88200897	-2.529488774	3.14E-19	5.03E-18

RPS26P47	-2.104652064	-2.891563054	3.17E-19	5.07E-18
EVX1	6.685575825	-1.181859832	3.18E-19	5.09E-18
SMYD3	1.493393432	2.598000058	3.39E-19	5.41E-18
TP53I3	1.837981617	4.796622913	3.39E-19	5.42E-18
MAMSTR	2.017285525	0.309368703	3.52E-19	5.62E-18
NNMT	-2.239829285	9.312737455	3.56E-19	5.68E-18
RANBP3L	-2.233009155	1.198488715	3.65E-19	5.82E-18
AC017083.1	1.930395939	-2.471975978	3.77E-19	6.00E-18
TM4SF19-AS1	1.882510192	-1.764899225	3.86E-19	6.13E-18
NCAPD2P1	5.416554525	-1.112290838	4.07E-19	6.46E-18
AC009148.1	2.149981598	-2.703027857	4.29E-19	6.79E-18
SRD5A1	-1.338075464	5.689202623	4.38E-19	6.93E-18
MCM8	1.354541766	2.83673167	4.38E-19	6.93E-18
BOP1	1.642551539	6.18282259	4.39E-19	6.93E-18
LINC02561	3.359141714	-1.998585313	4.54E-19	7.16E-18
INKA1	1.452915833	0.431941708	4.62E-19	7.30E-18
C20orf96	1.010257803	2.150628351	4.64E-19	7.32E-18
AL121832.3	2.213547559	-1.635025702	4.67E-19	7.35E-18
DUSP10	-1.14854775	5.220416486	4.73E-19	7.45E-18
SSUH2	3.877498904	2.293963906	4.88E-19	7.67E-18
AC099508.2	-2.380409435	-1.176051442	4.98E-19	7.82E-18
TRAF5	1.476919004	2.576035414	5.08E-19	7.97E-18
CEP72	1.355130755	2.298257934	5.10E-19	7.99E-18
BLNK	-1.043443866	4.423314465	5.19E-19	8.13E-18
AGXT2	-1.807294945	5.563641928	5.35E-19	8.37E-18
LRRC37A6P	2.908423786	-0.65530336	5.63E-19	8.79E-18
DNM3	1.551708117	0.573231762	5.86E-19	9.15E-18
STAB1	-1.127256179	5.789105073	6.07E-19	9.46E-18
AFP	6.96218248	9.109408127	6.09E-19	9.49E-18
THBS1	-1.677093616	6.917331137	6.13E-19	9.54E-18
SFN	3.802546176	4.758258945	6.26E-19	9.74E-18
AL121899.1	2.284818452	0.196192972	6.42E-19	9.97E-18
CHML	1.726691275	3.565327651	6.52E-19	1.01E-17
RNF165	-1.77973401	0.679642376	6.75E-19	1.05E-17
EBF3	2.248013633	-0.223835629	6.82E-19	1.06E-17
AC099520.1	5.459977444	-2.981026713	7.06E-19	1.09E-17
CYB561D1	1.195537411	2.231930275	7.16E-19	1.10E-17
FAM72B	2.24387953	-1.212360716	7.42E-19	1.15E-17
L3MBTL1	1.391801701	1.151411168	7.99E-19	1.23E-17
CDCA7	3.722449786	1.879061502	8.18E-19	1.26E-17
AC112777.1	3.291575228	-2.100104451	8.26E-19	1.27E-17
SORL1	-1.330267635	6.613981677	8.48E-19	1.30E-17
ASIC1	3.350965384	1.453130578	8.53E-19	1.31E-17
CTSV	4.534524585	2.172265933	9.07E-19	1.39E-17
AL359878.1	2.378653633	-2.506044895	9.21E-19	1.41E-17
RDH16	-2.030078654	7.723432412	9.25E-19	1.42E-17
PPT2-EGFL8	1.087517854	-0.480703384	9.41E-19	1.44E-17
MCAM	1.357081988	5.562378191	9.78E-19	1.49E-17
KIAA1614	1.834669863	0.809829885	9.81E-19	1.50E-17
SIGLEC1	-1.38620482	3.502272581	9.89E-19	1.51E-17
AC025171.2	1.536203004	0.116219244	1.00E-18	1.52E-17
LINC02585	2.250193847	-2.370566392	1.00E-18	1.52E-17
PCBP4	1.093867648	4.477405277	1.01E-18	1.53E-17
ACSM5	-1.862497574	6.726233577	1.03E-18	1.56E-17
APOBEC3A	-1.812721781	-1.576079231	1.05E-18	1.59E-17
ADH1A	-1.793774998	8.935858426	1.05E-18	1.60E-17
TFAP2A-AS1	3.953629524	-1.378916008	1.08E-18	1.63E-17
CD300A	-1.292035972	2.450354919	1.08E-18	1.64E-17

CYP1A2	-3.238253937	7.131914441	1.09E-18	1.65E-17
CP	-1.540977458	9.698252973	1.12E-18	1.69E-17
SBF1	1.023161702	5.434026122	1.13E-18	1.71E-17
S100P	5.875593235	5.799657451	1.17E-18	1.77E-17
RMI2	1.926973944	2.570066686	1.21E-18	1.83E-17
MUC13	5.19400767	6.91101768	1.22E-18	1.83E-17
AQP7	-1.246436324	3.479043679	1.22E-18	1.84E-17
SIRT7	1.013976676	4.216978393	1.23E-18	1.85E-17
AC009005.1	2.177365258	0.022602777	1.26E-18	1.88E-17
AC108463.1	1.844513961	-2.020416164	1.31E-18	1.96E-17
ATAT1	1.083088613	2.601266534	1.31E-18	1.97E-17
MAGEB17	6.597199346	0.548683556	1.31E-18	1.97E-17
LINC00528	1.689132324	0.248631659	1.32E-18	1.98E-17
SPOCK1	4.469698224	2.026182212	1.33E-18	1.99E-17
AC114316.2	4.150682122	-2.162725719	1.39E-18	2.08E-17
AC232271.1	1.359937008	-0.37394619	1.44E-18	2.15E-17
MATN3	5.249612023	1.760981374	1.46E-18	2.17E-17
AC048341.2	1.629010357	-1.134002189	1.46E-18	2.19E-17
AL161668.4	-1.895483956	1.008602534	1.49E-18	2.22E-17
ORM2	-1.581603788	9.719710432	1.58E-18	2.36E-17
ACACB	-1.211305908	6.499030549	1.63E-18	2.43E-17
IGFBP4	-1.065881402	9.843893142	1.70E-18	2.53E-17
HAAO	-1.372150558	7.235553171	1.71E-18	2.55E-17
CD1D	-1.610316992	2.755539426	1.72E-18	2.56E-17
THUMPD3-AS1	1.092393541	2.914757375	1.74E-18	2.58E-17
TRIM7	2.512190464	0.157844206	1.74E-18	2.58E-17
ZNF581	1.210943121	3.536619234	1.83E-18	2.71E-17
FCAR	-2.036708384	-2.741142734	1.86E-18	2.76E-17
MIR3945HG	-1.774755033	-2.772024763	1.96E-18	2.91E-17
SPIRE2	1.280593965	3.969606231	1.98E-18	2.93E-17
TMEM81	1.030944665	1.602987782	2.00E-18	2.96E-17
SPINK5	3.206931124	0.644227963	2.01E-18	2.96E-17
INCENP	1.267096203	2.969861151	2.03E-18	2.99E-17
LRRC1	2.319392363	3.165441387	2.03E-18	2.99E-17
ADM2	2.16574684	3.87609286	2.03E-18	2.99E-17
ACAN	3.600902755	0.633273684	2.09E-18	3.08E-17
C6orf48	1.207766186	5.757581656	2.11E-18	3.11E-17
UBD	2.566710874	6.041675396	2.14E-18	3.14E-17
AC111000.4	2.47640509	1.397998034	2.20E-18	3.23E-17
MIP	-1.679748626	-1.174162182	2.27E-18	3.33E-17
HAO2	-2.372104296	6.146779344	2.30E-18	3.37E-17
AC024230.1	5.670860775	-2.254318282	2.33E-18	3.41E-17
LINC00634	3.548835595	-2.907731001	2.35E-18	3.44E-17
RFXANK	1.079157214	4.745224216	2.40E-18	3.51E-17
PDIA2	6.071041656	1.604164696	2.43E-18	3.54E-17
MEP1A	6.535283679	2.928007374	2.54E-18	3.70E-17
MAFA-AS1	7.565073695	-0.754928049	2.58E-18	3.76E-17
AL138831.3	1.792936473	-1.528063388	2.65E-18	3.85E-17
AC091987.1	5.721055636	-2.573516642	2.67E-18	3.89E-17
LINC01004	1.36154384	-0.314565936	2.71E-18	3.94E-17
DCST1	2.193618209	-1.194916429	2.72E-18	3.94E-17
RDH5	-1.527137362	3.13331504	2.78E-18	4.03E-17
PLA2G6	1.320976519	3.70605776	2.79E-18	4.04E-17
CXCR2P1	-1.940087309	1.53088455	2.80E-18	4.05E-17
GC	-1.24067073	11.39402796	2.81E-18	4.07E-17
CSMD2	3.108551438	0.528291856	2.82E-18	4.07E-17
PDLIM7	1.233651799	4.191174621	2.85E-18	4.12E-17
OSR2	4.142335276	0.477720148	2.88E-18	4.16E-17

SLC2A5	4.295089069	2.999763674	2.88E-18	4.16E-17
ERRFI1	-1.357738621	8.229850089	2.89E-18	4.16E-17
LPL	2.472458382	2.066596323	2.92E-18	4.20E-17
KCNK9	6.220914542	0.033530936	2.97E-18	4.27E-17
AC004160.1	-2.331799026	-0.145074346	2.99E-18	4.30E-17
BGLAP	1.777963841	-0.675088773	3.15E-18	4.53E-17
GSDMC	3.854806077	-0.898223296	3.18E-18	4.57E-17
AL022322.1	1.886774919	-0.771813581	3.20E-18	4.59E-17
GLUD1P3	1.148434781	-0.791805105	3.21E-18	4.60E-17
AC034229.4	2.559179523	-2.90550347	3.26E-18	4.66E-17
DTX1	-1.835418995	4.39909383	3.33E-18	4.77E-17
TLX1	2.85770512	1.664971239	3.47E-18	4.96E-17
MOGAT2	-2.237297096	5.266832306	3.49E-18	4.98E-17
SYPL1P2	2.363335382	-2.609079156	3.50E-18	5.00E-17
FGD1	1.381350771	2.710928347	3.67E-18	5.23E-17
MT-ND2	-1.081727048	12.43170791	3.77E-18	5.36E-17
POU5F1	1.980561281	0.786775565	3.79E-18	5.38E-17
SNHG25	2.379622491	0.339051987	3.91E-18	5.55E-17
AC078778.1	2.056756152	-1.651436847	3.95E-18	5.60E-17
LYPD8	4.898498015	0.222297959	4.14E-18	5.86E-17
PRND	3.924374309	-0.408746935	4.16E-18	5.89E-17
SLC22A1	-2.387903616	8.203894945	4.19E-18	5.93E-17
HIST1H2AM	2.433889269	-2.142695865	4.20E-18	5.94E-17
ANKRD13B	1.697752259	1.051848026	4.24E-18	5.99E-17
GAD1	4.6610037	-0.295272447	4.28E-18	6.05E-17
PPP1R35	1.078526735	3.919533476	4.29E-18	6.05E-17
KIAA0040	-1.102844008	4.302406164	4.34E-18	6.12E-17
CLN3	1.087549697	4.989312848	4.39E-18	6.18E-17
AC097641.2	1.76029932	-2.660935441	4.39E-18	6.18E-17
DNA2	1.371997952	1.73691999	4.57E-18	6.43E-17
AL024498.1	1.930773862	-2.793705135	4.62E-18	6.50E-17
ARIH2OS	1.039181551	0.160673921	4.71E-18	6.61E-17
RBM24	2.597254603	2.420256498	4.74E-18	6.66E-17
FGB	-1.592992717	12.90072733	4.78E-18	6.70E-17
AC003102.1	1.559645842	-0.153020649	4.89E-18	6.85E-17
MAFF	-1.251012294	4.728028544	4.96E-18	6.95E-17
PKN3	1.419067003	3.201954228	5.03E-18	7.04E-17
GPR158	3.398243559	1.502276727	5.12E-18	7.16E-17
LCN2	4.709637226	7.630631946	5.16E-18	7.21E-17
SYDE2	-1.257845107	1.075440566	5.19E-18	7.25E-17
SLITRK6	-2.848732834	-1.998594425	5.26E-18	7.33E-17
TTC39B	-1.174599314	3.181713644	5.27E-18	7.35E-17
PGC	10.50064323	6.733405667	5.31E-18	7.39E-17
WNK4	3.715778548	2.669998573	5.39E-18	7.51E-17
NLRP12	-1.365206383	-2.57442744	5.73E-18	7.97E-17
DNMT1	1.171082767	5.074186916	5.75E-18	7.99E-17
HIST1H2AI	3.530901133	-2.062524207	5.84E-18	8.11E-17
TCTEX1D1	-2.101691707	0.491438222	5.85E-18	8.13E-17
IL33	-1.625538874	3.392143206	5.88E-18	8.15E-17
CD81	-1.037795224	8.992803979	5.88E-18	8.15E-17
C9	-2.61552567	8.79931701	6.00E-18	8.31E-17
CD14	-1.423048187	8.663843796	6.14E-18	8.48E-17
REEP2	3.055191388	0.115434783	6.22E-18	8.58E-17
PRIM1	1.283377874	2.881652309	6.26E-18	8.62E-17
STAU2-AS1	2.406183515	-2.421378198	6.45E-18	8.88E-17
HRAT92	2.98960965	-0.119387913	6.50E-18	8.95E-17
BMP5	-2.503716382	-0.089933425	6.53E-18	8.97E-17
AZGP1	-1.577188321	9.871561594	6.56E-18	9.01E-17

C1orf162	-1.230607721	2.901268407	6.58E-18	9.04E-17
AL021707.2	1.572869567	-0.449949744	6.62E-18	9.09E-17
AC090502.1	7.963087409	0.74442857	6.63E-18	9.09E-17
AC011462.4	1.923288284	-2.186975584	6.68E-18	9.15E-17
EML6	2.014620655	1.226086602	6.68E-18	9.15E-17
NANOS1	2.642553217	0.449968483	6.96E-18	9.51E-17
CLCN2	1.029711276	2.644282394	7.02E-18	9.60E-17
LINC01451	3.162500949	1.690344337	7.03E-18	9.61E-17
FAH	-1.00856432	7.528762074	7.10E-18	9.69E-17
GRAMD1A	1.429673249	5.308721538	7.13E-18	9.71E-17
AC110285.7	2.030736121	-0.008972061	7.50E-18	1.02E-16
CD109	2.458322059	3.517043657	7.90E-18	1.07E-16
HCN2	3.276237011	0.686195968	8.11E-18	1.10E-16
PDE4C	1.912216005	0.623619348	8.19E-18	1.11E-16
MAFA	6.535580875	1.293476028	8.22E-18	1.11E-16
DCST2	2.065814157	0.745642496	8.25E-18	1.12E-16
MT-ND6	-1.324612773	10.47166377	8.29E-18	1.12E-16
SNHG3	1.665868943	3.41034368	8.34E-18	1.13E-16
AC015908.3	-1.365223921	1.039319477	8.43E-18	1.14E-16
CSMD1	6.446869067	1.1825757	8.50E-18	1.15E-16
AC069544.1	1.343587364	-0.903594395	8.56E-18	1.16E-16
LINC00628	2.701588806	-2.798916446	8.77E-18	1.18E-16
TMPRSS2	-1.294491683	5.264169267	8.80E-18	1.18E-16
HOXD1	5.435912338	-2.051723466	8.99E-18	1.21E-16
TMEM147-AS1	1.209138789	1.446637946	9.01E-18	1.21E-16
DSTYK	1.024971338	3.354464814	9.83E-18	1.32E-16
SAA4	-1.846704632	6.840857029	1.03E-17	1.38E-16
AC073842.2	2.229128493	-2.566009589	1.03E-17	1.39E-16
TIGD3	2.156227987	-0.228890431	1.07E-17	1.43E-16
AL117379.1	1.622567827	-1.406146978	1.14E-17	1.52E-16
DLK2	1.944444529	0.560716852	1.18E-17	1.58E-16
AC004540.2	-2.39257682	-0.546047966	1.19E-17	1.59E-16
AC026740.1	2.631185638	-0.126489713	1.24E-17	1.65E-16
5-Sep	1.972526469	2.769200249	1.27E-17	1.70E-16
RPL23AP7	1.046669303	0.527040806	1.28E-17	1.71E-16
HIST1H4H	2.586512	0.095598062	1.32E-17	1.76E-16
NCAPG2	1.241106762	3.427644669	1.34E-17	1.79E-16
EPPK1	3.351951476	3.184074054	1.35E-17	1.79E-16
LINC01121	4.203213722	-2.310409916	1.35E-17	1.79E-16
KCP	2.946851872	0.32933305	1.39E-17	1.84E-16
CCL25	6.889079204	4.49560121	1.40E-17	1.86E-16
AC110285.2	2.123234856	0.97843208	1.44E-17	1.91E-16
CDH8	3.658097171	-2.069531364	1.45E-17	1.91E-16
AC013731.1	1.924801115	-1.97593106	1.45E-17	1.92E-16
MYO1A	3.01428481	1.522428045	1.48E-17	1.95E-16
COL2A1	9.256576405	4.268804616	1.53E-17	2.02E-16
SEZ6	6.939232284	2.56186759	1.54E-17	2.03E-16
QRICH2	1.489002127	2.153374687	1.58E-17	2.09E-16
PAFAH1B3	1.953187482	3.964418377	1.60E-17	2.11E-16
TIGD7	1.162658504	0.493660504	1.60E-17	2.11E-16
BANF2	4.934159045	-2.064515906	1.61E-17	2.12E-16
GLA	1.165561197	4.325281643	1.65E-17	2.17E-16
AC096887.2	1.447630583	0.166924369	1.66E-17	2.18E-16
SIPA1L3	1.171709289	4.538768113	1.68E-17	2.20E-16
ADH6	-1.523259893	7.979668729	1.79E-17	2.34E-16
SNHG12	1.262714159	2.3081636	1.91E-17	2.50E-16
ADM	-1.347463592	4.327872904	1.92E-17	2.51E-16
GALNT10	1.023170906	4.127972388	1.93E-17	2.52E-16

AP000553.2	2.958068746	-2.940719429	1.95E-17	2.54E-16
LINC00665	2.804053193	2.899382603	1.99E-17	2.59E-16
NTN4	-1.270534401	3.877570011	1.99E-17	2.59E-16
AL139412.1	2.341186405	-0.890152941	2.03E-17	2.64E-16
ATP6V1FNB	2.78405641	-0.25138596	2.04E-17	2.65E-16
AC068669.1	2.652287121	-2.943084597	2.11E-17	2.74E-16
SLC30A3	4.90667487	0.912374885	2.11E-17	2.74E-16
EPHX4	2.60027875	-1.729383108	2.15E-17	2.79E-16
SLC19A3	-1.493492058	4.008784409	2.22E-17	2.87E-16
SMC1B	3.714429473	-0.017387508	2.25E-17	2.91E-16
AP001412.1	1.844606262	-2.801736095	2.25E-17	2.91E-16
KCNN3	1.50867213	1.589259881	2.28E-17	2.94E-16
AP003352.1	1.420480688	1.409977043	2.35E-17	3.04E-16
RPL32P3	1.036137471	1.691433532	2.39E-17	3.08E-16
QDPR	-1.073655917	6.88292882	2.52E-17	3.25E-16
TMEM164	1.344563175	3.76769941	2.59E-17	3.34E-16
C19orf48	1.313868583	5.532609232	2.64E-17	3.40E-16
MT-ND5	-1.277885185	11.69449196	2.71E-17	3.48E-16
WDR17	-1.6980281	-2.709359291	2.86E-17	3.67E-16
FNIP2	-1.26480094	5.464974824	2.92E-17	3.74E-16
FGL2	-1.292801163	4.064994602	2.92E-17	3.74E-16
TRIM50	5.362693307	2.480849472	2.97E-17	3.81E-16
TMCC1-AS1	1.571936902	0.071985197	2.99E-17	3.83E-16
PRR7-AS1	2.66604746	-2.504094021	3.00E-17	3.84E-16
ABHD6	-1.065887953	5.092881572	3.03E-17	3.87E-16
ATP6V1C1	1.000751693	6.02599654	3.06E-17	3.91E-16
SFI1	1.270492281	3.702342543	3.07E-17	3.92E-16
AC105118.1	7.552404351	-0.684398153	3.08E-17	3.93E-16
ZNF572	1.412541054	0.819705991	3.17E-17	4.04E-16
AGO2	1.01393965	4.629773546	3.20E-17	4.07E-16
INKA2	1.212988522	2.137160182	3.37E-17	4.29E-16
CD226	-1.444713699	0.724824914	3.49E-17	4.43E-16
UGT2B7	-1.817364646	8.42910933	3.59E-17	4.56E-16
ADRA2C	3.303296375	2.330025416	3.64E-17	4.62E-16
UNC119B	1.169493693	3.649570435	3.65E-17	4.63E-16
LINC01970	3.131303024	-2.000005845	3.66E-17	4.64E-16
AC023355.1	2.072405438	-2.17983351	3.67E-17	4.65E-16
MSX1	2.003102965	1.026203246	3.67E-17	4.65E-16
SLC7A8	-1.459246088	2.85431402	3.74E-17	4.74E-16
GTF2IRD1	1.110642363	3.572991515	3.75E-17	4.74E-16
C20orf144	2.278699867	-2.771579146	3.75E-17	4.74E-16
HERC2P2	1.314781568	3.406726035	3.94E-17	4.97E-16
ZFPM2-AS1	4.064598881	1.363842721	3.95E-17	4.98E-16
ELFN2	3.691437027	1.619536479	4.05E-17	5.10E-16
PFDN6	1.038243585	5.69534016	4.12E-17	5.18E-16
LINC01679	-1.368714607	-1.003067437	4.17E-17	5.24E-16
AL365181.2	3.744487611	1.199620933	4.19E-17	5.27E-16
PPM1K	-1.270442634	3.203833479	4.21E-17	5.29E-16
HRCT1	2.848351445	2.986568574	4.27E-17	5.36E-16
ARID3C	-1.578010131	1.953014982	4.28E-17	5.36E-16
FLJ31104	2.093046675	-1.372218453	4.31E-17	5.40E-16
PROCA1	1.499334992	0.389970099	4.38E-17	5.48E-16
NRM	1.467797241	3.542550876	4.42E-17	5.53E-16
RSAD2	-1.654905197	2.740584903	4.44E-17	5.55E-16
AC016737.1	2.099188857	-2.283407681	4.46E-17	5.58E-16
SLC9A9	-1.221892612	2.032868205	4.57E-17	5.71E-16
SSX1	8.719323811	2.962453349	4.65E-17	5.80E-16
HMGCLL1	-2.307309312	-2.051392853	4.65E-17	5.80E-16

AC091133.4	4.481533967	-2.657592679	4.90E-17	6.11E-16
VASH2	2.37093102	1.049798509	5.08E-17	6.32E-16
ZFPM2	-1.368396537	0.882458475	5.32E-17	6.61E-16
FGF23	-3.576960484	-2.238966769	5.37E-17	6.66E-16
RBL1	1.261729005	2.29080155	5.58E-17	6.92E-16
AC112484.3	2.178030694	-0.75688872	5.73E-17	7.10E-16
ASMTL-AS1	1.572731798	1.666278547	5.73E-17	7.10E-16
WISP3	-1.455485088	-1.535187623	5.78E-17	7.15E-16
SULT1C2	3.570044225	3.918195301	5.81E-17	7.19E-16
SPINDOC	1.20779477	2.896790208	5.82E-17	7.20E-16
YJEFN3	1.752678747	0.740825538	5.85E-17	7.24E-16
GRIN2B	-2.299229855	0.103254265	6.01E-17	7.42E-16
ARID3A	2.496812818	3.958215936	6.19E-17	7.62E-16
ASS1P9	-1.730873209	-2.603195545	6.22E-17	7.65E-16
CKAP2	1.243089567	3.3289574	6.24E-17	7.67E-16
AC005332.1	1.910615809	-2.799006601	6.31E-17	7.75E-16
WDYHV1	1.128239601	3.052126671	6.60E-17	8.09E-16
AC006252.1	1.965646759	-2.544999553	6.91E-17	8.46E-16
AC126118.1	2.019814461	-2.721883884	7.06E-17	8.64E-16
COX7B2	8.901193291	2.22946428	7.35E-17	8.99E-16
AL133215.2	2.52810576	-2.549890626	7.52E-17	9.20E-16
BX842568.4	-2.121711584	-1.313738932	7.67E-17	9.37E-16
AC004890.2	1.875427005	0.375944448	7.75E-17	9.46E-16
LINC00894	1.582642997	0.064949655	8.22E-17	1.00E-15
AC092535.4	1.925773143	1.045601954	8.30E-17	1.01E-15
SLC38A4	-1.50690939	8.230390742	8.38E-17	1.02E-15
ZEB2	-1.171108674	3.657068651	8.41E-17	1.02E-15
CASTOR2	1.567777028	-0.177183079	8.49E-17	1.03E-15
GNG4	5.435178304	3.271583073	8.58E-17	1.04E-15
SNCG	3.473946833	4.434576437	8.58E-17	1.04E-15
AC022306.2	1.357171229	-0.496315481	8.67E-17	1.05E-15
GLP2R	-2.29207625	-0.925625011	8.88E-17	1.08E-15
MAPK12	2.169482375	3.212904723	8.92E-17	1.08E-15
AP000523.1	3.036443742	-2.608103451	9.12E-17	1.10E-15
AC012615.1	1.280178722	0.956772883	9.30E-17	1.13E-15
RHPN1	1.841484852	2.66439336	9.32E-17	1.13E-15
DAPK2	1.605659521	3.432059864	9.47E-17	1.14E-15
SLC17A8	-2.431190978	-2.246711009	9.58E-17	1.16E-15
EMX1	3.840940757	0.651678233	9.68E-17	1.17E-15
FP671120.4	-1.743914248	-0.873260041	9.79E-17	1.18E-15
CFL1P1	1.427082384	-1.806056539	9.84E-17	1.19E-15
AL445228.2	2.963194662	-2.787968539	1.00E-16	1.21E-15
AVPI1	-1.040228345	4.661498069	1.01E-16	1.21E-15
CKS2	1.33116471	3.927177057	1.04E-16	1.25E-15
BCHE	-1.879405512	5.729701473	1.05E-16	1.26E-15
ZNF296	2.15318156	0.255970303	1.07E-16	1.29E-15
DYNC1I1	3.845709984	2.382364826	1.10E-16	1.32E-15
BFSP1	1.46659384	0.137779612	1.10E-16	1.32E-15
C1QTNF3	4.037580002	4.504621516	1.12E-16	1.34E-15
MKRN3	3.962564095	-0.676973547	1.16E-16	1.39E-15
HAND2-AS1	-2.378348675	0.348514384	1.18E-16	1.41E-15
AC024595.1	5.831206441	-2.706025137	1.19E-16	1.42E-15
ZHX1-C8orf76	1.436620334	0.103540003	1.21E-16	1.44E-15
HES6	1.402699947	2.815626664	1.22E-16	1.46E-15
SPP1	4.284306157	9.196720526	1.24E-16	1.47E-15
ZNF341	1.181544976	2.417035738	1.25E-16	1.49E-15
AP003068.4	2.82767501	-0.086786155	1.26E-16	1.50E-15
ARHGEF26	-1.277119898	4.639012394	1.30E-16	1.54E-15

LRRC77P	3.102415179	-1.746935245	1.31E-16	1.55E-15
SKAP1	-1.559601067	3.196226337	1.36E-16	1.61E-15
RAB6B	1.466978341	1.802715573	1.38E-16	1.64E-15
AVPR1A	-2.471983493	5.015281477	1.38E-16	1.64E-15
SLC36A1	1.023500747	2.938971906	1.38E-16	1.64E-15
LINC01535	3.444073031	-1.530213621	1.42E-16	1.68E-15
AL031186.1	1.550355438	-2.175321512	1.43E-16	1.69E-15
LINC00511	3.327845026	1.868925309	1.44E-16	1.70E-15
MCM3AP-AS1	1.082117207	0.292862348	1.44E-16	1.70E-15
LINC01116	3.224917182	-0.530358285	1.45E-16	1.71E-15
DQX1	5.980100762	0.925608383	1.48E-16	1.75E-15
NAP1L4P1	2.927822984	-0.482960395	1.48E-16	1.75E-15
SPIC	-1.860078326	-2.097191769	1.50E-16	1.77E-15
GPSM1	2.012909707	3.554308746	1.53E-16	1.81E-15
AC009159.3	-1.26835522	0.093900587	1.54E-16	1.82E-15
ANXA2R	1.513102749	1.013350389	1.57E-16	1.84E-15
AC011005.4	2.499949846	-2.256127378	1.58E-16	1.86E-15
ATP5MC1P4	2.689076179	-2.399383391	1.58E-16	1.86E-15
FAM86JP	1.180751062	0.891644569	1.60E-16	1.87E-15
AL691420.1	7.280092076	-1.491162429	1.60E-16	1.87E-15
NADK2	-1.044299336	7.628458967	1.60E-16	1.88E-15
AC103706.1	1.8071604	-0.281216834	1.64E-16	1.92E-15
PIAS3	1.013104891	3.348319258	1.65E-16	1.93E-15
ANXA10	-1.852292704	4.262455725	1.67E-16	1.96E-15
DOCK3	2.574758096	-0.806149936	1.68E-16	1.96E-15
BCAS4	1.685270815	1.939486528	1.70E-16	1.99E-15
C1QL1	3.931585972	2.863278656	1.73E-16	2.02E-15
AC087392.1	-2.455131523	-0.158415168	1.78E-16	2.08E-15
ADGRG6	-1.32659171	5.934988141	1.86E-16	2.16E-15
MEX3A	1.866547196	2.482533958	1.87E-16	2.17E-15
LDHD	-1.308348477	6.520980768	1.87E-16	2.17E-15
STK24-AS1	1.779623311	-1.799988139	1.90E-16	2.21E-15
DNASE1L2	2.659893516	-0.200587002	1.93E-16	2.25E-15
NFAM1	-1.287832343	2.245456691	2.04E-16	2.37E-15
FUT2	2.974645224	1.210460161	2.06E-16	2.39E-15
CWH43	-2.787413369	-1.831333039	2.11E-16	2.45E-15
DCAF13	1.02856504	5.014441109	2.12E-16	2.46E-15
GNG12-AS1	-1.31876375	-1.036419328	2.14E-16	2.48E-15
AL138828.1	4.437054029	0.78743494	2.16E-16	2.50E-15
CPA6	5.741837477	-0.476578331	2.17E-16	2.50E-15
C1orf198	1.139048007	5.543281551	2.17E-16	2.51E-15
AC005041.3	1.656316691	-1.121360181	2.20E-16	2.54E-15
AL035461.2	1.733457977	-0.15731378	2.25E-16	2.59E-15
ACTN2	4.743754165	3.255712012	2.27E-16	2.61E-15
MBL2	-1.83909667	6.688765079	2.32E-16	2.67E-15
RGMA	1.912285286	-0.075390871	2.32E-16	2.67E-15
RPLP0P2	3.051512058	-0.641440877	2.34E-16	2.69E-15
A1CF	-1.003174323	7.537151008	2.34E-16	2.69E-15
SLC6A8	2.891659293	4.699954157	2.36E-16	2.71E-15
LINC01833	7.199248001	-0.679512335	2.39E-16	2.74E-15
CUEDC1	1.046695859	4.059843391	2.43E-16	2.79E-15
GNAL	2.03240064	2.370705723	2.51E-16	2.88E-15
AC099778.1	1.281251778	-1.090039365	2.54E-16	2.91E-15
CEL	3.226097775	0.191137787	2.56E-16	2.93E-15
DPY19L1P1	1.00981895	0.222517794	2.69E-16	3.07E-15
AC117386.2	5.149532654	-1.21689472	2.73E-16	3.11E-15
COL24A1	3.942793452	-0.089305732	2.78E-16	3.17E-15
FIBCD1	5.641225918	-0.800032295	2.87E-16	3.27E-15

FIRRE	3.171712098	-1.912608966	3.05E-16	3.46E-15
UROC1	-2.298252215	5.920001491	3.12E-16	3.54E-15
CISH	-1.210985289	5.182356278	3.14E-16	3.56E-15
MAGEC2	8.566094965	2.857245239	3.16E-16	3.58E-15
AP001453.3	1.31111931	-1.227756068	3.17E-16	3.59E-15
ZNF580	1.119811587	3.211029318	3.24E-16	3.67E-15
AC011447.7	2.930418359	-0.823827363	3.25E-16	3.68E-15
GOT2	-1.003392338	8.080278231	3.28E-16	3.71E-15
MYCN	3.654240032	1.145070613	3.32E-16	3.75E-15
CTNNA3	-2.217874829	1.270618561	3.48E-16	3.93E-15
AP006623.1	1.603850905	-0.949858004	3.51E-16	3.96E-15
WFS1	1.008317847	5.195892953	3.54E-16	3.99E-15
DUOX1	2.272407269	1.676801387	3.60E-16	4.05E-15
SFRP4	2.941742308	2.147899161	3.61E-16	4.06E-15
CYP19A1	6.859573394	1.357526606	3.62E-16	4.07E-15
Z97832.2	1.621119842	-1.565517133	3.65E-16	4.10E-15
HK3	-1.420575091	1.856604435	3.73E-16	4.19E-15
STRC	7.871761989	2.111521899	3.74E-16	4.19E-15
DSCAM	-2.069493867	-2.850924011	3.82E-16	4.29E-15
DKK1	5.788251577	3.857785147	3.85E-16	4.31E-15
RPL18P13	4.751621582	-1.821287696	3.87E-16	4.34E-15
AC068987.1	2.225018667	-2.581843187	3.90E-16	4.37E-15
PSRC1	1.551152962	2.15407137	3.90E-16	4.37E-15
ANKRD23	1.154528262	-0.78987213	3.91E-16	4.37E-15
STK39	1.999440587	3.313477198	3.93E-16	4.39E-15
FOSL1	-1.692904946	0.946116794	3.95E-16	4.41E-15
B4GALNT2	7.250308831	1.949987494	4.07E-16	4.54E-15
DLX5	5.285166059	-1.237992855	4.09E-16	4.56E-15
REXO5	1.377028909	2.176904231	4.09E-16	4.56E-15
LINC00482	1.770343362	1.862872842	4.09E-16	4.56E-15
PSAT1	-1.294715424	6.483182285	4.37E-16	4.85E-15
ADRA1B	-1.566946776	2.026870786	4.47E-16	4.97E-15
JPT1	1.2928025	5.425739036	4.48E-16	4.97E-15
GLDN	3.838874289	2.081156942	4.55E-16	5.05E-15
AC022007.1	1.310865277	0.257535889	4.57E-16	5.07E-15
AC091057.4	2.277734076	-2.693165445	4.58E-16	5.08E-15
MFSD2A	-2.271722939	5.975344973	4.65E-16	5.15E-15
LDLRAD1	4.117102187	1.453502764	4.69E-16	5.19E-15
HIST1H3B	3.154671444	-1.630076702	4.77E-16	5.28E-15
PLEKHG2	1.118836023	3.66743416	4.79E-16	5.29E-15
RHO	3.551530909	-2.786397919	4.86E-16	5.37E-15
AC005393.1	2.148910713	-2.649649515	4.91E-16	5.41E-15
TM4SF20	7.987120829	3.276829271	4.91E-16	5.41E-15
AC087741.1	1.312776269	1.59891614	5.18E-16	5.70E-15
TMEM71	-1.126232141	-0.563372309	5.21E-16	5.73E-15
BEST4	2.27222944	-1.309431643	5.29E-16	5.81E-15
AC096642.1	1.713803567	-1.713524789	5.31E-16	5.84E-15
AC022211.2	1.496615487	-0.519983601	5.52E-16	6.06E-15
MYBPC1	3.712529925	-0.749385965	5.54E-16	6.08E-15
ST6GAL2	-2.147249884	0.468012697	5.62E-16	6.16E-15
TEX41	3.267581047	0.929657008	5.64E-16	6.18E-15
PRRX1	2.409180559	1.482223044	5.68E-16	6.22E-15
PHPT1	1.202170767	6.098562268	5.76E-16	6.30E-15
TDRKH	1.217699382	3.318754387	5.76E-16	6.30E-15
SHMT1	-1.28674431	8.09525506	5.79E-16	6.33E-15
AL390728.4	1.552467987	2.669757165	5.95E-16	6.50E-15
ABCC9	-1.541840633	4.79814536	6.07E-16	6.63E-15
AP000347.2	1.743277919	-1.177625297	6.18E-16	6.74E-15

HAO1	-1.453009118	7.638248306	6.18E-16	6.74E-15
AL160006.1	1.257809696	1.534245859	6.24E-16	6.80E-15
DUSP5P1	4.742666784	-2.827205148	6.30E-16	6.87E-15
DLG5	1.623543527	3.80538452	6.60E-16	7.18E-15
UPK3A	5.677676693	2.996685419	6.66E-16	7.24E-15
TSACC	1.5639321	0.247573828	6.73E-16	7.30E-15
KCNC1	4.935474706	-1.664885503	6.81E-16	7.38E-15
AC127024.5	1.493469176	-1.484626848	6.92E-16	7.49E-15
UGT2B11	3.215723367	4.758568055	6.95E-16	7.52E-15
SPARCL1	2.768556647	7.076279427	7.03E-16	7.60E-15
LINC01235	3.092901372	-1.053224635	7.07E-16	7.64E-15
FLVCR1-DT	1.602455284	1.546856079	7.10E-16	7.66E-15
UCHL1	4.744489925	3.583413611	7.12E-16	7.68E-15
LINC01224	4.513110291	-0.705838052	7.17E-16	7.73E-15
IBSP	6.632646002	-0.531096592	7.18E-16	7.73E-15
ETV4	2.982192047	4.20547647	7.27E-16	7.82E-15
CHKA	1.118836549	5.221946796	7.40E-16	7.96E-15
PI15	4.05681933	0.926565228	7.50E-16	8.06E-15
AP003469.4	1.473236119	1.968993407	7.52E-16	8.07E-15
TLR3	-1.180420018	2.988016588	7.67E-16	8.23E-15
FAM167B	-1.043158756	2.411731352	7.78E-16	8.35E-15
AC093895.1	5.182288944	-1.608598533	7.84E-16	8.40E-15
VAX2	3.338334763	-1.13977285	7.91E-16	8.47E-15
SOX12	1.384930093	4.585056018	8.00E-16	8.56E-15
HOXA3	2.828344456	1.32260043	8.06E-16	8.62E-15
FAM50A	1.068763478	6.262440944	8.36E-16	8.93E-15
NES	1.257795473	5.033667792	8.37E-16	8.94E-15
AL355388.2	2.470571529	-2.15820377	8.47E-16	9.04E-15
BRICD5	1.390652323	0.891319082	8.76E-16	9.34E-15
CACNB4	3.152699544	-0.093581459	8.78E-16	9.36E-15
AP003469.2	3.862797441	-2.346064501	8.83E-16	9.41E-15
THBD	-1.268247587	3.471602407	8.84E-16	9.42E-15
PLXNC1	1.849871205	3.87849991	8.85E-16	9.42E-15
PGP	1.13664433	4.3231187	8.92E-16	9.49E-15
TNFRSF25	1.514187121	2.459647716	9.08E-16	9.66E-15
CPXM1	2.367146281	1.447282239	9.18E-16	9.74E-15
GINS4	1.746782906	1.346818356	9.27E-16	9.84E-15
SNX15	1.15092401	-0.929564158	9.38E-16	9.94E-15
PLXNA1	1.299251645	4.170652128	9.39E-16	9.96E-15
AC020978.4	-1.740055183	0.522941452	9.45E-16	1.00E-14
AL731563.3	1.19593216	-1.066847476	9.79E-16	1.04E-14
AC005089.1	1.784987274	-0.336531072	9.84E-16	1.04E-14
HOXD-AS2	4.002888646	-2.074083904	1.01E-15	1.07E-14
DYDC2	3.442043111	0.902820869	1.01E-15	1.07E-14
CYP2C9	-1.797828125	8.873884454	1.04E-15	1.10E-14
DAND5	2.863224966	-2.515046317	1.05E-15	1.11E-14
AC011445.2	2.586486644	-1.103201114	1.05E-15	1.11E-14
LINC01980	7.158793678	0.413065695	1.05E-15	1.11E-14
CCDC78	2.302296442	-0.376806825	1.06E-15	1.11E-14
SERPINF2	-1.274754116	10.25895367	1.08E-15	1.14E-14
AC233280.1	2.324659956	-2.651611675	1.10E-15	1.16E-14
SOWAHA	1.868215238	3.50231181	1.12E-15	1.17E-14
PLEKHH1	1.386518466	1.897786313	1.12E-15	1.18E-14
MTHFD1	-1.224663586	7.980719336	1.12E-15	1.18E-14
AC005829.1	2.095635813	-1.790310998	1.13E-15	1.19E-14
STX11	-1.350570549	1.270861837	1.14E-15	1.20E-14
ACTBP9	-1.466617762	-2.347695368	1.15E-15	1.20E-14
IL17D	3.154108441	1.119751253	1.15E-15	1.21E-14

AC006538.1	2.33687244	-1.534746468	1.16E-15	1.21E-14
FGA	-1.386409747	13.4978252	1.17E-15	1.22E-14
OLFML3	-1.537420517	3.443549557	1.20E-15	1.25E-14
LINC01426	2.844345314	1.17885735	1.20E-15	1.26E-14
LINC01287	8.584185268	2.933194629	1.22E-15	1.27E-14
PRICKLE4	1.524830216	-2.139151368	1.23E-15	1.28E-14
PRRT3	1.05683654	0.624710474	1.25E-15	1.31E-14
CROCCP3	1.139701818	-0.007035744	1.26E-15	1.31E-14
GAREM2	2.158928317	1.152424965	1.27E-15	1.32E-14
PCDH17	1.373965189	2.556332358	1.29E-15	1.34E-14
AL450992.2	1.51763109	0.997337357	1.31E-15	1.36E-14
PDZRN4	-2.282748346	-1.630940985	1.31E-15	1.37E-14
MPP3	1.876414314	1.008527872	1.33E-15	1.38E-14
AC005785.1	1.527314052	-0.999162332	1.37E-15	1.42E-14
ANO2	3.011104092	0.005790892	1.37E-15	1.42E-14
ADGRA3	-1.183881287	5.72839105	1.37E-15	1.42E-14
PINK1	-1.024621334	5.158302828	1.39E-15	1.44E-14
POC1A	1.108681894	3.233921947	1.40E-15	1.45E-14
AC132872.1	1.243733376	0.981370773	1.42E-15	1.47E-14
FANCC	-1.055142892	4.027984085	1.42E-15	1.47E-14
SEMA6C	1.498152294	4.090351776	1.45E-15	1.50E-14
P3H4	1.530258473	3.579227192	1.48E-15	1.53E-14
LOXHD1	2.645956559	-2.434464823	1.53E-15	1.58E-14
ISL2	3.882771983	-1.314624526	1.54E-15	1.59E-14
MIOX	4.116284067	0.089888183	1.56E-15	1.61E-14
TMEM266	2.051444462	0.382806588	1.57E-15	1.62E-14
FBXL19-AS1	1.551926354	0.176606578	1.58E-15	1.62E-14
RHBDL1	1.634211559	1.08984067	1.58E-15	1.63E-14
AC245128.3	-1.994514685	-2.778721927	1.58E-15	1.63E-14
LINC01194	7.118495268	-1.179251633	1.60E-15	1.64E-14
RPSAP70	-1.197560376	1.19413297	1.61E-15	1.65E-14
DEPDC7	-1.228203713	4.677485974	1.61E-15	1.65E-14
LINC01767	-1.582340073	2.587349254	1.67E-15	1.72E-14
SPAG5	1.342447349	4.646249076	1.69E-15	1.74E-14
FO538757.1	1.017738996	0.759387438	1.71E-15	1.75E-14
MPEG1	-1.13860192	4.606793449	1.74E-15	1.78E-14
SPDYA	1.343961218	-1.564682061	1.74E-15	1.78E-14
MSS51	1.24994481	-0.455562855	1.74E-15	1.79E-14
AL078581.1	-1.110851759	-2.213766742	1.74E-15	1.79E-14
HIST2H4A	1.999746857	-1.43408167	1.78E-15	1.82E-14
RFLNA	4.523447661	1.207541163	1.78E-15	1.82E-14
PHACTR3	-2.162690972	-2.045406937	1.85E-15	1.89E-14
RASGRP4	-1.060929055	0.131611696	1.87E-15	1.91E-14
PELI2	-1.481142864	1.082844341	1.92E-15	1.95E-14
ALAS1	-1.241848374	8.189352655	1.94E-15	1.98E-14
KRT16P1	-3.891403074	-2.246465145	1.96E-15	2.00E-14
AC008764.8	1.237440089	-1.42322177	1.97E-15	2.00E-14
AP000769.1	2.600457249	0.827758101	1.98E-15	2.01E-14
TINAG	6.995992693	2.099434381	2.04E-15	2.07E-14
AC010331.1	1.698779525	-1.560611716	2.07E-15	2.10E-14
SYTL5	-1.633984287	0.984958586	2.14E-15	2.17E-14
ZNF337-AS1	1.033583847	1.089894411	2.15E-15	2.17E-14
PES1P1	5.91548998	-2.627995911	2.16E-15	2.19E-14
DCAF4L2	9.374409481	3.083262319	2.23E-15	2.25E-14
MT1H	-2.932561106	5.371614848	2.25E-15	2.27E-14
TSSK5P	2.224086215	-1.848097359	2.26E-15	2.29E-14
FAM171A2	2.094511751	0.291781711	2.27E-15	2.29E-14
DACH2	5.015268181	-2.270025531	2.27E-15	2.29E-14

TMEM150B	2.22226307	3.228324347	2.31E-15	2.33E-14
DRP2	3.692906886	-2.656591068	2.37E-15	2.38E-14
LRRC19	-2.190720245	-0.461970735	2.44E-15	2.45E-14
AL592424.1	1.934023983	-0.653857037	2.45E-15	2.46E-14
IL10	-1.580624851	-1.647165177	2.46E-15	2.47E-14
AC016394.1	1.465070278	-0.532610043	2.52E-15	2.52E-14
PROZ	-1.594941967	4.662336003	2.53E-15	2.54E-14
GPC2	2.064245153	-1.492112616	2.57E-15	2.57E-14
KCNE5	3.117577316	-0.269108279	2.58E-15	2.58E-14
AJ003147.3	1.612506669	-2.486880288	2.62E-15	2.61E-14
ELF3-AS1	1.694092126	1.023034063	2.64E-15	2.64E-14
AP000347.1	1.518096717	-0.923722524	2.66E-15	2.66E-14
LINGO1	2.539302549	2.727345035	2.67E-15	2.67E-14
AP003057.1	-1.528902699	-2.711223912	2.69E-15	2.68E-14
SLC9A3	4.008927953	1.556712211	2.70E-15	2.69E-14
TDO2	-1.808812332	7.898743361	2.74E-15	2.73E-14
PRRT2	1.579025106	-0.794764017	2.96E-15	2.94E-14
ZNF300	2.077684974	1.178204382	3.00E-15	2.98E-14
HMOX1	-1.386484683	6.181820416	3.00E-15	2.98E-14
AC124016.1	1.389622063	-1.961066715	3.00E-15	2.98E-14
F9	-1.885682076	7.976294962	3.01E-15	2.99E-14
SLC6A9	2.109271734	2.206180423	3.05E-15	3.03E-14
MYO15B	1.414960224	5.531034002	3.05E-15	3.03E-14
LINC01011	1.069107684	0.058743675	3.06E-15	3.03E-14
RHBDL3	4.221043536	0.963686789	3.07E-15	3.04E-14
REG1A	10.375688	6.898353877	3.08E-15	3.05E-14
CERS1	4.349128677	-0.403466257	3.14E-15	3.10E-14
IL1RAPL2	-2.061741778	-1.175217873	3.17E-15	3.13E-14
LRRC69	1.930763395	-0.522657374	3.24E-15	3.19E-14
RGS5	1.435096843	5.464650953	3.24E-15	3.19E-14
ASB9P1	3.388257116	-1.629834015	3.26E-15	3.21E-14
RET	-2.240894344	1.263686426	3.26E-15	3.21E-14
LRRC37A3	1.394147976	1.386957444	3.27E-15	3.22E-14
ALDH4A1	-1.071820776	8.221400597	3.32E-15	3.26E-14
DCST1-AS1	1.465569749	0.4638091	3.34E-15	3.28E-14
FIGNL1	1.10064759	2.641627481	3.37E-15	3.30E-14
AC024560.2	2.481691286	-1.714901555	3.41E-15	3.34E-14
SERPINA10	-1.167941722	7.954578291	3.45E-15	3.38E-14
TMEM132A	2.485708924	3.658362573	3.46E-15	3.39E-14
SERPINI1	1.900093548	2.604751678	3.46E-15	3.39E-14
RAB20	-1.00560102	4.769275901	3.47E-15	3.39E-14
SPART	-1.368996151	3.092638324	3.53E-15	3.45E-14
TMEM154	-1.77523668	1.814121901	3.54E-15	3.46E-14
LINC00893	1.442695688	-0.218457535	3.54E-15	3.46E-14
ATP2A1-AS1	2.277375123	-1.869591214	3.55E-15	3.47E-14
LPIN2	-1.056022094	7.504028714	3.57E-15	3.48E-14
MIR2052HG	4.58999011	-2.389552421	3.58E-15	3.49E-14
SYN3	2.620032189	-0.621288523	3.59E-15	3.50E-14
PEX11G	-1.120697408	3.096971553	3.60E-15	3.51E-14
STEAP1B	3.27204996	-1.75481434	3.62E-15	3.52E-14
ZNF213	1.019604983	2.64733199	3.62E-15	3.52E-14
APOL1	-1.031851828	7.755905064	3.65E-15	3.55E-14
LINC02365	4.538380864	1.592859821	3.66E-15	3.56E-14
BRCA2	1.329314964	1.489212185	3.72E-15	3.61E-14
ADH1B	-1.797736525	10.90040659	3.77E-15	3.65E-14
NEB	2.920281188	4.197441252	3.79E-15	3.67E-14
C5AR1	-1.121799425	3.022920753	3.88E-15	3.76E-14
PHGDH	-1.517872252	6.379695178	3.88E-15	3.76E-14

FER1L4	2.935341023	1.462158759	3.99E-15	3.86E-14
SEZ6L2	3.436289358	4.961604239	4.05E-15	3.91E-14
WNT3A	7.761797082	1.001239592	4.07E-15	3.93E-14
F2RL3	2.298605471	1.870500247	4.11E-15	3.97E-14
FABP5	1.672678631	2.50260454	4.17E-15	4.02E-14
RFC3	1.067999951	2.902792848	4.20E-15	4.05E-14
TMCO3	1.260155287	5.153099059	4.21E-15	4.06E-14
LINC01611	6.334987823	-2.093556081	4.31E-15	4.14E-14
LINC02466	5.37869922	-3.035510695	4.41E-15	4.24E-14
MTMR11	1.920547556	3.48885236	4.48E-15	4.31E-14
AC004263.1	2.155920606	-2.485334127	4.59E-15	4.40E-14
AC016885.3	5.920130953	-1.877295523	4.64E-15	4.44E-14
FGG	-1.398959506	12.6634294	4.64E-15	4.44E-14
AC073585.1	2.875766837	-2.489048857	4.66E-15	4.46E-14
GPD1	-1.629918369	6.460111148	4.70E-15	4.50E-14
AC098934.1	1.841456579	2.30871782	4.93E-15	4.71E-14
DHTKD1	-1.038055284	7.253023272	5.02E-15	4.79E-14
DCAF4L1	2.466715288	-1.069612477	5.09E-15	4.86E-14
AC139100.1	1.980182726	-0.588440576	5.14E-15	4.90E-14
DNASE1	1.063950272	2.505091104	5.18E-15	4.94E-14
CDNF	-1.013090874	1.26960229	5.30E-15	5.05E-14
AL163953.1	3.696664832	-1.939014922	5.30E-15	5.05E-14
FBXW10	4.164086552	0.315400277	5.31E-15	5.05E-14
NECTIN1	1.235395875	3.936369862	5.34E-15	5.07E-14
TPBGL	3.031780854	0.086979631	5.37E-15	5.10E-14
TRIM16L	2.416389952	4.47562888	5.38E-15	5.11E-14
PCSK1	6.925349066	3.501886558	5.47E-15	5.19E-14
TRIM54	4.305351005	0.550040648	5.47E-15	5.19E-14
WDR97	1.539981727	0.855362436	5.48E-15	5.20E-14
AC079061.1	-2.143822359	0.527277811	5.49E-15	5.20E-14
CXCL2	-1.571254549	5.351356027	5.53E-15	5.23E-14
AL121658.1	1.766368581	-2.539942036	5.53E-15	5.24E-14
CYP17A1	4.215351551	6.034087849	5.56E-15	5.26E-14
UNC13A	3.623255266	-0.083907745	5.61E-15	5.31E-14
AL731684.1	4.91771819	-2.225709991	5.77E-15	5.44E-14
DCAF8L2	7.385378573	-0.971416107	5.77E-15	5.44E-14
AL162413.1	6.692512855	-1.238441237	5.78E-15	5.45E-14
DSCR8	7.627885065	0.432473764	5.82E-15	5.49E-14
SCG2	3.60542462	-1.454229893	5.82E-15	5.49E-14
OSBP2	1.719616989	1.959479389	5.92E-15	5.57E-14
GOLGA6L9	1.180933557	0.276261246	5.94E-15	5.58E-14
TCHH	-1.697978609	-1.55679562	6.05E-15	5.68E-14
TEK	-1.383468973	2.657374314	6.06E-15	5.69E-14
BOLA2B	1.669789582	-1.028364962	6.11E-15	5.74E-14
HOXD10	4.30269798	-1.330635109	6.30E-15	5.91E-14
MSI1	3.47579139	2.67596246	6.33E-15	5.93E-14
CCL4	-1.379861014	2.33027092	6.39E-15	5.99E-14
LINC01186	2.797650926	-1.126003942	6.43E-15	6.02E-14
ABLIM3	-1.055610226	6.032229945	6.51E-15	6.09E-14
TKT	1.497395701	8.068278203	6.60E-15	6.17E-14
HEY1	1.320853681	2.387509045	6.64E-15	6.21E-14
AC011337.1	1.620508247	-2.512418029	6.67E-15	6.23E-14
FOXF1	-1.367027093	1.027802943	6.85E-15	6.39E-14
OXLD1	1.001881107	4.183395154	7.14E-15	6.66E-14
AC092809.2	-1.705090683	-2.265671867	7.18E-15	6.70E-14
LPAL2	-1.262316325	2.010613359	7.25E-15	6.75E-14
AL021807.1	2.327259364	-1.621109124	7.37E-15	6.86E-14
AC245100.4	1.492110786	-0.535358664	7.49E-15	6.97E-14

AL358072.1	1.686326371	-2.925047836	7.56E-15	7.04E-14
LGI1	-2.510429242	-2.044098578	7.59E-15	7.06E-14
PHOSPHO1	-1.194656012	-1.922481541	7.62E-15	7.09E-14
AC145285.6	1.263266714	-1.692654644	7.90E-15	7.34E-14
AL359313.1	5.713081436	-1.935186236	7.95E-15	7.39E-14
GNB1L	1.013550829	2.319579642	8.04E-15	7.47E-14
NME1	1.174307942	5.750333588	8.17E-15	7.58E-14
AC107419.1	4.471475436	-2.305102242	8.31E-15	7.70E-14
GPR35	2.991923943	2.214601998	8.38E-15	7.76E-14
TBX15	-1.779790676	4.239000748	8.48E-15	7.84E-14
GBP2	1.379098848	6.305725612	8.62E-15	7.97E-14
SLC39A10	1.257386949	3.144324565	8.67E-15	8.01E-14
RTBDN	5.216152022	-0.791482084	8.70E-15	8.03E-14
HIST1H3D	2.202894997	-0.667843751	8.76E-15	8.08E-14
SHLD2P3	-1.308183146	0.102038256	8.85E-15	8.16E-14
CCDC180	1.76366275	-0.916890266	8.85E-15	8.16E-14
GIPC2	-1.296032059	3.780423085	8.95E-15	8.24E-14
HERC5	-1.3983624	3.364626951	9.12E-15	8.39E-14
AC010300.1	1.673680391	-1.031034616	9.21E-15	8.48E-14
SLC35E4	1.349325022	0.800109691	9.35E-15	8.60E-14
PRR18	-1.472757755	1.994813883	9.40E-15	8.65E-14
SRRM3	3.541509072	1.783659795	9.82E-15	9.01E-14
AC080129.2	2.035490525	-1.65421424	9.91E-15	9.10E-14
C14orf180	-2.87354565	-0.304405938	1.02E-14	9.33E-14
AC239809.3	3.734079849	-0.100364492	1.02E-14	9.35E-14
PLPPR4	2.818035717	-0.875040234	1.02E-14	9.36E-14
LINC00221	7.672808834	1.380564189	1.04E-14	9.48E-14
CD200R1	-1.247027372	-0.27784756	1.04E-14	9.56E-14
PCAT6	1.605744668	0.656396937	1.05E-14	9.59E-14
AC078909.2	2.918155643	-2.840803313	1.05E-14	9.59E-14
IGDCC3	5.959099197	0.229709331	1.07E-14	9.81E-14
AL355802.2	1.596921355	-1.701882422	1.10E-14	1.01E-13
ARFGEF3	2.952218312	2.870815885	1.11E-14	1.02E-13
REG3A	10.52369764	8.635738422	1.12E-14	1.02E-13
SRGN	-1.122135103	5.029462319	1.14E-14	1.04E-13
AC141557.1	2.380394161	-1.985079891	1.17E-14	1.07E-13
LINC01234	5.698178566	0.624486104	1.19E-14	1.08E-13
LINC01355	1.295015577	0.915594394	1.32E-14	1.20E-13
CALN1	-2.663791648	-1.990176807	1.34E-14	1.22E-13
BARD1	1.26698617	1.727923556	1.34E-14	1.22E-13
SRD5A2	-1.996060284	4.732287289	1.35E-14	1.22E-13
AC012409.1	-1.63479033	-0.294479361	1.36E-14	1.23E-13
LINC00942	6.975181474	1.440448609	1.40E-14	1.27E-13
ANXA2	1.163423807	8.141975444	1.41E-14	1.27E-13
GPR65	-1.188706217	0.918870974	1.42E-14	1.29E-13
FBXL6	1.106054343	4.76157341	1.43E-14	1.29E-13
MPZ	2.989587372	3.750684315	1.43E-14	1.30E-13
FBXL22	1.140774596	-0.852886723	1.46E-14	1.32E-13
CRHR2	1.710187562	-1.566541213	1.47E-14	1.33E-13
RN7SL2	-1.136922654	4.333140088	1.48E-14	1.33E-13
IYD	-1.605026249	4.527077702	1.48E-14	1.34E-13
KCNH4	2.254338636	-1.124909822	1.51E-14	1.36E-13
NPFFR2	6.10626423	0.213811582	1.51E-14	1.36E-13
PRDM7	3.332235877	-2.184156949	1.51E-14	1.36E-13
DDX11	1.238468476	3.342202395	1.53E-14	1.38E-13
AC245060.6	1.672024139	-0.85549793	1.56E-14	1.40E-13
PRKAR2B	-1.66606467	0.973246099	1.58E-14	1.42E-13
C8orf76	1.010046486	3.16767087	1.59E-14	1.43E-13

LUCAT1	4.079984525	0.452403826	1.62E-14	1.46E-13
AL035563.1	1.445859512	-1.382583756	1.64E-14	1.47E-13
PLIN1	-1.644210246	3.398404731	1.64E-14	1.47E-13
PSMG3	1.04108224	4.551962495	1.64E-14	1.47E-13
ACP4	4.023293326	-1.143046084	1.66E-14	1.49E-13
GOLGA2P7	1.703572197	-1.823115616	1.66E-14	1.49E-13
AC079466.1	6.330618504	4.030681876	1.67E-14	1.49E-13
CATSPER2	1.228878704	0.41470086	1.68E-14	1.51E-13
AP002807.1	1.596903867	-0.224043434	1.69E-14	1.51E-13
FGF14-AS2	-1.264864953	0.851927397	1.70E-14	1.52E-13
BDKRB1	2.713929201	-0.443762532	1.71E-14	1.53E-13
PAEP	9.414356183	3.775237616	1.71E-14	1.53E-13
PXMP2	-1.065614103	6.104939541	1.71E-14	1.53E-13
AC010894.3	4.619601208	-0.664741203	1.72E-14	1.54E-13
ATOH8	-1.676726277	4.10635033	1.73E-14	1.54E-13
CCDC154	1.890230183	-0.260275733	1.75E-14	1.56E-13
AC008894.3	1.829592204	-2.193683902	1.76E-14	1.57E-13
PRR36	2.353930258	0.873883486	1.80E-14	1.60E-13
MNS1	2.685970006	2.288633388	1.83E-14	1.63E-13
MMS22L	1.302864714	1.936264113	1.85E-14	1.65E-13
AC114401.1	4.347260679	-2.669164562	1.87E-14	1.66E-13
PCK2	-1.242586369	8.796511581	1.87E-14	1.66E-13
RASGRF1	4.110108725	1.511980944	1.90E-14	1.69E-13
AC022107.1	1.185425683	0.182670799	1.96E-14	1.74E-13
C1QL4	4.071722382	-1.307942474	2.00E-14	1.77E-13
ARHGAP27P1-I	1.049976718	0.571897616	2.00E-14	1.77E-13
RAP2A	1.016631834	4.883864367	2.01E-14	1.78E-13
DLL4	1.079775109	3.631324739	2.01E-14	1.78E-13
SNRPEP4	1.377935758	-2.109005676	2.03E-14	1.80E-13
AC092384.2	-2.290765321	-0.230456363	2.05E-14	1.81E-13
SNHG10	1.055895758	1.332823089	2.09E-14	1.84E-13
MAPK11	1.402222275	2.927254983	2.10E-14	1.85E-13
KCND3	-1.904129486	4.026031453	2.10E-14	1.85E-13
DIO2	4.362500104	1.613887053	2.12E-14	1.87E-13
CLVS1	2.787952902	-0.632768275	2.12E-14	1.87E-13
AC074212.1	1.40417032	0.550787379	2.12E-14	1.87E-13
AC090809.1	6.588374315	-1.877497396	2.17E-14	1.91E-13
RELL2	1.32231263	0.235198294	2.20E-14	1.94E-13
AP000346.2	1.959147479	-1.279652565	2.24E-14	1.98E-13
GPR146	-1.211772832	0.861631022	2.29E-14	2.02E-13
AP000593.3	5.297565599	-0.387590301	2.29E-14	2.02E-13
PRDM15	1.289647874	2.871527682	2.37E-14	2.08E-13
RNU6-8	2.035206668	-1.468474362	2.41E-14	2.12E-13
PAGE4	8.585982249	4.673202943	2.43E-14	2.13E-13
AL078590.3	-1.579414349	-1.376127127	2.43E-14	2.13E-13
SERPINA1	-1.095396856	14.37455089	2.44E-14	2.14E-13
ZNF618	1.044022285	3.705439605	2.44E-14	2.14E-13
CASKIN1	3.451670983	-1.793559935	2.50E-14	2.19E-13
TSPO2	2.150253509	-0.504036435	2.58E-14	2.26E-13
SLC7A10	6.055067872	1.093787087	2.59E-14	2.26E-13
LIN28B	9.452827987	1.105429839	2.59E-14	2.26E-13
FRMD6	-1.128426798	3.287361848	2.60E-14	2.27E-13
COBLL1	-1.070966001	6.301055695	2.61E-14	2.28E-13
C11orf80	1.246793674	2.227749189	2.62E-14	2.29E-13
RIDA	-1.234414483	8.227010415	2.63E-14	2.29E-13
FAM133A	5.35747057	1.570078237	2.70E-14	2.35E-13
ZNF99	4.279738894	-0.75652576	2.71E-14	2.36E-13
AKR1D1	-1.915703179	6.317886577	2.73E-14	2.38E-13

LINC02313	3.470476025	-2.385380979	2.76E-14	2.40E-13
AC011503.1	4.71725166	-1.451852901	2.77E-14	2.41E-13
SASS6	1.043721203	1.626974303	2.77E-14	2.41E-13
MCMDC2	1.219667804	0.55468155	2.78E-14	2.42E-13
ZNF695	4.370333806	-1.779252339	2.81E-14	2.44E-13
BNIPL	1.677075372	-0.428376632	2.83E-14	2.45E-13
MAGEC1	8.395651271	1.819674134	2.83E-14	2.46E-13
AC063948.1	1.640511175	-2.68238503	2.86E-14	2.48E-13
TTLL4	1.436329376	4.330686813	2.92E-14	2.53E-13
MAGI2-AS3	-1.683630768	3.376766185	2.94E-14	2.55E-13
AP006621.2	2.035843092	-1.278953165	2.95E-14	2.56E-13
ALPK3	2.047385993	3.937936529	2.98E-14	2.58E-13
CUZD1	3.731133198	0.284697578	3.03E-14	2.63E-13
AL117329.1	5.305978854	-2.490874479	3.06E-14	2.64E-13
PDE1C	2.594003209	-0.157465234	3.07E-14	2.65E-13
CA2	-1.34184477	5.992193826	3.08E-14	2.66E-13
EHHADH	-1.28672317	7.827364139	3.12E-14	2.69E-13
AC116407.4	1.512585051	-2.114327899	3.12E-14	2.69E-13
AC090192.2	5.224917229	-2.305971721	3.17E-14	2.73E-13
AC078993.1	4.335373354	-0.185575882	3.18E-14	2.74E-13
DUSP13	5.401362225	-1.33160013	3.19E-14	2.75E-13
AC012313.8	1.575125256	-1.925181206	3.25E-14	2.79E-13
CHGA	6.966720762	2.149171591	3.28E-14	2.81E-13
AC073195.1	1.329899458	-1.677021976	3.28E-14	2.81E-13
AMOTL2	-1.039809728	4.70066969	3.35E-14	2.88E-13
C8orf59	1.006473312	4.807507133	3.42E-14	2.93E-13
LHX2	-1.733074632	0.423008265	3.43E-14	2.94E-13
CCDC3	-1.242518449	3.850240222	3.46E-14	2.96E-13
FAM24B	2.10231219	-0.939744572	3.65E-14	3.12E-13
UNKL	1.044792951	2.889905718	3.67E-14	3.13E-13
SPRED3	1.820287842	-0.933752468	3.78E-14	3.22E-13
SPOCD1	2.698516176	-0.634700583	3.80E-14	3.24E-13
C7	-2.066161117	6.603581067	3.83E-14	3.27E-13
CCDC170	2.519342477	2.935381208	3.86E-14	3.29E-13
ACTG2	1.869169902	2.469654524	3.90E-14	3.32E-13
HIST1H2BG	2.175382503	-0.276739287	3.93E-14	3.34E-13
TNFSF4	1.754744527	1.712327195	3.95E-14	3.36E-13
MYBPHL	5.445070419	-0.848751897	3.97E-14	3.37E-13
LINC02580	-1.546057887	-1.961938667	3.97E-14	3.37E-13
AC245884.8	1.471424184	-1.612853318	3.97E-14	3.37E-13
LINC01762	2.456035217	-2.250043478	3.99E-14	3.39E-13
AC012236.1	2.477751108	0.55358759	4.04E-14	3.43E-13
AL133410.1	1.808922714	-2.122448331	4.09E-14	3.46E-13
CALY	4.02488216	-1.032054278	4.12E-14	3.49E-13
ZNF681	2.135140639	0.561682646	4.13E-14	3.49E-13
LINC02367	2.084154759	-1.760218196	4.14E-14	3.50E-13
ZNF883	2.791572809	-0.05540384	4.17E-14	3.52E-13
CRYGS	1.96152879	1.240971041	4.17E-14	3.52E-13
FAM81A	2.067808902	-0.019052446	4.28E-14	3.61E-13
HIST2H2BF	2.015634455	-0.867487165	4.34E-14	3.65E-13
TRIM60P18	1.453631787	-1.030326612	4.37E-14	3.68E-13
TAL2	2.517219069	-2.524854481	4.38E-14	3.69E-13
FAM163B	-2.326409527	0.085631137	4.45E-14	3.74E-13
ZNF37BP	1.035907141	2.158402239	4.47E-14	3.76E-13
RNVU1-6	2.225342749	-2.868770995	4.47E-14	3.76E-13
DDX53	7.237422033	-0.848795287	4.52E-14	3.79E-13
C2orf48	2.312994645	-1.884742912	4.67E-14	3.91E-13
AL359921.2	1.213781962	-0.263218923	4.72E-14	3.96E-13

CYP4F2	-1.601548244	6.847034234	4.74E-14	3.97E-13
AL031985.3	1.030477875	0.562980204	4.75E-14	3.97E-13
BRSK2	3.425499487	-0.055178032	4.79E-14	4.00E-13
DLX1	5.271022152	-1.026837132	4.81E-14	4.01E-13
GLUD1	-1.066988514	8.982022449	4.84E-14	4.04E-13
GAPLINC	3.085640741	-0.206951245	4.86E-14	4.05E-13
PNMA3	3.660625322	2.345750475	4.87E-14	4.06E-13
MCF2L2	2.946301087	-0.002951772	4.95E-14	4.13E-13
CLDN15	1.813469774	5.36068728	4.98E-14	4.15E-13
MIR600HG	1.412839996	1.003822123	5.04E-14	4.19E-13
FAXDC2	-1.151905916	6.354174854	5.04E-14	4.19E-13
ZMIZ1-AS1	1.821345653	-0.186568408	5.04E-14	4.20E-13
STXBP4	1.055907134	2.622030205	5.04E-14	4.20E-13
SERTAD4-AS1	2.968581173	-0.7898393	5.09E-14	4.23E-13
HIST1H4E	2.438723477	-2.261484436	5.17E-14	4.29E-13
LRRK2	-1.215670008	2.661832503	5.18E-14	4.30E-13
AC145343.1	1.736393753	-1.097778837	5.28E-14	4.39E-13
SLC22A11	4.269173157	4.088675943	5.37E-14	4.45E-13
AC011290.2	1.606898008	-2.309050803	5.38E-14	4.46E-13
HAVCR1	5.130786661	0.396205109	5.49E-14	4.55E-13
NCOA7	-1.063601892	5.222331607	5.50E-14	4.55E-13
CCDC188	1.348255195	-2.20785191	5.59E-14	4.62E-13
TMEM198	1.434502369	2.528793655	5.60E-14	4.63E-13
ALPL	-1.592554177	5.735900805	5.68E-14	4.69E-13
AC106900.2	4.390013666	-2.487492719	5.72E-14	4.72E-13
PPIAP77	2.321684455	-1.508121116	5.74E-14	4.74E-13
HOXC9	5.638427218	-0.576008385	5.90E-14	4.87E-13
SYP	1.785743289	1.124883224	5.98E-14	4.93E-13
AL391056.1	4.404649674	0.547243867	6.12E-14	5.04E-13
NR6A1	1.256772961	2.590242871	6.21E-14	5.11E-13
FER1L6	6.06602826	1.01687797	6.25E-14	5.14E-13
CEND1	2.251717793	-1.993893624	6.28E-14	5.16E-13
AL390728.6	1.292212317	1.983205134	6.42E-14	5.27E-13
NRIP2	1.155117889	1.370497726	6.53E-14	5.35E-13
FOLH1	-1.313332573	3.665421438	6.57E-14	5.39E-13
ITGA2	1.970823258	2.989110617	6.59E-14	5.40E-13
LINC02377	6.253299803	-1.638088245	6.63E-14	5.43E-13
PHEX	3.164103958	-0.270115267	6.70E-14	5.48E-13
AC007066.2	1.067018772	-0.567268664	6.70E-14	5.48E-13
AMDHD1	-1.355839676	6.242375356	6.72E-14	5.50E-13
STEAP4	-1.68512258	4.265391317	6.86E-14	5.60E-13
LINC01572	2.142243512	-2.353076284	6.89E-14	5.63E-13
AL353807.3	1.288513473	-1.911915483	6.90E-14	5.63E-13
UCN2	3.883313863	-2.920585399	7.00E-14	5.71E-13
AC011498.6	1.722210644	-2.835163377	7.02E-14	5.73E-13
CCNO	3.20245021	0.586684269	7.03E-14	5.73E-13
BX842568.2	-1.691864802	0.24014193	7.06E-14	5.75E-13
SNX22	1.82030535	3.400852877	7.11E-14	5.80E-13
AC034236.2	1.041161501	-0.209846022	7.19E-14	5.86E-13
LZTS1	1.393533057	2.14124031	7.24E-14	5.89E-13
MINCR	1.524994274	2.088839907	7.27E-14	5.91E-13
AC012146.1	1.550542388	1.411284331	7.33E-14	5.96E-13
HOXA11	5.430754035	-1.729819338	7.36E-14	5.99E-13
CXCL17	4.406397759	1.113171013	7.63E-14	6.19E-13
ADAM11	2.285429196	-0.368595313	7.72E-14	6.27E-13
TRIM17	3.396045351	-0.667466941	7.98E-14	6.47E-13
AC138696.2	1.532815348	-0.87719712	8.04E-14	6.51E-13
SLC34A3	3.001550262	-2.583206367	8.21E-14	6.64E-13

FSD1L	1.158835467	0.891370812	8.44E-14	6.83E-13
LINC02428	-1.967349272	2.374088662	8.47E-14	6.85E-13
LINC01136	2.471618479	-2.10358081	8.50E-14	6.87E-13
MT-ATP8	-1.135598572	9.087260345	8.70E-14	7.02E-13
MIR194-2HG	1.401410003	0.608001792	8.70E-14	7.02E-13
BRIP1	1.415964563	2.127495087	8.77E-14	7.07E-13
AC007639.1	4.173672432	-1.470575341	8.77E-14	7.07E-13
AP003419.3	2.088294568	-2.219680504	8.79E-14	7.08E-13
HIST1H2BN	1.53039852	0.997729977	8.83E-14	7.11E-13
MTND6P4	-1.586787402	-0.448875805	8.85E-14	7.13E-13
PEG10	4.583285942	7.339791265	8.99E-14	7.24E-13
VCX	5.837561156	-1.798810921	9.06E-14	7.29E-13
LIMK1	1.210626811	3.919143046	9.25E-14	7.45E-13
GATM	-1.197653489	9.938974679	9.31E-14	7.49E-13
GLOD5	-1.500543484	0.819159329	9.52E-14	7.65E-13
CTD-2201I18.1	1.791073484	-0.736727253	9.53E-14	7.66E-13
ABCB5	6.296171811	0.281805411	9.63E-14	7.73E-13
RGS18	-1.252227919	-0.037056714	9.72E-14	7.79E-13
LRRC4C	-1.723602726	-1.135052554	9.80E-14	7.86E-13
AC098820.1	1.461875704	-1.113667986	9.90E-14	7.93E-13
NKD1	3.941694367	4.972725768	9.94E-14	7.96E-13
ALDH3A1	5.250784831	7.172742975	9.96E-14	7.97E-13
HCG27	1.246677449	-0.165213632	1.00E-13	8.03E-13
PFKFB2	1.208466802	3.57383239	1.01E-13	8.05E-13
TSPEAR-AS2	2.715186615	1.211554737	1.02E-13	8.15E-13
DDX39B	1.005889388	5.275476673	1.03E-13	8.24E-13
STRA6	3.72234209	-0.32636991	1.05E-13	8.35E-13
AC242376.2	1.676292829	-1.097737425	1.05E-13	8.39E-13
LINC02588	5.714879297	-1.926828499	1.05E-13	8.40E-13
LINC02335	5.540451817	-2.414912768	1.07E-13	8.49E-13
HGH1	1.04648144	5.464885113	1.08E-13	8.57E-13
SQLE	1.629963683	6.414247657	1.09E-13	8.66E-13
FOXH1	4.126018097	-1.373081662	1.09E-13	8.70E-13
Z97634.1	1.327272468	-1.220184982	1.10E-13	8.70E-13
RHBDF2	1.053354836	4.294598968	1.10E-13	8.74E-13
AC109830.1	5.273704904	-2.8877886	1.12E-13	8.88E-13
CDK5R1	1.27118671	0.882869179	1.14E-13	9.02E-13
LINC01389	1.863223996	-1.291647151	1.14E-13	9.02E-13
ODAM	7.576387904	2.471073941	1.15E-13	9.13E-13
C18orf54	1.398518666	0.358939808	1.16E-13	9.17E-13
AC008610.1	1.721138653	-1.412480961	1.18E-13	9.32E-13
GSEC	1.317374322	0.698475996	1.18E-13	9.32E-13
CASP17P	1.182741656	0.860196026	1.18E-13	9.37E-13
SLC4A11	2.761457872	0.78666438	1.19E-13	9.42E-13
VWF	1.547539787	6.167814365	1.19E-13	9.42E-13
AL080317.1	1.072475381	0.414657534	1.19E-13	9.44E-13
CMTM4	1.271153561	3.455250341	1.20E-13	9.46E-13
AC092910.3	1.264989072	-1.215868352	1.20E-13	9.47E-13
DPY19L2P2	2.020873134	-1.123053751	1.20E-13	9.51E-13
LAGE3	1.118311256	4.438051363	1.22E-13	9.61E-13
PRAME	6.765145168	2.989807224	1.22E-13	9.61E-13
CAMK2N2	2.38568629	1.08977465	1.23E-13	9.71E-13
CEMIP	2.625283976	2.376291418	1.25E-13	9.88E-13
ECEL1	6.43562381	3.212757739	1.25E-13	9.88E-13
RHEBL1	1.141149146	-0.436270559	1.26E-13	9.93E-13
AC109460.3	1.226406343	-1.282457742	1.27E-13	1.00E-12
DBN1	1.731076209	4.539187696	1.30E-13	1.02E-12
S100A10	1.245370456	6.950115347	1.31E-13	1.03E-12

FHAD1	2.141071654	-0.413684977	1.32E-13	1.03E-12
ZNF71	1.092876274	1.878741777	1.33E-13	1.05E-12
ATP2A1	1.543788645	-0.295610662	1.36E-13	1.07E-12
AC006077.2	1.614407101	-1.447758516	1.38E-13	1.08E-12
PPP4R3C	7.943412821	0.42112699	1.39E-13	1.09E-12
C1QTNF6	1.188634501	4.048752193	1.44E-13	1.12E-12
ZMYND10	1.519344944	-0.388858933	1.45E-13	1.14E-12
BPIFA2	6.385898093	-1.399803449	1.47E-13	1.14E-12
KPNA7	2.133047593	0.253440872	1.49E-13	1.16E-12
AC106820.3	1.664519285	-1.514446714	1.49E-13	1.16E-12
CLEC2L	6.514366837	-0.032742836	1.50E-13	1.17E-12
TRAM1L1	2.951551743	-0.27762541	1.50E-13	1.17E-12
ELL3	1.177199933	-1.385263725	1.51E-13	1.18E-12
COL22A1	4.61965561	2.216327864	1.51E-13	1.18E-12
AL671710.1	1.097167711	-0.883365316	1.51E-13	1.18E-12
AC020904.2	1.510822407	-1.498749381	1.51E-13	1.18E-12
AC021321.1	1.820655957	-2.803894861	1.51E-13	1.18E-12
HEXA-AS1	1.760467278	-1.76768844	1.52E-13	1.18E-12
SLC27A2	-1.329880942	7.336327371	1.53E-13	1.19E-12
ALPI	8.32735974	3.358533387	1.53E-13	1.19E-12
SCML2	1.330946994	1.57803417	1.53E-13	1.19E-12
CSKMT	1.064771603	0.920353501	1.55E-13	1.21E-12
AL807752.1	1.644678104	-2.396756726	1.56E-13	1.21E-12
GGT5	-1.418752278	4.80861272	1.58E-13	1.22E-12
PLCB1	1.53776976	3.769768808	1.60E-13	1.24E-12
PFKFB4	1.615972185	1.862887171	1.62E-13	1.26E-12
KLF13	1.055156119	5.78291469	1.65E-13	1.28E-12
IL6	-1.964760653	-0.267970554	1.65E-13	1.28E-12
AC084018.1	1.550276671	-0.901625939	1.69E-13	1.30E-12
LINC00906	5.375864013	-2.615089062	1.69E-13	1.30E-12
BRINP2	-1.922475705	-2.812148404	1.69E-13	1.31E-12
CGREF1	1.9599066	4.532528906	1.70E-13	1.31E-12
AC027796.4	1.761772084	-1.182172297	1.74E-13	1.34E-12
AC007364.1	-1.218292697	-1.170907442	1.74E-13	1.34E-12
LAPTM4B	1.542957368	6.686041507	1.77E-13	1.36E-12
DNM1P35	1.941250195	-1.037387633	1.80E-13	1.38E-12
SLC16A4	-1.585131619	2.108423179	1.82E-13	1.40E-12
AL160314.2	1.564093409	-2.513392784	1.82E-13	1.40E-12
GLYAT	-1.973439573	6.551615661	1.84E-13	1.41E-12
FXYD1	-1.856207375	4.532818883	1.84E-13	1.41E-12
AC109460.1	1.264715251	-2.276346321	1.86E-13	1.43E-12
HIST1H3H	1.996588731	1.661306041	1.87E-13	1.43E-12
SHBG	-1.785269524	5.213926492	1.90E-13	1.45E-12
AL591895.1	1.166294158	1.561396732	1.90E-13	1.46E-12
KLRD1	-1.29460048	0.892255528	1.91E-13	1.46E-12
CD69	-1.388347199	1.353019426	1.91E-13	1.46E-12
SHOX2	3.019908646	-1.305970112	1.93E-13	1.48E-12
ASPG	-2.075611282	5.261917418	1.93E-13	1.48E-12
P3H2	-1.366871119	2.086756152	1.94E-13	1.48E-12
AC087741.3	1.38689423	-1.416486936	1.96E-13	1.50E-12
MORF4L2-AS1	1.260432549	-1.835448527	1.99E-13	1.52E-12
CDKN2B	1.315575111	2.810932678	2.00E-13	1.52E-12
CBR3	1.920981441	0.489666738	2.09E-13	1.59E-12
AL032819.2	3.799064436	-0.577969784	2.10E-13	1.60E-12
AL121772.1	2.086350455	-1.973955167	2.11E-13	1.61E-12
AL355987.4	2.289044086	-0.117361643	2.13E-13	1.62E-12
FBXO32	1.669064983	3.505604514	2.13E-13	1.62E-12
DNAH17	1.517674426	0.391162088	2.17E-13	1.65E-12

HNRNPA1P16	1.257962753	-0.989566678	2.18E-13	1.66E-12
CKLF	1.026968306	2.505503025	2.19E-13	1.66E-12
LINC01943	1.713881246	-1.111172415	2.21E-13	1.68E-12
ZNF607	1.563015516	1.177693612	2.22E-13	1.68E-12
GLP1R	6.208448946	-0.988616778	2.23E-13	1.69E-12
BAGE2	6.715562273	-1.337245567	2.27E-13	1.72E-12
PKN1	1.0448284	6.620142803	2.30E-13	1.74E-12
LAMA3	2.312951351	4.665591879	2.33E-13	1.77E-12
SLC9A3-AS1	2.111365114	3.406118325	2.34E-13	1.77E-12
MT-ND4L	-1.19467861	9.630082693	2.35E-13	1.78E-12
ACOX2	-1.141415747	6.752282394	2.36E-13	1.79E-12
SP5	2.502369924	3.201205692	2.39E-13	1.80E-12
AC009533.1	1.148552702	1.332473931	2.44E-13	1.85E-12
AC112178.1	5.981069288	-1.965467956	2.45E-13	1.85E-12
AC008870.2	1.212483701	-1.75569007	2.49E-13	1.88E-12
AC023090.1	5.11120084	-1.855660187	2.50E-13	1.88E-12
GRPR	4.04282365	-0.037962676	2.53E-13	1.91E-12
TSPAN7	-1.069058799	3.649141022	2.54E-13	1.91E-12
ADGRB3	-1.901477588	-1.617552229	2.57E-13	1.93E-12
E2F5	1.163943734	2.258420743	2.66E-13	2.00E-12
AL356234.2	3.426461972	-1.313754945	2.77E-13	2.08E-12
PAQR6	1.560089344	1.056561124	2.81E-13	2.10E-12
AC002116.2	1.433513542	-1.849018455	2.89E-13	2.16E-12
PPP1R14D	4.143296055	-0.269650843	2.90E-13	2.17E-12
KIRREL2	7.680110915	1.638082426	2.91E-13	2.18E-12
AC012358.2	1.014564355	-0.664265874	2.91E-13	2.18E-12
AFF2	4.772302864	0.259117998	2.94E-13	2.20E-12
MEGF10	-1.915002803	-1.735702506	2.96E-13	2.21E-12
ZP3	1.606141831	0.929820461	2.98E-13	2.23E-12
AC108748.1	-1.628534067	-2.593029791	3.05E-13	2.28E-12
CCER2	1.749776136	-1.089857841	3.07E-13	2.29E-12
FAM198A	-1.852681819	2.208261011	3.08E-13	2.29E-12
AC009509.4	-1.185502793	-2.370755491	3.10E-13	2.31E-12
HES2	3.008029026	-0.42561269	3.11E-13	2.32E-12
FANCA	1.02269283	2.883559156	3.13E-13	2.33E-12
NKX3-2	4.387354863	-2.103469255	3.18E-13	2.36E-12
AC114786.2	5.552172743	-0.250451203	3.19E-13	2.37E-12
TMEM82	-1.749689319	4.30304702	3.19E-13	2.37E-12
PALM2-AKAP2	-1.576053158	-1.063508585	3.19E-13	2.37E-12
LINC02323	3.904413276	-2.151057277	3.19E-13	2.37E-12
ZNF385D	3.881626941	2.110730745	3.21E-13	2.38E-12
IGHA1	-2.014399991	6.931554753	3.23E-13	2.40E-12
IGDCC4	3.100271532	0.227105686	3.29E-13	2.44E-12
SPATC1	1.571953821	-1.501048069	3.29E-13	2.44E-12
CYP2W1	2.840016305	-1.250789368	3.32E-13	2.47E-12
TMEM136	1.34252746	1.372357534	3.33E-13	2.47E-12
KAZN	-1.507231265	1.527576615	3.40E-13	2.52E-12
SPSB1	-1.014774798	5.488874111	3.41E-13	2.53E-12
SERPINH1	1.076304527	6.43927945	3.42E-13	2.53E-12
MIR4664	2.099461045	-1.471690507	3.43E-13	2.54E-12
MAGEB2	9.160161946	1.350188817	3.50E-13	2.59E-12
INHA	4.719195772	1.015897015	3.51E-13	2.60E-12
GABARAPL1	-1.068566657	6.558427978	3.56E-13	2.63E-12
AL139023.1	5.249252572	-2.298614698	3.57E-13	2.64E-12
AC138393.1	1.071183392	0.555143119	3.60E-13	2.66E-12
CEACAM20	5.076078903	1.263056853	3.63E-13	2.68E-12
ANKRD34A	1.094131322	-0.729875835	3.78E-13	2.79E-12
TEAD2	1.375855445	4.128290777	3.79E-13	2.80E-12

PLXNA3	1.401795917	3.701994224	3.80E-13	2.80E-12
ZG16	-1.76959438	3.814241506	3.84E-13	2.83E-12
AC147055.1	3.399642533	-0.326253483	3.86E-13	2.84E-12
KCNJ5	2.132885657	2.319345839	3.89E-13	2.87E-12
AL590705.1	2.478078226	-1.170770699	3.90E-13	2.87E-12
MAGEA6	8.034788856	2.746311864	3.90E-13	2.87E-12
AC011468.1	1.334321308	-0.521399864	3.93E-13	2.89E-12
AKAP3	-1.314332476	-0.608762331	3.97E-13	2.92E-12
AC245100.3	9.155204532	1.669372283	4.03E-13	2.96E-12
SYT3	4.765292094	1.057637645	4.08E-13	3.00E-12
DNER	6.919057195	1.58336803	4.09E-13	3.00E-12
PNMA5	6.613654643	1.544179996	4.10E-13	3.01E-12
RFPL4B	7.321581684	-0.101729538	4.11E-13	3.02E-12
AC002128.1	1.39947334	-1.853768502	4.19E-13	3.07E-12
LINC01748	5.098273794	-1.598085346	4.23E-13	3.10E-12
ACTL8	7.383032085	0.83188223	4.23E-13	3.10E-12
AC100803.3	1.995977984	-1.892758348	4.27E-13	3.12E-12
AL135999.1	1.313124737	0.827929374	4.27E-13	3.13E-12
FER1L5	2.70968402	-1.746772189	4.28E-13	3.13E-12
PSCA	3.902464243	-1.050626765	4.39E-13	3.20E-12
ASPDH	-1.603022396	5.691855297	4.43E-13	3.24E-12
HMGA2	5.836678958	1.423707707	4.49E-13	3.28E-12
LINC01126	1.801968665	-1.87556866	4.49E-13	3.28E-12
F11	-1.207884267	6.019885636	4.54E-13	3.31E-12
ODF3L1	-1.494530764	-0.68670622	4.61E-13	3.36E-12
AL080317.3	1.269480898	0.064085312	4.63E-13	3.37E-12
LINC01667	7.600398992	0.261819792	4.67E-13	3.40E-12
RNF24	1.006608937	2.80763225	4.72E-13	3.43E-12
HOXC6	4.635706737	-1.027540728	4.72E-13	3.43E-12
MIR25	1.537819641	-2.175060535	4.74E-13	3.45E-12
AL138847.2	-1.316522965	2.982863804	4.76E-13	3.46E-12
GJA5	1.513679671	2.764516962	4.77E-13	3.47E-12
GADD45G	-1.376195703	6.261262194	4.78E-13	3.47E-12
FBN3	3.465494979	1.14448931	4.85E-13	3.53E-12
DPYD	-1.010890172	5.581472492	4.87E-13	3.54E-12
AL136537.2	6.198181609	-1.988442306	4.90E-13	3.56E-12
ANKRD65	2.485296815	2.719674562	4.93E-13	3.58E-12
STMND1	3.192694806	-0.085992069	4.95E-13	3.59E-12
AQP7P3	-1.947101073	-2.623583947	5.02E-13	3.64E-12
MYMX	3.173826708	-1.267857026	5.02E-13	3.64E-12
STRA8	3.897226776	-2.072806353	5.03E-13	3.64E-12
TNNC1	3.028817607	1.335266479	5.11E-13	3.70E-12
AL109933.4	-1.58665414	-2.280020308	5.13E-13	3.71E-12
SLC45A4	1.980313015	3.15352262	5.14E-13	3.72E-12
PABPC4L	1.441443172	-1.164251394	5.21E-13	3.76E-12
PCDHGA1	2.36938658	-0.561264056	5.33E-13	3.85E-12
AC106822.1	-1.841646991	-2.240466849	5.34E-13	3.86E-12
HAO2-IT1	-2.11684884	-2.641786299	5.44E-13	3.92E-12
GOLGA2P5	1.253776586	3.662032368	5.45E-13	3.93E-12
STEAP3-AS1	-1.394114407	-2.300945278	5.54E-13	3.99E-12
KCNQ3	2.074939984	-0.951214027	5.74E-13	4.13E-12
ADCY10P1	1.24206742	1.13382296	5.76E-13	4.14E-12
PTOV1-AS2	1.077183774	0.394566128	5.80E-13	4.17E-12
LINC01612	-3.10010094	-1.368239807	5.95E-13	4.28E-12
AC027601.1	1.075483696	-0.54280904	5.98E-13	4.29E-12
BFSP2	2.919711154	-1.954741693	5.99E-13	4.30E-12
FRRS1L	5.377385042	0.813046518	6.03E-13	4.33E-12
APLP1	2.936723575	1.895590273	6.09E-13	4.37E-12

AKR1B10	3.613498433	8.99727795	6.11E-13	4.38E-12
CCSAP	1.028558134	2.872263739	6.17E-13	4.42E-12
FAP	2.362225375	1.126801922	6.26E-13	4.48E-12
AKR1C7P	2.15892694	-1.420100392	6.27E-13	4.49E-12
ESCO2	1.645470905	1.010653839	6.28E-13	4.49E-12
CPNE7	2.700353764	1.431184271	6.33E-13	4.53E-12
SMPD5	1.855153835	-1.318343129	6.36E-13	4.55E-12
PFN1P11	4.206943681	0.694101628	6.37E-13	4.55E-12
CYP4A22-AS1	1.923802256	-2.842506871	6.46E-13	4.61E-12
ANKFN1	8.225446171	2.053108196	6.47E-13	4.62E-12
DPPA4	-1.905371705	-2.181867584	6.55E-13	4.68E-12
ZNF530	1.123268988	0.879558124	6.60E-13	4.70E-12
ARHGAP22	1.402005681	0.603341564	6.71E-13	4.78E-12
TGFBR3	-1.140039609	4.617494306	6.74E-13	4.80E-12
TECTA	-1.082945638	-0.250996508	6.74E-13	4.80E-12
TPSP2	2.159974554	0.63783858	6.96E-13	4.95E-12
MFGE8	1.188052743	4.490600767	7.07E-13	5.02E-12
AC005253.1	1.107067565	-1.045252907	7.15E-13	5.08E-12
DIRAS1	3.498310209	0.409380991	7.16E-13	5.08E-12
PCDHB2	2.914215242	0.31422127	7.18E-13	5.09E-12
STC2	2.042768996	3.218537109	7.26E-13	5.15E-12
AL118511.2	3.352890997	-2.73065218	7.44E-13	5.27E-12
TCAM1P	4.740001437	-1.106382278	7.66E-13	5.42E-12
DMC1	2.290180516	-1.210461592	7.68E-13	5.43E-12
C9orf163	1.392381515	-1.681008107	7.83E-13	5.53E-12
DPF1	2.372212399	-2.32569603	7.84E-13	5.53E-12
CFAP53	1.36799938	-0.73530981	7.87E-13	5.56E-12
TBKBP1	1.053143567	2.705333609	7.90E-13	5.57E-12
AC122688.3	1.629445824	-1.31848519	7.92E-13	5.59E-12
ASNSP1	6.331150083	-0.369479717	7.96E-13	5.61E-12
DTNA	1.744621978	3.97670749	8.10E-13	5.70E-12
AC006486.2	1.378082646	-1.723584483	8.21E-13	5.77E-12
HIST1H3E	1.689010013	-0.694478277	8.23E-13	5.79E-12
FLJ12825	2.777622069	-2.552583766	8.32E-13	5.85E-12
LRRC10B	1.678007816	-1.127474012	8.39E-13	5.89E-12
AC005225.4	-1.088705969	0.424692731	8.39E-13	5.89E-12
POPDC3	6.205152955	-0.041381889	8.43E-13	5.92E-12
ANKRD29	1.690945226	2.89335679	8.44E-13	5.92E-12
ZNRD1ASP	1.079768771	1.048287691	8.50E-13	5.96E-12
FTCD	-1.401308183	8.456931532	8.52E-13	5.97E-12
PKM	1.801386568	7.235732256	8.59E-13	6.01E-12
VSIG10L	2.072734816	3.026305254	8.70E-13	6.09E-12
CNTD1	1.014297439	-0.524996028	8.75E-13	6.12E-12
KCNN1	2.547740605	-1.436153315	8.84E-13	6.18E-12
ENO3	-1.627970485	5.312359621	8.95E-13	6.25E-12
ALS2CL	1.395000573	4.754953525	8.97E-13	6.26E-12
ACTBP11	1.872200212	-2.214156242	9.02E-13	6.30E-12
UGT2B10	-1.613472522	7.842617301	9.14E-13	6.38E-12
VAMP1	1.039829816	2.378474653	9.24E-13	6.44E-12
UNC5A	2.814039639	-0.353856274	9.33E-13	6.50E-12
CRLF1	2.609867051	1.320238954	9.40E-13	6.55E-12
AC025171.4	1.608184963	-1.67160323	9.41E-13	6.55E-12
EIF4E3	-1.166042675	3.320572838	9.81E-13	6.82E-12
SNORA60	1.583996568	-2.91480349	9.83E-13	6.83E-12
AL031673.1	1.233101319	-0.365496529	9.88E-13	6.87E-12
AC007773.1	1.68541063	-0.702257453	1.01E-12	7.01E-12
OR2B6	2.236514766	-1.965678431	1.03E-12	7.13E-12
KCNU1	7.38770004	1.579012193	1.03E-12	7.14E-12

C19orf57	1.346756558	1.169893419	1.05E-12	7.26E-12
KDM4A-AS1	1.1670169	-0.476609702	1.05E-12	7.26E-12
CARD18	5.156403335	-2.696217483	1.05E-12	7.30E-12
LINC01719	1.574497069	0.17187485	1.09E-12	7.55E-12
AC091182.1	2.170105848	-1.243131348	1.09E-12	7.56E-12
ZSWIM5	1.948850434	2.683622845	1.10E-12	7.59E-12
AC106820.5	2.035400595	-2.69240471	1.12E-12	7.73E-12
PYCR3	1.011999673	4.728857463	1.13E-12	7.78E-12
AC005077.2	-2.195260542	-2.222804596	1.14E-12	7.85E-12
RHPN1-AS1	1.469955966	-0.37027916	1.14E-12	7.90E-12
AL158819.1	6.321069829	-2.051072561	1.15E-12	7.94E-12
BPIFB4	4.122901281	-2.054579494	1.18E-12	8.09E-12
IRX3	2.505306271	2.164115678	1.18E-12	8.10E-12
KCNJ15	-1.416726648	0.753868991	1.19E-12	8.16E-12
GOLGA2P10	1.26496239	0.329137828	1.20E-12	8.24E-12
ADAMTSL2	-1.51180477	4.656337592	1.20E-12	8.24E-12
SLC27A5	-1.515953811	8.990845338	1.20E-12	8.28E-12
AC007277.1	3.816020806	-1.572586543	1.21E-12	8.33E-12
FCN1	-1.310296291	1.820244644	1.22E-12	8.38E-12
AL008729.1	1.332240778	-1.309250205	1.24E-12	8.48E-12
DNAH12	2.690034532	-0.185699803	1.24E-12	8.51E-12
KLHL31	1.248489843	-0.018108096	1.26E-12	8.64E-12
TESC	2.749552753	4.608579076	1.26E-12	8.66E-12
HSP90AB3P	1.291770135	-0.703332508	1.28E-12	8.75E-12
IFI27L1	1.03691227	3.145089907	1.29E-12	8.83E-12
AC104966.1	-1.407992029	0.631845117	1.29E-12	8.85E-12
AL049830.3	2.947344655	-2.858190055	1.31E-12	8.92E-12
FAR2P1	6.792135992	0.273830753	1.31E-12	8.92E-12
RAET1E	1.780426872	-2.682915803	1.31E-12	8.92E-12
LAMB4	2.77051446	-1.267826382	1.31E-12	8.97E-12
EEF1A2	3.932506567	6.303536079	1.33E-12	9.09E-12
SRPX	-1.765342735	2.346716923	1.34E-12	9.17E-12
INPP5J	2.065578594	0.113773694	1.35E-12	9.23E-12
LINC01607	1.877322494	-1.354296162	1.37E-12	9.32E-12
LILRB1	-1.169701322	1.829749019	1.39E-12	9.45E-12
CCDC102B	1.265710469	1.514218294	1.39E-12	9.48E-12
FHDC1	1.978092403	0.324795801	1.40E-12	9.53E-12
AC092957.1	5.508562304	-2.399509202	1.41E-12	9.60E-12
STPG3-AS1	1.908275871	0.267675679	1.43E-12	9.73E-12
PAIP2B	-1.388621353	3.976522906	1.45E-12	9.82E-12
SPATA3-AS1	2.646285704	-2.744005279	1.46E-12	9.92E-12
MAGEA12	7.687460004	2.111337463	1.46E-12	9.93E-12
CEP83-DT	1.426993888	-2.28446878	1.49E-12	1.01E-11
MDFI	2.382761458	1.537600195	1.49E-12	1.01E-11
GDPD1	1.170909417	1.159623101	1.50E-12	1.01E-11
TXNRD1	1.394867648	7.28844545	1.51E-12	1.02E-11
LPCAT1	1.397104038	5.171175986	1.52E-12	1.03E-11
PYCR1	2.499855263	4.169987239	1.54E-12	1.04E-11
F11-AS1	-1.177473251	2.749585619	1.55E-12	1.05E-11
BX276092.7	7.880076007	-0.938580477	1.56E-12	1.05E-11
SNHG21	1.008417973	-0.752379322	1.57E-12	1.06E-11
CTD-2297D10.2	5.523880731	-0.853669779	1.58E-12	1.06E-11
CNIH2	1.645743722	-0.899746254	1.60E-12	1.08E-11
ZNF385C	1.899905218	0.315877979	1.61E-12	1.09E-11
AC098934.2	1.941889302	0.317781459	1.63E-12	1.10E-11
AL031123.2	1.36321831	-0.16551229	1.63E-12	1.10E-11
CIART	1.504590562	2.751856906	1.65E-12	1.11E-11
PCDH9-AS2	-3.059189055	-2.416602454	1.67E-12	1.12E-11

GEMIN7-AS1	1.147831569	-2.168181861	1.69E-12	1.14E-11
S100A1	2.658397669	3.164456127	1.70E-12	1.14E-11
PPP2R2C	4.694646294	1.694926347	1.71E-12	1.15E-11
MTND5P11	-1.586469874	-0.193384714	1.72E-12	1.15E-11
MIR647	1.504276495	-1.643359196	1.72E-12	1.15E-11
AC092117.1	1.522237631	-0.899566838	1.72E-12	1.16E-11
LY6G5B	1.37499123	0.01690973	1.73E-12	1.16E-11
AL512770.1	1.227669138	-1.38112688	1.73E-12	1.16E-11
KAZALD1	1.736197253	1.439223253	1.75E-12	1.17E-11
BREA2	1.387728437	-1.885607808	1.75E-12	1.17E-11
MAGEA3	7.113802462	3.000138296	1.75E-12	1.17E-11
AC108751.4	1.797506917	-1.410444979	1.77E-12	1.18E-11
PLA2G4C-AS1	1.714350705	-2.537038096	1.80E-12	1.20E-11
MT1G	-2.491775749	8.572657576	1.82E-12	1.22E-11
AC026401.3	1.136832925	1.253074729	1.83E-12	1.23E-11
MAGEA8	6.779288512	-0.231464176	1.84E-12	1.23E-11
FHL2	-1.222699876	3.594212172	1.84E-12	1.23E-11
EGF	4.729469904	0.207363306	1.84E-12	1.23E-11
AC009137.2	-1.16556075	-2.694274555	1.84E-12	1.23E-11
PNCK	6.047195041	1.894657059	1.85E-12	1.23E-11
CNTN4	-1.362342485	0.795028114	1.87E-12	1.25E-11
SLC22A8	7.077577629	-0.084759088	1.88E-12	1.25E-11
AL031665.2	1.274697504	-2.082276707	1.90E-12	1.26E-11
AZGP1P2	-1.623522404	-1.616827096	1.90E-12	1.26E-11
LMNB1	1.216410635	4.352250045	1.91E-12	1.27E-11
AC119424.1	-1.679786869	-1.628562784	1.91E-12	1.27E-11
AC025254.1	6.84036137	-1.868443999	1.92E-12	1.28E-11
SSTR5-AS1	6.390553122	2.221514926	1.93E-12	1.28E-11
SP8	6.282006927	-1.157376658	1.95E-12	1.29E-11
TMEM253	1.580033821	-2.481685216	1.96E-12	1.30E-11
CTNNA2	5.839952348	2.861474465	2.03E-12	1.35E-11
ATP5MF-PTCD1	1.562188797	-2.707304061	2.04E-12	1.35E-11
SNHG6	1.345304823	5.642171123	2.11E-12	1.39E-11
DMBX1	4.896103483	-1.895633003	2.11E-12	1.40E-11
ADAMTS16	3.678603971	1.647682951	2.13E-12	1.41E-11
SELP	-1.511210571	1.160374586	2.15E-12	1.42E-11
BEND3	1.144306615	1.73359497	2.15E-12	1.42E-11
AC090559.1	-1.275554155	-1.24153187	2.16E-12	1.43E-11
AC009065.4	1.198752843	-0.841410784	2.17E-12	1.43E-11
RMST	3.645539917	-1.45598416	2.17E-12	1.43E-11
AC090164.2	2.577299129	-1.532093383	2.17E-12	1.43E-11
AC099343.2	1.416017307	-2.335641734	2.20E-12	1.45E-11
LYG1	1.133193708	-0.468347784	2.20E-12	1.45E-11
FABP4	2.736443819	3.519426052	2.21E-12	1.46E-11
FAM229A	1.044019903	0.969752423	2.23E-12	1.47E-11
LINC01225	2.835266179	-2.323046609	2.25E-12	1.48E-11
KCNH6	4.894345658	-0.190075415	2.29E-12	1.50E-11
AC092803.2	1.074923598	0.630963112	2.29E-12	1.51E-11
SLC16A2	-1.172558555	6.188020911	2.29E-12	1.51E-11
TNFRSF18	1.929510499	0.607434535	2.31E-12	1.51E-11
AC112484.1	1.613741062	-1.349205036	2.34E-12	1.53E-11
NTM	2.942424943	0.846106016	2.34E-12	1.53E-11
DRC1	2.228977158	-2.395621263	2.37E-12	1.55E-11
TAS2R4	1.47534346	-2.430781218	2.38E-12	1.56E-11
ANKS1B	2.64777516	0.340792803	2.39E-12	1.56E-11
SMKR1	2.81553766	-1.554696155	2.39E-12	1.56E-11
AC068987.4	1.848493433	1.440440476	2.40E-12	1.57E-11
LY6E-DT	-1.808309701	-0.073593877	2.41E-12	1.57E-11

PTK7	2.210731294	3.615254494	2.41E-12	1.57E-11
SULT1A1	-1.132418117	6.521295327	2.46E-12	1.61E-11
ZNF433-AS1	1.036247925	0.443327863	2.50E-12	1.63E-11
DNM3OS	-1.333199529	-0.319723284	2.52E-12	1.65E-11
AC245595.1	2.353158304	-2.081759284	2.54E-12	1.66E-11
AL121772.3	1.595540414	-2.628831755	2.55E-12	1.66E-11
CAVIN4	1.679700264	-1.424701324	2.55E-12	1.66E-11
TTR	-1.518420437	11.27921155	2.56E-12	1.67E-11
BBOX1-AS1	4.632646959	-1.566524403	2.58E-12	1.68E-11
RNF126P1	2.54066683	-2.297931541	2.61E-12	1.70E-11
LINC00235	1.458491051	-0.936883485	2.61E-12	1.70E-11
FLNC	3.274384836	4.286406142	2.65E-12	1.72E-11
C19orf81	4.658374726	-1.624678762	2.69E-12	1.75E-11
AC007038.2	1.063912858	-1.315727469	2.70E-12	1.75E-11
AL357079.1	1.395307465	-2.273714378	2.72E-12	1.76E-11
TENT5C	-1.176312877	3.500118546	2.73E-12	1.77E-11
LINC01608	6.461463181	-0.726347475	2.74E-12	1.78E-11
DPT	-2.025255383	2.87456365	2.74E-12	1.78E-11
ARTN	1.745522802	-1.254118639	2.77E-12	1.80E-11
PCDHA1	4.858829697	-0.49297663	2.81E-12	1.82E-11
CYP2J2	-1.115849471	6.296628353	2.87E-12	1.85E-11
KIF5A	3.027680536	-2.033180825	2.88E-12	1.86E-11
AL355574.1	1.335172365	-1.893114705	2.88E-12	1.86E-11
FHL1	-1.090447306	4.232431376	2.90E-12	1.87E-11
AC007611.1	1.19529489	-1.375979095	2.90E-12	1.87E-11
AL023806.3	1.71883008	-2.910423788	2.90E-12	1.87E-11
CASS4	-1.053183505	0.773013357	2.95E-12	1.90E-11
AC073611.1	1.187166463	-1.208059833	2.95E-12	1.90E-11
LINC02404	6.664342798	-0.593637473	2.99E-12	1.93E-11
EPHB2	2.515554447	3.277164573	3.06E-12	1.97E-11
LINC00607	2.414145374	0.166403639	3.06E-12	1.97E-11
CAPN9	2.651203925	-0.972323875	3.11E-12	2.00E-11
C1orf61	3.111211032	-0.603870666	3.12E-12	2.01E-11
MICE	1.441198188	-0.752650976	3.25E-12	2.09E-11
NAT8L	3.480196278	0.822358678	3.26E-12	2.10E-11
GPR82	-1.332353887	-1.685148111	3.28E-12	2.11E-11
PRSS2	9.249702142	2.564015367	3.32E-12	2.13E-11
KIAA1211L	1.31052368	1.173038277	3.35E-12	2.15E-11
MCCD1	6.217648565	0.993955652	3.35E-12	2.15E-11
TNXB	-1.402899173	3.134409124	3.36E-12	2.16E-11
AL138762.1	1.36987253	-1.954384652	3.36E-12	2.16E-11
AC069224.1	1.340586194	-1.145818258	3.37E-12	2.16E-11
DHRS1	-1.026640234	6.122495666	3.41E-12	2.18E-11
FOLH1B	-2.055446616	1.869606227	3.43E-12	2.20E-11
APOA5	-1.500984821	8.061437398	3.45E-12	2.21E-11
AC010333.2	4.015323471	-2.964543715	3.45E-12	2.21E-11
ALDOB	-1.547420935	12.06296093	3.46E-12	2.21E-11
AC087164.2	-1.807473746	1.31883046	3.46E-12	2.22E-11
AC099568.2	1.448986097	-1.682663465	3.48E-12	2.23E-11
TEX11	4.046537059	0.236841333	3.50E-12	2.24E-11
CHRND	5.610064627	-1.083145161	3.51E-12	2.25E-11
FAM162B	1.437877531	-0.46775067	3.57E-12	2.28E-11
RNF180	-1.166185572	1.680171437	3.60E-12	2.30E-11
CA4	3.225933092	0.360239829	3.61E-12	2.30E-11
SOX6	-1.373430527	3.052034794	3.62E-12	2.31E-11
ZFHX2-AS1	1.183121133	-0.825459723	3.62E-12	2.31E-11
MYBL1	1.213354057	1.857866806	3.62E-12	2.31E-11
AC245052.4	1.089795635	-0.606239685	3.64E-12	2.32E-11

APC2	1.044872328	1.388290177	3.66E-12	2.33E-11
GRIN2C	1.196421864	-0.862684064	3.67E-12	2.33E-11
COLEC11	-1.438224449	5.427723198	3.68E-12	2.34E-11
SORT1	1.204559573	5.525188046	3.69E-12	2.34E-11
MMP12	4.163721087	1.212840358	3.69E-12	2.35E-11
HSD17B13	-2.211476566	7.537025704	3.71E-12	2.36E-11
AC092667.1	2.241582279	-1.98151085	3.76E-12	2.39E-11
BLVRA	1.245372036	4.17581622	3.77E-12	2.39E-11
AC010333.1	4.169997635	-3.000705771	3.79E-12	2.40E-11
AC092171.3	1.21975531	0.928779738	3.81E-12	2.42E-11
OR51E2	2.913308874	-2.742609886	3.83E-12	2.43E-11
AL359513.1	1.587583734	-1.696217848	3.86E-12	2.44E-11
AC015813.1	1.242303642	1.921772929	3.88E-12	2.46E-11
FAM182B	2.070580701	-1.925687649	3.90E-12	2.47E-11
PHBP11	1.901729984	-1.204762806	3.93E-12	2.49E-11
CAPN8	2.768844457	0.81561771	3.95E-12	2.50E-11
TMIGD3	-1.237845918	1.576131517	3.96E-12	2.51E-11
SMPX	3.273290728	0.006886571	3.98E-12	2.52E-11
RNU6-850P	2.05706362	-2.961351022	3.99E-12	2.52E-11
DMBT1	6.088002762	2.199611887	4.01E-12	2.54E-11
LINC01480	3.4531614	0.22629242	4.04E-12	2.55E-11
AL353801.3	1.646372441	-2.817717809	4.13E-12	2.60E-11
PAGE1	9.29952067	2.455666508	4.18E-12	2.64E-11
AP000525.1	2.631609909	-2.545049828	4.20E-12	2.64E-11
HSD17B6	-1.379485755	8.842828357	4.23E-12	2.66E-11
AL137026.2	4.333779235	-2.776741273	4.28E-12	2.69E-11
INHBC	-1.376407888	5.607630123	4.32E-12	2.72E-11
AL157778.1	5.559365796	-2.468639851	4.33E-12	2.72E-11
CDC20B	4.161267336	-1.074353635	4.33E-12	2.72E-11
AL139130.1	3.600100128	-2.780570133	4.34E-12	2.72E-11
PART1	7.042084076	0.534298199	4.36E-12	2.74E-11
OGDHL	-1.422276947	6.641591852	4.37E-12	2.74E-11
AC106771.1	5.354318869	-2.816886598	4.38E-12	2.74E-11
MIR548XHG	6.530959734	-1.667305396	4.46E-12	2.80E-11
FBLIM1	1.325738799	4.46638686	4.58E-12	2.87E-11
DRGX	5.336570662	-1.497480255	4.63E-12	2.90E-11
NCKAP5	-1.27849216	1.434427612	4.67E-12	2.92E-11
CASC20	5.800634161	-1.943634477	4.67E-12	2.92E-11
PADI3	7.860100009	1.259850781	4.69E-12	2.93E-11
AC112907.3	1.231165031	-1.821248684	4.69E-12	2.93E-11
GOLGA8A	1.734107986	2.521455538	4.79E-12	2.99E-11
AC145207.5	1.085499078	0.641043869	4.79E-12	2.99E-11
HTR1D	4.296656326	1.347064173	4.81E-12	3.00E-11
FLJ45513	1.430410682	-0.632279913	4.81E-12	3.00E-11
GCM1	3.635726285	-2.531696527	4.82E-12	3.01E-11
AC069277.1	5.095968739	-2.089329131	4.86E-12	3.03E-11
SMYD2	1.018008657	5.692315113	5.04E-12	3.14E-11
EXTL1	2.525443806	-1.781058812	5.10E-12	3.17E-11
AF279873.3	6.654139539	-1.193591973	5.13E-12	3.19E-11
CST1	7.560575968	3.537699063	5.14E-12	3.20E-11
BRINP3	6.004165613	-1.307123183	5.17E-12	3.21E-11
CCL2	-1.383558487	3.95511258	5.17E-12	3.21E-11
AC124242.1	-1.178213189	-1.505801021	5.18E-12	3.22E-11
LINC01108	2.908967074	-1.57136222	5.21E-12	3.24E-11
BCAT1	1.710674438	2.624931761	5.22E-12	3.24E-11
SRARP	5.238756094	0.367549357	5.25E-12	3.26E-11
CDRT1	3.125145755	-1.286342428	5.28E-12	3.27E-11
SLAIN1	-1.584421324	1.434272499	5.29E-12	3.28E-11

GBP1	-1.053348235	5.698514446	5.40E-12	3.34E-11
NRG2	2.319864679	0.289974444	5.51E-12	3.41E-11
BORA	1.045790944	0.564692001	5.52E-12	3.42E-11
SGK1	-1.140153457	6.016583266	5.54E-12	3.43E-11
AL136295.6	1.051267582	-1.398429571	5.65E-12	3.49E-11
LINC00106	1.450351116	-0.268578578	5.66E-12	3.50E-11
CEACAM3	-1.559843005	-2.618113175	5.74E-12	3.54E-11
Z95331.1	1.171966488	-1.216142079	5.92E-12	3.65E-11
AC073529.1	1.375589608	-2.252396716	6.12E-12	3.77E-11
SMIM32	4.60752734	2.054489911	6.28E-12	3.87E-11
ADGRB2	2.838325338	0.207595318	6.29E-12	3.87E-11
HIST1H1PS1	2.569196484	-2.708657249	6.34E-12	3.90E-11
C12orf75	2.159321239	3.464219125	6.43E-12	3.95E-11
HIC2	1.25709153	2.64091203	6.43E-12	3.96E-11
MAP7D2	3.668695595	1.700943514	6.56E-12	4.03E-11
ZNF738	1.59702709	0.277590877	6.60E-12	4.05E-11
IMPDH1P6	2.021092653	-2.055010534	6.64E-12	4.08E-11
NAA11	7.774036018	-0.362315945	6.65E-12	4.08E-11
C12orf56	4.316839228	-0.939417114	6.66E-12	4.09E-11
PLA2G4C	1.238150187	4.293656544	6.72E-12	4.12E-11
HYI-AS1	1.417810432	-1.92295415	6.75E-12	4.13E-11
LINC02438	3.415488201	-2.85066245	6.77E-12	4.15E-11
OTOG	6.307460292	-0.338130726	6.78E-12	4.15E-11
ADAM23	3.134035917	1.59221169	6.98E-12	4.27E-11
AL031777.1	2.186136894	-1.467394503	6.99E-12	4.27E-11
IRX6	5.390521892	-1.582150854	7.07E-12	4.32E-11
AC010595.1	5.255737511	-2.574843219	7.10E-12	4.34E-11
LINC01977	2.963124685	-1.870861959	7.19E-12	4.39E-11
CHST6	2.693777984	-1.907490579	7.19E-12	4.39E-11
CDC25B	1.004564493	5.533738187	7.19E-12	4.39E-11
LINC00944	2.793202889	-0.548588585	7.22E-12	4.40E-11
LINC00491	5.644010839	-2.646251045	7.39E-12	4.50E-11
AC068831.1	1.253107479	-2.507318355	7.48E-12	4.55E-11
STRCP1	3.178837249	-0.796682539	7.63E-12	4.64E-11
AKR1B15	3.862569678	2.44970969	7.70E-12	4.68E-11
SATB1	-1.089552348	3.418764328	7.71E-12	4.69E-11
FGF17	3.30276411	-0.998978498	7.76E-12	4.72E-11
KREMEN2	2.339196118	-1.307422299	7.80E-12	4.73E-11
PRDM9	3.790151931	-2.752634868	7.92E-12	4.81E-11
IGHA2	-1.850984571	3.635664789	7.97E-12	4.84E-11
AC010542.5	1.214179644	-1.68257722	7.98E-12	4.84E-11
PFN4	1.529083329	-0.912393079	8.05E-12	4.88E-11
AP005057.1	3.4258019	-2.139576365	8.05E-12	4.88E-11
PLP2	1.507127354	5.071658093	8.08E-12	4.90E-11
AL928654.1	1.14603998	-0.01616438	8.11E-12	4.91E-11
VSIG1	4.036012572	1.711260009	8.34E-12	5.04E-11
AC113404.1	2.975970742	-1.885042995	8.34E-12	5.04E-11
AC122710.1	5.616177951	-2.457334021	8.39E-12	5.07E-11
SLC12A5-AS1	2.487397545	-1.357109431	8.42E-12	5.09E-11
AC003072.1	1.028518675	-0.552200045	8.42E-12	5.09E-11
IGF2BP2	2.280460287	3.879805232	8.43E-12	5.09E-11
AC015909.5	1.498733837	-2.809681305	8.45E-12	5.09E-11
AP002478.1	5.231971152	-2.365048908	8.51E-12	5.13E-11
STAG3L5P	1.105528998	1.720393293	8.62E-12	5.19E-11
GDPD3	1.523888001	0.497358119	8.68E-12	5.23E-11
MICB	1.266754609	1.995481564	8.68E-12	5.23E-11
GABBR2	4.41832435	1.793311168	8.74E-12	5.26E-11
CLCNKA	2.616624837	-0.344597964	8.79E-12	5.29E-11

AL117336.3	1.098551151	0.01596422	8.82E-12	5.30E-11
CSF1R	-1.0543556	4.777632728	8.91E-12	5.36E-11
EGFL8	1.197995862	-0.389127615	9.06E-12	5.44E-11
CLDN18	4.219301241	-0.58578845	9.11E-12	5.48E-11
AOC1	-2.07401809	1.812711113	9.26E-12	5.56E-11
AC004076.2	1.368928199	-1.609747768	9.26E-12	5.56E-11
CRYBG1	-1.017618627	3.875506172	9.49E-12	5.70E-11
LINC02159	4.560732755	-1.765090697	9.51E-12	5.70E-11
PPP1R16A	1.140851192	6.536440875	9.57E-12	5.73E-11
AC012617.1	2.375149313	-2.713965962	9.64E-12	5.77E-11
EIF4HP2	1.570001732	-2.072967943	9.69E-12	5.80E-11
SIGLEC14	-1.721204859	0.227003637	9.70E-12	5.80E-11
PPEF1	2.238440381	-2.403233701	9.83E-12	5.88E-11
FCGR2B	-1.708549713	2.727890082	1.01E-11	6.02E-11
AC241584.1	2.040916744	-2.744171387	1.01E-11	6.03E-11
COL7A1	2.736798146	4.208625251	1.02E-11	6.07E-11
PDZK1P1	1.673463191	-1.845376238	1.02E-11	6.08E-11
PDGFRL	1.577263061	0.653712282	1.02E-11	6.09E-11
ALDOA	1.102650247	8.500472399	1.02E-11	6.11E-11
PRDM12	1.813737626	-2.307052842	1.03E-11	6.14E-11
CDA	-1.371748963	4.253952798	1.03E-11	6.16E-11
AC048341.1	1.070419265	0.189242179	1.07E-11	6.35E-11
PGLYRP2	-1.561576546	7.374085725	1.08E-11	6.43E-11
ZNF57	1.189609205	1.663290631	1.10E-11	6.55E-11
NAALADL2	-1.28970052	1.682964174	1.10E-11	6.55E-11
CSPG4P12	1.571244137	-2.204439897	1.12E-11	6.65E-11
KIF20B	1.000726685	2.303975022	1.13E-11	6.71E-11
RNF224	2.674289585	-2.926187067	1.14E-11	6.74E-11
MAEL	3.957599562	1.341263047	1.17E-11	6.95E-11
AC005150.1	7.864553534	-0.510873103	1.18E-11	6.96E-11
TNNI2	2.591862187	0.86118773	1.18E-11	6.98E-11
LTO1	2.050100044	4.155425971	1.18E-11	7.00E-11
CALML6	1.78202148	-1.026249636	1.21E-11	7.15E-11
MPDZ	-1.100622219	5.69681992	1.21E-11	7.15E-11
KLHL23	1.010180478	3.741595935	1.21E-11	7.17E-11
GTSF1	4.227299502	2.317354796	1.21E-11	7.17E-11
AGBL4	-2.029720754	-1.413398679	1.22E-11	7.19E-11
MEFV	-1.089403008	-1.171519202	1.23E-11	7.27E-11
LINC00672	1.335178869	-1.05811174	1.24E-11	7.33E-11
LINC00624	2.758200983	-0.023483295	1.26E-11	7.39E-11
SAA2-SAA4	-2.295019012	5.780905845	1.26E-11	7.43E-11
FCGR3A	-1.184802816	4.954641608	1.27E-11	7.46E-11
AC025171.5	1.529803737	-2.330132193	1.27E-11	7.47E-11
AC015802.4	1.277877124	-1.916566145	1.29E-11	7.56E-11
AL139220.2	1.958810652	-1.969195507	1.29E-11	7.57E-11
POU3F2	3.467014041	-2.094693495	1.29E-11	7.60E-11
GJA10	6.775406585	-1.353433223	1.30E-11	7.62E-11
GPR180	-1.033626925	3.409110828	1.30E-11	7.64E-11
RGN	-1.08094351	7.248406079	1.30E-11	7.65E-11
DKK4	5.068515436	2.328323263	1.31E-11	7.68E-11
PCDH9	-1.71042811	0.975465851	1.32E-11	7.74E-11
PP7080	1.317119806	3.296609077	1.32E-11	7.74E-11
TRPM8	-1.486273351	4.598900138	1.34E-11	7.86E-11
ALX3	5.660163891	-2.304731615	1.35E-11	7.93E-11
FEZF1	4.861650974	-2.059787384	1.37E-11	8.01E-11
AC036108.2	2.319851074	-1.169232221	1.39E-11	8.15E-11
CCL26	3.03093623	-1.477233253	1.41E-11	8.23E-11
BGN	-1.243646176	7.878922983	1.45E-11	8.48E-11

RPL22L1	1.433017513	5.266864909	1.45E-11	8.48E-11
HPDL	2.252093029	-1.123572115	1.46E-11	8.50E-11
RPSAP7	1.726417568	-2.025936601	1.46E-11	8.50E-11
AC007292.1	1.178328703	-0.532645846	1.48E-11	8.62E-11
CDH19	-2.046744958	0.854700562	1.48E-11	8.62E-11
CPB2	-1.046194214	9.053256942	1.48E-11	8.63E-11
JCHAIN	-1.861953058	4.385423055	1.49E-11	8.66E-11
AC008735.2	1.329947358	-0.46860213	1.49E-11	8.67E-11
MIR4292	1.578317653	-2.80062379	1.49E-11	8.69E-11
AP005230.1	3.89373643	-3.000662929	1.52E-11	8.84E-11
GABRR3	3.91292949	-0.577192645	1.53E-11	8.89E-11
FCRL6	-1.195550759	-0.316535204	1.54E-11	8.93E-11
AC107959.3	2.157641188	-1.435636132	1.54E-11	8.97E-11
SPERT	4.957895339	-2.718265714	1.55E-11	9.01E-11
ZNF716	5.642870042	-1.30959657	1.57E-11	9.09E-11
LTK	-1.433506395	0.527213674	1.58E-11	9.19E-11
AP002360.3	1.698153225	-2.322391966	1.63E-11	9.47E-11
OR2I1P	2.094755017	5.731540733	1.64E-11	9.50E-11
RGS20	2.474758541	-2.341706569	1.66E-11	9.60E-11
AC108062.1	1.220506812	0.207545717	1.66E-11	9.62E-11
AC112493.1	3.738878687	-2.648097438	1.67E-11	9.68E-11
GLS2	-1.969835581	3.325514649	1.68E-11	9.71E-11
FAM155B	2.653483283	0.561245732	1.68E-11	9.72E-11
AC092171.5	1.25013766	0.386166451	1.68E-11	9.72E-11
PDGFRB	1.152084974	5.896064785	1.69E-11	9.75E-11
AC012676.1	1.388259244	-0.898548995	1.73E-11	9.98E-11
AC022497.1	1.601440728	-2.119451545	1.73E-11	9.98E-11
U91324.1	-1.292393509	0.591572384	1.73E-11	1.00E-10
HIST1H2BJ	1.816810702	0.513339055	1.74E-11	1.01E-10
PLEKHG4	2.227556211	1.369099343	1.75E-11	1.01E-10
AL590652.1	2.455464859	-2.94967856	1.76E-11	1.01E-10
ATP8A2	3.047708276	-0.531371798	1.77E-11	1.02E-10
CECR7	3.943698944	-0.553814228	1.77E-11	1.02E-10
GJB2	-1.08929849	4.863549189	1.77E-11	1.02E-10
TMEM178B	2.740278696	1.002580041	1.79E-11	1.03E-10
AP006621.5	1.471903634	0.87202112	1.80E-11	1.04E-10
HIST1H2AD	1.989939326	-1.488022142	1.81E-11	1.04E-10
PLA2G16	-1.034522087	5.770526525	1.81E-11	1.04E-10
CNTNAP1	1.540270558	1.846350506	1.87E-11	1.08E-10
AC239803.3	2.687184137	-2.509071238	1.92E-11	1.10E-10
AC118344.2	1.305625136	-0.4553349	1.99E-11	1.14E-10
C2CD4B	-1.538312054	-0.002695408	2.02E-11	1.16E-10
AL109615.3	3.1252528	0.386184757	2.03E-11	1.16E-10
SLC22A4	1.924042036	1.106256245	2.05E-11	1.18E-10
DCAF8L1	7.299407791	-0.980383067	2.06E-11	1.18E-10
GCKR	-1.114180842	5.684661969	2.06E-11	1.18E-10
AC016710.1	6.25875087	-2.095989764	2.07E-11	1.19E-10
SLC46A3	-1.366835137	5.601627227	2.07E-11	1.19E-10
AC009690.1	2.260892452	-1.707384194	2.12E-11	1.21E-10
NT5M	1.291278067	1.422691886	2.13E-11	1.22E-10
LINC02492	5.472246328	-2.779340149	2.13E-11	1.22E-10
AOX1	-1.435080195	9.650700894	2.14E-11	1.23E-10
AC105411.1	2.999908917	-0.245252238	2.16E-11	1.23E-10
DNAJC12	-1.406533921	4.080058257	2.16E-11	1.23E-10
SORD	-1.091286368	7.680346212	2.17E-11	1.24E-10
FAM78B	1.256174398	1.224528078	2.19E-11	1.25E-10
AC073655.2	1.193424933	-1.826888666	2.20E-11	1.26E-10
AKR7A3	-1.476702901	6.273896686	2.22E-11	1.27E-10

AC007099.1	5.13822709	-0.411850588	2.23E-11	1.27E-10
RARRES2P8	6.522395986	-1.342076475	2.23E-11	1.27E-10
AL353708.1	1.141256837	-1.394345716	2.25E-11	1.28E-10
AC145207.8	2.498256581	-2.084610649	2.26E-11	1.29E-10
TMPRSS15	8.588368656	1.415372124	2.29E-11	1.30E-10
MYC	-1.191374548	5.785572565	2.33E-11	1.33E-10
BX649601.1	1.809662414	-1.809304104	2.35E-11	1.34E-10
AC009275.1	2.437524607	-0.398732907	2.37E-11	1.35E-10
DLX6-AS1	5.333685716	-1.027148192	2.39E-11	1.36E-10
IZUMO1	1.682891022	-2.205015545	2.40E-11	1.36E-10
UCN	1.33283123	0.204889242	2.41E-11	1.37E-10
LINC00310	-1.12604408	-2.370916995	2.51E-11	1.42E-10
RNF157-AS1	2.572242575	-0.669719149	2.51E-11	1.42E-10
EVA1A	-1.042077437	5.727497931	2.52E-11	1.42E-10
DLG2	-1.154380969	0.576442582	2.62E-11	1.48E-10
GATA3	-1.313959214	0.596137871	2.65E-11	1.50E-10
CDIPTOSP	2.369724514	-2.475381366	2.66E-11	1.50E-10
PTGIS	-1.654497395	2.764405988	2.68E-11	1.51E-10
MYCBPAP	1.73197548	-2.134391755	2.68E-11	1.52E-10
HIST1H2BO	1.912029548	-2.211940671	2.69E-11	1.52E-10
LYG2	2.462184368	-2.506138626	2.70E-11	1.53E-10
AMBP	-1.034663823	12.57290703	2.74E-11	1.54E-10
FAM178B	4.606942875	0.078072306	2.75E-11	1.55E-10
AC135586.2	-1.116319669	-2.135039958	2.75E-11	1.55E-10
AQP8	3.76218391	2.104012903	2.76E-11	1.56E-10
ADAMTS18	3.058097354	-1.731992151	2.78E-11	1.57E-10
MST1	-1.010762621	7.920037082	2.79E-11	1.57E-10
EPHA10	3.416107095	0.086840329	2.79E-11	1.57E-10
SBSPON	2.366096387	1.554697957	2.86E-11	1.61E-10
AC100872.1	2.886609419	-2.877340141	2.89E-11	1.62E-10
SH3D21	1.186123299	0.689099015	2.91E-11	1.63E-10
PTGDR	-1.181575463	-1.411005578	3.02E-11	1.69E-10
PON1	-1.372399999	8.364897714	3.04E-11	1.70E-10
DBNDD1	1.584505329	4.942309034	3.07E-11	1.72E-10
ATP6V0D2	2.926324759	0.100543149	3.07E-11	1.72E-10
MFSD6	1.204797745	3.305324031	3.08E-11	1.72E-10
HMGCS2	-1.193022172	10.32758148	3.09E-11	1.73E-10
AL669831.5	1.211457212	-1.516304074	3.11E-11	1.74E-10
AC087521.1	-1.444523238	-2.673379779	3.17E-11	1.77E-10
C3P1	-1.561100318	6.657613491	3.17E-11	1.77E-10
GABRA3	5.823179717	-0.312103584	3.19E-11	1.78E-10
LINC01549	3.450771115	0.446995133	3.19E-11	1.78E-10
RPS6P25	1.076969216	-1.959447681	3.19E-11	1.78E-10
AC002550.2	1.125958927	-2.490438512	3.20E-11	1.78E-10
ITGB1BP2	1.344977474	-0.305526087	3.21E-11	1.79E-10
BTNL8	3.625242859	2.262897011	3.22E-11	1.79E-10
SOX11	3.468363009	-2.130078257	3.24E-11	1.80E-10
NTS	6.787022924	7.03243038	3.28E-11	1.82E-10
TSPEAR-AS1	2.177352764	0.861334816	3.31E-11	1.84E-10
ATP13A4	-1.893473911	-1.625214837	3.34E-11	1.86E-10
RTEL1-TNFRSF6	1.074841679	0.187572171	3.35E-11	1.86E-10
LINC01269	3.548991645	-2.597295192	3.46E-11	1.92E-10
SLCO6A1	5.90208045	-1.879002899	3.47E-11	1.93E-10
AC010615.2	2.443726276	-0.878625921	3.49E-11	1.94E-10
CHST7	-1.06118434	2.643967558	3.50E-11	1.94E-10
ATP6V0E2-AS1	1.337498135	-0.181002496	3.53E-11	1.96E-10
SRXN1	1.392774591	1.760156941	3.54E-11	1.96E-10
IGF2BP2-AS1	3.362517048	-2.154024467	3.54E-11	1.97E-10

CCDC187	3.728935974	1.294214851	3.55E-11	1.97E-10
AC242426.2	1.101373717	1.51287921	3.57E-11	1.98E-10
ACSL4	2.158198156	7.949310431	3.65E-11	2.02E-10
BX649632.1	1.540547239	-2.811006084	3.71E-11	2.05E-10
PTGDR2	1.950093956	1.447641473	3.76E-11	2.08E-10
CACNG4	4.028976866	2.273659801	3.76E-11	2.08E-10
AC109588.1	6.167711144	-1.971305269	3.77E-11	2.09E-10
AP006222.1	1.786502881	-2.024347627	3.81E-11	2.11E-10
PTGIR	-1.125428446	1.500673616	3.81E-11	2.11E-10
SEMA4F	1.390704984	2.233778556	3.87E-11	2.14E-10
PPIAP29	1.776340909	0.719444792	3.88E-11	2.14E-10
KRT12	6.977220844	0.268776735	3.89E-11	2.15E-10
TTC9	1.820840786	4.305548375	3.89E-11	2.15E-10
SPTY2D1OS	1.075154146	-1.236188486	4.06E-11	2.24E-10
AC093458.2	1.701024279	-2.231696709	4.08E-11	2.25E-10
KCNJ6	4.392184087	-0.285432332	4.10E-11	2.26E-10
AC093788.1	1.195701287	-1.884506735	4.19E-11	2.31E-10
BAALC-AS1	1.630136263	-0.792113563	4.22E-11	2.32E-10
LINC01666	3.918632801	-2.39682407	4.24E-11	2.33E-10
COLEC12	2.405845174	1.917505085	4.24E-11	2.33E-10
LINC01446	4.592601053	-0.848440428	4.31E-11	2.37E-10
AC011481.1	1.484839343	-2.835363215	4.33E-11	2.38E-10
AC129507.2	-1.228046249	0.054008977	4.33E-11	2.38E-10
SLITRK3	-2.703837643	1.484441205	4.34E-11	2.38E-10
CDHR2	-1.92455654	5.277936423	4.35E-11	2.39E-10
RDH8	5.174382419	-1.72495697	4.36E-11	2.39E-10
CYP27B1	1.670941349	0.052987722	4.38E-11	2.40E-10
AP001350.2	1.104166796	-1.579407859	4.40E-11	2.41E-10
AP001160.1	1.126373299	-1.696312196	4.40E-11	2.42E-10
AC022154.1	1.424102365	-2.922298656	4.41E-11	2.42E-10
SPAG4	1.421166859	2.470685214	4.43E-11	2.43E-10
LINC01101	4.013759594	-2.375077158	4.49E-11	2.46E-10
GHRHR	3.697244853	0.630332556	4.50E-11	2.47E-10
SAMD5	-1.626548218	2.982245698	4.50E-11	2.47E-10
SLCO1B1	-1.286361961	7.059446964	4.51E-11	2.47E-10
HIST1H4K	1.857453273	-2.303592314	4.57E-11	2.50E-10
PLCXD3	-1.769967986	0.90110067	4.60E-11	2.52E-10
SLC22A12	5.097221364	3.505438294	4.66E-11	2.55E-10
AC007298.2	-1.648253514	-0.737220986	4.66E-11	2.55E-10
AP001992.1	1.376113559	-0.442049893	4.67E-11	2.55E-10
UPK1A	2.890605283	-1.09050769	4.71E-11	2.58E-10
FGF13	1.925421082	2.496341746	4.73E-11	2.58E-10
SNRPD2	1.018390659	6.762386559	4.74E-11	2.59E-10
MMP14	1.324003802	6.244984862	4.75E-11	2.59E-10
LINC02388	-1.787684307	-2.390367146	4.76E-11	2.60E-10
GPR137C	1.223336221	0.716261023	4.86E-11	2.65E-10
ASPHD1	2.732547876	2.56130967	4.86E-11	2.65E-10
ADGRL1	1.826025207	3.266719264	4.92E-11	2.68E-10
TMEM132E	-1.509017487	-0.484508514	4.92E-11	2.68E-10
KCNA3	-1.451467198	-1.698424313	5.07E-11	2.76E-10
FBXO15	-1.099663415	-1.213623745	5.07E-11	2.76E-10
IL2RB	-1.157537405	2.914268302	5.11E-11	2.78E-10
AC104118.1	1.166967505	-1.403048497	5.23E-11	2.84E-10
AL359715.4	-1.213465963	-2.25631659	5.26E-11	2.86E-10
LINC00896	2.111727894	0.289424577	5.32E-11	2.89E-10
C5orf58	3.139761679	-0.159287456	5.33E-11	2.89E-10
PDE6G	-1.034668684	0.086564722	5.43E-11	2.95E-10
BBOX1	-1.767623117	4.128470333	5.61E-11	3.04E-10

SMC4	1.26924915	3.965317466	5.64E-11	3.06E-10
AC010761.1	1.061611123	-0.756397856	5.65E-11	3.06E-10
AC013275.1	2.503730044	0.581934657	5.69E-11	3.08E-10
FZD10	3.031123769	-1.338256064	5.80E-11	3.14E-10
HOXC-AS2	3.906763014	-2.864854538	5.82E-11	3.15E-10
AL512306.2	1.623700962	-0.510027376	5.85E-11	3.16E-10
GAS2L3	1.154357511	2.296640412	5.93E-11	3.21E-10
SAA2	-2.29656779	9.103676772	5.98E-11	3.23E-10
RNF144A-AS1	2.742749542	-1.213830066	6.01E-11	3.24E-10
ARHGAP20	-1.349162878	0.323470768	6.01E-11	3.24E-10
ZNF92P3	2.503230588	-2.868019759	6.10E-11	3.29E-10
AC020594.1	1.461056055	-2.555637544	6.14E-11	3.31E-10
AL031600.1	1.642391135	-2.31049069	6.14E-11	3.31E-10
AC092115.3	3.507004092	-2.700207069	6.15E-11	3.31E-10
LINC01134	1.251478678	0.251217957	6.20E-11	3.34E-10
PHYHIPL	2.037074128	3.115956084	6.23E-11	3.35E-10
HOXB8	3.863933848	-2.486661473	6.25E-11	3.36E-10
CEACAM4	-1.172620984	-1.932986271	6.26E-11	3.36E-10
FAT4	-1.202175855	2.666273764	6.28E-11	3.38E-10
KLHL38	2.932449347	-2.383910926	6.38E-11	3.42E-10
PPFIA4	2.53686132	-0.463657731	6.38E-11	3.43E-10
CENPX	1.070632165	5.74424182	6.42E-11	3.44E-10
RBP2	4.28602695	-0.08059922	6.43E-11	3.45E-10
AL139100.1	1.52889195	-1.715231784	6.45E-11	3.46E-10
AC011632.1	5.625963556	-1.509011763	6.47E-11	3.47E-10
TREML3P	4.685295558	-0.544648401	6.49E-11	3.48E-10
ARHGEF2	1.029779886	4.637653132	6.50E-11	3.48E-10
AC080112.3	1.129396461	-0.710482204	6.53E-11	3.50E-10
ENTPD2	1.66517874	2.020206909	6.54E-11	3.50E-10
MFSD10	1.072952791	4.473775844	6.79E-11	3.63E-10
AL449212.1	1.291545049	-1.920132957	7.06E-11	3.78E-10
SLC1A1	-1.42107193	5.367165049	7.08E-11	3.78E-10
ANGPTL3	-1.150751676	8.456813971	7.08E-11	3.78E-10
KIF21B	1.50326927	3.240049295	7.10E-11	3.79E-10
RNASE4	-1.057982961	4.356084218	7.18E-11	3.83E-10
RPL10L	4.976793795	-1.658584378	7.19E-11	3.84E-10
AC026250.1	1.462280716	-1.422700609	7.41E-11	3.95E-10
AL021392.1	1.98919484	-1.955818742	7.56E-11	4.03E-10
PTGER2	-1.206290844	0.038991324	7.69E-11	4.10E-10
AC009121.2	1.972475031	-2.970611422	7.82E-11	4.17E-10
TRIM46	1.667855065	-0.922799859	7.91E-11	4.21E-10
AP000253.1	-1.236219611	-2.276927385	7.92E-11	4.21E-10
MIR663AHG	6.17033931	-1.682258798	7.98E-11	4.24E-10
SYNPO2	-1.111903512	3.284817418	7.99E-11	4.25E-10
PRKCB	-1.167934033	1.968260672	8.01E-11	4.26E-10
LINC00173	2.044402029	-2.451736447	8.03E-11	4.26E-10
CDH9	6.892647258	-1.178295495	8.14E-11	4.32E-10
SPTBN2	-1.241250131	5.09300437	8.26E-11	4.38E-10
COLCA1	1.815841089	1.329663921	8.27E-11	4.39E-10
LINC01615	2.806922706	-2.607567145	8.33E-11	4.41E-10
AL162726.3	2.868056755	-1.586028313	8.43E-11	4.47E-10
VWA5B2	2.323901113	-1.045795742	8.44E-11	4.47E-10
ITIH1	-1.094752047	10.71412402	8.56E-11	4.53E-10
LY6K	3.929189488	0.234306916	8.59E-11	4.55E-10
DLX6	5.820434914	-1.35401233	8.68E-11	4.60E-10
PRR19	1.997160588	0.499601	8.69E-11	4.60E-10
NR0B1	6.471585566	-0.48951482	8.75E-11	4.63E-10
HHATL	5.884941138	1.677079837	8.79E-11	4.65E-10

BICD1	1.204458412	1.634575493	8.85E-11	4.68E-10
AC106782.5	1.63614311	-1.77725312	8.86E-11	4.68E-10
CYCSP6	4.174574035	-2.990479509	9.13E-11	4.82E-10
CTXN1	2.294391343	-0.309055305	9.14E-11	4.83E-10
NAALADL1	2.198333775	2.45668223	9.15E-11	4.83E-10
CNBD2	1.095178361	-1.58977935	9.18E-11	4.84E-10
AL391832.1	2.660084886	-1.646396014	9.20E-11	4.85E-10
VCX3A	6.096338672	-1.575068171	9.28E-11	4.89E-10
TNNT1	3.562345646	0.383125001	9.39E-11	4.94E-10
CPA2	8.586607952	2.094856561	9.41E-11	4.95E-10
CACNG1	4.87858512	-1.984120712	9.43E-11	4.96E-10
PLA2G1B	2.240266341	0.558070433	9.44E-11	4.97E-10
LINC00346	1.781337468	1.154788959	9.60E-11	5.04E-10
AC079766.1	-1.086737737	-2.629291711	9.66E-11	5.08E-10
G0S2	-1.507083757	6.635253743	9.68E-11	5.08E-10
FOXF2	2.456427406	-0.810172751	9.72E-11	5.10E-10
HTR3A	5.117108134	-0.266376202	9.78E-11	5.13E-10
MNX1-AS1	6.409723646	-1.198000304	9.89E-11	5.18E-10
MSL3P1	1.395661303	-0.912093307	9.92E-11	5.20E-10
BPIFB2	4.774019335	3.805962665	1.01E-10	5.28E-10
DRD4	2.023194687	0.355888194	1.01E-10	5.29E-10
ANKRD33	5.229818409	-0.16885968	1.01E-10	5.30E-10
XAGE5	7.087213321	-0.522979402	1.01E-10	5.30E-10
ACRV1	1.798103455	-2.835955916	1.03E-10	5.39E-10
LINC00622	1.525999562	-1.507159051	1.05E-10	5.48E-10
RNF183	3.038870157	-1.671779342	1.05E-10	5.51E-10
TEX45	2.062243578	-1.937797924	1.05E-10	5.51E-10
AP002364.1	1.521151578	-2.906560742	1.06E-10	5.53E-10
BX322639.1	2.422344175	-0.267000972	1.06E-10	5.53E-10
AMH	2.451400708	-0.740798253	1.06E-10	5.55E-10
HBA2	-1.56543151	3.29718955	1.07E-10	5.61E-10
LINC01019	6.488094374	-1.291385051	1.08E-10	5.64E-10
KCTD17	1.661825809	3.452007228	1.09E-10	5.67E-10
PLGLB1	-1.224858482	1.421915172	1.09E-10	5.71E-10
ZEB2P1	3.534470098	-3.027982925	1.10E-10	5.74E-10
AC005381.1	4.897161517	-2.412880954	1.11E-10	5.77E-10
SPATA25	1.170547382	-0.183878286	1.12E-10	5.84E-10
AC020928.1	2.916471557	-2.284480499	1.13E-10	5.86E-10
CABP1	1.711425786	-1.703039334	1.13E-10	5.86E-10
MTUS2	-2.045656917	-0.388320084	1.14E-10	5.93E-10
COL8A1	1.663080221	2.576932923	1.15E-10	5.96E-10
LEAP2	-1.177822401	6.789005643	1.15E-10	5.99E-10
MYH16	1.753977819	-2.773892274	1.16E-10	6.01E-10
LINC02525	5.488119179	-2.30999783	1.16E-10	6.01E-10
AC003973.3	2.733163042	-0.115410054	1.16E-10	6.02E-10
ABCA8	-1.437452374	4.819677321	1.18E-10	6.13E-10
LINC00664	2.705089865	-2.207761553	1.19E-10	6.16E-10
SHC3	1.703314375	-0.01432522	1.20E-10	6.22E-10
TRIM31	2.303517375	3.20346332	1.20E-10	6.23E-10
AC234582.1	1.211350764	-1.573544729	1.20E-10	6.23E-10
FRG1JP	1.446730831	-0.446042152	1.20E-10	6.24E-10
ENPEP	-1.056869415	5.485871016	1.21E-10	6.26E-10
C1QC	-1.077379563	6.544362824	1.22E-10	6.30E-10
AL365226.2	5.646176476	-1.944141028	1.22E-10	6.30E-10
PLAC8L1	1.441691051	-1.67933241	1.22E-10	6.33E-10
TSPAN5	2.235134105	3.086570105	1.23E-10	6.36E-10
CAPS	1.079495344	3.540996858	1.23E-10	6.37E-10
SPON2	1.072865808	7.421500325	1.24E-10	6.41E-10

AL354798.1	3.001825798	-0.015644488	1.26E-10	6.52E-10
PIK3R2	1.235630814	0.309742527	1.27E-10	6.56E-10
HOMER2P1	5.960553673	-1.754459376	1.28E-10	6.59E-10
HAPLN1	4.860954748	-0.609634977	1.29E-10	6.64E-10
SOSTDC1	3.018328002	-0.644064742	1.29E-10	6.66E-10
ZBED6CL	1.077893397	4.197907322	1.31E-10	6.77E-10
CNTNAP4	4.483973483	0.371552646	1.32E-10	6.78E-10
WNT6	2.807263406	-0.756225117	1.32E-10	6.81E-10
AC020765.2	1.196403925	-1.366027153	1.33E-10	6.83E-10
CARMIL3	-1.200988568	0.820057684	1.33E-10	6.84E-10
AC010168.2	1.131178804	-0.192809573	1.35E-10	6.92E-10
MCCC1-AS1	1.431647875	-2.201638261	1.36E-10	6.99E-10
H1FX-AS1	1.064891513	0.492528281	1.37E-10	7.05E-10
LINC01929	3.454814487	-1.667113729	1.39E-10	7.13E-10
LINC02275	-2.048813954	-1.198689729	1.40E-10	7.21E-10
AC120114.3	1.174978266	-1.036308951	1.41E-10	7.23E-10
TMEM47	-1.061159809	4.091819445	1.46E-10	7.49E-10
AL137186.2	1.10237004	0.503482306	1.47E-10	7.52E-10
OR11Q1P	5.296065131	-1.577604991	1.48E-10	7.56E-10
CCDC183	1.478168472	0.477072433	1.48E-10	7.56E-10
WFDC21P	2.773653829	0.778276015	1.49E-10	7.61E-10
SIGLEC5	-1.176456704	-2.146615587	1.50E-10	7.65E-10
AC024600.1	-1.755040382	-2.757379333	1.50E-10	7.69E-10
KLK4	6.89051937	0.661724863	1.51E-10	7.72E-10
PI16	-1.843518887	-1.699337453	1.54E-10	7.85E-10
LINC01341	1.436163775	0.894815707	1.55E-10	7.91E-10
KHK	-1.02237605	7.704419634	1.55E-10	7.92E-10
ILDR2	2.299118177	2.642399	1.56E-10	7.99E-10
MT1M	-2.260796053	5.411570549	1.57E-10	8.00E-10
AL121832.2	1.185045933	0.736305126	1.57E-10	8.01E-10
BHMT	-1.616041184	8.623086751	1.59E-10	8.09E-10
DDN-AS1	1.87224377	-2.531029478	1.59E-10	8.12E-10
RNU6-529P	-1.123525186	-2.402709404	1.59E-10	8.12E-10
HIST3H2A	2.695834133	1.056932657	1.60E-10	8.13E-10
LNCOC1	1.603835488	-1.493629031	1.60E-10	8.15E-10
TMEM249	1.876210006	-2.622435896	1.60E-10	8.15E-10
RPL8	1.135980853	10.42276177	1.62E-10	8.23E-10
KRT20	6.56156519	2.39904613	1.62E-10	8.24E-10
GXYLT2	2.479643675	0.283776591	1.62E-10	8.24E-10
GAL3ST1	2.46942963	3.875878323	1.63E-10	8.30E-10
HOXB13	5.748588808	-0.457748341	1.64E-10	8.34E-10
FAM87A	3.059947168	-2.370792213	1.64E-10	8.35E-10
AL109976.1	2.185148911	-1.879991013	1.65E-10	8.38E-10
SLC6A17	2.964985785	-1.599536474	1.65E-10	8.39E-10
CMBL	-1.078917913	7.398447923	1.66E-10	8.44E-10
AL441992.1	1.01396654	0.696155761	1.67E-10	8.46E-10
AC015922.3	-1.023514363	-0.878703607	1.67E-10	8.49E-10
PDZK1IP1	2.981708267	5.738244349	1.68E-10	8.53E-10
AC137723.2	-1.546880704	-0.680409236	1.69E-10	8.56E-10
GAP43	2.847686056	-1.960183755	1.71E-10	8.68E-10
PODNL1	1.765590561	-0.837869738	1.72E-10	8.72E-10
TCAP	1.995957127	1.188099844	1.72E-10	8.72E-10
AC012435.1	1.207131319	-2.531328785	1.74E-10	8.79E-10
SULT1C2P1	2.751946665	-2.719327215	1.76E-10	8.90E-10
AC021146.9	-1.523630466	1.021197597	1.76E-10	8.92E-10
DGCR9	2.068577096	-1.174713506	1.77E-10	8.96E-10
KLF4	-1.161418667	3.512920492	1.78E-10	8.98E-10
AC108134.2	1.386087972	-1.286894342	1.80E-10	9.07E-10

AC020917.4	1.069029394	-0.581094765	1.80E-10	9.07E-10
C5orf30	1.689620371	1.640556034	1.80E-10	9.10E-10
AC018755.4	-1.587515935	-2.050826973	1.81E-10	9.12E-10
KLHL30	2.467926804	-0.343253517	1.82E-10	9.19E-10
TUBA3C	7.20258311	1.451992088	1.84E-10	9.30E-10
AL035252.3	1.641367058	-0.862836574	1.88E-10	9.47E-10
ARHGEF37	1.459078405	3.027870439	1.89E-10	9.54E-10
B3GALT1	4.39015305	-0.771372165	1.90E-10	9.58E-10
AC245060.5	1.221832784	-0.733434926	1.92E-10	9.66E-10
KCNE1	-1.43436015	-1.924036936	1.92E-10	9.68E-10
APOA1	-1.359868267	12.9530708	1.93E-10	9.72E-10
NPTX1	3.763356213	-1.190391954	1.97E-10	9.92E-10
PKD1L2	2.566350469	1.126423088	1.99E-10	9.99E-10
AC010148.1	1.730112459	-1.792655173	2.00E-10	1.00E-09
LINC01630	5.092379679	-2.056753687	2.02E-10	1.02E-09
DHDH	1.952806655	-1.017327961	2.03E-10	1.02E-09
SUCLG2P2	-1.082583622	-1.328980175	2.03E-10	1.02E-09
SSX5	6.004956985	-2.106759095	2.05E-10	1.03E-09
MAGEA11	7.071509236	-0.875299377	2.05E-10	1.03E-09
TMEM132D-AS	7.465254736	-0.424769338	2.05E-10	1.03E-09
AC092683.1	1.794491694	-2.849525524	2.05E-10	1.03E-09
RPL12P35	2.149817537	-2.584369648	2.05E-10	1.03E-09
SLC6A7	2.995953896	-2.214340506	2.06E-10	1.03E-09
PIEZO2	1.137383345	4.620063614	2.07E-10	1.04E-09
HIST1H2BE	1.670064228	-2.782780666	2.08E-10	1.04E-09
LUZP2	4.249380632	-0.091126467	2.09E-10	1.05E-09
KCNH2	3.580996209	1.434960516	2.09E-10	1.05E-09
DCC	4.092173124	-0.418192823	2.10E-10	1.05E-09
RBPJL	7.329979094	1.118387271	2.14E-10	1.07E-09
HOMER3	1.237448663	3.41541084	2.15E-10	1.08E-09
FAM96AP2	2.383847467	-2.011785544	2.16E-10	1.08E-09
TMEM163	3.382773517	1.970632429	2.17E-10	1.09E-09
PLAG1	1.821628473	1.15384294	2.19E-10	1.09E-09
SLC7A11-AS1	3.733577579	-2.834767481	2.19E-10	1.09E-09
TFR2	-1.027319564	9.574566248	2.20E-10	1.10E-09
REG1B	10.50171311	3.060653961	2.23E-10	1.11E-09
PAIP1P1	1.305888051	-2.172040194	2.26E-10	1.12E-09
AP002360.1	1.100512093	1.456511459	2.31E-10	1.15E-09
AC104170.1	-1.083915554	-1.542799129	2.33E-10	1.16E-09
AP001830.1	1.202576607	-0.022704249	2.35E-10	1.17E-09
PGGHG	1.647343958	5.72968835	2.39E-10	1.19E-09
KISS1	1.729209762	1.026955143	2.39E-10	1.19E-09
ESYT3	2.199108963	0.184262663	2.39E-10	1.19E-09
ERICH4	4.164304744	-2.737414929	2.39E-10	1.19E-09
AC034213.1	3.842710844	-1.945038488	2.41E-10	1.20E-09
AL391095.2	-1.302671309	2.083077431	2.44E-10	1.21E-09
PTPN14	1.213555185	3.260278824	2.46E-10	1.22E-09
MSH4	2.196055999	-2.665627246	2.49E-10	1.23E-09
KCNJ11	1.717317371	1.444653386	2.49E-10	1.23E-09
C4orf48	1.88648932	0.693545158	2.51E-10	1.24E-09
AC113368.1	-1.402923646	-2.284451587	2.52E-10	1.25E-09
AOX3P	2.317363439	-0.964603176	2.52E-10	1.25E-09
NALT1	1.345303887	-0.330186641	2.56E-10	1.27E-09
COL11A1	3.911708213	1.387271232	2.58E-10	1.27E-09
B9D1	1.039530876	3.139861195	2.59E-10	1.28E-09
SVEP1	-1.496013429	2.723848581	2.59E-10	1.28E-09
APOBEC3B	1.879144043	1.800731073	2.61E-10	1.29E-09
CLTCL1	1.152696208	3.644045822	2.63E-10	1.30E-09

SSTR5	5.765576544	-0.135375479	2.64E-10	1.30E-09
TNFSF15	1.872859833	1.058476882	2.66E-10	1.31E-09
GNRH1	1.010480057	-1.096742945	2.66E-10	1.31E-09
MDGA1	2.125740446	2.457596177	2.67E-10	1.31E-09
MTRNR2L8	-1.288939051	-0.452015245	2.67E-10	1.31E-09
NRG1	-1.891726058	2.727146058	2.67E-10	1.32E-09
WDR88	1.033107189	-0.889438188	2.69E-10	1.33E-09
SHISA2	2.359749894	-1.34188749	2.70E-10	1.33E-09
LOX	1.630089215	2.970092723	2.71E-10	1.33E-09
DSCR4	6.990613575	-1.294662601	2.71E-10	1.33E-09
AL133370.1	4.409239916	-2.393090219	2.72E-10	1.34E-09
AC087289.2	1.096605939	-1.930641436	2.75E-10	1.35E-09
FAM186A	1.385253714	-1.460311455	2.77E-10	1.36E-09
AC022532.1	1.604998531	-2.024216815	2.77E-10	1.36E-09
GRIK4	2.584530138	-0.429114939	2.77E-10	1.36E-09
SLC2A2	-1.166105745	8.633758795	2.78E-10	1.36E-09
RPS10P5	1.608553842	-2.50801835	2.78E-10	1.37E-09
AF196972.1	1.309267318	-2.259304287	2.83E-10	1.39E-09
NT5C1B	-1.051945389	-2.032185535	2.83E-10	1.39E-09
AL139289.1	1.227141995	-2.10656037	2.84E-10	1.39E-09
ASPSCR1	1.23229534	6.456372805	2.85E-10	1.39E-09
LINC01979	-1.624408632	-0.958031379	2.85E-10	1.40E-09
CELA3A	6.024515679	-1.352418811	2.87E-10	1.40E-09
AC005618.1	1.892139671	-2.178468226	2.92E-10	1.43E-09
PDZD7	1.419212934	-1.390257034	2.94E-10	1.44E-09
DGAT2	-1.069351395	7.401448793	2.95E-10	1.44E-09
C22orf23	1.108408632	-1.281991482	2.95E-10	1.44E-09
TDGF1	2.501241901	3.018062631	2.97E-10	1.45E-09
CHGB	5.038482044	0.659784576	2.98E-10	1.45E-09
PRKAA2	1.894995795	3.615687043	2.98E-10	1.46E-09
AC018553.1	2.65864132	-0.362359695	2.99E-10	1.46E-09
SHISA4	1.402263353	3.793521934	3.02E-10	1.48E-09
Z99289.1	-1.61753741	-2.745453853	3.03E-10	1.48E-09
PLEKHS1	3.673133954	-0.004414306	3.04E-10	1.48E-09
DLX2	4.350182724	-2.236196011	3.04E-10	1.48E-09
NRXN1	-2.211478007	-2.868969021	3.09E-10	1.51E-09
EDARADD	2.259709845	0.540263963	3.11E-10	1.51E-09
AC004160.2	-2.005373821	-2.554187078	3.11E-10	1.52E-09
CAND2	-1.385319726	0.8515072	3.19E-10	1.55E-09
WNT11	-1.509655984	2.231101465	3.19E-10	1.55E-09
CACNA1S	2.658839334	-2.655026798	3.22E-10	1.57E-09
ZNF541	2.265951641	0.727242993	3.26E-10	1.59E-09
MAPT-IT1	2.624339172	-2.875682175	3.28E-10	1.59E-09
SELENOM	1.753462002	5.122680646	3.29E-10	1.60E-09
ZIC4	3.410393985	0.936061763	3.30E-10	1.60E-09
CST2	3.014004955	-1.377825311	3.30E-10	1.61E-09
AC010809.2	1.182283717	-1.551066222	3.32E-10	1.61E-09
ABLIM2	1.441897333	2.211431907	3.32E-10	1.61E-09
ZNF233	1.513527921	-0.099082515	3.33E-10	1.62E-09
AC022400.7	1.035082836	1.499521063	3.35E-10	1.63E-09
AL662844.3	1.456557716	-2.984368452	3.37E-10	1.63E-09
Z84485.1	1.342773632	-1.178871707	3.37E-10	1.63E-09
KIF28P	1.547914158	-1.164535618	3.42E-10	1.66E-09
LHFPL4	4.462602062	-0.053735439	3.42E-10	1.66E-09
PGBD1	1.046878415	2.119198297	3.57E-10	1.72E-09
PTGES3L	1.648808938	-2.124960692	3.57E-10	1.73E-09
AC097634.1	1.701950043	-2.899439141	3.59E-10	1.73E-09
AC124944.2	1.924355987	-2.590705064	3.60E-10	1.74E-09

AC141557.2	2.128989423	-3.001056962	3.61E-10	1.74E-09
AC112907.2	1.677395218	-2.440212203	3.66E-10	1.77E-09
ZSCAN31	1.371080951	3.516857332	3.74E-10	1.80E-09
AC105105.2	-1.108701362	0.859976281	3.76E-10	1.81E-09
LVRN	2.377149406	-2.258120222	3.78E-10	1.82E-09
NAT14	1.238536983	2.676874111	3.79E-10	1.83E-09
PURPL	2.415193679	0.566247455	3.86E-10	1.86E-09
AC108136.1	2.823285748	-1.868925193	3.89E-10	1.87E-09
LINC00941	2.662601607	-1.547493053	3.90E-10	1.87E-09
AC097461.1	1.168767301	-1.033935289	3.94E-10	1.89E-09
AL161669.3	1.375420943	-0.00636212	3.99E-10	1.91E-09
ZNF385D-AS1	5.424538148	-2.988250583	4.01E-10	1.93E-09
AC004923.4	1.441427652	-2.635913096	4.03E-10	1.93E-09
ZNF85	1.362458494	0.275187645	4.07E-10	1.95E-09
KRTAP5-1	2.264203316	-2.805305196	4.08E-10	1.96E-09
CREB3L1	2.334889307	2.680080766	4.09E-10	1.96E-09
ZNF192P1	1.50590968	-1.283903981	4.11E-10	1.97E-09
LINC01431	1.222339748	-1.308790459	4.13E-10	1.98E-09
CD180	-1.035939274	1.123450354	4.16E-10	1.99E-09
ERC2	3.024538529	-2.085077264	4.16E-10	1.99E-09
MAP1A	1.758149855	1.742713747	4.16E-10	1.99E-09
CHRNB2	2.190004471	-2.160845041	4.18E-10	2.00E-09
POLH-AS1	1.04157441	-1.682063254	4.20E-10	2.01E-09
PLCD3	1.357842301	2.598091895	4.21E-10	2.01E-09
KRTAP5-5	5.439441355	-2.537312362	4.26E-10	2.03E-09
VEGFD	2.681535799	2.24018654	4.28E-10	2.04E-09
PKP4-AS1	1.483823842	-2.884151166	4.30E-10	2.05E-09
FRMD3	1.898990943	2.291362597	4.38E-10	2.09E-09
CHODL	3.847589135	-0.146222424	4.39E-10	2.09E-09
PDGFRA	-1.602842156	3.744867087	4.42E-10	2.11E-09
SEMA6D	-1.174630711	1.341659651	4.47E-10	2.13E-09
PLEKHN1	1.786682991	-0.039149892	4.47E-10	2.13E-09
UGT1A10	4.994411867	0.203965911	4.47E-10	2.13E-09
CR381653.1	1.292332916	-1.65724004	4.50E-10	2.14E-09
LGI3	3.239248065	-0.31503091	4.51E-10	2.15E-09
AC020907.1	1.983390829	-0.193814536	4.65E-10	2.21E-09
PTGES	2.698447533	2.176231327	4.72E-10	2.24E-09
SULT4A1	4.523215241	1.290012388	4.73E-10	2.25E-09
LIPG	-1.109105361	5.372146205	4.79E-10	2.27E-09
AC096564.1	-1.276318641	-2.271981441	4.85E-10	2.30E-09
AC020907.4	1.659453611	-2.544133652	4.87E-10	2.31E-09
EYA1	3.936205812	-1.711690631	4.87E-10	2.31E-09
AL391244.2	1.911436447	-2.764825882	4.89E-10	2.32E-09
TSPEAR	1.934452897	-0.404117182	4.90E-10	2.32E-09
CCDC196	-1.984457533	-0.316384479	5.01E-10	2.37E-09
LINC01091	1.891116472	-0.906413247	5.02E-10	2.37E-09
LINC01730	1.89337114	-2.633727729	5.05E-10	2.39E-09
INSRR	3.100690353	-2.219699712	5.09E-10	2.41E-09
IGFBP1	-1.379119471	9.574666718	5.12E-10	2.42E-09
CAPN12	1.538471739	3.680700198	5.16E-10	2.44E-09
SPP2	-1.61755124	6.690040895	5.20E-10	2.46E-09
MSC	2.574312772	3.836448419	5.22E-10	2.47E-09
KLF2P1	5.529106811	-2.668808303	5.26E-10	2.48E-09
MC1R	1.175004204	0.772260478	5.33E-10	2.51E-09
SLC5A11	2.367076627	1.261279631	5.33E-10	2.52E-09
LINC00862	1.637044051	-1.139471652	5.33E-10	2.52E-09
TMEM132B	2.658149047	-0.71890791	5.34E-10	2.52E-09
MYEF2	2.288125817	1.420984922	5.35E-10	2.52E-09

AVIL	2.052941377	2.90271732	5.42E-10	2.55E-09
FABP6	4.364188773	-1.321333361	5.48E-10	2.58E-09
C15orf48	2.299341211	2.608489006	5.50E-10	2.59E-09
C16orf89	3.739214572	1.79695429	5.56E-10	2.62E-09
ESPL1	1.379923871	3.250762935	5.60E-10	2.63E-09
RBP4	-1.029575817	12.35331691	5.61E-10	2.64E-09
AC233280.2	1.959092735	-2.979265188	5.61E-10	2.64E-09
PLPPR1	1.649137702	4.082629025	5.61E-10	2.64E-09
CA8	2.26883043	-0.924954695	5.79E-10	2.72E-09
AC005186.1	5.153580039	-0.783541911	5.86E-10	2.75E-09
AP001273.1	1.017159785	-0.507654802	5.89E-10	2.76E-09
CASC8	3.506053253	-2.20905235	6.01E-10	2.82E-09
AC073263.1	2.227208232	-2.754911334	6.12E-10	2.87E-09
CORIN	2.354791833	0.162324074	6.18E-10	2.90E-09
BSND	3.629100495	-2.724249878	6.24E-10	2.92E-09
CHRNA3	3.918044804	-1.6397706	6.25E-10	2.93E-09
SLC16A3	1.676047968	4.417092312	6.37E-10	2.98E-09
RBMY2FP	6.833429412	-1.875786302	6.38E-10	2.99E-09
AC139769.2	4.575779724	-1.656429604	6.39E-10	2.99E-09
NAV3	2.243781255	2.373785409	6.40E-10	2.99E-09
AC018665.1	1.394888178	-0.25016116	6.45E-10	3.02E-09
TAGAP	-1.107332505	1.262935787	6.56E-10	3.06E-09
FAM99A	-1.897008132	3.97660719	6.58E-10	3.07E-09
LINC00648	5.235738812	-1.04816908	6.58E-10	3.07E-09
SLC25A5P1	1.28421134	-2.473711662	6.71E-10	3.13E-09
AC007405.1	1.234071635	-1.607796046	6.74E-10	3.14E-09
AC020915.2	1.469342538	-2.683003403	6.76E-10	3.15E-09
ARR3	1.313004187	-2.616463444	6.80E-10	3.17E-09
AMBN	7.781825546	-0.185785228	6.82E-10	3.17E-09
GAST	7.826999671	-0.121206517	6.83E-10	3.18E-09
AL356512.1	1.229881352	-1.931184383	6.85E-10	3.19E-09
AL133467.4	4.600563348	-3.055456931	6.85E-10	3.19E-09
AC120498.4	1.30990455	0.269784032	6.86E-10	3.19E-09
SLC38A8	4.990010183	-1.357102693	6.87E-10	3.20E-09
TKFC	-1.090371792	7.404945406	6.93E-10	3.22E-09
CA12	2.825654284	4.416228806	6.98E-10	3.24E-09
FNDC4	-1.074800928	5.656902061	6.98E-10	3.24E-09
AL139125.2	2.370143512	-1.999630778	6.99E-10	3.24E-09
MAP2	1.571019627	4.307123698	7.02E-10	3.26E-09
CTAGE7P	1.325098703	-2.318700832	7.05E-10	3.27E-09
PAGE2B	6.21218197	1.226463189	7.06E-10	3.27E-09
AP006216.2	-1.47345334	-0.635612692	7.09E-10	3.29E-09
XDH	-1.283579499	6.053419539	7.11E-10	3.30E-09
ANG	-1.171749253	8.877437948	7.14E-10	3.31E-09
IL11	3.681710098	-0.289415375	7.15E-10	3.31E-09
HIST1H4I	1.032427445	3.696756317	7.17E-10	3.32E-09
CFAP61	2.906536275	-2.336153364	7.19E-10	3.33E-09
AC114485.1	5.892872112	-2.646815491	7.23E-10	3.35E-09
CKB	2.050887611	5.701002801	7.30E-10	3.38E-09
TGM4	3.146943168	-2.416500138	7.31E-10	3.38E-09
KISS1R	4.226379038	-1.428027736	7.36E-10	3.40E-09
AC092809.4	1.324088819	-2.442069794	7.38E-10	3.41E-09
B4GALNT4	3.649660115	1.496853953	7.42E-10	3.43E-09
TMEM74	1.667626389	0.677998501	7.50E-10	3.46E-09
MMP10	3.541181919	0.095737851	7.50E-10	3.46E-09
AC114811.2	3.574480805	-1.983304302	7.51E-10	3.47E-09
ARMC9	1.037848851	1.461134269	7.51E-10	3.47E-09
PRR16	1.338515033	-0.571790282	7.59E-10	3.50E-09

GABRG2	5.666423857	-1.942929634	7.60E-10	3.51E-09
SCARA3	1.547769552	4.169450788	7.61E-10	3.51E-09
AL109811.1	1.341268802	-2.960150917	7.70E-10	3.55E-09
AC110285.1	1.905065928	-2.895860294	7.79E-10	3.59E-09
PPP1R14C	4.111689815	-0.447786591	7.83E-10	3.61E-09
PRTFDC1	1.236777242	1.321643702	7.89E-10	3.64E-09
ABCA17P	2.147503419	-1.411570371	7.93E-10	3.65E-09
FAM3B	3.05104557	4.159661568	7.97E-10	3.67E-09
CPN2	-1.097867391	7.92587682	8.10E-10	3.72E-09
AC024940.1	3.613116047	-1.624919482	8.13E-10	3.74E-09
FAM230C	5.938898252	-2.139482412	8.24E-10	3.78E-09
MT3	4.154683093	-0.287881331	8.25E-10	3.79E-09
SATB2-AS1	1.710590086	-2.015006466	8.33E-10	3.82E-09
AZGP1P1	-1.212260034	3.539879806	8.35E-10	3.83E-09
PRAMEF4	5.204220594	-0.123860272	8.35E-10	3.83E-09
FERMT1	2.258578077	2.145448472	8.39E-10	3.85E-09
SMIM25	-1.215756396	0.627031542	8.42E-10	3.86E-09
DGUOK-AS1	1.126930449	-1.620803367	8.60E-10	3.94E-09
AC126323.1	3.276665973	-0.35835847	8.64E-10	3.96E-09
FAM21EP	1.050398821	-1.785892784	8.74E-10	4.00E-09
ASNS	1.714710974	3.136187696	8.80E-10	4.02E-09
AP003174.1	2.071151732	-2.156659315	8.91E-10	4.07E-09
AC139491.2	2.079429888	0.480694605	8.93E-10	4.08E-09
LRRC26	3.81522569	-2.309413428	8.94E-10	4.08E-09
RNF144A	1.131166711	2.447445182	8.94E-10	4.08E-09
A1BG	-1.226939761	6.696821391	8.95E-10	4.08E-09
AC124067.4	3.051521388	-0.289139662	8.98E-10	4.10E-09
AC104260.2	-1.271311304	-2.732421282	9.00E-10	4.11E-09
PTPRVP	1.275783195	-2.208163892	9.00E-10	4.11E-09
STAMBPL1	1.150835142	2.033168633	9.07E-10	4.14E-09
MCTP1	1.550285435	2.732077035	9.08E-10	4.14E-09
G2E3-AS1	5.037081121	-2.231776137	9.10E-10	4.15E-09
GRIN1	2.899950644	-2.39799924	9.10E-10	4.15E-09
AL359075.1	2.096303183	-2.871234507	9.14E-10	4.17E-09
CPA1	7.09784727	2.011814936	9.22E-10	4.20E-09
INAVA	2.406337332	2.57514149	9.24E-10	4.21E-09
ZNF431	1.098741573	1.710650033	9.25E-10	4.21E-09
DPYS	-1.22681867	7.975017006	9.35E-10	4.26E-09
PLK5	2.160573496	-2.5298143	9.40E-10	4.28E-09
TSPAN10	1.662219009	1.805324396	9.44E-10	4.30E-09
AQP10	4.39092778	-0.644825964	9.61E-10	4.37E-09
C10orf95	1.199974566	-2.516113201	9.66E-10	4.39E-09
AL021328.1	-2.118989431	-0.121386538	9.69E-10	4.40E-09
LYZ	2.194801492	8.034336822	9.78E-10	4.44E-09
LINC01436	3.420729114	0.311422962	9.85E-10	4.47E-09
GSTA4	1.065481333	4.601844743	9.88E-10	4.49E-09
MCIDAS	3.465420379	-3.044827758	9.89E-10	4.49E-09
CIB2	1.626455182	1.345438478	9.96E-10	4.52E-09
C9orf57	3.825733729	-1.638062519	9.98E-10	4.53E-09
AP1M2	3.065450583	3.939750511	9.98E-10	4.53E-09
AP006284.1	1.304846367	0.651187139	1.00E-09	4.54E-09
AC004492.1	1.054111505	-2.127696207	1.01E-09	4.59E-09
HES5	1.696323455	-2.384880741	1.03E-09	4.65E-09
AP005901.1	5.117248146	-2.995156365	1.03E-09	4.65E-09
PAGE5	3.902378622	2.00549279	1.03E-09	4.65E-09
ABCA3	1.634054142	3.218081221	1.03E-09	4.68E-09
AC006065.4	5.732447355	-1.478340613	1.03E-09	4.68E-09
TET1	1.428667713	0.442207447	1.04E-09	4.70E-09

LINC02327	4.52879936	-2.561810262	1.04E-09	4.71E-09
ADGRD1-AS1	6.174674641	-1.859737687	1.04E-09	4.72E-09
RNF17	5.498721632	-0.941147004	1.04E-09	4.72E-09
HIST1H2AG	2.148051154	1.788383088	1.06E-09	4.78E-09
DLK1	7.021931625	6.996532129	1.06E-09	4.78E-09
NPHS1	4.297284738	-1.318556637	1.07E-09	4.81E-09
AC133644.2	2.410186599	-2.255104027	1.07E-09	4.83E-09
IDO2	-1.86221996	1.015725382	1.07E-09	4.83E-09
TFAP2E	1.597998137	-1.339696	1.07E-09	4.84E-09
GCNT3	2.533958548	2.68588942	1.08E-09	4.85E-09
AC103740.1	-1.359548944	1.904193003	1.08E-09	4.88E-09
SERPINA4	-1.176587581	7.981722231	1.09E-09	4.90E-09
AP001453.2	1.404191975	-0.576425063	1.09E-09	4.91E-09
EXTL3-AS1	1.231376847	-1.53863983	1.09E-09	4.91E-09
AC105052.2	1.255436479	-1.767384081	1.09E-09	4.92E-09
C1orf220	1.250104886	-0.620321191	1.10E-09	4.93E-09
PROK1	4.472522547	0.649742579	1.10E-09	4.95E-09
CCR3	3.654684982	-1.10983058	1.11E-09	4.99E-09
RCOR2	2.314364128	-0.367992431	1.12E-09	5.05E-09
AC124947.2	1.36001192	0.448850111	1.13E-09	5.06E-09
TTC39A-AS1	2.331809964	-2.624166938	1.13E-09	5.09E-09
AL139011.2	-1.167735601	-1.872643668	1.14E-09	5.11E-09
TYRP1	4.045730035	-0.414647459	1.14E-09	5.12E-09
AC006449.5	1.217085141	-1.668222518	1.14E-09	5.13E-09
ADAM12	2.25063256	1.583549715	1.15E-09	5.14E-09
ABCC4	1.586618196	3.455750292	1.15E-09	5.15E-09
AC026254.2	1.61606833	-2.849734402	1.17E-09	5.24E-09
GGN	1.532721366	-2.241760921	1.17E-09	5.24E-09
BCAS1	2.448534218	1.493282624	1.18E-09	5.26E-09
AC135506.1	1.183288563	-0.955259204	1.18E-09	5.30E-09
TYMSOS	1.288403195	1.333842722	1.19E-09	5.32E-09
GNGT1	4.743304184	-2.021337535	1.20E-09	5.35E-09
UCKL1-AS1	1.350444455	-1.79114526	1.22E-09	5.43E-09
SOX9-AS1	1.824756042	2.699872217	1.23E-09	5.51E-09
SYT5	2.567335238	-1.537499962	1.25E-09	5.57E-09
AC139887.1	1.278263995	-2.404537493	1.25E-09	5.58E-09
UPK1A-AS1	3.316362753	-2.017116922	1.25E-09	5.59E-09
AC006504.8	1.137394324	-1.260762379	1.25E-09	5.59E-09
MIR9-3HG	2.929344379	0.772018599	1.28E-09	5.70E-09
PLEKHG4B	2.984281587	-0.116351564	1.28E-09	5.70E-09
MLANA	2.979728762	1.196590345	1.29E-09	5.73E-09
TTYH3	1.003094415	6.03652286	1.29E-09	5.73E-09
TLX2	3.65178066	-2.877310917	1.30E-09	5.79E-09
CALCA	4.01994225	2.502490034	1.31E-09	5.83E-09
SLC6A3	4.79722882	-0.748709926	1.33E-09	5.90E-09
AGXT	-1.205557599	10.3504468	1.33E-09	5.92E-09
CTAG2	6.416252573	2.13729562	1.34E-09	5.96E-09
AL121583.1	1.353059914	-2.559997212	1.36E-09	6.03E-09
TREM2	1.585049265	2.217610218	1.37E-09	6.06E-09
NFATC4	1.303981425	2.570111319	1.37E-09	6.06E-09
FBXL16	2.403382672	1.533541874	1.37E-09	6.08E-09
CFHR3	-1.585221822	7.076197137	1.40E-09	6.22E-09
DLX4	2.610384079	-1.711506038	1.42E-09	6.28E-09
AC099552.1	6.335638912	-2.286201572	1.42E-09	6.29E-09
FREM2	-2.269467722	2.021567593	1.43E-09	6.32E-09
DLGAP1-AS2	1.147795511	0.178206611	1.44E-09	6.36E-09
FGF12	1.978255495	1.943775098	1.44E-09	6.36E-09
PSORS1C1	2.01156909	-0.565424925	1.44E-09	6.38E-09

KCNK12	3.467538246	-2.312635326	1.45E-09	6.40E-09
TFDP3	7.539571614	-0.084098812	1.45E-09	6.42E-09
AC004870.2	5.940406189	-2.390441407	1.46E-09	6.46E-09
LINC02188	3.646768974	-0.855365304	1.46E-09	6.46E-09
AC125603.2	2.9681761	-1.721634383	1.48E-09	6.54E-09
KCNQ1OT1	1.218421789	1.119241416	1.48E-09	6.54E-09
PRSS1	9.207754146	2.480969806	1.48E-09	6.54E-09
LINC01315	1.376581941	-0.453180153	1.49E-09	6.58E-09
PLCD4	1.054227707	1.054153339	1.50E-09	6.61E-09
C10orf91	4.623087121	-1.0773659	1.51E-09	6.64E-09
LINC02027	-2.117402134	0.702751997	1.51E-09	6.64E-09
SP3P	4.527492238	-2.434151298	1.51E-09	6.67E-09
AL031719.2	1.237795448	-2.569615336	1.52E-09	6.68E-09
AP001271.2	1.539997758	-2.681463644	1.52E-09	6.69E-09
KNOP1P5	5.585772132	-2.483083322	1.54E-09	6.76E-09
KRTAP5-10	1.921337699	-2.438327479	1.55E-09	6.80E-09
TEX37	5.814971482	-2.71989302	1.56E-09	6.85E-09
LINC02593	-1.638450391	-0.56691651	1.58E-09	6.93E-09
FDCSP	5.383004848	2.178661007	1.58E-09	6.93E-09
AXDND1	2.023598532	-2.191664457	1.58E-09	6.94E-09
CACNB3	1.099319874	1.404759108	1.59E-09	6.98E-09
AC008060.4	5.853029645	-2.712450417	1.59E-09	6.98E-09
ADH1C	-1.553558894	9.097336931	1.59E-09	7.00E-09
AL591845.1	1.227187769	-0.621313004	1.61E-09	7.06E-09
CACNA1I	3.316741378	0.368646526	1.61E-09	7.06E-09
SAA1	-2.046389719	10.49351767	1.63E-09	7.14E-09
TACR1	3.281835995	0.048744492	1.64E-09	7.17E-09
BX322650.1	1.684438005	-2.670550148	1.64E-09	7.18E-09
TMC5	2.948469966	3.095524292	1.65E-09	7.21E-09
C1QTNF1	-1.38955195	5.144282818	1.65E-09	7.21E-09
FGL1	-1.299685024	10.60159773	1.68E-09	7.36E-09
LRRN2	1.819706858	1.778396489	1.69E-09	7.39E-09
SPECC1	1.528838013	2.876112791	1.70E-09	7.46E-09
CILP	-1.411232955	0.447079089	1.72E-09	7.54E-09
SCN4B	1.214940856	1.275032747	1.73E-09	7.58E-09
GLUL	2.065540756	11.50595155	1.75E-09	7.67E-09
TMSB10	1.351124405	8.813616087	1.75E-09	7.67E-09
TYRO3	1.672022958	3.116335904	1.76E-09	7.71E-09
AC021092.1	1.944219977	-2.850260185	1.77E-09	7.72E-09
RAMP3	-1.000777768	3.784819101	1.77E-09	7.75E-09
MAGEB1	6.862708555	-1.320782847	1.79E-09	7.80E-09
AL161891.1	1.195194566	-0.676070388	1.79E-09	7.80E-09
RTL1	6.548773023	-0.092570385	1.79E-09	7.80E-09
MFSD2B	1.891605154	-0.579103077	1.79E-09	7.82E-09
TF	-1.048708314	12.86134998	1.79E-09	7.82E-09
SLC4A4	-1.241242056	4.881145108	1.80E-09	7.83E-09
ASB15	4.155099149	-2.00821829	1.80E-09	7.84E-09
AC104809.1	-2.338241753	2.004899357	1.81E-09	7.87E-09
CD24	2.03541093	6.788866088	1.81E-09	7.87E-09
AC008549.1	-1.622946852	3.188060404	1.81E-09	7.88E-09
NMUR1	-1.052635837	-0.421017841	1.82E-09	7.93E-09
RHOF	1.368387209	0.170527794	1.82E-09	7.93E-09
AL080317.2	1.195318988	-1.435624197	1.83E-09	7.95E-09
GUCY2C	3.023937008	2.240772532	1.83E-09	7.98E-09
PRSS56	6.179472275	-0.497438655	1.84E-09	7.98E-09
AL445647.1	3.734249953	-2.909322134	1.85E-09	8.05E-09
AL139327.1	7.067844591	-1.059425821	1.87E-09	8.11E-09
SEMA3G	1.118793675	3.204506706	1.87E-09	8.13E-09

CYP2C18	-1.216207665	5.671777547	1.87E-09	8.14E-09
ZNF382	1.309575477	0.561209062	1.93E-09	8.39E-09
CFAP74	-1.449571386	-0.746491455	1.95E-09	8.44E-09
AGPAT4	1.342567834	1.984681907	1.97E-09	8.53E-09
AP002761.4	1.429634303	1.104202893	1.97E-09	8.54E-09
C1QB	-1.072225502	6.907501721	1.97E-09	8.55E-09
AC068580.1	1.363071855	-2.212992739	2.00E-09	8.64E-09
NMNAT2	2.338120658	-0.317021564	2.00E-09	8.65E-09
FAM71F2	1.341080789	-0.493282943	2.00E-09	8.65E-09
AIFM3	1.253265319	0.918590471	2.01E-09	8.71E-09
AKR1B10P1	3.357039144	0.886429835	2.02E-09	8.71E-09
AC016717.2	5.953389493	-0.844660001	2.02E-09	8.72E-09
AC010327.4	1.17249608	-1.086994355	2.02E-09	8.72E-09
LINC02413	5.085188731	-1.564168006	2.04E-09	8.82E-09
COL1A1	1.77198197	8.524523778	2.05E-09	8.86E-09
HOXA9	2.543928414	-2.492910328	2.05E-09	8.86E-09
PKDCC	1.264075714	4.093791015	2.06E-09	8.88E-09
B3GNT4	1.372921047	-1.314381862	2.07E-09	8.94E-09
AC005324.3	2.81254314	-2.846329424	2.07E-09	8.94E-09
CD248	1.102416174	3.522866541	2.08E-09	8.95E-09
OAS2	-1.156818338	5.220370801	2.08E-09	8.96E-09
SQSTM1	1.068539653	9.338063854	2.08E-09	8.97E-09
LUZP4	6.348082386	-2.273789548	2.09E-09	8.99E-09
FAM110C	-1.211532115	3.972823785	2.11E-09	9.09E-09
ALOX15B	3.237052068	2.121118775	2.11E-09	9.09E-09
AC131009.1	1.303507337	-2.240180251	2.12E-09	9.12E-09
CES5A	-1.446988693	1.309740564	2.12E-09	9.13E-09
BCYRN1	2.080351574	-0.682620368	2.13E-09	9.15E-09
AC005165.1	3.692991591	-0.90771468	2.14E-09	9.21E-09
NR1I2	-1.365608857	5.144873041	2.15E-09	9.24E-09
MTNR1B	6.880912358	-0.855848999	2.15E-09	9.24E-09
TLL2	1.971150474	-1.321121561	2.15E-09	9.25E-09
AL139246.3	1.432302537	-2.116822634	2.15E-09	9.26E-09
TUBB1	-1.047896352	-2.241104252	2.16E-09	9.29E-09
HIST1H1C	1.438255078	7.055680976	2.16E-09	9.29E-09
AL355472.1	1.061617751	0.815417837	2.17E-09	9.31E-09
ANXA2P2	1.039067694	0.972460077	2.22E-09	9.55E-09
NKPD1	2.311380098	-2.653229351	2.23E-09	9.56E-09
MECOM	1.163938617	2.487624375	2.25E-09	9.67E-09
SCGB1D2	5.485235344	-1.239996224	2.28E-09	9.76E-09
AC011815.1	1.201751804	-0.868228005	2.29E-09	9.81E-09
LINC01956	4.730235623	-1.888929565	2.32E-09	9.93E-09
AC104534.1	2.041062486	-1.239906461	2.33E-09	9.98E-09
AC008060.1	5.298862695	-2.793442987	2.34E-09	1.00E-08
ADAM22	1.695742345	0.475658275	2.37E-09	1.01E-08
AL162582.1	3.506141529	-0.253765233	2.38E-09	1.02E-08
AL365203.2	1.006098534	1.922424178	2.41E-09	1.03E-08
TMEM191C	1.31440621	-1.953170839	2.42E-09	1.03E-08
FOXD4	1.624097458	-2.234567418	2.43E-09	1.04E-08
AC084125.4	1.257583696	-2.098437913	2.45E-09	1.05E-08
HS6ST2	3.871991862	1.233482496	2.47E-09	1.05E-08
HIST3H2BB	3.049909734	-1.988443391	2.47E-09	1.06E-08
LINC02600	2.577046386	-0.858621419	2.49E-09	1.06E-08
DNAH8	2.155892916	-1.865050579	2.53E-09	1.08E-08
LINC00114	2.905912747	-2.83410861	2.54E-09	1.09E-08
MIR325HG	2.777292274	-1.254619834	2.55E-09	1.09E-08
ZNF724	1.425627978	-1.38371054	2.59E-09	1.10E-08
SLC6A14	5.223091268	-0.504636637	2.62E-09	1.12E-08

MYRF	1.08113422	5.708843615	2.63E-09	1.12E-08
CAMKV	4.690787872	-2.267691568	2.63E-09	1.12E-08
AC022306.3	1.297200169	-2.487546667	2.65E-09	1.13E-08
PCSK1N	4.062735802	2.131671619	2.65E-09	1.13E-08
AC093616.1	1.18500281	-1.830933308	2.66E-09	1.13E-08
PRR7	1.213414462	1.320866207	2.67E-09	1.14E-08
PRRX2	2.853738306	-1.786296943	2.67E-09	1.14E-08
AC124067.2	2.151807561	-2.8752425	2.69E-09	1.14E-08
RTN2	1.275680408	2.925504851	2.69E-09	1.14E-08
LINC00702	1.301258958	-0.811375184	2.72E-09	1.15E-08
AC115619.1	-1.047734317	5.610675184	2.72E-09	1.15E-08
AP000350.5	1.450153105	-2.902445943	2.73E-09	1.16E-08
CPT1B	1.064901918	0.458116013	2.74E-09	1.16E-08
LINC01637	1.348975783	-1.0564317	2.74E-09	1.16E-08
SYNGR4	2.090161418	-1.767360617	2.75E-09	1.16E-08
ST6GALNAC5	2.658094452	-1.629018921	2.77E-09	1.17E-08
CNNM1	2.636048065	2.471209555	2.80E-09	1.19E-08
BMP4	1.759921593	3.393670681	2.81E-09	1.19E-08
SCN11A	-1.294402388	-2.0544175	2.83E-09	1.20E-08
GPR1	3.035066978	-1.905126368	2.85E-09	1.21E-08
GRM4	3.312889501	-2.462325455	2.86E-09	1.21E-08
B3GNT5	1.658889457	3.186698114	2.86E-09	1.21E-08
AVPR2	1.631075818	-1.848997574	2.91E-09	1.23E-08
KIF7	1.224773608	1.496533504	2.97E-09	1.25E-08
SAMSN1	-1.030931392	1.698431852	2.98E-09	1.26E-08
PACSIN1	2.158564163	1.161915533	2.98E-09	1.26E-08
CACNB1	1.047518152	0.214666884	2.99E-09	1.26E-08
AC010247.2	2.688294346	0.224878426	2.99E-09	1.26E-08
LINC01151	2.243178014	0.562508194	3.01E-09	1.27E-08
AL592295.1	1.182291267	-2.855178191	3.03E-09	1.28E-08
FOXJ1	3.218421661	1.361883945	3.04E-09	1.28E-08
EN1	4.919728227	-2.386166579	3.07E-09	1.30E-08
HOXC13	4.84481666	-2.533730047	3.08E-09	1.30E-08
GLS	1.066992779	5.008820639	3.09E-09	1.30E-08
AL008721.2	1.214742445	-1.169431957	3.13E-09	1.32E-08
KITLG	1.140092587	2.71775747	3.14E-09	1.32E-08
AC068620.2	1.206131993	-2.84118797	3.14E-09	1.32E-08
CNR1	2.625450886	-0.070321398	3.15E-09	1.33E-08
AL442128.2	1.35567281	-2.872846297	3.17E-09	1.33E-08
AC136632.2	1.155931474	-1.155432152	3.18E-09	1.34E-08
CEBPA-DT	1.083093208	3.206965472	3.18E-09	1.34E-08
ZNF492	2.779536638	-2.086849112	3.18E-09	1.34E-08
STK19B	1.778443597	-0.347359111	3.21E-09	1.35E-08
PCP4	5.770015319	0.681921834	3.21E-09	1.35E-08
AC090772.3	1.646058647	-1.557607549	3.25E-09	1.36E-08
VNN1	-1.460805503	7.167710824	3.25E-09	1.37E-08
FIGNL2	1.557660537	-0.693512946	3.26E-09	1.37E-08
CHRNA5	1.712171802	-1.306686979	3.29E-09	1.38E-08
TPM3P9	1.084818714	1.917751276	3.29E-09	1.38E-08
AP001767.3	1.49634337	-2.656617517	3.32E-09	1.39E-08
AC108752.1	5.962372958	-2.178767932	3.33E-09	1.39E-08
IGHM	-1.683586066	6.039887451	3.34E-09	1.40E-08
CCDC13	2.604433564	1.261371369	3.34E-09	1.40E-08
NAT16	2.856936761	-1.663731871	3.34E-09	1.40E-08
KCNMB3	1.198083909	0.215318346	3.45E-09	1.44E-08
ZNF730	2.761241139	-2.629543113	3.46E-09	1.45E-08
MMP9	2.038249123	3.97239733	3.46E-09	1.45E-08
S100A3	1.939864379	-1.002209706	3.46E-09	1.45E-08

AC010643.1	2.251843853	0.466713066	3.46E-09	1.45E-08
CCDC114	1.521482542	-1.633065174	3.47E-09	1.45E-08
CABP7	1.33411055	-1.483923157	3.48E-09	1.45E-08
MTND2P28	-1.063563954	7.71135554	3.50E-09	1.46E-08
MGAT3	1.55182002	0.220917519	3.51E-09	1.46E-08
KCNJ14	1.015662825	-0.150192369	3.52E-09	1.47E-08
DBIL5P	1.122258224	-2.566146205	3.53E-09	1.47E-08
HCG20	2.017709057	-2.299970222	3.54E-09	1.47E-08
HGFAC	-1.772397531	6.657629571	3.56E-09	1.48E-08
TMEM151A	2.465771111	0.960362625	3.59E-09	1.50E-08
AP002847.1	2.113597344	-2.29214343	3.60E-09	1.50E-08
LINC02037	-1.38965216	1.016947592	3.61E-09	1.50E-08
AC079760.2	3.089771528	-2.718981724	3.64E-09	1.51E-08
AC090921.1	4.653294541	-1.437329138	3.64E-09	1.51E-08
RNF2P1	1.319846729	-2.115964202	3.77E-09	1.56E-08
YEATS2-AS1	1.088596059	-1.935113507	3.80E-09	1.58E-08
AC024580.1	1.397979003	-1.878118527	3.80E-09	1.58E-08
CLRN1-AS1	-1.933625798	-2.428518764	3.81E-09	1.58E-08
IKBKE	1.185279407	2.573690096	3.81E-09	1.58E-08
AC011481.3	1.185608587	1.824349527	3.81E-09	1.58E-08
RPL34-AS1	-1.032411379	-2.793326032	3.82E-09	1.58E-08
PWWP2B	1.022435613	3.702310845	3.84E-09	1.59E-08
MAGEB16	5.670828367	-2.829191585	3.85E-09	1.60E-08
AC099792.1	3.167567791	-2.949218279	3.86E-09	1.60E-08
AL591848.3	1.009158974	-1.812555933	3.86E-09	1.60E-08
CRLF2	3.423482052	-0.934659447	3.87E-09	1.60E-08
TM6SF2	-1.147623592	4.02700819	3.91E-09	1.62E-08
AL662907.1	1.113530719	-2.604531545	3.95E-09	1.63E-08
PAX8	1.271659575	1.746109392	3.96E-09	1.64E-08
CAVIN3	1.20901087	2.929283724	4.00E-09	1.65E-08
AC010970.1	-2.604976022	0.609502105	4.06E-09	1.68E-08
ELFN1-AS1	3.639004857	-0.318263784	4.10E-09	1.69E-08
EN2	3.755351482	-1.772446729	4.11E-09	1.70E-08
PLAU	1.241750585	3.099569291	4.14E-09	1.71E-08
CD274	-1.027478029	1.087031844	4.16E-09	1.72E-08
C2CD4D	1.346340358	-1.763600317	4.17E-09	1.72E-08
TBX2-AS1	1.523629306	-0.2949728	4.18E-09	1.72E-08
AC006128.1	1.121757428	0.464201686	4.18E-09	1.72E-08
AC127496.1	1.423207564	-2.87813967	4.19E-09	1.72E-08
SLC4A3	2.609483084	1.207761409	4.25E-09	1.75E-08
SLC12A9-AS1	1.680527145	-2.280220329	4.36E-09	1.79E-08
AC137630.3	1.49590181	-2.659061399	4.38E-09	1.80E-08
AC090409.1	1.432742924	-2.576066646	4.39E-09	1.81E-08
ICAM5	2.49593893	-0.98718147	4.40E-09	1.81E-08
AC116914.2	1.12160287	-2.719047686	4.40E-09	1.81E-08
MNX1	4.648729864	-0.704922975	4.43E-09	1.82E-08
HNRNPA1P10	1.113566943	-2.009866169	4.48E-09	1.84E-08
BIK	1.885148194	0.606610835	4.49E-09	1.84E-08
DKKL1	1.936830941	-1.80501007	4.52E-09	1.85E-08
LGALS14	10.45457109	1.849885212	4.57E-09	1.87E-08
GNMT	-1.500274097	6.556841868	4.62E-09	1.89E-08
IGSF1	2.335810172	2.91825741	4.62E-09	1.89E-08
MGC27382	-1.108179489	-0.525404882	4.66E-09	1.91E-08
SRC	1.150663713	4.78556603	4.68E-09	1.92E-08
CACYBPP2	1.195295895	-2.799210995	4.72E-09	1.93E-08
SLC12A5	1.631497088	0.257831495	4.74E-09	1.94E-08
REG3G	11.31311002	2.719137914	4.78E-09	1.96E-08
PITX2	4.803973206	-1.018780325	4.81E-09	1.97E-08

AC007406.2	2.359255787	-0.810236281	4.82E-09	1.97E-08
GAGE2A	6.57114926	-1.849052825	4.85E-09	1.98E-08
SSC4D	1.515092786	2.525616634	4.86E-09	1.99E-08
AC121338.2	1.627901201	-1.019146865	4.86E-09	1.99E-08
CKMT1A	3.420011207	-1.483336682	4.90E-09	2.00E-08
GP2	5.648062257	3.179653311	4.93E-09	2.01E-08
SPATA21	2.462646555	0.8979491	4.96E-09	2.03E-08
SLC26A9	3.693380903	0.576132911	4.98E-09	2.03E-08
SOCS2-AS1	-1.118759065	0.921339167	5.01E-09	2.04E-08
SPHK1	2.093228068	4.119193334	5.01E-09	2.04E-08
AC010536.1	1.155151919	-1.87199743	5.02E-09	2.05E-08
F13A1	1.773058586	3.236542164	5.04E-09	2.05E-08
SCAMP5	1.349613635	3.990042863	5.05E-09	2.06E-08
TH	-1.989784416	-2.320730961	5.08E-09	2.07E-08
CNFN	1.962405926	-0.180919316	5.12E-09	2.09E-08
LINC01697	4.84231582	-1.111145382	5.14E-09	2.09E-08
AL163952.1	3.079939561	-2.421640132	5.16E-09	2.10E-08
CYTL1	1.556494296	-1.943658675	5.23E-09	2.13E-08
IGF1	-1.314130497	3.190695035	5.26E-09	2.14E-08
MTTP	-1.07264149	7.421680281	5.28E-09	2.14E-08
BEX2	2.574052783	2.717744574	5.28E-09	2.14E-08
AC113410.3	1.735719255	-2.773174024	5.32E-09	2.16E-08
CKMT1B	4.506839848	-0.297165089	5.32E-09	2.16E-08
HABP2	-1.122663291	8.486746243	5.38E-09	2.18E-08
AC026356.2	1.355613048	-2.748523781	5.38E-09	2.18E-08
DLL3	3.937980279	-2.614235133	5.41E-09	2.19E-08
AL139289.2	1.197595236	-1.575300119	5.44E-09	2.20E-08
CCDC112	1.031228773	1.140367043	5.45E-09	2.21E-08
WSCD1	1.404375297	1.974615118	5.49E-09	2.23E-08
EFHD1	-1.312854333	4.447161874	5.52E-09	2.23E-08
MEOX2	1.57204517	0.105058325	5.53E-09	2.24E-08
GUCY2D	2.094410589	-0.371014843	5.54E-09	2.24E-08
NPM2	1.932360735	1.884134998	5.56E-09	2.25E-08
PCNX2	1.437852946	1.830210419	5.69E-09	2.30E-08
AC138965.3	3.914364452	-2.261924404	5.70E-09	2.30E-08
TRPM5	2.901099515	-2.605383539	5.76E-09	2.33E-08
TCL6	2.797709697	-1.433645616	5.80E-09	2.34E-08
GLB1L3	3.291488091	-1.350682474	5.80E-09	2.34E-08
APOC3	-1.193116436	12.00975613	5.87E-09	2.37E-08
C3orf85	-1.736719868	1.018444745	5.91E-09	2.38E-08
LINC01876	1.925754231	-1.734805813	6.01E-09	2.42E-08
RPRML	3.05854736	-2.224237064	6.03E-09	2.43E-08
CNGA1	-1.268137555	3.717187396	6.06E-09	2.44E-08
PYY2	1.343477734	-1.83144983	6.18E-09	2.49E-08
AIRE	2.881817017	-2.83450235	6.19E-09	2.49E-08
CCDC185	3.759085975	-2.886184791	6.20E-09	2.49E-08
VSX1	2.188422171	-1.619008649	6.28E-09	2.52E-08
KRT86	2.256421171	0.216373959	6.28E-09	2.53E-08
PSORS1C3	1.903368471	-1.064223234	6.30E-09	2.53E-08
C1QTNF12	1.654538089	-0.098125509	6.31E-09	2.53E-08
CASC15	1.850791976	0.005550004	6.38E-09	2.56E-08
CCDC162P	2.436579229	1.168168034	6.52E-09	2.62E-08
LRP8	1.558098532	0.581004089	6.60E-09	2.65E-08
TMSB15A	2.159463801	-2.20670216	6.61E-09	2.65E-08
SHLD2P1	-1.089403469	-0.877298963	6.63E-09	2.66E-08
ART4	-1.113063613	4.375998893	6.69E-09	2.68E-08
LINC01694	2.857612708	-0.867130108	6.70E-09	2.68E-08
STPG3	1.541329554	0.089697799	6.77E-09	2.71E-08

SH3GL3	6.094322351	0.5520251	6.82E-09	2.73E-08
TMEM155	3.073849299	-2.173151652	6.93E-09	2.77E-08
SLC25A25	-1.001932445	6.437609206	6.95E-09	2.78E-08
ELOVL3	2.402665702	-1.698258144	6.98E-09	2.79E-08
Z97192.2	2.479978159	-1.318238411	6.99E-09	2.79E-08
ZNF320	1.456486964	2.714407743	7.01E-09	2.80E-08
AQP5	2.593417033	-1.736510781	7.02E-09	2.80E-08
ONECUT3	3.516516474	-2.158182073	7.05E-09	2.81E-08
AC090502.4	4.569791952	-2.944595639	7.06E-09	2.82E-08
AC068580.4	1.407484752	-1.147775558	7.13E-09	2.84E-08
ENOX1	1.829444869	-0.216917936	7.14E-09	2.85E-08
AL109933.2	-1.848467689	-2.538539244	7.21E-09	2.87E-08
STOX1	1.846440296	0.308360978	7.27E-09	2.90E-08
AC006946.2	3.356714189	-2.896876382	7.28E-09	2.90E-08
SOHLH1	5.504072932	-1.483307988	7.29E-09	2.90E-08
LINC01792	4.095215802	-3.116401606	7.30E-09	2.91E-08
MPPED1	-1.46024931	3.479413459	7.35E-09	2.93E-08
AC145423.2	1.611261707	-2.904632803	7.36E-09	2.93E-08
14-Sep	5.813125056	-1.26052144	7.36E-09	2.93E-08
SLCO1B3	-2.028117306	4.700116549	7.42E-09	2.95E-08
CHST1	1.335152351	1.867581316	7.51E-09	2.98E-08
AC007998.3	1.384776573	-2.299448469	7.52E-09	2.99E-08
TM4SF1-AS1	1.553883097	-1.882985605	7.52E-09	2.99E-08
ZNF560	5.260091412	-1.990932015	7.52E-09	2.99E-08
ITGB4	1.466795472	4.092300414	7.54E-09	2.99E-08
NOL4	-2.026083192	-1.03567146	7.59E-09	3.01E-08
MICAL1	1.02831097	3.716142926	7.60E-09	3.01E-08
DMRTB1	6.287373919	-2.338124022	7.74E-09	3.07E-08
SACS-AS1	6.57165795	-1.346547143	7.78E-09	3.08E-08
HIST1H2AE	1.564366763	1.121544124	7.80E-09	3.09E-08
RUNDC3A	1.834106094	-1.105386523	7.87E-09	3.11E-08
PPBP	-1.898227686	-1.984632275	7.88E-09	3.12E-08
MROH3P	3.622369771	-2.197823916	7.90E-09	3.13E-08
CLDN19	2.878956381	0.084933893	7.95E-09	3.15E-08
AP000892.3	-1.033836093	0.203406751	7.97E-09	3.15E-08
ENPP6	2.894973409	0.234632698	8.02E-09	3.17E-08
AL450306.1	1.547073754	-2.709297871	8.07E-09	3.19E-08
LINC02109	3.847313175	-2.269356202	8.09E-09	3.20E-08
CLUL1	1.855752992	-2.061877303	8.22E-09	3.25E-08
CSAG1	4.478460013	1.932996867	8.27E-09	3.27E-08
USP32P3	2.558863245	-3.100946848	8.31E-09	3.28E-08
CASP12	1.439655851	-2.517866393	8.36E-09	3.30E-08
MYO16	-1.089904234	1.394728404	8.37E-09	3.30E-08
OPRPN	4.104526267	2.22340632	8.58E-09	3.38E-08
LINC02253	4.730976733	-1.972870314	8.60E-09	3.39E-08
KRTAP5-6	1.645600464	-0.83914623	8.60E-09	3.39E-08
TKTL1	3.915266624	1.223465939	8.67E-09	3.42E-08
AL118505.1	2.486050494	-2.189769513	8.69E-09	3.42E-08
APOH	-1.008963908	12.08008054	8.70E-09	3.43E-08
MYH7B	2.131720896	2.469863147	8.80E-09	3.46E-08
TMEM52B	3.178450647	-0.826408399	8.80E-09	3.46E-08
LINC-ROR	3.584694536	-2.740395123	8.85E-09	3.48E-08
PTH2R	3.63560668	2.269097553	8.92E-09	3.50E-08
GRM2	1.661964244	-1.235188967	9.01E-09	3.54E-08
RAB3D	1.579388717	2.565522311	9.09E-09	3.57E-08
AL451069.3	4.109156566	-2.31696195	9.12E-09	3.58E-08
AC012510.1	1.075124537	-0.59454723	9.13E-09	3.58E-08
TM4SF19	2.310292419	-1.507567477	9.17E-09	3.59E-08

SBK3	2.601805343	-1.878070839	9.22E-09	3.61E-08
P2RX3	-1.303580737	-0.894498304	9.23E-09	3.61E-08
AC025575.2	3.237871415	-2.152070001	9.25E-09	3.62E-08
TMEM74B	1.369772153	1.954102974	9.27E-09	3.63E-08
PRR34-AS1	1.262528793	1.752625873	9.30E-09	3.64E-08
BHLHE40-AS1	1.760029649	-0.12359588	9.33E-09	3.65E-08
DNAH3	2.293787214	-1.899590641	9.41E-09	3.68E-08
ZNF43	1.316888812	1.809606999	9.42E-09	3.68E-08
SEMA7A	1.33493821	3.130573781	9.46E-09	3.70E-08
TMEM98	1.20924998	5.33002956	9.48E-09	3.71E-08
RAPSN	1.338931551	-1.345197401	9.51E-09	3.72E-08
BPIFA1	8.162220964	0.092144327	9.51E-09	3.72E-08
ZSCAN12P1	1.02633505	-0.52597027	9.57E-09	3.74E-08
TMIE	1.698301014	0.831709632	9.57E-09	3.74E-08
MTMR7	1.830909728	1.978113337	9.58E-09	3.74E-08
ACADL	-1.420042111	3.541534009	9.59E-09	3.74E-08
SPTA1	3.452798804	0.113335531	9.67E-09	3.77E-08
CXorf67	5.560369417	-1.811979189	9.69E-09	3.78E-08
CES3	-1.134976995	5.232907131	9.74E-09	3.80E-08
CA5B	1.324771489	2.020689568	9.77E-09	3.81E-08
DGKK	4.033753743	-1.189588589	9.80E-09	3.82E-08
TMEM25	-1.065980133	2.97662423	9.81E-09	3.82E-08
PIGZ	1.011335796	2.240107524	9.86E-09	3.84E-08
CHP2	6.210734743	0.247017452	9.90E-09	3.86E-08
CAMK2B	-1.624999326	2.220466936	9.93E-09	3.86E-08
PGF	1.148924337	2.915969369	1.00E-08	3.90E-08
TMPRSS5	1.349805838	-1.251755986	1.01E-08	3.94E-08
AC092506.1	2.562429479	-2.600380588	1.01E-08	3.94E-08
PTPRN	2.333675773	-2.801545222	1.02E-08	3.98E-08
HDGFL1	6.754272915	-1.195378305	1.03E-08	4.01E-08
TPH1	1.803199744	-1.602746469	1.03E-08	4.01E-08
MEF2B	1.048388997	-1.723124968	1.05E-08	4.06E-08
DISP2	1.547169627	1.232020569	1.05E-08	4.06E-08
NAMPTP1	-1.221477561	2.471659608	1.05E-08	4.06E-08
CCDC13-AS1	2.197821384	-1.205037473	1.06E-08	4.11E-08
FAM166A	1.736825455	-1.710928104	1.06E-08	4.12E-08
KIAA0319	2.046584634	-0.819149586	1.08E-08	4.17E-08
AL139042.1	4.832976131	-3.177862052	1.08E-08	4.19E-08
MPIG6B	-1.235126897	-2.865766578	1.08E-08	4.20E-08
ANGPT2	1.058657744	2.693496616	1.08E-08	4.20E-08
ERP27	2.288690174	0.937952293	1.09E-08	4.21E-08
AL590483.3	3.206785041	-2.401046739	1.09E-08	4.21E-08
TAC3	2.928097931	-1.89812307	1.10E-08	4.25E-08
IGFN1	4.679655839	2.440864448	1.10E-08	4.26E-08
HAPLN3	1.373545277	1.520824368	1.11E-08	4.28E-08
CLEC9A	-1.240648621	-1.561944618	1.11E-08	4.30E-08
HOXB7	1.636517379	0.474968835	1.12E-08	4.33E-08
AC008109.1	3.044204566	-0.862513106	1.13E-08	4.36E-08
GDAP1L1	3.061514886	-2.411767276	1.14E-08	4.39E-08
AC005154.2	1.044845834	-1.662794523	1.14E-08	4.39E-08
ALX1	5.720965812	-2.595552945	1.14E-08	4.40E-08
INSC	1.857819609	-0.982926148	1.14E-08	4.40E-08
TPRXL	3.120310403	-1.780018233	1.14E-08	4.41E-08
DDIT4L	2.348665071	0.169094752	1.15E-08	4.42E-08
LINC02267	4.965967435	-2.750182358	1.15E-08	4.42E-08
SSX3	6.688866412	-1.003100862	1.15E-08	4.44E-08
RNU6ATAC	4.631455224	-0.927708794	1.16E-08	4.47E-08
AC090826.1	1.829578546	-2.64408432	1.16E-08	4.47E-08

MUC15	4.907639438	0.111946511	1.17E-08	4.49E-08
LHX3	3.734841676	-0.215913929	1.19E-08	4.56E-08
CHRNB4	1.767581819	-1.86870522	1.20E-08	4.60E-08
SMARCD3	1.311839629	2.852548151	1.21E-08	4.65E-08
RTL5	-1.093243379	2.351745785	1.21E-08	4.66E-08
FAM99B	-1.675077164	1.240478214	1.21E-08	4.67E-08
DUOX2	3.810142869	4.772374455	1.22E-08	4.68E-08
AC244107.1	5.707734805	-2.289007767	1.22E-08	4.68E-08
OBSCN	1.991184997	3.206377217	1.22E-08	4.69E-08
FLRT1	1.371995489	-1.423773559	1.22E-08	4.69E-08
VN1R1	1.303127304	-0.803491112	1.22E-08	4.69E-08
TSPAN8	1.820618006	6.33266369	1.23E-08	4.72E-08
AL161772.1	2.298467543	-2.168470361	1.23E-08	4.73E-08
MMP1	2.542265649	1.313752104	1.23E-08	4.73E-08
LINC02577	4.486085289	-2.92733474	1.24E-08	4.76E-08
GLI1	1.348428696	0.096876379	1.25E-08	4.77E-08
PTPRG-AS1	1.703865539	-1.973553131	1.25E-08	4.79E-08
SYT9	-2.318198944	1.011825326	1.25E-08	4.79E-08
AC097478.1	3.588926223	-1.069972313	1.25E-08	4.80E-08
CBSL	-1.748363723	1.172061572	1.26E-08	4.82E-08
CAPG	1.33002842	4.765581931	1.26E-08	4.83E-08
FOXC2	1.718112098	-0.719328303	1.26E-08	4.83E-08
AC016405.3	1.524839269	-0.429920667	1.27E-08	4.85E-08
RAET1K	1.776958902	-2.976823524	1.28E-08	4.91E-08
SIK1B	-1.130733438	4.033023873	1.29E-08	4.94E-08
PCDHB8	2.48081626	-0.914832495	1.31E-08	5.01E-08
AC074135.1	3.195679587	-2.950717342	1.33E-08	5.06E-08
TREX2	1.720176227	-0.95112878	1.34E-08	5.09E-08
AC099489.1	1.325785127	0.555649366	1.34E-08	5.09E-08
HSPD1P6	1.245322252	-2.913019878	1.35E-08	5.13E-08
CPE	1.310689627	5.729752531	1.35E-08	5.13E-08
HES4	1.184983645	1.676890158	1.35E-08	5.13E-08
YWHAEP1	3.128679596	-2.781997514	1.35E-08	5.16E-08
DAGLA	1.423960514	2.086072886	1.36E-08	5.17E-08
AIF1L	1.390025382	3.211648195	1.37E-08	5.21E-08
AC062015.1	6.242199835	-2.191033658	1.38E-08	5.25E-08
AL954650.1	5.572096122	-2.62527672	1.41E-08	5.34E-08
AC010969.1	-1.879886064	-1.200116276	1.41E-08	5.36E-08
C1QTNF7	-1.245041764	-0.430665732	1.42E-08	5.38E-08
AACSP1	4.885683069	-1.929375395	1.42E-08	5.39E-08
RND1	-1.023770752	5.876811413	1.42E-08	5.39E-08
SLC25A15P3	3.50984436	-1.832989687	1.43E-08	5.41E-08
SSPO	1.443229559	1.311949248	1.43E-08	5.42E-08
AP000866.2	1.125782295	-2.035160779	1.45E-08	5.47E-08
GOLM1	1.253513347	6.321487066	1.45E-08	5.49E-08
AC022413.1	1.04114889	-1.342490982	1.46E-08	5.51E-08
GPAT2	1.598088279	1.029230451	1.48E-08	5.60E-08
TMEM61	2.33208589	-1.39984705	1.48E-08	5.60E-08
MROH6	1.313916677	3.248003065	1.49E-08	5.63E-08
CPLX1	1.502187995	3.22830819	1.53E-08	5.77E-08
NR0B2	-1.0840511	6.048397364	1.53E-08	5.78E-08
PTPRR	1.949356963	-1.471834087	1.54E-08	5.79E-08
ZNF826P	1.798212664	0.699233123	1.54E-08	5.81E-08
ACSM2A	-1.183342818	7.756581106	1.54E-08	5.81E-08
AC025580.3	1.472870262	-2.518378532	1.55E-08	5.82E-08
ANKS6	1.363257015	3.365706478	1.56E-08	5.86E-08
CTSK	1.9699724	4.25931303	1.58E-08	5.95E-08
CAHM	1.099160455	-1.356160788	1.58E-08	5.96E-08

PDCD1LG2	-1.102108966	0.50756067	1.62E-08	6.10E-08
NOS2	1.488130623	0.607634304	1.63E-08	6.15E-08
CKMT2	2.61547024	2.397125589	1.65E-08	6.20E-08
AL117335.1	1.536869795	-1.443864061	1.66E-08	6.22E-08
WFDC1	-1.097740826	0.890014345	1.67E-08	6.26E-08
ZNF208	2.114012448	-1.182137898	1.67E-08	6.26E-08
SLC22A31	4.100487838	3.90432786	1.67E-08	6.28E-08
COL9A2	1.741680541	1.871095737	1.68E-08	6.29E-08
DGAT2L7P	1.610525646	-2.343548669	1.71E-08	6.40E-08
EXPH5	-1.388035893	2.761072815	1.71E-08	6.41E-08
OVAAL	4.792423859	-2.630769121	1.73E-08	6.48E-08
SLC6A12	-1.139408672	5.749171254	1.74E-08	6.52E-08
HKDC1	1.627921217	5.103218737	1.77E-08	6.60E-08
MRAS	1.06603903	3.804616161	1.77E-08	6.63E-08
AL031722.1	-1.049086538	-1.97794441	1.78E-08	6.66E-08
AL513534.1	1.136478014	-0.147729597	1.79E-08	6.67E-08
FAM171B	1.182045928	0.626102132	1.79E-08	6.68E-08
SLC10A1	-1.514905361	7.367009548	1.80E-08	6.73E-08
MFAP4	-1.526139008	4.18357073	1.80E-08	6.73E-08
CACNA1C	1.196233018	1.462682029	1.82E-08	6.80E-08
TMEM108	1.529883689	-1.003064498	1.83E-08	6.84E-08
SERHL	1.123157861	-1.686595619	1.84E-08	6.86E-08
PABPC3	1.135945486	-1.563909913	1.84E-08	6.88E-08
AC004253.1	1.172582048	-2.431316229	1.85E-08	6.91E-08
AC010624.2	4.406755942	-2.598748796	1.86E-08	6.93E-08
HNF4A-AS1	-1.281735454	2.639943582	1.87E-08	6.96E-08
PAGE2	6.894095922	1.999512913	1.88E-08	7.01E-08
HOXC4	2.044920453	-1.273177667	1.89E-08	7.03E-08
MIR217HG	3.683817886	-1.991632673	1.90E-08	7.08E-08
PLCH2	2.166887047	3.304468484	1.90E-08	7.08E-08
ERVMER34-1	3.026850286	0.151270408	1.91E-08	7.09E-08
TTLL6	1.985305936	-1.412273329	1.93E-08	7.19E-08
AC073575.2	1.003633871	-2.587127937	1.95E-08	7.24E-08
RPL39L	1.694387679	2.50201919	1.96E-08	7.29E-08
RIMS2	2.971748104	-1.001595059	1.98E-08	7.33E-08
SCUBE3	1.429452665	-0.846034856	1.98E-08	7.36E-08
WDR72	-1.323395047	4.676105249	1.98E-08	7.36E-08
AC004801.2	1.306791768	-2.187652345	1.99E-08	7.37E-08
RIMS3	1.121985124	0.763697874	1.99E-08	7.38E-08
DUTP6	1.033591764	-1.520540126	2.00E-08	7.43E-08
ZNF781	1.568094334	-0.37941117	2.01E-08	7.43E-08
ZNF676	2.828515093	-0.666189346	2.01E-08	7.43E-08
SCGN	2.157305694	3.594697889	2.01E-08	7.44E-08
AL133467.2	3.799339283	-3.114291217	2.01E-08	7.46E-08
EXOSC4	1.010517774	5.184188522	2.03E-08	7.52E-08
AL354760.1	1.20822855	-2.694576599	2.05E-08	7.59E-08
PHYHD1	-1.24560862	4.44656046	2.06E-08	7.62E-08
ZNF703	1.337439199	3.85028669	2.07E-08	7.65E-08
KLK2	5.405001868	-0.602042786	2.07E-08	7.65E-08
IMPG2	1.093619776	-2.154195194	2.07E-08	7.65E-08
ZNF83	1.408036715	3.543565985	2.11E-08	7.80E-08
ZNF648	2.250453815	0.402685297	2.12E-08	7.83E-08
APCS	-1.171206484	9.777209671	2.12E-08	7.84E-08
AC104958.2	1.130400012	3.618845555	2.12E-08	7.85E-08
SOHLH2	5.12342042	-0.951047743	2.14E-08	7.89E-08
TRMT112P4	1.520001361	-1.364155085	2.17E-08	8.00E-08
GOLGA6L10	1.128543857	-1.993053711	2.18E-08	8.03E-08
DPYSL5	4.204017942	-2.889659527	2.18E-08	8.05E-08

RASD1	-1.244499074	5.688017767	2.19E-08	8.06E-08
FXYD3	2.871183437	2.839046412	2.19E-08	8.06E-08
RNY3P8	2.120843601	-2.669343017	2.19E-08	8.09E-08
AC007663.3	1.303130618	-2.477071661	2.20E-08	8.09E-08
FNDC10	1.706976259	1.474312446	2.25E-08	8.27E-08
LINC00488	3.506536241	-1.48486124	2.25E-08	8.29E-08
STUM	1.688830113	0.029271606	2.25E-08	8.29E-08
TNP1	8.815368546	0.784160242	2.26E-08	8.30E-08
CLCN1	2.101534084	-1.620219566	2.26E-08	8.32E-08
AC068580.3	1.000625374	0.783257414	2.29E-08	8.41E-08
SPINK1	2.881691696	8.230534637	2.30E-08	8.45E-08
CDK3	1.036631637	-0.902702022	2.31E-08	8.50E-08
AC022893.1	1.111047908	-1.319990644	2.31E-08	8.50E-08
BNIP3P17	2.628177975	-3.081620353	2.32E-08	8.52E-08
NXPH3	1.515207036	-0.095888193	2.33E-08	8.55E-08
AC006480.2	1.046959975	-2.069378111	2.33E-08	8.56E-08
CACNA2D3	2.091084933	-0.104203891	2.35E-08	8.62E-08
AC104667.1	1.748235241	-2.802987341	2.36E-08	8.66E-08
PRG4	-1.34140807	7.321295968	2.37E-08	8.68E-08
DUOXA2	3.906857514	2.379440637	2.37E-08	8.69E-08
AC083809.1	4.167844423	0.237247423	2.39E-08	8.76E-08
FOXL1	1.669361525	-0.458911784	2.41E-08	8.81E-08
LINC01139	3.731612535	-0.054862412	2.43E-08	8.88E-08
GTSE1-DT	1.118898888	-2.417974925	2.45E-08	8.98E-08
ZNF32-AS2	1.124293959	-2.048458252	2.45E-08	8.98E-08
AL355803.1	1.481169176	-2.784443159	2.46E-08	8.99E-08
MRPS30-DT	1.097737168	-1.652803998	2.46E-08	8.99E-08
AXIN2	2.093509793	3.349072839	2.46E-08	8.99E-08
TNFRSF19	2.21991299	3.285608027	2.47E-08	9.02E-08
LINC02119	5.423636034	-0.943629973	2.48E-08	9.07E-08
HIST1H2BH	2.487153434	-2.828439246	2.50E-08	9.13E-08
AC140059.1	5.463826313	-2.953702645	2.50E-08	9.15E-08
CRYBA2	4.847215479	-1.506487559	2.51E-08	9.17E-08
AHRR	2.04939676	0.628757281	2.54E-08	9.26E-08
ACTBP8	3.087868118	-1.431912194	2.54E-08	9.29E-08
RGS17	1.438286371	-1.22458247	2.55E-08	9.29E-08
ZNF556	2.50318302	-1.931738389	2.56E-08	9.35E-08
ARHGAP4	1.044023814	4.693812543	2.58E-08	9.40E-08
RHOXF2B	5.907658263	-2.610443747	2.58E-08	9.41E-08
LRGUK	1.309160198	-1.662348122	2.59E-08	9.43E-08
LINC01132	1.112155232	-0.885811558	2.59E-08	9.44E-08
CACNA1E	3.06499731	-0.379056862	2.60E-08	9.48E-08
SIGLEC8	-1.238863572	-0.407824423	2.60E-08	9.48E-08
AC136601.2	-1.649176103	-0.091040353	2.61E-08	9.50E-08
ISM2	4.697276592	-2.029757386	2.63E-08	9.56E-08
CCDC155	2.876855443	-1.640214089	2.67E-08	9.73E-08
CLIP2	1.218768248	4.098644114	2.72E-08	9.88E-08
PRR20G	5.036590366	-3.229525634	2.72E-08	9.91E-08
TRPV4	-1.291505112	2.906831631	2.75E-08	9.99E-08
CPA5	2.355235245	-2.26503678	2.76E-08	1.00E-07
AC244197.3	1.275921011	-1.171897551	2.76E-08	1.00E-07
AC092490.1	3.125659756	0.464953769	2.76E-08	1.00E-07
LIX1	5.209325988	-1.925305263	2.79E-08	1.01E-07
TNNT2	2.151266413	-0.699013827	2.79E-08	1.01E-07
AL645608.8	2.0775109	-2.096949426	2.80E-08	1.01E-07
SCIN	1.865991244	-0.309009599	2.82E-08	1.02E-07
BSN	1.317117323	1.94825329	2.84E-08	1.03E-07
SLC6A15	6.218141224	-0.706936224	2.84E-08	1.03E-07

SOX21	4.019134683	-2.42197226	2.85E-08	1.03E-07
ANKLE1	1.40675945	-1.176262147	2.86E-08	1.04E-07
GJC3	-1.08690732	-0.405363984	2.86E-08	1.04E-07
AK8	1.916656608	-0.49402973	2.86E-08	1.04E-07
ATF5	-1.292998644	9.369037576	2.86E-08	1.04E-07
ABCC8	2.586022754	-0.803118102	2.91E-08	1.05E-07
MEP1B	-1.891962946	-0.396018651	2.91E-08	1.05E-07
SYT2	2.125795678	-0.817490135	2.91E-08	1.05E-07
OXCT1	1.76672223	2.306487053	2.92E-08	1.06E-07
SLC13A4	1.06163275	-0.639869615	2.94E-08	1.06E-07
LINC02055	3.34936929	-0.313302873	2.95E-08	1.07E-07
AL391427.1	1.376805855	-0.735824318	2.96E-08	1.07E-07
FABP7	5.21462918	-0.752313818	2.99E-08	1.08E-07
AL353622.1	1.006970807	0.43939162	3.02E-08	1.09E-07
LINC01605	3.06007841	-2.77671342	3.03E-08	1.09E-07
AL117339.4	1.279064628	-2.593887137	3.03E-08	1.09E-07
RPSAP18	1.129726698	-1.556573637	3.04E-08	1.10E-07
MAP1B	1.066219194	3.695446557	3.06E-08	1.10E-07
ACOT12	-1.174132624	5.266096908	3.06E-08	1.10E-07
DRC7	1.241255368	-0.231146715	3.07E-08	1.11E-07
IFIT1	-1.111314023	5.568589441	3.07E-08	1.11E-07
AC009271.1	3.999613599	-3.033285025	3.12E-08	1.12E-07
GPR37L1	1.371417451	-0.765916035	3.18E-08	1.14E-07
LINC00879	5.668320459	-2.228149002	3.19E-08	1.15E-07
AL137230.1	1.376744132	-1.719059155	3.19E-08	1.15E-07
ENTPD3	2.72597063	-1.401599162	3.20E-08	1.15E-07
GNAT1	1.393677054	-0.632403454	3.22E-08	1.16E-07
AC011944.1	2.514683262	-2.702874112	3.23E-08	1.16E-07
SRPK3	1.437315952	-0.84551677	3.23E-08	1.16E-07
CT55	5.386134415	-2.638299545	3.24E-08	1.16E-07
VAC14-AS1	1.812959835	0.238616414	3.25E-08	1.17E-07
TDRD5	3.834993274	-1.521968356	3.28E-08	1.18E-07
ATP8B3	1.100461268	1.206039853	3.28E-08	1.18E-07
NDRG1	1.165610321	7.67943258	3.29E-08	1.18E-07
ZBED2	2.357696914	-1.533523158	3.30E-08	1.18E-07
ANKRD45	1.78838716	-1.608955903	3.36E-08	1.20E-07
YWHAEP7	3.017703383	-2.172188651	3.37E-08	1.21E-07
SCGB1A1	5.760156092	-1.795325636	3.42E-08	1.22E-07
AL592166.1	1.113058512	-2.681837541	3.43E-08	1.23E-07
UNC5D	5.331473553	-0.112315559	3.44E-08	1.23E-07
SIRPB1	-1.105934138	-0.191357089	3.44E-08	1.23E-07
LINC00689	3.429105137	-1.127414086	3.48E-08	1.24E-07
RSPH14	1.58589525	-1.353269649	3.49E-08	1.25E-07
ANO4	2.098148229	-1.565901937	3.50E-08	1.25E-07
AL590079.1	-1.63390382	-2.350564289	3.51E-08	1.25E-07
CASC22	3.281168441	-2.472239623	3.54E-08	1.26E-07
AL445483.1	2.277625981	-1.777969057	3.61E-08	1.29E-07
LINC01511	4.65220285	-3.021245728	3.61E-08	1.29E-07
AC093724.1	1.270030593	-1.730653233	3.63E-08	1.30E-07
LGI2	1.427594898	0.519306233	3.64E-08	1.30E-07
HPSE2	2.766973308	-2.724639193	3.64E-08	1.30E-07
ASLP1	1.314018166	-2.432148316	3.65E-08	1.30E-07
AC083880.1	1.070506928	-1.516350916	3.67E-08	1.31E-07
ZNF732	1.755798862	-3.008607995	3.69E-08	1.31E-07
AP006285.2	-1.600419253	-1.863963487	3.70E-08	1.32E-07
MYCNOS	2.711989408	-2.715743401	3.71E-08	1.32E-07
LAMP5	2.892583187	0.873833043	3.72E-08	1.32E-07
HIST1H3G	2.863500403	-2.109957056	3.72E-08	1.32E-07

AR	-1.136223992	6.456333753	3.74E-08	1.33E-07
PI4KAP1	1.089601149	1.148042032	3.75E-08	1.33E-07
DCLK3	1.349520683	-2.051292298	3.77E-08	1.34E-07
VCAN	1.913775152	4.94505479	3.80E-08	1.35E-07
AC015922.2	-1.14625374	-2.171236794	3.83E-08	1.36E-07
AL583810.1	1.243499094	-2.89846014	3.88E-08	1.37E-07
BRDT	5.067620327	-1.455731009	3.92E-08	1.39E-07
MELTF	1.667545877	3.216020964	3.92E-08	1.39E-07
NPNT	1.739645752	3.909029288	3.94E-08	1.39E-07
CMPK2	-1.029770131	2.929838488	3.97E-08	1.41E-07
LINC00856	3.261725024	-3.115788752	3.98E-08	1.41E-07
LINC00885	-1.78635196	-0.079555753	3.98E-08	1.41E-07
AC026368.1	1.971221348	-2.805596631	4.00E-08	1.41E-07
SLC30A2	3.023219199	1.718579772	4.01E-08	1.42E-07
LGALS8-AS1	1.281510058	-1.669852317	4.04E-08	1.43E-07
C2orf40	-1.309603807	-1.379505693	4.04E-08	1.43E-07
SERPINA3	-1.131954418	4.450103642	4.05E-08	1.43E-07
GAGE1	6.430783377	-1.382282121	4.06E-08	1.43E-07
IL13RA2	-1.696297732	0.941909384	4.08E-08	1.44E-07
DOC2GP	1.281089162	-1.36588637	4.08E-08	1.44E-07
PMPCAP1	3.570370518	-3.018042882	4.09E-08	1.44E-07
AC055822.1	1.0219785	-1.739355941	4.10E-08	1.45E-07
WIF1	5.293132893	-0.692027729	4.14E-08	1.46E-07
AC061975.6	5.242934986	-1.420177706	4.17E-08	1.47E-07
LIN28A	5.088922476	-2.09839768	4.19E-08	1.48E-07
SLC22A15	1.712046771	1.653185385	4.20E-08	1.48E-07
AC138150.2	1.262761464	-1.406700026	4.21E-08	1.48E-07
FMO3	-1.130971516	8.515884182	4.23E-08	1.49E-07
ATP5F1AP3	-1.014563067	-2.056418761	4.32E-08	1.52E-07
OTOP3	5.330583759	-2.082314882	4.32E-08	1.52E-07
AC112206.2	-1.58120607	2.323311689	4.37E-08	1.53E-07
PDK4	-1.199525801	7.407543227	4.37E-08	1.54E-07
MYOM3	1.425472683	-1.367965009	4.39E-08	1.54E-07
COMP	2.827770046	1.370195557	4.42E-08	1.55E-07
Z98257.1	1.597988324	0.867045516	4.45E-08	1.56E-07
FAM86GP	1.354566495	-2.703810446	4.45E-08	1.56E-07
TSPY2	6.53133298	-2.129870272	4.51E-08	1.58E-07
AC091390.3	1.158697573	-2.623478966	4.52E-08	1.58E-07
EVPL	2.67695931	1.795996701	4.52E-08	1.59E-07
KLHDC8A	2.294388306	-0.975315942	4.54E-08	1.59E-07
AC078925.4	5.410210807	-0.91772312	4.55E-08	1.59E-07
AC040970.1	1.871816961	-1.189355691	4.57E-08	1.60E-07
CR382285.1	3.160082934	-2.548241631	4.62E-08	1.62E-07
AC104986.2	1.031914991	-0.269681191	4.64E-08	1.63E-07
FAM83F	-1.784153719	0.911527436	4.64E-08	1.63E-07
JPH2	1.676595501	-0.100037674	4.65E-08	1.63E-07
STX16-NPEPL1	1.102991483	-0.766604741	4.66E-08	1.63E-07
MUCL1	5.969534252	1.020296022	4.66E-08	1.63E-07
LINC02487	1.420457473	-0.472396139	4.71E-08	1.65E-07
HCG9	1.677421143	-2.76732635	4.78E-08	1.67E-07
PPP1R1B	3.532178163	1.300476869	4.80E-08	1.68E-07
ADAMTS14	1.443995146	0.148618009	4.81E-08	1.68E-07
MSANTD1	1.013772862	-2.005658048	4.83E-08	1.69E-07
ALX4	3.666543073	-2.439086196	4.86E-08	1.70E-07
IQANK1	1.484682603	0.568690271	4.87E-08	1.70E-07
AL121672.1	1.492074662	-2.954130175	4.88E-08	1.70E-07
AL354707.3	1.285488064	-2.180016569	4.88E-08	1.70E-07
OASL	-1.118528511	4.690351404	4.95E-08	1.73E-07

AC010491.1	1.046621744	-1.22776465	5.00E-08	1.74E-07
TAGLN2P1	1.037799845	-2.347565204	5.00E-08	1.74E-07
AL355102.4	2.039023301	0.452803897	5.02E-08	1.75E-07
CLPS	10.35911706	1.808262951	5.07E-08	1.76E-07
SPAG6	2.694020632	-2.795945645	5.11E-08	1.78E-07
CT45A10	5.678760343	-2.810423233	5.13E-08	1.78E-07
CAGE1	2.62474707	-2.610752182	5.15E-08	1.79E-07
SLC4A10	-1.290103178	-1.973101	5.15E-08	1.79E-07
RBP5	-1.023816568	6.721930914	5.17E-08	1.80E-07
NUPR1	1.038016742	7.957109383	5.18E-08	1.80E-07
AL139349.1	1.212869315	-2.153347404	5.22E-08	1.81E-07
ANKRD20A19P	5.931397758	-1.151338416	5.29E-08	1.84E-07
CTB-178M22.2	1.755321777	-2.903895182	5.31E-08	1.84E-07
AP003119.1	1.653361275	-1.08403343	5.32E-08	1.85E-07
ATP6V1B1	1.9232031	-0.849451556	5.35E-08	1.86E-07
HSPB1P1	1.21597265	0.417506564	5.36E-08	1.86E-07
TRARG1	5.730923495	0.99972928	5.38E-08	1.87E-07
RPL7P1	1.074813985	0.31607487	5.50E-08	1.90E-07
HSD17B1	1.008552796	1.009011317	5.55E-08	1.92E-07
CDH12	5.488424308	0.743440929	5.58E-08	1.93E-07
ZNF467	1.134830548	3.323935517	5.58E-08	1.93E-07
AC005726.3	1.015876017	-1.733656952	5.67E-08	1.96E-07
LCNL1	3.23270458	-0.58474238	5.69E-08	1.97E-07
LINC02158	3.580512159	2.136926413	5.69E-08	1.97E-07
AL445437.1	2.38854743	-0.932855178	5.78E-08	2.00E-07
AC018638.7	1.144353072	-1.332752709	5.79E-08	2.00E-07
AC105339.2	1.121964567	-2.191240427	5.81E-08	2.01E-07
LINC00501	2.89499106	-3.054802556	5.82E-08	2.01E-07
MRPL35P2	-1.116665759	-2.870577235	5.82E-08	2.01E-07
RPL17P34	1.379977189	-2.725468098	5.86E-08	2.02E-07
LRRC39	1.206592212	-0.505603266	5.86E-08	2.02E-07
LINC00880	2.045940642	-1.333852912	5.88E-08	2.03E-07
AC025265.1	1.202538344	-0.878969184	5.97E-08	2.06E-07
AC253576.2	1.312605473	-2.881536829	5.97E-08	2.06E-07
AC087741.2	1.211560402	-2.132563177	6.02E-08	2.07E-07
RASGEF1A	2.089619435	0.812574759	6.09E-08	2.10E-07
SCGB2A1	2.911604288	-1.485917465	6.10E-08	2.10E-07
AL022393.1	1.707124286	-2.664161599	6.11E-08	2.10E-07
AP003559.1	1.955775664	-2.68513194	6.12E-08	2.11E-07
AL512598.1	1.723084829	-2.464670421	6.14E-08	2.11E-07
AC025048.4	1.205208484	-2.356742264	6.19E-08	2.13E-07
AC090515.2	1.001697718	-2.214048428	6.20E-08	2.13E-07
RHOXF2	5.701523543	-2.331702802	6.21E-08	2.13E-07
GPR150	1.860390939	-1.303565695	6.21E-08	2.14E-07
FAM19A4	4.895290129	0.115493019	6.23E-08	2.14E-07
LINC00494	3.372475103	-0.389182564	6.24E-08	2.14E-07
WNT2B	1.320531799	0.547716894	6.31E-08	2.17E-07
TSPAN15	1.23095384	3.891559978	6.31E-08	2.17E-07
OCA2	3.115986322	1.267179732	6.42E-08	2.20E-07
TFAP2A	1.998642301	0.806199895	6.45E-08	2.21E-07
GPAT3	-1.044352386	3.975626057	6.45E-08	2.21E-07
MAST1	1.397263393	0.935728937	6.49E-08	2.22E-07
KRBA1	1.067126968	1.940114774	6.53E-08	2.24E-07
FAM227A	1.640740771	-1.362585958	6.54E-08	2.24E-07
CDH22	4.469315552	0.423651199	6.63E-08	2.27E-07
GRP	3.811681615	-3.089981712	6.70E-08	2.30E-07
AL353150.1	1.586823447	-1.076265272	6.75E-08	2.31E-07
MMP24	1.550787765	1.748351363	6.85E-08	2.34E-07

ACBD7	1.573559111	-2.24526881	6.86E-08	2.35E-07
RFX6	3.574857409	-1.85919582	6.91E-08	2.36E-07
SLC16A8	1.386718328	-1.326640927	6.96E-08	2.38E-07
SCN7A	-1.589062265	-0.248943332	6.96E-08	2.38E-07
LINC01474	2.214311878	0.515627308	6.96E-08	2.38E-07
CYP3A43	-1.415374326	2.187077148	7.01E-08	2.39E-07
AL645939.1	1.289766605	-2.438817449	7.01E-08	2.39E-07
PIP5KL1	1.407794768	-1.620906764	7.03E-08	2.40E-07
AC022809.1	1.659561345	-2.685054706	7.03E-08	2.40E-07
AC087752.4	1.028682928	-2.569074369	7.06E-08	2.41E-07
KCNH5	4.639783321	-2.345849538	7.12E-08	2.43E-07
MT-TY	-1.04110742	-1.057290978	7.16E-08	2.44E-07
Z82246.1	2.256848158	-0.807498755	7.18E-08	2.45E-07
AC006329.2	-1.311475067	0.493754989	7.19E-08	2.45E-07
MEIS3P1	-1.192789803	-1.841597233	7.21E-08	2.46E-07
HAND2	-1.608524159	1.411177919	7.41E-08	2.52E-07
GXYLT1P6	-1.741340754	-2.800634829	7.42E-08	2.53E-07
AC138207.3	1.730314852	-2.920285456	7.57E-08	2.57E-07
MYH13	3.571327634	-2.814472965	7.60E-08	2.58E-07
SNORC	1.564857366	4.572700195	7.60E-08	2.58E-07
AC023043.4	1.09576874	-1.453767876	7.60E-08	2.58E-07
HIST2H2AC	1.047139048	-0.165133861	7.61E-08	2.59E-07
PIK3C2G	-1.215026582	3.253593021	7.62E-08	2.59E-07
KLHL35	1.815346752	-1.693070677	7.64E-08	2.59E-07
MYO1H	1.639744867	-2.891630259	7.65E-08	2.60E-07
AC019257.1	-1.676743836	-2.177734718	7.67E-08	2.60E-07
ROS1	2.67083978	-0.980513374	7.67E-08	2.61E-07
NRCAM	2.446125217	3.306813228	7.77E-08	2.64E-07
NKX2-2	4.545042221	-2.92894357	7.88E-08	2.68E-07
JAKMIP3	1.669969617	-1.007854407	7.94E-08	2.69E-07
MRLN	4.225906788	-3.066606148	7.94E-08	2.69E-07
PAQR8	1.30940602	2.543779678	8.14E-08	2.76E-07
DWORF	4.814046579	-1.201774388	8.14E-08	2.76E-07
ADRB1	-1.365808189	-0.71184178	8.15E-08	2.76E-07
AC006305.1	3.136019243	-1.719288765	8.20E-08	2.78E-07
EGFEM1P	2.873360403	-2.148246064	8.22E-08	2.78E-07
CCDC80	1.769842233	4.700320053	8.26E-08	2.79E-07
USP54	1.144725657	2.646658657	8.28E-08	2.80E-07
SEMA3B	1.544824965	3.853551043	8.32E-08	2.81E-07
AL035701.1	1.512832846	-1.920071528	8.40E-08	2.84E-07
USH1G	3.423059605	-2.783767565	8.41E-08	2.84E-07
PRAMEF8	3.667121038	-2.482079172	8.41E-08	2.84E-07
AL133338.1	1.060488162	0.470096606	8.49E-08	2.87E-07
AC133919.2	1.739656958	-1.329192825	8.52E-08	2.87E-07
CCL13	1.854940268	-1.227348589	8.53E-08	2.88E-07
AC139887.4	1.382616251	-2.854630876	8.55E-08	2.88E-07
C1QTNF1-AS1	2.373584197	-1.093732095	8.61E-08	2.90E-07
HIST1H1E	1.368372091	-1.866755408	8.61E-08	2.90E-07
GIPR	1.906514642	0.188477521	8.66E-08	2.92E-07
LINC01589	3.277630526	-1.803323565	8.69E-08	2.93E-07
LINC02200	4.814317748	-1.229091914	8.70E-08	2.93E-07
PTPN5	1.848411857	-2.679001491	8.70E-08	2.93E-07
GDAP1	1.007470715	2.202149768	8.72E-08	2.94E-07
IL4I1	1.520350724	1.882866369	8.72E-08	2.94E-07
HOXA-AS2	1.390056802	0.948106426	8.75E-08	2.95E-07
TRIM6	1.296335961	0.925484978	8.78E-08	2.96E-07
CYP8B1	-1.511562112	8.571769246	8.80E-08	2.96E-07
RNU4-1	-1.566707514	-2.420806503	8.81E-08	2.96E-07

CRISPLD2	-1.087788629	4.151052921	8.83E-08	2.97E-07
YBX2	1.968900113	2.219337205	8.89E-08	2.99E-07
MS4A10	4.85800672	-0.725600513	8.91E-08	3.00E-07
AL078604.2	1.551809586	-1.350612837	8.97E-08	3.01E-07
TRNP1	1.729769141	4.175371932	8.97E-08	3.01E-07
LINC02041	2.801999194	-2.128623457	9.11E-08	3.06E-07
DISP3	2.298225857	-2.090015299	9.14E-08	3.07E-07
NAT8B	1.750329898	1.596800009	9.20E-08	3.08E-07
GTF3C2-AS1	1.149905801	-2.878102362	9.22E-08	3.09E-07
APOBEC2	1.570675344	-2.540694402	9.25E-08	3.10E-07
AC135584.1	-1.8005462	-1.326940833	9.26E-08	3.10E-07
NKAIN4	2.902674813	-2.416788327	9.27E-08	3.10E-07
AC244197.2	1.517130749	-2.414969276	9.34E-08	3.13E-07
GRIN2A	2.793249896	0.184883911	9.41E-08	3.15E-07
ZNF728	3.480059321	-2.24251355	9.46E-08	3.17E-07
AC084018.2	1.016735648	-2.020613036	9.56E-08	3.20E-07
BLK	-1.351234465	-0.703216263	9.57E-08	3.20E-07
PHF2P2	5.200427658	-2.50643588	9.72E-08	3.25E-07
PRRT3-AS1	1.315100126	-0.040532373	9.85E-08	3.29E-07
AL356019.2	1.034992484	-1.46017411	9.86E-08	3.29E-07
TLE6	1.301864905	1.647514986	9.90E-08	3.30E-07
HCAR1	2.009055631	-1.956911779	1.00E-07	3.34E-07
TTPA	-1.052829474	5.794591549	1.00E-07	3.35E-07
AC127070.1	1.317457897	-2.665958939	1.00E-07	3.35E-07
SI	6.569744398	0.259630339	1.01E-07	3.37E-07
CTRB1	6.164642479	-1.545340369	1.01E-07	3.37E-07
TUBB4A	2.554904876	3.370156536	1.02E-07	3.41E-07
BSN-DT	1.577310534	-2.449702044	1.03E-07	3.43E-07
AC015912.3	1.390944475	-1.049006316	1.04E-07	3.47E-07
AC109635.4	5.679366229	-1.576718122	1.05E-07	3.50E-07
AC003985.2	6.361068092	-1.118220692	1.06E-07	3.53E-07
AL022322.2	1.086460029	-2.641962898	1.07E-07	3.56E-07
RNU4-2	-1.62963928	-0.98675197	1.07E-07	3.56E-07
AL035446.1	2.516207571	-0.666885622	1.09E-07	3.60E-07
RHBG	2.707228262	5.431057797	1.09E-07	3.61E-07
NEFL	3.925967378	-0.848636595	1.10E-07	3.65E-07
IGFL2	2.907651432	-3.033171961	1.10E-07	3.66E-07
LINC01353	1.590137865	-2.796662883	1.10E-07	3.66E-07
FAM160A1	-1.182320265	-0.973155669	1.11E-07	3.67E-07
CERNA1	1.641030547	-0.701637813	1.11E-07	3.69E-07
KCNG2	1.426475436	-2.6417635	1.12E-07	3.71E-07
CDH11	1.551281711	2.788626846	1.12E-07	3.71E-07
FTHL17	5.521030828	-2.888682642	1.12E-07	3.71E-07
AC016708.1	1.647859187	-2.803814314	1.12E-07	3.73E-07
ZNF793-AS1	1.725773591	-0.704055878	1.13E-07	3.73E-07
SLC30A8	4.110682274	-2.125348899	1.13E-07	3.74E-07
ADGRE4P	-1.08106507	-1.789950581	1.13E-07	3.76E-07
AANAT	1.548850172	-2.972021284	1.15E-07	3.79E-07
AL359265.3	1.092976954	-2.5579691	1.15E-07	3.79E-07
ACTL10	1.227899013	-0.516670724	1.15E-07	3.82E-07
CT45A1	6.011598426	-2.550497513	1.17E-07	3.87E-07
AC007743.1	1.419602713	-2.396804019	1.17E-07	3.88E-07
AL121758.1	1.246397436	-2.759229436	1.18E-07	3.89E-07
COL21A1	1.394786412	1.55302765	1.20E-07	3.95E-07
OSR1	1.770389519	0.29412902	1.20E-07	3.96E-07
CCDC192	1.469451757	-2.475956167	1.20E-07	3.96E-07
LINC01948	1.70242948	-2.746060269	1.21E-07	3.98E-07
SCARA5	-1.760172543	0.708126864	1.21E-07	4.00E-07

TNNI3	3.465957091	-1.150732405	1.23E-07	4.05E-07
CASC9	3.329322655	1.505864502	1.23E-07	4.06E-07
BCAM	1.002922827	7.365880012	1.23E-07	4.06E-07
ZFR2	2.720611167	-1.356173869	1.24E-07	4.07E-07
SCUBE2	2.19161815	2.249315816	1.25E-07	4.11E-07
FBXW11P1	-1.092746263	-1.320525241	1.25E-07	4.11E-07
RARRES1	1.640265088	3.281372017	1.25E-07	4.12E-07
AC012073.1	1.144366367	-0.999242927	1.25E-07	4.12E-07
AC005837.1	1.161645392	-2.604580498	1.29E-07	4.22E-07
AL513329.1	1.206588128	-2.757894532	1.29E-07	4.22E-07
NUGGC	-1.159033974	4.07251176	1.29E-07	4.24E-07
CASQ2	1.382655298	0.163543875	1.29E-07	4.24E-07
CHST10	1.228032234	1.467023198	1.29E-07	4.24E-07
PINCR	4.730374491	-3.156985709	1.30E-07	4.25E-07
ERVMER61-1	4.49492534	-2.738184977	1.30E-07	4.27E-07
FMO1	2.206486957	2.150450685	1.30E-07	4.27E-07
LINC00535	1.64998194	-0.01753414	1.31E-07	4.28E-07
AL365361.1	-1.095391801	-0.338510003	1.31E-07	4.29E-07
LINC01714	-1.347078608	-2.006664754	1.31E-07	4.29E-07
LINC00839	1.727424686	-0.790495147	1.32E-07	4.31E-07
POLN	1.153247507	0.731213762	1.32E-07	4.31E-07
CTRB2	5.61196492	-0.899063878	1.33E-07	4.34E-07
AP000894.4	1.037420159	-0.844230922	1.34E-07	4.37E-07
MAGEA10	5.409591249	-1.527586113	1.35E-07	4.40E-07
AC005911.1	1.061431949	-2.48256322	1.35E-07	4.41E-07
EIF5A2	1.383068887	2.55174068	1.35E-07	4.42E-07
SYT8	3.215589573	2.478205794	1.36E-07	4.45E-07
TRIM72	2.517945058	-1.07909356	1.36E-07	4.45E-07
ZNF300P1	1.971443167	-0.098316149	1.37E-07	4.47E-07
COL1A2	1.392148537	7.76222179	1.37E-07	4.47E-07
NFE2L3	1.033970842	2.744838583	1.38E-07	4.51E-07
ZNF257	1.647608557	-0.443807204	1.39E-07	4.55E-07
PA2G4P4	1.018638036	-2.416257594	1.40E-07	4.56E-07
DKK2	1.757293037	-0.098195247	1.40E-07	4.57E-07
ADAM32	1.962561142	-2.051866735	1.40E-07	4.57E-07
DNAJB13	1.870438684	-2.560766568	1.41E-07	4.59E-07
PRODH2	-1.038622847	6.433638699	1.41E-07	4.60E-07
AC023906.5	2.312016358	-1.144635382	1.42E-07	4.62E-07
LINC01164	4.557786611	-2.475943745	1.43E-07	4.64E-07
AL451074.6	2.598700809	-2.567548412	1.43E-07	4.66E-07
AC010336.2	-1.161506335	-1.166677472	1.43E-07	4.66E-07
PAQR5	1.821872598	3.345755067	1.44E-07	4.66E-07
AC011447.3	1.486024079	-2.274795528	1.44E-07	4.67E-07
KC877373.1	2.420914397	-2.626814445	1.44E-07	4.68E-07
LINC01681	3.14120233	-1.719556116	1.44E-07	4.69E-07
C1orf158	3.887643436	-2.842300727	1.46E-07	4.73E-07
CLIC5	1.392790334	1.549598928	1.46E-07	4.75E-07
ZDBF2	-1.161973234	1.33593294	1.47E-07	4.76E-07
STAR	2.696875578	-0.987421855	1.48E-07	4.79E-07
INHBA	-1.016231448	3.676639273	1.48E-07	4.81E-07
ZNF723	4.378558291	-2.466548451	1.48E-07	4.81E-07
CDH10	5.280727857	-1.332506248	1.53E-07	4.96E-07
COL5A3	1.302393561	5.717488476	1.55E-07	5.01E-07
SLC1A3	1.184187059	2.73882896	1.56E-07	5.04E-07
TRIM74	1.710677128	-2.196722737	1.56E-07	5.04E-07
TTYH2	1.135295481	2.128872471	1.56E-07	5.05E-07
AC005005.3	1.08673227	-1.692032094	1.57E-07	5.09E-07
RAB34	1.443117586	4.155867388	1.58E-07	5.10E-07

MSMB	2.584968513	-0.935776768	1.58E-07	5.10E-07
AP000695.2	1.604953207	-2.272797954	1.58E-07	5.11E-07
MPPED2	1.390783439	0.299582271	1.59E-07	5.14E-07
FTLP12	1.521908538	-2.534519352	1.60E-07	5.15E-07
SLC44A5	2.335526755	2.055016318	1.60E-07	5.17E-07
HNRNPA1P21	1.454994126	-1.513428389	1.60E-07	5.18E-07
ATP1A2	2.094127249	1.907738574	1.61E-07	5.20E-07
PNMA6F	5.00266437	-2.801390198	1.61E-07	5.20E-07
SNPH	1.201108123	0.251636461	1.62E-07	5.22E-07
AC010457.1	3.224872314	-2.611639552	1.62E-07	5.23E-07
MYLK2	1.570226462	-2.376898419	1.62E-07	5.24E-07
AC025165.4	1.03621124	-2.886833362	1.63E-07	5.25E-07
ADAMTS6	1.271518294	-0.42530655	1.64E-07	5.27E-07
FLT3	-1.130459818	-0.918850677	1.64E-07	5.29E-07
HMGN5	-1.007660975	2.337403076	1.64E-07	5.30E-07
SLC51B	1.811953038	3.020805686	1.65E-07	5.30E-07
AC011487.1	1.924361021	-2.515457149	1.65E-07	5.31E-07
ADAMTS20	3.446480259	-3.058758524	1.71E-07	5.48E-07
CBLN1	1.556281699	1.143631742	1.72E-07	5.54E-07
LINC01301	1.141621799	-1.044999448	1.73E-07	5.54E-07
IGFL2-AS1	3.78563807	-2.753122357	1.73E-07	5.55E-07
SLC5A1	-1.955017324	1.323173074	1.74E-07	5.59E-07
C1orf229	1.336407467	-1.162669077	1.76E-07	5.66E-07
TMEM119	1.659103662	1.96303547	1.78E-07	5.70E-07
AC134682.1	1.622910852	-2.083295668	1.78E-07	5.71E-07
ZNF793	1.269780738	0.431241897	1.79E-07	5.73E-07
LINC02362	-1.217582755	3.037512941	1.79E-07	5.74E-07
CLRN3	-1.554706306	3.654187697	1.79E-07	5.75E-07
LINC01448	4.074380659	-2.773395354	1.79E-07	5.75E-07
SULT1A2	-1.027781076	4.10496754	1.80E-07	5.76E-07
NKX1-2	4.973097603	-1.450275145	1.80E-07	5.76E-07
SCNN1G	4.689717837	-1.235228265	1.80E-07	5.77E-07
CNGB3	2.275247761	-2.866191028	1.81E-07	5.80E-07
AC025271.4	1.186352341	-1.573374674	1.81E-07	5.80E-07
HIST1H3C	1.526077014	-2.778642074	1.82E-07	5.84E-07
ABCA4	1.191177536	1.176434448	1.84E-07	5.88E-07
LINC02587	3.175960574	-2.21690894	1.87E-07	5.97E-07
AC105446.1	2.802763415	-1.920240341	1.87E-07	5.99E-07
C10orf55	1.705666623	-2.885938462	1.89E-07	6.04E-07
LINC00887	2.106744692	-1.153288785	1.89E-07	6.04E-07
AC008622.2	1.556103493	-2.671579487	1.93E-07	6.15E-07
GABRA2	5.178894049	0.706874037	1.95E-07	6.21E-07
LAIR2	1.939228467	-1.490327386	1.95E-07	6.23E-07
SSTR3	2.789765612	-1.708420314	1.96E-07	6.26E-07
SH2D5	1.977148224	-1.880197226	1.96E-07	6.26E-07
LINC00958	4.189677644	-1.400226129	1.97E-07	6.27E-07
LINC00239	2.184337625	-1.274783363	1.97E-07	6.29E-07
CAPN14	1.940584104	-2.889895596	1.98E-07	6.31E-07
DDN	2.07763204	-2.610234128	1.98E-07	6.32E-07
AL445673.1	1.917579889	-2.691751634	2.03E-07	6.46E-07
AC109460.2	1.146714067	-1.827535824	2.04E-07	6.49E-07
NELL1	6.445678168	-0.471226766	2.04E-07	6.51E-07
AL513318.2	3.52555657	-1.62265841	2.04E-07	6.51E-07
PERM1	1.535223223	-0.432860457	2.05E-07	6.52E-07
RTL9	2.056498606	-0.166992178	2.06E-07	6.55E-07
MAPK13	1.571165523	3.95085536	2.08E-07	6.61E-07
MMP13	4.471818985	-2.383538006	2.08E-07	6.61E-07
AC006378.2	1.380243639	-2.65535648	2.08E-07	6.63E-07

COX6B2	2.268280445	-2.367644995	2.08E-07	6.63E-07
TPTEP1	-1.26030054	-1.45100887	2.08E-07	6.63E-07
SMR3A	7.247169335	-1.53325792	2.09E-07	6.64E-07
GBP7	-1.251186299	4.824793852	2.10E-07	6.68E-07
ULBP1	1.882470544	-1.655411007	2.11E-07	6.71E-07
MFAP2	1.79898708	1.260243616	2.12E-07	6.74E-07
SLC52A3	1.655892045	0.856074625	2.13E-07	6.75E-07
AC012213.3	1.242822004	-2.864408916	2.13E-07	6.77E-07
KIF1A	2.809315857	2.546678927	2.15E-07	6.83E-07
GDNF-AS1	2.538410501	-0.427222541	2.16E-07	6.85E-07
CACNG8	1.371563376	-2.079883421	2.20E-07	6.99E-07
HOXC8	3.710697606	-2.471228847	2.23E-07	7.07E-07
AC108865.1	3.900444396	-2.945390302	2.26E-07	7.16E-07
SNORA33	1.002086435	-0.913113362	2.26E-07	7.16E-07
PLA2R1	-1.057596474	0.638688197	2.26E-07	7.16E-07
STK32B	1.419808291	-0.693651317	2.27E-07	7.17E-07
ADAMTS9-AS1	-1.017937726	-1.634473873	2.27E-07	7.17E-07
DCX	2.261404269	-1.776225437	2.27E-07	7.19E-07
NKX6-3	6.618720271	-1.157812342	2.31E-07	7.30E-07
CPT1C	1.004366229	0.8086143	2.31E-07	7.30E-07
AC009686.2	1.332302884	-1.431552335	2.31E-07	7.31E-07
ROCK1P1	1.174479789	-1.509779445	2.31E-07	7.31E-07
WISP2	-1.562065448	1.246176408	2.32E-07	7.33E-07
TNFRSF9	2.00277673	1.359609659	2.32E-07	7.33E-07
DAPL1	5.135475951	0.053359318	2.32E-07	7.34E-07
AL645940.1	1.056326549	-2.375203834	2.33E-07	7.36E-07
PDZD2	1.05196077	1.254812217	2.35E-07	7.42E-07
SLC28A1	-1.252486211	5.406990336	2.36E-07	7.43E-07
SNHG27	4.510860355	-2.522743684	2.38E-07	7.49E-07
ZNF788P	1.191927806	0.545256435	2.40E-07	7.57E-07
PRAMEF7	3.621350586	-2.594735252	2.41E-07	7.60E-07
SNORA11F	1.145949469	-2.817470011	2.42E-07	7.63E-07
AC253536.6	1.428048612	0.022467119	2.42E-07	7.63E-07
NLGN1	2.91366061	-1.476145581	2.43E-07	7.65E-07
AL033384.1	2.066554241	-2.574498542	2.44E-07	7.67E-07
HOXC10	4.884183879	0.035990837	2.46E-07	7.73E-07
KIF26B	1.733534681	2.017079705	2.50E-07	7.85E-07
ADRB2	-1.006456274	2.89507499	2.50E-07	7.86E-07
AC005740.4	1.017100183	-2.853495898	2.53E-07	7.96E-07
SLC5A5	2.463025748	-2.185329905	2.54E-07	7.97E-07
SOX21-AS1	3.504459807	-2.612664623	2.54E-07	7.98E-07
AC011379.2	1.104760728	-1.079448691	2.54E-07	7.99E-07
AC008738.2	1.368083483	-2.389685091	2.55E-07	8.01E-07
ANK3	-1.13739454	3.017182625	2.57E-07	8.08E-07
C6orf222	3.467185736	-1.046712413	2.58E-07	8.09E-07
AC005519.1	1.080352099	-1.922351113	2.59E-07	8.11E-07
FOXN4	2.377005501	1.59918314	2.59E-07	8.11E-07
MKRN4P	3.668993915	-2.256711291	2.59E-07	8.12E-07
CHST5	4.221515646	-2.069265112	2.60E-07	8.14E-07
PFKP	1.511177521	4.055854785	2.63E-07	8.25E-07
AL591686.1	5.473214207	-2.488551486	2.64E-07	8.25E-07
PQLC2L	3.345858629	-1.867002147	2.64E-07	8.25E-07
LINC01702	-1.434633336	2.923662809	2.66E-07	8.31E-07
AL390728.2	1.529260404	-2.839064198	2.67E-07	8.36E-07
RGSL1	3.125734908	1.078212304	2.67E-07	8.36E-07
NAP1L6	3.235575198	-1.775464019	2.68E-07	8.38E-07
AL358394.1	4.781192973	-3.209004603	2.69E-07	8.40E-07
AC111182.1	1.716912109	-1.600645909	2.70E-07	8.44E-07

ZNF714	1.338704902	0.361471324	2.71E-07	8.48E-07
HSPA7	1.644344851	1.68505533	2.74E-07	8.56E-07
AC008105.1	1.424947214	-2.044471344	2.75E-07	8.59E-07
TUBB3	2.027320798	-1.438446031	2.75E-07	8.59E-07
GATA2-AS1	1.550975944	-0.413909467	2.76E-07	8.62E-07
TACSTD2	-1.518462845	2.543679033	2.78E-07	8.69E-07
FBLN1	1.465928548	5.339780608	2.81E-07	8.76E-07
KHDC1	1.910956432	-2.041129246	2.84E-07	8.85E-07
CCNI2	1.558866236	-1.869462606	2.86E-07	8.90E-07
PLAC4	5.161450423	0.565961339	2.87E-07	8.93E-07
ERICH3	-1.191136365	-1.645181983	2.87E-07	8.94E-07
CYP27C1	1.57037266	-0.64100937	2.88E-07	8.98E-07
AC138904.1	2.13084993	-2.921646473	2.89E-07	8.98E-07
CFAP43	1.081054424	-0.380307352	2.91E-07	9.06E-07
UBA52P6	1.218256597	-2.898287279	2.92E-07	9.07E-07
ANO9	1.604500366	2.057332858	2.92E-07	9.09E-07
AC068134.3	1.169136405	-2.682026591	2.93E-07	9.11E-07
LINC02298	1.7607234	-0.505930169	2.95E-07	9.17E-07
AL158071.3	1.563211611	-2.866250903	2.98E-07	9.26E-07
AL355482.1	1.916105827	-0.296646553	2.99E-07	9.28E-07
SIPA1L2	1.102430001	4.808591703	3.02E-07	9.37E-07
TEKT5	1.487276052	-1.090171859	3.02E-07	9.38E-07
KRTAP5-AS1	1.809161887	-1.441165816	3.06E-07	9.49E-07
WNT2	-1.78631723	-0.757212999	3.07E-07	9.51E-07
AL118511.1	1.190356337	-2.807314137	3.07E-07	9.53E-07
AF274858.1	1.085080441	0.075534749	3.07E-07	9.53E-07
VSTM5	1.689372099	-2.709340624	3.10E-07	9.61E-07
TSPY1	5.829713095	-2.68748993	3.11E-07	9.62E-07
GAL3ST4	1.195156779	1.187354156	3.11E-07	9.63E-07
AC034228.1	5.147010438	-2.676456891	3.12E-07	9.68E-07
AC006449.2	1.090786598	-2.963209286	3.13E-07	9.71E-07
ACTA1	1.360028655	-1.70158498	3.14E-07	9.72E-07
AC073389.3	1.127891192	-2.220051699	3.17E-07	9.82E-07
ABCA12	2.494982858	-1.569990995	3.19E-07	9.87E-07
AP003716.1	-1.35150877	2.753915251	3.23E-07	9.99E-07
C2orf27A	1.149834639	-0.209952241	3.24E-07	1.00E-06
GREB1	1.494696584	3.794531646	3.24E-07	1.00E-06
PPIAP9	1.253931006	-2.82620089	3.26E-07	1.01E-06
RIMKLBP2	1.28550121	-2.650847503	3.26E-07	1.01E-06
AC011815.2	1.298970405	-2.474385052	3.27E-07	1.01E-06
VNN2	1.679890541	3.831980673	3.27E-07	1.01E-06
RBM12B-AS1	1.018078796	-2.434404278	3.28E-07	1.01E-06
AC005534.1	1.178071192	-2.541189944	3.29E-07	1.02E-06
MYH3	1.638133109	2.211315537	3.31E-07	1.02E-06
AC026469.1	-1.278421301	-1.543133807	3.31E-07	1.02E-06
SMTNL2	1.817808425	-2.17845803	3.33E-07	1.03E-06
DCXR	-1.018019479	9.434421916	3.34E-07	1.03E-06
AL122125.1	1.306028863	-2.897138853	3.37E-07	1.04E-06
KLHL34	2.676656081	-0.873074344	3.37E-07	1.04E-06
MUSTN1	1.940452799	-1.92317966	3.38E-07	1.04E-06
HSP90AB2P	1.032190038	-0.866897537	3.39E-07	1.04E-06
AC102953.1	1.268441165	-2.874424128	3.40E-07	1.05E-06
COL4A6	2.361200065	-0.214595827	3.40E-07	1.05E-06
LTBP2	1.209131011	4.687227192	3.43E-07	1.05E-06
AC006435.2	1.068008204	-1.753278507	3.46E-07	1.06E-06
CHN1	1.081573182	1.579353323	3.47E-07	1.07E-06
SSC5D	-1.100838745	1.563839608	3.47E-07	1.07E-06
PTCRA	-1.115632165	-2.305589288	3.47E-07	1.07E-06

AP001972.4	4.305508154	-2.200434753	3.48E-07	1.07E-06
STC1	1.091522602	3.593391054	3.48E-07	1.07E-06
CRISP2	5.465682047	-1.338418479	3.53E-07	1.08E-06
SGCA	-1.194665311	0.650987751	3.53E-07	1.08E-06
MCOLN3	2.529428547	0.887468568	3.54E-07	1.09E-06
AL353572.1	2.033682492	-2.651125126	3.56E-07	1.09E-06
CYP21A1P	1.143034719	2.264089408	3.58E-07	1.10E-06
NRSN2	1.299886427	3.537480147	3.58E-07	1.10E-06
AC132938.5	1.287264338	-2.443367402	3.60E-07	1.10E-06
AL121601.1	1.235489201	-2.709288709	3.64E-07	1.12E-06
FSTL5	3.672841199	0.489274314	3.64E-07	1.12E-06
FOXD1	3.088226202	-2.324846573	3.66E-07	1.12E-06
ADAMTS9-AS2	-1.015972717	-2.120517016	3.70E-07	1.13E-06
ARHGDIG	2.771744109	-2.572414235	3.71E-07	1.13E-06
PLA2G4F	3.088900412	-1.5686415	3.71E-07	1.14E-06
GRIK2	2.618424668	-0.738857203	3.76E-07	1.15E-06
AL138781.1	1.192954139	-1.80269858	3.82E-07	1.17E-06
CA9	3.088135263	3.74795352	3.83E-07	1.17E-06
UGT1A1	-1.284683268	6.394595728	3.83E-07	1.17E-06
CT83	4.951809582	-2.592214899	3.84E-07	1.17E-06
MISP	3.105489617	2.860532399	3.86E-07	1.18E-06
AC040173.1	2.96924141	-2.451798997	3.92E-07	1.19E-06
SYNGR3	1.830050892	-0.542952038	3.94E-07	1.20E-06
RYR2	2.168437343	0.633874987	3.98E-07	1.21E-06
KIAA1549	1.471034739	2.259922803	3.98E-07	1.21E-06
AC006213.3	1.891684488	-2.32111315	4.00E-07	1.22E-06
AC005224.3	-1.272623145	-2.520513251	4.07E-07	1.24E-06
STAC2	3.124169059	-2.474847179	4.08E-07	1.24E-06
AC083843.3	1.066047702	-0.073924514	4.13E-07	1.25E-06
CCDC144NL	3.998765308	-2.875170326	4.14E-07	1.26E-06
REN	1.963366901	1.103053715	4.14E-07	1.26E-06
AC022098.2	3.567270049	-2.723356927	4.15E-07	1.26E-06
GAS1	-1.151308692	0.735948822	4.20E-07	1.27E-06
GCG	5.377450628	-1.10360869	4.24E-07	1.29E-06
AC026992.2	2.270453455	-2.583183348	4.25E-07	1.29E-06
PKIA	1.998861749	-0.130849312	4.27E-07	1.30E-06
SLC47A2	1.123419495	-0.922271012	4.29E-07	1.30E-06
RIPPLY3	1.808761346	-0.550031521	4.31E-07	1.31E-06
ERVH48-1	2.862737086	-1.026197897	4.33E-07	1.31E-06
GPR1-AS	4.989768306	-1.380774818	4.34E-07	1.31E-06
AC095057.3	1.017801689	-0.802632586	4.38E-07	1.33E-06
CRACR2B	1.292362913	2.711818986	4.38E-07	1.33E-06
AC012485.2	1.440412807	-2.9103128	4.40E-07	1.33E-06
AC126175.1	4.21864402	-2.888674967	4.42E-07	1.34E-06
SLC45A2	1.864572746	0.178836104	4.46E-07	1.35E-06
C19orf33	2.162376888	0.771913657	4.47E-07	1.35E-06
ZNF735	5.320621043	-2.584639396	4.47E-07	1.35E-06
TMEM45A	-1.201346693	5.282841254	4.49E-07	1.36E-06
DLGAP2	-1.640666619	-2.070667633	4.52E-07	1.37E-06
GJB3	-1.561074647	-0.048076148	4.53E-07	1.37E-06
PCDHB3	1.906487312	-0.346531	4.53E-07	1.37E-06
HBA1	-1.486007087	-2.00787794	4.54E-07	1.37E-06
SLC5A2	1.298660791	-1.227114658	4.58E-07	1.38E-06
AL590004.3	1.748786907	0.447231821	4.58E-07	1.38E-06
BDKRB2	1.389091317	2.466197631	4.61E-07	1.39E-06
UTS2B	1.938582682	-0.810115631	4.80E-07	1.45E-06
GOLGA2P11	3.135536163	-2.762846421	4.85E-07	1.46E-06
AL807757.2	1.464590988	-2.09338274	4.88E-07	1.47E-06

ASRGL1	1.184207192	2.651898716	4.89E-07	1.47E-06
C5orf66	1.523107032	-0.128792715	4.89E-07	1.47E-06
AL356490.1	1.664661466	-2.329105984	4.89E-07	1.47E-06
ADAM21	1.565054083	-2.224050426	4.92E-07	1.48E-06
ARMC12	1.030831433	-0.934560035	4.95E-07	1.49E-06
PEX5L	1.341035174	-2.847505856	4.97E-07	1.49E-06
SLC2A6	1.089646958	3.150271044	5.03E-07	1.51E-06
GFAP	2.28848044	0.571067712	5.04E-07	1.51E-06
OR7E29P	-1.380086587	-2.489890867	5.04E-07	1.52E-06
ZNF726	1.26823929	1.071301322	5.08E-07	1.52E-06
ATAD3C	-1.252771263	1.777913673	5.08E-07	1.52E-06
MGAM2	3.241835183	0.748559323	5.14E-07	1.54E-06
TRPC4	1.394177362	-0.880319935	5.15E-07	1.55E-06
AC064807.2	2.46842462	-2.463759423	5.16E-07	1.55E-06
UGT2B27P	-1.213264899	1.958981839	5.17E-07	1.55E-06
PTF1A	4.818475164	-2.433005608	5.19E-07	1.56E-06
AC092598.1	3.620250569	-2.819579963	5.24E-07	1.57E-06
NWD2	3.12550549	0.091480054	5.27E-07	1.58E-06
ZNF93	1.164561077	0.472168186	5.29E-07	1.58E-06
PLPP2	2.080817399	4.331416412	5.29E-07	1.58E-06
FZD9	2.39113901	-0.23038911	5.30E-07	1.59E-06
TSPY10	5.921171624	-2.636410934	5.32E-07	1.59E-06
TMEM72	2.445416721	-0.424733976	5.34E-07	1.60E-06
TCP1P3	3.149971076	-1.205470793	5.40E-07	1.61E-06
CDH16	2.499279718	1.3950544	5.42E-07	1.62E-06
SULT2A1	-1.121965844	9.0960308	5.52E-07	1.65E-06
ULK4	1.115052935	2.637837382	5.52E-07	1.65E-06
LRP4	1.650269693	1.881666926	5.56E-07	1.66E-06
BMP7	3.652433956	0.25564083	5.57E-07	1.66E-06
SNCAIP	1.45728633	1.600711009	5.60E-07	1.67E-06
CLVS2	4.877743617	0.234613855	5.62E-07	1.68E-06
HSD17B3	1.216799286	1.234068407	5.62E-07	1.68E-06
GSPT2	-1.112572831	2.904007727	5.62E-07	1.68E-06
DNAH10OS	1.085392863	0.270727869	5.65E-07	1.68E-06
AC018638.6	1.472058324	-2.333424776	5.68E-07	1.69E-06
AC006213.4	1.047114678	-1.058639591	5.72E-07	1.70E-06
EDDM3A	7.119059832	-1.385767818	5.75E-07	1.71E-06
OLIG3	5.426267727	-2.82307886	5.79E-07	1.72E-06
SST	8.512359201	1.497775189	5.84E-07	1.74E-06
MATN4	2.852334581	-0.638492122	5.86E-07	1.74E-06
TRAPPC13P1	2.233711902	-1.509527639	5.86E-07	1.74E-06
PNMT	2.757678385	-0.931768697	5.94E-07	1.77E-06
ARG1	-1.08565853	8.532640876	5.95E-07	1.77E-06
LINC00479	1.582450148	-2.048180814	6.02E-07	1.79E-06
AP000844.2	4.471427682	-2.659778007	6.15E-07	1.83E-06
TIMM8AP1	1.265974387	-2.23706094	6.16E-07	1.83E-06
OTC	-1.129505075	6.729874701	6.17E-07	1.83E-06
TSPAN11	-1.128393785	-0.110328216	6.19E-07	1.84E-06
AC067930.3	1.36838217	-2.910211652	6.25E-07	1.85E-06
AC006330.1	1.045172946	-2.943033	6.26E-07	1.86E-06
SPATA31D1	4.929811287	-3.112100316	6.26E-07	1.86E-06
REC114	3.188028571	-3.030650695	6.35E-07	1.88E-06
USH2A	-1.211211476	3.379984587	6.38E-07	1.89E-06
FAR2P4	4.129307055	-2.006712008	6.41E-07	1.90E-06
AGR2	4.000206632	4.660621717	6.44E-07	1.91E-06
LINC00668	3.572936606	-1.154792813	6.49E-07	1.92E-06
PLPP7	1.437546535	-0.43466152	6.50E-07	1.92E-06
MLIP	-1.068253116	3.964362582	6.52E-07	1.93E-06

AL109917.1	1.128214486	-1.196580526	6.55E-07	1.94E-06
AL109918.1	1.176186479	3.747194196	6.60E-07	1.95E-06
CASQ1	1.577576675	-2.18703761	6.60E-07	1.95E-06
LINC01238	1.043196331	-1.401368855	6.62E-07	1.96E-06
SOX4	1.212184699	4.964511345	6.62E-07	1.96E-06
AC024560.1	1.629606158	-2.324287101	6.68E-07	1.97E-06
TRIM73	1.333866356	-1.656929581	6.71E-07	1.98E-06
ZNF878	-1.136959504	-2.536546991	6.76E-07	2.00E-06
NKAIN2	2.309917682	-0.084135822	6.78E-07	2.00E-06
HOXB9	3.397261522	-1.083819652	6.81E-07	2.01E-06
MUC5AC	5.584828605	2.409153749	6.84E-07	2.02E-06
LINC00189	2.015147505	-0.839755325	6.92E-07	2.04E-06
AC069218.1	1.276093414	-2.515788486	6.93E-07	2.04E-06
KLHDC7B	1.407111724	1.14435954	6.94E-07	2.05E-06
AC123912.3	3.911621741	-2.754233487	6.95E-07	2.05E-06
AC011503.2	1.310680834	-0.273959933	6.96E-07	2.05E-06
CRYBB1	2.110313301	0.579283741	6.99E-07	2.06E-06
CCR10	1.295405233	-0.943393279	7.02E-07	2.07E-06
AC006042.1	1.284040782	-1.707540953	7.03E-07	2.07E-06
HSD3BP5	1.575231659	-3.013809592	7.04E-07	2.08E-06
FASN	1.003962727	9.374362016	7.06E-07	2.08E-06
PCDHB11	1.541368825	-0.021830472	7.11E-07	2.10E-06
AC239859.6	1.850921482	-2.606866092	7.12E-07	2.10E-06
AC023824.6	4.486554001	-2.633048885	7.17E-07	2.11E-06
LINC02506	3.32576685	0.477969829	7.20E-07	2.12E-06
SOX2	3.336153803	-0.435738921	7.23E-07	2.13E-06
AC233992.3	1.111308502	-2.68251612	7.24E-07	2.13E-06
AC069061.2	5.161615679	-2.992589254	7.26E-07	2.13E-06
RN7SKP23	1.174386674	-2.955176798	7.27E-07	2.14E-06
AC097512.1	3.612569609	-2.816679942	7.34E-07	2.16E-06
ZNF571-AS1	1.286251278	-2.053416351	7.40E-07	2.17E-06
FRZB	1.096866253	2.90310396	7.42E-07	2.18E-06
IGLV8-61	-2.018204435	2.086948467	7.46E-07	2.19E-06
AC073073.1	1.341566507	-2.011808337	7.52E-07	2.21E-06
GLYATL1P4	2.740568882	-0.666170589	7.53E-07	2.21E-06
TWIST1	2.040043246	-0.493668495	7.66E-07	2.25E-06
HS3ST4	5.82487253	0.611168294	7.71E-07	2.26E-06
AC020978.9	1.325444843	-2.388229681	7.72E-07	2.26E-06
DEFB132	2.626792786	0.622607653	7.75E-07	2.27E-06
UAP1L1	1.328341725	2.92996019	7.78E-07	2.28E-06
FOXG1	5.670367912	-2.238219611	7.86E-07	2.30E-06
NOV	1.113555659	1.261488105	7.95E-07	2.32E-06
SAGE1	5.146231424	-1.749253614	7.99E-07	2.34E-06
SLC5A4	1.301400435	-2.094576815	8.03E-07	2.35E-06
AL360270.3	1.11073755	-2.304272423	8.05E-07	2.35E-06
MLLT11	1.320836521	2.46298771	8.09E-07	2.36E-06
AC005387.1	1.1068511	-2.769541731	8.10E-07	2.37E-06
ANKRD26P1	5.237477178	-0.731122702	8.14E-07	2.38E-06
EYA2	-1.324534127	0.519347734	8.25E-07	2.41E-06
MED12L	-1.002547787	-2.02079934	8.27E-07	2.41E-06
LINC02384	2.162969578	1.846154368	8.29E-07	2.42E-06
HOPX	1.250450354	1.494387102	8.29E-07	2.42E-06
KIAA1024	1.507478247	-1.662207295	8.31E-07	2.42E-06
CCDC141	-1.232357348	-1.134847318	8.42E-07	2.45E-06
LINC00513	1.401186403	-0.066102757	8.50E-07	2.47E-06
AC006206.2	3.497212432	-1.969830042	8.54E-07	2.49E-06
AC112219.2	1.453665082	-2.874959728	8.56E-07	2.49E-06
MIR17HG	1.120922911	-0.330193825	8.68E-07	2.52E-06

SLC44A3-AS1	1.268498258	-0.651795878	8.70E-07	2.53E-06
AP001065.1	-1.044896311	1.0232027	8.72E-07	2.53E-06
CORO6	1.112774955	-0.643749361	8.74E-07	2.54E-06
CSAG4	4.146525325	-2.702772475	8.74E-07	2.54E-06
PRRT4	1.685977224	-0.685035364	8.77E-07	2.55E-06
EGFR-AS1	2.486580693	1.031826902	8.88E-07	2.58E-06
B3GNT3	1.862984944	3.718176136	8.90E-07	2.58E-06
VGF	2.077824127	0.016097424	8.91E-07	2.59E-06
ZNF534	2.572398522	-2.679811714	8.92E-07	2.59E-06
ATP10A	1.510188035	1.862934379	9.06E-07	2.63E-06
IQCH	1.47546524	1.113317432	9.08E-07	2.63E-06
KCNK17	-1.337572062	-0.096457326	9.13E-07	2.65E-06
LINC02154	3.262930169	-3.124727499	9.26E-07	2.68E-06
BNIP3P11	1.168385904	-2.111936856	9.31E-07	2.69E-06
SMC2-AS1	1.251243841	-2.984491207	9.32E-07	2.70E-06
C9orf152	1.824957117	1.386078146	9.42E-07	2.73E-06
SERTAD4	1.833114958	-0.184608821	9.49E-07	2.75E-06
PRAMEF15	4.091498045	-0.516404354	9.54E-07	2.76E-06
SIGLEC15	1.662759761	1.450633705	9.62E-07	2.78E-06
ANO1	-1.12341785	6.113439549	9.70E-07	2.80E-06
RAVER2	1.0642901	2.475093088	9.73E-07	2.81E-06
RNA5-8SP6	-2.816982449	-2.802460077	9.74E-07	2.81E-06
TUBB8P7	2.133794077	-2.952384928	9.74E-07	2.81E-06
AC118754.1	-1.110503606	-2.442618858	9.79E-07	2.83E-06
NKX2-5	4.018542752	-2.660169317	9.79E-07	2.83E-06
KCNF1	2.325289641	0.436805984	9.80E-07	2.83E-06
ZIM2-AS1	2.206724786	-2.349648677	9.87E-07	2.85E-06
AHNAK2	2.042098563	2.473920244	9.88E-07	2.85E-06
AGTR2	4.602129856	-3.01985153	9.89E-07	2.85E-06
DRICH1	1.149479917	-2.382871143	9.90E-07	2.85E-06
SPEG	1.791786804	1.011093677	9.92E-07	2.86E-06
SLC10A4	2.145302331	-3.022338945	9.94E-07	2.87E-06
NUDT11	2.400193404	-1.705793261	9.95E-07	2.87E-06
AC011840.1	4.823352296	-2.455262829	1.03E-06	2.96E-06
AC067930.5	1.456091131	-2.466044383	1.03E-06	2.97E-06
AC092159.2	1.980645048	-3.051385482	1.04E-06	2.98E-06
LINC01625	-1.534413035	-1.063805325	1.04E-06	2.99E-06
SLC39A5	-1.147120078	6.872342178	1.05E-06	3.02E-06
MYBPC2	2.37390229	-2.012077947	1.05E-06	3.03E-06
SPEF1	1.399309654	-2.208043893	1.06E-06	3.03E-06
AC027117.1	1.313341874	-1.153564416	1.06E-06	3.03E-06
CELA3B	3.779222222	-2.946068358	1.06E-06	3.05E-06
GRIA2	3.454481248	-2.13367162	1.06E-06	3.05E-06
ITGA11	1.074646216	2.028393524	1.07E-06	3.08E-06
PTGFR	2.062646758	2.608952746	1.07E-06	3.08E-06
PODXL2	2.051989083	3.084091892	1.08E-06	3.09E-06
AP001331.1	3.109977573	-2.412628677	1.08E-06	3.10E-06
FMN2	3.483298092	-1.385738961	1.09E-06	3.12E-06
GPX8	1.007051848	1.999811887	1.09E-06	3.13E-06
AL136528.1	2.379331462	-2.16180882	1.10E-06	3.15E-06
GUCA2A	2.811990329	-0.767673174	1.10E-06	3.15E-06
GDF10	2.763000807	-0.415930143	1.10E-06	3.16E-06
AL356966.1	1.373307691	-2.372310957	1.10E-06	3.16E-06
AP003171.1	4.873546123	-2.79821354	1.11E-06	3.18E-06
TMEM40	1.95360825	-1.007378046	1.12E-06	3.21E-06
AC019109.1	3.317992459	-2.655573833	1.12E-06	3.22E-06
IGHV2-70D	-1.917994289	-2.305314476	1.13E-06	3.23E-06
SALL2	1.471315515	2.04040636	1.14E-06	3.25E-06

AC026356.1	1.045254062	-1.761796465	1.15E-06	3.29E-06
AC068506.1	2.098244164	-1.079791692	1.16E-06	3.31E-06
AQP6	2.887027686	1.469394076	1.16E-06	3.32E-06
DAO	-1.040066158	5.824519913	1.17E-06	3.33E-06
LINC01754	1.945479388	-2.593116706	1.17E-06	3.35E-06
MKRN2OS	1.473873188	-1.215206537	1.17E-06	3.35E-06
SLC39A4	1.697449121	4.206123807	1.18E-06	3.36E-06
SH3RF3	1.255445368	2.505424034	1.18E-06	3.37E-06
ADRA1D	2.431016282	0.043235807	1.18E-06	3.38E-06
SCN5A	2.047773689	-2.270178058	1.19E-06	3.38E-06
GPR50	6.897817247	0.170283453	1.19E-06	3.40E-06
AC126603.1	2.136950346	-1.940371431	1.20E-06	3.42E-06
AC087239.1	1.14364538	-2.480562403	1.21E-06	3.44E-06
CYP2E1	-1.531529064	11.65959573	1.22E-06	3.46E-06
XKRX	1.607843484	-2.442010727	1.22E-06	3.48E-06
AL139246.2	1.40034281	-2.785900487	1.23E-06	3.51E-06
MAGEA4	6.422955888	0.315210567	1.24E-06	3.54E-06
SLC13A5	-1.123004486	8.165620849	1.27E-06	3.60E-06
SCUBE1	2.011628112	4.223269431	1.27E-06	3.61E-06
PDE6A	1.415125767	-2.79233487	1.28E-06	3.62E-06
QRFP	1.12884695	-2.839547079	1.28E-06	3.63E-06
UGT1A7	2.394105342	-0.528814488	1.28E-06	3.65E-06
GPR61	1.168372016	-2.621099939	1.29E-06	3.65E-06
ZNF90	1.429293559	-0.689329812	1.29E-06	3.66E-06
ERICH5	1.55869368	5.095841554	1.30E-06	3.67E-06
HERC2P3	1.622222395	0.789702766	1.30E-06	3.69E-06
AC009093.5	-1.363337925	-1.386580431	1.30E-06	3.70E-06
LRFN2	2.229969304	-2.382579972	1.32E-06	3.73E-06
DNAH11	1.579144838	1.277862655	1.32E-06	3.73E-06
CARM1P1	4.596088325	-2.819896553	1.32E-06	3.74E-06
SHISAL2A	1.708314449	-0.509177726	1.32E-06	3.74E-06
TECRL	4.610241842	-2.431906036	1.32E-06	3.74E-06
PHLDA3	1.253180495	4.139396307	1.37E-06	3.86E-06
USH1C	2.677811731	3.62748751	1.38E-06	3.90E-06
FZD10-DT	1.64504239	-1.952244363	1.39E-06	3.92E-06
AC020661.1	1.12550315	-2.809832324	1.39E-06	3.93E-06
MKRN9P	3.462371204	-2.954004823	1.40E-06	3.95E-06
UGT1A5	2.651604932	-1.441580395	1.40E-06	3.96E-06
CACNA1G	2.060952487	-2.764476026	1.41E-06	3.99E-06
SGSM1	1.293387307	0.411832365	1.42E-06	4.00E-06
DPEP1	2.29591567	1.028669105	1.42E-06	4.00E-06
QPCT	1.378551238	1.714421974	1.42E-06	4.01E-06
FETUB	-1.212596196	6.671178074	1.45E-06	4.08E-06
ADIG	2.303594633	-2.774303089	1.46E-06	4.10E-06
SLC8A2	1.90904098	-2.538044596	1.46E-06	4.11E-06
PROKR1	2.420708261	-2.409632888	1.46E-06	4.12E-06
LINC01793	4.280865737	-2.497604367	1.47E-06	4.12E-06
ATP4A	3.32621252	-2.321300876	1.50E-06	4.21E-06
CUBN	1.101267762	-0.454102995	1.50E-06	4.21E-06
HSPB3	4.322599661	-3.012100262	1.50E-06	4.22E-06
LINC01559	3.690768661	-0.677171468	1.51E-06	4.24E-06
GALNT13	1.913256652	-1.728694808	1.51E-06	4.25E-06
PRELID3A	1.214246634	-1.023250324	1.52E-06	4.26E-06
AL356317.1	-1.111558817	-2.414255623	1.52E-06	4.26E-06
GALR2	1.403936023	-2.433866755	1.52E-06	4.27E-06
TNNT3	2.172794064	-1.60750687	1.52E-06	4.27E-06
EFHC2	2.344111631	-2.216526179	1.55E-06	4.33E-06
AL442125.1	1.070845657	-2.08198877	1.56E-06	4.36E-06

AC006205.2	2.343554707	1.895609744	1.56E-06	4.38E-06
RBM20	1.536089322	-0.293452875	1.58E-06	4.42E-06
PKD1L1	1.166829515	-0.93583952	1.59E-06	4.44E-06
DPYSL4	1.802528815	-0.365468878	1.59E-06	4.45E-06
CD177	2.462955714	-0.416628657	1.60E-06	4.47E-06
KCNIP3	1.545850839	1.707129368	1.61E-06	4.50E-06
SOAT2	2.015139064	3.136038793	1.62E-06	4.53E-06
AC144833.1	4.4826163	-2.022013506	1.62E-06	4.54E-06
SALL3	5.516872492	-2.209730008	1.63E-06	4.54E-06
IL31RA	2.344197862	-2.411760959	1.63E-06	4.55E-06
PPARGC1A	-1.066247016	5.91155415	1.63E-06	4.56E-06
LINC01348	-1.15121386	3.930401193	1.64E-06	4.57E-06
MYRFL	1.495954914	-1.861125364	1.64E-06	4.59E-06
LINC02127	1.42815233	-2.44545032	1.65E-06	4.59E-06
OFCC1	3.886198097	-2.895156816	1.66E-06	4.64E-06
MRC1	-1.116541426	4.956009061	1.67E-06	4.65E-06
AC027279.2	1.506594192	-2.82298045	1.68E-06	4.67E-06
SMIM31	3.552539277	0.062385563	1.68E-06	4.69E-06
GJA3	2.148033095	-2.132400121	1.69E-06	4.70E-06
ADGRG1	1.058969917	4.508987932	1.69E-06	4.71E-06
AMN	-1.24354927	5.26438624	1.72E-06	4.79E-06
HTR4	1.871595043	-1.427282725	1.72E-06	4.79E-06
NDST1-AS1	1.689233185	-1.131868291	1.75E-06	4.85E-06
MUC12	2.463340699	0.716214737	1.75E-06	4.85E-06
AC006273.1	1.799661593	-2.262053441	1.75E-06	4.86E-06
AC060780.1	1.001803833	-0.354305377	1.75E-06	4.86E-06
ZNF239	1.366044367	0.45923595	1.77E-06	4.92E-06
LINC01018	-1.458551201	5.611792316	1.80E-06	5.00E-06
PDCD6IPP2	1.458183817	-1.481026229	1.81E-06	5.01E-06
ZNF426-DT	1.231573554	-1.677473212	1.81E-06	5.01E-06
AC092045.1	1.247420301	-2.843531628	1.83E-06	5.06E-06
JPH1	1.809815816	0.573546997	1.83E-06	5.06E-06
SEMA3B-AS1	1.252421924	-1.142952186	1.84E-06	5.08E-06
UBE2SP1	1.204638231	-1.954863938	1.86E-06	5.15E-06
MGAT5B	1.797900914	-1.400163565	1.88E-06	5.19E-06
AC243965.2	1.218404338	-2.496579551	1.89E-06	5.22E-06
NLRP4	3.859529625	-2.383275151	1.90E-06	5.24E-06
KRT81	2.15247341	-0.759002002	1.90E-06	5.25E-06
C1orf105	1.126160207	-0.91188269	1.91E-06	5.26E-06
SLC7A14	2.474012533	-0.863979885	1.93E-06	5.31E-06
CR589904.1	4.133523001	-2.046775209	1.93E-06	5.32E-06
MT1JP	-2.010545986	-1.556646159	1.94E-06	5.35E-06
LRRN4CL	2.192192106	-1.401860792	1.97E-06	5.42E-06
AC009108.3	1.17440923	-2.319677329	1.97E-06	5.44E-06
LINC01361	3.533574358	-2.283764634	1.98E-06	5.44E-06
MMP17	1.806628686	0.828901909	1.98E-06	5.46E-06
AC010616.1	2.309226245	-2.153360444	1.99E-06	5.49E-06
ITLN2	1.822904302	0.011494026	1.99E-06	5.49E-06
ZBTB32	1.086070015	-1.596173952	2.03E-06	5.57E-06
BCL2L15	1.520659763	-1.021060034	2.04E-06	5.62E-06
AC119150.1	1.939029622	-1.751863154	2.04E-06	5.62E-06
CNN2P1	1.139717904	-2.119066386	2.06E-06	5.65E-06
AC005329.1	1.428138929	-0.880056095	2.07E-06	5.67E-06
HNRNPA1P8	1.137242193	-2.947022509	2.08E-06	5.72E-06
KEL	2.406933398	1.61804784	2.11E-06	5.78E-06
ZNF610	1.344438206	-0.378122386	2.11E-06	5.78E-06
LINC01819	3.571406719	-1.551160519	2.13E-06	5.83E-06
LINC01843	1.154852086	1.709571079	2.18E-06	5.97E-06

CACNA1B	3.010393609	-2.738705123	2.19E-06	6.00E-06
SLCO2A1	1.200647474	3.48873075	2.20E-06	6.02E-06
SLC12A1	2.983869192	1.569644218	2.21E-06	6.05E-06
PCDHB10	1.232017769	-0.728505693	2.22E-06	6.07E-06
FSIP2	1.593151219	-0.839139413	2.25E-06	6.15E-06
SLITRK4	1.793821765	-0.971670156	2.25E-06	6.16E-06
FAM135B	1.836154943	-0.133841095	2.26E-06	6.19E-06
GLULP4	1.989791537	-2.496208692	2.27E-06	6.20E-06
GUCY2EP	1.924649508	-1.649639632	2.31E-06	6.32E-06
TMEM270	1.920848259	-1.897297105	2.32E-06	6.35E-06
AL162741.1	1.102831914	-2.5586051	2.33E-06	6.36E-06
AC099684.1	-1.080291674	-0.186524805	2.36E-06	6.43E-06
AGAP1-IT1	-1.166600037	-0.221251597	2.37E-06	6.45E-06
AC010547.2	-2.05218513	-1.044203871	2.37E-06	6.46E-06
DUSP15	1.558451364	-0.301990064	2.37E-06	6.46E-06
AC139769.1	1.73808004	-1.125878629	2.37E-06	6.46E-06
AL390719.1	1.287492978	0.894467951	2.37E-06	6.46E-06
AC103770.1	4.041241861	-2.3040976	2.38E-06	6.48E-06
COLGALT2	1.565252191	0.013159296	2.39E-06	6.50E-06
ARHGAP40	2.714365199	-1.43182879	2.39E-06	6.51E-06
COL4A5	1.738316443	2.822616056	2.39E-06	6.51E-06
TAC4	1.203522893	-2.966285259	2.39E-06	6.51E-06
CHST4	-1.914178422	1.989940666	2.41E-06	6.56E-06
NBPF13P	-1.131403315	1.962002777	2.42E-06	6.59E-06
NTN5	1.097853205	-1.748407446	2.43E-06	6.62E-06
HRG	-1.170621359	11.21046171	2.44E-06	6.63E-06
AC011247.1	1.282828052	-2.547149436	2.46E-06	6.68E-06
HRK	3.027644932	-2.251370592	2.46E-06	6.70E-06
CRYAB	1.700478008	4.269452715	2.48E-06	6.75E-06
CEACAM7	6.160210642	2.916255273	2.50E-06	6.80E-06
PRELP	-1.146373483	4.326852444	2.51E-06	6.81E-06
GLDCP1	-1.011741001	-1.166713991	2.51E-06	6.82E-06
NCR1	-1.103464575	-2.149957937	2.51E-06	6.82E-06
HEPACAM2	2.949270419	-2.390970008	2.52E-06	6.82E-06
FGF22	1.536163287	-2.734567221	2.53E-06	6.85E-06
AC022150.4	1.557381605	-1.374208945	2.53E-06	6.86E-06
QRFPR	2.137811321	-2.753502566	2.55E-06	6.90E-06
VGLL2	5.357835597	-2.611768961	2.55E-06	6.92E-06
HERC2P4	1.732472408	-2.442023246	2.56E-06	6.94E-06
ARG2	1.506560114	2.179743097	2.57E-06	6.95E-06
SAMD11	-1.155790626	1.09695777	2.58E-06	6.99E-06
PMEL	1.978856109	4.682925993	2.58E-06	7.00E-06
USP2-AS1	1.26293225	0.586930402	2.62E-06	7.09E-06
GRIN3B	1.599216896	-2.265100511	2.63E-06	7.11E-06
SNHG28	1.2399375	-2.578045885	2.64E-06	7.13E-06
AP000757.2	2.934297524	-1.769904317	2.64E-06	7.14E-06
AC114488.1	1.875941357	-2.448804317	2.64E-06	7.14E-06
AC087273.1	-1.011773384	-2.347377443	2.65E-06	7.16E-06
SLC38A5	1.403984578	0.921482259	2.67E-06	7.22E-06
RSPO3	-1.690549687	1.318106787	2.70E-06	7.29E-06
LRP12	1.051715697	1.977749516	2.74E-06	7.40E-06
SRSF12	1.138826779	-0.026865574	2.74E-06	7.40E-06
AC068756.1	3.089624688	-2.852561119	2.75E-06	7.41E-06
SLCO4C1	-1.498407026	2.268862175	2.76E-06	7.44E-06
PLEK2	-1.189251274	3.403543954	2.77E-06	7.46E-06
AC007114.2	1.438992042	-2.623775152	2.79E-06	7.52E-06
LINC02334	2.497419245	-2.396554841	2.81E-06	7.56E-06
GTF2IRD1P1	1.182228977	-2.578308695	2.81E-06	7.57E-06

SNORD99	1.090216933	-2.57455138	2.84E-06	7.66E-06
ELOVL4	1.573783013	-1.074525987	2.87E-06	7.73E-06
AP000851.2	-1.813426896	-0.64161535	2.90E-06	7.82E-06
AC027117.2	-1.048712373	2.172825364	2.90E-06	7.82E-06
KAAG1	1.725454971	-0.683562499	2.91E-06	7.84E-06
SAMD12	-1.158227014	1.395880724	2.91E-06	7.84E-06
HCG17	1.048503727	-2.159818028	2.93E-06	7.89E-06
VNN3	-1.041622668	4.032050788	2.94E-06	7.92E-06
BMP8B	1.450207639	1.528936143	2.95E-06	7.93E-06
CD70	1.656653334	-1.326042981	2.96E-06	7.95E-06
FAM241B	1.036311765	1.906801447	2.97E-06	7.98E-06
NANOGP1	1.974708301	-2.791852888	3.01E-06	8.09E-06
NHLH2	4.155633011	-3.03244945	3.02E-06	8.10E-06
TMED3	1.202104226	4.940826601	3.02E-06	8.10E-06
PICSAR	3.86898601	-2.952236884	3.04E-06	8.16E-06
SPDEF	2.777661967	1.080187029	3.04E-06	8.17E-06
SLC16A11	1.47422053	4.487024333	3.05E-06	8.18E-06
ACSBG1	1.825857323	-1.471604542	3.07E-06	8.22E-06
NOTUM	2.288712017	6.585480171	3.08E-06	8.27E-06
AC136475.10	1.083164213	-2.384153315	3.10E-06	8.31E-06
AC244517.1	1.913446764	-2.864591176	3.10E-06	8.32E-06
IGHD	-1.502532322	1.193794033	3.10E-06	8.32E-06
KBTBD12	2.914649214	-2.160039741	3.14E-06	8.40E-06
HID1	1.051642079	3.701741342	3.15E-06	8.44E-06
PPP1R1A	-1.142656808	6.266344441	3.17E-06	8.50E-06
AC092042.3	-1.229410509	-2.703287294	3.18E-06	8.51E-06
AL356652.1	1.154230015	-2.763222001	3.18E-06	8.52E-06
RIPPLY2	3.903837203	-2.685724617	3.20E-06	8.56E-06
LILRA4	-1.014596193	-1.724645413	3.21E-06	8.58E-06
FOLR1	2.150565386	0.305416726	3.21E-06	8.59E-06
HTR2C	3.920254367	-2.598716436	3.21E-06	8.60E-06
AC007966.1	1.716716589	-2.921082949	3.26E-06	8.72E-06
SLC46A2	2.35096759	-1.828025707	3.26E-06	8.73E-06
AC073610.1	1.145643266	-2.07037222	3.27E-06	8.73E-06
RNF43	1.115176809	4.834605082	3.30E-06	8.81E-06
LINC00322	2.823192331	-3.339134068	3.30E-06	8.82E-06
APELA	4.07666185	-2.894206312	3.31E-06	8.83E-06
IGHV3-72	-1.591828911	-0.99065799	3.32E-06	8.87E-06
FKBP10	1.255110094	4.73010911	3.34E-06	8.90E-06
TAS2R5	1.193581599	-2.67877624	3.34E-06	8.90E-06
GPA33	-1.380951013	-2.991561468	3.39E-06	9.04E-06
FMO2	-1.016354616	1.328667893	3.40E-06	9.05E-06
SNORD17	-1.030908882	-2.039982893	3.43E-06	9.14E-06
CDH7	3.438668447	-2.447324175	3.44E-06	9.16E-06
TNFSF9	1.385730418	-0.389352985	3.48E-06	9.27E-06
CCDC144NL-AS	1.509852215	0.471733338	3.49E-06	9.28E-06
PKIB	1.484353005	2.821694809	3.52E-06	9.36E-06
LINC01518	3.983536363	-3.047809701	3.52E-06	9.36E-06
AC015961.2	1.246605002	-2.975530902	3.52E-06	9.36E-06
TAT	-1.288080121	9.980467138	3.53E-06	9.39E-06
DRAXIN	1.159825521	-2.029602495	3.54E-06	9.41E-06
CCDC74B	1.760715965	-1.964730303	3.58E-06	9.51E-06
AL365356.4	1.470238228	-2.539895071	3.58E-06	9.52E-06
LRCOL1	-1.382689247	2.812438341	3.60E-06	9.56E-06
CNTNAP3B	1.570452405	0.826310426	3.61E-06	9.58E-06
ARHGAP28	1.286063449	0.346890639	3.62E-06	9.61E-06
LRRC38	4.579480867	-2.403591019	3.62E-06	9.62E-06
AC018467.1	-1.038312586	0.889795333	3.63E-06	9.62E-06

ZNF468	1.02982471	2.788718911	3.63E-06	9.62E-06
FCGBP	1.442756603	3.466883952	3.66E-06	9.69E-06
CNGB1	2.589214528	-0.962069647	3.68E-06	9.75E-06
SMILR	1.892106444	-2.344063879	3.71E-06	9.83E-06
ITIH5	2.063074022	4.332568684	3.72E-06	9.86E-06
KIAA1324	1.662653777	1.551869453	3.73E-06	9.88E-06
JPH3	2.234068623	-1.192087831	3.75E-06	9.92E-06
CST4	4.369825548	-2.425852912	3.79E-06	1.00E-05
ZCCHC12	2.306592187	-1.703351464	3.79E-06	1.00E-05
AC027688.1	1.331808055	-1.656870118	3.83E-06	1.01E-05
AATK	1.303669997	1.499500464	3.83E-06	1.01E-05
INSYN1	1.526024038	-0.169678156	3.83E-06	1.01E-05
Z82249.1	5.932323849	-2.176011605	3.85E-06	1.02E-05
UBXN10-AS1	1.492163438	-1.792386749	3.86E-06	1.02E-05
DLGAP1-AS5	6.003988315	-0.039101111	3.91E-06	1.03E-05
AC108676.1	1.453135105	-1.668174443	3.91E-06	1.03E-05
KRT85	2.636956479	-0.865819988	3.97E-06	1.05E-05
AC093627.7	1.455073148	-2.7623054	4.01E-06	1.06E-05
NLRP14	-1.184314115	-2.234231288	4.04E-06	1.07E-05
BIRC7	2.333507827	-0.58990958	4.05E-06	1.07E-05
AC079062.1	3.427137991	1.004688717	4.07E-06	1.07E-05
TTC9B	1.112807247	-2.708340416	4.08E-06	1.08E-05
KCNG1	2.037342483	-0.599217397	4.09E-06	1.08E-05
FGF19	3.405279639	3.886884474	4.12E-06	1.09E-05
AL626787.1	-1.102003389	-2.763065014	4.12E-06	1.09E-05
AC025259.3	-1.014833746	-2.370558241	4.13E-06	1.09E-05
AL161645.1	-1.281551357	-0.567601121	4.17E-06	1.10E-05
C5orf38	3.608780986	-2.837250823	4.20E-06	1.11E-05
FZD2	1.197456116	0.12310036	4.21E-06	1.11E-05
SGIP1	1.200367628	0.468965813	4.25E-06	1.12E-05
GCGR	-1.415398156	5.389297804	4.26E-06	1.12E-05
LINC02363	1.022148765	-0.447613097	4.29E-06	1.13E-05
PTPRD-AS1	-1.114753036	-1.992100794	4.32E-06	1.14E-05
SYT14	4.293840469	-1.692547542	4.35E-06	1.14E-05
FLJ16779	3.441874645	-2.644957583	4.36E-06	1.14E-05
EHF	1.468384012	2.730568299	4.36E-06	1.15E-05
LYPD2	-1.788503584	-1.941960373	4.38E-06	1.15E-05
SPATA17	1.725810036	-1.098695136	4.39E-06	1.15E-05
CXCL5	2.886574504	2.437910108	4.40E-06	1.15E-05
MPP2	1.384135775	-0.570112849	4.42E-06	1.16E-05
AC007608.3	1.389710055	-1.289025451	4.42E-06	1.16E-05
HNRNPCL3	4.679571439	-3.080301252	4.48E-06	1.18E-05
AC091153.3	1.109159389	-2.239672878	4.52E-06	1.19E-05
AC020656.2	1.486991239	0.657232606	4.55E-06	1.19E-05
SPATA18	-1.122454443	2.261870997	4.56E-06	1.19E-05
UBE2U	3.365349816	-1.999378713	4.58E-06	1.20E-05
DERL3	1.24608108	3.092302993	4.60E-06	1.21E-05
MGAT4C	2.359919293	0.842916443	4.61E-06	1.21E-05
GOLGA7B	1.775116994	1.863668952	4.62E-06	1.21E-05
AC111149.2	1.930657775	-0.801487548	4.67E-06	1.22E-05
AL445309.1	1.061701074	-2.445680672	4.71E-06	1.23E-05
ZNF365	1.659778895	-2.557838232	4.71E-06	1.23E-05
IGLON5	-1.170177981	-0.148004018	4.80E-06	1.25E-05
SOX9	1.243003973	4.913553083	4.82E-06	1.26E-05
ZNF391	1.415211344	-0.122397854	4.83E-06	1.26E-05
AL139385.1	-1.256858617	0.674629236	4.86E-06	1.27E-05
LINC00514	1.023394637	-0.35300631	4.87E-06	1.27E-05
PTGDS	2.458128102	7.355264256	4.93E-06	1.29E-05

PARM1	1.117989516	2.666364254	4.95E-06	1.29E-05
CYP3A4	-1.665309358	10.44538623	5.00E-06	1.30E-05
MXRA5	-1.038280345	3.114419334	5.03E-06	1.31E-05
VLDLR	1.56857166	2.848162204	5.03E-06	1.31E-05
ELOVL7	1.696215267	3.000398126	5.08E-06	1.32E-05
SULF1	1.371737259	3.906479548	5.11E-06	1.33E-05
C2CD6	1.34743752	-2.257943398	5.11E-06	1.33E-05
ALOX15	1.519628903	-1.672567366	5.16E-06	1.34E-05
SLC26A3	2.24406273	1.591436468	5.16E-06	1.34E-05
AC016924.1	1.08330659	-0.89554603	5.17E-06	1.35E-05
AC148476.1	2.389352123	-1.957055765	5.20E-06	1.35E-05
AC131532.1	2.904521899	-2.866228366	5.21E-06	1.35E-05
GLIS2	1.018912943	3.355149223	5.22E-06	1.36E-05
AC025580.1	2.283254491	-2.898741279	5.24E-06	1.36E-05
UGT3A2	1.94961884	1.025365594	5.27E-06	1.37E-05
SMIM24	-1.581755116	4.024604368	5.31E-06	1.38E-05
RHOV	2.323871637	0.603516045	5.31E-06	1.38E-05
PRR5L	1.192743287	2.526094017	5.32E-06	1.38E-05
AC073321.1	-1.457207841	-2.652864496	5.36E-06	1.39E-05
SIK1	-1.499472979	2.259201946	5.38E-06	1.40E-05
NEURL3	1.401083417	2.312105158	5.41E-06	1.40E-05
AC010624.1	1.10512907	-2.766117397	5.44E-06	1.41E-05
CRH	3.911733683	-3.26498119	5.49E-06	1.42E-05
AC008033.3	2.140230219	-1.489898972	5.50E-06	1.42E-05
STXBP5-AS1	1.119544454	-1.253757499	5.55E-06	1.44E-05
LEP	3.500000118	-1.765101386	5.56E-06	1.44E-05
FAAHP1	1.146528387	-2.005648394	5.56E-06	1.44E-05
AL160408.1	2.264555417	-2.414645355	5.57E-06	1.44E-05
DEFA5	6.209729061	-2.189362471	5.62E-06	1.45E-05
EGLN3	1.41960781	3.112621532	5.67E-06	1.47E-05
FLJ31356	1.647957012	-2.773460098	5.67E-06	1.47E-05
CLLU1	3.976434161	-2.4987313	5.68E-06	1.47E-05
ALDH3B2	3.860564847	-0.071924596	5.69E-06	1.47E-05
MRPL23-AS1	2.599543	-1.287669372	5.69E-06	1.47E-05
PLCB4	1.129239603	0.110662388	5.70E-06	1.47E-05
MEOX1	1.525842015	-0.821650666	5.79E-06	1.49E-05
CALCB	1.265929128	-2.178750432	5.81E-06	1.50E-05
CPS1	-1.16101886	10.66375108	5.84E-06	1.51E-05
FMR1-IT1	1.151778713	-2.935232956	5.84E-06	1.51E-05
CLDND2	1.02635826	1.012087172	5.85E-06	1.51E-05
GJB6	3.058025622	-0.611097023	5.96E-06	1.54E-05
AC006213.6	1.026253	-2.466853335	5.98E-06	1.54E-05
THRSP	-1.54381959	6.014570528	5.99E-06	1.54E-05
CYP7A1	1.854384577	6.200435019	6.10E-06	1.57E-05
SNAI2	1.084344183	3.457318361	6.12E-06	1.57E-05
AC099066.2	1.573402861	-1.953726991	6.13E-06	1.58E-05
INSL6	3.589912152	-2.294502825	6.16E-06	1.58E-05
HTRA3	1.325653583	2.777611007	6.17E-06	1.58E-05
TMEM59L	1.61570963	-1.710279437	6.17E-06	1.59E-05
LINC01029	4.632718738	-3.286453754	6.18E-06	1.59E-05
SERHL2	1.291082351	0.748403497	6.20E-06	1.59E-05
IL21-AS1	2.736317504	-2.566661278	6.25E-06	1.61E-05
GLIS2-AS1	1.282411571	-0.84116021	6.31E-06	1.62E-05
ANKRD30A	2.709859112	-3.172950121	6.31E-06	1.62E-05
U62317.1	1.033161994	-1.137468352	6.33E-06	1.62E-05
PLXNB3	1.62698225	1.342130323	6.42E-06	1.64E-05
ZNF285	1.067329898	0.193617829	6.44E-06	1.65E-05
DCDC2	1.645147223	4.831039419	6.44E-06	1.65E-05

TRIM63	3.424293116	-0.067443053	6.44E-06	1.65E-05
LINC01124	1.252795588	2.69403849	6.47E-06	1.66E-05
KIAA1257	1.338271125	-1.854028908	6.55E-06	1.68E-05
SNAP25	1.659756862	2.100064104	6.57E-06	1.68E-05
PDPK2P	1.052680298	-2.121976819	6.61E-06	1.69E-05
POU6F2	-1.701284282	-0.981653573	6.63E-06	1.69E-05
HK2	1.532947999	3.256547639	6.71E-06	1.72E-05
GMCL2	2.905940369	-2.646029303	6.72E-06	1.72E-05
KRTAP5-7	2.080538989	-2.835773571	6.72E-06	1.72E-05
AC106897.1	1.071576389	-2.538706157	6.78E-06	1.73E-05
MAGEC3	3.824958107	-2.405060996	6.79E-06	1.73E-05
CHRM3	1.272596505	1.769309936	6.83E-06	1.74E-05
TNR	-1.64118527	-0.138797243	6.87E-06	1.75E-05
LINC00632	3.074936883	-2.396911538	6.87E-06	1.75E-05
LHX8	5.412313219	-2.240392253	6.93E-06	1.77E-05
KC877982.1	6.01012703	-1.284682054	6.96E-06	1.78E-05
PTCHD3P2	1.962080504	-2.500891148	7.04E-06	1.79E-05
RAMP1	1.091201906	6.517708601	7.07E-06	1.80E-05
AC009414.2	1.247604947	0.007822824	7.10E-06	1.81E-05
CRYBA4	3.268407304	-1.582873499	7.10E-06	1.81E-05
SEMA3A	1.474332733	0.185151742	7.25E-06	1.84E-05
DOK7	1.382016595	1.441629777	7.26E-06	1.85E-05
AC005537.1	2.763642089	-2.592985384	7.32E-06	1.86E-05
MSLN	2.307249631	0.643624892	7.33E-06	1.86E-05
AL354872.2	-1.008517528	2.355029251	7.36E-06	1.87E-05
ASIC5	1.493698292	-2.954019833	7.42E-06	1.88E-05
LINC02228	3.149960631	-2.73411491	7.42E-06	1.88E-05
PRR15L	1.68562828	2.702432574	7.44E-06	1.89E-05
PTHLH	2.012256795	1.469985982	7.50E-06	1.90E-05
IL12A	1.133188576	-2.042114203	7.51E-06	1.91E-05
PPIAP21	1.089814493	-2.649433681	7.52E-06	1.91E-05
AL355303.1	1.08097664	-2.366567384	7.55E-06	1.91E-05
BOC	1.113557543	0.819285216	7.56E-06	1.92E-05
CLLU1OS	3.807186489	-2.037244237	7.63E-06	1.93E-05
PLP1	-1.869285678	-2.320007888	7.65E-06	1.94E-05
AC007731.2	1.114849743	-2.173240842	7.65E-06	1.94E-05
MST1R	1.573637645	1.391885094	7.73E-06	1.96E-05
AC022034.1	1.916450808	-1.179935187	7.75E-06	1.96E-05
G6PC	-1.01058215	9.133929274	7.78E-06	1.97E-05
ULBP2	1.326464139	-0.425264664	7.81E-06	1.98E-05
LMTK3	1.661599747	1.058262354	7.82E-06	1.98E-05
LGALS17A	2.22878132	-1.984173076	7.85E-06	1.99E-05
ACTL6B	1.356320605	-2.442658391	7.92E-06	2.00E-05
SCG3	2.26732976	-1.707699817	7.99E-06	2.02E-05
SDK1	1.558838638	1.869850854	8.04E-06	2.03E-05
PYDC1	3.516438215	-2.670248258	8.08E-06	2.04E-05
CYP2A6	-1.474501446	9.664367557	8.13E-06	2.05E-05
HHIP-AS1	-1.39322594	-0.962914262	8.13E-06	2.05E-05
AP003119.2	1.111856919	-0.771144695	8.14E-06	2.05E-05
CAPN13	2.676638556	0.387006142	8.14E-06	2.05E-05
NDRG4	1.677794063	2.13148214	8.20E-06	2.07E-05
FGF4	5.218898072	-1.501261554	8.27E-06	2.08E-05
NBL1	1.142641563	2.601863038	8.27E-06	2.08E-05
LINC01508	3.881836639	-1.883829359	8.31E-06	2.09E-05
CNTD2	1.447228775	1.794924032	8.43E-06	2.12E-05
LRRC52	2.860493743	-2.448848576	8.55E-06	2.15E-05
AC090578.1	3.61443963	-2.599631271	8.60E-06	2.16E-05
RFX3-AS1	1.026240811	-1.7374283	8.69E-06	2.18E-05

LINC00200	4.059433037	-2.260792494	8.74E-06	2.19E-05
C8orf37-AS1	1.29895053	-2.655162588	8.75E-06	2.20E-05
CATSPERB	1.658469561	-0.688142838	8.76E-06	2.20E-05
PRSS21	2.556012214	-0.634263948	8.78E-06	2.20E-05
NPTX2	2.3594083	3.432006555	8.83E-06	2.21E-05
OVOL1-AS1	2.674363286	-1.177810582	8.94E-06	2.24E-05
ADIPOQ	4.392036253	-2.290012896	8.95E-06	2.24E-05
FSCN2	1.390583926	-2.510411512	9.00E-06	2.26E-05
LINC01831	-1.434175369	1.13424124	9.10E-06	2.28E-05
BX537318.2	3.045442282	-3.035256833	9.11E-06	2.28E-05
AC016722.2	1.001896261	-2.506160029	9.11E-06	2.28E-05
AC104046.1	1.107953868	-2.684187625	9.15E-06	2.29E-05
AC090971.3	1.066944276	-2.580762653	9.16E-06	2.29E-05
CYP1B1	1.461264183	4.896658679	9.29E-06	2.32E-05
MAP3K15	1.551159014	-1.442340026	9.31E-06	2.33E-05
LGALS12	2.379629316	-1.260967493	9.43E-06	2.36E-05
AL121845.4	1.26084072	-0.711758137	9.44E-06	2.36E-05
AC026403.1	1.11686261	1.502419072	9.57E-06	2.39E-05
SUN3	2.409869593	-2.872727262	9.67E-06	2.41E-05
GPR63	1.370152108	-2.484866938	9.71E-06	2.42E-05
PCDHGA4	1.445988948	0.188589133	9.92E-06	2.47E-05
AC022784.1	1.201180765	-0.468149576	1.03E-05	2.56E-05
CALML3-AS1	1.440322259	-1.001829169	1.03E-05	2.56E-05
ST6GALNAC2	1.039769336	2.002602427	1.03E-05	2.56E-05
HSPB8	1.423321777	4.033215648	1.03E-05	2.57E-05
LRRC3B	1.502089648	-2.671048148	1.04E-05	2.58E-05
AC026523.2	3.322499421	-2.643006375	1.05E-05	2.60E-05
AP001527.2	1.022411626	-2.833061236	1.06E-05	2.63E-05
LMOD1	1.238424665	3.502420563	1.06E-05	2.63E-05
ANKRD34B	2.833083916	-2.638616976	1.06E-05	2.63E-05
RPL12P14	1.450922139	0.113866327	1.06E-05	2.64E-05
AC140479.2	1.690544063	-2.880146752	1.07E-05	2.64E-05
AC016738.1	1.180889359	-2.134932446	1.07E-05	2.65E-05
AL357146.1	2.79299896	-2.809861281	1.07E-05	2.66E-05
SOX14	5.412017037	-2.419377499	1.09E-05	2.70E-05
PAK3	2.190300094	0.555561948	1.10E-05	2.72E-05
GULP1	1.796889021	1.650995884	1.10E-05	2.73E-05
PDE11A	-1.030580877	2.738741418	1.11E-05	2.75E-05
HOXD11	3.563925728	-3.15888702	1.12E-05	2.76E-05
AC132938.1	1.469955288	-2.53309862	1.12E-05	2.78E-05
MT1L	-1.366602438	3.555590321	1.13E-05	2.80E-05
AC068533.2	-1.056833489	-1.416308651	1.14E-05	2.83E-05
SLC35F1	1.429090012	-1.642023894	1.16E-05	2.86E-05
SLC5A10	1.524620324	0.030127025	1.17E-05	2.90E-05
AL713998.1	4.819500682	-2.253105832	1.18E-05	2.92E-05
VN1R81P	1.511332538	-3.037186997	1.18E-05	2.92E-05
TRABD2A	1.44805686	2.374510011	1.19E-05	2.94E-05
TMEM54	1.045859871	3.580287945	1.20E-05	2.95E-05
AL365295.1	1.153491995	-2.067612684	1.20E-05	2.95E-05
LINC01632	3.476414021	-2.7376576	1.20E-05	2.96E-05
CFAP65	1.306025453	-2.115959953	1.20E-05	2.97E-05
CCND2P1	-1.144662407	2.883135615	1.20E-05	2.97E-05
RDH10-AS1	1.068640537	-1.983607535	1.21E-05	2.97E-05
LOXL1	1.364722347	2.583357092	1.21E-05	2.99E-05
GRM8	-1.477578821	0.333326228	1.23E-05	3.02E-05
CRTAC1	2.793873851	1.720247228	1.24E-05	3.05E-05
LINC02212	4.259725645	-2.131490431	1.25E-05	3.06E-05
AC136621.1	3.796074582	-0.037604775	1.25E-05	3.07E-05

PCDHB6	1.357902405	-1.05193514	1.26E-05	3.09E-05
AC025164.1	1.053009002	-1.442022898	1.27E-05	3.11E-05
AL356056.2	1.177755723	-1.699297288	1.27E-05	3.12E-05
CRHR1	4.513305346	-2.771464691	1.27E-05	3.13E-05
CHRM1	3.292190155	-2.021818716	1.28E-05	3.13E-05
TUBA4B	1.409610645	-0.373499344	1.28E-05	3.13E-05
DMKN	2.031250084	3.206491484	1.28E-05	3.14E-05
KIF5C	1.399091913	0.793777865	1.29E-05	3.16E-05
XG	-1.24635497	-0.151538788	1.29E-05	3.16E-05
ITGAD	-1.076479072	0.722373286	1.29E-05	3.17E-05
PPIAP39	1.65254933	-2.658391771	1.31E-05	3.21E-05
MYO7B	1.339406669	2.555652382	1.31E-05	3.22E-05
AL627309.7	1.185868245	-1.807415236	1.32E-05	3.24E-05
AC009646.2	5.782525052	-1.805972012	1.33E-05	3.25E-05
SULT2B1	1.97294961	0.299981876	1.35E-05	3.29E-05
AL022341.1	1.015999842	-2.836655659	1.35E-05	3.30E-05
LINC02562	2.735609242	-2.417872594	1.35E-05	3.31E-05
SLC16A14	1.649851021	3.250000431	1.36E-05	3.31E-05
SRGAP2D	1.383769462	-2.535667004	1.36E-05	3.31E-05
DDR1	1.173896244	5.119316499	1.36E-05	3.32E-05
KSR2	1.664738629	-1.571589469	1.36E-05	3.33E-05
PSAPL1	3.047167685	-2.086052327	1.37E-05	3.33E-05
SLC2A1	1.109084305	3.255224336	1.37E-05	3.35E-05
EPHA5	3.873199891	-2.445488127	1.38E-05	3.36E-05
AC117402.1	1.402135569	-2.137294132	1.39E-05	3.38E-05
C16orf74	1.096496616	-0.31132377	1.39E-05	3.38E-05
TNFSF11	-1.142742438	0.444313947	1.39E-05	3.39E-05
TMEM191B	1.387131213	-2.41748687	1.40E-05	3.41E-05
PLD5	2.46223022	-0.117484464	1.40E-05	3.41E-05
FABP1	-1.050712404	10.3282613	1.42E-05	3.45E-05
SCN1A	2.877579266	-1.666298673	1.43E-05	3.47E-05
CCDC36	1.28902678	-2.37430643	1.44E-05	3.49E-05
BMP8A	1.014143769	-2.1485978	1.46E-05	3.55E-05
NEGR1	-1.102635427	-1.696943499	1.47E-05	3.58E-05
ANGPTL7	4.044229567	0.201274855	1.48E-05	3.59E-05
CPA4	1.837152625	-1.510476621	1.49E-05	3.62E-05
AC009093.1	1.073139089	-2.799081759	1.51E-05	3.65E-05
TMEM130	2.194597482	0.513365626	1.51E-05	3.66E-05
GAL	2.934001794	-1.054724857	1.51E-05	3.67E-05
ADGRG2	1.604707043	2.440169834	1.52E-05	3.69E-05
CTSE	2.733536768	1.631560714	1.52E-05	3.69E-05
C7orf61	1.254039352	-2.324856447	1.53E-05	3.71E-05
OLFM4	4.022824965	2.24608776	1.54E-05	3.72E-05
SCTR	1.956840129	2.622407285	1.54E-05	3.73E-05
SLC9C2	1.861991898	-1.752958032	1.54E-05	3.73E-05
ADORA1	1.551397349	1.459358846	1.54E-05	3.74E-05
SYCP2L	1.239819912	-2.378610267	1.54E-05	3.74E-05
AC134878.2	1.190747769	-1.954342944	1.57E-05	3.79E-05
SLCO5A1	1.411869773	-1.017943844	1.57E-05	3.80E-05
AC005357.2	-2.10852091	-2.434806609	1.58E-05	3.82E-05
AL354719.2	1.418364701	0.422933168	1.58E-05	3.83E-05
CAMK2A	1.92516057	-0.342889545	1.60E-05	3.86E-05
AC010907.2	2.106601821	-2.745942023	1.61E-05	3.88E-05
AC073365.1	4.174970617	-2.949776291	1.62E-05	3.90E-05
TRIM67	1.243370731	-1.154672306	1.62E-05	3.92E-05
DNAH6	-1.074786103	1.878968757	1.63E-05	3.93E-05
Z97056.1	1.5508267	0.397946815	1.63E-05	3.93E-05
AC243830.2	1.086398315	-2.752067548	1.64E-05	3.96E-05

ITGA3	1.196673669	4.200882613	1.66E-05	3.99E-05
TREML4	2.619199228	-2.715161977	1.66E-05	4.00E-05
LHFPL3-AS2	2.355640196	-0.210097304	1.67E-05	4.01E-05
SERPINB4	5.025491284	-1.483926661	1.67E-05	4.03E-05
AL035661.1	-1.224817946	1.747938582	1.68E-05	4.04E-05
GSTM5	-1.280862307	0.620423955	1.69E-05	4.07E-05
SMYD1	3.155251912	-1.954891349	1.70E-05	4.09E-05
AC015849.5	1.24998959	-0.653984625	1.70E-05	4.10E-05
CTNND2	2.403809817	3.040429419	1.71E-05	4.10E-05
CHST8	2.418196083	-0.032918564	1.71E-05	4.10E-05
RIC3	-1.284584005	-0.23302768	1.71E-05	4.12E-05
AL137802.2	1.20551223	-2.93305166	1.73E-05	4.16E-05
IHH	1.22026987	2.503141142	1.75E-05	4.19E-05
AC010487.1	1.426024316	-1.068183081	1.76E-05	4.23E-05
LINC02449	1.008604554	-2.660815411	1.77E-05	4.24E-05
PDCD1	1.461448932	1.099759192	1.77E-05	4.24E-05
LECT2	-1.136661338	6.474212089	1.78E-05	4.26E-05
ZNF813	1.024945704	1.713583402	1.78E-05	4.27E-05
CAMK1G	1.816110867	-0.251206463	1.78E-05	4.27E-05
ZNF790-AS1	1.00419574	-1.000653301	1.79E-05	4.29E-05
AC097063.1	-1.214607368	-2.598714486	1.80E-05	4.32E-05
AL928921.1	1.279075419	-0.026703448	1.81E-05	4.34E-05
ADGRB1	1.485548862	-0.352866134	1.82E-05	4.36E-05
NOVA1	1.432293259	1.046641997	1.82E-05	4.36E-05
CELF5	1.774895128	0.591412111	1.86E-05	4.44E-05
ACSL6	1.385814114	3.746157622	1.86E-05	4.45E-05
AC145138.1	1.002652922	-2.345202919	1.87E-05	4.48E-05
INSM1	3.154231562	-2.565448474	1.89E-05	4.52E-05
DKK3	1.164525237	4.75181985	1.90E-05	4.53E-05
CLDN6	2.023390127	-1.518218295	1.90E-05	4.55E-05
SLC1A7	1.823820935	3.135207437	1.91E-05	4.55E-05
C4orf54	2.058094436	-2.334385533	1.91E-05	4.56E-05
PTPRD	-1.04556051	3.007393242	1.92E-05	4.59E-05
PAPLN	1.311359553	3.589257917	1.94E-05	4.62E-05
ITPR3	1.312149115	3.401739183	1.94E-05	4.62E-05
CELF4	1.466879138	-1.535925795	1.94E-05	4.63E-05
AC007938.3	1.229731454	-1.774080657	1.95E-05	4.65E-05
LINC01550	-1.07630648	-1.84678762	1.99E-05	4.73E-05
SIX3	3.1804313	-2.915167311	1.99E-05	4.73E-05
IGKV2-24	-1.526382456	0.005571616	2.00E-05	4.75E-05
AP000808.1	1.759113416	1.494869792	2.05E-05	4.87E-05
SPRY4-AS1	1.404628221	-2.935843967	2.11E-05	5.01E-05
OXT	-1.647587936	0.499077848	2.11E-05	5.01E-05
RHCG	1.888388475	1.486892721	2.12E-05	5.01E-05
KCNJ4	1.519850001	1.791950506	2.12E-05	5.02E-05
FAXC	1.923896371	-1.597888522	2.12E-05	5.02E-05
SELE	-1.064180077	1.562954225	2.12E-05	5.03E-05
KLC3	1.817672833	-0.535190707	2.13E-05	5.04E-05
AC009041.2	1.070864421	-1.044253268	2.13E-05	5.05E-05
UGT8	2.217263212	-0.855456579	2.13E-05	5.05E-05
AC108868.1	3.944180591	-2.863188052	2.16E-05	5.11E-05
AC096711.3	2.094682529	-2.749068143	2.21E-05	5.22E-05
ST8SIA5	1.222770522	-2.358286379	2.21E-05	5.22E-05
CADPS	2.439815958	0.270020841	2.23E-05	5.26E-05
TMEM35A	1.783292888	-2.563267889	2.23E-05	5.28E-05
BTNL3	3.342333424	-0.103480571	2.24E-05	5.29E-05
CFAP45	1.144780688	-1.246866513	2.24E-05	5.30E-05
BLACAT1	2.459697662	-3.00764122	2.25E-05	5.32E-05

DUXA	4.460892945	-3.110355575	2.27E-05	5.37E-05
AL109659.2	1.005250736	-2.808158713	2.31E-05	5.46E-05
INTS4P1	1.111624211	-2.309366183	2.36E-05	5.55E-05
AC015802.5	1.044833879	-2.857603869	2.36E-05	5.55E-05
LINC01510	2.218947373	-2.683460787	2.37E-05	5.59E-05
ARHGAP36	5.589467007	0.511659158	2.38E-05	5.60E-05
AL049794.1	1.036094805	-2.748336202	2.39E-05	5.62E-05
AC008554.1	2.171663084	-2.785932417	2.39E-05	5.62E-05
AC106795.2	-1.210726264	-0.751227413	2.39E-05	5.62E-05
AC007495.1	-1.309809922	-1.719864205	2.41E-05	5.66E-05
MYH1	2.795226546	-1.912779633	2.41E-05	5.66E-05
S100A6	1.113863236	6.230112936	2.43E-05	5.72E-05
ARHGEF38	1.437325282	-0.014682142	2.44E-05	5.73E-05
PNLIP	4.704107186	-3.018840972	2.49E-05	5.84E-05
COL11A2	1.635293353	1.462001044	2.49E-05	5.85E-05
TWIST2	2.046314466	0.265071152	2.49E-05	5.85E-05
REG4	1.933241096	-0.149728558	2.50E-05	5.86E-05
ATP1A4	1.765173314	-2.684951211	2.50E-05	5.86E-05
KLK1	2.038345698	-2.761023336	2.50E-05	5.87E-05
FGF8	3.587376891	-2.5030628	2.50E-05	5.87E-05
MEG3	1.684168849	4.91971962	2.52E-05	5.90E-05
AL033397.2	1.085014837	-1.74520114	2.53E-05	5.93E-05
LCTL	1.087608964	-2.104468025	2.56E-05	6.00E-05
HMGN2P15	1.182721386	-1.383243964	2.58E-05	6.05E-05
ZNF454	1.198320808	-2.048852939	2.60E-05	6.08E-05
SNAP91	3.041826009	-2.609747723	2.64E-05	6.17E-05
PRDM16	1.387077988	0.847513938	2.66E-05	6.21E-05
TDRD12	1.188514626	-2.808884483	2.67E-05	6.23E-05
CLGN	1.344927832	3.494557266	2.69E-05	6.29E-05
AC112206.3	1.964824824	-1.421796173	2.74E-05	6.39E-05
LINC02315	2.371359608	-1.240421745	2.75E-05	6.42E-05
AC087273.2	1.813202962	-1.786389805	2.79E-05	6.50E-05
AC099552.4	2.042809169	-2.014151385	2.79E-05	6.50E-05
AC092645.1	1.074743809	-0.126642566	2.83E-05	6.58E-05
LINC01914	1.626486404	-2.425727268	2.85E-05	6.64E-05
ANGPTL1	-1.105727063	2.97443441	2.87E-05	6.67E-05
FAM183A	2.086927047	-1.818307697	2.87E-05	6.68E-05
AC004687.1	1.129665762	-0.134823824	2.90E-05	6.74E-05
LGR5	2.155723444	3.369879072	2.93E-05	6.81E-05
GDF6	-1.116989952	-1.388501541	2.94E-05	6.84E-05
AC104971.3	2.475352904	-2.585206639	2.95E-05	6.85E-05
SALL4	1.640474332	0.57777185	2.96E-05	6.86E-05
ZNF98	2.342426422	-2.676256524	2.96E-05	6.87E-05
SYT15	1.141063668	-1.277574704	2.97E-05	6.90E-05
AL590326.1	1.040339994	-2.039101305	2.99E-05	6.93E-05
CHRNA1	2.420109837	-0.738302005	3.05E-05	7.08E-05
LINC01579	2.75821737	-3.121515662	3.06E-05	7.08E-05
PNPLA5	-1.728053562	-2.498898955	3.08E-05	7.14E-05
JSRP1	1.187483716	-0.416641583	3.09E-05	7.16E-05
GRIN2D	1.016365417	0.216782658	3.10E-05	7.19E-05
C1QTNF9B	2.00648808	-2.880935921	3.11E-05	7.19E-05
SMCO2	1.20745772	-2.330690789	3.11E-05	7.20E-05
AC112206.4	1.757138568	-2.581742884	3.12E-05	7.22E-05
SLC6A11	2.439038915	3.325398184	3.16E-05	7.32E-05
NTNG2	1.17517015	-0.769893905	3.17E-05	7.34E-05
LINC00982	1.53179336	-0.188412211	3.18E-05	7.36E-05
PODN	-1.023911496	3.247670836	3.19E-05	7.38E-05
FGD5P1	2.348366887	-3.040513986	3.23E-05	7.45E-05

LINC02054	3.139724024	-2.43097346	3.25E-05	7.51E-05
HBE1	2.970552094	-1.854303571	3.28E-05	7.57E-05
AC027288.3	1.596674358	-2.527018384	3.31E-05	7.63E-05
AC009549.1	1.07825537	-1.086616871	3.35E-05	7.73E-05
RAMP2-AS1	1.152414784	-0.064903744	3.37E-05	7.77E-05
MGP	1.028810785	5.161683143	3.42E-05	7.87E-05
MIR181A2HG	1.215419046	-2.592533268	3.42E-05	7.87E-05
AC116025.1	-1.116943683	-1.427598661	3.46E-05	7.96E-05
MDGA2	2.823985565	-0.746742302	3.47E-05	7.97E-05
EFCAB12	1.147281464	2.108340413	3.48E-05	8.00E-05
LAMB3	1.073863177	4.714166182	3.51E-05	8.06E-05
S100A8	-1.122482386	2.455759115	3.52E-05	8.09E-05
SERPINE2	1.179015018	4.470751476	3.60E-05	8.26E-05
DDX11L2	1.04308765	-0.879798344	3.63E-05	8.32E-05
ADGRA1	-1.750837735	-2.027327599	3.63E-05	8.33E-05
NECTIN4	1.685739804	0.232463278	3.64E-05	8.35E-05
SBK1	1.096426771	0.561721776	3.65E-05	8.37E-05
ANKRD2	1.406794603	-1.609033252	3.67E-05	8.40E-05
CYP4F32P	3.597816278	-2.43555959	3.71E-05	8.49E-05
AL353747.3	2.445250053	-2.87721985	3.71E-05	8.50E-05
MAPK15	1.755858479	-0.339182496	3.73E-05	8.53E-05
CITED1	1.293193792	-2.013405393	3.78E-05	8.65E-05
TGFB2	1.178340533	1.83018718	3.82E-05	8.74E-05
CLIC6	-1.354790202	1.152976029	3.83E-05	8.75E-05
OBSCN-AS1	1.27040812	-1.886714465	3.83E-05	8.76E-05
MCHR1	1.218252497	2.002698114	3.91E-05	8.92E-05
FOXP2	-1.22910463	1.518871598	3.91E-05	8.92E-05
AL451069.1	2.063743884	-2.750109596	3.91E-05	8.93E-05
CDH18	3.259954909	-2.464327455	3.94E-05	8.99E-05
CARNS1	1.17006818	1.034667376	3.95E-05	8.99E-05
SLC6A2	3.232372605	3.130003312	4.00E-05	9.12E-05
RHEX	1.618886292	1.797315952	4.01E-05	9.13E-05
AC132807.2	2.546238608	-2.88204269	4.01E-05	9.13E-05
PSMA8	2.835390751	-2.961007875	4.02E-05	9.15E-05
AC079305.1	1.188366587	-1.925944482	4.04E-05	9.19E-05
AC073349.2	1.304413538	-2.734326326	4.06E-05	9.24E-05
SLC16A9	1.911844395	2.624659032	4.06E-05	9.25E-05
AC016395.1	-1.007780055	-1.700179313	4.07E-05	9.25E-05
IRGM	1.825483715	-2.742653564	4.07E-05	9.27E-05
UTS2	1.801851441	-1.470175717	4.09E-05	9.29E-05
SNAP25-AS1	1.344296067	1.014388393	4.11E-05	9.33E-05
WT1-AS	2.432154745	-1.884985152	4.19E-05	9.51E-05
IGFBPL1	1.98529911	-1.024958762	4.21E-05	9.55E-05
TNFAIP6	1.645554026	-0.567893202	4.22E-05	9.56E-05
MYT1	1.403996553	-1.295078077	4.23E-05	9.60E-05
XCR1	-1.05661093	-0.637346164	4.24E-05	9.61E-05
LARGE2	1.240867205	2.720846622	4.26E-05	9.65E-05
NCCRP1	1.844681682	-1.279738907	4.30E-05	9.74E-05
AC156455.1	1.15636528	-0.960407504	4.32E-05	9.79E-05
CDH17	2.594199709	0.928195171	4.33E-05	9.79E-05
SMIM22	2.121888742	0.64618742	4.34E-05	9.82E-05
CKM	1.485588477	-2.634078181	4.42E-05	9.99E-05
KCNC2	2.856154589	-0.272719711	4.42E-05	9.99E-05
OR51B5	2.297158663	-2.60611386	4.48E-05	0.000101153
CALCR	1.969233896	-1.942271196	4.48E-05	0.000101291
MOGAT3	1.035103882	4.44857409	4.51E-05	0.000101781
CLEC18B	1.062944323	-2.548896228	4.54E-05	0.000102459
OLAH	1.850754601	-2.795024197	4.55E-05	0.000102828

MIR3681HG	1.696218044	-2.472413394	4.65E-05	0.000104732
TMEM63C	1.701067871	-0.374383204	4.67E-05	0.000105253
AP000688.2	2.438718674	-2.641963736	4.75E-05	0.000107013
PCDHA3	1.975563328	-1.8680227	4.77E-05	0.000107266
TNFRSF11B	-1.009108652	3.008629816	4.80E-05	0.000107959
BPIFB1	2.582822927	-0.691089623	4.84E-05	0.000108924
SPTSSB	1.806440505	0.371788623	4.85E-05	0.000109143
EMID1	1.078602427	3.485936145	4.87E-05	0.000109519
RPS3AP54	1.01749972	-2.936434347	4.96E-05	0.00011131
RASAL1	1.614031254	0.4541569	4.98E-05	0.000111778
PPIAP46	1.205060369	-2.500670432	5.01E-05	0.000112436
LINC02269	4.007630033	-2.984366129	5.02E-05	0.000112644
SLC38A1	1.002571701	5.073077264	5.02E-05	0.00011272
U91319.1	-1.189879237	0.697777768	5.04E-05	0.00011317
NNAT	1.081924677	-2.042312799	5.07E-05	0.000113678
NOX5	1.335328604	-2.5885767	5.08E-05	0.000113909
SNURF	1.006196621	-2.535172049	5.10E-05	0.000114165
MT1A	-1.188350031	4.008286497	5.14E-05	0.000115191
FOXO6	1.539211333	0.008443228	5.16E-05	0.00011555
OTOGL	1.476188042	-2.865618135	5.17E-05	0.000115691
NKX2-3	1.83726874	-2.605086081	5.17E-05	0.000115709
FRAS1	1.710910265	3.080537064	5.19E-05	0.00011612
TGFB2-AS1	1.512905936	-2.523218527	5.20E-05	0.000116421
RHOXF1-AS1	1.381247097	-1.135671555	5.23E-05	0.000117026
ZNF711	1.267775633	0.772791993	5.24E-05	0.00011719
TNXA	1.005923154	0.997967472	5.25E-05	0.000117376
WTAPP1	1.760480984	-2.600087327	5.29E-05	0.000118278
APOD	1.148509098	1.089197966	5.32E-05	0.000118825
FHL5	1.278297222	-1.060416308	5.32E-05	0.00011885
CD1A	1.675771623	-1.752113874	5.34E-05	0.000119226
AP000345.2	1.124930338	-2.272821191	5.34E-05	0.000119237
AC008514.1	1.797525419	-3.009897186	5.39E-05	0.000120296
AC237221.1	1.809313806	0.056699323	5.41E-05	0.000120848
OVOL1	2.026995709	-0.046059873	5.42E-05	0.000120931
AC093720.1	1.721351543	-0.418369542	5.45E-05	0.000121578
SEC14L5	1.13086832	-1.087922182	5.47E-05	0.000122007
PCDHB16	1.185622982	1.099112925	5.51E-05	0.000122866
APOA4	2.646833921	8.601014726	5.58E-05	0.00012436
LINC01978	1.272082679	-2.241310466	5.83E-05	0.000129579
AC079145.1	1.088243287	-2.964126607	5.86E-05	0.000130031
RGS13	1.910695902	-1.782253574	5.90E-05	0.000130932
AL627309.6	1.086400427	-0.492449497	5.93E-05	0.000131706
NPY5R	-1.252868246	-1.805840175	5.94E-05	0.0001318
CLIC3	1.269415853	0.283575814	5.94E-05	0.000131806
AQP7P2	-1.201052135	-2.423608912	5.95E-05	0.000132128
SIM1	2.171577536	0.530073033	5.96E-05	0.000132293
PCDHB9	1.210441488	-0.878863904	5.97E-05	0.000132435
KCNT1	1.173376273	-2.526407912	6.03E-05	0.000133567
PCDHA4	1.70402399	-2.437720737	6.07E-05	0.000134373
LINC01807	2.155309659	-2.381210375	6.07E-05	0.000134378
ALLC	-1.218354132	-1.700401527	6.10E-05	0.000135074
AC139749.1	2.219240978	-0.309241455	6.12E-05	0.000135455
LTF	1.407490328	1.767454856	6.18E-05	0.00013674
ST6GALNAC1	-1.040794186	-1.590234291	6.26E-05	0.000138363
C9orf84	1.247191829	-2.537065253	6.28E-05	0.000138784
TRPM3	1.417444937	0.084738444	6.30E-05	0.000139181
NRK	-1.098310508	-1.597887347	6.33E-05	0.00013985
LINC01249	3.51753509	-3.18059863	6.35E-05	0.000140182

HLA-DOB	1.123594757	1.02181035	6.37E-05	0.000140703
GFRA3	2.634246545	0.118870051	6.40E-05	0.000141281
RHOBTB1	1.197263886	4.526633651	6.44E-05	0.000142154
SLC6A1-AS1	1.152877979	-1.707900629	6.44E-05	0.000142157
LOXL1-AS1	1.293975685	-0.604370134	6.52E-05	0.000143762
AC131009.4	1.042666812	-2.694274912	6.58E-05	0.000145024
DNMT3L	-1.183561557	-0.608619252	6.59E-05	0.000145217
GATD3A	-1.152974559	1.184058205	6.62E-05	0.000145853
SHISA6	1.454022803	-2.621668559	6.62E-05	0.000145863
BEND6	1.536375053	-1.893532456	6.66E-05	0.000146584
DCLK1	1.188626052	0.305134468	6.68E-05	0.000146933
LINC01836	1.153537437	-0.688631774	6.68E-05	0.000147117
LINC00460	3.057901098	-3.178924368	6.75E-05	0.000148477
LRRC37A7P	-1.056698022	1.821967163	6.77E-05	0.000148906
HMCN2	1.40997381	3.213440485	6.80E-05	0.000149651
MEGF11	1.290195181	-2.663202162	6.91E-05	0.000151784
GALNT5	2.122319236	-0.750918999	6.93E-05	0.000152185
CNTNAP3	1.275794311	0.158205703	7.08E-05	0.000155403
ZNF578	1.208638383	-2.473100991	7.11E-05	0.000155925
AC004231.1	1.79947122	-2.607406788	7.13E-05	0.000156216
RUFY4	1.127104296	-1.347650223	7.18E-05	0.000157181
CYCSP34	1.053069459	-2.56620214	7.30E-05	0.00015983
AL049873.2	1.028083697	-2.814014552	7.34E-05	0.00016067
ADCY8	2.991865723	0.556572889	7.38E-05	0.000161295
AC016205.1	1.786908954	-0.129040168	7.39E-05	0.00016154
PRSS50	1.206512661	-0.489405077	7.40E-05	0.000161732
SYNGR1	1.061298382	3.619667251	7.41E-05	0.000162048
MATN1	1.169838433	-2.80654951	7.48E-05	0.000163341
NRXN3	1.472217828	1.61006686	7.49E-05	0.000163609
AC008592.5	-1.089409307	-1.702782144	7.52E-05	0.000164309
AC004836.1	1.045137238	-2.034929338	7.62E-05	0.000166262
PCDHA7	1.711506456	-2.404448531	7.73E-05	0.000168546
BACE2	1.312191566	5.048576825	7.79E-05	0.000169699
PLEKHD1	1.276088689	-2.395133433	7.89E-05	0.000171584
SLC47A1P2	1.0905067	-1.974053745	7.91E-05	0.000172172
MROH2B	2.481302277	1.655099906	7.99E-05	0.00017368
ALG1L13P	-1.113937729	-0.729017781	8.01E-05	0.000174066
TREH	-1.069711017	2.249355069	8.10E-05	0.000176069
FAM90A1	1.256223107	-1.877285162	8.10E-05	0.000176069
OXTR	1.42133883	1.049615484	8.21E-05	0.000178354
BEGAIN	1.467116432	0.060028954	8.25E-05	0.000179098
SYNDIG1	1.862074619	-1.747402467	8.26E-05	0.000179284
C7orf77	2.251933158	-2.393830871	8.26E-05	0.000179354
ASB11	2.048959726	-2.514170024	8.29E-05	0.000180012
AL590822.1	1.208468856	-2.848846996	8.36E-05	0.000181436
SLITRK1	3.13195421	-2.740892628	8.36E-05	0.000181436
RANBP17	1.133780484	1.340067239	8.41E-05	0.000182376
GAPDHP14	1.485688904	-1.599614021	8.57E-05	0.000185798
POSTN	1.35934298	4.132007596	8.58E-05	0.00018588
CLPSL1	4.084908578	-3.127205565	8.63E-05	0.000186951
GAD2	4.25342158	-2.784287475	8.67E-05	0.00018774
DMRT2	2.848667179	-2.897437212	8.72E-05	0.000188666
TCF23	1.312313008	-1.944974744	8.81E-05	0.000190728
ADAMDEC1	1.51448615	0.591337917	8.82E-05	0.000190848
MB	1.305467559	0.371265318	8.82E-05	0.000190859
DPP6	1.390558581	-2.9586103	8.98E-05	0.000194205
SLC29A4	1.397707249	4.479546565	9.05E-05	0.000195594
ZNF727	1.570093048	-2.132627643	9.11E-05	0.000196693

CDH3	1.500325431	-0.69962669	9.22E-05	0.000198938
AL513314.2	1.078418997	-2.807015668	9.27E-05	0.000199964
TP63	1.218148841	-0.347182959	9.30E-05	0.000200609
LINC02381	1.245176955	1.29218983	9.34E-05	0.000201443
AC009093.7	1.786064385	-2.634697816	9.36E-05	0.000201727
TPSG1	1.707259134	-0.985206626	9.36E-05	0.000201751
LINC02211	2.47180898	-2.844022364	9.57E-05	0.000206005
LINC00844	-1.325153786	3.657424053	9.62E-05	0.000206975
IQCA1	1.374034512	-0.228232953	9.62E-05	0.000207071
TUBA3E	1.924353105	-2.257778346	9.75E-05	0.000209709
CDH26	1.120952323	-1.625720631	9.82E-05	0.000211148
SPRR1B	-2.148702109	-2.666207698	9.85E-05	0.000211694
AKR1C2	1.035217442	8.240989083	9.98E-05	0.000214245
AP001626.1	1.785572366	-2.728182881	0.000100212	0.000215146
ZNF114	1.034210221	-2.350672498	0.000100449	0.000215593
SGPP2	1.542866868	1.386635567	0.000100573	0.000215837
FXYD2	1.989172176	4.253999477	0.000100916	0.000216532
AC152010.1	4.842009556	-2.377007237	0.000101009	0.00021669
ABHD11-AS1	1.486010614	-2.368215665	0.000101094	0.000216831
LINC01303	1.530842183	-2.468947224	0.00010323	0.000221264
AP000924.1	1.602224153	-1.024844019	0.000103541	0.000221823
SPIB	1.801129209	1.334250839	0.000103607	0.000221923
ARHGEF16	1.007145327	3.780026676	0.000105391	0.000225528
AC079209.1	1.321506324	-1.019431829	0.000105726	0.000226223
CHEK2P2	3.493528285	-3.112005984	0.000105813	0.000226389
SULT1E1	-1.086003344	3.123113169	0.000106136	0.000226949
OPRK1	3.649164265	-1.156175007	0.000107255	0.000229209
FCGR2C	1.203461643	0.436740219	0.000111161	0.000237172
FRMPD1	1.286573346	1.478120041	0.000111674	0.000238221
ADGRF1	2.570772518	-0.715983809	0.000112046	0.000238992
GALR3	1.385197202	-1.509271868	0.000112556	0.000240011
APCDD1	1.691484069	3.780656864	0.00011338	0.000241681
PIANP	1.484743097	-2.405821537	0.000114474	0.000243823
AL592293.1	1.741964867	-2.905558093	0.00011515	0.000245098
CSMD3	2.488428977	-2.309001047	0.000115262	0.000245315
RAB3C	1.690612375	1.579319478	0.000116865	0.00024849
LINC00504	1.161364338	-0.832981038	0.000116939	0.000248624
LY96	1.02274503	2.899770532	0.00011759	0.000249793
AC005162.3	1.105434306	-2.250446591	0.000118573	0.00025181
KRT1	-1.735332979	-1.287022114	0.000118657	0.000251964
BSPRY	1.201644037	1.670468277	0.000119865	0.000254362
IL1RAPL1	1.785320799	-2.435814773	0.000120304	0.00025522
AL358115.1	1.088714921	-2.935868025	0.000120353	0.0002553
FAT2	1.151146421	-1.906017526	0.000120522	0.00025561
PCDHGA2	1.228346295	1.252213789	0.000121026	0.000256606
AC106869.1	1.073079089	-2.554492468	0.000121668	0.000257918
KCNJ10	-1.095509356	0.613373088	0.000124292	0.00026323
AC010501.1	1.130493153	-1.492971323	0.00012483	0.000264271
TRPC3	1.887251801	-1.434088482	0.000125257	0.000265125
LANCL1-AS1	1.058872632	-2.824039335	0.000125286	0.00026516
LINC01619	1.056337364	-1.12143874	0.000125746	0.000266108
AC131097.2	2.008923767	-1.590296124	0.000125986	0.000266591
DLX3	2.237652463	-2.605426683	0.000126217	0.000267054
PNLDC1	1.561452666	-1.788542064	0.000126628	0.000267824
AC097658.1	1.076771774	-2.286810959	0.00012716	0.000268822
RAP2CP1	1.486846641	-2.802000343	0.000127603	0.000269682
LINC02038	1.380882841	-2.357998717	0.000128764	0.000272032
LINC01152	1.475847618	-0.671386101	0.000130656	0.000275638

AL121949.1	2.62979936	-2.135902371	0.000130694	0.000275693
AL121974.1	3.016219762	-2.036079638	0.000132007	0.000278305
ACPP	1.730565644	-0.760663484	0.000133551	0.000281322
PCDHA6	2.057473884	-2.086415762	0.00013445	0.000283083
PRR15	1.653683887	0.589189375	0.00013499	0.000284193
TMEM45B	1.010034102	6.025537394	0.000135929	0.000286061
CNTN3	-1.30533025	0.925391587	0.000136222	0.000286651
FAM151A	-1.308987507	2.422587858	0.000137589	0.000289366
RSPO4	1.667183825	-1.885365679	0.000139304	0.00029278
EDA2R	1.1001242	0.589826706	0.00014013	0.00029446
ABHD12B	1.053057005	-0.15578789	0.000140646	0.000295489
TMEM246	1.020380441	2.917158432	0.000141446	0.000297113
FOXQ1	1.513363505	2.558930709	0.000141538	0.000297278
C6orf52	1.068450718	-0.906250797	0.000141729	0.000297653
GUCY1B2	1.709846614	-0.852916352	0.000142744	0.000299643
CCL28	1.057643424	2.421502161	0.000143244	0.000300665
AC020571.1	1.511159243	-2.202701435	0.000143697	0.000301446
LINC01993	1.642416199	-2.571737225	0.000144152	0.000302335
RASL10B	1.579017419	3.561542441	0.000144432	0.000302861
LMO7-AS1	1.228183771	-2.439130667	0.000144439	0.000302861
CPHL1P	1.275384594	-0.178018302	0.000145427	0.00030479
BARX1	2.086808618	-0.523292195	0.000146391	0.000306695
AJ009632.2	1.527429411	0.28469135	0.000146895	0.000307692
KLK13	2.842956193	-2.036222997	0.000147556	0.000308962
GSTA2	-1.174554523	7.461977695	0.000147999	0.000309831
ZNF66	1.071769832	-1.545398081	0.000149177	0.00031218
CTLA4	1.098644026	0.053008745	0.000149416	0.000312652
TERB2	-1.292325963	-1.140126915	0.000150139	0.000313988
MIR1-1HG-AS1	2.040849188	-2.69013185	0.000150665	0.000314972
GCSAML	1.903390071	-2.011453009	0.000152207	0.000317896
SUSD4	1.40256901	3.889027795	0.000155791	0.000324866
LINC01344	1.110577408	-1.109763702	0.000157782	0.000328835
RGS6	1.320022734	-1.386445927	0.000161365	0.000335771
MUC5B	2.65810681	5.72597098	0.00016252	0.000338048
MSC-AS1	1.17753798	1.648710403	0.000164536	0.000341988
NPIPA3	1.132504739	-2.517358436	0.00016471	0.000342318
LY86-AS1	2.061129313	-2.156421497	0.00016541	0.000343613
S100A14	1.347830967	4.619432014	0.000167016	0.000346659
AC100826.1	1.694099055	-2.507193818	0.000170232	0.00035298
TH2LCRR	1.249859864	-3.09010217	0.000172745	0.000357786
MT1DP	-1.225284542	1.438752133	0.000172917	0.00035811
BEND4	-1.213176773	-2.424123343	0.000173657	0.000359542
POU2F3	1.306992578	-0.632872904	0.000176071	0.000364136
AC131097.1	1.785110588	-2.66523258	0.000177197	0.000366397
STK31	1.26383576	-1.362017918	0.000178736	0.000369341
LINC02247	3.681720227	-2.616135475	0.000179406	0.000370623
OLFM3	2.950874774	-2.102288243	0.000179428	0.000370633
BX322559.1	4.267259773	-2.910297799	0.000179579	0.000370878
DUOXA1	1.644776425	-1.818215097	0.000179994	0.000371699
HYDIN2	1.452223318	-2.892404466	0.000180248	0.000372191
ZPLD1	2.434972602	0.725791982	0.000181847	0.000375387
ERVE-1	-1.62859736	-2.770096116	0.000182681	0.00037704
NECAB1	1.083558411	0.193615025	0.000189291	0.000389926
ASXL3	-1.096162688	-0.488734659	0.000192763	0.000396714
TESC-AS1	1.776249398	-2.55876533	0.000199032	0.000408801
TMEM200A	1.00865444	0.279353002	0.000199964	0.000410589
AC004593.1	1.248698853	-1.857240832	0.0002006	0.000411782
IGKV1-8	-1.413559387	-0.362642283	0.000205655	0.000421618

AP000424.2	1.437386162	-2.80310778	0.000207141	0.000424548
NFE4	2.458311939	-2.270921401	0.000207624	0.000425381
AC019117.1	1.129234417	-0.574549343	0.000208686	0.000427479
ERVW-1	1.836845566	-3.010210087	0.000210503	0.000430807
BX470209.1	1.98921853	-2.61171555	0.000213646	0.00043712
CYP24A1	2.573087382	-2.737945508	0.000217175	0.000444055
MTCO1P42	2.593813941	-2.281414655	0.000217703	0.000445012
NT5DC4	1.329218544	-2.684224401	0.000220617	0.00045068
MYMK	2.08135521	-2.418413103	0.000222686	0.000454741
AC080188.2	1.024819848	-2.907228611	0.000225363	0.000459998
AC120498.10	1.789262639	-2.621091437	0.000231345	0.000471263
SDK2	1.462616674	1.230590383	0.000235433	0.000479066
MMEL1	1.173077819	-1.604161925	0.000241521	0.000490741
BX539320.1	1.10326238	-2.368996343	0.000241713	0.000491086
ADAD1	3.930274641	-3.110822071	0.000242184	0.000491953
CCR8	1.328924386	-2.90857881	0.000242211	0.000491964
IRX5	1.472691413	-1.060565252	0.000243732	0.000494919
LMO3	1.018067585	-0.96835242	0.000245565	0.000498234
PLN	1.305486096	0.5356037	0.000248863	0.000504467
XAGE2	3.342552041	-2.576872281	0.000253514	0.00051343
VN1R48P	1.454374175	-2.780320574	0.000259954	0.000525853
SERTM2	-1.498106361	2.568229881	0.0002606	0.000527113
GABRG3	1.795439682	-1.716344092	0.000261292	0.000528465
AC048380.2	1.021853309	-2.672593204	0.00026402	0.000533741
AC021723.1	1.490581336	-2.848587344	0.000264161	0.000533978
CPEB1	1.763615558	-1.01367351	0.000264421	0.000534454
HPGD	-1.082685997	6.493826367	0.000267747	0.000540591
CCL20	1.305866117	5.098044663	0.000269283	0.000543448
AC026336.3	4.216429656	-2.466138699	0.000270539	0.000545786
MYBPC3	1.390495169	-1.845784183	0.000271204	0.000547078
HSPA6	1.238906778	3.060735333	0.000271544	0.000547712
AC016723.1	1.753481198	-2.195709272	0.000275285	0.000554611
LINC00323	1.723087037	-3.025876179	0.000276466	0.00055684
PAX2	2.302406227	-1.040688897	0.000280155	0.000563813
FUNDC2P2	1.57624403	-1.876792882	0.00028029	0.000564033
CPNE4	1.657882421	-3.083606711	0.000282099	0.000567469
COL10A1	1.678774936	0.421906837	0.000285466	0.000573933
AC131009.2	1.22072926	-1.655382669	0.000286834	0.000576477
KIAA1549L	1.41255863	-0.948726069	0.00028848	0.000579576
CST5	2.304402333	-2.237269284	0.000288769	0.000580052
LINC02544	1.59791502	-2.935356048	0.00029163	0.000585274
WNT10B	1.090398955	-1.69162167	0.000294377	0.000590416
MEG9	1.765618662	-1.303306776	0.000295365	0.000592218
AC010980.1	1.797110243	-3.034910056	0.000295381	0.000592218
SLC2A14	1.385166079	-0.221798565	0.000296939	0.000595129
SERPINB7	3.695821534	-2.260756186	0.000298418	0.000597986
LINC00470	1.548386382	0.076699106	0.000300874	0.000602584
AC122713.2	-1.085358964	0.339435167	0.000300982	0.000602746
ROR1	1.13679621	0.721727596	0.000302998	0.000606619
NMU	1.826662878	-3.093055841	0.000303557	0.000607684
PSD2	1.099519772	-2.136104046	0.000305744	0.000611516
GAPDHP1	1.079287124	3.0907175	0.000305913	0.000611799
NMRK2	2.671493599	-1.86439481	0.00030828	0.000616311
KRT6B	-1.639814992	-0.886465933	0.000308339	0.000616375
GPC5	1.470531368	0.922410752	0.000319617	0.000637553
CSDC2	-1.094865668	-0.778512739	0.000320183	0.000638568
TBX18	1.449109729	-0.205262032	0.000322506	0.00064303
KRT17P4	-1.196975215	-2.310613707	0.000323582	0.00064509

C6orf223	2.004670009	2.459933858	0.000324108	0.00064605
TDRD9	1.612589551	-0.314846588	0.00033159	0.000659907
AC136475.3	1.2663624	1.906262336	0.000332931	0.000662398
LGALS9B	1.928330049	-1.768052692	0.000333477	0.000663307
SPAG17	1.475876501	-2.511166303	0.000340867	0.000677283
LRRC36	1.244522401	-2.263088244	0.000342658	0.000680541
CALB2	1.6608171	-2.03597612	0.000342933	0.000681026
AC124798.1	1.045242111	0.529404237	0.000343981	0.000683047
CELF2-AS1	1.937040166	-2.922527898	0.000344721	0.000684334
CPSF4L	1.154331878	-2.9486551	0.000345445	0.000685528
NUP210L	1.427718721	-2.601804837	0.000349316	0.000692903
UGT2B26P	1.266743563	-2.553443013	0.000349626	0.000693395
AC104072.1	3.323357571	-2.699969252	0.000351631	0.000696815
NCAN	1.625153844	-1.64092407	0.000363481	0.000718833
SERPINB3	3.8709265	-0.779280517	0.000367907	0.000727073
BATF	1.022871183	2.13488493	0.000368359	0.000727901
COX6A2	-1.173312711	0.895441781	0.000369371	0.000729773
DIO3OS	-1.133637033	1.413829457	0.00037066	0.00073206
DUSP26	1.258649622	-1.394975828	0.000372634	0.0007357
RIMKLA	1.080207437	-0.529243422	0.000373911	0.000738012
AC104581.3	1.101316473	-2.877442541	0.000379201	0.000747676
LPAR3	2.244126103	-0.6239096	0.00038158	0.000751969
AL117382.1	1.083417176	-1.153049818	0.000387912	0.000763505
EWSAT1	1.039261663	-1.329757483	0.000388223	0.00076405
LINC00222	1.060249829	-2.120523487	0.000389317	0.000765935
FOXL2	2.350002075	-2.934076854	0.000393394	0.000773343
KCNA1	3.069413082	-3.3788435	0.000400546	0.000786296
DEGS2	1.114504307	0.633843739	0.000400868	0.000786861
SHC4	1.281689861	-0.632851923	0.00040127	0.000787581
ACTC1	1.674971828	-2.140819395	0.000403182	0.000791056
CLDN4	1.420713258	5.029117283	0.00040579	0.000795685
SYT6	1.300633719	-2.837411604	0.000410589	0.000804883
SNORA59B	1.082387665	-1.822015528	0.000416532	0.000815818
ADGRG5	1.131232765	2.615579754	0.000418668	0.000819786
PRTG	1.161641602	-0.872725232	0.000423595	0.000829143
VLDLR-AS1	1.335862125	-2.230745259	0.000427195	0.000835678
TTYH1	1.512085444	1.684112561	0.000427412	0.000835957
CDS1	1.196635398	2.279253832	0.000429631	0.000840076
AC013652.1	1.193109851	-1.401045949	0.000432131	0.000844595
PRSS16	1.933714273	0.504967621	0.000434757	0.000849284
AC027228.2	1.144035745	-2.862554696	0.000435323	0.00085024
ADGRF4	2.251008881	-2.402822579	0.000437346	0.000854042
RASEF	1.121866041	2.999089487	0.000437865	0.000854982
LRRC66	1.068288523	-1.625561987	0.000438478	0.000856028
C9orf106	1.101704443	-2.111684667	0.000441849	0.00086246
GAL3ST2	1.400884159	-1.947928142	0.000442924	0.00086427
ADGRG3	1.213187474	1.598499607	0.000445654	0.000869355
EPN3	1.497848046	-0.214886105	0.000446123	0.000870118
C11orf53	2.856942154	-1.592012627	0.000454773	0.0008856
AC018816.1	1.041425102	-0.842957236	0.000455226	0.000886406
AC124312.3	1.269918667	-2.577690829	0.000455729	0.000887231
ACSM1	1.286266693	5.303431476	0.000456438	0.000888455
COL9A3	1.169918361	2.586513183	0.000457986	0.000891082
RPS6KA6	-1.271078987	-0.687472268	0.000462159	0.00089842
AGAP12P	1.03642022	-1.933426676	0.00046354	0.000901026
XK	1.051418847	1.817956241	0.000465394	0.000904315
GSTM1	-1.402386865	5.920067865	0.000465633	0.000904672
AL023583.1	1.140183225	-2.223038405	0.000472057	0.000916227

ACTN3	1.297371819	-2.989506813	0.000479476	0.000929097
HLA-U	1.046458456	-1.74677463	0.000487114	0.000942673
SHANK1	1.148255878	-2.098401984	0.00048995	0.000947614
ADAMTS7P3	1.056776777	-2.84969065	0.000490897	0.000949255
P2RX2	2.472553617	-0.795374521	0.000493489	0.000954101
SMPDL3B	1.127369666	0.709387107	0.000497126	0.000960552
TSSC2	1.010254127	-1.434776778	0.000498854	0.000963645
CRP	-1.17940595	11.13050222	0.000502236	0.000969755
TRDN	2.914354051	-1.355402315	0.000511385	0.000986145
KCNH3	1.17331275	-0.252521883	0.000513129	0.000989423
SCNN1B	1.292061628	-0.152779896	0.000514547	0.000991815
SLC25A24P1	2.307312013	-3.077560534	0.000519437	0.001000723
BCL2L14	1.075744826	0.423737344	0.000527385	0.001015072
GALNT9	1.672728807	-1.712931195	0.000528726	0.001017393
LINC01671	1.211122469	-0.005528718	0.000529596	0.001018804
SPINT1	1.380348566	4.7564272	0.000531862	0.001022978
PI3	1.705978543	1.528322171	0.000533294	0.001025566
WNT4	1.304245379	2.649029908	0.000534767	0.001028221
SLC22A17	1.285912758	3.370454213	0.000535341	0.001029236
IGKV5-2	-1.379812747	-2.452769661	0.000540522	0.00103893
ST8SIA2	2.302842603	-3.022746136	0.000541293	0.001040233
FCRLA	1.511506234	0.610842048	0.000543431	0.001043983
LOXL4	1.114820028	4.658371333	0.000550274	0.001056223
MGAM	1.484772261	0.490027009	0.00056207	0.00107757
CCNJL	1.017301226	0.310131272	0.00057263	0.001096687
IGF2-AS	1.951634788	0.588084946	0.000574547	0.001100265
SLC7A4	-1.189691496	-1.689770482	0.000576513	0.001103652
ZG16B	1.440869541	0.782454383	0.00057761	0.001105373
EPCAM	1.73573775	5.305169323	0.00057761	0.001105373
PRIMA1	-1.392793488	-1.638161736	0.00058201	0.001113127
AP3B2	1.526413849	-1.924358149	0.000587919	0.001123469
AC100793.4	1.031850231	-2.801244904	0.000590189	0.001127613
KRT23	1.700531961	5.028885702	0.000590851	0.001128783
TRPC5	-1.068831933	-0.896036482	0.000591151	0.001129259
GOLGA6B	-1.190652795	-2.658168452	0.000600958	0.001147112
AP001476.1	1.784163927	-3.031923642	0.000604401	0.001153092
UGT2A1	-1.222052922	1.326027701	0.000608037	0.001159535
FBLL1	1.352625224	0.614914544	0.000619401	0.001179914
FGF20	2.226969401	-2.718045651	0.00062492	0.001190006
LHFPL3	2.05677631	-2.497397702	0.000628462	0.001196345
CR392039.2	1.529869166	-2.377290152	0.0006436	0.001223184
EPHA1-AS1	1.148377902	-1.895122115	0.000644666	0.001225002
PIFO	1.191162973	-1.231851415	0.000648112	0.001230818
HAP1	1.231394246	-0.99342996	0.000649063	0.001232415
LINC01705	1.519457239	-1.913057477	0.000649954	0.001234002
CR1L	-1.062782786	-2.555257865	0.000651238	0.001236126
SLC6A4	1.123697079	-1.66166995	0.000657688	0.001247521
NWD1	1.392994543	-2.686136981	0.000658608	0.001249161
PAX8-AS1	1.014029727	2.638210527	0.000658685	0.001249202
EYA4	2.08063692	-0.419574953	0.000667071	0.001263926
AP001636.2	1.159581523	-2.859743932	0.000676407	0.001280531
MIR646HG	1.237121567	-1.448965635	0.000679067	0.001285132
MYT1L	2.370951131	-1.889462151	0.000685347	0.001295592
HAS2	-1.073493744	0.491820358	0.000692072	0.001307753
NPBWR1	-1.283266754	-0.483762195	0.000696098	0.001314806
AC022167.4	-1.023995683	-1.644291919	0.000699918	0.001321352
KANK4	-1.146494342	0.978580402	0.000706388	0.001332555
ZNF750	1.162261724	-2.569815813	0.000707314	0.001334189

ZNF888	1.068385873	-2.607707954	0.000716376	0.001350599
EDN3	2.190864549	-2.662898815	0.000719112	0.001355416
RNASE2	1.275896974	-1.00196862	0.000719223	0.001355511
FAM9A	2.391934802	-2.868533754	0.000722902	0.001361985
GRM7	1.641870397	-1.846235125	0.000724405	0.001364701
GPR88	1.580866836	5.160407047	0.00072809	0.001371412
RFPL1S	1.173093011	-1.336925109	0.000737379	0.001387625
PRSS30P	1.020151017	-1.917677826	0.000744077	0.001398817
KLK3	3.972583366	0.529428602	0.000761084	0.001429107
KCNH7	-1.089001607	-2.132244292	0.000762753	0.001431641
VANGL2	1.200822353	1.270762397	0.000764917	0.00143486
AC254629.1	1.203286139	-1.920366964	0.000772062	0.001447414
CHI3L2	1.078051071	-0.346840601	0.00078058	0.00146277
PCDHGC5	1.339891448	-3.005158303	0.0007821	0.001465374
GPR27	1.544203107	-0.593815942	0.000785926	0.001472295
AC073283.2	1.52903638	-2.141512777	0.000789359	0.001478109
AL590560.1	1.00225319	-2.513872435	0.000791684	0.001481966
DIRAS2	1.550518273	0.257433086	0.000807371	0.001508932
TMC4	1.245912246	3.884755295	0.000808464	0.001510848
DHRS2	1.465207737	6.145610082	0.000810709	0.001514664
DOC2A	1.019080974	-1.410928526	0.000811168	0.001515396
AL117190.1	1.770222923	-2.353402338	0.000814913	0.00152201
LINC00923	1.516559221	-1.914261126	0.000841106	0.001568442
SNTG1	1.683929487	-0.160826331	0.000844739	0.001574694
PLA2G4E	1.239328434	-2.954427848	0.000854412	0.001591795
CA3	1.568014402	1.278698048	0.000857779	0.001597888
AC104692.1	1.349215237	-2.002729215	0.000867776	0.001614945
KCNK10	1.416353067	-2.313181479	0.000879111	0.001635632
TUBA3D	1.032940932	-1.29090366	0.000893753	0.001661079
AC008163.1	1.818746393	-1.532278232	0.000898063	0.001668566
WNT1	1.791955651	-2.811297617	0.000926487	0.001717778
PPP1R27	1.045148325	-2.323630396	0.000931562	0.00172633
ROR2	1.02850295	1.101546262	0.000943616	0.001746498
FGF5	2.863672114	-3.072074637	0.000944841	0.00174862
LMOD2	1.458368035	-2.883845066	0.000964202	0.001782388
CYP4F23P	1.323980709	-2.205513605	0.000971215	0.001794166
HECW1	1.086059496	-0.729309038	0.000990475	0.001827851
MEG8	1.935740966	-2.488258596	0.000990511	0.001827851
KCNK2	2.129478082	-1.022883934	0.000996345	0.001838162
RPS14P8	1.22205279	-1.556344784	0.001002287	0.001848361
SLC35F3	1.471609957	-1.465274545	0.001032682	0.001901593
ESRP1	1.992757629	2.603999224	0.001036321	0.001907666
ARSI	1.012559819	0.271613702	0.001045396	0.001923653
LINC01014	2.44236786	-2.887568891	0.001055815	0.00194157
CCDC169	1.959844071	-3.083943032	0.001057938	0.001944579
WFIKKN2	1.735581281	-1.828920109	0.001089872	0.001998351
AC103702.2	1.908354556	-2.825847839	0.001095347	0.002008062
LINC02232	2.719615816	-2.39907318	0.001100319	0.002016185
TCP10	1.683606772	-2.506793841	0.001116209	0.002043293
ERLNC1	1.027583199	-2.259204339	0.001127391	0.002062414
SPINK4	2.393352888	-1.703072385	0.001128311	0.002063928
HUNK	1.124445524	2.044862897	0.001131131	0.002068917
FA2H	1.655610594	0.744178693	0.001135945	0.002077042
BCL11A	1.021679402	0.128534528	0.001138434	0.002081083
ARNT2	1.088590195	2.083317867	0.001160807	0.002119731
VAT1L	1.2940086	1.698193286	0.001166335	0.002129304
AC147651.1	2.350130629	-1.352239701	0.00119092	0.002171529
SPATA46	1.515646551	-1.40782134	0.001191667	0.002172639

POF1B	1.421337508	1.763049339	0.001223474	0.002226715
HRASLS5	1.501946836	-2.146503992	0.001228267	0.002234893
UPK1B	-1.264948571	-2.487248522	0.001242556	0.002259423
CYP11B2	4.570345542	-0.260254689	0.001256207	0.002282205
TMEM255A	1.006355398	0.111959514	0.001266625	0.002298146
AC090825.1	1.072442067	-2.337487309	0.001267771	0.002299856
BDNF	1.079211605	-1.499508525	0.00126959	0.002302592
CSAG2	2.246839576	-1.458570147	0.001293704	0.002341771
SOX1	3.226094811	-2.225733844	0.001305727	0.002362195
PLPPR3	1.46504879	-2.813008646	0.001323072	0.002391448
AL162293.1	1.490069221	-1.865614308	0.001330049	0.002403034
ANXA8	-1.112596476	-0.945705837	0.001341663	0.002422703
SLC2A1-AS1	1.018243559	-2.386346358	0.001375534	0.002481863
AL138749.1	-1.602328056	-2.761247795	0.001376334	0.002482906
CDX1	1.331263291	-1.710892219	0.001417673	0.002554186
HOXD13	2.607936938	-2.783442407	0.001439407	0.002589714
SH3RF3-AS1	1.013956485	-1.267331779	0.001461388	0.002625969
ANXA13	1.089451051	4.615379639	0.001463397	0.002629134
PDPN	1.271140101	0.373767145	0.001465375	0.002632169
AC127496.5	1.162497642	-2.804840826	0.001465427	0.002632169
HCN4	1.698209253	-1.717882844	0.001471229	0.002642167
AC009084.2	1.06490976	-1.671668	0.001476893	0.002651699
PADI2	1.053784706	1.167555229	0.001483968	0.002663547
PIK3CD-AS2	1.049901401	-0.789441968	0.001484214	0.002663776
AGRP	1.216504229	-2.455171429	0.001492142	0.002677575
AC129492.1	1.34527697	-2.968482733	0.001506741	0.002702254
AC138649.1	1.12088299	-2.775228189	0.001521771	0.002727396
COL17A1	1.04713084	-0.983365497	0.001522231	0.002727754
MIR378B	1.017774855	-2.813635758	0.001571961	0.002811112
FAIM2	1.072035924	-1.637546444	0.001578648	0.002822393
HTRA4	1.119195228	-2.043474409	0.001614117	0.002880885
CBLN4	1.612225803	1.210833591	0.001614197	0.002880885
EREG	1.846601189	0.68510185	0.00163062	0.002908572
GLI2	1.302902691	0.773627693	0.001659515	0.002957331
OLR1	1.33092131	1.242045038	0.001659542	0.002957331
AL390778.2	-1.704062666	-2.298997762	0.001664121	0.002964075
GREM2	-1.015267963	4.377373382	0.001678729	0.002987717
SLCO4A1-AS1	1.945514524	-1.464190559	0.00172882	0.003071003
GRM3	1.76387837	-2.199093591	0.001739959	0.003090054
SLC3A1	-1.063883224	3.614654758	0.001749946	0.003106065
ITGB6	1.201091178	0.40544925	0.001755169	0.003114841
SLC22A2	1.337524229	-1.579738978	0.001771482	0.003142545
CNTN2	1.057123758	-1.967737269	0.001783112	0.003161671
AP005233.2	1.977154788	-2.810463663	0.001803648	0.003194703
ANK1	1.029249604	-0.611046702	0.001832916	0.003242785
LMX1A	1.521792318	-2.353274297	0.001833945	0.003244091
DNAAF1	1.268074554	0.759180713	0.001838416	0.00325123
SLC14A2	1.466164346	-0.545167499	0.001847803	0.003266539
AC002480.1	1.085948411	-2.899642181	0.001857702	0.003282224
GRAMD2A	1.103893887	-2.484323702	0.001885	0.003328352
TMEM156	1.011182125	2.159221154	0.001915867	0.003379919
GCK	1.863291991	3.857407327	0.001941421	0.003420148
AC106798.1	1.506715615	-2.85287698	0.001954553	0.003441927
KLK6	2.293879143	-2.013718287	0.00196132	0.00345167
KRBOX1	1.051594415	-1.926315461	0.001979211	0.003480965
AC098617.1	1.519537094	-2.990275111	0.002000941	0.003517249
RGS7	-1.3301073	-2.925901291	0.00200241	0.003519278
AC092068.2	1.139971318	-2.62438057	0.002006963	0.003527002

CFAP47	1.238363817	-2.061467277	0.002037279	0.003576066
RBBP8NL	2.060914168	-1.554508168	0.002041939	0.003583403
CGA	1.772963999	-1.148412864	0.002052236	0.003600343
C10orf90	1.881323652	-1.017437168	0.002076708	0.003638996
IGLV1-36	-1.21632155	-1.196894357	0.002082718	0.003649242
SLITRK5	1.134301304	-2.344088478	0.002085272	0.003652858
NEFH	1.020373171	-0.00117578	0.002114774	0.003699904
LINC01727	-1.256182427	-1.514860566	0.002131296	0.00372692
MLIP-AS1	1.134638552	-1.944730309	0.002162664	0.003778667
SPINT1-AS1	1.210971879	0.082352168	0.002166123	0.003784415
LINC01169	1.025875956	-1.313198966	0.002176419	0.003801513
AP000757.1	1.065393547	-1.577895939	0.002192725	0.003829098
AL445189.2	1.505940957	-1.98739459	0.00220789	0.003854075
NLRP2	1.248482097	0.546009882	0.002230562	0.003891526
LGALS9C	1.338570566	-0.439383785	0.002304052	0.004010046
AC010127.1	1.464114132	-1.944825428	0.002330063	0.004053111
RXRG	1.327230596	0.919209407	0.002404505	0.004176108
MYEOV	1.585208528	2.207363584	0.002417008	0.004195543
AL137798.1	1.24896752	-0.561514872	0.002522769	0.004370313
UGT1A8	1.340515994	-1.315332097	0.002590906	0.00447864
IAPP	1.262965856	-1.181897142	0.002592426	0.00448023
SYT13	1.713616986	2.69951602	0.002598294	0.004488984
EFNA5	1.228494485	1.397967337	0.002606208	0.004501266
AC079360.1	1.185051904	-0.6938705	0.002611586	0.004509183
PDX1	1.7176522	1.992396197	0.002611598	0.004509183
AC004540.1	1.003279953	-1.063096296	0.002651243	0.004569632
DNAJB3	1.129947956	-2.132656996	0.00268274	0.004619184
SHISA3	1.074641849	0.473305394	0.00269111	0.004632527
BMPR1B	-1.002444884	-0.688728657	0.002758467	0.004738997
IGHV3-74	-1.030815688	1.349935984	0.002760903	0.004742818
PTCHD4	1.079560612	0.673274259	0.002811714	0.004821589
AL445183.2	1.153679513	-1.467016443	0.002835122	0.004857262
AL355512.1	1.177297309	0.33822636	0.002843601	0.004871044
IGSF11	1.385976982	-2.442843174	0.002851503	0.004883832
AC243562.2	1.146632392	-3.097453949	0.002907125	0.004972246
LINC01587	1.446117989	-1.377336394	0.002907712	0.004972869
NPPB	1.77113594	-2.715773752	0.002965978	0.005064765
MALRD1	1.151427486	-2.492043174	0.002996193	0.0051113
SPESP1	1.25508849	0.14111709	0.003009756	0.005132091
CNTN1	1.995850733	1.859213774	0.003076829	0.005239672
AC114947.1	1.302871067	-1.672800661	0.003131552	0.005324757
FBXO40	1.89145106	-1.675328616	0.003196362	0.005428768
APCDD1L	1.624079396	-2.04533877	0.003223078	0.005470821
AC005304.2	-1.065359107	-2.514562854	0.003297403	0.005588925
AL021940.1	1.612452323	-2.560784718	0.003315142	0.005616863
DDX43P3	1.416730824	-2.68091858	0.003320486	0.005625491
WNK2	1.272384742	3.656070003	0.003325718	0.005633929
CHI3L1	1.194381209	8.979268254	0.003404692	0.005757689
IGFL1	2.61404996	-2.825065925	0.003412272	0.0057692
LINC01370	-1.114844153	2.059932733	0.003460365	0.005846977
LINC02029	1.052514189	-0.527574148	0.003471833	0.005864582
AC005550.2	1.901007447	0.442840286	0.003540062	0.005974423
SLC34A2	1.860664427	3.950868661	0.003559632	0.006004732
LINC01182	1.251533635	-1.653535948	0.003617354	0.006096128
FRMD5	1.281301209	-2.63064898	0.003653474	0.006150976
AL161431.1	2.761143866	-1.507070538	0.003662835	0.006165344
MT1B	3.607822046	4.470177471	0.003679982	0.006190481
AC110741.1	1.900598729	-2.857819539	0.003945005	0.00660947

KLK10	-1.108560905	-1.09937035	0.003995767	0.006689509
C1orf116	1.10101978	2.208891986	0.004010447	0.00671057
LINC02160	-1.354898309	-2.276239655	0.004075583	0.006815484
PEG3	1.258009258	4.186074029	0.00413983	0.006914653
AP000424.1	1.002455491	-1.037273085	0.004185547	0.00698729
KRT87P	1.21898947	-0.935657855	0.004186044	0.00698729
AC108860.2	1.724676558	-2.453253359	0.004268439	0.007114595
TFAP2C	1.526416812	-0.984405818	0.004271781	0.007119634
ASCL1	-1.255038788	1.736236264	0.004336517	0.00722
TMEM51-AS1	1.011731958	-1.041282253	0.00454354	0.007542788
SFTPA2	1.731786985	-2.722261688	0.004616198	0.007654323
BX470209.2	1.563741635	-2.346717155	0.004629922	0.007674804
KCNS2	1.309016005	-2.943449207	0.004648938	0.007702331
RSPO2	1.547547116	-0.052431477	0.0046758	0.007743969
CHMP1B2P	1.637508993	-2.276970961	0.004684162	0.007756669
ZNF812P	1.069770458	-0.36118465	0.004699226	0.007779887
PADI1	1.931239806	2.039693469	0.004702314	0.007784425
TRPA1	1.268092325	-2.066805532	0.004703626	0.00778602
NTNG1	1.791536207	-2.885193083	0.004749138	0.007854965
FOXE1	1.893524024	-2.381032234	0.00483247	0.007980405
ILDR1	1.055598232	0.763013371	0.004834348	0.007982329
BEX1	1.687214863	2.460847763	0.004868181	0.00803582
LMX1B	1.60806805	-1.193173748	0.004892693	0.008072114
SOST	2.403907236	-2.780772274	0.004893115	0.008072214
PRSS3	1.335327045	3.34908174	0.00498456	0.008212778
AC007663.2	1.119137176	-2.145524171	0.0050085	0.008249185
EPHA6	1.765417529	-1.855323325	0.005054087	0.008315699
PDZD3	1.094832256	-2.034879327	0.005112069	0.008402449
SEZ6L	1.515153347	-0.642965096	0.005117168	0.008410212
HRNR	1.008754786	-2.820372192	0.005154746	0.008467034
SDR16C5	2.142192051	-1.921298722	0.005204588	0.008541968
AL049836.2	2.593044459	-2.449444265	0.005242938	0.008598803
LPO	1.298859172	-3.121536597	0.005328538	0.008728113
AC090152.1	1.104653393	0.264658015	0.005428867	0.008878803
SFRP5	-1.294443622	3.395246404	0.005469927	0.008941381
LYPD6	1.005852416	-0.190671527	0.005613371	0.009157128
PRAMEF2	2.016853734	-1.903495512	0.005750462	0.009365744
AC023154.1	1.216974744	-2.692240579	0.005800885	0.009439622
ATP10B	1.565764782	0.386294929	0.00581829	0.009466568
LCN15	1.887279379	-1.664488459	0.005951278	0.009666074
ART3	1.136046891	-2.002861868	0.006005298	0.009748153
DPP10-AS1	2.383768903	-0.772220484	0.006013001	0.009759334
LAMA1	1.322661197	1.10857932	0.00624003	0.010106455
TEX15	1.827898688	-1.571610304	0.006358376	0.010285473
IGLV4-60	-1.11673338	-0.975689255	0.006363033	0.010292263
C6orf132	1.074001222	1.738747864	0.006496493	0.010490692
A4GNT	1.636591147	-1.135721519	0.00691343	0.011120633
AL008723.1	1.048196791	-2.652964704	0.007009407	0.011266917
AC104031.1	1.013403145	-1.323211233	0.0071165	0.011427565
MAB21L1	1.254918378	-2.79534131	0.007246597	0.01162146
GJB4	1.480172843	-2.578405277	0.007566827	0.012107248
AC097652.1	1.461170725	-3.058467109	0.007762488	0.012399919
CNTN5	1.231099597	-1.971795512	0.008209289	0.013055856
PKP3	1.305145978	1.701108817	0.008384778	0.013320748
OSTN	1.333245225	-2.899373638	0.009104231	0.014375947
PCAT14	1.469904852	-2.477777299	0.009178594	0.014486213
KCNH1	1.002595361	-2.748505858	0.009456055	0.014898897
CALB1	1.092692879	-1.086432782	0.009556447	0.015042245

AC026765.2	1.067212803	0.119569108	0.009692109	0.01523863
WT1	1.286856528	-0.853185109	0.009846039	0.015454586
UMODL1	1.276102244	-1.580457738	0.009862183	0.015476669
LINC01133	1.086712835	-2.579213178	0.010111707	0.015833819
MYH4	2.068038466	5.779859307	0.010125698	0.015851291
FAM83A	1.073475536	0.183719943	0.010132396	0.015859557
LHX9	1.011081437	1.058234641	0.010196387	0.015955252
TCP10L2	1.187743656	-2.592538589	0.010340654	0.016170822
AP000439.3	1.842421832	-0.120811329	0.010362429	0.016201478
PAX9	1.170631728	-2.573181154	0.010678676	0.016666809
CYP2A7P2	1.363201992	-2.72107975	0.010733873	0.016742448
ALKAL1	-1.053447601	-2.210348374	0.010854494	0.016922331
AP003071.4	1.000588451	-0.989173437	0.011289528	0.017551638
RASSF10	1.485411669	-2.830465468	0.011364527	0.017665783
FAM19A5	1.098233256	2.6835271	0.011709718	0.018173333
DSG4	1.092050343	-2.935055385	0.012166178	0.018846458
PCYT1B	1.075530968	-1.508522324	0.012608833	0.019472849
FAM167A	1.021150314	0.953695945	0.012681256	0.019573889
AL358334.2	1.21416448	-2.51078528	0.01297081	0.019990486
CSAG3	1.586995704	-0.618653561	0.012989435	0.020016432
LINC00992	1.079656888	-1.067800084	0.013332782	0.020515852
WFDC2	1.179161242	2.733340805	0.013739723	0.021101401
IL13	1.403528124	-2.015131551	0.013790874	0.021174714
TFF1	1.846604075	1.317661783	0.013843002	0.021249806
PSPHP1	1.264087031	2.549945084	0.013859989	0.021272526
WSCD2	1.012438836	-2.25842846	0.01399596	0.021458107
LINC00524	1.549959913	-2.583427385	0.014044904	0.02152577
SFRP2	1.581737586	0.554411717	0.014110677	0.021622134
TUBBP5	1.122714414	-0.115600558	0.014219374	0.021778253
CRISP3	1.870011678	1.460188201	0.014227898	0.021789817
LGALS7B	1.385156904	-2.857658601	0.014310487	0.02190131
FAM153B	1.053744322	-0.80208939	0.014373915	0.021987855
AL645608.2	1.444835282	-2.775884155	0.014655644	0.022391262
AL049629.1	1.248057309	-2.336421763	0.014936609	0.022786298
C1orf140	1.148408566	-1.647180627	0.015508891	0.023606221
AC007608.1	1.186959976	-2.736595179	0.015599062	0.023732168
RBM46	1.050249981	-2.207093199	0.015612414	0.023750868
STMN2	1.260610586	1.283332968	0.015735556	0.023917062
EEF1DP5	1.875151562	-1.836364556	0.015747117	0.023933008
LINC01721	1.265492834	-2.524803817	0.015798935	0.024000351
NEXMIF	1.114838424	-2.517609494	0.01662422	0.025143094
AC006262.1	1.239793897	-2.868858515	0.016958147	0.025604868
LINC00940	1.072593105	-2.843485345	0.017247428	0.026010056
ANKRD22	1.001924226	1.652342273	0.017968668	0.027021235
AL365357.1	1.083670722	0.837244553	0.018155528	0.027283896
CACNG7	1.836100649	-3.158989359	0.018209963	0.027356514
ANGPT4	1.060214607	-1.417802771	0.018463165	0.027708989
TRIM40	1.070726714	-1.280971996	0.018573686	0.027862896
SORCS1	1.336393839	-1.178317714	0.019679	0.029358423
MS4A8	1.619115196	-0.815526237	0.01990102	0.029665911
ROBO2	1.003174481	0.922338111	0.019967169	0.02975857
PITPNM2-AS1	1.713389731	-0.808721808	0.022466111	0.033224007
CCK	1.60829114	-1.940106966	0.023090038	0.03408226
PPDPFL	1.868577073	-3.007949023	0.023531997	0.034683383
FGF9	1.124587745	-2.657039658	0.02440241	0.035885897
TMPRSS4	1.005689907	-0.334627151	0.025603769	0.037504702
H19	1.030842058	9.130672572	0.025682094	0.037614508
DCAF12L1	1.781156197	-2.88349549	0.025805845	0.037781132

HS6ST3	1.254170601	-2.651140533	0.026477038	0.038692686
PAK5	1.775664042	-0.146895127	0.027374125	0.039902023
FMR1NB	1.150111034	-2.863417442	0.027434834	0.039980097
AC087379.2	1.253166763	-0.368418764	0.027456246	0.040006089
ZFP57	1.082902799	-1.991082154	0.027789337	0.040444024
LINC00348	1.227883613	-1.169373223	0.02796815	0.040672518
MMP3	1.324082838	-1.458154983	0.027982559	0.040688182
CLCA1	2.410729227	-2.603818584	0.028513948	0.041407034
IGKV1D-13	-1.01691632	-1.141828571	0.028606158	0.041535547
AL121949.2	1.112760873	-2.748245003	0.029162435	0.042302074
KIF25-AS1	1.263311904	-2.37400018	0.031115576	0.044882092
UCA1	1.214771461	1.765212826	0.032268782	0.046422812
LIPM	1.110705875	-1.952053859	0.03267548	0.046962607
ATP12A	1.68958011	-1.352784451	0.033452813	0.047984275
KCNS1	1.003475519	-1.886408918	0.034073253	0.04876481

al samples.

Table S2. Differentially expressed miRNAs between hepatocellular carcinoma samples and normal samp

id	logFC	logCPM	PValue	FDR
hsa-miR-424-5p	-2.315714938	7.414355076	6.40E-87	5.25E-84
hsa-miR-139-3p	-1.918062986	5.028321644	7.33E-42	3.01E-39
hsa-miR-1258	-3.439255981	1.632560493	1.23E-37	3.36E-35
hsa-miR-21-5p	1.807758583	17.84339206	4.69E-36	9.62E-34
hsa-miR-93-5p	1.662610238	12.56503952	4.42E-35	7.24E-33
hsa-miR-589-5p	1.518025628	6.643475824	1.76E-32	2.41E-30
hsa-miR-10b-5p	3.561733274	14.23129048	7.50E-32	8.78E-30
hsa-miR-4746-5p	2.473524666	1.937821615	5.16E-29	5.29E-27
hsa-miR-10b-3p	3.507086444	3.118445211	3.20E-27	2.91E-25
hsa-miR-500a-3p	1.541741589	8.599323444	3.64E-27	2.98E-25
hsa-miR-224-5p	3.325447897	7.481451851	9.18E-27	6.84E-25
hsa-miR-424-3p	-1.359153958	2.037153894	1.12E-26	7.64E-25
hsa-miR-7706	1.986090527	1.455603669	2.09E-25	1.32E-23
hsa-miR-490-3p	-3.316506322	1.002453779	2.92E-25	1.71E-23
hsa-miR-183-5p	3.873938071	11.86545899	3.96E-24	2.03E-22
hsa-miR-532-5p	1.36802815	10.56578218	4.42E-24	2.13E-22
hsa-miR-452-5p	2.444198694	8.04648455	3.06E-22	1.39E-20
hsa-miR-96-5p	3.767523025	3.785121139	4.49E-22	1.94E-20
hsa-miR-501-3p	1.380790671	5.964839052	5.86E-22	2.40E-20
hsa-miR-103a-3p	1.048798025	14.80699357	7.33E-22	2.86E-20
hsa-miR-182-5p	3.371304562	13.25715038	9.25E-22	3.45E-20
hsa-miR-139-5p	-1.44580026	7.222515522	1.21E-21	4.32E-20
hsa-miR-450a-5p	-1.192160603	3.386537736	1.06E-20	3.62E-19
hsa-miR-221-3p	1.517457767	7.045260645	1.18E-20	3.88E-19
hsa-miR-1307-3p	1.101445565	10.61210118	2.77E-20	8.72E-19
hsa-miR-511-5p	-1.243529964	3.898953455	3.08E-20	9.35E-19
hsa-miR-660-5p	1.270937157	6.459782638	7.67E-20	2.17E-18
hsa-miR-1248	-1.912411134	-0.237798332	3.93E-19	9.77E-18
hsa-miR-1180-3p	1.657882174	4.578617057	3.93E-19	9.77E-18
hsa-miR-1269a	5.744145629	9.294102373	5.17E-19	1.25E-17
hsa-miR-101-3p	-1.06735723	14.53583404	6.24E-19	1.46E-17
hsa-miR-222-3p	1.433392321	4.94811969	6.66E-19	1.52E-17
hsa-miR-767-5p	9.061032031	4.696567526	1.41E-18	3.14E-17
hsa-miR-33b-5p	-1.607996904	3.236724084	1.55E-18	3.35E-17
hsa-miR-105-5p	9.000179542	5.272757416	1.94E-18	4.08E-17
hsa-miR-1301-3p	1.519212081	3.404428477	5.84E-18	1.11E-16
hsa-miR-877-5p	1.735869074	1.181338396	8.30E-18	1.52E-16
hsa-miR-452-3p	2.331659593	3.514266566	8.32E-18	1.52E-16
hsa-miR-144-3p	-1.83319133	4.222446312	1.16E-17	2.06E-16
hsa-miR-34a-5p	1.363171694	8.576777831	2.28E-17	3.89E-16
hsa-miR-188-5p	1.476018628	1.966035854	2.69E-17	4.50E-16
hsa-miR-542-5p	-1.160766568	1.291611015	3.02E-17	4.95E-16
hsa-miR-195-5p	-1.2795388	5.160103415	3.60E-17	5.78E-16
hsa-miR-1266-5p	1.894033382	2.873182024	5.69E-17	8.97E-16
hsa-miR-326	-1.281589575	2.365120963	1.32E-16	2.04E-15
hsa-miR-891a-5p	6.404354024	3.958619451	2.20E-16	3.35E-15
hsa-miR-335-5p	-1.186201577	3.544988437	3.18E-16	4.74E-15
hsa-miR-501-5p	1.435658675	2.016649308	3.69E-16	5.40E-15
hsa-miR-34c-5p	3.981863393	1.585777545	3.98E-16	5.73E-15
hsa-miR-6503-5p	-1.428969697	-0.42233971	6.42E-16	9.08E-15
hsa-miR-9-5p	3.536816791	10.27010249	7.38E-16	1.03E-14
hsa-let-7c-3p	-1.350287261	3.605797414	8.34E-16	1.14E-14
hsa-miR-130a-3p	-1.036505845	5.869670124	1.72E-15	2.27E-14
hsa-miR-589-3p	1.39103194	1.155940825	2.01E-15	2.62E-14
hsa-miR-30c-1-3p	-1.053105845	1.106272476	2.22E-15	2.84E-14
hsa-miR-190b-5p	3.069643796	0.656046624	2.60E-15	3.26E-14

hsa-miR-3200-3p	2.457239258	2.127983758	2.79E-15	3.42E-14
hsa-miR-3144-3p	3.63433053	0.088233156	3.14E-15	3.79E-14
hsa-miR-196b-5p	3.369565806	6.442209497	3.65E-15	4.34E-14
hsa-miR-30d-5p	1.2071081	13.91959646	5.00E-15	5.86E-14
hsa-miR-500b-5p	1.133082111	2.635609634	6.66E-15	7.70E-14
hsa-miR-500a-5p	1.132680939	2.635256327	6.91E-15	7.87E-14
hsa-miR-3677-3p	1.605196162	2.039417373	7.22E-15	8.12E-14
hsa-miR-671-5p	1.164725651	1.929483175	8.34E-15	9.24E-14
hsa-miR-4652-5p	6.904568474	1.928900761	9.62E-15	1.05E-13
hsa-miR-4661-5p	1.890560756	2.914598015	1.01E-14	1.09E-13
hsa-miR-421	1.379539577	1.877147933	1.30E-14	1.38E-13
hsa-miR-217-5p	4.195259407	11.48136004	1.36E-14	1.43E-13
hsa-miR-301a-3p	1.32863315	2.887646943	2.97E-14	3.08E-13
hsa-miR-18a-5p	1.610928955	4.440739064	3.93E-14	4.03E-13
hsa-miR-454-3p	1.046426428	2.951150247	5.01E-14	5.08E-13
hsa-miR-19a-3p	1.512914812	5.94875708	6.83E-14	6.83E-13
hsa-miR-1251-5p	5.023086832	1.216735423	6.98E-14	6.90E-13
hsa-miR-3127-5p	1.184384076	1.799776504	7.72E-14	7.53E-13
hsa-miR-500b-3p	1.303345311	1.287416503	9.90E-14	9.33E-13
hsa-miR-362-3p	1.075609443	2.143907012	1.03E-13	9.63E-13
hsa-miR-135a-5p	3.857805209	2.709352217	1.31E-13	1.19E-12
hsa-miR-3662	2.771488047	0.357225591	1.79E-13	1.62E-12
hsa-miR-1269b	6.526606368	6.668463132	3.72E-13	3.30E-12
hsa-let-7c-5p	-1.098665593	11.39613058	3.74E-13	3.30E-12
hsa-miR-103a-2-5p	1.377026167	0.94461435	3.84E-13	3.35E-12
hsa-miR-122b-5p	2.36151502	1.667460704	4.35E-13	3.76E-12
hsa-miR-224-3p	1.907861473	2.819248935	5.09E-13	4.30E-12
hsa-miR-3614-5p	-1.243476167	1.743324033	7.09E-13	5.93E-12
hsa-miR-20a-5p	1.248336334	9.526190788	7.59E-13	6.29E-12
hsa-miR-369-5p	-1.601263391	2.431395485	1.14E-12	9.20E-12
hsa-miR-184	4.686484701	4.262140302	1.96E-12	1.56E-11
hsa-miR-581	1.344128365	0.167919996	2.09E-12	1.65E-11
hsa-miR-3923	7.505022881	3.971641646	2.97E-12	2.29E-11
hsa-miR-17-5p	1.136574953	9.70355291	3.32E-12	2.54E-11
hsa-miR-4664-3p	2.410461788	-0.257869032	3.62E-12	2.75E-11
hsa-miR-34c-3p	2.812217599	0.974084845	4.48E-12	3.37E-11
hsa-miR-4326	1.567643475	2.550017921	4.71E-12	3.51E-11
hsa-miR-214-3p	-1.42753586	2.015843983	5.82E-12	4.30E-11
hsa-miR-552-5p	5.188071887	5.44156116	5.95E-12	4.36E-11
hsa-miR-122-3p	-1.083456212	7.651470288	7.10E-12	5.15E-11
hsa-miR-199b-3p	-1.311521279	10.15030516	8.45E-12	6.08E-11
hsa-miR-199a-3p	-1.308461594	10.1554523	9.13E-12	6.51E-11
hsa-miR-512-3p	7.613974976	4.61901994	1.55E-11	1.10E-10
hsa-miR-216b-5p	4.056934361	5.903327434	1.64E-11	1.15E-10
hsa-miR-767-3p	5.271334557	0.349714661	1.96E-11	1.36E-10
hsa-miR-552-3p	5.346802378	4.70476037	2.98E-11	2.05E-10
hsa-miR-372-3p	7.317169491	5.816901221	3.91E-11	2.67E-10
hsa-miR-216a-5p	3.624188652	5.623477527	4.47E-11	3.03E-10
hsa-miR-1270	2.134343038	1.359877628	4.74E-11	3.19E-10
hsa-miR-33b-3p	-1.338521537	0.234140916	5.32E-11	3.52E-10
hsa-miR-183-3p	2.761593157	-0.361881993	5.51E-11	3.62E-10
hsa-miR-6502-5p	-1.23375954	-0.482315381	6.51E-11	4.24E-10
hsa-miR-301b-3p	1.725925433	0.724532467	7.29E-11	4.71E-10
hsa-miR-10a-3p	-1.095042152	0.875866878	8.02E-11	5.03E-10
hsa-miR-196a-5p	4.554700127	4.093602103	8.03E-11	5.03E-10
hsa-miR-520a-3p	7.516141424	5.205609598	1.01E-10	6.26E-10
hsa-miR-519a-5p	7.341092902	4.952006975	1.03E-10	6.31E-10
hsa-miR-502-5p	1.243973263	1.034981882	1.03E-10	6.31E-10

hsa-miR-520c-3p	7.243349789	2.261766499	1.04E-10	6.31E-10
hsa-miR-520b-3p	7.242444503	2.260902882	1.05E-10	6.31E-10
hsa-miR-34a-3p	1.174174066	0.62551281	1.07E-10	6.40E-10
hsa-miR-2114-5p	3.202602487	1.563328681	1.31E-10	7.79E-10
hsa-miR-34b-3p	3.427508626	-0.040700851	1.57E-10	9.26E-10
hsa-miR-643	1.587681064	-0.295170091	1.85E-10	1.07E-09
hsa-miR-130b-3p	1.186668559	4.906754717	1.96E-10	1.13E-09
hsa-miR-130a-5p	-1.060155529	0.007101334	3.39E-10	1.88E-09
hsa-miR-765	1.765794302	-0.293725733	3.55E-10	1.94E-09
hsa-miR-939-5p	1.15406053	0.425690172	4.54E-10	2.43E-09
hsa-miR-1226-3p	1.609768767	0.832546999	5.11E-10	2.70E-09
hsa-miR-4742-3p	1.219310336	0.428277744	5.87E-10	3.08E-09
hsa-miR-219b-3p	1.45195492	-0.258783579	5.96E-10	3.11E-09
hsa-miR-520f-3p	7.538893986	2.986876517	9.24E-10	4.76E-09
hsa-miR-937-3p	1.586894695	1.700792093	1.40E-09	7.11E-09
hsa-miR-520h	6.791320811	2.14177493	1.45E-09	7.33E-09
hsa-miR-526b-5p	6.440230522	5.592708303	1.55E-09	7.79E-09
hsa-miR-519c-3p	6.790869711	2.265532253	1.60E-09	7.99E-09
hsa-miR-216a-3p	3.584024345	3.030818448	1.67E-09	8.28E-09
hsa-miR-520a-5p	7.017726451	3.344987937	1.76E-09	8.63E-09
hsa-miR-541-3p	3.411096145	1.362437142	1.95E-09	9.52E-09
hsa-miR-5003-3p	1.658758123	-0.353041118	2.39E-09	1.16E-08
hsa-miR-518b	6.538667983	3.874428087	2.50E-09	1.21E-08
hsa-miR-518f-5p	7.239262549	3.253733989	2.69E-09	1.29E-08
hsa-miR-520g-3p	6.982233599	3.023733235	3.07E-09	1.46E-08
hsa-miR-520e-3p	6.388028191	1.682970317	3.55E-09	1.68E-08
hsa-miR-548d-3p	1.730960959	-0.383882682	3.95E-09	1.86E-08
hsa-miR-516b-5p	6.687919808	3.463421177	4.63E-09	2.15E-08
hsa-miR-5010-3p	1.06494234	0.467871759	4.79E-09	2.21E-08
hsa-miR-7974	2.288309724	-0.424976365	5.36E-09	2.44E-08
hsa-miR-5586-5p	1.186525529	1.891308142	6.15E-09	2.77E-08
hsa-miR-516a-5p	5.926509816	4.028955218	7.39E-09	3.31E-08
hsa-miR-362-5p	1.00413046	4.736130014	7.66E-09	3.41E-08
hsa-miR-4791	-1.045762406	-0.369355035	8.51E-09	3.77E-08
hsa-miR-548y	3.860193694	-0.332035539	9.48E-09	4.13E-08
hsa-miR-1323	6.074791429	2.50713321	1.00E-08	4.35E-08
hsa-miR-454-5p	1.075126966	-0.042386407	1.10E-08	4.73E-08
hsa-miR-519d-3p	6.280800143	2.514844093	1.11E-08	4.76E-08
hsa-miR-338-3p	1.377165705	9.274413003	1.26E-08	5.35E-08
hsa-miR-200c-3p	2.666399193	8.4472835	1.33E-08	5.61E-08
hsa-miR-3682-3p	1.046255915	0.991214433	1.36E-08	5.71E-08
hsa-miR-376a-2-5p	-1.474472089	-0.405626337	1.39E-08	5.79E-08
hsa-miR-9-3p	2.665460901	1.639408239	1.79E-08	7.36E-08
hsa-miR-6783-3p	1.900848298	-0.671109287	2.15E-08	8.78E-08
hsa-miR-515-5p	6.013132692	1.597490166	2.44E-08	9.89E-08
hsa-miR-519b-3p	5.864403411	0.966980275	2.72E-08	1.09E-07
hsa-miR-4536-3p	-1.121924643	-0.65977642	2.90E-08	1.15E-07
hsa-miR-3677-5p	1.284976979	0.574296205	2.90E-08	1.15E-07
hsa-miR-525-5p	6.269724332	3.227341042	2.98E-08	1.18E-07
hsa-miR-512-5p	6.106534351	1.532341351	3.04E-08	1.19E-07
hsa-miR-520d-3p	5.904450123	1.745527936	3.44E-08	1.33E-07
hsa-miR-760	1.626696753	-0.046533168	3.44E-08	1.33E-07
hsa-miR-518f-3p	5.700764857	1.226137227	3.74E-08	1.44E-07
hsa-miR-498-5p	5.994932946	1.469163259	4.51E-08	1.73E-07
hsa-miR-431-3p	2.109378198	4.541940167	4.53E-08	1.73E-07
hsa-miR-137-3p	3.941549782	0.495595407	4.55E-08	1.73E-07
hsa-miR-2114-3p	2.790448723	0.430612424	5.10E-08	1.93E-07
hsa-miR-1292-5p	1.196373525	-0.187158197	6.13E-08	2.29E-07

hsa-miR-615-3p	3.89746622	-0.040639046	6.19E-08	2.31E-07
hsa-miR-1276	1.609854338	-0.462145665	7.06E-08	2.61E-07
hsa-miR-466	5.229687037	0.869401927	7.13E-08	2.62E-07
hsa-miR-517-5p	6.663367276	3.047269209	7.18E-08	2.63E-07
hsa-miR-522-3p	5.878398919	1.655735808	7.56E-08	2.75E-07
hsa-miR-518c-5p	5.895496678	3.430341126	8.05E-08	2.91E-07
hsa-miR-20b-5p	1.3777977	4.300458216	8.38E-08	3.01E-07
hsa-miR-6514-5p	1.424729242	-0.555330993	8.52E-08	3.05E-07
hsa-miR-548x-3p	3.579647364	-0.505796793	8.81E-08	3.14E-07
hsa-miR-524-5p	5.002139273	0.487077096	1.14E-07	4.00E-07
hsa-miR-6844	2.09262335	-0.57694252	1.15E-07	4.00E-07
hsa-miR-653-5p	1.576262647	3.812615373	1.26E-07	4.36E-07
hsa-miR-1229-3p	1.409849039	0.088526109	1.35E-07	4.65E-07
hsa-miR-509-3p	2.789319038	2.927332295	1.37E-07	4.69E-07
hsa-miR-146b-5p	1.160793083	10.67769925	1.41E-07	4.83E-07
hsa-miR-518e-3p	5.605976314	2.237580851	1.56E-07	5.31E-07
hsa-miR-518a-5p	5.518171429	1.331957531	1.58E-07	5.34E-07
hsa-miR-527	5.44739177	1.272255002	1.81E-07	6.07E-07
hsa-miR-5187-5p	1.117966635	0.211272125	2.14E-07	7.14E-07
hsa-miR-431-5p	2.169733895	2.875289701	2.44E-07	8.05E-07
hsa-miR-518a-3p	5.464166037	1.777373696	2.63E-07	8.64E-07
hsa-miR-508-3p	2.473662313	4.571553543	3.02E-07	9.68E-07
hsa-miR-1295a	2.170627979	1.510231487	3.19E-07	1.02E-06
hsa-miR-523-3p	5.332491757	1.538576919	3.29E-07	1.05E-06
hsa-miR-18a-3p	1.19256297	1.14368575	3.30E-07	1.05E-06
hsa-miR-517c-3p	5.169966847	0.934493603	3.47E-07	1.09E-06
hsa-miR-1283	4.889439485	0.753096585	3.69E-07	1.16E-06
hsa-miR-942-3p	1.309667924	-0.213481814	3.90E-07	1.22E-06
hsa-miR-3189-3p	2.370899679	-0.387548573	3.92E-07	1.22E-06
hsa-miR-514a-3p	2.570601869	3.786268565	4.22E-07	1.30E-06
hsa-miR-525-3p	5.029018248	0.877925138	4.47E-07	1.36E-06
hsa-miR-92a-1-5p	1.039330534	2.197814681	5.01E-07	1.51E-06
hsa-miR-548f-3p	4.418411287	0.146686257	5.13E-07	1.54E-06
hsa-miR-519a-3p	5.138347208	1.724134166	5.39E-07	1.61E-06
hsa-miR-3691-5p	1.251409595	-0.286781902	6.19E-07	1.84E-06
hsa-miR-4664-5p	1.452649223	-0.307422393	6.62E-07	1.96E-06
hsa-miR-518c-3p	5.221745303	1.981999701	7.00E-07	2.06E-06
hsa-miR-6716-3p	1.275694965	-0.375807521	7.05E-07	2.07E-06
hsa-miR-154-3p	2.150103843	2.709541589	7.86E-07	2.27E-06
hsa-miR-141-3p	2.403761063	5.255506089	8.04E-07	2.31E-06
hsa-miR-515-3p	5.353453772	1.4513257	8.21E-07	2.36E-06
hsa-miR-373-3p	5.000697193	2.569551192	8.90E-07	2.54E-06
hsa-miR-526b-3p	4.331144332	-0.101562202	1.06E-06	3.01E-06
hsa-miR-6715b-3p	2.161921034	2.312979101	1.11E-06	3.14E-06
hsa-miR-337-5p	-1.346687613	-0.498731491	1.27E-06	3.57E-06
hsa-miR-3117-3p	1.846402875	-0.182527916	1.44E-06	4.02E-06
hsa-miR-7705	1.053205079	0.256617925	1.68E-06	4.64E-06
hsa-miR-205-5p	4.076654197	3.314129002	1.68E-06	4.64E-06
hsa-miR-25-5p	1.356615128	-0.545505392	1.85E-06	5.08E-06
hsa-miR-521	3.888617104	-0.369397442	1.89E-06	5.17E-06
hsa-miR-6516-5p	1.266143182	-0.374447342	2.05E-06	5.59E-06
hsa-miR-5589-5p	-1.322233398	3.653734476	2.18E-06	5.93E-06
hsa-miR-493-5p	1.586901766	4.331267292	2.57E-06	6.94E-06
hsa-miR-92b-3p	1.045801651	4.231047628	2.85E-06	7.67E-06
hsa-miR-371a-5p	5.261472667	1.095146825	3.34E-06	8.89E-06
hsa-miR-3680-3p	1.372709951	-0.554920984	3.57E-06	9.47E-06
hsa-miR-577	2.288289828	0.558935228	3.65E-06	9.66E-06
hsa-miR-34b-5p	2.395859931	-0.34748982	4.34E-06	1.15E-05

hsa-miR-203b-3p	3.588270231	2.185404251	4.37E-06	1.15E-05
hsa-miR-6788-3p	1.170450327	-0.133574991	5.04E-06	1.32E-05
hsa-miR-892a	3.731614244	-0.432946958	5.65E-06	1.47E-05
hsa-miR-524-3p	3.706514413	-0.447634071	7.63E-06	1.97E-05
hsa-miR-376b-5p	-1.088267144	0.329120659	8.34E-06	2.14E-05
hsa-miR-517b-3p	4.262345403	3.366161696	8.76E-06	2.23E-05
hsa-miR-517a-3p	4.262177745	3.366001063	8.76E-06	2.23E-05
hsa-miR-5589-3p	-1.214075769	3.156151417	8.89E-06	2.26E-05
hsa-miR-410-3p	1.443630155	5.056970049	8.92E-06	2.26E-05
hsa-miR-520d-5p	3.991487261	-0.224757416	8.97E-06	2.26E-05
hsa-miR-301b-5p	1.575886681	-0.278189136	9.98E-06	2.50E-05
hsa-miR-412-5p	1.653487457	4.013436008	1.00E-05	2.51E-05
hsa-miR-6720-3p	2.295344184	-0.67619842	1.01E-05	2.53E-05
hsa-miR-6516-3p	1.169795098	-0.617904973	1.04E-05	2.59E-05
hsa-miR-6734-5p	1.184235043	-0.676628386	1.10E-05	2.72E-05
hsa-miR-561-5p	2.49426459	-0.307326147	1.12E-05	2.76E-05
hsa-miR-432-5p	1.548693048	4.564179199	1.52E-05	3.68E-05
hsa-miR-1224-5p	2.087227332	0.21975036	1.60E-05	3.86E-05
hsa-miR-485-3p	1.55927595	3.715534059	1.79E-05	4.30E-05
hsa-miR-371a-3p	4.792021802	1.046284259	1.80E-05	4.32E-05
hsa-miR-944	1.426232719	1.434314429	1.93E-05	4.57E-05
hsa-miR-3934-5p	1.38842242	-0.543398658	2.81E-05	6.63E-05
hsa-miR-509-3-5p	3.472901691	0.251899212	3.50E-05	8.18E-05
hsa-miR-3922-3p	1.01419497	-0.027703085	3.56E-05	8.27E-05
hsa-miR-7-5p	1.020840317	0.241447579	3.81E-05	8.75E-05
hsa-miR-496	1.407491235	1.991873999	4.84E-05	0.000110657
hsa-miR-520g-5p	3.490877812	-0.444260615	5.08E-05	0.000115346
hsa-miR-409-5p	1.232431735	4.024125173	5.20E-05	0.000117714
hsa-miR-147b-3p	1.493970583	-0.326938767	5.36E-05	0.000120818
hsa-miR-376c-5p	-1.008816714	0.409051499	5.64E-05	0.000126681
hsa-miR-382-3p	1.290325966	2.176962363	8.37E-05	0.000186056
hsa-miR-539-5p	1.314987414	3.760605392	0.00010071	0.000220218
hsa-miR-3131	1.957950666	-0.219414732	0.000101521	0.000221403
hsa-miR-31-5p	2.169776094	0.500722609	0.000114796	0.000249028
hsa-miR-499a-5p	1.868749052	1.508486596	0.000130938	0.00028107
hsa-miR-3660	2.78202356	-0.747090699	0.000148572	0.000315619
hsa-miR-141-5p	1.777415366	4.053589463	0.00015186	0.000321771
hsa-miR-5683	1.150100325	1.156786861	0.000218148	0.00045867
hsa-miR-129-5p	1.36725976	1.11834664	0.000262487	0.000547682
hsa-miR-889-3p	1.185834882	5.705693097	0.000271628	0.000563445
hsa-miR-204-5p	1.11346818	5.80935209	0.000272103	0.000563445
hsa-miR-653-3p	1.482283693	-0.135654614	0.000278415	0.000575064
hsa-miR-766-5p	1.009129609	-0.623711303	0.000309297	0.000632477
hsa-miR-551b-3p	1.608464194	3.063061896	0.000459137	0.000925043
hsa-miR-539-3p	1.385375736	1.465755547	0.000513632	0.001024765
hsa-miR-20b-3p	1.271641585	0.165301338	0.000595625	0.001176897
hsa-miR-1185-1-3p	1.512377636	-0.369927132	0.000686	0.001345742
hsa-miR-483-3p	1.864910932	7.899011155	0.000736673	0.001438266
hsa-miR-135b-5p	1.563895958	1.822679341	0.000760325	0.001480918
hsa-miR-4739	1.194013664	-0.135728858	0.000764772	0.00148605
hsa-miR-206	1.530962161	0.160385803	0.000797612	0.001542551
hsa-miR-199b-5p	1.213822648	4.654634761	0.000895993	0.001724682
hsa-miR-487a-3p	1.415772833	0.843793233	0.001009639	0.001929846
hsa-miR-499a-3p	1.792975932	0.722923543	0.001309947	0.002450224
hsa-miR-4784	-1.137826847	-0.321894509	0.001311766	0.002450224
hsa-miR-380-3p	1.468380508	0.218418501	0.001536483	0.002818605
hsa-miR-506-3p	2.072993069	-0.340691516	0.001577313	0.002887046
hsa-miR-346	1.529266052	-0.382230346	0.001711972	0.003126542

hsa-miR-483-5p	1.618509959	6.737162071	0.002078502	0.003729479
hsa-miR-668-3p	1.53060931	-0.640590908	0.002301439	0.004093667
hsa-miR-708-5p	1.021542634	1.785945723	0.002567161	0.004507649
hsa-miR-508-5p	1.991729259	0.165699044	0.002775224	0.004862572
hsa-miR-380-5p	1.162052018	1.177449148	0.003222386	0.005562855
hsa-miR-3681-5p	1.605495719	-0.098405234	0.004509185	0.007655344
hsa-miR-376a-3p	1.116162518	1.687583448	0.007279568	0.011986437
hsa-miR-802	1.696783777	1.529874674	0.01062045	0.017211007
hsa-miR-202-5p	1.270697399	-0.185620244	0.010740545	0.017324774
hsa-miR-211-5p	1.077149665	0.647949346	0.019160867	0.029533668
hsa-miR-1911-5p	1.78840199	0.924638825	0.022262968	0.03412268
hsa-miR-200c-5p	1.499437876	-0.033869131	0.023010543	0.035137142

ples.

Table S3. Survival information of differentially expressed miRNA in train group

id	futime	fustat	hsa-miR-424-5p	hsa-miR-139-3p	hsa-miR-1258
TCGA-5R-AA1D	1.230136986	0	9.508539136	7.691428022	1.415339214
TCGA-2Y-A9GV	6.936986301	1	8.948636434	7.181073945	4.125304723
TCGA-G3-A25Y	1.238356164	1	9.587966743	6.649037067	2.352950889
TCGA-FV-A3I1	0.676712329	1	9.20413251	5.381603011	0.859446017
TCGA-DD-A118	9.416438356	0	8.602372411	7.138478691	2.400863138
TCGA-DD-AAC9	0.950684932	0	8.894850601	7.05123077	0.03341657
TCGA-DD-AADU	1.517808219	0	8.166806818	7.023583071	0.228151848
TCGA-CC-A3MA	0.830136986	1	8.968961828	5.773376668	1.38891995
TCGA-DD-AACS	4.942465753	0	7.177416999	6.252679901	0.763420189
TCGA-G3-AAV6	0.178082192	1	7.838759653	4.997962159	1.2504032
TCGA-K7-A5RF	1.728767123	0	10.12410334	7.365444788	5.207347631
TCGA-ED-A4XI	2.243835616	0	10.50153444	6.290208082	3.473497586
TCGA-DD-AAW3	4.473972603	0	8.271199157	8.016325627	0
TCGA-CC-5264	0.279452055	1	8.30166083	6.192271607	3.340915642
TCGA-MI-A75C	0.797260274	0	7.702619771	2.952676258	0
TCGA-DD-AACA	6.304109589	0	8.616831185	7.405402585	1.286373915
TCGA-UB-A7MD	0.142465753	1	9.174536663	7.392628645	0
TCGA-CC-A5UE	0.745205479	1	6.731474886	3.365865599	0
TCGA-RC-A7S9	1.753424658	0	7.382774975	5.267713046	3.78357539
TCGA-5R-AAAM	0.126027397	1	10.52049921	8.535728773	4.114772483
TCGA-3K-AAZ8	1.084931507	0	8.17955773	7.181457393	0.400060789
TCGA-DD-A4NA	2.761643836	0	8.049464052	5.701362172	0.11763996
TCGA-DD-AAE6	0.38630137	0	7.583465637	5.538852282	0
TCGA-ZP-A9D0	2.989041096	0	9.191930856	7.311744055	0.202332748
TCGA-DD-AAVS	4.994520548	0	8.085436396	6.550756892	2.130210393
TCGA-2Y-A9H5	1.520547945	1	10.11356802	6.504818162	1.905830411
TCGA-ED-A8O6	0.153424658	1	7.608457885	4.058963497	0.010292929
TCGA-G3-AAV1	0.983561644	1	8.877430391	7.371616148	0.187538477
TCGA-DD-AAED	2.090410959	0	7.504399734	4.533656582	2.239227407
TCGA-G3-A3CG	1.843835616	0	9.04201839	6.806774336	2.492691355
TCGA-EP-A3RK	0.994520548	0	9.036467192	5.99723472	1.429216227
TCGA-DD-AADJ	2.920547945	0	6.757944234	6.679573321	4.077368488
TCGA-ED-A8O5	1.112328767	0	8.618167411	7.433768456	0.059867061
TCGA-2Y-A9H6	0.978082192	0	9.100944447	6.815138716	0.673044292
TCGA-DD-A3A0	2.150684932	1	7.821608015	6.075238807	3.306653267
TCGA-DD-A1EJ	2.753424658	1	9.023538812	2.898599437	3.281459003
TCGA-G3-A6UC	1.838356164	0	6.488726365	7.489993248	0.09977175
TCGA-PD-A5DF	1.750684932	1	9.124581418	4.095088834	1.06182241
TCGA-2Y-A9H3	4.153424658	0	8.632352501	7.527245859	1.166072485
TCGA-UB-A7MA	2.323287671	0	10.37442157	5.000170644	0.024737137
TCGA-MI-A75G	1.912328767	0	8.092014056	5.727731552	2.946932943
TCGA-BD-A2L6	3.734246575	0	8.096878199	7.354317725	1.02569718
TCGA-DD-AADL	1.742465753	0	8.313642825	7.050773466	1.43965192
TCGA-G3-A7M8	1.178082192	0	8.698674309	8.471275682	0.178849905
TCGA-DD-A1EL	1.136986301	1	8.940671902	6.517901813	1.516234097
TCGA-G3-A25Z	1.794520548	0	9.269339834	7.551951891	3.6789035
TCGA-DD-A73E	0.120547945	0	7.618218108	6.901613935	0.256072613
TCGA-DD-AACO	5.139726027	0	8.639934088	6.375551939	2.468168
TCGA-2Y-A9HA	0.098630137	1	8.931219718	5.477829915	0
TCGA-WX-AA46	2.071232877	0	11.00895772	8.953269809	4.792773972
TCGA-G3-A7M7	0.989041096	0	9.230892212	6.641415283	3.027668812
TCGA-BC-A10X	2.109589041	1	12.34809414	8.983690375	5.203719224
TCGA-DD-AAD1	1.545205479	0	9.018601367	7.033092808	1.362889313
TCGA-DD-A4NB	2.709589041	0	10.41083481	4.460288783	2.200029638
TCGA-G3-A3CK	1.602739726	0	8.013557037	6.665134825	0.029873328
TCGA-G3-A3CI	0.493150685	0	9.225979345	8.274650891	0

TCGA-DD-AAEE	2.219178082	0	8.594427843	7.602640591	0.104421376
TCGA-CC-5261	0.265753425	1	10.22184376	6.449995904	0
TCGA-CC-A3MB	0.863013699	1	9.289510186	7.137019973	1.614091245
TCGA-RC-A7SB	1.610958904	0	7.600552896	6.67005441	4.261422648
TCGA-ZP-A9D2	2.095890411	1	10.18441774	6.733846218	0.529118532
TCGA-DD-AADD	3.37260274	0	7.116887508	5.540142608	0.08389656
TCGA-2Y-A9GY	2.073972603	1	8.53889729	4.285543879	0.006484722
TCGA-DD-AACL	0.293150685	1	10.0812811	4.211382199	1.119433037
TCGA-K7-A5RG	1.421917808	0	8.80296616	5.654701918	1.963492855
TCGA-HP-A5N0	2.060273973	1	9.8477064	7.368526718	4.249941624
TCGA-ES-A2HS	1.884931507	1	8.137924729	6.819181604	6.157599274
TCGA-DD-A4NE	1.808219178	1	8.865670125	5.128900839	2.449427953
TCGA-DD-A4NL	4.687671233	0	8.550911247	7.475438998	5.110557055
TCGA-O8-A75V	1.473972603	0	8.806196542	6.958345684	0
TCGA-XR-A8TD	2.821917808	0	9.627686943	6.631862616	1.912256073
TCGA-DD-AADO	1.24109589	0	7.422969357	3.008699957	0.03873945
TCGA-2Y-A9HB	0.712328767	0	8.484646297	6.839853418	0.041961577
TCGA-CC-A8HU	0.942465753	1	8.122613099	5.702825935	2.88690369
TCGA-G3-A5SJ	1.912328767	0	8.369862026	4.224499871	0.818000341
TCGA-2Y-A9GZ	2.323287671	1	8.648296309	7.987302305	3.413954955
TCGA-CC-5263	0.353424658	1	9.296191236	5.143027359	1.121333481
TCGA-UB-A7MB	1.646575342	0	9.00138845	6.515188024	0.16806202
TCGA-DD-AACN	3.567123288	0	9.464080884	6.902349849	2.293053469
TCGA-DD-A3A7	1.147945205	1	8.134972557	4.73473993	0.049701325
TCGA-DD-AAVX	4.706849315	0	8.172389343	8.135032808	0.150070129
TCGA-DD-AAVU	6.032876712	0	8.296245481	5.099679154	0
TCGA-UB-A7ME	1.331506849	0	8.72160614	6.405178446	1.277305601
TCGA-DD-AACG	1.284931507	1	7.895878846	4.197487134	2.682940226
TCGA-G3-A25X	4.873972603	0	10.22246786	5.589241655	0.918532366
TCGA-DD-AACW	3.901369863	0	6.203256804	6.558626997	3.599777315
TCGA-KR-A7K2	2.271232877	0	9.648202789	6.306157379	0.109276395
TCGA-ED-A5KG	2.339726027	0	7.208715611	3.988665119	0.653131307
TCGA-UB-A7MC	1.369863014	0	7.431744584	7.42707498	0.286311156
TCGA-EP-A2KA	1.717808219	1	8.333325637	4.956552774	2.587017905
TCGA-DD-A4NG	2.197260274	1	8.884745739	5.323279689	2.448683644
TCGA-GJ-A6C0	0.084931507	1	7.833940535	3.820006704	5.369346008
TCGA-DD-A3A1	0.638356164	1	8.292648067	2.810166235	3.715333789
TCGA-DD-A1EK	1.528767123	1	8.517914352	4.163358672	1.049090458
TCGA-KR-A7K7	2.605479452	0	8.165071403	4.446675729	0.7032128
TCGA-DD-A1EH	4.095890411	0	9.09056181	6.378096725	0
TCGA-DD-AAW0	5.520547945	0	10.57869261	7.070412276	0.855421309
TCGA-GJ-A9DB	0.183561644	1	8.279911151	6.056760398	0.081263733
TCGA-5R-AA1C	1.424657534	0	8.198926842	6.951243006	0.970099065
TCGA-FV-A23B	5.073972603	1	8.921847586	6.283368667	3.271095607
TCGA-CC-5259	0.684931507	0	7.003250927	3.615436127	2.307247184
TCGA-DD-AA3A	1.123287671	1	8.160103995	5.537286182	0
TCGA-RG-A7D4	3.008219178	0	7.544135016	5.230110129	0
TCGA-DD-A3A5	8.561643836	1	8.306763576	6.760276561	4.939473317
TCGA-BC-A3KG	1.863013699	0	8.305318671	5.795677902	1.293132065
TCGA-DD-AACM	4.846575342	0	9.49439626	5.594951643	0.392699912
TCGA-BC-A10W	0.249315068	1	9.830807215	5.994280022	0.010724722
TCGA-DD-A3A6	8.926027397	1	10.58411021	8.507307535	0
TCGA-DD-AACC	4.616438356	1	9.474674045	7.567800802	3.817250847
TCGA-CC-A7II	1.093150685	0	8.32419721	5.101268496	2.166717976
TCGA-CC-5262	0.282191781	1	10.0633645	7.182828445	0.649237
TCGA-DD-A3A9	2.550684932	1	8.764164845	5.752555857	0.013604435
TCGA-BC-4072	4.082191781	1	10.38189929	5.555779233	0.022288377
TCGA-ZS-A9CD	3.797260274	1	8.678592968	6.402554062	0.792894723

TCGA-BC-A69I	1.060273973	0	7.825656012	7.51968935	0.245242734
TCGA-DD-A114	3.147945205	1	9.864814866	7.77784427	3.286370551
TCGA-DD-AAD8	3.339726027	0	8.5660618	5.433674183	0
TCGA-GJ-A3OU	2.408219178	0	9.170060981	6.187876747	2.589860736
TCGA-ED-A7XP	1.095890411	0	8.833967251	7.293240495	0.02632058
TCGA-CC-A9FW	0.679452055	0	8.104321693	3.272977059	2.417331298
TCGA-2Y-A9GS	1.983561644	1	8.945588879	6.247287187	0.624711849
TCGA-DD-A39W	2.265753425	1	7.592582215	6.134860755	0
TCGA-G3-A5SM	1.424657534	0	9.750310871	5.676035598	4.02952993
TCGA-ED-A66Y	0.810958904	1	6.852468265	3.049535714	1.170739941
TCGA-BC-A69H	1.216438356	0	6.65429093	4.143918236	0.062775982
TCGA-DD-A73F	2.97260274	0	8.308352054	4.854017631	3.360493391
TCGA-CC-A8HV	0.764383562	1	8.236393179	4.967198706	1.932493027
TCGA-4R-AA8I	0.717808219	1	9.233702797	6.116872507	0.008538305
TCGA-ZS-A9CE	3.4	0	8.372852294	7.318098798	0.025342748
TCGA-G3-A5SK	2.038356164	0	8.929990698	6.859059184	3.35264955
TCGA-DD-AADC	1.164383562	1	9.60271045	6.382118912	2.130837254
TCGA-BC-A10Y	1.947945205	1	10.01368696	7.430634341	0.042043701
TCGA-BC-A10Q	3.109589041	1	9.518931924	5.775789702	0
TCGA-RC-A7SF	1.58630137	0	7.710616991	6.498715524	1.558467839
TCGA-G3-AAV2	1.019178082	0	7.771691206	7.002039498	0.061500863
TCGA-CC-A1HT	0.276712329	1	9.728560352	6.129440128	0.608794222
TCGA-DD-AAE7	1.764383562	0	8.8858183	7.362039563	5.403723094
TCGA-G3-A5SI	2.104109589	1	7.923258612	5.975425142	1.752891905
TCGA-XR-A8TC	3.668493151	0	6.891194268	6.621487514	1.498371425
TCGA-ED-A627	1.15890411	0	10.5267487	7.466896264	2.678818828
TCGA-DD-A1EC	1.649315068	0	9.26569462	6.443673803	2.724060909
TCGA-YA-A8S7	1.128767123	1	8.568636297	5.325455191	1.396488832
TCGA-DD-AADV	1.57260274	0	8.817078812	7.104504304	2.327344544
TCGA-DD-A3A4	1.676712329	1	7.919908105	7.334921173	0.273201808
TCGA-G3-A7M5	1.224657534	0	7.951732801	7.747568865	3.372142965
TCGA-DD-A39Y	0.468493151	1	9.00859779	5.189517544	0.750710889
TCGA-DD-AACF	1	1	8.670388701	6.627715382	1.6514909
TCGA-EP-A2KB	1.632876712	1	7.25319814	6.435229915	3.509950788
TCGA-DD-AAE2	1.747945205	0	8.755587654	8.338375369	0
TCGA-DD-AACZ	0.468493151	1	9.491054839	4.49404607	1.065430403
TCGA-DD-A116	4.443835616	1	10.24952147	7.65101385	0.055158321
TCGA-DD-AACQ	1.183561644	1	8.531800919	5.016590351	1.138366509
TCGA-2Y-A9H2	4.742465753	0	8.874889956	5.548734347	2.105763361
TCGA-DD-AADW	1.608219178	0	8.721640938	5.816245217	1.1529411
TCGA-DD-A11D	4.273972603	1	10.33220312	7.487736044	2.570026957
TCGA-MR-A520	0.62739726	0	8.833473822	7.366850104	2.001385845
TCGA-DD-A115	6.964383562	1	10.91416929	6.889716863	0.900111308
TCGA-DD-AACY	3.97260274	0	7.752379935	7.332440243	3.417870926
TCGA-DD-A73G	9.528767123	0	9.09661398	5.925022792	0.953951421
TCGA-CC-5260	0.238356164	1	9.855666288	6.161926013	0.600415654
TCGA-DD-AACI	4.432876712	0	8.803854714	5.661592386	1.271553605
TCGA-DD-A4NQ	1.021917808	1	8.678879189	3.931336512	2.898550432
TCGA-FV-A2QQ	1.997260274	0	8.989225893	6.843305772	1.642516108
TCGA-CC-A5UC	0.950684932	1	10.2287993	5.609950759	1.992060772
TCGA-DD-A4NH	2.512328767	0	9.232920722	3.784911484	0.813537342
TCGA-DD-AAVQ	7.473972603	0	9.593543456	6.61644334	0
TCGA-DD-AAVY	5.397260274	0	6.540502078	7.446948277	0.105732819
TCGA-UB-A7MF	0.58630137	1	8.150000768	3.721523513	5.04710645
TCGA-ED-A459	2.493150685	0	9.092519211	3.974687118	3.280724243
TCGA-2Y-A9GT	4.449315068	1	8.473604213	7.692797985	5.33156815
TCGA-2Y-A9H8	1.734246575	1	7.93725652	4.722280652	2.692407282
TCGA-DD-AAEI	4.194520548	0	7.779865419	6.448013139	0.11704542

hsa-miR-21-5p	hsa-miR-93-5p	hsa-miR-589-5p	hsa-miR-10b-5p	hsa-miR-4746-5p
20.60763773	13.13187235	7.459599462	15.50362823	1.418334712
19.52518808	13.82307799	8.484667452	16.5513193	3.208155592
18.8895184	14.43529359	8.299701676	17.44978644	5.203528313
20.48230647	14.8777952	8.222927972	13.61545008	3.364842724
20.02350107	14.22575759	8.723942952	18.70082134	3.409364729
21.1791041	14.20032767	8.3890444	12.81247468	2.159913107
20.62376238	14.70536572	8.671609147	14.84100061	3.580906405
20.33292671	15.74202526	7.826169791	11.19068521	4.863061763
19.72490597	15.47245054	10.25816653	11.93358984	5.384351718
18.67210264	15.88462163	8.854035345	18.99885368	6.170974332
18.26534165	13.82807074	7.240004567	15.82802532	0.132488497
19.67605847	14.58416988	7.616538266	15.80094445	1.356170411
19.33595789	15.490015	9.903795032	15.22059337	3.383388421
20.26495001	15.68157307	8.724373418	14.58134018	6.076429523
21.03406057	15.02469495	9.262778035	15.98920375	1.957621556
20.55607648	15.59766283	9.346072135	14.51202866	4.507706739
20.02863351	14.23487951	8.491887417	16.83416978	3.752349788
20.20030181	15.51472881	9.053858493	16.61613211	3.74916121
19.09299759	14.52007648	7.437871849	8.848502359	5.625078089
20.0538381	14.20096171	7.994696904	17.42118108	3.403162346
20.18516164	14.51762755	8.093581958	16.20729013	3.04036133
21.1735756	15.69031389	8.945971322	16.13675424	3.373880469
17.87476801	14.11887358	8.596267657	17.49802934	4.065517639
19.39572768	13.57450641	7.925653633	12.84173051	2.389001376
19.7400463	14.80298336	9.312714622	13.49171892	4.584045257
19.8239165	13.92163084	8.910772962	12.01752742	2.364848203
18.81527996	14.25448413	7.82314444	18.17864971	3.758334
21.20054315	14.53178986	9.369943467	18.00605094	3.14734339
20.32360851	15.60716642	9.208651361	13.52405818	2.576989557
19.92072929	14.69868794	8.556754827	13.37967418	2.987829153
20.52821464	14.6176226	9.045020734	16.88342232	4.787211207
18.6842149	15.73217947	8.910199666	18.68384693	3.985669337
18.49638174	15.11771533	8.466596672	17.6478099	4.1259341
20.19387566	14.05332922	7.487002548	13.8419018	3.16161143
19.43663374	15.15368492	9.7371481	19.4414285	3.675065041
19.19615374	14.74967321	7.305075925	14.56809072	4.3611259
18.82723059	14.92500856	9.765474837	16.14104145	2.451921983
20.32682696	15.23960358	8.607183948	14.23227175	4.264515331
20.80614753	14.54426786	8.490425079	18.54149469	3.410732239
20.9393176	14.66553418	7.873193239	13.07410006	4.894709447
19.87229495	14.62602048	9.098396904	17.07321948	3.090264906
17.53141585	14.45930424	8.865002254	16.17804682	3.846563561
19.49278241	15.21201501	9.411602062	14.12654909	4.080121671
18.95879831	14.23330081	8.577038356	17.49262594	4.00321196
19.83180963	14.12281405	9.568765291	18.16167355	5.322365071
20.80938105	13.41257183	8.561821637	12.18546973	4.387481996
19.29223389	15.48759412	9.618546952	16.85329574	2.575238662
20.40750423	15.35262816	9.927542064	16.58338931	3.097461271
19.77244215	14.92240898	8.948475644	17.5411213	2.583033941
18.61423929	14.42934389	8.068093803	11.63710304	1.69718392
20.23994719	14.07268835	8.49920029	13.83135009	1.338950405
19.39320831	12.74816858	7.271633053	13.75268672	1.268113937
19.89357139	15.0056786	8.111059449	18.33409461	3.026968069
20.11789648	14.46180015	7.734197003	12.97500257	1.538519706
20.03896607	14.08835504	9.001122584	15.57331984	3.864463054
18.58274649	14.12030652	8.353968496	16.97468138	2.976641112

20.45587643	14.87031395	8.83726962	8.561025887	3.688773744
19.43010702	13.5590293	8.099366177	17.97299617	4.448065461
19.0400391	15.32986825	8.990486169	10.95886681	4.79256393
19.19276543	14.72356465	9.56652752	16.06095805	3.09160916
20.49161498	14.23190679	9.608701517	13.53351802	4.76259373
19.2748265	14.94199875	9.662947139	14.47263803	4.900067004
20.74067562	15.31972842	9.578240305	14.61414606	5.73828879
18.91110428	14.95217166	8.748428547	16.43393225	4.602615758
20.85536687	15.21762112	8.209922291	14.72878776	2.774103909
17.56448402	14.41399494	7.847701832	18.26107046	2.323812103
18.98673395	14.15718438	8.568856967	12.91374533	3.718784576
19.77764439	16.04798658	8.571901119	11.47826944	4.800514018
19.07010744	14.45882474	7.804652167	17.32473809	2.833533191
21.22316198	14.40332157	9.44842754	18.00728791	4.236964247
19.81327157	14.43181732	8.064686066	16.24663871	5.478026699
20.67655596	15.38170197	8.550242973	15.34568782	5.169802934
20.24649382	14.11233386	8.421350282	12.0023316	3.811208326
19.76647817	15.15560425	8.409765436	16.51127014	5.196048312
21.07893124	15.41387325	8.182321584	17.13083773	5.284550721
19.61634049	14.55584556	8.844860139	17.27427492	2.715344839
19.70048016	14.7602264	8.937674018	12.97702493	5.355991154
19.61005424	15.23535491	9.011466793	17.86508045	6.491359694
19.44543432	14.83315935	8.714785622	17.80434812	2.630035239
20.85241843	14.34174333	10.43727503	15.8290626	4.915867346
21.56988587	15.11406639	8.084211226	16.03925749	3.000458842
21.68297344	15.5919924	9.280401231	15.47319324	4.293112679
19.3624857	14.72386934	10.22545679	13.3641236	3.981723637
19.79878481	14.92953515	8.71828438	15.65669676	4.015280888
20.66622791	14.26734332	7.811268875	16.61277967	4.791942721
18.29060493	15.46769923	7.886010169	10.56956686	4.628088927
19.76094703	15.09550052	8.644949958	17.42216114	4.006151458
20.47240676	15.46650807	8.925528171	18.66641063	3.93212337
19.98605271	15.64074787	9.422111493	12.70644572	5.583957731
21.5824037	14.21080148	8.355555572	12.48345704	4.702754593
21.10618983	16.10073087	9.180501408	18.88035789	3.300492585
20.29220514	14.97383494	8.3726748	16.91195296	4.413949637
20.20076415	14.23508777	8.677479287	15.15618168	4.046878021
19.18769798	14.0261174	9.093183834	15.71734505	3.881918636
19.75439105	14.82042501	9.092843583	15.83777168	4.743306991
19.16187648	14.01039166	8.576742073	19.03488977	3.843380903
20.23572204	14.29364415	8.999454416	13.03941335	2.591739185
20.81985881	14.23502971	8.311212685	14.79334933	2.373463195
20.98129914	14.84753408	9.772710205	17.60510681	5.378805677
19.90756784	13.90759839	8.357588189	17.82426682	4.357043244
19.30620135	14.18084701	7.665226565	15.83491189	4.194765554
20.39242383	14.03302304	8.088730332	12.70960944	4.106492452
19.91207168	14.88367679	8.53644934	15.8716088	4.936710906
19.74065551	14.53638752	9.667053352	12.11890205	4.091730217
18.5379853	15.36602901	10.11621139	15.71862923	6.513845966
20.32563525	14.95210969	8.724120477	17.98720826	3.446737608
20.47467507	14.0287023	8.494664245	15.8134998	4.309142457
17.96033011	13.54367528	7.875489965	16.28867547	2.766615156
20.14925727	13.92741135	8.032626243	14.35519315	4.45083883
18.76348355	15.72388963	7.492224004	15.22969337	3.73003829
19.90428038	13.70993422	8.510029864	17.52726979	3.79551868
21.34933292	15.78888247	8.30994054	17.70844227	3.073652386
20.67925623	13.77741102	7.803709201	15.67460862	4.447450281
20.17440668	13.68645064	7.743678346	17.16276475	1.71909381

19.57455235	15.1768513	8.1108795	16.43508398	3.643550425
20.05355079	13.81938296	8.34388342	15.4970023	5.151772436
19.74357885	15.63726128	8.973382992	16.47551127	5.042154146
20.78084129	13.90927879	8.733191064	15.04057363	3.271191881
20.6707652	14.59241718	10.03723107	15.50259248	4.146427426
20.58769458	14.72282742	9.437484996	15.02375485	2.767962883
20.25048367	14.64407578	8.831410331	15.2832657	3.450266952
17.7566255	14.50497115	7.445455027	16.86671882	2.766207524
20.20089668	14.9303342	8.285258266	16.38406632	2.026551953
19.51135885	16.8498476	8.502352556	15.2165923	4.161266536
20.5134247	16.51872985	8.65640159	15.68955939	4.140340671
19.75792269	14.96111739	9.187335066	16.65823578	3.521746479
19.77752279	14.93646639	8.294264797	11.59202276	4.237544753
20.25503805	13.58094187	9.277719408	17.26144516	4.508509491
18.50889366	14.145701	9.303798581	15.61540411	2.449535615
18.82256962	14.87775613	8.053037196	13.43383028	2.335155901
19.81023018	15.39221062	10.76755494	15.61294466	5.777188325
19.14969626	13.78759788	9.53215171	19.13513971	6.384234395
21.55198854	14.68682874	9.423630929	13.40424669	5.168639716
20.53672832	15.11753878	8.871983516	16.34644907	4.357377655
19.29714197	14.15765286	8.896795899	16.21855381	3.315960886
20.3357478	14.3158621	9.182783795	16.58819048	2.932487118
19.76301491	14.46063078	8.84636965	13.26058978	3.51955717
19.68548713	16.33822056	8.881334961	15.86580602	5.424782045
19.15628413	13.76504496	8.057578738	13.3090486	4.157815811
20.2393014	14.66370554	7.837613787	14.94425212	2.41588018
20.805844	13.48009335	9.012296483	17.60436146	4.230157736
20.08042544	14.66429042	8.539892664	15.12145294	4.279704397
19.81837269	14.65487719	9.370644174	14.50580495	4.529794734
20.80442815	13.40951291	7.522049926	15.797438	0.316533539
18.06980284	14.49617945	8.986064102	13.02551301	1.633291152
19.91300581	15.23346682	9.436465661	13.54740808	3.760365014
19.83958977	15.40813243	9.215930582	16.47217207	5.256037339
19.79968273	14.98055602	7.603856593	15.41574984	5.016935387
20.123208	14.24021957	8.737554408	11.51686839	3.704447852
20.15648771	14.46434234	8.860027259	15.57371963	3.095457137
19.13861029	13.32554754	7.62482954	15.52651031	2.259002292
19.09952813	14.81744292	9.467415883	18.0661085	3.108400043
21.39995367	14.60550603	8.611416121	13.90212985	3.341315069
20.26669608	15.59691352	8.588682474	14.94626794	4.926087135
18.68830967	12.83883646	8.583117986	17.43921016	2.753180119
18.04861829	14.55554208	7.880172496	17.91102033	2.031375439
19.50722514	12.99259554	8.787695315	16.41404645	3.429901802
20.78726542	14.42733804	8.419407101	16.43342987	2.398992489
20.06859558	14.62092194	8.884204464	17.33858937	3.928279225
21.29026035	14.85899316	8.403456808	16.05881479	4.68904687
21.85697669	14.54601427	9.592814007	16.30225927	2.686299748
19.76442765	15.19894967	9.114328677	15.82365985	3.801152985
19.92775239	14.61827315	8.247449177	17.67328369	3.436090094
18.85260396	14.36960906	7.739738047	16.25545161	1.349391392
20.95008527	16.34856822	9.764261601	16.86068311	3.786749738
19.72472364	15.331274	9.21044938	18.39610764	4.433373951
19.28602838	15.16515689	9.23299082	15.85964876	2.029584722
21.46954019	13.37850785	8.583381319	16.56835024	3.479002983
20.16578662	16.17313817	8.306602102	16.17451255	5.569163659
19.9346938	13.51001317	8.744571297	16.04657886	3.283407441
19.15887763	14.61023435	8.595337619	16.21407981	3.379407614
20.22942272	15.30951054	8.235330779	12.88794549	4.761432801

hsa-miR-10b-3p	hsa-miR-500a-3p	hsa-miR-224-5p	hsa-miR-424-3p	hsa-miR-7706
3.912814192	9.740525587	6.965210358	4.172486504	3.900986213
4.68435656	10.54761043	8.150235271	3.208051195	2.357401462
6.322172946	10.40730158	8.578194206	3.80154138	3.848701033
3.239800977	10.56069262	10.54033294	4.297943792	2.788183137
7.27340518	10.94180009	7.875837098	3.995928728	4.41128986
2.488776357	9.71620961	4.647706303	3.501305427	3.765331923
3.589879628	10.23344123	4.758331964	3.810347177	4.004077019
1.391136827	10.40088242	11.40181828	4.182996481	3.822268651
1.679522961	10.60273431	9.79921082	2.94917265	3.321811197
7.309159546	10.44593944	6.21968689	3.15069008	3.468251635
3.911966722	10.21175316	8.827525624	3.375148217	0.129012527
2.453585156	11.50781006	9.631660369	5.726937328	3.959993876
4.920033099	10.69842773	11.52799861	3.922702587	3.246877951
4.37321409	11.40235647	11.04423749	3.354345059	3.525628641
5.341005184	10.31499184	11.20024376	3.122197144	3.411189524
4.003608884	9.413983954	6.081027958	3.205742247	3.401062854
5.55184393	10.81468746	9.243968278	3.509686743	2.700728416
4.460458257	12.62469275	6.882209837	2.023944707	4.612808534
0.155446239	10.90568965	11.02050762	1.469411949	2.929961147
6.18812228	10.01341837	7.348895187	4.941808667	2.532194492
6.150952383	10.66884255	11.13802227	3.033539714	3.857616177
4.404318716	9.851258545	7.720768772	3.371772004	4.036827825
7.114169811	12.20943929	8.602739296	3.40704606	3.407718381
0.240071163	10.36056938	11.08733664	4.38797512	2.847698727
2.601789652	10.58050391	8.67169197	3.95027545	4.102536966
1.70442372	10.20615522	4.901905208	5.333581364	3.006248658
6.105156083	11.83492589	9.986561407	2.37449991	5.518372107
6.646347719	10.2984833	6.929279461	4.45871165	2.33311404
2.847206499	11.39553028	11.18559678	2.846086769	3.975866287
0.790851179	11.32038129	11.06903619	4.457626081	3.248089062
5.587811073	11.13233376	10.21332239	3.767302749	4.059321048
8.231347137	11.38907655	9.872278184	2.484030665	4.083971414
6.759083104	10.44911918	9.062155304	3.773090881	3.109616049
3.36109781	9.48144656	10.45063205	4.529693992	2.796392677
8.240097269	10.50933547	10.1538626	2.776078011	2.77701694
3.877510437	10.49233447	8.774109829	0.735057363	3.733240468
4.213532288	10.49959471	11.40063823	2.005990104	3.663107207
2.081029072	11.76046604	10.70594396	3.260834058	2.406634686
7.468004566	10.29413431	8.519065537	4.708584688	4.051847059
1.089948276	9.782270294	8.680767457	5.093570633	1.695119101
5.773728181	9.645118786	9.531956359	3.571141096	3.091517876
5.169981407	11.02540387	10.74125828	3.623726263	3.207813213
3.181345887	11.85301762	9.428558132	2.107803042	2.89849597
5.832041182	13.19316379	8.056694315	2.303841705	3.617958972
6.780631068	10.55666737	10.2913863	2.652395608	3.502226894
0	9.955753077	8.134100751	3.452950294	2.461704281
5.766114304	10.49583298	8.613320569	3.043006632	1.859554292
6.022801597	10.45603167	10.5025611	3.326055403	2.819698798
6.644116916	10.37268405	6.701802769	3.34977385	3.669791369
2.393789823	9.353333968	4.269751987	5.195983686	2.391046731
1.986285625	10.91074088	5.147883575	3.043993363	3.271431568
2.916079974	9.155941831	5.750828796	6.283870001	1.954352768
7.274922535	10.7385313	9.379122929	3.724560119	3.954638936
0.181096208	10.88377852	9.39036807	5.398000098	3.522971516
4.49035971	9.507874509	9.013830811	3.62139954	3.157715235
5.736610227	12.95911698	8.677725065	4.224758308	2.978296197

0.120325937	9.901782806	7.066445675	3.686574363	4.10611144
6.718943051	10.3617495	3.377755184	4.318365429	2.415077939
0.007287673	10.32001961	5.247913193	3.62145563	3.943588127
4.266852319	12.0926033	9.227677438	2.897530237	2.897246305
2.661122172	11.1211952	8.304873343	5.007301261	3.564143794
4.138601099	10.81668262	6.434118625	0.092086317	4.431939442
3.844410034	10.11325277	8.526897823	3.205603366	4.737549979
6.362250037	11.57704791	9.112089987	5.482072693	3.084716818
2.773664008	11.0297247	8.089956031	3.833466514	3.181546675
5.461604159	10.89400188	11.81158408	4.089556419	2.001797421
1.524925753	9.818558801	8.388257024	3.006365379	0.166610666
1.468489824	11.85849707	6.23409321	4.170486057	4.019728564
4.084987952	11.48184701	9.188948107	4.069088905	1.67455044
6.340062098	10.55488322	9.508578989	4.171023691	2.602099491
5.383618133	9.802427959	8.269084926	3.89599956	3.704418472
5.348284595	11.84082563	9.381403154	2.184378304	5.300576753
1.151376846	10.15926457	5.664339717	3.024050238	5.087483464
4.61629117	11.66243263	9.13028997	3.55553897	1.755785617
4.827040394	11.22703487	10.991389	2.889319972	3.709726813
7.177579953	10.52285317	8.053403685	3.510537407	2.716464117
2.981011864	10.9662702	10.12820776	2.980106729	3.345811642
6.120035978	11.72673485	9.099930613	2.723226558	4.492519926
6.952288475	10.64264949	8.728186044	4.778514047	3.524646888
5.230978815	10.2496774	9.252346762	2.565949753	2.23386673
5.396160653	9.860920453	6.624467103	3.882291148	3.702997853
5.877165224	10.96269201	5.057904236	3.672222894	3.556986407
1.662878664	11.67558999	10.11386446	4.23253195	4.542917612
5.504756909	10.11482013	8.057084139	3.274817563	3.274404173
5.986096146	9.596386131	4.586176955	4.293919514	3.318523248
0	11.14948972	6.107310904	1.731687463	3.386650524
5.616033807	11.40010402	9.752469096	4.003511932	2.042593604
7.093791095	11.64658437	9.847724864	3.126846856	4.362530335
1.948318367	11.80872291	10.51093424	2.673438904	3.492165964
3.172138563	10.09781006	9.081896001	3.324145366	2.587064146
6.916636904	11.41076605	11.22822397	4.012621173	3.570779413
4.938619375	11.8037662	9.110885751	0.195589482	4.520279623
4.54985921	9.809469069	8.865465432	3.503451166	1.430102921
5.16480287	11.53100171	9.251624114	3.394836458	3.532467059
4.847448913	10.83128026	9.408644829	3.103496554	2.35210843
7.792165543	10.78575739	8.79316661	4.209318067	4.344227178
2.947851505	9.961840587	9.066818588	5.973732223	3.099514834
4.234386788	10.25698884	8.154346851	3.581254315	3.733727274
7.482876573	11.27900291	12.45770123	3.41406532	3.539547925
6.522329352	10.46336973	7.637827173	4.01465288	2.909368243
5.308998653	9.781139106	9.730013546	2.925259496	3.621449653
0.826666797	8.862750665	9.92900607	1.761790635	1.367346114
5.23016941	11.10076849	7.181584932	2.791198382	4.230806173
2.76132757	9.289489934	8.831309818	2.910408902	2.911487485
5.358725246	12.27800308	9.392030113	2.937784661	3.860011011
8.005659329	10.21636025	9.723298966	4.837279891	4.572655339
5.42050784	9.850578791	6.151120734	3.518955857	3.644512854
4.68067143	10.48649449	7.37020954	4.915848091	1.323704265
2.818675038	10.15722426	5.845350608	4.761175301	2.629720445
4.625054867	9.728041485	4.27862964	4.344654378	3.911078089
6.97877293	10.10355295	8.567649142	4.207029891	4.857027288
4.779305184	11.53096927	11.44705834	3.983287542	3.396396532
4.7124949	9.117416028	8.697557719	4.584651227	2.434122419
5.458522188	10.24277991	5.580349491	3.574144643	2.998769329

3.891178927	11.70623568	7.045954715	3.356680357	4.065943652
4.509080131	10.53225992	10.92441463	4.315019544	2.78907344
6.053605663	10.49558058	9.328649227	3.397196251	2.875707131
3.967065326	10.0493916	8.991652161	3.584794574	3.129683002
3.730519961	11.48956998	6.783040149	4.316271016	3.602083612
4.070858415	10.99513723	9.589740923	3.936314116	3.465357411
4.203227198	10.4738881	10.16851854	4.394550431	4.30807293
5.813548798	10.74570857	4.773705693	3.258327227	3.627961467
4.118688918	11.54590042	9.387988498	3.351948673	2.349634124
4.063333983	11.09742319	13.18568262	2.553196875	5.682484131
4.338208488	9.962340921	10.3232492	3.321036403	4.580324968
5.017488814	10.1875679	10.05359849	4.291490599	2.177306276
0.094888255	11.00116662	9.595395588	3.41526006	3.880708223
6.870843577	9.767308227	9.909322431	3.748397016	2.629766218
3.135862976	11.1442266	10.24581494	3.306236873	4.388407495
0.082656898	11.21052501	10.01395663	4.286061574	3.36498825
5.142176569	10.62070247	8.994670819	4.289917315	3.881304521
7.505903498	10.60979628	8.411864062	4.551840146	5.230184574
2.857359625	9.634825089	6.631428969	3.384382455	3.295349939
5.758888226	10.34131701	5.720443557	2.936651771	2.804624429
4.50319365	10.5669547	5.92682611	3.487661157	2.286407629
5.549384631	9.430217519	9.555138687	4.023489383	2.68207563
1.133190147	10.2971542	5.296738919	3.783964468	2.997953075
3.008512909	12.35182923	13.8802049	3.374404783	4.670518126
1.498886498	10.39444981	9.068062822	2.176231255	3.678640141
3.100093536	10.77713343	8.31437984	5.149746122	3.099137417
5.738246028	9.784798462	5.263043824	3.717193235	3.909736912
4.274290417	11.85683804	10.68305968	4.146475165	3.574258603
3.9130821	10.27095812	9.518264825	3.813458096	3.912982152
5.03774036	9.715368469	4.37600686	3.102028982	5.189060611
1.628186548	11.27495566	10.06581796	2.322372869	1.633563726
3.401206191	10.83696279	5.638958028	3.680163418	4.174550372
6.2171842	10.95119904	11.79192871	2.398048911	2.662982364
4.649017892	10.97091085	6.249539192	1.386295902	2.827591338
1.852682419	9.78393486	9.493963691	4.308834306	3.980146518
5.14708744	12.33953629	9.021063818	4.421821443	3.798313231
4.387811966	8.89017608	9.834545154	4.88301186	3.455518291
6.671969951	11.42094269	10.36536448	3.976657221	2.253224889
3.216379295	9.227595649	8.999914064	4.073118767	3.08281184
4.211310139	11.04366906	9.502534002	3.997792341	4.657394641
6.804929792	9.26016344	8.76668661	4.594501906	1.781030404
4.676069482	13.6854641	9.104681659	3.586582183	2.028338383
5.813344455	9.624498174	9.466367366	5.394049612	2.660175116
5.63715048	10.27230494	11.23527636	3.435248728	3.761240659
6.075046197	10.39273018	8.887079125	4.571452043	4.387633846
4.81300503	9.311005247	8.478571327	4.790549977	3.637339292
5.769605623	10.58950238	9.339338041	3.185863982	3.18427577
2.638864707	11.46604811	9.521191636	2.638075912	3.135401295
6.418855912	9.976843683	8.652020761	2.805277967	4.035897031
1.998372562	11.87556158	8.046472745	3.057429014	1.999514772
5.331950826	12.18563221	10.5910765	3.869508362	3.610019238
7.494511239	10.58187083	10.58939832	4.297575081	3.358081676
5.010156552	11.10669265	11.79659806	1.375345507	3.689535575
5.609971745	10.14536877	8.330589312	2.383034088	3.702862478
3.67809201	12.31803854	6.846197817	3.498071791	4.091542724
4.593417192	9.740451511	8.792376246	2.778491505	1.994759968
5.821840937	11.34170982	10.55835446	2.981684096	3.640190149
3.374733721	10.73864228	10.80453212	3.369216277	3.894178807

hsa-miR-490-3p	hsa-miR-183-5p	hsa-miR-532-5p	hsa-miR-452-5p	hsa-miR-96-5p
0.108370222	13.01669882	12.07186147	8.914264238	5.787837693
0.006451093	11.57026669	12.20986102	9.268182003	4.288121728
0	15.39938552	12.21071625	9.462831008	6.319273604
0	8.484489864	12.58032175	11.44907606	0
0.014774051	13.54418904	12.03838425	9.025383315	3.547942078
1.72766352	15.04224122	12.51866865	6.322078997	7.568038092
0.204886805	11.95280325	12.83085952	6.737308164	4.640584162
3.532754653	17.47416117	12.44391683	10.8674877	9.324918089
0	11.57083817	13.97461573	9.4705898	4.066056604
0	14.17823615	11.91636365	7.351105442	5.467204746
3.696026619	10.97874622	10.80380384	8.604969949	2.871199574
4.709001825	14.31352985	11.56170174	9.402603581	6.088052455
1.936222139	9.978602792	13.68583063	11.91788121	3.623156005
0.064267258	17.02861508	12.65487702	12.16244346	7.205951259
0	13.94763483	13.29696878	11.28407647	5.755710168
1.912761601	13.19346965	13.07890135	7.45201817	5.861556056
0.704506064	9.517602027	12.27680722	9.968066378	2.351996174
0	9.916995868	12.39232872	6.563214288	1.330078285
0.121887896	13.68451312	12.10902615	10.62902622	5.97302524
5.853790579	12.58916851	12.62486454	8.323074506	5.262388061
2.183679913	8.64605999	13.70118727	11.44464418	0.512760048
0.107495125	18.09594307	9.886977935	6.88727298	9.585236558
0	8.070595624	14.97598823	10.2248996	0.887732573
3.121789326	13.45911544	11.95271276	10.68865073	6.867716331
0	15.10621779	12.76019336	9.929979999	6.723865517
1.920887821	14.86151204	12.58407761	6.533947612	6.601430647
0.00957149	12.65098423	13.45331455	10.76567488	3.379172633
0.169480471	10.96618465	13.1594429	8.219496725	3.871272795
0.048001306	16.81394217	13.90488556	11.57924639	9.520178628
0	8.79463191	12.91035056	10.97588379	0.791763025
0.112150309	12.72292994	13.60182008	10.08177394	5.923436878
0.030080364	12.15162447	13.17601579	10.66509372	3.498000129
0.055218011	14.18289878	11.59550535	10.65199313	6.963830839
0	15.22258632	12.03805686	9.935002397	8.464340818
0	14.63985585	12.62824118	10.61370661	7.626871596
0	15.94370631	12.26107116	10.29766675	7.160774756
0.091429905	7.236938889	12.92414001	12.688817	2.450496728
0.015540951	14.83796763	12.06538047	10.34747059	7.132067832
1.163536716	12.21933271	11.82063459	10.46787766	4.500654087
0.022948275	11.85397107	11.965929	8.637344462	5.428306986
0	13.45507422	11.61206823	11.03026551	6.50457469
0.006434602	8.721117961	12.87282105	10.88089673	0.007800002
0.114999663	14.3899431	14.17306545	9.983688392	7.0829701
0.161848613	11.85428237	14.84257498	9.510007829	4.957082403
0.136084209	9.31825526	12.35553232	11.60581371	0.17652995
0	13.44282092	12.24322953	10.07689044	4.815493216
0.228977054	7.45542791	12.81817165	10.79437605	0.312593116
2.786133398	12.53778469	12.67097065	11.28578992	4.641489574
0	13.47055402	13.38301832	7.79654952	5.857931267
4.534363052	8.703971832	11.45400595	6.579651096	2.860889392
0.087305476	13.29135732	12.28903603	6.578762649	4.822851029
4.963263055	11.41088923	11.34924011	8.174899578	2.916479322
4.414150426	14.6069558	13.20955941	9.508468684	5.237447646
0.140639243	12.37890863	11.91487885	9.241235391	4.341640409
0.027689536	9.219062987	11.77757094	10.23682213	1.107846472
0.781887753	10.05668522	15.19616819	9.385417148	2.009456587

1.360685474	11.90610107	12.77338106	7.854895376	5.070214287
1.127029159	14.27436466	12.89368515	6.352516606	5.612116787
4.426443804	12.27048954	12.94616023	6.465193599	2.842406152
0.015779663	16.03219644	12.87176682	10.78843005	7.744089976
4.165452003	15.85751337	13.45650119	10.40675819	7.634347423
0.077079514	8.920151948	14.1185415	7.15916964	0.096905015
0.006034024	10.50672462	11.97613804	10.26311266	2.355099048
0	14.10104417	13.19108787	9.910429177	5.450065288
0.749219451	15.2485254	12.53662359	7.796330072	8.160681356
3.461336154	9.690467468	11.63339577	12.39426735	1.001335215
1.510838474	9.543744372	11.8061132	8.863740855	0.179482941
0.905125133	14.41117429	12.34310048	5.749324374	6.48582982
4.268114131	11.31489799	11.90184182	8.588568117	3.469549198
2.134434812	13.06164231	12.99720443	10.66105088	5.05729153
4.990616795	13.3777966	12.074961	9.106339886	5.698527847
0.035855093	8.856944184	13.67670868	10.88867088	1.139283188
0.038816738	14.06024642	12.27373498	7.317648757	6.854121079
0.200062236	11.24968212	12.69909599	10.6228518	5.134729911
0	13.95781744	12.34706174	10.49804204	6.232385584
1.359076863	9.647971434	12.41412197	9.926520148	2.52776025
1.119614798	15.6066838	11.95930155	11.20783935	5.804765097
0.152344255	11.99560193	13.18141457	10.30408656	4.911721591
3.280263614	12.97514462	13.39283963	9.004820952	4.870980453
0.045918042	14.0121126	13.00644661	9.998087717	7.101307328
1.504676072	9.665118106	12.35205265	7.879590455	1.515791869
0	14.6351323	13.42686695	6.569893684	7.149929504
2.801134244	14.10179151	13.78738818	10.6375743	5.383114178
0.017980037	11.02325543	12.53642786	8.502676833	3.27553961
0	14.74205699	11.88399106	6.537388599	6.417216937
0.803427636	7.861724373	13.69996307	6.771648728	0
0.09998685	15.7095427	13.47865904	8.718945445	8.055677598
0	15.17480787	12.5845609	9.655076659	6.876550017
0.254842292	13.48392431	13.66037423	10.59580456	5.689799757
1.001463035	8.478142859	12.74766862	10.75813551	1.001452908
0.205149944	15.0890785	13.32549756	10.38844393	7.662271651
0.158473444	15.60804977	13.52487198	9.252985625	7.502594485
0.875129947	13.30341835	11.94602122	9.622990294	6.53200049
0.012323441	14.6878467	13.8811086	10.17464146	6.537824083
0	14.58672356	12.92475869	10.2115543	5.439756664
0	17.23784614	12.39899	10.30550019	7.512914182
4.485779198	9.598446207	12.98471561	8.960783668	1.806933783
0.074692491	13.44286636	12.54665955	9.698160389	6.223087843
0	9.140760695	14.11326441	13.5621439	2.763662761
0.017949055	12.20877138	11.79592488	8.869407168	3.104234894
0	12.7166096	12.918378	10.19556899	4.242003607
1.768909409	14.43258114	10.49366834	10.60548211	7.638881439
0	9.217857375	12.49727554	8.611210681	0.989093911
1.267011465	12.14753248	11.76378175	10.13093982	5.283413508
0	13.71592964	14.204755	11.35268645	5.684064219
2.420645679	14.42021891	12.60079802	10.69211272	6.069058253
0.009972303	10.83169403	12.18718112	8.162225187	1.632747944
0.523102232	8.935894641	12.35446803	9.462296623	1.331169609
3.911428333	10.64402045	12.20441246	6.753966129	2.629261622
0	15.78458358	11.3054098	5.622151034	6.27362226
1.150169308	14.9216987	12.23400729	10.27385535	6.332245643
1.644209139	15.41791001	12.44955746	11.05713951	6.900679579
0.020684976	12.0598843	10.74087877	9.219044708	4.935648989
0	12.41398437	12.3521413	7.795324707	4.60851537

2.915619516	11.92140251	12.72165965	6.917103853	4.576642988
2.119600922	15.47695501	11.93541535	11.56868973	5.518111905
2.316275345	14.4714114	12.29665056	11.05208779	7.016318551
0.427347439	13.62309734	12.83536777	9.934366004	5.652379178
1.095430049	13.27923241	13.20938762	7.605382973	5.008859337
0.086564807	16.45795104	12.7089132	10.17314987	8.335269456
0	13.99307123	12.09434667	11.28463573	6.437380613
0	11.95385681	13.00540508	6.251393545	4.48006711
1.60870344	12.39297261	12.41529773	8.924410968	5.119399228
0.04342455	8.487933606	12.89120469	12.14772448	0.053646308
0.057873538	13.65323214	11.31118595	10.3189772	4.510203748
0.034494752	11.44012411	12.23573668	10.61050172	4.45613326
0.076148384	16.81071818	11.94969788	9.965772108	8.700182419
1.62233738	12.89942339	12.14182653	11.74529119	6.004859244
4.128724833	14.89569043	12.8253922	10.48228589	7.287356465
0.066649158	11.92193095	12.48544747	9.147572209	4.292538916
3.610620396	13.10866232	12.45187631	10.38752229	4.33357371
1.150914925	15.57052746	11.31792831	10.42970073	5.523235452
0	17.08452335	10.29795877	7.638604046	7.938810397
0	14.83581237	12.36263077	7.275916949	6.850775812
0.056709803	10.72274644	12.42221651	7.45364193	3.12040262
0	13.65593073	10.85156562	11.35102477	4.593119544
3.350960261	13.76443728	12.05927162	6.515676915	5.825033987
0.035726332	15.05117046	12.98739477	11.76312183	6.63035531
1.4863358	10.94197527	12.74887568	9.635350439	2.977903787
5.710237825	12.56256456	11.8707762	8.181248438	4.861957129
0	15.84579941	10.58400766	6.659195057	6.364150542
1.404105928	13.7321928	13.55958942	12.07231553	6.431661644
4.590640548	10.14734516	12.37229444	10.71387502	2.813166098
0.243657285	11.48910539	12.9230776	6.624251973	3.99588616
0.166372085	11.1531371	11.83495713	10.88305972	3.419009177
0.752238659	14.76464333	12.52186236	6.312159224	5.947475362
1.054700558	14.45522005	13.42727153	12.18209769	7.207468246
0.099704762	7.921792213	13.78896623	7.791330076	0.126851977
1.864636474	7.93925796	13.02988034	10.65628004	1.186108636
1.0645915	9.669397161	13.31327003	10.97257039	1.663478665
0.05091412	10.91400279	11.87403554	11.10427557	3.459483228
0.645170077	12.68101513	12.83004436	11.35744828	5.636182465
2.587519879	14.61536388	11.45647258	9.809129058	7.404918669
0.656368958	14.88115143	13.0025908	10.22178906	6.995230074
0	15.47466756	11.4886891	10.78474822	7.546936047
3.107282249	9.524216082	14.00338518	8.653038474	0.387855287
2.447429988	12.08410035	11.97308472	11.15519301	4.362508746
0.080099945	9.073626566	13.70009959	11.30373208	2.736552731
0.954424385	14.5414163	12.58718421	9.230217158	6.745013835
1.086209258	17.07422738	10.98594828	9.595298513	7.525690548
0.069804445	13.82834205	13.31131639	10.27405791	6.701327053
0.060213348	15.42648854	11.80087808	9.886131248	6.96267802
2.293324202	14.69619851	12.52539921	8.672679845	6.660825895
3.444631524	12.00059538	11.35975524	7.495703758	4.600097976
0	16.54959256	13.96461023	10.61410816	8.941327006
3.175923972	15.57415765	12.72338064	10.18868552	7.337391874
0.096799631	8.767222611	13.73161127	12.0005254	1.369492951
0.181288685	10.09637725	12.70255799	10.31274907	3.48590709
0.092688785	13.78706293	12.82889456	5.895670966	4.613807824
1.975720969	12.02942304	11.56957226	10.3464379	4.594393438
0	15.23519684	13.06080135	11.39637424	7.307046944
0.106962042	15.29405078	13.19531802	10.58799323	6.784602807

hsa-miR-501-3p	hsa-miR-103a-3p	hsa-miR-182-5p	hsa-miR-139-5p	hsa-miR-450a-5p
7.269961824	15.83275034	15.11748311	9.631854355	6.345345909
7.942541761	16.3967951	13.93294014	9.692818819	5.085133502
8.282616886	16.37235283	16.17764993	7.780360737	5.176801503
7.769366329	16.55044212	9.800648632	7.564677995	5.322502954
8.149762087	17.50897823	14.58776572	7.971005466	3.789881585
7.363726404	16.31471155	16.63472534	9.28459258	5.143827129
7.524308586	17.66389667	13.65544513	9.64221086	4.36540668
7.606674649	16.96757663	18.51309405	8.353563011	5.973963572
8.026433076	16.25346369	13.27319353	8.686731848	3.081973971
8.484368604	16.89239745	14.77541627	7.305761935	4.629658502
7.254538401	16.24574828	13.7513456	9.628146169	6.371518763
8.288517798	16.98547524	16.49453182	10.03334629	7.599465779
7.449591001	17.21293358	11.44044944	10.58059897	5.129297228
8.07494581	17.1429008	18.15197466	6.761870862	3.825615458
7.755096903	16.58432476	15.42245502	5.15810655	3.410616616
6.724719677	16.78888415	14.92945962	9.775505451	4.820229206
7.683171384	16.13451818	11.62065509	9.480283985	6.008186
9.906828348	17.42492258	11.32969007	6.199620013	2.492470288
8.010172942	16.46835034	15.70755964	7.410604408	3.979300088
7.539401823	16.80982543	14.18693895	11.01343269	6.169054462
8.127369521	16.83841554	10.22743546	9.886301025	4.819507442
7.175901848	17.40280699	19.68258117	8.798930588	5.609718668
8.874486241	16.12067644	9.910092235	8.174778111	4.514668011
7.516786625	15.88901586	15.3879217	10.25999888	5.288387755
7.745514294	17.84789493	15.95597716	8.588042931	4.861032879
7.512298152	16.96974419	15.96515711	8.089799987	6.431649237
9.862587659	17.30441488	13.52417912	6.690708591	4.451851257
7.501426447	16.49911995	13.06334005	9.604490733	4.476150754
8.445146088	17.05242389	18.11151118	7.392877788	4.189356263
8.615167845	16.82383065	10.29349356	8.873188671	6.407520049
8.38225473	16.32503397	14.09253321	8.098768889	5.274696687
8.87900112	18.75028309	12.56694122	9.476909312	3.879521821
6.979761001	17.31374395	16.23557896	10.4371445	4.971798593
7.058277565	16.48863005	16.38779123	9.571834495	6.692885925
7.208780033	15.85605863	16.23959268	7.641185292	5.593028511
8.359511876	15.37519454	16.3382283	5.730517862	3.727736645
7.011825662	16.35922448	9.406485696	9.719028791	4.095841793
8.95735691	16.7583142	16.54737526	7.087733979	5.741481874
7.82345197	17.16782542	13.9736066	9.267456317	5.465561667
6.536858018	16.07637729	14.18263446	6.58753132	7.721141417
7.140640489	15.97131987	15.65180797	8.642351676	5.396052647
8.429615897	16.25632027	10.19650667	9.327055444	4.994782621
8.376555208	17.02729293	16.16221751	9.252124685	3.613637388
10.36974443	17.05615588	14.22573447	10.77249216	4.559026908
8.324216752	16.90259129	11.04486551	8.036389844	4.914345035
7.509262471	16.1542986	15.0652035	8.412522549	4.669286907
7.206163091	16.34837765	9.428293688	9.089514213	3.408632697
7.912687354	16.89059003	13.72393581	8.957790964	4.877326668
8.401212514	17.50250734	14.56490615	9.029404889	5.478825904
6.879558271	16.61516125	10.52514563	12.00841642	6.985578183
8.336018177	16.64394481	14.43462616	9.082661489	5.257957231
6.958755294	15.90003483	13.3134649	10.3729315	7.003965599
8.788861728	17.53694315	15.16651656	9.630475593	5.694094786
7.99156694	16.58917538	14.89925639	8.150141492	7.320058461
6.616431477	16.19927171	11.33535929	8.081270762	4.977262639
9.811046887	16.87977735	11.74115608	10.07547908	5.410513291

7.300544776	17.22454598	14.04072716	10.01905238	5.068445578
7.974728962	16.45118243	15.50005412	7.813812676	5.456326658
7.89088083	16.18349404	12.80289755	9.004989639	5.286277199
9.025689939	17.2531623	17.52313833	8.79440159	3.553854952
8.759160206	16.53907763	17.12014901	8.733236449	5.914303751
8.286536881	17.65024728	9.992562975	8.488280312	3.753354092
8.490242697	17.17906064	11.8376034	5.653250426	4.285487274
9.512586578	17.37367694	14.29238388	7.488651423	5.880633137
8.398559611	17.45187624	17.82858507	8.63368989	4.964057035
7.79662676	16.5275718	12.77577035	10.52283554	7.100219867
7.363265517	16.55720626	10.85965693	9.513138827	4.840926018
8.797715902	17.12032757	16.26868871	8.63172362	5.797306661
8.849742879	17.13163929	13.60948497	10.33257433	5.266140476
7.881375749	17.14728722	14.40496268	9.146586994	5.87935226
7.540958222	16.77248421	14.62336535	9.470798565	5.891399258
10.08809511	17.94194936	9.910662185	6.0289138	3.530577617
8.27471363	16.61274558	15.75438121	9.887408972	4.989920879
7.996154333	16.39190344	12.8274295	7.910521188	4.734528031
8.380493909	16.6558348	15.92536241	7.048825963	5.368392632
7.38370205	16.63136365	11.05270248	11.00017554	4.598281473
8.220501473	16.52146254	16.20970654	6.476892567	4.355276373
7.849453673	16.49450708	14.33293951	8.665099259	3.967965934
8.251846572	17.45902198	13.85992589	9.22275456	5.386865169
7.089683187	16.24507409	15.63851835	7.165111579	4.077274004
7.413411158	17.2879124	11.93398599	10.93062389	4.191047756
8.485521378	17.6476865	15.62406854	8.046642916	4.140970361
9.06525923	16.37857474	14.77905613	8.304210304	5.823506259
8.343378218	17.61569298	11.87601755	6.589711228	1.671035814
7.034472829	16.13503697	15.98292096	6.876651004	5.113552917
8.95173807	17.67842993	9.147270834	8.921304763	3.011352652
8.496730831	17.73869268	17.51927104	8.718791634	5.781579896
8.918016924	17.42925192	16.56192689	6.428400499	4.306467846
7.388527222	17.5475634	15.52922758	9.656255359	3.517031768
7.617114625	15.84998601	10.32321386	6.646252949	4.757229091
8.519871233	17.87965708	17.03300798	8.747086168	6.031846663
9.175799149	17.11896852	17.43783134	6.517768264	4.543168147
6.823061145	16.24003142	15.23372646	5.406581407	5.078775628
8.637516635	16.45898858	15.48188386	6.268336627	4.245906385
8.840364259	17.04185922	15.16190387	7.47357276	4.579569772
8.137056985	17.0394314	18.10567579	6.484679115	4.491504014
7.936565935	17.02457857	11.02391147	9.819549898	5.119076957
7.445975628	16.89150787	14.42330773	8.447158149	4.335067021
7.738958907	16.73175517	10.94524156	9.591179858	3.953244078
7.800765114	16.75335963	13.40198381	7.786726454	4.502858797
8.02443018	16.38187427	13.71965223	5.511010529	3.69171617
6.302405983	15.26279838	16.33912796	7.948695303	4.567064508
8.574324212	18.35544088	11.91514482	6.625812387	4.304459275
6.872005624	15.92626182	13.54916406	9.234664254	5.142201178
9.055339921	17.24661309	14.95967267	7.722275211	4.369825419
8.544239121	17.02348331	14.86489481	7.863963742	5.923981635
7.750611485	16.02775663	11.77730703	7.421628458	4.454314486
8.194628484	16.86123843	11.06606823	10.47432874	6.883043443
7.95391189	16.86205858	12.41937395	9.977333294	5.846341241
7.915913452	15.69192292	15.34012414	6.620696756	5.339568582
7.362991334	16.09779607	16.16468831	8.161159104	5.057538584
8.329019957	17.69437005	16.52864462	8.99707024	6.327909501
6.858778144	16.25373742	14.18847697	7.598828796	4.882746154
7.662598355	16.81467865	13.59253767	8.65359628	5.673136713

8.33823037	17.39781309	14.44086733	10.00110601	5.840653324
7.92245572	16.4852604	16.13271798	8.059197189	5.520991957
7.669165695	16.78426414	16.01576722	7.685937655	4.009465386
7.920698646	16.92040026	14.61876339	8.459964452	5.628189119
8.344151945	17.08836364	15.23144585	9.255209653	5.675134117
8.494936469	18.61197616	17.48240631	6.289069491	4.070725041
7.840240451	16.4418527	15.29901575	8.437551775	5.009389664
7.49394068	16.33293226	13.74318688	8.466387974	3.951845867
8.722593974	16.88213251	14.93637152	8.76732403	6.52401949
9.544945538	18.76141064	9.667872692	5.657279512	4.163631905
7.966806868	17.92547232	14.69984065	6.540084547	4.244082005
7.844686879	16.40635509	13.01908454	7.157479757	5.603770325
8.262193476	16.73956994	17.67642507	7.573266722	5.87284751
7.518769101	15.96276052	15.28841463	8.14400534	5.29811155
8.246791389	17.39220295	16.66442408	10.16725346	5.731153676
8.5365465	17.16178274	14.5200208	10.49826315	5.854188381
7.759632846	17.3360398	14.18796038	8.66419189	5.637705054
7.885435157	17.3834353	16.96487794	8.391644159	3.393701748
6.358048702	16.6312979	18.31180886	6.53608606	4.406087493
7.756372436	17.82084216	16.14664144	8.662719099	4.652812629
7.737594454	17.02956981	12.64939035	10.14238247	4.7946386
7.442364871	16.77576675	14.63678975	5.757367118	5.410962621
7.460709403	16.38192645	15.17576638	10.0225134	5.162360054
9.77194123	18.19155642	16.59296096	8.961252603	5.388152621
7.524686826	17.53965537	12.46256807	9.017370452	3.490811401
7.998435144	17.112345	14.789557	9.759188815	7.185291406
7.931835976	16.3901245	18.02116577	7.25278837	3.815513934
9.044362732	16.5693536	15.08145038	7.818440222	5.44746479
7.457701314	16.42643058	12.17048284	9.960708197	5.093816452
8.27647603	15.25790078	13.41177116	9.345631633	4.37652195
7.940205938	17.35170941	13.54362739	9.915108407	3.137845062
8.75781683	17.33712599	14.85788739	7.451315383	6.173298113
8.383738083	16.60387766	16.15452135	9.223661445	5.444934765
8.105548096	18.63355674	9.641582932	8.008413749	2.040577852
7.731592954	16.54201108	10.07377297	10.53107375	5.218722631
9.73813402	17.49859089	11.56731516	7.234092111	5.35081444
6.786634064	16.0106415	12.2808312	8.517090863	5.891861583
7.9094473	17.13921696	14.37253307	7.429059577	4.698889458
6.597292982	16.10066029	16.59398495	7.846767713	5.624938634
8.492894216	17.73182595	15.50065293	8.717159109	5.502193453
7.074740087	15.43091894	17.33479367	8.936917861	4.940937697
10.46602558	17.23287912	11.87431624	10.66222016	5.177692322
7.023935553	15.15585353	13.673773	8.433462784	6.102679464
7.655873645	16.67083966	11.1110588	9.957353369	4.458029114
8.246212283	16.74076977	15.59709484	8.363031823	7.27381852
6.396804497	16.9656999	18.0816161	7.346874318	4.781804309
8.233397742	17.23226111	15.40359627	8.099196947	5.540231301
9.052419253	16.56689497	17.53667469	7.555013926	5.214849589
7.350161517	17.15620843	15.70036145	9.341821741	5.309080806
9.062528871	16.30382295	14.17834243	8.871257338	6.86307546
9.013283924	17.28247846	18.23554554	6.554354444	6.21005168
8.32408196	18.06422542	16.29738726	8.692369448	5.715521964
7.164965875	17.63490733	10.75461051	9.37835432	3.854629171
8.139308509	15.75422389	12.77284055	6.567816079	3.924040529
10.39104304	17.12114052	15.37765593	7.363915367	5.390065529
7.45455767	16.08544709	14.16974555	9.655140056	5.033702947
9.136025356	17.24011477	15.73164047	7.42650285	4.486393097
8.174861505	17.14501781	16.61852503	8.76584314	4.292551393

hsa-miR-221-3p	hsa-miR-1307-3p	hsa-miR-511-5p	hsa-miR-660-5p	hsa-miR-1248
7.472265715	11.52168058	5.746249619	8.045543718	0.082451569
8.001698886	12.26521442	7.82931272	8.284883299	1.612805047
8.751199772	11.84664629	4.792808627	7.623521122	0.386766004
9.442684223	12.73429984	5.089981317	8.381850296	0.867984878
9.042996509	13.30377536	4.615288352	7.537327708	0.011736745
8.182931827	11.65469409	5.190176514	8.493505178	0.024402563
9.322071359	11.99825693	5.066215369	8.617206065	3.344681275
9.079869253	12.2385246	4.387152659	8.522606963	1.978222677
8.295396303	12.52821995	3.780093589	8.964173183	0.775864569
9.695728757	13.72778386	4.586324509	7.644369303	0
8.334662872	11.71913989	6.995619351	7.148588101	2.434296938
8.295537645	12.12147506	6.578671633	8.483197863	1.316993934
9.585059547	12.82530977	4.380186311	9.173018709	1.941998674
6.846435677	12.6213687	5.187341734	7.67361157	0.0498804
9.891582893	13.45806591	6.340538869	8.833346637	0
9.331020163	12.66995973	5.339331904	8.513239974	0.056976036
8.650155393	12.0557722	6.083116865	8.411638688	0
8.367047175	12.89048908	4.971714192	8.300836475	0
8.838247496	12.71597443	4.578612364	8.377508502	0.092185429
7.7338578	11.81037951	6.297577849	8.632852678	2.762062254
10.22614588	12.38647123	5.379792387	9.915798859	2.017964739
8.506440904	14.1823067	5.468587785	6.642029963	1.366593996
8.494864153	12.81080633	4.274430306	9.673981185	1.864238209
9.019520714	12.71787401	5.05277194	8.145118759	0.134450349
10.10637095	13.04413458	4.529406647	8.653580593	1.430758576
9.544383996	12.25727135	5.247907974	8.47650429	1.536319786
8.829878017	13.59062312	4.229197089	8.646629624	0.007622821
9.332123393	12.70001891	6.62204239	8.28258675	0.125586576
11.88662558	12.98561747	5.575767293	9.027004411	3.217519559
10.52400373	12.43128945	6.471153965	9.130635263	0.798823159
9.187795515	12.93721071	8.708342023	9.183682099	2.77865073
9.546773497	14.38584042	3.498030684	8.811114698	0.023722399
10.36849718	11.49039599	3.77695237	7.94430996	1.196049793
9.179511299	12.50493282	5.51531562	8.862811582	1.895218923
9.904334442	11.55739013	4.93024994	8.731173197	0.761412497
9.230820441	12.97889611	4.592067596	7.645061521	1.671045239
7.603540182	12.29850793	5.481783831	9.022539319	0.070090919
8.874219033	12.47137673	5.71257663	8.736017995	0.012341436
9.531026218	12.9289093	6.012893228	8.042756306	0.033103359
9.4401927	12.18055262	2.938064494	8.325286932	1.682048805
8.042344149	12.22762855	5.366448557	7.82467207	0
8.998748446	12.69768924	5.768938058	7.972319639	1.612734435
8.209415856	12.67513637	3.78956097	9.60883255	2.024575622
6.534105647	12.40546164	6.168369872	11.68586177	1.530462898
9.014095295	13.84917102	6.18474582	7.835182156	2.094331618
7.038293179	12.57937297	5.414871128	7.224620918	0.92005474
8.933989582	12.28128048	4.474064584	8.578088248	0.165577568
8.868699849	11.74322573	4.641189306	8.361422467	1.336686281
9.092801653	13.73106842	5.07338147	8.996665411	1.416601384
7.539594135	11.52554419	5.736969948	7.846799649	2.680838273
8.691352333	13.39965676	6.719824395	8.243864997	1.302337463
7.823588334	11.38297696	7.144573647	6.85262468	2.978963306
9.344959318	12.48488673	5.455676076	8.912433165	0
8.308602746	12.12847052	5.58383112	8.527700421	2.109041527
9.090496266	12.64910085	6.547666489	7.60043914	1.100492527
10.09384957	12.81029236	8.092602042	11.07242094	1.732397943

8.566669984	12.58233572	5.637915715	8.322238509	1.957886143
9.470132124	11.94694544	5.391299048	7.5720489	2.655040274
9.338425894	13.4742896	5.593148161	8.126621055	0.004830396
8.150681165	12.9242715	5.932396161	8.678313283	1.057823101
8.746042461	12.95476347	4.568534697	9.015470162	0
9.757867121	12.8624161	5.204557215	9.457886924	2.320936461
8.823702258	13.35541742	6.921340269	7.975651815	0.004813775
8.441329492	13.88322659	7.219875775	8.82254973	1.149070719
9.023539993	11.99822203	6.136657884	9.128186566	1.689711809
9.713022971	11.4642096	6.287591722	8.33760381	1.001260347
8.231816073	13.23180049	5.463911113	7.586468725	0.103785204
8.860106263	11.98333825	5.013887168	8.605461705	0
7.685729542	13.25545747	6.983673855	7.712870454	0.131171174
9.077816523	12.83364727	6.563913486	8.949676445	0
8.238447118	12.37705181	6.060924278	8.508086629	0.937762999
10.265771	14.31998622	5.306777476	9.401373912	0.028199435
9.003190149	12.93226232	5.093891919	8.445998281	1.139623731
8.268536394	11.80692714	5.81174363	9.028056235	0.146375202
10.38781364	12.46945966	6.737355482	8.5169129	0.828410328
7.721847575	11.66211782	3.86657061	8.939862381	0
7.7470919	13.07278777	6.400018972	7.33180553	0.024201305
7.324022684	11.75355953	4.911087252	9.089944608	0.113711797
10.61643277	13.22977465	4.838341117	8.969429169	0
8.709757104	13.06214425	5.184013865	8.697042763	1.164235136
8.493162419	12.53613417	5.211426872	8.295147355	1.455384515
9.656277465	13.5231318	5.803117045	9.489424778	0.982856303
10.63769572	13.46161782	4.535087658	9.416244115	0.764341451
9.744118626	13.39957386	5.112133828	7.889293965	1.065768559
9.911093403	11.62094397	5.153530248	7.494765208	2.538998132
10.82933836	13.6152407	3.269714898	9.077323778	0.8130762
11.40855572	12.84487299	5.967219245	9.935690533	1.342924704
10.70032147	12.95521178	4.207666119	9.013575797	0
7.533363822	12.54310251	4.586360307	10.20621457	1.784541491
9.547376085	11.86582625	6.541543811	8.809764677	2.323678345
8.878338124	13.28996412	6.641325893	9.635275787	1.65250795
9.153420726	12.69143655	7.314229079	10.00361658	0.117978252
8.509847078	12.41747815	6.598063514	8.069683032	3.645077261
8.562950034	13.30495733	7.009151463	9.343609834	1.045285239
9.777094893	13.24776448	6.290597956	8.806954613	1.938375589
10.03749679	12.64177161	5.257539165	7.621221268	0
8.73644033	13.20903645	5.617843489	8.382151834	0.864154473
8.886236748	12.08172671	4.826320327	8.90675341	0.05769558
9.064689164	12.14962955	5.858526753	9.742105691	1.552283051
8.536521391	12.67479051	4.570304857	7.362088667	0.014237314
10.59461171	13.09820701	4.951341215	7.757074713	0
8.957773993	12.36508104	3.623374707	6.667700187	3.354157605
9.711462217	12.51052034	6.191523338	9.156777963	0.989690585
6.933634668	11.8094407	4.263332707	7.733853978	0
7.902552736	13.64368203	3.949923736	10.01102049	0.980878383
9.037939786	11.60290568	4.581733343	9.06422117	0.408101307
8.708092707	11.87612696	6.107816712	7.182964079	2.368473831
9.279749842	13.02394712	8.201559158	8.47304749	1.91017218
9.355212014	11.81354797	5.732343806	8.653729632	1.857489462
8.5672609	12.99829157	3.908289962	7.107584605	1.867094991
8.974495804	12.90282036	4.908789154	7.201027299	1.17449419
8.352599592	13.66241167	6.188699967	9.354989788	0.01005484
8.869606535	12.26776725	5.298802553	6.872346317	2.416959403
8.524779859	12.28323028	5.953108259	7.944743494	1.350085429

8.191156466	12.39908925	5.53272264	9.112730045	1.691887991
9.816540812	11.77334741	5.718900572	7.623767075	1.036443087
9.400257587	11.05744275	4.438788963	8.289126865	1.36913309
9.471475539	12.05397604	6.948463717	8.864825787	0
8.87560968	13.62302792	6.737858244	9.04627508	1.688056654
9.351956494	13.09781221	6.312814282	8.852210358	0.066506934
9.330349478	11.72275401	5.8231513	8.437634391	0
10.21018307	11.86645149	3.506007811	8.683913158	0
9.167054353	12.27167509	6.161498373	9.192490779	1.01859978
10.53708913	13.58694414	3.838946923	8.916285905	0.03403136
8.571804722	13.91222238	5.555618794	7.460265026	1.205048832
8.49179677	12.89167316	6.045657727	8.34486274	0.027146973
8.268881767	12.45777766	3.748741274	8.039768691	0.058781147
9.944635181	11.68755183	5.179507539	8.350465096	0.006330191
7.741138287	12.59396868	6.213162696	8.58349982	0.018597025
7.488892407	12.61306105	6.607205853	9.020862972	0.051672467
9.222580732	13.24460151	4.719905977	8.029939247	1.22979895
7.391098893	13.36985026	5.281355609	6.649955761	0.030544324
8.646839748	13.08088528	4.010647868	6.51623518	0
8.65066396	12.1473866	4.867092121	8.633764331	0
9.433726366	12.41484393	6.570834461	8.133560764	1.201109796
10.35737617	12.36377851	6.286270235	6.930123282	0
7.947825531	11.8015521	5.253285405	8.149798657	1.727352049
10.32137546	14.20878817	4.860955921	9.150925703	1.128825661
8.73440274	13.57365421	3.691818174	8.699649714	0.098782395
8.502147423	12.22867748	6.015272589	8.471745221	0.013654884
9.846225792	12.81406985	6.737581372	6.869983686	0
10.94483762	12.40848621	6.132904753	8.987894526	0
7.561082747	12.29423114	4.650175993	8.287273107	0.000798644
10.18434445	12.83983725	5.040821557	8.396350482	1.755509997
7.002303187	12.27306942	6.032710659	8.654170904	0.12344489
7.951918388	14.06551038	6.690434743	8.226312122	2.261145461
9.616740529	12.06664296	4.945005369	9.305966685	2.386501868
11.04207312	14.12715883	3.869735644	9.234084374	1.342030264
9.083149963	12.26446217	5.243565298	8.599480624	0.691382455
9.38828849	12.56169084	8.023167863	9.710663288	3.538292029
9.318421497	12.3040543	5.831248268	6.94052186	3.690561938
9.02867946	11.68023	5.828484856	9.154449754	1.167442996
8.126123424	12.02520475	4.698788036	7.539337867	0.854152105
9.743355235	13.33406255	4.21091707	9.094740547	0.669956248
9.366983682	11.47242087	5.611694134	6.865741183	1.805163217
8.727853968	13.3308902	7.01709566	10.44228907	0.196260973
7.878083779	10.41258501	5.450935834	6.973673965	2.461297087
8.907724458	12.4834526	4.85535731	9.181144274	1.902565251
10.51378339	14.05929495	6.334426485	8.708856538	0
8.702256113	12.26067248	3.226296979	6.221031885	0
9.144857968	12.41290411	5.456115352	9.275117969	1.864862468
9.056183323	13.81958144	6.033141263	8.009417353	0.046821429
8.994833555	13.64232576	6.574839978	7.908590522	2.20562654
9.0273943	13.38720717	6.569140484	7.649056669	0.069086195
9.649297411	13.23201035	4.597668874	10.72921159	2.567338777
10.46577808	13.40647199	5.218167945	8.978854522	0.930570866
10.03157165	12.63244822	3.855043455	9.704728754	2.990845928
10.90419225	12.98969655	6.402063237	8.804890933	1.586121252
8.622217649	13.29257413	7.07876342	9.290438314	0.071015796
8.124871526	11.88851767	5.874269681	7.600997242	0.06828436
9.593732558	11.93972737	4.419385947	9.704573997	0.49205254
7.78997531	13.46733201	4.29342593	8.615285468	1.364922192

hsa-miR-1180-3p	hsa-miR-1269a	hsa-miR-101-3p	hsa-miR-222-3p	hsa-miR-767-5p
5.828783413	1.406351954	16.70920381	5.829036762	1.410207086
4.625500746	11.60118772	16.97733307	5.532987719	0.007862549
6.462180513	10.68309316	15.35040576	6.871825366	0.387421793
7.694924308	11.21368042	15.96551242	7.334042833	7.334113251
6.964414787	12.94124017	15.67539947	7.694203064	1.65524223
5.715305395	12.1713153	16.90727134	5.806685993	0.038218998
6.257590245	10.38044777	16.54151558	6.736249349	0.278445698
6.814401134	1.045556353	16.0266311	7.590293745	0
7.767583133	2.451739271	16.22307058	5.765504587	3.082850358
9.529518687	7.370154009	14.68326916	7.788443823	6.403297765
4.605163315	9.888327132	15.65008855	6.265058508	0.139270064
5.891182263	3.827297844	15.22047907	6.859404989	2.002952073
4.242941293	11.46524719	16.88637824	6.258511811	0
7.903127526	13.08657597	15.03460018	5.469811807	7.682084442
6.591861441	15.04644816	17.27531624	7.913572517	0
6.051523964	8.713549902	17.14734711	6.218958965	2.977924096
5.461704113	12.99336769	16.54331791	6.446648639	9.438657445
8.80065681	1.332794903	15.02370367	6.763160699	4.297803792
6.941405223	5.491827997	14.56700088	7.614477683	0.158075651
5.069843615	6.96197108	16.6713597	5.225823067	1.540569819
6.468731167	6.099564859	16.81891514	8.157162344	2.278003406
5.743542046	2.518863502	14.31503744	7.698344737	2.070309088
6.439066084	12.79739855	16.14416355	5.764670862	0.888251703
5.935339882	3.484022326	16.88776191	7.499459838	0.24475895
6.384254826	3.369257205	16.04424005	6.978336719	1.417942876
7.733057516	4.132822733	15.80619048	6.715101271	0.459007838
8.819728466	9.363233306	14.76743935	6.992114358	0.011684781
6.350334208	4.044284926	17.30406737	6.051444082	0.225722865
5.876911039	7.008571889	16.60986601	8.909757594	2.242679554
6.457465942	12.3268742	16.18131119	8.19402696	0
7.198844992	9.16056558	16.22399558	7.036719956	5.390115261
8.647926518	13.18539135	15.86049768	7.165489568	0.037121107
6.288416912	0.069509821	16.26051317	8.243450334	0.069061203
5.596396387	8.400103388	16.06055532	7.15663112	1.183061604
6.294806977	10.16588842	16.37946651	7.410570357	1.277827427
7.60347721	11.41010809	15.37760276	7.731756425	7.770736827
4.607479202	12.0929671	19.39174447	5.483222696	0.116610177
4.681576353	8.680626923	14.82231352	7.375146774	8.46109033
5.684244796	12.42963197	15.69357313	7.15527428	0.052434778
6.27344248	11.49888011	14.97738083	7.803238955	0.028213029
5.939847677	12.81839168	17.94821973	6.053521863	4.753495335
5.39850944	13.51173284	15.5897555	7.533341523	8.963377609
6.240622287	2.09800475	16.43074979	6.210456097	9.157043519
6.532711377	0.216457621	17.13044271	4.868669536	0.214632
6.253581936	11.92463565	13.86729767	7.051239983	9.276166339
6.181051508	8.958480339	16.33178073	5.61458652	3.040319711
5.266712891	13.69613293	17.02419631	5.834834204	0.315555934
7.441250523	3.326862525	17.01654347	6.719541081	0.125467946
6.555397643	13.04817125	16.10123659	6.866554693	8.43697913
4.114305085	0.249072337	17.59117723	5.50598404	0.246861448
7.14264173	9.012601868	16.00607737	6.81678749	1.983172856
5.864436296	5.207667416	16.69014633	6.019490078	0
5.543444156	2.092015895	16.10535831	6.70216946	0.558125315
4.460274346	2.666066072	15.47469192	6.514114074	1.52829183
6.967928753	13.55561219	17.35713153	6.817381936	1.107304499
5.557951119	2.478362625	17.64534547	7.927168957	0

5.457546051	4.444224255	16.51133347	5.722603664	2.019240788
7.08828927	5.535254118	16.22224066	7.773544341	0.64126731
8.574353382	10.12272676	15.42027579	7.732561257	0.007378163
6.422021739	14.18690262	17.01946582	5.772090257	10.06214134
6.933303559	11.72309149	15.71281823	6.725983767	0
7.816076876	13.40676758	15.73608281	7.458582268	10.84741578
7.93373908	6.342535912	15.65154707	6.682678067	2.031501708
6.78717428	9.755171128	14.51768487	6.424019934	7.007806416
5.154397076	3.673017871	16.02344923	6.981890622	10.16937449
4.394457591	8.869494425	15.70504122	7.743675112	1.001328749
7.133522198	1.514350616	17.40245849	5.899336371	0.180863853
6.251254713	2.447840392	15.23785534	7.595930062	0
5.116579728	1.655363559	14.69763307	5.865351748	1.66213393
6.69942668	9.102541719	16.65593667	6.614141172	1.417560463
6.230448886	11.61654682	15.61835173	6.429588442	8.741660114
9.439984289	6.746156194	16.25911445	8.267708856	0.044383228
5.190857579	10.54800296	16.88048281	7.691945884	2.528749534
6.977603183	11.61172366	14.88685302	6.624059878	2.453513247
5.624776673	3.516879313	15.75587621	8.721469826	7.238384101
6.060357004	11.01852919	17.91441309	5.663444171	8.531817599
8.435363279	3.990140822	14.44033238	6.121671056	8.352141219
7.035027725	9.719954786	16.2361836	5.535396078	0.200951851
7.401761606	2.634154611	16.82639704	7.95166782	0
7.790107478	12.73215624	16.34414064	6.656591135	1.799241229
6.318367965	2.647590351	16.80260013	6.349389945	1.512936457
6.912485183	3.431414578	16.58825448	6.764385323	0.981966522
7.775272583	10.13582015	16.46186975	7.988147808	3.500830761
7.862429175	3.915890415	15.41439832	7.436163499	4.75545548
5.799285492	4.209360275	15.21170341	8.061014261	0
7.67960046	2.864896391	16.3418186	8.423827392	0
5.969064658	9.225151121	15.87462785	9.531546612	0.128120671
6.166640104	4.442749679	16.96159012	8.807846809	1.526095329
6.006464048	12.8782508	16.50959711	6.062599266	2.671351722
7.743867828	1.586724378	16.07492424	7.35995281	0.000449003
5.783719735	9.745296804	16.36040429	6.902010778	2.473965066
5.320402975	9.978330268	15.22457096	7.944214258	7.285583374
6.855966345	8.124246273	16.03290849	6.000132289	4.438996223
6.207695867	13.92036537	17.34193287	5.833108189	10.24262626
9.197093438	11.51710202	15.56841418	7.556529075	4.914532435
6.863627471	1.940226326	16.04079594	7.355030329	0
6.855680195	3.234714705	16.35350362	6.512737295	0
5.762829761	11.92498601	15.90002876	6.920387581	8.977520283
5.652046075	13.33106819	17.24581318	6.443164867	0
5.241621269	12.352195	16.15912662	6.85530582	6.539914092
8.22534276	13.44787734	14.69340664	8.864433468	2.924744419
6.28773178	1.766046377	13.97795112	8.054305632	0.828209185
6.424939039	8.723318543	15.39415804	7.57437254	3.442522746
7.480715455	9.595054559	17.54793259	5.079722686	6.88138767
7.186914285	14.08214117	17.28329815	5.985258607	10.10998288
5.29208196	3.447149618	15.18618585	6.508241261	6.683856052
8.340153525	4.817383538	16.1079892	7.515217769	0.012176651
7.034808052	0.991889182	16.14525908	7.268989582	0
5.823392714	1.841659627	16.05251224	7.047620956	0
6.581773413	12.13875407	15.13231285	6.305606972	9.904153338
5.771414744	12.83395522	16.56235049	7.004685878	7.340788247
6.999577786	2.390390107	15.42829881	6.767195981	1.049250617
5.451297162	4.712510817	14.37239763	7.506046921	0.025400128
7.081412102	2.998687878	15.66415544	6.542065971	0

4.826985508	9.07190982	16.20493732	6.404603982	0.301076303
5.99856121	4.358630936	15.76119458	7.439350518	0.331605456
5.999176679	10.60599622	17.46709517	7.222325054	8.983217717
7.202755515	2.336785393	16.34438044	6.844457917	0
6.362608942	13.41701352	16.55964149	6.753452968	2.125595992
7.98600149	13.01683557	16.00587511	6.756804634	10.33290435
6.710833438	10.77863595	15.63921309	7.568610058	7.817247204
7.549153231	6.422501355	16.73270478	8.152657157	0
5.458372982	9.320352574	15.60561528	7.39004188	4.616633466
8.544076605	13.10067019	15.41179897	8.492202315	1.167425538
7.105515236	12.02137736	14.36530186	7.373541994	8.959101776
5.797900096	5.941104425	17.04546029	6.279924148	9.302680673
7.096473782	4.014910135	15.35697641	6.38209772	0.096317866
5.635548556	12.21030573	17.52710189	7.792601861	8.829243687
5.70149886	13.10739553	16.28088881	5.606238263	1.091226568
6.443275021	12.31802263	16.63396516	6.041803038	1.89233405
8.545738138	10.19454066	15.32075765	6.828368972	9.624189871
7.306873115	9.52534814	16.78097153	5.560252865	6.632605693
7.544899104	3.293110572	15.5147634	6.71249127	0
6.91558626	12.01226765	17.22933489	5.564928571	0
5.926712578	14.64321826	16.41027263	7.164507735	1.217751527
5.707727951	10.73997349	15.5172062	8.424247133	9.834397499
5.765138636	10.06071943	16.79352154	5.702338212	0.042174782
8.21101407	3.528698429	15.27844231	8.31001871	2.182246956
6.162887086	0.172296922	15.37277234	6.493787275	1.493668702
4.807468389	5.107431723	15.69975881	6.877436765	4.353813614
6.133487788	2.913650389	16.02841188	7.645552985	0
6.411034471	12.91992782	15.43888727	8.762341537	1.923511683
6.800882717	10.14222474	16.564148	6.050838135	8.664702496
5.242034516	2.617729566	18.57323048	7.945384864	0.33868226
6.8664099	1.615284791	15.83685053	5.423511211	8.749615487
6.740113191	13.09461292	15.41567154	6.125802907	8.493571694
6.802878983	3.404291198	17.11650787	7.596688569	0.017027659
9.040342711	11.26353917	17.75491183	8.978165993	0.127739505
3.972435715	11.75645367	18.2171716	6.579736964	8.075753839
7.352569099	12.26425238	15.24773606	7.726853474	6.253958119
7.118175471	4.470604311	16.47651168	7.491287776	0.063531815
5.859522314	1.807592069	16.54379749	6.993608116	0
5.849218019	6.007187679	15.45860357	6.397918173	0
6.089336657	3.018944987	15.90956711	7.193481227	0
5.994476331	12.45828499	16.8644837	7.3259661	4.057910954
6.10103761	3.59632602	16.31095025	6.87057093	2.015584739
5.879667923	12.27356838	16.50295517	6.434547083	7.419087379
5.422469472	9.635076962	16.14192512	6.702590778	1.305480867
6.757562424	6.582877112	16.32882035	8.396045849	1.529569327
7.018467809	4.987690297	16.00961263	6.823930604	0.611023822
6.482573053	8.777818438	15.56020645	6.893329534	1.266996024
6.839271158	11.02669491	13.36647992	7.659331017	2.909368166
7.430622272	12.3483385	16.68226205	7.128491589	9.924795065
5.845828571	3.662036854	14.15175966	8.337221176	0.114782597
5.989049007	1.752202639	15.43048883	7.842970812	1.358436388
6.623551526	2.958638978	16.14332447	7.558341344	0.477850402
5.952298483	8.412755598	16.85518273	7.236989351	0.123820813
6.535566942	3.919856034	16.56586408	8.550533605	1.675330714
7.04972013	2.451496234	14.0028257	7.772284476	1.352279276
4.083725609	11.42887849	17.56943917	5.803498584	0.113327589
5.905549493	12.66154373	15.23580404	7.410523141	1.263482199
7.109028312	2.516456385	16.11426392	5.564575071	0.137583146

hsa-miR-33b-5p	hsa-miR-105-5p	hsa-miR-1301-3p	hsa-miR-877-5p	hsa-miR-452-3p
3.149540212	1.409164955	4.510503519	3.138645575	3.381568406
4.625349738	0.007877932	4.898668011	2.033870064	4.898685109
2.212578185	0	6.87605166	3.221447534	4.606325775
6.106964435	8.112475714	5.916217339	3.243164993	6.648207265
4.483531205	1.057376269	7.148927442	3.787818378	3.67401178
3.884413257	0.038299214	4.509688431	1.122762032	1.120679995
4.505927006	0.279347892	4.639209921	1.771057262	3.305872657
4.129498755	0	4.883895381	3.066993741	6.146060647
6.26966298	3.423900497	3.780123317	1.678028601	4.688789698
3.148461049	7.438002519	6.985119817	4.762746694	2.892513073
4.297982488	0.139628752	3.912241894	1.418256286	4.053337646
3.969531197	2.002071711	5.659683953	2.449893618	5.436341213
4.313377537	0	3.50807824	2.252866722	7.037672248
4.06969343	8.502124459	5.629639117	3.153781449	6.632391279
7.340699186	0	4.806980771	2.76113578	6.842957211
4.74856258	2.977392549	4.596403236	1.288134819	0.092237533
6.367498698	10.32353191	4.914798547	3.831989746	5.318895319
4.179201479	5.62387731	6.994319988	4.417018852	2.845516484
5.200853387	0.158496872	5.933765527	4.452147564	5.814964161
2.753241427	0.964022812	4.263515806	2.532409432	4.029434065
4.635730407	3.038902202	2.288962451	4.352364321	6.457919641
2.526125713	2.865588409	6.82529487	3.891325228	0.136857177
5.134671003	0.88848316	4.064026691	2.638776102	4.941881772
4.958768502	0.245512375	6.472063771	1.690499691	6.890053014
2.958234263	1.418256443	5.771117996	2.793125425	5.90007335
4.653662137	0.459839347	6.15825199	3.633753597	2.366564516
3.758689243	0.011707851	6.038107777	4.812965901	7.409879166
4.203759198	0.226397112	4.048277756	2.329320273	3.668861888
5.874589043	1.184370171	5.575537132	2.243194137	7.223531211
7.484904211	0	5.706024708	3.126278548	6.471211694
3.77461937	5.218448675	5.333051644	2.093007023	6.161851259
4.268614956	0.037198828	7.295695385	3.759024048	5.914239888
1.843754989	1.211613988	5.7911336	3.303583796	5.917229957
5.496024085	1.555289921	5.90247561	3.627843786	5.636849987
7.475936962	0.755513523	4.743962328	2.61329008	6.413345958
6.597262542	8.743294492	5.519978931	3.813173218	6.095383798
3.826921601	0.116897997	3.497092885	2.447942402	8.064967207
2.406896809	8.041723821	6.098048253	2.080933913	6.713095295
1.784336724	0.052548334	5.649367322	1.784681531	4.574977724
3.304058462	0.028270919	5.352021406	2.118737447	4.896621892
8.532100219	5.633192958	5.211720457	2.367694676	6.727093027
4.740807482	9.365774647	4.499708485	3.207578859	5.822387749
3.413414636	8.997528247	4.087090545	2.105066669	5.402891984
2.30435884	0.215261737	6.129604885	1.615991605	2.766900092
3.513722828	9.993798406	5.611799498	3.496068369	7.119066534
5.253355122	3.452191718	5.319937157	2.682187458	5.070306856
1.857232659	0.316634904	4.473403083	3.028551609	5.994941379
5.918738228	1.371089353	5.562012139	2.816624332	6.11977849
4.882146094	8.592184316	5.305514737	2.941614803	3.568062598
2.394774489	2.386222902	4.11395964	0.219940664	2.861650295
6.339209174	1.331476285	5.204067251	1.339698146	1.985486377
5.693065504	0	4.851492474	1.267258224	3.488189478
2.420727125	0.558865515	6.110149973	2.930479151	4.351733745
3.535060464	0.184816279	5.475152618	2.665928085	4.938324107
4.415802616	2.470604944	4.416183157	1.717260732	4.813616284
3.810012506	0	5.12155629	2.008611438	4.840382839

4.62687039	0.122536974	5.355524278	3.505672587	3.31891211
5.078722883	0	5.455438208	2.725244431	2.720790925
7.038612873	0.007392581	6.921433589	3.49533729	2.84242866
3.262853484	10.68767214	5.268751634	2.407580108	6.347652132
2.38902463	0	5.285001757	3.634218204	5.136120652
2.996167384	11.06838781	3.753566969	3.217845387	3.225827531
3.035509146	3.49585556	5.766374138	4.680600999	5.497819003
2.228232184	7.453229657	6.026466739	4.175551961	5.429599068
1.656951546	10.5896777	5.757843941	3.679070294	3.395990344
3.323980366	2.001778384	4.172050975	2.80931285	8.552944719
6.318929419	0.18136539	4.658385573	1.529499952	4.926446609
4.095104486	0	6.217492992	3.438388469	0.905971916
0.231359391	0.238384404	5.65185479	3.67815331	3.705751088
5.295768552	1.417874716	5.231099819	3.591879041	5.878807461
5.592254355	9.603993433	4.478085308	3.804389141	4.892266868
6.000946089	0.044477667	6.613524151	5.210757414	5.679582121
5.880581923	2.797604087	4.555362796	2.528044867	3.3932891
2.460655168	3.566147089	5.043473679	2.450515105	6.346901528
4.286108431	7.976227593	4.906613579	2.724524583	6.563538648
2.057100487	8.525229678	4.086960409	1.748199097	4.086911941
5.187880335	10.68452128	7.714529415	4.428112304	7.429486431
3.075127749	0.201528326	5.639937441	2.715909489	5.532597202
3.342452195	0	6.416607448	3.035897881	4.502729324
8.882743683	1.798803686	4.271793448	1.801656897	5.23146488
4.830861338	0.178848853	4.191824408	0.160739536	1.516878302
3.556630472	1.562834242	5.327927216	3.431778555	0.981844582
4.827924344	3.759792223	6.702606039	2.93217288	6.017113714
5.756241673	5.156794416	5.112066255	3.69050895	2.910269811
4.322275461	0	5.431821643	3.817816568	1.53733881
4.373240546	0.807472469	6.265277715	3.274423677	0.805878439
4.466278202	1.378332328	4.734571954	4.122343724	4.734830117
2.612951344	1.820056537	7.565128838	4.271231214	5.344198704
0.345402286	0.357860141	4.042947906	1.947773228	5.943137037
6.556961081	1.001442708	5.494220632	3.002157909	5.702812979
3.590070732	0.279749561	5.661665048	2.933817018	6.759467587
3.102575107	7.124537209	5.616378235	4.125193389	4.544795695
6.780371466	4.188873605	5.185142693	2.81163834	5.002666116
5.247171638	10.47461923	3.072025131	1.049204157	6.05538225
4.302562268	5.673625483	6.098755757	4.15133325	6.721001128
3.26986393	1.699965465	5.094731483	3.092038223	5.189929966
4.717333525	0.859330983	4.849534127	1.406945885	4.716695039
1.289016497	8.689835734	4.126216231	2.370548999	2.372455045
2.542103665	0	4.537438468	1.548489785	7.199316381
3.275387199	7.480441339	4.969625926	2.908806614	4.811838159
6.068696502	2.925261278	6.591717895	3.051166361	6.177172903
3.884029883	0.828553747	6.179668738	3.889867459	6.573651602
2.306519724	3.30516686	5.191684279	5.304758601	4.626238515
4.605296643	7.065640366	4.207430982	1.94861978	4.72887423
5.225519485	10.29710747	5.90226788	3.369173419	6.45922916
3.120514711	6.888267499	4.867360614	4.708676263	5.416697276
7.150744113	0.01220072	6.367229242	3.228035855	3.645391108
2.375532926	0	5.249192065	3.484982264	5.289191407
2.410412119	1.440044085	5.124815693	2.411501709	2.410739187
2.830741949	10.75279275	4.572634712	4.630154976	2.164543179
5.951928922	8.206652021	4.845426857	1.809020663	5.426311839
1.645788802	1.049140575	7.724278105	3.882558079	6.209453472
5.034834324	1.080167156	5.801836093	3.57994046	4.448885519
5.433306719	0	4.561760973	3.577488643	3.473292642

0.292322279	0.302084685	5.131553424	2.526631534	3.654282711
2.0872594	0.709362416	4.829792934	3.082152787	6.666696319
5.964862633	9.058226074	4.491019819	2.050797817	5.744657564
4.815489791	0.442247678	6.208836519	3.896864693	5.80423153
4.608662352	2.125376064	5.544000523	1.09703509	2.721045559
5.141268249	10.65406083	5.963232518	3.264363313	6.61461289
4.923369399	8.231440496	5.441958086	4.005239555	6.780122696
2.858754206	0.435765965	5.676992178	2.1786302	1.660887107
3.731221544	5.241479209	5.424327884	2.349533892	3.937952436
3.955165243	0.054087239	8.249269271	4.249992555	8.086705876
1.225015102	9.285994049	5.87041754	4.651337983	5.389863714
5.380527781	9.830246083	4.794525786	2.176728875	5.255566796
1.294531027	0.096546367	6.256090358	4.593896625	6.203858068
2.041722156	9.868230891	4.509054613	5.177719337	6.050618318
4.142260306	2.120822034	3.725307707	2.448571434	5.761316284
6.624101323	0.08406556	5.092670095	3.5350427	5.394087434
4.643827629	10.05705629	5.636904399	2.522512285	5.656466101
2.199415306	9.099713091	6.897518005	2.528431063	3.930023478
4.97185001	0	6.670722513	2.421744794	2.238062798
6.151385268	1.562008138	5.024908976	3.062610422	2.310389052
3.3174679	1.217231765	4.795117941	2.285307651	2.892303473
4.122835527	11.4924729	6.395848156	3.726574157	7.362036607
4.598248336	0.042264085	3.659644189	0.038898472	2.505046777
5.075004883	0.044314878	6.775151425	3.790350904	8.054112828
0.166874307	0.171412615	5.311748612	3.242832105	5.185267456
2.415874916	3.911626019	4.965114546	2.90470057	3.688409588
5.332148413	0	6.314313776	4.369042982	1.922804868
3.566831816	2.857854634	5.887869248	2.741333447	7.590970215
4.913218939	8.954866889	4.591272176	2.005269315	5.621112974
6.77142135	0.339878277	4.378379072	3.715916132	0.333824264
4.460254601	9.297946771	3.419317695	3.120328928	6.714392398
6.4067312	9.526686902	5.130482284	5.232190609	1.278921121
5.610906489	0.017061705	5.041299934	2.397604547	7.154469956
4.137481913	0.12806152	4.886935368	2.485557585	3.107528601
4.899437789	8.728994401	4.797035198	2.494159437	5.389689402
4.423156516	6.274202524	5.312469655	3.094299988	4.423411108
7.877593698	0.06367272	5.212327281	2.257343143	6.752114796
6.203357893	0	4.466238911	2.254653608	7.160742723
4.270932705	0	5.718971141	3.755991957	5.785818822
1.818400825	0	6.781614841	1.817765812	5.480104019
6.28381358	4.818733337	4.901225071	2.922541473	5.484265039
2.028498919	0.393414545	6.035190099	2.741648972	3.607387312
6.617295394	7.973370252	4.362603634	2.182239444	6.120856408
6.808504269	1.950922539	4.547095344	3.232739983	6.084714661
5.808241191	2.931663875	6.867484207	2.51932221	4.626805531
2.359484355	0	5.512714645	2.523862351	4.715966781
5.732683259	0.088196913	5.734482079	3.705383139	5.110175017
4.155905994	1.863075757	7.08986661	3.795068443	5.000373776
4.997452724	10.47106935	5.92568529	2.796392192	4.605977634
2.785797716	1.341691444	5.730782294	3.65217809	4.089354305
1.356209523	0.818654007	5.95309083	3.704226493	6.650968017
4.214275056	0.47866707	6.689984943	4.413246945	6.305075294
4.941105125	0.124130653	2.476119242	2.810933024	7.56170987
5.547151495	1.673495009	4.760456749	3.183259967	5.425635353
4.219219601	2.005670044	6.76726424	3.294004997	2.456846996
4.900401197	0.113605548	4.902125851	3.473420013	4.756470093
4.386889755	0.490481258	4.996934489	4.57017486	6.486222647
3.573922344	1.403993508	4.770312392	2.070956135	5.932590488

hsa-miR-144-3p	hsa-miR-34a-5p	hsa-miR-188-5p	hsa-miR-542-5p	hsa-miR-195-5p
3.581363022	10.43052092	3.907247006	3.89151578	8.284724678
8.731466644	9.832949723	4.209627547	2.033791384	5.796128125
4.391862774	9.026444806	3.453370046	3.411373435	6.986317042
4.525001477	10.56150357	4.233204395	3.589295755	6.105236001
4.55105637	8.958828009	3.547419663	2.401942498	7.223451705
4.186226595	10.31230793	2.488849586	2.979116701	7.542361096
5.395370227	12.26964689	4.017389358	2.927052831	7.865106928
5.435268779	9.333703825	4.432614762	3.256727955	7.84530427
3.612615052	10.08672759	4.069157551	2.451376113	5.503431308
6.010542053	8.261371354	3.027066927	3.032805566	5.394244922
3.912397634	10.91422942	2.078958325	2.521537174	7.444819655
3.674026482	10.68275434	3.495854313	3.058333734	6.581003937
5.007246761	12.45387512	4.169843265	4.011392028	6.88422
3.159897795	9.85305624	2.930389358	2.924571083	7.46414918
4.197579949	12.26779702	4.409348959	1.546073761	7.242488421
5.719972294	10.9103702	4.882328941	3.395713356	6.898648375
4.979560392	11.90640497	4.253437452	3.326743504	6.492051555
1.32995207	11.15169563	3.749168935	0.791409582	5.233072182
2.134126748	9.73843383	4.913806769	2.581804548	6.473464744
7.270718731	9.933632692	2.94518393	4.528255622	7.967429342
5.49207436	11.61031003	2.293562765	2.998000682	7.340206588
0.13718452	11.32908742	1.415057677	2.071743077	4.049618722
4.338235223	10.78677096	5.17205079	1.846950167	5.741264507
5.618808125	10.28629646	4.09556027	3.691921225	6.445626266
5.231406451	10.25940039	3.244752046	2.604068363	6.127359072
2.912894863	10.91859244	4.740435804	4.37434231	6.972057402
4.229207285	9.547573648	5.674442992	1.631223038	4.703853186
4.478219385	12.16337463	3.861660767	3.405516744	5.725909268
3.270844926	12.08532917	4.18668664	2.573708646	4.371888132
4.107731106	10.53599425	5.105572103	3.661721899	7.183164806
5.276841748	10.39084193	4.865959396	3.580612127	6.751947536
3.344479877	8.398489823	3.635554479	2.74935626	4.181094716
6.140440856	8.279809317	3.308077228	4.117697316	6.006057171
5.04498517	11.00689214	4.348535025	4.208963372	8.125052749
7.680074993	9.749394091	4.042853405	3.595868227	6.975001783
6.29493111	9.898365239	3.880306071	1.939192952	4.755496921
4.844444797	12.86142495	3.493729718	1.354971157	6.391696803
2.896487067	12.01769833	2.672327882	3.412062087	5.931205847
6.70205503	11.00781674	3.564510825	3.697972183	6.526508958
4.30989888	10.42761692	2.119310661	3.718111424	7.253345534
7.407434301	12.04031392	3.848334985	2.775929049	8.516246718
4.209926083	9.747311335	3.84656249	4.038527574	6.17053907
4.21593978	10.92405914	4.434130207	2.891706654	6.384544398
6.93188319	10.52897248	6.386338767	0.18915764	7.927579458
5.337267462	10.38484377	3.708877538	3.26084081	5.211532794
4.505991257	10.21855336	3.673798532	3.569904731	7.326043097
3.408517457	11.79103589	3.046959725	0.272853601	5.578613641
4.002069122	10.20966649	2.482376655	4.225840361	8.201427075
4.796759253	12.29288644	4.884340794	3.353061287	6.725061292
6.115897736	10.93039907	3.490136786	4.916123849	8.257032794
5.147678047	11.63311089	5.855396733	3.929650397	7.321220141
6.917052783	9.611182762	2.417970131	5.103666166	8.912455473
3.944173724	10.14920707	4.190489336	3.308181871	7.351606936
3.301448832	10.627811	3.730091143	4.039946329	6.997549218
7.09192837	11.76356095	2.471671421	2.961535349	6.150144173
7.814585267	10.74611061	6.989370315	3.558104703	7.512443025

4.235750444	11.81432402	4.337644445	2.80311614	6.879338197
3.947279435	9.108488344	4.60715226	4.077151614	8.199830064
5.49797687	9.231517385	4.79256254	3.034963263	5.167728922
6.894439238	10.61675773	4.681721029	0.017688985	5.421207976
3.087510308	9.015160178	4.87490548	3.571766876	7.175017479
4.69357122	11.16872605	2.384751619	2.380310252	4.972267473
4.622878534	9.729101798	3.205700132	3.205085436	4.792957751
5.072042855	8.911738389	5.578421601	3.549272651	3.184972278
2.609581743	10.97047608	3.397714227	3.061960935	6.422981119
7.07901269	10.59186915	3.17195179	4.089516641	7.19202955
4.926929438	9.86721727	3.286323238	3.69950351	5.899240115
3.311191268	11.05402514	2.187467264	2.66747116	6.661673881
1.666352242	11.55535741	1.674470913	4.586582757	5.959374694
3.587701724	11.04424429	4.633990439	3.691846079	6.98754167
5.89104805	10.41503241	2.900418718	4.067370188	7.079988493
5.467776048	9.868014667	4.212288732	0.040422822	4.609249633
4.481109367	11.01013968	2.79837568	3.21597597	6.89835675
4.00948752	9.879086302	3.27892455	2.446362079	5.51585065
4.652371657	11.48219129	3.794276884	2.725460679	6.3928131
6.479710073	10.24434489	3.786941664	2.718618764	7.245945407
4.355566046	8.16997008	4.270654773	1.121739964	6.806351599
3.590188426	9.633944727	3.350579175	2.257395146	7.186085218
4.871066569	9.747783545	3.678734658	3.611418753	7.088420389
2.233396654	9.880031985	4.51823836	3.058165726	5.407838793
5.664090456	11.64352787	1.522908304	3.691526809	7.888129094
4.555667684	12.63796901	4.219159282	2.558856605	5.992470889
4.389387535	10.47942822	4.831391294	3.601064125	6.230700302
3.275553	10.01114688	4.10866359	2.093198488	3.566647934
2.890564122	9.636126266	3.313996755	2.996308797	8.208439457
4.528938142	8.547315542	3.588593996	1.732412466	3.270253816
2.040243918	11.323603	4.87976997	2.484851226	7.181068557
3.012647467	10.1340258	2.89201586	1.520280082	7.164251831
3.804474364	11.81578341	4.025711376	0.305963974	5.949530259
4.757233857	10.60881866	4.70275451	2.809476983	7.079214094
4.511690379	11.87079022	2.948991752	1.76988851	6.087568674
5.865397384	11.61640026	3.098987995	1.602546137	6.545582049
4.700998838	11.40448722	3.887274059	2.980325082	6.075993149
9.064471294	10.88016636	4.245222393	2.064204894	5.24762509
4.947861037	10.87525406	6.134979148	2.54003284	4.021475376
2.870982764	8.843453707	3.956876819	2.990869904	7.538513356
5.66653405	10.04375196	3.578662119	4.231607781	7.921893183
4.12639976	10.75073993	4.004752792	3.727646845	8.259753277
5.199842052	12.29684416	4.040941408	3.655396048	7.4668468
7.04425	9.420642274	2.093700654	2.09304048	5.018853025
4.887514224	9.909735042	4.606134067	2.090980923	4.190341615
4.034289003	9.204095709	3.306690894	1.367443045	6.416730606
3.889622979	9.88270119	3.568025115	2.306646629	5.596811406
4.514663578	11.07862462	2.411123172	3.286216866	8.15300178
5.950812884	9.793324391	5.524513562	1.803321707	6.413297076
5.962247909	9.992284154	3.758927662	3.573809686	6.292687131
6.272977442	9.226673271	4.705502511	2.052067042	8.434761244
7.477603593	12.57456672	4.656995682	5.102528597	8.867755813
4.932506629	9.462201428	3.273283403	3.515678107	6.706850834
5.370057187	8.474150365	4.213148908	2.833250278	5.307196136
5.26515718	9.200651935	3.861600682	3.505483788	7.974273313
3.535361647	9.760741986	2.880005418	3.533412851	7.447173885
3.291105048	9.830347024	3.119461393	2.433296376	5.521816016
4.300443134	11.31220155	4.9784949	3.374782447	7.850685101

7.013333022	11.33646716	4.25749125	2.521748482	6.198670472
4.459136781	9.285315717	3.932483868	2.329090244	8.6073954
10.04458412	9.984476199	3.158796527	2.522856789	8.28598953
4.201940566	10.49000681	5.435020181	3.417478067	7.996842923
6.322679687	12.0869611	4.671548099	1.701676884	6.545682192
5.502391012	11.28650525	4.736553204	2.424007237	4.819301122
6.703558051	10.62377453	5.149757901	2.406519955	7.56396329
8.789350388	10.96219093	4.193868253	1.852643852	3.770702428
3.19989221	10.97883851	3.731038206	3.199279564	7.192338025
1.168395438	9.505122109	2.553564346	1.789839906	7.074772908
3.918733638	9.318788569	2.292886485	1.855360822	4.143837791
5.671719643	11.15419273	3.521741531	3.192832729	6.423446444
1.935999781	9.489305038	3.219242472	3.58065315	5.973432796
7.45178655	11.54071879	3.748570782	3.216097254	6.510355297
4.313221761	9.800149394	4.311651264	3.595177832	6.639773715
7.432465808	10.01956102	3.170880942	2.331556527	5.605611872
4.474115631	9.349479887	3.658463932	3.744219911	6.308722561
5.324678585	8.7966728	3.02501233	1.769030139	10.04402004
1.788462217	12.30903027	3.091316378	1.786328849	6.915699589
8.403669369	11.59069755	3.914892881	1.556125862	7.390928231
4.981022592	11.11728735	3.488793172	1.850128739	6.297035059
4.121741976	9.390031103	3.244169148	1.454954635	6.770173244
5.418877606	10.09477016	3.519552286	0.038428083	6.953645879
4.019815178	9.849092933	4.382307491	0.040276187	6.936092643
2.173292531	9.644502243	4.40358661	2.963633718	5.047537842
5.236942578	10.28617817	2.415879533	3.908530024	7.318836983
1.92287142	11.12281259	3.495260433	1.922722046	6.870080723
4.138475142	9.491398052	5.565838765	3.898052023	7.098185048
5.215833116	10.11503956	2.005288281	2.327413566	7.415872444
5.773681287	10.95053361	5.299546816	1.90535072	5.146463461
6.8861931	8.897601215	0.208873792	1.631129004	5.657972145
5.528020491	11.56720842	5.195326586	3.599679862	4.747439412
10.78382976	9.979572352	5.173509328	2.662321938	7.789344112
6.066179716	9.241623361	4.949295295	2.038589717	4.959416147
6.213534704	10.17197322	3.912845107	3.062566016	7.156977326
7.170372941	8.55634635	4.744755363	3.264615825	3.907195826
6.924390243	11.474923	3.611914292	3.451804614	8.71269956
5.073245103	9.72751145	4.904516427	0.644091384	8.666699042
5.849657551	11.11422148	4.392473373	2.578070472	7.316339921
4.60817187	10.21148002	3.872142075	3.946015577	7.04465418
7.296829412	9.533802382	3.549461477	3.07347347	8.717161858
3.895484047	11.18259549	6.605385339	2.734318666	6.536630242
5.228855628	10.71743629	2.849992184	3.171263112	8.062755413
5.221078431	10.60077433	3.436831371	3.230398727	7.832929582
2.518949042	11.05916202	3.252107768	3.253286827	6.31711741
1.088303705	9.79095001	1.731393428	3.494797066	7.018213222
5.053467177	10.69929225	4.204159873	3.703004312	6.416491839
3.138800364	11.97497423	4.044975202	3.792977488	5.057955674
4.358244058	10.9065749	3.156198732	3.645843455	6.960082844
3.060202752	10.34596958	3.288784397	3.649116445	6.409144273
3.606291871	11.05144182	4.501819517	3.514668996	5.835290592
5.310129325	10.32295904	5.045378481	4.030088341	7.500947166
5.197636453	11.9533559	3.694627284	0.111041631	4.546616452
4.761555881	10.04558834	1.687855762	0.21306818	5.781752133
4.329426203	11.14823782	2.797320401	2.451908443	4.329456584
6.351738725	9.859326588	3.053492929	3.044649272	6.104479958
5.583816932	9.640418717	4.207764899	2.68261903	4.384967952
5.858787763	10.70842595	3.900335609	1.413172927	5.781877033

hsa-miR-1266-5p	hsa-miR-326	hsa-miR-891a-5p	hsa-miR-335-5p	hsa-miR-501-5p
4.854690489	3.378654468	3.148904064	5.00210498	4.048119184
4.362104664	4.127132738	2.35735598	4.432616288	3.208170779
6.102416766	3.659819545	0	5.784352309	3.753592941
5.29181614	4.232953905	4.355887222	4.295051077	4.358031024
6.485416426	5.551091988	3.547927303	5.707679055	4.550022925
2.488890275	4.272386044	1.120300348	5.319492294	3.348637545
1.769743932	4.019062405	5.601497284	4.366225947	1.773578476
2.726010394	4.945681049	0	6.23772054	3.874765172
5.431755464	2.948280013	0	3.318088393	3.932734008
5.6039027	3.262553247	4.825245253	6.477433732	4.674941413
6.422874105	4.768612999	0.138534296	4.692597024	3.37759199
4.424712169	3.067929231	1.350651319	5.852686094	3.965724961
2.734262637	2.925899041	2.925885169	6.204496607	3.246372508
6.025254163	4.175208143	3.685926343	5.997165898	4.369433414
6.662653977	3.410814502	7.455751714	4.704006563	4.120119756
2.706217503	4.117243478	10.65983382	4.120935649	2.368945125
4.915846679	4.885813512	3.213564052	5.042594594	3.89455164
8.30483164	4.513467124	3.747077365	4.898420525	4.975592723
5.587711838	3.817776264	1.461808483	4.761366944	3.642382992
3.265816475	4.642109686	2.945135699	5.07000143	4.264030195
3.539393218	0.480188149	0.514356254	6.084250153	3.884014588
5.967982336	2.525941381	7.72807789	4.851587352	2.525857729
5.098149639	2.418538717	6.253056026	4.064077531	4.339944717
3.928413085	4.524169116	2.384848463	4.407209678	2.389158531
4.419352102	3.244608107	0	5.553028961	3.589327344
4.857336122	3.830959412	1.213060698	5.26690805	3.730688191
6.568528851	3.865518176	3.056043865	5.104654501	6.892872517
4.702875735	4.692737678	5.371982933	3.43329663	2.336309894
5.611146687	4.530268106	1.809429325	5.991261239	4.927796907
3.972063175	4.855995822	1.32512049	4.852742231	4.894513299
0.141555797	5.266191809	5.334477242	3.599872501	3.595660595
4.827240934	2.1555086	0.036961434	4.08734959	4.350035043
4.229190141	3.308342442	2.612976878	5.540087688	2.884197706
3.837006492	3.161319149	2.093795773	6.344960655	4.568720134
4.784075194	5.303622873	1.659956111	6.123585174	3.976174198
4.83242023	3.470725136	12.40141703	6.59590922	4.01606207
4.321520088	3.494244863	3.671765115	5.047074824	3.49402715
4.903975958	3.41390028	1.060705692	5.097077758	4.000905684
4.250402364	4.154822052	0.052201536	3.043622024	2.816415717
4.896199428	2.119314678	2.118529178	5.464550021	1.695066702
4.510244405	3.090132691	4.797768859	5.038357169	2.94031857
5.768768623	4.499320466	5.040732881	3.037846509	4.287626989
4.636851933	4.326517135	3.789507783	5.168371244	4.208599989
5.190789147	4.430872263	0.213341766	3.633850769	6.536720139
4.317392588	3.885205673	5.663192899	4.436849381	2.197658919
5.070714414	3.673677846	7.242460567	6.319097738	2.681096744
3.930139504	3.684252915	4.867779429	4.615359126	3.047560852
4.550862863	4.941736986	2.031100669	4.640544984	3.097688418
4.398655244	3.928934228	5.036760807	4.707236369	4.563258218
1.693311042	4.1033724	0.24530025	6.987343914	2.860238789
4.667299375	3.802844695	0.110543431	3.946854547	4.070212969
3.579906405	5.907011391	1.271921009	6.233892072	3.050969685
4.538087188	2.813519883	3.026101649	4.352148195	4.233840535
3.535193346	3.020043111	0.183248396	5.947526915	4.062446458
3.865616926	4.165669135	1.716035879	3.749511592	3.748412782
4.677100307	5.719664926	0	5.056472532	6.740062538

3.517798089	4.533475235	4.540451623	4.235216856	2.811455928
4.484944114	4.643630212	0.640149227	6.399459721	3.827630859
5.286228277	4.944692058	1.614024252	3.496061641	4.359361293
6.121534621	4.3442268	2.081961834	4.621321887	3.902499313
7.131421961	4.321794254	0	5.687886014	5.372192718
5.202775527	2.384850507	4.24767225	3.225925072	4.017502843
6.981014962	5.24724078	3.737348253	2.842327489	3.94381207
4.212417339	4.165879795	5.782600548	4.260038562	4.906700291
4.482104289	1.958865437	10.50508296	4.929206611	4.532075396
5.172087221	4.324044147	0.000410991	6.757076411	4.172032759
3.724566139	4.547221694	0.17983573	5.283696745	1.529372644
6.543272244	2.187458107	0	4.369467293	3.756482671
4.937189323	3.899346516	0.236179146	4.742764246	4.3732376
4.168572948	4.361700079	0.864687953	4.359933688	4.026401932
4.846827599	4.980812813	1.911742573	4.799419701	4.594386469
5.834194847	4.118931436	0.044189228	4.302394538	6.217106929
4.480468787	3.024749434	1.150471075	4.480835666	4.317181226
4.944403799	4.473541402	2.456363088	4.008636091	4.719658458
5.528820619	2.533930647	1.756070311	5.625813208	3.516812212
4.691181066	2.527156254	3.60652843	6.480474497	3.50955457
8.102971006	6.49109762	1.119303563	3.76680383	3.639145791
5.875162249	3.072487833	2.724277676	5.963470637	2.265608536
4.806014256	3.246902514	2.630833226	5.976764553	3.67847943
4.07707732	4.175489049	1.799949027	3.725792739	3.967278188
5.451476931	3.511386081	0.177346851	5.274976938	2.199079927
4.725579598	1.975517888	0	6.099506127	4.363441173
5.459753285	4.172191936	6.781623069	5.409358414	5.105618363
5.504539444	2.420045068	6.0675489	4.696201317	3.275170186
5.928921911	4.868280969	0.491061187	7.567502676	3.238435907
4.578331679	3.680418411	0.806428037	5.367980241	4.429558346
3.539555426	2.832314417	0.127457568	5.858597276	4.72694634
5.849189808	2.759162839	4.716079989	4.680715148	4.825701123
1.950127291	1.953690282	5.379364912	4.425538436	0.333635291
4.909235329	4.757207289	1.001451286	5.461796535	4.461739115
3.306838047	3.301585725	2.95064892	4.209130191	3.582773751
4.153111673	2.752033553	5.563728754	5.17722417	5.428772361
2.401761354	4.046673761	0	2.620133363	3.887161467
4.245847473	5.661714781	5.435369192	2.65449584	4.719874301
4.739200158	4.985357764	2.141700483	4.70030489	5.078320333
6.451914933	2.982128573	1.057669486	6.815371467	3.585407833
3.768475339	4.520420112	0	5.825953004	3.677271851
3.411589779	2.373552662	1.929063473	4.008786159	4.749447062
1.548686996	1.548518168	1.548915364	4.412059557	3.125091252
5.397324269	3.566043967	3.428295041	5.321538712	1.671363175
4.983585039	5.132918399	4.750954208	2.790765904	4.246381402
5.612684921	3.7169668	1.763916679	7.121271522	4.10632981
3.683326875	0.98893867	0	5.474043385	4.304603337
4.880944633	3.574085087	4.60400513	4.806716875	2.197816524
6.189698215	4.514333804	2.47886227	3.998028762	5.665353713
4.293293511	3.193260229	1.611941609	5.446974672	4.091601057
4.14893153	6.945157806	0.012127184	5.791101423	3.519181574
2.875392676	6.419108311	6.168666016	7.108090449	4.656488497
3.272573131	3.512135352	0	5.326142841	3.618340774
5.570761723	6.324920588	4.816744301	9.071479885	3.285691285
3.005208868	5.09052173	2.445829745	5.0287759	2.606456737
4.602060646	0.014912705	0.015397228	5.123699523	5.032466811
5.486195471	4.770228023	0.025293724	5.338202174	2.434516943
2.847703513	3.751448265	0	4.561911766	4.657584183

3.013528626	1.826965098	3.892079116	4.933015463	4.08177251
4.247548094	4.253524647	6.587436039	7.045645896	4.195032155
5.003680796	2.874242089	5.51164062	5.745084976	3.396255138
3.92434433	4.503443833	0	5.259819859	4.748876175
4.318232518	5.055848297	5.611536103	3.140950148	4.234308545
4.816949859	3.79908216	3.802745389	6.147708579	5.623187199
5.266687915	3.606851382	5.915116575	5.523276164	5.149298336
2.441175129	4.805991739	2.947954308	3.770236065	4.264270144
4.786548155	2.349718242	2.836499467	5.617003812	5.35348591
5.584897077	2.82283056	3.245951083	4.257590818	4.57882965
5.804801752	0.06958668	1.854065097	4.338292465	4.140580289
5.297982268	4.375549514	4.530555472	3.661838242	3.903368108
3.748261866	2.717633483	0.095848837	5.575230236	4.129672784
6.094806374	5.093932333	7.94582422	4.573228819	3.748598594
4.603341913	3.947903248	1.696531015	5.979486162	4.229024602
4.473971457	0.080441223	0.083473122	5.250780358	4.382198815
6.875470993	3.807756334	2.829962449	4.99403589	3.491240334
8.096491166	4.035940627	6.854751631	6.32601007	1.153137
6.55072481	4.788978136	3.38079075	7.567677883	2.979286563
3.70382308	3.548384086	1.855919001	5.025212824	4.152684678
3.784692673	2.891957494	5.733073265	4.13727611	3.909613965
6.528519619	6.088152505	1.46058141	5.233732975	3.649046512
3.786101035	2.999220942	3.902762376	5.378817265	3.658357605
6.559323081	3.528803696	1.753249842	4.972127531	5.259633765
5.247013449	2.632277669	1.495791445	5.586566599	3.686196549
1.6674763	2.089891347	1.067182039	6.258068681	3.687747438
6.475072293	5.789116698	2.913081133	6.171861825	3.908922248
3.881305654	3.569343025	6.742725132	2.96580689	5.226877346
5.135632354	2.813167391	6.960656617	5.135645089	3.591041787
5.231852072	2.633584813	1.902592403	4.809248409	5.804601602
6.146603621	4.874416074	4.034362714	5.944965736	3.647592215
4.534494602	5.666313419	3.296577714	4.666257988	4.391609536
4.407825364	4.893272362	1.054389523	5.895140317	4.786182523
4.465052681	3.709667258	0.127078844	5.025434449	5.203139589
4.611970471	4.094457854	3.166946639	5.48008961	3.70421008
4.560994324	5.856916001	0.020076663	2.677438852	5.494069318
2.862502372	5.659925683	4.695467608	5.259575421	3.612085942
5.797539221	4.141892093	0	5.710193493	3.913914829
7.341122764	4.654159051	2.1035209	6.548233335	3.839142753
4.889621925	3.128463068	0	6.063650212	4.053735106
6.014789128	5.125748515	0.841177526	6.682258254	2.920078904
4.514151982	4.319552083	2.020205813	3.894496883	6.265173693
4.010677671	5.361990029	7.541783856	6.507880296	3.305201525
3.011272282	3.437262016	2.397260341	4.783724615	4.359158973
4.880433367	4.784925802	1.939219935	4.319232532	5.056043212
4.904186897	4.159622754	4.000176287	7.693389878	2.519913354
3.560000215	3.187336231	2.685795178	4.574990194	4.399021191
6.290135296	2.304261877	2.638083722	4.156460476	5.106819101
3.879004824	5.07025963	0.227289964	4.057264794	3.871507108
3.664456057	3.956922041	3.059909261	6.355880417	4.67638074
5.495523051	2.310759445	2.715441507	5.242853054	5.713200224
5.201410696	4.18000532	4.357823689	5.844203391	4.057042783
2.818096643	3.32240611	2.816863637	4.717741867	3.09177833
5.547793452	3.714412307	0.241543443	3.721981945	3.713879756
5.619966863	3.302194175	0.117695798	4.102568448	6.254992182
6.158885601	3.481983723	1.991791052	4.593578892	1.345163034
2.782604695	2.427790197	2.134782799	4.777442848	6.183065044
4.596419297	3.371858144	6.990732884	4.503015543	4.286471128

hsa-miR-34c-5p	hsa-miR-6503-5p	hsa-miR-9-5p	hsa-let-7c-3p	hsa-miR-130a-3p
2.528364061	0.077170605	10.02185032	5.615023827	8.981107416
1.025753643	0.004863409	7.721145953	5.947998757	8.6001168
3.147133666	2.202081828	7.250975048	3.95796968	6.55449863
1.810926718	0.870190432	12.59028256	6.051869197	6.944210764
1.656286912	0.011083813	11.95934219	3.409820199	4.847111575
2.488512313	1.109701981	12.51331063	4.435276307	8.136583102
2.476887895	0.138914316	11.54467538	4.366326531	7.573538644
4.35694384	0	10.5242586	4.030922704	8.136455343
0	0.779248816	9.004767474	3.930287705	6.086400272
2.395818098	0	8.137509855	4.007523886	8.455142873
1.418340379	1.35948493	12.00788788	7.42692957	9.158528481
2.007733285	2.693044461	9.836358256	6.555400122	8.695180475
0	0	6.562373606	5.873425448	7.361191224
0.076097184	1.221370335	12.09026386	1.248539674	5.080443433
1.546011528	1.550873492	9.322644263	5.234124985	6.439846807
0.087618571	1.251958644	7.842133128	4.596187946	6.724644147
2.699989947	1.247731392	15.81215158	5.131314096	7.693032603
2.492919125	0	16.16824693	5.797194013	5.171956971
0.147581294	2.033859923	9.002771869	6.858372796	6.306475987
0	0.966285736	8.749908989	6.297680569	9.005481191
0.464659756	0.22222052	8.039634642	7.348518524	7.902714585
4.837908692	1.986952215	11.61824887	3.145380596	8.106544857
0.885744845	0	9.319934291	3.520205701	6.038836625
0.226204371	0.125058456	10.89377955	7.567166492	8.123741217
3.369819417	0	7.860881932	3.243944234	6.61457184
2.611194819	0.465826957	8.878824428	5.013383166	7.757443062
2.862367215	0.007202628	9.326176825	2.374478251	6.968302503
1.645092235	1.53695333	11.87080436	4.705055973	6.995343403
1.811717188	0.035331475	8.04353735	3.270954211	6.703277315
2.017821477	2.538389315	8.643236141	5.422315774	7.506059931
2.545429636	1.371432476	9.531691765	3.775354544	6.666061126
2.976579403	0.022367827	7.130859813	4.768512283	7.417314519
1.844641793	0.040478732	8.522289486	5.083714932	7.921714501
3.971851128	0.692076142	9.025071586	4.643527518	8.816846855
2.211035834	2.266252881	8.380798377	5.570336118	6.999896854
0	0	7.830982828	1.637832914	5.472443435
0.109400259	0.065701716	8.492771686	6.66027223	6.962114863
3.413424383	1.649677442	9.246691632	4.797078284	8.389304415
1.785119777	1.148163658	10.35486899	2.215580222	6.599861332
4.307511296	1.081892874	14.41744822	2.938131821	8.96554189
0	0	11.31745104	5.110266442	6.016523209
0.007452681	0.004851024	7.842473072	5.94789453	7.678160315
2.107591185	0.08160402	10.47485024	5.736863457	8.019172409
2.303372056	2.592422272	8.497590105	6.814501361	7.595212531
1.522251511	0.095505933	13.9025992	3.514289318	6.154195674
0.91464807	0	7.652557136	5.320022318	6.39798942
2.57178547	0.153461682	6.846958533	7.602750831	6.593465856
0.11758779	0.070188826	8.437954823	4.001731497	10.0992109
2.109358189	1.824535575	13.48075768	4.398332497	7.294027902
0.228091523	0.125942335	7.742450435	6.768201698	9.682247721
1.339563328	1.918750085	10.26378509	7.452569502	7.907567447
0.745743606	2.988612549	8.04047121	6.069518504	8.885261877
3.12495191	1.715715003	10.64205606	4.982022573	8.265982744
1.539104183	0.098470562	9.046643557	5.35778719	8.593227299
2.963508116	1.098405681	8.654339221	6.439005068	7.63353101
2.260493538	2.719780107	8.142365904	6.615137764	7.81790867

1.371007444	1.320932826	8.700778259	4.539548742	7.536617545
3.379652849	1.844876294	9.716129763	4.905399528	7.361323657
1.024175466	1.021930181	8.486272103	4.622964626	6.608818127
1.661089524	1.05666508	14.02851342	5.684127051	7.357975714
2.662831337	0	8.657433369	6.369077023	7.981348467
0.091719289	0.055824096	8.903680033	1.941020565	5.846874517
1.024092216	2.026842929	9.953495067	3.035504747	8.8301913
1.484760078	3.074779041	10.06243647	3.882735236	8.104982638
3.589020152	0.764180928	12.89338359	4.528480985	7.7917928
2.809326755	1.586404755	13.43293289	6.956386617	8.699180313
0.168406193	0.096886589	7.929706462	7.127697208	6.474865037
1.467535532	0.911997855	10.20337997	5.269911917	7.364566427
2.367607704	0.122055102	9.593599024	6.313355773	8.665968977
1.41482076	1.433670688	11.02667956	4.773176865	8.034431888
1.503317409	0	10.1875771	4.846635478	8.576267592
1.141702481	0.026573867	8.679234204	2.183961116	6.895193992
2.199059456	0.02872032	10.84837547	5.236066745	7.92757088
1.756260166	0.135962157	9.826264141	3.805624205	6.879465504
4.098256182	1.383848947	11.73756898	4.506654816	6.714115383
2.882553976	0.827038863	13.24513992	6.162131024	7.677487841
3.174766028	1.710384342	11.68202266	6.95872215	4.508271051
3.068347419	1.488952255	12.0930858	6.157472148	6.572320113
2.074727435	1.198369481	7.129307802	4.544462617	7.775666274
1.802160145	0.033837887	10.4500981	2.233849378	7.102458065
0.166120233	0.095726307	10.91984946	3.515879778	9.133568775
0.981558568	2.300574321	7.327438313	4.363178889	7.858600873
1.96466538	0	11.09861143	5.069725446	7.155630392
0.020916285	1.06444234	9.183528387	0.021794608	8.944301955
4.136878897	0.500186285	13.24676194	4.586113254	7.956056403
0.802821739	0	6.740074802	4.839486797	9.350031056
3.334861487	1.334404655	10.97463111	4.88802859	7.85908855
6.353447459	1.57060881	10.96898522	3.865334769	8.400811238
1.952853887	0.16873569	12.48658912	4.246851549	5.621786828
3.002175043	2.001657931	8.657046227	4.250245126	6.046781533
3.294706845	0.139074952	10.16340898	4.868374129	7.326813913
5.1542905	0.109949791	15.37251834	5.102235854	8.494788977
1.430154209	1.447559031	13.74489679	4.549796251	6.877089078
2.064590562	1.044388186	12.6670722	5.832761417	7.899169875
1.59220099	0.721339355	11.22737266	2.140598628	6.31441412
3.2742661	0	7.275163522	2.981096951	6.346783887
1.805413351	0	11.36372843	5.009578967	8.252479612
2.70917373	1.874618125	8.424473958	5.079268088	8.852046678
1.548469327	0.972720729	11.15055748	5.706649126	7.140968191
0.020879998	0.013440886	8.573903301	5.469825071	6.88210718
2.482760829	1.208980375	8.881832906	4.136850671	6.934272638
1.367441295	0	8.936360884	2.072202318	9.533482385
2.983632547	0	10.19371924	2.983539707	6.85230585
0	0	8.511271296	6.153593188	7.738997417
3.033799786	0	13.84725575	4.405380425	6.359009306
3.051134443	1.460158445	8.611158244	4.085369531	7.8859161
1.633264507	0.007502479	16.86398203	1.632899086	6.490073159
8.876321878	2.770020644	8.803210708	7.211543345	8.819507895
3.273955156	1.860935512	9.739653751	4.803864569	8.577084792
2.164080755	1.870325839	14.36840454	5.275389804	9.012149341
2.052794884	2.124032985	7.8289404	4.298741521	7.040271368
5.363246824	1.045529753	11.83369882	5.505918696	7.987915414
3.581221569	1.073962013	15.46905672	6.469625864	8.497656629
1.718973294	1.75417507	9.372796234	5.458363122	8.241768331

3.635995857	0.147871205	8.445682961	6.734208446	7.751044054
4.616938512	2.081344009	9.495402186	5.555410945	7.225689333
1.741429089	0	8.032055345	2.304449799	7.992134989
4.352479115	1.759590916	9.832077814	3.879042408	8.537954238
3.139902414	1.087001161	17.17515908	4.235645438	6.908624759
3.039857285	1.91619193	9.313789197	2.768616062	6.34077822
2.842169587	2.087171915	9.348318946	6.031871104	8.191688131
1.430308029	0	9.641456069	1.436263068	5.821313262
3.489603675	1.018239986	10.63721651	5.646762996	8.015550657
4.899896851	0.032045599	8.448673666	1.168978922	7.989414841
2.626147611	0.042362421	13.82701642	3.497021809	5.516879398
2.506967312	1.725245761	12.79311035	5.420769559	7.37489197
3.217427559	0.055177918	10.02781767	4.133796058	7.067980456
4.508197313	1.028717692	12.51260817	4.634695001	7.399862805
1.697783885	1.083848466	8.596005527	6.100918068	8.251994609
0.07898986	1.229116454	10.48660459	5.092408024	8.407513373
4.184640037	1.236240144	12.98842445	3.655737039	8.354972197
1.769750408	0.028774904	12.59342733	4.693882276	6.840649469
4.277813439	1.162926014	11.74166189	4.120152966	6.876887112
2.0990134	0.696668202	9.384312175	5.415341964	6.954683774
0.066953944	1.196587196	10.01184924	5.894669028	6.883366745
3.246153357	2.062855316	9.91710705	3.497652923	8.311485376
2.17554513	2.735943195	10.71356786	5.964857168	8.432266698
3.007205817	0.026480385	9.668379757	6.94835786	7.117013199
2.175942335	0.092271909	8.634446871	3.867751478	6.261575255
3.270290053	1.06171936	10.1666435	5.317023242	9.197246045
3.082382505	0.946651477	12.87030077	4.299558743	6.133418302
2.302586774	2.213124292	9.186286219	4.650949245	6.449066354
0.001154277	0.000755297	9.512324634	5.621104614	8.377186247
0.309603806	2.38464368	8.044744215	4.199022547	6.759817726
1.633725989	0.114970086	12.52530873	5.900389575	6.936463099
0.751887589	2.488042978	8.911955109	5.303280447	7.045048033
2.072840075	2.06120056	9.40216011	5.788171861	6.883186488
0.119684713	0.07132939	7.84865839	4.649262393	8.414914788
1.553977507	0.695592899	9.009750377	5.48003516	8.572055368
2.085939858	1.653401523	10.0477892	1.064585717	6.496749928
3.992808811	0.03741399	11.9647271	5.212118518	7.288747516
1.505986653	0.663416281	8.031139452	4.18980892	8.056077788
4.556754777	1.414657368	8.89517877	4.070252438	9.313596972
2.896407452	0.674338539	7.888477672	3.418606561	7.943327942
2.091780604	0	9.005722903	4.590753771	6.906125415
0.35607154	0.18127998	8.50039752	7.685249169	7.461550395
0	0.908157208	7.936971114	6.390088408	7.513395197
0.095424549	0.057915736	8.826035473	4.987152366	6.950398908
2.257796929	1.535616155	9.003655072	4.511553485	7.531731483
3.894098773	1.119955541	8.315825992	5.042160361	7.404875174
1.272914886	2.284517652	9.680398146	4.208391699	6.844060625
2.637827797	1.208115495	10.50491933	3.664244664	6.471818685
2.342828841	2.185899248	10.34519646	4.057323308	8.458742397
4.197639448	1.928241497	11.14432943	6.01388591	9.564441276
6.474110644	0	13.34218808	3.784843007	9.231273521
3.284570839	0.939483087	10.25210417	4.426154534	7.75759864
2.475644432	0.069359216	10.11716429	5.84205528	7.223243888
0.224689281	0.124347266	9.929635268	6.618230307	8.30227015
2.011362691	1.31184121	10.07962892	4.850161258	7.866078412
2.439564123	1.300257	9.110793549	5.467950286	8.252493297
1.252991738	0	8.286165208	2.674207672	7.22919517
1.413695855	1.355725527	11.86927566	5.818462072	7.262428767

hsa-miR-589-3p	hsa-miR-30c-1-3p	hsa-miR-190b-5p	hsa-miR-3200-3p	hsa-miR-3144-3p
2.076631344	2.073468456	2.853483373	1.417816347	0.107620201
2.033853365	2.843951379	1.616045041	1.616237068	0.006415061
2.759234311	2.219902662	3.301244328	5.989760543	0
2.789314901	2.125116762	0.85938777	4.526875718	2.390040954
3.54601007	1.058921306	1.655682903	3.673581073	3.250146459
3.174388511	2.158053255	0.033718321	1.732650178	1.120438486
3.279243766	1.766042881	3.254961218	3.301638708	1.732782138
1.91094873	2.736645098	1.388289847	6.440218511	0
4.07543763	3.433016559	3.43590312	2.79980996	0
3.730937558	2.186156151	1.250083346	6.360157733	0.732155985
2.075907651	2.85758331	0.119700672	2.871350926	0.107451375
3.486789325	2.445911096	0.101287112	2.793883405	0.091240799
4.315475671	4.383422973	0	1.524245414	0.950785675
3.349712443	2.318919944	1.881529855	4.765438896	1.872259481
3.274331762	2.953600972	3.952314801	5.563809215	2.763576408
3.864094984	2.968821389	2.360542074	1.286944959	2.678721975
2.14088936	2.541244692	2.543756602	1.215661146	3.54134178
4.568842637	1.326991973	0.791330084	6.205098535	5.491630181
2.583852265	3.949248819	6.819874789	4.115475257	2.105349017
3.266793813	2.946253803	0.963050635	2.270497287	0
3.006717113	3.454522675	0.406625717	3.043934598	0.346660911
4.748554291	2.515658336	0.118904045	4.500006932	3.322781132
2.638900846	3.525434234	1.847918327	2.160586598	2.646974191
2.380234333	3.45258465	5.21076536	3.486227999	0.180993652
3.691511805	1.415485537	0.861964461	4.682681545	0
2.369539299	2.730353577	0.871443201	3.564445425	0.876045555
2.374167821	1.631129038	2.049581511	6.774395168	1.629854182
3.409876001	2.778441263	0.189818957	2.336552964	0.168175019
3.591341988	2.241471272	2.570663745	2.577169447	0.047706837
2.01878452	2.993862199	2.491802365	3.900225212	3.268395797
3.583551028	1.431640318	3.372450605	2.095938759	0.111368655
3.632546761	2.153703889	2.152834276	6.353953156	2.74069656
2.880330024	2.276082539	2.606071068	3.11211818	0.05487404
2.488340424	2.655046286	2.097111454	3.265251205	1.181468714
3.298956134	3.188565653	1.963959682	3.588142677	2.442472163
2.189224872	4.693951172	1.637829415	0.736116789	0.73530135
3.058254917	3.478909453	0.100807624	3.669017116	0.090817173
3.550419888	3.549457574	1.65930954	2.081285148	1.060956275
1.166754699	3.55770971	2.21214559	3.043184778	2.533337248
2.711701728	3.300189206	2.709769301	2.446621009	3.122466788
3.573843915	1.400996933	0.850330493	2.110156426	3.105291723
3.359810812	4.208299092	2.033433642	2.03391094	4.205983981
3.925964126	3.589219548	1.440548916	3.610448917	0.114193927
3.099975719	2.747154632	0.18099336	3.115344053	0.160617619
4.882887328	3.486135546	3.478479232	3.510664543	0.135093891
2.873218302	4.190354221	1.480664676	3.567178695	2.207483298
4.08897889	4.400890782	3.85633297	4.301528216	3.284785664
2.816096562	1.379085033	2.469959849	2.482690437	2.784638548
3.57279766	3.468880032	1.799484286	2.365367025	2.118109734
2.846241545	3.458418691	0.206505307	0.234727153	0.182397768
3.637970787	2.761791993	1.337887472	4.071198211	0.08672506
0	3.499234777	2.926309583	1.651455097	0
3.46247508	1.361199979	1.362265441	4.962016775	1.659371335
3.008262928	2.20679994	3.498202253	2.216866792	0.13960758
2.142602396	3.743605415	1.109698359	2.143417008	0.027527083
3.2389778	2.833601708	2.265167357	2.260105029	0

3.504974446	3.498845879	0.105515392	2.811862065	2.440187165
0.632263132	3.389759768	2.586041988	4.48812488	1.795781285
4.560326184	1.614245827	1.024204291	2.61935077	1.023868948
3.551790885	3.550813251	3.549965212	3.795392888	3.67147693
3.00062402	2.237237484	4.849219576	4.89972748	1.631422872
4.750765306	4.00251264	0.08474052	0.093804037	2.962967088
4.284418061	2.841602124	2.35457261	2.031810587	4.788828835
4.454048888	2.416147119	0.629180687	6.288746183	1.4954725
4.048038698	3.596708099	3.190050011	2.773806585	3.616241995
2.809311148	3.586976908	2.586857714	1.001352996	0.000337764
3.27352902	4.022737595	0.153817762	4.193604417	0.137193465
3.177139507	3.661313534	3.662594563	4.095810105	0.905220069
3.166686907	2.354223352	0.199264206	1.673912589	1.643141268
2.96053287	0.861601442	3.114137856	3.368176632	0
3.358571489	2.710876322	1.912059921	1.503481674	0
3.525878093	1.754581439	1.141577331	6.817923867	0.035640868
3.021731784	2.196822599	3.384250613	1.7692474	2.18893935
0.239633378	3.756186134	0.22545209	4.333935248	4.05447067
4.03172007	2.065193343	2.322015397	1.75407033	2.330016569
3.612408071	3.794430212	1.355488954	0.815227339	0
4.085667073	1.121665129	4.983773045	2.980742651	2.968153001
3.059173847	3.557060313	5.519029756	1.581162244	0.15120397
3.036332742	1.831740392	0.662715725	5.247579414	0
2.832786051	2.562086709	2.229986248	3.585323216	0.045637611
2.647075168	2.189637109	2.185321262	0.170470623	1.503551784
3.431809179	1.56252582	0	5.292629222	4.617551439
4.450841066	0.751488929	2.788977046	5.361749301	0
3.690376622	3.425252457	0.019533682	4.431689926	3.419587047
1.963452654	2.572333604	4.029426014	4.431486906	0
2.511276105	2.296481619	0.801962547	4.717068328	0
3.098773811	2.482362183	3.51390755	3.338525291	0.099305685
4.115740753	1.149910267	1.522016723	5.22906966	0.654866407
3.757637759	2.646522948	3.444481943	2.678065363	0.252607341
2.587053423	1.586757714	1.001480916	3.324155253	1.586710725
2.932545282	1.766968426	0.231421926	3.584777731	2.410253283
2.284316521	2.733229134	5.919871844	5.012109421	2.24347489
3.710872464	2.981250283	2.146877804	3.502543922	2.151985207
2.877377452	3.879753761	2.65278594	2.389539913	2.061705539
3.325684066	1.214685123	0	3.894387147	0.704973907
3.09258714	1.935206609	0	5.939844558	0.577782652
3.236978322	2.3765413	2.120300576	1.805536891	0
2.705799508	1.928983619	0.082076853	4.122910615	0.074208188
4.124159894	3.540361224	2.543049452	1.548536207	0
1.671283826	3.272418355	0.019499902	4.695058147	0.017846062
2.648907156	1.845043073	1.846671904	3.766485679	1.179225232
2.900744762	3.052632649	0.823673084	3.526017475	0
2.983774545	1.571469142	0.988881301	3.790242896	0
3.898668087	2.916599309	0	1.947764854	4.769858698
3.36988693	3.045162833	3.4542543	5.471807402	3.334826423
3.570647169	1.800686146	1.97555355	5.455374475	1.995812513
2.864167296	3.643373677	2.639993195	5.869839233	0.009916137
0.5232827	3.419712946	0.522024563	2.517468041	0
2.820750849	2.631793936	1.840457475	1.838936189	0
3.000949232	1.447432612	1.447779915	6.747390235	0
2.447756846	2.894060518	1.516321111	3.005964051	1.823335489
3.659577029	2.065641254	2.389849103	5.570671124	1.049669342
3.441649332	3.11675544	0.02248457	2.107738624	0.020565535
1.719411044	2.02843536	2.029695141	2.996844451	0

4.382711274	2.515694161	1.812437954	2.53929476	0.217826332
1.639565102	2.952611776	3.813099189	1.951806385	0
3.162321891	3.288786666	1.743548216	3.158427636	2.891859185
3.476939177	1.442860998	1.869844482	4.79649861	0.427524529
1.701824351	2.125369916	2.124681589	3.465126013	0.024268662
4.280405378	1.336114861	1.97833356	6.140590395	2.40189692
2.713505107	3.18859597	1.773862323	2.220084251	2.739423531
1.655962619	2.874077506	1.432732992	2.765597746	0
3.351601266	1.020144441	2.835734164	3.352086387	2.025397852
3.413322949	2.219307946	1.170945032	7.132115508	1.167860302
4.65079647	0.06468997	1.226525398	4.862512845	1.222059445
2.176621125	2.504336984	1.136280875	2.775562251	4.272962658
3.212747102	1.296763718	3.57341487	4.238787102	0.075653196
2.852705049	3.368138506	3.50546115	3.632979462	3.043247815
3.595762159	3.303277	2.120170294	1.69765088	2.442204463
1.895135393	1.893587983	1.892007159	3.171467989	1.254592224
5.157708725	2.837607452	1.580585221	5.580871311	0
1.769297677	3.020114852	1.153381572	1.769596963	1.762638358
3.633607177	2.241761674	0	2.580087422	0
2.807120203	3.560291631	0	3.169477467	3.0884974
1.850577389	2.885085804	3.476774437	4.417365715	2.271042543
3.342278424	2.821193315	1.456662292	2.1929033	0
3.654790999	2.502332961	1.134753389	3.519933844	1.132465118
3.200227453	4.204606054	3.785633137	5.889140867	2.993325827
1.503321137	2.168027816	1.49948823	2.632573272	2.140943238
4.008779148	2.088962922	1.667181393	3.270852782	0.017110099
2.722580714	1.922929213	2.502813216	3.610157478	0
2.465788496	2.469384176	3.963583652	3.069425519	1.04401117
3.465384341	2.813056759	0.001081247	0.001175695	3.327484036
0.295901023	0.285330666	2.594802632	0.319804277	0.241554091
4.298740058	3.989985865	1.626044217	2.323774481	2.720167487
3.189127412	2.7852559	1.662090628	6.177308022	2.229806605
2.662550246	3.540209563	4.939811943	3.542547497	0.01383715
0.11573429	1.385369952	1.38409629	1.385278423	0.099025882
2.658111201	2.661240196	1.180943283	2.800279593	3.305758378
3.264979135	3.264051672	0.017870661	3.419293426	1.064507701
2.257168393	1.823274175	3.447197308	2.592005192	0.050599834
3.316194743	3.656194864	3.502470223	3.211684572	2.062583903
3.220237396	2.768489724	2.580375439	4.391868225	0
3.744767515	2.460272973	1.524508905	5.856907866	0
2.75547126	3.074702553	0	3.205119704	1.389047553
0.339346484	4.251862503	0.316443552	0.368592469	0.274029664
2.660918839	3.654854294	0.900060892	2.181569385	2.859214013
2.73171831	3.597105158	3.885775362	2.399183054	0.079574937
1.938952457	2.932848747	2.741496575	4.175576189	1.530234142
2.181185432	2.673402812	0	4.003680869	1.746883004
3.181205016	1.272661247	5.55245829	3.384948847	0.069356288
4.33960926	2.299805453	4.331144076	5.524149202	2.885853496
3.141002192	2.786736375	2.778997324	3.873411522	2.739333052
4.492137251	1.995651807	1.348202635	3.05954162	0.089461944
4.699458915	2.0586848	5.228701053	5.083778402	0.815061041
3.136367116	2.770732256	1.50908828	5.405763567	2.13693519
3.510656633	3.307246841	2.801478759	0.118835738	0.096144173
3.182018183	3.447888534	5.408417699	3.481226624	0.179865199
4.202710959	0.104509007	4.579488305	3.302669982	0.092066102
1.345960868	1.345181083	1.344132923	1.344810189	1.97473561
4.097282713	1.960797422	1.253627976	6.95102328	1.542151525
2.070641288	1.412395451	0.118301704	2.523692738	2.487303081

hsa-miR-196b-5p	hsa-miR-30d-5p	hsa-miR-500b-5p	hsa-miR-500a-5p	hsa-miR-3677-3p
4.408894925	15.37702057	4.297911273	4.297909396	3.147488983
5.923941105	14.86150209	3.846879901	3.846879825	2.844682349
10.71616882	15.24772951	4.274487382	4.274489394	4.278999985
4.577354436	15.76860109	5.228304962	5.228305719	4.940989311
6.914360394	16.78924122	3.897001816	3.897001634	5.261375197
5.320119276	15.84483878	4.356311275	4.356310759	1.12216865
7.18416056	16.17864351	3.58785166	3.587849353	4.352843629
11.62904714	19.07348537	4.130161252	4.13016261	3.411298337
3.206471273	17.18974232	4.510864144	4.510865101	5.188021717
9.214527084	14.55703423	4.630731289	4.630732426	5.789483693
4.604378627	14.87176614	4.852345504	4.852343118	3.146677576
6.859974073	15.29349453	4.982111835	4.98210975	2.793594386
5.089563794	17.61380869	4.313472973	4.313473166	3.383335997
10.71689064	16.11455038	4.06923846	4.069237526	5.181787204
6.423725577	17.10974886	4.856066369	4.856066527	3.950402346
12.22906423	16.38327695	1.927285092	1.927285676	4.882970723
8.774681512	15.47127353	5.48783209	5.487833719	4.199049531
3.66162212	16.01008787	5.350518553	5.350519689	4.05093209
9.34267068	16.52979463	4.841824654	4.841821942	4.360035586
8.700911564	15.96476121	3.750013007	3.750013105	2.753305761
7.431388489	17.1216727	5.111999933	5.111989201	0.479923851
5.194251718	15.17881772	4.047746065	4.047744461	2.867660002
11.34443655	15.49354197	5.920489509	5.920490235	3.521023264
7.100181201	15.64122006	4.532217275	4.532213473	4.253895169
2.385627793	16.38046224	4.29995189	4.299952408	2.958637844
9.298658459	15.46792626	4.810413577	4.810415887	4.574717908
6.537034519	16.50360124	6.452853608	6.452853332	3.758385755
5.560235931	15.82192903	4.202308797	4.202305844	4.583583342
5.717872547	16.42303384	5.417236822	5.417235636	1.811652181
4.551765041	15.72897073	6.116823259	6.116824613	3.563278121
5.925171011	14.11758277	4.425566084	4.425564004	4.616293181
9.69101449	16.78253819	4.503848562	4.503848025	2.484299443
8.599441474	15.87399803	4.911446748	4.911445564	0.066298638
10.1277751	15.51409204	5.239282734	5.239284401	2.091134867
5.063439768	13.83135649	5.245607685	5.245609	3.756168429
5.158225772	13.68920739	4.795916303	4.795917503	5.717409037
3.967015039	16.09086337	4.421489942	4.421488282	1.354374156
4.417807946	14.52901883	5.050808451	5.050808114	3.261158458
7.66205877	16.84487071	4.249997417	4.156228151	2.547424882
10.15959017	15.65112419	4.138797909	4.138797573	1.695055653
7.114632342	15.73700391	4.5106245	4.510625122	2.583965196
3.84676822	15.13863708	4.287868689	4.287868588	3.498155063
5.170507313	16.01616694	4.63540463	4.635402282	2.902682302
3.394294919	15.96211407	7.180800769	7.180795001	2.766561703
6.436248998	14.96803604	4.827687278	4.827684225	5.058347476
2.681913104	15.8943475	4.185578144	4.111836486	4.112504111
13.20110143	15.7877832	4.738783186	4.738777742	1.860633237
4.641101194	16.40653978	4.354336545	4.354334813	4.124743739
3.568812867	16.19261562	4.840825418	4.840826131	4.709485934
4.11198421	15.75051691	2.862585624	2.862585189	1.696991519
3.043879388	17.14312382	4.958349542	4.958347584	4.187615028
6.174205849	14.68803672	3.897688909	3.897689625	2.916664627
10.22489777	17.6415622	3.59133954	3.591340448	3.122522196
3.532299603	14.77507995	5.94222822	5.942224004	2.674233943
6.582856657	15.11861433	3.749210664	3.749210358	4.16565061
6.000758682	14.79210235	7.611007713	7.611009343	3.348195691

6.140170256	15.98848726	4.443038174	4.443036413	5.23956786
10.65154278	15.86679687	3.825202701	3.825203643	4.816446391
4.896229883	15.78594555	3.844469947	3.844469876	4.561152857
4.905792711	15.63179479	4.798156279	4.798155965	3.902534107
4.783264474	15.54823035	5.385906893	5.385909286	4.819022085
4.246836893	16.12298494	5.030671039	5.030669286	3.890270719
4.037253863	16.7310738	3.737281034	3.737280969	5.73834525
7.361966331	14.07118517	5.276720219	5.276722104	3.886117484
4.701958393	15.40901577	5.501596898	5.501598305	2.921989349
5.932929278	14.45252832	5.809523575	5.809523566	2.001799449
1.516188596	16.17454188	2.206144688	2.206145415	3.286963758
5.23562197	15.82058722	4.974085217	4.974085701	4.306789977
5.598809693	15.40241905	5.752083778	5.752078455	2.834068241
4.025150906	17.64378486	4.299428336	4.299428856	4.98424092
4.142079396	15.73118434	4.84702961	4.847029936	4.537853543
11.67321451	16.57967528	5.773382741	5.773381794	4.212537034
11.34504191	15.93664929	4.554414829	4.554414115	2.798476214
3.278776752	15.28607826	5.363492159	5.363486543	4.331785963
3.706596061	17.10597111	5.090052927	5.090053856	3.173333269
5.046085095	15.72910293	4.646103461	4.646104273	2.71525088
8.656295144	15.02071449	4.830478548	4.830477922	6.802079217
3.789060144	15.53964109	3.074847114	3.074846389	4.714831542
7.233969765	16.84778297	4.873581258	4.873582787	3.601723463
9.190436874	16.3941336	4.859172946	4.859171987	4.795498407
4.548184428	17.14055926	3.514701371	3.514700049	3.278782109
3.142355033	16.30062187	4.924856253	4.924856347	2.972666185
6.971670785	15.7823388	5.436030851	5.436032216	4.753299168
9.137057949	15.39088295	4.197098391	4.197098119	4.279163093
10.33472708	15.4579171	4.323144696	4.323146462	4.941753825
3.489729038	16.75344616	4.715645438	4.715646321	2.696764786
7.61639864	17.25575213	5.522308948	5.522306296	3.866513627
9.828469779	15.7729106	5.601274002	5.601275925	2.759180089
5.6934494	15.79553341	3.514829674	3.514826874	3.507723403
5.002357073	16.21226789	4.089764236	4.08976423	2.809507342
7.773008643	16.64087742	4.634224981	4.634220413	3.301485944
5.320245294	14.99649626	6.503548808	6.503543554	2.291051028
3.608431778	15.26715596	4.839008118	4.83900872	2.144273935
6.521587553	14.82424315	4.32388146	4.323881262	3.881989575
6.995640749	15.41536679	4.740000608	4.740001905	5.107727205
3.898029785	15.32395635	4.108405825	4.10840713	4.4584526
4.970820227	16.14262843	4.014845842	4.014846299	3.769606459
4.125507996	14.89007288	4.678346408	4.678344912	3.877867621
4.810174209	15.8505099	3.6542616	3.654261673	1.960301643
9.752970018	14.87454568	3.692573057	3.69257287	4.501871218
9.949504964	15.90656512	4.434265076	4.434266323	4.683008256
8.329985632	15.84221527	4.61643919	4.616439951	3.962854451
7.441330194	15.6231377	5.408546219	5.408546284	4.374960518
4.514950965	15.08335614	3.48116977	3.481170257	4.091356007
10.288961	14.4354815	6.291558281	6.291561277	4.641323203
10.07785295	15.73770362	4.159693984	4.159695815	4.536287384
5.017006025	16.16558028	4.309563787	4.309563628	6.570271733
6.007713233	14.01582036	5.007674361	5.007676537	4.266596297
8.046939298	14.69241137	4.199076064	4.199076493	2.818871412
2.999535083	13.8333382	3.630324607	3.630324867	5.210425211
5.381170375	15.37039889	3.408718506	3.408719087	4.563495174
8.815874004	14.66259346	5.401121655	5.401121354	2.391264699
6.300377266	15.68799463	4.213199434	4.213199118	4.447599121
3.750472373	17.11046028	5.296769024	5.296770137	4.12012256

7.575677272	15.32064859	5.521978354	5.521971823	2.538804696
10.05015012	14.68665063	4.734004591	4.73400726	5.45774282
6.865334747	14.79396257	4.727867517	4.727868375	2.519969876
7.053832202	15.47513318	5.154383332	5.154385988	4.589881034
5.399793762	16.89552875	4.541124946	4.541124505	4.469582901
8.589031411	15.18995273	5.821621257	5.821618841	4.882802326
4.921259727	14.50879972	5.359051695	5.359053643	3.996005365
6.152801577	13.9412007	5.348460508	5.348463337	2.440919028
4.490973199	14.42881413	6.353931132	6.353930989	4.279037572
4.784737021	18.51869127	5.30599306	5.305992022	4.717061155
10.7061127	14.93024704	4.799964826	4.79996363	4.335035362
4.377430614	14.90748689	4.455183963	4.455183359	3.196535752
9.01385376	15.62213302	4.963883441	4.963881747	3.885438618
7.279956892	15.2296284	4.508871023	4.508870884	4.218767855
3.459934841	15.49345829	5.002548775	5.00254827	3.598188113
5.348912656	15.44531748	5.677846671	5.677844896	3.835689598
7.800126096	15.34998693	4.179391632	4.179392654	4.833719258
4.626616218	15.84944905	3.929409609	3.929409107	4.478967081
3.624029148	16.57908917	2.857247131	2.857247263	5.698520609
6.278173191	18.63591815	3.846256741	3.846257591	3.169809611
4.981177687	15.9082886	4.330805129	4.330804176	4.32869605
5.160043236	15.79525064	4.399846594	4.399848026	6.164734662
5.495361977	16.25460385	4.203721797	4.203721273	0.040578868
1.751884032	15.99638933	5.610108278	5.610107365	4.460916714
2.626191647	15.91429361	3.865914736	3.865912977	3.486690388
5.631900797	15.03780917	5.151272672	5.151272287	2.089891497
5.115112073	15.95752996	3.716444235	3.716444385	6.491257051
9.759891829	15.15632948	6.427237377	6.427240069	4.321597956
5.529974749	16.02293632	4.176152976	4.176152962	3.005895365
3.751796107	15.22455204	5.132417866	5.132411158	3.461976065
4.889267602	15.45437145	4.882508669	4.882504751	1.63308369
6.079012872	14.70886485	4.667505828	4.667506902	6.654629163
6.428430828	15.40516482	4.993521658	4.993521362	4.333036567
3.868083782	16.69649472	4.809135782	4.809133635	4.250528136
4.761393577	16.31678369	4.572487194	4.572488503	2.933552653
7.770297773	15.52392739	5.803300652	5.803300222	3.095513736
6.753821517	14.78464619	2.592126138	2.592126192	5.346037148
4.507671662	16.05906448	5.523265218	5.523267154	3.485465128
8.551785788	17.2305929	4.390868	4.390868607	4.392208921
8.959511533	15.90592578	4.160679042	4.160680166	3.995434171
4.540290156	14.64772966	3.825868327	3.825868769	3.908769311
3.236903039	15.35908499	7.613237608	7.613226235	2.031424645
5.006862701	14.67921627	3.749166056	3.749166317	4.300047313
5.050193597	17.48579981	5.162976278	5.162974376	3.239483409
9.633258344	15.70352453	4.627107982	4.62710819	5.284126459
4.648011933	16.6934705	3.22645798	3.226458466	4.386403463
4.4032913	16.47525653	4.57345133	4.573449998	3.712645257
8.348781042	14.18062643	6.139397598	6.139395864	4.742732132
5.314162655	16.28412982	5.158244669	5.15824024	4.20491719
5.378869541	13.85435252	5.803392904	5.803390392	3.956863876
9.416893045	15.08373356	6.698548052	6.698549346	3.699879145
9.07522655	15.90681377	5.259914835	5.259917421	5.087158202
3.853414588	17.31576584	3.523187079	3.523186169	2.476897565
10.94940764	15.87538027	4.527034641	4.527030876	4.091857781
2.794304248	14.97667222	6.233420631	6.233417844	5.655267635
5.035512489	15.26721068	3.813872478	3.813871379	3.481939465
9.46188943	14.94576832	6.196428863	6.196431942	2.427801675
4.174447155	17.34110777	5.124396103	5.12439353	2.865159691

hsa-miR-671-5p	hsa-miR-4652-5p	hsa-miR-4661-5p	hsa-miR-421	hsa-miR-217-5p
3.146742254	0.132827203	3.912357407	3.57619157	9.991450919
3.846636172	0.007573233	3.946603489	2.034000845	12.75047109
3.397009009	0	3.392908686	3.96542092	10.23214621
4.771969125	1.41225585	5.495697851	4.23349968	13.52561686
4.791213084	1.05864793	4.792244765	6.068811408	13.44930653
4.184147476	0.036714602	4.509339822	3.348368663	8.798059151
3.298632096	0.261925604	6.860472012	4.015415976	6.257837717
3.694670736	0	4.264565822	2.847075716	7.152379095
3.523041177	1.677881906	1.983203036	3.083275328	15.59053683
3.801844151	5.496784799	6.060114976	5.099253399	6.509899025
3.905924802	0.132608839	2.872174394	0.131922697	9.429221675
4.512973854	0.111793058	4.323844423	3.06709855	10.04164563
3.829572822	0	3.829026008	4.00978857	6.204120107
4.831312363	7.184217353	3.530888248	5.073112347	10.72844647
3.273698654	0.968227208	7.518176264	3.122116973	4.341921161
3.40412842	0.089039316	4.595487219	3.871789298	10.14656968
3.752620943	4.403983512	5.5525218	3.215139745	8.192277966
4.56277991	5.176746353	8.79114172	4.784418894	5.454989443
3.972289418	0.150267716	3.446630152	1.469108416	6.116165751
3.266065109	0	3.528089299	3.528442668	12.87881689
3.880280417	0.47809291	4.1920916	3.038117857	5.950168951
5.643567712	2.867436316	1.412029142	3.571951213	6.596300967
4.516518257	0	3.626373124	2.637498284	4.208191829
3.918418929	0.230915053	5.358434881	2.853063253	9.594280958
2.383826658	0	4.099080594	3.949753238	7.943022314
2.718449949	0	3.083719413	3.165571132	7.323861971
3.758295348	0.011251037	6.943455243	5.344900557	8.027401206
2.798477505	0.213309707	5.552830638	3.146351309	7.288218648
5.238535646	1.188501557	3.443639457	4.280002355	8.548287396
4.555486359	4.456117317	3.244961112	3.122991573	15.29723012
2.09591337	3.595456256	4.87399707	3.395500176	14.5418181
4.179118965	0.035663364	2.977647442	5.467653785	16.51008386
3.63487266	0.066163499	3.637134425	3.111385195	10.92396579
4.300441059	0	3.361182024	3.161988968	12.10240771
4.106648337	0	3.056776149	2.610660209	10.02674227
3.730420643	5.851441605	4.717579612	4.799610114	13.8563889
3.06523697	0.111253077	4.213020639	2.451715505	4.422329726
4.795834035	3.413844157	3.261482086	2.67228122	15.14097074
3.042759555	6.59359345	5.009310372	3.829801496	8.456387796
3.303403689	2.71305377	6.391072962	2.712964102	13.44426085
3.570553551	2.366390366	4.754283789	2.940532252	7.166196123
3.62383552	1.025616436	5.084993152	2.844546488	15.10940164
3.783306779	4.535363856	5.102190389	2.559431777	16.30054969
4.939593519	0.203025196	3.117698218	3.392087182	17.21156169
3.508496994	4.637328045	8.485555304	6.412296321	9.748977786
3.67387528	0	6.088932909	4.256776276	16.63801767
3.91701289	0.295874955	4.612925943	4.117854237	4.130409656
2.822493586	3.526532185	2.033606464	3.699945296	8.188134222
3.463564133	6.981613755	5.532395675	4.080543619	15.13276615
3.489420661	0.232855912	2.862654556	0.231429454	5.504180417
4.186558282	0.106047393	3.946395139	2.771200895	7.613151252
3.287376463	0	3.050031091	3.582257361	7.716843893
3.594494683	0	4.70203404	3.594804318	11.67329485
5.281210099	0.174797842	4.56679406	1.538749112	9.07474186
4.07182055	0.032795884	5.25783316	3.621787493	6.599639024
4.680431416	0	1.702856768	2.662415442	17.69887367

2.811091638	4.621255617	5.189115105	2.810915416	12.86876848
2.853256425	0	4.443476556	4.27008026	7.4833063
4.359304403	0.007106974	4.49728324	4.037073574	14.63132069
3.262425532	4.620264782	6.421254857	3.415113288	16.9137155
4.792080271	0	4.193667813	4.110807291	7.090559786
1.942882779	5.14258044	5.620022348	2.721718291	5.97822798
4.792400176	8.151714183	4.94491046	6.2077583	15.04975371
4.876761832	1.485325468	4.057070138	3.616356476	10.00618268
5.356388552	4.104841506	4.618527787	3.056736394	15.98453497
4.089563387	0.000397698	2.809335801	2.809330962	10.89172233
2.206424769	0.171588341	4.555048131	3.717659516	5.220326788
4.704620071	0	2.187670945	3.024697807	9.189174285
4.617809205	0.224357399	4.84255516	2.369083641	8.32186216
3.9491155	3.687635164	7.018162368	2.958383658	13.82232412
2.709260668	5.384941111	3.703009876	4.479340705	13.33421103
5.169573908	0.042613126	6.42967933	5.463617371	8.624757566
4.754744749	0.046190482	3.929313353	2.199253033	15.09542454
1.756260629	6.078496164	3.285033997	2.459664719	7.283972517
5.092625111	0	7.421858896	2.317632129	15.89986714
3.509836224	4.08928828	4.691116349	3.290327254	13.06847343
4.82871824	5.612736271	4.184374749	4.270375205	8.247972985
3.783369955	0.190311986	1.577944173	2.265294745	16.84793
2.909272681	0	4.545112675	4.231226596	14.55854794
2.566284543	6.48419354	5.133993411	3.258236101	13.06215028
4.182133105	0.169246545	6.757875711	4.309679763	7.276840243
4.430561871	0	5.725241032	5.643210363	6.656478454
3.186060585	0.753464364	3.75955452	4.173106413	7.699275172
4.50182816	5.754932635	5.19937052	3.807990991	15.51508046
4.214624342	0.484158998	1.764960047	4.364908632	13.39058131
4.375853145	0	4.839958034	4.532783303	7.327854214
4.133400901	0.122111735	4.01109044	3.336864056	13.85005836
5.888231922	0	4.102977056	4.051463165	10.49296915
4.226635383	0.33313724	4.973419081	3.504176043	5.825475276
4.956516458	0.000432764	3.002188249	2.001958097	13.71289823
5.133235366	0.262292571	5.461865784	3.815099825	12.54143668
4.74089201	4.53218268	3.383269288	3.377045425	14.44315728
4.190740037	2.144277693	4.255870408	3.503060333	15.46810953
1.644525238	4.934181342	4.398153202	1.644532372	15.39225692
2.535797878	4.886759048	5.418183887	4.744013183	7.550690613
2.6203425	0	3.952783637	4.57140152	7.477613125
3.472809342	0	4.158018764	3.358541391	8.875185887
2.710182854	4.512953536	4.334759648	4.229209564	15.01571322
2.54213704	0	7.411033385	5.733895466	5.784466117
2.685597402	1.071175122	3.916021633	2.419820849	8.243684402
3.767707684	6.185175227	6.412432366	4.085610009	7.010931348
3.963284545	0	3.961266554	3.886019887	12.64265508
3.305201327	4.506176692	5.408499728	5.111797084	13.28759769
3.819279852	5.443391814	2.19835493	3.16695795	15.77242027
3.285288747	7.243407611	3.1189691	5.129194731	4.958762511
4.591651682	0	3.046365292	4.718518036	10.95255272
3.381235395	1.039651669	4.454272574	5.616650528	4.383740253
3.249030159	0	2.218253561	4.184536861	6.442194804
4.450990029	0	3.27256536	3.512471484	12.24348499
3.730145309	3.410241723	4.625239631	4.405368133	8.526623762
3.496501239	5.476550732	4.346050589	3.654142433	9.351180358
5.400303989	0.014881681	3.777141466	4.076796365	10.12430652
4.212251289	2.925245164	3.444173728	4.517797995	8.056162258
3.573343766	0	4.901562283	3.911042009	11.51765623

4.413450892	0.282655107	3.890164406	3.876799872	8.134250324
3.56565088	2.321969918	3.069853579	4.803004497	9.237412175
3.779324571	7.687860216	4.491503936	3.39664842	16.25038203
3.046790092	1.862964232	4.664963551	5.199599526	7.739174622
4.395062419	5.85007766	5.643214892	3.464711004	11.53305456
3.798141366	3.938670631	5.357197607	4.65696562	4.396981022
3.450549973	4.73698277	4.47157444	4.836906961	15.46230472
2.557860771	0	4.035045382	3.324727554	5.675256731
4.616206839	1.60939572	3.615604086	3.489705314	13.32499774
4.425640002	0.051773951	4.257292352	4.503834189	9.364466515
4.422422145	6.699218866	6.822812748	3.494589265	5.299477372
3.368132138	5.936821497	3.001823267	3.001074162	16.3731683
3.590046588	0.092056389	2.991508526	4.602797246	10.19080911
3.632857359	3.955359368	4.693569957	3.507054916	13.66402717
3.840249229	0.02779415	5.145198271	2.449486556	14.57634333
4.776639841	0.080248153	4.710560994	2.942173032	9.820824531
4.523490504	0	5.423248921	5.000209635	10.67443448
4.478468851	5.878080498	3.221404054	4.754983591	14.22613015
4.686540293	0	3.19333938	4.454355006	6.266391806
3.1702757	6.396637966	6.164614652	4.53716852	16.3520419
2.287203755	0.067993882	6.039944255	4.498839342	3.643497174
3.965992486	4.983751638	5.694034144	5.631754168	7.6187531
3.658113019	1.134512399	3.786127217	3.365863512	9.308939567
4.67275571	0.042457798	2.513828542	3.007748276	11.36690314
2.631745586	0.162320296	3.867066508	3.253030529	10.34693653
4.274370039	2.681555641	3.271128801	2.089882409	10.47305877
6.154534495	0.941990091	3.494811715	5.036540001	5.519954566
4.481405061	0	4.68462509	5.11171925	8.457019006
4.465652142	6.156066656	3.813520737	3.591031955	16.89017715
4.357333831	0.3169191	5.137177368	4.902120975	5.329126015
3.646263183	6.168930396	5.351724544	4.178700966	16.57421609
3.760596669	6.250929974	7.925782359	4.44269666	11.59920212
3.891337504	0.016387887	1.651815352	3.891286866	6.843624214
2.830458184	0.121752505	4.364114966	4.003853572	8.453053431
4.205134226	6.055434753	2.799927136	3.704976099	16.26270141
3.799097097	5.387027909	7.293507429	4.744585725	9.257497995
2.861727004	0.060895634	5.861035807	4.467067151	7.079413644
3.108609771	5.869014601	3.106830885	4.293483694	14.52965156
3.660257352	0	4.95366633	3.660376735	6.815971573
3.99624703	0	1.156172647	3.806624614	10.61446325
3.827299894	0	1.781820325	2.564495002	14.06430573
5.039128804	0.365044752	3.606034564	0.362349233	17.90066608
3.750023348	0.900666894	4.966874133	2.181598168	14.25742302
5.041699588	0.096996513	4.629460791	1.95627066	13.3892356
4.571183275	0	3.735637168	4.097974416	10.34090137
4.469102432	0	2.915697466	4.161024398	7.108984368
4.203817749	0.084159885	6.412760617	3.383780947	11.39824737
5.209563352	6.398757762	5.850514868	4.670809804	12.66745117
4.590297114	2.807689686	4.909241035	3.434859002	13.36285645
5.510434256	0.109526972	4.415548903	2.784428054	12.84682949
5.184192146	0	3.866666452	4.156225542	12.6982077
4.058003541	0	2.757657918	4.705551536	7.833745309
3.321605808	0.118057944	5.683685181	4.933445231	5.510710266
4.844482281	3.479279775	1.683776975	1.688035307	4.957303645
5.694447852	0.112845622	4.101987975	3.968746746	10.22933116
3.655168746	0.108152357	1.994101728	3.949999618	16.11469271
3.852384455	3.30773227	2.978431671	4.676727136	5.793171073
3.744271353	1.412999079	3.749245167	3.899433962	15.79954634

hsa-miR-301a-3p	hsa-miR-18a-5p	hsa-miR-454-3p	hsa-miR-19a-3p	hsa-miR-1251-5p
3.912234018	5.828640676	3.756212102	8.235026697	3.139951648
4.795407339	6.28929578	4.362112484	7.096866463	0.007331006
5.275372765	5.887333032	4.727639173	6.474967049	5.3157
4.471353213	7.264293902	4.813909654	7.769367704	1.411936114
2.07716739	5.848334731	3.89712102	7.02494937	1.05892226
3.17757867	4.092541843	3.177585711	5.947164949	1.732620093
4.364057718	4.972959311	5.314068181	6.175668843	0.248714403
6.518283441	5.944194877	6.124896568	6.849830518	0
5.184725359	4.770340719	5.529199347	7.243499105	1.291278906
7.540968497	8.795625513	6.368926638	9.646216123	4.460507711
4.853789426	5.135950527	5.132519098	7.948388539	0.127152612
5.615563153	6.026010997	5.777004356	8.783831765	0.107362386
4.091419599	5.504158542	4.727721204	7.137453021	0
4.622864341	4.070471391	4.697561369	5.188030277	3.677893004
5.408179314	6.391011563	4.807084664	8.394773748	0
4.32950907	5.521834551	5.130093494	7.417888542	0.085658142
3.892003267	5.925547918	3.891936527	7.247286412	1.214126086
6.232539041	5.967770522	5.506806465	8.424033571	4.247127604
4.366216739	8.242201433	4.997647984	10.23004536	4.820552952
4.189719952	4.798813668	4.111829856	6.858484419	0.963045345
4.975307096	6.026357438	4.976292648	8.631882763	0.44671769
6.741816715	3.576559498	7.271792757	6.94258168	0.126294677
3.145236039	5.693317305	4.720561764	8.157264255	0
3.928374819	6.444754851	3.7263177	10.39564621	3.190399034
5.058632619	6.427762512	4.632529888	7.650922282	3.783354085
4.597629584	6.571458291	4.17034286	7.702258498	4.97030366
6.418252946	8.132912271	5.452699133	9.133130855	4.305780244
3.869698663	4.349091372	5.072661039	7.288344887	0.203291635
5.042258821	7.06201142	4.5327544	7.634812077	1.189213964
3.972072248	6.310042395	4.689018393	7.190495031	0
4.200159238	5.961314901	4.622747958	6.731521049	2.88288064
5.313867215	7.545728345	4.504217877	8.015350232	4.264171736
4.494457608	6.709923879	5.499700435	10.33751346	0.063761753
4.523423243	7.029890525	4.391367566	9.199656552	0.673892944
4.040233391	5.613300698	4.040167351	6.460970735	0
5.160030394	6.66782783	4.7568507	7.188169573	2.753374441
3.67136858	3.671514	5.479597961	7.645827148	0.106848178
5.454803029	7.72833605	5.769958608	9.344950551	0.017713264
2.547491508	7.35834451	3.948484611	9.084996445	0.048540359
4.784905959	7.242724756	6.666831385	8.978314928	0.0262195
4.280098846	5.997897427	4.708657027	8.267384755	0.850494448
3.846822747	5.500431743	3.946502636	7.007249169	0.007312226
2.904099962	5.605579345	4.086117368	7.531415848	1.443779334
3.633141878	4.773388271	4.439809057	8.428215708	0.193639945
4.435438974	6.808475762	4.435673717	7.66330121	5.178502121
5.031259965	5.145906494	5.533016028	6.891906114	2.202442558
4.860939868	4.315958063	5.700880246	7.315313474	4.095272279
4.639433892	7.902069653	4.246815111	8.909058689	3.091270719
4.922579496	5.703598046	5.037627621	8.008800584	2.773797272
4.769011229	4.66438361	4.769407302	8.73056138	0.221593981
4.401059457	7.320706205	4.892025521	7.96671028	3.466444589
3.897279506	4.922565256	4.215587061	5.561607976	0.745004114
4.572456324	4.603945156	4.792233635	6.353613637	3.032548705
4.457374488	5.951797846	5.160983237	8.792902497	0.167072439
4.073245837	5.300621031	4.490180145	6.758577138	0.031685822
4.38948313	6.149225822	4.841197201	7.542119283	0.780752355

4.343059803	5.192203234	4.78956518	6.228077806	0.111899318
5.051238512	6.79198043	5.232147704	6.634932739	4.793091071
5.167531258	6.921599402	4.681844681	7.506704229	3.736727985
5.456267969	5.34708815	5.185936293	7.300266939	1.6610271
4.756394159	5.26322548	4.69674913	7.395470008	8.068172361
5.306419634	6.478683214	5.25569613	7.253236874	0.089650631
5.710733051	7.439754967	5.682171117	8.635226801	3.35758362
5.251853593	7.47467712	4.520516329	7.161039773	1.11866965
4.102240475	6.49583176	5.50059637	8.136289985	0.748159023
4.909045343	5.932927617	5.250092222	8.131495172	0.00038519
4.75094469	5.722182189	4.199057589	7.767989785	0.164044496
5.951725589	6.622979926	6.032900525	9.810643771	0.904246028
4.241359462	4.2442731	5.264847704	6.774303822	0.213642904
5.094531173	6.178698665	5.129995387	7.932522158	4.423911926
5.798511128	5.672427451	5.999980546	7.67123478	0
7.027808938	8.294479228	5.774061287	8.171967859	1.755424018
4.819059834	5.966629893	5.235579923	8.667667027	1.153492993
5.571328334	8.549872132	6.053127604	12.24669147	0.242461825
5.503360903	6.015105397	5.761457471	9.047885214	3.17613811
4.017533154	5.730506675	4.86026846	10.15614795	0
5.889566039	4.355613074	4.433508763	4.888379239	0.035161531
4.394902234	6.953077326	5.528817076	9.901327978	1.581697273
5.243831926	6.862076775	5.026741883	7.859839023	4.9497127
4.975736978	8.452324551	4.359462807	9.178231469	3.581196471
4.048964376	5.277985769	4.4373062	7.5247315	0.161834247
4.555746268	6.738182461	5.462267407	8.737998834	1.562453683
4.901403875	7.285420365	4.53586604	8.751887063	6.126829403
4.918808132	6.683704479	3.916205124	7.352733978	0.020509651
5.608532995	4.523258303	5.155666519	5.068542672	0
4.991404923	7.998166495	4.197502757	8.312957886	2.294488863
5.090221319	7.912454411	5.152370657	8.878704127	2.487636673
6.143913742	6.350642403	6.46501242	8.805012684	0
5.607955605	6.521448424	5.083304593	8.684727998	1.949107288
4.757226585	7.306178755	5.809721496	8.328837212	0.000419151
5.828182047	4.871848546	5.773806282	8.230714829	0.249056005
5.099741064	5.955686317	5.314437415	7.737089298	0.189387074
4.319741798	6.298215805	4.439467464	6.877056495	2.402648302
3.533428099	5.96090343	4.600117587	7.067523474	0.014031234
6.240255318	9.116246172	5.394435275	8.802618023	4.26041652
4.415017803	5.674370504	4.565027536	6.661366993	2.479336393
4.349648629	5.617392893	3.9370801	6.96669703	1.805657536
4.334643842	4.681044584	4.233966651	7.35272939	2.706861105
3.41371886	3.953216594	3.413715306	7.25489686	0
4.432189574	6.067466806	4.015925705	7.471727074	5.195791275
3.691918097	6.648631926	3.044807904	6.050269103	1.173378673
5.612701943	6.078221877	5.771497164	7.86581666	0
5.536810571	9.131659394	6.02655203	9.303785988	1.571424719
3.739763527	4.914791161	3.73971396	6.504963642	1.948376203
5.031294712	8.254165623	4.295511191	8.683655321	0.502762614
4.953222504	5.514767268	4.58349871	6.370221181	2.260014452
5.016745792	5.99427328	4.920465224	6.040898214	3.51841399
5.762795098	5.814888273	5.099992787	5.894854349	0
4.449268938	5.845419177	4.973615328	7.669564139	0
5.138540594	8.495948973	4.068079573	8.427206972	3.412169799
4.251105502	4.390142418	4.299307174	4.908344243	0
6.033935279	6.102713132	5.98664352	8.260993591	1.050466089
4.650760282	5.830290487	5.91045282	6.899064589	0.023616056
4.562389704	6.348235442	4.699032881	8.176883459	0

4.571669259	5.309187656	5.7814664	7.251766747	4.884164491
3.996719469	4.40842217	4.669003601	4.939379097	1.639117689
4.852309121	7.326704533	5.295399848	8.146237569	1.348861234
5.208137307	6.827637074	4.405931408	7.556753963	2.036789878
4.147610484	6.456974053	4.470809552	6.826134012	1.097018132
6.433615539	8.033623275	6.547756333	9.070651803	5.611425346
5.144061627	6.640319655	4.662238436	7.524588022	1.478107415
4.775589466	6.181113287	5.136720969	6.134151081	0
5.031773395	6.299112918	5.279805107	8.093267294	0.005715141
8.559515329	6.881724718	6.937171769	8.857420723	0.049949222
5.702631254	7.064699563	5.737642783	7.974151099	5.032235381
4.60059018	6.213585802	5.254876014	7.963571479	1.136607962
6.146955548	7.652752765	6.061235062	8.553047624	3.214165756
3.633162924	6.792418563	4.907869464	8.473313139	2.629628886
4.230111593	4.729770269	4.667686384	7.497265178	0.026863636
5.034544209	5.789094128	5.034687468	8.577061042	0.077252374
5.825417059	5.222746643	5.638184419	6.092994624	2.685958413
4.231489752	5.094700017	4.878696212	6.469112608	1.1537873
5.056821178	4.405157904	5.937146827	5.438632439	0
4.96510069	7.356172326	4.80008972	7.796230345	0
4.581309703	4.137534657	4.858849507	7.76314763	1.222886519
5.56923734	7.24822333	5.303578811	6.504145869	0
3.659481199	5.338785504	2.999684044	6.60436111	4.522089079
7.687459584	7.116614452	6.679071433	9.565175067	5.298291685
4.164852455	7.199995631	4.968426309	9.466681827	3.475540447
4.914125014	5.693323832	5.751613499	7.431481211	0.019624809
3.815560406	4.993265669	5.60603551	5.762868314	6.016884443
4.437717271	6.58605302	4.716712213	8.409203585	0
4.76120305	5.65027516	5.050738488	8.876868934	0.001132844
3.755134178	5.643231109	4.375290137	7.844911171	0.299697367
4.460584615	5.859054936	4.790334296	7.836448869	0.199354689
6.066028216	7.87763802	4.863509114	7.802091618	5.047104817
4.786977529	7.539994341	5.086947532	7.977806587	0.015853043
4.885224377	7.910965661	5.210282565	8.867984455	0.116838933
2.932712064	4.864460076	3.972882575	7.021223541	0
4.960239293	8.568290911	4.909569175	8.836406474	0.018759922
3.996901166	3.997356342	4.826412391	3.613088614	1.824619154
4.699412441	7.259185227	5.102246244	8.336640148	0
5.32820814	5.988410198	5.386884119	7.552260864	0
6.695233916	7.948196995	6.478986767	10.17020335	5.884729257
2.752832576	4.257988887	3.82558165	5.794051082	2.754932277
4.335344541	5.920589923	5.277652885	9.26273172	0.343969397
3.650081803	4.088078559	4.010655425	4.482444236	0
2.737458475	6.144133915	3.613549027	8.476702467	4.025262586
4.511667056	8.069143801	3.735637311	9.310080174	3.101363143
4.96147508	2.79702884	4.649234854	4.902052594	0.601693164
4.574248895	5.45700577	4.402760863	6.607158261	2.683151587
5.401510119	6.428787739	5.715852357	7.564751276	0.06964461
4.813914299	7.611170703	4.6031829	7.84921128	2.33876513
5.648157069	8.036675874	6.16486212	8.542097417	0.105204177
7.952898049	7.238333808	6.773795179	8.354966194	4.945559692
6.968183341	7.107962651	6.215224478	7.325539206	4.305617256
4.122868529	5.19838713	4.544727454	6.990885444	0.113325176
3.923239381	8.54868384	4.529634233	10.70920908	0.218321702
6.802609217	6.153828162	6.580160619	8.898328445	4.757430604
3.814497174	4.832007348	3.814585379	7.369160145	0.103894639
6.345107388	7.350531412	5.586737603	9.365844285	1.252278986
4.847507081	7.108778526	5.061479751	8.029486201	0.125646084

hsa-miR-3127-5p	hsa-miR-500b-3p	hsa-miR-362-3p	hsa-miR-135a-5p	hsa-miR-3662
4.290774868	2.525520508	4.598369008	3.911889782	1.411825794
3.846562011	3.207758093	4.432296342	0.007717724	1.615742666
3.613284735	2.845224931	3.072969242	4.817516936	1.768458479
4.528109284	3.481613038	3.77605966	0.86126796	1.413935402
4.411937104	3.408344603	4.260124459	1.058258414	2.399887329
3.501502001	2.755003355	3.767263015	1.732217255	0.03233199
3.296547114	1.77191342	4.197125166	5.148831356	2.887470885
5.643908463	2.115481304	3.81628021	1.037015379	0
4.363348089	3.786978698	4.463972858	3.930776737	1.29384554
5.217407643	3.561692549	3.556553817	3.463770889	1.251910403
2.869682381	2.076875532	3.378250483	3.148664094	3.341427942
3.823730099	2.006552031	3.966500531	2.794438114	1.351535638
3.24653891	3.624543767	3.508386053	0.950790382	0
4.617559678	2.926944314	2.930763392	5.589177174	1.248928204
4.037925787	2.53951729	4.3421927	0.968440937	0.968205116
2.368595544	3.202226101	3.207565	0.091076971	3.552344047
3.962194561	3.105932941	4.49555859	4.400784883	2.549898967
6.079288407	4.567464515	3.471901241	5.507229819	4.43128853
2.932985694	3.63182453	3.211806088	7.024623513	2.117145513
3.403275433	2.270733698	3.265969374	0	0.963206466
3.517292522	2.280661756	4.793346269	0.49803173	2.216936824
4.169148892	1.415758595	2.526073052	0.134983688	0.113133184
4.516915867	4.278762264	5.273305601	1.445070053	0
3.915919841	2.843710739	4.396730768	3.490190888	2.350301759
3.688675124	3.246821474	3.108587315	5.444991478	0
4.178234547	3.514678419	4.054936284	5.232961098	1.207136859
4.966080921	3.757567745	4.644251836	4.381272616	0.009975665
2.335473586	2.330722759	4.584959003	1.641528773	4.613401136
4.450471222	4.182143194	5.092407106	4.677694588	0.05022097
2.988280718	3.567188715	4.931312645	0	0.78764287
2.888064295	2.542429958	4.421840549	5.719464285	0.118086948
3.761493055	3.875597203	2.751832972	4.087034858	1.725683243
2.613236349	2.881255613	4.227058388	0.067606688	2.6009459
2.794902206	2.092405868	4.959692252	3.160108223	0
3.399821798	2.612753047	5.067924444	1.657970566	0
4.017322123	2.901432665	3.267817955	2.400362795	2.412406723
4.089139932	1.35535685	4.512709133	0.113915282	1.992905598
3.090090167	3.550760737	4.182068221	1.061266811	0.016206892
3.702806155	3.40775532	4.05481657	0.051368038	1.780533792
1.695127942	2.712041082	4.783828829	4.84150347	2.931707696
3.848887228	3.352560004	4.401170524	3.089603996	0.850773662
4.126874245	2.844261576	3.498182816	0.007697935	0.006704205
2.901265727	4.19655148	4.882036377	5.552239205	1.435802401
3.624090215	5.838671759	7.037461337	0.208766273	2.723426842
4.037873225	2.19419114	2.197719325	7.766851533	5.671647157
2.872033515	3.454012326	3.673588997	2.871600455	0.914715026
3.396694022	3.03174942	4.458369266	8.272301458	0.243878504
2.481869876	2.478812891	4.242730215	3.704342558	1.374139755
3.929744085	3.227243011	4.216547141	2.365472386	2.372247161
2.858141262	0.221792601	4.955181682	0.239772591	2.808422642
4.185340215	1.986746986	3.943355589	3.277345861	3.02008667
3.287877241	2.419788429	2.603809592	1.269688486	0.744958064
4.278561325	3.460361141	3.296757539	3.120959626	0
3.903039166	3.010947328	3.53154928	3.301341588	3.480345599
3.158458071	3.481492659	3.483743016	2.739060662	0.028911202
3.454421099	5.194286269	6.760096616	2.260361158	0.78085774

3.314934358	2.806495141	4.930202313	4.538022063	3.057601703
4.120558969	3.958581517	3.542987865	4.87605026	1.493672154
3.495778426	2.619089707	4.207286137	3.844494043	1.024084455
4.343806819	3.414117595	4.266148087	2.082390324	1.659660488
4.401539498	4.773450584	5.187555909	7.772246574	2.685536074
3.888496331	4.600210204	4.018160476	4.020590626	3.860757393
5.710118942	3.205329655	3.035440234	2.842341691	5.079399173
3.082635615	5.19338719	4.165430521	1.122705198	2.589655549
4.166837622	3.499827127	4.38567306	3.829982372	2.937686685
3.809434707	1.586618025	3.461491682	1.001345904	2.001753256
2.206118943	2.20297636	3.520255677	0.176183773	2.181686252
4.0188279	3.552915208	3.848977425	4.430964378	0
4.227328874	2.823822709	3.184219959	0.230930343	2.784624448
4.237564426	2.792298833	4.633499519	5.702352813	0.862077925
4.538296035	3.070146953	4.064863756	2.228704828	0
5.214851525	4.532006973	4.018977457	1.140423626	2.77371882
3.024233574	2.796536561	3.546135801	2.199084913	2.521448178
4.595676433	4.151858413	5.557780951	1.752942619	0.212482069
4.910956765	2.724096543	4.096247156	5.718210484	3.630100354
2.715641062	3.033484757	5.151038409	1.356245958	2.318999827
5.709273174	2.486430493	2.755153059	1.120417259	1.120710826
3.581333458	4.96153988	4.259519723	5.873878033	3.899089133
2.630489883	3.252025302	3.819972266	5.539366881	1.539337698
3.062156058	4.352341975	3.585238697	5.320639555	0.048031383
3.508579811	2.992306103	3.887529361	2.198341477	4.36972731
3.971485822	4.495090102	5.180198488	5.292427608	4.673353391
5.07561256	4.590582671	5.104972695	5.163521054	0.751957641
4.356884354	3.564788731	3.104257989	1.070824866	2.905378643
3.909544584	2.566306111	3.38463408	1.534692579	2.154243335
3.490835178	3.852792025	3.146937648	1.341761243	0.802450183
3.105840575	3.528246109	5.262743997	7.096953013	2.805626348
4.312310646	3.590118035	4.684995069	1.522025744	2.282045341
1.953483337	4.199482665	5.066425607	6.938905768	1.914782105
3.587207776	3.324127628	5.002329432	0.000440877	2.001907757
3.578676491	3.284960373	3.302401707	1.771403873	1.750000742
5.004651966	4.989072485	4.4164249	0.204068767	2.260070976
1.830873385	0.874110078	4.497117754	6.406726326	0
4.07477125	4.466737515	4.934349449	0.014783896	0.012847366
3.320671323	3.513731436	4.305023076	3.213782709	2.363917158
4.920188985	3.278058609	3.653619475	3.269926436	0
2.117478561	3.941515896	2.948142397	2.117695473	0
2.709781973	4.114656485	4.67571261	4.824456695	2.358719735
2.763709023	3.861218402	4.47649452	2.280353486	0.97020771
2.68550867	2.684869328	3.692202974	5.199036679	2.09098738
3.537953211	2.484473714	3.157984526	0.676348145	0
4.107215489	1.367311557	3.62457834	0.826114881	0
3.568062441	2.791294158	4.506143438	1.985308143	5.71162093
4.212027111	2.912682658	3.279394622	3.042812893	0
4.842884258	5.027036311	5.93615625	0	0
2.97133344	3.671948174	3.5572428	2.885695175	2.407867805
5.347241675	3.057696841	2.641371281	3.51944964	3.378123728
3.477440201	2.521396843	4.559176014	0.982670655	0
3.717735487	3.138811973	3.809545286	1.438271513	2.826193528
2.422151107	2.422878385	2.42198371	0.889167217	2.427390539
4.304895278	2.609583848	3.006046601	1.148523525	1.519685826
3.073527354	2.066084985	4.399579602	1.049894413	2.064441715
4.447043937	3.289248167	2.434545564	3.444129214	4.286815545
3.369999918	3.258720469	4.515796698	0.795784568	0.793328905

2.536930174	2.528866185	4.560257614	9.007815394	0.233739596
4.285745455	3.321397705	3.779064482	4.832603797	0.689861404
3.502567611	2.709937019	4.772275078	3.022807409	0
3.639020018	3.05325559	4.643847599	2.873216033	1.178230569
3.95258497	3.310150093	4.792490006	5.279924928	2.939398885
3.797102053	2.426361243	4.951107741	5.356415137	1.972579056
4.209348129	3.182388881	4.834721795	2.839807159	3.969964593
1.655344511	2.023537755	3.031065481	0.429455628	0.41990609
2.836459994	3.937268936	5.675541326	1.019906467	2.835233601
4.254157527	3.242249571	3.24539204	2.221955241	1.785936691
3.321376247	1.227370412	2.898072573	5.989186689	5.441950029
2.775120309	2.176947431	4.908167925	1.748695346	0.036041188
3.21836223	2.985865929	3.885874207	4.241278387	0.079893363
4.048447631	2.852831804	3.855749041	4.693517719	0.008276186
4.311211723	2.715064924	5.143916708	0.028350599	0.024534631
4.286653512	1.895581377	4.70726869	1.258628532	1.257882326
4.649199317	3.202384467	3.490821956	2.962261432	4.033735722
5.847786162	3.681925397	2.199691312	0.04724822	1.152367561
4.614311339	1.492259447	2.236395196	0	0
2.803142846	2.495413002	4.03795085	1.185048978	0.678167417
3.488026188	3.31210925	5.088260106	5.036232781	0.05938373
4.928484269	2.194793907	3.783312335	1.746241349	0.609102745
3.519091558	1.747295381	3.366356858	4.907398457	1.133895977
4.604867612	4.37877636	5.345111031	6.960836228	1.75133188
2.631074062	0.155417149	3.687191182	4.888311934	1.493644618
3.687438173	2.680821777	4.010929582	3.562173705	0.017949078
4.715100154	2.913854855	3.370110923	7.12438121	3.086119142
5.670922786	4.490367313	6.030282442	0.57516321	0.568467677
3.175869493	1.589828014	4.706704654	3.706576893	0.001039925
2.631060717	2.621082763	4.185591444	0.327617945	2.570403521
3.844747199	3.400402248	4.570744926	0.215086668	0.176118118
3.50165661	3.301579835	4.33899419	3.758329869	2.62716342
3.542204826	4.543379015	5.086207423	5.040985662	1.055150699
3.862796634	2.48645105	5.145101862	0.124726185	2.468488147
3.461895556	2.937531392	4.203850681	0	1.182857049
3.266169632	5.269512719	5.146049127	1.064847852	5.000873425
1.200050936	2.589796458	2.862101021	4.205160698	4.082587209
3.401274308	2.878962645	5.185508611	1.14135081	0
5.653189942	1.791242009	4.141722899	0	0
3.936599088	4.002665903	4.491925355	6.915411765	1.154542997
2.564606382	1.781206468	3.826724939	3.739029843	0.838029748
2.758020788	5.112210303	5.883517193	3.246034733	2.683077982
2.850181523	2.851153752	3.544414533	1.866646429	0.900410626
3.009019225	4.444521346	3.239785516	6.533980098	0.084072289
3.629586109	3.389900395	4.88097532	0.954470989	2.259690115
4.584815794	2.800361473	2.664377216	2.179094765	1.447648904
2.957738899	3.552201554	4.096960364	6.456440588	0.073191215
4.993617569	3.330237514	3.662371921	3.803458121	3.315203737
3.667090814	3.144570773	3.157591096	3.44162989	3.803199939
3.057884514	3.65363946	2.785208494	1.347245034	3.456315541
4.396269721	4.159851565	6.04743016	8.581312636	0
3.970895889	2.96073117	4.17938197	5.942665401	0.463878134
2.476252604	3.315131508	3.521312044	1.372557284	2.455968858
3.196323543	3.696939445	4.848969063	2.851765073	1.668864542
4.60410321	3.961054631	4.844216491	5.619269968	3.794743621
3.282308431	0.103970484	3.951865257	0.110724578	2.421954768
3.070377695	3.520838566	5.004275284	1.957475712	0.476003692
3.898491752	1.413762822	4.17005413	0.134279306	2.836058346

hsa-miR-1269b	hsa-let-7c-5p	hsa-miR-103a-2-5	hsa-miR-122b-5p	hsa-miR-224-3p
0.1403126	13.65067336	2.520768042	2.869866845	3.756006545
1.025042067	13.72689713	2.8440344	2.033989824	4.898553752
0	12.20354049	1.565500556	1.923719986	4.657552356
10.11742521	13.42470398	2.382169249	3.777222976	6.070920846
2.401936894	12.35157288	2.401762149	3.256399833	2.892258159
10.12675916	13.73829956	1.732090212	2.488592925	1.121347049
6.217900669	13.24563409	2.462067465	2.477508327	0.270643234
0.576612514	11.45285166	3.424522469	0.566340893	7.932465356
1.682973122	11.98138415	2.639413275	5.871051704	5.154585757
6.751028102	12.26954857	4.842675998	5.653328696	2.3953462
0.14007874	13.44031551	4.027757067	0.130741916	5.067299057
0.11783473	12.62001085	4.076786456	2.453135612	5.333676355
11.30756097	14.37388413	2.514356166	0.950334424	6.294355328
2.319709421	11.34106489	2.319552737	2.323330715	5.13298865
0.969009421	13.66896753	2.953494554	4.757253604	6.13834878
7.463168872	12.98478384	1.925280155	4.944161901	2.706239753
11.14708265	12.63440608	2.986987763	3.41832341	4.536881438
12.09202961	12.80483873	2.684123805	8.133321678	2.679527782
0.159025426	14.42819547	2.920454458	1.469439334	5.811081942
3.114629148	14.1486887	2.271037296	1.950940867	2.531980778
0.524217521	15.01025444	2.990581257	0.4671512	5.76396357
0.139110962	9.389479888	4.570842726	0.129853927	3.576552067
1.447173094	11.7009315	2.161981346	4.770375991	4.621429858
9.528825912	13.73434438	3.687549606	3.713231143	6.618586136
0	12.74685475	2.794382261	2.384015529	4.681367444
0	12.05573813	2.372538547	1.473134031	2.492273452
8.41921006	10.52734295	4.379088147	2.862403448	4.644658393
0.227243837	12.6441587	1.641294361	3.85683022	2.335856315
1.18369468	13.06838846	2.573038087	4.982604348	6.480304333
10.87771221	13.64150202	3.366318446	1.321637044	6.799560703
0.145516809	11.31675569	2.537441124	5.074987633	5.273389924
11.35166392	13.43323671	2.973904634	2.751342307	4.504121055
0.069410425	13.89193423	3.765716591	1.216792659	5.539080445
0	12.21419819	3.464407857	3.455886731	5.51729438
2.61307966	13.03744599	2.92951834	2.21094396	5.404808671
0	9.642596778	2.190553178	6.041899496	3.560717408
1.345388013	14.58322031	3.054634797	3.06393388	7.518198864
7.750301045	11.77775323	3.675618646	2.08125045	5.741292303
1.161859698	12.25162475	1.166660015	1.166434933	4.250334015
0.028343455	11.45350943	3.300546334	1.091051428	2.938169283
0	13.5410798	3.354792074	2.584359774	6.109055186
1.6155821	12.87183134	2.843941337	1.61620372	6.548767647
9.748390074	14.01491295	2.10223463	2.90059723	4.887849016
0.216052462	13.92625598	2.749342792	4.154665652	3.633002009
0.178987887	12.6171873	1.520225333	5.863683628	4.188490944
0	13.26396407	1.480309611	3.040657068	4.033985828
1.839917979	14.72039187	1.85197624	3.676265758	5.634513293
1.369623555	13.70408622	2.812622338	2.821360118	5.604204423
12.44767899	13.39103252	3.09329719	4.615011141	2.365506614
0.248581487	14.8272788	3.184513239	2.393232075	1.69358678
0.111711846	13.08387916	2.426256406	0.104626478	1.336648506
0	14.6526748	0	1.954117861	3.390617729
1.653341158	13.2596474	2.087886029	0.549397474	4.185811857
4.210794577	12.50315123	4.176329178	0.172148509	4.068903627
12.21603948	13.74558524	2.469630816	2.738472893	6.102088897
0.787136074	13.42466354	2.009659137	0	4.879673442

11.10382634	12.53538205	1.370402022	4.529426066	3.692436079
1.787927519	12.94088602	1.121606398	4.318555949	1.782082495
0.007410643	12.77334424	2.031674289	2.031913374	1.614216663
2.407306832	12.9258645	2.081807246	4.417767398	6.289012971
0	14.86298895	2.901312277	2.389530388	4.603039606
1.292364689	10.51117205	2.379344826	4.75895446	1.298042899
0.007384986	11.55974873	3.204974152	4.036834932	2.842341314
1.784340309	10.27149795	2.415296758	4.267249076	2.968752284
10.329239	12.68107355	3.76389987	5.968138362	2.608894227
1.586585344	12.99586508	2.809299884	2.001799034	6.211631047
0.181994892	14.06933318	2.650146085	2.661551152	4.839531081
1.469962093	11.70020166	2.667805031	3.024945875	2.446331679
2.358089795	13.39720398	2.356155501	0.220673126	4.74009476
9.146597353	12.97114657	2.604053797	0.862216685	5.326721772
0.935232159	12.85163129	2.229528687	3.896032327	3.068927471
1.137758879	11.28363294	4.206014643	2.184362596	5.07576428
1.148991254	13.08754145	2.794614447	3.391314279	1.76865066
9.878325765	11.60985245	3.959450609	2.924530671	3.567947318
11.39043049	11.79730197	3.960325607	3.174225004	5.783006158
1.751862334	14.01466585	2.530587722	3.290895367	3.606675486
13.19142834	14.24680123	1.730885955	1.121477352	4.887553697
1.566881331	14.05376241	3.758461393	4.380494724	4.512888651
5.429703586	13.55062062	2.916914718	2.910151865	3.676079208
11.7282758	11.17122151	1.181024903	1.180812207	3.852469832
1.509471886	12.91726835	3.259553569	4.816551129	3.892013947
5.42970093	14.04430127	1.975686287	5.100288228	2.296299553
7.311793985	11.88265578	1.661934631	5.537554014	5.685140852
6.263486144	8.223116424	2.908247593	3.56573023	3.104493625
0	12.80483416	0.918211025	3.387753981	0.925625867
4.478945481	13.83190197	2.29604554	4.483390597	1.341875699
0.128849303	12.87141904	3.323831532	2.04319163	4.732797638
0.663848123	11.62943848	4.371685551	2.760272036	4.886493719
10.69444914	12.83930168	3.464440894	2.673142869	4.723389384
12.1968614	12.01608596	1.001482643	1.001477954	4.324246383
0.280885573	12.26611108	4.904662496	0.257731629	5.715381432
8.290109123	13.70133267	3.586272798	4.139397079	3.383093961
5.698086753	12.17259605	3.507024955	0	5.699435387
2.653951148	13.15327274	3.070420743	3.658660425	5.164640477
10.15649809	8.279371618	2.987452746	2.981190716	4.14084394
0.5892321	12.72341898	1.406541357	5.494269274	4.3750418
6.688234896	13.0595311	1.407164946	2.781414037	3.768510423
0.094904864	13.82092907	2.368292397	1.291510444	3.740576454
12.47888421	14.14162284	2.542698491	4.997348844	6.962902558
10.37431663	12.35130698	1.071512409	3.807502764	2.685762726
10.91186381	11.09776862	1.173405834	4.896320665	4.856284141
0.829076658	10.4616204	3.052042044	0	5.133383473
9.018025009	9.636472805	3.89019224	2.79120125	2.306519934
1.269003134	14.22892855	0.740294323	2.910478098	4.84475539
1.579769227	13.0254871	2.183564409	0.950129613	5.827240949
1.136356178	12.10237374	2.264171182	2.889031718	4.15878123
0.012230874	11.3293744	0.011078963	1.633261796	2.641397583
0	14.90320408	2.776297377	0	4.619748604
1.841337415	12.59855607	2.631459289	2.410594456	0.881490448
0	11.55856021	3.413857046	0	0.889235739
3.410298404	13.12492209	0	2.753382596	3.493864117
8.041090264	12.32002706	4.534763437	3.396684636	5.249152492
0.025516792	13.72910418	2.106761664	1.082134632	3.29111534
1.723515725	13.24143389	4.192406291	1.328797078	2.494689807

1.806923433	14.78171152	3.328337988	2.535968542	0.292365764
9.370446468	14.59423409	1.956424933	0.694062551	5.371745705
10.08232699	12.08652153	2.523451764	3.50292329	5.324658979
0.443503072	12.99370884	2.581504292	2.328665088	4.203582776
0.030173727	11.96614459	2.452564098	4.145735191	2.454349274
1.3274534	10.22919691	4.793439217	3.0402576	4.187609251
0	12.72429615	3.692278655	4.305425312	5.379958974
0.437027525	9.3918813	2.026708237	2.558792312	2.023348527
7.890552327	13.26674474	3.351389607	3.937566934	4.423749339
10.87272223	9.682770723	4.33507437	0.051152245	6.218091444
0.072854758	11.8789178	3.314461892	6.010928574	2.627295344
3.522149747	13.10794629	2.504713654	3.000702294	5.16429992
9.850208261	11.76978388	4.74141952	4.754086824	5.026581759
10.82529876	13.64816068	2.852434334	5.178500143	5.137472926
0.02904324	13.88107275	3.303643331	2.71582252	5.274598063
2.331020743	13.40438403	3.824563914	1.260750281	5.198787536
1.210599233	11.72088834	2.836702031	5.476766789	3.656064654
2.797481407	13.41586972	1.768772907	4.316260986	3.547451133
1.792311059	10.68319854	1.493017588	0	1.786514062
10.45795814	12.82799352	1.556447411	1.852487504	1.853760446
1.84653762	13.85047092	3.773475438	4.576036659	2.892581143
8.839950314	12.27775758	2.541743017	0	4.51970955
8.889349227	13.98447279	1.135037423	3.365389788	2.175486491
1.751812443	11.91179117	4.907514321	3.909262662	8.458177836
0.171997409	13.15901271	0.150443331	1.50385183	2.978855128
0.021188632	13.41698656	3.097776311	1.667900357	3.271116761
0.943409448	12.20168058	2.723033865	4.230566958	2.24059248
0.581471605	13.37148785	4.207062391	0	5.772805012
10.25799445	14.09845058	2.813069384	3.005877287	5.432631146
0.341382319	13.55742562	1.903085149	4.507061646	0.328371317
9.845460553	13.7338225	2.768008264	1.633714781	5.649420014
10.46987059	12.50669634	3.410572391	2.428306203	3.183284748
1.65085728	13.59828182	2.39719271	2.398039883	6.982663507
1.375799184	13.99484182	3.093411019	4.129512271	4.009420349
2.102363588	14.28271452	2.100301598	3.273774268	5.024157455
0.020251581	9.059389779	3.796632703	5.054899174	5.272548877
0.06384934	13.86570208	2.255964351	5.053362481	4.623426649
2.600395054	12.60321531	2.440293638	1.506048036	6.228371682
0	10.84017661	2.578575424	0	5.550216941
10.55974118	12.66528186	3.947823338	4.363271257	5.097014388
2.56620617	13.77172282	1.384958399	1.78096448	5.68418661
0.395284248	13.39397852	3.546050302	2.756381428	4.945026383
2.183233008	14.51733793	0.90000798	4.163847823	4.792278119
8.197829547	13.68843433	2.393274151	1.956171766	5.635846191
1.529845513	11.30508443	3.93007851	2.518925049	4.249077312
1.979209248	13.87720055	1.080834023	0	4.503009776
1.265337646	11.33010401	3.178336547	0.083079408	4.308414278
0.075897924	12.76875944	2.633400767	3.333172376	3.509295896
1.635636102	11.91202073	2.332071387	0.212560858	4.711909324
1.992891766	11.38260447	4.393062681	0.10804968	3.291199219
0	12.48648965	4.560367514	3.78781589	5.148681412
1.247712453	12.05132074	3.79159574	5.871907978	5.239862732
2.021887047	14.83957901	2.469197493	2.475907742	6.014658696
1.670552155	15.107924	2.370546692	2.846310437	4.399759945
2.004211938	10.25197943	4.499690114	1.35985309	0.115743017
0.113954155	14.15567867	1.991647603	1.345802111	5.153973991
2.787520335	9.911002264	3.817981528	2.675674129	6.318344764
7.464872077	13.76251569	1.412768954	5.346523113	4.993233464

hsa-miR-3614-5p	hsa-miR-20a-5p	hsa-miR-369-5p	hsa-miR-184	hsa-miR-581
2.527129708	11.41039594	4.99378578	2.871236671	1.406673334
3.497919092	10.25854276	2.844681651	2.357320888	1.615389477
3.567017088	10.71224198	3.841150151	1.098204495	2.373768137
3.105961193	11.94473374	3.240493922	0	2.131074055
1.656276878	10.99183996	2.402785546	5.302347701	2.663238458
3.988851617	10.6612869	3.176998528	2.158726248	2.745747264
2.474477519	10.79279669	3.300329643	5.724523351	2.413568767
1.032402312	11.39733274	3.874582799	3.245113724	2.627238257
1.291365419	11.40770523	1.292142939	3.205325244	2.464373784
0	12.34333801	3.262587828	4.58603304	2.766829542
5.451259875	11.08321476	5.558336159	2.074259608	0.108574738
4.506884058	12.37937558	4.910094804	0.116865684	2.426214033
3.509074042	12.10707069	2.925903928	0	1.525629679
4.614282054	8.893027345	2.323789429	2.32174862	1.246401132
3.122375868	12.19459266	0.968241661	2.760767683	1.547008777
4.414350118	11.71141831	3.207283737	5.757365403	2.680345658
2.848234227	10.60956143	2.698896499	2.141679247	1.219273961
2.024228097	11.30588849	0	1.33065059	3.685795957
2.58755333	13.55738884	6.978880207	0.157615653	1.454480926
3.750780105	11.0386019	5.226686667	1.951365336	0.963441776
4.752874365	12.69357967	2.294032762	2.281363538	0.351884784
2.523184091	10.67498397	2.525916782	1.407953894	0.107866946
2.419085411	11.46131601	8.57465529	6.039036175	1.851343775
4.083038735	12.5552764	3.204297774	7.311008174	1.661648126
0.862266522	11.72710104	7.058902724	4.418789585	2.39307013
2.721429227	10.78414003	3.831054002	3.163543776	2.528975632
3.055593729	11.96605182	1.63128986	5.943620159	0.009598398
2.33292056	11.3282125	2.336369294	3.149818164	3.576239406
3.859305785	11.99754155	0.057449184	2.243061831	2.232338979
4.695561348	10.7751602	3.122579789	0.792289549	2.028365731
4.303806832	10.03731631	3.39675614	1.425128377	2.069325592
3.170838243	12.98122258	1.118751362	0.037036125	1.117331022
2.279141455	13.49865219	1.844606654	2.278178702	2.863746946
1.845402697	12.53890022	4.759831488	5.834997852	2.10817211
3.678043028	10.22049787	2.923351069	2.212803626	2.441266215
1.93787809	11.5920886	5.163709291	0	2.417951618
2.005412892	11.19079808	0.111486725	0.116295723	1.345906363
2.895883175	11.94938652	4.094292048	1.658990052	0.01558526
3.236749867	13.11241347	1.166016592	4.422199636	2.534218083
0.026504125	12.56597391	3.944626058	0.028149725	2.439880097
4.460376798	11.15227567	1.401052127	1.403389255	1.804650468
2.621310546	10.76535903	2.621479173	1.615795579	2.032537161
0.13729168	11.50367533	1.442964565	6.270111575	1.43019383
3.992583653	12.2135159	3.627585308	0.213944713	2.706841611
2.195653574	12.01792443	2.654142669	2.192411601	1.504058089
3.86674895	10.44077082	0.914941235	0	3.048379801
4.279748673	10.99843377	1.860639499	6.366084877	1.813876685
2.819533736	13.28063861	3.328329234	1.372902288	3.652786143
4.082373809	11.07299508	2.109209395	6.01565541	3.239902604
0.225088658	11.77822003	4.530289976	0.246029687	2.339033868
3.936998384	12.07496968	3.473557338	0.110803788	1.330963954
4.434972875	9.663095228	6.756323971	2.20664742	1.659678465
4.006576804	11.04197272	4.000237007	1.368680262	1.657984079
2.669820804	12.21048716	5.151111435	2.672101373	1.519831787
4.920564672	10.24357579	1.109398145	10.36247475	1.713101755
4.395555025	12.10419548	3.34820481	0	0

3.683494368	11.16030249	2.811540872	3.692807468	2.003232778
4.176011659	11.09594394	4.220454911	7.811377082	1.794525359
3.73689916	11.17047425	4.124709129	1.02368995	1.613531232
3.678499496	10.38876897	1.661022257	2.897713935	2.403718097
4.827244477	10.42162345	3.088685539	4.275828486	1.34417743
0.09076663	11.62698704	7.468756808	1.939100717	2.362628847
2.842084823	11.50978706	0.007094285	0.007336906	2.353570439
2.851425086	10.66556065	6.167731639	0.638387464	2.249028035
3.295109774	11.9097052	3.831826707	7.332360422	2.440010916
3.32395718	11.32414662	4.461553068	0.000411754	2.323721524
4.04136477	10.82037541	3.286937267	1.520691777	2.170357888
0.904355212	12.72692188	1.871916855	0.906397586	0.905079062
4.487299003	10.6376772	3.69804795	1.663793236	1.645596362
4.028463238	11.60060005	2.601301796	1.815824846	1.819804773
4.066025724	10.06694033	4.751644322	3.802091072	1.914168946
3.202401793	12.19692884	2.184524213	3.008788632	2.175710195
5.229619322	11.54120889	2.529866949	1.150211261	3.203922697
2.92014168	14.72851757	3.561356437	8.774072478	2.396644404
3.039663642	12.39603614	3.411085123	1.363548373	0
2.056983761	13.43965948	2.527165081	2.312328828	2.066350091
3.879926541	9.114789392	1.73149799	0.037807035	0.030787211
0.184370791	13.57034152	1.581532894	0.200322599	2.223698663
3.250350149	12.01826105	3.030557994	4.902871891	2.803188926
1.802049902	13.54144012	0.054907056	3.259335395	2.551887658
3.271838259	11.57330315	3.000915254	0.177818725	1.505230351
0.981535493	12.69127172	1.975517574	1.975727845	2.782001957
2.782101933	11.82957867	2.429115936	3.500611538	2.950169574
1.671560222	11.38457973	3.808225189	0.021998358	2.414881862
2.435728002	9.34299996	4.917743074	2.985844148	1.781189433
2.698089608	12.97111407	5.371575113	6.594624642	2.305629004
2.489155385	12.87339036	4.360389027	5.784878773	0.100327789
5.019326797	12.08902739	0.656407203	2.892144763	2.076409984
3.772929993	12.81212586	0.334136908	1.940811995	2.583391109
3.809603717	11.47966639	3.461671593	1.001448899	2.001881379
1.774026598	11.32391837	2.480917021	0.277859597	2.414489051
2.746515424	10.54677585	3.812973802	7.974293454	0.159074059
2.810804245	11.20940004	2.809840258	0.876936113	1.836254881
3.394509027	10.28015548	3.881986523	8.391710021	1.642777736
2.848697691	11.72600154	5.900208322	2.846901787	0.704681302
1.049563285	10.47583442	5.174175361	4.201119195	2.504538248
3.679149004	11.69072786	3.578496534	3.855050745	2.602696181
2.372075116	10.42393113	4.749653185	5.833337032	1.284900841
2.280437648	11.90252992	1.54851627	2.280567365	2.282249461
1.071445869	11.50161743	5.503159656	1.06993692	3.906340844
3.265852072	10.02501254	2.481890617	1.551426573	1.178566568
0.824137283	10.27657395	5.639280599	3.88370933	1.37143884
3.889912753	13.03422079	7.383208299	2.569140758	1.985843853
3.168987928	10.5106549	3.16644352	4.149003406	3.191301592
0	12.50355	2.177989626	2.182139129	1.820329788
0.801651437	10.38554771	4.362036092	1.612833545	2.538328984
2.376565062	10.91998493	3.645042811	1.038963139	3.224683216
3.677927585	9.409613695	1.325155672	5.575458008	2.06327503
4.268898493	11.41366898	7.022949755	2.819094591	0.880888127
0.88792205	11.2230438	7.630764203	1.449111218	2.429806904
4.755945126	9.157167574	4.9757424	0	2.282342367
1.646085248	11.92759001	3.776707353	4.400303913	2.652132022
3.706869048	11.43720979	6.603538808	0.0253435	2.428527591
1.719029005	10.83076594	4.244767396	1.332518501	2.036576897

3.349931313	10.92771196	3.009672343	3.653522305	0.22057535
5.178121014	8.728559764	5.134998366	2.326421766	0.691773689
2.709222527	12.57832701	2.707930192	0.813553329	2.315984215
2.96722763	10.75986177	3.124912098	1.185785498	2.361882071
3.463719643	11.20527323	2.454360583	6.960819797	2.121065812
3.793556107	11.63109444	1.984776325	7.404076387	2.403635144
3.819488591	10.43528823	3.273805637	1.120805789	1.487903609
2.022652705	10.96444045	5.823572485	3.817728132	2.048783341
1.02012054	11.35515977	4.118452839	1.019692964	2.3483453
3.047716994	12.90258744	0.051870258	4.06354654	2.807202472
1.227284952	12.13924879	6.627453031	1.853681966	1.845955754
3.901262953	11.22453415	1.749233923	5.165461162	2.496896804
1.938292387	10.80604985	5.483476946	0.096068167	1.923258041
4.572149061	11.24674708	1.031587568	7.510463891	1.622371341
5.60150599	10.13212283	3.306742245	7.337924155	2.708171477
5.335947607	11.72686137	4.706732824	1.892916401	1.883357013
2.684797888	10.45893707	4.067493534	3.197132586	2.354400973
6.063749454	10.48317797	2.530257579	6.193685856	1.763198947
2.860743562	9.888341611	3.382567089	2.726782378	0
2.657858278	11.8108731	1.852598457	1.560644582	1.56505283
1.851242514	9.946412934	1.222016867	9.290066937	2.272115817
6.534146092	10.11220537	3.243838751	9.204568882	1.464404501
3.517895987	11.20880548	3.901215363	0.042077141	2.494984973
3.201702039	12.59286643	1.754967338	0.044117977	3.188418431
3.852970589	12.07155064	7.243890862	3.867877001	2.143325995
3.560794567	11.20873072	5.721218531	0.021046154	2.086004306
4.862509564	9.363140179	1.922364972	0.942987077	2.244129937
2.123477106	10.76738829	2.301833439	6.59486688	1.693987394
1.589830064	11.81347048	3.005895234	5.254316953	2.590347233
0.305015563	11.58582336	0.317849118	0.337378765	1.86072709
3.637438182	11.25394461	1.633093717	6.400849604	4.087853501
1.275245728	11.79116468	2.778985973	2.61503949	1.975158192
4.171305599	11.75157918	2.398151304	1.054298399	1.650156715
3.703472407	12.95104844	2.830939926	3.338474367	2.794075921
3.781210199	10.93889874	2.49096781	1.559067336	3.084748045
3.265669136	12.60243425	3.095511343	2.411398547	2.895517733
5.203623088	8.588735707	2.861971105	7.091928747	1.815914594
2.253295234	11.97853943	2.59663977	1.14502197	0.645071383
3.082880445	10.32723927	4.6541995	7.270598267	1.397626266
2.897530646	13.43709217	3.420656452	6.033163694	2.477085102
4.062261704	9.238618505	3.205307579	0.84142199	1.388628643
3.223692001	12.71659788	3.595359536	2.018233394	3.109625666
0.900232698	8.425987068	4.425203252	1.464158047	0
4.144730382	13.02433502	2.736877366	3.61358016	2.375762682
2.931899196	11.92478279	3.389086512	6.666208332	1.530096842
3.772640812	9.267826993	4.81863782	1.976304165	1.45196775
4.300043861	10.56296235	1.272221585	1.906937853	1.896676996
1.236296349	10.60147315	1.235484769	0.075323199	3.633056582
2.341160743	12.03834461	3.436937112	0.227946944	1.625103012
1.999457881	11.28141537	7.256051972	2.443334326	3.445422865
3.789106395	11.79031266	3.607733879	8.716768978	0
3.881161925	11.62468581	2.403660652	3.716372628	0.901961086
4.111667965	11.8815213	2.817156609	2.474491169	1.36527303
3.702350083	14.26227503	3.199370291	0.242257515	2.329837989
2.010893412	11.28716238	3.071936059	1.353106992	0.093000405
4.580591322	9.933486312	3.053802643	1.339294284	1.337019418
2.984011688	12.09586198	5.594400878	11.93278692	1.540707433
3.366198817	12.48673322	2.07363275	1.405985121	2.048754649

hsa-miR-3923	hsa-miR-17-5p	hsa-miR-4664-3p	hsa-miR-34c-3p	hsa-miR-4326
0.138782949	11.40711782	0.095389509	2.861276754	3.580125743
1.025276284	10.46596666	0.005813068	2.033778961	3.739795539
0.385905647	11.48934449	0.377061012	2.567773814	4.36623523
2.380705283	12.31729982	2.809072104	3.108084215	4.677447593
0.018012727	11.1547753	0.013286739	3.787247629	3.89696848
0.038058234	10.61991493	0.027733909	0.034790118	2.756716145
0.276644238	10.81709391	2.36038316	2.926204146	4.360933073
0.57357307	11.81691319	2.508759933	4.148359779	4.829675042
1.29554765	11.56440733	2.006458485	0	4.923742819
1.634662594	12.96792953	1.651732759	1.249358099	5.095460423
0.138552286	11.35518052	0.095243283	1.417702755	4.997780145
0.116602408	12.27854678	0.081154565	2.004715157	5.332062999
0.951234338	11.87967619	0.951926722	0	4.169390813
9.914372047	9.711438683	2.289991046	0.07290398	4.543274317
0	12.08486654	1.548364908	1.957853916	4.271677712
0.092695506	11.73232529	2.329216607	0.083858843	3.579957378
7.975906099	10.74850451	1.22862061	1.214149949	4.196967483
0	11.72600707	2.045820867	1.717662897	7.082713956
0.157232977	13.70897992	2.079238023	0.140525628	4.669097703
0.963815226	11.02339788	1.954728203	0.963017233	3.40295369
0.514470105	12.33576763	2.102297379	2.269190919	3.048237606
0.137597713	10.93921165	0.094637747	4.573119347	5.126634854
4.137559565	11.30188175	1.451749998	0	4.672252703
0.243253733	12.73948415	1.627806132	0.213956373	0.236280798
1.41743075	12.14953325	0	0	4.236234134
1.213103471	11.19012266	1.228269473	1.473496892	3.503250303
0.011638528	12.8390534	3.634916804	1.63121249	6.037676562
0.224375424	10.94390249	1.587651873	3.83538038	1.642504887
0.059540352	12.3650271	1.79362152	1.189158073	5.193964021
12.1025164	11.26045825	1.333410765	1.321502471	5.286890609
1.425873724	10.47599744	1.402481569	3.382210595	4.620764252
1.116922	13.6380882	1.113733925	1.119317889	5.739245506
0.068750771	13.23486422	0.049193628	0.062480098	4.717533743
1.181897	12.6645298	0.679657864	3.169320936	3.361571571
0.754243172	10.57015681	1.285654541	1.65741805	3.974441468
0	12.64653184	1.656057841	1.939298954	3.371837987
0.116034016	10.64557192	0.080785101	2.445925768	0.113344933
1.060700595	12.31117904	1.05932482	2.405989254	6.141748057
0.052207244	13.08220976	0.037723778	2.214162535	4.337906344
0.028096993	12.12123718	1.087174428	2.118377061	3.45707922
5.423745618	10.88797609	0	0.85031782	0
5.398479825	11.55839014	1.024711482	2.357057425	4.499540413
5.291880524	11.76648491	1.411984941	2.103156343	5.03197628
1.60781037	11.64735898	1.5637083	0.188715506	3.83309964
3.715257523	12.73062502	2.569665683	0.157522415	5.959071046
5.717703259	10.77131085	0.917207164	1.886202646	1.885873016
0.313402795	10.64362921	0.196365263	3.368856528	4.130386964
1.37354955	13.40376773	0.086481076	0.112253334	4.549352528
2.772269678	11.27298115	1.409218342	2.367428888	3.667052003
0.245338379	11.30388051	0.159174846	1.69370527	0.23828363
1.333650737	12.44641942	2.715520893	1.33936928	4.665895553
1.956562567	10.12563785	0	0.744619729	2.91607325
1.018495014	11.40606242	1.921657476	1.88252883	4.67228463
2.212319827	12.1478753	0.122911589	0.163004615	6.019850411
0.033985198	10.48996494	0.024826648	1.717008291	2.964428577
0.785372955	12.25374295	0	0.780444398	4.097004419

2.811204522	11.04504639	0.084406879	1.370657123	4.442718835
0.640174223	11.74447559	0	3.703637925	5.233790252
1.614022237	11.97737464	2.02927295	1.024238047	4.124891699
2.673521804	10.63439541	0.014187755	2.672454651	3.902957802
0	10.48975891	2.101610619	3.099288347	3.55676784
0.097080239	11.76467662	2.673543862	1.94048215	5.201285868
0.007323813	12.01246961	2.838357379	1.614196235	4.895864856
1.490270289	11.33176076	1.134263209	2.022457244	4.707672982
2.609860372	11.58078502	0	0.747781598	0.750926861
0.000411031	11.32977119	0.000306487	1.586615038	4.089577005
0.179860858	11.42592076	3.181338095	0.160070685	3.01085784
2.85629502	12.81055579	0.907281114	0	4.932432667
0.236215339	11.02317183	0.153991195	1.671400524	5.80215226
1.417047204	11.67793246	2.62132193	1.414911687	4.360633431
0	10.30419485	0.93549934	2.229393504	5.384114853
0.044193976	12.79678337	3.348470082	1.754965105	5.973761462
0.047918093	11.48159943	1.145931552	1.768627616	2.798799325
0.269400774	14.60867938	0.172615941	0.235815342	5.56663326
0	12.56165492	1.370943047	2.892581199	6.310388778
6.729101269	12.71314454	0	1.748608808	3.78561016
0.037734654	10.16607276	2.959597515	1.731036021	5.773301424
0.199799352	13.07057219	0.13280066	0.177156264	5.067665488
1.168791487	12.5106552	1.177493678	1.162224174	5.085550154
1.177589072	13.57263481	2.539409585	2.563105496	3.85209194
2.194647424	11.56356316	0.11933656	0.157928593	3.714912889
0.981900989	12.78292468	1.563833474	0	4.877586361
0.757189627	11.98174824	2.818279153	2.619558662	5.899108331
2.093223484	11.89131091	2.410200999	1.07164565	4.634215591
0	10.07516049	1.805344738	3.923608905	5.349148762
0	13.41532231	1.351516628	1.732472624	4.427860843
0.127473784	13.31553181	2.434097882	2.039587353	4.955985099
0	11.96914548	0.659835762	4.872792969	5.945751513
0.353997031	12.52216489	2.513862031	0.305111696	4.036840274
1.001451166	11.55796189	1.001425051	2.809475719	4.394635312
0.277040428	11.2620388	0.176814933	1.769548745	4.633398603
0.208536822	10.62472065	0.137959372	3.081290321	4.750168078
1.432167398	11.60135238	0	1.430258545	3.127350329
12.91793684	10.96877863	1.640533526	2.064180288	3.659290794
0	12.24045867	4.515806599	1.214497257	3.8931129
0	11.09168177	1.064951856	2.626940159	2.870341178
1.409332552	11.89994441	0	2.951495615	4.225189159
0.093955254	10.47323402	1.274404436	2.975566562	3.740005718
0	11.37242284	0.971153703	1.960517993	2.28033464
1.070049005	12.12136368	0.016130057	0.020099347	4.196864124
2.091039955	11.01418785	2.976268562	2.93104754	4.342316637
0	10.51544812	0	1.367466134	5.10017238
7.618583348	13.20356036	1.57226269	2.98383839	5.889126875
0.7462101	10.36875349	2.228026395	0	4.420980573
4.294082483	12.77376865	0.507353193	1.569467665	5.468263621
0	10.74273861	2.687789744	1.799279332	4.913900998
0.012128395	11.79141778	2.371414878	2.052047413	4.705955905
0	9.955789015	0	7.47868544	3.162408514
1.840206097	11.48368226	0	2.412085749	4.329826239
0	11.82432056	4.09313267	3.152278228	5.985918223
2.607481124	9.881053283	1.537875075	0.649611978	4.479020626
0.015398777	12.05967103	1.048358471	3.242964613	0.015108979
0.025296329	11.72698236	1.078693848	2.106917242	5.172240423
0	11.48577892	2.523014574	2.027738078	3.571898009

0.299063552	10.96401482	0.188740298	2.984433587	3.88741027
0	9.049651402	0	3.673536613	3.561513221
0.813283932	12.53303018	0	1.742212359	4.49217868
1.184948198	11.03361727	1.913702824	4.333734683	4.204804803
0.029910265	11.18472227	1.693653554	1.097034412	4.235149529
7.236383789	12.04242865	0.076570559	3.032807457	5.991631706
0.633561749	10.77296935	1.502542652	2.847966219	4.154770912
1.17452282	11.36774336	0.425923999	0.837721053	3.1084941
4.201259911	11.37418744	0.004535405	2.613188939	5.704307754
1.16807535	13.29606944	3.019007309	2.550540103	6.60318877
8.453981178	12.45701747	0.051527936	1.855266795	6.685183487
3.522904143	11.07641139	1.736967158	1.748671206	4.205879122
0.09586031	11.37589285	1.907012811	1.29712258	5.936106331
4.04902869	10.93783054	0.007154147	4.571374556	3.507306034
2.941049455	10.37194009	1.089266838	1.697402274	4.728849295
0.083482868	11.78479441	1.246111441	0.075656455	4.473010285
1.207813809	10.79929429	2.373268693	3.49992767	5.470358849
3.02538251	10.76499233	2.998083126	2.795481937	0.047041667
0	10.22573287	0.641090791	2.586072131	4.223364853
1.187887764	11.75270946	4.377210695	2.10153669	4.765968663
0.070663361	10.27729755	1.829359034	1.222780567	0.069164357
7.934415618	11.07202552	2.237382978	3.978134323	4.951373084
0.041995818	11.35010309	0.030531006	1.746855154	4.00980688
1.138615373	12.97968851	2.493325648	0.040205246	4.800592798
2.172431252	12.11698222	1.46520275	0.151586251	0.165678263
6.795487504	11.13379415	1.065618785	0.019266293	4.964544479
0	9.903675469	0.943675422	1.513781112	4.077975524
0	11.13000783	0	1.394278378	5.804770356
7.441095857	11.58441887	1.003849102	1.00400552	5.093791518
0.336296457	11.80862028	1.810222334	1.904857637	3.467977271
0.21988326	11.3429518	0.14458772	0.194238558	4.970721502
4.38786086	12.72163292	3.448509905	1.27523947	5.037726287
0.016959407	11.8379699	1.647610474	3.079564896	4.671119413
0.127095	13.41906624	0.08793258	1.385974346	5.086906742
3.701482719	10.78866518	0.683254927	1.180267802	3.544872458
12.1220952	12.63500703	1.062773619	2.676129436	5.857703179
0.063249528	9.126609298	1.189916378	3.279444464	3.459113008
0	12.36240412	2.080570962	1.137430163	5.523613737
0	10.81035223	0	4.147733395	5.498473104
1.158747476	13.6112146	0	2.901963303	4.858680848
11.50480046	9.737424263	0	2.567034875	4.59170156
0.388967545	12.82013271	0.234833834	0.333135503	4.330871612
9.722987606	9.01580596	0.903256756	1.866875172	3.429335907
0.101045739	12.68456708	1.922455662	1.312119686	4.544796989
0	12.06745992	1.943432599	2.519569822	0.954390165
0	9.491331511	1.467091648	4.071678062	4.2052264
2.685779227	10.76296159	0.061980965	2.346198281	5.162119394
0.075166918	11.07234178	0.053582379	1.236251009	5.213201093
2.340184559	12.70562732	0.148891921	0.20053519	4.70927162
1.996510883	11.83964237	1.32768382	1.349768385	6.219168479
0.817678301	11.75793399	0	4.788348941	6.317182901
0.909827943	12.01468653	2.602660244	2.26886582	5.332948317
0.123200639	11.86842494	1.350710803	2.470216819	4.121718749
0.24158077	13.84251813	2.727339594	3.686168289	1.685661178
4.525239694	12.03193665	0.081874161	1.359647294	7.191955165
1.991762672	10.16834282	0.078659575	1.992275357	4.19911442
0.488285703	12.33961132	2.34847134	0.914397205	4.036845396
2.521622864	12.85469297	2.793643982	2.069253136	4.683324187

hsa-miR-214-3p	hsa-miR-552-5p	hsa-miR-122-3p	hsa-miR-199b-3p	hsa-miR-199a-3p
4.842981521	3.912239772	7.787298883	14.07837496	14.08159838
1.61628408	1.615742134	9.89352827	9.582878289	9.592528736
4.081131404	0.788199676	8.254687237	12.35104073	12.35381826
2.955193793	6.06913442	9.255062656	12.90712192	12.90903289
1.656285009	2.891819288	7.70914904	11.20001004	11.20719592
4.271172973	1.119490186	9.486864572	13.51669938	13.5195145
0.257107987	4.510432553	11.23977895	9.370182695	9.370182539
4.136706745	1.396205155	6.892097583	12.90205806	12.90604321
1.291596774	1.986030209	9.414432813	9.857684121	9.860023796
3.466883776	7.740232194	8.271527605	11.55050985	11.55812431
4.916742664	2.868680986	10.39670695	13.58438745	13.60136327
4.510231601	3.828968219	9.822349115	13.20884953	13.23130683
3.623913936	0	10.04978042	11.73844351	11.74163086
3.527150518	1.880974934	7.797795219	11.55532156	11.56346421
0	1.958148034	10.24679882	7.287651306	7.296527865
2.704663864	1.28083445	10.0123594	11.13893326	11.13980281
3.103191419	8.48106959	8.88060186	11.76639622	11.77243517
0	3.252907038	9.751959394	6.699471292	6.720968275
0.147956571	0.158588845	8.205813207	7.410453896	7.424168358
5.433486296	0.964041344	9.555851299	13.65969748	13.66630897
2.290352103	5.69772313	11.01242145	11.69481199	11.69627155
3.570006929	0.138742504	3.373806559	11.60329129	11.63232658
0	3.817817813	10.27369726	8.41199791	8.411997961
3.199555787	3.725042821	9.513477388	12.72465829	12.72763851
3.109378692	3.108922974	8.680702948	11.19583646	11.20106146
5.368389694	0	8.518077114	13.54275506	13.5449954
2.374488864	4.867536069	7.992359643	11.05118005	11.05472395
2.334853964	7.560670058	10.27605738	10.03448898	10.03448902
1.811729948	3.26998072	11.17105292	8.115581429	8.1283006
2.017786197	0	10.19290685	12.28170808	12.28619805
2.887303681	1.422770073	8.938557708	11.57966057	11.58630144
1.11907535	1.116145461	8.577091113	8.413033129	8.413033072
3.110660023	1.840598947	10.0589414	10.29653749	10.30377019
4.994483557	5.635549006	10.06050502	13.02733566	13.03125765
4.044510927	1.66156046	10.10826511	13.07820484	13.07943637
0.734978179	2.190798162	8.535827219	8.139708129	8.143409374
0.109659383	5.659610903	10.44480081	8.913989305	8.918331288
4.795366438	2.671878877	9.276894016	12.67371849	12.69182161
2.815760459	2.545807386	9.082019212	11.14711	11.15091989
5.348990931	4.139140143	9.383986435	12.15662666	12.16255735
1.798506012	0.85451379	10.69706458	9.553026327	9.556246121
3.037630492	7.211407938	9.759347268	11.55691244	11.5578979
3.407169703	2.555456378	10.76208548	10.13989569	10.14192133
2.303647916	6.672258354	10.23681997	11.32516491	11.33978341
0.166121888	5.615097385	8.571431772	8.026988142	8.036406635
3.191581778	1.482026143	9.463262318	11.3439879	11.34850638
1.86052808	10.00586704	9.2742641	9.799542158	9.803298435
3.095624814	0.125851639	9.375320485	12.36718381	12.37286905
1.797619012	9.668352481	9.209771061	10.68633797	10.69146782
5.70584516	6.034109068	11.71866674	13.53258404	13.53649188
3.47107671	5.46132097	9.420739073	12.3025728	12.30869247
5.979606908	0.751767181	9.943930501	13.9403592	13.94204405
5.018783225	3.212609184	8.534310938	13.93692297	13.94260789
5.211500263	4.069806928	7.129699463	13.66167594	13.67724241
0.032395856	4.49058015	9.99331061	9.87693825	9.887293621
4.224969975	0	10.00584949	12.08872748	12.09406912

3.08406996	8.449010537	10.57035294	11.13210975	11.13873957
5.191669338	2.723046609	8.052031612	13.53487055	13.53694066
3.943749224	0.007395727	8.683054806	12.10131219	12.10232403
1.661087677	3.679779223	10.5635312	8.275782906	8.285734366
5.103473771	0.542494393	7.936704988	13.62388882	13.62649601
0.091929135	3.224244148	10.58398249	8.355152531	8.355152371
3.205587382	0.007370122	8.578218245	10.86408232	10.88382955
1.776753717	0.639807217	8.401581668	8.638673532	8.643221443
3.496961102	3.671995874	10.43597536	11.15449183	11.17074509
4.394423015	1.586589636	9.552963379	12.61301303	12.63112002
1.530054043	0.181474916	9.513331773	10.80797424	10.81074161
2.856633777	3.550712749	8.832030002	11.52503528	11.54591853
3.892485834	1.659900708	9.548550605	11.51242919	11.53173418
3.949885566	4.168349776	9.248956404	12.39032285	12.39534395
5.141879898	2.900672791	9.332531238	13.01955042	13.02469079
1.141663422	3.530095775	8.71174715	9.977398204	9.980738194
2.529432941	0.048250761	10.20761893	12.41007409	12.41320577
4.166039942	4.186513122	9.41609824	11.23578537	11.2433679
2.317835634	2.065682293	9.620759544	10.6799319	10.70245057
1.354842967	4.087136821	11.67469264	9.12108822	9.127309552
1.121489724	0.037990753	7.49615394	10.01485253	10.01803762
2.722914939	0.201654233	9.576846727	9.974377593	9.976966945
4.743459361	2.470507216	8.84033589	13.51867555	13.52142762
3.72262568	2.564713021	9.319856447	11.41522353	11.41779424
3.706678972	4.049569305	10.24302343	11.16628183	11.17804418
0	0.982014977	9.524364734	8.400259842	8.400259851
3.405541651	5.003583807	9.737646938	12.73259003	12.73447491
0.020957385	3.808787642	9.269560304	7.454225598	7.45422554
5.0833699	0	7.606841348	13.09960877	13.10299259
1.340253995	1.344780696	9.611194438	8.129450287	8.12945039
4.130715415	3.870206647	10.30112377	11.95469791	11.96448357
2.893136986	1.158719128	8.035695723	12.3719377	12.41020061
0.32637092	4.041458064	9.001705722	9.050734771	9.064374864
2.001957502	1.586733043	9.514164508	11.29588568	11.29875671
2.945268877	0.279947106	10.12173197	11.12967406	11.14758537
1.604766973	1.591038369	9.515355009	11.13844622	11.18835195
0	0.877492772	10.03643922	7.198758169	7.207213575
0.014332843	1.643533226	9.352994787	9.234022833	9.239076415
1.59222438	5.293107339	9.26423698	8.276108588	8.279344597
3.656806511	0.588340127	8.215311528	11.64342622	11.64657923
5.01357222	1.808010727	9.03001612	13.94348362	13.94472009
6.043308552	3.212113414	9.051190637	14.39109775	14.39420963
1.548472189	6.665482005	9.64092246	10.36452446	10.36874897
1.07134552	0.022063015	8.872732505	8.323881077	8.323881041
2.482620639	0	8.49333105	9.897663607	9.90470669
4.619817857	0	4.615509018	11.01404292	11.02082629
3.790349137	6.852306828	9.292456699	10.42269848	10.43304036
2.762508027	5.762364641	9.64603255	11.94022898	11.94210573
1.801324152	0	8.586883236	9.543526329	9.547545866
4.98639646	3.658630045	8.535305177	13.64741704	13.65186539
2.641226142	2.376286447	8.421769487	10.8300374	10.83417484
5.524316539	0.535505339	0	12.64048932	12.64291736
4.201104774	1.840967838	9.254028195	12.71248345	12.71789402
3.631761623	0	2.642214057	12.14742865	12.15049604
4.717694751	2.754407534	8.745663993	13.47530494	13.47784342
4.662358339	2.065515515	7.650881134	13.4364006	13.45173989
4.12399892	1.080123531	8.170317084	10.86810557	10.87401331
5.30140311	3.474031956	9.532117072	13.1787438	13.18202346

1.827030621	10.1691142	10.03597434	11.21598474	11.24978522
6.047617404	0.709652728	5.727112999	13.89982424	13.90231698
3.39732913	5.886786712	10.22465022	10.57991895	10.58442223
5.470740143	0.863004588	7.769839966	13.78757487	13.79002118
3.464320436	6.895186513	9.607881179	11.61930336	11.62541667
1.336692482	3.272037833	10.80890863	10.11125878	10.11497815
5.851375651	8.501696435	9.353929663	13.61105836	13.61350737
0.840445405	0	10.76459841	9.608933942	9.613144017
5.353259677	0.006142349	9.463090157	12.80478179	12.81955568
1.171147082	3.955270781	1.166264224	10.21905876	10.24937543
1.227049993	1.221483001	8.396962577	10.11143009	10.14274626
1.749325954	2.774578534	10.20890899	10.23702073	10.24118078
4.753914175	2.989367841	7.604137674	12.15287267	12.16024385
2.853105369	4.441883399	10.22627036	11.8195644	11.82369469
3.306169778	0.028981758	10.55211569	10.91487238	10.91818337
1.896266247	0.084107912	10.56411317	11.40744953	11.43596589
4.128218558	7.857118427	9.265057419	12.5233472	12.5287257
2.798249521	10.07476485	8.456268934	9.920043459	9.921804166
4.413548443	2.422158796	1.501411209	11.85929606	11.86519013
2.309616239	2.658218618	9.616028277	10.52442602	10.52903134
1.2225653	12.29311145	10.49239805	8.03913844	8.046096738
4.447853531	0.620006335	5.509027984	12.18023865	12.18156817
4.289115627	5.066654445	10.54454931	12.44200006	12.44350282
0.041928777	1.752275231	10.25127472	9.511052842	9.517962399
2.974195348	1.49207295	8.480195775	10.44229428	10.4492412
5.564987013	2.415131023	8.58152386	13.78613233	13.80322538
1.513683437	0.943253372	8.1984639	10.12041124	10.12646002
3.168023828	0.580568651	6.118945911	11.08362079	11.08783014
4.093609613	7.115006168	10.21596719	12.86002329	12.86508266
0.310620558	1.895347497	9.646587787	10.00905366	10.01245578
1.633720871	3.652359311	10.59370557	8.599294491	8.599294176
2.779857914	1.281271156	8.678915793	11.44369226	11.44675829
1.055392787	0.017069132	11.32652387	8.86730802	8.870590936
1.386349191	0.128131818	9.6736047	9.216392912	9.22364723
3.629503861	3.625853862	9.663715739	12.20721712	12.21049243
2.08595495	1.662957017	9.759868808	8.620840848	8.628704121
4.201156956	10.09543287	10.41102737	11.89582986	11.89771038
2.743545719	1.805656249	10.52165436	8.823061612	8.823061715
6.104119694	1.793817964	6.007083116	13.20253789	13.20432282
4.408830733	3.232592551	8.981856164	13.03916558	13.04485375
3.648999584	2.349060346	10.57027827	11.50272832	11.50560082
2.756080449	0.393739464	10.48197763	10.32518352	10.35810671
4.300965002	2.441273333	9.899434143	13.24789065	13.24960705
1.312137199	1.950733242	9.943254961	11.21869875	11.22290912
4.098208275	4.248958137	8.14825918	12.64046385	12.6447448
4.586032208	1.738384087	0.612984252	13.37220876	13.37631239
2.685441071	3.982520238	9.181952284	11.73782826	11.74290961
1.867294837	1.230273674	8.278729488	9.788574373	9.836162899
3.431809923	6.181316539	9.197981615	12.75435648	12.7575739
4.751212243	0.115126541	9.506226326	12.37128728	12.38500766
5.248212006	3.698792086	8.772342402	12.32945201	12.34465918
4.652318331	0.47885989	9.069722309	12.6845647	12.69496051
2.475842779	12.14265239	10.66232473	8.578652641	8.589804019
2.846115076	3.482958288	9.211243005	10.95643642	10.96364902
2.011438302	4.525030379	7.683895985	9.755637781	9.775010793
4.075598093	1.337456956	9.898915227	12.11669176	12.11902996
2.981937234	8.663483937	10.34400108	10.93821117	10.94225305
1.413635712	0.138013509	9.695971365	8.509819329	8.509819125

hsa-miR-512-3p	hsa-miR-216b-5p	hsa-miR-767-3p	hsa-miR-552-3p	hsa-miR-372-3p
0.139441357	4.181875743	0.114380645	3.380419342	0.140090514
0.007859942	7.107674965	0.006736262	2.033667169	0.007887715
0	4.448894909	0	1.356378119	0.788760916
5.439143884	7.752434178	2.963488703	5.193653435	0
0.018079254	8.208229864	0.015440723	3.085914177	1.057282532
0.038205405	2.981465699	0.032407868	1.119829832	0.038350244
0.278293039	1.75616342	0.21842939	4.511071957	1.756356275
0	0.576108846	0	1.91511925	2.120007531
0.769484052	9.495218603	0	2.236256401	0
1.936323324	0.738364703	0	6.103516279	2.186192059
0.139209319	1.407679129	0.114198928	0.139273149	0.139857119
0.117132982	3.497395495	0.096784032	2.00294519	0.117655886
0	0.95159678	0	0.95140955	1.934652983
2.657646837	5.288198333	1.87783436	0.080772108	2.929223857
0	0.96896118	0	0.968840077	3.535975349
4.889421535	4.00315967	0.077535688	1.281682242	6.13796345
6.774991942	0	5.789772139	7.318773659	2.354133676
0	0	0	1.331093471	0
0.158004324	1.457676429	0.128829839	0.158079273	1.4577827
1.54055656	6.612114472	0	1.951442008	0.964078962
0.518644462	0.522956091	0.378848166	4.647662155	0.522791524
0.13824907	0.138916645	0.113446631	0.138312348	0.138891262
0.888215386	0	0	3.281665307	0.888650321
0.244631438	5.49936514	0.194035997	0.244765426	2.379120472
0.865425389	1.418515455	0	1.417945372	2.129058369
2.076272634	0.887779169	0	0.459014591	1.710953632
1.630662191	3.865805633	0.009997896	3.51703341	1.037179829
0.225608739	2.326385474	0.180022224	6.972342748	2.794134978
0.059781694	2.84552762	0.050343567	2.242676228	1.184051653
0	9.029385045	0	0	0.79338388
0.144604688	8.375458084	2.078453626	1.424105524	0.145284292
9.508281558	10.35402302	0.031488601	1.116475152	5.139355535
13.24303336	5.580180974	0.057956438	1.212115245	7.369801569
4.984821856	7.021630211	0	4.084169883	0.682686271
0	4.384217904	0	1.660872937	0.755851909
1.639936169	8.673004731	3.392344518	1.640031268	1.641052228
0.116561428	0.117101751	0.096329821	5.483166363	1.346107403
2.406404799	8.725066034	3.895456017	1.060473067	0.019094412
0.052415537	1.781658771	0.044249494	0.052435755	1.78169557
0.028203219	7.668724498	0.024004533	3.132359566	0.028307743
0.85409976	0	1.802245593	0	2.112448003
1.615748519	9.710519031	4.944236001	6.308075305	0.007867482
7.991261754	10.13888967	5.488480804	0.148580089	2.554924036
1.605463726	11.41187237	0.171779574	5.196943532	0.21566293
8.176917719	4.189484549	3.832464285	2.650946546	7.454433471
0	10.83155192	0	0	0
0.315373406	0.317400098	0.244718329	9.337603858	1.841989779
1.37216844	2.477761305	1.374375435	1.372016333	0.125983377
9.942902048	10.49788127	1.80136988	8.135061285	8.364986798
0.246732421	1.681105682	0.195573535	4.11412946	0.24810967
1.332428482	0.111563634	1.334972641	3.276440446	1.33086998
0	2.770514038	0	2.605374309	0
0	5.193513998	0	3.122382756	0.559393811
2.210478518	0.185180429	0.14894001	1.52828084	0.18514371
1.715647971	1.715124692	1.109081616	3.749408252	1.715148619
0	11.52966684	0	1.317513556	0

0.122180379	7.158684065	0.100787807	7.708522098	2.017627356
0	3.286588432	0	3.189201568	0
0.007375719	8.827080618	0.00632231	0.007378287	1.023549845
2.407684341	10.88392848	5.973040719	2.081736139	1.061159824
0.541437695	1.625165552	0	0.541563686	0
12.39732468	1.937063967	3.722514943	0.097546741	7.943493299
6.546577735	10.07386476	0.006300478	0.007352744	1.023468752
0.638910558	4.001521458	1.785257081	0.639017222	1.128619484
0.754183552	9.300332633	5.993456118	1.659748564	4.781371764
0.000412487	4.52570744	1.001355052	0.000412628	2.809323765
0.180778938	0.181720639	0.146322794	0.180868166	0.181684818
0	4.095022199	0	2.447069171	0
0.237538463	3.182255335	0.188830254	0.237667134	0.238845417
0.865120825	8.239187107	0	3.24385837	0
7.482542703	8.444331265	4.071114079	1.504596186	2.229573383
0.044367225	3.912353276	0.037556555	2.513432704	1.752693242
0.048107491	9.798073935	0.040671444	1.149876805	0.048293919
1.74306464	0.272621079	0.213179748	3.804980872	0.272558945
0.822577082	9.378531541	3.42476914	0.822635677	0
0	8.908996063	2.725583966	3.289457416	2.716691179
6.634394463	3.882833237	4.258667412	0.03789462	1.729017155
2.722607326	11.33749285	0.16153246	0.200956807	4.822801999
0	8.69677372	0	0.671656257	0
0.05712373	6.954596339	0.048148081	0.057145989	0.057349481
1.5131431	0.179197661	0.144410513	1.512925824	2.190348212
0	0.98203737	0	0	1.975839222
0	1.666084891	0	3.678352224	0
0.022039575	9.379099448	0.018797746	3.10421923	7.180351798
0	7.811481058	0	0	0.49385455
0	2.295849671	0	0.807093202	2.042728931
0.128065931	7.88699672	0.105439799	3.107476896	0.128649636
0	4.156469533	0	0	1.159080202
0.356351739	0.35877549	0.273085312	1.93835803	1.933663657
0.000448857	7.816186986	0.000385663	1.001445846	0.000450415
0.278692566	6.376993766	0.21871586	0.278853304	0.280325821
4.757388657	8.738787016	2.260834211	0.209762526	0.210756781
0	9.087968013	0.8743694	0	0
8.027253838	9.548783907	5.714824581	1.64367705	2.388906685
0.710933251	2.538565628	0	3.892207781	12.7373428
0.58735384	1.938492447	0	0	1.060679833
0	3.676509099	0	2.375155618	0
8.726741351	10.76032152	3.849946146	3.212722373	4.959198201
0	0	0	5.73292159	1.549152034
1.069811387	2.685032085	2.681528494	0.022009167	0.02208162
1.180752389	1.182143232	0	0	0
0	6.37479501	0	0.828211963	3.420286113
1.985423442	7.66839274	0.988927979	5.567070296	9.205861699
3.958848432	10.20559025	2.61010752	5.70298993	1.650380127
2.007662871	0.962462424	4.55977998	0.513974273	5.392350714
0.407991484	5.30799782	0.392510233	3.259705321	0.409608622
0.012172572	2.051728726	0.010417007	2.051895301	0.012216029
0	1.846728854	0	0	1.613417069
0	6.067323408	0	1.439772077	0
0	0.890943445	2.646977848	0	0
7.929141355	4.390611417	2.063756831	1.813328657	4.635485073
0.015455302	3.535140087	0.013211508	0.015460786	0.015510909
7.767626212	1.682628892	0.021631885	1.080347596	3.290675296
0	5.798500969	0	1.332958332	0.798899164

0.300905701	0.302799721	0.234532442	8.252511354	0.302727592
0	3.417306592	0	0	0
0	10.71444645	3.954578282	4.328449689	0.814547523
1.870265005	2.043526385	0	0	0.863535708
1.700409416	4.794184598	0.02553834	5.82501727	2.453207771
4.398745812	0.110571766	4.62080086	3.471695922	16.40084959
3.363293019	10.36382565	3.477247525	7.665372288	0
2.180898579	0.436440927	0	0	3.508169906
0.006125783	6.617388036	1.020030276	0.006127909	0.006147338
0.053950099	3.417798927	0.045521529	2.220263885	0.05416185
0.072457824	0.072765323	0.060760637	1.222137737	0.072753638
11.67204057	10.77131839	4.584280169	2.176123637	4.853938047
0.096279159	5.253235847	0.080100845	2.377612508	4.015993603
2.629790303	8.359580925	5.689890368	3.748878909	0.009718573
0.028899314	9.606301873	0.024591199	0.028909893	1.695778195
1.255257108	3.368467523	0.070038643	0.08387318	1.254091817
1.583551595	4.793053428	3.759576406	6.921446647	3.198256598
4.320101198	8.892953338	3.380290116	9.590177697	4.037515818
0	1.135253998	0	1.133855616	1.135186236
2.657429093	10.18972003	0	2.657516092	0
0.070956618	0.071256803	0.059531557	10.57883582	1.216851409
8.798410784	2.538704229	4.306138331	0	6.424239017
0.04215965	3.78585377	0.035714408	4.010414139	0.042320897
0.04420488	5.679492389	0.037421177	0.044221627	0.044374717
0.170867744	5.373793912	0.138741535	0.17095065	0.171709366
0.0210855	3.911553116	0.017989836	0.021093081	2.088764937
0	0.943322611	0	1.514789034	0
0.579569789	2.85847107	0	0	1.05035203
7.901302903	11.70347587	4.529393022	6.196273854	3.005879083
0.338479961	0.340726664	0.260800569	0.338692535	0.340641072
0.221083158	11.3988467	4.49459004	3.653511867	0.222268017
0	5.189526408	3.077555854	0.757201303	1.281540914
2.397682056	0.017085786	0.014542903	0.017027952	3.25173123
0.127684967	2.485461524	0.105139222	1.378237195	1.376591462
2.310122691	11.22284076	3.872950304	3.843501245	0.686194765
0.020153222	2.901194021	1.065156897	1.064007017	0.020226535
1.19591586	2.860662026	0.053414898	10.09815564	0.063762375
0.653000963	8.342357971	0	0.653104455	1.513823275
0	0.849382772	0	1.397015187	0.849356285
0.663880697	5.392010911	0	2.274190279	0.665021873
1.783424462	9.080494118	1.386804903	1.388067664	0
2.756770071	11.80710617	0.296981262	0.391941514	0.394362615
9.369143548	9.918290788	4.849671885	0.902560051	0
0.101491812	7.406353244	1.308281145	3.438773044	1.950341732
0	4.319228621	0	4.014983395	1.529755581
0.610916629	0.612245721	0	2.182605263	0
1.267096719	5.457163412	0.073378721	0.087993387	2.346520341
3.138154861	5.946775185	1.233765562	1.23095657	0.075791914
4.488139899	7.206979107	4.904645762	5.08796829	0.229819649
12.3947305	5.378986494	0.094877054	0.114785025	8.06182341
0	6.254368364	0	0.818294489	0
0.911436938	0.913502531	0	0.911610431	0.479248368
0.123768336	2.472160203	0.102044703	11.14152303	0.124327895
0.24294542	0.244346875	0.192800675	1.675316227	2.844593065
0.118247253	3.676725844	0.097669048	2.006551475	3.070734595
0.113280508	11.04620241	0.093719202	1.338495086	1.337041348
0	0.491124086	0	8.61366697	0
1.405179798	9.885658088	0.112877741	0.137586184	0.138161287

hsa-miR-216a-5p	hsa-miR-1270	hsa-miR-33b-3p	hsa-miR-183-3p	hsa-miR-6502-5p
4.182069241	0.128426774	0.104024946	0.091330507	0.074927517
6.984075579	1.616279568	1.61502244	1.0245031	1.0231477
4.555465615	1.343945916	0.775994285	1.816404243	2.611476494
7.769412521	3.778557626	3.258352768	0.864804081	0.871198517
7.822324907	0.01694686	2.397333645	0.0128079	1.052612968
3.348838287	1.122701053	2.150073303	2.733898166	1.108646992
1.756916842	2.473284357	1.722669226	1.68270371	0.134425452
0	2.59881286	1.039901785	4.68589555	0.58657727
9.575862032	1.291318538	1.997862161	0.77105249	0
1.636499718	0	0	1.656098827	0
1.408043339	1.418471996	0.103862859	1.383227371	2.414735778
3.828846091	2.007011283	1.981312029	0.077790903	1.305824174
1.525243466	0	2.739680619	0	1.532132904
4.902216163	4.270152184	1.243676908	1.234448687	2.2603173
0	0	3.277827325	2.282219379	0.971130035
4.120751488	3.573325366	0.070957978	1.26723434	0.052010324
2.143127444	3.679665642	0	0.711480382	1.250593695
0	6.031798223	0.793904711	0	0
0.158674401	0.145135272	2.098124527	0.102318742	0.08363165
7.01640744	1.951003398	1.541509594	0.964727353	0
0.522295563	3.8515063	2.157074531	0.276384196	2.625608667
1.405458573	4.671096758	0.103191726	4.64828342	1.353262976
0	3.628995012	2.169388897	0	0
3.928261504	2.384179107	1.650533375	0.150524091	1.565098183
2.385359697	5.237561621	0	2.404882356	0.873597364
2.07803688	3.933763467	1.726831815	2.138341721	0.917205416
1.630514603	3.964513711	0.009257436	1.036274799	1.034246909
1.630257594	0.205236225	2.272807765	0.140348536	0.113288833
3.269901226	0.055697727	2.228475352	2.544297998	0.034386606
9.229310459	3.904221662	4.206888475	0.794373948	0.803373554
8.354664849	3.159267395	0.107622898	2.035057432	0.077374618
10.26852615	4.177018504	0.029047121	0.025953905	0.021786194
4.71961274	3.305141864	0.053218229	1.202692202	0.039384425
6.968770426	2.092021048	0.675928919	3.225638783	0
4.482724892	1.960491269	1.668779901	3.345474558	0
8.337158012	6.108342856	1.954292932	0.741690641	0
0.117019266	0.10770895	1.341621525	0.077439078	1.928454946
8.181418048	3.260314759	1.06054295	1.05864983	0.011358113
3.411281505	1.784960289	2.797207046	0.036289754	1.146695821
6.912976259	8.993360298	2.112242825	0.01984387	0.016694319
1.801291626	2.585049412	4.305524834	1.412107125	0
8.561229244	5.209534128	2.35511406	0.005592814	0.004729804
10.14868974	3.931124509	2.071810997	1.405492065	0.079212448
11.20030559	3.383382159	0.154741828	0.134317325	2.149343767
3.890297055	5.246620961	0.130357214	0.113768309	0.092635624
11.1651049	1.480101914	1.483606589	0	0
0.317090189	1.858848981	1.798424369	0.186530674	1.685556829
3.328766568	0.115741779	2.005744091	0.082860802	0.068175409
9.359348129	4.403363395	0.851816747	1.411340086	0
1.681770111	2.389852793	1.655134572	0.151634575	0.121956275
1.981927601	1.339772228	2.732072708	1.313555648	1.291786121
2.91781801	1.267319924	3.425165495	0	2.483270557
5.594105565	3.1274126	0	1.029485279	1.970062996
0.185032989	1.539001403	0.134674938	2.134812541	0.095495615
1.715218229	0.031903829	1.711410552	1.10360663	1.09746776
12.09547825	3.733599811	0.782999808	0	1.342427362

7.625613395	3.508681565	2.433734674	0.080887043	1.317348506
2.975373621	3.767173538	0.634356976	1.141409348	1.162717883
8.204048782	1.024213654	3.202707223	0.005263428	0.004451786
10.66503963	3.41434702	0.015254541	3.078886346	0.01153085
1.858664873	4.883791216	1.349114203	3.269603263	2.50658857
0.097874449	3.884373825	1.290230185	0.065563864	1.879323473
8.969773538	4.68084181	0.005837648	0.005245298	1.610120291
4.05693851	3.085517203	0	2.0648341	1.159813878
9.750890913	3.978977307	0	2.650984296	0
4.646008151	2.001796356	0.000328727	0.000295767	0.000250619
0.181576804	0.165512865	3.959485762	0.115469286	1.443018678
3.935344286	1.872016446	0.905727537	0.908166317	0
1.659473759	1.6742505	3.086459044	0.146755573	0.118216592
7.972219426	4.533896834	1.419906123	1.829130146	0.873306201
7.844056377	3.483021812	1.506124129	0.936136727	1.513872151
3.669074563	5.256789621	2.172899135	0.030881552	0.025876458
9.136231711	1.153446283	3.005581788	0.033402028	1.740153006
2.450656985	0.244929886	0.190743458	0.164268316	0.131589119
9.211991854	3.710274963	0.820338739	0.824471179	0
8.036076127	1.354667812	0	0	0.828323721
2.156768965	4.08702146	1.724751252	0.026480566	0.022224047
10.0310292	3.064260844	1.551473939	0.126770984	0.102781737
8.441772755	1.162409298	0	0.67166034	0.684036827
7.050424705	1.801972904	4.145280126	1.781759634	0.032935829
1.510953065	0.163277234	0.130677592	0.114039704	2.073131823
1.562854935	2.558687001	0	1.977806316	1.565505113
1.665889998	1.660987555	1.672965173	1.99424104	0.769469883
9.32162995	3.274211188	2.088433639	0.015571698	1.063844815
7.554152206	3.244217373	0.925783128	2.365004143	1.613835522
2.29570114	0	1.742117989	0	1.768085857
8.130226454	2.488615702	0.096038316	1.356069793	1.330638095
4.484322016	1.519743346	0.656015117	1.548694822	1.189664573
0.358404732	0.318211758	0.242017359	0.206165482	1.749367362
8.002406046	3.461649514	4.461490641	0.000321838	0.000272708
6.300162927	2.93921788	0.195521994	1.683472264	0.134579763
8.297783818	5.14616068	0.151552578	1.543369685	0.106561377
9.640524227	4.606597965	1.434899885	1.844831297	0
10.06069269	3.658189777	1.048096	1.04661333	1.04398343
1.598338974	2.537546704	1.222454611	2.394778494	1.250920082
2.753738269	4.77832562	1.712857315	2.823570041	0.599398482
3.472145336	1.805541255	1.412246482	0	0.867448373
10.02652007	4.667995853	0.071880441	0.063578832	2.892288213
0	0.970089592	0	0	0.972954378
3.103865314	0.020611379	1.667683331	0.015545016	0.013098302
1.181900287	0.672450419	3.813145377	1.574351145	0
7.177151779	4.416130772	0	2.0941051	0.837803063
7.709814756	3.683595739	0	0	1.987657515
10.15574269	3.169476077	2.622022302	1.978288342	0
0	5.70620993	1.305405896	1.323279349	1.356083359
5.658177526	2.259101352	0.80588889	2.19512238	1.466870085
1.038753282	2.86443695	3.639212929	0.008658126	1.626325528
2.223327537	3.079194466	1.338083202	0	1.930736544
6.175432301	2.819992908	2.161838001	0	2.441224956
1.851574387	4.82136077	0	3.549534854	0
4.434316367	2.755163955	3.264904055	1.543165056	2.534350693
3.073560903	2.65585091	1.049337862	1.64112925	1.637224152
3.290713508	4.029728119	1.080348895	1.077779807	0.015069059
5.896400264	0.793340746	0	1.343149308	0.808753387

1.809502593	1.825590845	1.769309262	0.17939143	1.66203962
4.157745566	3.011712911	1.257087789	1.503969142	2.366371826
10.40134093	2.05034121	1.751604399	0.815718254	0
3.522984638	1.441564682	0.85354676	2.408510075	2.157485907
5.796462939	3.601780526	1.094622975	0.021099013	1.086179484
1.328342471	1.336965997	0.083230715	2.704362636	1.289491582
9.608055478	5.022973927	2.880167397	3.144876936	0.647003954
0.85432879	1.163847617	1.449981466	0	0.443578629
6.690645694	2.349607367	0.004868138	0.004375196	0.003701739
2.551899892	4.645629125	1.780417068	0.03731444	0.031194586
1.2213592	5.378455216	0.055766239	1.829943875	0.041212691
10.28176437	2.177155902	2.166585498	1.128456132	1.723618129
4.437687246	3.215333768	0.073273406	2.662554337	0.053640801
7.933840467	3.74806902	0.007682547	1.030134683	0.005832383
8.869912506	1.697730407	1.692734631	2.109331199	0.017095363
2.94144617	1.261062849	1.252380217	0.056869379	1.226709086
4.382187983	1.20210444	0.696378985	0.702155725	1.239172899
8.572673422	4.75202778	0.037530719	1.144388093	0.028016112
1.134988044	2.028985258	0	3.45805294	0
10.58016522	1.55554645	3.894340931	2.355539395	1.219989022
0.071210993	1.222845743	1.215913142	0.048533868	0.040412196
2.933873678	4.027437755	1.469031733	1.485039851	1.816355718
3.785923963	2.175199017	1.131475627	1.732958587	1.119357173
4.918005759	4.015266035	3.508290707	1.132928039	1.728347809
4.023910608	4.392358602	0.125666292	0.109790214	0.089514732
3.099655171	2.415475876	0.01663415	0.014907228	1.061148369
0.943281294	0	3.503278014	1.929299871	1.522974223
2.465666187	2.463863616	0	2.177974943	2.0053374
10.78950399	5.215647738	1.003922721	0.000869485	2.004422643
2.623801273	1.911036946	3.314129791	0.197706453	0.156743809
10.43917868	1.633330884	0.159013702	0.137895111	0.111396745
5.893700748	3.596069034	2.233493052	2.973378555	0
1.65100373	1.65216406	2.068761687	2.389391318	2.060454171
0.128197207	2.041002162	2.01237187	0.084241635	0.069278804
11.30847793	2.656381432	1.869365174	0	0.69754309
2.411070293	4.622915534	3.257701475	0.014257111	2.394853596
3.458649301	2.258043869	3.582216233	1.18744944	2.209936272
9.016691006	1.505889345	1.520073914	1.535217792	0
1.7939008	5.477067558	0.847042349	1.405615654	0
5.121794373	4.115514117	0	1.551113362	1.191776337
8.977900145	3.551691165	3.670782124	2.37154427	0
11.29699751	2.748517088	0.26220945	0.222431728	1.801324239
9.603387158	3.019224992	2.669808494	0.904175784	1.477712739
7.594716322	0.09398829	4.568161319	0.068071637	0.056293547
3.252070707	0.953948362	3.842230852	2.264419779	0
3.482997968	4.351248471	0	2.730997234	2.270605861
5.736195044	0.081613738	0.067201436	0.059505217	1.853807366
5.489206034	3.797103889	0.058010463	1.220271002	1.81951235
7.297898412	2.340315297	1.614927959	1.583302325	0.11452413
5.103538788	3.054596986	2.410744387	0.076312992	1.300306731
5.875177861	0.813891236	0	2.080254097	2.356709895
2.409156237	2.864310772	0.905208635	1.548927054	0.487668677
0.124261206	1.375642011	0.092999322	0.081857115	0.067372749
0.244132707	1.687543929	3.796196558	0.149631037	1.562049563
2.795050736	2.455289128	1.34609271	1.331690475	0.064664939
10.33589026	1.34609331	3.430562131	0.075414633	1.296947702
1.537412767	3.313629903	1.266430846	2.977525014	0.960066792
9.989471392	2.520180219	0.102684101	2.01634175	2.410601079

hsa-miR-301b-3p	hsa-miR-10a-3p	hsa-miR-196a-5p	hsa-miR-520a-3p	hsa-miR-519a-5p
1.41656383	4.017760816	2.5278179	0.13982657	0.139679158
3.622659629	2.033469079	0.007837713	0.00787643	0.007870123
2.675046566	2.86096164	1.929871408	0.388207849	0
4.030407599	3.3719483	1.414363053	4.897699382	4.164476549
1.058867903	2.076031807	10.69222754	0.01811811	0.018103246
1.122536715	2.156890481	2.982123709	0.038291385	0.038258494
1.762598799	1.759922983	0.276994303	4.367471894	3.30265676
2.121006414	3.65116815	0.573797035	0	3.156163304
1.985823385	1.680286829	0	1.296877296	0.769766982
5.689985612	1.63368772	6.682680029	1.636151105	0
1.415934803	3.352918647	1.410975901	0.139593727	0.139446623
2.001554536	3.277804318	3.829837998	0.117443302	0.117324558
1.934476307	1.524544877	0	0	0.951458029
2.317353959	3.145771409	7.086624652	3.685324713	3.158968122
3.123373943	2.278374002	2.7607481	1.546677744	0
1.287035802	1.922411072	3.406457719	7.590271482	6.368834864
2.543095663	3.431692089	1.596124104	6.457992073	5.653343019
3.992429015	0.791359895	1.719631059	0	0
1.466283664	0.134342877	0.157396895	0.158455738	0.158282981
2.271328058	2.754748539	0.963838509	0.964014736	0.963982095
2.251518012	2.960442033	0.515354654	0.521101567	0.520159987
2.067544647	2.848214463	7.413973715	0.138630152	0.138484321
0.885346766	0.885398453	0	0	0.888361687
0.206240016	1.681668911	2.384364951	0.24543877	0.24512971
1.816024945	2.606519761	0.865087404	0.865715331	0.865599488
2.83600018	3.02397431	8.187840534	1.216040449	1.709498343
4.304393477	2.373420993	11.06633311	1.630567553	3.22652914
0.191072689	0.188474964	0.224637381	2.327972161	0.226054678
2.839672039	0.05217677	0.059591676	0.059922732	1.184599656
4.416409037	2.680612149	1.713863226	0	0
4.038811201	3.567959784	1.425563999	2.089227615	0.144853635
2.153065173	1.118982517	0.036995671	9.219529617	10.41038621
2.274782114	1.841042012	0.0688112	14.84722258	14.29577512
1.176105354	3.378970957	10.4005939	4.140111467	4.522078751
1.658681993	3.41163037	0.754387195	0.755464669	0
2.907413727	0	1.258393148	2.40290006	0.740761032
1.999734954	1.352946993	0.116146129	0.116869896	1.347202276
3.258354997	2.079919531	2.406546862	0.019066364	2.671974416
0.046320201	3.034963593	3.832151805	2.213989994	0.052490687
1.694236872	5.454977524	10.11205247	1.088891041	0.028241533
1.401298284	0	0	0	0
2.356780355	3.359019401	1.02519523	1.025069237	1.025092633
2.545287124	0.126586896	2.103723386	8.538300357	9.212459703
0.182171415	2.284813248	0.213618018	0.215200224	0.214941918
4.006894874	1.516735227	3.279765311	7.381928521	7.410802728
3.45650171	2.204057875	0	0.916684057	0
3.351028026	1.842749535	4.748617652	0.316529459	0.316086791
1.378195785	1.3774498	2.48042308	1.371187556	0.125622977
2.587307638	2.112457761	1.799478208	10.56356243	10.55406094
1.688842774	4.470099341	0.245634427	0.247549352	0.247236615
4.048835907	3.453050614	0.110662752	1.982425613	1.331911101
1.653062416	4.589407342	2.419321289	0	0
1.00905806	2.94165945	8.128006586	0	0
0.157583705	3.693749021	3.535294756	0.18476607	0.184555214
1.716342894	2.140764194	2.142424552	1.715372809	3.48412911
0	1.704078204	1.316849311	0	0

0.10611518	2.016091259	0.121740404	0.122507199	0.12238214
0.631520315	3.911892534	7.469563733	0	1.130597591
3.034570118	1.614114062	0.007354884	0.007391174	1.023615219
2.406553712	1.660350947	1.660174557	2.897520867	3.262637855
2.5433786	2.545497081	8.085349572	0	0
3.579058582	0.084243589	2.995342779	13.29467951	12.47234328
3.620241858	1.024110051	2.031571932	6.038684059	6.167997198
2.23490126	0.629171191	1.126194886	0	1.782654047
1.962369031	2.215371422	3.971921844	1.2773179	1.277083654
2.001777576	3.323901569	1.586597852	0.000413336	0.000413011
2.644151532	2.641016667	1.521234331	0.181316408	0.181110701
3.55577971	2.189759918	0	1.873474124	0
0.200600789	3.144163035	3.186124256	0.238313726	0.23801695
4.642522324	2.964071495	1.417137771	0	1.417708888
1.911958061	3.223595414	1.504388597	8.18447644	8.377719324
3.660611884	1.141487234	11.71966856	0.044468449	1.13842415
1.767624298	3.675292457	3.686178748	1.14956722	1.149714342
1.745761335	0.22376373	0.269738009	0.271920165	0.271563688
4.237439855	1.361105049	2.534737081	1.757093774	0.82280146
2.721114596	2.722088285	0	1.751151355	1.358769003
2.484046487	2.155614826	4.272708724	5.399024954	5.834343078
2.25029804	0.168874303	0.200023457	0.20147202	3.071939724
2.291186892	2.477220123	9.938892734	0	0
1.799926559	3.712635015	1.177466691	1.176528007	0.057206467
2.6366716	2.184062095	1.51472248	0.178800758	1.512304483
0.981518151	0	0	0	0
2.622490939	1.27717171	9.912958882	0	0
3.562552009	1.071479691	6.637300356	1.670164815	2.092897552
2.907751406	4.465469472	4.755923403	0.493198701	0
0	0.801993311	0.806559196	0	0.807271968
2.817043451	2.03447103	4.468449859	0.12841232	0.12827977
2.459395794	3.24551105	7.801014886	1.158464486	1.158152921
0.292595051	0.287972146	0.354496663	1.93598394	0.357204652
2.587028331	3.702612991	2.587067064	0.000449782	0.000449428
1.763515873	3.252845082	1.76373461	1.759098754	1.75999248
1.598709172	1.597020009	3.382776562	4.426519365	3.996850336
0.873973253	0.874026531	1.432250002	0	0
3.392770163	1.643999008	0.01501769	9.889641852	9.627846255
3.608469877	0.703195024	2.141878528	0	0.71127566
2.325134808	1.696878116	1.057964514	0	0
0.855339938	3.365621045	2.374792605	0	1.807759618
3.19849988	4.101196873	2.983373223	9.042907474	9.027661919
1.548671204	2.76481694	0	1.549092988	0.970830552
1.071425869	3.561856457	1.070002234	1.670019692	2.092739739
1.846230562	1.174192746	6.895491182	0	0.680273772
2.547730684	4.309501471	5.893514267	0	0
4.56826569	1.57152572	3.305133942	0.989167636	0.989157692
0.740201	2.418494282	0	6.409063181	3.889559124
2.619303113	2.010426477	0.959280685	3.032334498	2.183444015
2.989946735	2.993164996	1.612337621	2.126412639	0.816153538
2.640093763	1.039779358	0.012137793	1.038803033	1.038839471
0.976629316	4.591146926	0.987101145	0	0
2.413379503	2.991923917	3.396975603	0	0
3.290564596	3.003888102	0	0	0
0.649248694	3.944896347	3.221273528	7.650377247	8.191018206
2.065393678	2.065203488	7.146741292	0.015488314	0.015475686
0.022591846	1.683408799	3.93235482	5.128814309	6.638433761
4.991735674	3.845385604	1.721709767	0	0

2.509087494	0.246583373	0.299454599	0.301986145	0.301572451
1.810093342	3.855332876	3.465156848	0	0
1.74330932	1.74381751	1.352306372	2.521602936	0.814086678
3.221154303	4.478837274	1.674871659	0.442160562	2.782696054
1.096884515	2.451202662	1.094997303	3.140453923	1.094600137
4.463571388	1.334787792	7.108440575	1.979205466	3.041548099
4.689264002	3.38276943	2.015915778	2.962676106	4.251035556
2.454218399	1.857840266	0.851158526	0.853694689	1.177067588
2.835789347	2.612831246	0.00610853	0.00613858	0.006133685
3.943419787	0.047161303	0.053780743	0.054075792	0.054027706
3.311331792	1.226348829	8.145219857	0.072633405	0.072566229
3.361807467	2.174257069	2.506725707	11.70586885	11.20336742
1.933968489	2.370864023	0.095949359	0.09652406	1.290396663
1.031735286	3.367620147	1.623011338	3.216953863	3.748863886
3.455201244	2.936797551	1.696461201	1.091040784	0.028938635
2.66190752	1.891533364	1.255997257	0.084046617	0.083967042
2.839813261	4.307382764	7.724008664	0.702082555	0.701849524
2.525655317	0.042202941	2.529529694	4.40303884	2.528990355
1.494119822	2.034907044	2.858313486	0.644596441	0
2.946737511	2.666851871	0.685470883	3.461606881	1.857349576
2.281843685	2.28082945	0.07072572	0.071128023	0.071062445
1.991513215	2.546889216	3.146373091	6.464897093	6.190813052
1.745999905	0.036972445	2.174563701	2.998851176	0.042218751
3.902210897	1.75305052	1.138520878	1.13779792	1.137932832
1.500057686	2.162766366	0.170195905	0.171367108	0.171175992
2.679329937	3.799383415	3.56222626	0.021131135	0.021113678
1.514028445	1.923364894	5.190962709	1.514908805	0
4.015962428	2.751113739	1.401202566	0	0
2.813026086	2.327351235	1.003917842	9.916394006	8.215945557
3.059993322	0.274757599	0.336759828	0.33976138	0.339270667
2.76024088	1.625069916	0.220138127	3.652719874	2.782657953
4.642374483	4.125652601	3.907795911	0	0
2.07188708	0.015030155	2.072352795	0.017058382	0.017044423
2.034827342	0.109436004	2.038094011	1.37739298	0.127898017
1.180788859	1.556606579	1.558784325	2.934600644	1.560061499
2.899097584	0.017779865	2.085376519	1.063879277	0.020180097
1.199957017	2.253418807	1.822498955	1.195428452	0.063601187
3.12162842	1.804166182	0	0.653658703	0
1.394487198	3.088443325	2.103618786	0	0
2.625884244	2.27941931	3.127801937	0.664522599	0.664266805
2.350999796	2.351705214	0	0	1.388237975
0.318967103	2.003129777	2.019337589	0.393269287	0.392659611
2.183556409	3.173551918	6.464194595	9.437281432	9.091139969
2.390371456	2.994709645	0.101140573	1.304808351	0.101652846
3.254083743	3.391539777	10.92944882	0.955129927	0
0	3.24618437	3.316859587	0	0
1.906285904	2.341793169	3.386106878	1.905731649	0.088092687
3.127353813	0.065601521	2.302487684	0.07566587	2.637144297
2.781005167	3.625537732	1.642400833	2.804176263	4.819008902
2.435415389	2.433621815	0.11432741	10.85832576	11.9413671
3.955659244	2.532977088	2.881613241	1.358810741	1.358634092
4.678453837	2.666733963	1.243344187	1.51652724	1.245393043
2.465486641	1.372915469	1.368742871	2.023511656	1.367067459
0.204901296	0.202053621	9.927758105	0.243745082	1.674467642
2.783809844	2.00370319	2.455791968	1.35149219	0.118441064
1.989254873	1.343791875	1.991535356	0.113578409	0.113464419
3.534843524	1.253955861	7.424629131	0	0
4.014848806	1.410497132	3.1419695	1.404101567	2.067548539

hsa-miR-502-5p	hsa-miR-520c-3p	hsa-miR-520b-3p	hsa-miR-34a-3p	hsa-miR-2114-5p
2.07537615	0.134499637	0.134495829	3.123727717	1.419015367
2.033787511	0.007646377	0.007646211	3.206813838	0.007461437
1.757057841	0	0	0.769894105	2.075290059
2.600195059	1.811141895	1.811141322	2.960912243	0
1.656089787	1.05847964	1.058480082	1.05878929	1.058820025
2.158609695	0.037094168	0.037093306	2.156688064	0.036135458
2.926787017	0.266023611	0.266014251	4.129120604	6.770251195
2.854735018	0	0	1.388938429	0.565989958
3.621520312	0	0	2.809511909	1.982500508
2.750129256	0.733400777	0.733398742	1.633944763	0.731680952
3.890529197	0.134277842	0.134274043	2.068069822	2.868374968
4.078906011	0.11314538	0.113142303	1.354255161	0.109739105
2.735301641	0	0	3.511347234	2.926183735
1.883936095	0.078174685	0.078172702	2.918252891	0.075974027
3.75912594	0	0	4.652565999	2.277585445
2.699788958	4.118646176	4.226892459	1.286360955	3.869634934
3.761221946	4.950984054	4.950988502	2.99256405	3.963574224
3.668817803	0	0	3.761553013	3.572127122
3.197126849	0.152221441	0.152216992	2.571254985	0.14730746
1.951187503	0	0	1.951654206	2.945369836
2.269936781	0.488065124	0.488042213	3.76931359	2.289062569
2.071670655	0.133359912	0.133356145	2.509487296	0.129194787
3.909601441	0.886429062	0.886428186	2.42218205	0
1.688809603	0.234357651	0.234349794	1.680784057	2.849858133
3.954070991	0	0	2.387993081	2.127130967
2.617905048	0	0	2.627818213	1.472975455
4.37931078	0.011360667	0.011360418	3.054078462	0.011083511
3.405257483	0.216400778	0.216393727	1.635292111	4.573557344
3.264408335	0.057961692	0.057960282	3.064037752	2.57630091
3.830290167	0	0	2.681044353	0
1.432466127	0.139433301	0.139429318	1.429191312	7.094051486
3.757962473	4.429060869	4.35074142	2.152382128	1.728698818
3.30187566	9.954980605	9.971315479	1.216060229	2.882978251
3.463002897	2.793751693	2.793752561	2.807028244	5.167996093
3.405716288	0	0	2.217016235	1.960159912
3.03821649	0	0	1.638412727	0
3.482290692	0.112597456	0.112594397	3.937581312	2.789561118
3.99864339	1.061499406	1.061499871	3.896931534	0.018036381
2.544519457	0.050844084	0.050842866	1.166066626	3.828617746
1.091273052	0.027400686	0.027400063	1.090999943	4.661875296
2.368327617	0	0	3.358080645	0.850852978
2.033701653	0.007626774	0.007626609	2.033345989	0.007442317
3.395726912	6.316699013	6.316688292	2.09614123	0.138614047
5.826230789	0.205917785	0.205911189	3.078388726	1.617159067
2.190959593	6.503258256	6.503245062	2.181746722	2.995661167
0.914358182	0	0	0.914449224	1.480120564
1.853827412	0.300743726	0.300732596	3.618027878	5.599377296
1.379717654	0.121080042	0.121076699	1.37713669	1.380109224
4.0854069	8.391533485	8.384286751	3.23255625	4.512713673
3.188013793	0.236338173	0.236330225	1.685745805	3.204270828
2.764222894	1.338065084	1.3380674	3.255538389	1.339603744
0.744636389	0	0	0.744519822	2.204884026
3.036298076	0	0	1.887236341	1.643432386
4.532583234	0.177171623	0.177166213	2.199965978	0.171206702
2.469963502	1.109136663	1.109137479	1.715909899	5.811244871
5.315357515	0	0	2.98629672	2.663048082

2.465086948	0.117982332	0.117979094	3.66386413	3.511427123
1.488550975	1.124364344	1.124361684	1.122478139	1.122314301
3.034956019	0.007175537	0.007175381	2.031390145	2.031903457
3.09006661	0.018781109	0.01878069	2.671156204	1.661089828
3.430692459	0	0	2.062437952	0.987583552
4.122374715	10.7525095	10.75364201	2.708945069	0.091566096
1.614200664	2.355229956	2.355229963	2.031282464	0.006977957
3.698997504	0	0	2.026945191	0
3.681068161	0	0	0.747689103	3.180802949
4.86005512	0.000401473	0.000401464	2.323771388	3.809428205
1.528614295	0.173905536	0.173900256	2.639774689	2.660791707
3.852348584	0.905053868	0.905053136	2.66944635	0
3.671413322	0.227667323	0.22765977	2.799349333	1.674781856
1.81468426	0	0	2.129778007	2.126679814
2.71045616	5.284949777	5.284950941	2.711855126	1.503312576
4.206835631	0.043059545	0.043058531	2.509303576	3.666876522
2.795182321	0.04667824	0.046677132	2.79166361	2.529290618
2.446165177	0.259164537	0.259155512	1.742158097	1.756201081
3.042181863	0	0	3.625860204	0
2.718663863	0.815500353	0.815498932	1.750394776	0
1.121738315	2.158588085	2.1585883	0.033127104	2.158420267
0.177435437	0.192966207	0.192960156	2.697953799	1.582271865
2.471986832	0	0	1.536655713	2.777598074
3.844247885	0.055394199	0.055392859	2.827639274	2.565701166
2.192348405	0.171522761	0.171517575	2.183101449	2.197955767
3.67270392	0	0	4.55738463	0
3.601149241	0	0	2.624201984	3.91413291
2.684460305	0.021420999	0.021420519	1.071448976	0.020886264
2.140754012	0	0	1.258732313	0
3.016439476	0	0	0.802022715	1.340187145
3.096466142	0.123618482	0.123615052	2.813700547	3.706861298
4.17206558	0	0	3.141540317	1.519796399
1.944137801	0.338916456	0.338903232	2.629592449	5.413738253
2.323959174	0.000436871	0.000436862	2.323933819	0.000426483
1.769782743	1.773537055	1.773540606	2.448573283	5.206178311
4.894742718	1.603112102	1.603115503	2.722837336	0.194258677
2.980360468	0	0	2.147327246	1.430141472
2.38882537	5.121139767	5.121138912	3.978369272	0.014284946
3.516935447	1.216396627	1.216394556	3.334796349	1.891600151
2.320990175	0	0	0.57622143	0.578857215
3.101660885	0	0	2.120804786	1.406980621
3.72752843	6.544399486	6.544392594	2.697790843	1.291633922
2.542606844	0	0	4.125349309	8.142604525
1.671175454	0.021383793	0.021383314	2.682765021	1.07138181
2.302161927	0	0	2.492297702	3.160295449
2.328402968	0.825458842	0.825457497	2.5489207	3.964633279
1.98536094	0.988964955	0.98896487	3.153888338	1.98528521
1.261906571	3.278964137	3.278965701	1.952401025	0
3.641836476	0	0	2.956482997	1.568512899
2.809757923	0	0	2.994444375	1.382089834
1.039859294	1.039537191	1.039537491	1.039763728	0.011549379
3.416067146	0	0	4.689426581	5.157166074
2.631079967	0	0	2.155614838	1.437397972
0	0	0	2.425585755	0.888048733
1.144691818	5.269896577	5.245352642	0	0.651193917
4.933907428	0.015027947	0.015027615	1.645466096	2.066243399
1.684119904	4.373842169	4.37384103	2.921431501	0.024052891
3.00164251	0	0	3.584823722	0

4.043912435	0.287217107	0.287206681	1.809608623	5.095851502
2.948444126	0	0	0.990561408	2.323219899
1.742171633	1.349856221	1.349854972	2.882387191	2.708747993
2.579937803	0	0	2.801369983	1.862918498
3.309073918	0.02916603	0.029165364	2.451013524	4.052010429
3.631928906	0.106369533	0.106366678	3.775366072	1.984518805
2.969412366	2.562971443	2.562971834	3.194768548	1.769735227
2.674608501	0	0	3.135483857	3.454259797
4.888509024	0.005960008	0.005959879	2.02611098	0.005816423
3.56572409	0.052327428	0.05232617	2.547134084	0.050930218
3.119589688	0.070194169	0.070192417	1.852537888	0.068249546
3.192787879	8.992149946	8.969011983	2.994596322	1.136413451
3.210933013	0.093127325	0.09312489	0.082846323	1.938566242
2.041539827	1.031510138	1.031510378	2.364672755	3.506867414
3.456947714	0.028075704	0.028075065	2.119826018	3.72349975
4.069350472	0.081159508	0.081157436	1.891168768	2.334499448
1.878310347	0	0	1.88167207	0
1.769012977	1.152744954	1.152746083	1.767323623	0.04553631
1.786364534	0	0	2.429375292	0
2.101432692	1.183901805	1.183899526	3.566258189	1.55555856
2.284335436	0.068746556	0.068744845	2.881328934	6.189336507
1.45497351	6.32620324	6.337248999	1.993272107	0
2.50312532	0.040922898	0.040921939	1.134601027	1.134880684
5.252837942	0.042902437	0.042901427	1.140914187	2.513307508
3.667524955	0.164476838	0.164471925	1.498339237	1.503942742
2.089250899	0.020494971	0.020494513	2.088247794	0.019984422
1.922729566	0	0	2.242101112	1.513672751
3.716562833	0	0	2.985067034	0
1.589819846	5.983632592	5.983632511	2.32734409	0.001152696
3.706146972	0.322293731	0.322281437	2.589554029	0.308862942
3.822697946	0.212122169	0.212115304	1.624316003	0.20456986
2.78369899	0	0	4.246100597	0.751821325
2.886013417	0.016549534	0.016549167	1.05539623	1.055421501
2.483490201	0.123253792	0.123250375	2.032666123	0.119473711
3.062655609	1.182485096	1.182482808	2.501292946	0.678375329
3.904160296	0.019589989	0.019589552	1.663462249	0.019102983
2.588223572	0.061559616	0.061558108	1.821563204	6.791559716
2.602251124	0	0	2.608854838	0
1.393968865	0	0	3.224990875	2.35855662
2.458530744	0	0	2.909155353	1.15259448
2.09304647	0	0	0.837632238	0
6.147487885	0.371657558	0.371642413	2.001267695	2.754491228
0.900020776	7.352512586	7.320633099	2.663365101	2.659964303
3.754513281	0.09813616	0.098133567	2.388206389	1.956058947
2.932523679	0	0	3.102942482	1.938768108
0.600997183	0	0	1.081631376	0
3.179569852	0.085123378	0.085121188	2.944967555	4.093227834
4.143790788	1.23495352	1.234955207	4.329267435	0.071072016
2.334135464	3.15887236	3.158867751	3.393015077	1.652511049
3.04971319	7.944212167	7.935791074	1.347580965	0.107523225
5.400168083	0	0	2.316044886	0.814213719
3.840714039	0.470861805	0.4708581	2.276443712	0.467579818
2.470414856	1.373876943	1.373879454	3.823605856	5.862912309
2.831851923	0.232767907	0.232760123	3.156846197	0.224214926
5.028469419	0.114213466	0.114210354	3.804344809	2.011300878
1.345704309	0.109451392	0.109448437	2.42805525	0.106178892
4.10067461	0	0	2.142146969	1.757146978
3.556179602	0.132666072	0.132662329	3.117410242	0.128527745

hsa-miR-34b-3p	hsa-miR-643	hsa-miR-130b-3p	hsa-miR-130a-5p	hsa-miR-765
0.104173996	2.025214953	7.325840558	3.075621546	0.093300431
1.615034745	2.030896649	9.28006523	2.840906996	1.024638121
0	0.378404187	5.255142061	0	1.381991609
1.417093996	2.619942871	7.908626864	1.82209723	0.864188361
1.057597624	2.070013041	5.552650054	1.056835351	1.650918481
1.725960829	1.72071685	6.921228567	1.723256906	1.116264243
0.195602211	0.171838968	6.411683121	0.182646913	0.172161495
2.636368332	2.661660844	6.288411861	2.502343974	2.165181776
0	0.770202215	6.388422965	0	1.305693375
0	3.075229418	8.935805136	2.426086292	1.961763094
0.104011632	0.093005802	4.605113165	2.472229575	2.02490575
0.088406333	0.079298037	5.960452115	1.337063228	1.96422654
0	1.528123627	5.6469703	1.938188477	1.939282048
1.243769288	1.85735212	6.846018715	0.058701125	2.616531459
0	0	6.439873185	0.969010785	2.54416834
0.071053266	0.063955805	6.62853002	1.273500643	1.26911072
0.705746654	1.922165341	7.896836589	2.38364402	0
1.333368323	3.514435713	5.985484374	0	0.797625578
0.117053599	0.104398296	7.170332387	0.110204851	2.074384499
0	0.96455952	7.681008752	0.964131694	1.542867535
0.330981887	0.283769424	7.183944553	0.304931511	0.284393713
2.043088373	2.457878645	4.774171983	3.072133173	2.790481443
0	1.858268131	6.202196906	0.888289121	0.889460681
0.174437152	0.153859453	7.21939033	1.635542351	0.154139927
0	0.866744011	5.094039991	0	2.14404972
1.216388206	0.452105282	7.132352193	1.502608093	2.907927687
1.037424972	1.036461954	7.427769715	0.008829707	1.036477299
0.162204777	1.581893201	5.438269893	1.594680926	2.237773857
1.183700496	0.04192846	6.893841821	0.043972323	1.178224932
0	0	8.308029755	0.791607652	0.79352098
3.333366076	0.096301857	8.606490339	1.406624494	0.096460411
0.029082775	1.112992244	5.771635708	1.719463575	2.465910379
0.053287104	2.249178029	6.088768618	2.254813345	1.204033138
2.113847771	0.680853233	6.744568758	0.678283891	1.19261834
1.668660577	0	6.399401782	1.67379134	0
0	2.437998102	5.966152628	1.264910584	2.626637873
0.087998121	1.329700154	6.820456793	1.335581744	1.329884937
1.656779793	1.654246099	7.482742276	1.655477797	0.013715188
0.040779532	2.786812297	4.713524626	0.038713502	1.768050153
2.437951097	1.086621699	5.787527852	2.435225467	0.020211286
0	0.855408802	4.561789024	0.853896359	0
1.025204357	1.613934641	7.984030539	2.031427835	2.030837331
1.424563595	0.098675152	6.047213574	2.832149283	0.098838342
0.154984607	0.137211426	6.095692523	2.228031151	1.558651209
2.151852879	3.165975204	6.764718857	0.122738003	0.116328921
0.915779519	0.917638351	7.087329471	0	3.056012982
1.798929612	1.759096282	7.961978145	0.203308603	0.191282952
0.094297283	1.987047186	7.125341155	0.088997342	1.987327201
0	1.410367779	5.996653449	1.407849034	2.38653909
0.175775967	0.155000868	5.568313794	3.083927005	0.155283958
1.963324325	0.075459795	6.989076772	1.321233325	1.315848858
0	0	6.188983166	3.328032088	0
1.374915794	2.136150759	5.079000859	1.672936326	2.135812485
0.134879019	2.579159597	4.939798553	2.594755204	2.140298756
0.026776018	1.70681279	7.440641782	1.105662512	2.454884376
0.782957219	0.786918856	5.929781752	0.784897262	1.721506716

0.092001996	0.082463912	5.070834568	1.986875062	1.978354987
0	0.639524946	5.592536532	1.134644809	0.639435803
0.005864667	2.029013986	6.83339262	1.023421488	3.031028557
1.061483188	0.013905693	6.786814657	2.07620328	2.0747312
2.084009956	1.654106794	6.932952656	1.645209418	0.537031697
0.074264372	2.669591759	7.780790005	0.070242935	1.280723288
1.023640664	2.028914635	7.481861159	2.352383701	1.023051856
0	0	6.291186535	2.764956499	0.637125807
0.750373137	1.286399289	5.654582119	1.980912038	0
1.001334963	2.001639525	6.716447126	1.586527952	0.000300991
0.132565691	0.117874401	7.203026645	0.124603363	0.118076346
1.471316547	1.882055184	7.949494295	1.473063707	2.460421431
1.635252327	1.606856931	5.913145002	0.159069179	0.150258288
1.419850404	0.866439418	8.603801578	1.422265275	0
0	1.508551952	5.978206598	1.507347753	1.508513914
0.034651064	1.741397521	5.921738944	0.032917888	3.175835875
1.149307495	0.033990809	6.614908575	0.035617778	1.753898777
1.707715854	1.674455739	6.23598969	1.690299163	2.336428675
2.0757357	1.372295029	8.182811966	1.767561789	0
0.815979022	3.326259167	7.892756259	1.761774993	0
1.724814326	2.142623975	7.381848422	0.028208399	1.719696223
0.145982239	0.129466162	7.956673236	0.137019547	1.528913
0.665561864	2.114052684	5.797665971	1.855534683	1.862502134
0.044342898	2.537115529	5.448638647	1.787110358	2.537273209
0.130874215	1.478025552	8.339716002	2.913946155	0.116607712
0	1.563990965	7.309769298	0.982081514	0
0	2.654083742	6.12730295	1.287360149	0.758407248
0.017399488	1.068099389	6.435726761	3.092687644	0.015857125
2.477160444	0.486587032	6.381095833	2.346694491	1.285803099
0	2.320061902	5.190776132	3.041593463	2.319902331
0.096173845	0.086131509	7.791633137	1.365128928	1.994635887
3.548406965	2.313512702	5.31983388	2.30468569	1.167191097
0.242452398	0.211158294	5.693347493	0.225318511	0.211579133
0.000358116	1.001416838	6.872761798	2.001818607	0.000327523
2.396954068	0.172048493	4.512180541	1.705912485	2.352407032
3.516087588	1.547764737	6.781399024	0.142382815	1.548129087
0.875855121	0	5.572798065	0.877137825	1.439167168
2.061032107	1.640093852	8.622001326	3.064049436	0.010885283
0	0	6.525624328	1.226650152	2.175611137
0.578904335	1.72972905	5.785149471	0	3.519182856
1.813596978	0.860313649	5.846311165	2.131808704	0.860266079
0.071977148	1.898295903	8.993202719	1.276915259	0.064875534
0	2.284447863	6.080704505	2.767848484	0
0.017369547	1.664774251	4.280016098	2.086454958	2.409241832
0.674140251	2.342260579	5.282738158	2.119437313	0
0	1.379202404	6.389005191	3.074048272	1.379110814
2.307460083	1.57235799	8.699569619	0	1.986560716
0	0.747374931	7.215319114	1.970179733	0
0.955730882	3.118112639	6.579634411	0	1.607251099
1.400982271	0.39970844	7.356142277	2.317831632	0.399611936
1.038983323	2.047546443	7.086296959	1.630469702	2.047576076
5.683636085	0.52992525	5.383906935	2.427601447	0.994796433
0.881641767	1.446144668	6.632893688	1.444074997	0
0.889419139	0.891774136	7.08929159	2.435389703	0
0.652065669	0	5.701218559	1.534466699	0.657104895
2.3866679	2.383837795	5.289188721	2.385191069	2.060117851
1.080373808	1.078199417	5.9113675	1.079279784	1.676722004
2.040113357	1.341875701	9.155511422	0.797228749	0.79910557

0.209486755	0.183559524	5.389409123	1.749989606	1.732656466
2.374025804	0.321085868	5.312557901	1.681377442	0.320992817
1.355236608	2.548823666	8.350862504	2.543546005	0.814931364
2.733657622	1.715881662	7.862773452	1.907022407	1.715528938
2.119263534	1.09205033	5.707023415	2.442221091	2.928248542
0.083345272	1.943943172	8.305335677	0.078754244	2.70835037
2.03665914	0.632767413	9.438345115	2.045809966	1.130571125
0.421877647	0.854860875	5.774139453	1.69248929	1.468876429
1.608449446	1.607652077	6.261158622	1.019475343	1.019235189
2.80309706	1.161650396	5.850472082	0.039812621	2.526225837
1.220284472	2.260359596	7.42800405	0.052921794	0.050492453
1.133002105	0.03024189	7.563646931	0.031676809	1.129246358
0.073372266	1.277353173	5.806723964	0.06940589	2.666040025
3.502684781	0.007014216	7.399818186	1.030685087	0.007023769
0.022739291	1.088699574	9.028202016	1.69076858	1.088739076
0.064264247	1.866779507	7.283190095	2.63389574	0.058012973
1.894616332	3.140860615	5.822327923	2.72273462	1.218556732
0.037577668	0.034056172	8.307459038	0.035686517	1.145290654
2.052199572	1.819863781	5.61692523	1.812243468	2.91899863
0.680598263	0.685541586	7.319695102	1.19426746	1.19862183
0.054720691	1.209231972	6.803255762	1.212556668	1.827300592
1.100104712	2.029070733	5.955209873	2.232090413	2.028789508
0.032961827	1.127752065	7.245827814	2.161175382	0.029944332
1.137656399	0.031311069	5.541613742	3.503065079	2.165093405
0.125853684	0.112053151	5.778494711	0.118378896	0.112243112
0.016653995	1.661543116	4.565895413	2.895494977	0.015180044
1.516139872	1.928801619	4.993254923	0.943319553	1.928760455
1.046451733	2.174013725	9.628659171	1.051565352	0.576245031
0.00096764	1.003826856	6.826649768	0.000922712	0.000884865
0.231989724	0.202433338	6.725815759	0.215822506	0.202831622
0.159264989	0.14088498	6.525723444	1.584786992	0.141136267
0	1.290138231	7.677544647	0.755484011	0.757613127
1.054279731	1.647112152	6.355272318	2.067355195	0.012286841
1.371065085	0.085894887	4.813191161	1.364159595	1.357822291
0	1.883485962	8.26095734	1.573631378	0.684359807
0.015924866	1.658245769	5.747666275	0.015159965	0.014517627
1.812695371	0.044437617	4.695540067	0.046616158	1.188671624
0	0	8.505005873	0.648964347	0.65154901
1.799650299	0.850078912	5.849148413	2.375928016	1.404534199
1.835921767	2.818347686	6.547245914	0	1.850663738
1.390436538	0	6.084994585	0.841344227	1.395804566
0.26269438	0.227949629	5.076031199	0.243636802	2.565055147
0	1.876848902	7.621923843	0.90267719	0.90371718
0.077157386	1.290989246	6.427189381	0.072956288	0.069472978
0.954726028	1.532412144	6.731998237	2.938372592	0.955760705
2.70309145	1.099113765	4.539242536	1.759582403	0.608430977
1.26397738	2.907016497	6.087971459	0.063695657	0.060708508
1.852075574	1.221639829	7.271208187	0.055038288	2.599320904
1.615264256	2.243954545	7.69517922	0.153758633	1.588556407
2.744086013	0.077787097	5.652234731	3.885263503	1.957458938
3.973306718	1.765477121	8.09242355	2.331462356	0.819343277
0.466020903	1.262482154	7.086597422	2.46271798	3.135836466
1.360807505	0.083455985	7.5650303	2.427373494	1.348047761
3.100327855	2.718147821	4.652505016	0.16225163	1.618451257
0.089201548	1.967095949	6.030791513	1.339947338	1.967358912
1.970018184	0.076868742	7.610775939	1.327049724	0.076990626
0	1.281547374	7.431677472	0.480778241	1.281301589
0.102830858	1.381858968	8.226560954	1.388996416	1.382082575

hsa-miR-939-5p	hsa-miR-1226-3p	hsa-miR-4742-3p	hsa-miR-219b-3p	hsa-miR-520f-3p
2.506718817	1.417389013	2.06379505	0.094466422	0.136969385
1.615782954	1.616134803	1.615790212	1.024715129	0.007753587
1.34995284	3.582844169	1.349839548	0.784823772	0
3.113865013	2.382697701	2.963174599	0.863839486	3.23980485
2.400041775	1.655900469	2.400069809	1.056285629	0.017828724
0.032495559	1.7318214	1.121844493	1.116665819	0.037651454
2.890238049	1.765247799	2.890742927	1.693101284	1.768827172
2.127801284	3.170157219	1.391199667	1.413263305	0
2.458725829	0.763363288	2.813052792	1.304819208	0
1.63595527	5.535526157	3.043398361	3.311880371	2.183117203
1.411790754	0.121966973	1.411895514	0.094321865	0.136742492
2.770833641	2.002930757	2.435721066	0.080390324	0.115139826
2.73714253	2.253360592	3.098313498	0.952032447	0
1.24920671	1.251444194	1.249256587	0.056623943	1.884269796
3.275954055	4.536470337	3.275933895	0.969280912	0
1.284723209	0.082422658	2.68868951	0.064809678	1.926546382
1.59686606	3.111352773	1.596789767	2.389340325	5.807172732
2.690735832	2.851625631	2.857599224	1.339142236	0
2.118296195	1.467299778	2.901881562	1.433274472	0.155109707
1.540494824	2.271169118	1.540485292	0.964442472	0
2.220762514	0.418503749	2.221459768	2.096342371	0.503133911
1.409227985	1.414143199	2.503214544	1.385558763	0.135803493
2.834061678	1.84741723	1.84927855	1.45211742	0
1.674076197	1.685966284	1.674311971	1.625220694	0.239473085
2.38981273	3.87363313	2.798963567	1.827611604	0
2.244292734	2.50323197	2.943614952	1.229712924	0
3.961779743	6.186614841	4.054999222	1.628112146	0.011521389
0.180669216	2.777100105	2.303966808	0.145629025	0.220988672
2.236742077	3.262368452	3.580497363	1.178880646	0.058873767
1.715500755	2.490327026	2.850985036	0	0
1.426127116	2.088777055	2.522988649	1.401084468	0.142017366
2.478325094	4.697892702	3.867683141	2.466683085	5.770541251
1.214967743	1.842443098	3.291639559	2.57933584	10.73165707
2.314122812	1.848002331	1.553768095	0.680138899	3.767616586
2.219965462	2.930756173	0.748827564	1.677386527	0
2.411748081	3.961565351	2.196815472	0	0
2.433672797	2.443407919	2.433854456	1.331282593	0.114580122
1.658646469	2.080393122	2.07913508	2.39816722	1.06112052
0.044372416	2.213208774	1.165331337	1.768889059	0.051631795
1.693103508	0.025343423	1.693131389	1.086950262	0.02780329
2.372820542	2.112269892	1.802057936	2.386493414	0
0.006736157	1.025743771	2.033070624	1.024652224	1.025384769
1.436440057	0.129844442	1.43655483	1.410543562	6.956174749
2.274624663	1.612478406	1.602996048	1.561490029	0.210208261
1.512044796	2.187516767	2.174955085	1.479525528	7.079857975
1.888014476	2.463151296	2.205193166	1.486255834	0
1.831576638	0.265756611	1.831918366	0.194114122	0.308009167
3.297568552	1.378888594	2.462528651	0.085658243	0.123247404
3.35986032	3.094107795	2.780414012	1.811747229	9.396559025
1.6789507	1.69100164	0.196541185	0.157452952	0.241513192
3.248764072	1.983391842	3.021477204	0.076491239	0.10918609
0.744866062	0.744480809	0.744850149	0.750960895	0
2.837820458	4.530053049	3.407774153	1.38752156	0
1.528217888	2.657672832	1.528374048	1.494198991	0.180686271
1.714942398	1.109849647	1.714976745	1.104583471	1.108465582
1.706081238	2.263845723	1.315032557	0.786360428	0

3.286712469	3.497905411	1.365917063	1.345541366	0.120081669
2.592727464	2.98487532	1.493121813	0	0.638046457
3.619620532	2.031602405	2.61805466	1.023203526	0.00727603
1.062531522	2.895791103	3.410435216	0.014071859	0.019051552
2.247396615	1.61777166	1.861778559	1.651693094	0
2.703946743	1.939093402	2.370356391	1.281861709	11.39576078
2.031015321	3.494751232	3.033831867	2.838224214	4.497174618
2.241190494	3.472118493	2.869068882	1.13516949	0
2.936922964	1.656967228	1.273602273	1.676288853	0
0.000355307	2.323785979	0.000355611	1.00130657	0.000407003
1.519574545	3.494082974	0.146989135	1.486360721	0.17733571
2.450752885	1.87282335	0.904471231	0.907473918	0
0.189518775	2.353105204	1.657751385	0.152339421	0.232583535
3.253773803	3.112851903	3.253688713	1.423974238	0
2.231241015	3.898650746	2.904417348	3.495297758	5.978831936
3.364860772	4.729842098	4.369154663	2.758986544	0.043715213
1.152222047	0.04303159	2.193187493	0.034416222	0.047394774
1.73432763	1.748293305	0.214264352	1.678660722	0.26504646
3.049549417	2.538054269	2.324950826	2.083523769	0
0	2.887125669	1.356756615	1.764584483	0.816614428
1.728707587	2.484667276	2.481484186	2.734990382	3.34798266
0.162095228	1.578255318	0.162287768	2.18391811	0.196899937
1.837247865	4.083887305	2.64641934	2.111900221	0
3.569770216	2.231159686	1.179531855	1.784265671	0.056261012
2.962590408	0.154830135	2.176286487	2.128750655	0.174891778
2.297056082	1.562537261	2.297047843	0.982240227	0
3.202213834	3.194146248	3.202066168	0.757871118	0
1.670038599	4.011797012	2.905648467	1.665294498	0.021731381
2.153171862	1.966612019	0.478824223	1.56813254	0
3.284417825	4.968885352	2.518279224	1.749222988	0
1.381838871	2.481713004	3.30667634	0.087334089	0.125842085
2.46608412	2.901576834	3.528120988	2.663892641	0
1.917030755	0.29763683	3.071676816	3.562230324	0.347562008
2.00191085	1.001482251	2.323915034	2.323779157	0.00044289
2.891218195	0.236732141	3.234379599	3.137384171	0.272512327
1.591010621	2.277627938	0.168933453	0.136518707	1.599863351
2.407291215	1.832000398	1.834013088	1.438650916	0
1.048913016	2.653180849	2.651809415	0.010999302	5.750146438
1.216815268	4.419021229	3.339950133	2.744569214	0.708321227
2.640467849	3.371600072	2.332445318	1.432635396	0
1.408781325	2.119502178	1.408749785	0	0
0.078791938	1.928671542	1.28838726	1.273553059	7.286438068
0.970187903	1.548608693	1.961191641	1.963521673	0
2.416494354	1.071480921	2.416528717	0.015997701	1.670904777
3.066125175	3.371605544	2.31113193	2.693524125	0
2.077173729	3.312987952	2.908259878	1.777842606	0
1.985625226	2.983933938	2.569653206	2.570762459	0
1.954956344	2.41618649	0.740586023	1.666161832	2.908869039
1.812329144	3.046049483	2.498061757	2.67701753	0.509958813
3.299100552	2.393272822	2.919267012	0.398894935	0
3.964046228	2.863620518	1.039629533	1.038114914	0.012006186
0.522021619	3.179073847	2.233976716	0.529108868	0.983755878
1.439012253	1.839936976	2.41570009	0	0
4.354305853	5.251095749	2.427049978	3.018447267	0
0.649516406	2.267291225	1.816208827	0.656452981	6.814147412
1.64505584	2.654930127	3.656721082	2.384198244	0.015242426
2.920016489	2.106496388	0.021708514	0.018423476	6.237969245
2.693090122	1.720398305	3.145492883	2.048821156	0

0.235450927	2.514127301	3.28849283	1.736842341	0.294018817
0.6897105	1.454628612	1.244535865	1.863277979	0
2.055971076	1.349291335	2.055899351	2.328642726	0
1.876270259	3.217731817	2.904925491	1.200776552	0
2.123164778	2.942832736	3.595382137	2.114933866	0.029596326
2.412325077	1.335957817	3.245579317	1.946035288	2.768791011
1.483212335	1.772447126	1.483128106	1.129385988	2.221005965
1.665330279	2.028172352	2.335615572	1.466550527	0
1.020038123	0.005532848	1.609035348	2.024268947	0.006043232
2.215097537	6.270955408	4.698911932	3.535471594	0.053140771
2.282822581	4.3221941	1.850314337	2.261935316	0.071328091
1.7460006	2.175397668	2.767334813	0.030617199	9.899483939
0.080340764	3.404173217	4.862624302	1.905761676	0.094704881
2.040743183	2.041378373	1.031591452	1.030397094	1.623238509
2.118714443	1.093412665	2.935009669	1.089036769	0.028488872
1.888422396	2.663418778	2.32323595	0.058688256	0.082501034
2.533592153	1.579662286	1.583272585	2.729175461	0
3.209252336	3.214710535	2.789574284	0.034482465	2.530226101
2.744781353	2.990009282	0	0.641597039	0
2.672129103	1.855297632	2.819910457	1.579519432	1.558178182
2.608706308	2.615158812	2.277536008	1.210131129	0.069853688
2.832692233	3.863363	3.360789963	0	6.537299886
1.744220411	3.360704558	1.134041465	1.734780087	0.041543048
2.506563255	3.003174828	3.655548931	1.74165051	0.043555482
2.940578899	2.958252392	3.442822962	0.113696817	0.167667339
1.666440763	1.667438889	1.068159524	0.015340676	0.020791289
3.614938159	2.91489044	1.514589596	0.943798039	0.942516849
2.991897249	1.681948898	2.625575058	0.575540844	0
1.589755781	0.001097096	2.005161545	1.589506567	6.52997897
3.650924738	1.900055	2.573938436	0.205887233	0.33032529
0.177294918	3.615174018	1.618761499	2.231365919	1.628754395
4.414405425	5.238355482	2.626362952	2.461871516	0
2.070956341	1.651825122	2.39560332	1.053048181	0.016786586
2.028049469	4.111502845	2.469800832	3.031786869	0.125469287
1.858721373	1.180507376	1.558999384	1.196129183	0.682303266
3.090995884	2.410425654	1.065194266	1.062616471	0.019872619
1.819592334	2.255213763	0.05361799	1.189372972	0.062535877
0.643927362	1.507192911	2.26532863	1.531046412	0
1.79469048	1.792268664	2.10745236	0	0
2.284084396	2.904598188	2.283941429	1.547030627	0
0.837952193	1.385136072	1.38666865	2.11033053	0
1.987626075	2.012497118	1.988094078	2.57196735	0.381571545
3.024529035	2.442349747	0	0.903456892	8.339147311
3.215376621	2.391949839	2.717941204	1.292338796	0.099815431
1.529453653	2.258532755	3.25532934	2.263659381	0
2.684425385	2.52829036	0.600616409	1.097727944	0
3.688287202	1.272553647	3.16701249	1.880846905	0.086541987
2.293625449	2.299466269	2.625479276	2.27180032	1.233528604
3.104401724	3.129278676	2.311665153	0.14730824	2.34363206
1.987988137	2.773939767	1.988128667	0.078855338	9.082303382
2.062481798	2.314144345	2.725033672	3.1996769	0
2.425378272	4.16394933	3.239674688	1.260374445	0
2.457040198	2.024028751	2.016186992	1.984864132	0.12163608
2.348037016	1.681916863	1.670388675	0.155352738	0.237835714
2.774546858	2.786067814	0.098075513	1.335575291	0.116230934
2.422921877	0.09983252	1.983568159	1.322891771	0.111366824
1.767947229	3.922695253	3.548622451	2.008353065	0.922657672
2.837430218	3.72444359	2.058040065	2.458916671	0.135093767

hsa-miR-937-3p	hsa-miR-520h	hsa-miR-526b-5p	hsa-miR-519c-3p	hsa-miR-216a-3p
0.131030296	0.133943668	0.140006058	0.134514506	1.414790665
3.037737869	0.007622111	1.025102589	0.007647025	4.362137827
2.337518541	0	0.388683365	0	1.926500572
3.777186714	1.81106699	5.351883923	2.123286828	5.811428606
3.256412258	0.01751925	1.057318454	0.017577875	5.820870444
2.981502434	0.036968185	1.119432511	0.037097535	1.731951559
3.810141748	1.773041056	5.397998176	2.480566445	0.272313509
3.15808404	0	0	0	0
2.234134718	0	0.770199754	0	7.013581565
4.305133308	0	2.398166036	1.631759561	0
2.078651102	0.133723016	0.13977284	0.134292681	0.136818163
1.356691519	0.112695983	1.347929679	0.113157396	2.006402144
3.623883155	0	1.93462012	0	0.950917925
4.0649377	0.077884883	4.178438854	1.885308437	0.079498228
6.358827014	0	0	0	0
2.368406485	4.226297549	6.547335684	4.42166918	2.368508889
3.595778623	4.703107134	6.930861401	3.318351205	0
3.909261769	0	1.720919658	0	0
1.469424241	0.151571774	1.458120118	0.152238819	0.155198445
2.532082078	0	0.964057465	0	4.748399089
2.290746838	0.484729707	0.522250215	0.488154608	0.503603219
0.129926989	0.132809806	0.138807714	0.133374624	0.135878515
2.827783303	0	0.888586374	0	0
0.227235251	0.23321132	0.245815267	0.234388335	2.388276828
3.10931631	0	0	0.863293194	0
4.519720832	0.451330888	1.905969285	0	0.880658097
8.621726724	0.011324294	2.049699366	0.011361639	0.011526302
3.656623727	0.215371813	0.226668134	0.216428316	0.221129975
2.244492517	0.057755687	0.059988408	0.057967198	1.186796719
4.111909833	0	0	0	6.996387939
2.545822608	0.138851726	1.42254079	0.139448855	5.633481046
3.877410767	7.451211663	11.04702686	7.384524206	7.786674596
1.84466963	10.45506684	13.30440984	9.52902656	2.884821612
0.675080217	3.362353234	4.193931301	2.301267411	4.391074574
3.181700327	0	1.661679337	0	2.424565901
2.751268386	0	3.805060221	1.636216457	5.299102691
3.491774199	0.112150702	0.117013599	0.112609401	0.114640946
2.081253292	1.061564132	3.794321932	0.018496635	6.287753085
1.785130679	0.050666114	0.052593923	0.05084884	0.051655906
2.119243229	0.027309634	1.088779067	0.027403119	4.6001464
2.940863628	0	0	0	0.852725868
4.039268322	0.007602572	0.007863882	0.007627421	7.610997813
2.107783255	6.370730247	9.715334034	6.732509363	7.381055297
0.199928709	0.204955061	1.602855152	0.205943548	8.343305521
2.196973303	4.312371992	9.712759103	7.407667029	3.514141632
3.674252337	0	0	0	8.657075218
1.860595155	0.299120661	0.317068905	0.300787196	0.308233683
3.698351589	0.120591853	0.125909388	0.121093096	0.123313915
4.081171669	7.037217663	11.38982731	7.965575179	6.804641937
1.697492138	0.235178596	0.247930333	0.236369212	0.241672702
5.135822604	0.1068944	3.805562904	0.107327175	0.109243452
1.267679425	0	0.751865644	0	1.270317741
4.193155067	0	0	0	3.12087392
1.539037477	0.176381949	0.185022861	0.177192749	0.180794355
1.717485252	1.109250039	2.470495113	1.109133468	0.033640309
2.977354697	0	0	0	9.305741795

3.084261602	0.117509402	2.01789749	0.117994979	4.711193564
1.780401809	0	0	1.124374752	0.638122906
4.285107511	0.007152791	0.007398368	0.007176145	6.305412283
3.414905556	1.062504755	4.00298985	0.018782744	8.509118346
3.492900054	0.534673335	0	0	0
2.384226649	10.1653056	13.67925246	11.02897886	0.095956401
4.895380083	3.621538562	6.168009311	3.495840779	6.530378301
2.019827362	0.633287848	0	0	2.968785619
1.271701094	0.750159805	1.277626437	0	6.361949294
0.000393616	0.000400221	0.000413732	0.000401506	2.323809755
2.661631089	0.173134729	0.181566924	0.173926157	0.177441183
2.856597751	0	2.44734948	0	1.872405282
2.831009861	0.226565295	0.238675246	0.227696819	0.232735016
6.11473562	0.862831724	0.865563019	0	4.902189582
3.597449854	5.958466998	8.522759377	5.447077977	4.799491419
6.405332352	0.042911359	1.138110762	0.043063505	0.043735277
1.769408424	0.046516323	1.149373821	0.046682567	7.227081462
4.464759733	0.257847964	0.272354482	0.259199785	1.751176772
4.34801074	0	1.757323327	0.819803941	6.951126502
2.527586276	0	0.818921588	0	5.148434132
3.880910767	3.347464856	7.651774726	4.090031749	0.037348514
2.723152899	0.192083009	2.256754427	0.192989839	7.297080063
3.248534891	0	0.672514551	0	5.693717759
5.225487338	0.055198395	0.05732013	0.055399432	4.271532688
4.633945182	0.170765615	0.179046733	0.171543017	2.197516137
3.294161285	0	0.982023114	0.98166592	0
5.83073307	0	0	0	1.279111404
3.565748125	0.021350787	0.022109635	1.071091429	7.939854608
2.98773954	0	0.49363626	0	5.789780027
4.262116392	0	0	0	0
3.863307953	0.123117682	0.128573703	0.123631874	5.153012771
2.4515297	0	2.272348087	0	2.451211444
1.953199236	0.336988606	1.934451761	0.338968107	0.347829586
3.172124404	0.000435509	1.001441292	0.000436908	5.757255875
3.296145637	0.265030619	0.280113091	0.266435239	2.480517565
3.096211077	1.60356582	3.996095908	2.291188546	4.754173967
2.620451452	0	2.146094001	0.875186485	6.835607979
3.532778333	6.121508133	9.919899229	6.452386232	7.111575621
7.08582505	1.216109562	0.711798404	0	0.708384767
2.872613905	0	0	0	0.583968897
3.234743925	0	0	0	0.857667949
1.932205522	5.681251831	8.424031906	4.1237401	6.417335726
4.345677861	0	0	0	0
0.020944385	0.021313709	2.09257778	1.070969662	0.021703081
4.685888476	0	1.553143586	0	0.67712659
1.367495943	0	0	0	4.464537971
3.790341677	0	0	0	3.683319798
2.762422781	2.197812607	4.64681935	1.263418156	7.580760229
1.801321421	0	2.727760598	0	0
5.820664943	0.400064157	2.127071554	0	3.046385547
5.869234082	0.011800565	0.012210381	1.039536016	0.012011311
2.375948162	0.527601065	0	0	0.530755523
0	0	0	0.88097069	4.329319039
5.141299433	0	0	0	0
3.497809194	5.812554309	8.859073903	7.145232446	2.053866808
2.066268741	0.014979416	0.015503681	0.015029244	1.645700113
2.700441578	3.443802175	7.5320123	5.256897135	0.025044589
4.828585433	0	0	0	2.494834344

0.277792349	0.285696662	0.302490247	0.287257823	0.294228889
2.526393597	0	0	0	1.643631431
2.520398194	0	1.745086713	0	8.328574481
5.067605816	0.43358306	1.454022409	0	0
3.602725052	0.029068723	3.465260225	0.02916863	2.946296289
4.483624568	0.105952506	3.271904567	0.106380683	0.108276514
3.532626088	1.115212472	3.364068242	0.629290849	7.266751159
2.950200232	0.843153813	1.442661239	0	0
1.609414864	0.00594117	1.019588752	0.005960511	3.352179689
2.553181166	0.052143678	0.05413432	0.052332339	1.169222164
0.068592482	0.069938191	0.072715173	0.070201011	0.071362824
3.195815601	9.243678422	10.95630362	7.710715358	7.759940617
2.379837784	0.092771552	0.096638131	0.093136837	1.93723948
1.623482433	1.031543531	3.507251218	0.009420757	5.37215068
4.310984317	0.027982266	0.028992644	0.028078201	6.123830036
2.669993017	0.080856807	1.891401935	0.0811676	1.894901377
2.964271166	0	2.338654719	0	1.878271259
5.08944466	2.530346871	4.231808889	0.046774884	5.965725635
1.126246593	0	0	0	1.13026001
3.980007031	0.681189973	3.911931719	0	7.107263028
1.222532196	0.068496609	0.071207846	0.068753236	0.069887598
4.899869397	6.423732538	8.149950479	5.59101379	0
2.77288486	0.040782726	3.194253949	0.040926644	2.17532801
2.183728227	0.042754842	2.78107326	0.042906381	1.754110411
3.857472906	0.163759583	0.171599843	0.164496025	0.167765407
1.068407526	0.020427938	0.021152379	0.020496762	2.089637722
3.370493622	0	0	0.942163429	0
4.048418844	0	0.580715568	0	1.044639794
1.589829499	5.734253875	8.992344638	5.983632908	8.166304171
0.311200848	0.320501362	0.340359444	0.322341744	1.908634832
1.633709406	1.632568279	1.619127325	1.632094918	7.380405343
6.760647458	0	0.7578724	0	4.043931615
2.398044983	1.05515824	1.651006817	0.016550967	1.054747622
3.103897443	0.12275481	0.128190866	1.384793902	0.125537281
1.180965405	1.55476819	2.656458169	1.182494044	8.032224159
3.095200736	1.065162418	1.063799709	0.019591698	1.663809733
3.088256982	0.061339148	0.063729293	0.061565509	1.197958198
1.50606008	0	0	0	5.303418027
3.217867764	0	0	0	0
2.061821352	0	0	0	2.616104826
2.091728041	0	2.094058057	0.839189927	6.635284915
3.586313682	0.369450369	0.394012541	0.37171671	9.536187792
2.181665294	6.935213255	9.4828154	7.848016092	7.201716534
3.608757126	0.09775753	0.10187415	0.098146283	5.27241722
3.62966989	0.954248467	0	0	0.95458906
2.797303168	0	0	0	0
3.185818373	0.08480334	1.266054847	0.085131934	3.855129305
3.506088179	0.072841119	4.257136887	0.073116511	3.509460385
3.866078077	2.808835763	5.884576757	3.158890317	4.356369562
1.349908402	8.882722484	13.40075671	9.009652706	5.376266793
2.056550192	0.815192596	0.81881528	0	2.714893522
4.143910015	0	1.94178161	0	0.473763291
2.815649284	0.119023019	2.472501714	0.11951627	0.121701523
2.846440713	0.231632166	0.244117996	0.232798307	1.682995381
3.070105934	1.358722431	0.118707448	0.114225621	0.116292829
2.439830332	0.109019729	0.113717186	0.109462934	7.858055205
3.070974154	0	0.490836792	0.482799618	0.48564221
5.802952697	1.412407593	4.046346863	1.412028616	7.831458043

hsa-miR-520a-5p	hsa-miR-541-3p	hsa-miR-5003-3p	hsa-miR-518b	hsa-miR-518f-5p
0.137780495	2.078342304	0.09051646	0.138654347	0.137593607
0.007788588	2.62137959	1.613730512	0.007826184	0.007780533
0	4.011601141	2.658891269	0	0
4.094528143	0	0.86506812	4.577176221	1.413617735
0.017911156	0.01697017	2.069235205	0.017999715	0.017892182
0.037833639	2.159484429	1.115264253	0.038029457	0.037791698
1.766771475	0.251805349	1.67995639	3.589894041	0.273676932
0	1.909905108	1.419256591	0	0
0	0	0	0	0
1.253202165	4.253783232	1.965316158	1.935373027	0
0.137551918	0.128439596	0.090378983	0.138423952	0.137365418
0.115794166	3.063489916	0.07711533	0.116498749	0.115643428
0	1.52411982	0	0.951206594	0
0.079879505	1.885230791	2.283419037	2.658416016	3.160087655
0	0.968085275	0.969595812	0	0
5.989432041	1.288132898	2.651288632	5.521394993	4.675042186
4.351318407	1.214240614	1.233020892	6.196577022	2.69882695
0	0	1.738601423	0.79596141	0
0.156059082	5.829188358	2.067762646	0.157082347	0.1558403
0	0.96307245	0.964804942	0	0
0.508174956	0.453955637	0.273110363	0.513658459	0.507009315
0.136605965	1.415836664	0.089809636	1.408928241	0.136421068
0.887420484	5.516986175	1.453769373	0	0
0.241161401	0.222389613	0.149035236	0.242984958	0.240772028
0	5.170371706	0.867564485	0	0
1.210994927	1.203109683	2.140628935	1.212802816	0.455619491
1.037616317	0.010974349	1.036188474	1.037483586	1.630969925
0.222501509	0.205642751	2.668633306	0.224134765	0.222152666
0.059172225	0.055781285	0.040857816	1.185640585	0.059103504
0.79120555	0	1.336846339	0	0
1.427760423	0.133316031	2.03327704	0.143780853	0.142670628
9.010729079	0.034736908	2.953035305	10.09941056	8.269352912
11.56169377	0.064330569	0.046892616	12.68102976	11.57934784
3.36110089	0.67424128	0.682374799	4.139775408	1.551989881
0.753329272	0	1.681476106	0	0
1.257163427	4.313480083	0	3.371601418	1.256992534
0.11523058	0.107888548	0.076767024	0.115930975	0.115080737
0.018848102	1.659930151	1.058506929	2.080764786	0.018828092
0.051889473	0.048959397	0.036000457	0.052166521	0.051830146
0.027934849	2.119025662	0.019691374	0.02807622	0.027904565
0	0.850627643	0.856286403	0	0
1.025315563	1.616201693	2.353333151	0.007806112	0.00776058
7.130619789	0.136841834	0.095859081	8.459386821	7.224366994
0.211622284	0.195843975	0.133024251	0.213148446	0.211296259
6.950165811	0.163153786	2.116982604	7.584477851	7.502110487
0	1.480100889	2.214363138	0	0
0.310415251	1.859060057	1.748972525	0.313018671	0.309859978
0.123958745	2.033843387	1.982140437	0.124724857	0.123794865
9.930782569	0	0.855659352	9.959492202	9.772305657
0.243221345	0.224233748	0.150132007	0.245066424	0.242827389
1.335113669	1.339765531	0.073397999	1.333861416	0.109658192
0	1.954468283	1.284666405	0	0
0	2.263430914	0	0	0
0.181842951	0.168889888	2.132519318	1.530873536	0.181576334
1.108165881	1.109868015	2.127642751	1.716090774	1.108239471
0	0	0.788100905	0	0

0.120770581	0.112999577	2.406972071	0.121512479	0.120611872
0	1.121899449	2.069978907	0	0
0.007308839	1.024210522	3.030598128	0.007344077	0.007301288
1.061884116	1.062874208	2.397939827	2.081989834	1.061925861
0	0.531388599	1.007748443	0	0
12.0274267	5.435880289	0.065008125	11.74988842	11.94913263
5.323699057	0.006909893	1.022871964	5.208228012	3.737347459
1.124510877	6.594627253	0.638832798	0.637736669	0
0	0.748411975	0	0.753327523	0
0.000408808	0.000388164	0.000293596	0.000410746	0.000408393
0.178464407	1.530049177	1.478331316	0.179681619	0.178204248
0	2.187796145	1.47569553	0.906204098	0.905824154
0.234205551	1.674344704	0.145312971	0.235957191	0.233831492
0	0	1.829645557	0	0
6.44548276	0.933306498	1.509225882	7.029981207	4.846614128
0.04392962	1.755555919	1.133044655	0.044160101	0.043880259
0.047629123	1.769286734	2.176479496	0.047881063	0.047575169
0.266990199	2.454674039	1.665904028	0.269091013	0.26654181
0	0	0.82479686	0	1.362418787
0	0	0.820554618	2.312014035	0
5.046124203	3.17405919	4.238908368	5.997756084	3.766821432
0.19819555	0.183715107	1.522478529	0.199593447	0.197896864
0	1.16244425	1.182268691	0	0
0.056544626	1.181069539	1.169179702	0.056849592	0.056479325
0.176000222	0.163578642	0.11297997	0.177195519	0.175744737
0	1.975550836	0	0	0
0	1.276232092	1.685542059	0	0.756135435
1.070461498	3.565128356	0.015453201	0.02194172	0.021809438
0	1.962119959	1.290435928	0	0
0.805705768	4.998331824	0	0	0
0.126571973	0.118344388	0.083729801	0.127358109	0.126403816
0	0	0	0	0.6598738
0.350430872	0.31895756	1.821043332	0.353538262	0.349768544
0.000444854	0.000422387	1.586622	0.000446963	0.000444402
0.274533412	0.252152814	0.166498245	0.276718242	0.274067147
1.598207637	0.191527417	2.192206952	3.383082252	1.598622978
0	2.144666872	0	0.876684903	0
8.11186049	2.06450357	2.057934534	7.111992832	7.632946271
0.709043848	7.067364425	0.712205826	1.219893289	0.70886845
0	4.57766078	1.43853147	0.586058512	0.584617773
0	2.117839195	1.82163161	0	0
7.18375554	1.291812023	0.063042076	7.455212904	6.221831937
0	0	2.28487104	0.970678229	0
1.070340812	4.355724208	1.067456276	1.670485121	1.07038834
0	4.689441996	1.193162519	0	0
0	2.899493215	1.38083041	0	0
0	4.737665098	0	0.989100648	0.989055321
4.317353459	0	1.279408829	4.688266834	1.949201697
1.297828844	0	1.612725522	1.29945652	0.956772943
1.128867071	2.709796529	1.426129201	0.406390157	0.404586837
0.012060939	1.039817563	3.957710093	0.012119754	0.012048337
0	0	0	0	0
0	5.299467795	0	0	0
0	10.18125351	1.456506236	0.89008931	0
7.816192856	2.053320299	2.312224865	7.111186629	6.682461953
0.015312473	0.014512636	1.04769955	0.015387722	0.015296351
4.032308879	3.442460603	0.017762351	6.774240641	3.932319078
0	1.719062421	0.800301234	0	0

0.296269751	2.998450634	0.177533886	0.298704406	0.295750353
0	2.210689429	2.171859304	0	0
0	0.809440714	2.332686597	0.813183633	0
0.438244309	0.848235111	1.926194372	2.330859236	0.437957765
0.029736946	0.028133885	0.020936408	2.125983698	0.029704576
0.108826596	0.101971851	1.939670776	1.981429956	1.332568006
2.563691871	0	1.509238875	2.840711665	2.01433376
0	1.850550053	0.429066634	1.174210046	0.431451614
0.0060704	1.609414278	1.019085705	0.006099581	0.006064148
0.053406852	0.050381755	1.160273543	0.053692944	0.053345589
0.071699356	8.357470609	0.049106341	0.072098712	0.071613862
9.915890982	0.039896241	1.128120261	10.10476111	7.132182786
0.095221955	4.332344269	1.27472765	0.095778461	0.095102861
1.031230488	0.009101054	0.006848448	2.365498049	0.009585942
0.028623887	0.02708452	2.108939205	0.028768975	0.028592808
0.082940497	0.077961272	0.056395807	0.083413336	0.082839289
0	1.877013077	2.381280134	0	1.206405627
2.799035742	0.045051597	3.996642992	3.686642318	1.151490861
0	2.028906158	0	0	0
1.186616482	0	2.141480387	2.10120831	0.683974889
0.070216157	1.22283123	1.207368311	0.070606038	0.07013269
6.331382672	0.610452744	1.112934309	7.547333953	4.355608
2.175095217	1.135015737	0.02915731	1.745827356	1.746365468
0.04376903	0.041338496	2.163048853	0.043998587	0.043719866
0.168716686	5.609290798	0.108777722	0.169848054	0.168474838
0.020888087	2.681025481	1.064708054	0.020992087	0.020865806
0	2.240740557	1.518895169	0	0
0	1.038893121	1.968340618	0	0
7.643072624	2.005279759	1.003799156	7.381503805	5.787764606
0.332988082	0.303712433	2.455247551	0.335870951	0.332373433
0.218059918	0.20164538	1.565507544	0.219649109	0.217720463
0	0.751418271	2.824341348	0	0.755346897
0.01686401	1.652168879	2.653284451	1.054417017	0.016846189
0.126196502	0.117998695	0.083500841	2.038567093	0.126028961
1.185209571	0.677833455	0.685956128	0	2.308247172
0.019964942	1.664285834	0.014148947	1.064286128	0.019943691
0.062855392	1.200438345	0.043296898	0.063199012	0.062781821
0.650807245	0	1.838210177	0	0
0	0.845183228	0	0	0
0.661741885	2.062444791	1.854982888	1.15855429	0.661542578
0	1.781076371	1.397407992	0.841103401	0
0.384867042	0.348867583	0.219978508	0.388439785	0.384105963
8.453230981	1.462391576	1.878063061	8.273157181	6.210607398
0.100365975	0.094140864	2.346870913	0.100958575	0.100239166
0	2.519108987	1.532884484	0	0
0	2.667287749	1.474051136	0	0
0.087006788	0.081742977	0.059007003	1.907532507	0.086899741
1.865477617	0.070273664	2.595453999	2.3027402	1.865617892
3.160447082	0.208264235	0.140568021	4.358456153	3.44246366
9.928808899	5.750876527	1.952739469	11.15995015	10.56964969
0	1.747777307	1.368614035	0	0
0.474803747	1.233502983	1.550618349	0	0.907484519
0.122335978	0.114442517	0.081140591	0.123089735	0.122174736
0.239508177	0.220908925	1.610458857	0.241314536	0.239122466
0.116892861	3.296982444	1.962485988	0.117605633	0.116740374
0.11199513	0.104900439	0.074762739	0.112671619	0.111850395
1.259678097	4.7405674	0.485477087	0	0.486396125
2.522826278	0.126914233	2.449670426	2.069843755	0.135707308

hsa-miR-520g-3p	hsa-miR-520e-3p	hsa-miR-548d-3p	hsa-miR-516b-5p	hsa-miR-5010-3p
0.137061947	0.131281698	0.089096881	0.13800526	3.117262023
0.007757586	0.007505246	0.005492437	0.00779827	2.84320517
0	0	0.38118729	0	1.572329992
2.597823473	3.105141413	1.827692338	3.943532459	3.113347096
0.017838142	0.017244384	0.012541526	0.017933959	1.655161721
0.037672265	0.03636226	0.026128941	0.037884051	2.483105563
1.768618102	0.258162503	1.675120528	2.477836689	1.752447095
0	0	0.57404277	0	2.469789176
0	0	1.702964798	0	2.458050015
0.735051802	0.732002634	1.967192001	1.253420054	2.406002265
0.136834862	0.131066488	0.088961926	0.137776215	3.344833058
0.115214514	0.110542118	0.075936452	0.115975429	2.436672607
0	0	0.952699201	0	2.254353919
0.07950691	0.076493663	2.281442722	1.248044968	2.914723898
0	0	2.959259715	0	3.275828649
4.595856436	2.368495957	1.265029658	3.736053662	1.285092995
5.131690339	3.896180185	0	4.811039461	2.361967775
0	0	3.173925233	0.795457007	2.690075623
0.155218027	0.14846378	2.83260521	0.156322232	2.565250894
0	0.96316088	0.964943798	0	2.271916294
0.503706837	0.469037654	0.267450638	0.509580131	4.24959604
0.13589507	0.130175765	0.08840301	0.136828331	2.061138616
0	0	2.658510667	0	0
0.239665542	0.227748909	0.146446314	0.241629979	3.150039835
0	0	0.868030958	0	2.608380974
0.880685481	0	1.520653545	1.211425243	2.085714091
0.011527386	1.038139071	1.036034272	0.011588388	3.513929972
0.22116116	0.210464384	1.571111291	0.222921263	2.757813887
0.058907853	0.056765825	2.214357033	0.059254835	2.567452209
0	0	0.795400107	0	1.715118371
0.142114231	0.136067801	3.065420776	0.143101464	2.080162655
8.326362693	6.826214303	1.111559155	9.489024655	2.15123933
11.21565928	9.004040809	1.201112025	12.14455776	2.87100101
3.902792278	0.675185394	1.196835549	3.767365672	3.18130855
0	0.749920162	0	0	2.434627222
0	0	0.742891366	1.638282998	1.25538399
0.114654367	0.110009452	0.075594263	0.115410767	0.097265094
2.406841049	0.018144973	1.058251811	1.060901192	3.086279371
0.051661226	0.049810684	1.155199309	0.051960789	2.806743087
0.027818319	0.026871522	1.085551609	1.089706956	1.69327551
0	0	1.413346365	0	1.801812448
1.025377411	0.007486012	1.024280697	0.007778271	0.006772727
6.938021883	5.991863594	1.401753884	7.240620081	2.536278376
0.210369502	0.200361227	0.130774016	0.212014555	3.068645366
5.753145612	6.491764585	3.377959039	6.431808391	1.512959755
0	0	0	0	2.876651237
0.308283239	0.291411719	0.18118856	0.311083764	0.247778793
0.123328592	0.118252938	1.979437576	0.124155823	2.022064937
8.093309672	8.193035079	2.130832934	8.869703043	2.590056564
0.241707906	0.229653598	3.312964096	0.243695443	1.680426676
1.986453953	0.10487366	0.072284054	2.431812197	2.416652286
0	0	0.753642953	0	1.655435654
0	0	0.556915605	0	2.277331433
0.180818208	0.172608658	1.482944333	0.182163687	2.194174533
1.716703674	0.03248388	2.126822439	1.108073332	1.109237076
0	0	0.788775759	0	2.013820715

0.120160299	0.115243258	1.338512143	0.120961435	2.01207642
0	0	0	0	2.244171067
0.007279779	0.007043243	2.351302625	0.007317913	2.354327697
1.660606688	1.062741261	0.013390421	2.082131963	3.258156698
0.537812248	0	0	0	2.803640194
10.89094176	10.36365388	2.662262266	11.1670283	2.37112612
4.497180353	2.031807589	3.615584251	4.359601969	4.122447478
0.635869169	0	1.140897226	0	1.120441902
0.751981061	0	1.289793395	0.75272956	2.218087279
0.000407209	0.000394189	1.001287373	0.000409307	3.586927857
0.177464459	0.169451009	2.120267826	0.178777368	1.520511776
0.905661619	0	1.47622528	1.872664043	1.87425277
0.232768448	0.221312285	1.59542882	0.234655679	1.658921454
0.863852051	0	1.830560593	0	2.131014379
6.430285578	5.319929757	1.509602912	6.414401604	0.933415523
0.043739704	0.042198838	2.162538246	0.043988953	4.785430671
0.047421541	0.045737905	0.032674693	0.047693978	2.193535518
0.265267972	0.25158192	0.159720293	0.267529884	2.875258729
0	0	0.825381963	0.821491746	3.721235673
0.81666516	0	1.369704631	0.817232162	2.062204924
4.090847383	3.63847591	1.113793589	3.640284762	2.154245569
0.197047696	0.187866021	0.123469034	0.1985549	0.163300506
0	0	1.565295737	0.669701467	2.2962701
0.056293404	0.054257441	1.779907556	0.056623125	3.709012037
0.175018221	0.167146771	0.111133993	1.516939989	1.514164895
0	0	0.982495704	0	1.562741578
0	0	1.294488853	0.756472743	1.665284062
0.021742965	0.021012856	1.663674011	0.021860828	1.071242433
0	0	1.578942815	0	2.31058135
0	0	1.753834672	0	2.873791502
0.125925387	0.12071863	1.35291017	0.126774197	1.382412136
0	0	0	0	2.910433124
1.94885117	1.953341153	1.814726354	0.351228473	2.615946614
0.000443114	0.000428944	3.586855751	0.000445397	2.809424556
1.769570974	0.258522162	0.163524262	1.767052623	2.438653079
3.102664765	0.195912972	0.128184805	2.288224969	3.055068166
0	0	0.879640835	0	2.984961795
6.721948451	5.599775312	1.0463009	5.885364633	3.529340948
0.708398892	0	1.928227236	0	2.362404137
0	0	0.587021806	0	1.941763404
0	0.856022166	0	0	2.598543392
6.370730766	4.817779519	0.06210478	7.250083728	1.288702482
0	0	0.971627567	0	0.970166651
0.021705172	0.020976393	0.015219685	0.021822819	2.091326471
0	0	2.703964229	0	2.310412641
0	0	0	0	1.765468178
0.989036059	0	2.308311404	0	1.571600667
3.042794906	2.198010257	1.280659833	1.949505175	1.954400331
0	0.504804195	1.615644254	0	0.949307521
0.810100733	0	1.662380719	0	1.808656716
0.012012442	0.011617856	0.008479776	0.012076084	2.639316975
0.530776298	0	1.359428638	0.532038566	3.192113343
0	0	0	0	2.15671061
0.889456968	0	1.457119479	0	1.448529729
6.108534246	6.171780738	1.166601487	6.173533031	1.518721803
0.01525043	0.014745771	1.640648598	0.015331849	4.160092903
4.650898596	5.170561556	0.017522683	5.652888472	2.430827742
0	0	1.344782774	0	2.286938953

0.294275256	0.278470296	0.174308285	0.296895016	0.237420947
0	0	0	0	2.345368858
0	0	0.816663784	0	2.884803783
0.856365104	0	1.725688859	0.85809318	2.595893546
1.095695831	0.028600547	0.020651868	1.095378327	2.940068071
1.33317394	0.103953153	1.308388405	1.982275217	3.769407157
2.013882	2.012438032	2.061999332	2.01474564	1.777220971
0.430674268	0.424804884	0.859966575	0.432098251	1.664411966
0.006046336	0.005850438	1.019005829	0.006077915	2.348882697
0.053171161	0.051260529	1.159501606	0.053480494	3.557765779
0.071370488	0.068708894	1.827567004	0.071802137	3.305535947
9.809262071	7.859011251	2.482790413	9.638095326	2.172697741
0.094763914	0.091064814	1.898195008	0.095365151	3.858947354
1.031306873	1.031664836	1.029936154	2.041599607	1.62291434
0.028504296	0.027532685	0.019894154	0.028661242	2.711079983
0.082551213	0.079403867	1.240781534	0.083062178	2.323883523
0	0	2.572100387	0.6997821	2.532700039
2.530207652	0.045828166	0.032737342	1.768288135	0.041103164
0	0	0	0	2.433060358
0.683470772	0	2.359529311	1.559190948	3.571061947
0.06989508	0.067296201	2.250591336	0.0703165	2.278072103
6.927531117	4.774691746	0	6.788536374	1.996285928
0.04156621	0.040108676	1.126108176	1.746177369	2.170799212
1.139548739	0.042045161	2.488258478	1.139082698	1.14043996
0.167787049	0.160330251	0.10701367	0.169007602	1.495286231
0.020802348	0.020105293	1.064424106	0.020914865	0.018137513
0	0	0.944571851	0.942717925	1.514514474
0	0	1.724521035	0	1.686882498
6.31019454	4.913129748	0.000851899	6.346282434	2.590464837
0.330628528	0.311997405	0.19195088	0.333728184	2.576785852
2.322332901	0.206341933	0.134235697	0.218468364	3.086738712
0	0	2.826550891	0	1.969970607
1.05474472	0.016237697	0.011816937	0.016885427	3.077417685
0.125552285	0.120364425	0.082208559	0.126397984	4.095988583
1.184456464	0	1.201403614	0.683316657	2.319203463
1.064674119	0.019218285	1.657118704	0.019990482	3.793835897
0.062572367	0.060279963	0.042678921	0.062943835	2.250987959
0	0	1.840552958	0	2.053524784
0	0	0	0	1.794388978
0	0	1.173739347	0	1.824362806
0	0.838349312	0	0.840702984	2.353631076
0.381946617	0.359004174	0.215727064	0.385783767	2.690731818
7.175241377	6.988500159	0	6.933014434	2.185043412
0.099878282	0.095941567	1.286648081	0.100518451	1.308897463
0.954591427	0	0	0	2.259455439
0	0	0	0	1.738680671
0.086595057	0.083267461	0.058136837	0.087135492	2.671292196
1.865973783	2.638156396	1.836756915	1.865297222	3.121624803
3.160616623	1.652501389	1.577211257	3.678103994	1.637730143
10.9564059	8.481372099	1.320074479	11.45619863	1.345877454
0.81655764	0	2.750157113	0	1.356425547
0.473785729	0.468224665	1.793068519	0.475146478	1.946235055
0.121715964	2.029432138	0.0798905	0.12252988	2.458249525
0.238026368	0.226219794	0.145583278	0.239972343	3.650383604
1.35616797	0.111580387	1.328803849	0.117076229	1.355569929
0.111438543	0.1069505	1.316443709	0.112169172	0.094624468
0	0	0	0	3.339789612
2.071961753	0.129502443	2.01167602	1.408130869	0.114009219

hsa-miR-7974	hsa-miR-5586-5p	hsa-miR-516a-5p	hsa-miR-362-5p	hsa-miR-4791
0.087245448	4.17456741	0.138851904	5.662653576	2.001788627
1.024203753	3.623916528	0.007834667	6.924150464	1.02381014
1.39543401	4.280998663	0	5.27096078	1.843592653
0	5.471181634	4.524931405	5.494499321	2.148927458
0.012319167	2.07732298	0.018019701	6.159791742	0.01172256
1.717498298	4.184036453	0.03807366	5.583819929	2.461967435
0.159475782	0.260330219	3.827226364	7.116330215	0.149372739
0	2.727064099	1.913820155	5.983689923	0.579419673
0	2.448273554	0	6.994061991	1.315063561
0.740860021	3.465883626	0	5.291685781	1.275196398
0.087113763	2.078904463	1.411128865	7.824664101	0.082212684
0.074397455	5.273048379	0.116657985	8.390918414	0.070308178
0	2.925984561	0	7.118063977	0.953661816
0.052586578	3.528275074	1.24710782	6.112732574	0.049814789
1.54939736	3.95050852	0	6.439885296	0
1.886366811	1.287521892	4.003959908	5.92827586	0.056899979
1.236264541	3.82581006	7.187847064	7.398400839	1.241571854
2.054244597	6.027438906	0	7.438115532	0
2.49533009	4.112415832	0.157313748	7.625960599	0.092053583
2.539006666	4.029996137	0.963826593	5.894202106	1.544567758
2.049374653	2.292839189	0.514905796	7.681240147	0.241319832
0.086568377	5.054900244	1.408522445	4.928325435	1.366396869
0.891136316	0.885978165	0	7.92784709	1.45759393
1.604117173	2.388626675	0.243397891	6.193377932	0.134243132
0	2.126977979	0	6.091814468	1.835585676
0.4563571	3.880351466	1.902744157	6.858395353	0
0.007997252	2.050313692	0.011642967	7.600606229	0.007613696
4.140525036	3.659578756	0.224504495	7.214151286	2.968457377
1.786645555	3.978207208	0.059565644	6.848369859	2.207141553
0	2.839206829	0	7.572691608	0.798887716
1.389470143	5.831812883	0.143987645	7.273981816	2.447828159
1.110865477	3.496807117	9.385389168	6.141412005	0.023692924
0.045335861	2.613427121	12.75691879	5.977362347	0.042980691
0	3.363611426	6.319158942	6.51072609	0
0	3.058086685	0	8.747031825	0
1.274134704	2.750783368	2.189362138	6.445650205	2.237040677
0.074063215	2.006126211	0.116089262	6.41634491	0.069994846
0.012954778	4.618647607	1.659069223	7.713755446	1.056935483
0.034832241	3.410481716	0.052229074	6.719351113	1.151372071
2.917731017	4.137697675	1.089379882	6.252692493	0.018137
0.857463825	4.401974149	0	6.737622637	0.859410179
0.005382882	2.033914559	0.007814573	6.56490367	1.023749595
0.092361114	2.559452436	6.905046613	7.467036157	0.087115842
0.127848142	3.392155184	0.213493872	9.194162008	0.120137493
4.816423987	4.039245547	7.2721538	6.809006644	0.102138775
0.918899116	3.673934074	0	6.009006636	3.476570605
1.736121712	3.680797435	1.849586057	5.578466316	0.165309254
0.079211777	3.327331553	0.124898019	5.694233576	2.397825129
1.413655805	3.349336911	9.517265317	7.834624596	0
1.608251812	3.488871276	0.245484244	6.877911708	0.135206808
2.698766935	1.988043764	3.277083972	7.142799901	1.302182116
0	2.418070996	0	4.957078607	2.6605701
0.558298139	4.856749699	0	6.293792705	0
0.111924158	4.201410538	0.183372664	7.929540731	0.105349102
1.703950448	1.717455521	4.255106604	5.493723739	1.701387653
0	3.889566943	0	9.084744456	2.045123677

2.998320984	2.024417277	0.121680161	6.805021942	1.95765648
0.643339595	4.316841247	0.640270768	5.411815141	2.488878057
0.00506601	2.355322912	0.007352029	6.24830809	2.027650586
0.013152547	3.679208459	4.096540243	7.17723302	1.652262581
1.665765785	3.179271642	0	5.380778879	1.676542978
2.658836002	2.994470496	10.70122361	7.389000262	1.891406779
2.837138973	4.991771463	4.992388485	5.921275388	0.004808052
1.520506771	5.209817963	1.126075783	6.348160085	0.644769817
2.249871131	5.457697804	1.275984658	8.106360339	1.296132181
0.000284801	3.461486717	0.000411184	8.364152471	0.000271381
0.110080178	0.170683322	0.179956975	6.231678605	2.100849188
0	3.849398425	0.906285307	7.527631431	0
2.681891194	2.832801985	0.236353794	7.626887856	0.130970122
0.868363429	4.477282146	0	7.202908578	0.870183197
0	4.800769525	7.009317144	5.748943797	0.937786278
1.131580446	3.667826388	0.044212134	6.152396219	1.734281477
0.032068669	4.399531155	0.047937944	6.071891217	0.030447698
0.155974557	2.459698107	0.269566927	7.048735562	1.638120007
2.92715133	4.560269608	0.822085578	7.830743581	0
2.084342747	1.747873169	0	6.385140245	0
1.716427262	6.186450969	3.347863197	6.235427617	1.111087569
1.514654026	1.581947399	2.260919257	6.625091581	2.145906726
1.185802561	3.031114651	0	5.763122675	1.575267956
0.037833089	2.566234392	0.056918453	7.07633607	1.164159357
3.567178008	3.709441399	0.177465907	6.120274756	1.455124906
3.783780442	3.778966672	0.981906713	7.036040769	3.43659253
0	4.173076021	0.757259258	6.526139204	1.694887844
1.663083937	3.807999319	0.021966307	5.470421545	1.065740707
2.523436673	3.754440655	0.4912158	4.49140445	1.305946337
0.810688747	2.50917744	0.806499637	6.617821624	0.813137193
0.08074181	4.133028238	0.127535805	8.016757032	0.076251482
0.664770188	4.892069393	1.524974281	7.209485309	1.866455604
0.195105012	3.149982736	0.354243173	7.200989974	1.78400759
1.586601513	4.324224621	0.000447439	6.394708742	1.586568784
0.15966643	4.491631996	1.764030913	9.047631574	0.149548427
0.125329371	5.649624526	4.297546865	8.337468272	1.520340043
0	4.552096725	0	7.876822047	0
0.010285551	3.394992249	6.602046474	6.73685533	0.009789694
1.236600773	3.215593754	0.710179743	6.956315551	1.630581186
0	3.272571987	0	5.517622833	1.081022084
0	5.190626195	0	5.781375529	0
1.266396302	3.737125398	7.326821837	5.901008078	0.05761845
0	3.761272044	0	6.160858061	0
0.014948179	4.014956416	2.685476596	6.067510951	2.080619535
0.682729634	2.791562237	1.179793694	5.929413455	1.20234033
1.382977053	3.42098822	0	5.348791388	0.833956454
4.073291458	4.789928087	0.989110384	7.069408309	1.57287143
0	2.909882701	1.950283205	5.927553043	0.753494561
0.512933583	1.801397823	0	6.777778162	1.33986102
1.433573284	4.129872359	0.406766154	6.515383482	0
1.037315136	5.106234895	0.012133026	4.061395196	1.03670061
0	5.217811778	1.331853145	7.471033433	0.538234843
1.854569783	4.806274067	0	6.067432576	0
0.893381354	1.84954668	1.448969802	4.816684052	0
0.660917288	3.726831368	6.354854076	5.027445874	2.683604554
0.010551729	4.778279333	1.645367713	8.230676282	1.046431513
3.100427051	4.585067856	6.8853942	4.448962569	2.416900598
0	4.466566469	1.332261292	6.856390112	0

0.170127269	4.255296378	0.299256212	7.0186815	2.345926157
0	5.422328537	0	5.663944628	1.03669979
0	4.011980265	0	5.655336817	1.369224751
0	5.43635219	2.192867908	7.123459355	0.879203406
0.020278887	3.729331583	3.846744675	6.934951546	0.019279402
0.070247686	3.797862811	2.427424653	7.063904528	1.299788925
2.781699275	5.551231048	4.624502449	6.258964614	0.640365428
0	1.430933523	0.433582521	7.063722473	1.49407572
0.00421143	4.938247546	1.609120389	8.367912261	1.018592242
0.035812538	1.170915948	0.053757541	6.533536711	0.033988094
3.072098092	4.79634681	0.072188907	6.049181082	1.204951021
0.02854514	3.786705157	9.967891042	6.120430591	1.124451974
0.062033043	3.21884442	1.291801107	6.205547546	0.05870239
0.006640126	3.369252789	3.046576163	6.050773724	1.029277742
0.019535471	2.122150847	1.091541849	6.557226316	0.018574186
0.054487009	2.335052813	1.893250402	7.801741675	0.051604244
3.267304083	4.182733023	0	5.768834817	2.179170908
1.750013704	4.755008571	0.048033004	5.043903588	1.746358155
0.646150886	4.367667805	0.643035475	6.034819625	1.535659649
0	2.935995424	3.546352529	6.735753783	1.90223345
0.04653452	2.620932824	1.218535273	6.430530887	1.815556097
0.620805051	5.06686442	8.338256883	4.39758968	1.123167945
0.028224949	3.519342398	2.999353503	5.015859889	0.02680982
1.131120505	1.140963634	1.138569268	8.036142595	1.128767973
0.104715989	2.631573199	0.170103951	6.93017191	0.098639377
1.064041401	5.691097085	0.021015558	6.584809997	1.062959829
1.930469567	4.492856327	0	5.39712423	2.513746231
0.580401629	5.494088694	0.578564196	6.620017007	0.584395273
2.004698605	3.813473496	5.39871606	6.028809309	1.003702407
0.187224161	0.31483093	0.33652474	6.691814767	2.412211005
0.131214896	2.784910817	1.624005946	7.161629753	1.54321804
1.998196739	3.980745685	0	6.740029976	1.299780557
2.388095183	3.2516392	0.016965958	7.07742391	0.011046513
0.080522295	0.121165134	1.37966475	6.900829503	1.971920107
0.688012686	3.055978698	0	5.456500311	0.691469113
3.888727737	6.032457337	0.020086517	6.596251775	1.655082855
3.236387357	0.060635317	0.063276608	5.832136732	1.793350533
0	1.799536069	0	6.354541157	1.167592255
0	4.072489089	0	5.86990303	0.854201975
0.666457324	2.454045815	0	6.547370867	1.564330776
0	1.385009895	0	4.940151837	2.380087833
0.210232356	3.591446967	0.389250734	10.75394128	0.195928023
0.905201511	3.842750685	7.389012416	6.120374456	1.474261605
0.065156672	2.398844021	0.101092471	7.29483541	0.061637255
1.53350249	3.389197266	0	6.479335801	0.957146017
2.240504569	3.561228079	0	4.612278249	0
1.871498564	3.978587605	0.087619922	7.516262119	1.244901205
0.049344402	4.596407057	1.864480218	8.509584165	0.046760237
2.223098398	4.343144827	4.358544936	6.769438181	2.642604481
2.379126338	4.966712688	12.22090436	8.114351873	1.311071314
0	4.941117078	0	9.432801997	1.374515342
0	5.210547389	0	6.707197421	0.480001707
1.339817421	2.476535552	0.123260101	7.208082468	0.073926576
0.142245324	3.197563154	1.678008282	5.615925934	0.133467878
1.956576041	4.516824539	0.117766722	8.786962034	0.070918277
0.072139567	3.05308224	0.112824497	4.594606297	0.068191099
0.94003291	2.980636024	0.488408902	7.667113691	0.492159572
2.441798699	3.569412877	2.521514488	6.258418092	0.081316905

hsa-miR-548y	hsa-miR-1323	hsa-miR-454-5p	hsa-miR-519d-3p	hsa-miR-338-3p
0.09296292	0.135482595	1.399131064	0.135511744	10.68636043
0.005690323	0.007689161	2.031962668	0.007690427	10.00613278
0	0	0.777338528	0	11.26032045
2.811098706	1.412832106	0.861808438	2.597696561	10.16499815
0.01300128	0.017677044	0.014083495	1.058350856	9.527231832
0.027118389	0.037316428	0.029456722	0.03732301	10.93786465
0.171447649	2.951391535	2.38854098	1.771418484	11.42328078
0	0	3.211570315	0	9.893034084
0	0	2.660175035	0.766454472	10.14735533
4.003483641	1.251480832	3.765123516	0	12.67731576
0.092821069	0.135258776	1.398541018	0.135287864	11.66240108
0.079144646	0.113939537	0.086839044	0.113963079	10.48736576
0	0	0.951223552	0.950712549	10.3793713
0.055787153	0.078686415	1.867176342	0.078701578	9.1999125
0	0	1.547610795	0	10.50694852
0.063835836	3.208516786	2.670669101	3.87495245	10.41570236
0	3.213472716	1.910847043	5.013626934	10.48970712
3.954037854	0	0	0	9.358104506
0.104186461	0.153370522	0.114865132	0.153404606	12.10311931
0	0	1.953641001	0.963426945	9.994557881
0.283012566	0.494012673	2.834419255	0.494190038	10.46850922
0.092233627	0.13433248	2.812383332	0.13436132	10.74278505
0	0	2.170254172	0	9.433871582
0.153519112	0.236389047	0.170843594	0.236449377	11.61473183
0	0	0.864353893	0	14.70458598
0	1.47592362	1.932511202	0.452939606	10.90572705
0.008435314	1.037872118	2.85779135	0.011426699	11.60627137
0.143092886	0.218223427	0.15892844	0.218277543	10.68495624
0.041853369	0.058325293	1.182850074	0.058336063	15.4902419
0	0	0	0	10.49467174
2.474933472	0.140461646	0.105796747	0.140492143	10.95218526
2.46568486	8.092500985	2.471588909	7.489385577	9.64667672
0.048050526	11.21403121	1.209477257	10.77468821	10.93050992
0	2.926409596	1.564192409	3.264395138	12.20200512
0	0	1.280890951	0	9.159623486
2.794396812	1.255462938	2.780489827	0	9.315184725
0.078785748	0.113386931	0.086439289	0.113410335	10.37406663
0.013673215	1.061368268	2.665202825	1.061364022	10.55899369
0.036868169	0.051158154	1.16134763	0.051167456	10.74293261
0.020148497	1.090396811	2.437105864	0.027566042	10.33056434
1.411235461	0.852109477	0.852857275	0	10.02334416
0.005675804	1.025487484	1.02510886	0.00767071	9.52951426
1.408151022	5.967787306	0.10845217	6.072587905	10.63584812
0.136916281	0.207622683	1.579363787	0.207673294	11.3624239
3.391576192	6.729502862	0.128053345	5.268632751	9.598175143
0.917675132	0	0.916052276	0	9.602578034
0.190465985	0.303624478	0.21342781	0.303710123	10.37241832
0.084317428	0.121942903	0.092606698	0.121968485	12.91198306
2.386813751	7.950066906	2.121362307	8.035769845	10.74320458
0.154657358	0.238393137	1.650704351	0.23845417	10.16266696
0.075314931	1.337394897	0.082576158	1.987435156	10.96963605
0	0	1.664720067	0	9.565731675
0	0	1.666660294	0	11.1905637
0.119627116	0.178569132	0.132276816	0.178610601	10.41878878
0.024279184	0.033328686	1.710732917	1.108898103	9.698307643
0	0	1.320770694	0	10.52134911

1.977751468	0.118818167	1.35463639	0.118842947	10.57895096
0	0	3.018508662	0	12.90112813
0.005341467	0.007215641	2.61662427	0.007216828	8.924909456
4.080802311	2.082443741	2.077337876	1.062301619	10.36314961
1.004835993	0	2.431624565	0	9.662610362
0.06667675	11.19898618	2.353509112	10.56207799	10.62246465
4.353352095	3.844332728	1.612952014	3.621623783	9.886165741
2.776049479	0	1.500827084	0	10.95752079
2.648577534	0.750956854	1.669103288	0	11.01999429
0.000300099	0.00040368	1.001330068	0.000403746	10.94473283
0.117629287	0.175269556	2.597617667	0.175310029	8.670103933
0	0	0.906005554	0.905264571	10.61757534
0.149656975	0.229619903	1.630675456	0.229677887	10.44544185
0.866495337	0	1.420579891	0	10.61245639
0.935872263	4.751009718	3.491956375	5.101984816	10.71127062
0.031367814	0.04332101	2.499081219	0.043328754	9.825931368
0.033930981	0.046963956	1.148644093	0.046972418	10.6671656
0.167612525	0.261498983	0.187021098	0.261568341	12.81290249
3.993970467	0	1.367218511	0	10.93060656
3.326485451	0	2.54639635	0	10.31961229
0.026893368	2.980985388	2.147922681	3.501261672	9.170239713
0.129191377	0.194529801	2.206470239	0.194576209	10.45669356
0	0	1.171754452	0	10.05538749
0.040059884	0.055739775	2.217599355	0.055750011	9.737097725
0.116167372	0.172862533	0.128366767	0.172902285	11.04069879
0	0	0.98192858	0	10.51761402
0	0	1.982263811	0	13.69509504
1.068063235	0.021544827	1.667395859	0.021548493	10.52847116
0	0	2.176478506	0	9.884220891
0	0	2.054635161	0	10.07908005
0.085962668	0.124503684	2.010587944	0.12452993	9.739993211
0	0	2.298155359	0	11.55469252
2.500750444	0.342341327	1.870993625	0.342443211	9.761307049
0.000326552	0.000439273	2.323836861	0.000439344	9.955154208
0.171656567	1.772413428	2.837497339	0.268896734	11.64225955
0.134177221	2.290925416	1.568279377	1.602050075	11.11580216
0	0	1.435521989	0	10.84707345
1.640056795	6.206834749	2.060640469	5.935447294	10.46080458
1.231086812	0	1.607022381	0.707231105	9.8966167
0	0	1.058579146	0	13.33976928
0	0.857071639	1.412945925	0	12.84371051
1.27213468	6.795447008	1.90993246	6.344283537	9.895042234
0	0	0.97072081	0	10.52926717
0.015781252	1.671141593	1.667256285	1.070815715	9.506907455
1.875801502	0	0.674836443	0	9.459025191
0	0	2.561208519	0	11.50050488
0.989358667	0	1.98611933	0	10.15175648
3.090854787	2.198005058	1.270534074	2.76117824	13.16107483
5.696870274	0	2.036810953	0.50828729	9.241257939
0	0	2.164553223	0.401766816	13.25819339
0.008787557	0.011905419	1.631041053	0.011907399	10.00976025
0	0.529012436	1.340147034	0	10.87893811
0	0	2.642177586	0	11.26458314
2.850455014	0	0.88976308	0	9.551958124
0	5.193586124	1.154313826	5.534414883	10.37567751
0.011133419	0.015113526	2.386210596	0.015116059	14.24221721
0.01817282	4.712009039	0.019705402	4.125395477	12.79083584
0	0	1.730969318	0	10.17805279

0.18313378	0.289914844	1.76362977	0.289995031	10.79296242
0.321199718	0	2.77356267	0	9.330010884
0.815063046	0.811349656	1.356184109	0	10.03029863
0	1.179218355	2.234107521	0.435226177	10.16639614
0.021423858	1.096112135	2.933410784	1.096105502	10.37991402
3.201292006	2.429537013	1.957705316	1.33457714	11.37372944
3.894395614	2.012953906	2.25149021	1.480205967	10.37135242
0	0.428668257	1.684507211	0.84503741	8.865429695
0.004439926	0.005993221	0.004801454	1.019938052	10.82870381
0.037910548	0.052651708	2.206340857	0.052661312	14.26301136
3.080590172	0.070646094	1.219220604	0.070659483	11.73760848
0.030189101	8.136536744	2.75875499	9.285833683	9.97089787
0.065887861	0.093755754	2.949841069	0.093774378	9.919557234
0.007002611	1.623346016	1.621733838	1.623344294	10.52709843
0.020636479	0.028240517	1.692166145	0.028245397	9.067878844
0.05781728	0.081694049	1.251212632	0.081709888	10.01589681
3.460498045	0.697671974	2.550189752	0	13.85825277
3.189575225	1.769262058	2.185722457	1.769254559	9.397053055
0	0	1.50833922	0	13.42207854
0	1.853292084	1.873095482	2.492678825	10.94795938
0.049328537	0.069187817	1.835692206	0.06920089	9.860208345
0.617000456	6.244634155	2.224956466	5.762739105	10.30021975
0.029849236	1.133854814	1.130947379	0.041177534	13.33044993
0.031256283	1.140155808	1.746008339	0.043170569	13.67011051
0.111822574	0.165745817	1.477092309	0.165783464	14.39487998
0.01513343	0.020613191	1.066728762	0.020616691	11.17747269
0	0	1.926514875	0	11.58223103
0	0	0.571627756	0	10.01797242
3.59018028	4.465699487	1.589618069	6.235199047	10.2872866
0.201950209	0.32547663	0.22677829	0.325571291	9.080680038
3.272075381	0.213896392	0.156063474	0.213949067	9.215342953
0.757772351	0	2.451970988	0	10.25555011
0.012249365	0.016644116	2.392565593	0.016646916	11.21734143
0.085726587	0.124135774	2.009488441	0.124161924	8.99688088
0	0.681105737	2.122052244	1.183153618	10.66221201
3.082863342	0.01970275	1.0638318	0.019706088	11.09966376
0.0443576	0.061948776	0.048344148	0.061960304	11.30469987
0	0.648660162	0.647193817	0	9.957300143
0	0	2.372869078	0	13.20789752
0	0	3.858066952	0	11.7279929
0	0	1.391269846	0	8.934417402
0.22738517	0.375581824	0.25653723	0.375698626	10.62722498
4.11243669	6.719007331	1.46689188	6.731162564	9.217084612
0.069233083	0.098805035	1.29964352	0.09882486	10.84105366
0	0	1.530952743	0	12.55457116
0	0	1.99988444	0	10.72322885
0.060502581	0.085688586	0.066146539	0.085705335	11.57006669
0.052325951	1.866729627	0.057116767	1.866719078	11.87404956
3.019594446	0.221062982	0.160802892	3.877299639	10.19190354
0.077637076	8.899615783	2.407698289	9.882058362	11.10030895
0	0	2.327852377	0	13.85739353
0	0.471875266	2.816151383	0	11.43773168
0.083293234	0.120352497	0.091463829	0.12037767	9.67408178
0.152603789	0.234780463	0.169795754	0.234840233	10.51100367
0.079843626	0.115016771	2.41849001	0.115040585	11.47876335
0.076720761	0.110214148	0.084140282	0.110236758	9.346155039
0	0	2.476092252	0.483813984	11.7062645
0.091789202	2.073085015	2.476253679	1.411222688	11.40790047

hsa-miR-200c-3p	hsa-miR-3682-3p	hsa-miR-376a-2-5	hsa-miR-9-3p	hsa-miR-6783-3p
6.322906406	0.123998471	0.078019564	2.079310549	0.072447657
5.655872406	3.207422076	1.02343413	0.007483963	1.022907728
7.701113617	3.226697465	0	1.564032865	0.397685757
6.707033074	3.483556813	1.430562447	3.586039637	0
6.202279614	4.087288712	0.011189589	3.085600333	0.010489595
6.421736753	1.122736707	0.023232878	3.98976507	0.021741206
7.096038644	3.27134335	0.140621528	2.477287944	0.129499229
7.865584596	2.29821525	1.070637174	0	2.218582515
6.648632711	3.08887965	0	0	1.325307681
6.543628773	3.651826952	0	0	2.468220087
10.6854149	2.859761496	0.077903868	2.868914217	0.072341373
8.716034828	1.356400472	1.936059157	0.110151698	1.301940094
6.276197786	2.253135818	0	0.950325893	0
7.168900201	4.056213466	0.047360046	3.354111724	2.583282358
7.382406368	1.957879715	0	1.957630203	0
5.015329166	3.394763764	0.054056621	0.087788184	0.050363498
6.630011473	3.327570744	1.246677908	6.760081825	0.725062172
7.659773671	3.756134335	0	5.487494718	2.331103925
9.082785909	2.580683262	3.066240784	1.469469058	2.452244038
5.962344642	1.539967407	0.966179941	1.95094306	0
8.609740167	2.267206742	0.225310906	0.46624397	0.205363418
7.564897774	1.414913453	0.07742464	2.075549734	0.071901092
6.285492073	0.885322966	5.105236561	1.444268144	0
6.689254311	2.834705556	1.575205188	2.387335633	1.556856595
6.339317977	2.385836002	2.82820891	0.862502656	0
11.85063078	3.014458764	2.180087466	1.700464729	0.471286849
16.26063677	3.225348543	0.007270731	1.631334482	2.041267602
5.776633255	4.163715491	0.118319677	2.796187442	0.109276816
7.738096494	3.068446207	0.035687872	0.05658595	0.033336562
6.670055441	2.843598765	0.801415845	1.71103518	0
6.948585768	2.538042178	0.08058338	1.433093924	0.074802091
7.510572985	3.757589764	0.022586981	0.0352151	1.104487753
8.326009508	4.310624804	1.192385259	0.065275802	0.038169092
6.788542311	0.673280941	0	0	0
6.803939689	0.74854861	1.301585512	0.749769762	0
5.807353976	3.478932549	2.244097494	0	1.290326308
5.888911489	2.445437517	0.066408256	0.109621309	0.061764524
7.699154228	2.405912512	2.068496529	0.018092213	1.054717038
7.272088963	3.233530579	1.148707521	1.785127133	0.029430268
7.200268705	3.717796077	1.082177711	4.662105854	1.080241398
6.192463592	2.94387444	0	2.110264911	0
5.398478421	3.037069514	0.004896633	1.025681038	1.022849524
6.588707104	2.890462308	0.0825095	1.44375474	1.999564458
7.233882601	0.187922603	0.113415677	0.199532098	1.50204978
7.888316049	5.796661705	0.096593875	5.590791293	0.089469404
5.352087064	3.676674591	2.220537731	0	1.494479432
6.368304217	3.018932058	0.155392602	0.290026608	0.142834023
7.313434255	2.475184278	0.070950431	0.117829129	1.320499982
5.632252792	3.673012284	0.860690346	4.965649418	0
7.967712767	0.214760415	1.579078826	0.228657834	0.117575206
6.394571069	3.2642806	0.063550601	1.987877323	0.0591302
6.656481814	2.421755387	2.259376466	0.745826142	0
8.636896611	2.427046707	1.048234742	2.564838272	0
8.422554109	0.162348721	0.099598431	0.17192671	0.092215144
6.681313459	2.961242563	0.020819542	1.717486338	0.019489654
6.954787227	2.009490433	0	0	0

6.227805184	2.464373532	0.069298414	0.114832577	0.064426752
15.47182922	3.387816815	2.311163753	2.023679118	1.1666008
7.38782226	2.354968904	0.0046087	1.024168943	2.612186533
7.715450237	3.551199845	0.011944414	4.095139757	1.055545111
11.8015094	2.900679574	0	0	0.555580174
7.186682561	4.229152522	3.648642358	2.720961166	1.875107526
7.98153666	4.791327308	0.004592851	2.031801746	2.026155749
7.235162961	2.723687739	2.09200755	2.410271388	0
8.454026378	1.960733753	0	4.388384208	0
7.736921025	0.000377368	0.000259361	4.089554814	1.001213013
6.129307371	2.198791699	0.097993082	0.168785148	1.436934061
8.741375221	2.858456857	0	1.467546814	1.48344055
8.748938981	2.357038879	0.123513068	0.220365153	0.113995761
6.928084425	2.385372583	0	2.383574375	0.874453947
8.485857695	3.222217116	1.923548366	3.220671341	2.245687527
7.985194065	3.371275893	0.026836736	0.042069275	1.726922615
6.409604087	1.768524597	0.029005799	1.153222224	0.027120799
7.323058806	2.444581471	0.13762494	2.458081285	0.126788357
8.007924878	0.817943761	1.383150715	2.889427829	0
6.425949388	2.530367765	0.826567384	4.221124884	0
6.946832708	3.984412436	0.023041769	3.174861173	2.717575126
7.521346937	2.256254767	0.107258634	3.954872964	1.476161645
8.3126587	3.256099759	0.681300974	1.162939341	0
6.211550434	2.562848734	1.161240234	2.234216989	0.031933173
6.72105655	2.643349833	0.096817205	2.198263207	0.089673568
7.30080082	3.431972897	0	0.981563752	0
10.80417335	3.60182868	0.767260191	2.616057257	0
6.668589921	3.80550056	2.078971535	0.020951348	1.063160661
8.888616849	1.965004489	0.95837144	4.294484375	1.620949543
6.496494412	3.016884261	0.815524129	0.802884262	1.770892109
6.709788453	0.114144409	0.072298482	3.10499169	0.06718804
14.46884468	3.594833556	0	1.519855174	1.193381206
6.933792	2.651603031	0.170909878	3.498922297	2.823268811
8.939049047	2.001944896	0.00028222	0.000427748	1.001319911
7.895678213	2.464194805	1.636355786	2.47833372	0.12964669
9.011354036	3.080053142	0.11123558	5.294002737	0.102830115
6.329377734	2.403614643	0.883529007	5.603504121	0
6.122789992	3.393527295	0.009346532	3.881575233	0.00876421
7.16619258	2.705109595	2.201117571	1.89155406	2.211834696
15.79954725	3.79667009	1.08751162	1.406254228	0
7.620918144	3.102002296	0.866035217	2.374184243	0.868642343
6.766594921	2.703774075	0.054735151	1.932178199	0.050990804
7.121058732	3.41485176	0	3.414091703	0
5.470145772	3.27282201	1.659787414	0.020914998	0.012716531
7.617009638	3.628074439	0	1.546257648	1.911608071
7.750720731	1.762666703	2.366360342	0.824447224	1.394384467
7.285688181	2.56929583	3.308593463	2.791204736	2.309447561
6.950840167	1.949573793	0	0	0.760660706
5.496382897	4.833907768	0	4.141828354	1.36206122
7.541992919	1.605610529	1.178195985	1.604122628	0
7.67145142	3.517845649	1.626955642	9.196284288	2.042969697
5.639204767	1.323849366	0	0.525388688	0
7.485735969	2.412228298	2.178098746	2.152790954	0.891216171
7.288592604	2.643096885	3.859803474	4.464729866	3.187203741
7.033050697	4.111859208	2.526859441	1.145498878	0
9.029583657	2.87881119	1.63802547	2.066261635	1.044637976
7.831744673	2.106837146	4.183343437	6.634427694	0.014627888
6.091064565	2.02791004	2.067051278	1.718974214	0

7.020915086	2.982719976	0.149715222	0.277171048	0.13771413
8.944093247	1.955985885	0	1.638821301	1.332151403
6.152567277	2.523227639	1.372774292	0.809847463	0
6.928343982	2.039504037	0.886552799	2.681362665	0.453219174
6.948204029	1.701588133	1.685908002	7.883427701	1.085239622
7.049227628	3.032174886	1.293896179	0.103590629	1.285852001
6.372011667	3.541994416	1.149565398	1.478240709	1.158146804
5.976191183	2.449128135	0.877312897	0.840412499	0.446556132
8.363520724	2.613160686	0.003831972	1.02009116	1.017890717
8.251685253	1.789656995	0.032366509	0.051100005	2.178235642
7.844020548	2.28952488	2.833694116	3.91397475	1.196011636
6.07146645	3.192429286	1.122308507	2.507026844	0.024169998
16.52539505	2.710724306	0.055758648	0.090755161	0.051936815
7.039773493	3.045672425	1.618493493	3.046364975	0.005665196
6.147509693	3.303812988	0.017716779	1.697783514	1.082154952
6.721907818	1.894242135	1.230010512	2.334676035	1.838978855
8.026045441	3.888019009	1.623951108	2.335609621	1.242554654
8.12446205	4.979910476	0.029060961	4.034496845	1.134007829
7.139271146	2.031228267	1.543127611	2.581216481	0.658852154
7.485406041	2.809141404	0	1.852495156	1.22350505
4.98084625	3.481223783	0.041962324	1.222573544	0.039162483
10.85829051	3.58855741	0.62843679	2.812893058	1.822091294
8.196230581	1.135055478	0.025549979	2.504987216	0.023901503
7.277686476	2.511193903	0.026742273	1.141166607	2.47336326
9.514755657	3.467576084	0.093316702	0.159710101	0.086472117
8.046393877	2.08916121	1.061930387	3.099442652	0.012198081
7.547835718	2.722954364	0	5.036928366	0
6.677558915	3.176936713	0	1.394268799	1.086539443
6.800890687	3.590905	0.000762328	1.589829759	1.003564487
5.24005101	3.075337329	0.164241995	1.913102743	0.150801134
7.002121904	3.820421403	0.116326582	4.018160189	2.153303875
8.480737312	2.932840224	0.766486478	1.275491031	1.706901656
6.934292945	3.079465774	1.050224752	2.072850634	0.009886304
7.463863423	3.094108522	0.072105126	0.119931333	0.06701014
7.637217065	2.496130063	0	0.678569712	0.699814145
6.805041283	3.264420322	0.012450348	2.085952847	0.011669323
7.7878404	2.856951075	0.037793124	3.876513384	0.035292465
7.270155629	2.256428256	0.662659442	0	0.667907188
9.669889385	4.004331984	1.414052766	0	0.858922053
9.474973763	3.137288705	0.673596047	1.152744554	1.888440245
6.668993676	3.554564932	1.808979624	1.780965626	0
7.812937346	2.017537231	0.183660226	0.357133682	0.168227902
6.66726687	2.182737976	0.907884809	1.866300552	2.206584703
5.598378709	1.953425614	0.058528743	2.398327032	0.054495864
6.794936974	1.939104758	0.957781837	0	0.958677657
8.015902933	3.23850417	1.118655182	0.603065276	0
6.893687593	2.345856137	0.051287338	1.272864482	1.233920135
8.290678177	3.499235732	0.044470069	1.236066269	1.81624306
6.845202723	3.842346759	0.119621216	1.652529572	0.110460018
9.207734003	1.99665205	1.304909225	1.349958245	2.347434969
14.3777908	2.885770136	0	4.864545262	0
8.979919625	3.790631832	0	2.533014974	1.305657747
6.369647471	3.974434195	0.070110576	0.116304114	0.065174491
7.78363254	2.371459757	0.125838478	2.382731212	2.195198925
7.229892779	1.359527393	0.067278064	1.359886499	0.062565953
6.789082346	2.434288888	0.064708793	0.106575359	0.060198122
5.868947336	1.252425513	1.310878949	1.253099646	0.964841219
8.551891704	2.515098472	0.077061972	4.491452136	0.071567862

hsa-miR-515-5p	hsa-miR-519b-3p	hsa-miR-4536-3p	hsa-miR-3677-5p	hsa-miR-525-5p
0.130666231	0.124575822	0.064237465	2.067725762	0.137534447
0.007478066	0.007205798	1.022048118	2.033373124	0.007777982
0	0	1.182598984	2.579911174	0
1.810923291	2.381817468	1.440433884	3.372864239	2.787030883
0.017180484	0.016540996	0.009432918	3.405780468	0.017886174
0.036221523	0.034815679	0.019499455	2.484146209	0.037778417
2.47709573	1.768760276	1.583156686	2.444991854	2.95139128
0	0	1.776607579	3.354460848	0
0	0	0	4.018612262	0
1.24967373	0	0	3.659659788	0.735433252
0.130452273	0.12437412	2.97883423	0.117570432	0.137306381
1.35676808	0.105100426	1.907496674	0.099545332	0.115595708
0	0	0.956903878	2.253991529	0
1.885552484	0.072962115	0.039408327	3.931004818	2.323047652
0	0	0	3.27548182	0
1.287888575	1.287936007	0.044875187	4.483277257	5.386300488
4.357985678	3.32691304	1.659879004	2.359797276	4.578741167
0	0	0.814943204	1.719916317	0
0.147745802	0.140653266	0.071529902	3.181658479	0.155771049
0	0	1.548024622	2.271644025	0.963637709
0.465473304	0.431416088	1.898528664	2.237102135	0.506640847
0.129566723	0.123539215	0.063760456	0.116791064	0.136362539
0	0	0	2.642015256	0
0.226492079	0.214176369	0.102561431	0.200645092	0.240648816
0	0	0	2.130542189	0
0	0	0	3.111524279	0.881492306
0.011108426	0.0107006	0.006138166	1.630815964	0.011557966
0.209334251	0.198240412	0.09613551	2.311233751	0.222042273
0.056536132	0.054246283	0.029818999	1.188204401	0.059081744
0	0	1.763299405	2.848432628	0.791034896
0.13542429	0.12905943	1.345555549	3.138832183	0.142608713
5.673311699	5.176669664	0.018961796	1.726743471	9.237018853
9.178453288	9.072443598	0.034103816	0.059481525	10.40121945
4.351107608	3.056340787	1.931616946	1.176833752	2.793834568
0	0	0.775450774	2.432702069	0
0	0	1.301243723	4.273941735	1.939139912
0.109513861	0.104599228	2.925537929	2.437888826	0.115033301
1.061835702	0.017403358	1.052615331	1.659056983	0.018821756
0.04961212	0.047631247	1.742867521	1.16592359	0.051811361
0.026769717	0.025751835	0.014551856	0.024589595	0.027894975
0	0	0.868466782	1.401834234	0
0.007458902	0.007187345	0.004137524	0.006876144	0.007758035
5.088198645	5.135694619	0.067831573	2.540392021	7.449019782
0.199302769	0.188901779	1.477290663	0.17741363	0.211193083
5.121826019	3.260483718	1.410377666	2.969036917	6.713155651
0	0	0	2.684678465	0
0.289643834	0.272433711	0.124804856	1.839426306	0.309684316
0.117711817	0.112350095	0.05855745	2.803491039	0.123742986
6.661639281	6.404193142	0.867885586	3.472304213	9.454225306
0.228382492	0.215929324	0.103258857	0.202252053	0.242702729
2.770188181	0.099764266	0.052583345	1.979520684	0.109613459
0	0	0.771764824	2.426407693	0
0	0	0	2.83243515	0
0.17173804	0.163158664	0.081417094	0.153631674	0.181491949
0.03235924	0.031113724	0.017489263	1.109499118	2.142932044
0	0	0.804891233	1.314162183	0

0.114718896	0.109521661	0.057226263	3.064494432	0.120561629
0	0	1.180787795	3.6418832	0
0.007017764	0.006762526	0.003894645	3.356685255	0.007298896
2.082469591	1.062931064	0.010066443	1.660180742	0.019113118
0	0	0.566102658	4.140745533	0
8.64064203	8.89928739	0.046792459	3.728392411	12.30765481
2.031799859	0.006739149	0.003881274	3.942079967	4.792882682
0	0	1.177967597	2.986909825	0
0	0	0.774702875	2.78474325	0
0.000392786	0.000378718	2.586423039	1.001359137	0.000408261
0.168600923	0.160221194	2.044909284	2.188761765	0.178121906
0.904499486	0	0.916505389	2.449708064	0
0.220103237	0.208247921	1.516905995	0.195206281	0.233713124
0	0	0	3.376403402	0
4.538571981	4.289419762	0.942081508	3.707952055	5.935626374
0.042033387	0.040381617	0.022494821	3.520154074	1.139823485
0.045557183	0.043753577	1.128332267	2.194661162	0.047558084
0.250141952	0.236066213	0.111141144	3.227358353	0.266399938
0.818725061	0	0.839200714	1.756703585	0
0	0	1.390923764	1.750708634	0.816937092
1.121509416	2.15729971	0.019340397	2.974253332	3.766815625
0.186893776	0.177327783	0.087459831	3.036880864	0.197802334
0	0	0	2.293911356	0
0.054039071	0.051861563	1.150733079	3.711270642	0.056458648
0.16631156	0.158076534	0.079216012	2.970463271	0.175663873
0	0	0	2.559278914	0
0	0	1.321704302	3.075131696	0
0.020934308	0.020148521	1.060719908	3.423559507	0.021802047
0	0	0.986421763	3.184308548	0
0	0	0	2.516193563	0
0.120163663	0.114666108	0.059642632	1.383908643	0.126350583
0	0	2.160269863	0	0.659811768
0.325757583	0.30547066	0.136628277	2.625988794	0.349559047
0.000427417	0.000412108	0.000238879	1.001478934	0.000444259
0.257027023	1.769693026	0.11358106	1.758096925	0.273919623
2.749033153	0.184784559	2.100920572	1.59526836	0.206422585
0	0	0	1.833122772	0
4.244804739	4.073186268	1.04186148	2.38780004	6.819701283
0	0.703434645	0.733231985	3.612471476	0.708814106
0	0	1.491917031	2.635778314	0
0	0	1.436132893	3.105687323	0
4.74600989	3.573179397	0.045427725	1.289674595	6.570384831
0	0	0.97419017	0	0
0.020897988	1.671169804	1.65437364	2.417288607	0.021764145
0	0	0	3.787820469	0.677595429
0	0	0	0.823787192	0
0	0	0	2.569528521	0.989053079
1.644990288	1.261911953	0.767808713	2.606796293	3.042848255
0	0	0.538289005	3.593856723	0.95667651
0	0	0	2.996360511	0.404495142
0.011575369	0.011149948	0.00639303	5.058466566	0.012044346
0	0	0.559061851	2.050460701	0.984592102
0	0	1.462147627	0.879948115	0
0	0	0	2.645478847	0
3.927159469	4.267125365	1.590631937	2.766723382	7.422424723
0.014691447	0.014147667	0.008087423	1.050326039	0.015291245
2.107662574	3.117380904	0.013141492	1.082045402	4.213562759
0	0	0	2.502117259	0

0.276811957	0.260646601	1.617955503	1.807750135	0.295586031
0	0	2.289224939	2.447721412	0
0	0	1.385866308	2.054335362	0
0	0	0.915703248	2.701080999	0
0.028491765	0.027404307	1.678512596	3.306021546	1.095544588
0.103490268	0.098897183	1.273147568	2.416267699	2.768589747
1.478218611	0.625190198	1.558082698	2.230221462	1.482076774
0.424369053	0	1.260592448	0.837713713	0
0.005829334	0.005617902	0.003239196	1.60915567	0.006062167
0.051055545	0.049010914	0.027075995	3.940688659	0.053326191
0.068423805	0.065585032	0.035668242	2.617095284	0.071586793
7.872730047	7.628020508	0.021665548	0.037032041	8.592901224
0.090669427	0.086741065	0.046260695	1.931809212	0.095065156
0.009211745	0.008875017	1.616116367	1.623060729	1.031257884
0.027428218	0.026383789	1.078937378	1.696334523	0.028582966
0.079067085	0.075717127	1.21436299	2.658984537	0.082807245
0	0	0.72411697	2.977438032	0.699320923
0.045647051	0.043839546	0.024339657	2.524077373	3.025670462
0	0	0.66799194	2.873946485	0
1.555589882	0	0.709273943	3.481189658	1.18634666
0.067017793	0.064245157	0.034983465	0.06110345	0.070106262
4.959079359	4.374688238	0	2.548685744	7.104464087
1.747497634	1.135074599	0.021426213	1.74520324	2.17518096
1.141182955	0.040235144	0.022416407	2.179678194	0.043704298
0.159538509	0.151726679	0.076440235	0.143031466	0.168398289
0.020030295	0.019279956	0.010964869	1.666899085	0.02085875
0	0	1.526325609	3.614010861	0
0	0	0.604286219	2.314263053	0
3.813451815	3.706414061	0.000645139	1.589780243	5.983692049
0.310049309	0.291125362	0.131559896	3.668302946	0.332179004
1.633726066	0.194432034	2.122147913	0.182506753	0.217613039
0	0	0.777503409	4.689364184	0
0.016177668	0.015576865	1.639086164	3.40099902	0.016840546
0.119811454	0.114333471	0.05948704	3.835271383	0.125975924
0	0.676963459	0	2.814274002	1.184938838
0.019146743	0.018430917	1.055655083	0.017612393	0.019936962
0.060034225	0.057585266	0.031554344	4.982676941	0.062758525
0	0	0.676895363	1.138614574	0.650539219
0	0	0.863548908	2.771272973	0
0	0	0	1.82261248	0
0	0	0.85697139	2.095662271	0
2.030106313	0.33354258	0.146270091	1.99846929	0.383865263
4.69862952	5.842959958	0	2.443750823	6.479275487
0.095520975	0.091344228	1.2543069	1.309973238	0.100199019
0	0	2.534702964	3.739104862	0
0	0	1.525237329	2.934497447	0
0.082911521	0.079372349	0.042617468	3.170996877	1.269471195
1.867281662	1.865799992	0.037041279	1.862397584	2.303519701
2.342969136	3.136573303	0.097152563	2.772672037	3.442456557
10.43833927	8.100271637	0.054130231	0.097573629	9.90456647
0	0	1.390803798	0.813592196	0
0.467816274	0	1.869631225	2.882162891	0.474442536
0.116188654	0.110910825	1.30251732	1.372133614	1.370971961
0.22497438	0.212768621	0.102000013	3.857794727	0.239000413
0.11107621	0.106077213	1.290787282	3.64850101	0.116692101
0.106471508	0.101720018	0.053520326	1.343106254	0.111804574
0	0	0.513683062	1.531381633	0
0.128897301	0.122907995	0.063469579	1.409581341	2.523035115

hsa-miR-512-5p	hsa-miR-520d-3p	hsa-miR-760	hsa-miR-518f-3p	hsa-miR-498-5p
0.130174374	0.131696629	0.102826384	0.127465985	0.129668329
0.0074563	0.007523536	0.006182374	0.007335753	0.007433867
0	0	1.962711387	0	0
0	2.380062541	2.612588286	0.859537673	0.85977534
0.017129322	0.017287389	1.653020207	0.01684609	0.017076597
0.036108876	0.036457005	0.029594167	0.035485805	0.035992818
2.476350873	0.259170569	1.719162221	0.248968127	1.773388904
0	0.566764289	2.329562968	0	0
0	0	0.76645632	0	0
0	0	3.764189252	0.730985974	0.731501048
0.129961415	0.131480574	0.102666526	0.127258497	0.129456395
0.109645104	0.110878083	0.087296334	0.10744851	0.109234964
0	0	0	0	0
0.075913163	1.251452754	2.299335293	1.251957068	1.251816684
0	0	1.959904907	0	0
2.704254407	0.088342082	0.070191089	2.973968676	2.367832625
2.981240115	3.320106797	2.3771049	3.90048439	1.89126799
0	0	1.33407976	0	0
0.147172186	0.148947951	1.446453953	0.144016207	0.146582175
0	0	2.535817879	0	0
0.462641602	0.471453953	0.324976332	0.447309523	0.459743616
0.12907999	0.130586358	2.040677033	0.12639968	0.12857921
0	1.44433093	0	0	0
0.225489307	0.22859752	0.171889777	0.21999345	0.224459123
0	0	0.864231951	0	0
0.448421868	0.449459247	1.217992171	0	0
0.011075814	0.011176561	2.047339235	0.010895225	0.011042203
0.208432298	0.211227239	0.15988257	0.203484796	0.207505443
0.056352346	0.056920501	2.227444095	0.055336755	0.056163051
0	0	1.328892808	0	0
0.134910062	0.136501671	1.412932194	0.13207914	0.134381038
6.158662665	7.002867857	2.737722682	7.06202161	6.975536673
9.684667559	9.462088121	1.829892285	8.888607274	9.38540604
2.649055196	2.485240434	0	3.166040227	2.796080076
0	0	2.231457472	0	0
0	0.735269391	1.955507064	0	0
0.109117665	0.110343456	1.340441648	0.106933804	0.10870991
1.061862865	0.01819032	1.060392435	0.017725018	0.017968056
0.049453223	0.049944384	0.040314956	0.048574884	0.049289546
0.026688219	0.026940048	0.021956347	0.026237243	0.026604242
0	0	1.40659544	0	0
0.007437194	0.007504255	1.614845748	0.00731696	0.007414819
4.529319251	4.952284312	0.109047464	5.47978386	4.622253521
0.198457873	0.201075586	0.152792116	0.193821055	0.197589516
5.373469556	5.490162245	0.128781953	4.412508108	5.371776531
0	0	3.202534687	0	0
0.288234868	0.292606625	0.214825461	0.280537116	0.286788818
0.117279281	0.118617674	2.003821083	0.11489605	0.11683418
7.340141443	7.075717119	1.405808054	7.115231549	6.974796308
0.227368363	0.230511874	0.173203536	0.221810781	0.226326542
0.104031821	1.988008886	1.325819102	1.986751158	1.339689254
0	0	0.747648353	0	0
0	0	2.740979068	0	0
0.171042758	0.173195984	0.133035157	0.167221856	0.170327866
0.032259471	0.032567783	0.026480239	1.717344758	0.032156676
0	0	1.320457498	0	0

0.114299734	0.115596682	0.090838849	0.111989865	0.113868378
0	0	3.513046339	0	0
0.006997361	0.007060388	0.005802933	0.006884355	0.006976331
0.018302131	1.661074429	2.402005627	0.017998691	0.018245642
0	0	2.943364963	0	0
9.788269811	10.15872338	0.073357424	9.165154258	10.15601702
2.842149601	3.035352199	2.352928504	1.614253592	3.943507631
0	0	1.12780694	0.630306803	0
0	0	0.750803183	0	0
0.000391662	0.000395133	0.000325685	0.000385435	0.000390503
0.167922002	0.170024455	0.130760746	0.16419044	0.167223899
0	0	0.905915459	0	0.904404447
0.219138482	0.222128549	0.167436389	0.213849308	0.218147256
0	0	0.86392272	0	0
3.704181622	3.482297094	1.506362248	4.355456507	2.489090879
0.041900971	0.04231023	3.771842586	0.041168756	0.041764558
0.045412554	0.045859585	2.513468296	0.044612931	0.045263565
0.248993534	0.252554558	1.703906677	0.242706835	0.247814157
0	0	0	0	0
0	0	0.816334668	0	0
1.731581704	3.175155518	3.968997415	2.978868001	1.731566931
0.186117534	0.188522064	1.54914842	0.181854887	0.185319584
0	0	1.171343796	0	0
0.053864337	0.054404487	2.546625253	0.052898657	0.053684359
0.165644493	1.523275326	0.129097569	0.161977673	0.164958554
0	0	1.976860103	0	0
0	0	2.452547038	0	0
0.020871423	1.071402987	2.088070343	0.020523349	0.02080662
0	0	2.733092467	0	0
0	0	1.742928764	0	0
0.119720077	0.121092712	0.094948263	0.117276254	0.119263623
0	0	1.834949702	0	0
0.324091485	0.329264163	1.873223956	0.3150055	0.322382516
0.000426194	0.000429972	2.001847696	0.000419418	0.000424933
0.255834749	0.259532179	0.192892068	0.249310209	0.254610465
0.194065301	0.19660636	0.149653227	1.604143768	0.193222254
0	0	0	0	0
4.830349882	4.883240246	1.047978394	5.204260082	3.658428621
0.704662217	0	2.731053079	0.70392293	0.70450068
0	0	3.234417684	0	0
0	0	0	0	0
5.009655684	3.8762933	1.280692806	4.587950234	3.734275325
0	0	0	0	0
0.020835218	1.071280704	2.412768844	0.020487776	0.020770532
0	0	2.513306758	0	0
0	0	1.373992024	0	0
0	0	1.986094238	0	0
0.741412001	3.16721416	0.743403975	0.740780154	1.644981385
0.504132776	0.505077699	3.497789651	0.502806376	0
0	0.398070553	1.403042688	0	0
0.01154135	0.011646448	4.912421496	0.011352965	0.011506287
0	0	2.417147591	0.523766402	0
0	0	0.881896426	0	0
0	0	4.087470168	0	0
2.607733374	5.147053268	3.267288904	5.154354805	3.989062095
0.014647952	0.01478233	1.049217018	1.050464169	0.014603125
1.684423118	4.447086448	1.080143886	3.580255979	2.434185501
0	0	0.795744128	0	0

0.27549	0.279590922	0.206257008	0.268262953	0.274132974
0	0	2.133636226	0	0
0	0	1.355902151	0	0
0.849077914	0	1.463497782	0	0
1.096873328	1.096730726	1.696377965	0.027922853	0.028314957
1.336814131	1.336482865	0.082309037	0.101079644	0.102739261
0.626842506	0.627557589	2.60031671	1.770195589	1.113305825
0.424040162	0	0.422362215	0	0
0.005812435	0.005864639	1.019672104	0.005718827	0.005795015
0.050891514	0.051398555	2.206775036	0.049984858	0.050722553
0.068195738	0.06890091	2.271362193	0.066936156	0.067960874
8.161096698	7.852194964	0.032966086	7.562033734	7.315066199
0.090353238	0.091331209	1.916513993	0.088608815	0.090027734
1.031699584	0.009267991	2.362632614	0.009035726	0.009157074
0.02734459	0.027603003	1.692348419	0.026881842	0.027258419
0.078797712	0.079630736	0.063495095	0.077310751	0.078520355
0	0	1.592781938	0	0.694873162
0.045502109	0.04595011	3.786821523	0.044700755	0.045352797
0	0	3.13588222	0	0
0	1.182602323	1.570388128	0.678615756	0
0.066795065	0.067483713	0.054078541	0.065564891	0.066565695
4.489230657	5.996401893	3.704575892	5.903842333	5.484983239
1.134892664	0.04021406	1.131117685	0.03913402	1.134940463
0.041748479	0.042156109	1.137232964	0.041019169	0.041612607
0.158906087	0.160864265	0.124160252	0.155428558	0.158255706
0.019970252	0.020155769	0.016474212	0.019637889	0.019908375
0	0	4.239917392	0	0
0	0	1.701682961	0	0
3.175854287	3.913021195	2.004989053	4.761037522	3.912989531
0.308497278	0.313314559	2.517720173	0.300026625	0.306904914
0.204362753	0.207084854	0.156995885	0.199542899	0.203459936
0	0	4.326319536	0	0
0.016129606	0.016278096	0.013326674	0.015863516	0.016080073
0.11936946	0.12073716	2.781736476	0.116934369	0.118914643
0	0.678945268	1.568808323	0.677515567	0.678155244
0.019089465	0.019266434	2.670007748	0.0187724	0.019030438
0.059837612	0.060445453	1.811684388	0.058751318	0.059635115
0	0	1.820392731	0	0
0	0	4.023187504	0	0
0	0	1.537480012	0	0
0	0	2.581029168	0	0
0.35471695	0.360619374	0.258325734	0.34437073	0.352768853
4.698863505	5.988472452	2.189900008	6.455816835	5.948418517
0.095184658	0.096224964	1.932522333	0.093329616	0.094838457
0	0	2.937160831	0	0
0	0	2.705758311	0	0
0.082626842	1.272663817	0.066480637	0.081055678	0.082333741
0.071015653	2.909264554	0.05739987	0.069696419	0.070769643
1.652500166	1.652439578	2.27622164	3.144594042	0.210158751
9.018558763	9.592638572	0.085607187	8.48312186	8.989340985
0	0	1.758454868	0	0
0	0	1.764005385	0	0.467208966
0.11576294	0.117080192	1.359382027	0.113417127	1.375580858
0.223980691	0.227060676	3.594469975	0.2185342	0.222959813
0.110673159	0.111920185	1.344891861	0.108451686	0.11025836
0.106088561	0.107273303	1.331745887	0.103977425	0.105694424
0	0	1.990145711	0	0
0.128413681	2.073491111	0.101503944	0.125750423	0.127916097

hsa-miR-431-3p	hsa-miR-137-3p	hsa-miR-2114-3p	hsa-miR-1292-5p	hsa-miR-615-3p
5.571647159	1.414398882	0.115621393	1.392227101	0.104184439
4.499852245	1.615921164	1.025697784	2.617959462	0.006248714
6.294756616	1.347851	0.770355098	1.10623429	0
4.577126408	0.85952973	0	1.822742766	0
3.897191297	0.015756814	0.01557666	3.398717287	4.604135218
4.435530377	0.033098175	1.121957851	0.028161186	0.029929662
3.826641684	0.225054048	5.347173267	0.180219197	0.195625148
5.773338693	0	0.563311279	1.046016886	0.565864538
0.769371505	0	0	1.302943388	0
5.343925502	1.250789727	0	2.616151355	2.202610281
4.053310041	1.413775367	1.412508626	0.096934553	1.400896261
6.665860973	0.099149303	1.352557686	0.082556252	0.088414931
4.564228242	0	0.950327818	0.951739577	0
4.37382245	3.803761044	0.068185982	0.058075901	0.061984178
3.535886784	0	0	1.960676017	0.96864054
4.330717349	0.079357918	3.186763068	1.272528585	0.071059941
4.13951856	0	1.596343268	1.227212264	0
2.026296269	7.24897075	1.720871077	1.33743928	0
9.8331343	0.132178835	1.462135816	2.855655221	0.117065618
6.027419307	0	1.951943595	0.964221977	0
3.043183773	0.393245491	0.384974746	0.300133985	0.331028697
5.366090043	0.116311	1.409938861	1.389264114	2.043106862
9.968676078	3.001242722	0	2.172977431	0
4.538782337	0.199692521	1.675700163	0.161136505	4.508343997
8.799119356	2.388588546	0	0.865625263	0
4.946252471	0	0.872291164	1.504633421	3.227800399
3.379112505	3.376526618	0.010084784	3.219875454	4.576179687
1.633458208	0.185148339	2.30590294	0.15006382	0.162222799
3.597752435	0.051459999	2.237330155	1.794973777	0.046351668
4.107768639	0.787297766	0	0.792020515	2.505198449
4.877048383	0.121435622	5.894731852	0.100396479	0.107789029
2.751336609	1.118774405	0.031776316	1.114269628	0.029085273
1.212323187	0.059263218	0.058517763	1.824857148	0.053291929
4.823892184	0.673108278	4.055522113	0.678815957	6.662583816
4.222031292	0	2.219257383	1.28439876	0
6.997410009	1.254811286	0	2.621020471	0
2.449276106	0.098681864	2.770363989	1.33431679	0.088006672
5.652629508	1.658995028	0.016386822	1.655212593	0.015043159
3.948740664	3.033538539	2.209862162	1.159145003	1.775284052
4.462510827	6.131924329	2.932373245	2.109394952	5.795968133
3.225597858	0	0.8506131	1.409164355	0
3.739760664	0.00685371	0.006776964	2.031295871	0.006232748
2.901905151	0.124575922	2.091887852	1.414574534	0.110504816
4.313354402	0.176602516	0.173844499	2.224496758	0.155001619
4.649351695	0.147898683	0.145697856	0.12126078	0.130566979
3.673046691	0	0	1.485472151	0
3.932017482	0.252449971	3.602343203	0.200519997	0.218260488
4.001900642	0.105905004	0.104444014	0.087990664	0.094306559
4.278504794	0	3.473906846	1.810104295	0
4.114323514	0.2012894	0.198019063	0.162346431	0.175795938
5.08894054	0.094169958	0.092899577	1.320038484	0.084061944
6.019645269	0	0.744761263	1.669403228	0
5.796241473	0.546101283	1.010340552	0.551882544	1.016883425
7.197571058	0.152957017	0.150661019	2.150570148	0.134893319
3.973375361	0.029590934	4.243354116	0.025206588	0.026778305
4.095941876	0	0.780666834	0	0

3.989655063	0.10327055	3.060559421	1.348747076	0.092011007
4.264069015	0	1.787815216	1.135668251	2.891694418
6.188579135	0.006448916	1.024123999	2.029463056	0.005865144
4.003095921	0.016831957	2.08048029	2.075873891	0.015273952
4.315800939	0	0	0.534821248	3.749749962
9.899946147	0.083007981	1.297672735	2.944661799	0.074271395
3.621658558	6.123000637	0.006354749	1.612509757	0.005844912
10.13174199	4.630945815	0	1.132659652	0
6.519163722	0	1.96541327	1.982146304	0
6.192019172	0.000361412	3.702411426	2.586714871	1.001334989
4.058963382	0.150250656	0.148005738	0.123098744	0.132579688
5.543161756	0	0	1.473424284	0
5.95897322	0.194287547	1.659080778	0.157032772	0.169918565
4.727143372	0	0.861924777	1.825683447	1.41984651
5.383231789	0.933331766	0.933410713	0.935253451	0.934356912
3.669399113	2.508612302	1.140954162	2.985388015	4.890045803
4.402701475	0.041554184	1.152397832	1.756335731	0.037508267
4.009134311	5.33794168	0.215921489	2.351855452	0.191083122
4.224506504	4.360698452	0	1.768371231	0
3.60669276	0	0	0.818089039	0
4.434012602	3.16830999	0.032429823	0.027926967	1.118610925
3.354132921	1.572913605	0.163440484	1.537298908	0.145998018
5.163848247	1.163602346	0.662923381	2.107771122	3.867832873
4.521839961	0.049210668	2.557365902	2.541108181	0.044346837
4.919088234	0.148273658	2.964917146	0.121553984	0.130887992
5.292244567	0	0	1.563718821	0
4.708546245	0	1.665235904	1.288161144	5.96479568
7.28402925	1.071368409	1.07124951	2.410844694	2.901769488
3.996324603	0.478782624	0.47879155	1.563325651	0.481070183
8.03335046	0	0.802265894	2.059084798	0
4.563115235	0.108060981	3.308256292	3.039237039	2.451954316
2.760302962	0	0	1.541388014	1.833291862
3.803457636	0.28208519	3.926885349	0.222127347	0.242482891
4.525898286	4.90908234	0.000388938	2.323799225	1.001452652
5.159353538	0.225352226	3.237689748	1.702336628	0.195872791
5.447724571	5.099781528	0.170147053	1.556947774	0.151805639
3.799048567	0	1.4316108	2.417238209	0.87585313
4.720853	0.013138169	1.643646234	0.011253549	0.011931583
9.267340675	0	1.596729981	2.889618261	1.909139442
7.483423152	0	0	1.723134503	0
5.460192233	0.855506032	0.855641281	0	0
2.709229558	0.080407526	0.079351641	0.067357248	1.281724649
2.955675552	0	6.697110243	0	0
7.253454562	0.019152494	1.670040637	1.068714836	0.017370999
6.704989252	0	0.671509598	1.869986088	3.197207862
6.656483971	0	0.823970976	2.089209818	0.825988071
9.136723483	1.571562476	0	3.307131067	0
5.140810929	0	0.740497074	0	6.082188857
3.562589391	0.500802305	1.294859667	1.840600048	4.165957602
5.888216307	0.799687552	0	1.141273178	0
4.817507304	4.643177373	0.010507628	2.048291147	0.009656908
2.520385848	3.001252307	2.537424066	1.346947171	3.536087878
8.267028122	0	0.880099821	0.883139891	0
12.36022249	0.887839684	0	2.436046606	0
3.981307792	0	1.518664518	2.086616031	0
4.247962252	0.013480524	0.013327208	2.061028658	4.927202037
7.912348751	4.366535107	0.021825112	1.677971587	0.020014715
3.908353199	2.031065625	0.793141531	2.302937724	0

6.198059676	0.241827356	2.948621838	0.192686317	0.209511852
5.431159446	0.991009464	0.991723557	1.684130851	0
4.328423281	0	1.350759412	0	0
3.785707793	2.047590398	0	1.473233989	0
4.471226466	0.02607375	2.940108941	1.694307779	2.119267835
3.943115834	0.093360311	1.332977465	1.950088891	0.083353297
3.272844667	1.113980436	0	2.260437909	0
7.126655041	0.419910392	1.166519594	1.89691239	0
5.91489176	0.005358054	1.020055896	2.024514232	0.004874182
6.552106365	7.112837776	0.045950705	1.163574479	0.041946415
12.13374843	0.062138954	0.061352644	3.086491881	1.220290151
3.788439332	0.036902554	0.036460043	1.130672427	0.033342674
7.613083619	4.729523984	1.2941923	0.068650381	0.073379192
3.633159669	1.031664129	2.628368444	1.030601212	0.007692149
4.391563529	0.025105613	2.711116325	2.111987086	0.022741216
5.482932642	0.0716591	0.07073447	1.871365131	0.064270201
4.992910501	0.69358306	0	1.215258947	3.531322983
3.929958255	0.041635438	0.041130988	2.181958044	0.037580957
5.259320042	0.634373483	0	1.514322803	0
3.546615536	0.677833469	0	0.683519083	0
4.420618354	0.060878425	5.26335411	2.26000258	0.054725661
2.537123793	1.093058214	0	2.419609981	0
3.366792196	0.036480914	1.134169083	1.736233468	0.032964683
4.120063385	0.038227433	1.140455967	2.494632582	0.03452996
9.521096797	0.142414897	0.140315781	2.116642545	0.125866814
6.82340323	0.018360353	1.068216553	0.015698988	0.016655385
4.229265028	0	0	3.380485302	0
3.419445469	0	0.56831966	1.955500869	0
4.465730182	0.001062794	0.001051056	1.003865079	0.000967717
3.109027859	0.26924184	0.26440524	2.48901409	0.232018464
3.856460355	0.181665315	0.178804718	2.239952462	0.159282598
5.18950565	0	1.277182816	1.677875581	2.233323603
4.545862388	1.055336245	1.055247296	2.067065698	0.013472145
3.106363315	0.107751396	0.106259772	1.362750658	0.095913948
3.843406878	0	0	1.193805926	0
3.557747165	0.017553462	0.017351933	3.254918248	0.015926193
2.861523425	0.054607444	5.565128448	1.807084966	3.42944102
3.781698573	0	0	1.527779444	0
4.831229251	0	0	2.376815681	0.847006479
4.788955667	0	0	0.660671715	0
3.548312128	0	1.386468376	1.393772833	0
4.341056377	0.307108651	0.301301329	2.587407765	0.262728373
4.74447327	0	1.463684058	1.468532088	4.763314219
3.009992773	1.309752945	1.308944477	1.294921982	0.077164725
5.210966839	0	1.939985905	2.263049297	6.001088007
5.45264626	0	0	1.464850257	0.60316365
3.385826898	0.075088397	1.270408517	1.884029012	0.067297262
5.569605946	2.627865549	1.861049043	2.274992134	0.058091618
4.607113762	2.317851788	0.184451152	1.598314504	0.164146782
10.26770131	3.462565027	0.095868214	2.998859908	0.086700468
5.544374277	7.415903691	0	2.741339827	0.815867551
4.093364895	0	0	1.256408056	0
2.024702877	0.104564643	4.588596062	1.352800231	0.093138936
5.049487325	0.198409686	0.19520068	1.628929043	3.372582086
7.387952873	1.356630409	0.098694985	3.009626251	0.089210237
3.053929285	0.095995602	0.094696034	1.9594258	0.085658977
6.459443974	3.094460571	0	2.182975927	1.783378012
2.06842743	0.115724627	0.114098705	2.02787571	0.10284114

hsa-miR-1276	hsa-miR-466	hsa-miR-517-5p	hsa-miR-522-3p	hsa-miR-518c-5p
1.374054162	0.123265701	0.13711958	0.131085487	0.137941885
0.005292299	0.007146437	0.007760076	0.007496588	0.007795541
1.400317696	0	0	0	0
0	0	3.478149772	1.810917278	3.583984509
2.391390725	0.016401716	0.017844005	0.017224027	0.017927531
1.113215564	0.034510114	0.037685221	0.03631742	0.03786984
0.155336586	4.575883414	0.272498223	0.257686382	2.950983254
0.576975315	0	0	1.033198158	0
0	0.763402862	0	0	0.768058512
1.663298745	0.730450188	0	0	0
0.085115243	0.123066604	0.136892375	0.130870677	0.137712972
0.072731378	0.10403465	0.115261016	0.110383218	0.115924322
0	0	0	0	0
0.051458933	0.072267648	0.079536811	1.885585697	0.079963132
0.970063257	0	0	0	0
0.05880681	5.215533535	4.120771114	2.368426591	3.406762011
1.238364912	5.437679154	5.733216636	3.215878429	5.214533244
0.801366185	0	0	1.326919495	0.795411223
1.416648651	0.139130474	0.155285474	0.148234864	0.156248031
0.965343283	0	0	0	0.963690508
0.252394454	0.424363716	0.504063866	0.467898789	0.50918356
1.371268458	1.414630267	0.135952089	0.129981604	0.136765633
0	0	0	0	0.887485383
2.249461589	0.211555425	0.2397854	0.227347984	0.241497825
1.833538489	0	0	0	0
1.246200459	0	1.209843538	0	0.456150869
3.046767496	1.038256992	0.011531119	0.011136179	0.011584296
1.560445014	5.243386475	0.221268578	0.210103915	0.222802885
0.038693699	0.053749671	0.058929073	0.056692633	0.059231546
0	0	0	0	0
0.088085023	4.915964915	0.142174542	0.135862644	0.143035134
2.134856239	0.033526722	8.764287815	7.476833932	8.518390522
0.044378177	0.061946352	11.13077067	9.998255278	10.57397849
0	0	2.793710072	2.301709888	3.264115992
0	0	0	0	0
1.276071889	0	0	0	1.257316939
1.319500645	2.444648035	0.114700593	0.109851479	0.115359964
1.652111333	0.017256524	0.018777284	0.018123508	1.659301214
2.186320079	0.047201322	0.051679548	0.049747413	0.051940684
0.01869406	0.02553037	0.027827675	0.026839087	0.027960987
0.858231569	0	0	0	0
0.005278808	0.007128138	0.007740176	0.007477376	0.007775549
0.090221497	0.131032404	6.956589371	5.918969186	6.958792298
0.124695526	0.186681515	0.210469912	0.200023642	0.21190393
2.102321061	6.198583997	5.841352221	4.898700626	6.282111025
0	0	0	0	0
0.172085462	7.469998614	0.308453952	0.290847533	0.310895191
2.734798612	0.111195064	0.123379141	0.118080443	0.124100256
3.387043895	4.62084131	8.71184222	7.021127161	8.088122738
0.140475651	0.213279692	0.241829171	0.229248114	0.243561732
0.0692542	0.098762896	2.432447296	0.104724546	2.77195593
0	0	0	0	0
1.402686339	0	0	0	0
0.109238641	0.161321576	0.180900365	0.172331032	0.182073242
1.702924918	5.249772298	2.964457474	0.032444169	1.108099894
0	0	0	0	0

0.075596251	5.374894689	0.120209256	0.115076109	0.120907624
0.644795093	0	0	0	0.639101216
1.611420032	0.006706876	0.007282113	0.007035126	0.007315355
2.660171585	0.017522668	2.082277588	1.661085208	2.897879877
2.75661123	0	0	0	0
1.272224399	2.985740191	11.84550715	9.915196794	10.58025967
2.614152369	0.006683694	4.285490958	2.842197502	4.124889691
1.523637121	0	0	0.631671031	0
0	0	0	0	2.210581422
0.00027936	0.000375649	0.000407338	0.000393742	0.000409167
1.466995805	0.158426226	0.177544631	0.169179936	0.178689117
0	0	0	0	1.468708867
0.136036131	0.205723133	0.232883606	0.220926619	0.234528731
2.149535932	0	0	0	0
1.510673375	0	5.672895467	4.479647062	5.672684041
3.338719049	1.141866681	0.043754949	0.042146121	0.043972226
0.031410125	0.043361953	0.047438205	0.045680321	1.151004169
2.302510094	0.233078716	0.265405934	0.251122503	0.267377667
0.827079959	0.817920092	0	0	0.821451373
1.372444596	0	0	0	1.357247863
1.112290481	0.034219001	3.501502903	0.03600983	5.398522553
0.117799016	0.175282912	0.197139709	1.582209866	1.57543789
1.875375271	0	0	0	0
1.166021997	0.051389188	0.056313569	0.054187858	0.056600995
0.106136052	0.15631211	0.175096955	0.166880445	1.517072361
2.29966452	0	0	0	0
1.691418233	0	0	0	0
1.066423447	0.019977446	0.021750176	1.671571465	0.02185292
1.300104067	0	0	0	0
1.358650704	0	0	0	0
0.078911559	0.113482137	0.125977253	0.120541718	0.126717179
1.861250519	0	1.522865845	0	0.660252067
0.189764047	4.174047611	0.348092127	0.327181703	0.351003465
0.000303984	0.000408768	0.000443254	0.000428458	0.000445244
1.662458441	1.767868949	0.272886076	0.258045121	0.274936342
0.122251979	0.182627271	2.753066675	1.604742879	2.28834167
0	0	1.831622416	0	0
1.045727472	0.013672463	6.692810861	5.285468	6.885705668
1.933982822	3.110597148	0	0	0
0.589922251	0	0	0.579266848	0
0	0	0	0	0
1.887063028	0.084218247	7.084917518	5.127141328	5.724748905
0	8.678714521	0	0	0
1.066310589	0.019942863	0.02171237	1.071328502	2.093276935
0.684098183	0	0	0	0
2.099766511	0	0	0	0
0	0.9888696	0	0	0
2.242121632	0	3.572295516	0.741689789	2.411797096
3.555379666	0	0	0	0.511282845
0.405597433	0	0	0	0
2.855757335	0.011057236	0.012016336	0.011604321	0.012071815
0	0	0	0	0.53194757
0	0	0	0	0
2.44355084	0	0	0	0
0	0.649447931	6.780189774	5.250754928	6.079899238
0.010345245	0.014029201	0.015255412	0.014728465	0.015326387
0.016866527	0.022998949	5.33810775	2.700449898	5.414408635
1.745620062	0	0	0	0

0.165638639	4.211737836	0.294434981	0.277941115	0.296718648
0	0.316122347	0	0	0
1.366978609	0.808960969	0	0	0
0.874691227	0	1.181584455	0	1.183091226
1.689351028	0.027167747	0.029622391	0.028565889	2.946220945
1.303584307	2.759084085	0.108332164	1.98467661	0.108947291
2.786546826	1.478931994	1.481617452	0.627254193	2.014678306
0	0.420901961	1.171121189	0.840614918	0.848995254
1.60697505	0.005571796	0.006048269	0.005843716	1.019793167
2.191356991	0.048567221	0.05319008	0.051195212	0.053459734
1.207357884	0.064970171	0.071396881	0.068618043	0.071773161
1.730777351	0.038490808	9.45154623	7.873114449	9.105665518
0.060677173	0.08589228	0.094800667	0.090938796	0.095324779
2.359644056	1.623342089	2.041674493	0.009234655	1.031211744
2.695721035	0.026156564	0.028513898	0.027499402	0.028650712
1.237227938	0.074992391	0.082582452	0.079296536	0.083027873
1.613323137	2.1274747	0	0	0.699717728
2.172380545	1.153707679	4.231710659	1.769758209	2.52996957
0	0	1.495618818	0	0
1.897329762	0	1.558308962	0	2.100496035
1.203367105	7.15367829	0.069920849	0.06720748	0.070288212
2.258577845	0.609110327	5.549573939	4.89977283	5.43048858
1.729184386	0.038049484	0.041580629	0.040058801	2.77343626
2.158996136	0.039877768	0.043595059	0.041992654	1.139117088
1.445336237	0.150051659	0.16786159	0.160077798	0.168925569
0.014052711	0.019116582	0.020809232	0.0200814	0.020907316
1.931067631	0	0	0	0
2.542874487	0	0	0	0
1.589352745	0.001104736	6.692923695	4.591172313	5.621111652
0.182157039	0.287136126	0.330817417	0.311375623	0.333519406
0.127960951	0.192126168	0.21686013	0.205990869	0.218353176
1.6903507	0	0	0	0
1.051573659	0.015445996	2.072669731	0.016218573	0.01687939
1.346314887	0.113153669	0.125603961	0.12018815	0.126341175
0.689368276	0.6768071	0	1.554038529	1.185395189
2.075114749	0.018275043	2.085749305	0.019195493	1.064476372
1.183387105	2.856412052	0.062595083	0.060201656	0.062918902
0.656821838	0	0	0	0.650989356
0	0	0	0	0
3.095348601	0	0	0.657075311	0
1.80288041	0	0	0	0
0.204353292	0.328704211	0.382180282	0.358242047	0.38552513
0	0	7.995119211	4.884505556	6.668019021
0.063723658	0.090442257	0.099917412	0.095807512	0.100475462
1.533897399	0	0	0	0
0.613984974	0	0	0	0
0.055766057	0.078607002	2.349785049	0.083154021	0.087099207
1.215360591	0.067637922	0.074369746	0.071458025	3.509533164
0.131660633	0.198332123	2.809577292	1.652518387	2.80884946
0.071363079	0.101956431	7.790918678	8.440086207	12.82428588
0.822775144	0	0	0	0
0.477412938	0	0	0	0
1.336937537	2.468837379	0.1217657	0.116551458	0.122475209
0.138653188	0.21017063	0.238145102	0.225822515	0.239841435
1.323600763	0.104999529	0.116353528	0.111419675	0.117024529
1.939480953	0.10069517	0.111483196	0.106797821	0.112120102
1.824144324	0	0	0	0
1.369569786	0.121619369	2.523325757	0.129309526	2.865844937

hsa-miR-20b-5p	hsa-miR-6514-5p	hsa-miR-548x-3p	hsa-miR-524-5p	hsa-miR-6844
5.315427984	2.427713002	0.083967173	0.117112956	0.079037805
7.03016627	2.029052942	0.005225368	0.006863824	0.004963785
4.105604966	1.649403515	0	0	1.140863894
3.943538266	1.83439818	0	0	0
4.735891644	1.647280716	0.011921928	0.015739323	0.011316045
5.097084372	1.110830975	0.024799179	0.033059948	0.02350292
4.870524755	1.641659257	0.152711977	1.755859104	2.28916635
4.174704713	1.43859912	0	0	0.582438443
5.325354149	0.777607465	0	0	0
4.896800423	0.745776053	2.637112487	0	2.456091575
5.19791399	0.079536795	0.083841228	0.116925947	0.078920369
6.393377921	0.068070583	0.071668272	0.099017927	0.067554631
5.896209912	0	0	0	0.954180425
5.469576835	0.048292305	0.050738207	0.068986378	1.223247411
4.03736104	0.970616273	0	0	2.284502098
4.675820634	1.254854802	0.057970695	2.357741739	0.054728772
4.774321548	1.244693897	3.58725399	2.147019471	0.719613245
8.72310723	0	0	0	0.80479319
5.14049679	0.089002928	0.093912124	0.131992536	0.088300722
6.77264918	0	0	0	0
4.828507278	0.23131876	0.247503903	0.392434799	0.229042462
6.747793567	1.361761427	0.08331959	0.116151777	2.423103014
5.764797437	2.183799412	0	0	0
6.025815997	0.12946203	0.137168627	0.19937687	0.12836515
4.168691125	0.871555873	0	0	0
3.919970072	0.463223046	0	0	0
6.210831889	2.042913129	0.007741912	1.038127267	3.952422029
6.597278775	0.12099395	0.128086965	0.18486248	0.11998336
4.67904215	1.779481853	0.038166127	0.051398124	1.168999131
6.431931064	2.315563438	2.309948562	0	0
6.131965683	0.082281377	0.08675918	0.121267794	1.374924351
6.942691815	3.308034594	1.712031157	5.563441381	1.709110759
6.794297507	1.193763915	0.043765744	7.560560822	0.04138606
6.205761421	0.690014666	0	1.851291954	0.690515846
5.125737125	1.299847244	0	0	0
4.076323826	1.281895035	0	0.733659528	1.282576371
4.983880494	0.067768562	0.07134802	0.098551226	0.067255205
6.307942863	0.011978969	0.012536441	0.016558273	1.056233837
5.650099101	1.14973717	0.033652121	0.045163982	1.149351309
6.603752725	0.0176189	0.018450178	0.024478229	0.017498927
4.753514189	0	3.389308151	0	0.860838368
4.432569181	0.004984093	0.00521205	0.006846261	0.004951141
5.349356507	1.384769535	0.088858071	3.903209964	0.083595766
6.721960301	2.166177234	0.122691876	0.17633367	0.114996684
5.711463575	0.098692538	2.8660402	3.66788528	2.084364906
3.86462648	1.491472062	0	0	1.491717747
7.773574858	0.159131006	0.16910152	0.25201746	0.157716962
4.879314383	0.072417182	0.076279439	0.105762844	0.071863589
5.656443622	1.418432858	2.805999441	6.034266009	0.860227985
5.98762444	0.130383897	0.138158176	0.200970414	0.129277509
4.585826883	1.298523075	0.068248792	0.094046381	1.922236289
4.095941404	0	0	0	0
5.76720167	2.355653646	0	0	1.046587999
6.209643488	1.461445888	0.107529846	0.152733342	1.459971178
4.628727836	2.121028476	1.101059574	0.029557026	0.021060194
6.824381217	1.735526354	0	0	0

3.989774266	1.324804236	1.331267188	0.103132623	0.070187373
4.005186999	1.155808854	0	0	0.649836517
4.037457414	2.027284327	0.004905352	0.006441913	0.004659968
5.186393985	1.057222401	1.058136859	0.016813226	0.012079643
6.642780186	1.388147301	1.016539635	0	0
4.768634926	1.887125278	0.060518401	8.751002557	1.264972947
12.29511858	2.027190989	1.611188706	3.204280165	1.022012299
7.625241436	2.088364249	3.555478894	0	0.647559236
9.110482426	0.762457954	0	0.747787144	0
6.956397206	1.001247915	0.000275869	0.000361025	0.000262215
5.153573571	1.454284689	0.105772002	0.150031972	0.099321475
8.551940069	2.689502647	0	0	0
6.354059581	0.126330271	0.133808239	0.19398309	0.125265527
5.792328978	2.82621669	0	0	2.635311749
6.020079032	1.51234629	2.240903848	3.224222172	0
4.741133405	3.333058288	0.028661757	0.038321143	3.484720375
5.939028403	0.029554104	0.030988564	0.041505282	0.029347389
6.062914531	1.628668635	0.14939475	0.219233138	0.139624899
4.50627052	0.829827477	0	0.818103868	0.830151889
6.884008264	1.376772107	2.756427254	0	0
3.990855821	0.023471433	3.952788399	1.729402197	1.109653046
5.477466954	0.109635315	0.115928473	0.165739384	0.108737439
4.73445102	1.579567091	0	0	0
3.06299705	0.034831733	0.036543139	0.049151782	0.034585272
5.456827271	2.087257313	0.104484924	0.148058597	0.098126804
4.555655103	0	0	0	0.9830603
5.640533191	0.766149135	0	0	0
5.971331032	2.079656901	3.256308846	0.019164179	1.064924228
7.879800693	0.497618346	0	0	0.498240315
5.060193635	1.762995111	0	0	2.915464391
5.745077622	1.338475232	0.077744253	0.107915331	1.337473763
7.33749489	1.183041183	0	0	1.87265811
5.194797143	0.175123209	2.451369045	0.281581037	0.173528913
7.377436784	0.000287204	0.000300185	0.000392853	0.000285327
6.582462141	0.144091629	0.152892564	0.224981454	1.64010324
6.45645459	1.513104309	2.940124579	0.172568008	0.112780194
4.118760508	0.882896793	0	0	0.883133081
7.546349187	0.009515283	0.00995542	4.241285474	1.04471435
5.958855503	1.245024453	1.628301855	0	4.183338857
2.752062744	0.594739459	0	0	0.595317095
4.890856163	2.146043528	0	0	0
5.483694722	0.055829604	0.05870432	4.205267366	0.055416703
5.237247368	0	0	0	0
4.503096503	1.064970034	0.014463291	1.071235667	1.064814068
4.887178276	2.151579264	1.200380297	0	0.688820617
6.940582864	0.83528116	0	0	0.835597057
5.026632067	0.989791162	0.989638207	0	0
5.801056904	0	3.231107548	1.263396416	0
8.74852197	0.98534301	5.716000739	0	0
5.292293877	1.893956934	0	0	1.691259501
7.394808819	1.03634151	0.008064645	0.010616041	1.627157955
7.932721642	1.377391098	0	0	0
5.659352044	0	0	0	0.888627658
7.689696863	1.461602645	2.1838654	0	4.106815945
3.650699653	0.66671901	0	4.280632261	0.667242066
7.481628882	1.638437334	0.010212849	0.01346564	0.009695801
3.708789896	1.074700482	0.016647401	1.081995836	1.074519636
5.457422594	0.805873016	0	0	1.752104633

7.090093915	0.153284142	0.162794642	0.241419478	0.151934397
7.18319186	0.3320076	0	0	0
8.110072754	0	0	0	0
5.031850519	0	0	0	1.514361042
5.675846674	1.087879578	0.019613198	0.02604411	1.087664409
5.790157586	1.920778195	0.067691099	0.093237991	1.919647188
5.115768796	0.642629398	0	1.481721761	1.148095659
7.32124985	0.438430651	0	0	1.220196034
6.354254043	0.003900239	1.018697003	0.005352251	1.018370919
6.939382872	2.185809071	0.034596679	0.046464971	3.51676546
5.203209992	2.242993506	1.8218635	0.062062527	2.242147891
6.167650189	1.123137357	0.027590976	6.239415343	0.026140182
4.688860378	0.056875965	0.059811199	0.081888333	2.639614956
7.345632438	2.03569144	1.029444422	1.031659796	0.006106071
4.84557207	1.084692779	0.018895243	0.025077137	1.084485976
7.342494835	2.276718754	0.052564495	0.071569207	0.049655787
6.811367342	1.233129233	0	0	0
4.32013098	1.138327482	3.180277139	1.152963413	0.029403243
3.546505149	0.652085513	0	0	0.652621241
6.106279741	0	0.691357766	0	2.568072739
5.896074561	1.198755663	0.04491891	0.060803746	0.042470836
3.498014432	2.625500962	1.1209727	4.928450151	0.627501796
5.01581204	0.026029169	0.027282141	1.745003807	1.725027745
4.972681763	0.027245194	0.028560459	0.038182775	1.127080443
5.373403584	1.433375113	0.100655881	0.142210457	1.432013138
5.78084854	1.062329082	0.013871317	0.018339849	0.013163305
5.605541701	1.932822025	0	0	0
5.240087842	0	0	0	0.587399414
5.937170482	1.003667269	2.326689437	3.005677535	1.589236536
5.043330523	0.168249164	0.178950426	0.268769262	0.166733128
6.691917554	0.118946543	0.125893413	0.181386434	0.117956528
5.274899662	0	0	0	3.83929147
8.685033971	0.01073598	0.011234081	0.014823539	0.01066403
5.091964234	1.337595086	0.077534115	0.107606249	2.396064951
5.603531112	1.212342388	3.453099748	0	0.694043642
6.596161757	1.059634917	0.013267774	0.017533895	0.012591473
3.286259026	0.038521724	0.040430465	1.820847145	1.789937117
3.398338071	0.661243705	0	0	0.661771598
6.858447018	0.855391341	0	0	0
6.410594601	0.672206759	0	0	0.672724851
3.907111308	0	0	0	0
6.099844852	0.188276377	0.200638883	0.306540675	0.186529112
4.791652458	0.907372096	0	4.241023477	0
6.581347361	1.275464919	0.062808629	0.086189714	0.059263293
5.807759557	2.267522532	0	0	2.267698289
5.391804688	0.618676125	0	0	0.61923775
7.766622307	0.052305407	0.054978064	0.07499354	0.051921358
6.005390337	0.045340002	0.04762136	0.064594781	0.045011895
4.716876108	0.122331118	0.129520045	0.187139654	0.121307029
7.156996687	0.066801539	0.070322758	6.814616992	1.306390898
6.673040237	1.376644617	0	0	0
5.878128531	2.373473888	0	0	0.483221076
4.124105839	1.328508306	0.075367126	0.104424641	0.071011414
5.049240121	0.128720338	0.136372609	0.198096705	0.127631093
6.844823156	1.315579794	0.072291867	0.099927336	0.068137612
5.765364448	1.303828205	0.069504583	0.09586918	0.065531236
6.228670744	1.596367233	0	0	0.952716474
5.191548586	0.078674573	1.367464194	2.061070724	1.989499172

hsa-miR-653-5p	hsa-miR-1229-3p	hsa-miR-509-3p	hsa-miR-146b-5p	hsa-miR-518e-3p
5.570608923	0.106638263	2.078324132	11.36373814	0.134380755
6.01851197	0.006367735	1.025467224	11.190286	0.007641192
5.224042213	0.37243176	1.758262021	9.643161286	0
6.123368936	3.381096561	3.104043597	11.95524323	2.954346284
3.99709304	2.886043482	3.086151206	8.958823992	0.017564149
5.319915974	1.120208281	1.732048294	12.79909546	0.037067246
1.764511075	2.405605738	4.507027021	12.08725452	3.302900624
3.690396695	2.139786321	2.293512912	9.748881262	0
3.521002574	1.297140386	1.679209848	11.783906	0.765886167
6.662712987	2.41542095	0.734810188	12.65033591	0
4.181935494	2.050137407	1.41454162	12.73730427	0.134159205
4.325160211	0.090433767	0.114956827	12.9302175	0.113049299
4.009026395	0	2.252579561	14.18244122	0
8.16104624	1.24523742	2.659282417	12.46966144	1.250529527
4.703935102	4.202603423	3.535857415	15.6645544	0
3.406649035	0.072626059	2.368645404	10.98392047	3.734189299
5.130544869	0.704978614	2.350923342	11.03924432	4.197857221
10.77746238	3.0157441	3.128680775	13.12020647	0
2.589699707	0.119891362	2.136247622	12.37040346	0.152082508
5.069895231	0.963557048	0.963546154	12.64314304	0
4.64623538	3.323231531	0.501732827	12.25605344	0.487350197
5.899421913	2.047364265	5.466182804	11.41807928	0.133242285
3.145343009	0.88669128	2.418633179	12.6650016	0
4.40870213	1.656816539	5.132976317	10.28637861	0.234112373
3.947785441	1.821166802	1.416433747	10.12179824	0
4.623623463	2.10154072	1.209253127	12.47730387	0.451750876
3.642984298	3.753271697	2.374524518	12.13325986	0.011352895
4.348887739	0.166469219	4.046980984	13.26705821	0.216180639
5.460169958	1.801890435	4.283103993	13.34510776	0.057917664
5.034182263	2.859182193	1.323597824	10.43121272	0
5.680858219	0.110345427	5.386047573	12.46714725	0.13930894
1.727291599	2.740103448	3.49797303	12.53735415	8.250842969
2.278727808	1.211700278	2.613833857	9.495157663	10.7531591
4.718184218	1.860948802	6.221730896	11.82333796	3.7691655
3.586677168	2.798430083	1.961015146	10.41879444	0
5.862898185	1.64591204	3.646637636	13.04803118	1.254885589
3.671841272	1.344067272	2.451875331	10.563406	0.112501941
5.621431213	1.060849198	2.672425365	12.70206884	1.659801412
5.64980051	0.04162516	0.051559664	10.04179008	0.050806049
4.309908863	0.022640224	3.132704812	10.29670553	0.027381229
3.349259298	1.405103271	4.562387473	12.86212321	0
4.847945941	2.032333479	2.033826212	9.463736206	1.025548179
4.087390914	0.113139268	2.90407961	11.92135037	6.621782626
4.171109214	3.538575661	1.612623972	13.7480699	0.205711832
4.190647069	1.500885072	2.196408422	12.41991593	4.313329577
5.947030262	2.468219962	0	13.26860895	0
1.851175162	0.224518915	1.856429312	12.00779099	0.300396287
6.399859884	1.367648639	1.376512628	11.58569271	0.120975662
7.231900609	1.404310591	3.348164997	12.13451379	7.492849463
3.733752825	0.180504589	0.241036393	11.96164847	0.236090058
5.363526961	1.966652703	2.432637266	11.29636119	0.107225791
4.922820977	0.746498706	2.205035955	12.43669708	0
5.473473407	2.732574679	4.045959099	14.60310051	0
5.018234624	0.138258298	2.215574356	11.8613733	0.177002722
3.159255319	0.027313855	2.739033525	10.81598475	1.717264419
5.21387427	0.7822985	3.45221089	10.73374978	0

4.791562874	0.094127062	2.023071537	13.25416708	0.117881217
4.483591941	1.128122072	3.460536527	10.71480729	0
3.62181252	3.203069743	1.614194381	11.44120969	0.007170677
8.967568829	2.403128257	4.905478793	9.822127739	2.673750329
4.354727602	0.99223621	3.087070111	11.39109437	0
5.150067712	1.292192207	1.941583928	11.5608145	9.903949624
9.566304855	2.353349666	1.614093906	13.09289178	3.844264683
7.50123516	0	5.571889663	12.13339522	0
5.568043319	1.66563316	3.055406978	12.86825685	0.750369278
3.323980212	2.323709259	1.00134363	11.30199899	0.000401205
3.901956443	1.508160152	1.525765475	12.11959878	0.173740674
4.489186768	1.470672622	0	10.68187761	0
5.813269589	0.174425079	0.232130716	12.99808872	0.227431535
6.5107824	1.820750818	0	12.42443268	0
4.064142685	3.36496806	2.709115745	12.38356828	5.725233809
4.120835155	3.891136798	3.376818422	12.86728614	0.043027877
4.69378782	2.783046192	0.047329169	15.22451811	0.046643637
4.187709546	0.196331668	1.751823897	12.19427448	0.258882786
4.742061559	0.819717142	0	13.61972211	0
4.644784873	0.815385649	1.3565373	10.86193428	0
7.168560364	2.477121664	2.487501645	11.01652036	3.175854081
4.625723406	0.149713179	1.577786963	11.64703405	0.192777283
4.734672087	2.094357808	3.029476348	10.7431235	0
7.420844539	1.792853435	2.566486935	9.787660089	0.055352351
3.716974078	1.502046224	4.190647319	14.64595276	0.171360823
5.72510793	1.563146834	4.292868847	12.89715081	0
4.227420424	1.670959707	3.909567704	10.72164784	0
5.697923039	1.070306935	0.021702985	12.28418549	0.021405997
5.512693431	0	0.488068888	11.12416089	0
3.923916316	1.740433374	4.373051526	13.14624899	0
4.562865086	2.017640103	3.340455293	11.68432984	0.123511404
4.7859503	1.830437903	9.714553335	12.28752433	0
3.517183535	1.888149659	4.040819499	12.06535649	0.338503667
5.956576461	0.000364868	1.586753529	12.5648954	0.00043658
5.241992754	0.201294678	3.306975204	14.86699855	0.266105843
6.328466522	2.68730384	2.290014153	13.5796105	1.60321634
5.624542813	1.837127054	0	13.69362957	0
5.206848358	2.061518519	2.389478204	12.51600624	6.832250275
5.368562789	2.874352451	2.351364072	10.68274573	0
4.005423482	1.709444343	2.137611524	12.99803771	0
5.567146863	0.856914981	2.59162376	13.78241333	0
5.932624314	1.914923791	2.710921681	12.47239766	6.342309141
6.343772813	0.970495181	4.122942853	13.05760091	0
4.197304264	0.017710838	4.109369336	12.77067535	0.021368818
6.291819901	1.557561446	2.790601316	10.9333411	0
3.046902782	2.339125768	1.76278758	9.774299827	0
4.230468854	1.985929273	2.306525435	13.45438507	0
4.419257964	2.618782825	2.198393478	10.31431291	1.645628986
2.607010801	1.585580546	2.934875675	8.809068405	1.569979565
4.55492087	2.941617275	0.403340119	12.98687784	0
6.883877167	0.009842375	1.039320676	11.98916439	0.011830403
6.045045969	1.62081472	2.875418232	10.35281637	0
5.684171139	0	2.628919025	13.37848227	0
7.890295116	3.538550678	5.101995081	10.59753954	0
3.722967995	1.824433091	1.149088197	11.1529823	5.861177977
6.695150151	1.049583527	2.65631925	13.9432888	0.015017578
4.651162622	0.020408728	1.081268792	11.78278177	5.081524712
5.044274007	1.334025086	2.494761197	12.80284968	0

3.368774682	1.777503327	4.429905388	14.51410891	0.286891665
4.644930611	0.315406304	3.30856	11.31625715	0
5.609361278	0.810794343	3.157831384	10.70236345	0
5.49857054	2.496796214	1.671010021	13.80857611	0.852666046
5.766944745	1.697618792	4.793717864	12.20155604	0.029145237
5.996823051	1.326563999	2.429172759	12.97809789	2.429584116
5.089877084	2.593577642	1.481237752	12.1190936	2.401660647
2.026123534	1.676855445	2.315000876	13.66709619	0
4.353392253	1.019812597	3.199905702	13.70642662	1.019986943
3.246032379	5.873802472	5.936249085	13.28654437	0.052288157
8.885593882	2.605674031	1.855592832	15.49519798	0.070139456
4.91066984	1.13369976	2.177368928	12.3898268	9.417656838
4.134148838	1.920948049	2.38012744	11.85026385	0.093051271
5.09459175	1.622175786	3.633172487	13.53771998	1.623401726
4.845489255	3.448104378	1.092273674	12.2652277	0.028055738
3.838627726	2.314279606	3.838219331	12.90216136	0.081094804
5.350236705	0.695550885	3.196538737	13.37087811	0
4.48156727	1.762136029	2.799190378	10.32185761	1.152779824
5.235778367	1.131825147	0	10.36881736	0
6.162234416	2.831983929	1.853998714	12.09443763	2.309083864
5.293048979	1.217250532	7.407089625	13.39490548	0.068693132
4.311401164	2.565939718	3.042452716	12.65549132	6.561150316
5.208913652	2.166266322	1.746688762	12.13078283	0.040892943
3.795560011	4.097442489	3.529816228	11.96669254	0.042870895
2.977937903	2.138827122	0.1673741	13.15926678	0.164323439
4.498585208	1.067318596	1.667439826	13.23596606	0.020480648
3.995448594	1.92555434	0	11.88268982	0
6.621464697	0.570057011	2.30249387	12.50921906	0
6.398759611	1.589672352	2.813175249	13.01477262	5.006304207
1.903860273	0.238812347	0.329583069	9.245817106	0.321909994
6.191169971	3.752307954	2.787542642	12.74195804	0.211907863
7.684026896	4.66355581	0	13.36871043	0
3.252018506	1.05453556	2.07271296	10.00369772	0.016538074
2.488784176	2.455448864	4.364221534	13.79369845	1.384883034
6.659928685	2.116422866	2.307784736	12.04490015	0
5.147328603	1.661453672	1.663862667	12.42359581	0.019576327
4.105501639	1.195639363	4.387551204	13.59055348	0.061512496
5.579147196	2.276086584	1.141924779	10.79660626	0
3.454441004	2.111936907	0	10.87709345	0
5.175327804	1.533785169	1.818671972	10.39869319	0
4.377532427	1.7885759	2.347409044	11.42074773	0
5.711756759	2.642502806	0.380653733	12.25808424	0.371184849
5.158578562	0	3.017784539	11.52147365	7.056080234
4.988058605	1.303172948	1.309135756	12.39517498	0.098055217
5.135164221	2.522696561	4.01504474	9.499677335	0
4.203094366	1.087328256	13.16481678	10.73451039	0
6.392571747	0.068768837	2.34995297	11.05483429	0.085054967
4.156766842	1.230251994	1.233691651	14.29136421	0.07305201
5.312875501	3.074346622	1.647960226	11.58894194	0.218984283
3.664897524	1.339161427	1.346662712	12.30728169	9.31504962
5.98953279	1.35967551	1.74875498	13.38705064	0
4.489908824	2.441985142	2.858660553	12.73962079	0.903133582
0.122940924	1.36328799	3.093620518	9.674074326	0.11940071
4.261010155	0.178005862	4.258545862	11.30428302	0.232524898
6.319999117	1.988419544	3.677867826	12.4685017	0.11411628
5.035281822	3.013837467	1.342569807	11.39997145	0.109359105
5.553165164	3.120326108	3.067226675	11.00857909	0
5.064115368	2.045264981	1.409924866	12.41862413	1.412122443

hsa-miR-518a-5p	hsa-miR-527	hsa-miR-5187-5p	hsa-miR-431-5p	hsa-miR-518a-3p
0.128472395	0.127910644	1.407699657	1.415397623	0.131896921
0.007380685	0.007355625	1.025509904	3.037947632	0.007532355
0	0	2.700288334	5.55971482	0
2.78843446	2.788632836	3.253900934	3.861915486	0.860133431
0.016951637	0.016892767	3.080372614	2.402746347	0.017308127
0.035717884	0.035588424	2.4801182	3.349407141	0.0365027
2.938272869	2.472265748	2.869370092	1.76968493	1.773828425
0	0	1.395296807	4.081248633	0
0	0	1.993005703	0.767070236	0
0	0	2.765601129	4.751163468	0
0.12826288	0.127702261	1.407091519	2.529270661	0.131680458
0.108265173	0.107809396	1.348268275	3.069433093	0.111040227
0	0	2.255415702	1.524503988	0.950390949
1.885190804	1.885052433	2.907444376	3.160094651	0.076815733
0	0	1.546927385	1.95773757	0
2.367216559	1.927223854	0.074590108	2.368717747	1.287602274
4.258457719	4.205171419	1.218752333	1.593815805	3.895564029
0	0	2.695257974	0	0
0.145188422	0.144534048	2.889416314	8.271767506	0.149181704
0	0	1.952620039	2.753244288	0
0.452956012	0.449797	2.191792208	2.292674494	0.4726242
0.127395684	0.126839746	0.108838105	3.145377959	0.130784554
0	0	2.16677986	8.511127026	0
0.22203057	0.220892766	1.663819469	0.238619658	0.229007528
0	0	2.134173482	7.414589163	0
0	0	2.646409536	2.912087195	0.449608541
0.010962532	0.010924992	2.048370272	2.050200815	0.011189777
0.205319487	0.204294867	0.171846558	0.22022369	0.211595753
0.055714848	0.05550391	3.250365848	2.577182758	0.056995115
0	0	2.499906133	2.485789014	0
0.133130953	0.13254384	1.421178762	2.890754813	0.136711115
6.908105694	6.893988667	3.160425512	1.117886251	7.729730163
9.143598901	9.037071563	0.0558387	2.279709171	10.18636223
2.302742768	2.303026685	2.107035506	2.091980678	3.266501512
0	0	3.203484101	2.21115255	0
0	0	3.49736597	5.672697032	0.735343107
0.107745744	0.107292605	1.346719507	1.351983065	0.110504652
0.017836301	0.017774231	1.657836676	1.659555283	0.018212188
0.048901936	0.048719482	2.80214695	3.411984439	0.050008875
0.02640526	0.026311542	0.023188332	2.446557058	0.026973095
0	0	0	2.58389252	0
0.007361776	0.007336781	2.619533299	1.025415935	0.007513051
4.525149727	4.423055711	1.431308689	1.440092771	6.36472672
0.195540987	0.194580522	0.164079086	0.209493105	0.20142064
4.62475187	4.623018914	2.609724396	4.188765355	4.634796695
0	0	1.889301134	0	0
0.283385624	0.281793941	0.232491874	1.856825109	0.293184302
0.115781928	0.115287497	1.370561314	3.099558826	0.118793715
6.280338065	6.265077666	0.850725058	3.568185778	6.338728471
0.223870678	0.222720138	0.186475941	1.693256137	0.23092656
0.102736461	0.102308535	1.331728073	2.772459092	1.339230981
0	0	0	4.157323926	0
0	0	2.448069128	2.262452003	0
0.16864016	0.167848282	0.142507005	3.535215449	0.173479603
1.717416102	1.717379081	1.713427369	2.964472274	0.032608248
0	0	2.489536761	2.661951844	0

0.11284854	0.112369302	0.09678667	3.31885061	0.115767258
0	0	3.32048036	2.232248901	0
0.006926477	0.006902984	1.613596967	5.323720766	0.007068655
1.661064543	1.062896784	0.015941986	2.082347525	1.661069446
0	0	0.99027559	3.260846853	0
8.611170028	8.527817998	1.29430876	9.15727989	9.080103424
3.205412737	3.205367334	2.61739761	2.031739358	3.357944008
0	0	3.113355082	9.074450572	0
0.748373856	0.748260156	1.275754988	3.493854075	0
0.000387756	0.000386462	2.001733755	1.586609105	0.000395588
0.165575694	0.164802285	1.512849965	2.664262408	0.170301364
0.904312303	0.9042766	2.192771483	2.187657832	0.904641989
0.215810147	0.214714997	2.76951805	1.670575443	0.222522905
0	0	1.417954863	3.107654962	0
4.595777151	4.539457463	1.913950525	3.068924474	4.538203552
0.041441449	0.041289328	4.281873403	1.754880411	0.042363956
0.044910703	0.044744588	2.190246759	0.047275911	0.045918275
0.245035637	0.243734708	2.400169249	1.752188671	0.253024598
0	0	1.363947621	1.361855869	0.819006646
0	0	1.754473598	2.715004844	0
3.637180454	3.63689917	2.151704413	2.48752584	1.731570869
0.18343654	0.182553369	1.56173986	1.578084456	0.188838912
0	0	1.841913282	2.467388738	0
0.053258191	0.05305761	1.794708795	4.733643647	0.054475418
0.163338974	0.16257895	2.611064873	3.892228565	0.16798221
0	0	3.144040224	4.970580762	0
0	0	1.280600899	2.429244389	0
0.020653047	0.020580705	1.669092221	6.68277496	0.021091216
0	0	1.779744964	3.311135858	0
0	0	3.029120535	7.331061099	0
0.118184598	0.11767762	0.10121812	1.384072795	0.121273269
0	0	2.472815117	1.153902851	0
0.31836446	0.316487078	0.258906493	1.950061464	0.329948356
0.000421944	0.000420535	2.586963536	3.809638237	0.000430467
0.251726673	0.25037671	0.208185354	2.952863561	0.260020316
1.604502121	1.604318583	2.249106406	3.99590426	2.750666545
0	0	2.409774212	3.263237114	0
6.265357157	6.162813294	3.389984507	4.163217448	5.630983286
0	0	0	9.078362823	0
0	0	3.627517177	5.920562025	0
0	0	2.960568606	4.158045858	0
4.418439729	4.11532498	2.353452558	3.213715914	5.516884749
0	0	1.961678969	2.280368936	0
0.020617238	0.020545028	2.089896806	7.231785424	1.071263673
0	0	1.855083169	5.96396101	0
0	0	0.824805573	3.525144253	0
0	0	1.571752207	7.647431768	0
1.261898828	1.261861446	1.266335116	4.149691779	0
0.503250907	0.502996132	1.818904193	4.256731853	0.505214256
0	0	2.74766363	3.499728371	0
0.011423176	0.011384017	3.962843791	4.762756579	0.011660235
0	0	2.913256721	0	0
0	0	1.843651125	8.091309804	0
0	0	2.170025727	10.75946354	0
4.530050035	4.402599076	3.035601477	3.117017072	5.849194003
0.014496878	0.014446821	2.387921071	0.015206879	0.014799959
3.289547365	3.118367521	2.428880917	6.572372666	3.9310493
0	0	0	1.720138497	0

0.270938194	0.269443461	1.786720784	1.823152346	0.280132621
0	0	1.978632207	2.860844778	0
0	0	1.35279174	3.395115136	0
0	0	2.359734123	2.961322517	0.431820101
1.096977636	1.097000657	2.713224176	2.454331057	1.096708088
0.101838413	0.101414966	3.603889305	3.043371392	0.104415622
2.013126011	2.01333839	2.735807261	1.771607391	0.627662072
0.423024398	0.422727739	2.475622342	6.994764123	0
0.005753719	0.005734259	2.348428201	1.609279512	0.005871487
0.050322442	0.050134111	3.033210087	3.711772609	0.051465133
0.067404944	0.067143388	2.278318722	8.248311629	0.068993545
8.210929572	8.158983806	1.134485791	3.522873174	8.446859746
0.089257687	0.088895601	2.692628143	6.000819698	0.091459753
0.009091297	0.009060303	0.008022353	1.031354434	1.031641852
0.027054242	0.026958078	2.117048861	2.121929512	0.027636915
0.077864016	0.077555304	0.067416674	2.671181831	0.079740196
0	0	2.988525806	3.196557394	0
0.04499917	0.044832696	2.785821304	2.19936814	0.046008928
0	0	1.12994279	3.465923329	0
1.55554408	0.678727975	2.32775904	1.557844425	1.182672908
0.06602275	0.065767293	1.218681675	2.892576932	0.067574174
5.91661951	5.857137958	1.463538028	3.14572439	6.648658247
1.74739294	1.747342502	2.167948137	1.746748707	0.040264889
0.041290782	0.041139264	2.994909453	1.139762708	0.042209622
0.156719811	0.155998928	1.488202053	7.540004806	0.161122109
0.019761736	0.019692658	0.017386294	3.271125344	0.02018011
0	0	0.942187511	3.369836362	0
0	0	1.692843383	2.302392207	0
3.590980593	3.590971778	1.003960057	2.327460353	4.176100785
0.303159428	0.30140864	1.864084759	0.328981558	0.313951466
0.201330446	0.200332182	0.168682613	1.629490154	2.322978037
0	0	2.947673538	4.168377379	0
0.015962677	0.01590737	2.069998999	3.784730526	0.016297577
0.117839476	0.117334306	3.662524068	2.831948211	0.120917066
1.180414937	1.180345816	0.677877964	1.852179419	0
0.018890549	0.01882465	2.082541593	4.007004338	0.019289653
0.059155697	0.058930089	2.576440104	3.459429418	0.060525285
0	0	2.059961742	1.508633781	0
0	0	0	2.575503346	0
0	0	2.640232689	2.615962783	0
0	0	3.566228029	3.20457657	0
0.348192396	0.346055941	2.659693539	0.379910163	0.361400764
6.05001443	5.907024512	1.870425604	3.429199488	6.496656558
0.094019549	0.093634537	0.081048218	1.955081621	0.096361718
0	0	1.529981335	3.513917356	0
0	0	1.085398866	3.226213578	0
0.081640217	0.081314046	2.663969999	1.909319991	0.083622947
0.070187373	0.069913444	1.231794685	2.303939073	0.071851397
1.652077847	1.651838467	0.173917928	1.648286926	2.34401775
9.106038167	9.04402822	0.091151646	6.164697584	9.629930721
0	0	2.539774563	2.526972013	0
0.466563352	0.466288788	2.435258721	2.649075395	0
0.114289131	0.113802449	0.097984285	1.372134343	0.117253446
0.220553102	0.219425485	2.332934884	3.721105648	0.227466945
0.109277577	0.108816647	0.093814024	3.973944553	0.112084181
0.104762387	0.104324313	1.977463007	1.994164604	0.107429089
0	0	2.821745006	6.414774029	0
1.413834968	1.413805731	0.108297003	1.41014353	1.413358281

hsa-miR-508-3p	hsa-miR-1295a	hsa-miR-523-3p	hsa-miR-18a-3p	hsa-miR-517c-3p
4.054142613	2.078889304	0.130209464	3.137553855	0.124144499
2.844616329	0.007436296	0.007457854	3.497598014	0.007186287
2.840094947	2.754289927	0	3.155048666	0
4.524915745	0.859782626	1.411985204	4.237376531	0
3.547862122	3.256180872	1.058824123	3.407979912	0.016495209
3.64159966	1.122613785	0.036116917	1.732451083	0.034715204
6.549648633	2.475619461	1.773601817	0.245218824	2.925146093
3.979374238	1.032625877	0	1.911072229	0
1.29624131	4.516352463	0	1.983545101	0
1.255057745	3.264866781	0	4.257166122	0
3.147321548	1.418441593	0.129996434	2.862678689	0.123943656
2.792384472	0.10927935	0.109673539	3.486405696	0.104749642
2.925978386	4.244330746	0	0	0
3.825653996	4.27177721	0.075931575	1.251919571	0.072733643
4.341766426	0	0	3.274360482	0
4.889414345	0.087122546	4.113568914	0.084747382	0.083658504
3.415443749	4.5443139	3.510479383	2.141006798	2.852289196
4.971297769	4.359762438	0	3.667714732	0
4.674153121	4.743937321	0.147213104	5.604179043	0.140151814
2.753441034	2.753526156	0.963119044	2.270844411	0
3.538276165	0.460056446	0.46284313	2.274947525	0.429084014
7.306875018	4.037829538	0.129114715	2.519837124	0.1231123
2.419411173	5.516302556	0	2.829270897	0
7.365330021	2.386035057	0.225560799	4.367034941	0.2133124
2.791621042	0	0	2.961125757	0
2.229876678	2.91669216	0.873671707	3.250092705	0
2.862466063	2.638905705	0.011078143	4.701730807	0.010671384
4.349031365	1.645011424	0.20849661	0.200627411	0.197460828
3.981133493	1.811663054	0.056365465	3.437470596	0.054082945
1.714337692	0.787928053	0	3.36438836	0
8.129406183	2.886014524	0.134946747	2.540561106	0.128608881
4.939441762	3.171083825	5.853687825	4.345987848	6.498700031
4.129281022	3.109951622	9.487725441	3.629224881	9.027807741
8.065290961	0	1.176084028	4.153184097	2.48989911
0.754889304	6.333633071	0.749599177	2.779134479	0
6.280647284	2.587129545	1.635486406	3.03711445	0
4.096407442	3.292241545	0.109145934	2.44733697	0.104250453
2.40642524	3.793106591	0.018027501	3.412363967	0.017355087
1.162952645	2.54660114	0.049464565	2.813506817	0.047489856
5.049202882	7.424855169	0.026694037	3.592510606	0.025679022
5.997844944	5.564554446	0	2.111159709	0
1.025136934	5.76782868	0.007438744	3.945483608	0.007167884
3.412757734	1.443893225	5.584240839	2.89362514	5.255176047
3.39734358	1.617114716	0.198518121	3.376428079	0.188170153
3.279067455	0.164603046	5.120366847	3.86747894	3.259453074
3.040279751	0.91458095	0	3.041922077	0
4.47516128	1.859923181	0.288335273	1.855762111	0.271234007
2.029907719	3.094335936	0.117310141	2.815790113	0.111969896
4.454331604	4.081960058	6.662046327	2.941921559	6.170136833
2.388510455	0.226439197	0.227440663	2.385393979	0.215055877
1.983434049	1.339682952	1.33961662	3.931712749	1.339297587
1.655148007	2.418800885	0	1.651460234	0
6.324369163	2.565578494	0	1.881713369	0
1.528732377	1.539134074	0.171092346	2.664801341	0.162553523
4.490664112	2.738186946	0.032266594	2.142553503	0.031024675
4.95180996	0	0	3.458646239	0

4.117953337	0.113915058	0.114329641	1.370967861	0.109153018
5.495011938	1.488078932	0	2.725871924	0
2.031627352	4.284965905	0.006998817	3.843596627	0.006744234
6.920493524	3.091140476	1.062805998	3.090356525	1.062928176
4.664002531	0	0	1.616116384	0
1.938659485	1.942579043	9.211281444	3.217059013	8.730524252
2.619090435	5.246801026	3.35782256	4.206336149	2.354864591
6.843628645	3.461749541	0	4.014820582	0
4.781206804	1.271481843	0	3.060973314	0
2.809328957	4.461532524	0.000391742	2.586883231	0.000377709
4.058935984	0.167299418	0.167970425	2.654342896	0.159629952
3.17507497	7.046178017	0	3.177220887	0
3.185106147	1.674684102	0.219207267	1.67266533	0.207415719
1.417460026	0.862036223	0	3.110937847	0
3.596585144	1.911083127	3.357619741	1.911335157	3.705851649
5.68016175	3.202700907	0.041910424	4.964729137	0.04026363
3.68605951	3.809473538	0.045422878	2.197956107	0.043624788
2.92741954	5.098019172	0.249075395	5.517528421	0.235081106
2.319105305	1.360192897	0	3.301051739	0
4.818774737	3.511348167	0	1.354703209	0
2.980522865	0.035700648	3.174653813	1.121750727	1.121725779
3.35406618	8.800042461	0.186172891	2.259111693	0.176654064
5.081963361	1.162654406	0	3.25379294	0
3.586291204	2.565505928	0.05387681	4.724177105	0.051706202
5.931136751	0.165032759	0.165692071	1.522687647	0.157495371
5.327854324	2.558639312	0	3.294482288	0
4.439465039	1.276343297	0	3.190694266	0
1.0699574	1.071536589	0.020875913	3.690300296	1.071642682
1.769560856	3.457878699	0	0.918529874	0
5.572756609	3.590421553	0	5.105355345	0
5.618995384	3.53191198	0.119751725	3.327860471	0.11427637
12.15385679	2.761076893	0	2.764252918	0
6.310271378	0.322567184	0.324210182	1.946597055	0.304061614
3.809639371	0.00042507	0.000426281	4.172171979	0.00041101
4.369263536	0.254742823	1.77455138	1.771294576	0.241401032
4.757384948	2.288789319	0.19412379	2.740474093	1.602101759
1.832670527	0	0.874386561	2.811879334	0
4.536111709	7.863821753	5.958654784	2.653753318	3.979823615
3.318700876	0.704517607	0	4.752784737	0
3.510874673	3.847501021	0	1.693170997	0
4.407671114	1.805445352	0	2.118420475	0
3.411115376	0.088293245	4.421058625	1.930697794	4.411415432
6.377615228	0.970118225	0	0.970056848	0
5.434550212	1.071414095	0.020839699	0.020284677	0.020057488
3.76442537	4.898208102	0	3.051842555	0
2.898184951	0.824244368	0	2.545047473	0
3.982680964	0.988892764	0	4.567935385	0
3.166095682	1.947853094	2.597893558	1.261839425	0.740338302
3.562624101	2.478569781	0	3.967852533	0.501692054
0.815237116	0.802049216	0	1.60473677	0
1.038908438	6.789571249	0.011543779	1.039856511	0.011119472
5.92109339	0	0	2.042704149	0
4.265228513	1.838889776	0	2.820835915	0
6.911389047	0.887986432	0	4.347372015	0
2.608189685	1.145194384	5.324770015	2.447938749	4.217623584
4.400299901	1.646091569	0.014651057	2.06596179	0.014108724
1.080407751	0.023968642	4.71005338	3.70575142	3.288175301
4.973967503	3.133645125	0	2.4971428	0

6.514400186	3.001579327	0.275584213	0.264107597	0.259518271
4.956747317	3.570634142	0	2.213341601	0
2.708705642	5.178907778	0	4.393515743	0
4.608959214	1.174856284	0.43062497	2.578235048	0
6.54562694	1.702037529	0.028410901	3.84283659	0.02732653
3.64661282	2.427925765	0.103146588	3.460654449	0.098571036
2.223576446	3.610643518	0	3.75799573	1.478677442
3.447775142	0	0	0.421804807	0.421168739
4.423843694	2.34964942	0.005813641	2.836165068	0.005602747
7.486757924	2.552784005	0.050903223	4.337261551	0.048864992
1.22231258	2.292194456	0.068212015	4.576168926	0.06538277
4.456180484	3.195286086	7.582048849	3.19345758	7.652552365
3.888754413	1.297268949	0.090375802	3.409266996	0.086461772
4.857398376	3.369017172	0.009186745	3.954433036	0.008850889
3.307057028	4.896603087	0.027350561	2.121464277	0.026309082
6.205635064	0.078550377	0.078816936	2.332306534	0.075478688
4.681352476	1.578238176	0	2.522974024	0
4.403132391	6.801539272	2.199383143	3.807272296	1.15375631
1.790036113	0	0	2.030121776	0
3.169388724	2.804276666	0.679442521	2.660205722	1.854304979
10.29776772	0.066590525	0.066810962	0.064847861	0.06404758
3.646349916	1.744557042	4.709868334	3.856932797	4.971789385
3.90267576	1.134935635	0.039835804	2.174609118	0.038277412
4.802083036	0.041627318	0.041757894	4.294855988	1.141405866
2.170904378	0.158326067	0.158951196	2.965924862	0.151175013
2.905693446	0.019915075	0.019974538	2.415100596	0.019226247
3.36993908	0.94177482	0.941801049	2.914134358	0
2.126921009	0	0	1.394029029	0
3.706580221	4.398482653	4.864195256	2.590606052	3.813338834
3.995297412	0.307077	0.308607862	3.083301333	0.289808897
3.41780386	4.666229577	1.633712985	3.118597736	0.19367214
1.280413733	2.780413263	0	4.053594541	0
2.88713675	0.016085437	0.016133037	4.475727728	0.015533844
6.249053143	2.828112529	0.119400995	3.697792371	0.113945107
2.655450222	6.625651879	1.180743775	2.310195625	0
3.557741748	2.901106997	0.019093555	4.742450478	0.018379674
6.685358188	0.059657037	0.059851646	2.257065773	0.057410638
1.145498976	2.744290803	0	2.880509437	0
1.39690018	0	0	3.34464417	0
2.895472437	2.764088289	0	3.135041741	0
2.753672252	5.093798271	0	1.384764194	0
3.605477035	2.752773847	0.354852292	3.839875001	0.331944917
4.881579044	3.930738791	7.446750517	1.462461522	4.368146196
1.951929427	2.734847846	0.095208657	3.60199526	0.091047415
5.013239875	0	0	4.321347233	0
14.60081227	1.442160899	0	1.442266176	0
3.187845715	3.184723303	0.082647158	1.909072569	0.079120537
1.863831583	1.236227464	1.867239004	3.921110268	0.068069876
4.21598524	4.037575854	0.211166917	2.336378226	1.649154176
3.059238772	1.350129731	8.540514893	3.945312927	8.212860534
3.943706129	1.747702509	0	1.748161861	0
4.722072726	0.467240614	0	3.216431273	0.464865252
4.452038329	3.318365773	0.115793314	0.112054102	0.110536519
4.403710247	0.223070206	0.224051535	5.615784259	0.211912235
6.49473797	1.360056364	0.110701918	3.958191717	0.105722504
3.484032055	0.105737079	0.106115885	2.436119424	0.10138272
4.775968453	1.526543568	0	2.893016798	0
2.520546539	4.754742408	0.128448184	2.857012482	1.412979898

hsa-miR-1283	hsa-miR-942-3p	hsa-miR-3189-3p	hsa-miR-514a-3p	hsa-miR-525-3p
0.121615018	2.031606509	0.089622678	2.872026713	0.123405116
0.00707124	1.6143256	0.00551952	2.621514361	0.007152768
0	0.782838732	0.380800022	1.571680173	0.372692288
0.859344054	0.86331269	0.865364462	3.36394668	1.811839929
0.016225354	1.056562076	1.650007764	2.402515143	0.016416566
0.034123515	2.145710495	0.02626415	3.50296576	0.034542684
0.235128099	1.699055646	0.164426034	6.702378433	2.461624622
0	1.703675643	0	3.329155466	0
0	2.260991249	0	1.680483009	0
0	0.736173004	0	0	0
0.121419187	0.096158467	1.380581388	1.411864653	0.123205742
0.102690604	0.081913223	0.076373211	0.116364104	0.104148104
0	0	0	3.50809114	0
0.071390549	1.860905391	2.282177887	3.685975876	0.072341619
0	0	3.865392031	4.341771071	0
0.082079623	0.065998931	0.061665728	4.00396622	1.925186663
1.593428851	1.612709967	1.233884075	2.536048512	1.592809345
0	3.871385329	5.355335067	3.128898033	0
0.137213287	1.436341707	0.100366301	3.446721347	0.139292472
0.963027934	2.274960663	0	0.963768628	0
0.415608389	0.296860019	0.269539684	3.906857148	0.425109786
0.120608552	1.388180755	0.088924017	5.253171379	0.122380461
0	2.173406019	0	1.445923461	0
0.208267168	0.159693056	1.611227671	5.497219785	0.21183386
0	1.423491736	0	2.791253024	0
0.871358323	0.450210401	0.454532526	1.212427097	0
0.010499143	1.036768565	0.008180503	0.011619483	0.010621196
0.192904903	0.148743857	0.137487604	3.871273951	0.196126295
0.05312192	0.043205284	3.226831754	3.737372065	0.053802586
0	0	0	0.791742996	0
0.125967204	2.816110007	0.092636206	7.449961269	0.127836604
6.666559355	0.027179785	0.025529099	5.229617303	6.242279433
8.198664058	0.049624133	0.046468221	3.482719841	8.919773955
0.673073254	0	0	7.102951239	1.175671152
0	1.284968356	1.290327291	2.212228324	0
1.253754171	2.216906133	0.742601787	5.933085048	0.733603011
0.102203158	1.333430682	0.076028759	3.968395912	0.103652354
0.017070603	1.059468475	0.013255202	1.060781288	0.017272179
0.046657701	1.158789664	1.155478682	2.215089376	0.047247137
0.025250057	2.109056615	2.69567278	2.712972574	0.025553979
0	1.409540794	1.815817824	5.879785237	0
0.007053137	2.840535691	0.005505442	1.025244963	0.007134452
4.506873648	2.056137357	0.094903018	3.41357324	5.364332489
0.183892595	0.142264359	0.131606785	2.301171729	0.186917496
3.483274267	3.403183149	1.469810089	2.193874388	4.015526479
0	1.485700469	0	2.20251729	0
0.264247796	0.198609806	0.182441711	3.408391555	0.269183193
0.109738951	0.087298064	0.081335935	0.124578441	0.111318003
5.693916225	1.810584074	0	3.66662541	6.447945196
0.209955724	0.16088928	0.148485942	4.272714945	0.213561166
0.097499757	0.077928933	0.072696787	1.984907724	0.098869504
0	1.670152696	0	1.956276351	0
0	0.552306379	1.395096096	4.980993671	0
0.15901115	1.497873492	0.115120849	1.531301342	0.161516926
1.109819137	2.945872476	0.023519125	3.159254341	0.030871764
0	1.718841526	0	4.159390286	0

0.106989597	1.347810593	0.07940411	3.849081366	0.108520906
0	0.637774455	1.821336155	4.215869683	0
0.006636377	0.005499812	2.837608064	2.031723569	0.00671281
0.017333764	2.887630223	0.013457702	6.165839953	2.081784798
0	3.163032412	0	2.780745618	0
9.172497835	0.068949701	0.064397363	2.72181421	8.239157455
1.614110111	3.353589339	4.200749774	1.613953874	2.618697211
0	1.807114041	0.639414372	5.799313986	0
0	3.097109205	0	3.752529262	0
0.00037176	0.000308885	0.000291204	2.001787852	0.000375976
0.156168457	0.122064982	0.113213466	3.725429385	0.158617108
0	1.880719274	0	2.446695972	0
0.202554618	0.15563549	0.143732314	2.834293465	0.205991383
0	0.865529567	2.625958202	0.864530834	0
3.805936642	2.236452037	0	3.59650271	3.071095894
0.039568998	2.167801221	4.496506179	4.609166027	0.040061064
0.042866675	0.035007524	0.032846281	1.767654188	0.043403689
0.229334507	3.994952593	0.160788025	1.747096102	0.23339596
0	0.822637395	0	0.821816383	0
0.813552673	2.075926061	0.820927293	4.549130524	0
1.121601183	2.960277403	3.735203754	2.754991212	1.121699695
0.172712894	2.189386908	0.124245293	2.261807918	0.1755003
0	1.858160128	1.183208047	4.459933372	0
0.050792007	1.17230473	2.53340334	2.835929556	0.051439522
0.154092535	0.120535987	0.111817421	5.339141511	0.156499751
0	0	0.982469254	5.642666397	0
0	1.679709344	1.996324864	3.978690246	0
0.019760859	2.085961309	0.015322727	0.02192092	0.019995686
0	2.190257513	1.291991884	1.538186409	0
0	0	0.809604651	5.252274596	0
0.111989703	1.362725197	1.98759783	4.889114064	0.113608151
0	2.660332661	0	10.11661395	0
0.295869449	2.965881259	2.482540956	5.288772858	0.301654479
0.000404535	1.5866556	1.001402994	3.172137129	0.000409124
0.235444456	1.699850949	0.164624164	0.276300032	0.23965399
0.179916926	0.139392636	0.128997414	4.297397478	0.182856577
0	0.877608519	0.879479229	3.263300753	0
4.159997998	1.04725527	1.639368499	3.395413645	4.658469113
0	1.228192172	1.62088514	1.595958311	0
0	1.72439347	0	2.871078569	0
0	0.859369855	0.861470217	4.288084282	0
5.316975073	0.066848187	0.062452133	2.710108525	3.991783511
0	2.76817487	0	6.719566303	0
0.019726668	1.068575899	0.015296486	4.109540749	0.019961069
0	0.677466389	4.107166623	2.645369373	0
0	0	2.764029327	0.827416267	0
0	1.572224853	0	0.989092778	0
0.740208351	0.745920857	0	3.658259846	0.740283138
0	0.962928562	0	3.997661407	0
0	0.397839321	0	1.130427261	0
0.010939811	1.630184464	1.629105834	1.039076464	0.011067122
0	1.636666087	0.999230944	5.027934681	0
0	1.849601631	0	2.986488076	0
0	1.454173012	0.892705716	5.487638333	0
3.017546349	2.301664694	1.54552877	1.518833186	4.166831198
0.013879178	0.011468177	0.010795015	4.165308883	0.014041833
1.082266947	1.078888618	0.017611582	0.025253598	2.923024168
0	1.339781883	0.800705235	3.749630142	0

0.252943963	0.190872346	0.175500994	6.055878101	0.257589033
0	2.152663233	0	4.187081978	0
0	2.545489851	0	2.708177179	0
0	1.474674235	0.436395251	2.456887498	0.846694787
1.09694124	0.022084109	2.437632288	6.644878151	0.027192965
0.096656057	1.316638253	1.3090233	2.427871169	2.759256096
1.479513605	1.501362611	1.510544557	1.483333045	1.112748097
0	1.187184877	0.859267514	2.316974559	0
0.005513386	1.607898642	1.0190357	3.937955809	0.005576713
0.048006226	1.775254028	1.15978988	6.148174683	0.048614502
0.064193348	1.215524674	2.256035709	1.223327713	0.065035671
6.502993518	2.488362318	1.12774529	3.36920675	7.309248968
0.084820964	0.068130192	1.273809364	3.419138649	0.085982666
0.008708639	1.030542017	2.360448856	3.748919177	0.008809443
0.025868969	1.690073258	0.01999564	2.121466514	0.026180787
0.07407718	1.246836597	0.055875177	4.558436763	0.075069583
0	3.792929209	4.465332761	4.602106447	0
0.042950737	2.181369953	0.032909282	3.930049442	1.153716457
0	1.515288257	2.290236651	0.642544021	0
0	0	0	1.187480028	0
0.062885641	0.050948547	0.047699872	8.220139032	0.063708515
4.195440053	2.734030165	0	3.497735507	5.43688739
0.037619895	1.735808247	1.731930908	2.174796159	0.038085679
0.03942573	1.13493797	0.030262816	3.668826523	0.039915857
0.147943919	0.115993564	0.107666821	0.169631733	0.150229814
0.018909738	1.06577882	0.014669243	2.415593696	0.019134001
0	1.927968909	2.732703941	0	0
0	3.496160927	1.060880532	1.046795679	0
3.175729647	3.17518565	1.589428655	3.591075421	4.175940487
0.282149678	0.210742848	3.91977665	3.111498736	0.287559299
0.189230484	0.14610715	0.13509564	1.625149055	0.192371222
0	0.756268529	2.976090146	0	0
0.015280278	2.066863706	1.052309193	3.404769963	0.01545995
0.111666468	0.088765643	0.082687273	5.478077662	0.11327924
0	2.130593925	0	3.70142635	0
0.018077687	2.403194195	3.667309774	2.411547325	0.018291662
0.056383339	0.045798436	0.042908027	6.141585204	0.057110917
0	1.152125031	0	1.144036375	0
0	1.804301228	2.383048133	0	0
0	1.166530345	0.664888247	2.762458242	0
0	1.394176358	0	1.782707157	0
0.322668774	1.907873797	0.217297833	0.387755079	0.329217038
4.016680117	1.468795291	0	3.928410759	5.1293833
0.089304055	1.294168224	0.06685446	1.953286726	0.090538298
0	0.955439798	0	4.014952782	0
0	2.009825441	2.235303905	14.01592954	0
0.07764067	0.062538242	1.252112702	3.386291383	0.078688514
0.066824745	2.603385756	1.218943275	0.075025408	0.067706493
0.195313013	0.150468124	0.139050992	2.808033195	1.648433549
7.380666852	1.328725945	0.074926211	2.784854612	7.444574439
0	0.818247884	2.749550005	2.057905796	0
0.464266356	1.77670219	0	4.357914123	0
0.108340006	0.086231585	1.343136593	3.523934913	0.109894713
0.206910951	1.626814021	0.146534071	3.722247928	0.210446634
0.103640527	1.337759597	0.077044009	5.784372938	0.105114248
0.099402533	2.392506596	0.074046568	2.439109632	0.100804274
0.476338799	1.276652379	0	3.895945258	0
0.119995641	1.386381007	1.376543088	2.522045575	0.1217565

hsa-miR-92a-1-5p	hsa-miR-548f-3p	hsa-miR-519a-3p	hsa-miR-3691-5p	hsa-miR-4664-5p
4.177470686	0.109860091	0.131542	0.093234809	0.092461527
2.033996468	0.006522372	0.007516724	0.005704132	1.613937059
4.94656135	0	0	1.611730566	0.787142602
4.940571257	0	1.412092074	2.399911022	1.825525194
4.614463908	0.014940578	0.01727137	2.394085649	0.012941999
3.502284047	0.031317932	0.03642171	1.116241337	1.11596898
1.773129606	0.208216591	2.945688053	0.172022639	2.348021035
2.956829013	0	0	0	2.663385557
5.029161734	0	0	0.770162745	2.265622966
4.404598024	0.731615999	0	2.433125343	1.265540695
4.048653046	0.109687025	0.13132626	0.09309247	1.38494406
5.043561493	0.09307992	0.110752892	0.079369994	0.078728986
4.444816724	0	0	0.952177608	2.742906296
2.930861904	1.246980668	0.076629971	1.857451844	0.055507648
2.760661249	0	0	0	2.766244428
3.873512615	0.074674964	2.705048537	0.064012079	1.893622291
3.593520143	0	3.895916425	0.710451316	1.231202476
3.366370107	0	0	2.304865927	2.882215141
7.169340731	0.12360673	0.148767507	2.074230402	1.429833218
2.753277956	0	0	0	0
5.453627125	0.357108874	0.47055223	0.284124888	0.280967591
3.375117861	0.108970497	0.130433346	1.383759048	0.091736854
4.400768193	0	0	1.858223587	0
6.690527986	0.185288094	0.228281153	2.279861676	0.152597853
4.944921421	0	0	0.866717925	0
3.676525815	0	0	1.953151055	2.295941859
3.056017527	0.009678046	0.011166352	2.369010125	2.045530013
3.429247153	0.172080206	0.210942862	2.237549698	1.580099246
3.862749872	0.048584805	0.056862876	1.791851208	1.791202356
3.973480973	0	0	1.730609017	3.026630035
3.771679388	0.113703253	0.136339981	0.096392159	2.037507882
4.705302759	0.030431692	6.82641209	2.955081696	1.11275772
7.122896909	0.055899801	10.17804247	1.821217049	2.248388935
5.020416299	0	3.049958166	0.680805206	0
2.210671042	0	0	0.755459163	0.755829323
4.505808952	0	1.253880093	2.437871949	0
2.452199926	0.092646211	0.110218995	1.329805453	0.078372263
4.417100011	0.01571644	0.018173429	0.013707031	1.654070813
4.336932796	0.042723185	0.049894574	0.036964287	1.156940087
6.024185554	0.023211943	0.026914521	0.020199084	2.107329326
4.281299834	0	0	1.813381564	0
4.625005811	0.006505696	1.025660242	2.030829152	1.024518817
1.442570841	0.116600642	5.804480625	0.098768094	1.407341028
4.162816921	1.594512181	0.200809299	2.212206099	0.136117168
4.429878057	0.138053656	4.728159246	1.477113949	0.115237863
3.452327758	0.915091854	0	1.486639296	0
3.68626677	0.232839527	0.292161042	0.19112402	1.756475032
3.52763293	0.09934284	0.118481758	0.084559953	1.985780467
2.58289713	0	6.397903388	0.854750644	2.607020804
4.403945533	0.186735621	0.230191902	0.155162085	0.153727534
4.491873851	1.331830172	2.770817165	1.946897502	3.397806169
2.916434785	0	0	1.282405699	0.751912136
2.563490713	0	0	1.92596568	1.682919129
4.205325175	0.142691298	0.172977074	0.119994674	0.1189496
3.483814063	0.028011177	1.109621854	0.024340721	2.128744286
3.811257936	0	0	0.786881386	0

3.317190435	0.096901727	0.115464982	0.082539209	1.976850723
4.782522343	0	0	0	1.81664054
2.842426213	0.006121757	0.007054002	1.612244867	2.028933005
3.091740323	1.658964207	1.062723138	2.074710844	1.655114103
1.851148015	0.990198425	0	2.289776956	1.364814863
5.028038045	1.294395199	9.285003982	1.90767798	1.906587362
4.35931243	0.006100628	3.357919911	3.616278037	3.490562031
4.355521545	0.630290174	0	2.274872987	1.137217085
4.896978824	0	0.749205945	0.754663334	0
4.089570278	0.000343331	0.000394781	1.5865025	0.000298771
2.206635784	1.513044036	0.169810721	0.117989412	1.482337201
5.304484451	0	0	0.907738988	1.882354886
3.46528141	0.180384773	0.221824249	0.150141337	0.148764596
3.948483735	0	0	1.424566974	4.058382622
1.503489341	0	4.893964151	1.918325264	2.237762563
4.212903227	0.036279376	0.042268732	2.165615104	2.165051417
3.220427752	0.039279124	0.045814254	0.034018868	1.144637061
7.417439684	0.203288827	0.252191918	0.168171212	1.672244916
3.875421883	0	0	0	2.562348645
4.821727859	0	0	0.819475615	0.819774197
2.487587925	0.031055616	1.121405485	2.957976929	0.026766781
6.578471246	2.226501971	0.188277527	0.129595105	0.128447305
3.949032936	0	0	0	2.115192581
5.028262593	0.046473679	0.054349704	1.170692816	1.782676345
4.540340956	0.138397629	0.167500178	0.116522085	2.895484116
3.556721016	0	0	2.29884147	2.976124325
4.171668457	0	0	2.464742704	2.972842625
4.754265808	0.018184238	0.021046029	2.409363051	2.409057137
2.788968035	0	0	2.359717789	0.937218594
4.627209281	0	0	0.808084245	1.353423136
5.518400265	0.101339474	0.120953312	1.35868088	1.357629945
4.824524703	1.826974969	0	2.313355204	0.661461976
4.840078902	0.259308772	0.32873653	1.832990535	2.498028297
4.172222994	0.000373598	0.000429589	0.000327334	0.000325107
3.820346235	0.208485391	1.774792008	0.172232449	0.170595953
2.752560971	0.160859155	1.604668025	2.200899498	1.546115765
1.830983342	0	0	0	1.439546272
2.654518784	0.012461539	5.324084158	2.058601965	2.38210997
3.319194327	1.21902943	0.705159365	1.922442671	4.425880389
2.870854164	0.57720772	0	2.941771649	1.068101365
3.472302706	0	0	0	0
2.710717504	1.28560504	5.855846729	0.064832388	0.064323705
3.125044706	0	0.970174228	2.546768523	0
5.918719031	0.01815289	0.021009506	1.068000367	0.015708829
3.616998641	0	0	2.845125756	2.982135855
4.035935747	2.080014473	0	0	0
6.026713579	0	0	2.570840276	1.572387959
2.909257624	0.741452573	1.262382817	1.667436225	2.231876795
4.299297727	0	0.504974312	3.020090458	2.917702514
2.498254466	0	0.3979495	1.420366877	2.697815655
3.868004528	0.010083428	0.011635798	2.047563328	2.37078652
1.325607824	0	0.52584296	0	0
5.418969764	0	0	1.446107303	0
4.905773116	5.043133569	0.88820995	2.65888752	2.850798378
2.606230557	0	4.947940551	1.842327612	1.541644642
4.077103644	1.049851316	0.014768712	1.048076229	0.011082876
5.171626121	1.08122399	4.372852685	2.097336551	0.018088959
3.131763654	0	0	0.799132658	2.050812872

3.88266394	0.22330981	0.27917306	2.397025727	0.181981838
3.00472721	0	0	0.321032791	0
4.729179802	0	0	1.361405705	0.815261065
1.863257382	0	0.431551056	1.715680628	1.48222571
2.126763153	0.024692244	1.096747556	0.021477804	0.021324256
4.293101134	0.087705309	1.336525889	0.07490163	0.074301992
3.363998438	0	1.113948881	3.026403108	1.506480498
3.109531516	0	0	0.427234441	0.42773527
2.026547162	0.005086909	0.00585935	0.004450668	1.019191151
4.061727001	1.783807966	0.051347134	1.77300602	3.380542642
4.86260909	0.058592186	0.06882937	3.080985377	1.212871102
3.368616612	0.03490169	8.613246512	0.030266646	2.159052336
4.130645648	1.290968685	0.091231949	1.904069151	1.27667968
3.748678464	0.008030236	0.009259563	1.620674565	1.030231541
2.941096647	1.092177159	0.027576809	1.088722088	0.02054071
3.368653017	0.067492252	0.079546207	1.24435474	2.295877299
2.96281762	0.694709388	0	2.161049156	1.603977416
3.685532688	4.016823527	0.045904681	3.189846765	1.144910699
2.418383144	0	0	0	1.520323123
3.628862453	0	0.679972335	0	2.353569029
1.221780031	0.057412253	0.067413852	0.049460658	1.208754606
4.402087106	0.60986616	5.501174747	3.11280294	0
2.773477713	0.034504663	0.040174801	1.734074962	1.12748289
5.998251459	0.03614897	0.042114777	2.165045992	2.491008849
5.673491509	0.133019859	0.160665235	2.10699808	2.10501752
3.423686645	0.017403761	0.020136966	0.015170985	1.065083565
3.610144122	0	0.941886546	2.73142958	0
2.461281401	0	0	2.173827573	0.576770848
4.465675362	1.003960817	3.591024423	2.32689939	0.00087833
4.79301692	0.247858518	0.312823347	0.202660141	0.200643351
4.571557968	0.168910756	0.206807913	0.141028088	0.139754413
4.338768491	0	0	1.990840998	3.453322073
4.944115269	0.014072798	0.016263048	2.389975589	1.05275197
4.006382205	0.101052832	0.120598262	1.357734006	0.085270566
2.933261383	0	0	1.197278662	0.684825937
7.091234475	0.016640535	0.019248499	1.65826914	0.014406897
2.259060927	0.05153696	0.060383798	0.044475147	2.230187992
3.484925627	0	0	1.532651301	2.085548169
2.102243649	0.8459033	0	2.121159316	1.404989411
5.664594849	0	0	0.662712886	1.170211782
1.385324604	0	0.838403061	1.39584131	1.396278127
4.800807873	0.281526716	0.360016892	0.228214651	1.889434028
1.462705977	0	5.593936914	1.876809706	0
5.160136618	0.081141629	0.096119367	1.291079094	0.068876552
4.68181399	0	0	0.955767999	0
3.13096099	0	0	0	0
1.271940634	0.070693273	0.083417906	0.060668477	0.060196484
3.335022774	2.289044518	1.86732497	2.599230868	0.052065676
4.049939594	0.174155205	1.652466792	1.588383227	2.68295742
5.906937991	0.091258823	9.434958313	0.077857473	1.324240263
2.5269118	0	0	2.07849768	0.819667036
4.674452038	0.901319709	0	1.989063242	2.732990988
4.119599698	1.366269196	0.116946427	1.347962592	1.981242929
6.815651577	0.184124862	0.226747193	1.61826552	1.616066986
4.324525002	4.716445857	0.111793565	0.080071278	0.079423721
2.440776497	0.090152867	0.107153018	1.321462471	1.320549678
4.24478902	0	0	1.565649367	3.162955815
4.98824955	0.108428602	1.413466485	1.381986386	1.380845524

hsa-miR-518c-3p	hsa-miR-6716-3p	hsa-miR-154-3p	hsa-miR-141-3p	hsa-miR-515-3p
0.133105264	2.016977318	5.365450687	3.580027786	0.129509647
0.007585425	0.005498997	2.621600653	2.621373225	0.007426824
0	2.158001157	4.209664863	3.394685545	0
1.810984939	1.82760823	3.104076739	3.584374607	2.123514776
0.017432943	0.012556757	0.017735126	2.076466906	0.017060046
0.036777832	1.114780663	3.177544263	2.158221258	0.035956391
0.262605072	0.163593419	1.770600566	3.30199836	0.253875645
0	1.716370634	3.058760307	3.81369799	0
0	0	1.293409787	2.801157119	0
1.932870229	1.658501882	3.555274473	3.872155097	0
0.132886331	0.089088953	3.91095458	7.445358799	0.129298035
0.112017995	0.076042169	3.828864438	4.847137225	0.10910633
0	0	2.513395852	1.525178742	0
1.885553949	0.053697536	1.249614603	3.158773325	0.075564186
0	0.969705438	3.121926424	3.857159371	0
3.207275448	0.061406193	1.927101752	1.281055996	0.086990467
4.355177198	1.234274577	3.317762862	3.102146749	2.981712405
0	0	0	4.239548394	0
0.150592427	1.42406868	7.772500462	6.182784158	0.146397197
0	2.276345655	3.528116047	1.951517902	0.963096161
0.479738267	0.267955449	0.497482091	5.601292716	0.458838082
0.131980223	2.448548681	2.075066913	4.177927041	0.128422177
0	1.454337605	10.31195915	2.638200454	0
0.231486236	2.264774971	3.490087061	3.725402966	0.224136402
0	1.42696967	6.702072628	2.791914533	0
0.450579466	2.705106018	2.817947476	8.504060036	0.873277805
0.011269309	1.627285534	2.050232813	13.17113021	0.011031651
0.213822761	2.223657261	1.641663528	3.147716659	0.20721504
0.057444551	0.040329753	1.187334811	3.864076371	0.056103649
0	0.795339964	2.987049743	3.464335029	0
0.137974796	0.092221906	2.095114378	3.598564033	0.134215159
8.078581154	1.111605951	0.036370844	2.153849316	6.409772329
9.941868053	0.046278743	1.215034427	5.084718565	10.07484263
3.049000033	1.579574827	3.047950551	3.695472307	2.486203383
0	0	1.657924647	3.831306023	0
1.254357769	0	3.646878498	2.587952374	0.734584984
0.111476695	1.324785564	0.113843269	2.448235723	0.108582023
2.08129143	1.058274985	3.090536641	4.851825032	1.061894767
0.050397282	1.155267169	2.816845398	4.05650891	0.049238181
0.027172034	1.684917936	2.119151175	5.142536118	0.026577883
0	0.856740155	2.939867714	2.773887845	0
0.007565981	1.024290023	1.616061929	0.00785657	0.007407794
6.34076941	0.094476767	0.144841521	2.900770375	5.211391685
0.203505412	0.130975487	0.20861028	4.170367387	0.197317413
5.197044107	1.468983885	1.518905111	4.190072003	4.418514815
0	1.487952117	2.461278698	2.681659399	0
0.296681618	1.743929091	1.85779339	3.691641723	0.286336119
0.119855461	1.979681425	3.099464329	4.00144691	0.116694591
7.67138113	1.815272036	4.148694501	2.36698972	6.483728543
0.233433652	0.147753184	1.694112773	5.440886567	0.226000182
1.988034517	0.072383958	3.046967445	3.475001086	0.103526111
0	2.242523228	2.417937942	3.580376416	0
0	3.012054577	3.591091018	4.465322984	0
0.175192163	3.168584676	4.338444578	5.687052644	0.170103789
1.717398352	0.023427935	1.108751693	2.738170841	0.032124411
0	0	2.260340155	3.1115242	0

0.116795979	1.338685466	0.119301361	2.018399857	0.113733095
0	1.520847085	1.490520562	11.75853164	0
0.007118402	1.022863143	4.845398073	4.125001252	0.006969728
2.673679871	1.059159766	1.660762018	4.003028135	0.018227909
0	2.610806869	4.317808687	8.825219182	0
9.677146006	1.276558186	9.578954308	4.247368266	8.628306247
3.844172034	2.028443015	0.007213717	4.430066126	2.355132449
0	0.639678968	7.711477502	3.754645385	0
0.749791098	0.756682726	5.909289013	5.183944101	0
0.000398328	2.001613213	2.586903209	4.461574688	0.000390139
0.171973289	2.120624305	2.205906182	3.007551992	0.167005078
0	0	2.856024779	5.091494038	0
0.224906647	2.249613274	1.671412476	5.271728924	0.217836719
0	1.426602206	3.243510423	3.77893913	0
5.416533594	1.919597019	4.14216163	5.249090925	3.896408286
0.042687484	0.030250982	1.755036779	4.609202231	0.041721745
0.04627172	1.143113232	3.812647926	3.024375758	0.045216807
0.255867731	0.159978543	2.460861012	4.345747603	0.24744479
0	0	4.161580478	5.088169339	0
0	1.369617552	2.71497172	3.785339943	0
3.347229902	1.113841425	1.120445837	4.508311657	2.754267105
0.190752748	0.123656846	0.195435271	3.967825532	0.185069512
0	1.565119894	3.342540643	4.804206722	0
0.054902652	1.168432613	3.586254163	2.564840342	0.05362788
0.169624703	2.114590593	4.318224676	2.651381826	0.164743541
0	2.299244689	1.975564649	3.971198795	0
0	0.760436712	2.615101638	7.587162089	0
0.021244667	0.015264422	6.112502829	3.808816656	0.020786277
0	2.650466903	2.137179897	5.110970195	0
0	0	7.158177765	2.510737241	0
0.122362304	1.986819229	2.491753082	4.468227036	0.119120477
0	0	0.65843062	10.85473428	0
1.953703264	2.480357253	0.344331627	3.513439854	0.321847711
0.000433448	1.001401358	3.702712398	5.556965275	0.000424537
0.262973366	0.163790284	0.270232001	4.030779036	0.25422706
0.198964559	0.128381403	2.753405427	5.320642251	1.604724684
0	0	1.431037606	2.810470893	0
5.03054526	0.010482954	3.982173782	2.878010168	5.535124167
0	0	8.110564778	4.2498471	0.704452367
0.580497271	1.736853029	4.41549829	10.61034753	0
0	2.397624957	4.088247743	3.855265655	0
5.237768641	2.323342211	4.678771963	4.755741975	4.326113818
0	0	0.970419934	3.413810808	0
0.021207782	0.015238285	4.969206922	0.022049954	0.020750227
0	0	4.822912579	3.449875306	0.672789649
0	1.381662277	4.663306675	5.658140062	0
0	1.986782387	8.513508646	4.374796277	0
2.596918454	0.74939163	2.761172131	3.739968651	0.741231269
0.506105219	1.326866468	0.95456125	1.578020799	0.503767819
0.399265665	2.597847293	4.194060634	4.259480225	0
0.011743204	0.008489976	1.633047536	3.381411105	0.01149528
0	1.650036065	0	2.650443112	0
0	0.885240964	7.020944687	4.390208045	0
0	2.180587833	10.14375541	3.28552296	0
5.454791059	1.16646148	2.444203088	2.447411376	4.528415582
0.014906057	1.640676428	2.391252481	5.634666186	0.014589053
4.82688298	1.675287414	6.923716442	3.824756197	4.211221692
0	1.741779974	2.847694953	3.473939442	0

0.283411164	2.378467383	2.539675308	3.890197966	0.273708087
0	2.82348403	3.415001275	5.137857916	0
0	0	3.777565233	2.521611868	0
0.432814477	2.270233074	3.123008791	3.967350572	0
1.096550171	0.020677425	2.72122968	2.453383854	1.096920532
1.984777212	1.937526896	1.334142553	4.07086218	0.102619784
2.012388344	2.275621891	3.811940212	2.710762367	0
0	0.859796386	5.297980103	1.44201687	0.423621607
0.005912689	1.019013089	3.489881037	6.098402988	1.020114122
0.051866125	0.036541834	1.790001873	5.351470402	0.05066953
0.069551672	0.048460493	9.663984613	4.588909493	0.067887182
8.709383804	1.127576008	3.001806597	3.522533646	8.261548738
0.092234593	2.329090994	4.761190154	13.510681	0.089925624
1.62344635	2.037342786	2.041737595	4.573298288	0.009148363
0.027841061	1.687291065	1.092452728	3.306891425	0.027231371
0.080399839	1.240895226	3.837868717	4.292349892	0.078433339
0.696268029	1.608012709	3.196642178	4.602046723	0
3.025134613	1.143383587	2.199478044	3.929774684	2.199255474
0	1.826300691	2.857222979	2.727869732	0
1.183166983	0.68783899	1.184976833	4.689881384	0
0.068119188	0.047504863	1.22074661	2.284484719	0.066493726
6.971434423	0.619409451	3.417719677	6.678076224	5.853769828
1.747408144	0.028789941	2.505424397	4.204353785	0.039657282
0.04253186	0.030143689	1.139958191	4.608453178	1.141301833
0.162678625	0.10717172	7.300566779	6.784923091	0.158051824
0.020326621	1.660400801	4.353459856	4.565873733	0.019888951
0	1.929894458	2.501044325	4.430438507	0
0	1.061368214	2.46139093	3.88128272	0
4.813621321	0.000852906	2.59066512	2.813146573	4.254072496
0.317809159	0.192277214	0.327325556	0.339788165	0.306406516
0.20961226	1.562100729	0.214924298	4.191472827	0.203177053
0	1.685852975	2.214436401	5.595229246	0
0.016414826	0.011831264	3.081208929	3.404586427	0.016064524
0.122002169	1.985796178	1.383539264	4.466776897	0.118772009
0	0	2.307529314	4.649693419	0
0.019429407	0.01397662	2.677507994	3.799785545	0.019011909
0.061006191	0.042734392	1.824303512	4.205848388	0.059571574
0	1.159328708	0.649126286	3.484255831	0
0	1.406820074	2.76424911	6.826605217	0
0	2.3235408	2.615871069	6.606205867	0
0	0	0.839753445	2.920618705	0
0.366138676	0.216106799	0.377864195	3.892938896	0.352159416
6.605908303	1.471394213	2.659424145	2.182861407	6.029781973
0.097186132	0.066570112	1.309729672	1.95098572	0.094729861
0	0.956236803	2.518790536	4.09728108	0
0	3.573806984	3.226331244	2.523130985	0
0.084320237	0.05821491	2.350181166	2.957695718	0.08224179
0.072436137	0.050379623	2.910618704	4.81519291	0.070692456
2.344703538	1.577561957	4.055090534	4.35798341	2.34170788
9.334335592	1.950499915	8.309597722	5.98438091	9.198520725
0	2.082256965	3.402710696	11.70488968	0
0	0	2.403787476	5.197693653	0
0.118298345	0.08000259	0.12084336	2.472976935	0.115187457
0.22992296	0.145813273	3.720389591	5.360612724	0.222640003
0.113073136	2.393124583	4.428459351	3.677243544	0.110128266
0.108368446	1.316602891	0.110655009	3.955137498	0.105570804
0.481550155	0	6.530537241	3.306786108	0
1.412869032	0.088105521	1.410699894	5.06482001	1.413815293

hsa-miR-373-3p	hsa-miR-526b-3p	hsa-miR-6715b-3p	hsa-miR-337-5p	hsa-miR-3117-3p
0.135695904	0.101805939	0.134555958	0.073539709	1.393420925
0.007698425	0.006132305	3.498340923	0.004666159	1.024931736
0	0	2.558199788	0.817474248	1.372998396
0	0	2.954320202	0.871843686	1.419662401
0.017698851	0.014030184	0.017582128	0.010627865	0.013584733
0.03736458	0.029341226	0.03710692	0.022035436	1.117801561
0.268971542	0.190422508	2.480575418	0.131663902	0.182066505
0	0	0	1.078155329	1.945587884
0	0	3.521836643	0	0.768179598
3.026074997	0	1.93310209	0.750408046	2.614902795
0.135471645	0.101647972	3.147852568	0.073431595	1.392842382
0.11411181	0.086455216	4.688216441	0.062952369	1.336762064
0	0	4.505990322	0	0
0.078797356	0.06067998	2.323849979	0.044794373	0.058551971
0	0	3.535969837	0	0
3.208588715	1.276431123	2.706162232	0.051089331	1.273270698
0	1.911374941	1.216083303	0	1.61095834
0.794074021	0	1.327797845	0	0
0.153619959	0.114329871	2.935580518	3.640317891	0.109895186
0	0	0	1.54614752	1.542240807
0.495311955	0.320474327	2.294722698	0.209211613	0.30378218
0.134543529	0.100993875	1.413995918	1.350692862	0.097168841
3.28144609	0	4.064828795	5.476630488	0.888349
2.389399253	0.16996694	0.234473884	0.118671529	0.162737678
0	0	4.68223445	0.874232014	0
0	0	3.163374188	0.469963468	1.224467533
0.011438689	0.009095151	3.642800806	0.006908933	1.628620611
0.218619535	0.158128734	2.336619713	0.111041101	0.151527474
1.187502643	0.045407483	1.811426848	0.033799674	1.18074173
0	0	2.838584785	0	0.791704718
0.140684824	0.105311927	1.431415895	0.075934848	1.406237533
4.706210247	2.961205186	2.155475878	0.021424443	0.027579545
3.112712257	6.754903826	4.571485442	0.038705001	2.850747589
0	0.676752479	2.091283497	0.695305737	1.188451561
0	0	2.922915766	0	1.283781495
2.750012783	0	3.267359047	1.994826104	0
1.352772497	0.086057526	5.379141531	0.062675878	0.082887011
0.018624209	0.014757137	5.003151722	0.011173927	1.059716876
0.051226211	0.039962161	0.050862099	0.02983557	1.771303298
0.027596075	0.021771888	1.090579056	2.097611693	1.087769221
0.852184913	0	2.110205938	0	1.408755529
0.007678687	0.006116641	2.357427314	1.022956047	2.354352999
1.44105964	3.534099612	1.4420616	0.077733389	0.103812522
0.20799313	0.15113571	1.615460004	0.106494145	1.569708042
6.480295405	3.789751482	4.824989469	0.090862796	0.122385201
0	0	1.480450201	1.494004122	0
0.304251515	0.212257625	3.050418974	1.679680488	0.20264168
0.122130106	0.092192786	0.121129491	1.947503288	4.36662546
7.026462767	4.95285385	4.841457334	0.862836251	1.407975994
0.238839896	0.171261904	3.728972578	0.119501183	1.639250197
0.108222221	0.082214204	2.433053671	0.059998011	0.079207227
0	0	1.268826431	3.47924299	0.749453547
0	0	1.012931394	0	1.38260928
0.178872632	0.131640874	0.17725165	1.446500054	1.501061173
0.033371308	0.026255486	2.143386376	0.019752048	1.105588113
0	0	1.314042128	0.797888339	0.784996433

1.368530461	0.089957601	3.317782039	0.065382519	0.086619086
0	0	2.852335755	1.852668185	1.134885994
0.007224325	0.005755979	3.035597202	2.026407909	0.005576667
1.062273863	0.014983388	3.262712539	0.011343788	5.17059415
0	0	2.889919393	1.033651217	0.999521464
5.941553696	7.159243907	3.891688487	3.901790167	2.946224626
1.023891763	2.839246302	0.007153001	0.004365692	3.201712788
3.537106023	0	1.777436839	3.085087615	4.014678634
5.779568987	0	2.209456663	0.766868172	0
1.586611744	0.000323084	2.323812634	0.00024666	1.586526635
0.175565765	0.129395749	2.664239636	0.092165033	0.124244007
0	0	3.311406651	0	0
0.230044336	0.16557682	3.701305203	0.115851544	1.619739634
0	0	3.588281673	0	1.422383513
3.70311339	3.229708989	3.069026553	1.514339258	1.916931426
0.043377661	0.033965666	0.043074545	0.025442875	1.136029375
0.047025864	0.036758528	2.798719768	1.13443766	1.756869574
0.262006715	0.186036322	2.461142659	0.128898112	0.177926322
0	0	1.361067969	0	0
0	0	2.881376353	0	1.364174212
1.120554357	0.029096548	1.731328111	0.021854664	0.028141296
0.194869466	0.142410784	6.251876315	0.100779029	1.53914506
0	0	2.284120194	0	0.6681252
0.055814665	0.04344732	2.234488575	0.03237541	1.173120617
0.173153464	0.127753871	2.199059559	0.091070674	0.122681722
0.981714174	0	0.981667459	0	2.975505167
0	0	2.429008896	0.770508738	1.287549208
7.210700278	0.017068303	2.910281792	1.658239861	0.016522661
0	0	0	1.325940193	1.561811048
0.80454125	0	2.293212643	0	0
1.384928359	0.094019888	1.385825856	1.328265054	1.364794138
0	0	1.152314845	0	0
0.343087337	0.235580731	3.512104549	0.159532128	0.224555026
0.000439793	0.000351565	0.000437009	2.001625863	1.001433841
0.26935237	0.190661609	4.857906518	0.131814361	0.182292127
4.293140396	1.567301287	3.381149674	1.496027174	4.150556523
0	0	0.875197025	0	1.437185517
1.644328438	2.06054255	6.362742677	1.04372596	2.383545348
10.34522353	0	3.824312392	3.197766843	0.708440416
0	0	1.407925002	1.470067263	1.427883084
0	0	3.233652662	1.429073943	0.858915205
1.289506674	2.942679916	2.37365875	0.051726646	2.337062544
0	0	5.23772888	0	0
0.021534166	0.017038954	1.07096379	1.063357429	0.016494287
0	0	2.644425966	1.601657777	1.56632936
0	0	0	2.371601174	0
6.815057384	0	3.6833697	2.794522514	0.989227575
0.743772747	0	3.481651569	0.75979351	1.27348954
4.667027054	0	7.427027367	0	0
0.401987413	0	1.384678354	0.417662666	0.811158056
0.011919908	0.009475568	0.011842351	0.007196349	0.009177836
0	0	0.980962815	0	0.527158361
0	0	0.880980742	1.863444454	0
0	0	0	2.673244519	3.429427789
1.809313957	2.926815878	3.859808718	1.186912655	0
0.015132059	0.012010333	1.645954757	1.044849854	2.872455177
0.024848172	2.100643507	1.081706445	2.088275334	1.678244441
0	0	1.329686877	0	1.338756216

0.290501896	0.203822916	4.087456354	0.140047537	2.418364627
0	0	1.455689472	0	1.008559235
1.742140818	0	3.281772196	0.824821104	2.733809796
0	0.429431406	1.178161342	0.8950408	2.520054012
0.029374852	0.023155482	1.701820537	0.017450204	0.022405125
12.41988134	0.081523637	0.106411768	1.287466342	0.078545832
0	0	3.2728604	0.648382695	1.499317412
0	0	5.866196484	2.593918466	0
1.019929158	0.004783673	4.031012558	0.003642775	1.608020927
0.052721979	0.041099927	1.790399151	0.030665787	0.039715449
2.627351749	0.05468357	2.627306202	3.566589791	1.216592071
3.661510426	6.288433297	1.13564764	0.024498922	1.130979548
0.093892029	0.071799557	3.220423752	1.255158752	0.069225621
1.623333091	0.007548364	4.57293513	1.617761944	1.030665301
1.092536136	0.022301205	3.840884852	0.016814479	0.021579897
0.081809941	0.062911623	1.896006626	0.046385979	1.248098202
0.697821192	0	4.431603862	0	0.698789507
2.530378506	0.036829647	2.199662488	0.027544467	0.035600199
0	0	0	0	0
0	0	1.183935694	0.699867081	0.683114652
2.287180123	0.053591203	0.068771861	0.039713531	0.051738553
3.042574594	5.189042873	0	0	2.58250329
0.041223793	0.032312242	3.785505227	0.024226574	1.73669763
0.043219281	0.033844201	1.754777826	0.025353597	1.13555176
0.166021341	0.122879199	7.151662506	2.484851144	0.118041276
0.020638795	0.016337538	1.068053388	2.073815322	1.662842523
0	0	0	0	0
0	0	4.870262468	0	1.051823192
3.465501308	3.175319822	1.589817597	0.000724954	3.464770712
0.326169715	0.225510213	2.634909824	1.715750946	0.215101233
0.214281946	0.155281928	2.323823744	0.109193654	0.148829027
0.754055136	0	0.753437537	1.309209861	0
1.054954266	0.013216917	2.663413361	0.010016435	4.160920052
0.124327132	0.093757645	3.106976317	0.068009398	0.090253461
0	0	2.307262491	0	1.876866616
0.019727172	0.015622768	0.01959646	0.011823576	1.063190129
0.06203312	0.048146598	5.347514471	1.17443322	0.046500693
0.648830849	0	1.140107348	0	0
0.846799408	0	0.846409569	0	0.848598995
0.659818063	0	1.523496905	0.677681957	1.843949777
0	0	0.839203499	0.851562481	0.841423794
0.376437149	0.255041066	4.662867523	0.171217675	0.24278955
0.901226346	4.90245226	0	3.464461746	1.874767719
0.098950094	0.075491175	1.955986494	0.055288058	1.295742666
0	0	1.528857601	1.948735975	0.955275919
0	0	0	0	0
0.085811135	0.065865997	0.085155785	0.048487265	0.063532134
2.304164173	0.056879002	2.910380665	0.04207482	1.846168555
1.649607794	1.609814392	4.208896329	1.531409742	3.537952928
6.519863541	5.191702731	1.348450895	1.921086904	1.964994608
0	0	4.018220689	0.82904459	3.195640869
0	0	5.125648882	0.48928304	0.468737124
0.120536704	0.091055923	3.696809361	0.066142807	0.087669725
0.235217973	0.168925959	1.686630507	0.118003884	2.293258933
1.357510928	0.087229893	2.798186592	0.063490634	0.084009094
0.110379599	0.083770338	1.995121961	0.061083536	0.080697388
0	0	0.919593614	2.068555809	2.180910304
0.13384201	0.100499112	0.132721438	0.072644785	3.815697551

hsa-miR-7705	hsa-miR-205-5p	hsa-miR-25-5p	hsa-miR-521	hsa-miR-6516-5p
2.499181493	6.477299063	0.079980799	0.090622517	1.380084365
2.032800356	0.007785074	0.005014207	0.005570873	0.005485179
1.090581619	0	1.85152652	0	0.79159162
0.860243004	1.812003412	1.429313344	0.865033403	1.82778555
0.015034017	0.017902877	1.647375579	0.01272369	0.012524674
2.153400533	0.037815339	0.023752307	0.026520701	0.026092719
3.488617583	4.969832701	1.642846859	0.166519668	1.674638088
1.394441214	0	0	0	1.716887354
1.295197593	0	0	0	1.703112372
2.410869352	4.788563929	0.745546484	0	1.2686542
2.056829023	7.707863499	0.079861747	0.090484851	2.450492728
1.990534364	1.352269006	1.313430631	0.077203365	0.07581953
3.513430305	0	0.954028869	0	0.95271785
1.874926129	0.079837237	1.224178379	0.05448056	1.23244771
1.958985595	1.957824475	0	0	0.969728574
3.700196182	0.092032135	1.875572481	0.062316262	1.888793819
1.599577805	8.20616102	0.718896892	1.925654822	2.181889214
3.770441678	7.029349936	1.350453091	0	1.74005403
1.456854528	2.135166007	2.041271838	0.101509152	1.42358041
1.540833298	0	0.965941535	0	0
3.700130013	3.049098282	0.232522837	0.273535712	0.266892941
1.405546949	3.377350718	0.07936863	0.089914723	2.013282386
2.645353058	0	0.893478618	0	0.89063727
0.186904012	1.685639217	0.130040933	0.149229033	0.146190173
2.611156689	0	0	0	0.86807811
1.484258206	0	0.462895239	0.453810484	0.45502874
2.636354676	5.18964343	1.034935396	0.008257102	2.044853898
2.292619825	0.222349257	0.121527208	0.139161259	2.222716077
2.23383904	0.059142239	0.036508669	0.040901086	0.040220064
2.02694241	0	0.800237732	0	1.338024328
2.514895908	2.093565117	0.082619324	0.093675672	1.39232725
1.117727243	0.036729995	1.107916524	4.608096279	1.111507271
1.835928732	5.75780527	2.826075117	6.960781521	1.201010375
0.674038721	0	0	2.13410863	1.884452471
0.749511312	1.961576524	0.763017966	0	0.757617258
2.200607935	0.738654138	1.281538917	0.742062901	1.971847637
2.427534524	4.322532524	1.31207871	0.0768546	2.387786277
2.891332069	6.326959328	2.069204481	0.013380828	0.013171216
1.164247976	0.051863587	1.757487634	0.03603818	0.035444358
2.440657523	2.446395025	3.106449517	0.019711264	0.019397988
2.117318824	0.85303575	0.860420071	0	1.413426236
0.006545573	2.62146559	1.023548414	0.005556662	0.00547119
2.083960998	0.146624622	1.385385084	3.306161417	0.094190177
1.596026396	2.302733203	2.167322416	0.133192606	1.547731516
0.139164429	0.175422096	0.099110566	2.1172714	2.112705312
1.889035668	0	1.491343448	0	1.896555222
2.505006613	4.473230784	0.159877805	0.1848321	2.404644586
1.371418325	0.123887231	0.072708963	0.082228777	0.080740553
3.863144531	2.109948566	1.418215362	2.799594804	2.130944746
2.796255477	2.393092185	0.130967832	0.150327586	0.147260902
1.971790277	2.432065245	0.065102154	0.073481175	0.072173558
1.658342071	0	0.759180222	0	0.753719011
2.591866784	0	0	0	0
2.63033198	4.853948253	0.102208043	0.11646792	0.114223694
2.464420105	13.62991834	1.099537512	0.023747424	2.452161063
0	0	0	0	0

2.780258359	1.366338328	0.071010275	0.080272425	0.078825041
0	0	0	0	0
1.023981931	5.464743187	2.349987621	0.005229431	1.022844656
2.404244091	2.082187852	1.057293776	0.013585308	1.059110106
1.342395532	0	1.682069091	0	1.009710706
3.980950751	0.09637945	2.911526939	6.30741436	0.063941494
2.030693574	5.360233621	2.027235895	1.022879026	2.61470571
2.742023114	0	0.646737977	0	0.639858712
2.792283208	3.39596716	2.003946943	0	0.75682608
2.586807452	6.97947193	0.000264848	0.000293879	2.001611364
1.514244531	5.853298888	0.100552939	0.114533631	0.112334169
1.875583589	6.217524583	1.47994827	0	0
0.181945345	7.983671503	0.126892174	0.145500758	1.595023535
2.133247905	0	1.43189566	0	1.830652426
0	0	0	3.371679367	0.936540688
1.139931503	0.043908082	1.12801773	0.03067	2.488711664
2.190831232	0.047605581	1.138252325	0.033171933	0.032628729
0.205113656	1.749554013	1.629824689	0.162823267	0.159434812
2.545715531	5.942186251	0.829658135	0	0.825441154
1.358164268	0.817037248	0.82550909	0	0
1.727360125	0.037493998	1.110070756	1.114359403	0.025876705
2.228578083	1.575978386	3.172839032	0.125723137	0.123261375
1.166390833	1.167368736	0.679664765	0	1.183921396
2.82106061	0.056516133	0.034961518	0.039151871	0.038502406
4.24145999	0.175888724	0.099340773	0.113117988	1.469483335
1.976322165	0	0	0	1.978023461
0	3.59192077	0.765932772	0	0
1.070791441	0.021822594	2.403758399	0.015468657	0.015225174
0.921409492	0	1.843433265	0	0.489501813
2.048361776	0	0	0	1.753961396
3.070110403	0.126498592	1.339000436	0.083826632	0.082304861
2.286183651	5.434275014	1.182607074	0	0.663627052
3.391089895	3.158443036	0.175965507	0.204232671	1.81409738
2.001891523	6.324308282	2.001681895	0.000319783	1.58661236
3.489582731	6.034744994	0.14475179	0.166720994	0.163230197
1.584451093	1.598391591	2.157031795	0.13054471	0.127967436
1.432822454	1.431626735	0	0	3.292002614
1.64304664	5.325490088	1.637181602	2.868651818	2.057540636
1.599962889	3.213710054	0	0	1.928431175
0.577024485	0	0.594438634	0	1.737359658
1.409917117	0	1.424715619	0	0.861701011
1.919178682	1.93024094	1.258364804	2.325622129	0.062011773
4.044322954	0	0	0	0
0.018267278	0.021784655	0.013866453	0.015442158	1.067129545
1.554192543	1.844853623	0.688055653	0	1.881894307
0.824636516	1.763236849	1.388229175	0	1.381837435
2.307151165	2.306545237	0.989779191	0	2.308320174
1.958085635	0	1.289607266	0	0
0.501402216	1.573425145	2.091577236	0.969470698	0.971603219
1.989863674	0	0	0	0.821630883
2.050512648	1.039197753	0.007736621	0.008601751	2.633829774
1.615805754	0	0.54043568	0	1.650582053
0.880620172	0	0.888282899	0	0
1.449614157	4.211562255	2.668223832	0	0.892891312
1.82009393	0.656177065	0.666446414	2.949815547	1.166756807
2.388127375	3.073876951	0.009795416	0.010896729	1.640617759
1.681747018	0.025139453	1.671712148	1.675789641	0.017498863
2.290518442	0	0	0	1.742030644

0.225377519	3.654181193	0.153996889	0.177775568	1.716702102
1.656180592	0.703465387	1.308694111	0	1.02320401
2.314508736	3.022867408	0.821108354	0	0.816725249
2.606479004	1.6724159	1.511798725	0.435673375	0.86942582
2.937795125	2.126284894	1.087992169	0.020957616	1.090865504
0.088347192	1.982610546	0.064573489	0.072874475	1.308217621
2.027994121	0.632189499	1.146761157	0	1.13531343
1.170109526	0	0	0	1.199526424
2.025621725	2.836540802	0.003913772	0.004346997	1.018997778
0.04424072	0.053380121	0.033105394	0.037055184	2.194879553
2.878560767	2.292113372	2.836725006	0.049159276	0.048326376
1.134712119	0.04221342	1.727229857	6.168355404	2.156535355
1.925302615	1.293264405	0.057098061	0.06431302	2.328641695
1.03142706	1.623168005	1.029028464	0.00685516	2.037299084
1.694686301	0.028610327	1.08480099	0.020188164	0.019866963
3.146141914	5.523625978	0.050213896	0.056457538	0.055486466
2.703830657	0	0	0	1.222740488
2.19118765	0.047699913	2.167722097	0.033235605	1.751235629
0.635265201	0	0.651806353	0	0.644976713
3.302423125	1.186525458	0	0.686988087	0.688006125
3.46557287	0.070179736	1.199034436	0.048188455	1.206220847
2.961897225	0	0.626656748	3.005407412	1.787900812
2.496168211	4.010431503	0.026124274	0.029187402	0.0287136
2.504135616	4.97224634	2.1547262	0.030561119	1.131894313
2.929941747	0.168611141	1.434089953	0.108909595	2.53283284
2.676494112	2.681581864	0.013299664	0.014808736	1.660320259
1.515123285	0.942654221	2.25269807	0	3.246324303
1.934051095	1.045539055	0	0	0
2.590382788	0.001201907	0.000778478	1.003800305	0.000850786
0.25023904	0.332719773	0.169049993	0.195875762	2.890185258
1.611489669	2.321413367	0.119468919	0.136733401	1.561388624
2.797132769	1.278621885	1.302164069	0	2.659070822
1.054902332	0.016856234	1.643814382	0.01198828	1.052201322
0.10181196	5.819969498	1.338118614	0.083597365	1.351781043
1.862329415	0.68299815	1.904788553	0	0.686912703
1.064833537	0.01995567	3.247309036	0.014163056	1.061606844
0.051886969	0.06282329	1.790925172	0.043342994	3.044785564
2.270491193	0	0.660968603	0	0
1.796400204	2.764421514	1.412686592	0	2.383635953
1.828766547	0	1.568164864	0	2.109112815
0	0	1.404293746	0	0
2.665024286	0.384534784	0.18919976	0.220297488	0.215307425
0	1.463571032	0	3.673924157	0.904759759
1.941856304	0.10031064	1.275874011	0.067567307	2.344126707
1.94067306	0	0.957486642	0	2.26501643
1.450261242	0	0	0	0
1.898518412	1.269309328	0.052507728	0.059071944	2.899799285
1.232243218	8.18885834	1.210729147	0.051109081	0.050239628
2.749940178	0.225261937	0.122871515	0.140748231	3.002891
3.026410376	2.444931924	0.067067706	5.162470433	1.319895542
2.728407475	5.641189328	0	0	2.559812074
2.794730038	0	1.817357199	0	1.269676055
2.78538583	0.122265615	0.071845348	0.081233956	2.408107364
0.18572759	4.95730368	0.129295202	0.14834555	0.145329011
3.038051366	3.678214219	1.942786272	0.077882574	0.076484731
1.978680299	4.201124089	1.30428857	0.074847691	1.316267317
0.917479812	0	0.494416552	0	1.574416111
3.10033982	4.686629752	1.991654229	0.089483375	0.087841462

hsa-miR-5589-5p	hsa-miR-493-5p	hsa-miR-92b-3p	hsa-miR-371a-5p	hsa-miR-3680-3p
5.196950089	6.813131895	6.98100175	0.126070657	0.079436038
6.484172716	5.210878154	5.398596065	0.007273183	0.0049851
1.351547121	6.685164978	6.033644843	0	0.389727605
4.723666471	5.351971432	6.585234863	0	0.869215541
5.584766021	2.892036361	5.340853473	0.016699165	1.053576243
6.321034107	5.439032467	7.301730861	0.035162956	0.023608319
2.951343979	4.641198263	5.536913843	0.245639565	1.64085809
1.675061976	5.066655222	9.417576022	0	2.198768916
5.183874321	3.318472065	6.452305326	0.763640921	0
0	5.394399398	5.369491635	0	0.745931637
5.069311352	4.694019722	5.522725704	0.125865969	0.079317921
2.453355918	6.489957817	5.854982052	0.106315414	1.938834503
5.726641789	5.378749116	5.276979961	0.950169548	1.942825255
2.659026043	3.825760008	5.187913343	0.073752689	0.048167488
5.036300697	3.535872019	4.703915331	0.968046992	0.97063917
2.979222053	3.876115622	5.187070897	2.97258028	0.054991418
5.416693234	4.446169238	6.037035914	0.703382842	1.634427114
0.795168012	1.330666468	5.348241449	0	2.063320527
2.935912878	10.21111746	3.647033263	0.142392006	0.088753572
5.188538262	6.893924756	6.640242352	0	0
6.154016546	4.440407997	4.647422668	0.439577701	0.230509349
2.073689533	4.405468791	8.592218387	0.125018724	0.078828671
4.27451316	10.70831376	4.338194056	0	0.893668802
6.788213895	5.218310384	5.78597013	0.217178964	0.129072369
0	9.596715249	5.26355306	0.862010751	0
3.30808266	6.136422618	6.527861316	0	0.463445569
2.374469153	4.146602557	6.169581657	0.010801511	0.007383885
1.638796154	4.349112677	5.616722991	0.200948386	2.62459026
1.810271504	4.371938428	4.872839507	1.189219913	0.036281036
5.789626696	4.346552927	6.33817427	0	0.800560046
5.216214803	5.091223289	6.396036162	0.130621113	2.440107174
3.344530109	2.483576636	3.636694137	2.154584066	0.022951442
0.068235787	1.212580889	5.418777812	1.217124579	1.806606331
5.712998798	5.729800315	6.179456568	0	1.208141114
5.524580073	3.903920115	6.091181341	0	0.763384206
1.256991045	7.651372135	2.898348018	0	1.282136088
7.190219099	2.449647226	2.790887452	0.10580724	0.067586306
5.0971111	5.557347552	5.770521715	0.017570112	1.650385658
2.547254748	4.056722493	5.615198737	0.048120131	1.149600556
4.785210086	5.500715878	7.334443071	0.026003435	1.082644747
4.798115042	2.584651948	4.707924155	0.850396028	0.860660746
6.085444192	3.3605169	5.564576165	0.007254554	0.004972402
6.206582949	1.435587439	4.728453046	0.134058151	0.084020673
4.771239028	4.671393699	5.267957186	0.191442422	0.115615806
0.175276341	4.048909576	5.510063496	2.988122945	2.850546833
7.789914519	4.719454229	5.217955132	0	0
0.309855039	3.932329325	4.31600528	0.276610798	0.15862853
4.94915442	5.316741233	5.369535217	1.380030455	0.072220632
4.454588034	5.364961847	4.339510921	4.221311785	2.139016122
5.142068528	5.684663326	5.50500084	0.218965026	1.583683014
5.311508142	5.364095711	6.417974497	0.100905561	0.064671417
5.470158192	6.490063455	5.96997546	0	0.75955071
0.555133986	5.752290815	7.377049787	0	1.957925408
3.736839948	7.342508162	6.936185793	0.165258392	1.460923174
5.456543446	3.973404733	4.628721289	0.031421476	1.699799088
5.153248775	4.44067023	5.776457077	0	1.338028592

3.693001197	4.344727201	4.540518176	0.110798679	0.070534933
4.638529569	4.809996213	6.848785387	0	1.536650443
5.992822789	5.562182005	4.561574273	0.006825698	0.004679964
6.143891931	3.79588708	4.683022727	0.017841294	3.073803408
2.661311718	5.507178997	7.470029772	0	1.930015111
2.384079032	10.26759946	3.893808514	4.597825957	0.057392775
0.007275949	3.205789012	7.616287955	0.0068021	2.349809453
0.636434165	9.725760112	4.558142892	0.629941981	2.658821125
3.395965548	5.323555718	5.124299873	0	1.299110552
4.646005784	5.359729846	5.04656241	0.000382201	1.586419935
6.700263521	4.200924543	4.927614605	0.16227251	0.099841386
3.848163591	5.200920586	5.091545773	0	0
5.339207837	4.635532688	5.710315222	0.211139574	0.125952038
4.474808203	5.057148535	5.998479017	0	1.43223371
4.352879749	6.157478972	7.070227775	0.933219066	1.512414816
4.861517617	3.796221596	4.538488357	0.040789485	1.732204969
6.714321551	4.626430214	6.020052874	0.044198832	0.029480743
1.749842599	5.580319889	6.274094147	0.239492167	0.140408757
3.409958699	4.0245524	5.960195248	0	1.382006902
4.645090757	4.277924055	4.153396083	0.813727658	0.825798731
0.037470205	4.710888246	8.943519896	0.034865906	2.133463153
6.044683606	2.723476369	3.96885168	0.179666497	0.109316411
3.245386352	5.338670094	6.07718744	0	0.68011161
7.931571877	4.27198677	6.509739894	0.052398817	1.162218252
6.80545036	5.45688081	4.998161389	0.16009275	1.448528951
3.293960048	3.142257341	5.099686621	0	0
2.429609702	4.58026927	7.423077699	0	0.766295752
1.670919251	7.30386368	3.91632873	2.908867536	3.08379449
2.138509058	5.212489829	8.66695892	0	1.31214514
2.293846745	8.281188166	3.488850102	0	0
4.467642881	5.784896034	5.379797803	0.116016411	0.073596309
0	1.525413619	7.131864156	0	0.670719303
3.158509794	3.803891773	5.825318565	0.310381805	0.174556596
0.000444398	4.525898289	8.131682153	0.000415899	0.000286538
5.239343943	3.828125351	6.25758907	0.24597538	0.14364736
5.816996935	5.025795174	5.384986619	0.187252678	1.512505497
6.598356475	2.810090462	3.501949583	0	0
7.985268088	3.982329373	4.935276934	0.013918805	1.044782645
3.1012211	8.897669891	5.593065114	9.085978118	1.24528672
0	7.912525568	5.60706351	0	1.085315406
5.046889981	6.133574253	6.616030221	0	0
5.344039222	5.867439104	7.087953101	0.085992383	1.878204327
5.237363194	2.542271523	5.62406911	0	1.965832041
4.918873251	7.814390897	3.104161455	0.020307582	0.013784269
0.677653057	6.266305038	4.786188347	0	2.368620728
0	5.812291797	9.536015183	0	0
1.571532665	10.02631213	4.737215645	3.790568033	0
7.465047832	4.419171991	5.437178947	0	1.290192378
3.683894422	2.479445684	5.117636903	0	1.345925706
3.753507778	6.851918448	6.058815814	0	0.411063612
4.309753852	3.968100249	5.586848082	0.011255209	2.631414277
0	2.378296395	6.460757983	0	1.377850927
4.662275657	9.317144184	5.778482871	0	1.452852379
0.889645968	10.43867628	8.722975978	0	0
4.0393156	4.876788724	6.907663635	2.264350303	1.866230449
1.049672776	4.663830133	6.619685725	0.014282186	1.638382614
6.621747539	8.479691938	6.012623872	0.023422594	0.015862054
4.823374537	5.350874338	6.932625761	0	0.806000425

4.432062782	5.669460301	5.388370181	0.264573704	1.680059037
2.323713989	5.285183294	7.45658631	0	1.310262867
5.407639869	4.384569557	4.541038557	0	0.821403851
2.572077686	5.541376294	6.885683475	0	1.513273107
2.946259678	4.735173873	8.681274277	0.027673074	0.018681896
0.108685568	4.188936573	5.456926621	10.74615509	1.295863373
3.528014434	4.695801698	6.81122643	0	1.147528976
0.431449086	7.909497849	4.672990167	0	0.438654485
4.118699588	5.760116682	5.890204238	0.005670236	1.018398319
2.553474521	4.90586677	2.552828058	0.049515485	1.153727072
1.855070301	11.50922963	4.244640041	0.066284758	1.816554039
5.569244651	3.36905686	5.38178961	1.748938022	0.026258111
3.593367224	6.991293642	6.809190232	0.0877079	0.056726324
6.75114418	4.441910829	5.858013573	1.031765985	1.618716492
6.276187943	4.313253789	4.603829186	0.026641939	1.084619579
6.772326648	4.475260474	3.838554099	0.076542259	1.231481115
1.206403916	5.595264794	6.245236834	0	3.169679897
8.534187734	4.758796065	4.138230889	0.044285762	0.029536867
0	4.568198792	5.919037723	0	1.159697949
3.703496115	3.27313161	6.30376491	0	1.600466849
5.037432833	4.332306652	3.317242322	0.064928647	0.042669451
0.61649352	3.242760617	7.305567994	1.454699677	2.272264627
4.453646703	4.730898411	6.165274253	0.0387751	0.025965063
2.513637838	3.375919164	4.972663788	0.0406414	0.02717791
0.168472686	10.5560212	4.808992765	0.153640184	0.095061472
1.667272187	6.991802885	6.258524816	0.019465507	1.062276246
0	3.369902383	5.762964387	0	2.252892955
1.04538396	3.936074371	9.829222667	0	1.446333203
5.7343228	5.006366647	5.937169364	0.001124041	1.003664321
8.498085075	3.469243538	4.682826277	0.295711715	1.742211167
4.581235728	4.034236856	3.856812756	0.197071294	0.118594881
6.187489936	4.387840964	5.456352894	0	2.484285204
5.041269407	4.841856688	5.702678194	0.015725476	2.872307192
1.381741777	2.487832423	4.888002518	0.115679	0.073398983
8.94106308	4.252037384	5.700036987	0	0.693721843
2.901715514	4.625604418	4.188335062	0.018607944	2.883840896
4.624147445	4.38831363	6.51922126	0.058189251	1.179126479
3.397895778	3.107663984	4.084131333	0	1.171448254
0.847624948	5.064798571	8.761283376	0	0
1.819390692	5.500121248	6.143953172	0	0
6.137860659	4.377485188	4.433724178	0	0
3.89476558	4.824875956	5.558528288	0.339116171	0.187655323
7.589913715	5.158456279	5.879762486	0	0
7.55909873	4.45909996	5.42223037	0.092371853	1.275188457
3.834854697	4.626742437	5.54001753	0	0.957567587
0	6.021456791	7.12913199	0	1.116535164
3.715330231	4.654565818	7.046427943	0.080243859	1.860523057
1.865619103	5.216331132	5.645664775	0.069014288	2.251745595
5.378709213	5.003546106	6.425039588	0.203490372	0.121967341
1.998046732	9.982360067	6.048021963	3.280644396	1.932604204
3.784825893	4.446429828	6.28484331	0	1.376823065
1.739530861	4.667421024	6.48061892	0	0.935521706
6.112557019	3.523581823	3.092676773	0.112207514	0.07136363
7.020717791	4.762432357	6.814225846	0.215744728	1.576618692
5.232213888	5.997734026	7.387291339	0.107305829	0.068473649
8.365064162	4.678150516	5.645957501	0.102888173	0.065852736
5.265364719	6.926111104	5.319034741	0	0.494960496
6.311766647	3.749601818	4.996671006	0.124378195	1.359734029

hsa-miR-577	hsa-miR-34b-5p	hsa-miR-203b-3p	hsa-miR-6788-3p	hsa-miR-892a
0.118054421	0.092390051	1.417593785	2.036830091	0.088082881
0.006907482	1.024576486	0.007628444	0.005992439	0.005440055
0	0	1.346728013	0.780200764	0
0	2.141249708	0	1.419208334	0
0.015841574	0.01293354	1.65616551	0.01370428	0.01241992
1.730849795	0.026972457	1.121976006	0.028635867	0.025867621
0.226853616	0.170237857	0.265014068	1.707484999	0.161215213
0	2.351788474	1.672197596	2.903926167	0
0.763453286	0	0	1.693516647	0
4.268023324	0	0	0	0
2.067402808	2.023029296	3.752726408	0.098823012	0.087949719
0.099786791	0.078669721	4.320669759	1.337904173	0.075093777
0	0	0	2.257768109	0.952834897
3.34019635	0.055467786	0.077960485	1.240318564	0.053057194
0	0	0	1.547957897	0
2.358718408	0.063464296	0.089816064	1.274142198	5.090570818
4.104866643	0.71090021	0.706095205	1.609888416	0
0.791421319	0.798008128	2.492230571	2.706467022	0.799956711
0.133083308	0.103530848	0.15174118	0.111078766	0.098607329
0.963087183	0	0	0	0
0.397198091	0.28067673	2.294664981	2.125278493	0.263445731
3.871731624	2.020462697	3.905057524	2.033522338	2.01141875
2.163595245	0	0	2.171956547	0
0.201226655	0.152466621	1.691199116	0.164661224	0.144602793
0	0.86697441	0	2.140300973	0
1.477107788	1.233095825	0.876786055	0.448814245	0.455696957
0.010254054	0.008391833	8.8084399	1.037008834	0.008061988
0.186537351	0.142128709	0.215640018	3.036960073	0.134917033
0.051760019	0.041620728	0.057809435	0.044277532	0.03986217
0.787240653	1.335481657	0	0	0
3.13947563	2.037353832	0.139003388	1.407705942	1.390876659
3.489586096	0.02621641	1.118560463	2.143257529	0.025144298
0.059614558	0.047779879	1.844463421	2.255630523	0.045735354
0	2.533935514	0	1.187596844	0
0	0	0.751024377	1.283060176	0
1.254581275	0	0	0	5.98483672
0.099315768	0.078313308	0.112267225	1.971923424	2.386001269
0.016666051	0.013601863	2.40694685	0.014413777	0.013060889
1.782216748	1.156904275	2.216205377	2.198289278	0.035132204
0.024640522	0.020041771	0.027333394	0.02125721	0.019233157
2.371123057	0	0	1.408276553	0.857153283
0.006889805	0.005646738	0.007608888	0.005977137	0.005426182
3.15209848	0.097863885	0.142719083	2.061733235	0.093256395
0.177908678	0.136003312	1.615857087	0.146558716	0.129170317
4.48995349	0.115144944	1.520832327	0.123733919	1.466596426
0	0	0	2.471402147	1.488343406
0.254553867	0.189081931	0.299543496	1.78072889	0.17877941
0.106594892	0.083806349	2.823468561	1.997402197	0.079960273
4.721362318	0	0	2.79222025	0.856529349
0.202839783	0.153595084	2.395360623	1.642043787	0.145659084
1.337039233	0.074866376	0.10700434	1.322022799	0.071487633
0	1.283093427	2.768460914	1.667630962	0
2.831929898	0	2.420401269	1.671710072	0
0.154043307	0.118853048	2.216858428	1.503026041	0.113049284
2.140413553	1.103944067	0.033049506	2.132237047	0.023166209
2.837335698	0.78725011	0	1.322738384	0

1.367936107	1.34289346	0.117632748	2.756723266	0.078066091
1.785866582	0.63997515	0	1.50793342	0
0.006482846	0.005314135	1.614314018	1.612771977	0.00510673
3.67523053	0.01380983	0.018735891	2.076421937	0.013260328
0.986773054	0	0.990053191	2.094038725	0
3.728894812	0.066286435	0.094039053	1.286031059	0.063343704
2.841166934	0.005295829	1.023974399	0.00560542	0.005089147
2.421106786	1.513011787	0	1.80322184	2.070949107
3.068470607	0.755069008	1.656023774	0	4.347663006
1.001359426	1.001299432	1.001352107	1.58653163	0.000287064
0.15131266	0.116870845	0.173335696	1.494992297	0.11118306
0	0.907925943	2.187462975	0	0
0.195767144	0.148637471	3.90169826	0.160443995	0.1410171
1.415832588	0.866670253	0	1.824626442	0
4.757057724	0.935963834	2.228617903	1.916640954	0
0.03858332	0.031197408	0.042950025	2.496661522	0.029907857
1.766799255	0.033745604	0.046558571	0.035860492	0.032343137
0.221332318	0.166436956	2.929860538	1.692602885	0.15766613
6.253688903	0.82405805	0	0.821766091	0
4.795349506	0.819802132	0	1.363599766	0
5.819129842	0.02674872	0.036687203	0.028397447	0.025653578
0.16719336	0.128341281	0.192313253	0.138159956	0.121973786
0	0.671038304	1.164366162	0	0
0.049496178	0.039838074	0.055249482	0.04237061	0.038161063
0.149318035	0.115420308	0.170963024	1.489106193	0.109817
0.981543386	0	0	2.298300097	0
1.664029233	0.758839596	11.15699748	0.755985141	1.998252551
4.273636146	0.01572545	3.808436752	1.666546796	1.66335279
0.478809502	0.937304234	9.321738444	0.930184802	0
0	0	1.340955729	3.522867556	0
0.108767832	1.992918877	0.123248292	2.774436554	0.081507063
4.949135488	3.487383274	0	1.539186511	0
0.284538764	0.20907069	0.33749072	2.981577141	0.197354463
1.001479248	0.000324901	0.000435864	1.001437213	0.000312367
1.758696813	0.170444902	1.773887604	2.376086971	0.161408479
2.269268576	2.63866176	0.20061476	0.143578981	0.126619789
0.874082735	1.844224918	0	1.840694326	0
1.049069101	1.046756331	0.014610017	3.06427498	1.639038966
2.146663092	0.711167514	0.706361614	0	0
0.576219972	0	0	1.426553156	0
0.855453948	0	5.490441623	2.131304544	0
3.856263999	1.271584073	0.091029579	3.163505272	0.061434281
0.970109388	1.551021328	0	2.545826021	0
1.071295885	0.015698495	1.071027548	2.41139635	0.015071193
0	0	0	1.185370146	1.883279515
0	0.829658842	1.368079911	1.77464397	0
1.985522031	0.989374845	2.569079433	1.572118538	0
0.740286216	0	0.742895221	1.661761798	0.750021783
2.854387303	0.967315444	0	0.505422618	0
0	0.400197735	2.382494727	1.138557838	0
0.010684175	0.008742223	0.011810468	2.048633706	0.008398331
0	5.707945205	0	0.988847856	0.533456771
1.840935671	0	1.437804195	0	0
3.155258703	1.455721522	0	0.890421599	1.45756732
2.619178604	0	0	1.533586572	0
0.013552651	2.383628002	1.64598525	3.874430591	0.010637665
1.683230191	1.676409419	5.712841655	1.079430477	0.017350814
0	1.738904649	0	0.796964938	0

0.243811087	0.181817803	1.826450352	1.752599144	0.172014807
0.689304085	1.277910398	3.071076754	0.698478225	2.309950266
3.704236353	0	1.349727212	1.755072865	0.817111667
0.846848548	0.434463427	1.443667144	1.194047577	0
0.026217409	0.021310045	1.70187542	1.09352234	1.090605842
3.620287834	0.074246548	0.106061279	3.214262387	0.070899715
0.624722666	2.055848988	0.628988877	0	1.136329056
0	0.855767352	0.427187719	1.690927586	0
3.198699433	1.607590832	2.026544544	0.004674911	0.004245211
1.170640129	3.015779686	0.052191621	1.777120775	0.036121614
1.852243604	1.212819854	0.070004972	0.053296271	0.04788887
1.136057065	0.030025527	0.041295704	1.738936219	2.155892564
0.0825029	1.276609087	0.092864356	0.069914198	0.062599257
1.623078846	3.040399215	0.009397769	1.621369294	3.209609423
3.718101701	0.020527045	0.028006649	1.090112655	0.019697966
0.072095106	0.057484896	1.259768954	0.061288636	0.054976591
4.256997605	1.604063764	2.682598667	0.698333957	0
3.383241477	1.144878023	0.046650617	0.035929736	1.142822605
3.980044802	0	3.291092077	0.639287729	0
1.857882729	1.199491284	0	2.342877543	0
0.061240579	0.049049938	0.068561817	1.213025164	0.046945635
3.931899458	0	0.613304622	2.230709336	0
0.036686711	0.029687626	1.747295566	1.737244723	0.02846444
0.038443925	1.133373984	0.042793354	2.986385115	0.029801862
0.143407605	0.111109045	2.632732318	0.119331868	0.105754845
2.08814526	1.065070046	4.011049728	0.015956034	0.0144535
1.923517431	0.944085085	2.50103429	1.517093452	0
1.684750785	1.423801385	0	1.050850065	1.062792189
2.590504042	0.000877772	0.001178098	2.32701789	2.00471593
0.271541266	0.200457299	0.32096824	0.217862505	0.189357238
1.623585777	0.139636786	0.211381554	3.296883452	0.132579862
1.968341992	0	0.753211295	0.755186003	0
0.014919632	1.646939354	1.055143636	1.053663082	0.01170255
0.108455804	0.085205552	0.122884946	2.773297334	0.081285139
2.666213108	0.684866247	0	1.572796463	0
2.084343097	0.014397448	0.019542736	0.015258549	0.013823808
0.05492798	1.188112574	5.729776344	0.04694158	0.042236376
0	0	0.647655069	0.648606811	0
1.394972253	0	1.394460528	1.802637712	0
1.153101314	0	0	0.659773646	0
0	0.843222035	0	2.584236504	0
0.309873964	0.225641637	2.031214025	0.246034472	0.21271074
2.184265083	0	0	2.672455849	1.47184638
0.086842194	0.068825712	0.097856293	1.296706944	0.065755225
0	0	2.518785795	0	0
1.445122066	0.608988043	0.605049112	2.216710778	0
1.271358592	0.060152831	0.084886826	1.886256007	0.057514224
5.085386888	0.052028554	1.235128196	0.055429497	0.049782972
3.115881783	1.586142414	1.651303001	0.155075745	0.136444425
2.432223387	0.077172572	4.506512863	1.331657961	0.07367454
1.355757766	3.910198656	0.815243123	0	0
3.954929767	0.916743499	2.40350382	1.771262658	0.475105204
0.105244044	0.082789362	1.374135065	0.088574634	0.078997115
1.675947299	0.151559145	1.687037307	3.078375475	0.143753215
1.357067605	1.965781806	0.113877461	0.084869985	0.075751658
1.343259161	1.951831127	0.109132318	1.962360796	0.07281171
3.663967771	0.484193174	0.919119496	1.555166633	0
0.116492707	0.091224821	0.132262059	0.097711969	0.086980952

hsa-miR-524-3p	hsa-miR-376b-5p	hsa-miR-517b-3p	hsa-miR-517a-3p	hsa-miR-5589-3p
0.086538945	3.099646078	0.137806029	0.137805711	5.469985715
0.005359911	0.006425425	0.007789689	0.007789675	7.190881663
0	2.107056143	0	0	0
0	1.415786214	4.356034611	4.356034683	3.682796379
0.012233939	1.653963593	1.057954509	1.057954577	3.086147055
0.025468279	1.727456504	0.037839368	0.037839297	4.889094202
0.158012005	1.733367648	5.720246887	5.720245664	0.270737038
0	1.037201984	0	0	0
0	0	0	0	4.46179571
0	1.945986798	0	0	0
0.086408503	1.405088448	0.137577399	0.137577081	6.703290895
0.073809733	2.758630061	0.115814759	0.115814502	3.673475165
0	0.950770136	0.951042673	0.951042617	4.56448882
0.052189052	1.245909347	2.322884307	2.322884511	1.249373597
0	1.547086687	0	0	4.408933887
1.262407163	0.073388562	4.749889223	4.749888983	0.091241912
0	1.601771152	5.674560077	5.674560401	5.695701299
0	0	0	0	0
0.096844928	5.94645332	0.156088975	0.156088602	3.44650434
0	3.407316154	0	0	5.18869364
0.257407163	0.347657269	0.508334406	0.508332418	5.367272773
0.085868257	2.489926349	0.136631226	0.136630911	0.13524968
0	6.153780686	0.887430615	0.887430489	4.207978286
0.141804925	2.331003418	0.241214616	0.241213953	5.131890892
0	3.966836346	0	0	0
0	1.921830579	2.609977203	2.609976922	3.162507433
0.007942483	1.037674764	1.037612791	1.037612835	2.050210888
0.132347931	0.168549939	0.222549182	0.222548588	1.641103592
0.03922773	0.047791508	0.059181611	0.059181494	1.810857839
0	1.326365904	0	0	5.449073173
0.089431135	3.115556708	0.142892946	0.142892613	5.087377939
3.95228387	0.029953961	8.707014954	8.707014716	1.728061174
7.008228023	1.212256359	12.17368299	12.17368251	0.067723371
1.199608953	2.508671865	5.130602586	5.130602866	6.114522503
0	0.750156351	1.275936333	1.275936051	4.931446081
0	3.401533602	3.031503654	3.031503577	0
0.073478517	0.090993281	0.115251051	0.115250796	6.738358939
0.01286502	1.657494773	1.060947094	1.060947166	6.345678213
0.034578704	1.163363367	0.051897577	0.051897476	2.816839556
0.018940626	1.691352671	0.027938985	0.027938934	5.229361724
0	0	0	0	4.006206964
0.005346245	0.006409001	1.025313197	1.025313226	6.500403975
2.468969859	0.114425454	7.922531792	7.922530856	6.627531016
0.126734276	0.160971181	0.211666837	0.211666282	4.439320836
4.025230434	0.135378399	6.373287592	6.373286687	0.173666839
0	1.482720855	0	0	5.504561783
0.175130138	0.227597611	0.310491157	0.310490211	0.306354485
0.078580127	1.368811881	0.123981135	0.123980856	4.550711686
3.38566536	0.851049819	9.012785232	9.012785511	4.611838228
0.142835929	2.336068629	0.243275187	0.243274516	5.054523463
0.070273817	1.968214215	3.476260075	3.476260079	5.457496899
0	3.610697864	0	0	4.377745113
0	2.289684274	0	0	1.014866438
0.110975767	2.177519063	0.181879383	0.181878929	3.912087241
0.02281072	0.027573803	2.142853384	2.142853481	4.813081241
0	0	0	0	4.879646304

0.076723532	0.095158821	0.120792263	0.120791993	3.69246026
0	1.127357104	0.638943356	0.638942991	1.490820739
0.005031556	2.030548509	0.00730987	0.007309857	3.737440556
1.058650051	0.015715485	3.262868223	3.262868233	6.228459447
0	1.871109265	0	0	3.260883642
6.38791268	3.849070133	11.25020477	11.25020409	1.298011609
0.005014234	0.00601004	4.896059094	4.896059074	1.614113942
0	3.579800872	0	0	1.122964192
0	3.080691561	0.752560849	0.752560585	4.163065918
0.000282886	2.32371629	0.000408865	0.000408864	6.509974863
0.109150477	1.510024756	0.178499956	0.178499513	5.56766828
0	1.470377093	0	0	4.844814607
0.138304577	2.313775186	0.234256672	0.234256035	7.123964705
0	2.802979469	0	0	3.865696579
0.936975225	1.505418666	7.723489515	7.723489632	5.724589558
0.029442002	1.139203479	0.043936362	0.043936278	3.530484407
0.031836657	1.150471119	0.047636492	0.0476364	5.235431956
0.154550882	0.198908649	0.267051486	0.267050722	3.804212953
0	1.761435997	1.362551747	1.362551543	3.874312027
0	0	0.817104107	0.817103906	5.243709049
0.02525773	2.477799024	3.640280239	3.640280227	0.03720681
0.119701546	0.151531509	0.198236366	0.198235857	6.856752863
0	2.305726349	0	0	2.28454887
0.037555906	1.177349193	0.056553546	0.056553435	7.35588976
0.107814243	1.503877175	0.176035132	0.176034697	4.993730212
0	0.981752511	0	0	3.293966384
0	0	0	0	2.215797134
0.014869567	2.679528272	0.021835965	0.021835925	2.420014782
0	1.986134285	0	0	1.262018508
0	2.050354252	0	0	2.040095198
0.080096052	1.375842975	0.126594947	0.126594661	4.886529381
0	0	0	0	0.658736973
0.193214335	0.253248671	0.350521424	0.350520295	4.243826729
0.000307821	0.000368126	0.000444916	0.000444915	0.00044153
0.15820046	2.411068788	0.274597143	0.274596349	4.636677208
0.124242163	0.157619167	0.206882036	0.206881498	7.307335618
0	0.875213786	0	0	6.184326303
1.638700921	2.385870829	7.570264728	7.57026464	8.533357199
0	3.245107217	0	0	1.594097755
0	2.506170221	0.584884876	0.584884469	0
0	1.41085002	0	0	4.349683456
1.265650664	1.284304263	7.197231682	7.197231141	5.829963775
0	0	0	0	4.810333256
0.014844129	2.679368986	1.070334172	1.070334255	4.109316653
0	0.673000641	0	0	1.548451179
0	2.746311473	0	0	0
0	3.154489639	0.989063595	0.989063583	2.569080756
0	1.267289002	3.739438379	3.739438415	6.412498413
0	0	1.573591346	1.573590845	3.901386415
0	1.820887894	0	0	3.191020908
0.008273742	2.050095045	0.01206266	0.012062639	2.641396728
0	0.981748419	0.985026713	0.98502619	0.530002023
0	3.994102595	0	0	3.396920169
0	4.693291209	0	0	0
3.402427901	1.524579695	6.845723651	6.845724153	2.053470092
0.010479029	1.049687692	0.015314675	0.015314647	1.645782477
0.017088039	4.020207644	5.172992468	5.172992386	4.650735118
0	2.037250364	0	0	4.118201421

0.168539435	0.218351791	0.296340749	0.296339864	5.454122711
0	1.830502998	0	0	1.642660577
0	1.353569212	1.743097624	1.743097432	5.513174746
0	2.221414429	2.038047454	2.038046995	3.197434769
0.020135994	1.697985096	4.054294341	4.054294306	2.721219319
0.069697267	0.086155321	1.982497416	1.982497758	0.107800745
0	2.031616594	2.014538909	2.01453859	4.203836566
0	3.16371563	1.172375351	1.172374727	0
0.004182846	2.025414065	0.006071254	0.006071244	5.031766293
0.035551306	0.043236621	0.05341522	0.053415116	2.553729055
0.047113647	4.887569152	0.071711034	0.071710889	1.855761935
4.557438414	1.743253221	10.17951535	10.17951506	5.380559571
0.061554925	2.959130641	0.095238225	0.095238022	1.294487308
0.006594796	1.031288452	3.04663014	3.04663015	5.372069344
0.01939805	0.023410411	0.028628132	0.028628079	6.504024642
0.05407349	2.31567246	0.082954322	0.08295415	6.83852969
0	1.588343741	0.69958222	0.699581906	1.580266441
0.031897585	0.038725524	3.221397316	3.221397345	6.32520216
0	2.047240667	0	0	0
0	0	3.62696023	3.626960252	3.056314624
0.046187179	2.271249807	0.070227558	0.070227416	4.237333388
3.129942792	0.610385951	7.05821524	7.058215812	0.615240115
0.028022475	1.13252029	4.666200741	4.666200641	5.014779282
0.029337766	0.035574304	0.043775745	0.043775661	4.213334742
0.103840095	4.533426657	0.168749732	0.16874932	2.175821603
0.01423605	2.410699256	0.02089113	0.020891092	3.100091082
0	1.515543435	0	0	0
0	0.569706375	0.57706756	0.577067147	1.397604584
2.812305349	1.58968615	6.621145403	6.621145397	5.135630542
0.185430821	0.242157679	0.33307211	0.333071063	5.231275864
0.130065116	0.165463305	0.218106307	0.218105729	6.256346305
0	1.28051235	0	0	5.506344706
0.011527602	0.013859904	0.016866444	0.016866413	3.891967811
0.079878566	0.099220987	0.126219392	0.126219106	0.12496731
0	0	0.683107526	0.683107198	8.515563325
0.013616133	0.016387303	0.019967844	0.019967808	4.006983995
0.041560742	2.244950667	0.062865441	0.062865316	2.862506388
0	0	0	0	4.899535442
0	1.797754871	0	0	0.847080436
0	1.532713049	0	0	1.155950605
0	2.10095907	0	0	4.776820665
0.208150318	3.103939577	0.384971107	0.38496981	5.276302537
3.040733195	3.027953227	7.549162962	7.549163132	5.944836883
0.064651304	0.079725849	0.100383299	0.100383083	5.751827212
0	2.522442082	0	0	2.257780728
0	1.086544611	0	0	0
1.249156365	1.895789513	0.087021411	0.087021229	2.686860561
0.048973901	2.287005022	1.865457784	1.865458032	3.93085842
0.133839622	1.623221648	3.442466283	3.442466307	3.44187551
5.565806305	3.443500333	10.18582853	10.18582773	3.06061065
0	0	0	0	3.288818067
0	0.902520772	0.474842048	0.47484157	2.403946729
0.07763611	0.096332552	0.122358007	0.122357732	5.50723884
0.140975638	0.18027397	0.239560892	0.239560235	3.720909332
0.074454712	1.349662094	0.116913694	0.116913434	6.28954653
0.071572206	0.088552091	0.112014903	0.112014657	7.284689851
0	2.714936545	0	0	5.79764894
0.085459483	1.400695003	3.906079148	3.906079023	5.462799296

hsa-miR-410-3p	hsa-miR-520d-5p	hsa-miR-301b-5p	hsa-miR-412-5p	hsa-miR-6720-3p
7.210318818	0.097048945	0.093979842	2.871717053	2.411528049
5.626140467	0.005896366	2.353901511	5.170196102	0.004664691
7.872193544	0	1.809301662	5.986984783	0.396370137
6.240879037	0	3.266991315	2.124384284	0
3.256814305	0.013480583	0.013121199	3.789924964	0.010624474
6.192603274	0.028152403	0.027376854	4.357121288	1.107964318
5.665834206	0.180144376	0.173600913	4.640906841	0.131610619
6.277709823	0	3.029876369	5.631091449	0
3.78047163	0	0	0.768777191	0
7.306174492	0	4.068547509	5.764006607	0
3.146721558	0.096899707	0.093836162	1.411315612	0.073404804
5.28459409	0.082527386	0.079987306	6.025420707	1.303620438
6.027304398	0	1.527967733	4.505449099	0
5.023511664	0.058056576	2.288124553	4.624238037	0.044778949
4.534178602	0	0	4.993173515	0
5.683372061	1.2724982	0.064494713	4.330727157	0.051071506
4.659848689	2.57328468	1.229840279	4.914215722	0
2.026708118	0	2.30405315	3.128998169	0.808250006
9.747157012	0.10886793	1.432448629	8.588883055	0.082026759
7.270496545	0	0	5.110481749	0.966759509
4.989869213	0.29998654	0.287185216	0.514344555	0.209116696
4.601474352	0.09628171	0.093241155	4.049950791	1.350642834
10.88203429	0	0.889288537	7.848234752	0
4.965403878	0.161071631	0.155391275	3.930261407	0.118624533
10.36243816	0	0.866496674	8.663714275	0
6.556247815	0	0.888222406	6.136987727	1.56094737
4.451955547	0.008742832	3.048950425	3.75875152	0.006906748
3.148201627	0.150004506	0.144807354	3.871219579	0.110997699
4.679029836	0.0435042	0.042265614	1.185544944	0.033788307
4.400644866	0	1.729950715	4.290452984	0
4.319607662	0.100360152	2.477419682	4.530325919	0.075907009
2.976973928	4.739269109	1.717418583	2.751580985	1.705636865
2.277480065	6.714307174	0.048530225	3.309406174	0.038691846
5.673880618	0	0.680398797	1.553389743	1.604032001
4.383969014	0	0.75511084	1.27689213	0
7.898761413	0.738968278	3.52286272	6.422052368	0
3.065422965	0.082150662	0.079623987	2.002492464	0.06265349
5.141828443	0.014178114	0.013799532	2.672205508	0.011170357
5.813005578	0.038306007	0.037227344	5.256435604	0.029825623
5.352428692	0.020904193	0.020337479	3.304001735	1.080623736
4.079424371	0	0	2.36721522	0
4.94758225	0.005881313	2.030921559	4.039792042	0.004652817
3.788710756	3.098613947	0.09956571	3.180637526	0.077704793
4.313023043	0.143456791	0.138539353	0.213338526	0.106452849
4.547468824	3.40573807	0.117213206	4.049003542	0.090828538
4.861109013	0	1.894541847	3.45194883	0.922522413
5.834956417	0.200434095	2.42896187	3.408012556	0.145214243
5.518018626	0.08795957	1.353054332	4.552877454	0.066904769
5.95822074	5.110012078	0	3.462376208	0
5.793967434	0.16228094	0.156546995	4.272854641	0.119453792
5.148442542	0.07850861	1.948093929	3.046316602	0.059976694
7.345594082	0	0	3.050492648	0.763873587
6.392723536	0	1.388243841	5.2156976	0.571189643
5.992317026	0.125162972	0.121002443	6.935541592	0.093623171
4.693093998	0.025198779	1.707196274	3.623038393	0.01974561
4.800705332	0	0	0.785355144	0

5.505386615	0.085843999	0.083185204	4.791748414	0.065359038
5.534725697	0	1.514198384	4.904424663	1.164910707
7.333668746	0.005534711	2.029111072	5.593198888	0.004379372
4.683023397	0.014395189	0.014010621	2.673525656	0.011340162
6.493027668	0	1.362465718	3.4160942	0
10.80853904	7.464617419	1.281389567	9.566430906	0.053282941
4.124880622	0.005515634	2.615421582	4.359621164	0.004364319
10.63884924	0	3.146222433	8.982726289	0
5.776104432	0	0	4.742068761	0
0.000413063	0.00031082	1.00130493	2.586897344	0.000246583
4.659596276	0.12305231	2.133686192	0.179833148	1.439562019
5.33490133	0	0	1.872934222	0
4.386500711	0.156969977	0.151470225	0.236175426	0.115805884
6.144238066	0	2.982191892	5.836097906	0
6.723157682	2.497500839	0	5.213598521	0.939716624
5.429605599	0.032575759	2.166153184	2.782548827	0.025434475
5.447015038	0.035245388	0.034259386	4.231612954	0.027483241
4.619227403	0.176060037	0.16970461	5.135263766	0.128846185
5.187287761	0	2.341770212	3.172798985	2.104820883
5.686086337	0	1.366520165	4.777263071	0
4.646477143	0.027918262	1.115544553	4.646456521	0.021847507
3.353287593	0.135276768	0.130702277	1.573297362	1.479084571
6.697088399	0	1.178814764	4.584027054	0
5.924039796	0.041633583	0.040452915	0.056887492	0.032364555
6.520358743	0.121508258	0.117494564	5.615497894	1.434523083
7.429334624	0	0.982262029	5.099693944	0
5.219914759	0	1.290481847	5.003734392	0.770529117
8.502458677	0.016395122	0.015955133	6.380345238	1.063457561
5.633502643	0	1.808370714	5.289062013	2.073673991
10.29410133	0	0.807789762	7.680149772	0
4.46832626	0.089688287	2.763030698	0.127455902	0.068165681
1.819713396	0	0.660597984	1.818119388	1.577202707
3.514101048	0.222029094	0.213458597	2.674812741	0.159464671
6.250317727	0.000338219	2.001789859	4.757234787	0.000268316
4.209897642	0.18036694	0.17381318	1.764391079	0.131761004
5.385738202	0.140555209	0.135760248	3.820431111	0.10443097
3.799152327	0	0	3.885794139	0
6.487978513	3.233961438	2.646610475	5.601514617	0.008876439
10.48232931	0	1.921444783	7.680412547	0.724387598
8.85744614	0	0	5.903192449	0
7.033467395	0	0	5.118405578	0.868122831
6.711081804	2.33571026	0.065322034	1.929065936	1.868679339
4.859005765	0	0.971253519	3.654194753	0
8.284149597	0.016366975	1.068143835	6.157620573	0.012880911
7.567714382	0	0	6.497474596	0
7.014600975	0	0	2.731525753	1.39346979
10.11887041	0	1.572322164	8.521104675	1.573354083
6.195503059	0	1.276517497	5.228040786	0.75981459
5.512349584	0	1.319019859	4.255283819	0
7.553014785	0	3.298863901	6.114762186	0
5.522723692	0.009108194	1.629822823	4.521646479	0.007194071
2.877963738	0	0	0	0.547236974
10.13445463	0	0	8.075934103	0
12.79381081	0	4.016305301	12.29605556	1.872694571
6.007265357	2.300381055	0.656721804	5.111158913	1.571914072
4.400281782	0.011541995	0.011235655	2.880034281	2.054594673
8.510603343	0.0188512	1.078385906	2.925320508	1.668955417
5.235739603	0	3.409128827	3.749658566	1.758392421

5.039827286	0.192604751	0.185476954	6.104808544	0.139990088
5.954872638	0	0	5.285582049	2.097484856
5.585097926	0	1.360931293	3.777164252	0
5.703147784	0	1.917189956	4.581535722	1.244425759
5.578502502	0.022229833	1.092273125	5.105351982	0.017444546
3.802170422	0.077853948	2.977942783	3.47227763	1.287426889
5.789376855	1.126893222	2.766301313	4.510382162	0
8.907419168	0	1.190825603	5.076168512	0
5.491560298	0.004600193	0.004480046	3.352163082	1.017971752
5.512491352	0.039392558	1.16207075	5.351200341	1.14975523
11.47626916	0.052347631	2.261356464	10.70827369	1.197029139
5.672182142	4.760737232	1.12951141	5.299031742	0.024490851
7.846272273	0.068627035	1.905096146	8.022383778	0.052669351
5.298326668	1.03059858	1.030357642	1.623035304	0.005737203
5.145920184	0.021411378	2.924318797	4.536472831	0.016809035
4.977323452	0.060179682	0.058406598	1.893358078	1.225157533
6.496082682	0	1.217940754	4.474001219	5.554569047
5.143846553	0.035313352	2.179574614	4.232044831	1.739256616
6.193062266	0	0.641870818	4.9401173	0.657780311
4.148858465	0	1.580106695	4.034801795	0.699891685
5.38418657	0.051306976	1.209802015	5.956131022	0.039700003
4.070468765	2.990066821	1.109178093	3.903344421	0.632860831
6.044050126	0.030994656	2.158365281	4.204497548	0.024218597
3.668530125	0.032459622	1.134025033	3.668798292	0.025345228
10.17062232	0.116921498	0.113090003	9.665759839	0.087778345
6.29877569	0.01569423	0.015273719	6.237991161	0.012355566
5.5489529	0	0	4.491678171	0
4.616480984	0	2.668484525	3.880606814	0
6.156196499	2.326979145	0.000890142	3.465505503	1.589124706
2.626050094	0.212714436	0.204610084	3.995887535	0.153346152
2.782565985	0.147339899	2.229750035	3.85693435	0.109151111
5.742264088	0	3.642535613	4.106956131	0
5.257504886	0.012700122	1.052977562	4.40805696	0.010013243
3.105752944	0.089440198	0.086651266	2.03838169	0.067984846
5.680852891	0	0	3.909194931	0.698823678
5.563050866	0.015008589	1.658468312	4.625586111	0.011819792
4.695522545	0.046117084	0.044796906	4.827553314	1.174411033
5.099932494	0	1.5316753	3.107378059	0
5.522724046	0	0.849806672	4.952816519	1.417354112
6.526885572	0	0.662295473	4.355669904	0
5.052166361	0	0.842676276	4.257981375	0
3.241424099	0.239987655	0.230494173	0.388885926	0.171144004
5.943652165	3.768914877	1.469609282	4.881639313	0
4.154783936	0.072131765	1.291844442	3.010565825	0.055268601
6.094814562	0	0.95568591	5.510317988	0
6.355056891	0	0.608026893	4.612372993	3.260161052
5.269639236	0.062989078	1.25603961	4.403888395	0.048470439
4.257336929	0.054438522	1.22225087	5.56926491	0.042060415
4.487933897	0.151747123	1.590341753	0.227286435	0.112203167
8.964914916	5.378456258	0.078461202	9.960755513	0.061768403
5.855375808	0	0	5.815224757	0
5.670585957	0	1.987242633	3.482972366	0
3.091642656	0.086883648	1.983992787	2.475025152	0.066119017
5.882727108	0.160099318	0.154461936	5.780057513	0.117957205
5.117477671	0.083261147	1.334985708	6.51697351	0.063467917
4.594533978	0.079983546	1.954451756	1.991793957	1.294819813
10.04054652	0.481391888	2.190714179	5.301028994	0.501421039
4.29344771	0.095814208	1.383072641	3.142132692	0.072618321

hsa-miR-6516-3p	hsa-miR-6734-5p	hsa-miR-561-5p	hsa-miR-432-5p	hsa-miR-1224-5p
1.355699975	1.975603464	2.025431156	5.828765727	0.110821363
0.004736542	1.611340776	0.00570506	4.741076876	0.006568154
1.151391526	0.399216678	0	7.088236397	2.368876063
0	1.435173081	0	5.32163167	0
0.010790493	1.051775981	0.01303554	3.673944586	3.40360682
1.108600121	0.021416219	1.720771676	4.997269976	0.031550865
0.134230014	1.610257652	0.172061295	3.826638397	0.210374406
1.448590019	0.590652441	1.41504609	5.184803377	1.394336547
0.780876049	1.72458858	0	2.449735259	0.764402196
0	1.288130164	0.737609068	5.62280085	3.286249895
1.983730617	1.3484315	0.093110707	4.694055926	0.110646469
1.305672509	0.061027528	0.079385135	5.780946175	1.349148398
0	0.955533143	0	4.874080757	0.950572512
1.833963996	1.21512349	0.055948801	3.825669722	4.143956148
0.971141132	1.965062945	0	4.197580069	0.968321825
1.867465037	0.049563286	0.06402392	4.121225009	0.07528466
0	0.72614149	0	3.318197298	1.218198331
0.807391049	0.809781597	3.514288664	0.796586608	0
0.083520415	1.387964936	0.104518599	10.14117137	0.12471641
0.966585612	0.96706787	0	6.55448623	0
0.213794588	0.201160473	0.284199718	4.197811188	0.361657186
0.074262375	0.070709148	4.391078109	4.601575269	1.40566676
1.461575456	0	0	10.4436151	0
0.120933855	0.114664689	0.154052795	3.206278035	0.18713918
0.873641659	0	0.866712443	9.706513951	1.41800897
0.917393217	1.278835087	0	5.639901691	0.444934908
0.007013714	2.040981332	1.036472539	4.146595636	1.630198108
2.605765717	2.157541835	0.143581696	2.797219714	3.584690762
1.165874685	1.770850916	0.041971089	3.981142895	1.186272724
0	0	0	3.653779363	1.717613256
0.077272919	0.07355521	2.039206503	4.530430279	1.422340088
1.706483671	1.103906334	0.026429638	2.154201277	0.03065758
1.189609937	0.037577798	0.048187496	1.841460912	2.26721151
0.694128619	0.697482335	0	5.235838296	0
0	0	0	4.10308429	1.663832077
1.287423508	1.686873997	0	7.005572125	0
0.063751486	0.060760516	1.329827591	0.116482934	0.093430381
0.011345133	0.010867481	0.013709302	4.851833567	0.015829199
2.17605258	1.144190189	1.768015777	4.339128773	1.164283199
1.678514907	1.079802997	4.508802799	5.569578038	1.692158242
2.144084833	0.864602546	0	3.569328205	2.117254303
0.004724483	2.02768289	0.005690502	3.946539814	0.006551359
1.375409066	1.368182616	0.098787652	3.179891974	2.084168955
0.108481225	0.102970725	1.558535069	4.313353409	1.59624351
1.436729545	0.08793705	5.115051748	4.438164501	0.139325948
0.922133289	1.90487087	0	3.673047022	0.914995386
0.14816963	0.140159123	0.191168264	4.136727077	0.235345284
1.324508061	0.064866754	0.084576249	4.458350679	0.10019475
1.421790562	2.407099873	0	5.582530486	0.850554528
0.121782597	0.11546085	0.155196013	4.876541877	0.188605577
0.061022048	0.058173999	0.07554205	4.669386339	0.089199989
0	0.765673735	1.282390974	6.454033387	0.745512343
1.423667732	0	0.554108654	5.796239858	1.012516374
1.449713771	0.090627936	2.913246633	6.895765539	1.522922314
1.697163859	0.019199789	0.024344854	3.973375051	0.028217956
0	1.346267182	0.786873514	4.38864158	0

1.317189976	0.063373994	0.082555053	4.629314019	2.780486012
0.653722974	0	0	4.963804695	1.496113027
1.610194403	0.004262476	0.005355288	6.324266023	0.006164685
1.056119821	0.011032563	0.013918965	3.679897136	0.016072505
1.398636829	0.556998198	1.363579324	5.018841728	0
0.05419884	2.300015881	0.066874349	10.15778799	1.295003501
2.026506477	1.609649425	5.353356016	3.20577374	0.006143406
0.65145969	0.655092421	1.136395721	10.14946	2.035135133
1.3041673	0	1.286315275	6.120398365	0
1.586388596	0.000240065	0.000300866	5.08963314	2.586808191
0.093840258	1.433942541	0.118013615	3.523341062	0.141507842
0	2.212265087	1.474712936	4.430465253	0
0.118049305	0.111958017	0.150173896	4.847606026	0.182172439
2.158134479	1.437667972	0	5.385473569	0
0	0.940262714	0	5.913200912	0
1.728766537	1.12318795	0.031454026	4.676713424	1.750615303
1.135228908	0.026710544	1.14499497	4.481200799	1.763852391
0.13139869	0.12447355	0.168208771	4.738673521	2.856479506
1.385823905	1.388989306	0.823730569	3.173054805	1.363538773
0.828381168	0	0	4.597641818	0
0.022198006	0.021240771	4.153820325	4.99573489	4.628380276
0.102640153	0.097477146	2.620574288	3.354129244	0.156080484
0	1.594366799	0	5.536003591	1.166327402
1.158966978	0.031445035	4.54696438	5.084528604	0.046831179
2.505646866	0.088137151	0.116545924	5.970922563	1.508187756
1.979459683	0	0	5.493807515	0
0.769542293	0	0.758429439	4.169169501	3.207020109
0.013105601	1.657349293	0.015850286	7.19089284	0.018315461
0	1.331757104	0	4.755801562	0.921345437
0.817430953	0.819720322	0	8.917502439	1.343614893
0.06937364	1.324271888	0.086227385	4.960845453	0.102211867
1.88104151	0	0.661005982	2.271094098	4.30036732
2.837582206	0.153794459	0.211448379	3.154915664	0.262209782
1.586514253	0.000261223	0.000327387	4.172239149	1.586723529
0.134384051	0.12726875	0.172271165	3.304921476	2.422145526
0.1064136	0.101026722	0.134628618	5.867604576	0.162401923
2.43401404	2.176268484	0	4.549507027	0
0.009014564	1.043288525	0.010880643	4.66186304	1.643062872
1.641888961	0.726399612	0	9.458130316	0
0	1.09894264	0	7.701533519	1.413944894
1.42816691	0	0	6.116023923	0
0.052594482	0.05017959	0.064844401	4.3358775	1.286188465
0	0	4.604131256	4.122878713	1.961568948
0.013083273	1.062713695	0.015823109	7.589721084	1.070683642
0.692445697	0	1.571901096	6.803108105	0.672273491
0.837857875	0.839933403	3.455965694	6.496896116	2.336109651
0.989976707	0.990124306	0	10.16181232	0.988973906
0.758793892	0.761637691	0	5.170395066	1.265818681
0.524747012	0	0	3.997856415	0.951373026
0.844891987	0	0	6.790267843	0.392805503
1.035653531	1.625544418	1.037989389	5.107325829	2.05052979
0.545760287	0	0	2.520389393	0
0.890244292	2.183297668	0.884107458	9.448578783	0
1.464163974	0	2.658875588	11.3916759	2.839555571
0	0	0.657131931	5.403379044	1.148795511
0.009247133	1.044400773	0.011162627	2.656030087	1.644557078
1.073240106	0.014412716	3.689877901	8.085213875	1.081347515
1.756849611	0.811192421	0	3.908353782	0.793840187

0.14281511	0.135155625	0.183801427	6.364839687	1.789854982
0	0.341690524	1.015688807	5.58674748	0
0	1.378869614	0	4.72630117	2.532103093
1.241042172	0.454730477	0	4.237661551	0.849266427
1.684291448	2.102220512	0.021481428	3.846686714	2.121828116
1.289349514	1.284053881	0.074915793	1.98026735	1.329841636
0	0.65075861	0.632705434	3.528526036	0
1.514978539	2.322644748	0	7.98124952	1.440941289
0.003697585	1.605576021	0.004451389	5.353995763	0.005122492
2.506638482	0.029787752	0.038016264	5.909412258	1.168885416
1.19826889	1.19484777	1.831935866	12.32346932	1.847519802
0.024886147	1.118664493	0.030271855	3.661709053	0.035163384
0.053573541	0.051108962	0.066082808	7.720095673	0.077763651
1.617976641	0.005583578	2.361086585	3.748890219	1.622551979
0.017075564	1.081705439	0.020691832	4.900186809	0.023952018
1.226602406	0.045013531	0.057985524	5.789165779	0.068035204
1.629058595	2.2006948	0	5.195905526	2.351898418
1.135482671	1.133245826	0.034090204	3.220987736	2.519674312
0	0.660092572	3.910358941	4.361369253	2.254794657
0.69870128	0.702022405	0	3.273306346	3.083313531
2.231882194	1.802204484	0.049469533	4.42061814	2.274034797
0.631519545	0.635267278	0	2.376861367	0.60965333
0.024609256	1.117358718	0.029930995	4.851721322	1.133233885
0.025755113	0.024635941	0.031342168	2.182369872	0.036420775
0.089394357	1.414184018	0.112184104	10.05367387	0.13423472
0.012549433	0.012019648	1.661572746	6.837084265	0.017529122
0	0	5.169850305	3.369933945	0.942108403
0	0.595568939	0	4.315973651	0
0.000735784	0.000705549	1.003827927	5.363951773	0.00101653
1.721586291	0.147942375	5.033107	2.628107572	0.250585862
0.111240949	0.105564398	0.141058205	1.622216236	1.611715929
2.27310502	0.771529825	0.757635366	4.578813175	1.973453052
1.049576308	2.059192783	1.052870565	3.892056121	3.885248859
1.329616632	0.065909661	0.085990456	3.33821355	0.101922275
0.697630549	1.223926325	0	4.796316627	0.677633429
2.65877609	1.651341823	0.014511385	4.625607307	0.016760184
1.785412221	1.172509764	2.231022415	4.388299751	2.247418334
0	0	0	2.59843788	1.513930159
0.857701229	0	0.85004416	3.919383014	0.845751044
1.577105537	0.679937578	0.662702523	4.487298897	2.076688165
1.408130049	0.852814981	0	4.487850401	2.574994077
0.174770448	0.164955623	0.228270434	4.341054658	0.284776307
0.90893073	0.910178848	0	5.642960335	0.900728049
0.056222576	1.883251889	0.069439326	3.613422177	1.306129947
1.536152172	0	0	5.095610147	1.940654723
0.623300724	1.129666456	1.767491185	5.81982927	4.986280088
0.049295208	0.047046515	1.87933333	3.715090517	0.071265758
2.245061377	0.04084088	3.609248466	5.000974417	0.061448564
2.17700825	0.108497246	0.145231522	4.607113649	1.629238959
0.062848938	1.916465866	0.077872281	9.568513684	0.092029144
0.828278094	0	0	5.663333872	1.357982411
0.4877808	1.308788335	0	4.13424156	0.900805944
0.067285902	0.064107963	0.083548401	0.123683842	1.367097493
0.120250879	0.114023945	1.618317244	5.555999807	1.662338503
1.308225345	1.302519059	0.080086574	5.859772107	0.094721897
2.34895223	1.291344407	0.076952768	4.832028951	0.090912194
1.609392923	1.619833398	0	7.529316526	2.70809741
1.979702879	0.070382195	0.092075056	1.405427916	1.403753061

hsa-miR-485-3p	hsa-miR-371a-3p	hsa-miR-944	hsa-miR-3934-5p	hsa-miR-509-3-5p
6.665653085	0.125493415	0.128871013	1.366280039	1.410180165
5.128326622	0.007247205	3.497900652	1.61258843	0.006635628
4.748141642	0	2.213415426	1.412268208	0
3.943510965	0	3.106040523	1.833618687	0.860032628
1.057835072	0.016638181	0.016993343	0.011520274	0.015205326
4.186223343	0.035029025	1.732740124	2.135442739	0.031894527
2.477063319	0.244267734	4.830389522	0.146021555	1.744764645
4.425990974	0	0	0.580893098	0
3.318185079	0	0	0	0
5.030583795	0.730667767	0	1.276957961	0
4.181886009	0.125289881	2.077736255	1.365752476	0.112066752
5.741084463	0.105846369	0.108588495	0.068934698	0.095034847
3.729716373	0	3.246933851	0	0
1.883188211	0.07344763	6.709994131	0.048880745	0.066366661
4.037359343	0	2.952950297	1.550333322	0
2.705486638	2.97197778	3.573853054	0.05581756	1.283430426
4.139490657	0	2.536847131	0.718368454	0
0.795702345	0	3.573140935	0	0
10.09785152	0.141720419	1.469593444	0.090180006	0.126360081
5.612687366	0	1.950988478	0	0
3.047766403	0.436411306	3.02244378	0.235155899	0.368463113
4.773608289	0.124447408	0.127790172	1.995071121	0.111331424
10.95162365	0	2.160927422	0	0
5.137365837	0.216017949	0.222839088	2.23157897	0.189888394
9.404026868	0	1.415141137	0.871133792	0
4.731094217	0	0.872955835	0	0
3.056075395	0.010762607	0.010989124	3.746425935	0.009847389
1.636896132	0.199901542	3.417790401	0.122690187	0.176258885
2.243640805	0.054593249	1.189137073	1.780494174	1.805317568
2.9873008	0	1.321416773	0	0
2.092648312	0.130018017	4.411680657	1.377959262	1.423782893
2.483902778	1.728315464	3.342248963	0.023272834	0.030990834
1.842504316	3.106773752	1.21705846	0.042186165	0.056986786
4.13981998	0	2.486552056	0.689191264	2.815500754
3.903847649	0	1.27241187	0.762547862	0.749221692
5.067905232	0	7.68229946	0.748103304	2.601634879
0.115672629	0.105340892	0.108067193	0.068628319	1.348676084
4.343721757	0.017505817	1.061920485	0.012113494	0.015995451
3.04345678	0.047931554	1.785008369	1.150375987	0.043530282
3.132626178	0.025906415	1.091195406	1.083049581	0.023631365
2.77311564	1.400726297	2.110526654	0	0.850997515
3.037821619	0.007228644	1.025721603	1.023609867	0.006618658
2.104836326	1.443340271	0.137081063	1.386718988	0.119163941
3.633987949	0.1904604	0.196223215	2.937743266	0.168236236
3.891816785	3.262631225	3.501454224	1.450398518	1.508916023
2.681186817	0	0.914520257	0	0
3.694389296	0.274994221	8.839827141	0.16150806	0.239074542
2.823126173	0.113158715	1.380240203	0.073344508	0.101455285
4.881325755	3.933517564	3.4651999	0.859497657	0.850352702
4.415055675	0.217791178	3.200595727	0.132241636	0.19138299
4.585529559	0.100465	2.430812132	0.065662608	0.090298194
4.696125285	0	2.918868967	0	0
4.536473626	0	2.422627012	1.411342028	0.546544994
5.419144055	0.164447105	0.169202411	0.10315303	0.145982841
2.471331219	0.031302794	3.482094518	0.021449113	0.028522987
3.347049814	0	2.260927857	2.047178375	0.781131631

3.849080834	0.110305656	2.46906255	0.071628881	0.09895239
3.617053955	0	0.633060099	2.08964812	0.632120436
5.286380211	0.006801345	1.614367757	0.004742727	0.006227953
3.415685068	0.017775965	2.673210171	0.01229805	1.062334387
4.664395644	0	3.267817471	2.138235249	0
10.01733673	3.413371659	2.719611278	0.058259325	0.079666586
2.355112508	0.006777832	4.621975657	3.028893601	1.023949132
8.197325815	0	2.973013326	1.834296634	0.629798375
6.121203352	1.65603103	5.006689553	2.002927084	0
5.046560653	0.000380859	1.001360672	0.000266822	0.000349203
2.662757479	0.161479963	0.166124807	0.101479808	0.143430585
3.550398192	0	0.904340513	2.206537952	0
5.115004862	0.210021556	2.365545733	1.570338236	0.184826895
4.528830208	0	0	0	0
5.2839654	0	2.709698938	1.512025848	0
3.376812951	0.040632173	3.007245054	0.027659718	1.140284874
3.025116426	0.044027093	2.528873383	1.744788117	1.15165984
4.618538136	0.238167016	1.755598294	2.282811392	0.208486514
5.960580357	0	3.039761338	3.086442108	0
3.289024403	0	1.747910112	0.825137006	2.064108108
2.755036051	0.034733194	3.987280571	3.141271965	0.031627039
2.725035804	0.178762682	2.262776479	1.498187661	0.15825638
4.064653055	0	2.469009859	0	0.663403899
3.586551656	0.052191569	0.053400367	0.035243929	0.04735896
5.143838374	0.159313789	5.046680517	0.100254429	0.14156548
4.292817407	0	1.975546994	0	1.976228456
4.488079712	0	1.276250587	1.696924384	0
6.855101096	3.806012862	1.071576741	0.013997107	0.018508955
2.680736709	0	0.48161494	1.598494131	0
7.405344607	0	0.802497594	0.814021363	1.34308208
4.012040655	0.115495056	2.042434981	0.074744735	0.103502821
1.817555971	0	3.128814231	2.693585598	4.795546141
2.676223341	0.308480181	4.006497724	0.17780496	0.266532279
3.172137458	0.000414438	2.001955336	0.00029034	0.000379988
2.951523904	0.244600981	3.803147592	0.146192372	1.745643118
4.545159199	0.186298748	2.287892783	0.115292094	0.164690789
2.809829768	0	1.430096799	0	0
6.207514374	0.013868304	2.654127498	0.009621516	0.012681053
8.697835157	9.105754404	1.592188092	0	0
5.607767428	0	3.590563348	0	0
4.849356671	0	0.855667082	0.864881686	0
5.34479493	0.085627784	2.708048222	2.308823953	0.077186971
2.542195485	0	7.191432927	0.972371984	1.549146553
6.043765139	1.071525722	4.355801555	0.01397321	1.669444692
5.5533758	0	1.546171339	0	0
6.497556173	0	1.761877151	1.789302373	0
9.600357341	4.737853323	3.442718909	1.987286845	1.571682079
5.080179382	0	1.948007518	0.754581761	0
2.935533943	0.948222384	0	0	0.501146873
4.950498511	0	1.119932879	1.171929657	0
2.864870176	0.011214627	1.03981227	2.044920254	0.010260037
1.609197268	0.523080633	2.650283316	0	0.522310641
8.476006135	0	3.3990871	1.858612269	0
10.10811974	0	0	0	0
4.672579962	0	2.608555671	1.559232992	0
4.247955528	0.014230322	1.050429828	2.38015996	0.013011131
8.141788043	0.023335717	2.107515461	2.416057764	0.021297401
3.831107329	0	2.683633553	1.75031881	0.79359619

3.892119218	0.263054145	5.901662327	1.685000689	0.229202638
3.362635483	0	3.141889329	1.733152453	0
4.438730004	0	2.050252524	0	0
4.16529146	0	2.574764094	1.50990327	0
4.471167329	0.027569432	1.09695799	0.018940929	0.025139955
3.042962866	10.78281205	1.336948536	2.948731	0.089527655
4.470386472	0	3.180812401	1.145774352	0
6.455298336	0.421646399	3.19038327	0	0
4.423834049	0.005650061	1.609415422	0.003943197	0.005174931
3.712007165	0.049320853	1.171363909	0.033371717	0.044779622
10.87849604	0.066014783	0.067590379	1.203543012	0.05973815
3.522962188	0.039074815	1.136535402	1.727720764	0.035549536
5.874559932	0.087334754	1.938238627	1.885428419	0.078699243
3.856044391	0.008926221	3.216572214	1.029104602	0.008170188
4.142570808	0.026542393	2.121895983	1.683541076	0.024208404
3.838628296	1.261016917	0.078082916	1.232760695	0.068837489
4.283575393	0.693892859	0.694632587	0	0
1.151054342	1.153798695	6.088662679	0.029956725	0.040093444
3.290108287	0	1.125636735	1.538440518	0
2.935114706	0	1.18190105	0	0
2.620777674	0.064664941	4.495228527	0.043293861	3.29584606
2.195126011	1.988097604	2.537057169	1.502380523	1.09510232
3.367039769	0.038626221	2.175284037	0.026331173	0.035144562
2.782016858	0.040484711	0.041398162	1.12793645	0.036821867
9.379621062	0.152900974	1.503946338	1.43563972	0.136034921
5.752090554	0.019393963	1.66788471	1.657682129	1.067932552
1.514455514	0	3.082649296	0	0
3.494518976	0	5.803539294	4.417238545	0
4.398603545	0.001120085	3.813423462	1.589268453	0.001026833
0.334805686	0.293936242	0.304405084	1.747761859	0.254647566
1.625596883	0.196051071	2.320069863	0.120608108	0.17299037
3.183410753	0	3.063809361	0	0
2.3979201	1.05553919	2.072754188	1.050742815	0.014321652
0.12669082	0.11515949	1.386576874	0.074543881	2.800952232
3.70141182	0	3.464550807	0	0
3.095718795	0.018539687	2.411494052	0.012819413	0.0169366
2.591457829	0.057956243	4.818993524	0.038981307	0.052529662
3.107131358	0	1.137762266	0.660376848	0
4.552581489	0	1.393832009	0	0
3.418687578	0	2.062368814	0.671355835	0
2.753176197	0	3.650129751	0	0
2.761526093	0.336957115	2.027812633	0.191216938	0.28962289
3.54379013	0	2.181887599	0	0
3.010856519	0.091975219	1.312399985	0.060453529	0.082805317
4.681150954	0	1.528689345	0.957382482	0.954198921
4.501444314	0	1.972619165	2.893759993	10.27534154
3.715388835	0.079907538	1.273119309	1.243166272	0.072111798
4.156742639	0.068731599	1.23631274	1.826827266	0.06216322
3.160021823	0.202425937	1.65221884	0.124050104	0.178399183
9.058562736	3.805040102	1.350198863	0.067647376	0.093168559
4.153236302	0	0.813958935	0.825032352	0
3.204442707	0	3.881540362	1.573484324	0.900113714
0.122811691	0.111706887	3.843983939	1.330111203	1.368269
2.381345651	0.214593979	0.221354364	0.130549168	0.188687461
5.894312214	1.359867453	1.360121983	1.31710589	0.095900896
2.779740231	0.10243723	1.994632672	1.305285425	0.092035293
7.592899869	0	0.915582319	1.836453506	0.476196816
3.142473645	0.123810479	6.260776891	0.079694538	0.11077534

hsa-miR-3922-3p	hsa-miR-7-5p	hsa-miR-496	hsa-miR-520g-5p	hsa-miR-409-5p
1.399690946	1.408381928	3.577157269	0.086805017	6.264600539
1.614912588	1.025534964	3.03783912	0.005373756	5.398581908
2.906011299	1.578906978	4.247771814	0	7.576768214
2.612665311	0.860287162	3.683929087	0	4.897775144
0.014134509	3.251053976	0.017404466	0.012266061	1.0577286
2.149371186	1.121039825	3.990733179	0.025537225	3.641723765
0.192411857	0.209419824	3.299668867	0.158562873	4.368075004
1.937358395	1.394703595	3.874946369	0	5.472584674
0	2.651037192	0.765238025	0	3.205149534
0.733638967	2.913865773	4.193186637	0	5.892347703
0.102558079	1.40777229	2.078965464	0.086674107	4.053486948
0.087206803	0.093519999	3.67014107	0.074031101	6.664930613
0.951183279	0	2.252487417	0	4.242933712
0.061180533	4.507005265	0.077303137	0.052338815	3.953380047
1.547565844	0.968339096	0.968227302	0	3.535854003
0.070121514	1.914817841	3.40448475	1.262684721	4.003968239
2.16228453	1.21840969	1.592153897	1.236719093	4.446209325
2.295199827	2.288508564	0	0	1.719474185
0.115378239	0.124225912	5.957918175	0.097148556	9.504744189
1.953575136	2.534507289	3.942584943	0	5.225868421
2.148919643	0.35964217	0.478104379	0.258442755	3.906787882
2.813430241	0.109501689	2.525796477	0.086131927	5.657328727
0.887368429	2.425686591	8.581495797	0	10.21221885
0.1716848	0.186320473	3.203763762	0.142286311	5.980057862
0.864255611	2.1338371	6.882281697	0	9.877402942
1.931597133	0	3.239789937	0	6.258740304
1.037315481	1.630154462	2.63909751	0.007963126	3.965675663
3.051685162	1.621501499	2.336246125	0.132790078	2.798742542
0.045770021	1.186128052	2.244730191	0.039337219	4.533610698
0.790128047	1.32524466	1.322016941	0	3.561807735
2.832279524	1.421894669	1.432443777	0.089707624	4.530321023
0.028733766	0.030557823	0.035663792	5.003029078	3.172545149
1.209717973	2.266836774	2.280010324	4.956650377	3.48265361
1.865418389	0.674104704	2.647836922	0	4.479457156
2.803445124	1.664110881	1.960053244	0	3.753980985
1.261813964	2.20090078	4.016185698	0	6.493489161
1.340332941	0.093083868	0.111254638	0.073698746	2.45019472
2.076307114	2.891238784	3.090261894	0.012898849	6.206882511
1.774390854	0.042905176	2.547376022	0.034674238	1.163485366
1.68981404	2.440538946	2.446570651	0.01899114	5.390829502
0	0.851263181	2.366390202	0	4.21585517
0.006161272	1.025469901	2.357415871	0.005360054	4.432565626
1.422630133	0.117177219	2.559821253	2.801079978	3.180648443
0.1526156	2.263461312	1.616638132	0.127153592	3.116548966
2.148383773	5.46499861	0.168805271	0.107904484	4.31971633
1.891423146	0.915037288	1.480260109	0	3.672974911
0.21455152	1.81898531	3.683080563	0.17575741	5.349795174
0.093003323	0.099818279	1.379382947	0.078818028	4.00208749
1.806840801	1.803029553	3.847201068	3.706616778	5.073166347
1.651843954	0.187778513	3.490454101	0.143321651	4.545107501
2.397053869	1.332234751	2.432775664	0.070483099	4.669240751
0	1.271424252	3.826461509	0	5.864833373
1.666008999	0.546885367	3.122765132	0	5.767570292
2.608107848	1.522305228	4.331302648	0.111332847	6.715762536
1.106825673	0.02812664	1.71741861	0.022872099	3.865823465
0	0	0.782055025	0	2.827997204

1.35511243	1.362461582	1.370324889	0.076954967	5.761507085
2.049730417	1.496459553	2.023530835	0	5.076350279
1.613084247	2.030773498	3.621589797	0.005044542	6.342598061
1.657558712	1.062167533	1.661036148	0.013095738	3.795910126
1.638160304	0.529836618	1.61602192	0	4.600875426
0.073284243	2.364805213	7.564736454	6.177386761	9.367774245
3.032288493	3.203455425	1.024024991	2.028139952	2.842271605
3.33059172	3.31501711	7.260284389	0	8.556942427
2.802176493	4.002238119	1.95884258	0	5.241651003
2.001697462	1.001348514	3.002005707	0.000283608	5.783545531
0.130615341	1.5138149	1.52944321	0.109500526	4.200945724
0	2.192529359	2.665800043	0	6.181961526
0.167238171	1.649080397	1.674414103	0.138771326	6.24534092
2.806604799	0	2.790758772	0	5.677185757
2.234428908	2.905823991	4.28688788	1.920400896	5.414980077
1.746800695	0.036431732	2.514457526	0.029522421	3.376738895
2.185687243	1.763655003	2.199297285	0.031924082	4.137804205
2.822077138	2.854734997	2.46028594	0.155086677	4.346400041
1.764521097	2.54595075	2.317560126	0	5.577057067
0	1.754114446	2.715316913	0	3.165666999
1.118222533	2.152017179	1.731520651	0.025326072	4.995638964
1.548934951	2.227832475	0.190315062	0.120092748	3.589986986
1.548953976	0	3.142964907	0	5.913341009
3.556723432	0.046673276	2.835825721	0.037660347	4.596298596
2.592130136	0.13910967	3.710710844	0.108159169	4.745722848
1.563365725	0	3.971402563	0	4.877404079
3.093756568	0.752592899	3.185837827	0	4.487974421
1.069756063	2.415392387	4.633454223	0.014908851	6.361472159
0.926834024	2.714824372	2.789510876	0	4.808762034
0.804833029	0.803027155	5.343895733	0	8.194581028
2.44909603	3.296920463	1.386783277	0.080339263	5.214645767
3.372622417	2.471868035	0	0	3.124594126
0.238216519	1.902087578	0.333143914	0.193925615	3.804335922
2.58691346	1.586721908	2.587072491	0.000308606	3.809640479
1.719670676	1.740997043	0.262297321	0.158752153	3.828448977
2.22970264	2.250858303	2.290950149	0.124651454	5.025368934
2.82446756	2.409425584	2.144277657	0	2.977411493
2.060733556	2.06221236	3.533049675	2.056984906	5.287131484
1.223331822	1.600184269	7.297649347	0	8.950037998
0	2.156509448	5.026172883	0	6.914346356
2.387452115	0.856277189	4.290253134	0	4.970797786
2.675554708	1.918875272	3.211974735	0.060588129	4.680924248
2.283032961	2.544167301	1.548508128	0	2.280524492
0.017166552	0.018226037	5.064523965	0.014883343	7.273581918
2.678972326	3.07285815	3.836619275	0	6.228658859
2.342194421	0	3.306636723	0	6.872399365
1.98609913	2.792034829	6.322216064	0	8.559375101
0.743440462	0.741338089	3.166587584	0	4.879535977
2.655392306	1.298889903	2.00189308	0	5.222204551
2.4351914	1.392686067	4.393170836	0	5.009867639
3.513220875	4.226222601	2.052405555	0.008295263	4.231669672
2.073927502	2.06063848	0	0	2.519431606
3.001618389	1.440071993	6.73476427	0	8.166125844
0.889679999	4.004243765	9.860374756	0	9.754258407
0.652603725	2.280140455	2.752413772	2.319507392	4.778280187
1.643309512	0.012836606	0.014881851	0.01050643	3.984273913
2.100969968	3.92182963	4.031230546	1.076663928	7.470502429
1.336007303	1.332370651	3.132127296	0	3.831126705

0.205997374	2.471472621	3.009113937	0.169136928	5.956195554
2.378314754	2.465019216	3.072293729	0	4.094378382
1.355957	2.532446571	2.049859606	0	4.541068759
2.626776256	2.216132034	2.78051146	0	4.049333978
1.094354285	1.698728572	1.701982054	0.020189845	5.151256177
0.082225444	2.740051059	0.10511594	0.069904593	4.297666802
3.005742207	1.486488813	2.220120559	0	4.891047106
0	1.441437339	5.836085351	0	8.508751332
3.349176893	1.608815309	2.349714648	0.00419362	4.938748783
0.041424992	2.212299207	3.245109398	0.035649739	5.35119556
2.271246324	1.223337817	8.313270188	0.047247401	9.83253445
0.032935898	0.03504781	2.775403744	3.865668795	4.668723047
1.285847213	3.385425301	5.648546694	0.061735033	7.671472782
1.621781188	2.850646163	1.623453578	0.006611882	4.297329223
2.114382682	0.023875254	1.697719017	0.019449839	4.049032425
1.25151509	1.256121972	2.670746025	0.054229275	6.01186172
1.896252016	2.987530673	2.68294163	0	5.196176526
0.037118197	3.376903953	0.046282295	0.03198519	4.75873412
2.054292513	2.04403879	2.580334239	0	5.82552694
2.524021268	1.563741207	2.655972828	0	3.460801304
1.83607772	0.057664687	2.891826307	0.046318043	4.332291097
3.294182564	4.169090712	0.612467775	2.041454815	3.497833184
1.73914358	1.133138238	2.505327963	0.028098774	4.0105112
0.034107598	1.1393655	2.183871294	0.029417881	4.463314293
0.124023805	2.14673444	6.241768203	0.104169903	9.167151269
2.409065909	1.067748993	3.687717393	0.014273605	6.836913112
3.619042057	3.238718558	1.513759949	0	3.716293015
3.47918048	2.487241342	2.856188019	0	5.101738832
1.589623893	0.001013443	3.327933666	2.004689419	4.960556115
0.22799629	0.249378863	3.106833659	0.186105533	4.812867491
2.257768136	3.068814667	2.323491436	0.130497938	3.65449182
4.019108577	1.667210255	2.214146673	0	5.099041984
2.65709893	1.650416113	2.398141329	0.011557819	3.251994549
0.094584453	2.02212994	1.385721327	0.080121014	2.038400944
1.87089233	1.862620127	2.654068942	0	4.35034506
3.257192065	2.896065344	2.411830047	0.013652	5.190499851
0.048533298	1.197143462	0.060896406	0.041677333	4.550043992
1.146734938	3.024646124	1.506504527	0	4.465811017
0.847346063	1.39706431	2.93169938	0	4.788426399
1.837696346	1.156305544	3.587360695	0	5.620103976
1.391070241	0.838545473	1.781035167	0	4.194294923
1.937531561	1.970581484	0.365052381	0.208933475	4.680528981
1.466760431	3.554701071	3.842536657	0	5.006857986
0.076133214	3.207970818	1.956261907	0.064841671	2.736334466
1.530888758	0	1.938749756	0	5.135137371
1.753121123	1.743732274	1.731423924	0	4.681962597
2.654200587	2.66485399	2.686345326	0.056728341	3.560139671
3.888497306	3.310406291	2.638699857	0.049113487	5.057713465
1.611939428	0.175107979	2.344579991	0.134287894	5.381571966
1.335499357	3.448868882	7.098825206	7.094165968	9.838803615
4.250121285	2.896220772	2.71514314	0	4.936760358
1.248852556	2.290512783	2.403738842	0	5.178793495
0.091854691	2.010331672	0.118059622	0.077870719	0.123173944
2.309928937	0.185148821	3.713509895	0.141453545	3.925095533
3.248294656	0.094369714	3.071714166	0.0746783	6.950065199
0.084494722	0.090576667	1.345222283	0.071785909	4.083862786
1.267987294	1.976508305	6.514920331	0	6.872270638
2.47720338	1.403336435	1.412998209	0.085721692	3.3743706

hsa-miR-147b-3p	hsa-miR-376c-5p	hsa-miR-382-3p	hsa-miR-539-5p	hsa-miR-3131
0.091852053	3.105093219	4.294901748	4.605652998	0.096855258
0.005633758	0.006535717	1.616235567	3.208287565	1.024866037
1.114609642	2.370453137	3.451952011	6.710178869	1.977541646
1.42257044	1.415044546	2.787080499	4.094456343	0.863161194
0.012869802	1.654356145	2.077321675	3.086086736	4.325413296
0.026835193	2.153139203	3.991139432	4.435472526	0.028103659
1.684453707	1.739467395	2.480288699	3.590073734	0.179729418
2.353045766	1.035804434	3.755867819	4.991241152	0
0	0	0.765618439	1.98426428	0
0	1.943936965	3.464843681	5.977818157	6.143643338
0.091712192	1.407513481	3.378486489	4.604788182	0.096706372
1.329549384	3.033288365	4.320219875	5.387530447	0.082367223
0.952346126	0.950614673	2.734305151	4.620753624	0.9517643
3.762491812	1.247122188	1.250788151	4.070511195	0.057949335
2.544433049	1.546893878	1.546085327	2.539312841	0
1.267737403	0.074852537	2.369053392	4.229757199	0.066353186
1.923936838	1.902774042	1.592425412	4.081352964	0
2.882997324	0	0	1.329968884	0
0.102915306	6.056259952	7.017127575	10.42417544	0.108645809
0.964678427	3.406835273	4.526652096	5.612681082	0
0.278492667	0.358428939	0.483747162	4.439341134	3.579052431
2.454828662	2.494454797	3.904615057	5.971787574	0.096089834
0	6.321915351	9.397657595	9.799556859	0.888595984
0.151479611	2.338364746	2.389506743	3.727400379	1.63185418
0.867140861	4.253777542	7.547718218	7.96401861	0
0	1.917872887	3.729574432	4.090687407	2.12370326
2.045414699	1.037812373	2.050309533	3.22665344	1.036819401
1.578494911	0.1725695	1.644081284	1.636742545	0.149675501
1.177388747	0.048694193	2.244769148	3.59799955	0.043426282
0.794143366	1.325351598	2.672947958	3.97161459	0
1.397016726	3.348977551	2.095933302	3.932051946	1.404656599
0.026083219	0.030497517	1.728547813	2.483884753	3.151429763
0.047525432	1.213257587	0.066579105	1.21334504	1.824646702
0.681586604	2.505477825	3.539293969	3.452087263	1.872901454
2.818938975	0.749626416	2.210670267	4.03964248	0.753843324
0.741421363	3.496763222	3.729395514	7.377919791	2.78836282
1.960138383	0.092874549	0.112018544	2.003058	1.334057613
2.073176316	1.657908873	3.678108499	4.904311215	0.014154339
1.156633081	1.164083814	2.81659764	3.832189592	0.038238171
1.685959871	1.691980702	3.837421719	4.785378319	2.109295866
0.855833198	0	2.110158345	2.77313233	0.85427895
1.613799676	0.006519006	1.025576801	3.360547389	2.840618683
2.482744521	0.116901425	2.108160786	3.180953064	0.102645392
0.13514681	0.164760422	1.616086464	3.117030031	0.143145612
0.114445759	0.13842392	1.521049441	4.437875229	0.12096227
1.487080894	1.482236222	1.885730743	3.864598818	0.916939625
0.187785103	0.233567897	2.576340247	3.40849861	0.199957724
1.350234527	1.370929244	2.482701839	4.248086262	1.356708213
0	0.850659118	3.088948578	5.478148406	1.81024463
1.622018812	2.343504855	3.727183683	5.059966662	0.161917693
1.944649307	1.971109849	1.33846845	3.806602591	2.386815447
1.283538385	3.607365373	4.034921354	5.649461595	0.749906008
0	2.447323424	3.78123291	4.761015976	0
0.118126559	2.182776452	4.451455038	6.241331852	0.124899972
0.024027245	0.028071435	2.143416026	3.484412338	0.025155438
0.787489762	0	1.313763234	1.316332168	0.785406478

0.081339677	0.097142537	3.086665105	3.51805869	0.085676318
1.517090646	1.496671763	2.973690489	4.483630903	1.135879395
1.023038863	2.03075159	3.495925543	5.711048322	2.029437694
0.013741634	0.015991337	1.660983453	1.660325032	0.014371037
1.00613367	1.867806583	4.013792988	3.415913312	3.253138204
0.065919647	3.979877657	8.009965941	9.563044129	0.069322036
0.005270196	0.006113097	2.355228642	2.842292619	2.838566154
0	3.725515052	7.05286982	8.637539495	0
0.755332844	3.077730113	4.16489161	5.093721921	0
2.80908582	2.323729354	2.586902721	5.359727452	1.00131433
0.116158988	1.513447753	1.528897746	1.522625132	2.141321138
2.682072252	1.46984155	2.187457928	4.888584289	0
0.147681309	2.320884391	2.36990991	4.386506683	0.156621674
0	2.801443538	3.779695677	4.631455921	0
2.49941503	1.505031862	4.478897314	5.591738512	0
1.133616711	1.139796604	0.042867492	3.009037171	3.9890261
1.144357516	1.151122696	2.798602312	3.393250118	0.03518339
1.670270054	0.203896709	3.281213741	3.568957232	0.175657098
1.772352092	2.328154862	2.722031606	5.122289898	2.916287744
2.079917453	0	2.05665878	3.698475225	1.364797054
3.32107808	2.479063177	0.036617677	5.61594506	0.027869948
1.525592168	0.155040309	1.581126367	2.724953194	2.191001187
0	2.488305974	3.750515673	4.416078635	3.493612303
2.2082514	1.178146773	4.075600044	3.725855064	0.041559316
0.114719105	1.507237218	4.987009892	5.711841931	1.48518804
0	0.981691723	4.293037501	3.778729487	0
0	0	2.42904269	3.678016524	2.816178312
0.015647448	2.904010643	4.108945656	6.78605348	1.068791441
3.079136254	1.982256422	2.890504356	4.320541553	0
0	2.048739263	6.514542889	7.858413972	0
0.084948991	2.022902303	2.043383025	3.871192941	0.089511788
0	0	1.520658549	1.524304307	0
1.826938739	0.260151756	0.336419718	4.043602541	0.221484285
5.729845148	0.000374351	3.587217212	3.324161208	0.00033767
0.169309103	3.212924574	3.302862696	4.511717831	0.179951323
3.466715581	0.161308006	2.752532794	5.179335063	2.212193297
4.220891638	0.874870717	0.875010036	2.977318795	0
2.645997281	2.386358521	3.242591991	4.984195221	0.011231438
1.23210251	3.24151696	7.283119687	8.990758543	2.38609006
0	2.502100887	4.799371948	6.663569615	0.582202154
0	1.410091223	4.622796912	4.620802579	0.859214837
1.271061937	1.285777213	2.983472701	3.881313174	0.067207627
0	0	1.960309521	3.12498463	0
1.067727624	2.903847899	4.431519589	7.6682359	0.016339389
3.221673511	0.672423002	3.157864317	6.649671343	1.870296641
1.379993062	2.744398743	3.71667993	5.562698553	0.828091536
0.989390243	3.306270649	7.449888877	8.215592149	0
0	1.266136925	3.960938153	4.024958947	0
0.967959866	0	1.569612697	2.724953467	0
1.149651392	1.816860988	5.86838597	5.086310942	0.397542353
2.634521672	2.05043334	2.05239878	4.647330222	0.009093102
0	0.980353763	0.980268336	0.986037399	0
0	4.144106879	7.958102309	9.16193364	0
2.851218352	4.787758824	8.858253324	9.663642045	3.430210181
0.657951646	1.522623649	3.318904145	4.908589083	0
0.011021318	1.049872672	3.244455617	3.884455088	1.642245088
1.077901915	4.202223292	4.64991433	8.792398299	0.018819244
0	2.035575334	2.9968294	3.663269402	0

0.180584552	0.223998459	4.08476724	5.222883816	2.413478199
2.897342217	1.977770335	3.6091849	4.245765721	1.010115747
1.36230673	1.352629514	3.282031629	2.874143993	1.756646003
2.406882794	2.216645032	3.635842056	4.201933425	1.473654421
2.113661394	1.698656598	2.721132229	3.465656386	0.022191857
0.073829121	0.087919383	3.042400738	1.981805077	1.949740284
2.456808291	2.028679048	3.072928077	3.992060912	0
0	3.157128816	5.384998109	6.35384494	2.509576791
1.019158611	2.348462655	3.489793388	4.353390327	0.004592652
0.037505143	0.044045095	2.822966619	5.392977353	4.387958385
1.212431904	5.006759812	8.029737493	11.04447373	1.835528777
0.029871679	1.744235257	2.77557017	1.747793724	0.031293811
0.065140902	3.189437053	4.829181809	6.38064669	3.360575814
1.030179656	1.03140157	2.853264762	2.365525674	1.030583935
0.020424095	0.023828844	1.697646059	3.307242788	2.111885488
1.243189429	2.318270551	1.89617329	4.637138112	1.247264237
0	1.58652756	4.181056588	4.474108546	0
2.177887057	0.039442385	0.046560242	3.547597642	1.756512537
0.643124137	2.044349459	1.785246492	5.306963915	1.139555695
1.200014286	0	1.852723726	1.187346087	0
0.048788025	2.273413669	0.068422637	3.490751344	0.051213537
3.869452519	0.609801914	1.094434253	2.811524421	0
0.029535623	1.133079508	3.194556491	4.204471201	0.030940647
0.030926865	0.03622817	2.18388301	2.513376959	0.032402876
0.110439249	4.722796112	7.044519187	9.457245159	0.116679415
0.014979633	2.411387818	4.806288148	6.518141535	0.01566782
0.944161978	1.515202044	1.513821106	1.514468124	0
0.577162929	0.569131658	2.604995648	2.125301703	1.710771331
0.000873569	1.589711311	2.005287213	4.465728141	0.000912318
0.199058844	0.24865115	0.319972354	4.200454443	0.212199532
0.138751958	0.169388508	1.632687339	2.785998995	2.23920987
1.992476582	1.279388276	0.753069231	4.225998227	0
1.052659201	0.014102112	2.398167668	3.252025555	0.012678899
0.084716149	0.101305974	1.385249176	1.380518082	2.000105467
1.885378998	0	3.54591084	3.458675079	0
2.077387046	0.016675407	3.266514387	4.561279289	0.014983373
0.043876639	2.246868308	1.824900416	3.880573035	1.190757408
2.80261141	0	2.043526938	1.510635712	0
3.48836102	1.796655722	3.340949681	3.216485937	0
0	1.530772908	3.420168076	3.584635557	0.660784939
5.20263345	2.099590957	1.781138409	3.907069558	2.366778094
0.224009365	3.118290832	2.031333608	2.761367217	0.23938356
0	3.026766943	3.544360308	5.15860144	0
2.349189601	0.081337114	1.311155075	3.439469455	0.071994635
1.94418791	2.521968018	3.735922929	3.62898985	1.943131882
0	1.08516344	3.403052635	5.06754321	0
0.059824115	1.898000254	3.713193033	2.95909329	0.062871534
1.839807654	2.289320245	3.335002676	4.156752078	2.871473318
1.584704376	1.627905742	1.651533908	3.880593626	1.597737991
0.076736183	4.456635182	6.469807932	8.957753998	1.329341379
1.367736133	0	3.289481378	4.498650353	2.332725833
0.472371776	0.901166343	3.126562261	2.405692623	2.466383585
0.082316052	0.098345391	1.374312782	2.026407332	0.086713593
1.61432158	0.184658863	1.68726796	4.2609417	0.159742234
0.07891314	1.99321408	4.216028806	6.843565537	0.083099328
0.075832094	0.090373978	3.054239103	3.484628504	0.079829094
0.484552576	2.709619705	7.332869534	7.993360896	2.489974845
1.379936832	1.403081766	0.131957881	1.407468165	0.095623433

hsa-miR-31-5p	hsa-miR-499a-5p	hsa-miR-3660	hsa-miR-141-5p	hsa-miR-5683
3.348782311	0.129633416	0.069954954	2.871646289	3.565557059
1.025728921	1.616286148	0.004468701	2.033754234	2.357242096
1.086520543	1.085878992	0	3.996052705	0.770866425
0	1.411956706	1.436103926	2.124415138	2.124187442
0.015719412	1.656284273	0.010171992	2.076786412	3.787677708
1.122140124	1.122621111	0.021066147	2.15888387	1.122766471
0.224263096	2.941303457	0.124585464	3.304978583	2.931158943
2.745239127	1.032593443	0	3.409153565	2.116375707
0	1.677698067	0	3.612692618	0.763635284
0	2.894640212	0	1.254317419	0.730741192
1.413521743	1.418448845	0.06985282	5.827206203	2.862870823
1.3533493	2.452337412	0.059943764	3.970575409	0.106277837
0	0.950269882	0	1.524893797	3.247426668
0.068888287	1.885419605	0.042728165	4.373824811	1.251926676
2.278597644	1.546007064	0	1.546496313	1.95777611
0.079141717	5.813295481	0.048703052	0.092717829	2.701060454
3.231271676	3.964312864	0	3.213592973	2.353329982
1.328220578	0.792064519	0.810683087	2.278366319	2.494719254
0.131780587	0.146541476	0.077995005	4.250242406	1.469074623
0	0	0	2.753369601	3.751251927
0.391513915	0.459544255	0.196687788	4.198180578	0.439323299
1.410941915	0.12854466	0.06942972	1.408590786	0.124972947
0	1.444219346	0	2.161506837	0
0.199017903	2.385916426	0.11242074	2.384685782	3.186446666
0.862130232	1.814490387	0	0.86502546	3.247516379
0.444517671	1.203398621	0	7.228679409	0.446329766
0.010176006	0.011039882	0.006615065	10.68526913	1.038259573
0.184537365	3.141196637	0.105264186	2.33173229	0.200864453
0.051327696	0.056149983	0.032275293	1.809381504	0.054793709
1.322954192	0.787909078	0	2.019680682	3.660560338
1.427955123	0.134344542	0.072217081	2.545177503	2.540724926
0.03207866	0.034956096	0.020483277	3.636719916	1.119332868
0.059108306	1.21698494	0.036941606	2.884215406	3.107210635
0	1.175952168	0	3.048524493	6.118409692
0.748609839	1.272495266	0	3.495255165	0.748758452
0	0	0	1.258266044	0
0.098402572	3.062556279	0.059682059	0.116063264	2.003898633
0.016537285	0.017964218	0.010694042	1.6590787	0.017564965
1.165767626	2.815290973	0.028501107	2.546727114	3.825468069
0.024446629	1.091151991	0.015706792	2.44610979	0.025995667
0	2.36688161	0	2.111415346	2.111126809
0.00683778	1.616206437	0.004457332	0.007813183	2.357152412
0.124205737	1.443899002	0.0739151	2.103938226	3.770629852
0.176027889	2.302510233	0.100995237	2.765574347	1.615679431
7.339127662	7.920408255	3.501576392	3.891774255	1.52149926
0	1.885729163	0	2.681296725	0.914387806
0.251525749	6.497004826	0.137301491	1.849734297	1.855949815
0.105601087	2.480630584	0.063706148	3.098801282	0.113626514
0	2.772972086	0	1.799409326	3.851142767
0.200607657	1.697282873	0.11319832	2.860244739	3.192351103
0.09390576	0.103620292	0.057146706	0.110584625	3.038391008
0.7446325	1.954281308	0	3.050523292	2.920891468
1.009902021	0	0	3.83567406	4.062138436
0.152478903	0.170278562	0.088926603	2.672716874	3.284097655
0.029518428	0.032149578	0.0188875	2.471160151	1.109943312
0	1.701559707	0	2.009712913	2.008773188

0.10297568	3.684391042	0.062243383	3.08708678	2.466523536
1.491829635	1.122126445	0	12.00502005	2.234428014
0.006433941	1.614370706	0.004195491	2.619259087	0.006823749
0.016791902	2.407959094	0.010856422	3.553844006	5.139383524
0.529101239	0	0	7.833095562	3.187100884
0.082780174	5.344327749	0.050801176	2.995429265	3.741509381
2.618141751	1.024110819	1.021201114	2.842267161	2.354973012
0.62923605	0	3.679123089	3.280751266	0.629931245
2.432127201	0.74864462	0	2.609887869	6.821172185
0.000360585	0.000390423	0.000236328	2.809331533	2.586883704
0.149783207	1.530130344	0.087520465	2.202030091	0.162208986
0	0	0	3.024396042	1.872251125
2.794103729	0.218078922	0.109776949	2.36507777	1.672768543
0.861817552	1.414773609	0	2.958071555	1.814450072
0.933346855	0	0	4.647529959	3.221702142
0.038270109	1.141794095	0.02431546	3.008904608	3.52581217
0.041449619	1.153354426	0.02626879	2.52919737	3.021673382
0.21882593	1.755972853	0.121998027	3.284156567	2.449366018
0.818118869	0.818527051	0.835410588	3.296331572	2.891685305
0	3.609932307	0	2.058296704	2.717929712
0.032738843	2.158335041	2.938597546	3.766829793	2.157657062
0.165457044	1.582247693	0.095624861	2.724167223	3.058921595
1.163714801	1.829322309	0	3.518760886	2.286961158
1.798423391	1.180984566	0.030919599	1.177557609	3.426529481
0.14781395	0.164911245	0.086489976	3.281460325	2.989518448
0	1.975535318	0	1.56272449	1.975610308
1.664567043	1.660915626	0	6.442978715	2.217304117
1.071345209	0.020802145	0.012350553	3.275503595	2.093371286
0.918917277	1.531400423	0	4.554542774	2.438584471
0.802140589	1.731709864	0	0.806472751	2.040653438
1.38337584	0.119232129	0.064899078	2.489620762	2.486285289
3.825960763	1.814384888	0	8.56543256	4.059876745
0.281007979	0.322264807	0.150593214	2.674599885	0.310229194
1.58674401	2.001956337	0.000257156	5.046753062	3.002155318
0.224559865	2.476509664	0.124726187	2.95051736	4.166598188
0.172270632	1.604740612	1.485675506	3.101368437	3.085273203
1.43133927	0.874315873	0	3.127466658	0.874022128
0.013107173	0.014228291	0.008499892	3.0719463	1.049208982
2.860509022	0	0	2.846661258	0.703644119
0.57624476	1.692382051	0	11.14442182	3.09273056
1.808150449	0.855763763	0	3.676025677	1.40697017
2.966084227	1.291725868	0.049307567	2.709818857	2.977257807
0	1.960327005	0	3.276577451	0
1.071223242	0.020766066	0.012329548	1.070036973	1.671276881
0	0.672831817	0	2.791556649	0.671871713
1.368797703	1.367369299	0	4.104076598	1.367354574
1.57156972	0.988891942	0	2.306582283	2.569233295
0	1.644982117	0.762712552	3.383300014	6.402330705
0.948869821	0.94945442	7.125724434	0.959057251	2.481935912
0	0.801992615	0	3.191714413	1.604688265
0.010602771	2.86456212	0.006890055	3.519476034	0.011251961
0	0.524747794	0	2.044294911	3.413665621
1.84125144	1.838894587	0	2.98657266	3.139391265
0	0	0	3.285188422	1.849937755
1.146160473	1.514066457	0	2.884935406	2.264380298
0.013448695	0.01460003	0.008718909	3.397276296	2.065972954
0.022028078	0.023955311	0.014180817	3.119742749	2.923782002
2.50271951	2.02635575	0	0	0.793023547

0.240955743	0.274039467	0.132421433	2.534902532	3.850696974
0.314710858	1.240242925	0	4.78973546	1.805243666
0.809152715	1.741456906	0	2.874328489	1.741889562
1.675207825	0.848755299	0	3.198079052	1.174082324
0.026010369	4.051733626	0.016690249	2.125919029	3.600803502
1.333702644	2.42787162	0.056689957	2.767644486	0.099991699
1.77605717	1.113296281	0	2.403745249	1.770645456
0.837875945	2.559900561	0	0.850907493	3.882804789
1.609122982	0.005793812	0.003488987	3.489893397	4.352422132
1.170423419	0.050710889	0.02929179	2.552933977	3.413245783
0.061975542	0.067944663	0.038647079	3.323496905	0.066263137
1.135896429	0.040150916	0.02341553	2.176578564	3.518376749
0.081768802	1.938433281	0.050219113	10.34315808	0.08767801
0.008441713	4.440895186	0.005495558	3.507359592	4.955157265
0.02504472	0.027252469	0.016083233	2.941040577	2.448453282
1.890038005	1.260959288	0.044239517	1.893290151	2.666747392
1.882828863	1.202288227	2.421615371	3.196944994	5.693988498
0.041530649	1.769712117	0.026318443	2.798688911	8.432123645
1.791193754	1.125815356	0	2.581492686	0.635060467
0	0	0	2.935427665	2.660097844
0.060718751	5.884761398	0.037900449	1.218570403	1.850545769
5.882322426	1.092513052	0	6.704935123	1.454706682
0.036390137	1.134943498	0.023155852	4.375457494	2.503629473
0.038131941	1.755023542	1.122000417	2.182715119	0.040628854
0.141977883	1.503975187	0.08341963	4.523320874	1.503281457
1.068307572	1.068490568	0.011827311	1.66694453	2.415121964
1.514363513	0.941770258	0	3.60980077	2.722598551
2.315348659	1.039202652	2.019167618	1.922180174	0
1.00399263	1.58983027	0.000694554	2.00524876	2.813101272
0.268232055	0.306795221	0.144889884	0.336417299	3.083832196
0.181069249	2.320959182	0.103530091	3.418706588	2.315992493
1.663433194	0.751647678	0	4.486514644	1.660196778
0.014804826	1.055450888	0.009587379	1.651399898	2.397537918
1.382337117	0.118883262	0.06472801	3.106984938	1.386341287
0.676750618	3.274945779	0	3.054944039	3.376323905
1.065334975	1.664304128	0.01131505	3.095658728	2.085563662
0.054466102	2.258413325	0.034164292	4.47144532	1.824314087
1.138878882	2.043891853	0	2.743013076	0
2.363631603	1.79104238	0	6.062478158	1.393873838
0.654914828	0	0	4.754640464	3.944049467
0	1.384752806	0	2.093038815	2.566370341
0.305895167	0.35263471	0.161465436	2.019912498	0.338942857
2.184603149	2.181796455	0	2.440741268	2.182333153
0.08606282	1.312331353	0.052684359	1.306657642	1.954541181
0	0	0	3.513912173	3.515352139
3.152627905	1.731216207	0	3.557685506	3.492822285
0.074885587	1.273045493	0.046234151	2.685746408	2.682089243
5.37337122	0.070752663	0.040144681	2.63804068	1.866311247
0.186809306	0.210093754	0.106395929	2.807367978	3.418226702
1.346701974	0.107003743	0.058843879	4.207157668	1.350057829
2.890745958	0	0	9.486117629	1.354558893
3.154633722	3.613120335	0	4.250286452	2.656070199
0.104265338	2.475038849	0.062962622	3.092846771	1.375429286
0.197740772	1.687991316	0.111794922	4.403700298	0.215652453
0.099776205	0.110229739	0.060453205	2.455940231	2.791097413
1.342660239	1.346017522	0.058174612	4.411379048	3.472614048
0	2.429592603	0	0.488350699	5.81087771
0.115386327	0.127881769	0.069109476	4.175754789	0.124332706

hsa-miR-129-5p	hsa-miR-889-3p	hsa-miR-204-5p	hsa-miR-653-3p	hsa-miR-766-5p
3.738746286	7.471907629	6.047466411	0.099816253	1.355287371
0.007257217	5.595756303	5.94820046	2.031691133	0.004724462
3.466287343	8.470408551	5.475200561	0.374817361	0.815327054
1.811403863	5.673002251	5.381554438	0.862389383	1.432826999
5.789611479	4.676849922	5.378426381	0.013801395	2.651327453
2.487256066	6.763183183	6.050431511	2.474243373	1.108493813
2.467441201	5.891874269	8.685767862	0.186103118	0.13378795
7.044411781	6.940088896	3.926830242	1.043428065	1.748542228
1.291304967	4.35882188	5.26998946	0	2.03287604
1.249287333	8.216898047	7.680473529	3.302897189	0
2.075574009	4.509979533	7.775866404	1.395368484	0.074497764
1.356772546	6.581461374	7.493485611	1.338817202	1.305328718
1.934010243	6.276171703	10.38050943	0	0.954927481
2.925467128	5.023435046	4.461942853	2.893638287	0.045407669
0	4.806917265	8.300850225	1.547866885	1.551283311
2.972211676	6.109830799	9.407631184	0.068259958	0.051798206
1.214094984	5.730955822	9.415943334	2.884593009	0
1.717387266	1.331785331	4.651408052	5.528630485	2.547757662
0.141979044	11.00334262	6.57584695	0.112043787	0.083268702
1.539915599	7.288266861	7.772355166	0	0
0.43762857	5.953354808	6.170403972	0.311806547	1.966457461
2.072579777	6.501624029	2.52113482	2.03511617	1.352673631
0	12.25750157	7.056947835	0	0
6.090351728	5.888346183	6.822705346	0.166232728	0.120544266
1.415210807	11.57158689	5.498884885	0	1.435706538
0.871832844	7.212622971	5.510101587	1.22186215	0.917819785
2.374131298	5.453047835	4.644853033	0.008948526	0.006995729
1.643130994	4.348113372	9.339527928	1.59854649	0.112770247
0.054677205	5.290022571	7.687282406	0.044613833	1.774386478
2.018916193	5.808423618	7.127851436	1.725113484	1.75001039
2.092313481	5.091310401	8.947043781	2.05273815	1.36653526
2.482715552	3.636335562	6.943103259	0.028034043	2.94014296
1.843722311	2.883049834	8.273657602	0.051265158	1.189412741
4.450814228	6.039051437	6.434285337	1.869004203	0.694328703
1.657125416	5.568889852	6.896293787	0.752666713	1.305373716
0	8.004037645	2.899525825	1.263762074	1.287688942
1.355163073	2.789015907	9.524370776	1.337325537	0.063566558
0.017530593	6.438568294	4.681527522	0.01451609	1.055230944
3.698747188	6.259460155	5.163138436	3.383027201	0.030230897
0.025943801	5.698163909	6.294062882	1.08818054	0.016631268
3.093164088	4.84064569	7.248108765	0	1.422676908
1.025752395	4.287988312	6.835849146	1.024976253	1.023058514
0.133675166	4.333497551	6.464897359	0.105818941	0.078871534
0.190838467	5.12150614	5.041912094	1.57396628	0.108139154
2.98740739	4.318939216	9.366482512	0.124834109	0.092226842
1.886050128	5.145896304	5.444909836	0.916434511	0.922198224
0.27561628	6.485554825	12.09885271	0.207283075	0.147670669
1.379964628	6.427135325	4.725110516	0.090423097	2.379618565
1.39990608	7.495184443	7.896525384	1.808474253	1.421950927
0.218243025	6.974566869	7.227434272	0.167491474	0.121389712
3.637302279	5.678518757	6.034128356	1.322879673	2.670922209
3.178995796	7.736995143	6.89784449	0.74871808	0.763033515
2.569250763	6.9820631	6.343755062	1.380147832	0.569984803
1.538096535	7.199142727	6.06913963	1.504602605	0.095072014
1.10994422	5.258737132	8.919279136	0.025815307	0.020007828
2.477663656	4.878309002	6.432071242	1.322219278	1.342752019

2.466212812	5.681799493	8.043937591	0.088238125	0.066316755
2.234634589	6.110234927	4.442542195	0	1.545525325
1.61432551	8.12128702	5.653393086	1.023507977	2.026565018
2.082070565	4.345111916	5.308586796	3.990639671	0.011487818
2.393455552	6.585551865	4.63307144	0.533439937	1.0317147
1.941127631	12.14869413	7.998847239	0.071326555	1.878784425
1.024152891	4.681824508	3.944041097	4.733068144	1.610080203
2.230700787	11.58775491	4.869069381	3.019533931	1.542322504
1.271312299	6.662318998	6.773530328	1.281477264	0.766056675
0.000381376	5.428445404	8.579641873	0.000317983	1.001223904
0.161785134	5.082280741	5.722828176	0.126738564	1.442240514
0	5.864552035	5.864551731	0.906427388	0.912813904
0.210451932	4.745005633	7.034408747	0.161964547	2.628479182
0	6.073033196	5.769808412	0.864618356	2.158329454
1.91135137	7.01937182	5.845025869	0	0
0.040692787	5.429605134	5.542396889	0.033386448	0.025776674
0.044093265	6.121889912	6.715905078	1.147731297	1.135093796
0.238677043	5.581348392	5.375833978	0.181844754	0.130967949
0.818042575	6.050404512	7.214747321	0	0
2.057796757	6.060153234	6.39870029	0	1.381043452
3.344194103	5.775373722	5.095569534	0.028605648	1.107610798
0.179110663	3.967357499	6.93384581	0.139420351	0.102319817
1.162199042	6.785745956	4.995258359	0	0.684330883
3.426292361	5.601204519	5.488327664	2.810539935	0.032806829
1.522610636	7.564180209	7.577225126	0.125137895	0.092438371
1.562471495	6.73813882	4.87740122	0	1.565535311
2.431893458	6.157444897	5.33042151	1.286265963	1.704910189
1.071647537	8.87946039	7.313977761	2.087123889	1.063757963
2.296991908	6.309335444	6.016202648	1.27621305	0
1.732203249	10.832835	6.984791383	0	1.768442509
7.365348017	4.562741325	6.828877294	0.092208891	0.069170158
0.653713713	3.126029793	4.257943044	1.161294985	0.675012444
2.657790475	4.586937	9.344566144	0.229872173	0.162223349
1.001482535	5.702819856	5.00235789	2.32381941	0.000271717
0.245129944	5.069671996	6.41383	0.186335215	0.133941369
1.603220583	6.396533048	5.620303502	2.221165782	0.106079263
0	3.502338175	6.419406005	2.826463816	0.884912886
0.013887765	6.75115168	5.121938659	0.011516471	0.008991339
1.592595438	10.86091955	9.027259278	2.381736189	0
1.406085317	9.188084169	5.299844959	0	0
1.406987437	7.232800877	4.620811814	0.858425263	0
1.930616527	7.340557384	6.250564998	2.339844208	0.052445324
1.548505457	5.199783575	7.558411285	0	0
0.020261615	8.718007599	6.97195842	0.016759176	1.063649887
2.485707678	8.371179475	7.132421244	2.331277082	1.90728853
0.823763481	7.032464805	4.35395413	0	1.79515964
1.985341423	10.45759166	7.632947308	0	1.573291135
1.948901502	6.251824759	4.843236002	0.744483814	0
1.291014503	5.318979646	7.51644481	0	0.524996343
1.96959503	8.227435693	5.226422454	0.809380172	0
1.039857439	5.870879667	5.017027982	1.038624115	0.007286818
2.379902845	4.04341845	11.19818473	2.26172187	0
0.879851028	10.97750454	6.354755734	0	0
5.909615761	12.68870653	3.821685675	3.303014457	0
1.514367864	6.661108342	5.381054394	0.653803442	1.569880746
2.390675776	5.861621848	6.23024942	2.873000723	1.045053758
2.107116837	8.838749263	6.397317545	0.019305603	1.073170396
2.027313365	6.057449652	6.090918326	1.73279106	1.757107585

2.524459472	6.108223629	9.637237366	0.19910515	0.142338229
0.690696512	6.276255285	7.088107167	0.697744361	1.325027197
0.809094613	5.655251295	5.072509123	1.754468164	0
2.037990917	6.359275973	6.112118303	2.240226424	0.892935221
3.462432874	6.083736676	7.948627676	2.11739999	1.684176886
0.099758578	4.58163356	5.863281149	1.954172461	0.060356073
1.478324599	6.727341429	5.837423107	1.497080015	2.093189715
1.656134209	9.318411596	8.303371696	1.182153151	0.443951709
1.02014772	6.182084831	4.78665885	0.004707332	2.022108892
1.171448503	6.642200379	4.652809961	0.040388137	0.031072871
2.623452683	11.96917438	4.66343986	3.989036827	0.041048367
1.136627308	5.767580484	5.2560016	1.739380068	1.120515456
0.087478494	9.272128379	6.004805658	0.070474752	0.053421301
8.695787583	5.408022455	9.301570663	2.362168586	1.028421697
0.026580752	4.788793875	8.591740892	0.02193062	1.08294382
0.076346514	6.443731052	6.508041901	1.249485073	1.842144767
3.498417243	6.836780033	5.248995619	0	1.938328147
2.796116067	4.820439608	4.9911594	1.758327308	0.027907562
4.506221019	6.254089872	2.980610595	0	0
1.555900652	5.458549802	6.290635744	1.192972331	1.220433757
2.617695966	5.626281099	12.32758388	1.213532508	0.040251339
0.609574868	4.664466576	4.889390973	1.473772987	0.63174267
1.135093582	5.91140645	6.142123639	0.031763211	0.024543537
1.754592718	4.463185907	5.67949754	0.033267207	2.740241549
2.623565159	11.17073984	6.021775703	0.120384468	0.089121978
3.098334741	7.289399185	6.039518832	0.016069707	0.012516831
4.716835119	4.905951941	3.815630564	0	2.254670715
1.919691284	5.444702471	5.28326622	2.507749705	0
1.58982359	6.050840233	6.17637724	1.589584932	1.589147487
0.294619369	5.043515602	4.812871527	0.220122879	0.155973834
0.196443832	3.652075555	5.812162122	1.588558937	1.520740929
1.660259441	5.129705709	5.572954595	3.720055246	0.768938996
0.015690258	5.373117083	4.172920191	1.648537204	1.049530026
1.386280987	4.256166939	8.189305398	0.091952591	0.068986297
1.180231457	6.37279111	5.939535293	1.191555343	0.697829171
1.065605114	6.126549065	5.911351402	1.063477718	2.071087051
2.858151334	5.260663441	9.139013358	0.047300185	0.036265332
1.80057706	4.379772766	7.057228179	2.47279937	0
0.844918405	5.296620924	4.070419181	1.802131861	0
2.900641061	7.000776733	4.719462827	1.841418563	0.676670042
2.092738406	5.156136956	6.621327715	0	0.850997345
0.337787618	4.680151656	6.04524326	0.248693513	0.174157748
0.90004757	6.082849986	7.13784106	0.902370852	0.909005728
6.118837283	5.050398993	6.280159581	0.074088048	1.889233937
4.683631829	6.539373119	5.783045195	0	1.536203834
3.493283534	6.311618193	6.099199649	1.0926208	0
3.704310193	5.3664467	5.969875911	1.887225002	2.280158257
2.905200702	4.815115467	5.787488692	1.226253624	2.571642784
2.794449985	5.510370094	7.026729958	0.156532227	0.113998774
0.103901028	10.45007822	6.048907086	1.96790507	1.299821918
0	6.626430392	4.973994416	0.817098355	1.380918188
1.935732212	5.815864306	5.344381333	0	1.300216651
0.111899708	4.635126273	6.585700649	0.089311817	1.945764114
0.215036943	6.022114948	5.133639848	0.165220778	0.119863938
0.107014207	8.067993033	4.220064424	1.341721195	2.361335884
1.345901733	5.470210604	5.727218861	0.082187997	0.061948783
0.477410603	11.3994217	5.952520011	2.482215396	0.500150025
2.070313735	4.687424573	5.467559755	0.098539717	1.979178589

hsa-miR-551b-3p	hsa-miR-539-3p	hsa-miR-20b-3p	hsa-miR-1185-1-3	hsa-miR-483-3p
3.581192589	2.867846893	0.108739167	2.018313599	2.523315726
1.616079856	1.02578271	2.619502735	2.616880929	7.735161189
7.189156898	3.459248209	1.35644488	1.820547997	12.27515178
7.889988778	1.41194027	0.86054536	0	3.105386209
5.046429711	0.017021886	1.654120747	0.01263164	10.82689632
5.234224769	2.159564814	1.120689791	1.115016602	2.981071764
0.272239573	0.252995048	1.735794643	0.164902662	2.466008782
1.67438509	1.671382043	0.564447396	0.573509608	4.081989771
6.377726364	0.764160922	1.686010956	0	9.128322825
6.342970307	3.646341996	0	1.966190664	12.37807822
2.077342658	2.077880127	0.108568227	0.089714295	7.681620889
3.069439571	1.356963451	0.092159868	0.076562486	9.872594621
0	1.52411827	1.52563052	1.528765449	4.091504128
5.467757424	0.075372126	2.906141361	0.054048535	1.879671398
0.968520035	0.968096472	0	0	1.958302115
0.091613382	1.288096599	0.073963068	0.061814087	4.002423156
1.89259319	1.891355631	1.219277511	0	1.897318138
5.456024046	0	3.494358688	0.799129344	0
7.331677358	6.72792914	0.122313422	3.986295908	5.936927699
1.540193656	3.266405993	0.963442114	1.543369658	5.366623562
0.503418905	2.286007506	0.351854355	0.270447839	2.264690147
0.135849059	2.07486025	0.107860486	0.089149848	4.176431247
4.514677438	8.927174719	1.851346189	4.601226192	8.747864538
3.208592499	0.22339358	1.661633514	0.147819697	8.758562625
0	6.558172525	0.863108621	2.626196809	12.00082777
1.209675987	1.472820823	1.211555723	0.893290325	12.51683378
1.037713958	1.038210878	2.048232113	0.008197988	13.17601047
5.770279	0.206546512	2.735607971	0.137868796	4.705322955
1.810584015	1.189114469	2.829316868	0.040585919	10.67980507
3.653657096	0	2.500807217	0.795046845	3.246723252
3.399906507	0.133833069	2.069314362	0.092873137	2.540350206
7.710292708	1.119225112	2.741361882	0.02558591	12.5760623
1.214437578	0.064548373	2.265343397	0.046576509	10.74842298
3.618425214	2.091775411	2.108179768	1.196048286	8.64620528
1.658480826	1.272438523	0.749937979	1.290130995	2.427953726
1.637491968	3.565580002	0.735021867	0.742477593	12.29111679
0.114617065	0.108287383	1.345900861	0.076217051	7.237152473
3.901736474	1.659940233	1.061072809	1.058388196	3.089916961
2.547401681	1.166642461	0.042342134	2.190545377	12.29280804
1.090018582	2.11908118	2.439877452	1.685170015	7.156197529
0	0.850688666	0.851533131	0	0.855088011
2.357378067	1.61620476	2.032536546	0.005517134	2.844337013
6.974478496	1.443915741	0.115396068	0.09514679	1.430939433
0.210288485	2.30198044	2.258971434	0.131968008	2.758755412
6.310594078	1.522273565	0.136571635	0.11184788	1.507302094
6.367599753	0.914538567	0	0	1.482427141
0.30814552	1.859490294	3.290753086	0.182985734	3.926712304
8.13574498	2.034064538	0.098349048	0.081539484	10.92696188
0	3.090523038	0	0	4.611453713
0.241610069	1.697121907	0.184561422	0.148905316	7.357186393
6.061397262	2.43099451	0.087590867	0.072875638	2.767769967
4.157096799	3.17620605	1.272224108	1.285241239	7.201340004
3.025295302	2.816645299	1.899060306	0.556376196	10.25118563
4.211240267	1.539101247	0.141147067	1.484569601	3.531164137
0.033634385	0.032050031	2.136850142	0.023571225	6.758571929
6.671272209	1.312870343	0.781805946	0	4.915919458

3.989225302	2.023654696	2.003223564	0.079602074	2.465355486
1.491388695	2.024080149	1.794532515	0	11.2779798
7.554427532	1.61436917	0.006071505	1.02290595	11.36005368
3.415690994	0.018187026	0.015823896	0.013486828	3.091276009
7.392185378	2.231925763	3.388534052	1.369058315	2.996148686
0.095937126	7.953651998	1.293656888	4.441295095	4.02009795
6.305276489	1.02411947	7.834870891	1.022827249	1.023391957
7.394330704	6.747471586	3.72844018	4.257718309	6.834995036
6.924522303	2.210146096	4.858419451	1.289138998	4.039303245
0.000407106	1.586618724	1.001345287	0.000291815	5.324103992
0.17739977	0.166501171	0.138699831	0.113514271	12.38363541
4.545860901	1.467503674	2.19313526	0	1.470116306
2.83589447	2.827479092	2.316590721	2.251924264	7.792054836
1.416154634	1.814093626	1.418265598	0.867478596	1.418586622
0	2.900925333	2.906374055	0	3.802439767
0.043727399	1.755593112	1.749709124	1.132753818	12.90373088
2.529826018	3.023235703	1.150734268	0.032920585	3.023612848
0.265156668	1.755745494	0.200859741	0.161251389	13.26287508
4.982646856	2.723375572	1.364332528	2.345849001	1.758084552
0	1.35469653	2.066354469	0	5.147671654
7.156081757	2.158263187	0.030785637	0.026104664	1.117959967
4.623466861	1.582188197	0.152905681	0.124581963	1.565798741
1.536610243	2.468855808	1.543369048	0	12.65017359
7.479450057	0.053497702	1.177671015	1.16879697	1.797776369
3.003032784	2.995320658	2.163553448	0.112113768	2.188145659
0.981780823	2.296348809	0	0	0.98209143
4.22790863	1.964875138	0.752956037	0	13.92167317
1.070645435	4.632183941	2.679842116	2.083666599	10.6383899
6.428773774	1.961929332	3.862999384	0	4.042978468
8.32328218	5.581742643	1.344458254	3.188495895	12.83498726
0.125883534	2.042558887	0.100321866	0.08312178	2.034815206
3.227885164	0	2.638838052	0	11.67764296
2.678731016	0.320617731	3.037838552	0.202132969	2.662693864
6.172298825	2.001955717	3.702526579	1.001403922	2.809492254
0.27262691	2.475656375	0.205974268	0.165101519	3.298779846
5.718188071	0.192349832	1.580970236	0.129349877	1.58776976
8.706835643	0.87426141	0.875073564	0	2.403617953
1.048511958	2.877808703	2.061913775	0.010545164	4.398171482
0	6.21104777	0.704682928	3.966276872	1.599525389
0.583935213	4.344368613	0	2.82742542	11.97993667
3.676066735	3.099547413	0.856540679	0	7.311521997
4.429255291	1.931859069	0.074930561	1.269077205	2.368439211
1.548726018	0	0.97040106	0	3.125246943
2.09344386	4.107418065	0.017999625	0.015329734	10.96091604
6.922372197	2.646684232	0.672761048	0.681060557	0.681839338
0.826538587	3.627015869	3.644501006	1.381256376	3.420777028
1.571513111	6.828095997	0	4.892507912	1.571712037
0	1.644999468	0.741707974	0	6.409037612
8.766013343	1.568462706	4.764502606	0.970446385	1.304867728
0.40372375	3.393035441	1.127204491	1.862390185	8.18206707
7.777716791	1.039805264	4.962968515	0.00854012	2.376097179
1.840841946	0.5245035	3.207621528	0	2.770931059
4.055056528	6.588032162	0.880889078	3.539767541	11.96375301
4.861463822	7.646300966	4.005375621	5.937745976	12.59916954
2.606345789	1.514050065	0.650664257	0	3.008603329
1.049768208	2.066190333	1.049762678	0.010818232	11.94780211
0.025040264	5.295236941	2.693751057	0.017650068	1.079764973
0.796278126	1.328604363	1.72667037	0.800601289	3.47474737

2.538873168	0.272730507	1.783832866	0.176018719	6.107660134
5.093926707	1.4528558	2.836423882	0	3.07561449
0	2.050201951	2.315989253	0	3.59992567
5.841682705	2.457274477	0	0.868238914	2.336260182
1.095709959	0.028221864	1.095492242	0.020803096	4.235386056
1.333223488	1.336930865	1.328269249	0.072274657	9.195797887
6.37569148	2.843265371	1.117932461	0	1.124100527
0	3.115029023	0.420612985	2.094840028	12.57913984
0.006044776	2.02651559	1.608724349	1.019048543	2.836314183
8.944938405	0.050547314	2.211156858	1.159913975	14.13279238
5.630812507	8.104150313	1.845950706	4.973188998	1.220423286
5.164643021	0.040024356	2.169278087	0.029297361	3.195897649
3.419206801	4.333100712	1.290242092	2.330137122	13.36778646
0.009555063	0.009128277	3.629211313	0.006806245	1.622664332
0.028496547	2.121926906	0.023574825	0.020039579	3.134931231
3.172453559	1.26101075	2.64832888	1.241451452	6.041785875
6.384837816	3.200816989	2.70652711	0.703013627	3.0869678
0.047501993	1.153691161	0.03900709	0.032983739	2.196728918
0.640870897	2.420039621	0	0.644386427	7.052362135
7.400487051	0.679082136	0	0.687453393	2.805461275
1.220134038	0.066327861	1.218242398	0.047811324	1.84613686
5.530700578	1.09237774	0.61014211	0	2.539871608
7.350168699	1.747441186	2.986040426	1.732069482	10.8958028
6.81992411	1.141331315	0.035831148	0.030330883	3.528839465
0.167726901	6.728209329	0.131604466	4.64864147	12.33538354
1.067632022	2.415565882	0.017257074	2.080157028	5.723110224
1.922640591	1.922418867	1.925125556	0	1.515231666
0.576104387	1.039039901	0.569465207	0	1.926501675
5.05074371	1.003999163	0.001001352	1.003791879	3.00586605
0.330476151	0.305260118	0.244694752	0.193886476	6.727307852
0.216671252	2.320399338	0.166999998	0.135468672	0.222955492
7.911981552	0.75152915	0.752151469	1.292853148	1.968405366
5.92058811	1.652177479	3.777487821	1.052345738	4.545690348
5.426583689	0.118443422	1.375703797	1.353056899	13.61033225
2.098041763	0	1.563467362	0.68635891	2.102870437
4.493824808	0.018969189	1.064631688	1.061778932	2.084678693
0.062554034	0.059425123	0.051068539	0.043007257	3.45793736
5.7283737	0.645225902	0.645073072	0	2.745997894
0	0.845246837	2.945497254	0.851221607	4.390408988
5.67840269	1.817116044	1.15723068	0.664743556	14.28583884
0.840182327	1.384730211	0	0	1.784692539
4.818176665	2.750927057	0.277761918	0.217980123	6.905274656
0.901582305	2.181853456	0.90097401	0	4.088499718
5.636806871	1.955778464	0.080358053	0.067017015	3.007624706
2.931369669	0.95397169	1.530097624	0	11.42643402
0	2.919709595	1.745579086	0	1.740151764
3.982651943	0.082029898	2.931683826	0.058599055	1.264892434
6.167146693	2.303256411	2.287924833	0.050706629	3.50767563
7.098192523	0.209183416	1.625089668	0.139437545	2.332007868
4.906971649	6.86250513	3.249652326	3.402753046	11.24391202
1.748878116	2.056809142	1.754867573	0	11.49564396
0.906525941	2.108716227	1.519982427	0	12.67213791
0.121675828	0.114870935	0.097121981	0.080554311	4.717965505
1.683056947	1.687839928	1.657831855	0.14694659	9.603618991
4.920693083	3.495434143	0.092994991	1.329788752	3.06944484
2.78042768	0.105285989	0.089266773	1.317382131	6.789135168
1.530948235	6.290758801	1.979255444	3.972696267	12.04603011
7.457483577	0.127400641	1.401651833	0.088722791	3.138323388

hsa-miR-135b-5p	hsa-miR-4739	hsa-miR-206	hsa-miR-199b-5p	hsa-miR-487a-3p
1.418656815	0.098715365	1.406172615	9.68730518	2.85763264
1.025716224	1.614555313	1.615355272	2.844613044	1.616137687
2.074857917	0	0	6.568100739	4.066200351
2.598039224	0	0.860622089	8.049453719	1.412332532
0.017303423	1.652160376	1.058037301	4.735899327	0.016292788
2.159870984	0.028570683	1.727628057	8.524713788	1.731837854
0.259547189	0.183725871	0.204681526	1.760540922	0.236603823
1.909384518	0	1.036919705	8.043436405	1.031703797
0.764895035	2.002977156	2.248389365	3.521331323	0
7.001887798	0	0.731988197	8.737971467	3.035181517
3.574946447	2.035685845	0.108108046	8.059727083	1.416796497
2.792803775	1.337618041	0.091781255	8.658109804	2.782342056
0	0.951567933	0	3.623218218	0
1.251414338	2.294277573	0.064217184	5.135272455	0.071725621
7.098995625	1.547986436	1.547048526	1.958041015	0.968043651
0.088437638	1.273923996	0.07366996	2.704414984	0.08247339
3.319992438	0.707834845	1.904535684	5.550073164	0.702962694
2.680103806	1.731612438	0.792846431	3.129341719	0
0.149128666	0.110779858	0.121781673	2.131804036	6.310942631
0	0.964092648	0	8.163537178	2.532851807
3.518867914	0.307072042	0.349708249	4.197683291	0.418933455
1.415373648	0.097932507	0.107403911	8.655229931	2.515059175
0	0.888179349	0	0.888194827	6.010595182
0.22891448	0.164175038	0.182252321	5.499095044	2.372018988
0.862683049	0	1.418798155	4.680569715	5.760035037
3.003167552	0	2.256632216	8.441234678	2.076842116
6.209412167	1.036986171	1.037702388	5.893872465	1.038246599
4.972405583	0.152840898	0.169320033	1.633190713	0.194037219
3.441672012	1.796241796	2.231971051	1.808799011	1.188958329
2.270684824	0	0	6.894054979	0.7871302
3.395104745	2.05054066	0.112054311	5.848330861	0.126625138
0.035447966	1.114764727	1.117230853	1.116542631	1.727776024
1.216611277	0.050751648	0.055175796	1.841381191	1.216778684
1.548934863	0.678055704	0.674619922	8.27615645	2.095361463
0.750111501	0.753088813	0	7.873336877	2.214603377
1.25396142	0.738270865	0	0.740403819	4.32172185
0.11046808	1.336133348	0.091354672	0.116533394	0.102713431
3.26096376	2.399551625	1.061025508	8.960229142	2.080404798
2.216103783	1.159869651	1.163507107	3.411701504	0.04686546
2.119286305	0.021209625	3.293498935	6.334049597	2.444559912
1.400861471	0.853759644	0	2.77358712	0
0.007511055	2.031485563	1.025387839	5.210783716	0.007081819
1.443486297	0.104638764	1.429650255	2.102578053	0.129931986
0.201342338	2.229585991	0.161697339	2.29846604	0.18495603
5.435901011	2.138518757	0.135962549	2.999071518	0.154542879
0.914782387	1.892789516	0	5.217905321	0
0.293053183	1.779787955	0.228739102	1.846732054	0.265980174
1.379745607	0.089443365	1.369230935	5.517864826	0.110294868
3.463788012	0.853123735	0	2.772581875	1.400246132
0.23083245	0.165413989	0.183669108	4.664398447	0.211222615
0.105306546	2.722056021	0.087234215	7.569241981	1.338769493
2.604249207	1.278270885	0.746076831	6.825342453	1.652456848
2.694661525	1.021590742	0	9.066236533	1.008876634
0.173415247	0.127428175	1.519110007	10.93802072	0.159892502
1.109583491	3.311770723	1.108046433	1.715670657	0.030631194
0	0	1.317666763	5.434999535	0.780348403

1.370683594	1.987714587	0.095539837	4.938115948	0.107528734
1.122889525	0	0	7.633995877	0
1.024140294	1.612749305	0.006050775	4.896255929	1.024229045
1.062700296	3.252158027	1.061955158	0.019316254	0.017405992
3.344102166	1.355696273	1.630594828	7.814551293	2.792297442
2.721569559	1.285800693	1.293344366	1.294385891	5.915673176
3.73702387	1.023368359	1.023812362	2.355012104	0.006640309
3.283175824	0	0.630688865	4.000848635	5.940046447
3.293154127	2.640510948	1.971109788	6.41061263	1.656939712
0.000395485	1.586530378	0.000339404	3.461497094	1.586611577
1.529826366	1.494510398	0.138078932	3.52324123	0.157029763
0.904636192	2.869782145	0	6.55585484	2.188976536
1.674724793	0.159973529	2.315158268	7.597744989	1.668976898
1.414938555	1.422047769	0	5.959393532	0.861602039
3.069345909	0	0.93394328	7.437000133	1.503571116
1.755645208	0.033064653	1.139321946	3.008641473	0.039742459
1.769397226	1.147301905	0.038788094	7.110600528	1.768092601
0.252917921	1.691817453	0.19986329	5.969616906	0.230761095
7.166574911	0.821847583	0	5.9411812	3.303666328
0.814677645	1.761414949	2.066665687	1.358469961	0
1.121363457	1.722372156	3.162615545	4.355600654	0.033982349
1.582047707	2.638898921	0.152204144	3.58920815	0.173693045
0.665232585	0	2.305131182	7.435087883	1.162437369
1.180653708	2.214551909	0.045883629	5.031819529	2.231199308
0.16792048	0.123692744	0.136300056	0.178225075	1.520560242
0	1.977177797	0	0.981956938	0
1.964572743	2.240632498	1.669776371	7.715343573	1.276537808
0.021085433	2.676118897	1.070513513	1.670357759	4.192754634
8.882563279	0	0.922800907	6.808050508	0.479319747
0	0	2.05003679	1.344123718	5.326790065
0.121232303	0.0912064	1.376273433	6.645679745	1.386252723
3.869850331	1.840300835	1.155286315	5.672602469	0
0.329793047	1.856392478	0.254567337	1.939014223	0.297898794
1.001476414	1.001436371	0.000369325	5.646234095	2.32395003
3.298362917	1.707482374	0.204944317	4.871939806	0.236922882
0.196865271	4.783557519	0.158326167	3.38195429	0.180950469
0	0	0	3.127664894	0
1.644539224	2.059872467	0.012314879	1.048033837	2.063949597
6.273672271	0	0	3.750184249	6.223115033
0	0.581248211	1.416226526	5.722877425	3.452225814
2.374027621	0	1.410699215	8.000232629	0.855314186
3.211238893	0.068423891	0.074633039	8.479253705	1.291140667
1.548486265	1.550325355	3.128117802	3.276620332	0
1.071267598	2.411300857	0.01793642	0.0219969	5.150498331
0.673718302	0	1.178836104	0.67984973	2.933505999
5.616609418	0	1.371621945	5.658230287	2.903026614
6.552278914	0	0	2.569154654	6.172922547
0.74195235	2.223869334	0	6.02798623	0
0.950678999	0	0.50193588	1.576803244	0
2.119201556	1.409897497	0.393253687	9.688375967	2.719623993
1.63324985	2.048587164	0.009965414	3.058292206	1.633024313
1.324500531	0	0	1.333128728	0.522321231
3.137332283	0.882760999	0	6.939406843	5.303899375
3.149702307	0	0	7.554520237	7.890916865
3.925733352	1.834368713	0.650785729	7.441107695	1.515343863
1.64607537	0.011706949	0.012635143	9.64511521	0.013936543
4.447143437	1.678485189	0.020670323	5.486981643	4.979689372
2.279406881	0.797054336	0.794349814	7.224053751	2.85398429

0.280009672	2.4218039	1.782464768	2.532489234	1.817048535
3.741511426	0	0.996750906	8.905315997	1.641966605
1.741456824	0	0.810435712	3.598965453	0.808942221
3.126236066	0	0.42736832	8.48075642	1.174424812
1.702028414	2.931421256	1.698117465	5.10544324	0.026982811
6.778747055	0.079141048	0.086494253	3.646585741	1.980264354
3.451069564	2.044996616	1.118211379	8.502583523	2.016596261
0	0	1.674237647	1.85757277	2.183223405
3.615375651	0.004664845	0.005028071	9.667343447	0.005535724
1.170978994	0.03999286	0.043392181	5.512427465	1.789158996
2.62666796	1.217080922	0.057829044	4.589007327	6.302817307
2.507228023	0.031817648	0.03446954	3.522736424	0.038222082
1.938738805	1.282505284	1.289934495	7.234237937	3.731169641
0.009276426	1.6213394	4.366524129	4.957023511	0.008744194
0.027629222	3.443521981	0.023490976	3.460306014	0.025978918
3.17033138	0.061138954	1.254969287	3.543200034	1.260551351
2.830313148	4.239150037	1.587983765	7.455827247	1.202255941
2.530045972	5.105580528	1.150888074	2.529212641	1.153631601
3.383654006	0	1.130790893	6.38065295	2.73484007
1.555759571	1.193855764	0	3.546662684	0
0.067553653	0.052109493	3.095951669	0.070941037	0.063175593
3.420841583	0.61387442	0	5.366430154	0.608980784
1.747484713	0.031458157	2.16717269	7.341632867	0.037784098
0.042197483	1.135832147	1.138830796	5.711903986	0.039598508
0.161063603	1.471125522	1.486206391	5.430953261	5.634469702
3.561460805	1.066181754	1.665247975	8.376304186	2.903663633
2.913222768	3.242643266	0	5.035016868	0.94163556
3.257287733	0	1.404193278	5.753252805	0
1.003988614	1.589569486	1.589691124	6.774651212	1.004004339
0.313806896	2.945282399	0.243398574	0.338363665	1.900237191
0.207362286	3.295821362	9.254042305	3.135334151	0.190334529
2.214247149	0	0	6.031013153	0
0.016293159	1.053629549	1.054692099	2.663071757	1.055511241
2.042145162	0.090953374	2.019018669	2.037094308	0.112234236
1.850776051	0.681661222	1.563848595	4.528856873	0
2.901423772	0.015224862	2.407012563	2.677097014	1.065569236
0.060507175	1.191785358	5.313817379	3.880409848	0.056639743
1.506225753	0	1.516060446	3.107949927	0
6.565675236	0	0	8.558775924	2.104372039
1.522526268	0	1.157501811	8.407827553	1.152184127
1.384963131	1.793196018	0.838916063	2.920312938	2.924879785
0.361223377	0.245213288	1.961795384	4.681127731	0.324964638
0	1.467994337	0	6.063865166	2.442326235
0.096330693	1.296465459	1.304384156	3.909455115	0.089738746
3.51438646	0.955201523	0.954430381	6.28238476	2.519867872
2.178640568	0.605487363	1.746110831	8.667799875	0
1.272623332	2.317732722	1.266619625	6.029925574	0.078009793
0.071829382	3.465024479	0.060166827	5.718333622	1.235950905
3.434207145	0.15462505	0.171352319	6.458130131	0.196463997
2.784238932	1.331378776	0.089990014	8.180374164	6.57541791
0.814568975	2.073734739	0	7.175514972	1.354938647
4.015365123	2.826212164	2.135670293	8.405167798	1.738767781
0.117214138	0.088346183	0.096719147	1.367726168	0.108887367
1.688099073	1.633299412	0.181113716	4.960075117	0.208153403
0.112046974	6.110960718	0.092612329	5.1768807	1.358974939
1.345568554	1.327582981	0.088902104	5.516194928	0.099896122
0.917250984	0	0	3.631102325	4.9382564
1.41337299	2.031010533	0.106871371	2.864100034	0.120615444

hsa-miR-499a-3p	hsa-miR-4784	hsa-miR-380-3p	hsa-miR-506-3p	hsa-miR-346
0.120499419	0.083421248	0.11031473	0.09228617	0.088803481
0.007020161	1.023900305	2.355910098	0.005655891	0.005477301
0	0	1.354546632	0	1.391786118
1.412584603	0	1.816101612	0	0
0.016105604	1.64835453	0.014991227	0.012921242	2.392620077
0.033861219	0.024656514	1.72839116	0.02694597	0.026053406
1.762431805	0.151592577	1.739917699	2.347274358	1.674114106
0	0	1.035705837	0	1.963742799
1.679742554	0	0	0	0
1.633105659	2.448543564	2.411283055	0	5.234461046
0.120305787	0.083296252	0.110140801	0.092145533	2.015469472
1.355168158	0.071213274	1.348745635	0.078583584	1.95636197
1.934486587	0	0	0	0
0.070796437	0.050429461	0.065322632	0.055409842	1.232316289
1.546229641	0	0	0	0
5.761014271	0.057612632	0.074963416	0.063396895	0.06113212
3.226658214	0.71641495	0.704043564	0	0
0	0.802323002	0	0.798052432	0.799614796
1.466217933	0.09329002	4.295611806	0.103411982	1.423301501
0	0	1.540868414	0	0.964973057
0.409771412	0.245426116	0.359255128	2.082225145	0.26628861
2.067437274	1.368234893	0.109420699	0.091563112	1.377001253
1.847777044	0	6.611384679	0	0
1.68370371	0.136188306	2.338913177	1.619008232	0.145912403
0	0	5.185760198	0	0
0.444844049	0.902487926	1.484710661	0	0
0.010422686	0.007699091	0.009710449	0.008383939	0.008117535
1.637854958	0.127185407	1.621343774	0.141953997	0.136118976
0.052696363	1.172063527	0.048762481	0.041578511	0.040157544
0.787134421	0	0	0	1.338141611
0.124802424	0.086192106	0.114177204	3.300261431	0.091784647
1.119106091	0.023968888	0.030538604	0.026190708	1.111450827
1.841703873	0.043503309	0.056107459	1.203348971	0.046078595
0	0	1.8571649	2.131352958	1.197145691
1.273014131	0	0.749589875	0	1.993106288
0.733512783	1.277903173	1.948471898	1.269603343	2.227206294
1.353572713	0.070895341	0.093017147	0.078227621	0.07535175
2.080141462	0.012466317	1.657939104	0.013588909	0.013151952
0.046289072	0.033454687	1.778709035	0.036628652	0.035389833
0.0250598	0.018345555	1.090075237	0.020022397	1.684747383
2.112861553	0	0.851275809	1.411593326	1.413513123
3.036710541	1.023839525	0.006527313	0.00564146	4.61765447
0.12805045	1.391763814	0.117089303	0.097752706	0.094026914
0.182013059	0.121836226	0.165048568	1.556149256	4.5702714
7.265028922	2.532647145	0.138655238	0.115009912	0.11049077
0	0.919811531	0	0	2.215154415
5.163073826	0.167829737	1.818736174	0.188831305	0.180490454
1.378150439	0.075790778	0.099745794	0.083713661	2.412365326
1.40050876	0	2.116612659	0	0.856266195
2.373286557	1.596400583	0.187619401	0.153402628	0.146980601
0.096645152	1.303629735	0.088808701	0.074785017	1.939569479
0.744462406	0	1.964715171	0	0
0	0	1.368759175	1.028364473	1.03259191
0.157452314	1.470263616	0.143318216	0.118712738	0.114018294
0.030267629	1.100856977	1.10842941	0.024125799	0.023331569
0	0	0.78147954	0.787296091	0

0.106034617	0.074009605	0.09729293	0.081716353	0.078692379
0.631516369	0.646191777	0.632508602	1.516489718	0
0.006588489	1.611217694	1.613635413	0.005309173	0.005141666
0.017205505	0.012656408	0.016012175	1.655068295	1.059081694
0.529248088	0	1.342733952	0.537682984	0
2.376487983	0.060142657	6.375234333	0.06621563	0.063837028
0.006565723	1.022362642	0.006120879	0.005290883	0.00512396
0	0	6.293184075	1.81339113	0
0	0	1.969398485	0	0
1.001361359	0.000274371	0.000344455	0.000298307	2.586649883
0.154644953	0.105055992	0.140824014	0.116733363	3.750377874
1.468035489	1.478478298	0	0	2.683839609
0.200421089	0.132857278	0.181229711	0.148452751	0.142286533
0	0	0.86255421	0	0
0	0	0.933767517	0	0
0.039261163	0.028495442	0.036408516	0.03116648	0.030124631
1.153162482	0.030807818	0.039419875	0.033711959	0.032578846
0.226816442	2.294668209	1.723956036	0.166224014	0.159125253
0.817922249	0.827938704	0	0	0
2.315154607	0.823756146	0	0	0
0.033575992	1.111543462	0.031164874	0.026722466	4.237372162
0.170979996	2.586102863	0.155307013	0.128187211	0.123036147
0	0	1.542083974	1.180466866	1.184086336
3.055014222	1.164864606	0.046642859	0.039797821	1.779660222
0.152594662	0.103779265	1.507482971	2.122938402	0.110752787
0.981518869	0	0.981687416	1.564053254	0
0	0	0.752609736	0.758889695	1.997344658
0.01961382	1.065999674	2.680378226	0.0157104	0.015202799
0.479029378	0.494052432	0.479387653	0	0.489616544
1.34075932	0.812571532	3.028539271	1.353541262	0
2.035920394	1.344434281	0.10175211	1.357389836	2.419717474
1.521742755	0	1.154131814	5.240475769	0.663726758
2.63775672	0.184947312	0.260678931	0.208785179	0.19929743
0.00040166	1.001375954	0.000374821	0.000324601	0.000314479
0.232835357	0.151771517	0.209506761	0.170225434	0.162911335
1.598604684	0.1194603	1.583782791	0.133125309	0.127731685
2.814165882	1.444032719	0	0	0
0.013427172	0.009900069	0.012503537	0.010785718	0.010441104
0.703180687	1.240686421	5.023174776	0	2.587595354
0	1.748404018	1.414380036	0	0
1.807058283	0	0	0.860586397	0
0.082462485	1.262267927	0.075946011	0.064208285	0.061910857
0	0.972116373	0	1.963827933	0
0.019579895	0.014382026	2.903910662	1.067814153	0.015176771
0	0	2.502357325	0	0
0	0.833441507	3.323024632	0	1.782248983
0	0	5.711906954	0	0
0.740201561	0.752800229	1.65262038	0.747797953	2.811304462
1.292282977	1.628185805	0.95164729	0.509283155	0
0	0	1.392781861	0	2.203141205
1.632918111	1.627999258	0.01011722	0.008733992	0.008456241
0	1.662523728	0	0.995973692	0.532915888
0	0.887079139	3.147737312	0	0
0	0	7.608751638	2.85091892	2.441176991
0	0.663696444	0	0	0.659867755
0.013777302	1.046611158	1.049885853	0.011065178	0.010711409
0.022577448	0.016553391	2.694370984	0.018059598	0.017473011
2.029402347	0	0.793934277	0.799530161	0

0.250067367	0.161582186	0.224428989	0.18157948	0.17364377
0	1.525958842	0.314773871	0.321624353	0.709703133
0	1.368372615	0.810039343	0	0
0.846588074	0	1.181165001	0	1.490079762
1.701056125	1.089146654	0.024777866	1.09181516	0.020592903
1.978855175	0.06726472	0.088053067	0.074165961	4.389633394
0.624742196	0	1.78221856	0	0
0.837420609	0	2.593347457	0	0.430324484
0.005473708	1.606807204	0.005103754	1.019181836	2.346340139
0.047625839	0.034393284	1.168732616	2.197923125	5.730613918
0.063666992	1.821237031	6.006903059	0.049988283	0.048249939
0.037760735	1.124968746	0.035025544	1.128843501	0.028995652
0.08409573	1.267082664	3.710811301	0.065432922	1.27295612
4.439343665	0.006393332	1.622509016	1.030216687	0.006739669
2.44696333	0.018787987	0.023860464	0.02050718	0.019837451
0.073457327	0.052243418	1.256079249	0.057424593	0.055397375
1.580319725	0	1.586395794	0	2.57263694
0.042614113	1.747185021	0.039496594	1.144830448	2.501966994
1.125733514	2.088240513	0	0	3.371912648
0	0	0.678850921	1.199591737	0
4.959836092	1.201904471	1.218935056	4.441909261	0.047298877
0	0.623741486	0	1.110972468	0
1.134815136	0.027124317	0.034627	0.029658293	3.750220598
2.180828934	1.129304681	0.036277608	0.031055715	3.873293301
0.14652112	0.099980785	5.221777183	0.110979693	0.106649325
0.018769307	0.013793485	0.017463101	0.015039804	1.064364389
0.941661377	2.513346424	0	0	0
0	1.984020258	0	0.576882788	0
2.00522247	0.000806503	0.001012848	0.000876961	0.000849578
0.27880439	0.177584298	1.866070106	0.200187005	0.191199189
1.626796695	0.125010601	0.169687055	0.139465861	0.133756236
0	0	0	0	0
0.015167751	1.051249182	1.054870643	0.012174089	2.064797869
1.38440715	1.974162601	1.377159111	1.991680332	0.081941399
1.854557451	0	1.562211123	0	0.687008691
0.017943692	0.013193433	1.064795501	0.014383711	0.013920364
0.055928521	1.79444789	0.051726635	0.044064728	1.185814567
1.803258819	1.166206885	0.64469262	0	0.654333074
0	2.388710918	0	0	1.407066521
0.654841041	0	1.156349362	0	0
0	0	0.838558818	0	0.844543685
0.318627409	0.199057826	0.283061197	0.225326079	0.214852551
0.900042494	0.90625225	0	0	0
0.088533696	0.062416855	0.081459183	0.068751817	0.066269864
0	0.956994108	1.529913923	1.532564369	0.956288577
0.600469235	1.485553518	1.450586446	8.649595997	2.024049549
1.272136088	0.054640564	0.07096413	1.254548885	1.251341355
0.06627382	1.830152543	0.061193691	0.051974594	3.084362236
1.64508278	0.128606182	0.174962631	0.143580584	2.668577863
0.099756911	1.940082572	4.457049007	0.077088322	0.07426046
0.813459837	0	2.065161245	0	0.821170153
0.897827817	0	1.240824923	0	0
0.107370497	0.074885166	0.098498	0.08269798	0.079632039
1.679680698	0.135399265	0.184992602	0.151369878	0.145053272
0.102721316	0.071832346	1.993444033	0.079276835	3.872431103
1.344390756	1.309045833	0.09051206	0.07617946	2.37811514
0	0.491170355	3.623909629	1.282955069	0
0.11889818	0.082386797	0.108876107	0.091122478	0.087691026

hsa-miR-483-5p	hsa-miR-668-3p	hsa-miR-708-5p	hsa-miR-508-5p	hsa-miR-380-5p
3.378454965	0.074232457	7.012487663	0.109567323	2.864007256
7.735158006	0.004704005	1.025728867	0.006508396	2.357256091
10.88756841	0	5.23988925	0	3.008773592
0.863668837	0	4.580490556	0.860412868	1.411973086
10.95033114	0.010715304	1.656277251	0.014907921	0.016720125
3.641094402	1.108313242	4.829642747	0.031246863	2.754580956
2.942495181	1.621562066	1.773837058	2.868690888	0.246112366
2.73037745	0	3.876819957	0.56424489	1.910879468
7.695208749	0	1.677725451	0.764658259	0
11.44125541	0.749852587	2.581231043	0	2.397754734
7.100650929	0.074123177	3.574330919	0.109394814	0.126064224
9.445181338	0.063533055	5.271540514	0.092839677	1.356855827
3.383353799	0	0	0	0.950172645
1.244692709	0.045192312	2.323466718	0.064917371	0.073857606
0.969009909	0	0.968167301	0.968374082	0
2.976414856	0.051549257	1.287721563	0.074489126	1.288117767
0.712115682	0.72350898	2.535660986	0.70421269	1.214091841
0.79775852	0	1.326977878	0.79257539	0
4.673198227	2.457613873	2.589842735	0.123268869	6.175596219
5.399738854	0	4.799817049	0	3.751212023
0.524229348	0.211667981	0.469966172	0.355731402	0.440671714
2.864078626	1.351979969	9.041079842	3.100015233	0.125215334
8.328055612	3.947674978	0.885866783	0.886264809	8.022614335
8.654715318	0.119885746	3.480846358	0.18472529	2.380491349
11.36288518	3.530722621	2.791546653	0	6.119124892
11.04426857	0.469143214	4.982182027	0	2.497737054
12.30193944	1.034147836	2.050304766	0.009657152	1.631276817
3.664828099	0.112162303	0.21075784	0.171568654	0.201308979
10.3307273	0.034092875	0.056825361	0.048470296	0.054886087
2.84055115	0	3.975150573	0	1.71144331
0.145518735	0.0766535	4.191666582	2.070728459	0.130828674
11.20608028	0.021605198	0.035357253	0.030362772	0.034204163
11.29926352	0.039044374	2.279795295	0.055765981	0.063279597
8.622039147	0.69466938	5.245332887	2.671235627	1.548949871
2.427565001	0.767108757	4.864664514	0	1.272323036
11.08568688	1.682390727	1.937588649	1.643737575	4.688912166
7.121424295	0.063253692	0.110137987	0.092407289	0.105967689
3.552113545	0.011265978	5.877234392	0.015682025	2.406264534
10.40787627	0.030092119	2.547088483	0.04262376	1.16675516
6.744625577	0.016557219	4.723913469	0.023160227	2.44556333
1.404581096	0.863094346	0.851052982	0	0.85040905
0.007877023	1.611683108	2.621423814	0.006491756	1.616169371
1.431711231	0.078471627	2.558962421	0.116285994	0.134272521
3.11154512	0.107560841	0.200636039	0.163817154	0.191780646
1.508240003	0.091747442	0.166925994	0.137666412	2.192922837
3.040711949	0	3.191449905	0.915122409	1.885989705
1.839898895	0.146827627	0.291871229	0.232078504	1.856293834
9.624200119	0.067551184	3.32648502	0.099083318	2.032491467
4.278880231	0	1.400009071	1.80340351	2.774245126
7.392099509	0.120725628	2.393730141	0.186167099	1.695387298
1.33018026	0.060548145	4.185124668	0.088231177	2.428797637
6.834154836	2.008363233	3.287968099	0.745776822	3.292047688
10.21195298	0.570394133	5.332822424	2.286676787	1.881522969
3.297225499	0.09457526	6.867867015	0.142287771	1.538343971
6.728193063	0.019918055	1.717479338	1.713375182	0.031462273
3.960620823	0	3.731242378	0	0.780610104

3.515682022	0.065988572	3.314772801	0.096649766	0.110968316
10.16901785	0	4.88282465	0	2.234268245
10.67676087	0.004416256	2.031918758	0.006108652	2.355099848
2.407305689	0.011437275	3.091428899	2.079177112	1.062926746
1.346728011	0.553549528	3.560982487	1.629025738	1.852105982
3.752054524	4.125712472	1.300501244	0.077880141	7.420701297
1.023426278	0.004401075	3.035338126	0.006087569	0.006810439
7.028143819	2.822482322	1.484926353	2.247878888	7.16882614
4.039022247	0.766339771	4.335608079	0	2.211234361
5.494034823	0.000248639	2.809329264	1.001346935	2.001792584
10.38755402	0.093064527	0.169671639	0.139815897	1.529391343
1.469963769	0	3.312077039	0.90498167	0.904197781
7.396618756	1.54998055	3.459955091	1.647348244	0.211524733
2.128877794	2.158660388	3.949581177	0	2.384639633
3.220469484	0	3.703885501	0	2.489557798
11.19995406	0.025659506	1.755655409	1.750076017	0.040843567
1.765901689	0.027727524	3.683940624	0.039188401	0.044257877
10.79353116	1.609093388	4.168361052	0.202653498	2.449906777
1.757791979	0.8328196	3.708317744	0.819119729	0
3.785765595	0	1.747837293	1.358596883	0.813745385
1.728765235	0.022039231	1.121418539	0.030985185	2.486084768
0.202255011	0.101778201	0.188118411	0.154167117	0.179977741
12.26155784	0	4.232100943	1.16699952	1.533957754
1.175837015	0.032655374	2.2343247	1.177955751	1.801599015
2.649225788	2.503652289	2.198591898	0.138009185	2.194319607
0.982065773	0	0	0.981706136	2.296443077
11.89087359	0	2.929135625	0	0.751803093
9.341133703	2.077341268	0.021033207	0.01814419	2.908913805
4.208482017	0	4.896875425	0	2.296463986
11.28658431	2.342560158	2.039598013	0	4.926976593
1.37682665	0.068825932	3.105693116	2.022067595	0.116195808
10.72446375	0	6.664115172	6.466724649	0
0.359489614	0.161276326	0.328393402	0.258428239	0.311037736
0.000450951	0.000270553	2.001957864	0.000372809	0.000416401
0.280890211	0.133193259	3.578236754	0.207828554	0.246449068
3.614607809	0.105514008	3.375625152	0.16038983	2.284518253
0.877819176	0	0	0	0
2.653950286	0.008952011	3.532844058	0.012434458	1.64447425
1.59906605	3.697411593	0.70511959	0	5.651839959
11.37484941	1.093709886	3.182940101	0.577275807	4.470704943
7.166431711	0.867777555	3.234589579	0	2.950644155
1.926035225	0.052192929	5.88889032	0.075464553	2.370355799
2.280794433	0	2.280354504	1.549343212	0.970059921
11.07892679	0.012991621	1.671418287	1.070544946	3.102621849
0	1.213057513	0.673534533	0	3.268809386
4.836548385	0	4.467971614	0	3.05039711
0.989206433	4.074411921	2.98360888	0	6.77722595
4.768072952	0.759253315	3.661596781	0.741517179	1.94871107
1.579777306	0.525421291	1.568705057	0	0.948363049
8.048550365	0.416830144	5.808091647	0	3.948763108
1.038660531	0.007255073	2.052393423	0.010061638	0.011269156
2.651918479	0	6.227028876	1.332359657	0
10.93947271	2.854670135	2.819194763	0	5.978782893
10.64033468	3.448335232	3.631543108	2.429414635	8.465805298
1.154753018	0.671582573	4.154666925	0	2.264222902
10.49275233	0.00918293	5.778711018	0.012757925	2.065993466
1.079885718	1.073051966	3.707607139	0.020875022	4.70832721
3.255895181	0	5.211719208	0	0.793048795

5.536474349	0.141532429	0.278901263	1.786259357	0.265097544
1.653101368	0	5.424540543	1.249821136	1.805091846
3.396219469	0	0.809998219	0	1.348887275
2.044412653	0	6.346884017	0.427098326	2.332414293
3.603653688	0.017596087	2.454201269	2.713149946	0.027708699
10.75254537	0.060060905	2.767194458	0.087481301	0.100176616
1.487218205	0.64768902	4.930603154	0.625888302	2.222745359
11.62930581	2.713614113	1.165121227	0	4.347178284
2.613327867	0.003672248	5.914125555	1.019906102	1.609389666
12.37764091	0.030929964	3.048838399	2.211732659	1.171454298
0.072855596	4.830202134	1.226934494	0.058451124	8.072845482
1.746179729	0.02470707	1.749322421	0.034821853	1.74896682
11.48656168	1.874922483	3.589078861	0.07693784	4.746249636
1.030812854	0.005785681	2.629896712	0.008012968	1.623438454
2.71519356	0.01695484	0.027559754	0.023725137	2.448508946
7.759278633	1.841598992	0.079491184	0.06732673	1.261066722
4.004489419	0	3.303546295	1.206730873	2.522706949
1.766245076	0.027780105	1.76975622	0.039264629	1.153804289
6.465415699	0	4.228117473	0	1.125292081
3.17083062	0	0.679929023	0	0.678351743
2.890194065	0.040062508	0.067368373	4.205275284	1.850616989
2.379121124	0.632122718	5.399336969	0	0
8.544228511	1.118987479	4.108031281	0.034425804	0.038826282
3.668084253	1.124470993	0.042087868	1.749501225	0.040695268
10.168477	1.421155343	2.630946876	0.132650052	6.553332491
5.237042738	0.012461629	4.426414116	0.017365501	3.098471235
0.943410326	0	2.50116567	0	0.941654451
2.739734553	0	5.012342647	0	1.9194029
2.005211337	1.003591063	3.175868066	1.003959147	1.589825228
6.216094595	0.155071882	0.312503884	0.247030481	0.296324019
1.617213901	0.110292627	0.206627726	0.168411253	0.1974227
1.968009824	0	3.185226021	0	1.660149833
4.255538642	0.010098724	1.055358013	0.0140421	1.055534132
11.57933214	0.06864311	1.386206854	1.376478556	0.115857761
2.494844978	0	0.678833912	0.677915568	1.180238074
1.0636418	1.058335087	2.677257604	0.016604018	1.065600845
4.762793626	0.036096649	3.088471469	1.196831003	0.058269373
2.049595548	0	1.506135609	0	1.137442679
4.39025003	0	5.723622354	0	1.393861775
12.28060035	0	4.958869543	0	2.457249727
0.842538052	1.408594778	0.838382025	0	0.837674233
6.872722838	0.173122943	2.757664591	0.280540878	0.339861173
2.18323478	0	2.440377482	0	0.900067201
1.303550407	0.05579014	1.312000708	1.30528983	0.092508295
10.96511434	0.958381488	2.257757451	0	0.95390065
0	0	4.346297833	9.772609347	3.034563048
1.904568616	0.048921391	3.383198406	1.267376258	0.080359536
1.862138391	0.042446432	3.333526904	0.060817276	0.069111506
0.230247829	0.113382908	2.80582224	0.173635711	0.203857036
11.35768145	3.534574867	3.659435648	0.091024113	4.954684276
11.55929308	1.381249742	5.57514232	0.814669855	0.813636083
11.91830142	0	5.428021883	0.901483475	0.898278146
5.728808912	0.06675684	0.116859365	0.097845366	0.112379771
9.318297706	0.119209885	0.226543246	0.183566639	2.375968722
2.794117907	0.06407692	1.359734992	1.351151415	2.791387851
6.844509425	1.295906111	2.440027717	0.089921502	0.103043316
11.41672361	1.611085847	1.956072437	0.476656962	4.297936531
1.402370462	0.073327905	2.522621441	0.108140424	0.124573565

hsa-miR-3681-5p	hsa-miR-376a-3p	hsa-miR-802	hsa-miR-202-5p	hsa-miR-211-5p
0.100790597	4.592538303	2.078910701	3.073093646	2.069181435
0.006082297	1.616284381	0.007438405	1.02488959	0.006946847
0	2.840811827	2.212819253	0	0
0	1.412024554	0.859789011	0	3.69207661
3.53784375	1.058792884	2.402566652	0.013503064	0.015933793
0.029088823	0.036250118	0.036016293	1.117579794	0.033485176
0.188214613	2.477275898	0.254505144	0.180558276	0.228822906
0	2.727972178	0.565734722	0	0
0.767133156	0	7.380183282	0.76845561	3.331295122
0	3.028107679	0	0.735762292	0
0.100634505	0.130576986	2.526985862	1.39187127	0.118715602
0.085617793	2.007795206	2.007519723	0.082687017	1.354420765
0	0.95032544	0	0	0.950217457
0.060121718	1.251646004	4.953893726	0.058163439	0.069946053
0.968841761	1.957630314	0	0	3.123608687
1.904255121	0.087782963	0.087151961	0.066602644	0.080383063
0	2.847133309	0	1.916834871	5.322063139
0	0	3.474114583	0	2.027981632
0.113162998	7.38162356	0.146701436	0.10908936	0.134070512
0.963937326	2.945326372	2.945417286	0	1.540211102
0.316033105	2.29022911	0.460328206	0.300802502	2.243063485
0.099988161	0.129690386	1.415794164	0.09647297	1.41231066
0	7.928457697	0	0	0.885408987
1.642351196	1.692322734	2.848831657	2.738730623	0.202904276
0	6.461981594	2.602431901	0	0
0	2.225585978	0	0.884672948	0.444642389
0.00902046	1.631334538	2.374432405	0.00875725	3.376834647
0.156387574	1.645093502	0.20769272	0.150332595	0.188055657
0.045002916	2.244446776	4.277958912	0.043581835	1.808648904
0	0.78817571	0	0.791961767	0
0.10425474	0.135554947	0.13448798	0.10056106	0.123139191
0.028269664	0.035213227	0.034986613	0.027408351	0.032533117
1.208657061	0.065272095	0.064823933	0.050062683	0.059997267
3.092543476	1.845200939	1.17598167	3.597100414	0
0	0.749767216	0	0.753682192	1.273385918
0	4.838524382	1.635485142	0	0
0.085224596	0.109614504	0.10879235	0.082309447	1.352830903
0.014634334	3.260781098	0.017979303	0.014201796	0.016763259
0.039610265	0.049652461	2.215768105	4.212654209	0.045760869
1.689186507	2.446364359	1.09114309	0.020939661	2.443569047
1.407462958	0.850935465	0	1.810851431	2.370715367
0.006066763	1.025681257	0.007419345	1.024826228	3.358990327
1.419974698	1.443756344	0.138052627	0.10305978	3.753489479
3.883128581	1.61715311	0.197764984	0.143767087	0.17933608
2.143692172	1.522217537	1.522294546	0.121467346	0.150076492
3.203562339	0	0	0	0.91443615
0.2097138	0.290002358	0.287080854	2.891133701	0.256857972
1.361399884	1.380019278	8.471009752	0.088131525	3.830017798
0.852580719	2.58340709	0	0	0.849727897
0.169335309	0.228640392	0.226537024	0.162643209	2.369181008
0.081424406	1.339511948	1.339677291	0.078659267	1.98060865
0	2.91773365	0	0	1.268322745
0	2.262352418	1.88029221	1.91809889	0
0.13025521	1.53907384	3.72363934	1.499704119	0.155229645
3.845206266	1.109704569	0.032177468	0.025241956	0.029934172
3.266165173	1.701545069	0	1.323846149	2.265786093

0.089080346	1.370956385	1.371116989	0.086011129	1.368407637
0	1.122478326	0.633396917	1.809290231	0
0.005709082	2.842302406	0.006980585	0.005543768	0.006519753
4.543858241	1.062772805	0.018257069	0.014419246	5.70704428
0	2.782066647	0.531928496	0	0
0.071984686	7.726923014	1.300812708	0.069584241	0.084088287
0.005689396	1.024085921	1.614269844	0.00552466	4.283464773
0	7.282402662	0	0.634979466	0
2.232528495	3.588806062	0	0	3.99029224
2.001686537	2.809327515	0.000390737	2.3236488	3.461413273
0.128039069	2.66136909	0.167364991	1.491744737	0.152472386
2.868660198	1.87189765	6.446421288	0	0.904232291
0.163731236	2.367890226	2.366729804	0.157317328	2.800361827
0	2.126626264	0	0	2.964204905
0	2.900663377	0	0	1.503866977
0.033670471	0.042067002	1.141781537	0.032632511	0.038819936
0.036437095	1.153223347	0.045293699	1.146708235	0.042049381
0.18389394	1.756312184	1.756027975	3.13427851	0.223237296
0.821219406	3.877224002	6.345179403	0	0
2.072200213	0.814427425	0	0	1.750274702
0.028846387	1.121495892	8.648114449	0.027966394	0.033203352
5.074854224	1.582244944	7.146817667	0.135565267	2.24701183
0	1.829218821	0	0	0
0.043061842	2.565860934	0.053720759	3.688328866	2.827887354
0.126418273	2.198258433	0.16509719	0.121761158	2.633080747
0	2.780484227	0	0	0
1.285624053	1.276511053	0	0	2.221455523
0.016925506	5.664794369	5.664369146	0.01642265	2.683020726
1.557034443	0.920434302	3.523090425	0.484175442	0
0	4.582621177	0	0	0
0.093095868	3.104982159	2.829484357	0.089864209	2.477222132
0	0	0	0.658892721	0.653125736
0.232660292	0.326181687	0.32272757	4.002804143	0.287228083
0.000348737	2.001957461	1.001479613	1.001431485	0.000397531
0.188450123	3.295471327	0.254857765	1.702839656	1.760380963
0.146436571	3.09576948	0.193392613	0.140857718	0.175487519
0	0.874465523	0	0	5.014765496
2.384250771	3.881573618	0.014240134	0.011268837	3.069461375
0	7.040674293	1.214757583	0	0.703202153
0.580276565	3.273974489	0	1.06309394	0.57622658
1.815390323	2.78145952	0	1.817413437	0
1.279076253	2.98161577	0.088323125	1.276067353	1.926417054
2.545542523	1.548471638	0	0	0.970094096
0.016896412	5.016422945	0.020783617	0.016394454	0.019369342
0	4.440031132	1.173741267	0	2.095122489
0	3.718286566	0	0	0
0	7.286271509	2.306541337	0	2.306824951
3.202784822	1.262216842	0	0	3.055185339
0	1.291675699	3.805650316	0	0.50086132
0	3.665329253	4.021584616	0.811860929	0
0.009397683	2.052379836	10.02155444	0.009123228	2.37552335
0	0.525383732	0	6.243677033	4.451711784
1.847417943	5.163971778	1.437377131	0	0
0	9.508781379	0	0	0
1.155470942	2.753483623	0	0	0
0.011911025	0.014702486	0.014612193	1.642319196	0.01363112
0.019464636	6.681221997	0.023975708	0.018883033	0.022332992
0	2.026200647	2.279822669	3.029005559	1.329610138

0.201410782	1.82701312	0.274407053	0.19305598	3.584248243
1.999954057	2.322888727	1.240297495	1.490041769	0
2.06572796	2.049965463	0	0.813490257	0.809017463
0.856221089	2.189338864	0.848834589	0.85961683	1.175515319
0.022959044	1.702047497	0.028333107	3.294656608	0.026373755
0.080741532	0.103584273	0.102816279	1.317633844	0.094600883
1.123606415	3.940861075	0	0	3.769788802
0.846959382	3.872715196	0	0.424897236	0
0.004744787	3.199693547	0.005798539	2.347258116	0.005416753
0.040737119	1.171155191	2.55279977	0.039462229	0.047080818
0.054186699	8.281044752	4.331538846	0.052442836	0.062913354
3.7632754	1.749325604	0.040186377	0.031402944	2.502605569
0.071123892	3.743084081	0.090093552	0.068756138	0.08305827
0.007486602	2.365607762	1.031710282	1.030613103	4.134007605
3.292273357	1.697783553	0.02727585	0.021447749	3.301654366
0.062330169	2.669873438	0.078576441	1.871544146	1.89138164
0	2.335612323	0	0	0
0.036507542	0.045683848	0.045382996	0.035375249	1.153247624
0	2.419217935	0.636208367	0	0
0	0	0	0	1.182621918
1.834390043	0.067074348	0.066612081	0.051400082	0.061635085
2.227496081	1.092902325	1.454596724	0	2.547188478
0.032032441	1.747498757	0.039723897	0.031048463	0.036910743
0.03355014	0.041913849	2.781137096	0.032516157	2.776029395
0.121605621	5.567264059	0.15838716	1.468540892	2.162387652
0.016201085	3.423109669	1.068483616	1.065938361	0.018567846
0	1.922366346	0	0	0.941700968
1.704634401	1.394266674	0	0.573989263	0
2.590189906	1.003993458	0.001149191	0.000915281	3.175681857
0.222754623	1.913098067	0.307226456	0.213228158	0.274060706
3.727578166	0.205464618	0.203642361	0.147660709	4.722445926
0	0.751965431	0	0	0
1.053889891	2.072850326	0.016090093	1.05343521	1.05540967
1.367255372	0.11992374	0.119006592	0.08961555	1.383553486
0.680770587	1.554003515	3.849828525	0	5.770435985
0.015492501	1.664306622	2.677049992	1.659310965	1.663503421
4.065264828	0.060084147	1.200334591	1.190974797	3.446194992
0	0.645873687	5.858547095	0	0
0.847825004	3.082174267	0	0	0
0	2.271427332	0.65654494	2.095172454	0
0	0.838253752	0	0.841634319	2.095158074
2.609055905	0.357100951	2.029083828	0.240590488	0.312907134
0	1.462440114	0.90029366	0	3.173656094
0.074775514	1.312148544	0.094908458	0.072268429	0.0874319
0.955010049	3.100899648	0	4.891366822	0
0	2.79747356	1.081585347	0.606225466	0.600416874
0.065253554	1.272866109	7.001126727	0.063106214	1.905170869
0.056359811	1.236067781	0.070819393	1.224550773	0.065485094
0.158224143	0.21225224	3.429049272	1.598712345	1.642929779
0.083966454	6.812704632	0.10711226	0.081101171	0.098488161
0	2.527478596	0	1.364440438	0
0.907916396	1.735404253	0	0.911329993	0
0.090166083	0.116296802	0.11541342	0.087053144	5.869347449
1.638719264	1.688172942	0.223166068	5.722063405	0.201594147
1.342771156	2.011425972	6.402292094	1.973995432	2.003468917
3.00211982	0.106568782	0.105774113	0.080137482	0.097277872
0	4.211261589	0	1.796402568	0
0.099499253	0.129020174	0.128016688	0.09600437	1.410347929

hsa-miR-1911-5p	hsa-miR-200c-5p
0.123532671	0.103358718
0.007158557	0.006208418
0	0.77649036
3.483836167	0.861568646
2.401755764	0.014207654
0.034572472	0.029725829
0.239630403	0.193814119
0	0
0	0.766289341
0	0
0.123333044	2.817802197
0.104251898	1.342483388
0	0
0.072409283	0.061531977
1.546109658	0
0.083277075	0.070531842
6.192553484	0
1.326878825	0
0.139440698	2.096741219
0	0
0.4257933	0.327340399
0.122506715	0.102531866
0.885317622	0
0.212088706	1.648883262
0	0
0	3.306778449
0.010629864	6.281111726
0.196356357	0.160799061
0.053850985	0.046024445
0	0
0.127969828	0.106928985
0.033587169	0.028887564
0.062065171	0.052910123
1.84729389	0
0	0
0	0
0.103755556	0.08733033
0.017286497	0.014944125
0.04728904	0.040498654
0.025575571	0.02205234
3.23042762	0
0.007140226	0.006192557
0.131320296	0.109618663
0.187133463	0.153657509
1.520200625	0.129481361
3.676856733	0
0.269536474	0.216169521
0.111430478	0.093572955
0	0
0.213818795	0.174218567
0.098967034	0.083420864
0	0
0	0
0.161695688	1.51099107
0.030898167	1.711156122
0	0

3.967801218	0.091298403
0	7.416172534
0.006718237	0.005827356
6.617820293	0.015173344
6.393880383	3.479430347
0.087139187	0.073715854
0.006695016	1.0236031
0.62947206	0
5.978907403	1.279206505
0.000376276	0.000327038
0.158791778	0.131473421
0	0
0.206236901	1.633309333
0.861608544	0
0.933192285	1.506255552
0.040096038	0.03441558
4.747313599	0.0372485
0.233686355	0.189326574
0.817927483	0.820510196
0.81357826	0
2.752131696	1.118394536
0.175699239	0.14475113
0	0
7.638434074	0.044035113
0.156671455	1.496755664
0	0
0	1.673499463
2.093022036	0.017285759
0	0.481373916
0	0.804653011
0.11372344	0.095432453
0	4.044411454
0.302069003	0.24007698
5.461685696	1.58669169
0.239955032	0.194058775
0.183066431	0.150496302
0	0
2.388681523	1.048031235
0	0
0	5.90210945
0	0
0.084387373	0.07144796
0	0
0.01997772	0.017256022
0	0
0	0.826195079
0.988869293	0
0	0
7.795342365	0
0	0.394653668
0.011076163	1.038920062
0	0.984997882
0	0
0	0.889563233
0.649480236	0
0.014053385	0.012161525
0.023039433	0.019881431
0	0.795590457

0.25792141	0.20753074
0	0.69497408
3.945445983	0
0	0
0.027216029	0.023454716
0.098108204	0.082718503
1.478850489	0
0	0
1.60933684	0.004842853
0.048657746	0.041653089
1.227159898	0.055441646
0.038560979	0.033113866
0.086065349	5.805148053
1.623352455	0.007642375
6.460898063	0.022588829
0.075140193	0.063799114
2.339570807	0
1.768763536	0.037320646
0	1.134123131
3.559109128	0
0.063767043	0.054332398
2.379783527	2.011943692
0.038118784	0.032738658
0.039950693	0.034292382
0.150392832	1.478835659
1.068581624	0.016545326
0	0
6.141082151	0.571194431
2.81306884	0.000961555
0.28794675	0.229750548
0.192595497	0.157891459
0	0
0.015472711	0.013383777
0.113394124	0.095165548
1.852884245	0
1.663993644	1.06397998
0.057162657	0.048804701
0	0
0	1.399707463
0	0.657963289
0.83754152	0
0.329686664	0.260047213
6.252960175	0
0.090626157	0.076584199
0.953882876	0
1.975325203	0.603471972
1.272822938	0.066800926
4.242536091	0.05767122
1.648561944	0.16270158
0.102168484	1.973198328
0	4.310640925
0	0
0.110005449	0.092416032
0.210699254	0.17183099
0.105219201	0.088523044
0.100904087	0.085003676
0	0
0.121881965	0.102028112

Table S4. Survival information of differentially expressed miRNA in test group

id	futime	fustat	hsa-miR-424-5p	hsa-miR-139-3p
TCGA-MR-A8JO	0.904109589	0	9.064137916	6.497324861
TCGA-CC-A8HS	0.821917808	1	7.147126964	6.827287924
TCGA-G3-AAV3	1.128767123	0	9.045150771	6.033869453
TCGA-DD-AAEA	1.575342466	0	8.289036872	5.975940443
TCGA-DD-A4NS	6.728767123	1	9.848814853	7.301100551
TCGA-EP-A12J	1.561643836	0	8.629181977	8.060542544
TCGA-2Y-A9H1	3.367123288	1	8.187519115	7.116103245
TCGA-WQ-AB4B	1.082191781	0	9.667157064	6.155787904
TCGA-DD-AADP	1.254794521	0	8.974445515	6.798663173
TCGA-DD-AADN	2.460273973	0	7.786542	3.391590545
TCGA-CC-A7IJ	1.046575342	0	9.120299254	4.710109523
TCGA-ES-A2HT	1.2	1	9.334792601	7.620327595
TCGA-DD-AAE3	1.550684932	0	8.201152007	8.003645627
TCGA-CC-A7IE	0.594520548	1	9.255512632	5.430017747
TCGA-FV-A4ZP	6.810958904	1	8.218407526	4.119406178
TCGA-ED-A7PY	1.068493151	0	8.176451468	6.572493994
TCGA-CC-A3M9	0.821917808	1	8.613767481	6.140028979
TCGA-BC-A110	5.797260274	1	11.17380964	8.901262396
TCGA-ZP-A9CY	2.142465753	0	9.822335596	7.915538224
TCGA-DD-AADR	5.556164384	0	7.841393426	7.17812948
TCGA-DD-AAE9	1.978082192	0	8.886983744	6.303444811
TCGA-CC-A7IL	0.761643836	1	8.587013689	5.552572682
TCGA-ZS-A9CG	0.934246575	0	8.847341187	7.486056035
TCGA-2Y-A9H9	1.909589041	0	8.02524942	6.994460286
TCGA-ZP-A9CZ	1.934246575	0	8.572583864	6.498341912
TCGA-DD-A1EB	5.526027397	0	9.998017834	7.778376915
TCGA-DD-AADB	3.402739726	0	7.550607334	5.053740835
TCGA-BC-A10S	3.898630137	1	9.222130467	8.022111411
TCGA-LG-A9QC	1.164383562	0	7.727963135	7.780288934
TCGA-DD-A73B	0.775342466	1	7.938577556	5.522837691
TCGA-K7-AAU7	0.983561644	0	9.733967352	5.427023598
TCGA-CC-A9FS	0.578082192	0	8.361455009	7.621564865
TCGA-ED-A82E	1.117808219	0	9.258294181	4.487142927
TCGA-2Y-A9GW	3.482191781	1	9.552810978	6.683379779
TCGA-DD-A4NO	6.150684932	0	7.822694505	5.502138112
TCGA-LG-A6GG	1.060273973	0	7.545617953	5.762327285
TCGA-XR-A8TE	2.534246575	0	8.361924271	6.718533317
TCGA-DD-AAE1	1.512328767	0	6.467544596	6.989728879
TCGA-DD-AAW1	5.449315068	0	7.47333271	7.406150186
TCGA-DD-AACD	1.043835616	1	9.035302215	6.970741254
TCGA-G3-AAV5	0.969863014	0	7.193885017	5.759763803
TCGA-DD-A1EE	0.956164384	1	8.890510759	7.466134188
TCGA-DD-AADE	3.293150685	0	8.326587915	7.031167454
TCGA-DD-A4ND	7.523287671	0	9.380604517	6.177867464
TCGA-ED-A66X	1.112328767	0	9.082718437	5.622253454
TCGA-BC-A216	3.701369863	0	10.59031189	5.548613043
TCGA-CC-A8HT	0.383561644	1	7.95212742	4.881688717
TCGA-DD-AACJ	5.75890411	0	8.888589911	5.527816963
TCGA-ZS-A9CF	6.608219178	0	8.615863286	5.386579258
TCGA-CC-5258	0.353424658	1	8.706408204	5.679442789
TCGA-ZP-A9CV	2.980821918	1	9.252726176	7.432103192
TCGA-WX-AA44	1.684931507	0	8.911497264	5.299650815
TCGA-BC-A10U	2.293150685	1	8.3912003	5.020943859
TCGA-DD-AACT	4.279452055	0	9.222610338	7.74032655
TCGA-MI-A75H	2.046575342	0	7.882833968	7.378999232
TCGA-DD-AACB	6.367123288	0	7.884869656	5.466941715

TCGA-G3-A25V	2.356164384	0	10.72782317	7.202623635
TCGA-DD-A73D	1.898630137	0	8.120416479	6.00254248
TCGA-DD-AAD5	3.684931507	0	8.49375429	5.212821641
TCGA-CC-A123	0.6	0	8.188909333	6.206050445
TCGA-2Y-A9H4	3.978082192	0	8.209402752	7.350647105
TCGA-BC-A10R	0.843835616	1	9.843195753	7.234930397
TCGA-G3-A3CH	2.136986301	0	8.983562374	7.657848722
TCGA-DD-AAEB	1.309589041	0	8.109351937	8.319996213
TCGA-BC-A112	0.419178082	1	9.055542741	6.243295863
TCGA-DD-A39X	4.64109589	1	8.902526196	6.752713167
TCGA-DD-AACE	5.983561644	0	8.034410353	7.731726556
TCGA-DD-A1ED	6.304109589	0	10.20024747	8.495976299
TCGA-MI-A75I	1.726027397	0	8.785476755	5.58679888
TCGA-DD-AAD3	3.547945205	0	10.05897643	6.544226452
TCGA-G3-A25W	2.561643836	0	9.904144032	6.424666728
TCGA-DD-AACX	0.465753425	0	7.751607406	4.597340173
TCGA-DD-A4NP	9.063013699	0	9.661592673	7.763047681
TCGA-BC-4073	2.326027397	0	8.643774047	6.258612439
TCGA-DD-A3A2	5.838356164	1	8.236302732	8.217443717
TCGA-DD-A4NN	2.463013699	1	8.493053983	4.692606369
TCGA-NI-A8LF	2.189041096	0	8.327705854	7.382639496
TCGA-DD-AADS	1.298630137	0	7.721522625	7.593158881
TCGA-DD-AAD6	1.84109589	0	7.879993861	5.067746063
TCGA-DD-AAE4	1.665753425	0	8.922275663	7.054310438
TCGA-DD-AACP	1.136986301	0	7.237676521	4.926793484
TCGA-DD-AAVR	6.884931507	0	9.720152355	7.449012835
TCGA-BC-A10T	2.293150685	1	10.49799533	7.410285036
TCGA-DD-AACU	4.293150685	0	9.932810143	6.099509828
TCGA-DD-AADA	3.378082192	0	8.938117338	7.119212011
TCGA-DD-A73A	1.994520548	0	7.330608865	6.146648275
TCGA-DD-A11C	1.81369863	0	8.474475033	6.693711259
TCGA-G3-A5SL	1.701369863	0	7.89175308	6.887642973
TCGA-FV-A2QR	1.591780822	1	8.043034744	6.090737207
TCGA-DD-A1EA	6.616438356	0	8.786255493	6.597386689
TCGA-CC-A5UD	0.832876712	1	6.268787534	3.904837425
TCGA-DD-AAVP	7.539726027	0	8.969660235	7.754049683
TCGA-UB-AA0V	0.860273973	0	10.57969008	7.846903616
TCGA-DD-AADK	2.873972603	0	8.847194993	6.708377163
TCGA-DD-AADY	1.520547945	0	8.335326191	8.819778841
TCGA-BC-A8YO	1.539726027	0	7.645287814	4.618299914
TCGA-DD-A1EI	0.501369863	0	9.959704728	8.178062417
TCGA-G3-AAV0	1.304109589	0	8.678255776	8.270157901
TCGA-DD-AADQ	1.194520548	0	8.486873737	5.470751821
TCGA-K7-A6G5	1.402739726	0	8.564374358	6.48442123
TCGA-G3-A25U	4.482191781	0	8.829010905	7.686538385
TCGA-DD-A39V	1.761643836	1	8.937430683	6.25412719
TCGA-DD-AADI	2.97260274	0	8.743615289	6.600748565
TCGA-DD-AAE0	1.520547945	0	9.470677738	5.09653262
TCGA-DD-AAEG	1.969863014	0	8.966732856	6.510948353
TCGA-CC-A7IF	1.778082192	1	8.760447655	7.091688522
TCGA-DD-AAVW	6.347945205	0	10.01361599	6.431946294
TCGA-KR-A7K8	2.482191781	0	9.986923315	6.308182564
TCGA-EP-A3JL	0.830136986	0	8.331708172	7.272977126
TCGA-NI-A4U2	4.906849315	1	7.851523193	7.156503795
TCGA-FV-A3I0	2.323287671	0	9.067327461	3.674199871
TCGA-UB-AA0U	0.895890411	0	9.947965104	7.743991967
TCGA-DD-AAVV	6.726027397	0	9.436488215	7.163461854
TCGA-QA-A7B7	0.257534247	0	7.639397645	5.279977624

TCGA-DD-A3A3	1.465753425	1	7.983114573	5.830539179
TCGA-5C-A9VH	0.882191781	0	9.293533924	5.729886593
TCGA-CC-A7IK	0.717808219	1	7.713078363	7.180230805
TCGA-G3-A25T	4.254794521	0	9.55455877	5.863028684
TCGA-2Y-A9H0	10.06849315	0	8.220631379	3.529775199
TCGA-DD-AAD2	1.802739726	0	9.785394052	6.597029544
TCGA-EP-A26S	1.665753425	0	10.2271466	7.741870348
TCGA-DD-A1EF	1.079452055	1	8.664926399	5.892041544
TCGA-XR-A8TF	1.898630137	1	7.688050631	7.493399457
TCGA-CC-A7IH	1	0	7.642066427	7.867640585
TCGA-DD-AACV	4.194520548	0	8.404407541	6.090454053
TCGA-LG-A9QD	1.002739726	0	8.746772122	8.577423979
TCGA-CC-A3MC	0.994520548	0	9.478759291	5.206271828
TCGA-DD-A4NV	6.569863014	0	8.942985009	7.700679591
TCGA-DD-AADF	0.315068493	1	8.217041967	5.449052438
TCGA-DD-A11A	0.216438356	0	9.156883801	7.491693594
TCGA-DD-A73C	1.920547945	0	7.050346579	7.807261415
TCGA-DD-AAE8	1.819178082	0	10.03113977	7.122203667
TCGA-HP-A5MZ	0.249315068	1	8.671863787	5.72666794
TCGA-BC-A5W4	1.498630137	1	7.514172446	5.103778806
TCGA-KR-A7K0	0.178082192	1	10.0896129	7.434744034
TCGA-DD-AAVZ	5.205479452	0	8.019585968	6.803657112
TCGA-CC-A7IG	0.819178082	1	8.176529	4.953704637
TCGA-5C-A9VG	0.898630137	0	9.053736931	7.004158473
TCGA-DD-A4NF	2.580821918	0	7.936535185	7.477555885
TCGA-2Y-A9GX	6.690410959	0	9.739879081	7.035302253
TCGA-DD-A113	6.643835616	0	8.52660247	5.446046333
TCGA-2Y-A9GU	5.312328767	0	8.123029071	6.736631241
TCGA-G3-AAUZ	1.315068493	0	8.635290256	7.215791248
TCGA-DD-A119	0.610958904	1	9.810898657	6.907774701
TCGA-G3-AAV7	0.989041096	0	8.919092591	4.311547887
TCGA-DD-AAW2	5.082191781	0	7.711700519	7.910873521
TCGA-DD-A39Z	1.646575342	1	8.669845898	7.449970262
TCGA-DD-AAD0	0.375342466	0	7.328525879	4.678035313
TCGA-G3-A3CJ	1.62739726	0	8.801371592	7.993353637
TCGA-DD-A4NI	2.235616438	0	8.393295937	7.257777469
TCGA-DD-AADG	3.136986301	0	8.131309362	8.13694313
TCGA-ZP-A9D4	1.082191781	0	6.895616712	7.562573485
TCGA-RC-A7SH	1.282191781	0	8.9799111	4.828452299
TCGA-DD-A1EG	3.75890411	1	9.156802556	6.255606752
TCGA-G3-A25S	1.139726027	1	8.902922284	5.17479923
TCGA-G3-A7M9	0.153424658	1	8.731322988	5.915793519
TCGA-WQ-A9G7	0.082191781	0	9.612238224	7.079714552
TCGA-MI-A75E	1.389041096	0	10.22384201	6.796182005
TCGA-WX-AA47	1.523287671	1	7.509977203	6.195120375
TCGA-XR-A8TG	2.460273973	0	8.130975495	5.426278392
TCGA-G3-A7M6	1.731506849	0	9.774123159	6.217163038
TCGA-DD-AAEH	2.147945205	0	8.390157274	7.564086404
TCGA-ED-A7XO	1.169863014	0	10.57546288	8.042936855
TCGA-BD-A3ER	3.054794521	0	9.472834209	6.521629126
TCGA-BC-A217	3.82739726	1	9.545444466	2.818643558
TCGA-WJ-A86L	0.945205479	0	7.746537514	2.236148215
TCGA-DD-AAEK	2.923287671	0	7.929039262	7.313417024
TCGA-2Y-A9H7	3.2	0	7.083794286	6.154883567
TCGA-RC-A7SK	1.293150685	0	9.300969473	6.9840948
TCGA-DD-AACH	0.534246575	1	7.665349013	6.039313745
TCGA-BC-A10Z	0.093150685	1	8.889131769	5.850860938
TCGA-BD-A3EP	1.120547945	0	9.145352993	6.52385908

hsa-miR-1258	hsa-miR-21-5p	hsa-miR-93-5p	hsa-miR-589-5p	hsa-miR-10b-5p
0.096921423	20.43050627	13.61366479	7.442181572	17.92253364
4.125013033	17.96399545	15.44890011	7.120853702	14.62270436
1.718409561	19.63159735	13.89950544	7.842899009	17.38048744
4.407637624	19.98806561	15.51116037	9.570119404	15.92563812
3.295866316	19.27620054	14.42314154	7.649150521	16.20457808
0.044278428	19.27552744	13.39138614	8.231268361	17.20939501
1.48632026	21.42906184	13.77364269	8.74552929	16.87422265
0.018988326	20.47682455	14.25849761	8.35561023	17.42533695
0.926985765	20.5329713	15.29223913	8.526563479	14.52512069
0.041683654	20.69689169	16.03565009	7.861734319	15.31502558
3.754372803	20.22983222	14.76308057	8.527280197	17.79318662
0.195411215	20.31536672	12.98484958	7.823874499	14.31471454
1.341245955	20.05070728	14.73424916	7.629466203	15.80565347
0.981733394	19.11674376	14.75975061	8.453949265	15.30052551
0.16639415	20.27020041	17.87201896	9.379901659	13.59581546
0.037423904	18.41819352	15.46712311	10.42869432	11.62695285
0	19.85686443	13.97503889	8.997249669	16.37194977
5.485848901	19.3924793	12.73280044	6.491531155	14.67586005
4.879263236	19.9844671	13.35073317	8.533089971	16.38056421
5.002422669	21.11096351	14.92334654	9.560437697	16.90863534
0.153794371	19.80352945	15.31040852	9.13340164	17.50260895
1.822064125	19.53822699	14.85542798	9.831476918	17.25630904
0.024985425	18.68127923	13.7972306	7.901580671	17.53450657
1.449796781	20.54729973	14.72825174	8.721710871	17.19860388
2.88779437	20.29027215	14.60402683	8.846846729	16.2447768
5.428471523	17.64977781	12.4465053	9.70329705	16.08681492
0	20.31546134	15.84762575	8.379219496	14.52745268
3.270997018	19.98698872	13.91595564	7.726400167	18.01783701
0.075038446	18.60358848	14.37344692	9.701534362	16.76983942
3.64155954	19.54938721	15.37107122	8.932068121	16.84528382
0	20.99779618	14.46644806	7.763119437	14.30451629
0.999699858	19.72816797	14.75215203	9.027416911	14.03534013
0	21.06090952	14.12498571	8.672371506	16.03546909
0.758861369	20.73925642	14.14623332	8.806910375	14.29456612
0.084976358	20.59051935	14.96415904	8.584728514	18.01728703
3.788604707	20.45562734	15.43309796	10.01442952	18.90507394
0.111317737	18.83355714	14.02622907	7.535050446	13.63356822
1.357074396	19.15353802	15.16214914	8.160308661	17.18470434
0.073556953	20.48619776	14.88882584	8.290666175	15.17690695
0.01915044	20.47880395	14.89189569	8.366039407	16.55011829
0.088096228	20.3161046	15.66463706	9.43587939	14.95549739
0.058112628	20.16525689	14.30756479	9.282553412	17.38854348
0.110933275	21.06388387	14.94235511	8.091432103	17.53792176
4.175285757	19.55027779	14.6246755	8.066165359	16.09489409
0	20.45684677	14.59261811	8.462816552	12.78242205
3.973340686	17.87408919	14.38473703	8.173757039	16.62345745
2.600754521	19.34800417	15.00702797	10.59280394	16.03440218
0.166374122	22.55795009	14.85841269	9.375918008	17.37276221
4.73216839	19.88947033	13.30651705	9.090046892	15.38543923
1.818082034	18.94334845	14.72889055	6.858169725	15.4579435
0.002363321	20.70724849	13.51406484	10.44163987	14.0016078
0	19.98935932	14.74850709	7.999340853	15.68923146
1.173074071	20.14707949	13.83795247	8.830814998	16.61611628
3.097714768	20.38447524	14.63460747	9.328222136	13.03831831
1.046856822	19.30864536	14.83312032	9.323535524	16.53144449
0	19.63700231	13.44258852	7.204646441	16.34902604

4.666348407	19.83856113	12.93643383	7.308628267	14.85504564
0	19.34233222	14.5260899	9.982040944	17.79017203
0	21.05198514	14.20939952	8.37789485	12.72806526
1.840237526	19.83361461	13.81736858	8.955955977	15.08731291
2.740296732	19.21219048	14.17821686	8.09434363	17.37248624
0	19.74776026	13.01166035	8.22196445	15.77588654
0.68171932	19.10648176	14.46405746	8.144506476	15.98369619
1.030086216	19.34258523	14.74999363	8.406228306	17.86376095
0	20.90032899	14.76660451	8.780889656	12.86883422
1.210336057	19.29664911	13.28482558	7.928295442	15.30285365
0.054071017	18.8930099	14.86729347	9.660163591	15.9811999
3.978489689	19.03222019	13.23684496	7.252458168	16.51684534
1.977401523	19.46188804	14.99084095	9.796637898	17.02081909
0.573573692	20.1454545	14.61923197	9.584968125	11.54241061
3.356248226	20.16021129	13.7487965	8.36446747	15.86604631
0.049890108	20.58797462	14.3738115	9.556114321	15.90338502
3.332403547	20.2243694	15.19096082	8.011238649	13.1231734
0.001778216	20.58265434	13.29461276	7.935579699	16.53989661
6.247803342	16.13617002	13.86410031	8.987516549	12.38057903
0.00834252	18.8685223	16.73578275	10.96305854	13.82387903
1.879339581	19.67505376	13.98768936	8.83256226	18.06581887
4.714769821	20.48352614	14.61723293	8.474372469	15.93804743
1.767565951	21.50781104	15.57360111	8.556544443	16.10110265
2.359645503	19.90507784	14.95784149	8.425194268	13.85387607
0	20.16151948	16.84043739	9.125303668	16.57192229
0	20.42274201	14.01734198	8.3839757	15.27781203
2.195077955	18.71615095	13.2060439	8.241382784	14.82786239
1.975997686	20.62386266	14.72528634	10.62812271	14.30819579
1.423566582	19.43870321	14.80716339	9.129164744	16.20262876
4.769605179	19.71282937	14.3402793	8.435560449	11.30288971
4.443451707	19.00960274	14.46714926	8.173658669	18.26703726
0.130433043	19.91302578	15.8435359	9.222841512	17.70930276
0	19.92932631	13.85151018	9.627283453	15.12373986
0	19.68461173	13.17864099	8.798181437	16.01162034
0	20.38664545	15.89568158	10.05055328	16.73281381
0.027697323	19.44808965	14.81746972	8.979282205	12.5259064
5.594144926	18.59362826	13.42070417	7.660173862	14.77366661
0.001317225	20.77627486	14.06422048	8.260650788	14.52782678
2.276473827	19.60491458	15.33936898	10.55700938	15.48487284
4.465783618	19.49694808	15.26345234	9.086484493	14.76793292
2.670154671	19.82900034	14.28712214	8.544255934	14.34218498
6.66245516	18.6988991	14.23402833	8.532272083	11.65468132
0.06177317	21.47029164	14.78401324	8.512226216	16.67910368
1.994718565	20.48569587	14.84913798	8.320675564	15.42235798
0	18.67701673	14.40835218	8.181322323	17.64886332
0	20.73929385	15.23436189	8.874648847	18.33573785
0	19.97717703	15.20625756	9.320837528	13.92704392
1.420489589	20.27501007	15.26132759	8.929851903	16.85572021
1.687475522	20.12562022	14.15041168	8.908485899	15.83230584
0.004260016	20.50724633	15.06783315	11.25878126	17.33619828
0.025139785	19.98304514	14.02410725	8.130628358	11.26826556
2.691855789	20.16835286	14.0316128	9.381881348	17.57388068
2.037447236	19.71051768	14.42497059	9.82707086	13.6285371
4.475376114	18.02577406	15.02681987	8.099731267	16.11110012
0	21.14908901	13.58224734	7.312512853	11.19027228
2.680453529	20.6191059	14.68692272	8.482916854	12.93134513
2.483344392	20.68412038	14.48730421	9.778637604	18.53333444
4.448778672	18.04059753	15.08101789	10.00469378	14.04618059

0.054868545	21.59691399	14.93699822	8.299543534	16.38327801
1.085352121	20.03121691	14.03332743	9.007574857	14.40498284
0.103007521	18.65791768	15.22724472	10.02645691	17.29375374
0	20.0354446	14.3935694	8.46000676	11.09351868
4.047698825	19.29334616	14.81622845	9.162955685	16.06468837
1.726954994	20.76584009	14.30848701	7.896171688	13.16584146
0.021626853	18.23239773	13.74828487	6.741734873	18.93045152
0	18.78576169	14.528467	8.265210052	16.69437956
0.37426106	19.63250014	14.75922667	9.384908446	17.07163129
0.092589792	18.66654135	14.24414357	8.950767504	13.82473333
0.023370855	18.71875661	15.62759344	9.337409622	17.02112356
2.382905068	21.18266481	13.78928878	8.650477904	17.07318367
0.020487204	20.23642879	14.38414347	8.71731972	12.31459098
3.874079586	19.50198278	15.29046602	8.327229782	15.65526371
0.015626419	20.21787302	14.14089357	9.430650729	15.7895732
2.54434341	19.93528955	13.64354482	9.774073964	18.54341393
6.041990819	17.15156681	14.6532793	9.066527284	16.1089925
0	20.36604138	14.65632907	9.426602378	12.93474691
1.977182454	19.93789871	14.36227404	7.463339719	18.22022399
0.085991743	18.29556703	16.46177693	9.178495852	17.47989478
2.559112503	20.20549932	14.39782521	8.397648217	15.69304314
2.084048605	20.23222313	14.77524185	8.810345818	16.01916554
0.689411725	20.1212477	14.87208936	8.138558451	14.71650417
1.047051737	20.46772581	14.64777644	7.385849517	15.73712614
0.149065306	19.0905108	15.1524812	7.788643903	18.09704812
3.640238999	20.35242951	13.83391143	8.299097019	12.25780367
0.102007671	19.80810885	14.30850287	8.65267879	17.2394554
0.068413712	20.26035121	15.10233483	8.996707025	17.63611613
0.018181257	20.47247749	14.04321089	8.452080562	10.22561953
0.010458722	20.20368249	13.3588416	7.901785496	15.19251481
2.368307611	20.02634582	14.86038316	9.513632256	16.53403511
0	18.97616173	15.37152828	9.030882239	16.56193899
1.54211574	19.70090654	14.90777298	9.708416294	16.691035
0.027128098	20.69630247	15.14620275	9.270247588	15.40831283
0	19.65369075	14.37749465	8.990456723	18.37476558
3.817790427	19.54860271	15.32110247	8.970185682	14.14594132
0.087954606	19.79169766	15.09044752	8.524949012	16.93071589
0.0105163	20.55216081	14.58913906	8.282131642	17.03970602
0.564515387	20.08442878	14.38207537	8.971723597	12.77388067
0	20.16973616	14.61677303	9.293273337	14.40323167
0.030556729	20.42846674	13.91229731	9.654221752	16.65316333
3.376081673	19.29237408	15.10045805	9.079194126	16.32941927
3.685204796	19.09542838	14.83519365	9.267982783	13.38264886
3.645402508	20.25965447	14.0209597	8.597254968	13.40140584
0.08080285	20.97675322	13.75492955	7.643729263	14.61682115
0.045506092	20.51546991	14.39871526	8.936834657	15.68298242
2.022086347	19.72832276	14.71151696	8.082846978	15.10583793
0	19.43298261	14.88466219	8.613609197	14.74841264
4.631530543	18.53295919	13.88220797	8.323313312	14.97017165
3.070057448	19.66698613	13.99899117	8.282545715	13.98162688
0	19.06393178	13.75984515	9.346086378	14.94764436
0.0504706	20.08229655	14.53444909	8.991559121	15.39365135
0.946702209	20.37516024	14.43006324	8.131371025	14.39319492
4.215832512	18.9346713	15.77098471	8.523161477	17.09837533
0	19.77233979	14.60661943	10.2357286	11.4377073
2.533948069	21.59071167	14.85283811	9.423245862	15.41191847
0.036158611	19.16278417	14.85701992	9.328653548	17.66686858
1.342584769	19.26943442	14.73171449	7.767401779	16.7952132

hsa-miR-4746-5p	hsa-miR-10b-3p	hsa-miR-500a-3p	hsa-miR-224-5p	hsa-miR-424-3p
3.052933782	6.13038245	10.14401128	8.654145895	4.495433669
4.798239885	3.652895186	9.611827549	9.832184126	2.293729249
3.751396725	5.42207708	10.12351033	9.73642292	3.6244511
4.482486081	4.899183505	12.15755643	9.033960753	3.895896078
3.087486534	3.089499508	11.17227054	7.93724623	4.994588998
3.403776819	6.420426224	10.08742133	9.594270669	3.230935387
1.490936399	5.518639033	10.03601321	7.681409342	3.667055257
2.092069406	6.754922556	10.23288849	9.322030918	4.012789126
4.584294299	3.884800268	10.3424376	9.508333008	3.472597491
5.038155419	4.691880108	10.22479597	10.83469081	3.022678805
4.390723099	7.166386884	11.20719126	7.355256765	4.584122401
2.828248639	3.699756725	10.08853793	6.204290016	4.489555084
2.776594875	4.827248787	9.886985595	10.06968732	3.278751685
4.671741308	3.779046093	10.94664129	9.415114606	3.142673687
4.973296401	2.257807753	11.62819026	10.64322684	1.576789026
4.111802569	0.042288058	11.64431653	5.672984983	3.660542391
4.770275768	5.542867602	10.60328651	9.303624519	4.132183376
2.526407192	3.037654295	9.595420747	7.027440355	5.742604267
0.078256566	4.773492486	10.21922232	10.02168193	4.371673551
4.28945055	5.967228149	10.42622149	6.233245446	0.238567251
4.553181418	6.418843935	12.28794197	12.63892167	4.322440414
3.894186638	5.050519118	11.01426073	10.92862883	3.887918375
2.939098498	5.977414503	10.17637661	11.47913447	3.945267222
3.423076422	6.393818395	9.664135372	8.976957566	3.619650966
4.637814783	4.972852703	10.91278416	8.054883172	3.614580531
3.085095897	5.301428833	9.464401897	5.195835998	4.289535044
4.426497252	3.874156152	11.11907439	8.2965061	2.554914484
2.588753699	6.747841756	9.879369179	9.466730863	3.453617108
3.18313344	5.048358623	10.758644	6.343037049	4.090191458
4.587457261	5.901113186	11.16663891	9.67192723	3.510317395
3.343298334	3.340285401	9.09034708	9.871538809	3.105838157
5.208980969	3.458975077	11.18995226	4.523088767	4.391852223
2.071630697	3.436851531	10.00277755	10.47327202	3.513942137
3.693137467	3.074312499	10.16552325	11.05055069	4.643645979
3.755044829	5.154131072	12.24860344	10.78062441	2.72589226
3.850901988	6.509866115	11.98581684	10.10747866	2.510200182
3.115755732	2.049553349	10.56236003	4.266003387	4.257465199
3.870444931	6.402377896	11.6453363	10.46618779	2.529198273
3.373324289	4.716760555	10.86228843	7.264931458	2.675175769
3.806967468	5.944495579	10.70044549	11.31999465	4.277382187
3.769551424	3.443535814	10.85188183	11.65087929	3.241656519
4.009308502	5.907406982	11.19143833	4.84246555	3.298638413
2.839043907	6.36379303	10.09103402	5.487995142	2.049670677
4.282964843	3.600444786	12.00620798	10.32658919	4.928489153
3.485145063	2.231558825	11.55141351	7.593556002	4.421464719
5.71104902	5.604523264	10.13286723	9.805075944	4.051082208
6.540161543	4.283326334	11.71860662	5.474217243	2.620491373
4.496552203	7.000672057	10.25709421	9.873111257	2.716784985
3.29597857	3.968431309	9.806671032	7.614772589	3.294292311
3.246521492	3.949751322	11.27672162	9.461334876	3.247063632
3.598332432	2.334095468	10.60585882	10.67432953	4.335529523
3.935423034	5.563163975	10.79538494	9.4198829	2.763287354
3.420613472	5.436535491	10.30759027	10.73526613	3.051903938
3.689169715	2.09090311	10.83099335	11.01997465	4.353655829
3.068568201	4.880915828	10.85294982	5.203374126	3.068396292
4.938314357	5.738786717	10.46115493	8.200045369	3.218330549

3.388399245	4.187568419	9.610836111	8.838211836	5.008983659
3.372409386	6.418175159	11.30011958	9.653965543	3.593532627
4.6240235	2.977898383	9.651285734	8.504770061	3.102199727
5.83138505	3.682932039	11.8844072	7.527140581	1.168014812
2.477805326	6.245020258	9.371620159	10.38642941	3.627990108
2.085769795	5.081470095	9.671884653	10.12388217	5.371583046
3.28152753	4.908180706	10.35051158	4.874612023	4.848981919
2.363451179	7.507587071	9.894246473	10.18574475	3.85304718
4.766492363	3.130961669	10.31061182	4.602652524	3.545451037
3.300155609	4.221276697	9.717245975	5.93965922	3.471803027
2.586754741	4.998978207	10.8809895	8.113935379	2.253898851
2.301765131	4.883766607	9.268567969	7.499876802	3.785097412
3.433157203	5.616050169	11.74107502	10.34344026	3.780814643
3.080444813	1.051884131	10.67590737	9.934926262	5.597845493
5.097963385	5.057168139	9.98778454	4.931654473	3.699261244
5.444328512	5.135399901	10.72686082	4.52228152	2.566832542
3.603656947	0.203519053	11.30426238	6.356076232	4.272794057
5.295822549	4.53399474	9.502643485	9.651034769	3.469414066
2.721773313	2.724270357	9.757411873	4.910133036	3.546666954
5.092737422	2.364617235	13.46582258	11.13831852	3.954060357
2.619072613	7.072500742	9.75673464	6.05082121	4.336063838
2.876979349	6.076616607	9.628625122	9.798972738	3.393593285
4.360653319	6.4087236	10.39173449	8.176564058	2.546953271
4.508120327	3.407163395	10.41180079	8.661632754	3.206137806
5.49817538	6.307626714	11.76414399	5.466538333	2.891754568
2.372389022	4.568973918	10.34291989	9.01650424	5.08551425
3.024616693	4.03727376	9.411717498	10.21340017	4.551421248
3.142433514	3.778869313	11.66163053	10.1285125	5.015539758
2.800593598	5.104925912	10.9861995	10.31623069	3.791197046
2.284657193	1.217927411	10.47149764	10.609052	2.888310453
4.075414018	6.912879772	9.93963606	4.942707333	2.832235987
3.958692775	5.879036705	12.03799851	11.72762476	2.91889907
3.461845266	4.763025609	10.83814063	10.4482838	3.463231649
4.566364227	5.511664567	10.30545315	3.484234897	1.646468692
5.685160259	5.079860026	11.2285329	10.19742112	0
2.727918345	2.132840682	10.29307388	6.604222072	4.15315202
1.544708963	1.95633043	9.708988386	7.611266329	4.992401854
1.590935645	3.914494107	9.655457325	9.640992861	4.255508048
4.971179299	4.722524029	10.9665497	10.07967522	3.637339181
4.671004814	3.815183993	10.80478035	12.43349163	3.625508515
2.260023456	2.661948025	11.29024029	5.152530746	4.099977494
3.269896	0.966098369	9.934997311	9.961684319	4.116464994
4.653703881	6.296251238	10.00791966	9.298993317	3.909341811
2.752859094	4.124835721	11.0824182	12.06773047	3.578634586
4.892546912	5.887742527	9.727619572	10.16083941	4.159239042
2.463696658	7.726255923	10.23223799	9.675899456	3.868672404
3.779228982	2.789657005	11.3348953	9.369801143	4.300792272
4.314886865	6.476886442	9.802909545	9.710590804	4.474768464
3.295657294	4.833584716	9.987345088	9.396833692	3.828619064
3.345687667	6.069747833	10.19944911	9.614475379	4.024278144
3.458644597	1.091395152	10.43569577	9.075623829	4.948716013
4.305653848	6.41964505	10.40008866	8.807095742	3.987235625
3.294427207	2.421214128	10.73821289	10.43970968	3.295822032
2.784004768	3.489345261	10.78288889	10.80971711	3.813240173
1.731467795	1.074677005	9.319727946	10.15661001	2.725429711
3.359250778	0.790501187	9.608268712	8.498405681	5.347007278
4.978363239	7.907131067	10.63898039	6.328577356	4.384245434
5.583605632	3.184616631	10.88307035	6.496001108	2.455722944

4.821543936	6.51603913	9.677752774	11.24300209	3.45541723
2.364256836	2.921050558	10.5089718	9.22922238	4.515942064
5.743289667	5.348104963	10.64408699	9.282689741	2.018714381
3.966363323	0.472883321	8.905262578	9.132615488	3.665537005
5.732033377	5.132912855	12.0039502	9.153166858	3.35344925
3.9856584	2.977614745	10.29131678	8.840179629	4.824353541
3.116005348	7.345373553	9.257076585	6.29658858	3.819915894
5.431123649	5.530285186	10.1374948	4.716011179	3.199740275
4.525238719	3.819386898	11.31912967	5.862607627	3.433569514
3.790299406	1.326878302	10.55703598	11.42104653	3.928110611
5.525423736	6.562100621	10.90691017	10.58183658	3.448875028
1.307122229	5.953946042	9.971174587	7.578111977	3.231844516
3.433956742	0.023036968	10.30866318	9.728589079	3.921074725
0.233389126	3.938431908	10.63939165	9.029150173	3.488522459
4.844266378	5.763812851	10.62548072	7.616373425	3.545632677
3.415192908	6.975246298	9.848606666	5.785779893	3.415325425
4.192894573	4.088528653	9.682508571	5.530544254	2.33967328
2.232250009	2.852880172	10.87349791	9.297691869	4.97475457
2.974146248	5.586640589	11.95721659	10.91682892	3.432983047
4.144080526	4.916276718	12.82534081	12.46150008	1.95122421
2.920935738	3.633263574	10.86502711	8.418311257	3.800257339
3.830167781	6.286752626	10.59627858	10.34442369	3.369450402
4.379093782	3.996218544	10.7066257	9.486109388	2.328775899
3.08186158	5.401239886	9.75111428	8.004613841	4.418747945
3.50646076	5.270236321	11.92618641	7.843327703	2.194680777
3.288364179	1.450310048	10.16699756	7.331339156	5.143989674
3.677935888	5.537238853	9.929667876	4.105232741	3.303736401
4.059990175	5.659735832	10.43917167	11.2459195	4.94897815
3.559752123	1.665809382	9.58164523	9.265248117	3.908568562
2.863408098	3.380101489	11.00380844	9.480566279	3.866155529
5.385477117	5.404065781	11.49993604	10.69212605	3.837233906
4.042596746	5.822277889	11.34608152	8.727065503	2.879392594
2.755611467	5.873564423	10.66208157	10.457552	3.530950288
4.737480387	5.477800959	9.817454382	10.00941645	2.130484034
2.968634294	7.896425806	10.33050502	10.05009433	5.136605742
2.470936861	2.470670473	11.65324836	10.84581698	3.686177526
4.153292996	6.379989716	10.62875449	9.191872962	2.40152188
3.866702074	6.084422668	9.40513394	6.039660874	2.863594416
2.959977463	2.728654376	10.79114964	7.52637076	3.416023496
3.185024905	4.000426736	10.40207544	9.890859547	4.006526432
5.457699674	5.496753863	10.85109368	11.01783432	2.741899691
5.595655109	6.239747572	10.20333936	8.781227443	5.468730673
2.677219607	2.340928599	11.11274322	8.533922349	4.293029758
2.27132506	1.746388272	9.885754033	10.09913208	5.142746316
4.002584999	4.515634471	9.206938229	6.372002539	1.289886908
4.709076933	5.008836201	9.832923823	9.99415992	3.701708685
3.485068291	3.025070856	10.41241272	9.876241284	4.274103225
4.427287252	4.300741539	11.09852988	9.120819395	2.661591325
2.926767502	3.047315427	10.60335222	8.081316234	4.031209833
2.776286211	4.104244329	9.968261032	9.062303902	4.972489718
6.207068535	3.918560141	10.26186607	8.690601437	2.913550379
4.443762514	4.923404634	10.6412131	8.658319232	2.23773443
3.916859392	4.374594596	9.928836971	9.128829057	3.503278606
3.873595537	6.440923295	11.65510819	11.82654473	2.775966798
2.840838739	0.895785462	10.32588868	8.894687633	5.11532129
4.012255242	5.427784684	10.06424433	11.19731399	2.835468229
5.411783511	6.160587888	10.98968914	8.677254305	3.190278372
3.013966034	5.953694022	10.65827466	8.885138169	4.433012947

hsa-miR-7706	hsa-miR-490-3p	hsa-miR-183-5p	hsa-miR-532-5p	hsa-miR-452-5p
3.049677087	0.088858719	12.19130953	11.86133216	9.150927833
2.897126776	0.058109926	12.25185983	11.63840271	10.16187986
3.332102063	0.028228082	11.58966725	11.31128822	10.07589659
4.481538503	0.014732945	10.06887463	13.76937562	9.90501675
2.025159757	6.002819818	12.29764921	11.92994926	8.206425141
3.694337257	0.040944359	12.28673905	12.90305873	11.43637319
2.613919677	0.127811887	11.81062158	12.84201103	8.81122131
3.563278231	1.667210738	13.34604071	12.54316523	9.751054596
3.588039389	2.705929194	13.4273087	12.51706613	10.89780085
5.720768623	0.038561406	14.73696107	12.7498006	11.31188592
2.884389479	1.424234274	13.88328311	12.6785658	9.06575409
3.881762865	2.310951241	12.87886071	13.19837551	7.112807808
3.474237425	0.088359684	14.9213555	11.94688562	10.95347285
3.431892648	0	10.19769851	12.34917546	11.23920497
4.485816406	0.150871991	15.37576634	12.02985175	11.03192643
4.452224596	0.034644998	13.32559601	13.49493527	6.859412318
3.757867425	0	11.82848421	12.01445668	9.539762339
1.318279533	4.847722814	12.13421606	11.41183898	9.252790274
2.661320095	3.330667848	14.04806454	11.92537371	10.6785398
3.510221237	0.19077718	15.09045121	13.10941292	7.201262076
3.518819035	0.139725232	7.752814481	14.6819607	12.88027389
2.546911939	0.223276023	8.054993024	12.29967819	10.78151896
2.938360232	0.023177657	10.34309297	12.06677649	11.60501832
3.94606516	0.117775098	13.25835889	11.9602913	10.27467377
2.906334979	2.528716318	11.11935511	13.20586015	9.073260353
3.200380771	0	9.699505717	11.33263792	8.014186261
4.552293799	0	12.17648856	13.40637997	9.30539917
3.084067661	0.050312716	14.10023204	12.06462251	10.21191661
2.952233454	0.069040346	8.51906347	13.16516719	8.590804271
3.356155156	0.032567527	10.70927982	13.78444602	10.30637933
2.7110991	0	14.30938798	10.99544168	10.00806001
2.806920505	2.321532625	13.88186653	13.77727908	6.918376344
3.368361271	0	16.10994644	11.52090116	10.41775786
2.942611006	0	15.49793796	12.55772838	10.59783069
4.608204132	2.69912502	12.40504276	13.07219513	9.613651405
3.323509357	0.213079379	14.0001822	11.79841702	11.30855661
3.714041634	2.467468198	9.914632104	12.10122742	5.628365523
3.513397566	0	15.28707077	14.17396742	10.0349528
5.141846562	0.067693576	11.35894093	13.66054001	7.720453876
3.806042915	1.069755151	11.98092503	13.48815272	11.73840955
5.040163008	0.080882915	7.660214505	13.0872867	11.5423149
4.307996052	0.053615191	13.11825639	12.95073765	6.7858422
4.007490467	0.101475876	13.16611929	12.65025553	7.790911751
4.867480255	4.063246379	13.15224111	12.06127997	9.896722103
4.653011879	0	13.16214444	12.68816152	7.43232566
2.21608241	0	14.21539254	11.72325697	11.20799175
3.237703256	0.128849884	14.04139909	12.7564658	6.185245504
3.94372295	0.150854308	10.98802634	13.81112723	10.27387351
1.355677584	0.091518221	9.154259472	12.09300015	8.88813336
4.638530189	0	9.576906422	12.2501603	10.48028186
3.182946992	4.099905961	14.24488964	12.62997209	11.55009899
2.618168133	0	11.78780646	13.21415514	10.1996515
3.051000566	0.044029127	15.98724872	12.02990157	12.236837
3.100565278	4.418645911	12.73484493	13.76975161	10.5679425
1.641848448	0.011759881	7.183828454	13.18637074	7.493261168
4.20858452	0	11.37000875	13.3984332	9.534075911

2.3463501	2.187290267	9.272169417	11.58118818	9.73332015
3.954742121	0	6.445473396	13.87726291	11.53191096
3.754918311	0	14.13082345	12.08497027	9.705186531
2.638490604	0	11.11029824	12.74098455	10.18877512
3.753485095	0.028853437	15.39883188	11.98166513	10.3555583
2.341765124	0	14.21911016	12.36696829	11.32906092
2.944931466	3.313175159	10.99011441	12.6677161	6.831272365
2.627499759	0.007537201	9.430283939	12.8303555	10.52247058
1.965624988	0	15.93862741	12.24082281	6.40076268
3.763465712	1.206889643	11.85989887	12.59102223	6.850691
4.094010952	0.049919362	7.019635358	13.46986638	10.21414386
2.786282507	2.565346022	11.14301618	11.28797299	9.273163265
3.144198957	0.982880931	13.59002772	13.41030509	11.11312374
0	4.583147567	8.584232865	13.20665692	11.02273719
2.705210932	0	13.29487068	11.82987823	6.896346555
4.073140995	0.046091029	7.190279389	13.73088055	5.89572539
0.190256228	3.024560973	10.56823099	11.78872188	6.620916017
3.469372551	0.001655925	13.27817281	10.48551604	10.15672558
4.256145392	4.442646574	11.31597188	12.03863872	6.316372024
6.156521004	0.007760303	13.83873879	13.63118767	10.0544066
0.300672471	0.240119677	10.07259324	11.98482626	7.889667414
3.979736933	0.01211035	13.86789226	12.20044638	10.19553857
3.312288806	0	10.93191324	13.12339357	10.28558714
2.703999497	1.281405378	8.492158981	12.68208454	8.777951027
5.408441149	0.928110752	13.21089725	13.48781699	6.465516513
2.116252756	0.85557554	13.51884546	12.48456559	10.70402555
1.769341207	3.907755988	14.7396147	11.59406329	11.15633276
4.495056989	3.296159092	14.92502765	13.00467857	11.42316129
2.393286224	2.980377601	16.47416398	13.17181875	11.42946998
2.617058938	0.056176859	13.94420541	12.29240166	11.40288759
1.675523505	0.177890453	14.69225932	11.41521927	6.172418531
3.796141659	0.118941539	10.53956178	13.48494574	11.31951604
2.656698597	0	15.29557089	13.32330642	11.04877389
2.913215985	0	12.17319621	12.89062981	6.013556422
4.386960278	0	11.73253717	13.53730648	9.720375119
4.677279148	0.025681827	8.575886763	12.76969228	8.007606179
2.537823683	5.122483732	11.18951331	11.65180408	8.743482114
2.328708652	0.001226732	12.67289726	12.8576061	9.575954218
4.569494351	3.092043982	13.63135235	13.27926812	10.72864355
3.280787083	0	16.2797068	12.27277443	13.28549979
4.284843517	0.78180324	12.26917034	12.12330985	7.200799398
3.647935016	2.952185263	14.23438856	12.14311332	11.1177619
2.287607937	0.056958363	14.21055305	12.21040777	10.4938984
4.464336571	0.274521656	10.76095433	12.57378001	10.93231513
3.677726592	1.810467935	11.78824961	12.49509212	12.07081451
3.456011042	0	8.399842768	12.90409876	10.15049285
3.867859549	1.819237131	14.8694845	14.02801483	9.391861866
3.610869326	2.463162196	13.32353239	12.26396941	9.626623498
3.712024833	0.021572992	13.10141756	13.41933038	9.465784531
3.930987551	1.015668189	9.872825699	13.20864689	9.908410268
2.448102999	1.091218103	12.63086686	12.81738576	9.987151214
2.028188028	0	8.87120791	12.00594016	10.60179995
2.862900557	0	10.91603428	13.67243134	10.8200818
3.284160512	0.08990397	14.12485812	11.33325308	11.30372517
2.727479278	0.360150231	15.38817583	12.39860391	10.37705629
3.123944247	4.583028347	9.916884569	12.35276369	9.787389466
3.611742505	4.1153148	8.865350366	13.38457001	7.412873159
4.716243931	0	12.64518537	13.09027203	7.390749728

3.454108332	0.050649043	11.4746854	13.28002676	11.13653263
3.413171099	0	15.50879195	12.81688238	9.75691513
4.216821667	0.094345951	8.729332756	12.2279767	10.84298865
3.92620475	2.29221768	16.38575071	10.99790914	10.19317029
3.52473198	0.06409435	13.92854847	14.46412295	9.785120273
3.170887621	2.475060616	10.450282	12.79028931	9.028381722
2.430867317	0.020073239	13.98961933	11.60365852	8.9669589
2.408642946	0	14.63698181	11.8738528	7.046551717
0.425825306	0.328791872	14.41999707	12.35846908	6.921150987
2.420109029	2.396125691	7.64194644	13.31592175	12.51298717
3.448328497	1.685753783	10.4665651	13.08978315	11.77225989
3.001194202	0.078920037	13.21466055	12.04908945	8.797249998
1.676502756	1.673570736	10.1967891	12.87187491	10.0885188
3.483141503	4.000675441	9.964982643	12.0023728	9.012483445
2.401138266	0.014518289	11.70556374	13.47970421	8.064452706
2.543465971	0	15.03916042	11.80243173	8.596181841
3.173503515	0.067676713	12.09119041	11.69906382	7.462422734
3.697508039	5.265334704	7.904814065	13.08335602	11.04204671
3.143949361	0	13.73187886	12.95987561	9.309021652
4.612949066	0.078977644	18.17848223	13.3094559	10.55120889
1.45891702	3.56952956	9.970274102	12.29041961	8.878895601
5.551929516	0.542429874	11.40573797	14.13224983	10.44349014
2.677707054	0	13.11480915	13.43318168	10.1883586
3.902404545	1.947449433	14.74992813	12.1564343	8.647707902
4.297728256	0.135530225	9.496641354	12.82157801	7.104084248
2.424941422	2.172876585	10.87694887	12.19125959	8.809478176
3.499255955	0.093445217	15.0925054	12.05403082	6.325769322
5.06231785	0.06301304	17.26197382	12.49060401	11.23276081
3.80082203	4.951776117	13.15737965	12.32750415	10.12058818
3.965564023	3.223264876	12.44621606	12.55613633	10.74399052
3.114431556	0	15.55074803	13.30826795	11.6757054
3.575904595	0	10.04947311	14.31135169	9.682854013
2.947777379	0	16.08287769	13.17947095	11.41147631
2.724403603	0.02515639	9.465263464	13.48397362	10.83083126
3.290164755	0.979042152	7.829560765	12.93410515	10.59098483
3.081849925	1.360396953	10.38184899	12.14455445	10.17256657
3.609854046	1.941960957	8.546742082	13.13710614	10.29843584
5.189368353	0.009778842	11.32344382	12.25417964	8.282145011
2.296531301	0	14.8636945	13.25677212	8.132775242
3.285181502	0.630348405	11.23988936	13.10827005	10.53770484
3.62419428	0.028319784	9.118072655	12.78375405	12.55697782
4.298757384	0	13.96160462	11.69742121	10.97015881
0.079860624	2.322119703	15.87553432	12.4533936	9.577702482
1.240083949	3.549368866	11.31194857	11.90458175	10.37560036
5.005883965	1.283122394	14.04756236	12.13377054	7.289023798
2.21493049	0.0420711	13.80563808	12.1363451	10.27383176
2.022426376	2.021530965	13.15095676	11.71763759	9.75893877
2.183516718	3.442516094	8.074056197	13.48547337	10.85139296
2.301818181	5.891086204	11.25756271	11.7069	8.711392074
2.212173975	0	13.09713002	12.49375744	9.41137888
4.581588813	0	15.02795645	12.16047582	9.971687639
2.237324238	0.046622879	14.21739008	12.84877155	9.176559389
4.309172242	0	9.791618033	12.64604629	9.77622591
3.667634738	0	16.28977407	13.28940763	12.31035811
3.29686367	3.431894122	14.34326969	12.01712343	9.539089965
3.499489269	1.21091636	13.23387388	13.0544039	12.32372388
1.132542523	0.033480635	14.20944703	11.59695891	9.794828293
2.69981915	0	12.4706482	12.64697766	9.633461682

hsa-miR-96-5p	hsa-miR-501-3p	hsa-miR-103a-3p	hsa-miR-182-5p	hsa-miR-139-5p
4.083244931	8.160706112	16.76133639	13.81129959	9.004754954
3.920025474	7.147054743	17.11512082	13.18709679	9.221478059
4.419476488	7.296198248	16.66181189	14.09323316	8.552828052
2.66777489	10.07591515	17.13434816	11.66862753	8.557841978
4.793351529	8.376120767	16.97114395	14.41593982	10.15410153
3.698156916	7.904796176	16.6680301	14.51307096	8.835411383
5.296505797	7.168497008	16.57010973	13.71503353	8.816324558
5.838880347	7.913214267	16.83524271	14.73917668	8.802503202
5.834795541	7.64086606	17.34343388	14.51835371	9.448862098
5.235075035	8.794503676	18.20860128	15.12634008	5.760457621
5.797384554	8.534404606	17.40287314	15.29099142	6.398763277
5.109555256	7.977094103	15.89205962	14.49730551	9.605355591
7.460346118	6.785834832	17.60542221	16.51058128	10.76827127
1.975940083	8.767797782	16.7729079	10.85118965	6.840620896
6.534010416	9.319740044	16.95790898	16.92423793	5.961826563
4.378573121	8.888959552	17.85537579	14.33764279	9.020817437
2.723242739	8.904363612	16.22561954	12.22131161	7.554802148
3.469110086	6.880067083	16.39143429	13.87816445	9.498750988
6.116485868	7.741543525	16.26889979	15.95403079	9.78200783
7.13341353	3.524158002	16.42419944	16.66655496	9.976866016
1.527893669	8.759863601	17.54159726	9.906398152	9.396709882
0.303836606	7.03736608	16.94567579	10.4814566	7.310735765
3.839551376	7.135030114	16.94364122	12.20786815	9.92672637
6.502188769	7.606239271	16.23111194	15.19836049	10.20168297
2.566304445	8.11378553	16.54359253	13.14189508	8.419774398
1.20719374	7.465956369	15.43866318	11.32956351	9.225531468
2.776662271	9.218246538	17.14975342	12.53132783	7.623849396
4.691999147	7.406218134	16.83247021	15.87203602	8.631004904
1.265527758	7.309246903	17.44777271	10.81535195	10.47293239
2.765044375	8.203883556	17.06389978	12.30715967	8.388910705
6.726418693	6.72403177	16.33312997	15.40048179	7.337407261
6.538674334	8.247439025	17.99399607	15.47666748	10.04527027
7.652873704	6.834323281	16.42746759	17.67664721	7.196666354
7.299728679	8.245062763	17.1189373	16.25420302	8.504840443
3.428955022	9.411312771	17.7985623	14.17110669	8.589952431
5.872478223	8.297992608	17.62691526	16.13156969	8.812375591
2.498474769	7.206385392	16.0076813	11.59184022	9.676723267
6.287203291	9.235141104	19.11611151	16.11934318	9.18208903
3.375281757	8.521716321	17.45504905	12.94739353	9.972826098
3.915165228	8.195451397	17.26169703	13.36306953	9.488540221
1.310047431	8.294652243	18.05550063	9.409673952	8.61063828
3.300941349	8.585374529	16.97694847	14.52478008	7.414797601
4.968034882	8.24254696	17.34596356	14.47001808	9.739970424
4.192292691	9.558035531	16.96475145	14.67344603	9.450768051
4.355945749	9.719467431	17.36935283	14.36229874	7.835576681
6.575988605	7.459723441	15.50664463	15.33460983	7.258508313
5.808776748	8.9136096	17.08393582	14.81458218	7.140272481
3.583225682	7.283520139	16.80151191	12.2922232	8.726703006
2.003262252	7.237509341	16.08138901	11.46285659	7.72793101
0	8.893960411	17.42113709	10.295967	6.653560739
6.080239334	8.271689183	16.61226592	15.89612734	9.085782721
4.754865294	8.26723862	16.99107494	12.84334214	8.017583143
6.913853236	7.611587108	16.51509621	17.02910804	6.292476995
4.106623717	8.261060611	17.10138852	14.34789639	10.53675387
1.641248092	7.836997688	16.9210262	9.275305088	9.770861144
3.093511142	8.063541053	17.64171681	12.43590469	7.291127484

1.436043744	7.135733028	15.86588322	11.28099287	9.104039639
0	8.000626968	17.10213116	8.349040591	9.031196239
6.446829635	7.622416095	16.00473665	15.30450898	7.185558476
0	9.795187255	16.51477783	11.88863046	6.793213357
7.809753844	7.051258418	16.62371106	17.20473978	9.42490949
5.307576313	7.226576152	15.8624472	16.2713201	7.784422244
3.981677485	7.988172373	16.2916539	12.86515463	9.777393291
2.363305925	7.037191869	16.76765063	11.56053712	10.86972317
6.497074097	7.815575115	16.77654459	17.20317682	6.968915119
3.474650497	7.446819848	15.8849302	13.37478861	8.979969214
0.061877418	7.765630796	18.00213699	8.97229938	10.54076197
2.301844572	7.136218477	15.94164142	13.53929612	9.98467466
4.616363305	9.22199946	17.44692272	14.38639266	7.842098509
1.053781669	8.350270496	17.30002883	10.3117769	9.133533442
6.055850231	7.564364731	15.97137748	15.16122823	7.626302371
1.178473704	8.503431117	15.73119571	9.158122364	7.190396652
2.279992843	8.347602663	17.27456881	13.19830746	10.96159677
3.710620788	6.976520216	16.88825356	14.49178086	6.868713482
3.853441602	7.715194479	16.39817865	12.42783412	10.59143371
4.572166596	11.23501284	19.10571609	14.78736757	7.932709772
4.666693045	6.643806245	16.5616869	12.92515128	10.29784287
6.552795953	7.372194828	17.21685691	15.76316849	9.890033734
3.887706021	7.797956249	17.0869546	12.87367572	7.92903791
1.283477732	7.925440706	16.38600641	10.65143289	9.875346904
3.347560527	9.706325839	17.85764742	13.38735358	7.228855206
6.248752123	7.340122865	16.71243523	14.90714488	10.11297725
5.993280768	6.097193942	17.05651556	16.43728062	8.396275914
6.751547228	8.748027288	17.21718269	16.57535605	9.208328066
8.205790057	8.11392134	17.33050997	17.2564442	9.78516024
7.012400707	7.055738878	16.33449801	15.64311287	8.461783996
5.655278884	6.775580487	16.78242927	16.29729561	7.818629081
3.199612171	8.694153559	18.55529895	12.23815446	10.07857305
7.625740997	7.959943735	16.59420037	16.17928679	7.854751592
3.044883981	8.247618041	16.14631529	14.06925683	7.567827079
3.286567275	9.254931562	17.76045056	13.66472611	7.627272483
1.100092538	7.596522467	16.99736915	10.92741817	10.07679771
3.948312257	6.923341473	16.27839362	13.32267031	10.22074823
3.914498563	7.722220631	17.02872603	13.39965234	8.944630138
5.620834693	8.449389002	16.99684771	15.39062654	10.68138124
8.457886023	8.049170493	16.92553137	17.90118909	7.207194179
2.009331915	8.868008769	16.96277436	12.99849566	8.653016694
7.427738054	7.558378437	17.02905953	15.99850253	10.49327557
5.89704452	7.622724069	17.75130193	15.37763392	8.167332973
2.01430084	8.699249104	17.50690965	12.80662419	8.728023277
3.853638011	7.095557278	15.6633936	14.3436261	9.295875989
1.88868847	7.648816188	16.84791432	9.768885248	8.415274001
5.978410019	8.77766017	18.21838119	15.35473787	8.963500448
5.419813518	7.618365647	17.75476603	14.01750696	7.167218027
6.439377639	7.871458656	16.64628592	14.85889926	9.420149139
3.023363901	7.451936219	16.15104155	11.64416908	9.790628187
4.229319825	7.861756139	16.63366963	13.96135024	8.951931241
1.722996576	7.782293515	16.23810263	11.27476692	8.526956089
2.240686722	8.498268339	16.79254863	11.83726367	8.78435861
6.734453697	7.796102874	16.84200356	16.59030806	10.47940544
8.307794686	6.699489536	15.7913602	16.77281763	5.647839944
2.018908527	6.883550555	16.53315685	11.60156418	9.95021338
1.172585253	8.566259784	17.17206106	10.65029129	9.182309158
4.24886825	8.281830052	17.29403794	13.91300897	8.0656849

3.612478868	7.595700363	17.09994987	12.44453028	8.4657782
7.407798085	8.032062707	16.89921805	16.37539636	7.9807935
1.360356634	7.671884977	17.24086266	10.70128037	8.858186442
7.527593388	6.107697462	16.57318086	17.68313148	7.357496114
5.874172104	9.640711138	16.33391183	15.17022796	6.139055662
2.977428585	7.950320648	16.91796348	11.84630277	9.177833061
6.711506176	6.741723469	15.86987087	15.36538929	9.753494018
4.99066498	7.855142379	17.512585	15.70131858	6.573327918
6.833315938	7.12990307	17.14293846	16.24014922	10.05653031
1.325266333	6.9103798	16.69682847	10.01214261	10.39441111
1.688098001	7.982963291	17.43401438	12.04558408	8.976917797
5.415451838	7.434988584	16.90627559	15.01954989	11.0171353
2.425277537	7.728229406	16.08428744	11.45730673	8.015832771
3.939305792	7.744840466	17.73735995	12.16231872	10.4462133
3.408296633	8.387524363	16.98042123	13.3132464	7.535730466
6.507356279	7.31878796	16.14876047	17.22088687	7.753300013
4.788467873	7.26473453	16.2951798	14.26624252	10.17348181
0	8.208991534	17.28905366	9.807103607	9.738561212
5.46373981	9.736291962	16.93154826	15.49422297	9.151320165
9.927181424	9.994593911	18.47743639	19.90047588	7.755522769
3.199983701	7.552146455	17.10686654	12.26189609	9.246423413
3.201122191	8.967361968	17.54168768	12.01950415	9.271109134
5.105009792	7.597339249	16.5327532	13.84230199	7.139688805
6.972340038	7.529186294	16.73946312	15.72399401	9.371396754
2.191495896	8.795240265	17.40518955	11.71795334	11.52372176
3.287746706	7.291435067	16.37313551	12.98915028	9.170463452
5.826071541	7.247829247	17.29277755	16.53358937	6.904205345
8.60336106	8.195223514	17.07837663	18.12048929	8.91728095
5.629728303	7.491256551	16.64434295	14.8918649	9.559107506
2.375213252	7.497134201	17.51138226	13.7431464	7.790490205
6.3005004	9.440016746	16.81898371	15.37433621	6.374389841
1.892928339	8.18807051	17.75882149	11.33185937	10.73315632
8.54588228	7.549382371	16.60733735	17.24942616	9.500307709
1.704344157	7.245984958	16.98533897	11.49529124	7.298137609
0.97920707	7.623607479	17.03202695	9.406163709	10.10867762
3.086948914	8.637106578	17.14777052	13.10043718	10.53287214
1.309566953	7.619047868	17.58384499	11.04960866	10.73865676
4.868794364	6.662564215	17.85992467	13.34375238	9.948748934
6.471323725	8.254585147	16.72307226	15.70340917	6.894799932
3.080123023	7.879779034	16.86826832	12.22850688	7.983637094
2.145780929	8.276226319	16.97435645	10.8043878	7.367723083
5.891855337	7.342078605	16.66396782	14.4996219	7.73415706
8.128043563	7.637653082	16.72991885	16.34446368	9.619591627
4.932024585	6.9530649	15.70319254	13.29379952	9.142576514
7.04094296	7.466062414	16.04066113	15.77643373	9.433980908
6.858432614	7.041105399	17.20519851	15.5858363	7.536377354
5.486570722	7.727976878	16.53604409	14.89829897	8.354007436
0.903252716	8.184404125	17.53071997	10.24786242	10.08922217
3.766720753	7.107391934	16.31707773	13.8492767	10.72191975
4.783797223	7.63152562	16.47010166	14.30515928	8.77650685
6.519514067	8.490285528	16.65624461	16.42110553	4.650032727
6.166748865	8.235637919	17.10619542	15.0705135	6.643407909
2.729186895	7.667191889	17.49759071	11.20833076	9.564922644
8.31808608	9.159494365	18.18761156	17.22325492	9.737845404
6.813343799	6.879309087	16.71318029	16.47319096	9.389249244
6.099352425	8.120070138	17.11630146	14.55255401	8.775481066
4.847931839	7.740580217	17.2610377	14.97328115	7.531082442
3.999111878	6.393650227	17.35513149	13.54714617	8.570592225

hsa-miR-450a-5p	hsa-miR-221-3p	hsa-miR-1307-3p	hsa-miR-511-5p	hsa-miR-660-5p
6.100169389	8.782277571	12.2397845	6.008505231	8.369938222
4.245380014	8.067932386	13.12957528	3.324959295	7.51301651
5.3438391	9.013938811	12.37446747	7.176193836	7.777438665
4.483394531	9.063039154	13.59187388	5.518974968	9.495250352
6.743903485	8.585241713	11.923374	5.505606501	8.062071392
3.232651239	10.58314352	12.37557741	6.400897382	7.87584952
5.293774789	9.019117467	12.43183052	6.473638145	8.542211277
6.339655968	8.600327281	12.47575182	5.197602227	8.616288765
5.688484054	9.6668863	12.65096365	4.926241252	8.928918647
3.023917179	7.561444434	14.20080686	5.790866359	8.140663549
6.382697295	9.027368269	11.92960046	4.644293818	9.295327416
5.899738006	8.74275513	12.17432673	5.806270047	8.850417487
4.080013752	8.544392357	11.53862385	5.093611458	8.7460204
5.180239717	9.334239969	13.63217794	6.670854894	8.577462091
5.465772077	9.495959795	13.42963965	6.039055755	8.638262597
4.60168583	10.79430263	13.8703622	5.534691615	9.348547385
5.756914015	10.49703856	12.77928439	8.162065368	7.589459273
6.641510268	8.533252333	11.07571721	6.571858356	7.110898898
5.778270123	9.312783282	11.9442657	6.78166458	7.844227413
4.444416506	7.52480263	12.55903817	5.175358549	8.410347297
4.453895214	9.986968877	13.44321605	6.53285783	10.47950898
3.669213656	7.912261401	12.62087032	3.027762804	8.442974261
5.353160229	10.16384183	12.2953306	5.729553708	8.276062937
4.740291673	8.478090072	12.01098293	5.362121772	8.077393364
4.646188585	8.286172633	12.35162096	4.974100074	8.614159036
4.642866307	7.488082484	12.50087309	5.300648825	6.671059081
3.96716783	10.51820951	14.9608354	5.175723791	9.055684136
3.456254077	8.513619997	11.9308661	6.495310853	7.385042873
4.487313725	10.11674055	12.19481139	5.730639032	8.911707518
4.195329489	7.796271918	13.47775214	4.657308957	9.698076941
5.593086473	9.13470645	12.73158769	4.554365481	7.378134459
5.284923639	8.860597815	12.82744817	5.727438739	9.680749202
6.187836444	7.849545263	13.3988373	4.214361146	7.490570493
5.798522274	8.909249848	12.38576884	7.809730677	8.492954858
4.53566229	7.562857522	13.23988471	6.589608561	9.187117209
4.673901859	9.355130692	12.20366359	4.546717794	8.487785095
5.710516404	10.12614573	12.38211373	5.439286456	7.782755214
4.280138896	11.41322171	13.08975338	3.608219509	9.788810149
3.84391192	9.92244042	12.78422872	4.855972858	9.098368821
5.63485675	9.96840432	12.83774091	6.089243702	9.232399801
4.266996242	8.376034623	14.33114406	6.382048401	8.676056459
3.768028559	9.350805062	12.42561775	6.051481382	7.920204556
4.966253228	9.347567606	13.64961076	6.042401175	8.392757404
6.651497361	8.364261135	12.91845234	5.149413885	8.690547687
6.234282252	9.352294219	14.02015556	5.065107998	9.022197427
5.08674682	7.929872652	11.81455492	5.086507309	7.094047006
5.032931878	8.756507826	13.33414523	6.367365242	8.28027525
4.616445508	8.695843635	12.44096531	5.408775736	8.78644025
4.213824857	6.893889946	11.86055033	7.379240108	8.022240465
3.867907939	8.952092793	13.94381011	5.795187917	7.332580604
5.505892871	10.25273432	12.69228234	6.10160042	8.117589976
5.228009872	9.388008799	12.42704889	3.66103359	9.281810906
2.556727043	8.842297578	12.66901462	6.023430949	7.431529537
5.864403635	8.748065888	12.36608214	6.502532197	9.147624024
5.956980629	10.61190601	13.40846094	5.56617095	9.067108474
4.011793168	9.550324243	13.1732163	5.364878089	8.798451472

5.836775458	8.706299814	11.69338562	6.275088798	7.314073051
4.239224234	9.604249375	12.26072263	3.782654991	9.900685238
5.101829137	9.111445817	12.65824007	6.747852949	7.869743853
3.60652329	9.876431133	13.85096242	5.481594	7.927455265
4.497150551	8.088361835	11.90222132	5.535987776	7.788659701
5.629512504	9.152472187	11.37573158	5.477228399	6.957399997
5.445986955	8.113082081	12.53573964	6.18380825	8.489528005
4.747726369	9.260612443	12.19121474	4.954426287	8.524412681
4.482485455	10.0828967	12.38894205	4.207077358	7.055706838
5.491643173	9.973009902	13.03753089	5.782931219	8.328597435
4.382145805	8.753941942	13.84238958	5.586747485	8.925213546
5.146727522	7.930487238	10.81464342	6.561469155	7.298259198
5.29421872	8.593750433	13.57700676	6.141962183	9.708085523
5.941862273	9.189127607	12.60651473	4.195903993	9.238713673
4.745781298	8.2898278	12.5246716	6.93004257	7.233696492
3.72667944	9.173379155	13.52289581	6.51001982	8.527545159
5.941276697	8.641103581	13.14405148	7.272259942	8.138649399
4.180265734	8.707748168	11.92817164	7.494508122	6.470142057
5.024973421	7.584846184	13.85133546	6.051286309	7.230779074
5.178262492	10.66764667	14.43568134	4.749196567	9.740147218
5.308249707	8.628891871	11.44480247	4.522704186	8.054957549
4.074318537	8.948183357	12.5445306	5.120501412	8.495615341
4.41452253	9.742063411	12.64930796	5.353491647	8.738192015
5.074033452	8.169984987	12.65039499	5.720103862	8.447796149
3.792759971	10.79852049	13.94346133	4.407250173	9.034588868
6.061781062	8.512776674	11.63981259	6.132311794	8.609446095
5.881101114	7.848551133	11.69683741	5.729508721	6.683114215
5.853072941	9.615572286	11.95536823	5.362796151	9.167146839
5.990985484	9.42200764	12.45319596	4.73804413	8.871045029
4.578721375	8.1628707	12.96138444	4.329195932	8.08404089
4.085902423	7.180126304	12.87250211	4.745371289	6.875859175
4.983691095	10.14159289	13.12928503	6.374233443	9.719971105
3.973589813	9.05157503	11.43828158	4.201301554	9.11604857
4.209579667	9.011601456	12.10485895	5.364668731	7.985331714
3.745556452	9.883364852	13.11384848	4.836213338	9.406733382
5.682786199	9.930604989	13.48029398	6.06700414	8.479610291
6.156082572	7.39717441	11.7615225	7.145967246	7.553275385
5.255744902	9.732510654	12.58472592	5.177752041	8.834538449
4.722385417	10.95997349	12.33020271	4.790576611	9.423441573
4.135305111	9.76271356	12.25560401	4.810448643	8.020276402
4.916352407	9.371996446	13.04086951	5.836460577	7.694372663
5.777378976	8.07287165	11.97548766	6.153664962	8.181992891
4.13848176	8.806931225	13.0285399	5.428017052	8.192607255
4.32885824	8.947185318	13.16408894	5.625742995	8.745681557
4.223243835	8.549331214	11.22516457	3.356049313	7.462772312
5.033788296	9.620427969	12.85844958	6.816091386	8.894062518
5.525328226	10.38174706	13.77198388	5.230033198	9.613299864
6.083150597	9.432490435	13.06537668	3.429954376	8.062061682
6.017377881	8.03166794	12.59668147	4.037590518	9.232411935
5.112596912	7.509498689	12.79686327	5.640042801	8.823397419
5.699612808	8.92916769	12.74378092	5.274058504	8.740199435
6.497066287	9.785323508	11.8207359	6.810146168	8.661397643
4.883100563	9.601428234	12.70491727	6.950317427	9.273247547
4.973901044	6.867473576	12.06883233	5.649822642	7.73884051
5.819560208	10.54656833	10.83563902	3.719342109	8.878880622
5.96128116	8.267347133	11.84244995	6.470511618	8.399226853
5.544561173	9.465095411	12.92883814	5.002157823	8.772450933
4.105208837	8.390445518	13.48351049	5.760198295	8.814146596

4.469315162	9.417187706	13.3990189	4.548363327	9.215018482
6.146277962	10.27214239	12.42368489	4.754426585	8.868491217
3.686386801	7.801302607	13.05792449	3.842585865	8.254261254
4.285436116	8.788779125	12.90245153	4.45557092	6.1580655
3.824689271	7.691087257	12.54652872	6.313756894	10.10597423
6.230511448	8.696871419	12.56692616	5.139050099	8.606863354
5.446991137	8.735031877	10.90186913	5.447301364	7.09300554
4.74677103	9.982685799	13.40295853	5.439982916	6.997524714
5.497733928	8.334082699	12.01201497	2.965766633	9.006657572
5.536646765	8.457650629	12.58754014	5.704006007	9.495347554
3.830721772	7.637268398	11.65757024	3.830803223	9.129224332
5.158512652	8.476151499	12.28545525	6.924991427	7.791720964
5.610680895	8.580559816	13.42087461	5.328241468	8.098297463
5.735939647	7.542308107	12.6304028	5.225466814	8.529113913
4.088847989	9.352785012	12.54411721	4.337507078	9.061663045
2.956790775	7.061021789	12.1184062	4.860389421	6.653023019
4.297444693	7.702067726	12.53223804	5.206135479	7.915394319
5.806492348	8.83580859	12.15606679	5.988853632	9.522726832
5.32956329	8.813767486	13.20857805	6.672141506	9.18573522
4.148482307	10.84878358	13.07865698	4.358493513	9.523748567
5.878944662	9.11879692	12.39017959	6.37463092	8.931737003
4.92016282	11.27002786	13.15456081	4.83737275	9.973181912
4.712938972	7.927923579	12.90768921	5.105299157	9.228009868
6.048977775	8.828497191	12.38948856	4.789844585	8.343908932
5.204924971	9.913319037	13.61580109	6.310965839	8.886707369
5.651196136	8.462680093	11.76797638	6.340681475	8.029687185
3.978162545	8.8442369	13.61348606	5.538499463	6.921729434
5.834061689	11.11542678	14.01166221	4.538384781	8.474488698
4.912280991	7.516983466	11.99410702	6.106444989	8.184549556
4.86831526	8.749168855	12.81265175	4.059903064	7.83075293
5.666354357	9.1697445	13.31711611	5.427029727	8.633475605
4.514062728	10.25604546	12.0506438	4.393937017	10.12449146
5.265073318	9.187446083	13.61125068	5.942680127	8.774817361
3.145154608	12.17082485	12.44331875	4.475561321	9.275437511
4.551384815	8.184513697	12.40742551	4.824179364	8.766227101
5.907141131	9.168340289	12.89765068	5.978001055	8.700112975
4.15782306	8.17382503	12.91772435	5.053156467	8.76604961
3.759976386	10.38724741	12.01476788	5.149128659	8.489240099
5.340757812	9.43070773	12.92618519	3.981837732	9.175839228
5.32053706	9.004270856	13.2227004	6.119964599	8.745730862
4.170641438	8.333120102	13.27595139	6.996853872	7.979058733
5.647637547	7.734136834	13.73813724	4.796201521	7.98751442
5.44491065	8.619261823	12.83173347	5.48927765	8.144116576
7.010089256	8.754000514	11.78565574	5.58099204	8.244520238
4.515469435	7.643762152	13.21934091	2.708326628	7.792932796
4.420852652	8.780515459	11.000247	4.712430501	8.587184154
6.312594538	8.527431755	12.58893006	5.486548818	8.120321428
4.839710501	8.860610278	12.61472738	5.645277185	9.409099344
6.502508842	7.470081986	11.58888154	7.06003592	8.213960566
6.270527424	8.403964815	12.59357354	5.74013144	8.265347826
3.671858889	6.153204771	12.42119472	5.915037835	8.222693911
4.670451036	7.662096159	13.37782364	4.802941535	8.364234913
4.669565796	8.996911079	12.87811544	5.199250127	8.40001585
4.136112838	11.18143729	13.48969559	4.965542932	9.460573378
5.633657645	8.080808996	12.8150244	4.914756683	7.76606339
4.069511867	8.930339483	12.89228321	4.725062451	8.954726776
2.502041933	7.151402242	13.22598829	5.563509987	7.398879237
6.20723931	8.680454304	12.50328396	6.585577421	8.63814324

hsa-miR-1248	hsa-miR-1180-3p	hsa-miR-1269a	hsa-miR-101-3p	hsa-miR-222-3p
0.068198718	6.162811166	3.952854535	15.16839565	7.02961453
0.045229783	8.259647296	10.00019555	14.64965672	6.480511348
0.0222812	6.746482081	14.11765776	15.62667061	7.443928534
1.054033454	5.450387529	3.547231772	17.00241754	6.95183007
1.330116634	4.346439496	9.030930831	15.69076482	7.203899764
0.032125017	5.97917666	5.002689879	18.01533627	7.815615984
1.429343664	6.212153435	12.50936622	16.37867856	5.928243252
1.660033815	6.378345216	10.16929025	16.1257018	6.587473069
1.504456353	6.868438558	3.587104384	16.27953686	7.647625674
0.03028926	9.131110039	13.74172462	15.07797959	6.018818867
0	5.176741321	1.069262967	16.19692091	7.489535404
2.220368029	6.203397174	5.404227828	16.08351954	7.171389986
0.067830967	6.349874865	13.07568158	17.29472606	6.101255157
2.97758569	8.606833395	9.142529869	14.72958814	7.840571618
2.141642697	9.717676275	9.919685099	13.15638402	8.349365218
1.729524668	7.157970335	0.043120815	17.12590252	8.146786655
1.061203322	5.504573047	4.126105471	14.19066259	9.070767553
0.372325375	5.369184417	2.315107519	16.97644372	5.910319966
0.050500746	6.117265177	10.56926571	17.32328303	6.694742371
1.612767524	7.863522552	5.331224682	16.10052151	5.660452876
0.104859411	6.588862234	12.89105317	15.76684468	6.973497956
0.161825196	3.668643684	9.045514755	16.46239604	6.026499003
0.018338784	5.636664152	11.02442063	16.28534715	7.076917621
0.089235822	5.75420546	10.23890085	17.03421556	6.602603847
1.395285806	6.420118847	5.748443966	16.51956819	6.733002597
0.707675725	5.923570497	9.467197569	17.54613161	6.002860433
0	8.605919826	12.20720053	15.72978342	8.305503119
0.039302852	5.859755717	11.01788552	16.82079225	6.006121385
0.053467638	5.362155927	13.22990945	16.82217084	8.297392371
0.025653627	6.469255176	5.522690626	17.76942315	4.954158467
2.417386739	5.243362236	11.0677084	15.38725524	7.498705727
0	6.523076527	2.806921616	16.25261885	5.906406853
0	6.453519872	2.257392774	15.98208293	6.150584274
0	6.012903697	11.54952163	16.21983469	6.596465538
0.060202817	7.218140942	12.051838	15.64523798	5.886190635
0.15507292	4.404741282	12.46200204	15.18454757	7.673510193
0.077694747	7.065959914	0.131561053	15.33505345	8.228970263
1.776264043	8.193061976	13.37066205	15.62653055	8.621561532
0.052457036	6.449095893	14.42065988	17.28668588	7.586656169
0.014105947	6.920113972	12.91898629	15.34608686	7.604899547
0.062301614	6.45435547	8.417938329	16.58941008	5.472915415
0.041818361	5.909113539	14.30608735	16.37264434	7.392940127
1.978311684	7.218073831	4.145665443	16.98571075	7.317317301
1.172957356	6.54384427	1.808982011	14.9100342	7.274247343
0.939587894	7.259733697	0.937259812	14.12409228	7.600620461
3.226554317	6.930420259	12.21881637	15.52077611	6.318346769
0.097154902	6.370211952	9.760269505	14.2711967	7.033318901
0.112671403	6.118535573	5.347547814	17.54303553	5.784800904
0.070155837	3.066398908	11.45013866	17.97789833	5.659986824
0	8.16277245	13.78469888	14.23828477	6.999605723
1.008153668	5.769027401	2.597379018	16.46234764	8.014278075
2.52544932	6.195995284	5.03920189	15.85954349	7.494370189
0.034495389	7.065228109	13.69407785	16.07686062	6.24717737
0.013853727	7.017696394	9.789283672	16.09761388	6.60254327
1.043234156	5.659514666	13.3160629	17.91843246	7.882715531
0	7.263509376	13.4008933	16.21037388	6.682568019

2.238545323	5.221979112	8.32885075	16.48158984	6.421294262
0	6.059020035	3.486854947	18.52496125	6.021521102
1.937325308	6.819328848	10.45632145	15.88770674	7.041120736
0	8.140119236	7.402804758	14.78849407	7.637578871
1.104618506	5.934415896	12.99375605	17.24756809	5.879950747
0	4.971510559	12.47822159	16.73859209	6.8991943
0	5.868345411	1.196520511	17.0013393	6.041204073
1.617449326	4.632264092	13.29166451	17.83063031	6.315093524
0	7.147556372	13.44729334	15.68881065	8.640600448
0.041898364	7.684949274	4.314687449	15.64425063	7.964463606
1.177980816	4.200467972	10.65274789	16.53498972	6.412840135
1.983225937	4.930992664	5.262110175	17.21413723	5.883412415
0	5.854661636	1.564330896	16.55440007	6.038058001
0	5.979127765	11.53510768	16.09967372	6.942005963
0	4.974521217	2.485531383	15.54337701	6.243108406
0.036075997	5.366815092	13.08898909	17.29579717	6.511160882
2.160072236	5.947554083	4.746742713	16.80642167	6.698701839
0.001323859	7.724984353	11.21108351	15.17338953	6.654569681
1.814345395	8.205281039	13.5078311	16.50595234	4.477963505
1.618401796	8.027231378	3.045228224	15.31507209	8.91730956
0.172864966	4.798062363	1.876263054	15.62242113	6.334471936
0.009632992	6.24702176	11.42831706	17.47877622	6.343964709
4.579093426	5.837121233	6.934716875	16.61304618	6.835455831
1.258266149	5.928631314	7.834028428	17.85979163	5.928813067
2.909372377	8.179364411	11.30165464	14.92262371	8.862991977
0	5.734622738	3.471060679	16.86561732	6.326137894
2.495708124	4.820085791	10.25594192	16.60188362	5.324633238
0	6.725013981	3.14253309	15.78370785	7.379438231
1.842916823	7.437382683	3.3789067	16.04260799	7.188544622
1.19930347	6.246464812	7.41879871	17.2281153	5.953663361
0.13135416	5.344375154	13.03727533	17.88278654	4.387826634
1.40217591	4.235565633	12.14590425	16.83868728	7.435117626
0	5.191573745	11.41859765	16.58923254	6.549950507
0.754417982	5.912075016	14.97590798	17.53199243	7.181259758
0	7.538295846	2.190359135	14.92524125	8.306135864
2.114208885	4.965851903	3.737205308	16.50749904	7.341520913
0	4.901257577	2.53813685	17.52178365	5.232189255
2.327730096	6.815296781	5.05220724	15.9782921	7.157702885
0.043527194	6.220383249	1.212693903	17.41675287	8.832288622
0	6.092927053	13.60773958	16.50281084	8.172519392
0	7.606652489	3.64331125	16.01992506	6.990262625
0	6.319042717	10.44728734	17.22632085	5.467933585
2.243148109	7.264450068	12.99785625	15.66602059	6.771482115
0.194987522	5.067218575	6.201612226	15.46718994	7.018422069
0.863373627	6.843694381	1.807735154	18.25379161	6.965452933
0	7.253585481	14.25987224	16.70464961	7.099640966
0	7.12498178	1.81655201	16.42563501	7.374475629
2.2649067	6.515810883	2.549221705	14.80263044	7.126763602
1.676380396	6.285505336	4.717852545	16.39690134	5.916812474
1.014671564	6.310922532	2.830186389	17.35322824	5.154526915
0.018450355	6.701342785	2.4470186	15.36811276	6.275784567
2.063310127	5.651470704	9.556882975	16.45847107	7.625595597
1.157233679	7.142667772	12.79225278	15.99388236	7.236510709
1.310717496	7.17113263	9.24862029	15.75058945	6.139397523
3.124088685	6.332088663	5.194115581	15.33540236	9.167328248
0	5.569486681	1.327213881	16.94870739	5.496907214
0	6.454177266	9.587962867	15.94504828	6.993392488
0.916363677	6.481653439	13.94794216	17.0804049	6.538685909

1.180478095	9.249713641	2.253788325	16.25861315	6.909192687
0	6.578368345	2.530413507	15.2779942	7.81667478
0.072231725	7.180341621	13.00003573	15.14219877	4.860241157
0.928937958	6.378673831	1.51769537	15.04813338	7.442155681
1.22598372	8.023145165	12.93935077	15.26491025	5.510878662
1.108952785	6.491348119	4.642417135	15.53463238	6.209712057
2.678586017	4.879448729	12.79504324	17.23974473	6.519101723
0	6.84755807	7.583363128	15.88973068	8.054815518
0.228798881	5.297112772	10.34323429	16.57751775	6.38075989
0.065311574	5.400906424	11.46172388	17.91087019	5.247848103
0.017170526	7.38496531	11.58086914	16.58227012	5.659371624
0.060843881	6.247151708	5.748957634	17.27046089	6.466728711
1.06951052	5.952675551	2.097595856	15.56167604	6.735115978
0.135805592	4.418111637	5.687107596	16.78154235	6.079916914
1.053255491	6.764809709	2.665910863	17.11592382	7.092550499
0.972975318	6.95259099	12.90170569	16.59444198	4.477381812
1.856966844	6.81473955	6.311362392	17.49202797	5.611338825
0.64731286	7.095684971	3.826935436	16.00841507	5.819883242
0	6.329207892	7.004959867	15.34024715	7.471367811
1.275255939	6.193845751	14.81350909	15.45346217	9.315511863
1.402417207	5.425027461	12.42049063	16.32105745	6.744847261
0	8.417323086	3.020293522	16.52113691	8.359464833
1.224723873	6.872210261	2.679603854	16.74992494	5.627666564
1.078361027	6.247073942	11.95265687	15.97921883	6.943549441
0.101895861	6.567985788	13.77824647	16.45367909	7.94495799
0	4.948334612	6.995801965	16.9587853	6.371083082
0.071571041	7.726853408	13.06541641	15.5832931	7.054178964
0.048935206	8.317195433	3.522243961	16.68518285	9.035772999
0.013399974	5.939505881	2.413008965	17.29548936	4.912623898
0.007744816	7.300770367	3.227186874	16.14566632	6.776546629
0	8.040914375	2.153581978	14.83520045	7.310573701
2.224186065	5.225876686	3.460143722	16.80879836	7.473783486
1.546259568	6.351775596	4.467440397	17.05429751	7.170983777
0.019885651	7.642203455	11.73771393	17.94499865	8.916963061
0.980321885	6.56547858	13.68550447	17.81679005	5.773084732
0.073105976	5.638988479	3.845971889	16.54271038	7.453057915
1.281063905	3.772438361	13.91977695	17.38557522	6.060240302
0.007787178	4.2304788	12.99514918	18.08564493	8.38027245
1.980303255	7.509593037	2.124083341	15.35746774	7.320015867
0	5.96706669	9.654505917	16.66542522	6.78623305
2.125493196	7.508503858	8.149796324	15.78241459	6.700814017
0.97529596	6.924398865	11.39956418	15.38315529	5.758884637
1.858041064	5.259442156	6.133365673	15.71319742	6.515130366
0.93620153	4.648543627	8.191688803	17.01714712	6.180439245
1.881333069	6.88491927	8.321330862	16.66974627	6.195401111
0.032991606	5.1131938	12.05454219	16.07961916	6.973684885
0.003395357	4.933853307	9.482944128	15.84951924	6.581998435
1.475540207	5.160395073	3.307390764	17.28651669	6.617845395
3.245088943	5.850952271	10.55928724	17.41317634	5.260471397
1.691443318	5.657087444	10.23123776	16.39668381	6.281930311
0.45974677	7.29732586	14.18999385	16.22871059	4.649199044
2.52840342	7.274794362	13.32040018	15.99939164	6.039403517
0	6.694047733	10.75670479	16.36687073	6.252685944
0.979986724	7.953537056	12.32825587	16.36338824	8.961973852
1.467441116	6.146769899	10.71024894	16.24784015	5.55353974
0	5.227904197	10.13318572	15.24912886	6.966947374
0.026361503	6.617467467	8.098189974	16.64517291	5.205499604
0.814161391	6.67686332	3.926984669	15.51094661	6.447161859

hsa-miR-767-5p	hsa-miR-33b-5p	hsa-miR-105-5p	hsa-miR-1301-3p	hsa-miR-877-5p
1.33807016	3.483776974	0.113449862	5.095894065	3.646267976
0.072790879	3.652638021	0.072955492	7.569551071	4.235322103
1.109371935	3.487402603	0.034873607	4.419353143	2.740156437
0.018034688	2.892025462	1.057217455	6.263051084	4.547929878
1.364391196	1.369190409	0.122995254	5.249575179	2.023786979
2.208062345	5.45771102	1.157618427	5.773500055	2.208645988
0.166302628	7.090083906	0.16675227	4.150080928	2.158863182
0.021616356	4.500809449	1.068406954	5.016686083	3.688509219
1.496976871	4.690003095	2.220657221	5.051622269	2.480394021
1.765699336	5.140541242	1.148559445	6.302932298	5.718237042
0.59271597	3.280335031	1.708515739	6.633640782	4.662553951
0.235828997	7.374651091	0.23654484	4.934716269	2.35692595
2.434734624	3.481258428	0.112781749	4.502072409	0.10252814
0	6.079160606	0	6.525046556	5.181193794
1.565465185	4.258819399	0.199414136	6.559260714	3.761155936
0.042857767	3.905533172	1.133642137	6.705965332	2.774209923
9.634816052	2.289646329	10.63344113	6.398622346	4.140172454
5.033027076	4.140397976	5.325530647	3.620484143	1.318002828
4.074830008	5.777412279	5.431128743	4.377978415	3.157945483
3.763686456	4.800114438	5.003987969	6.060272545	3.222747986
7.276140997	2.21208089	6.892346709	4.564204329	2.661997061
3.024083441	2.553973236	0.307715909	3.669810071	2.540568216
0.028498487	5.75864427	0.028557001	4.0470347	2.119900462
6.255050571	4.227857517	6.862990399	4.904060144	2.566305845
0.150928574	4.094234866	2.563412176	5.299082683	2.561597359
0.701383915	6.15340803	0	5.941324169	2.337231502
1.559700498	4.054568813	2.968913915	7.340934469	4.66801318
2.253946045	5.055233614	3.282471574	3.086839795	2.254427057
1.264131829	5.898925292	1.263491067	5.577684072	2.343998347
0.040243442	3.777317873	1.737852413	5.87335294	2.763231813
5.558626604	2.460878202	5.679160819	4.89140277	3.492674899
0	3.169475955	0	5.425784687	2.806922675
0.551646668	2.799850882	1.360646986	7.173434838	0.540844349
0	4.681622908	0.765576141	5.519948856	4.360670323
0.098835242	3.428841599	0.09907089	6.459557699	4.13040141
9.493676515	3.624398277	9.30308267	5.869548687	3.314008647
0.130602261	4.02007888	0.130933263	6.285507672	3.864111984
0	3.868718085	1.360275174	6.097498917	4.559440736
0.085233744	4.786895737	0.085431493	6.183643781	2.337639862
1.669387584	4.501713512	0.021846125	5.433216611	2.683782983
0.102569026	3.443297606	0.102815384	5.86540071	4.353062969
0.066999023	5.319916015	1.205572855	4.314880202	3.621438236
0.130134628	3.721768231	1.383816759	5.161781461	3.106404817
0.060382723	3.077017785	0.060515749	5.65048117	3.975922907
0	1.913883823	0	6.251761769	5.183981917
1.899971674	8.015683484	2.696247311	5.942246486	2.216397933
9.040242513	3.246625609	9.232971769	7.167772834	5.003898501
2.715911767	5.06313948	2.251288165	3.961638868	0.178567399
0.11672843	4.913689065	0.117016607	3.968682476	0.106299666
0	1.816553748	1.419607221	6.566560677	4.105991982
2.011524766	7.336038136	0.002681254	5.473491389	3.598135155
1.160028975	4.721277418	1.529360281	5.731782306	3.671333
4.966255439	5.265464439	6.29243505	4.349236915	3.952010737
1.667882307	3.425459028	2.906952594	4.915941763	1.668992419
0.014372856	4.465183117	2.061004312	5.027994889	2.65073218
9.385761957	4.70176571	10.14984828	5.165592624	3.950108416

5.267379511	6.406118182	5.332920307	4.596692855	1.960018502
0.867527045	2.794212044	1.420836613	3.485758131	1.817690515
8.118325158	5.976541729	8.652312463	5.613377718	3.105953259
0	3.148471892	0.676003071	4.878902627	5.188993635
10.00459788	4.422630585	10.85480123	4.344673984	3.487556837
7.433219761	6.542165226	8.148873626	4.725075372	2.086663635
0	4.497254575	0	6.086835346	2.31815481
1.620986636	4.368793039	1.029346557	4.439360758	2.039533626
0.974680538	3.767148233	2.548061615	5.888865413	1.96570094
0.06713426	6.12986926	1.205969515	6.078798664	2.270914514
2.585542894	3.084805754	3.60791824	4.294271075	3.867411002
0	5.40304344	1.567560459	4.500582217	0
8.233868397	4.432128013	9.059188335	5.431638042	4.06105823
1.410340755	4.52382905	1.056620046	4.757905602	3.086952574
6.260606052	4.473459725	6.69260877	5.930603509	3.21701032
1.177557285	6.137101775	0.057491053	4.078453521	2.565045366
0.207262687	4.286391913	1.585172716	5.661072821	3.079115882
7.993741006	5.917473938	9.733578878	5.098004936	2.594325115
1.979810156	4.902552827	0.376822102	3.853066528	1.988498925
0.009464701	2.628974874	0.009483289	8.630889619	5.691297725
0.333073182	4.359172035	0.334240322	4.795005329	2.604954516
0.014803923	4.396815661	1.64255635	4.074371816	2.652667031
0.830542583	4.414819345	1.768215409	3.719493094	2.548469242
6.77921697	5.184934977	6.96364747	4.330795669	2.701898046
10.44987412	2.891300042	11.01941482	6.273525517	5.500653061
0	4.619740023	0	5.402548031	2.781325998
7.295717191	4.692927836	8.700660314	3.220993498	2.52805146
4.725606364	4.363358899	5.725185375	5.670853459	3.878961729
0	4.592333576	0	5.803748156	3.381254162
0.070296542	5.504794245	2.281874447	4.329080873	0.064524168
8.586346812	3.705601692	9.907677345	3.706885384	2.362956774
0.15400381	0.150440395	0.154411205	5.188837665	2.912513459
9.167000126	5.546175745	9.201468209	4.651113055	2.494988059
2.202103508	4.691474462	1.651386537	5.39035111	2.201119622
4.93728557	4.374802007	5.516628558	7.064942886	3.373230117
2.132006314	3.319613463	1.099363544	5.407036055	2.726524189
0	4.591420192	0	3.120267003	0.966953769
1.004797799	4.651594665	0.00149392	5.467279117	2.591901713
0.069893788	5.948961317	0.070050896	6.966650731	3.899006247
0	4.618944903	1.44494438	6.234303286	3.627468545
0	3.550209365	0	6.681211216	3.352385801
0.966416461	5.645083324	0	3.946140613	0.965567093
3.786142819	3.31878735	4.658446849	4.98205646	4.021254463
0.388618513	4.122175215	0.390085461	4.510181463	3.555517887
0	6.653180527	1.409194647	4.761002866	2.117080486
8.000588735	4.325127698	7.974651056	4.908314357	2.204858163
0.864630259	2.600359459	0.864907194	5.652478275	2.383742059
0.427654057	3.304111758	0	6.312689912	3.561101608
1.687139509	5.419272118	0.026557151	4.888282467	2.111754926
0.004826721	2.607536676	0.004836095	5.112634527	2.607326524
7.065866189	3.459336722	7.180600771	5.354269042	2.447584105
6.076581148	4.611600075	5.879496877	5.353697957	2.686675314
1.792247377	5.622980923	3.197267339	5.544468894	2.732567202
0.114568765	5.099497271	1.34109945	5.101404005	3.051515764
0	4.553588468	0.376677786	5.697048245	2.98032212
0	2.986446665	0	4.040749358	2.675132425
0	4.706406213	0	6.070813328	2.919623231
5.377082078	2.675099808	5.313051273	6.119880679	2.676358132

1.194839974	6.086418675	1.194378744	4.386548095	2.857343498
0	4.00670981	0	5.698162855	3.963806334
10.00825	2.806372645	10.56645537	5.124714036	3.072702826
0	4.946019034	0	6.398471982	3.288238372
3.824655698	3.824394175	3.157841927	5.18612368	5.65020749
0.037118713	5.271877103	0.037196429	4.504544695	2.154679723
1.077989061	6.12540172	0.02469114	3.705247634	1.681383067
1.01044424	3.435669353	1.359584388	5.866996484	2.805224666
7.250128174	4.105119676	6.843113455	4.736251234	0.412351505
0.107960284	2.421745862	1.972076561	2.76063076	2.418177489
10.785739	4.038018151	10.17237116	2.931062016	0.024638954
0.099973494	5.100690441	0.100212393	4.848777443	3.597048575
0.023333942	5.510817594	0.023381287	6.141865884	3.108683644
3.938589871	0.241043263	4.274671624	5.145217395	1.697674443
8.396515816	6.791977027	8.796774732	4.948655485	2.07567655
3.415041557	6.061671668	4.27600574	4.708529946	2.281714357
0.085211738	3.703668864	1.258390234	4.197085628	2.672164399
4.400385013	6.277609815	4.601525451	4.994389923	3.701178564
1.563978717	3.673495927	0.982968206	5.994180732	2.974425219
3.903521511	3.235467071	5.215775293	7.933686478	3.424192707
0.154111827	3.432743483	0.154519586	4.985266614	3.418191911
0.553334931	4.811105097	0	6.275484544	4.319193968
0	5.548578831	1.578526835	4.749575786	3.398559015
0	2.47258414	0.586311482	5.209179634	4.118230916
0.177102504	4.044497671	2.18762921	5.796949325	2.190942369
0	4.774488187	0	5.211072427	1.852103476
0.119311471	5.289144066	0.119607482	6.29620728	2.795260585
1.241498715	7.318200544	1.240917413	7.83748637	3.343740537
0.020692211	3.686394672	1.065525208	4.628089004	2.90290235
0.011873624	5.148563267	1.037836818	5.061222901	2.050910665
1.605095882	4.78674518	1.904461931	5.817094294	4.684116361
1.487594299	4.624978909	0.921364646	4.570366374	2.880335601
0.965460222	4.403418604	0	4.528677064	1.541796707
0.030963826	4.545764835	1.097347938	4.909797288	3.142776305
0	5.392339098	1.972230672	4.551348863	2.292659125
0.122124142	4.709891145	0.122428756	5.593687519	2.466672793
8.880286567	3.912282572	8.699172143	4.040837489	1.957666165
0.011939213	2.375672064	0.0119628	3.380418491	3.056471977
0	4.132120242	0	5.762743011	3.639968573
7.356602948	5.321438689	7.962483568	5.968875526	3.379419533
8.713203911	6.634885539	9.800283033	2.146043996	3.622952702
11.03093952	2.171112092	10.90970939	6.589213829	4.379624133
1.260101364	6.424625221	0.085787545	4.718625558	3.835224694
0	4.812764616	0	4.56146333	2.118292357
0.093852428	5.757003248	0.094073988	4.823818235	3.400732808
1.781736105	2.546761522	2.545150433	3.947947454	3.559892278
7.993290481	2.345418002	8.63118016	4.670647157	2.345277586
0	4.884220996	0	4.090038718	1.868191378
1.554079476	4.790732312	1.554795053	4.389972502	1.548399157
0.755537316	5.033789912	0.756007356	5.216229864	2.614118117
9.660390961	6.470964954	11.12953683	6.742925375	4.298460305
9.980433736	4.363286014	10.27605448	4.181872991	3.723142645
0	3.502629463	0.948083408	5.083947769	0
0.978955331	4.773111586	0.979000899	6.565033704	3.289823942
8.224621725	4.583615405	8.843066508	5.954907026	1.858512732
8.760572072	3.946033334	8.711005045	5.403912134	2.972562013
8.168989679	4.788011053	8.909810639	3.517269272	2.17167986
8.571286402	2.510745173	9.195326246	5.526830981	3.85545239

hsa-miR-452-3p	hsa-miR-144-3p	hsa-miR-34a-5p	hsa-miR-188-5p	hsa-miR-542-5p
5.096180732	6.071886897	11.07075266	4.748387188	4.064378781
5.595504962	3.498256291	7.59166015	2.6281206	1.856422666
6.263073314	7.395595374	10.87941591	3.625042771	3.330169136
5.221672134	5.414587039	11.0552117	4.337837387	2.401723173
3.320594808	4.119773918	10.2781029	2.812900714	3.504181531
5.642134899	5.535142112	11.78406599	3.823061754	2.538108308
4.702663413	4.791996658	11.74991576	4.863732693	0.1479241
5.239468209	4.278118911	10.93604076	4.106603843	3.803758387
6.538506842	4.467775105	9.772243959	1.902581896	3.061269204
6.979089402	6.366614694	7.347857553	4.753507725	1.767506481
3.280291383	2.148501189	8.91575891	3.967661213	4.086137136
3.181748624	4.935927213	10.94722991	4.366728197	3.434622612
6.158294699	5.973694185	12.02300286	1.992682691	1.98978675
7.953348851	3.672245612	8.947387973	5.853657898	1.56272597
7.389061647	2.718536196	10.75122474	2.259407589	1.574930151
3.370097648	4.378551541	10.06553842	4.205401873	3.193640808
4.792851144	4.934087881	8.941185586	4.129740648	3.636485308
3.757616411	6.275163476	9.425689442	2.625574217	4.626392161
5.430082368	3.957816173	10.82479626	2.934570978	4.06144565
2.420659067	4.145863801	9.886493025	3.520227261	2.410212358
8.373268589	5.680367665	11.95169263	5.074395417	3.001260616
6.924242375	3.027344351	11.69924859	3.376444084	0.265620859
7.345241419	6.803752378	12.5939742	4.462154913	2.712543771
5.245416773	5.183850659	11.12994073	3.622303567	2.563807773
3.421991016	4.553010384	9.242386213	4.333903268	2.899405903
3.731895516	7.422514372	8.557219901	3.399745442	3.204862495
4.966371491	4.490216472	8.605817609	4.96680938	1.972249084
5.661309282	9.592334548	10.60307852	4.099114637	3.276535675
3.978942825	5.801570885	10.47181238	4.644510035	2.342800294
5.006483591	6.523928856	11.13446185	5.196673418	1.739778443
6.691834721	5.695035578	10.37870924	4.204423169	3.199480414
2.321513356	7.189339361	10.41736285	4.32146149	3.806904385
6.535348091	1.637807455	11.62455451	3.580620794	3.119467521
6.245400823	3.513114255	11.35712123	5.267539586	3.521594196
5.313526792	2.388075387	11.3578099	3.426466202	1.944913652
5.75820487	2.509935406	10.68729862	3.33384355	0.252742932
2.841441753	4.898482473	10.24431123	3.719476009	2.83174218
5.838128781	3.786730902	10.90127218	5.52628067	1.356097408
3.375392842	5.206439154	12.74226153	3.840788501	2.670761906
7.112376711	4.968715479	11.94136708	4.693731636	3.562824518
7.724753197	4.26772298	11.94844596	5.592919352	3.234219455
1.20755566	4.904407306	11.03355748	4.560923904	3.097921837
2.839801194	3.115943411	11.25251248	1.392444421	2.491532368
5.578318442	3.98375319	10.31833631	3.272098074	3.438894123
3.706018175	3.071971157	10.06771916	3.706682867	4.070405997
6.886821674	7.682652238	9.145837982	3.881256722	1.899340048
1.489025072	2.621084649	9.89756158	5.022309327	1.494296327
5.408632361	5.067581979	11.77371306	3.34385872	2.251360777
4.32319928	5.699598877	11.43349314	3.065907834	2.784040439
5.838873546	3.245800783	7.498148834	3.951318605	1.416657435
7.047607995	3.713961323	11.56556657	4.10105077	3.182808104
5.866863705	3.802583155	10.12576085	5.23404212	3.515862853
7.609785401	4.261254649	11.54258161	3.24831782	0.049753483
6.132365769	4.693204126	10.99806685	3.912366214	3.422966886
2.875083193	4.717332174	11.25498711	4.394064702	3.237978641
5.338054737	6.59561045	9.698685913	4.08090834	2.246723537

5.588531167	6.754193148	10.37272711	2.901815937	3.398512988
7.130949614	3.591669507	11.23112413	4.779662705	2.607270595
4.398738024	8.657045383	9.963123331	3.674662551	3.107761048
4.551487434	5.145061987	8.265393388	4.748158643	1.83675154
5.733322049	5.46380368	10.33290903	2.149302096	2.476037445
5.652706013	5.00907887	8.720716125	3.063338715	3.547504953
1.191929727	3.710710928	11.07130734	3.713740685	3.478518171
5.53972834	8.031361127	10.87213996	3.214536589	2.039441008
1.55363672	2.769485591	10.23242678	3.545324686	3.66141859
3.104819626	3.301542521	10.64224757	4.706675338	3.759114154
5.586701085	3.747948077	9.952895149	2.58675193	4.189723498
4.561945382	9.466261374	11.19841885	1.980749041	3.300423234
6.658480863	4.92655234	10.63876727	4.926921448	2.782448855
5.898646047	4.3681519	10.41240845	3.777857375	4.304794892
2.484619071	3.890030722	9.374385118	3.06465524	2.706036876
1.178834234	4.735525415	12.03488156	5.028226905	0.05211397
2.744666853	6.769306674	12.57237891	0.19596111	3.582409183
4.710921055	5.295981969	10.0674314	2.008763869	1.006640069
1.987529864	5.338693723	10.25633593	3.197926573	3.165153175
6.219442486	2.364536242	10.62100353	4.507394995	3.044720333
2.618715161	5.552961136	10.94871982	3.728979292	3.413581215
4.830644158	6.121262296	12.16936479	3.531775931	3.530337449
5.35352445	3.185966963	11.2046524	4.177258083	2.549269067
3.73646733	6.60750082	10.8746939	4.117095561	3.396103288
1.4969901	3.972536113	10.45137301	4.639333256	0.92732275
5.218469467	4.847586445	9.893799228	3.576973519	3.859573826
6.169098424	4.879041073	9.029610199	4.478251101	3.020954143
6.555210112	2.780538834	11.99017179	1.975626351	4.059578547
7.349890314	6.028709925	9.461085908	3.959393801	3.881649878
6.548350588	3.90947408	8.279436982	4.49649311	0.06370011
2.368110919	5.342528523	10.45679701	2.370537442	0.208869423
6.719135245	4.985669515	12.50425057	4.453398073	1.457698421
6.841796247	5.480092088	10.03922906	4.768158602	2.100394715
1.26710245	9.914655248	11.35980768	4.774674805	2.416126358
5.010260842	3.361627675	10.20497398	4.519445454	1.572636833
2.72796417	5.914944591	11.93156735	3.852908979	3.469095681
4.195847745	4.805172233	9.811506402	2.759091527	4.119598067
5.562447865	4.095117297	10.31974966	3.707934425	3.329169298
5.032994755	3.31297392	10.35151881	3.311492403	2.612855583
9.124544485	2.416789046	9.62095379	3.279679945	1.442494757
3.110411322	4.490874504	9.276701715	4.920006988	2.665787716
5.5591622	4.989186133	9.417198297	3.269896999	3.755331044
5.589027381	7.286983749	11.2137903	2.288449287	1.851246901
5.461418552	4.332118175	11.49314003	4.308700853	3.545263655
7.183138349	6.149786264	9.685383043	3.471361359	0.854535061
5.185280471	5.289374194	11.08624143	3.042839222	3.332535282
4.900887705	2.600754737	10.09585579	3.948071294	3.246821503
5.389745723	2.543091485	10.09812183	3.307179338	3.774340402
4.888340056	4.131119456	10.72965145	3.448640318	3.122346361
5.41759312	3.609154138	10.51963016	3.831589699	3.345255118
5.668919436	4.141465176	10.31413968	4.388787292	4.136646516
6.337365691	6.819945275	10.34471502	4.305663446	3.841822129
6.459845066	4.682203098	11.20857405	5.532345237	3.480237889
6.356130869	5.564802666	9.906247722	3.058033162	3.04906754
6.657103354	4.525588375	10.03464741	4.083675775	3.129176026
4.502458938	4.963547276	10.32024658	4.10999017	4.180936203
2.635940821	3.952418456	9.636786169	4.673558159	3.767387256
2.865740174	4.761849915	9.103814292	3.859328666	2.197333124

6.629446127	5.732383107	12.07027956	5.053733997	2.856122382
6.524184387	3.563009158	10.59917504	4.165848464	4.076711397
5.866812134	2.015780095	11.4622912	3.309887191	1.36593987
6.100096629	4.896536234	11.0103703	2.260902567	3.051914942
5.02187008	4.768747914	10.73410146	5.989620496	3.347325955
4.351898681	3.98729572	10.52362903	3.635543665	3.981419688
5.295065661	4.824770599	12.23872414	3.578265897	3.28495251
2.680156754	5.087732129	9.742470767	4.419884911	1.86966367
0.472767713	4.352065405	10.25661468	3.44171042	2.922694714
7.856378249	5.192170789	11.15649489	4.174894834	2.753221399
6.475972858	5.527677621	9.171202807	3.713756756	2.704518624
3.433492128	5.820666326	11.44021181	3.233214397	2.724676625
5.73395893	2.425311213	9.96049659	4.639612122	4.360463346
3.217048855	3.736349276	10.35237947	2.397075025	4.37043448
3.255543523	4.411698738	10.85384977	4.947469292	1.057928936
3.126219165	5.596907994	9.707165465	1.549513376	2.543775899
3.177645224	10.17007699	8.970692069	1.264233711	2.336393206
5.413644146	4.874601736	10.29371545	2.853737701	4.07782542
5.779190635	4.220434911	10.46523745	5.016827504	3.144218165
6.891982741	4.149091018	11.79381194	4.027023776	1.948942081
3.633443495	3.200091457	11.87814993	3.803176252	3.612432252
5.666381435	3.645763627	9.912683406	4.953573184	3.213282595
6.185127683	3.567407766	10.10752277	4.894961847	3.736842629
4.523808378	5.687702968	9.963528288	4.455247269	4.508188645
4.046149389	4.993059288	11.96484157	3.881619	2.188785615
3.992972876	3.824047779	10.7029071	4.821774188	3.915921107
1.357742227	4.106878051	10.68617806	4.771906494	3.492128612
6.104378082	5.802201494	10.95623737	4.612149082	3.341839947
4.3518183	4.190910637	11.03281215	3.421618975	2.67852569
5.646621038	4.520106169	10.35966609	4.146957655	3.378733069
7.558350395	5.169792283	9.867583291	5.546807484	3.844571042
5.190535353	5.29463848	11.85234268	4.194142162	2.469504473
6.896654264	4.643983563	10.40229772	3.945033138	3.117698807
5.582589508	8.196866672	11.81932381	3.733788761	0.028275267
6.155567096	5.747167322	12.17988821	4.551714903	3.29051218
6.662306707	6.91249971	11.53621548	3.514117044	2.464813075
5.169443888	5.720478081	11.34968016	3.613901067	2.397312041
2.863786153	5.920154867	12.81862536	3.22765919	1.632217954
4.308598352	1.678219978	9.704818668	5.428210555	3.071994293
5.799220564	4.669002973	10.4074319	5.10571268	2.855168302
8.306206323	5.699186597	10.64524449	4.756009251	2.473265442
5.746957957	5.853795854	8.486839196	3.792243233	3.435865171
6.404595787	6.23752857	9.223963493	5.482221642	2.337751763
5.456417927	5.814386129	11.49974536	3.883901703	4.357178086
2.370473257	5.864377166	11.65652901	4.672312748	2.36727245
5.541506712	7.718288174	10.28081699	2.815577593	3.939856948
5.885184838	1.016704743	10.18875856	2.832006618	3.484574896
5.987305572	5.720380256	10.23404063	3.546511939	3.022210376
4.95404573	8.951632463	9.484808824	3.362578637	3.370765358
4.576036226	3.831735881	9.911816276	4.388824678	4.54396125
6.04577951	1.478893156	9.20190114	3.30954769	2.498081722
4.738427842	2.839678923	9.795755902	3.434686866	1.804449511
4.499807372	2.920546472	11.01068976	3.377950732	3.726384071
7.679258681	3.426534656	9.448514549	4.666563635	1.55872526
4.00070983	3.918684538	10.49458937	4.223574666	4.083374946
6.629345656	5.379662828	9.66041173	4.293132284	2.343235581
4.788677544	6.962645367	10.27197104	3.516074962	1.132644928
5.224236438	2.295946001	10.24445474	3.769185823	4.328339393

hsa-miR-195-5p	hsa-miR-1266-5p	hsa-miR-326	hsa-miR-891a-5p	hsa-miR-335-5p
8.362439901	4.082193213	4.078424742	2.438228331	4.504393229
7.185135235	4.144526416	2.294083559	0.07245287	3.919850596
6.033941151	3.162369987	4.814765177	5.532481466	3.626143742
6.000241271	6.092699263	4.089488981	1.655276672	6.09295079
7.34151066	3.089501714	3.087873012	4.542210867	6.226060935
8.926013953	5.335534427	4.147682882	5.106511374	6.460133958
5.414436569	3.850958336	3.670851594	1.483582763	4.149779965
7.704565437	4.014021401	3.564197817	3.914269648	5.358121303
8.30981808	3.471721058	2.699818489	0	5.763597251
6.46697182	6.36522539	5.624640413	0.047591424	3.928057414
4.50133597	2.146838384	2.881364793	0.591487967	4.212513127
7.151419349	3.699023422	4.825383105	0.234362845	4.379462326
8.543006093	2.437653382	5.086112143	2.435905162	6.321226803
6.493893157	6.328142288	5.01553193	0	5.328265792
5.283876453	6.285389761	3.577928864	0.19768077	4.718833947
6.323282779	5.459676315	1.749927955	1.134477924	4.854386805
4.12430699	1.90758419	6.274659328	1.673574342	4.701310635
9.295193824	3.469252797	5.338394627	0.371840602	7.236225038
7.892147214	4.465515521	3.827488443	1.886406238	5.026879123
6.641175507	5.591364389	3.24034733	0.255528901	5.089433198
5.581752497	1.531698141	3.902294144	4.565196749	4.563704411
3.381794992	3.668503797	2.553211401	4.112115216	3.669586546
7.004097135	2.714479656	2.714346344	2.447344253	4.534288245
6.842514457	4.099682299	3.62303712	0.15157104	4.903543684
8.299066882	3.189414817	3.948736312	1.442737613	4.97312386
7.023661705	5.197729592	7.998503742	4.792594837	6.644530162
4.054520793	5.251755708	3.774929921	3.774731689	5.053828956
8.700654835	5.000681906	5.34272105	1.194340504	6.304563406
6.888994153	4.304430063	3.380627774	1.265209734	2.95557443
6.645673569	3.777356037	3.357695247	4.283599341	4.657105417
7.650652816	3.724261622	3.10380494	0.948126715	6.777039066
6.339364868	3.999533678	3.458977095	3.806891204	5.208974795
6.876240707	6.013928091	4.974804651	1.873369743	6.096517327
8.107466808	2.940039612	3.311545291	2.228484835	4.984016326
5.744607696	4.02600634	1.303659567	3.000368007	4.143861452
4.245494464	6.241381096	0.274308978	4.907055487	5.005772237
6.110811231	4.020240382	3.346932258	0.129923193	5.036357853
3.167772447	4.647416854	2.716899353	2.059776684	5.444588618
6.237193006	4.641532898	2.340224662	6.896712919	5.986306338
6.452423919	4.569017826	3.426901618	3.427323724	5.320517884
5.93480686	4.550149615	2.74070956	0.102063391	4.787894219
6.182950208	4.011799405	3.626792304	0.066691633	4.710065377
6.873677586	4.372997786	3.543944179	0.129458578	5.161281308
6.739268458	4.747987891	1.190158105	1.811487011	5.577862056
5.649165233	5.480025198	1.505825595	0	4.145103028
9.75273645	4.463597917	4.272269593	2.475977602	5.400336302
5.0361659	6.003074516	4.007134947	2.967055913	3.856701348
5.736910696	2.72004244	2.718971919	1.567863176	5.140816146
6.288692346	3.298489056	4.976071918	4.097005238	3.497508476
4.027177826	4.730307683	5.504153424	0	4.819838721
6.782416798	6.932974107	3.598344364	1.595551713	4.598838437
7.981735612	3.869051978	2.45387255	1.158778388	4.68409135
4.167318665	5.551768045	5.069656953	6.809273307	5.51486872
6.586397372	3.425470975	4.01233624	1.068162769	4.499980749
7.245169802	3.771841547	4.596060697	0.014314165	2.87511865
4.141435255	3.942357207	2.95943684	6.796130623	3.624241722

8.066155925	3.985032495	4.566131808	1.436473228	6.893150075
5.389596537	3.869680948	2.794466171	2.794624463	4.777305541
7.213448981	4.660396339	4.449652107	2.351467035	2.534628972
5.698759873	7.195313001	4.851181477	1.544163336	5.460709552
7.075524755	4.6349922	2.970526541	2.744961021	4.635289373
7.888658902	6.516277973	4.535781188	1.777393241	5.580464762
8.081913619	5.468872307	5.334188539	1.861947581	4.735313478
6.663097123	2.850988486	3.630507317	4.80233931	5.539660938
6.876612135	5.206426909	4.207419007	0	6.350384446
7.762270125	3.768437811	4.774610362	1.835344751	5.5316318
6.494236943	3.874676248	3.084013634	0.061974618	4.6184645
7.889225108	1.980798844	4.369610333	0.985760935	6.042694669
5.753396299	4.432119736	5.101917753	3.558398125	3.879970431
6.647970292	4.759238383	3.176304123	0	6.121211014
6.424614033	4.211058183	5.64279867	0	6.409613706
4.921218492	3.853484565	3.585990464	4.272960674	1.801639937
7.260177473	3.609779736	2.744537978	2.279370529	5.24034858
6.595671446	6.868605766	5.367994077	1.006499683	4.765369407
5.342044966	4.630651291	6.489811308	0.372582761	5.508167949
6.026900106	6.297240289	2.364679488	1.030394551	5.508626253
6.334208358	3.455024449	2.619695695	0.330686529	5.309917814
7.601078464	3.070812875	3.531841254	1.047360769	3.981012603
5.93637659	5.202840158	3.186595759	2.547210186	5.202303613
8.21584423	3.407019703	5.009703978	2.705520212	4.888657858
4.20620231	6.148477579	3.211212475	6.869319428	7.100212692
7.904937689	3.0959775	4.464448204	0.857538401	4.969483913
9.35217414	5.093074096	6.015603646	0.047914525	6.925096657
6.057686596	5.947736939	4.293208819	5.058024953	5.525063333
6.521172237	4.309692976	1.822721886	0	4.108899544
7.055335516	4.855789629	4.413942056	0.069971799	4.500332232
7.309180172	5.26570348	4.503791283	3.706789568	7.188966137
6.134847354	2.123863449	2.125042905	3.432402572	3.808636911
6.277260038	4.652091836	5.611911232	2.493149609	4.201579662
6.308260325	4.7710029	4.734827371	3.891075136	5.390644879
7.127647137	6.508939143	2.005480046	4.136402892	4.755414454
7.127263926	2.4610746	3.319066235	7.39003496	5.481025932
8.37222994	3.75608624	5.0351118	0	6.11650757
7.920855982	2.814503256	3.592431064	3.707984967	4.91467924
7.939942627	7.770660021	3.638877318	1.845018978	5.656559987
6.479479566	6.094213699	3.624776993	2.825170688	3.984049227
8.10705349	5.694767715	5.563068436	0.785274169	5.329422155
8.353070914	4.337933946	2.757031778	0.966292448	6.211083168
4.239498587	3.646495946	2.622460948	1.849946996	1.220781332
6.201658562	4.50574075	2.753766647	0.385621618	4.811435039
10.08061578	5.540810335	5.489684614	4.969393495	6.664471843
7.415741243	2.683414876	5.150433412	13.66918226	4.38885463
7.116063954	4.298194842	3.779030588	0	5.724094364
6.664120222	5.192456651	3.014825326	0.840730119	5.982518369
7.223821062	3.937414371	3.295754394	0.026391679	4.131024148
7.320252183	4.668793854	5.024849293	2.020794228	4.779916157
6.622495733	4.311781896	3.458762598	3.724206726	5.39268731
8.225552473	3.573599198	5.656560641	2.684566289	5.04693999
7.224405303	4.123180668	5.118145231	0.644151704	3.766265252
6.047771336	5.517435591	5.797566848	1.34337384	4.205765174
5.70572382	5.810199838	5.430707501	2.428261889	6.469922852
7.743756701	3.971444848	3.245340635	1.713221761	4.643815344
7.209105379	4.23299951	4.016405444	0.673814881	4.741672578
3.945395179	4.379968521	4.664074939	5.063658754	4.499704429

5.515487464	3.285146201	3.993468674	0.062904475	4.204284918
7.921688889	3.635543831	2.921904346	0.612594546	5.698644795
6.480238813	5.294125621	2.805509333	8.82713383	2.465031013
5.095132556	6.222296681	3.919291082	4.355144495	6.624656039
5.46882614	5.284937887	2.929730033	3.356343204	3.530133395
6.831795672	3.172696984	3.985861146	0.036959052	4.504452498
8.476099588	3.440626793	3.287195502	2.430838259	9.36238417
6.805161298	6.119870441	4.628293371	0.54964009	5.597555951
6.760183009	2.967318388	2.961437297	9.5290025	6.252033338
6.857847558	2.761040191	3.790846994	3.034651016	1.975774501
8.508024557	5.343966609	1.689080962	5.041496889	5.779210606
7.835250553	5.708063816	3.899848527	0.09948315	5.414242212
5.763474916	4.817996679	4.816961678	0.023236656	3.699044738
6.880885605	3.735070359	3.493762063	0.246143167	5.79033453
5.735660852	3.7883144	3.994867657	6.987125869	3.788382825
7.061078573	6.492203906	5.654150833	6.102582295	5.975393064
6.185415662	4.562762771	5.147612708	2.675265375	4.855378259
6.848786399	5.991124956	2.853497018	3.461282208	5.838271701
7.004985846	4.557314108	3.432881109	0	3.972731747
7.363563189	6.344868971	0.095828963	4.14914531	7.019400271
7.171729958	4.352100154	2.921348168	2.121172228	5.123816188
6.824618289	4.691833859	2.914369765	1.87632338	4.690806917
5.882977334	4.224992366	4.378554452	0.69662305	3.996151956
8.215361318	3.506740643	2.747429345	0	5.552168057
6.150966405	2.194271208	1.519358668	6.592229473	5.270602108
7.530055581	3.412138349	3.633610754	0.891749671	4.907039023
8.132638958	6.346357839	4.985974143	3.307787366	6.094618805
5.181137164	5.46028656	4.268978101	2.652077065	5.179840022
7.965481709	4.009568907	3.685892641	1.065924882	5.39106374
7.271845416	4.452743802	4.51947288	0.011825423	5.646530477
4.709671875	6.125250345	4.095206736	1.902842116	3.602181598
5.954800596	3.459517037	3.574886124	4.263067228	5.22625675
6.227809456	3.752386386	4.801998266	2.947455786	4.943331309
8.112787655	3.316643674	1.705473203	5.616045576	5.062057806
7.063519507	1.559272364	5.358713582	2.968600887	5.580636835
6.785562933	4.788991809	3.085837055	0.121499127	4.539086132
6.383257691	2.401978496	3.909318654	6.429759858	5.756876058
7.554624715	4.308476485	2.863741583	1.038273613	6.601380171
7.071982255	4.771278609	3.413802403	0	5.922285058
6.564440406	3.88258235	5.050764357	3.455662892	4.773026971
5.032402492	6.057809868	4.07578459	7.52598691	5.903402983
6.185498689	4.564857718	3.678802187	5.599463732	5.719836592
7.149143197	4.197948841	3.177925387	4.198890093	5.72396021
7.080462349	3.427070689	4.189182795	0.480432354	6.351322034
5.390920327	5.240434127	3.21009647	0.093397629	2.982009949
7.911040121	4.573494407	1.784363987	3.042530569	4.712154054
7.027453344	5.419316092	2.609149597	3.02503806	5.755057371
6.944833958	4.163931383	3.751672966	2.851773394	3.843389743
8.356997957	2.646863541	4.89576515	1.848169537	5.157607972
7.128616235	3.057641696	4.226124635	1.660666484	4.999939703
8.409120829	6.646145201	2.911706379	1.479767097	6.108151418
6.329471012	4.670149952	1.805322362	5.411621256	4.864325752
7.48987622	3.240692663	2.920604171	1.929598076	5.043407737
4.720539105	3.551874205	3.289385842	2.292124288	6.250601443
6.072803828	5.411794827	3.420294669	0.896368981	4.734851284
6.479314725	4.567033402	4.80337653	3.089472181	5.175072778
5.698626323	6.415580883	4.199011337	1.130006913	5.111447744
7.17279294	3.588947937	4.483540548	3.148425846	5.16208089

hsa-miR-501-5p	hsa-miR-34c-5p	hsa-miR-6503-5p	hsa-miR-9-5p	hsa-let-7c-3p
5.593151184	3.280589089	1.924101488	9.771082548	4.901191963
3.917160214	1.857757624	0.042529834	9.75749553	5.252633916
3.333356906	3.624226559	1.100272723	10.69578622	4.757569896
5.48362268	2.077061312	0.01105324	13.5773278	5.61621974
3.087706468	3.085359583	1.321983728	11.7923755	5.874342403
3.823214603	1.161545098	0.030257887	9.33655808	4.947792378
2.961582448	3.23518766	1.41808229	9.678162687	6.20788994
4.277047408	2.091999208	2.077127937	11.53528936	6.665706368
4.206523596	3.886183392	0	8.533716893	3.792054421
5.5173905	0.045211919	0.028535551	9.425142319	3.219604934
4.262572288	1.057171395	2.020590265	15.24365491	7.279953928
4.226306617	3.452181839	0.121275075	14.29859459	5.186456175
1.992674202	0.105601052	0.063601127	9.550642552	4.673846649
5.39732457	4.293587547	0	11.78705346	2.558847336
5.981600649	2.716131042	2.124373434	9.316744448	3.961489256
4.293450177	2.507748278	1.122385379	8.732367403	3.523816108
4.388924502	7.168502783	2.962188507	12.81285225	4.216047677
2.04438639	1.726184938	2.17249614	8.530004232	5.495179778
2.934701349	0.077097215	0.047454491	9.348241169	6.496624484
4.290385462	0.237340739	0.130243224	10.48127548	4.144907498
4.199066063	4.320891588	2.090159917	9.079460923	5.412837637
2.552999435	0.281394475	0.150047644	8.90831353	4.949211325
3.945915377	2.713949498	0.017303687	8.464271622	6.051570454
3.19185076	1.454171996	0.083451103	10.38771316	5.468835309
2.567608954	2.565914548	0.082742877	9.476818944	4.735581218
2.829444969	1.57797486	1.625008106	9.218755854	6.020324227
5.490195187	3.668319191	0.980829729	10.61926435	4.426166951
2.588811652	1.19790769	1.785698566	8.314585192	6.059094366
4.39495651	1.269990359	1.85443927	7.790153489	4.570887578
2.990810276	3.891248239	0.024182748	12.18501761	5.844576044
3.34301552	5.114861678	0.514036738	9.584221202	4.866500964
4.08699845	3.458979422	2.321582789	13.24521133	4.321456958
2.551153993	6.111529335	1.705299281	8.434981645	4.862060453
3.994440306	4.126658578	1.314808892	10.17861038	4.594030384
5.147983579	2.725534718	2.314842372	17.4698317	6.629312932
3.616549477	4.764654305	0.143897402	15.50149397	5.101190177
3.875388074	2.051006907	1.340034827	6.983268287	5.752168637
5.575695252	4.092654381	1.380037149	10.37916995	3.509943431
3.373514493	0.080259909	1.232503284	10.21791701	6.284756109
4.195268199	0.02068437	2.077767616	11.22867434	2.909203786
3.90973947	1.31517792	1.896430254	7.325248016	5.681366012
3.765909303	0.063225214	0.039339096	9.625523029	5.60726634
4.367613823	0.121894485	0.072527564	7.913274722	5.790109767
4.677737109	2.578681931	1.169150586	12.10141365	5.719133071
5.388058113	2.712352754	0.940673003	10.46072757	4.849815288
3.689284825	0	1.503247955	8.916064875	3.967508256
4.275635995	2.166309015	0.090769531	11.36321257	2.622063531
3.577449187	3.06177571	0.105070328	9.629889756	4.389468137
3.296226874	2.006254774	1.308197467	9.759051796	5.433778188
3.37069863	2.12870116	0.873642939	8.732614371	4.301218038
4.261576676	1.595679189	1.00799743	10.67878515	4.920846461
4.760797862	1.817031833	1.573009846	9.428035509	4.608812683
2.225163986	1.173541572	1.154285496	11.62605799	3.958891846
4.566150797	3.562622257	0.01307941	13.42793102	4.013119763
3.978352358	0.01364806	1.634169038	9.530980749	7.35089623
3.712487926	2.45936934	0	9.002226798	3.532517522

2.344918335	2.651113658	2.754953537	8.486086635	6.276091986
3.486746618	1.41783892	0	9.797792231	5.729166993
4.084066643	1.591138002	1.247158375	11.69763276	2.844725469
5.305681894	0.668486309	0	7.452805632	4.706518646
2.970484802	0.033704576	1.102438526	10.6536019	6.011713569
3.198814483	0	0	9.144056429	4.853926813
4.500801973	2.105366708	1.609462537	9.69246458	4.806859314
3.504719062	2.363386685	1.027263138	7.919002116	5.85528717
3.866770423	2.547919155	0.9763576	9.989514545	3.767111009
3.62722431	1.211082972	1.186696607	15.02867345	6.894558507
3.989316649	0.05878361	0.036703549	7.445517034	6.624689113
2.978517729	0.985492753	2.567941043	8.161156793	5.953894187
4.879608529	5.399073758	0.984231927	9.029672993	5.61603527
4.37403977	1.402222277	0.594501732	8.503350504	4.960615788
3.064618445	1.907037329	0	9.675675624	3.352022049
3.259545477	0.054195964	1.161226135	7.693318851	5.365832767
2.744346253	2.28240351	0.108843681	9.885622462	5.942644488
3.179795881	2.594382554	0.00125192	12.49738372	2.816995646
3.198754438	1.994269517	0.175495231	7.350568059	7.02732509
5.692454647	2.364604654	1.028064604	9.501385248	5.334516734
3.092868461	2.615200532	0.160084393	8.754950205	3.979727729
4.322123333	2.063485243	0.009099597	7.785031334	6.947275635
4.040469349	0	0	7.213409077	2.075688452
4.000435665	1.288213461	1.252209933	9.3706362	6.912390898
5.37531377	2.220437931	0	11.72271375	2.700417462
3.576863388	2.59058507	2.146928792	9.140228785	4.462485579
1.152848617	0.045517259	1.741996059	13.58981722	4.935492638
4.671540626	0	1.565385506	8.903614317	4.292971405
4.54165002	2.968768586	0.878234333	12.02465069	4.245626281
3.116808239	1.22056395	1.194827589	8.461826066	6.065387041
3.184395941	1.675780132	0.122222754	9.410932084	3.909143869
3.803269819	2.918230277	1.391800167	9.23478652	6.286666975
4.307459726	1.554686303	0	7.803617648	4.993817805
3.662241307	2.599444649	2.474809894	12.14737169	5.28605505
5.407692303	10.51130396	0	10.3113191	3.745377893
2.133232857	0.029963495	0.019145496	10.43398557	6.730136476
3.948703442	2.759214704	2.546056407	8.78816966	7.674288077
4.095051816	4.095004709	1.590165787	11.51133325	4.708202127
4.573549911	0.065934381	0.040937725	8.817013943	3.486250618
3.624843765	1.844339109	1.458616828	9.335037282	6.393031684
4.679755183	2.97746172	0	9.355061198	4.632278635
2.535649662	0.965657384	0.968606702	7.443527239	7.25700739
3.490293256	1.852540153	0.041713869	10.21061458	4.728754683
3.586722158	2.022841163	0.180128856	9.388757606	4.668416029
2.779669416	0.855079134	0.865645188	9.770043969	5.845853568
2.874302219	3.455649204	0.922968674	15.04580074	4.619528341
4.09909299	5.097300908	0.871798391	8.544424063	3.777796006
4.410175573	5.801108795	0.433575963	8.642895173	3.84646104
4.7754298	5.989612474	0.016120667	15.2402584	4.717884132
3.609016242	2.020934359	1.60172229	12.8903595	5.484692967
4.046693034	1.092552639	1.681123713	12.06131083	4.666789107
3.667079459	2.497160358	0.808486378	10.48210376	4.358930864
5.293538861	2.72939564	0.655532708	10.2614956	4.01329322
3.815752404	1.349405922	0.064658555	11.16633206	6.476725983
5.499291141	3.428049435	0.380155693	7.423824146	3.36417163
3.12204416	1.710534564	0	9.766958493	5.617035812
3.826654243	3.150595675	1.202442983	10.89964269	4.550737422
3.766550723	0.910745949	0	9.918499875	3.765694594

3.993316767	1.199209251	1.17648268	16.08460833	1.822404946
4.392252463	4.013429286	1.501073044	12.3968438	4.993841853
0.115065805	0.113017007	0.067690505	11.06123876	7.022693692
2.401311809	3.20673963	0.483986668	11.71438002	2.402302006
5.583985281	7.561815634	0.046753611	10.63664773	4.177592919
3.985772755	1.119101745	0.022366432	15.25322992	5.042351414
3.116049514	3.439502311	0.015013011	7.928430267	7.393098695
3.933699308	1.631384066	0.559979426	8.449463187	2.072396187
2.217893521	0.432508475	0.210596067	10.61647733	3.819824163
3.033927072	1.330617521	0.061258038	7.432803038	7.317515997
2.706170309	1.689166142	0.016203828	8.584761767	6.265050994
4.144114398	3.231311398	2.646200027	7.223714459	5.102032145
3.698326587	0.022133713	1.068104332	17.88792365	3.699047614
2.397245962	0.228854505	2.223223912	8.81388284	5.984938175
4.088163682	1.655064404	0.01089356	12.60516299	4.790828979
3.277967949	2.281533576	0	13.29681333	3.41497275
3.546726703	1.264768028	0.049268798	8.115190458	7.36911716
4.849593756	2.024312634	1.159806159	9.383353052	4.936231047
5.142301005	2.781935782	1.980322778	13.53384903	5.926043003
5.095962981	0.094046784	0.057139339	11.10760987	6.508540991
3.628687312	4.815521741	1.392033693	9.097957939	5.993000537
5.611232284	1.00375979	0	7.379149921	4.919644716
3.869858699	2.117700748	0	7.899341103	4.889612133
4.968892328	0	0	9.363569504	4.907128075
3.506901727	3.270537559	0.095144451	11.37129427	8.380055228
1.851808764	3.152651131	1.464732833	10.34045357	5.373142536
2.801094359	4.322917958	0.067076845	10.87361425	4.224080514
4.060244062	2.922832232	1.217279107	11.78624398	4.454826143
2.903978029	1.067160216	1.655153681	9.157750518	6.106260556
3.643555613	1.632076612	1.035110367	17.03126041	3.966581813
4.263669058	3.520427765	0.72663151	8.852692333	3.516358476
2.87911926	0.919424129	0	7.28424452	5.726077138
4.192573811	1.541774318	1.547145606	6.861058415	6.051258894
2.950107915	1.705563928	0.018759478	10.96989608	4.240044573
2.968624461	0.978838867	0	7.866235784	5.425402525
4.438507175	3.313279949	1.320684526	9.953863016	6.702466508
3.61418062	2.401270477	1.274366465	8.003691913	6.145296583
2.37569316	1.038992552	0.007357767	9.396214206	5.27113592
4.472865853	2.850759133	0	8.603804717	4.083235826
4.113719826	3.690215645	1.156044648	9.780560424	4.161266833
3.868180304	0.033074471	1.701013987	12.0983343	4.632154681
4.040546851	6.67560873	1.636949396	13.49441369	3.110493733
3.177800436	1.265874815	1.850573385	8.690668738	6.158224213
3.430021765	3.138685168	2.67145131	9.055284255	5.338583137
4.950138584	1.930281472	0.053875475	10.83061972	3.211620838
4.053648738	3.701358946	0.031070656	8.933393129	5.208987919
3.726339015	7.418897217	2.018966837	9.947977716	6.197137844
4.23513093	2.44222122	2.466164796	8.49016135	5.967084105
3.362374472	0	1.59727593	14.41909159	6.584807969
4.043522468	2.61206735	1.303112621	7.307226827	5.353395595
3.675961145	2.609616139	1.262648064	14.5161904	4.650424768
4.522403097	0.0548325	0.034343607	10.57232889	6.743359339
3.37791422	2.247623353	0	8.999550095	5.468297816
5.287791304	0	1.561996338	9.638886082	3.966349544
2.431431441	1.455041452	0.902600244	8.727133483	8.660943309
4.240854777	6.492572015	0	14.56457888	5.380463226
1.132057909	1.132422722	0.024847768	12.93690223	0.040896731
4.071264107	3.493353346	1.767041549	9.439812032	5.424228238

hsa-miR-130a-3p	hsa-miR-589-3p	hsa-miR-30c-1-3p	hsa-miR-190b-5p	hsa-miR-3200-3p
8.74825634	1.992815388	2.769021128	3.265073573	1.994790606
9.313811299	1.228271533	2.892073876	3.311752309	4.508077641
7.61199173	2.740018122	2.472122835	1.718696385	2.741851307
6.536672536	4.175303848	2.076338357	0.015995572	5.735535472
9.105521545	3.680167225	3.303290596	0.105897386	0.117759491
6.481187504	1.778754548	3.550936348	0.044688087	1.160924936
6.53020763	2.158441131	2.604074348	1.487389797	2.162774039
8.538637017	2.682881715	1.07023995	0.019154188	1.06982517
8.971912535	2.480465692	2.220864504	2.221398486	3.693463943
6.144671143	3.921838075	2.525037066	0.042067098	1.768064107
8.586326723	4.031681281	1.944327527	0.581649679	6.429597555
7.719693208	2.356195771	1.665039541	2.342993527	2.364811058
7.749578747	2.433514974	3.267275547	1.341832061	2.437893943
8.677509003	3.432104464	1.975977804	2.297012437	1.975782902
6.695900322	4.778485521	0.172605519	0.168346449	2.719459131
6.887971268	4.007720846	0.03850238	1.748307118	5.495343385
6.471805229	3.881377023	1.028969064	0.561249831	3.751828609
8.220340202	1.318023975	2.538148211	0.749720772	1.894376142
8.364652136	2.325077189	3.350006866	1.885157939	3.827948663
7.964148812	2.874515607	3.486770277	1.712121647	1.721901161
7.088545561	4.040048064	2.203086547	4.404252933	2.670586438
4.842740275	3.004614333	1.830441781	0.252788056	1.839777282
7.336231384	1.696017123	2.936347177	2.935321012	2.120480026
7.923723395	3.411589743	2.112815791	2.109335361	0.145943917
7.768108687	2.561056094	3.770700254	3.392972308	3.419304998
6.810145326	3.583713116	4.019590629	0	2.518909268
9.026294846	3.553330031	1.972328857	1.972456615	6.566326981
7.374162343	2.254254936	2.583981778	1.197518739	3.609265456
6.873037709	3.546734538	1.904148381	2.338947016	1.906907383
8.758520635	2.76306949	3.352734725	1.128767446	4.194046706
8.363727416	1.788094295	2.469961651	2.330812331	0.941190001
8.780871139	2.80692305	3.169487469	0.999699635	3.999535941
9.163411961	3.656109713	3.122768137	1.000273633	4.999145451
8.269897745	2.443358298	0	1.67357143	4.50431971
8.094401224	2.993813731	2.383052993	1.303156675	4.140529446
6.296560548	5.133642163	2.952188891	0.240750042	0.275728041
7.614664519	2.04869709	3.528093664	2.486713644	3.54627012
5.149229097	2.886352085	3.173901199	1.751367935	5.901346528
7.092988034	1.899786295	3.689566916	1.263951763	2.948216316
7.854668424	2.683699503	2.091878492	1.070735388	2.419000927
7.03369408	3.00754543	3.894444478	1.313791121	8.242586765
4.314864362	1.210940007	2.268430403	1.834257374	4.010090915
7.80993155	2.832100688	3.100102653	0.11211019	2.049854502
8.703136731	3.859181625	2.574743151	0.052964364	5.646692135
7.853127078	4.221682455	1.5059269	1.506150846	6.52483398
7.28423849	3.344689765	4.690477505	3.209793206	3.342919943
7.033944956	5.437613074	2.608528355	4.112347564	5.349817526
6.670587576	3.329347915	4.078424594	1.570650551	3.345514205
7.468072878	1.355559751	3.806913481	3.277558676	2.452594452
8.646480625	1.416566239	0	1.417260669	4.777369922
7.440539627	3.334951316	2.597297992	4.01302805	4.714229854
7.860801559	2.767114182	2.460351183	5.653053954	4.612767949
4.585451963	2.823029136	2.552621718	5.336455405	3.840762652
8.012100951	2.906009351	3.098931016	2.680508647	3.101326802
7.075273884	3.528599401	2.873657153	1.641387317	1.046663244
5.982120437	2.246083729	2.247786308	1.996142554	4.825308242

8.363804035	1.719046872	3.63198928	0.592366375	1.958702279
6.18267155	3.250814035	3.491821364	0.864079359	4.733394748
5.821607023	1.891450622	1.892946081	1.89450677	3.673851626
7.698313995	3.35803417	1.540519319	0.666578726	6.579115274
8.305499368	1.722664312	3.485590134	1.114255082	1.113842023
6.447598025	0	3.827215251	0.833538651	2.558040838
8.779236295	3.070007196	1.186977005	1.563572842	2.315617624
7.548416385	2.627272908	3.365804579	1.030110863	2.039684956
6.384082398	2.286108746	3.661665374	2.548648225	3.660546601
7.754302675	2.603106621	3.883505903	1.210612873	3.300670936
7.236400824	2.853341406	3.44473823	2.249144615	2.254185874
7.828060827	1.567272115	2.786566915	0	2.301760263
6.41483304	3.558953871	3.781445824	2.29832389	4.365204255
7.88080347	3.906136254	1.930716139	2.482531506	2.61493897
7.161159345	2.485251853	2.897841235	0	2.224392716
5.602206864	2.233725996	4.260689399	1.181248863	3.725350466
8.784820803	3.355006361	1.594583492	1.59172778	3.806412062
5.436879367	2.594317877	2.008686285	2.33092958	2.594409282
7.126066771	2.702714639	0.313853744	0.304328191	1.995306798
6.199451904	5.601939133	2.040472266	2.363833693	9.109579519
7.426786348	3.699849035	2.592225676	3.043574957	0.314641479
7.692324794	2.063227735	4.159040551	0.013141291	1.643450488
8.673644081	3.428410581	3.728086099	0.826071586	3.424293615
7.631002121	1.926893302	3.720195455	0.081089245	2.979010933
8.241884083	4.346623821	1.496327391	0.927366061	6.204650333
8.230560714	3.099276747	3.582478895	1.40639502	3.85449019
7.724797034	0.044218582	3.920556396	1.153093687	1.152669868
7.592061147	5.256998782	1.975827956	0	4.058869525
7.69163544	3.381462539	2.613966203	1.423346651	5.369334103
7.397100304	1.848172769	2.612929542	1.845688855	2.889394141
6.749211272	2.362215684	4.038568049	0.199575256	3.185652659
7.670061298	4.211895968	2.117667103	0.131868446	2.577994918
6.600579023	4.045981445	2.809441213	3.794919127	0.680456455
6.518479311	2.415301637	4.165727414	1.648770841	4.153516683
9.353004043	4.690747283	0.503293721	2.35027473	5.769107337
7.371207323	2.726399628	3.468149009	4.733327505	3.472376245
9.318727579	2.759576799	2.759941137	0	2.537628227
8.187888007	3.17714133	2.591846648	0.001328255	4.531236764
6.268909078	4.640104026	1.21932464	0.061155404	4.412734145
6.421712182	3.521375501	3.629434995	1.845900896	2.992857478
6.679439703	2.83106624	2.009910182	0	4.161547814
7.644112406	2.757558284	3.64890229	2.27488796	1.954323453
7.444361802	3.484648487	1.223529414	1.223023293	2.622576618
7.638236567	2.01568382	2.007099544	2.705422648	3.232958693
6.295761096	2.374105254	3.943405248	4.297894702	0
7.336783937	2.684697714	2.876637326	0.916027069	3.042689813
7.515098316	3.109961239	2.603603145	2.962804933	5.058726037
9.74162089	3.818975818	2.307476639	2.31150587	6.298215678
7.683549197	2.438280789	2.927632622	6.335223079	3.713371907
8.05360149	4.547706723	3.192958419	1.015865529	2.830372586
7.754763695	2.447491385	1.092693649	1.69585827	3.134500164
8.971711762	2.853311269	2.284432725	0	2.496504838
7.764569628	3.478283615	2.24390581	0.635598999	3.093020056
8.223040023	3.805370293	2.43709175	3.269987687	3.28888695
7.884248992	2.538407599	1.904126047	4.491553584	4.656764301
8.875228463	2.018256707	3.128652664	2.491246773	2.269986434
7.602021241	3.25944908	2.0837372	2.481994092	3.25253259
7.20046256	3.563273076	3.950306824	3.189061712	4.94345604

7.275707311	3.083732818	1.199234497	3.078613771	5.727885832
8.184459463	3.716532335	1.447278355	1.085047513	5.274348534
5.86895087	4.318910655	3.295686274	2.790325948	2.018819226
6.717287594	3.13382473	3.624379648	0.896258643	4.215298605
5.510914439	2.654347126	3.517442132	2.648983138	3.683093053
8.183129244	2.482832529	3.630612076	0.032757055	3.762327145
6.960003347	2.430350382	5.16292684	0.02181693	2.697399149
6.019819164	2.251744348	3.211838474	0.538820954	6.516927475
7.965371915	2.930472116	5.156516309	0.380211162	3.446322905
6.822598469	3.027187332	3.914386011	2.411574897	1.3295644
5.943565009	3.934733951	4.374825526	1.688214586	2.112859831
8.635020986	1.307867635	2.387017818	1.945461101	2.393620807
7.368685685	2.42476235	1.07572439	4.19769297	5.326822977
8.261518933	3.195065317	0.212769405	1.689819825	0.235520546
7.221937891	3.083302814	3.082541466	1.654472459	1.654967979
6.061286288	1.961536657	4.203887619	2.765914525	3.277921035
8.516228134	3.170546067	3.16674836	1.896516758	2.948130968
8.24567206	4.459186086	2.729936644	2.988615461	3.189881401
8.629660646	2.974449022	2.297958324	1.97712757	2.974117183
6.299638612	4.831164818	2.722722036	1.306621306	5.665132169
8.648745591	2.571243498	3.409808105	1.455289648	2.921771729
7.355113796	2.921425526	1.875265575	1.002594249	5.415449293
6.71151298	3.0835342	2.964574637	3.585042855	3.799803856
8.901124836	1.929796665	2.990138649	0.574350702	2.616302634
7.349637878	3.261614443	2.97845442	4.594189824	4.039979722
8.08224211	0.889369733	1.449469359	1.44981295	1.851855898
5.827429859	2.456747752	2.790263884	5.405026986	2.461535866
6.949871065	1.247120084	3.93153989	3.336286003	4.534754158
7.736940441	1.666227292	2.901994181	2.412224062	2.414178329
7.170956252	1.038871797	2.374657451	0.010548252	3.517795317
6.107250528	4.161779909	1.902422524	4.569348029	6.476724114
7.006083817	2.689400938	3.463076227	1.486554222	0.91978378
5.825201416	3.531599547	3.269618133	0	5.153161645
4.855443927	2.948302535	1.70489668	1.099766178	4.151150015
7.400051751	2.292677092	3.428026369	2.555312295	0.978930817
8.210775292	4.768867635	2.01910021	0.105425175	3.316718038
7.587827755	3.006923788	4.345435864	0.088846652	1.313666304
8.301323175	2.051162062	3.37869436	3.055460318	3.966554167
7.821966608	2.458959181	2.121287896	1.033740516	3.630120203
7.498371422	3.894973553	2.979437499	2.418469962	1.776649048
4.494288594	4.253478051	3.32967267	1.112166336	1.720300522
10.34260596	3.908706162	2.335007208	1.286099552	5.850196444
8.584277885	2.944221449	2.670228537	2.667485294	5.149922491
8.806616389	2.416803947	3.066811633	0.468105959	3.135371241
7.143526991	0.085538584	2.971465013	3.195512376	2.371640167
8.282831759	3.559590455	2.542775626	1.782230517	3.042404916
9.04993925	3.484675696	3.346785417	2.022142123	4.933542838
7.543264005	3.43391812	3.550248494	0.901236267	1.46414237
9.452828009	2.651298737	3.170868577	0	3.048066919
8.404290758	2.213345187	3.189422474	1.273794824	3.589029857
7.120161275	4.149431557	3.582920244	5.118877061	4.656526991
7.515577789	3.849355624	3.8461673	3.717050127	3.26271724
7.23434891	2.729914269	2.248395991	0	3.240886975
9.547829488	3.667906846	2.55461014	0	6.666023862
7.323652109	4.226615863	2.433494391	0	1.455326515
7.166948933	3.956537528	2.842316409	4.311096135	4.292036707
6.014813717	2.171575919	3.649206419	2.169580913	3.191113139
8.53741286	0.803262456	3.019654441	0	1.733503049

hsa-miR-3144-3p	hsa-miR-196b-5p	hsa-miR-30d-5p	hsa-miR-500b-5p	hsa-miR-500a-5p
2.411656318	4.830962831	17.02863687	5.938395435	5.938392897
0.057745738	7.12084587	16.47931279	3.919064654	3.919063897
0.028062269	9.74967128	14.52664781	4.340408318	4.340407853
2.398096495	11.189147	15.54651223	5.677191108	5.677190733
0.095310056	7.145611106	14.8470213	4.235200373	4.235198785
0.040696983	5.805578677	16.47524255	3.697697194	3.697696757
0.126895323	4.277992825	18.09878361	4.0064404	4.006438507
1.069081547	8.388558388	16.40734896	4.967177099	4.967176726
0.927767946	4.406655736	16.09938944	4.583467052	4.583467373
1.761178185	3.390904448	17.75123163	4.933003249	4.933002428
2.662844291	8.974525419	13.99080923	4.924418009	4.924419894
1.638998805	5.99884292	16.07469745	4.375615392	4.375611998
2.743109663	3.654414947	15.94453876	3.951020438	3.951019226
0	8.332083876	15.4691933	5.525382281	5.525382391
0.149745558	9.395280094	14.79714239	6.438130499	6.438125573
0.034438556	7.365759696	16.16552232	5.210685634	5.210684834
0.562709033	7.348076561	12.7653895	4.424208207	4.42420981
1.069476637	4.734518703	14.82306432	3.300141296	3.300142123
2.639818	3.689106732	15.37648685	2.935536024	2.935535844
3.149343975	4.575569136	16.46278364	3.964120379	3.96411746
1.51493523	3.729852508	16.48371701	4.928913694	4.928910435
3.265032912	3.019429561	15.52033407	4.284244739	4.284240443
0.023043058	8.02936526	16.01881151	4.726965544	4.785998581
1.438632412	4.904995665	15.86753419	4.098584756	4.098582922
2.86286393	8.028518678	15.06038271	4.093351882	4.093350072
0	3.656647788	14.5757063	4.064906542	4.064907494
2.778572334	9.522067647	16.74391858	4.824583125	4.824583228
2.242023163	5.920227971	16.9287694	3.283174483	3.283174157
0.068597784	5.902611646	16.04212039	4.203058507	4.203057413
3.495360691	9.723866044	15.92635099	4.58901299	4.589012385
2.496401085	5.099634188	16.24103538	3.48046843	3.480469309
2.584559413	7.706870981	17.73799158	5.169448666	5.169448668
0	1.877173349	16.85549878	4.340043343	4.340044945
0.760557062	4.948768024	17.08017445	4.183373494	4.183374316
0.077548369	5.984046899	16.33744018	6.040109373	6.040107125
5.107570509	3.859352096	14.93459011	5.859548257	5.859541509
0.101125197	10.13563085	14.27698049	4.264970498	4.264968776
2.900530758	10.04173298	17.48511433	5.756445972	5.756447087
1.258445012	11.28595194	17.94310784	5.203667962	5.203666354
0.017680022	7.764296224	17.18269721	4.810291367	4.810291011
1.941612591	7.931920614	17.15006096	4.040100407	4.040099235
0.053282332	3.894595354	16.78656039	4.011358417	4.011357674
0.100782612	4.37383713	17.23043537	3.877048225	3.877046891
2.562890303	8.945771762	15.35584528	5.096840711	5.096839615
0	4.802458753	15.66786567	5.895026537	5.895026952
0	9.947190178	14.45194328	3.342670808	3.20602936
4.199606145	5.175260462	14.26912926	4.955071062	4.955068062
4.022727505	1.561832072	16.887461	4.115977561	4.11597511
3.621539909	7.571231143	14.50323553	2.792795531	2.792795418
0	4.362361321	16.62777901	5.023560055	5.023560749
0.00218841	10.05461399	15.97692399	5.101395479	5.101395431
1.157958437	6.536878979	16.86551779	5.48314788	5.483149743
2.541966681	7.471366796	15.24483719	4.584143618	4.584142794
2.086942689	5.318906039	16.25245063	5.318058282	5.318057872
1.046218141	12.78515257	16.92267921	4.773619789	4.773619558
0	7.784105496	16.44486722	4.261678592	4.261679414

2.188436525	5.222125749	14.9802001	4.324528121	4.32452955
0	2.795568384	16.98924875	4.82230422	4.822304864
0.704232573	8.881961854	16.09028328	4.948801836	4.948803237
2.498304813	2.917783872	15.5282561	5.120543244	5.120544884
4.800711373	4.422951054	16.69506372	4.261238263	4.261237807
2.095405509	10.53316429	16.05505499	4.534296289	4.534296984
0.683478548	4.312184675	16.05723158	4.540562549	4.540563821
0.007494994	8.934396401	16.47826868	3.746378176	3.746378094
1.554228078	8.034381718	15.68602348	3.660431212	3.660431276
0.053386801	4.77865797	17.86593269	5.17731474	5.177313489
1.810848395	2.855264874	15.78428143	4.755629858	4.755628851
0	6.515568856	14.01924591	4.147213926	4.147213978
2.976105432	3.295831505	16.58780178	5.398625735	5.398625836
1.412873636	3.507106123	16.13440627	5.341098246	5.341100415
2.228984648	5.768589348	14.45723298	4.64318518	4.643185494
0.045809436	3.969909936	16.05244527	4.919300444	4.91929946
0.155559322	5.377073863	14.95979563	4.926337445	4.926333733
2.5938487	4.18026103	14.94545987	3.5950769	3.595076885
0.264102828	5.431611239	15.00632471	4.086282128	4.086277345
0.007716823	5.749951509	15.90611704	5.955993321	5.955993113
1.845646559	6.152373577	15.39327504	4.786914755	4.786908877
2.872331562	7.487005844	16.12905739	4.660144553	4.660144325
4.495101844	3.720219273	16.62975092	4.842023109	4.842023929
2.948219566	5.132636727	15.76839719	4.120138659	4.120137541
2.225083761	9.960533193	15.26868033	5.437070528	5.43707095
1.409656974	6.563202886	15.91002968	3.766985937	3.766986316
3.791154732	4.231205411	15.91872721	3.220878673	3.22087845
1.976626753	8.139886995	16.340036	5.100011612	5.100011711
0.869267954	11.21847366	16.51570014	5.242888313	5.242889028
2.866977839	6.317379694	16.34518073	3.781421739	3.781421083
0.176501287	3.906584099	16.12615517	3.469497177	3.469495472
0.118102146	4.352633139	17.62901267	4.827224447	4.827221822
2.32946552	8.520798299	14.38771303	4.613714916	4.613716246
1.962255759	9.748504893	14.28953943	4.518136328	4.518137371
0	4.260556959	18.85355181	6.13241271	6.132415625
0.025531835	11.35284631	14.98632684	3.961286811	3.961286476
0	4.269803506	14.63714507	3.271830882	3.271830931
0.001219965	6.400252947	16.14695813	4.255590997	4.255590978
0.055515886	9.610463064	17.28639183	5.54196441	5.541962979
3.532164443	7.645298836	15.95381296	5.202723828	5.202724453
2.019292398	2.977543422	16.55656529	4.633394556	4.633395498
4.594631886	4.851797905	15.18385095	4.7526278	4.700206365
1.218650126	4.861419639	16.7165403	2.288393266	2.288393512
0.272047727	5.3763776	17.29446559	4.932804205	4.932796874
2.124908958	4.287013712	15.76382949	4.156982537	4.156983039
1.892023467	4.565162172	15.23755107	3.193319448	3.193319554
0	7.106929847	16.80759498	4.727371202	4.727371836
0	10.3350839	16.76596261	4.551896529	4.551898709
1.084347165	8.960358463	16.77190938	5.220807111	5.220806617
0.003943389	3.483387985	16.95547565	3.931340867	3.931340818
0.023184782	4.390248449	16.54980529	4.66626027	4.666259826
0	4.053802334	14.64880031	4.120626419	4.120627102
0.637305098	6.538609517	16.41371126	5.140812983	5.140814769
1.339439475	4.907708488	14.96946955	5.214697442	5.214695257
0	10.63747299	16.48344411	5.557131362	5.557134592
0	3.740139283	16.18825021	2.986496896	2.986497047
2.311384159	8.923723029	16.5054244	4.468203873	4.468205158
2.682895088	8.626060493	15.03047958	4.2493574	4.249357732

0.05033657	8.951325557	17.17775236	4.621446203	4.621445234
1.457211296	3.324123626	16.7149649	4.881471178	4.88147295
3.634007729	11.31804671	16.17085655	3.510947472	3.5109466
0	4.487885202	16.99537658	2.755093166	2.755093221
2.903845598	9.159619569	14.37208565	5.700442913	5.700441202
0.029901037	6.187395214	16.61675107	4.180477868	4.180477414
2.425048204	8.595259179	15.04914504	3.705025361	3.705025149
0	7.892315333	16.1824973	4.806920287	4.806922255
4.480276964	2.951834873	14.85311398	3.814399567	3.814394354
2.394988904	3.263185224	16.67850077	3.035226592	3.035226256
4.118638687	3.587952597	16.07402923	4.524930955	4.524930567
0.078403975	5.268139351	16.98619695	4.029853596	4.029852463
0.018909646	9.221833334	15.41665726	4.022114974	4.022114716
1.665687182	4.665714104	15.17592544	4.769066646	4.769062405
2.885004523	3.67221681	16.28087637	4.613330598	4.61333033
3.281175191	5.537962579	15.98851626	4.202231676	4.202231783
0.067244097	6.865555665	14.9282263	3.374936679	3.374936167
2.255094249	4.062475736	16.19887269	5.025166354	5.025168094
0	5.141903034	16.31361373	5.926189842	5.926189957
0.078461147	9.31662623	15.60421195	5.919184626	5.919182398
1.443001224	7.726728248	15.56578549	4.560847863	4.5608455
3.156752592	8.650553141	17.35787479	5.373506633	5.373508965
0.691173276	8.906997691	15.29118466	4.468293469	4.468294673
3.017547526	6.996551259	15.8130621	5.068691802	5.068693807
0.1345449	12.32652584	15.67767785	4.987649258	4.987646048
0	3.91136394	15.23732815	3.82441014	3.824410446
1.992856722	10.46283722	16.41100983	3.680984117	3.680983086
0.062614079	8.932686825	16.51187482	4.365251556	4.365250472
2.408997408	3.686179261	17.32795604	3.42204408	3.42204395
0.009670651	5.734307293	16.8739261	3.96644768	3.966447554
2.567156042	11.30375796	12.94731773	3.969139652	3.969140503
0	2.689034765	18.44222978	4.869784431	4.869784826
0	5.434660101	16.21594885	4.528879825	4.528879979
1.098172812	5.990289563	16.24997359	4.73873354	4.738733047
1.559860922	6.232382396	16.8797682	4.288623686	4.288623768
0.094893584	4.866005476	15.85997364	5.244031924	5.244029571
2.979409356	7.629384857	15.47022609	3.91176773	3.911766661
2.049101755	6.830891908	17.3082098	3.518270308	3.518270224
3.929017653	6.551083259	16.23302697	4.916994267	4.916996214
0	4.704412302	15.9007588	4.396699922	4.396701287
1.715551375	6.806848959	15.60266262	3.869059439	3.869059094
3.245586862	8.438407582	13.03529317	3.616433497	3.616434516
0.067530906	4.089600041	14.72357849	4.197303125	4.197302052
0	4.591311273	14.99834039	4.185487764	4.185489428
0.073793242	3.582008934	15.58735119	4.5141689	4.514167514
3.219066548	8.796095882	15.63451335	4.337209925	4.337209224
3.023273657	5.114493715	17.20236669	4.113817663	4.113817602
0	4.233962598	17.038761	5.125009988	5.125010511
0	5.072979046	15.15710599	4.026658493	4.026659477
0	3.294970473	15.60334956	4.386304541	4.386305493
3.623536167	10.69264347	14.27414283	5.014658669	5.01466115
3.56570231	9.521351862	15.28986139	4.274809977	4.274809223
2.25111003	6.000424262	16.71020494	4.003642538	4.00364271
3.87635726	10.17294305	15.79802096	5.872335	5.872335139
2.439350303	3.009397846	16.40328438	3.832088831	3.832089126
1.592441961	11.86888655	16.53284573	4.525076651	4.525077862
1.13009095	7.457021572	15.55817639	3.516999676	3.516999382
2.052127317	8.582603484	15.0733779	4.260872823	4.260873534

hsa-miR-3677-3p	hsa-miR-671-5p	hsa-miR-4652-5p	hsa-miR-4661-5p	hsa-miR-421
4.404685586	4.302804964	0.10800569	4.199742986	3.949410941
3.49651449	3.49602073	0.069713886	2.293726546	3.789448073
3.975209496	3.751281389	0.033441298	4.631350423	4.562024887
4.412080657	4.675339745	4.549738722	3.256796254	4.337701975
1.371469403	3.985547054	0.116990301	4.344954499	2.025893997
5.705483902	3.03594503	0.04876592	4.947337424	2.540696501
3.238975214	2.96098819	0.157974716	5.998249855	2.616783447
3.806185128	3.806013501	0.020795787	3.102513078	3.690039254
1.495706315	3.885690195	0	2.890663732	3.792966288
5.691808425	5.723632405	5.138029113	6.712037551	5.088462608
4.21712545	5.384211794	0	3.280267296	4.61765088
4.069931378	3.456799762	0.222663616	5.020865045	1.670111322
1.992664447	3.652076009	0.107376236	5.415871787	3.050048833
3.431676642	4.141536539	0	4.725886097	6.629231071
5.668760195	5.391260548	0.188351984	3.960583683	3.95162184
3.787995958	2.508096971	0.041154014	3.905610017	4.205092289
4.307212905	4.969687529	0	4.988965584	3.556459648
3.418107513	3.473688379	1.537381564	1.896551818	3.474077055
3.533333885	3.532764918	0.078320536	3.957264411	2.32758324
2.889666587	3.238731735	5.448334891	4.997831997	4.557356162
3.726699085	3.725249588	5.278245897	6.019244485	5.211407283
3.660157383	1.840233547	0.287775688	6.245263353	3.022461864
4.045915785	3.133446752	0.027400095	5.698585446	4.66404411
2.120059753	4.548005621	0.144940013	3.426914718	2.571924253
4.964223559	3.417585068	0.143563316	3.953565203	3.417222294
4.478174378	4.607398918	0	4.794042613	3.658569257
4.873692531	5.096079302	0	6.05360407	6.233415738
4.381645666	2.588679635	0.06016105	4.943924605	2.255994924
2.346655503	3.847576237	0.083211644	2.955665874	1.907039283
3.776250792	4.442371813	0.038652487	1.127499783	2.167998449
3.414501982	3.784018785	3.414842403	5.102433125	4.284375926
3.169477272	2.80691856	0	4.391846056	4.458964476
4.130908161	3.827018187	0	3.577247533	1.632807012
3.514729986	3.925198032	0	6.188637853	4.457191738
3.600546957	4.441765571	0.094441564	4.535294875	3.894207529
3.617031173	4.38528388	7.37884536	7.560312879	4.22709317
3.34688568	4.959355616	0.124449919	0.127971422	2.84025491
1.749412342	2.528910566	0	4.395022992	4.221927177
3.373629238	3.546345608	0.081541803	6.072953589	4.292857494
4.568118437	4.863812315	0.020974085	6.220084434	5.35728281
5.464002078	3.242828551	0.097977326	6.495314959	4.261420649
5.223191633	3.103125461	0.064199452	5.813472468	5.563922138
3.873912551	4.366580332	0.124009388	4.740819315	4.257680938
1.813727714	5.041188943	0.057893197	3.273359114	3.598251131
3.360106361	3.706742344	0	5.321446867	4.146107313
5.007898491	3.055443865	5.760725987	3.205977336	2.69583274
5.83616739	4.500599516	5.659353277	4.794469191	4.499963329
3.06579201	3.576305768	1.576189369	6.252401127	3.951540626
3.964233404	2.006523555	5.160996438	2.45232878	3.065451201
4.906653164	5.025489233	5.099062254	6.98214374	4.907270301
4.768665796	2.820136067	0.002578789	6.714549743	3.920393618
3.736455339	4.216073729	0.657974805	3.418817742	3.737436922
4.507997159	3.248188735	4.962105306	6.376338475	6.285547071
4.012325366	3.56304625	0.020593133	4.631375023	3.689043117
2.875021089	3.529670194	0.01383606	3.771850238	3.068512113
3.71238205	4.264139881	4.144401797	7.0007436	3.436582875

3.212496864	3.617697194	1.718536495	3.683008132	3.213313821
3.112050819	1.418018924	0	4.172589424	2.962200283
4.814983234	4.664781962	5.847505824	4.139314076	3.59396013
5.669580645	3.828297997	0	5.2257133	6.1503813
4.260220964	1.722909844	5.46090758	5.906184988	3.870905472
4.682071013	3.641532991	0	4.584194653	4.123496817
3.855571284	3.922495002	0	3.919088202	3.379332486
2.363458512	3.044131041	0.008852272	3.214712342	2.627653834
6.466444222	3.660646252	0	5.450240235	2.769512294
4.959807896	1.837847656	0.064328275	4.403126133	1.837858944
3.989434398	4.197033056	0.059680789	3.874719218	2.586616203
3.678166894	3.562820601	0	2.786148147	3.884509756
5.101921442	3.5585608	4.365264783	5.949457626	4.673297183
2.746068823	4.492303627	0	4.000893831	3.649935115
4.348970298	3.215787662	0	5.279423981	3.891002568
4.91722336	3.259286482	0.055014919	3.853523206	3.259164968
3.805124159	4.999545024	0.196180561	4.928948354	3.602434499
5.139703456	4.01020098	5.534003618	4.917382013	4.097680829
3.5549401	2.721174373	0.350180578	4.774041792	3.196185509
4.295747289	3.954212692	0.009115147	3.216026862	3.631745855
4.645483116	3.727858366	0.311822947	5.469833926	3.090797479
0.014276418	3.980351837	3.980426673	2.653346664	3.39374176
2.734997707	3.530591919	6.356154823	7.030312677	4.177612786
4.00061415	3.872476169	1.287604659	2.70658285	2.705729045
4.58484537	4.691809319	4.528459724	6.553905815	5.991956844
2.372355017	3.09673853	0	4.569203347	3.577202827
3.811436312	3.21994794	2.199303609	3.812824892	2.798241952
3.779037906	4.36361415	0	4.82874949	3.67228604
3.254241709	4.311479645	0	2.800269417	4.311612778
3.313389205	2.618320656	0.067339712	3.781845062	3.116390676
1.675245267	3.463424963	0.224721947	2.370152596	4.073665758
2.125041377	4.098657693	0.14644911	5.42010437	3.427803659
3.847143654	3.778492593	2.65491338	4.253880596	2.934929068
5.444881371	3.045743407	0	4.952726065	3.744246083
4.379526336	4.008059896	0.507811919	5.28860157	5.043831555
4.679222186	4.060213827	0.030392334	2.953362012	2.952853331
2.951009697	3.120444549	0	3.649382719	2.275998274
3.329306899	4.592617606	0.001436987	4.007622122	3.592416098
2.616942033	4.572967224	0.066956263	5.332816146	2.887196441
4.513701107	4.457640714	7.810749635	5.537305062	3.279832828
5.490507119	5.157687108	3.11126464	4.879329442	5.219186906
2.27404469	4.116289035	0	4.530341752	3.532345594
2.893220124	3.91048438	3.317341122	5.426725926	4.50019367
3.871640394	4.783673389	0.362066118	5.617452138	3.583293752
5.462275748	2.947116323	3.471311587	0.856694696	2.947184139
4.037381287	2.683589332	0	5.929024467	4.910156479
2.957281815	4.025769532	0	2.382606574	4.300200502
4.138815752	4.61305302	0	3.430114372	4.282613513
3.448741623	4.938971319	3.448671347	5.040280426	1.688602498
5.417249609	3.193479989	0.004650355	5.112573887	2.607505258
2.71511954	3.458563852	0.027570315	5.899942364	3.45849515
4.41568409	3.575363855	2.49662324	4.465484872	2.99934902
4.791516449	3.385536713	0	5.603781909	4.073953714
1.99944128	5.208732908	0.109324977	5.099922936	3.287460278
3.11567315	4.011841084	0	2.892057618	3.046328494
3.121979383	3.464831886	0	2.270359032	2.838637407
4.01645184	4.017247876	6.549036311	4.777700011	3.827491658
4.664091707	2.455595929	4.943817368	4.027591741	3.859493569

3.876827876	4.382869867	0.06057181	7.047804688	4.544045252
4.009941702	3.233889278	0	3.770014442	2.801803929
4.526920324	2.465310739	7.430822427	7.876555998	4.613539861
4.881256952	3.872638223	0	3.659022644	4.328863021
4.692984272	3.948650109	6.441702622	4.899658323	4.538792334
2.48428941	4.428027083	0.035661072	5.829321708	2.751587516
2.104609846	2.921768403	0.023699307	2.697453562	2.921729472
6.161270228	3.012700354	0	6.142473261	6.476069935
0.446171773	0.442262374	5.883386248	3.817810946	4.081249467
4.384537011	1.977674622	0.103078571	3.638440536	3.033325582
1.68908397	4.21813405	7.12241204	4.525198592	3.296174057
3.604963785	4.352221711	0.095519694	3.902905361	3.603971913
6.117639739	4.63950992	0.022444785	5.733516755	3.433837123
0.234286461	5.660948599	0.233640677	3.499518787	3.491277274
5.482366996	3.994705229	4.67415974	3.255550077	4.087981787
4.124554925	2.956913343	6.461439337	6.721182598	3.861949014
2.675936503	2.675674442	0.081520907	3.971109641	2.675571778
2.853517905	4.172019506	6.19884385	6.989229648	4.913238834
3.673624093	3.879928737	0	6.238655426	3.43292914
3.233837198	6.008630468	0.095591763	3.607759563	4.617611832
3.429568925	3.958494339	0.146550443	5.054538171	4.226488421
3.515038247	3.113383949	0	3.768582901	4.980466538
5.167267191	3.078417649	0	5.610288501	2.512742627
2.472542234	4.455719007	0	3.948938824	4.494216647
3.274678119	3.705719709	0.168075105	4.989910951	3.505478077
3.412780831	3.633858532	0.890046238	2.424511383	2.833682248
6.721712947	4.426905279	0.113803368	6.446364973	3.304896683
4.757948511	4.362067797	0.075753236	2.924974146	5.366921334
2.414158997	3.097864884	2.414145375	3.686423382	2.414107141
4.704071743	3.86634016	0.011432681	6.631589487	4.22945134
6.909519974	4.507439971	0	7.425134599	4.316567499
2.688256441	2.879193681	0.919663197	4.514147649	5.04069117
2.947476785	3.530809501	0	4.336362531	3.116895006
3.733928829	3.469026865	0.029763968	1.098720203	3.849684153
2.77642335	3.289970954	0	6.155700948	2.292494083
2.024083028	5.180726859	0.116457525	5.067435491	1.370286433
3.44054438	4.261113605	4.919570692	4.461128306	3.012667432
2.640271865	2.863692654	0.011495768	3.644192992	3.759500698
4.714173312	3.49057532	0	3.487290738	3.249722888
4.113521978	3.757237983	4.215726771	5.451640004	4.166585658
5.873405934	2.146593546	8.499573615	6.848152659	4.926014988
3.993859199	4.253734295	6.114726079	4.324367607	4.778685659
3.548362631	3.177435526	0.081878959	6.016599231	3.547543068
2.540097824	2.86883335	0	5.025118783	2.767562636
4.747464625	6.014127801	0.089719401	4.822763134	3.209350374
2.546676133	3.702471531	4.709170791	2.546720196	3.563426713
4.11368048	3.93293311	4.485840281	4.274438489	3.024979758
2.441957757	3.307186874	0	6.085993916	3.431912034
3.62010119	2.300821506	1.176530043	3.537101629	2.090603175
3.181401344	3.676177856	0	3.398330952	3.676367212
5.636702504	3.442762838	6.954147879	4.595690741	5.221541108
3.588869893	3.434531138	5.978773465	3.436129937	2.839295674
3.37789321	3.824038156	0	3.617669129	2.920698424
2.968083218	3.552066364	0	3.289274325	4.358668039
4.474118887	3.641448104	6.017963104	5.709031341	3.009046096
4.612000885	4.768051238	8.593707085	6.329803437	4.073545183
5.72660689	2.172598531	6.412748762	4.10681701	4.724031471
3.590264066	4.138290834	2.510825215	4.627279953	5.318023397

hsa-miR-217-5p	hsa-miR-301a-3p	hsa-miR-18a-5p	hsa-miR-454-3p	hsa-miR-19a-3p
9.989121265	4.828951706	6.450557819	5.418499814	7.999652196
11.3826258	5.772848446	8.992996912	5.932082725	11.10231228
7.471971595	4.816303409	5.667160656	5.302938269	8.143384481
8.001118542	6.485154911	7.528523923	6.070002227	7.964557011
11.77727002	4.791130264	5.134525506	5.455699487	7.302350572
4.414111321	2.209581077	4.149899931	4.331276783	4.770551478
9.260845115	3.241816791	4.608671818	4.789363117	7.440559103
11.59953636	4.195175135	5.016901538	4.50086879	5.754963081
7.565828683	5.011340996	6.810549396	5.92547497	9.286355767
6.144468639	6.323347418	9.004072743	4.229654886	9.308776244
11.97032452	5.270720232	6.182873468	5.494821285	7.455473255
9.583925818	3.46367755	5.110636659	3.699161045	5.758963575
8.918956324	4.079599397	4.828761346	3.481374967	6.668448949
14.0094124	6.671001698	10.26712911	5.462583729	10.05093646
6.191359725	6.110452693	7.883992798	6.318355835	9.257587862
11.70889042	5.421109353	8.458993758	5.255756719	9.515820556
6.517744569	5.271832577	5.203133946	4.908738435	5.659011124
11.93982846	3.469265091	4.070172477	3.469194615	5.593300818
13.11263486	3.361692426	4.968694126	3.69007581	7.346571769
16.52926299	3.763334879	6.191972017	3.763532761	6.887354676
7.415364881	3.907869625	6.303318201	3.908030597	7.693627307
8.115620153	4.835963371	4.593328917	5.675734718	6.933538452
6.1678306	4.046836069	4.84359951	4.727279548	7.530226629
14.27932793	4.345909243	5.306426154	5.518105715	8.496924999
15.01006615	4.449542172	5.947938907	4.449741388	9.226325555
11.94571601	3.805084266	3.301000569	4.682406689	4.38189802
5.010756943	5.77356355	8.353712013	4.667057564	8.420930551
13.10661863	4.69095489	5.589674537	4.29598144	6.306538151
3.849308202	5.047588211	4.399783721	4.862096541	7.221652767
5.522633421	5.447440987	6.542336711	5.814900331	9.083649638
9.521062981	4.966379064	7.462038736	4.587141895	7.696958799
14.49286518	5.523082948	5.584480406	5.780879609	8.404652883
7.665692428	4.62480269	4.125728843	4.488836165	7.077945895
9.221095455	4.91465994	7.417512646	4.722982721	7.70774128
11.08336797	5.036977493	5.502099369	5.454609638	7.740996363
16.65223489	3.624614256	5.359203269	5.565443345	8.308055101
6.421542193	4.374725699	4.824108564	5.03517775	7.840765956
13.97381825	4.501370779	8.715950633	4.551645344	8.536295535
7.883393283	5.097159609	3.971697356	5.82615653	6.237460273
10.34001099	4.356993475	6.220878919	5.240037503	7.398514946
6.35960791	6.495058778	6.030100316	6.581611288	8.345088951
5.815530344	3.104375747	4.120457683	3.628452019	5.968414645
7.839100625	5.622339289	4.96852575	5.827154469	6.230281195
10.6964835	6.074053909	7.307883212	6.175626738	9.358957468
11.37252614	6.023981779	8.037972481	6.003191878	8.25497275
10.02023629	5.920984047	7.421917088	5.269469572	7.757321753
14.40146697	5.170889406	7.6606889	5.032113252	8.622202645
10.01542179	4.984383924	5.283533901	5.210199275	8.109425686
16.80373293	3.827475306	5.483462066	3.96802524	8.777239369
7.786909748	3.245747357	7.683553671	4.100925518	7.819976387
7.164011194	4.768783064	5.336001364	4.26168112	7.262198552
10.92583316	6.414309095	8.08987148	5.852446129	9.464941497
11.13319627	4.722865442	3.575934056	4.965044057	5.311696778
9.810267485	3.425469438	5.467207065	4.4292817	6.956438294
8.424282548	4.596646357	5.829543328	5.073668504	7.908346219
6.334351634	5.135634058	5.282168346	4.658759566	6.639015124

14.75311759	4.4476123	5.724052206	3.929395691	5.921410073
4.422963085	6.461282639	4.238943037	6.705281741	7.832569093
7.048456316	4.620091128	7.343147604	5.042425897	7.336065215
5.642930939	5.200019596	7.171323292	4.743459515	6.685923006
16.77994569	4.173616569	4.984778347	3.75610681	7.087808803
13.36975221	1.776243002	5.045229303	2.558070715	6.938213222
11.80115832	3.634512078	4.906975045	5.033491102	6.055791276
13.19885738	4.134054499	5.217714126	3.504936486	6.842884729
16.03053506	4.602719894	6.465643707	5.167959241	5.935229838
10.19196301	3.895557138	6.834298759	3.768478205	7.604145016
10.54215222	3.991399236	5.158266004	5.105920771	7.681175832
6.499988939	3.678056742	5.18616023	4.620861582	5.262082768
13.32009417	5.672676811	5.699981369	4.972423256	7.302696815
14.40354521	4.594985902	6.10647302	4.241944094	8.064148564
13.80477058	4.137275907	5.056847512	4.059494094	6.500041418
5.408417614	3.587326553	4.272954636	4.44346652	6.675111661
7.728824406	5.012373284	5.377007089	6.439981989	7.728636005
9.632843024	4.01028165	4.964737494	3.710613434	6.119209728
11.50537867	3.85076398	4.092820448	3.205866849	5.672646292
8.126898245	7.869620072	7.831279197	7.441771026	8.754003801
8.461837159	3.098163296	4.667537616	4.791027	8.905751055
6.26694356	3.65811289	5.398436823	4.775934302	7.325490521
5.685672805	4.521080226	6.814953101	5.754031246	8.377385687
16.96474103	3.736008545	5.075766365	4.120888311	7.429742927
11.40167385	6.373451137	7.993966406	5.525950409	7.202089269
9.433795267	3.095978348	4.968943715	3.67442545	7.427531985
15.76796272	3.547063964	3.929562473	4.137371819	4.758253734
8.362265013	4.55587803	6.16004306	5.015084563	8.10439885
7.715286779	3.598145093	7.027375905	3.598123611	7.968405016
13.06030185	4.234458214	6.799906873	4.025905419	8.927782973
13.85140412	1.671646162	5.412431646	3.188614641	6.049630843
7.588409641	5.121692747	5.477497663	6.128682259	8.430879318
15.72529256	5.054518658	5.389740396	3.973794699	6.4083801
9.654449708	4.26590678	4.916668684	4.691256988	5.172307671
5.32429413	6.822018877	6.747812322	6.710300682	7.894587702
6.137928425	4.242923945	4.74277502	4.548965199	7.044256935
8.954239632	4.118045665	4.75455923	4.035698257	7.155469868
9.057843323	5.095255649	5.329782979	4.762645442	6.030282616
12.03011591	4.974974627	6.404129891	5.377371913	8.485534951
16.40346725	5.938249791	6.588436255	4.98038189	9.580546937
13.79972514	4.587304154	5.534081924	5.357418557	5.775955891
8.052121097	4.193740153	5.921439547	4.530341964	9.052081419
9.917768825	4.504092593	6.494170919	4.921981643	7.073897248
7.265420191	4.807351574	6.251722657	5.764244767	7.996693165
15.21361961	4.156688674	5.116896886	5.045927105	7.139378492
6.827095978	4.450260514	6.219404555	3.774674544	7.475679528
12.25050624	4.528454379	6.495910429	4.859745145	7.42511287
7.529587906	6.307454903	7.849327564	5.773198328	7.698847343
11.11807496	4.717634981	5.178665213	4.453929842	7.80915639
7.625597788	5.85815819	7.244773909	5.858168449	9.694623445
9.976095355	4.311776219	6.355916827	4.843893075	7.297047098
15.09600247	5.382014771	7.631654517	5.298096922	8.633334408
11.40167108	4.533179799	7.567108099	3.766570424	7.521781749
10.69004894	4.759680864	5.844858428	6.134787927	7.228835865
6.160257492	4.527785812	5.193111166	4.24352271	6.840609321
8.31733294	4.400774914	5.746779766	5.034637939	8.200592985
10.2038824	4.467439366	5.436088861	4.942509825	6.528007316
14.78456081	4.610642188	8.070937987	3.945486272	9.771585008

5.515077652	4.88653702	7.538322852	4.622117075	7.548662015
7.664553857	5.733445592	7.13455873	4.994800197	8.132183802
12.87836903	4.531918057	5.450752609	5.294315022	7.280176166
7.10399384	5.767046368	4.578590123	4.945554509	5.376119685
11.26607827	3.684718747	6.811466709	4.460179399	9.042550657
10.5447216	2.977637065	5.355641426	4.574873037	6.846348889
4.370654376	3.116412851	3.705186901	3.928592585	4.768082831
10.022034	4.888988448	8.369529424	4.522501256	8.014523838
7.834129355	4.550403013	6.143106175	5.282380196	9.925274841
6.082607441	4.947111341	4.735859235	4.179434779	7.631607837
17.82811789	3.125558193	6.982329806	4.131561058	8.246053976
9.344543816	3.235206346	4.703756918	4.539870221	7.399885953
8.465406635	5.072186598	6.207438853	4.576497879	6.675047543
13.91317796	2.865122867	5.570464611	4.662506209	7.717527173
13.75706282	3.084679813	7.272410779	4.549269845	8.612562191
12.70775364	2.956787519	5.275082462	3.762223603	6.491785109
14.92156565	4.56274287	6.31104033	4.981046928	7.674039948
14.2289938	4.217618049	5.47544942	4.265773972	7.558440342
11.54075314	5.257471009	6.161514976	5.059586311	8.475268901
16.59140882	6.416517531	5.268500317	6.076512356	7.038680233
10.59351045	4.352067628	5.883264486	5.365604364	7.769660742
8.429531173	5.352570351	7.899309514	4.175340698	8.316600612
8.711364956	5.438634092	7.996461093	4.890345831	9.287793353
9.055153866	4.908286372	8.823154226	4.963136326	10.27664527
9.684744912	3.511226584	4.914658686	5.203999145	8.494960963
11.75224416	3.824236371	4.726641243	4.465030122	6.81600268
11.95584272	3.837275172	5.237876693	4.527429247	5.446211762
8.996837868	4.063011895	7.950080659	2.924974237	9.387506513
15.68830533	4.351598576	5.235082094	5.720459146	8.2318302
8.586221083	3.517997477	5.015496691	3.643872717	6.419781053
9.298947945	5.356142083	5.582262448	3.96857892	6.650951376
5.798340992	3.959568443	5.225889166	4.82379235	7.197340385
5.300528671	4.114300629	4.467298079	3.64569865	7.393286491
3.469018442	4.613135858	4.678033753	5.012769017	6.802099799
5.638236059	4.358908762	4.054269752	4.425964743	5.392133411
7.570414638	5.634825385	5.910562553	6.072975961	8.647754789
15.33275828	3.61686129	4.634660197	5.110856202	6.203996257
8.999670741	5.553556936	4.646167686	5.616347615	7.927090005
7.071478356	5.58602526	7.132729205	5.212893795	7.255879036
11.88900275	4.306784414	7.217433778	4.438341217	7.632367015
13.28678062	5.634104653	6.198991569	5.698393348	6.919030679
10.87311987	5.422509861	8.610820305	3.841090805	8.74390354
6.866952002	4.642987792	5.989754525	4.089745054	8.023893939
8.313264601	4.469138401	5.422090645	4.562741803	7.930790636
9.765261188	4.332417985	7.128699231	4.6771427	9.014708795
14.81994236	5.160914679	6.902966187	5.577173319	9.200368956
10.63681151	5.699483707	7.104253768	6.349089911	8.570603736
7.450611672	4.698211676	5.744354288	5.124641853	7.610627369
14.17296909	4.604479141	6.445327288	4.718861589	7.51834452
10.64820988	4.041137449	5.657147104	4.04107148	6.059959673
13.55464474	6.323299302	5.523633828	6.206401552	6.568606761
15.52942809	5.234465604	5.984645053	4.181465785	8.591180081
8.616034733	4.003543167	5.305916645	5.043546135	6.95687952
8.722948896	6.155133007	8.794566823	5.693304909	10.23385208
16.88822529	3.918900424	5.440594348	4.956919674	6.868428875
9.131732957	4.48035653	4.724646154	5.539918779	6.627283842
15.41654566	4.726456562	5.205450116	4.788142955	6.793493518
14.09234527	5.130381289	7.25014267	4.841938106	6.560264213

hsa-miR-1251-5p	hsa-miR-3127-5p	hsa-miR-500b-3p	hsa-miR-362-3p	hsa-miR-135a-5p
3.276623624	3.808672906	3.27675559	4.304539783	3.813650836
3.645836098	4.140552622	2.292450452	4.034383909	4.144301042
0.0323078	2.474376681	1.719869013	3.867869781	1.110821336
4.410499212	4.790735798	5.259295871	5.135609561	5.221267157
0.112309415	3.688956273	2.024317159	2.47313037	3.089437552
6.349270717	2.540577567	3.229004335	4.888429617	8.326914904
0.151184659	1.491346162	2.61166017	5.344284015	5.663816716
2.68311864	3.425721225	4.10500922	3.806277342	3.273546283
2.480261511	3.472488609	3.69506045	3.693497708	3.972076724
0.044284528	3.682600291	3.806566916	3.544719677	0.046839
2.633914067	3.096818889	4.399726112	4.468756672	2.991262183
3.164918788	2.363583514	3.443385291	5.093840168	4.077418523
1.343746981	1.9926231	2.434631369	4.076375467	3.811100045
0	4.495033343	4.059511876	4.878042944	1.562800732
3.33253538	5.123128825	3.763798079	3.779131082	4.505437278
5.526883139	4.730350662	3.784945414	5.066980711	7.190354078
0	4.541622444	3.248740507	3.151911767	0
0	2.806672951	2.630806371	4.003594785	1.540370268
1.255119134	3.826006618	3.158803157	3.827808002	1.888428819
5.718482257	3.952418503	3.737246108	4.292391436	6.655824151
0.16593589	3.013905938	4.98269318	5.148087901	4.205563947
0.272731758	3.654918005	1.837549209	3.897206332	0.29708427
5.308936487	3.304502143	1.092155023	4.896437242	8.121894717
1.454118538	3.190152033	2.567445164	4.340855589	4.901304381
3.178645286	2.909167077	3.607652876	4.545320812	5.041989771
4.529893361	4.007998502	1.876990383	3.399155523	4.864436425
0	4.289179165	4.137663953	3.427434128	1.559439383
0.058003677	3.873864397	2.586773708	4.688615213	0.061456411
0.080087203	3.846741454	2.34453258	5.100787922	1.267725294
3.355272422	2.764824599	3.996991759	5.483122571	4.589223631
1.278326815	3.344602002	1.787544375	4.075916779	3.102170888
6.74100175	3.321477806	4.584501217	5.523087154	8.550264994
0	4.977921319	2.252043655	3.198845967	1.635010862
0.759283799	4.35529649	3.611009759	4.405590802	1.976212837
0.090817999	4.611961108	3.593969471	4.248588259	2.727515497
0.259378011	3.61218629	3.317351458	3.618223826	2.51325949
0.119403099	3.114631614	2.496033646	3.54607833	0.127505311
2.313741087	4.696509999	3.51457257	3.947317956	3.032020925
7.623831265	2.675412103	3.834968602	3.968847207	9.02705048
0.020282456	2.684589488	4.630389303	2.68477539	2.684846868
0.094192827	3.613359318	1.958629976	3.615397332	0.100260663
0.061878245	4.216561468	2.60333469	4.400227867	0.065593898
0.118984401	4.365064327	3.107744181	2.04987394	2.498585863
1.81340551	4.088031514	4.278243727	3.272485928	3.07699956
3.361253852	4.94143588	3.987284259	3.223237057	4.067489321
0	3.689612756	1.899074363	3.788289683	0
2.958414569	5.789258995	4.588005089	4.276910851	4.508738359
0.179821528	2.717379759	2.711237898	4.60788572	0.193522075
0.106954519	3.492891202	2.787229932	4.209745873	0.114030208
2.794527663	4.637029158	4.175522731	2.960293672	0.864895435
1.595674084	4.183501632	3.598168193	4.714223457	3.820914358
3.133514525	4.055441046	3.237798322	5.181411663	5.836735652
0.050654709	3.573838167	1.793163189	3.574890131	0.053619939
1.669034027	4.011922535	4.748949118	4.630572138	1.668736013
1.04695274	3.391616668	2.874407808	4.880235799	5.431412389
0	3.872215784	2.460572055	3.71212007	0

0	2.505867729	2.347471358	3.544158129	1.433116496
0	3.692125504	3.69417973	5.77736647	0
0.703162196	4.199844146	3.218147901	3.673978618	1.891366486
0	5.180559144	4.295087366	4.131147782	0.671720511
2.476795486	3.164918906	2.743789542	3.978973546	3.629554482
0	4.430409413	2.748588706	3.819992402	3.321880723
0	3.379783696	3.181506871	3.984654403	2.31590767
0.008568297	2.363423245	0.008573395	4.046218988	3.630710385
5.004136205	5.046610758	2.54807663	2.961550748	5.935585595
1.211348753	3.47214513	3.468934129	4.118464959	2.877103019
0.057542741	1.820521282	2.584794981	3.280332404	0.060964468
0.985460402	3.300080622	1.980796015	3.148193074	3.678064824
0	3.780784297	3.781063914	5.142518019	3.673760653
0.575020172	3.267990414	3.785417384	4.373177663	3.264767579
0	2.89603817	3.799447541	3.592244111	1.907245913
0.053062867	4.17526289	4.791059673	3.725257935	0.056186236
0.187208175	4.274285841	2.279119307	3.371836782	2.283349872
2.594338562	3.595006969	2.594340121	2.81698933	3.917136248
0.33032955	4.269859119	3.810288683	1.995359795	0.362569766
8.883623638	5.474570302	4.294958082	4.047574182	9.012266278
0.294990541	3.72466802	2.607584579	5.200816802	0.322272588
0.013787652	4.161119247	2.387995483	3.241330621	1.643265653
0	3.425225162	3.427550227	4.669013073	0
0.085751384	2.977772487	2.702658149	4.420950995	0.091177196
5.792389429	5.132109733	3.349194044	3.587703357	5.341862724
0	4.01497752	2.591473337	3.470705896	2.116205172
0.044582993	3.926940754	3.21759615	3.392444994	3.929161383
4.615477645	5.397276948	4.61546833	4.556071219	6.310294703
0	4.111142278	3.256361827	4.036592546	1.823171276
0.064889464	3.312460711	1.220829343	4.232295227	0.068812265
0.213984178	3.46147217	3.682393068	3.185383577	0.231309491
1.458459364	4.097005281	3.792315661	4.346557993	2.578203949
0.677977396	3.057234487	3.173378008	4.256684159	3.702866221
0.741784529	2.599042627	4.572422997	4.565508147	2.412770401
4.462369342	6.120338159	4.717159538	3.857337722	7.057410822
5.613167148	3.318639991	4.239185736	3.147728105	8.586010143
0.966964554	3.120505844	0.966966065	3.756310374	2.276015679
3.466765801	2.814480457	3.007169843	4.007580964	5.293205143
2.28160489	6.235448245	4.318152303	4.719354715	5.918909986
0	4.813274718	3.281466101	3.816100651	0.885386338
0	3.889858742	2.476686195	3.110979558	0
0	3.75436781	2.949405849	5.371472181	0
1.22384486	1.852585569	3.117300267	3.784357829	1.221645669
0.341238541	4.63669415	2.737566075	3.588971776	2.021705192
0	3.357429217	2.116881205	3.576991282	0
6.648472408	2.20432304	3.043968511	4.509843057	7.63079749
4.239211057	2.600710254	4.102197783	3.94768673	5.019976222
5.044113786	4.249547448	2.429221248	3.370815925	6.056065828
2.438522896	3.124251901	2.704593399	5.039140214	5.560218148
1.015878013	4.024308971	2.83016231	4.931741163	2.830396803
3.837279077	3.838892961	2.120760265	4.228133504	5.143885993
0	2.027896673	3.480306361	4.906068535	0
2.731832613	2.982488665	3.776293633	4.887554986	3.383145893
1.998017883	3.813930719	2.779614761	1.999401066	1.346643
0	4.861335204	0.75491515	4.310169211	1.733908479
0	3.563590438	2.989906031	4.690692845	2.837865609
1.834910957	3.442487815	4.389489771	4.513213568	5.062601734
0	4.944342088	4.030487529	3.859087408	0

3.084606818	3.748860255	3.084673752	4.29578176	3.995272304
5.518101353	3.898202989	2.528177161	4.391438478	6.498613081
5.104513121	3.976227147	2.46207075	3.080321193	7.74403342
0	3.485354701	0.897011473	1.233984645	0.471454219
2.321018645	4.064613321	6.010473482	5.835358896	3.529806702
0.034446502	3.635112756	2.154864845	3.343899005	3.636548823
0.022913044	1.681509751	1.079900651	2.431275466	0.024169255
0	3.82669406	2.250737752	3.643691949	1.004864056
0.416398591	0.437995522	3.412115835	5.009756446	2.967159832
0.099058695	3.26236542	3.028759789	5.615864908	3.265521594
2.112440893	4.655011565	4.128508511	3.713984133	3.830589648
0.091847249	3.232290756	2.999444438	4.536180089	0.097734725
1.075735594	3.921218777	3.279846267	3.572341626	0.022888544
0.222327682	3.489844261	3.199033346	3.213677493	1.696058681
3.893719621	3.89473691	4.174426472	4.336748954	5.178087883
2.765198323	4.539398429	2.543574528	4.042147271	5.76060865
0.078470027	4.848532201	2.943973689	2.948060438	0.083357743
0	3.953534578	3.835361783	4.489037301	0.637612702
3.558613671	4.060418303	3.5586069	4.727350775	5.142054106
5.25006599	6.067275039	4.136509527	4.448169537	8.457162154
0.140372522	2.577120601	2.914307345	4.229345739	0.150301421
6.772796511	3.516832612	4.769266072	4.632796051	6.642821206
0	2.676434187	3.195143756	4.557231918	4.225265797
2.135013976	3.179100529	3.08721056	4.374671835	2.616140039
6.420143357	2.995028016	2.988351069	4.636586353	9.937526392
0	3.00183123	3.003024143	4.407454	0
1.362957251	4.519612105	3.06962248	2.014906021	3.97745305
1.247142809	4.058862513	2.920654738	4.82593988	2.317577018
0.019253421	3.685547233	2.413613037	4.74760198	1.066534831
1.038858829	4.519201442	3.64278095	3.379895496	0.011652859
5.885167243	3.906277408	4.797777499	4.506147542	4.926926497
0	2.87931292	4.458446784	4.042362925	1.48658807
0	3.405652987	1.541781863	4.89808666	0
0.028763183	2.458165951	2.948731129	4.796893968	0.030362587
0	2.968681148	3.290292605	2.968604605	0
1.370850113	3.686875903	3.309071767	4.929791849	5.546242006
3.901426629	2.739268163	3.901574923	3.243078687	7.36700044
0.011124744	2.863633783	1.039070196	4.382194963	0.011717195
3.638651301	3.698272047	3.340405153	5.195481003	5.191078578
0.629963003	3.616349818	3.953562139	4.399166621	1.122298825
3.865635675	3.625161071	3.331896517	4.257311813	5.600702992
1.561690124	3.621062401	2.721110768	3.556087069	3.194471742
1.266134813	3.701591648	3.542809529	4.478119836	3.179149381
0.904179465	3.137437029	1.743348615	4.373276551	2.961708171
0.086307946	4.001272522	2.976650156	4.673495849	1.287598832
1.166174807	4.052744087	3.039688172	4.894841336	2.215253034
2.608972464	3.8332689	3.194928782	3.195218648	6.197027167
0	3.844841961	3.308665838	4.596429019	0.902496375
0	3.454682198	2.091845136	4.393739464	1.550251666
2.613637457	3.400699498	3.40394733	3.83382492	4.279968079
1.201671278	5.509296389	4.223248514	4.011012989	2.816345181
1.1838667	3.261738519	3.063651027	4.444430506	0.056849009
0	2.920756199	3.09043687	3.377841712	2.508292776
0.978430797	2.775947193	3.667786156	5.095144152	1.971477003
0	4.002486301	3.161021245	3.420202124	1.455787844
1.88155421	2.968725287	3.818841563	4.342372134	4.00942005
1.744390682	5.008172143	2.500885054	3.191068635	2.996275667
0	3.591130231	3.593941906	3.14879991	0.805868914

hsa-miR-3662	hsa-miR-1269b	hsa-let-7c-5p	hsa-miR-103a-2-5p	hsa-miR-122b-5p
3.916017902	1.987685023	12.44335774	4.480958865	3.051442496
2.614695224	0.073161851	13.32903229	3.780735558	5.292732271
0.029475097	1.71740693	12.51077273	4.070308138	2.741376847
3.542273911	10.89936186	14.23910254	2.666577122	6.818885465
0.100876336	7.988464575	13.73747009	3.830011719	0.115390001
0.042807522	5.05566596	16.22920295	2.537551368	1.161486266
2.92492449	0.167316541	13.85794997	2.155514963	2.162146752
0.018391355	0.021714775	13.02523274	3.423579691	2.092020828
0.927222088	4.740188452	12.87161374	3.349653414	4.344299464
3.531363232	1.148023698	11.13982485	3.67738283	3.808809791
4.25459486	0.594467309	14.81026544	3.29419052	0.584533067
1.651994805	0.237443911	13.14165194	2.351258602	2.363011003
0.092817787	11.66737645	14.17572652	3.465707849	3.47645356
2.781818304	0	10.61393355	3.557711299	5.256682449
5.687582466	1.561585241	10.98171919	4.674956657	3.573272076
0.036198713	0.043062551	11.45184449	3.192993485	1.750026218
2.125132369	5.827996914	10.44632633	2.731618059	0
0	5.948910711	15.09656419	1.318491194	2.305931619
1.252027683	8.306308449	14.33035301	1.887127714	1.889280966
0.202436691	7.241058776	13.72376144	1.71740069	2.886003811
2.18764078	1.521406727	13.76382469	2.204413465	4.053340978
3.297835423	0.309017522	12.62245692	2.532118703	3.887383356
0.024189332	1.089436168	14.09828942	3.301816595	3.13311808
0.124078522	10.11684011	14.32943748	3.17633468	3.189299133
3.150262234	2.907028451	12.63131591	2.897377974	2.11505474
1.204339807	1.210338988	14.40476952	3.408988631	3.87899077
3.291776373	7.666074754	12.65638263	3.428171566	6.214703776
2.577443977	9.615374906	13.8367628	1.198030427	2.588255619
0.072384538	2.679680431	13.47544587	2.946977581	3.3788513
2.757052663	1.737355679	13.12060532	2.987190475	2.167899403
0.938745674	8.994985294	12.22792703	2.828444361	1.98702629
0.999701032	1.584604299	13.14878088	3.32148642	6.321456264
0	0.553511022	11.4935248	2.255362032	0
1.288074111	1.292099678	13.09298671	3.081660304	1.28584733
3.400604286	1.295991758	13.33597668	3.881959257	1.304340612
2.462262439	0.293118	11.39663078	2.95545582	3.329010662
1.388013345	0.131348477	13.62926031	2.046644921	3.113940541
1.753723378	0.820322104	10.95243158	2.315227919	2.88406393
1.892392754	10.5635328	14.63924267	2.669748138	2.675109628
1.070344067	11.18835346	11.70983136	2.417723488	3.690626538
4.12161366	0.103124321	13.44956469	4.24742127	4.360296511
1.208656613	3.299491284	13.64067855	2.268935656	2.875081153
3.508055475	1.382293505	14.29692707	2.490138389	2.497325198
3.962708873	6.178161668	12.83541258	4.444909509	3.443565763
1.915616507	0	10.81905554	3.07397511	1.91365958
0	0.926472454	13.02095545	0.92415419	2.475799617
4.094150587	0.168775038	11.20427803	2.951216587	3.472345982
3.719778173	5.41081448	12.83525767	3.751970096	3.340600435
1.350290493	0.117378044	13.94035125	3.283776765	2.006350624
3.119957821	12.14838964	11.89801538	0.863028416	3.783251175
2.597016468	4.183649118	12.9378561	3.598019491	5.223202716
0	0	12.51886786	4.279412701	2.061908391
0.046046846	3.841546758	13.3329794	2.553119539	5.546625412
1.069085966	1.667453372	13.20869589	3.26995997	0.020362336
1.046651872	11.55138547	15.12676755	3.390213446	3.238990855
1.999665689	0.77717416	12.19664873	0.770118573	3.332006997

2.362105514	6.743888497	13.75945262	2.169124885	1.7182721
0.864501703	9.713893701	13.48631903	0.864035748	3.112694404
2.362722111	1.221837959	11.52658195	1.592081236	2.13845184
3.985741981	1.547248441	11.61792887	1.167297873	4.385507737
1.113558922	11.56767786	14.90048536	2.967324897	2.970000099
0.834002248	1.778966911	13.57651684	1.379915269	2.341397462
2.116196487	0	12.94379984	1.86066852	3.471634827
1.029959596	12.12188359	14.70655932	2.039373797	5.570413291
4.354292627	2.286280051	13.28933834	0.974030684	0
0.056236593	0.067472362	13.50024421	0.060375022	5.121452927
0.052237719	9.688580535	13.66228289	3.445564304	1.196317405
0	0	14.26940328	2.978907414	1.980753275
0.982757021	10.78224284	13.56164477	3.144542467	3.78082289
0.573819427	1.412687627	13.86949703	2.317246377	2.867650936
4.288853171	2.225529277	11.58183978	0.930199582	1.499651112
1.797826353	0.057648434	12.35753931	2.563476832	3.584681445
3.317210129	3.370883169	14.30820706	3.347744516	3.088472586
0.00172448	7.04416336	11.17381887	2.008698356	1.006619105
0.284853887	0.378574617	14.1894322	2.69275125	0.343307245
3.213541789	1.030109779	11.52746668	4.133743529	3.74729442
2.558062962	0.335707948	13.74985093	3.060001043	3.087473536
2.873860102	10.30186275	14.90593898	3.23975034	4.395817119
3.982516614	6.082205358	11.83095353	2.905996766	3.051933124
0.077418665	1.9221593	14.73811423	2.69911486	3.99807728
2.704649269	9.990832675	11.40608193	2.89331997	6.033652004
0.854767044	1.409452373	12.84469596	1.80489193	1.803989557
1.765279842	8.638130408	14.82967217	1.153437586	1.769413952
1.562898676	11.2529959	12.55319794	2.296670884	3.6723585
0.86839085	0.871985005	11.63784106	2.613603229	2.800982419
0.058822531	5.336413234	13.29771291	1.220640191	1.849021409
2.333398745	3.702860463	12.18158388	0.206638691	5.625107991
4.294524799	2.917361986	14.80662735	4.077845416	3.425968342
0	9.536132949	13.25113156	3.177672839	1.554730924
1.265759209	4.51687174	13.03509771	0.741320946	3.046520553
3.322680254	11.57164638	12.76529612	4.57232512	6.019410238
1.705539914	1.099013204	14.58052379	2.950236273	1.708039321
0	0	14.21002647	1.54476877	0.967055185
0.001277463	0.001497519	12.92593711	2.591860466	4.467039355
0.058493489	2.614322263	11.84717106	2.612052556	4.127832924
3.634468507	11.03680955	13.1926201	3.820699726	3.625558758
2.269044798	0.787054042	13.56694885	1.312911298	2.47538644
1.95541197	11.17351029	15.01698993	3.40816668	3.64772012
1.221336351	3.31722234	12.72701505	2.887098155	1.223503826
2.676298554	4.670048328	13.35857183	3.538566057	4.096652666
3.867899443	2.374721588	14.00933106	1.805183135	2.590811171
2.207675107	3.043201602	12.9851531	2.205368493	3.194075327
0.861530257	2.128094463	12.22049282	3.247445909	1.414170593
3.277789137	1.16787584	12.39890525	3.38811888	0.830347801
0.022511009	2.929639543	12.65344444	3.292916297	2.112220074
1.603922958	0.004847839	12.66309072	2.020784412	4.272336669
1.694665605	7.999352254	12.59100081	2.44686325	3.946044195
0	0	12.66335232	2.854964122	1.330298404
1.499705663	1.137428462	12.24285655	2.589183446	2.421345412
1.344350945	2.439762604	13.96814014	4.067284285	3.056526317
0	1.083557576	11.21887568	1.903248395	0.757172939
1.323404097	0	13.7264013	2.843320012	2.270288007
2.653093499	0.676337319	12.21051549	2.789152623	2.63649267
0	12.53299836	10.57979619	3.036686237	0.910788265

0.053005147	2.254371827	9.105684246	2.586236361	5.385308754
1.086942566	0.615464629	12.36301577	4.180920354	0.605941856
2.780745225	0.12119632	12.36653973	3.297215246	5.050670619
1.744768243	0	11.29735361	2.409168203	0
0.067165878	0.080962856	11.75515477	3.149913164	2.657316603
3.163143215	2.153368083	12.38204455	3.631130635	1.728694344
1.079323494	0.024753972	14.82806511	1.079910047	5.122344221
2.571997986	1.361116001	10.98102376	2.25405658	6.460704851
2.150685365	0.48559671	11.05056973	2.916049271	2.953570726
1.965788265	10.38369034	13.70826586	1.33026211	4.055337949
3.44225652	1.083722608	14.28452799	1.688724874	5.686965272
3.208749209	4.917026865	14.39273562	2.723450365	3.42915716
2.687051884	1.0734961	11.37933442	1.075744161	3.813743025
0.195569608	3.935875893	14.3223683	2.841548134	0.229748639
1.653762171	11.08246934	13.25367047	2.889141604	3.671562174
0	10.53818944	13.68482967	1.961658451	1.961405223
2.659633602	1.257413544	14.44125803	3.167780518	4.082865017
1.789740503	10.37927013	13.68693511	3.297841197	2.976111455
1.977404617	6.36394032	12.34830862	4.728243213	2.974209495
2.37785192	1.944775671	13.30985842	4.33822996	4.350451675
2.550278964	0.155031224	14.22719317	3.938447034	2.919105963
1.004357324	1.364757204	13.79434381	2.258537308	5.057692165
2.128403061	9.431590572	11.69523379	2.963604083	0.691262227
1.048609981	1.413768808	13.24349965	3.365527842	4.458105656
3.230122026	13.00029157	14.1626059	4.506015802	3.70278227
0	1.452006824	13.49366713	2.836172279	0.889767475
3.047847922	3.305333997	13.54229566	2.010878702	2.460468527
1.244205869	1.874572114	11.13000568	2.64705917	3.15105827
2.675824771	5.275762633	14.3010304	1.666038858	3.097632942
1.038617762	3.05666609	13.12971425	0.010803951	4.644734899
0	6.150622398	11.05642712	2.550026041	2.148326995
0	11.86129025	14.12865516	2.209828478	2.468488714
0.964753848	3.851807943	13.59957614	2.756412212	2.272724323
1.703124052	0.031107871	14.18278465	1.705027819	5.239933492
0	9.745020032	14.15353625	1.972089257	3.427350175
2.009966633	9.938394505	13.72581988	4.601608241	3.685987695
2.385745551	2.736808357	14.60716183	2.396285706	4.033923045
0.010191985	9.155780754	14.57574448	2.639427699	3.759338021
0	0	11.25765516	3.26106546	3.989336582
1.489795856	6.718459236	12.84400223	3.194983995	2.409657839
2.142077182	0.035079766	12.65269765	1.112383954	4.256101617
2.622513158	1.572967957	10.68697718	2.606773156	4.370317097
1.893564333	0.086036691	12.56648448	4.547090354	1.265794277
0.467994694	2.278684843	13.15308529	2.88000413	1.9412332
3.554940675	1.281955814	10.44576926	3.569687507	4.225476412
1.164583828	1.16130994	13.47525498	3.404236307	1.784458809
1.016928904	7.741791928	12.41592844	3.346851888	2.34540196
0.901583176	1.869905441	15.13801741	2.854135085	3.307561791
0	1.851120781	13.20799381	2.934448175	1.175839791
1.968462189	2.215405932	13.22347353	1.65838298	1.273355542
1.205930556	1.710573913	12.75687518	2.617885824	3.971988301
0.048772091	1.801279113	14.12643786	3.428288528	2.838856275
1.930696058	1.521138118	14.80640374	1.519948011	1.92905407
0.978532685	0.979070649	11.89244578	3.13829755	6.340992597
2.656443276	9.573057452	14.42982818	3.29905419	2.431637137
2.852374062	9.563988249	14.39559543	2.841416509	1.206115428
1.131490968	6.273973969	10.2974019	1.132605226	1.744598686
2.709609892	11.21987568	11.59644682	2.514330383	0

hsa-miR-224-3p	hsa-miR-3614-5p	hsa-miR-20a-5p	hsa-miR-369-5p	hsa-miR-184
1.993781658	2.438124307	10.86677115	4.670423489	4.309664237
4.244995234	2.626629378	14.10903225	0.069848607	8.692384586
5.531463618	3.485193071	10.10585108	3.625185105	4.928411768
4.846467564	3.788217534	11.98742914	1.656026274	3.256691415
4.118456322	2.809837757	10.84246787	4.785513128	0.122354225
5.247721314	7.382113576	9.146150672	1.779165077	7.925408997
4.007377931	3.232671364	11.33954062	1.490734844	2.157623707
4.752877629	2.907570182	10.0433836	4.106714562	1.66883781
5.203757939	4.056058131	12.39874524	3.693588032	1.903465188
3.927785506	3.217067597	13.90273738	1.768133247	1.149235309
1.703241385	3.45215744	10.48243761	0.585825475	4.609484621
0.229624553	5.552780921	9.369925606	3.45838359	3.180273703
5.415434664	3.802522677	11.83836022	2.776811443	4.828725667
4.363537883	3.142777704	12.81536259	3.672406388	1.562987611
4.117284279	1.576768703	12.50263208	3.065846771	1.566858447
1.749325409	1.137109523	12.76297618	0.041228805	4.013600122
5.04046643	4.135568487	8.657098767	3.689738422	1.6742351
3.572276232	3.479452175	9.096675309	6.909080538	3.039152328
5.877236819	5.373578689	10.24447624	4.623853672	1.249879811
3.763135265	2.41851847	11.80692048	1.7222522	0.256300843
7.095628257	3.009899679	11.72948712	3.015849291	2.207656982
5.853329575	3.87934447	11.43988291	2.553148847	0.305551533
6.668085052	2.713655704	10.03887037	2.120484923	6.76053704
4.976611874	2.569271869	11.51016386	2.1200591	3.193441357
3.79697182	2.906123114	12.16626104	3.418553323	0.150494932
2.682137847	4.803487439	7.910942898	4.432760099	1.208020429
2.968781499	3.29040684	13.06420336	0.979062057	1.972341616
4.620053176	4.096000006	10.05942596	4.617471546	1.194000777
2.95566552	3.180269585	10.61010538	3.183390842	1.903546412
5.521501031	3.356083868	12.53319244	1.740244831	5.873978559
5.081037975	3.419915845	11.16519626	6.559770171	1.288025299
2.8069176	3.169480463	10.98036383	2.584534343	4.247456848
7.448600976	1.868995945	10.50196469	2.681236763	2.410947183
5.929326512	2.227557008	11.30639313	3.606539753	2.94080595
6.339641291	2.99680705	11.30366807	1.303685258	3.60301801
5.349588596	1.802519368	12.29454823	0.274144337	0.289903802
2.500101477	1.394532326	10.22079382	6.355634617	11.93568034
4.90161828	2.718180626	13.220563	6.610831352	7.657214898
2.339992111	5.911852469	10.73658711	1.900950872	11.33955255
3.914993611	3.425997747	11.47721992	2.68474229	2.092108539
6.758229463	1.959206846	11.64227924	1.314423918	1.309115848
0.065690752	4.895560719	10.07353538	1.210098892	0.066835399
2.498512368	3.867224641	11.0301492	2.049902219	7.288787105
4.874048838	2.246245666	12.48128808	4.454293622	4.09178072
2.231464243	2.712549893	11.5102147	6.77831164	2.712293384
5.368969067	2.21609788	11.14572149	2.475630056	0
2.16615752	2.165384449	11.18373783	2.62206308	6.533977093
4.50583649	2.713926692	13.19874929	1.576041821	1.566794132
4.095740875	1.355688104	12.11725985	2.452455323	0.116413587
3.590163446	2.604305425	11.85120424	6.404903216	1.419003548
6.080155699	6.036071163	9.859822899	3.335149181	2.334048201
4.721261418	2.764644411	12.85231453	3.232653383	7.001852059
5.821384558	3.954052732	9.170440634	4.064664943	6.610004665
5.632685047	3.100532164	10.81205151	5.151986171	2.416790504
2.386442701	3.877899557	11.45995335	0.013859682	3.068660383
3.710754084	3.219617854	10.84688395	5.342594815	5.947386283

4.090258494	4.458506899	10.02627438	5.946214426	0.602488719
7.217496435	4.106104985	11.67427671	0	0
4.020669451	4.407710128	11.5777144	1.890470253	3.212943005
2.782309178	2.785227638	10.51696559	6.786519474	1.174211019
4.634934521	3.753411095	10.47538453	2.149310058	9.884851518
4.427581602	3.064767665	10.4102058	4.483582898	3.639960094
1.859819976	4.050411745	10.76751679	2.105081963	0.689565548
5.8551375	3.043878951	11.18384767	2.363458232	5.405449462
0	0	10.56663786	4.281359042	1.96589107
2.877103766	4.396392097	11.77293213	2.272743177	6.158120754
3.991342328	4.093866346	12.80828817	1.195837962	0.062106888
3.784881377	4.369871672	9.785201704	5.999296309	5.224624885
6.181622858	2.560312903	10.40210866	1.563883365	1.977249294
5.768567389	3.180364926	12.36256326	4.154279719	1.409479902
0.931150035	4.139112982	10.30021895	4.13797681	0
0.056267555	5.399816608	11.08751833	0.055117973	0.057228221
3.375233138	2.739129234	11.23358956	4.407171526	9.555221806
4.533978676	5.332221026	10.02481441	3.817512901	0.002008921
2.725004222	4.43144874	9.162770148	2.722503383	2.719154793
5.178183028	3.747069667	12.45775039	2.040856605	5.664146252
2.621019899	0.300190052	11.37718453	2.619617601	3.454398187
5.66118205	5.118489234	11.97767114	1.643478486	1.04728161
2.734731229	2.903129787	13.10538562	2.735001458	0.830214437
5.13019048	3.868494362	11.68350527	1.287496451	1.282630801
0	1.903218927	11.87711197	1.903158794	3.885439972
5.04570295	1.804071309	11.94270551	3.936672182	0.857759334
3.547037874	2.797216573	8.761263906	6.116914373	4.626436568
5.828471594	3.557123218	12.13258885	3.294187995	2.296540377
4.642766258	3.119536088	12.22344653	4.541572446	1.425004401
6.194445877	2.283778168	12.27475716	2.618511994	0.070123678
1.671874679	1.675472171	10.76676502	4.374920212	1.664759955
6.025681435	3.795810691	12.3471838	2.125040621	1.450127177
6.533325209	3.551177659	9.968375215	3.056301369	0
1.950052846	5.521637337	9.449250915	1.264152242	7.238863856
4.98889818	1.294207782	12.40321886	0	6.592522435
3.472878406	3.851477713	10.75312501	1.707953454	6.981397681
3.534171964	4.036550874	10.26881444	4.270199241	0
3.814924628	2.591930525	10.36665312	4.255548487	5.217295832
6.334914078	5.023627662	12.45038159	1.218762952	1.844648828
7.28409091	3.280844854	12.58596838	0	3.143138072
1.314793713	3.113241768	9.866291833	5.218175563	3.452242857
6.229867966	3.854070368	11.35283976	2.535645434	0
4.980958081	2.287552484	11.70951538	2.622445814	0.071128626
5.690973834	3.568848832	11.45400293	4.488477171	0.387020672
6.218094279	4.160464534	11.82729473	1.406285895	0
4.993411718	1.482105513	11.14061024	3.193648507	0
4.580759119	2.126054976	11.97083008	3.481869774	0.864363609
3.704553314	2.007823672	12.05893171	4.378649682	5.134180502
4.887897099	2.929458894	10.58324734	2.930237346	4.131124577
5.195043704	2.02091587	13.17251319	2.607522975	7.11305098
4.390018752	1.092651305	12.00912763	3.723333544	4.390453816
4.303254686	3.135993869	10.9883207	3.912435647	2.497698384
5.526486832	3.096668033	11.4778494	3.958904207	2.422887362
6.352988528	3.65532108	10.75303079	3.486565146	5.731654136
5.778330209	1.32265975	10.00666419	5.961240819	7.365988151
4.400847628	4.557992259	12.20037587	4.348985948	1.713556869
2.47292569	3.444985578	10.38693894	2.07979612	1.838434389
1.475963282	1.475267068	13.22016801	0.911053058	5.211415242

5.303194931	3.281625821	12.20876963	3.087842313	2.86031994
4.99514441	3.643022806	11.17326054	4.855107549	2.922196619
4.782600435	2.802238219	11.50893539	1.365394266	2.46316808
4.800714524	2.262481901	9.860520614	3.770805423	1.24196574
4.371679132	5.412757886	11.62840395	1.884774086	9.526017519
3.34445697	3.495538198	11.34927957	4.350095063	1.116724759
1.681064637	4.290149379	9.562454182	3.440115716	5.826515713
1.353376494	3.01597717	11.60976202	3.510090315	5.675511993
2.216267946	2.211399708	11.84286227	4.52828767	0.478784816
7.353052637	0.10031523	10.37483008	3.034024049	4.949591065
4.777302982	4.129162645	12.63379579	0.025665281	5.134850566
3.607341766	4.765366059	10.73125432	3.604945954	0.099712443
4.364385925	1.676499077	10.90761057	3.281152216	1.07407913
4.543224495	1.699169732	11.64250819	4.532850497	2.861945423
2.075860176	3.254589657	12.996101	0.017130563	8.10381711
2.281513839	2.543474871	11.17141976	1.549524712	1.961735733
2.339911356	1.900592787	10.66277714	1.900875227	1.259610667
4.565125216	3.381908358	11.43156244	3.693050367	2.974704296
5.463876494	1.563651454	11.0948503	3.55826903	2.297793135
7.914473371	1.950803546	10.20304538	1.30718311	7.512638776
3.200331351	3.95163385	11.39176833	4.650063238	5.424792492
4.839375829	4.230294002	12.84773589	4.26587817	5.329507248
4.786983593	3.08083037	13.18512974	2.117404016	2.120289
2.313179259	2.618917697	14.12115638	4.734316716	4.369495282
3.88776531	2.991205898	10.89610215	1.519392916	12.69659638
3.632859097	4.073614959	10.3627912	4.21567203	2.167309312
0.116730854	2.459245183	10.53774671	0.114043544	0.118988076
6.584612002	2.6509674	12.82488426	2.317757421	6.105440972
4.42575542	4.271450986	11.5883046	2.903993719	1.065814005
3.517991417	4.146364809	10.57845805	4.760701616	4.584352157
5.428759437	2.546314713	9.573075597	2.544743037	2.361921027
3.574358306	2.879769687	11.62400346	2.879101827	4.329951806
7.377357401	2.755856213	10.7580159	0.964797893	1.542363402
5.403320268	3.848478218	11.62439678	0.029816693	1.097782398
6.760256231	2.968811712	9.751553632	2.776423863	7.866339465
6.432454242	3.838114098	11.93639369	3.844284772	2.019723392
5.110499423	4.148071094	10.77852505	1.959140861	1.308636906
1.632092552	3.759053093	11.7119209	2.863737938	8.424696947
3.487399462	2.60068369	10.54649013	4.271512883	3.062257043
5.43105686	2.228252743	11.85170825	3.281561067	0
6.007922513	4.416095981	11.00920367	1.111865121	1.109963353
3.942044701	2.470985396	12.56537451	4.662382784	7.445884969
2.677750413	2.340263052	10.41754318	4.086767556	2.949264176
6.281722249	2.272928385	10.78116703	4.338939744	1.521374468
0.091918482	1.290187291	11.90944716	2.371560039	2.981103983
5.298741843	2.214893913	11.93332863	3.946008256	1.162637367
5.419304903	2.831878542	12.10278453	3.610755216	2.609074
5.298479916	1.867968071	11.95481883	3.170600694	0
4.643789727	3.163866057	10.83415871	4.439651252	4.823271021
5.158746467	3.060794994	10.29118516	4.863726162	1.277938594
4.508096925	4.798200736	9.843961867	1.895888686	0.457456575
3.856383217	1.183773883	10.67718275	1.805329073	10.74897131
3.8234797	3.503641806	12.04704787	4.559441427	3.617665085
6.772610865	1.97144831	13.5600405	1.558712978	1.559035676
5.733378262	3.296916936	11.46414919	3.159405945	0
4.869760363	0.697420472	10.57666385	1.880985735	1.586927718
3.363533945	1.744538228	10.66529438	3.190947194	5.528207262
4.481430798	2.041570199	11.010236	4.629716152	1.735997699

hsa-miR-581	hsa-miR-3923	hsa-miR-17-5p	hsa-miR-4664-3p	hsa-miR-34c-3p
0.089155229	0.112616845	11.38463893	1.956289099	0.101498078
2.278448902	1.223913632	14.2398068	2.593058786	0.065805599
2.139435822	0.034655848	10.50003136	1.106860011	3.158780855
1.653931993	1.057560512	12.35189884	1.651229855	2.076561046
1.362246847	0.122075703	10.82493899	0.084700196	1.371931822
1.158826943	0.050600543	9.285067747	0.036597832	0.046144658
0.128270912	0.165403211	11.31908097	0.112017229	3.453752037
2.678047787	2.091443811	10.42327793	2.673079067	0.019742519
2.484927436	1.90335716	12.22703829	0.929389898	2.701225068
2.516630736	2.796948907	14.64718969	5.000744823	0.043430123
1.715546137	2.149076292	10.58979495	0.58840692	2.768810762
1.641428786	0.234398658	10.05178255	1.609115005	2.81133684
0.088654239	1.337584228	11.67168077	1.321827048	1.343345667
0	0.982093661	13.60260833	2.783591888	3.779847315
1.555035215	1.56787473	13.17549247	3.227354077	3.048843164
2.169992531	0.042675631	12.89872855	0.031012584	1.749366924
2.756360654	1.673607014	9.422270177	0.567956127	6.887829757
0.357634785	1.074199476	9.416211625	0	1.728315335
1.249458162	4.284124966	10.34995976	1.24059774	0.073855697
3.427552847	0.255569251	11.95987407	2.315066179	1.718459905
1.516671783	0.181992549	11.92638612	2.142807668	3.507049761
2.93002461	1.829446109	11.09185646	2.863584349	0.264921982
2.11510013	0.028381201	10.40331953	1.688295121	1.695849804
2.091454052	1.446622649	11.38706915	0.103450723	2.11479504
2.087277443	1.442697039	12.10438661	2.060426034	2.110332584
4.325503527	1.207551876	8.536032237	0	2.126421748
3.140528298	10.30224476	13.63073715	2.779971957	3.668841694
0.050467808	0.062482778	10.71797216	0.044874239	2.854228425
2.659199546	0.086586944	10.58650384	0.061313207	2.342616484
2.48748257	1.738654322	12.49194348	1.122937731	3.77160598
0	0	11.68582663	2.036626906	3.953460271
2.584557836	0	11.35706219	2.321551043	1.584597733
1.360043314	0	10.73299671	0	5.335066645
1.679675079	0	11.67007669	3.240317878	3.782164596
2.366891146	0.098363394	11.54300215	0.069179175	1.304374464
2.894255466	3.625463531	11.93483836	0.18334197	2.495875624
2.803098638	0.129939798	10.51403199	0.089756541	0.116729062
1.756434873	0.818805136	13.69976063	1.366930191	2.887321235
3.148180451	0.084837701	10.76769242	0.06013568	1.264905136
1.667978376	0.021713684	11.66630602	2.084724852	2.092151756
2.712239498	0.102075759	12.00991468	1.925502213	0.092181097
2.588186115	0.066699154	10.39079758	1.199643618	1.836093088
1.382058918	0.129475109	11.37485186	2.004097438	0.116321787
3.251532996	1.187251249	12.89252952	1.181027723	2.244520371
1.507603763	0	12.01265372	1.920106808	1.505817956
1.494370611	1.493376887	12.15646052	0	0
2.134509961	0.166840161	11.80449911	0.112901047	1.494171573
3.873110318	1.56781029	12.707622	1.528846146	2.705460245
1.986584742	6.510778619	11.85714057	0.080861523	2.446326752
1.821700909	2.129672571	12.69511144	3.617781425	1.816656269
2.011179595	1.595550979	10.11104984	1.594993657	1.008822221
1.157286412	0.662982406	13.10874914	1.167433186	2.274924477
0.044163014	0.054500284	9.60137155	1.164967833	1.173754441
0.017511391	1.668146828	11.04530972	2.083229902	2.090516246
0.011793113	0.014315602	11.36284385	1.638023912	0.013146024
0.771537534	0	11.0909329	2.015958247	2.246850111

1.732708853	7.93976693	10.49592178	0.599139598	2.168409394
2.396105008	0	11.12210469	0.868158069	0.864055092
1.218670171	0	11.67542265	0.708893646	0.702682075
1.548950875	0.674399328	11.44905781	2.518155979	0
2.735208707	0.03543507	10.41192978	1.109182603	1.114503443
3.215189896	3.062915834	10.3514983	1.389802936	0.833543251
1.192005332	0	10.89442835	1.201730295	1.860257436
0.007558287	0.009155355	11.07045298	1.028924744	2.36303029
1.966883634	0.974588798	11.15835113	0.974993998	1.553388069
1.207050042	1.207271168	13.25821392	2.245303986	1.211261455
2.241157736	0.061981538	12.81870944	0.044527449	1.196580811
2.302714858	0	10.16110833	1.568352831	0
1.564413601	0	10.54908339	0	4.558533096
1.411804609	1.408844641	12.25158711	1.42739197	1.402331738
2.228669071	0	10.84010728	0.932597013	2.225199132
1.178327092	0.057113911	11.05153068	0.04114903	1.18175559
1.575084699	0.206061634	11.20921544	2.201750739	1.596072602
2.008417691	2.816979688	10.12323397	1.006379651	3.710334284
0.267670895	0.372652183	9.84198498	0.226762409	0.320105653
2.626444034	1.030392024	12.91653193	2.847125518	0.008665358
1.849485775	0.330744765	10.90972762	0.205444058	2.598389901
0.0121446	0.014744892	11.80457987	0.010898033	3.530281738
2.341079265	0	12.87760293	1.38078691	0.826084934
2.680697357	3.208815633	11.64421111	0.065474998	1.926103621
1.498183438	0	12.48944371	2.903340883	1.903608115
0	1.408064501	11.8914269	0.858624048	2.949883419
1.150741035	1.150463034	9.096591938	0.034714363	1.153481016
1.976552627	4.219001976	12.17720181	0	0.981587603
2.401687214	1.424796407	12.54609163	0.87196481	2.136914255
1.838663274	4.026444969	12.20861271	1.827189271	1.847639831
1.646492174	0.236606406	10.94019792	2.272849089	0.208365372
1.444111176	0.153188747	12.15917081	2.068553858	2.568538244
2.114678015	10.84590132	10.36456903	0.683742419	1.555300364
2.617912854	0.74721286	9.980219433	0.74734465	2.416288104
2.877839931	0.514522045	12.33705292	1.606601854	9.349792984
2.127198526	0.031488291	11.00254087	2.121193605	0.028820576
0	0	10.35408746	0	1.956398692
1.590776241	1.590869	10.52665556	2.328088798	3.914264756
2.866803339	2.281616105	12.20604101	1.825915949	0.063222886
3.637944573	1.444246739	12.81087333	1.449979259	2.637190308
0.781719988	1.704231465	10.53621642	1.324980203	2.478204641
1.956004314	1.543491176	11.35866191	0.966811591	1.954560119
1.219174722	0.070982324	11.9651434	2.855128011	1.851154924
1.957540339	0.3856947	11.56009361	2.571615081	0.330527946
2.601492849	0	11.78894601	0.858832371	0
3.050738359	2.204899566	11.51325469	1.487880577	4.329356681
2.133472321	0.864162906	12.35606304	1.422884859	4.174285521
2.190867271	1.162814696	12.48901952	2.064691496	5.344648388
1.084515115	0.026394404	10.80786433	1.082024257	5.520580215
1.015677378	1.604020319	12.84216917	2.605166759	2.830015101
2.93121052	1.695646513	12.25885535	1.688915437	2.447093697
1.334557817	0.798704917	11.03750263	1.73791932	3.003707335
2.048134137	0	12.11160554	2.916583745	2.867597684
3.021985268	0.114004887	11.04817945	0.079464194	1.996289907
1.072508903	0.374269377	10.35359281	0	2.191779754
2.283098675	1.324694591	11.7632239	1.72825265	2.27251104
3.285332325	1.5433648	10.82210051	1.864397715	2.477725095
2.87373025	3.560457982	13.13334256	0.913397706	2.197382136

0.050805284	0.062911513	12.60918352	3.246019354	1.822675381
1.750059964	0.612620989	11.78680532	2.228186807	3.146122579
2.436761239	1.359884889	11.36763445	1.977213424	0.107932126
2.565763544	0	10.45262087	1.538910745	2.655729002
1.872522849	0.08017499	11.6684226	0.056985234	6.900745251
1.723865777	0.03696296	11.48575711	1.113726848	1.72815724
2.425449003	0.024540471	9.787840946	0.018029969	1.079922987
0.539881843	0	12.55254423	3.085943922	1.869822491
3.275593759	0.476949628	11.80276722	0.276314149	0.402081643
2.730158688	0.107435628	10.22894658	2.974246873	0.09692318
3.116452708	0.02653384	12.57491385	2.102832649	2.438508783
1.934788761	0.099495144	10.63010718	0.069929529	2.724405343
3.102491253	0.023239038	11.477808	1.670123722	2.098452985
1.668281195	4.878679543	11.52330794	0.159651426	0.216414503
0.014559583	1.056726026	13.03604312	1.05545844	1.057927936
2.283351641	0	11.2719221	0	1.961612427
0.067893103	0.084815815	10.62081438	0.060120932	0.076845006
1.795079001	6.354266041	11.5229473	1.812598492	1.122025893
1.564214473	0	11.75968272	0	0.982494012
2.370890937	0.099570805	10.60109279	1.918081113	0.089961568
2.096326904	0.153296035	11.39522563	0.104515138	2.568880811
2.097640914	0.552055846	13.14236087	3.280265409	1.002287693
1.883643895	0	13.33519649	2.366877409	2.681317602
1.703797888	1.694579266	14.06843916	0	1.403438615
2.160161092	0.17612665	11.29794518	0.118579665	2.188452208
1.452125953	0	10.43514724	0.892830322	2.645406038
0.093759844	0.118718936	10.83594691	2.408493564	4.593403051
1.241742928	0.078777613	13.05094828	0.056037746	3.514078495
1.663853008	0.020608562	11.29969129	1.064397571	0.018901987
0.009752744	1.038061955	11.08374702	1.037268049	1.038875815
2.731493465	0.715434683	10.19978647	1.235863274	3.232053045
2.886273653	0	11.65531215	1.4915952	0.919150953
2.274936386	0	11.24160594	0	0.964571499
1.098372304	1.097940533	11.24014926	0.022568785	2.723065924
1.55979594	0.979231807	10.03808121	1.560705482	0
1.36073501	0.121514412	11.92603868	0.084337594	1.370356008
1.307110074	0.101907083	10.56903938	0.071525412	1.95656218
1.630945571	0.011891925	11.20649262	1.037471536	1.632207155
1.038342599	0	11.22175422	1.952484476	3.259643338
2.037192027	6.972420815	12.30332349	1.133854758	3.698681575
3.321641515	5.304294167	11.59286655	1.107200713	2.145395138
1.293966767	3.98877252	12.63552373	1.3104512	6.576597856
0.068183068	1.89919778	10.85352497	0.060373599	2.671854992
1.761654034	0	10.53533884	0	1.744726462
0.074515147	1.284867818	12.04863444	0.065882751	1.928161332
1.777241639	3.042523426	11.79028621	0.037595131	3.233086598
2.60779524	1.605395602	12.12402578	0.003833804	5.58048711
0.902140458	0.903364625	11.98211396	2.19491635	3.434582721
0.67415027	0.680349812	11.02803201	0.679312084	0
0	1.277550601	10.78140491	0.754977014	2.214152262
2.254160035	1.901181885	10.69781178	1.742442377	1.472190315
0.046765456	2.839487241	11.01688991	1.174392676	2.235639706
1.521402377	0	12.03642354	0	1.519905044
2.555776945	0	13.86270023	0	0
1.862865042	10.57730041	11.44779502	1.462060459	2.174161725
2.144751675	1.211288892	10.57078597	1.909370116	6.350078958
2.491948005	0.041216466	11.13554932	0.029978433	1.132638094
2.051325951	0	12.0283481	2.319361252	3.391936634

hsa-miR-4326	hsa-miR-214-3p	hsa-miR-552-5p	hsa-miR-122-3p	hsa-miR-199b-3p
3.284806835	5.787177835	3.052337076	6.769638043	12.92658881
5.514586296	2.293668131	3.792056992	8.026097199	9.914854248
2.146041811	4.561343663	9.174864824	10.15387241	12.00907221
6.414403583	2.077074466	2.076212644	9.883962553	11.43045003
5.591675122	5.666872812	2.019560931	8.964076345	13.69011843
0.049571079	2.209668518	13.26823842	9.87283062	9.414300638
3.241247533	2.162095329	1.47996579	10.37549526	8.827116526
4.013837528	5.014551348	3.27323659	9.620388387	13.63970201
4.468296736	4.207135526	0	10.819244	13.01376481
6.301058727	0.045305769	1.148476184	8.923116149	7.261590825
6.054293687	5.447616652	1.067214987	0	13.21872786
0.227769073	4.360307298	0.236701221	9.299656537	13.6310866
4.078588295	1.992508703	8.391655494	11.19798418	10.96503342
5.803650333	4.363913363	2.559000854	8.362449341	11.07794477
5.819761317	3.340457156	1.563602265	9.162178636	10.55838608
4.964785316	3.660423095	3.001709766	10.31095847	11.0819653
5.804947741	0	0	0.574918067	11.30496189
2.88640526	6.483364514	1.549066881	9.928471919	14.00088988
3.829196374	4.621032134	0.082072033	10.59387014	13.07530243
4.29820402	1.722668906	3.762423623	9.161040431	9.648735613
4.661252742	0.170813675	9.040648185	9.465569145	8.772092285
4.941218181	2.550023993	9.283239687	9.509825968	7.504180075
4.046591897	2.938741352	12.5796195	10.0866275	9.642907312
4.737440409	2.570960311	3.191797583	10.83325477	10.93261832
2.911934453	4.438373688	1.439457545	8.732707304	12.92553694
4.383510703	2.123856992	4.333419888	10.46430218	10.5506163
5.848944069	1.559309534	0	8.079344518	9.504701291
2.589071138	3.085000211	1.192998051	9.714556848	10.49573341
0.084690508	2.681684801	9.206670696	10.64644521	10.82281859
3.511805223	0.038174875	5.287820254	9.980774327	8.811520839
4.279177531	6.009087648	3.104675664	8.05832547	12.94812972
4.169457197	2.321512669	3.321476265	10.75606674	9.979649971
5.585962434	4.223489501	1.01253445	1.643063554	13.4665263
0	5.351973052	0.765684152	9.058476787	13.39922114
5.406821504	3.752930714	3.229204147	9.491471521	11.83374739
5.345368388	0.268277601	6.450078961	10.15005831	8.308992098
5.531258733	4.257007605	0.131005524	10.34309259	12.18130266
5.446051165	1.356153234	2.530483993	9.665672186	8.62390862
4.390355751	0.080439341	12.42629144	10.26057012	9.405906666
4.014729147	2.90872169	8.346892029	9.518666085	11.19377969
4.366320446	1.959615442	1.307457027	10.3477947	9.606138366
2.876508992	0.06336165	8.425785653	9.613931819	9.028865548
4.145371287	3.540835559	0.130536096	10.17234954	11.18218412
6.149683319	4.53071371	2.244348201	8.856809973	13.04437399
6.402693469	5.388772722	0.936945378	6.700819383	13.29254345
4.878064767	3.343473543	0	9.277391954	9.753902783
4.79197437	1.495369672	1.483744673	7.389902436	9.516337186
4.503760861	2.258361651	0.199512891	9.687404209	9.407320989
0.113459159	2.790439703	4.518928589	8.843096946	9.906263609
4.683661238	2.128668542	0	8.65544324	9.159783485
3.335187967	4.261499698	2.011499893	8.948011584	12.30608795
5.483483441	4.40891785	2.457223651	8.444892834	12.50714
4.166317166	1.173495982	1.790133091	9.782435991	7.951530383
5.195858712	3.27180168	0.02145798	8.960097071	11.97775628
4.159662647	2.061676479	2.650968047	10.19589872	10.34364496
4.862092203	0.771245027	4.968869977	8.279773455	10.05648138

0	5.654116566	0.604009871	9.502282544	13.2957919
2.604863069	0	6.962262696	10.62768873	7.714385408
5.21620967	1.214040719	1.597139647	6.809801827	8.669053903
5.588314868	1.539431318	2.63909025	7.109306534	9.339006865
2.477912145	2.744720384	4.42309096	10.16625395	10.76595475
3.541793159	4.124319645	2.55957707	8.408023597	12.76886787
3.784426996	4.162486082	3.065096215	9.681910812	12.87378762
3.746363182	3.044024329	12.26117227	10.8407353	10.30025003
4.046935407	4.817320558	2.286201344	2.961756738	12.7412616
4.119797205	3.625705907	8.282497523	8.211263872	13.17304125
5.854156239	2.586225256	0.062425175	8.822608038	11.69812805
3.678083746	4.500946611	7.325068456	9.89956934	11.58401657
4.060454753	2.782168643	3.973013088	9.590058836	10.51178289
4.963331382	4.456801959	2.137501303	9.666499951	13.47678597
4.47365742	2.484616324	0	7.564328902	10.52651908
5.321037245	1.181500337	2.565593952	9.002296166	7.453644728
4.925667426	3.088273652	9.166687375	11.17252824	11.29387359
4.469815044	2.594386155	1.592888731	8.314556555	11.47176639
4.467245177	2.71764202	0.377126686	10.36824992	11.86771551
8.320700001	2.852066533	2.364313387	8.225023516	11.48431969
3.097299735	3.956696544	0.334495455	9.568065802	10.88975005
4.467181467	1.643532147	1.047050438	11.17461212	8.51658104
4.960393376	2.330145757	3.186727078	10.02186921	8.429299463
3.735381131	1.92824485	0.093505897	9.900297598	12.05620865
5.275038222	0.927586602	4.690290216	8.614489725	10.12161152
4.22345741	4.289675085	6.370845559	8.751654596	13.16547369
3.546805285	5.959989479	6.732014908	9.938785352	13.399711
5.292616372	3.971666438	10.10694079	10.54638533	13.15180922
5.952489644	3.878255561	1.425659806	8.317519982	12.14423415
4.790357258	1.849015872	1.215101436	10.70755681	9.182434938
5.752360607	2.36903217	8.122545246	10.50415391	9.916354704
3.631504784	3.42578954	11.78368094	10.16814031	10.71541375
5.026166777	4.039767147	0.686393099	9.70357777	12.79986666
3.961636301	0.742633423	0.748628913	9.552757998	7.881664212
5.996913526	1.294698666	1.581564727	6.967392449	8.183154569
4.325400987	2.460845779	11.47053859	9.098995082	11.23736947
2.759013005	6.287575421	2.276402295	10.23924732	13.6935076
4.007608424	4.814997553	2.591918505	8.953437304	13.71342186
5.189065017	3.114024374	2.886493976	10.52276342	11.39845721
4.13573779	2.158559634	1.445008629	9.046465903	9.017546502
4.221328953	4.766314122	2.010870966	9.097894657	12.50226094
3.753989239	3.40722817	0	10.88305311	11.7004184
4.656342327	1.223523435	3.317796847	9.64469044	8.254128645
5.609544386	2.74842071	0.390406251	9.783652907	12.04786451
2.590279589	1.406000438	0	9.98947687	9.524501936
3.569447222	2.204369821	0	8.651792712	11.82510529
4.942555479	4.237068328	1.815756173	8.760647817	12.82966834
6.374907228	5.676113917	5.115373098	6.41185397	13.22556735
4.940154508	3.828540229	7.296380639	9.221875872	12.63783836
4.112037449	3.483257848	9.102533559	9.941642841	12.34291482
3.306085048	4.309814362	7.598581716	9.503237934	13.33401481
3.475712472	5.808609577	0.7998656	9.178511467	12.72721552
4.573444941	3.771828231	5.213199431	9.241614754	13.01040955
5.976696534	2.444041972	0.114911927	10.9998718	9.790934563
4.245244257	5.227716933	4.984266172	0.773907387	13.56315009
3.244570793	5.07374148	1.714584918	9.295753532	13.57159876
5.567095562	4.384571601	6.409459741	7.719708116	13.10387276
5.498429717	0	4.248962745	8.001118583	8.235020249

3.87820924	0.05978731	2.255017205	10.16318013	9.274230783
4.162607522	5.209465914	2.672446144	8.799266832	13.16287558
5.121148028	1.366137881	8.281269627	8.281932346	8.496752315
3.039395539	3.92316306	1.514920604	5.342081402	11.79873906
4.068138059	1.884790633	0.080771199	9.050020036	9.342610729
3.879122123	4.880912753	5.273022604	8.712656502	13.76179131
2.697475863	3.286702682	5.90765059	11.29527969	10.78521864
5.387979672	4.178206252	3.108661081	8.300882919	11.23229973
0.458464681	0.434173322	0.48347854	10.7186701	8.170384992
3.463512373	2.421009796	10.05598534	9.887889511	11.02520416
4.833707006	1.086088003	8.375893158	10.12367308	9.080281525
1.305722101	2.729923856	1.945571726	10.27861805	11.68654904
4.924790694	3.571710955	0.023391616	8.706117672	13.04302875
0.239093534	4.254742169	0.248645168	9.865465903	12.72043392
4.549042738	1.057777053	5.220528932	9.993948717	9.12371485
3.655557191	2.281523585	4.654085507	9.296594131	9.219080418
4.917951982	2.946572449	1.258238991	10.12295175	10.22903872
4.677588704	4.012576692	6.8951034	9.548299377	13.26558633
5.14210484	4.294795385	5.526413472	9.015803716	13.21156424
5.951292664	1.951192024	1.300376473	10.65774463	8.788539761
3.631882536	5.040657112	6.656127907	10.08130802	12.71365305
4.036988618	2.412244073	2.558163784	9.536080793	12.01161483
3.568097374	3.19214277	2.678032029	6.969199248	12.1736667
3.08034101	5.749733247	0	8.962346527	14.18123581
1.517578576	0.16540921	10.78695412	10.37810818	9.768947559
3.633029116	4.992346936	2.167815016	8.132712913	13.89016732
4.525876039	2.799601637	0.1196721	9.045646869	10.36582965
0.077078825	0.074740923	0.079361742	10.1356009	9.498688575
3.269181602	3.097613734	0.020743043	10.30270773	11.99334152
4.382130724	4.760284328	7.384394773	8.713722161	12.90355825
5.429861969	2.990738315	0	6.016494252	11.37899917
3.779930866	2.468500649	11.28021055	9.377663639	10.55405912
0	1.541777837	0	10.84045241	9.457567185
4.677080397	1.099748366	3.469559065	10.23871874	7.147142356
4.214697535	2.292517834	1.972242497	10.78066462	11.69445887
5.451297975	2.809528677	2.808834284	11.11230224	11.61241059
4.784908524	3.011662886	12.18393872	10.76218041	11.25151422
2.375700215	1.038980346	5.347348633	11.11429239	9.422810389
4.577697572	5.776172973	0	8.013021238	13.22535585
0.634075106	3.540672578	1.782776508	8.706999029	13.27226269
3.976537607	2.146492949	5.460939382	8.717566065	8.017400725
0	4.089126175	8.159692918	7.812549523	12.3457252
4.981290161	5.091123188	2.338125949	9.462755772	11.9719622
4.299274346	5.766374161	0.482870689	9.946411716	13.89131185
3.582792143	0.088486495	1.282869193	8.416423083	10.13045128
5.060304205	4.333994571	4.777115902	9.453349099	12.82384996
5.384460735	5.070845151	1.016567749	7.828453979	12.39587445
4.012836298	2.661631441	10.54465339	9.743755267	11.25783257
3.767770988	6.051253294	3.836546431	10.39017215	13.25512005
2.775716082	4.626349814	1.964741009	9.074236224	13.23160913
0	2.609356537	5.842764425	8.739545559	10.27460662
5.186142186	2.237691026	1.179051873	9.963876206	9.593992807
4.438873616	3.241252112	0	9.612499111	11.9419567
4.28807025	0	0	11.06271902	7.913139795
4.222606652	3.296346917	7.109327795	9.699653613	12.76567526
4.128720142	2.688471254	1.885902698	8.712764424	11.93450551
4.106403534	1.744598479	11.13285675	9.137520179	6.922857137
3.490983882	4.67725232	7.565304167	8.509178295	12.36181341

hsa-miR-199a-3p	hsa-miR-512-3p	hsa-miR-216b-5p	hsa-miR-767-3p	hsa-miR-552-3p
12.93138397	0.113125365	4.677199585	0.093595616	2.437045856
9.918671725	0.072762991	6.744316918	0.061010335	1.854230711
12.01025986	0.03478884	0.034924885	0.029543826	7.7621548
11.43323792	0.018028563	2.89151907	0.015397695	1.057344998
13.70903878	0.122637017	4.630646483	0.101149378	0.122691538
9.41682115	0.050801721	0.051007575	0.04291035	13.63861555
8.832377994	0.166226492	3.850246756	0.135174748	1.481507138
13.64251041	2.091222532	5.319975915	0.018433161	2.908032906
13.0177689	0.928723604	3.210666309	0	1.903616912
7.261590687	0.047784569	1.148276184	0.040402824	0.047802816
13.2249422	0.59260522	6.347595613	0	0
13.63331364	0.235707838	3.176797705	0.187482941	0.23583515
10.97536962	0.112459576	3.479791723	0.09306511	7.389932733
11.0812095	0.982152445	8.305540862	0	3.142575175
10.58396104	1.565695658	0.199816835	0.159946918	0.198850588
11.08505644	1.134000311	5.382800156	1.136050534	1.133949521
11.30821143	1.674954552	0.574132725	4.957048909	0
14.00315456	3.415837753	5.967782433	1.73901451	1.331000827
13.07683987	3.957636741	7.892793968	1.88140289	0.081843927
9.648735548	1.709735802	10.72116588	0.203090974	3.242238656
8.789120505	1.525177635	2.203966934	1.524520332	8.547770438
7.522201547	3.024339337	3.665698148	0.238485514	7.671916178
9.642907305	2.939015793	1.089584452	0.02424506	10.91970896
10.93969201	6.128749248	9.111813257	2.883244117	2.11409827
12.92996696	0.150861336	10.10454172	0.123290346	1.440852546
10.55391637	0	7.745441339	0	4.333192515
9.504701293	0	0.979537526	0	0
10.49573344	0.062737644	7.416720115	0.052780612	1.193562601
10.826005	0.086958281	0.0873385	1.266996691	7.848937419
8.811520794	0.040229064	1.125477121	0.034101152	4.195558447
12.95159658	0.505868372	3.608638415	0.938621402	0.949873155
9.988195078	0.99970665	8.996690637	0	1.999606767
13.47018193	1.874751293	1.013070593	0	0.551652725
13.40181772	2.941069889	3.198542459	0	0.765125572
11.84625813	1.942841959	3.757557919	0.082125951	0.098837269
8.338827375	2.506039266	10.12016993	4.783601442	5.010214585
12.18879304	1.386212947	1.384283633	0.107394886	0.130605108
8.629771024	1.359794893	7.371637343	0	1.752212685
9.411544236	0.085200244	1.895775306	0.071143985	11.51453559
11.19976477	0.021794576	4.196167214	0.018590331	7.612854087
9.61130203	0.102527295	0.102989765	0.085123408	2.399853437
9.032330657	0.066973662	1.205171401	0.056264637	7.834827575
11.18686144	1.384937122	0.130697464	0.107026709	0.130137462
13.06564608	4.610751838	2.244081633	0.050820886	0.060383868
13.30635934	0	5.180271626	0	0
9.755433386	4.522000893	4.921626754	0	0
9.567902342	0.167672699	9.39217455	2.930067341	1.485299798
9.407320889	0.198724209	3.96005302	0.159927876	0.19882531
9.906263611	0.11667962	11.17863846	0.096423762	2.790540558
9.174753056	0	2.793863067	0	0.866548357
12.30867524	1.595520813	2.011487828	0.002296819	0.002676128
12.5098845	1.159914207	5.29960489	0	0.664026255
7.972503781	1.169786115	5.823613642	0.046158241	0.054740023
11.98661797	0.021397982	2.682357551	1.667558806	0.021405681
10.34715102	0.014368016	2.385866312	0.012286623	1.641012567
10.06264133	10.5716795	0	3.453257914	4.370500678

13.29887228	0	8.524372676	2.178895407	0
7.720234976	2.605774185	1.819949474	0	5.818362517
8.671516604	2.141390467	1.895646085	3.616246535	0
9.34342834	0.675316769	0	0	1.174896073
10.77158329	1.111811714	12.46302366	5.806137358	4.173944798
12.77192358	1.383056623	7.893291294	4.602727448	1.383117784
12.87768233	0	5.375050263	0	1.86304711
10.30261515	0.009188467	8.213551715	0.007871286	11.92357436
12.74453115	0	10.21725844	0	1.965944886
13.17539493	1.206536689	3.62832903	0.056375656	7.435896227
11.70670008	1.192146973	4.821888391	0.052365795	0.062258605
11.59093374	0	2.301962327	0	5.883415816
10.51371937	5.398407393	7.364092257	3.675348842	4.060373968
13.47979733	1.055601822	8.599792063	0	1.694861291
10.5283235	0	8.609214392	2.487888314	0
7.453644575	0.057344214	1.799217387	0.04833035	0.057366569
11.32205649	0.207160978	1.583868322	0.166270654	6.508344151
11.47789468	7.36828598	2.331014328	3.331376367	1.006475273
11.86872356	0.37519359	6.575694729	0.285893943	0.375441108
11.51015683	0.00946155	2.040326913	0.008104468	1.030279361
10.89522831	0.33287576	4.36275592	0.256920809	2.613257065
8.520733155	0.014798933	0.014854195	0.012653258	0.014804176
8.439491009	0.830489109	0	0	2.076965903
12.05851024	2.366669223	11.1687556	3.381053033	1.924011205
10.12755557	1.904151463	5.85831981	3.593708256	4.343263172
13.1693086	0	3.853715801	0	5.401946937
13.40349525	2.528803509	10.75830107	3.37990796	5.939207626
13.15568512	0	2.296647842	0.981682623	8.764421486
12.14859919	0	0	0	0
9.182434887	1.215819978	7.212063545	0.058968789	0.070297903
9.925149278	3.909050107	8.143354801	4.748478251	6.774214923
10.73732032	1.449225296	0.154699702	0.125677013	9.917613942
12.80204081	0	10.18518898	5.12075282	1.18826344
7.881664364	0	4.37125813	0	1.268836068
8.196053978	2.612312193	0	0	0.515874739
11.24337677	1.09962625	0.031730982	0.026871661	9.931756962
13.69759201	0	3.271990424	0	0
13.71698154	1.590852183	2.591912395	0.001280251	0.001491072
11.40656288	1.214639698	5.583846	0.058638827	2.615564518
9.022403181	2.417249555	11.96747563	0	0
12.50817926	0	7.903617349	0	1.705015502
11.70374926	0	4.193696738	0	0
8.260131486	0.071277102	3.913054421	0.059794049	3.491628992
12.06508215	2.008882388	0.391126527	0.294765667	0.388631113
9.529450369	1.80644796	10.02398559	0.854945269	0
11.82762961	0.918083258	2.205504495	2.207584453	0
12.83488227	0	5.76886771	0	1.416892374
13.23112302	0.84249374	0.429186101	0	3.704506586
12.6423127	1.083802847	5.420147826	0.02256267	5.993496985
12.34573922	0.004825132	2.020670699	0.004139607	8.154947591
13.33741674	1.695327323	2.714333433	2.117676698	6.87482647
12.72954524	3.665560859	9.127153914	2.506499873	0.799378685
13.01340346	9.34966028	5.398357051	0	4.407674062
9.809647757	0.114521056	4.205560969	0.094706962	0.114571188
13.56486204	0	0.377377759	0	4.445993427
13.57527075	0	2.988002171	0	0.792339167
13.10863886	1.174049068	4.509033845	0	5.143374804
8.23933218	4.027442749	9.224127029	0	3.034290734

9.280003254	0.063168362	0.06343124	0.053135318	2.860003182
13.16487767	0	1.095530061	0	1.094032225
8.525810527	3.983084426	7.447395549	3.803037832	6.798317929
11.80210726	0	2.113060782	0	0.910023358
9.345517475	10.54436924	5.906944453	1.248407516	0.080547303
13.76569458	0.037105544	4.707140216	0.031486596	4.504733421
10.79234775	1.680155796	0.024726613	0.020990788	4.824873074
11.23692411	1.010297347	4.125056295	0	2.252641392
8.216946267	0.480654782	2.195106389	2.152286165	0.481016078
11.02618616	0.107915896	1.322071553	0.08943848	9.178247616
9.086135066	2.111840113	12.25178038	6.210608001	7.413226761
11.69139691	0.099933027	0.100381491	0.083040515	0.099975549
13.0458152	0.023325919	2.424673737	0.019886194	1.073918559
12.74263188	0.247582149	7.898622957	0.196194808	1.686580905
9.131967448	8.174846462	8.321714499	5.083297753	4.675324953
9.223756715	2.956916399	7.112367971	0	2.28176915
10.23858442	0.085178248	9.802703543	0.071126136	2.33750039
13.26826689	4.777807604	8.793619839	0	6.364577892
13.22565967	0	5.141896453	0	2.974158327
8.82429793	1.301410019	9.638394097	0.083101632	0.100051647
12.720545	1.449512447	4.462715606	0.125760757	5.126286951
12.01432085	0.553217066	3.287431673	0	2.414898161
12.17515964	0	4.276365213	0	0
14.18461953	0	1.413091738	0	0.585607906
9.791328814	0.17701989	2.646304431	0.143453092	7.753080751
13.89383991	0.892100279	6.259472248	0	1.451357767
10.36743044	0.119261334	6.244671844	0.098473928	0.119314017
9.506468372	0.079110104	4.455022836	0.066192302	1.876147138
11.99562795	0.020685139	10.82123845	1.066848183	0.02069257
12.90925017	0.01186965	1.631209873	0.010158822	6.817182118
11.38241348	0.716256441	4.709901446	0	0
10.56277596	0	0	0	10.974135
9.459536655	0	0.965594834	0	0
7.158465023	1.097605106	0.031072987	0.02632042	3.144780008
11.69658202	0	1.559606422	0	0.979307285
11.63535621	0.122072549	0.122644409	0.100702411	0.122126762
11.25564031	8.698407676	9.968621488	3.413792846	11.28850069
9.425004706	0.011935216	5.309834861	0.01021471	4.060214712
13.22785821	0.575947968	1.918923645	0	0
13.27427879	9.24768626	6.143973453	3.104556895	1.491129181
8.017400656	8.740135371	7.912797505	4.328523024	4.49437642
12.34987598	8.134335868	4.654882232	4.981387771	7.599483
11.97434748	0.085555165	1.89697748	0.071431942	1.898173082
13.89281193	0.916912958	1.949766443	0	0
10.13220362	0.093814896	4.12352209	0.078114252	0.093854334
12.83051279	5.426316181	9.919081526	0.04409643	3.704218
12.40186529	11.44853808	4.026321674	3.023857827	1.01661281
11.26726167	0.903671591	0	0	9.858190777
13.2604343	4.602944392	8.127100484	0	3.264340517
13.23349244	0	4.821963386	0	0.755541112
10.27847394	2.718586677	9.054390738	6.062496904	4.535590578
9.596270624	5.775622938	10.09500019	4.840596004	1.179572874
11.94780934	0	0	0	1.520820079
7.924773585	0.978948198	3.289393887	0	0
12.77024905	10.97721852	10.29187922	3.749129916	6.316482239
11.93831346	3.090007854	4.069707772	4.09909323	0.704264613
6.936546063	10.13392107	9.625366902	4.092491958	10.18070592
12.36484821	7.541340267	7.658623255	3.026472856	6.380219887

hsa-miR-372-3p	hsa-miR-216a-5p	hsa-miR-1270	hsa-miR-33b-3p	hsa-miR-183-3p
0.113626498	4.593703631	3.475601386	2.405725269	0.075318698
0.07306024	5.934889389	5.464774954	1.221074002	1.212883665
3.161642051	1.717707063	2.740733444	0.027262369	1.105581809
0.018093822	1.057156866	2.667384146	0.014243815	0.012772428
2.468384313	4.793525348	2.024812424	0.092197331	0.081166195
2.539105658	0.050976166	1.161740193	3.015883077	0.035209915
1.479344678	3.673995813	1.491568138	3.396495724	1.446916773
1.068294622	6.177605079	0.020245034	2.086755674	2.082616351
0.929006789	1.903860154	0.927010639	3.068398709	0
1.148306637	0.047947574	6.205705364	2.779702476	1.7515161
0	5.453239278	1.701214898	1.966063563	0.590883487
0.237000974	4.380143996	1.669732906	3.778290801	2.694570213
2.43335239	3.810960616	1.34376439	0.084944912	2.97947432
0.982225234	7.592245238	1.975815776	2.560648976	0.98257329
0.199776227	0.199653782	2.256258199	1.546551869	2.608922281
0.043006473	5.256895837	0.040014112	1.133525897	1.129002092
1.67671801	1.041012719	2.111380441	1.931827483	0
1.333275034	6.823986524	3.04568284	1.073242457	0
1.24826782	7.56247239	2.660828232	2.304355427	1.858475038
2.412818511	10.28135407	4.41359286	1.677524422	2.299214412
1.522567599	1.522928954	4.540658497	0.133808776	0.116690168
3.904849657	1.822370401	3.642630577	0.212518967	0.182167795
4.389470398	1.089654734	0.026663629	0.02239348	0.020040798
2.912868156	8.341711319	3.416069255	1.433980596	0.098984492
0.151578451	9.076550799	2.563838334	0.1119602	0.098119725
0.702336809	7.023261082	2.684859521	2.145724842	0
0	1.559773203	1.97214331	0.979342882	1.56121168
1.818307326	8.319926557	1.198124719	1.810017531	1.799608214
3.978260685	1.263186906	1.270222889	1.888721624	0.058867363
0.040382267	1.125577202	0.037587635	2.750660665	0.028078366
0.507350497	4.554246607	3.677506474	0.944714541	2.537344544
1.999607957	8.67546845	0.999699621	0.999705436	0
1.361366436	0.55274037	1.350363516	0	2.993738997
0.765902708	3.691560896	1.671353444	1.292846148	4.118091294
2.724337348	4.252385794	2.387507144	1.293758975	0.066376691
0.292523325	9.65494315	4.753952302	0.202962779	0.174332248
1.384370535	1.384624061	0.120388468	2.459126322	0.085976341
3.033279801	8.072797398	3.294024399	1.759239658	3.330030848
0.085557339	2.33668621	0.079087515	0.065179773	0.057742404
0.021874163	4.108413948	0.020418387	1.069185812	0.015401149
1.30708258	1.95396838	1.315399658	1.303927209	0.068720987
1.205214605	0.067211757	2.873696702	1.824082317	0.045928475
0.130673943	2.043040621	2.048634777	0.097457599	0.085693394
0.060600389	4.285996001	3.269587351	1.802066025	0.04157249
0	4.21890245	3.806904003	1.917797192	0
1.90027105	4.049741575	4.273795057	2.703685972	0
3.243452227	8.287455249	5.497861392	0.123493888	0.107945153
3.063710627	3.064047338	1.576574621	0.144347432	0.125584397
1.999939861	10.68369826	2.788243122	1.979962797	0.077511853
0	3.110733065	2.961813157	0.865073948	0
0.002684543	1.595486985	1.008812561	3.011905456	2.010519239
0	4.855672434	3.877375141	1.536338846	0
3.052090591	6.314542657	3.953550289	1.168810161	2.526942522
0.021476049	3.689670234	1.669075338	1.067950598	0.015124952
0.014419579	2.874766171	0.013475383	1.045911199	0.010204438
12.25690747	0.776718096	4.791498756	1.306957005	1.315437433

0	9.283965141	2.165865645	2.378289857	0
2.606267961	0.86792558	0	0.866071489	0
0	2.537131228	5.330658801	1.221508561	2.393644973
1.176124667	0.676279048	4.642663842	0	0
1.111328707	12.01448908	3.753105504	0.027864982	2.458988424
0	8.173639222	0	2.932421939	2.365987018
0	5.421287519	1.186929702	2.528467355	1.20552759
2.039181047	8.048621024	1.030125028	0.007291051	0.00654891
0	10.3690403	2.76971311	0.974533707	1.555661964
1.205610516	4.012460969	2.874234984	3.595564658	0.046017121
1.191290008	4.618886655	3.603467066	0.048131034	0.042810552
0	2.301950868	1.980780208	3.301949646	0
1.977353708	7.381169309	3.780993333	0.983003969	0.983488988
1.05725899	8.63835531	1.401954093	0	0
0	8.118413117	4.061488134	1.911451588	0
0.057570943	0.057543936	1.181755698	1.791918571	0.039573509
3.37199149	0.208116823	1.59815085	1.566132022	0.130267081
4.59542224	4.469860835	3.179713565	4.653068708	0.001441451
0.377710801	5.5168803	0.334147385	0.252858752	0.214914958
2.852001535	1.621939167	1.622600073	0.007506735	0.006742287
3.450241894	3.090863715	2.610219653	1.83292069	0.19502101
1.642471192	0.014845765	2.063387243	2.059961679	2.057147351
1.37461783	1.768403022	4.474415295	0.828407191	0
1.922776429	10.89766532	3.203636013	2.342068933	0.062841648
11.80680234	5.496176419	0	0.92858475	1.502242938
0.858536241	2.947218191	2.116385986	1.812303611	0
1.149281061	11.2569363	1.769270854	1.760417942	1.144120234
0	3.294179837	4.363942288	0.981950286	1.564217364
0.871773785	0	3.119786381	0.869927006	3.406663731
1.214848809	7.589953525	1.848719387	1.213946639	1.20607579
1.659957283	9.174038213	0.216066883	0.169878928	2.25820981
0.154670614	2.117352025	3.421511803	0.114081869	0.099930918
0	10.44962638	2.937554606	0.680034128	2.858345169
0.748860784	3.961057666	2.600443792	2.790339062	0
2.344541848	0	1.571759079	0	0
0.03172632	0.03171226	1.101983754	0.024808113	0.022188862
0	3.120459783	2.537876285	2.540877302	0
0.001495747	3.914486681	2.006483122	2.32829688	0.00106799
2.61475428	5.46469584	1.219584235	3.086639268	1.821927884
0.887200226	10.54472397	4.67332714	0	2.654939965
0	8.284513987	2.007997796	0.782915547	0
0.966541122	1.954898851	0	1.95660283	0.967151979
1.84811808	4.239238407	3.314216501	1.838238088	1.826296647
0.391021477	0.390704815	3.565568143	1.937373545	0.220933816
3.470869443	9.476069073	0.854799485	2.963644704	0
0	1.484037701	4.116719542	1.485553972	0
1.815913432	5.813168537	1.414063836	1.821272304	1.424865092
0.429113992	1.429920681	3.557334039	0.833911113	0.846663872
0.026591607	5.134191904	1.688586656	1.684044852	1.08106818
1.603895137	2.343653043	1.015869811	1.603484544	0.00344931
0.028772348	4.047737335	2.714285117	0.022531003	1.08752637
2.031190264	8.876336548	2.282029563	1.336799776	0
4.609023758	5.935953951	2.730930208	2.266699486	1.146695486
1.340473366	4.205716442	3.283138041	1.973521824	1.32232545
0	1.554001291	2.050950101	0	3.60860204
0	3.46389975	1.320943295	2.031594505	0
1.545815858	4.330607361	3.152494258	2.502752568	0
5.525685756	8.77020892	4.66605664	1.478907975	0

0.063421251	1.194160354	1.823603505	2.240404739	0.043426685
1.095457597	2.92363462	0.604861653	0	1.775157624
5.868956278	6.277034095	3.075161825	1.991205328	0.079840016
1.244964838	1.939608967	2.532577835	1.759738959	2.172748968
1.244538817	5.740257755	3.678170033	1.868181229	1.854558932
0.037245867	4.707174322	2.975876677	2.145961063	0.025952273
2.103307664	0.024712256	4.442226821	1.677287175	0.017373235
1.87534578	4.653444203	0.999709649	0	1.673171098
3.448144471	2.954993473	3.418172119	2.096334864	0.260932434
1.9714596	0.108332747	2.757031494	1.953636663	0.072086554
1.687307292	11.71319706	5.130702294	2.696826681	0.018759425
0.100364444	4.624619811	0.092565619	2.965026819	1.28504644
1.073631592	2.424733653	2.690894414	0.018381498	0.016466225
0.24896597	7.848845272	2.392143112	0.176030646	0.152082958
1.056295971	8.041202191	3.083746753	3.886082923	1.054830063
2.765066253	7.49934586	0.970915225	3.281776057	1.552084855
1.257930401	9.007842754	1.265000753	2.315610535	1.246311926
0.642709202	8.358252614	4.177669923	2.892615233	0
0	4.43179818	2.560096982	0	0.983329139
0.100440897	9.68268565	4.611602772	0.075979504	3.949285712
0.154779228	4.235256774	2.915765076	1.438282493	0.099994413
0	3.203027699	3.021552162	3.165478	0.550431469
1.204902462	1.875706501	1.572364402	3.8443648	1.599661123
0	4.629156399	1.92881356	0.577039794	2.685769838
2.6464199	3.710841813	2.192803423	1.494833748	0.113323224
0.892520959	5.839468719	2.166691409	1.858420406	0.893835233
0.119795913	7.466546149	1.362990036	1.987629724	2.400342314
0.079437564	3.523373081	1.87955064	4.226644419	3.756188103
0.020760481	10.30502264	3.097141168	1.662857178	0.01462814
0.011911995	3.966522971	0.011136299	1.0380123	0.008444406
0	3.968632491	6.755940818	1.61313252	2.186770042
1.89362213	0.9214425	1.486014426	0.920530363	0.922626428
0	0	1.541772003	2.951629294	3.275810791
0.031068429	1.097242967	0.028959766	0.02430154	0.021738419
1.559601964	0	2.292586993	2.778751684	0
0.122622668	2.466691417	0.112711819	2.433404381	0.080821101
1.95320034	9.835918684	4.14707253	0.077678652	0.068614706
2.375197861	4.646140066	0.011197707	0.009457832	0.008490673
1.045540197	1.918601235	0	0.56721249	2.171683766
1.783174497	5.995451882	4.449507329	1.498512482	1.138013493
0.035034888	7.762956123	2.741225221	2.13769304	0.024448111
8.361339936	5.041953075	3.497961134	0.950216519	2.39850023
2.947960704	1.259172184	1.266107516	0.065440361	3.12120969
1.253116856	3.136729648	1.743071004	1.253803232	0
0.094214997	3.737963711	1.290228169	1.28009264	2.325090689
1.161620196	9.644456191	2.814007076	0.040588414	1.155844667
6.114808033	4.727288003	5.485726604	1.016672482	0.003698599
0.904049434	2.443038204	0.901356993	1.467798563	0
3.538180722	7.986012205	1.548201801	0.675581418	0
0	5.271650623	1.961254883	3.531237805	0
4.988517818	8.152551808	7.180929197	2.659061353	1.75168627
1.801662432	9.51870993	4.269184363	1.79420613	1.172161011
0	4.003525815	1.9291381	0.94759983	0
0.979033924	1.559157116	1.558680096	0	3.293953134
5.825147192	10.50542802	3.834603664	1.459187122	2.668765094
1.886231362	3.738121572	2.83721317	2.150319111	1.223861597
5.962256083	9.40674974	3.189187544	2.48898016	1.124768056
3.149341754	8.143857645	2.295862028	2.311231274	0.809970587

hsa-miR-6502-5p	hsa-miR-301b-3p	hsa-miR-10a-3p	hsa-miR-196a-5p	hsa-miR-520a-3p
2.677149211	1.343994105	2.764138454	1.339202623	1.337318144
0.04137516	1.227581593	1.853890985	0.072525325	0.072939423
1.699326702	1.111862302	3.156214209	3.487410707	0.034866533
0.010773043	2.076046929	1.655408058	2.076549124	1.057230874
3.192883999	1.370514174	3.296862303	1.365619784	0.122965343
1.142435413	1.161432998	2.535133975	1.777105658	1.157657009
1.413131735	1.487934469	1.486771527	3.241087185	1.480400436
1.062628509	3.422615402	2.905109963	1.06877294	1.068423078
0.933618899	2.893494825	2.481952621	2.220305619	0.928882158
0.02778346	7.523928029	0.041841405	9.21620212	1.148595675
1.775722766	2.773928399	1.416390688	9.109232584	0
0.117465998	0.199142241	3.139158649	1.659997947	0.23647491
0.061799703	1.986623221	3.261443396	0.11206202	0.11275485
1.979668822	3.432546807	1.562856834	10.27858692	0
0.101870227	2.244440892	0.167196084	3.961952497	0.199358616
0.025021731	2.504175267	1.748095939	1.134374825	1.747433574
1.445434679	2.856645013	3.505178738	7.710508463	1.392872253
2.327079822	1.73151962	4.002341143	0.372153908	5.530848295
1.221707488	0.071767483	2.653012482	2.325900724	4.703456048
0.126092227	3.478553533	0.21300759	0.255882541	0.257909322
0.094922846	1.531227012	1.529871392	2.208150626	0.183470775
0.145114679	1.826379305	0.250816139	0.305015338	2.54455266
0.016858782	1.092036191	2.11832759	1.694918391	1.69443225
0.080997066	3.170041487	3.395946362	1.446296721	7.388794846
0.080312832	2.552681303	4.055754185	2.910582325	2.563522228
0.713628067	1.880076841	2.342849223	0.700609455	0
0	1.972422362	0.97896006	0	1.972427389
0.035992029	2.251438634	2.850114942	2.588034832	0.062886592
0.048838395	1.269261071	3.691290754	1.264999431	0.087175372
1.716187951	0.035659928	1.128688184	1.126401705	1.125744824
0.516486541	1.557722312	3.366545696	3.544975513	0.506704203
0	2.321519295	2.806930469	0	0
0.563051578	1.635834175	3.534696367	6.814767828	2.554617488
0.776468091	3.321895373	3.209496112	0.764489862	1.291215644
0.054924211	3.411157232	0.085328617	1.298850772	0.099047884
0.139211185	1.791090309	1.788216398	0.289406395	5.195560187
1.96308419	2.487712985	2.042160701	1.387370641	0.130900943
0	1.7511338	2.061881356	7.680178166	0
1.230054568	2.666902048	1.896107283	1.259930566	1.258519104
1.657973436	2.091521575	3.099638086	4.69520506	0.021841823
1.892497741	4.017654805	2.727355188	1.309424867	0.102791332
1.795470381	1.210343928	2.266227008	1.83471226	0.067134099
1.335147052	3.325165349	1.39024245	1.386090654	2.494427825
0.034701455	2.573382157	3.066821297	4.457607947	4.875457987
2.246905354	2.714191065	3.075142718	1.506746715	0
0.931107329	3.209560716	2.217741225	0	3.342870868
1.41613904	0.1440111	2.153499586	2.16245919	1.484183245
0.10185927	2.244370485	0.167175932	0.197905105	0.199333252
0.063891584	2.000111377	1.353276041	2.791391827	1.34716289
1.436730725	2.797502213	2.131591784	1.41887762	0
1.594184114	0.002394001	2.597191584	0.002667735	2.597434872
1.191833051	3.24654945	2.46391134	2.064858302	0
0.031617252	0.0483277	1.790833271	2.555971279	0.054846508
2.399196237	1.668458158	2.904183644	1.66810066	0.021444327
2.051249064	3.527412424	1.641324331	2.061246934	1.045729658
0.787184163	5.330881442	0	1.690234282	10.7974773

1.490087544	2.661312365	3.322102779	0.602193243	0
0	2.390245313	2.133028169	0	1.420804143
0.722465902	1.214094951	2.991089567	2.140709043	1.596911635
0	3.456956338	0.666581885	1.544396577	0
1.101459031	1.114311676	2.146521904	1.112121902	2.476727965
0.847055069	0.833516594	4.133644389	1.777508791	0
1.921296242	1.187332006	2.955380806	0.689321606	0
0.005536413	2.626747011	3.213042789	1.62108089	0.009207804
0.976560766	0.974048464	4.604950937	1.965868655	0
1.184793341	1.21074803	2.26672465	2.876397147	0.067269647
0.035719133	1.196126346	3.074244676	1.192693982	1.191669591
1.56967075	0.985461782	3.148910371	1.980902354	0.985841336
1.566727387	2.298288094	1.564018908	1.977234344	4.830362342
2.415233191	1.691582059	2.48351642	0.583889657	0
1.510634641	1.50003995	1.500182538	3.064492678	0
1.159613116	0.050613137	0.050082336	2.233861321	0.057478797
1.495169999	2.721009291	2.264799883	2.743139302	0.207804822
2.329440721	1.006634093	1.592963242	3.817579373	7.510593624
1.7774123	2.6800062	1.969556786	1.983424027	0.376685931
0.005699486	3.214510722	2.040209772	0.00943469	0.009481475
2.363544001	0.274826104	2.579018574	2.615984256	2.61126102
0.008869958	2.062665856	1.048264177	2.388068598	0.014830494
0	0	0.826103635	1.767317103	0
1.249517439	1.287332325	2.360168526	3.208725027	5.434508692
0	4.28174527	0	0.928951401	0
0.86648528	2.1185634	2.376506951	2.590652606	0
0.027963962	0.042560562	4.124941866	7.862305023	3.812740633
1.56559289	3.432378816	2.296898931	4.92481491	0
0.879187605	0.867952439	3.260761398	8.123545869	1.425555524
1.19282969	4.311333794	2.278320094	0.070041412	1.215279238
0.11837791	1.667801983	1.665728043	0.236888051	1.661490709
0.081745472	0.132656076	2.559733986	0.153347299	1.448015017
0.698017664	4.112913459	2.316379792	1.855190046	0.686196028
0.75970196	1.952778316	2.420130552	2.763809454	0
0	3.143096782	0	0.514766131	2.840180953
0.018651202	0.028079199	2.457754068	1.099901482	1.706068781
0	0.966961152	4.904470781	0.967673177	0
1.590109838	3.177031632	3.914129871	1.004812247	3.17724175
1.191809243	2.609788182	3.471639009	1.21525487	0.07003556
0	2.160678607	0.883904232	0	2.159964468
0	4.110586218	3.824437828	0.785418165	0
1.961550491	3.53394377	3.119941682	0.966316896	0
1.195379198	3.632489791	1.222850807	2.893134762	0.071449396
0.173939931	2.708718605	0.311353065	0.38626231	2.746274491
1.427178419	0.854519766	0	0	0.858504252
0	0.916007864	2.877764528	1.483493104	0
2.157369793	2.128260307	2.795255268	2.95724528	0
0	3.329709814	3.578614607	10.94631552	0.428419468
0.015707281	0.02356618	3.581972136	7.439961427	0.026551864
0.002919371	2.829793237	3.482475071	1.015540423	0.00483518
1.082410029	2.936355872	3.129805363	2.714885471	1.69509583
2.072503916	3.58480587	3.381678143	1.333809329	4.824925533
1.16785126	5.059452726	2.594131853	0.644372305	8.226133743
0.062823413	1.993224533	2.43290808	1.995584169	0.114822939
0.38270112	2.992389591	4.813918689	1.550319419	0
2.732040353	2.490776294	2.99619002	0.791768763	0
0	2.085247647	3.625712572	1.173184618	0.675382432
0.918500563	2.678017483	0	2.456024076	4.610217255

0.03622526	3.865203253	2.851794432	7.768731092	0.06331847
0	4.136534409	3.919139323	0.612829914	0
0.065758026	1.364501429	1.363800536	2.463461093	5.755745982
0	1.5065906	2.115815136	4.664785452	0.477420555
1.218500221	1.881171298	0.069880774	1.881766833	10.84607151
2.127653692	2.480881127	3.754605199	2.154431551	0.037188844
1.071143112	2.103092456	1.680542773	0.024560045	1.077831095
1.043821896	2.924909781	1.35138036	11.52979834	0.551623672
0.203055709	2.18159185	2.888868859	0.477735004	5.045778623
1.284469264	1.971988959	1.970750916	0.107537726	0.108196755
0.015788232	0.023689866	0.023455722	1.687937125	3.125110565
1.885712698	2.991132913	3.585650374	0.099588237	0.10018907
0.013869845	1.675798097	2.688634543	4.286470438	0.023376666
0.122299402	0.208580315	1.688650893	1.689056154	0.248402976
0.010617513	0.01583587	1.654393396	1.056688313	8.201333822
0	0.970896635	0.970916218	2.764907547	4.538722773
1.2300006	2.93706665	2.331976195	2.675123468	0.085390128
1.545404507	2.865608225	2.587571786	1.129616217	5.454598117
2.564729497	2.782459056	3.559266058	1.977013816	0.982964347
1.26582251	2.385254899	2.989406853	3.903780491	1.946091053
1.387461122	0.132745603	1.454741231	0.153454728	1.448301299
0.564768677	2.929670467	1.639699325	1.876613041	0
0	0.689390306	1.575028844	4.224048404	0
0	2.321711091	4.360952977	8.258381231	0
0.09228684	4.271348318	2.18047613	2.649892105	0.177542788
2.192016312	0	1.853475754	1.853084433	1.451582215
0.065164145	2.450548457	0.102444966	0.118834233	0.11957858
1.215014987	4.152721183	3.13960099	7.390672759	0.079304463
0.012329875	1.06722007	2.086718399	0.020624851	0.020729866
1.034792715	0.010597188	2.638538029	3.05692121	0.011894789
0.728448446	0.708651429	3.441699759	3.832656513	1.22857433
0	2.882000246	2.2107572	1.487345053	0
0	1.953529117	4.034617504	0	0
0.018275567	2.128589608	0.027227462	1.097874566	2.949804131
0.980855967	1.559267749	2.293062853	0	0.979347069
0.066543672	1.368949657	1.368234282	2.468973458	0.122399014
0.056731956	1.313562567	2.726759977	0.102002886	9.949853149
0.007172831	2.639036087	2.050568985	0.011901134	0.011960498
0	2.610069713	3.584423717	2.297993901	0
0.651056639	3.388084713	2.235794428	2.411039692	8.412367546
0.020533402	2.144338751	1.112100289	0.034798469	8.774322781
1.349823739	3.702023663	2.339645752	9.027470736	7.262040283
0.048108815	3.534162579	1.897297631	4.922535838	1.2595373
0.491784335	2.553541915	3.583884337	0.915630062	0
0.052378545	1.289101591	0.081135003	0.093495103	1.283109053
1.146209396	1.782467973	3.034216026	1.162793781	7.498385095
2.018740017	1.605506196	1.605465289	1.60538432	11.69245796
0.909981805	2.855196885	2.44498532	2.851815815	0
1.90862005	1.550194014	3.280461218	1.553124507	5.615358123
1.7012544	1.659400427	3.069508393	0	0.755958585
0.916166908	3.399601649	0.443913044	1.212267865	1.214868504
0.033427082	4.067819345	1.183172147	2.236469277	7.966399857
0.951657271	1.520134004	2.24891262	0	0
0	4.874344423	2.776753002	9.808798778	1.559127677
1.47114046	1.455555472	2.434632693	0	10.35090211
0.716481602	2.696625396	4.96217226	10.07873551	3.737910644
0.024198114	3.997408345	0.036293783	0.041250605	10.87210197
0.818435822	1.34216225	2.872654253	0	6.720595485

hsa-miR-519a-5p	hsa-miR-502-5p	hsa-miR-520c-3p	hsa-miR-520b-3p	hsa-miR-34a-3p
1.337673112	3.46986842	0.109302592	0.109299642	2.427536851
0.072871921	2.29102329	0.070488407	0.070486646	0.063018292
1.109253187	2.472763713	0.033784467	0.033783688	2.14324719
1.057285054	3.787060657	0.017527126	0.017526737	3.25329164
0.122839707	2.466620643	0.118419774	0.118416521	2.459030115
0.050874301	2.538069924	0.04928381	0.049282634	2.534800308
0.166523943	2.156808476	0.160057338	0.160052594	3.209465263
0.021637866	2.682570311	0.02100305	0.02100258	2.904943827
0.928818755	3.21247576	0	0	2.702707229
1.148741786	3.678167382	0.046365884	0.046364784	1.765817787
1.06655188	3.20175442	0	0	2.155325593
0.236181271	3.434348997	0.225939607	0.225932132	1.659571947
0.112641866	1.989732934	0.108663992	0.108661063	3.629771242
0.982176866	3.294865354	0	0	2.297086104
0.199125462	4.225318257	0.190970142	0.190964174	1.569255127
0.042902516	2.505911251	0.041583745	0.041582769	3.36126536
2.293482628	2.850857945	0	0	2.119573115
2.966861664	1.537353548	2.180719667	2.0447336	1.903800056
4.842210557	1.887605651	1.253624816	1.253626624	2.319521966
0.257578279	1.718659988	0.246051191	0.246042791	0.211833232
1.524316855	2.658645042	0.175945442	0.175940082	2.196317705
0.307189913	1.833795447	0.292455507	0.292444811	3.319799515
2.71381469	2.119569884	0.027677405	0.027676776	3.299712403
7.569419923	3.408486163	4.224989178	4.224982553	2.553734548
0.151124014	3.403596099	0.145407204	0.145403005	3.760345062
0	1.20173545	0	0	1.202778881
0.979478036	3.553463815	0	0	1.559521327
0.062829607	3.276467482	0.060815988	0.0608145	2.250206183
0.08709231	2.342742865	0.084162433	0.084160271	1.901614283
0.040285217	1.739764772	0.039053838	0.039052926	2.492696103
0.950344596	0.49369554	0	0	3.126338355
0.999707055	2.999564141	0.999701835	0.999701833	2.999571388
1.011818411	3.119593867	0.545337959	0.545334663	3.386806685
2.793501053	2.444195738	0.761446107	0.761444279	3.52819107
0.098951248	4.235168236	0.095545531	0.09554302	0.084952716
3.623601391	3.817684759	2.98465216	2.984647464	1.787052541
0.130765196	2.831624488	0.125992357	0.125988847	2.821917583
0	4.790120436	0	0	3.620487761
0.085331097	2.941449223	0.082470321	0.08246821	3.16183836
0.02182375	3.272073004	0.021183214	0.021182739	3.560721735
0.102690303	2.39783265	0.09913075	0.099128127	4.013954052
0.067072718	3.09785393	1.20961851	1.20962003	1.209924975
0.130296838	2.491436273	1.391458643	1.391461246	3.092653759
5.502931664	3.438824733	0.058520376	0.05851895	2.241364544
0	4.070432164	0	0	2.233117685
3.342825062	1.899349489	0	0	1.492844753
2.160318314	2.161109569	1.493680446	1.493683507	3.443354501
2.715322946	2.251237131	0.190946183	0.190940216	1.569191992
0.11687029	1.355285233	0.112710765	0.112707703	3.79883232
0	2.795610286	0	0	1.81809243
2.011515434	3.182805311	0.002603395	0.002603339	2.597173467
0	3.136585331	0	0	2.628292163
0.054797655	1.792586073	0.053070373	0.053069095	2.220199054
0.021426598	2.681643605	0.02079828	0.020797814	2.414945049
0.014386918	3.237963302	0.013971713	0.013971405	2.872899495
10.80687964	3.09948602	8.742876552	8.724764775	1.996858312

0.603486566	1.430446595	0	0	2.79669384
1.819531351	3.115357785	0.865379509	0.865378471	2.968117014
0.710555233	2.851019226	0	0	2.358273258
0	1.836786923	0	0	2.299490951
1.720872306	2.476011706	0.034542574	0.034541776	2.473801828
0	2.749901262	0	0	0.833600401
0	3.289311339	0	0	3.078896465
0.009200407	1.621443481	0.008938033	0.008937838	2.362511193
0	2.286192796	0	0	1.553588727
0.067208125	3.294144575	0.065034852	0.065033247	1.83408991
2.251823574	3.274419224	0.060329828	0.060328353	0.054056904
0	2.564444704	0	0	1.56740675
5.557050537	3.1444706	3.880074241	3.880074424	2.975194635
1.410772753	3.790263678	1.048984415	1.048981269	1.693008116
0	1.499742842	0	0	2.226267846
0.057427309	2.233131537	0.055607212	0.055605866	2.230081854
0.207558381	3.582157931	0.198943857	0.198937556	2.715824977
6.098143059	0.001847955	2.816995765	3.009781701	2.816698458
0.376114369	1.984474179	0.356398836	0.3563846	1.967816091
0.009473854	5.506485288	0.00920351	0.00920331	1.622242237
1.88474053	0.286812934	0.317073098	0.31706109	2.57680852
0.014818421	2.387682818	0.014390341	0.014390023	3.23867796
0.830704403	2.076346286	0	0	3.319601287
3.735909105	2.365031426	1.286867364	1.286869379	2.359610081
0	3.590038962	0.927989779	0.927989225	2.894703624
0	2.374279541	0	0	2.376765339
1.149719247	1.153487654	1.76913271	1.152458386	1.152912518
0.982065992	3.557453219	0	0	3.557981356
0	3.881700072	0	0	1.824741292
0.070374401	0.063663041	0.068084113	0.068082421	2.277918299
3.185340248	0.208711012	2.37110601	2.371105946	0.197072795
1.448502881	3.183778336	0.148340014	0.148335708	3.597060863
1.560759016	2.941110429	0	0	1.85656588
0	1.647161842	0	0	2.773478362
6.815683914	4.536786537	0.509212727	0.509209224	2.014636533
1.706172	2.459498941	1.101289416	1.101290176	2.130638781
0.967801747	1.544735401	0	0	1.544992179
1.00479281	3.177113877	1.004878307	1.004878344	2.006390292
0.069971136	3.305205202	0.067695824	0.067694144	2.276448492
2.993185584	3.282671887	0	0	2.161311438
0	2.478104949	0	0	0
0	3.119166296	0	0	2.275024284
0.071383478	1.851218272	0.069055622	0.069053902	4.483252524
0.389340108	3.544793555	2.023768894	2.023771622	3.161891609
0	2.117566972	0	0	1.406902133
0	2.464922409	0	0	3.197945572
0	3.691013497	0	0	2.386728221
0.428061551	3.252617387	0.420286863	0.420282922	2.441909256
1.08368316	3.584262569	0.025742768	0.025742185	2.110166651
0.004831337	2.020816668	0.004694952	0.004694851	3.022562288
2.119987853	3.131964842	0.027849458	0.027848824	2.118891518
3.134012617	3.003581641	2.683875971	2.68387638	3.488106085
8.514050204	3.394516488	7.454750519	7.44948408	3.920591714
0.114707425	4.069352322	0.110641107	0.110638113	1.991347847
0	3.05840607	0	0	1.908925792
0	2.272428158	0	0	2.847169428
0	3.903404201	0	0	1.839370796
4.027483491	2.676837882	1.475627531	1.475626991	1.476015552

0.063261041	2.586901538	0.061231803	0.061230303	4.18665776
0	3.04190357	0	0	3.587415179
4.337958863	1.365920202	2.806100699	2.806099409	2.451906156
0	0.463831254	0	0	2.274093788
11.12381675	4.056192192	7.71860494	7.727477017	2.917414128
0.037156978	2.1542327	0.036028869	0.036028034	2.152372727
0.024665684	1.079924551	0.023937115	0.023936575	1.680455125
0	2.25303214	0	0	2.564642577
2.200089962	2.197790311	2.218278318	2.218279344	0.374097914
0.108089288	2.416519812	0.104304387	0.104301599	3.244452709
2.930625447	3.293930455	0.025878558	0.025877971	1.687995917
0.100091101	2.388767482	0.09663868	0.096636135	3.877720154
0.023357254	3.279020613	0.022669347	0.022668837	2.097361975
0.248088747	2.844584031	0.237139026	0.237131042	1.687749068
7.840915815	3.544163383	5.614098628	5.614097507	2.888007452
1.550056687	1.961603664	0	0	0.970925418
0.085309061	2.336337244	0.082449147	0.082447037	1.895661652
4.674602948	4.077963522	2.414049291	2.575585135	2.588263966
0	4.221350354	0	0	2.78259531
1.300945324	4.683317895	0.096711755	0.096709207	4.004723257
0.154312533	2.569144421	0.148443006	0.148438696	2.897793121
0	4.440833726	0	0	2.821585785
0	1.872017726	0	0	2.687218894
0	1.93063421	0	0	2.763322263
1.509058801	2.982758932	0.170330921	0.170325781	2.628654697
0	2.167402315	0.890374157	0.890373312	2.838211959
0.119457186	2.455252437	0.115185381	0.115182236	2.784196063
0.079230101	2.314329962	0.076605567	0.076603629	3.138944367
1.065602903	2.087596668	0.020106346	0.020105897	3.555721702
0.011885173	1.631958201	0.011544129	0.011543876	1.631608804
1.228307373	2.99579607	0	0	3.344283873
0	4.045397407	0	0	2.882646051
0	2.273099604	0	0	1.953697194
1.097464946	2.723123252	1.099234079	1.099234823	3.139463224
0	1.972048776	0	0	2.969609814
1.362407859	4.422064655	0.117879031	0.117875796	2.015166825
9.859283557	1.956666226	7.141143759	7.1691617	2.996988043
0.011950827	2.051059996	0.011607848	0.011607594	4.580947375
0	2.968606885	0	0	2.125413327
8.281551379	1.484670425	6.753029795	6.769897649	2.419948737
6.480851341	3.330634303	7.093226785	7.128562505	2.471075359
5.731023386	2.475402557	5.575934957	5.575943147	1.800845286
1.2598033	2.337695492	0.082811964	0.082809842	1.896923756
0	2.547674084	0.474910032	0.474906348	2.786485504
0.093961501	1.290023683	0.090758235	0.090755872	3.389784248
5.649443694	3.233208985	3.042598506	3.042597658	0.045494432
11.83651441	2.022317184	9.224212974	9.219288727	2.344907175
1.465908136	3.549388778	0.90212568	0.902124926	1.869567477
2.794956498	2.488899951	2.30068993	2.300689686	2.097627494
0	2.615356933	0	0	2.217802625
2.611327077	2.725633654	1.473719119	1.473715974	1.904432939
8.617921726	3.429174549	3.97212033	3.97211816	3.252129948
0	1.929393919	0	0	2.249002296
0	4.426525272	0	0	0
9.999691643	1.455145955	7.763412331	7.751589407	3.013799377
3.090274382	2.692794478	0.699747792	0.699745513	2.69876244
10.15328643	2.767267029	8.349549042	8.354634293	2.497319692
6.631291039	2.297274095	5.098869919	5.098873077	2.045351221

hsa-miR-2114-5p	hsa-miR-34b-3p	hsa-miR-643	hsa-miR-130b-3p	hsa-miR-130a-5p
0.106035449	1.332609974	0.076770677	6.94786283	2.395535476
0.06853447	0.0560659	3.44710728	6.305014717	1.837807378
6.785521501	2.463739148	1.106168161	6.220605377	1.107681407
0.01709344	0.014260713	1.650701306	6.047586683	1.651866728
1.372328622	0.092326477	0.082749388	4.793730392	1.988180093
5.372231218	0.039554445	0.035833093	5.74264799	1.154961196
2.61510615	0.122690171	1.450282691	5.670811748	1.459204396
0.020479243	1.66628124	1.066796974	7.52147358	1.067707949
1.495568759	1.49857991	0.92976325	6.479802742	1.908892448
0.04514338	0.037258989	2.177468703	10.01766115	0.035385127
0	0.584312331	1.985011007	7.51302031	2.682293237
0.217722753	0.168723819	0.148982207	6.659759914	2.277679316
3.048369546	0.085062225	0.076349625	7.134001631	1.958794893
1.562685946	0	1.564214982	6.413284702	1.977461635
0.184394558	1.546833452	0.128263132	6.081257477	0.135729629
3.902815468	0.033484624	2.160198886	5.705548081	0.031813913
0	4.53334236	1.412164641	6.962909386	1.697306557
2.044808721	0	1.088454023	6.050529653	1.769840615
1.254893396	0.062769246	1.86061816	6.883119113	2.297141123
0.236829715	0.182312527	3.084405092	7.538601439	0.170471077
7.478424975	2.165374199	0.119126162	6.481885427	1.498579312
0.280747467	0.212884731	0.18641909	7.555148804	0.198433241
7.929014052	1.691300904	0.020383385	6.927451624	0.021328153
1.45420473	1.43418182	1.417567654	8.904491792	0.106562566
1.450227349	2.078662229	1.414071769	6.308919199	2.505083935
1.57796011	1.209695053	0.70094901	6.560231479	0.698462166
1.559306612	1.973513504	2.972690042	6.693701814	2.29507377
2.255648779	1.192301396	0.043919878	6.087228327	2.233447353
3.705859098	0.066558147	1.252646152	6.366362192	0.063007023
1.128874932	2.157692761	1.727543816	6.22462719	3.335206581
2.710102313	2.504059149	0.955313035	5.856148388	2.211534178
0	1.999623488	1.999638023	6.208968579	1.999631009
1.632538405	3.99975537	1.018497256	3.765089795	3.355291724
1.285735347	0	0.765714097	6.985584643	0
1.304465528	1.293876769	0.067633087	5.54561113	1.288805647
0.266848376	1.749072186	1.71289301	4.797502266	0.189691324
5.609883461	0.097925237	0.087669428	6.288569975	0.092385205
0	0.81701693	2.337930482	6.241320295	0
8.31548345	0.06526627	1.247938769	6.335349307	1.25203292
5.318064543	0.017208117	1.664045717	6.134728138	0.016379225
5.885943695	0.077907119	1.293682781	8.187346271	0.073659187
2.604684791	1.204552442	0.046760867	6.434061144	0.049065344
5.563622004	2.019574513	1.999865816	7.60101361	1.370240336
1.190742925	1.185387108	0.04231877	6.128164612	2.225291978
1.505703424	0.936275097	1.510803565	5.252299849	1.509632562
2.215989086	0	0.927117021	8.178540146	0.926278058
0.15614067	1.472292793	1.453642843	7.538057185	2.111095101
3.061356257	0.144569904	2.94914137	8.426395006	0.135714132
0.109320309	0.088082553	0.079012738	9.557576147	0.083187821
0.863527227	0.865043476	0.867821876	6.443726658	1.423972033
2.01163406	1.008640422	1.594917023	6.643582054	2.01080412
3.018385529	1.160231401	2.316299567	7.52029158	2.307507835
4.843990345	1.78312346	0.03850039	6.71354059	0.040362498
2.417046987	2.085976188	1.066158492	6.754614885	1.664036719
6.687402891	0.011385486	3.059697317	7.629909054	1.638528508
3.43779597	0	0	8.917227725	0

1.071168452	0.595067469	1.458344941	6.852785743	1.993302954
3.785223162	0.866041193	1.831117418	6.860115411	1.425182914
0	0	1.921477036	5.76747894	0
0	0.669426975	2.334840493	7.608506077	1.559314331
2.744588697	0.027899095	1.711778477	8.032857121	2.137200131
3.199890209	0	0.839020933	6.461258749	0
0.683389705	0.684547682	1.20377978	5.817185381	2.703664011
4.631319099	0.007299559	1.028752721	7.103753825	1.619444004
0.974102015	0	0	6.24327485	0.974829576
2.272249449	0.051847598	2.242842316	6.392143769	1.201720894
0.05869185	1.190839239	1.799621793	6.697078856	1.187885924
3.300134738	0	0.986080447	5.334207634	2.303413434
4.365453018	2.97642698	0.9834087	6.398170927	0
1.927086216	0	1.725540037	5.748775775	2.520671112
0	0.931409221	1.914635409	7.142515032	0.932177232
7.130857939	0.044509379	1.783937275	5.772599466	0.042236949
5.634170033	0.15014852	1.542353353	6.220304571	2.210320834
0.001911829	1.006503898	1.006342672	6.502558122	0.001529822
0.340880551	0.253320331	0.22018798	5.516650782	1.888019821
3.747217814	0.0075155	1.029599251	5.40687591	2.03784147
4.488844853	0.228677355	0.199664504	4.364276426	0.212812202
0.014036814	1.047284074	1.046053074	5.98387399	1.046666331
0	0.828370739	2.763699927	5.54141157	3.214164033
1.928165483	0.071128358	1.269239732	6.743171781	2.664555986
1.496087574	1.499083591	2.49120398	7.524044326	1.50043881
3.0974534	2.126609381	1.818592448	6.326150566	2.389182746
2.199028225	0.037506225	1.14495247	6.699406067	2.182469188
1.562547146	0	1.564085475	6.509299897	0.982162087
3.119087789	0	0.87259417	6.084001507	0
1.220599632	0.054208577	0.048961104	9.347879076	2.58813668
0.219719528	1.636125778	2.706901861	8.651597191	1.621284861
4.222041689	1.438215994	0.101952748	6.535462889	2.074770752
2.936110464	0	0.684930125	8.775350696	0.682376747
6.598191221	0	1.668926644	6.123652229	2.226014387
6.941453936	8.626715849	2.55126204	6.229485578	2.392026585
7.556770751	0.024838273	0.022570074	5.80413077	0.023621698
2.95120151	0.967561791	0	5.406101231	3.277459088
2.006491083	2.591466883	1.004700131	6.878312557	1.590611549
2.282651845	0.05390827	1.823741083	7.373104098	1.828556739
1.844346862	0.885625584	2.173229617	7.581163806	1.448886899
0	0	0	7.837631076	2.027546761
3.407288129	0	1.958211918	8.554687762	1.545153903
3.315669466	0.054959523	1.210114162	7.908558514	0.052093486
0.352303351	0.26082361	0.226402614	5.975670011	1.911119853
1.804296221	0	1.818866602	6.601832813	0
3.776167003	1.893057189	0	5.354664023	1.895036366
2.600989821	2.614119455	0.865905291	5.576671571	0
1.417258233	2.993049165	0.844453459	7.05521102	2.490135207
1.688631743	3.816132379	2.42628819	5.993513705	2.103463321
1.604250347	0.003841413	1.015164443	5.883434229	3.020864959
1.696897013	0.022558252	0.020507983	6.100513143	1.689944543
3.135302409	1.3367233	1.34332802	9.616523586	1.735636341
1.12764897	2.054242627	0.643613425	9.08806947	1.514787617
0.107325688	0.086539109	0.077652233	7.434961251	1.330281341
4.541060403	0	1.787581187	5.471950031	1.774803005
0	1.327726682	0	7.64087386	2.51097909
1.538538992	2.816269942	0.673874499	6.486306018	0.671278731
1.880412424	0.911912451	2.208089817	7.252705816	2.687242472

3.874659832	0.048894448	1.802597596	7.216476534	1.190546814
1.976974786	2.841992915	1.102808114	7.576316458	0.608563428
4.427678815	0.090785384	1.339571904	5.755630747	1.981975722
0	0	1.781439448	4.578647845	1.77063897
0.075764638	4.910873893	1.235317166	7.268851034	0.058545005
0.03509985	0.029080934	0.026402039	5.941838324	0.02764348
5.444262167	0.019422057	0.017668367	6.90835445	0.018481943
3.365510603	1.00701349	0.546796282	5.110229634	0
0.431296723	0.311245472	2.707907444	6.841603819	0.287293999
5.689749993	0.081797138	1.307619143	5.6657632	0.077304387
1.689165453	0.020979672	2.101881345	6.878377479	1.08306318
3.428443685	0.076027403	2.346688264	7.678309995	1.291843907
0.022101855	1.073969908	2.089650669	5.820127608	2.681040255
0.228368195	0.176316758	1.627518123	4.666982093	1.641777765
0.016843032	0.014053164	2.657082965	7.450183849	1.055860424
0	0.971429856	0	8.428169088	0
1.900808671	0.065250093	1.870833065	6.848592555	3.326243354
1.122775217	0	1.515823448	7.489710764	2.270117873
1.563652164	2.299141573	0	6.463476725	1.564788225
2.729832252	0.076082577	2.945212262	5.319625303	1.921971445
5.604035331	2.863583231	0.102017772	5.960171084	2.513911553
1.353300624	0.543746018	1.917873028	6.401310498	1.01548745
2.329037365	1.888452539	0.697091289	6.183600685	1.591662502
2.133670909	0.576984925	1.064814442	6.353374859	1.963560583
6.708056507	1.495076917	1.475165494	8.157527856	2.575933349
0	2.434409319	0.893371514	4.773787865	1.455425923
2.799128689	1.348924853	1.336526588	5.446210857	0.084910523
1.24691457	1.863808399	2.281811823	9.381131748	0.057568462
2.413956124	1.065748228	1.063999195	7.638839196	2.081432313
1.038788497	0.00941721	0.008584835	6.570033685	0.00897143
1.221867128	0	2.184066016	6.557668287	0.713764665
4.194920451	0	2.219400673	6.608290601	0.921387182
1.541772313	0	0.966052833	6.227805499	0.96564227
5.676047842	0.02433105	3.448081329	7.524781291	0.023140628
2.968744139	0	0.979685043	6.489124812	1.560489062
3.083082172	1.356346542	2.412151738	6.643864947	1.986566711
5.36466224	0.077784391	1.921731897	6.542970052	2.36252087
4.583479586	1.03823124	1.628831448	6.454857435	0.009020646
1.034542647	1.041499976	1.051859175	6.577762562	0.569759772
0.631001464	2.887125582	1.511519861	7.055651184	0.634183997
2.741717904	0.027384183	2.131963775	6.849776936	0.026035519
1.561586406	4.064895586	1.841588142	8.006165546	4.305797573
3.175874611	1.884426754	1.248890339	7.537458299	1.253004026
1.513028995	0.910749478	0.475654227	5.651992462	2.325820405
2.370691232	0.071576483	0.064419941	6.885191917	1.275434852
2.21515177	1.774647406	0.036807865	7.270895736	1.159114653
2.022441118	4.778470692	2.020423886	5.64193383	3.482080666
2.442254096	0	2.196030343	6.790578593	0
1.84469926	0	0.680508094	6.178266724	3.096852219
0	1.280374816	0	7.082085677	2.237495221
4.216981889	0.877410489	2.131905249	7.070581926	1.939851721
2.237579476	0.045021202	2.212519686	8.959406495	1.790073184
3.241336455	2.25189347	0	6.141719937	0
2.292029888	0	1.973893243	9.521298637	3.877669762
0	0	1.462902424	6.250934801	1.461054842
1.582212509	3.992635872	1.222166844	7.330850882	0
0.039120207	0.032362069	1.125474673	9.461884416	1.127301231
2.511421515	1.347958557	2.064300077	6.864937502	2.31657327

hsa-miR-765	hsa-miR-939-5p	hsa-miR-1226-3p	hsa-miR-4742-3p	hsa-miR-219b-3p
1.952897284	0.093883578	1.990071902	2.757606223	1.322493799
0.050695489	1.851268576	2.891621001	3.631219626	1.215122384
1.106216317	1.111227965	1.11196261	3.153911608	2.132291185
1.05594946	2.075058383	3.670592747	3.404677867	1.056130574
0.082882124	2.012545852	0.107849745	1.367164357	0.083897066
1.152745618	3.808814569	0.04541342	0.043069824	0.036283415
1.450561186	0.135624404	2.603170351	2.588498114	1.45267123
1.066826034	3.097363878	2.416644714	2.414890071	0.015716336
1.501211875	2.483114268	1.495849101	2.895247826	1.500899084
0.033818494	4.383315003	6.450923976	2.787908818	1.753592994
0	2.783064927	1.41509237	1.951582779	1.735981933
0.149251595	3.397995264	3.149278567	3.123945951	1.606638981
1.3194436	1.339095012	2.429548358	1.33917288	1.95289708
1.564204179	2.55976637	4.616175339	3.295684697	1.977770833
1.524276202	3.524932808	3.042986424	0.16069278	3.223900159
2.486773398	1.136117319	3.363617713	2.993021032	1.130250788
2.161716304	2.978554836	2.733498611	1.676051332	0.568493784
0.76571483	1.323843347	1.318977419	0.750344555	0
1.238942306	2.648339295	1.254590529	1.252361785	1.239874548
0.160861597	2.837213903	3.207804185	2.383674983	1.650652362
1.488902182	3.249617925	1.532526712	2.637323995	1.491261022
2.408389279	2.957994703	0.258782157	2.501823651	2.41381955
1.087512734	2.11708587	2.445734207	2.117123921	2.434479145
0.101151293	2.101760962	2.112217991	2.884348045	0.10243449
1.414320659	1.442619244	0.131829877	0.123828954	0.101533849
1.21829502	2.133908694	1.202001181	1.582974952	0
1.561048623	3.139879435	5.325519313	3.554473948	3.142293992
0.043984928	3.267100186	2.852538464	2.578124036	0.04448177
1.252778388	1.267159885	0.077094677	2.666944845	0.060758224
1.122184626	1.737238933	1.128956486	1.128095865	1.72826297
0.50041287	2.179456463	2.470639094	1.562318009	0.953806223
0	2.321525101	3.999551277	1.999612214	1.584621088
1.670643127	2.266359654	2.256820316	1.879707537	1.377463929
1.300078781	1.982586044	3.320291102	2.805723585	1.2991919
2.673871187	1.937347848	2.989076706	3.993463204	0.068542938
2.377075663	1.778340431	1.793944384	2.465185964	2.382224893
2.76858257	2.036774595	2.489864199	1.388641588	2.003654788
3.910729682	3.304654025	4.406653468	3.304539629	1.367487015
3.123446044	2.930074813	2.93888266	1.262155148	1.249044148
2.408448782	2.904548359	2.417436101	2.904589427	1.66442646
2.685394559	2.991777991	4.137043421	2.387332922	1.924163535
3.959802969	2.593909563	1.210600886	1.831596467	0.047362815
0.087521399	3.836351882	2.826304889	2.035516377	0.088602969
1.179851556	1.189200339	3.069352972	1.808749705	2.549706522
2.239972038	2.715680154	4.223004694	3.364865456	1.920375312
1.498030429	0.924445897	2.217121391	1.493334937	0
2.537333676	0.136741559	3.452747431	2.931297769	0.111772702
1.524220459	3.524860091	3.549078491	2.232646026	2.178816
0.079138554	1.350746576	0.102749013	1.994208556	0.080100476
2.624933012	4.492248269	5.142510362	2.133309324	0
1.594922333	3.012394911	1.008819261	2.597045901	1.008454971
0	2.284160415	1.819807014	2.780506938	1.168304317
1.163912989	1.788960161	0.048871064	1.789018586	1.164507493
1.066187254	3.557519718	1.069552259	2.088742358	1.066403934
2.866677373	3.388208101	3.876372175	2.3843376	1.044893163
0.776736563	3.452830484	2.651191558	1.302045244	2.016937408

1.088371759	1.969721019	2.513767364	1.726352177	0.599666382
0.868757749	3.25751344	1.418178085	2.392830028	0.868418583
1.615435548	0.70279964	2.987659811	2.361878706	2.172774521
2.972702594	3.27627235	5.142698675	2.653008963	1.866580345
0.02537129	3.157129309	0.031910071	1.720314114	1.108895112
1.390969978	3.555400263	1.776722558	1.779224946	0
0.689379621	3.197627931	2.321257362	2.513902117	1.889120075
0.006666377	1.620938863	2.626909385	0.007898281	2.036239545
1.96853913	2.963217488	3.13198201	0.974148673	0
3.062842139	3.455822627	3.096237924	3.284215189	1.816309365
1.185108544	1.194795171	1.81859731	3.590211978	0.044138302
0	0.985521576	0	0.985519814	0.986033287
2.562921101	2.783111377	2.782598853	2.561084622	1.565219407
1.063676121	1.938155284	2.137661114	3.203215772	1.062417155
2.233618519	2.227122818	1.907751182	0	0
2.210371501	2.557554178	0.051185049	2.227811299	1.784981498
2.195278169	2.705286554	1.594111028	1.585133295	0.135080921
1.006345253	1.006605145	1.592993829	2.008552829	0.001482005
1.865748948	2.652395684	1.978286431	0.287512474	2.542947513
3.36192883	2.039817671	5.090898925	5.293014815	2.62412477
0.200055717	3.018793057	0.27944263	2.561525103	0.203056855
1.046072662	1.048071962	2.062847394	0.01269715	1.046220179
0.831668103	2.080621	3.056788128	1.372348163	2.094661803
0.064121343	2.958871544	0.082511796	0.077929121	1.270463974
0	3.064958887	4.932505157	3.353245427	2.903744947
1.415825347	1.807522552	2.118018489	2.955460543	0
1.14502088	2.193126686	0.043033031	1.152252861	0.034417353
0	2.559609002	3.879349745	3.558337303	0.982320103
2.411587899	2.808071917	2.137569604	3.263422445	1.835343646
0.049034409	1.218608304	2.280339395	1.218649928	2.254749702
1.608095151	1.658448895	1.669831581	0.190046082	0.152559628
1.421645517	2.889048875	2.11706052	2.106661538	1.423575057
1.580070801	2.108250901	0	2.506292785	1.196753005
2.955823685	2.77741606	0	1.95639998	0
3.213068157	3.240509499	3.381299271	2.357886151	3.454171802
1.698283771	1.705714943	2.458764683	3.140465879	1.097130228
0	1.54522971	0.966945747	0	1.959704989
1.590520675	2.591704021	1.590914204	2.591706537	2.328065011
0.048765716	4.301862999	1.845340331	3.099594917	1.825028651
0	3.000297744	2.63801124	2.421518104	1.450350985
1.326443734	1.705963513	4.647187719	2.014110247	0
0	0.965707951	1.543130629	2.537350256	2.540739459
1.210219854	0.059965795	3.308359179	3.104957318	1.8294638
0.226863857	3.139510906	2.715910116	0.296454898	0.230404808
0.859466627	3.687535112	0.854494484	0.854894183	1.415493053
0	3.336366555	2.68571506	1.48335596	1.488156871
1.423883298	3.116495396	2.96158387	3.37751936	1.423320577
1.689479601	2.677176212	3.186267768	2.320143308	0.842930881
0.019007168	2.109153645	1.688018799	3.442021593	1.680981889
0.003510714	2.34315197	1.604160516	2.020432221	2.605083736
1.688121786	1.694822926	2.712513702	1.694851305	1.088337722
1.34322395	0	2.03040371	2.03420191	1.342439577
1.144642675	2.747011491	2.427743105	2.880486303	2.46943951
1.956854097	0.094998978	1.994560149	1.344875353	0.078718334
0	1.743467852	2.056794291	1.328201747	0.767886542
0	2.278378693	1.321339856	0.787182792	0.792687944
2.333664473	1.168787266	2.084087586	3.275056318	1.562919078
2.468797492	0.910755452	4.184429843	1.882782963	2.689137308

1.18772262	3.974665828	3.448053984	1.818487856	2.231148521
2.23231472	2.380281653	3.581012599	3.161523639	1.101427002
1.339763834	2.78192771	3.294813601	1.36123793	0.082519057
1.260352424	2.280221957	2.871322279	1.235378706	0.469495766
0.055827146	2.311446146	1.250783689	2.311565024	1.236353916
1.113036831	2.969344826	2.748296136	1.725921073	1.717652753
0.017693043	1.679778986	1.680961654	1.079401351	1.076203173
2.470161319	3.678299642	3.899483306	2.264875635	1.911061058
0.268340621	0.356627663	0.390959365	0.357189342	0.272775842
1.307788282	2.405187642	0.095220447	1.326544753	0.074467299
0.019105879	1.085703653	1.688548505	1.687285872	2.915792134
0.068470918	2.712375144	2.721874046	2.712549835	0.069285423
1.669482496	3.807684833	2.689473621	2.911196982	1.669843159
1.627959256	2.359713074	2.379565132	2.813456671	0.15792304
0.012816264	2.887027976	2.075174346	1.057574512	1.650067173
0.972096896	2.544769888	1.549615212	0.971007973	0
0.058891295	1.26203823	2.334509844	1.892960059	2.302929606
0.639788992	2.42981256	2.028407346	2.034942992	0
1.565106099	0	2.97469827	2.782866493	0.983191521
2.677630934	2.378689984	4.011068657	2.378842223	1.916777776
2.064114932	1.450951296	1.456745404	2.889666893	0.103486433
2.127309109	3.044045303	4.720403879	3.616736001	2.124733513
1.902114793	2.691740579	2.683460912	1.878146164	1.595872015
2.368830651	0.574481167	2.481078508	3.295800297	0.581626271
2.122546434	2.95888738	0.153784824	1.510221458	1.477735478
0.893333629	2.170350106	1.852811725	1.450685297	0
3.748829674	3.276847427	3.061938918	3.049351296	0.081749005
3.297702244	3.501701037	3.338979137	3.794083759	1.232529843
1.660170191	2.899745716	2.67796228	1.664948729	1.660488886
2.369770879	2.861044045	4.450132478	2.049815235	0.008686125
0.716103483	2.723892647	3.529649032	1.22379639	1.236646784
0.922247651	3.204182191	2.470080888	0.91944125	1.491861768
0	1.954063486	2.756581789	2.949687322	0.965940517
1.094856039	1.09926292	2.456035905	2.943692378	2.118336557
1.560873332	2.293372394	1.972147964	1.972538213	0.979616731
0.082528556	0.101016551	2.461860632	0.101123971	1.345269047
1.293401232	1.948797063	1.955078873	1.948919355	1.923667485
1.037262221	1.63155055	3.226235355	3.962981124	1.037380359
2.920618261	1.393232055	3.075869133	3.853079252	1.708847093
1.811279008	3.565535668	3.294355895	2.424518237	1.134761442
2.132050262	2.142338083	1.719575795	0.029747029	0.02517523
0.960628319	0.496738012	2.72832652	1.569134523	2.90370665
1.872157491	2.329102377	0.075874969	1.263177687	1.250000946
0	1.243853245	1.514774697	2.132154722	1.787996058
0.064519555	2.69104637	2.365150853	1.921321374	2.330031102
1.156829981	0.044218885	1.782961133	0.044260739	2.787472908
0.003764481	2.830992424	1.605545277	3.346148584	1.60424331
0.904856604	1.870512711	1.464200467	0.901517701	0
0.680423813	1.553344872	0.672758794	1.852522193	0
1.988337511	1.967975813	2.215441732	3.074181972	0
2.131494368	1.205464013	2.372870999	1.709394894	2.288781121
0.040799608	2.230242282	1.183618615	2.560238013	1.173901377
0	0.946850803	2.509532819	0.946844888	0.94862673
0.979331126	2.555221485	4.426817493	3.428307637	0.979269829
0	2.436267244	2.653231858	2.656089425	0.897647805
1.912181572	2.138583918	2.345113083	1.888588813	1.910599095
2.481474805	2.167431879	2.170394526	0.035188689	1.125970531
0	1.343650846	3.393814631	2.047374681	1.353478103

hsa-miR-520f-3p	hsa-miR-937-3p	hsa-miR-520h	hsa-miR-526b-5p	hsa-miR-519c-3p
0.111214861	2.77789287	0.108871639	1.336849397	0.109314115
0.071627792	2.627542597	0.070231198	0.073021588	0.070495282
0.034288217	2.74139294	0.033670571	0.034902704	0.03378751
0.017778764	3.256175585	0.017470193	1.654845581	0.017528647
0.120528677	0.115453186	0.117944698	2.019360358	0.118432478
0.050044724	4.410696077	1.160865644	0.050974008	0.049288405
0.163137638	4.593106395	0.15936472	0.166932932	0.160075865
0.021307077	4.354648268	0.020934274	2.417355137	0.021004888
0	2.700044966	0	0	0
1.150533248	7.05129896	0.04620516	0.047945557	0.046370179
0	2.326381893	0	0.59372674	0
0.230804887	3.174011957	0.224848909	0.236832605	0.225968799
0.110562704	3.049081279	0.108236083	0.112892403	0.108675434
0	4.615320679	0	0.982214496	0
0.194849942	4.884273487	0.190098973	0.199642582	0.190993452
0.042214847	3.521861426	0.041441102	1.747247331	0.041587557
0.569156622	2.289683889	0	1.913011355	0
3.174516429	1.894030641	1.320947737	4.356465439	1.727512822
1.887967655	3.358563033	1.889141261	4.774400582	0.079214329
0.251522471	3.749664242	0.244825783	0.258312623	0.246083994
0.179428289	2.212208448	0.175162871	2.665274763	1.533974801
1.8353278	1.840105657	0.290895654	1.822425436	1.839187529
0.028084336	3.304355424	1.091583321	1.089659411	0.027679864
5.615702638	1.45407918	4.095875211	6.453005205	3.193351008
0.148131957	3.184421124	1.448878685	3.186556463	0.145423602
0	2.830396928	0	0	0.696842514
0	4.359573796	0	3.427408152	0
0.06177891	3.746630689	0.060598525	1.19290194	0.0608218
0.085562256	1.906994424	0.083846619	0.087276483	0.084170875
0.039643175	4.099088278	0.038920621	0.040362351	0.039057398
0.501943347	4.657694087	0	0	0.499261913
0	3.906430649	0	2.321515955	0
1.006847431	1.868680452	0	3.014973308	1.004121907
0	6.415737005	0	3.416247809	0
0.097171978	2.725856854	0.095178771	1.941634467	0.095555337
1.798478904	2.978218417	0.276292367	4.40365397	0.27778144
0.128268959	2.051150313	0.125479675	0.131066222	0.126006068
0.817640443	5.250034942	0	1.360463313	0
0.083837219	3.699626756	0.082161914	0.085510901	0.082478566
0.021489978	5.753015057	0.021113819	0.021863818	1.070313111
1.958252202	4.15165738	0.098747537	0.102914323	0.099140996
0.065941148	6.582372507	0.06467033	0.067208811	0.064910678
0.127811668	5.264615373	0.125034644	3.113637277	0.125558732
1.812674802	3.075311195	2.849380727	7.038439418	1.813510196
0	4.541579298	0	0	0
2.216048312	3.055672675	2.215851133	4.735622144	2.215861404
0.164549233	5.653714429	0.160734469	1.483477037	0.161453466
0.194825338	4.491515101	0.190075158	2.250607357	0.190969489
0.114695866	0.109916951	0.112263466	0.117132353	0.112722726
0	4.779006269	0	0	0
0.002639451	3.820781561	0.002595232	2.334004235	0.002603613
0	3.017813992	0	0	0
0.05389661	3.247538363	0.052883716	0.054905968	0.053075361
0.021099197	3.100917215	0.020730207	0.021465901	0.020800099
0.014170615	4.15891074	0.013926704	0.014412877	0.013972915
9.696524216	4.145924318	8.565957954	12.56246484	9.382572771

0.599677602	3.014352584	0	0	0
1.419128223	2.387156732	0	0.86791943	0.86538357
0	5.018428435	0	1.597278904	0
0	2.290996848	0	0.676265719	0
0.035058521	3.977893445	0.034425924	2.476587565	1.113678006
0	2.341384543	0	0	0
0	3.857702395	0	0	0
0.009063745	2.363405326	0.00890958	0.009216805	0.008938793
0	5.129116114	0	0.97475972	0
0.066073953	6.016259162	0.064800228	0.067344532	0.065041124
0.06128405	2.856021164	0.060114325	2.855657124	1.19538141
0	1.567258346	0	2.78627005	0
1.977126576	4.830874474	3.295781457	5.87888748	3.143986171
0.581287702	1.688114467	0	1.411669379	0
0	3.216025319	0	0	0.930846949
0.056477763	3.966867307	0.055410565	0.057541465	0.055612467
0.20304077	2.742103384	0.198024272	2.739606564	0.198968464
3.595088758	3.469412859	3.710575457	6.654575752	3.179814625
0.365711897	4.064794756	0.354323894	0.377382662	0.356454436
0.00933304	3.747302725	0.009174194	0.00949075	0.009204294
0.324915969	1.900557211	0.3153224	2.609923177	0.317119993
0.014595408	2.653155542	0.014343938	1.642504032	0.01439158
0	3.311361477	0	0	0
1.285143936	2.977384858	1.287144454	2.365609701	1.286859476
0	4.133934757	0	0	0
0	0.854951369	0	0	0
1.151522909	2.199138999	0.046517893	4.037150688	0.046684143
0	2.972866357	0	0.982103854	0
0.870027144	3.254911473	0	0.871700854	0
0.069179035	3.638957624	0.067836917	0.070518187	0.068090721
2.837064254	3.180517479	2.371042146	4.387151986	0.228068753
0.151134745	4.095844227	0.147711277	2.117379596	0.148356831
0	2.935604837	0	0	0.680931859
0.745635938	1.949247803	0	0	0
1.807488047	3.438347671	0.508717244	3.209337221	1.573310908
0.031156616	2.133147944	0.030599263	1.705931279	1.101286446
0	0	0	0	0
0.001470695	3.814848822	0.001446125	3.177237057	1.004878161
0.068783602	1.847605864	0.067450237	0.070113977	0.067702389
0	3.404686493	0.884826842	3.143543381	0
0	4.884331966	0	0	0
0	1.954317616	0	0	0
0.070168462	4.975223608	1.222688451	0.071529633	0.069062337
2.019852942	3.57836949	0.366418599	3.883624048	2.02375816
0.856829639	4.623111648	2.116256798	1.806973246	0
0.917273585	2.874594578	0	0	0.916766115
0	2.600822245	0	0	0
0	2.542837922	0	1.166560268	0
0.026119558	2.439083649	0.025657549	1.083498047	0.025745045
0.004760309	4.272341745	0.004680157	0.004839858	0.004695347
0.028259081	2.9395698	0.02775682	3.134230904	0.027851933
2.998354861	3.913882382	0.795975225	3.665934517	2.027995406
7.228597398	5.273089001	7.434650423	9.659795757	7.281540835
0.112581884	2.444137314	0.110203755	0.114963573	0.110652801
0	3.047675781	0	0	0
0	3.655996411	0	0	0
1.170788046	3.442996367	0	0.675706931	0
2.196642014	3.85984388	2.196399966	5.438363282	1.475629645

0.062202178	5.999253991	0.06101266	0.06338837	0.06123766
0	5.491039442	0	0	0
3.686351156	3.308073504	1.364973405	6.943695199	1.364627119
0	4.530198778	0	0	0
8.686680194	3.156662875	7.672441518	10.11415033	6.355418666
0.036568842	3.761241039	0.035906795	0.037227626	1.118457624
0.024286004	2.104552562	0.0238582	3.92855799	0.023939224
0	3.013901169	0	1.875069879	0
0.466872476	0.435124463	0.450465535	3.815910091	0.453523113
0.106111299	2.759138709	0.103897088	1.322368843	0.104315277
1.085113428	2.930509874	0.025792862	3.125024462	0.025880848
0.09828736	3.602764451	0.096266923	0.100308335	0.096648619
0.02299878	5.116101984	0.022594829	0.02340029	0.022671338
0.242338219	2.395623942	0.235974077	0.248785776	0.237170209
4.337382879	2.666515561	6.156689604	7.887225148	4.733555593
0	3.278112016	1.549551882	4.81148054	1.549574948
0.083815634	2.946692109	0.082140832	0.085488811	0.082457389
3.46114829	3.088001248	3.692284639	5.301321464	2.974029639
0.982752899	3.558378867	0	1.564038217	0.982646596
1.304685545	3.003043717	1.306812364	2.389895802	0.096721703
0.151240205	2.919192001	0.147813716	0.154683405	0.148459838
0	5.421126775	0	0	0
0	2.957113577	0	0	0
0	3.1796388	0	0	0
1.515555864	1.520125656	0.169580575	1.507748296	0.170350994
0	1.449219227	0	1.853771751	0
0.117223867	3.970857826	0.114726102	1.353793358	0.115197662
0.077859871	2.651985559	0.076322491	0.079394981	0.076613134
0.020396779	4.189407386	0.020040643	0.020750688	0.020108102
0.011707519	5.918357801	0.011507153	1.037799037	0.011545117
0	3.764241244	1.223084409	2.71068471	0
0.920392672	4.120873544	0	1.893569084	0
0	2.272721799	0	0	0
0.030511355	3.468608378	0.029966328	4.739583691	0.030069532
0	2.554696116	0	0	0
0.11997611	2.470154827	0.117406607	2.017551906	0.117891664
7.777811226	2.738925232	6.975288351	10.52909735	7.411348689
0.011772165	3.379945002	0.011570662	0.011972267	0.011608842
0	3.82214373	0	0	0
7.51834762	4.998010551	6.322432077	9.353241336	6.972818699
8.153457881	1.112152543	5.727928513	9.827582432	8.168769895
6.061012947	4.912435686	4.40100358	6.911166127	3.021972126
0.084185493	1.902183023	0.082502064	0.085867341	0.082820248
0	2.115880765	0.474388512	0	0
0.092288284	2.97971356	0.09041314	0.094162963	0.09076746
4.499293051	2.546237518	3.042463429	5.901693776	3.238364434
9.91904583	2.022448481	8.656297828	13.19954261	9.229121696
0	2.852083095	0	1.466130693	0
3.360495357	2.30114903	1.844948486	5.634879446	1.549301155
0	4.045236221	1.274230358	0.756216106	0
1.208422901	2.609214577	0	2.495805127	1.473731428
4.923227372	3.433757188	4.920922585	8.374700971	4.800609693
0	3.378193931	0	0	0
0	2.968172978	0	0	0.978604768
8.796755097	2.650840341	7.940874396	11.04429439	7.084196966
1.882407669	4.189093096	1.881128571	3.090741356	1.583148748
9.107872378	1.744598581	8.187408177	11.41903796	7.452835292
6.143970321	3.683614326	5.065495998	7.583247302	3.589616945

hsa-miR-216a-3p	hsa-miR-520a-5p	hsa-miR-541-3p	hsa-miR-5003-3p	hsa-miR-518b
2.439742698	0.111842125	1.994040061	0.074667757	0.11251749
2.628353639	0.07200085	0.067768627	1.212280863	0.072402138
0.034303627	0.034452877	1.111983963	1.105311927	1.1098543
2.076893625	0.017860956	0.016922712	2.393001017	1.057594468
1.368439977	0.121220743	2.470440873	0.080456668	0.121966045
0.050068013	0.050293618	1.161726178	2.185667794	0.050561211
0.163232303	0.164150521	2.160715681	0.106124716	0.165242454
4.195259965	0.021406395	2.091866913	1.66261329	1.068859611
0.928033216	0	0.927021927	1.911425774	0.92845364
0.047098895	0.047309744	1.152436482	1.142813273	0.047559819
2.486156015	0	0	0.591424449	0
3.464056112	0.23240996	1.669824846	0.144347363	0.234143225
0.110620944	0.111185502	1.34375818	0.074259933	0.111856048
5.015241371	0	3.672689391	0.982612402	1.562947813
0.194969319	0.196127666	0.181843608	0.124407338	0.197506191
1.135378341	0.042421207	0.040072122	0.029615786	0.04264303
0	0	5.175556845	0.571016354	0.571388462
2.526495685	3.238475973	3.309479428	0.365738826	3.41497979
4.62779442	1.887376287	1.888925701	0.055104606	3.361617495
8.168813947	0.253329302	0.23326947	2.295989193	1.71299474
1.5308919	1.529539323	1.535667624	0.115600677	1.527529568
0.299650854	0.301745712	2.545667164	2.395308284	0.304245119
0.028096781	0.028217311	1.092110411	1.686414167	2.119733411
6.188197327	4.557791578	2.118384673	1.41313479	4.980112309
6.051020357	0.149027288	1.450290796	0.097233978	2.910936545
4.757439152	0	1.201865916	0.702418042	0.700287043
0	0	0.97895983	0	2.292828384
4.620470332	0.062094048	1.822103324	1.798870425	1.194442634
0.085605154	0.08602088	3.17988655	1.250305779	0.086514372
0.039661206	0.039835855	1.740252941	1.121127488	0.040042963
1.98899539	0	4.039474598	0	0.504410256
3.806891439	0	0.999699662	0.999714668	0
0.547937375	0.54887687	1.000726258	1.021598022	2.68211467
1.673018203	0	0.759522948	1.30229358	1.977743804
2.38902322	0.097705138	1.304640731	1.281248024	0.09827899
7.096092444	2.985565773	1.802228179	0.17254114	2.509099012
0.128338836	0.12901633	4.880068789	0.085217774	0.129821342
5.048741239	0	5.906466272	0.821573471	0
1.899576142	0.08428502	0.079212111	1.245651586	0.084766846
2.092582048	0.021590192	2.092636816	2.671775402	0.021697864
1.311798619	0.101387685	0.095087395	0.068134783	0.101987528
0.065972897	0.066280506	1.210884556	2.239433444	0.066645494
1.389073575	0.128555734	1.393242612	1.359855145	2.496698461
2.579400774	2.579178856	1.813615072	1.79137083	4.934531853
3.22297613	0	8.724092911	0.938138303	0
2.47568793	3.055093312	1.898991277	1.498833237	2.886685433
5.847491753	0.165573431	2.617785627	2.862040095	1.48766355
0.194944695	0.196102848	0.18182114	0.124393411	0.19748114
7.629625539	0.115347126	0.107996141	2.391283687	0.116048386
1.816763915	0	7.179965514	2.407009014	0
0.002640553	0.00265122	2.334071488	2.595955016	2.820141195
2.454203817	1.157449105	0	0.664372036	0
3.249451665	0.054166919	1.793327927	1.162435816	0.054457562
1.668581365	0.021197499	1.669081918	0.015009973	0.021303116
1.641485898	0.014235571	1.04692481	2.055325637	0.01430535
0	9.916126724	4.942753649	0	10.27791676

6.196535692	0.600617974	0.594119026	0	0
0	0.866550226	0	1.832732602	1.818832991
0	1.217948013	1.591135085	1.618759109	1.218957676
0	0	7.043324723	1.187181103	0
8.212862367	0.035227173	0.033305755	0.024711113	1.112265073
4.972329307	0	2.915184285	1.793629004	0
3.063319981	0	0	2.551101564	0.68898918
4.506496842	0.00910479	1.03012118	0.006500115	0.009148878
7.38428145	0	0.974067771	1.968886432	0.974574218
1.836611504	0.06641409	0.062552852	1.812546205	0.066779918
3.281276948	1.193653531	0.058049302	0.042464431	1.192945519
0	0	1.567256027	1.568629465	1.980885868
4.972399833	4.220710639	1.977083822	2.300546473	5.14218231
6.085137051	0	1.40196552	1.066850499	0
5.719465239	0.931452691	1.499622037	2.496041658	0
0.056504414	0.056762602	0.05352549	0.039255778	0.057068888
0.203166866	0.20439053	0.189316156	0.129021654	0.205847071
2.331091092	5.40289769	1.593029353	0.001430841	5.68305283
4.473821574	0.368805123	0.334947651	0.212569637	0.372157152
0.009336999	0.009375332	3.04512815	0.006692029	0.009420759
0.325158489	0.327515493	0.29891613	1.780269753	0.330329464
1.047672076	0.014662378	1.04831718	0.01042838	1.047384657
0	0	1.765212016	1.383697662	0
8.689254691	0.092183611	1.288431992	1.266708956	3.73643774
4.206322079	0	0.927442753	1.911945689	0
0	0	2.11634677	0.860165089	0
6.852941673	1.768066234	2.198706008	1.752020288	1.150554206
1.562705145	0	0.981613156	0	0
0	0.870368527	2.969397305	1.837678706	0.870802102
5.141469622	0.069537494	0.065469725	1.205495878	0.069923057
4.633567415	2.836454181	0.21650238	1.600289509	3.90967808
0.151220596	0.152053201	2.574011135	0.09902579	1.4512101
7.921121104	0	0	0	0.684534189
1.266249843	0	0	1.980770163	0
0	0.959665481	0	2.558672886	2.009557568
1.100642227	0.031305177	1.101972923	1.697067401	0.03146483
2.27605797	0	0.966988583	1.54772011	0
0.001471307	0.001477234	2.32870415	1.590465505	2.006449652
4.024368073	0.069139717	0.065098374	1.204397292	0.069522754
8.007501273	0	0.88405942	0.88875652	0.886338839
4.389055367	0	2.663624411	1.328503329	0
0	0	0	0.967224434	0
1.221061704	0.070532804	1.852297826	1.208241123	0.070924704
0.378694585	0.38164562	2.020400457	1.87455785	0.385173581
6.976858216	0	0.854824266	2.975259139	0
0.91729281	0.917493741	2.464165798	1.489397112	0
3.586846874	0	0	0.866730059	0
0	0.424456955	1.836166322	1.184648504	0.425941083
2.111984557	0.026242673	0.024838608	1.080866031	0.026374965
1.015653332	0.004781644	1.01586772	1.602695653	0.004804558
2.448478778	1.696014934	2.120916941	1.087306675	1.695700553
5.652948389	3.256463706	1.720727356	2.311717843	1.723115192
3.195657177	8.280882838	2.584787188	1.826049211	8.451747609
1.997858959	0.11321854	0.106030599	1.321321853	0.113904039
0	0	2.050817622	0	0
0	0	2.017601661	0.794391517	0.791533533
2.913958848	0	1.538499537	2.123541421	0
6.296214529	3.445692438	0	0.914408681	2.197035993

0.062231891	0.062519762	2.256826867	2.55481792	0.062861303
0.610447106	0	2.804438325	1.776579285	0
3.842191716	1.361468091	0.111444625	0.079143875	3.686578814
0.904976838	0.473636853	2.759850052	0.917427025	0
2.658347445	8.513521516	1.251247816	1.233170797	8.729968294
3.344509513	0.036745359	2.155077861	0.02575006	0.036935079
0.024296673	2.104178914	1.079860299	1.075324886	2.10394705
1.354194435	1.355237077	0	0	0
0.467290099	2.212150303	0.422903812	0.257901734	0.476228862
0.106166714	0.106703863	1.330823983	0.071467508	0.107341788
9.257574645	2.11240559	0.024969356	2.690241456	1.688044674
1.949518043	0.098827837	1.950423231	0.066522077	0.099409579
0.023008853	0.023106406	2.098976283	3.416846306	0.023222046
5.680912433	0.24405442	0.224979286	0.150574842	0.245908232
5.706005361	6.377245793	0.016674903	2.068059117	6.339976584
2.764809823	3.861554622	0	1.965082308	3.655476163
6.956938048	0.0842633	2.338694987	1.867577903	0.084744981
6.101057712	4.061627643	2.024825673	0	4.264732318
2.559970997	0	0	1.565299414	0
7.418623658	0.098902891	0.092785026	1.911030425	0.099485161
2.578178137	0.152159477	0.141828477	1.417156903	0.153150203
0	0	4.394327098	1.386232866	0
0	0	1.196862091	2.563144481	0
0	0	1.046920455	0	0
1.515434052	0.174767861	1.520175144	1.469898218	0.175952253
3.287674672	0.891331782	2.425050704	1.458427475	0.89169805
3.681835298	0.117892721	0.110345414	3.223153781	0.118612969
0.077898299	0.078270673	0.073613648	1.229409066	0.078712627
6.792894515	0.020491653	0.019408957	1.659384429	0.020593585
2.050963846	1.038219151	2.863062131	2.36917413	1.038084271
1.901322415	1.225806274	2.710806384	2.593020089	1.60390374
0	0.920604699	0.919290842	0	0.920875059
0	0.965195469	0	1.545034234	0
0.030524951	2.13004476	1.705514547	0.021570643	1.704282411
0	0.979147513	0	0.979828168	0
0.120040457	0.120664279	2.023095886	1.340160382	0.121405374
7.028301648	8.55800771	0.094932409	1.290427827	7.920368915
1.632024996	0.011825821	1.039047837	1.036966932	0.011883458
0.572495477	0	3.636645634	2.173509362	0
2.409458828	7.875910961	0	2.065840386	8.214582005
5.303663407	8.627973991	0.032683654	1.708227415	8.49476906
3.021393746	6.070634623	3.852228748	0	6.378669875
0.084227584	0.084635474	0.079538079	0.057485888	0.08511965
0.91207941	0	1.512914708	0.477433428	0
2.708790835	0.09278973	2.705854844	1.268312187	0.093329386
5.841041849	3.947948313	1.166113659	0.035878148	5.748827032
3.025072173	9.001767714	1.017014258	1.016139343	9.523299653
1.464804786	0	0.901372837	1.472259171	0
5.577831348	3.536901162	2.302080297	1.194923663	4.521433062
3.057644056	0.75382694	1.961205497	0	1.277407587
6.088866884	1.477774354	0	3.104627917	1.900917045
7.443738714	6.166158628	0.054153236	1.783953463	5.709457096
0.947447514	0	2.729562536	0.949140866	0
0	0.978793232	0	0.979484779	0
8.26395934	8.922732533	0	0.898611261	9.50187294
1.584670907	2.52444794	0	3.15044755	2.130597654
6.06412983	9.367570511	2.172133822	1.124443007	10.36324956
5.551946788	5.501805595	2.29579526	0	6.32481514

hsa-miR-518f-5p	hsa-miR-520g-3p	hsa-miR-520e-3p	hsa-miR-548d-3p	hsa-miR-516b-5p
1.34117288	0.111286462	0.1068058	0.073531722	0.112015878
0.071914943	0.071670393	0.068996012	0.048590559	0.072104128
0.034414971	0.034307027	0.033122695	0.023849393	0.034498437
0.017842037	0.017788155	1.058600557	1.055255918	0.017883692
0.121061307	0.120607667	0.115668319	1.339634764	0.121412472
0.050236314	1.159670492	1.161437273	0.034440212	0.0503625
0.163917087	0.163253194	0.156052482	1.442667989	0.164431309
0.021383535	0.021318424	0.02060325	1.065988296	2.417956209
0	0	0	1.911927199	0.928267302
1.150217955	1.150489026	0.045432467	2.766451697	0.047374119
0	0	0	1.079121178	0
0.232039815	0.230987873	0.219649468	0.141859039	0.232855374
0.111042036	0.110633795	0.106184771	0.073131003	0.111358016
0	0	0	3.783788635	0
0.195833109	0.194995664	0.185939021	2.162622913	0.196482043
0.042373699	0.042238419	0.040755188	2.483458439	1.134919273
0	0	0	1.713395688	1.672352315
2.526954759	2.182326117	0.362726216	0.36676198	2.048061097
1.887526761	2.663314123	0.077499734	1.855774333	2.326704628
0.252912548	0.251728411	0.238989889	0.152748284	0.253830867
0.180310248	0.179559026	0.171423295	2.895971544	0.180892242
0.301212548	0.299698445	0.283483908	0.17698442	1.832412767
1.090735601	1.695548829	1.091924107	1.086392281	0.028254101
2.916778872	4.45560242	3.42098209	0.096529361	5.361967073
1.444972037	1.445780039	0.141860247	0.095689878	2.112656517
0	0	0	0	0
0.979284943	0	0	2.780775265	1.972250661
0.062021485	0.0618149	0.059553743	1.183890246	0.062181279
0.085915257	0.08561462	0.082330949	1.248998813	0.086147872
0.039791498	0.039665184	1.128760536	1.725243649	0.039889173
0	0.945085222	0	0.960328312	0
0	0	0	2.321561275	0
0.548627649	0	0	0.550260932	2.074262154
1.288746647	1.288340125	0	0.76769813	2.44150362
0.097582336	0.097232846	0.093419521	0.06482919	0.097852794
2.511627153	0.284471748	0.269396453	2.358268489	2.985307771
0.128844141	0.128354255	0.123023974	1.358931796	0.129223405
0.817996898	0	0	0	1.358586218
0.084181891	0.083888348	0.08068167	0.056419284	0.084409012
0.021567125	0.021501427	0.020779818	1.066542578	0.021617915
0.101259328	0.100894042	0.096909674	2.347430481	1.310724549
0.066202363	0.065979903	0.063545876	1.810727493	0.066374446
1.388345753	0.127896587	1.392916978	1.357799145	1.387731104
2.849512221	4.191894232	0.057311571	1.177333286	5.462463922
0	0	0	1.9220163	0
1.89938963	1.492899453	0	0.927873982	1.899484354
2.164859282	0.164666078	1.495281733	0.105224206	0.165857361
0.19580834	0.194971035	0.185915889	1.514542443	0.196457165
0.115197099	0.114770203	0.110119608	1.955639918	0.115527535
0	0	0	1.832357974	0
0.002648511	0.002640796	0.002555912	1.008329767	1.00866322
0	0	0	1.857338284	0
0.054104682	0.053927483	0.051986624	1.161651886	1.170961936
0.021174872	0.021110428	0.020402561	0.014808716	0.021224694
0.014220621	0.014178037	0.013710007	2.378516984	0.014253539
9.746444317	9.426173119	8.390224354	1.711880655	9.753544619

0	0	0	0	0
0	0	0	0	0
0	0	0	0.712301371	1.218188079
0	0	0	1.570719443	0
0.035188349	0.035077787	0.033864821	1.709754346	0.035273838
0	0	0	0	0
0	0	0	0.691823232	0
0.009095344	0.009068435	0.008772562	0.00641467	0.009116143
0	0	0	0	0
0.066335768	0.066112797	0.063673212	0.044987823	0.066508246
1.193786948	1.194138054	1.196260611	1.182503997	1.193485402
0	0	0.985504499	0	0.985716088
3.558391167	4.220732388	2.560246344	0	4.060369303
0.582025573	0	0	0	0
0	0	0.930492764	1.915977474	0
0.056697019	0.056510295	0.054465571	2.799655757	0.05684144
0.204079343	0.203194694	0.193634811	1.532565703	0.204764933
3.179828376	4.402716984	2.331094573	0.001412258	4.010296412
0.36809087	0.366064012	0.344496047	1.846147823	0.369665357
0.009365598	0.009337872	0.009033019	0.006604021	1.030491596
0.32691548	0.32521202	0.307013994	2.437681746	0.328237948
0.014646964	0.01460306	0.014120533	0.010290029	1.047512724
0	0	0	4.517671054	0
2.368571575	1.28505988	0.08818229	1.265296656	1.284094465
0	0	0	2.906176357	0
0.856904158	0	0	0	0
0.047576778	0.047423145	0.045739446	0.032675763	1.150971707
0	0	0	0	0
0	0	0	0.873833543	0
0.069454951	0.069219969	0.066649679	1.20446433	0.069636727
2.368921045	2.369688749	2.832558443	0.14300718	2.836187868
0.151841552	0.151239542	0.144702857	2.054042801	2.921960019
0	0	0	0.687309204	0
0	0	0.742773698	1.281925409	0
1.808253554	0.95852755	1.294867417	1.618863226	1.577134191
0.031270979	0.031173588	1.101769931	1.696427487	1.707000004
0	0	0	0	0
1.590891759	0.001471442	0.001424275	2.005816677	2.006460976
0.069057714	0.068824269	0.066270704	0.046748839	0.0692383
0	1.844984867	0.884358433	1.452654767	0
0	0	0	1.72537967	0.784648003
0	0	0	2.54180593	0
0.070448905	1.221049828	0.067597827	1.207193222	0.070633667
0.38089404	0.378761567	0.356097639	0.214287976	2.016495261
0	2.116736246	0	1.418309895	0
0	0	0	0	0
0	0	0	0	0
0	0	0	0.422620564	0
1.084498254	0.026133623	0.025247468	2.099584935	0.026276734
0.004776734	0.004762747	0.004608897	1.01499057	0.004787544
1.091289565	0.028274373	0.027311087	0.019736789	1.091149889
2.281768168	2.496709694	2.027917723	1.346221476	2.849886894
7.072207402	7.962614197	7.035718744	0.646142215	7.813389083
0.113071879	0.112654555	0.108107373	0.074370617	1.344545814
0	0	0	0	0
0	0	0	1.337485038	0
0	0	0	2.529066523	0
3.321052258	2.455630288	1.475327848	1.483481962	2.865736195

0.062446636	0.062238448	0.059959829	3.237748125	0.062607671
0	0.610465146	0	2.027978145	0
4.621710697	2.017253801	1.366037453	2.729931777	3.08140735
0	0	0	0.91927414	0
7.818439328	8.144580313	6.84902408	1.231971283	8.119247744
0.036704723	0.036589006	0.035319639	0.025396297	0.036794202
0.024373755	0.024299027	0.023478425	0.017008129	1.680685416
0	0	0	2.481040196	0.548490974
0.470319949	0.467382301	0.436431227	0.252658712	0.472605466
0.106567367	0.106178942	0.101944184	1.302965608	0.106867991
0.026352761	0.026271599	0.025380493	0.018363949	0.026415511
0.098703348	0.098349063	0.0944838	1.28265911	0.098977521
0.023081633	0.023011076	0.022236194	2.412519685	0.023136181
0.243658605	0.242533849	0.230423693	0.147952624	0.244530759
4.549412888	6.466711298	5.043366692	0.012325526	5.819523199
1.54978007	1.549732409	1.549486903	0	0
0.084160202	0.083866748	0.08066103	2.295185965	0.084387255
2.233504896	4.061970858	3.694997925	0	4.005898733
0.982788117	0	0	0.98343357	0.982813816
0.098778289	1.304595766	0.094554942	0.065572674	1.303563148
0.15194764	0.151345095	0.144802668	0.097509673	0.152414269
0	0	0	1.681459414	0
0	0	0	2.377451151	0
0	0	0	1.069527841	0.583273386
0.174514702	0.173794791	0.165994032	1.466983442	0.175072394
0	0	0	0.8944354	1.45058914
0.117738637	0.11730021	0.112525084	0.07720846	0.118078011
0.07817607	0.077906779	0.074963465	0.052602159	0.078384411
0.020469815	0.020407618	0.019724395	2.078418252	0.020517899
0.011748593	0.011713615	0.011329109	0.008270546	0.01177563
0.714223844	1.901333478	0	2.190199402	0
0	0	0	0.923078945	0
0	0	0.964708662	1.545238554	0.965224263
1.098418219	1.098594707	0.029482727	0.021277063	1.098267431
0	0	0	2.295894886	0
0.120505741	0.120054656	0.115142879	1.338246912	0.120854927
8.828623666	7.849281064	4.98148512	1.915593093	7.075446457
0.011813472	0.011778296	0.011391609	2.045841362	0.011840663
0	0	0	0.575326789	0
6.738914601	7.066492265	6.725115937	1.818247501	7.290891404
8.305903276	6.442557832	7.137862627	0.023926102	5.634808958
4.973772114	5.254993824	4.215663711	0.965810701	6.15670128
0.084531842	0.084236871	0.081014684	2.296345634	0.08476007
0	0.477818856	0	1.275892847	0
1.286307085	0.092345534	0.088757446	0.061765919	0.092928593
2.815931898	3.947791067	2.546302	1.764315814	5.112718668
9.69444278	10.0131136	7.691995824	2.342611451	9.980115692
0	0	0	2.198135031	0
2.092092859	2.793089765	1.548333002	1.883720226	2.926034135
0	1.275967332	0	0.758040734	0
0	0	0	0.454163222	0.881353208
5.529916808	5.490935077	3.853504002	2.208552604	5.530204069
0	0	0	0	0
0	0	0	1.560825701	0
8.142250876	8.497577874	6.556806663	1.464520963	8.917317347
1.585150682	2.129326835	0.698173879	2.171698051	1.883238341
8.341958528	8.843716912	7.202137864	2.478274473	8.441496178
4.759354558	5.551934235	3.149735285	0	6.025127174

hsa-miR-5010-3p	hsa-miR-7974	hsa-miR-5586-5p	hsa-miR-516a-5p	hsa-miR-362-5p
1.98379792	0.072048393	2.439792827	0.112670113	7.151159247
1.85177148	1.20977125	3.650151626	0.07249277	5.435296325
2.960127599	1.104183002	4.629344955	2.473979985	5.384238597
3.542742483	0.012285087	3.896133609	0.017969184	6.36034994
2.013490221	1.337066717	5.627966602	0.122134498	7.328161764
0.043283148	0.033798991	2.540703127	1.777160779	5.203270642
0.136586921	0.102180479	0.157160925	0.165489414	6.789238859
2.680202676	1.661602152	4.194156683	3.426441408	6.880024102
2.222376387	0	3.472364243	0	6.479830655
3.797108275	3.768859343	3.92585034	0.047616279	6.466930455
1.708523527	3.28530843	6.402826033	0	6.19427572
2.33179198	0.138626179	2.364042201	1.660251999	8.276829799
1.339573482	0.071656908	1.343136	0.112007581	5.800095633
3.29562399	1.564655846	3.878717324	0.982099324	6.852934021
0.161692822	3.616865993	4.377319331	0.197818125	8.986054488
2.993452778	0.028667823	2.508063137	2.177309215	6.970271411
2.123221635	1.963817664	6.15351497	1.038309914	5.134380284
2.32704086	0.368182857	3.526619339	3.358823475	5.086105623
1.882256339	0.05324435	3.953721073	4.466733919	5.81464653
2.385684861	2.282888092	3.753197923	0.255723609	5.772806029
0.149539491	0.111232724	4.197126544	0.182090161	8.160771477
2.504335997	1.720346106	1.840251025	1.829140929	7.426908881
1.091622345	0.019263621	2.447736307	4.047085463	7.157382751
0.125703926	1.40740108	3.952073625	5.753524195	6.452330704
0.124550579	1.404016278	4.441374389	0.150210305	6.02084321
2.133179637	0	1.202702907	0	5.873645291
2.777797838	2.296304507	4.359471096	2.776681291	6.931544367
1.81735211	0.041385018	4.098703326	0.062509487	6.814891672
3.360001166	0.056391495	1.907039003	0.086625851	6.936809861
2.163351454	0.026970222	1.740308133	0.040089716	7.109944983
2.480982176	0.962815834	3.93864846	0	4.156874191
1.584601952	2.321564435	3.699981611	0	7.128796381
2.823627571	0	3.284720526	3.01313683	5.179309658
2.235246972	0	6.335537157	2.44206911	6.327315558
4.412245624	1.902633761	4.138623213	0.098408645	8.712451195
2.923919679	1.691865732	5.728763737	3.625386375	8.168122762
0.108460209	0.082168239	4.014421708	0.130003312	6.566311626
4.237181761	0.822974143	3.610158409	0	8.726721822
2.661425902	1.242663022	1.901004025	0.084875687	6.990800248
3.269098552	1.662241098	3.426709463	0.021722164	7.031693068
2.722449955	1.91317192	3.242640192	0.102123063	6.725817988
2.594607186	0.044046504	2.875620948	0.066727921	6.801784936
0.108087631	1.355055788	3.717038358	0.129538341	5.535968968
2.569653584	1.78844776	5.240841791	4.749112315	7.525832609
2.233744574	0.93869583	4.219932162	0	7.621225009
1.900835049	0	2.215878667	2.475999991	4.522001749
0.137713962	3.711662873	5.509759631	0.166927332	6.184928194
2.683961767	2.156810425	3.951623533	0.197793022	7.362782842
2.435187895	0.074132355	4.316000139	0.11620687	5.529741695
2.610027657	1.429322724	4.303455644	0	6.231037192
3.472087466	0.001843953	4.101033448	2.334057399	6.406522755
1.824433997	0	4.761935755	0	7.565907549
0.046562132	0.036302793	5.391441012	0.054523186	5.515950387
2.414181794	0.014544789	3.272055835	0.021326952	7.39635267
3.065600296	0.009817339	4.071509362	0.014321097	6.582844443
2.823167902	1.7138038	4.14493653	9.918102621	6.293039428

1.434577128	1.467407802	4.239963862	0	5.513604815
0.864358343	0	0.864843342	0.867109228	7.472794188
3.232375876	2.906409888	3.593932162	1.219218459	5.185460085
2.65215855	3.608856751	2.916254675	1.173807867	6.695674387
1.720500134	1.708789612	3.870919329	2.477266245	5.794043169
2.09093361	0	3.063508897	0	6.024315285
3.083080338	0	3.922724031	0	4.970569591
3.042272156	0.006302549	3.504651063	0.009158826	6.156770074
2.963123957	3.667285	3.545363706	0	5.48170943
2.595113192	0.044131174	4.311479643	0.066862534	7.676227757
2.845023233	0.041067572	3.083587423	0.062008004	8.170006841
0.985510881	0	3.300057938	2.786196383	5.298596074
1.977546464	0	4.496491453	5.495694053	7.999690139
2.634345469	0.585731575	4.601252108	0	6.656416569
1.500609541	3.716836217	6.24560691	0	5.61405608
3.418203119	1.167863418	3.585428846	0.057138047	6.139564656
0.168105426	0.124034497	2.743519579	2.279139564	7.799612571
2.330833338	0.001387863	5.682852074	5.711073693	3.595081218
2.656255337	0.203246277	0.347767626	0.372917796	6.000216858
1.030891723	2.35915438	2.364659105	1.030382248	8.63870914
0.260192273	0.184720227	2.618270787	0.33096757	6.376198536
3.237960815	2.643610145	3.393746246	0.014750557	5.777275204
0	2.359667648	3.967458355	0	6.934993403
1.919886783	0.060177868	2.705744706	1.925029435	6.526223427
2.895649688	2.232268233	2.48030204	0.928928309	7.118745354
2.378263325	0	4.288892604	0	5.184993306
1.765870694	0.032069717	2.798250873	1.767410967	5.366565736
2.78154086	0.982669612	4.829087051	1.562827315	6.236979025
4.047823115	0	2.392808317	0	6.812906489
2.606899747	1.203080848	3.779116918	0.070010134	5.695577595
2.337492397	1.59084018	3.182903567	0.236745176	5.272846604
2.107712182	1.411102202	4.098212914	1.450759434	7.574911873
1.858838188	0	3.169323862	0.684779593	6.95396221
1.65087555	1.98500717	2.19948991	0	5.963703726
3.60321116	0	4.267282917	6.462093396	7.424138387
0.02709684	0.021324305	2.461013185	0.031500866	6.091366074
1.545176965	0	3.756451703	1.956504405	6.054880312
2.006348548	0.001028321	4.59261274	2.591943456	6.694440407
4.002019318	0.045855366	5.457493336	0.069609259	6.748210084
2.640808171	1.453504368	5.51230925	1.444283882	5.873882684
2.83946451	0.789612552	4.16273363	0	6.590361014
0.965683762	0.967529474	2.948998987	0	5.94413118
1.221833017	1.205787938	2.622181718	0.071013213	5.627579506
3.144154635	0.208840796	4.103328508	2.012561638	8.22840107
1.407571157	1.822824545	1.406142403	0.857800172	5.823595825
0.91623454	1.899266852	2.874411154	0	5.884482215
1.816405317	0	5.554901469	0	7.089325293
3.407921888	2.248583883	3.65871948	0.840939989	5.978971261
3.118870987	0.017941054	1.688601195	0.026404823	6.457847046
1.603974576	1.014905869	3.931171588	0.004809728	7.195542558
3.128222198	0.01938107	4.664898622	2.448071284	6.053126791
2.033807757	0	5.588458029	4.053192849	6.73669187
2.598789208	0	4.179525285	8.325413918	7.403058172
2.428100237	1.317162969	3.659561177	0.114058956	8.392884275
0.359534681	0	0.758244536	0	5.63598293
0.7871067	0.79595089	5.31790318	0	5.367371278
2.932941852	0.677506795	5.509992797	0	6.940081727
0.910700502	0	4.250429628	2.456001474	6.062074232

2.58029688	0.041656366	0.060313054	1.195536476	6.99575252
1.743190426	0.615285655	3.955418145	0	6.453895357
1.361678656	0.076343764	1.365771761	4.111556687	6.699812873
2.421575115	0.474565802	3.279910049	0.475403891	3.766884442
1.877922331	1.230364038	2.657833236	8.662099144	8.34099905
2.15123001	0.024932739	3.635346913	0.036977905	5.859209174
0.021164311	0.016703478	3.704262941	2.430813069	6.126445096
1.353469915	0	3.511150501	3.011736535	5.382514836
0.360151636	0.245899592	0.441090446	0.477336488	7.660131118
1.967315503	0.068975769	1.977669066	0.10748594	6.565551576
1.08578204	1.678786628	1.08596118	1.687966515	6.202491424
1.941747794	1.280616305	3.898691444	0.099541019	5.103515975
2.687529284	0.015832716	4.202359825	0.023248145	6.07411224
0.198470289	0.144547301	5.040853463	0.246328035	6.880609453
2.398882151	1.648294504	2.890558945	4.845536717	6.535254382
2.766427392	0	0.971021073	3.655481472	5.930112602
2.930993096	0.055305827	1.264393076	1.259960809	5.571551125
2.59225962	1.146479721	5.085594982	4.484173492	7.179741733
3.781848915	0	5.059928665	0.982874546	8.657994441
3.211400238	1.28080948	3.430660416	1.302405033	8.237555118
2.891150189	0.095451681	4.553562429	0.153374231	7.703059611
3.139401756	1.02807646	3.368215385	0	6.466198616
2.690777439	1.219378645	4.119172508	1.202728091	6.929892881
2.768230013	0	3.082280729	0	5.870064527
2.174023425	2.106098556	3.273280495	0.176220169	8.174210325
0.889606788	0.894958013	5.575840617	1.450978845	5.650359963
2.00301111	1.95932281	3.677261898	0.118775751	5.292912526
3.656339222	0.051584538	1.879961217	0.078812452	6.159300428
1.06693588	0.014067658	3.097831598	0.020616589	5.498492679
3.514794819	0.008125356	2.863372648	0.011831116	7.338512863
2.157436597	1.24340448	5.525578099	1.902945316	5.902824547
1.487125712	0.92347244	2.208788408	0	8.987840473
1.953984012	0	2.27268973	0	7.697010921
2.94400004	0.02089225	2.724985726	1.70419187	5.857586467
2.29331395	0	2.554663564	0	7.222866951
2.788586738	0.07727539	4.702899023	0.121572874	8.536173036
1.311336838	0.065673406	2.739602893	7.687091132	6.920549756
2.638274786	0.00816984	3.227610547	0.011896463	5.790156374
2.871344236	0	3.159505875	0	7.041004698
2.239734798	1.142648772	4.996104688	7.786065939	5.892531112
3.616769392	1.10451427	4.926033727	7.071950394	7.472247074
1.288474129	2.410124121	3.94734235	5.383697586	4.899116616
2.662699649	0.055535938	4.388283471	1.261048913	6.692238295
1.752614224	0	4.304668746	0.480588832	6.089272906
2.358502537	1.888425801	0.089312517	0.093451305	6.273518916
2.209116034	1.153679861	3.237515774	4.712641602	5.958273825
0.004474602	0.003560375	4.348221549	10.21233996	6.969957162
2.6662675	0	3.546657338	0	6.140720625
2.498067915	0	4.088165383	4.680246114	6.531064117
2.220113112	1.293053118	4.33722838	0	6.462234554
2.632144	0	2.717008611	1.90132908	5.705287428
1.182397622	0.038403553	3.588324535	5.139293835	6.117505634
1.520582311	2.518320497	4.722955105	0	6.082876768
2.96938202	0.979678268	3.966654129	0	7.231675252
4.010753613	1.465301643	5.151226146	9.607028172	6.146742115
2.850489447	1.227939056	3.405428726	1.586564935	5.961452318
2.167783894	1.123084489	1.744570533	9.418063001	5.821710434
2.519518469	1.756990524	4.001950246	8.144913808	7.116320043

hsa-miR-4791	hsa-miR-548y	hsa-miR-1323	hsa-miR-454-5p	hsa-miR-519d-3p
0.06810559	0.076622913	0.110064091	1.330795339	0.110086664
0.045170871	0.050527077	0.070942506	2.271806555	0.070955959
0.022253606	0.02474769	0.03398539	1.713113364	0.033991339
0.011690176	1.055896111	0.017627526	2.396634122	1.655844574
0.073312128	0.082588237	0.11925941	1.994839637	0.119284303
2.504567049	0.035769765	0.049587206	1.156599975	0.049596191
2.06698294	0.109080504	0.161282606	0.120373117	0.161318954
1.659994692	0.015503831	0.021124344	1.06835322	1.069571976
2.233804255	0	0	2.226185782	0
0.030251067	0.033710167	0.046649492	2.184209838	0.046657892
1.759024774	1.074647835	0	1.96882826	0.587705066
1.571849388	0.148655308	0.227872035	1.626655946	0.227929419
1.932017729	3.574499661	0.109420103	1.329028484	0.109442516
1.565055337	0	0	2.298264976	0
1.498086354	0.127991471	0.192512369	0.141750471	0.192558141
0.027229135	0.03031933	1.135960034	2.166217799	1.135950691
2.187752536	1.951719978	0	2.636715673	0.567638287
2.296184356	0	3.41558288	0.35943889	1.5395973
1.229208023	0.056489704	3.16414789	0.061711439	1.25308942
1.612384522	2.30563687	0.248223315	0.178509736	0.248287837
0.104705906	0.118877891	0.177330345	2.603364799	0.177371439
0.16156449	0.185984805	0.295223513	1.77483239	0.295305795
1.084388265	0.02034859	0.027839731	1.690694343	1.6958689
2.033632582	0.100779948	3.426170923	2.078926573	4.098367432
1.39506908	0.099896562	1.447644527	3.542375014	0.146523551
1.61716473	0	0	1.211002544	0.697488309
0	0	1.972138247	1.973673055	0
2.216471103	0.043840881	0.061199837	0.047776201	0.061211207
0.05339678	0.059852713	0.084720167	2.319263089	0.084736694
0.025621614	0.028517679	0.039288871	2.156611475	0.039295831
0.508926802	1.307951877	0	1.816449086	0
1.58463659	0	0.999702475	1.584612745	0
1.032705778	0	0.546242076	1.366993353	1.005098899
0.771525091	0	0.761952165	2.464399674	0
0.060121921	0.067505268	0.096193402	0.073902289	0.096212603
0.154825739	2.819453723	1.801165472	0.198927502	3.339604766
0.07758665	0.087497062	0.126898612	1.376355036	0.126925482
0	1.36841878	1.35727006	1.76037073	0
0.052387663	0.058706634	0.083014957	1.254999193	0.083031095
0.014088804	0.015635119	0.021305599	1.666503629	0.021309223
1.281339774	0.069895077	0.099807739	2.369523428	0.099827804
0.041764288	0.046676246	0.065317655	2.251982184	1.209170307
0.077335418	0.087208266	0.126447358	2.016383435	0.12647411
0.037835199	2.220205885	1.813260076	1.184527176	2.579590524
0	0	0	2.237146052	0
1.501429448	2.900158615	2.215904468	2.483401195	1.899065147
0.097014661	2.098257456	2.166728458	2.561976973	0.162710319
0.112504142	0.127977054	2.259240442	2.201107947	0.192533919
1.315742868	3.740216264	0.113501208	1.340195184	0.113524641
0.871532821	2.145759617	0	1.824746829	0
1.594505171	0.001943397	1.595638594	1.008608419	0.002618211
0	0	0	2.800820122	0
2.517488256	2.794776342	0.053399788	1.168100577	0.053409545
0.013836899	0.015354576	0.020918334	1.067698686	0.020921888
1.043216223	0.010357554	0.014051078	3.062297122	0.014053427
1.323098443	2.849938282	9.264365936	1.307961076	9.362917498

2.425577617	0	0	1.446584756	0
0	0	0.865668501	2.810451951	1.418641434
1.240991394	0.710127885	0.706482408	1.222695888	0
2.71085822	0	0	2.31995261	0
1.706114635	1.108413897	2.745364436	1.715874492	2.477909102
1.8002086	0.839085958	0	0	0
1.215313599	0.689564067	0	0	0
2.034347796	0.006646313	0.008988199	2.624296405	0.008989684
3.289840334	0	0	0	0
1.190867978	0.04676648	0.065449031	1.20396944	0.065461301
1.177897757	0.043502825	0.060710212	2.235542323	0.06072148
2.30468491	0	0.9856076	0.985786991	0.985608598
0.983937115	3.436740625	3.558432282	1.978351686	2.9743329
0.589705749	0	0	1.055063452	0
2.499499511	2.716372336	0	0.931636633	0
2.199389862	0.040208886	0.055954276	2.218378209	0.055964557
0.116600092	0.132769734	0.200572094	3.003234366	0.200620425
2.329750236	0.001462624	4.010265887	1.592677141	4.33228244
1.813783831	0.21964871	0.360086592	0.247457879	0.360196327
1.618384826	0.006842613	0.0092552	1.620898273	0.00925673
1.745748806	0.199189942	0.320181605	1.82648362	0.320274045
1.044491429	0.010665179	0.014472165	1.640803101	0.014474587
2.105783709	4.512680482	0.828195988	2.346005775	0
1.258137733	0.063902314	1.286287534	0.069909295	0.090790545
0.932272718	3.603275432	0	3.221225785	0
0.863218147	0	0.856052853	2.386294724	0
0.030448689	2.771239191	0.046965542	0.036906863	1.152126763
0	2.976174333	0	1.563489228	0
0.876190833	0	0.869511267	2.146527448	0
1.199208836	1.207212772	0.068520514	1.833456083	0.068533442
0.131152765	0.149872339	0.229996121	0.166670919	1.673150932
1.401954393	0.10174681	0.149451977	2.083340915	0.149484959
1.595126477	0	0	1.872165046	0
2.823807092	2.956360948	0	1.967370229	0
0.519064422	0	0.956471342	2.66024486	0.956504736
0.0202709	0.022531353	1.101074691	1.099094639	0.030888737
0	0	0	0	0
2.005640548	0.001083663	1.004867845	1.590680055	1.590917636
1.19814954	0.048604375	1.217814176	1.832143903	0.068142226
0.891265234	3.929298847	0.885198354	2.168223388	0
0.792265636	0	0	1.713902209	0
0	0	0	2.53931142	0
0.044299438	1.827962052	0.069499155	2.596475213	0.069512295
0.194657338	0.225843207	0.37247532	0.254725689	0.372590689
2.622851657	0	0	1.813506108	0
2.22031588	0	0	1.485987619	0
1.834521662	0	0	1.822156048	0
0.428326441	0	0	2.709183648	0.421397054
0.017061303	0.018948283	0.025893077	1.083439476	0.025897527
1.014665641	0.003500227	0.004721035	3.480969193	0.004721807
0.018427704	0.020472965	0.028012857	0.022209495	1.091844583
1.35159636	0	1.72136587	1.337726525	1.72137577
1.157385142	0	6.901957346	0.639131842	7.020259476
0.068874139	0.077502513	0.111413935	2.739770109	0.111436845
0.374897363	0	0.369247095	0.361889429	0
1.343016799	0	0	1.722766312	0
0.681029452	0	0	1.554130298	0.670663935
1.486023083	4.78588977	1.880601745	0	1.880605475

1.180393699	0.04412987	1.197787515	2.567845588	0.061630077
1.491515915	0	0	0.60673468	0
0.072132297	3.006962036	3.081373466	0.089168581	3.081383955
2.362138876	0	0	0.904988272	0
0.049684737	2.882523383	7.987737665	1.866460674	7.720286371
1.108904829	0.026356349	0.036244229	2.471579757	0.036250606
2.414174275	0.017638395	1.681208835	1.077706585	1.079140797
1.691925094	0	0.54554261	2.286984681	1.352487169
0.228392837	0.267063826	2.217624546	0.30347969	2.217592472
0.065222901	0.073326647	0.105024008	1.95125074	0.105045338
1.676797227	4.197514293	1.688832932	1.08386989	2.112764455
2.656561398	0.06823496	1.305685972	1.295424003	0.097314855
1.069480983	0.016717006	0.022800773	1.672071819	0.022804664
0.135595811	0.155116973	0.239203552	1.652542268	0.23926487
0.011521046	0.012777125	5.177927306	2.395565562	5.818867779
1.553206305	0	1.549623201	0.971530281	2.956796447
1.856807866	0.058692278	0.082993619	2.6442052	0.083009753
0	2.279717079	3.376399265	2.448815458	3.760102776
0.98378153	0	0	0.982896201	0
0.06080477	0.068283707	0.097369059	1.927286112	0.09738854
2.471593053	0.101811678	0.149555949	0.112196054	0.149588959
1.035000186	0	0	2.73310301	0
0	0	0	2.141924623	0
1.747739359	0	0	1.956660045	0
1.452444359	0.115434738	0.171658622	1.491915191	0.171698015
1.46212921	0	1.449816276	1.859141593	0.890617328
0.071472651	0.080478945	0.115997038	0.088325711	0.1160211
0.048870967	0.054717118	0.077105402	2.623574775	0.077120212
1.061791171	1.660076785	0.020222219	3.259240318	0.020225649
1.035799146	0.008570578	0.011609328	1.629911071	0.011611258
0	0	0	2.386892036	1.601352731
0.924596068	0	0	1.897742069	0
1.546278288	0	0	1.955603274	0
1.691054936	0.022073607	1.705191483	2.447845517	0.030249403
1.561858762	0	0	1.560200131	0
0.073005171	0.082236124	0.118713969	1.354354535	0.118738723
1.904743713	0.069786725	7.938556772	2.368985673	6.962523016
1.626974413	0.00861755	0.011673416	2.858869144	0.011675357
0	0	0	1.043014035	0
0.64445892	0	6.397497985	1.127825274	6.699324697
3.132118042	2.722821498	6.818821225	2.463131671	6.601168693
1.333527588	1.314216834	4.687704778	2.516278257	4.716480733
1.238792381	0.05893821	0.083359237	1.882432449	0.083375454
0.484130707	0.475773472	0	1.53616199	0
1.259678644	0.064298987	0.091367769	1.278757001	0.091385833
0.032949757	0.036742675	4.249080827	2.794925369	4.155333328
0.003391334	3.021815675	8.866311282	2.343780382	9.275034796
1.883577758	0	1.868127423	2.191729084	0
0	0.680610985	2.300822471	1.184592774	2.646761812
0	0	0	0.752297752	0
0.45985488	5.595654668	1.206379611	0.878935909	0.877417335
1.166585735	0.040666908	3.589938273	2.549362273	5.186209931
1.937871149	0	0	1.522385378	0
1.975083316	0	0	2.293994992	0
0.900922907	3.028317774	7.157129393	0	8.11153335
0	0	1.881607607	2.720627681	0
1.724780593	0.029309811	8.661058938	1.128604919	8.124636828
0	2.321853283	4.842710061	0	5.670308791

hsa-miR-338-3p	hsa-miR-200c-3p	hsa-miR-3682-3p	hsa-miR-376a-2-5p	hsa-miR-9-3p
11.4931023	6.843768889	2.433760012	0.064628213	2.439172895
13.30622302	7.235445696	2.893225972	1.201971592	1.857777287
9.493155422	6.008796119	3.482844223	0.021218076	2.741342114
10.67662503	7.406976756	3.671110394	0.011158717	4.613415202
11.6469248	12.19115435	2.021988749	2.976522594	3.085655449
8.923190568	6.229132268	3.933148975	0.030559688	2.209661211
10.16883257	6.555772954	2.156040145	0.091102981	1.491519879
11.0412019	6.82618217	1.669617932	1.063430465	4.106070271
11.13980239	7.586655403	3.212659227	0.932862791	0
10.59467146	7.648961851	3.920217455	0.028819076	1.768222958
10.95066485	7.983556001	1.056004236	0	6.163261838
9.620345855	8.522389747	3.155090073	1.558597065	4.910621187
10.77929255	5.210406252	2.43148862	0.06428218	1.343504947
11.50291534	11.57416467	4.220202954	0.983303673	2.97304448
11.59428297	8.708568101	2.704552868	0.106299872	0.185187676
14.80165847	8.017311793	4.448393975	0.025948443	0.040630454
10.0062113	6.596200379	2.731072144	2.835591205	2.844188182
8.577059904	7.567914	2.049450258	2.169034231	1.318818547
9.419216378	5.973908449	1.254894385	1.841358953	0.077243306
10.70374064	7.354010369	3.491863519	0.131820883	3.23498091
11.47270586	6.615553092	0.161253212	1.454867356	2.668039551
10.42273051	7.086692044	2.533403973	0.151925356	0.282151666
9.737648235	5.429770161	2.44622752	0.017471252	1.696223777
11.00519487	6.579020483	0.135106614	0.08438037	2.913203189
10.34728345	6.776114916	1.449133358	0.083663006	2.114995072
8.922513818	6.671162523	4.619491779	2.404213628	2.123866462
10.74360031	7.982416758	3.875163208	0	1.972106684
10.75672365	7.565809592	3.080369083	0.037359001	0.059363912
9.793619957	7.333407311	1.904835133	0.050746765	0.082055849
10.08245824	7.654949405	2.987399651	0.024420669	2.764628215
10.93880491	13.0715404	2.717491752	3.33420998	1.786887062
12.05851655	8.213830386	3.584515917	0.999726201	5.699968962
10.69505871	8.125395038	2.809904941	1.039488449	1.001591049
10.89579093	7.605961192	2.229687964	1.313918549	2.227108805
10.87404019	7.805005855	1.944503123	0.057098686	8.227884531
10.59976794	7.304835348	4.344883993	0.145680353	5.535203884
9.698174222	6.447008095	2.492206545	2.997299748	0.122579103
9.625098915	6.989273718	2.315039369	2.095456218	0
10.2066064	5.794907703	1.264842141	0.049792908	3.174895544
11.30345213	7.572630444	1.670329001	0.013444858	2.684492154
9.971984657	6.565366586	2.733213117	0.059076257	0.096576322
8.356013002	6.666009198	2.870624405	0.039739417	2.875005059
11.3068531	5.976960527	2.045546969	0.073318205	0.122146707
11.58887898	10.99054445	0.054543965	0.036015572	2.246668107
10.51573855	8.001543728	0	1.925815283	1.505714551
8.975056767	7.413909694	1.89942682	0	2.215951994
10.42016609	9.706610604	4.251888394	0.091794422	2.166450102
10.83310496	7.831236389	3.047611007	0.106288348	0.185164666
10.15840136	9.650957465	2.783115905	0.066469313	0.109730995
11.12366381	7.730092723	4.642216574	2.157167162	1.416605391
9.453120944	6.262126128	4.013218008	0.00167867	2.011636719
12.08992705	7.385369724	2.459157731	0.673640828	0.656997599
9.130566048	6.663020589	3.243082972	0.032806201	4.427750544
10.41879762	6.450485672	2.090449841	1.062826091	5.236113275
9.881414698	6.183425487	2.650382271	1.042521771	0.013670553
9.638091963	5.608565669	2.964921687	2.294627019	1.687123627

9.623984232	7.37242462	2.350310975	2.035294816	0.595067344
9.546088882	5.68122102	2.965286919	0.874227479	0
11.18432331	10.81713046	2.985742959	0	2.350305355
8.429661769	7.821475608	4.250253349	3.631965952	0
10.03985854	7.111684341	3.486289113	1.102800963	3.336172294
13.01254921	7.049844078	4.642300226	0	0.834248927
12.39720576	7.572113056	1.860467808	0	0
10.47293636	5.802988738	1.03015338	0.005732083	1.621530125
8.951854747	8.336913199	4.282459864	0	1.965599354
10.22868884	7.383168121	1.836409852	0.039815103	6.315736674
10.983327	6.370391668	2.251383322	0.037074905	0.058890811
9.926062511	6.385826995	1.980872939	3.441954459	0
10.9503624	6.779191733	2.560531087	0	0.982716999
10.87914784	7.312313959	2.622573266	1.084080516	0
10.81297847	7.132941725	3.066444106	2.501449109	1.907030717
10.06953154	6.452261241	2.832361906	0.034303591	1.802964503
10.94362863	8.194205469	2.72911422	1.503708136	0.19284231
10.3606295	9.016410593	3.710319835	1.006040538	3.469407982
8.702187057	7.768759898	4.023162555	0.177779762	0.342734967
15.70045661	8.729617523	2.851457502	1.028157787	2.364615369
11.87162586	8.156195177	0.28488378	0.162119751	2.615768651
10.68432249	6.947823799	0.013498735	0.009186028	0.014079859
10.76568726	6.393160587	2.549614071	0	0
10.96483914	5.79369233	2.699538172	0.054111794	0.087884155
11.38291787	7.411446953	4.588104412	0	3.473321331
15.54187711	7.04998671	2.374509079	0	2.116051099
8.684547584	7.021895399	3.020529758	2.491374398	5.40134606
13.20903283	7.17948617	1.97578136	0.983199566	0
15.84410063	9.347964715	3.603471671	0.877883975	3.493871846
9.90757882	6.698411528	2.613895722	0.04158003	0.066434035
8.560031249	6.551349185	2.358093552	0.123683144	1.675793725
11.17366617	7.339248591	2.119175931	0.085165099	0.144158417
9.999026303	6.560081762	2.941789693	0.695357417	0
9.131196756	6.781005142	2.416585058	0	4.815136592
9.703257321	7.675962494	2.950939537	0	0.506428876
11.06190551	6.310681352	2.13205564	0.019331691	0.030014779
10.86982026	7.733818141	1.956425326	1.549555766	0.967049599
13.12775668	8.192861344	1.004919842	1.004476593	2.591946438
10.85943678	8.061952601	3.89619979	0.041355762	1.847594612
9.208635179	7.594007782	3.407638025	0.892775729	0
9.356749941	8.082013214	3.35452014	0.794857439	0.781357475
9.423271659	5.699156315	2.949798364	1.548058242	1.542976419
11.15462942	6.176887823	1.223692892	1.198159849	1.2235317
10.91477604	8.272084849	3.191573961	0.182489962	3.223082361
9.328344119	7.441919411	3.100712193	0	2.947581858
9.729311643	5.991288221	2.876247571	0	6.620231422
11.77368981	8.355109188	2.603050773	0.871431958	0.861646226
10.49719364	15.99376004	2.855355668	0.432658259	0
11.10083376	9.584209002	3.122102893	1.674488051	6.859856242
14.37416758	8.292514353	2.829990047	1.014435959	3.608906914
13.23700147	7.819983891	3.131729735	0.017577332	2.939519942
11.19561466	7.429417205	3.263278507	1.754733143	2.02796186
10.19663967	5.980432283	1.49476102	0.654765918	2.983070899
9.940631665	8.4068362	4.068111248	0.065352414	2.444016833
11.93147827	7.352973667	1.542384177	2.318886948	0.757046733
11.25111883	7.858587365	1.321097782	0.801075297	2.017262649
11.38146264	6.496736251	2.7887132	0	2.473645871
9.141162069	6.119329144	2.867923129	0	1.475305892

13.10264802	10.70388553	0.057048779	1.177144876	7.790716416
9.859339135	6.683758245	2.369588533	2.267020471	4.216874164
9.227247841	6.432247812	4.679751627	0.068421346	2.464531133
8.701373604	7.852403616	2.267025339	0.483082859	3.127032169
10.02704278	10.23254507	2.319024917	0.047237037	1.250980737
11.00557576	7.265288266	2.154064491	0.022585571	6.539493275
9.558278013	6.98513132	2.919853575	0.015157619	1.681521462
8.958331009	8.834777208	4.94116136	0	1.000646141
9.534580136	6.720061879	5.052031503	2.599546476	2.21509211
9.900940076	5.743925137	2.752380616	1.288782738	0.101590048
9.877633383	7.336960988	2.704317926	0.016360342	2.439656361
10.49937238	7.289235522	2.99551631	0.057702812	0.09416034
11.36452469	9.613121487	4.018436643	1.068338463	8.301520351
10.81087395	7.661789222	0.215463846	0.127818756	0.229423391
11.49135656	6.647026595	3.543995443	0.010997474	4.336078588
9.953678529	8.20974119	5.20325223	0	4.954305394
10.10753466	6.358890878	4.073700622	0.049780957	1.264718255
10.86860251	6.386315644	3.388474713	0.651089286	2.576839184
10.61973679	7.502864804	0.982493931	0	4.294798983
9.736035386	7.962256872	1.948524524	0.057743161	1.307875397
10.26243087	6.984034177	2.568179957	0.085217731	0.144257755
9.709151927	8.272965859	3.900685323	0.561547813	0.544445178
12.50902735	7.571522987	3.303730625	0	0
9.578557646	5.826478839	2.479033421	1.757340167	2.313395984
11.51853374	8.768076062	0.15623214	0.096227549	1.520164007
9.66361589	6.355637057	2.167550738	0.897899698	1.851784097
11.6079427	9.555768956	3.958238805	1.315492923	2.460380503
9.613600909	7.590534352	3.667499535	0.046467267	2.923000282
9.420811923	7.073199414	2.087508831	1.060781866	1.067142925
10.74136074	7.593920992	3.642188916	0.007386992	8.867713964
10.52355895	9.935276989	3.527378653	0	0.710302965
10.59543006	4.823540781	2.209750791	0	2.468505879
10.08267619	7.541950867	0.964570871	0	1.541777263
10.38931139	6.899773459	2.129414171	0.018941756	3.14413739
9.422672482	6.340622138	1.559178961	0	0
10.0812208	8.137667812	2.020189974	1.948663687	0.114732633
10.71401347	6.029637623	2.396672433	0.058986658	1.314637782
10.03893288	6.454879748	4.306336538	1.035419625	2.863570233
8.98135642	15.08142494	2.969509251	1.443757801	1.034761206
10.1175896	6.963597667	3.194406101	0	1.484339369
9.940167491	6.534729757	4.25220741	0.021285914	3.867283698
11.45430705	12.18270039	3.571618978	1.879652247	5.344400787
13.71261793	15.8530896	2.337241082	3.097135886	0.080744703
10.68009607	6.120251992	1.745006783	1.298485971	1.742799774
10.0193183	5.566691024	1.289920995	1.254716543	1.930319175
9.933619888	6.58103538	3.037605049	1.148213663	1.784456177
10.61259454	9.162035671	2.022296671	0.003240036	1.016984745
10.28237049	5.924659148	3.173347703	0	1.463892444
10.90273395	7.086239475	1.84653465	2.156901695	5.13915134
9.960777896	6.497847908	2.214398451	1.302192163	0.750274345
8.291937765	7.187324924	4.707334672	0.46415872	5.710957878
9.850222638	7.275359502	2.835084462	0.034689285	1.805406519
11.82860927	7.010498705	3.504720849	1.527536697	0
13.38830862	7.45666448	2.554524252	0	0.978485395
10.61338217	6.868438326	3.298871368	0.902312967	0
10.9241308	10.12877152	3.673044131	0.713927446	6.940188337
8.343975015	6.548060009	5.95061958	0.025092601	4.368349386
9.394704495	5.856938056	1.734155474	0	0.804005681

hsa-miR-6783-3p	hsa-miR-515-5p	hsa-miR-519b-3p	hsa-miR-4536-3p	hsa-miR-3677-5p
0.060123833	0.106327588	0.101583778	0.053455159	2.426482633
0.040093114	0.068709561	0.065857306	1.188465035	0.062626428
0.01986158	0.032995421	0.031723668	1.692571575	1.111603164
0.010460701	1.058637186	0.016494783	0.009406999	3.405551576
0.064641982	0.115141599	0.109921222	1.920237625	1.369022441
0.028567302	0.048093593	0.046179424	0.0255811	3.393712779
1.407547902	0.15528761	0.147737634	1.388275473	1.485549099
0.012494549	2.092006666	0.019756534	1.059565813	2.680844244
1.507970778	0	0	0	0.927019927
2.484688239	0.045253071	0.04346265	0.024139172	4.215758931
2.032941204	0.584383298	0	0	2.338165508
0.113277145	0.218452648	0.206715232	0.099572232	3.134338705
0.059804798	0.105709907	0.100999022	2.323084485	1.340816451
0.983666052	0	0.981701544	0.984260884	1.976186997
2.108529713	0.184979832	0.175540452	0.086705075	2.68986434
1.722343383	0.040595904	0.039005482	0.021757229	1.747683405
1.077470938	0	0	0	2.861216698
0	2.04468187	0.358899987	2.068978863	1.321429775
0.044708803	2.327185494	0.073914706	0.039887596	2.651597152
0.121532178	0.2376479	0.224512896	0.106647587	3.895646647
1.441198594	0.170560405	0.162056158	2.807255299	1.528454436
0.139708042	0.281783474	2.535106802	0.122149185	0.247208929
0.016363228	1.091979559	0.026010915	1.077855336	2.117958459
0.078286071	2.913045755	2.563589677	0.069326101	1.449121555
1.375604579	2.566035386	0.134480399	0.068753254	1.445240448
0	0	0	0	3.213478018
2.560944869	0.97899525	0	0	4.216705292
0.034889234	0.059311339	0.056895425	1.164438387	2.848938538
0.047301833	0.081979676	0.07848659	0.042176257	0.074539861
1.112868329	0.038131164	0.036645133	0.020487547	1.128522191
0.519363478	0	0	0	1.280842672
0.999732228	0	0	0.999742102	0
0	1.350769372	0.540271037	0.576236549	2.262134662
0.778301989	1.975646283	0	0.785040591	2.802894656
1.259682345	0.093012015	0.0889642	1.24830819	0.08440038
0.134071348	0.2678132	0.252365281	0.117352933	0.235528597
0.068343453	0.122455967	0.116830537	0.060652881	1.390673207
0	0.81543887	0	0	1.752148941
0.046418982	0.08033858	0.07692631	0.04139733	1.895171319
0.012599608	0.020702184	0.019925512	2.071972356	3.686307701
0.055001435	0.096484031	0.092257521	0.048956763	3.746697296
1.182285429	0.063285036	0.060686407	0.033156389	3.286828251
0.068126097	1.393053207	0.116422831	0.060462871	2.039118174
1.773787079	2.848260896	1.190917934	0.030089288	0.052137483
1.516791762	0	0	0	2.494034583
1.505031487	0.924424069	0	0	1.492943701
3.294622386	0.156612671	0.148981382	0.075231211	3.441119246
2.108477876	0.184956857	0.175518993	0.086696001	0.165065549
1.923722606	0.109623417	0.104702881	0.054943209	0.099172802
2.161987732	0	0	0	1.818340809
0.001575868	0.002546765	0.002455095	0.001420132	3.012516886
1.195535813	0	0	0.687585409	2.280883047
0.030657434	0.051778411	0.049701722	0.027439492	3.043165941
0.012375098	0.020326403	0.019564467	1.058999941	2.089358298
1.041551344	0.013659622	0.013155214	0.007527588	1.046825299
0	7.016989613	7.579018145	0.795432591	1.690291053

0.617876711	0	0	0	1.433214769
0	0	0	0	2.133550366
0	0	0	0	2.992760834
1.597310052	0	0	0	3.461255848
1.100339414	1.114284062	0.03243216	1.096360012	2.739897036
0	0	0	0.853329849	1.380919193
0	0.683529497	0	1.240959138	3.193280367
0.005377819	0.008740696	0.008421532	1.025730957	1.030066876
0	0	0	0.977634845	3.76911971
0.037169912	0.063411779	0.060807279	0.033218644	4.201488338
0.034625328	0.058838708	0.056444354	0.030962609	2.57852879
1.569857748	0.985494813	0	0	0
2.787461654	1.977058153	0.982661727	0.985089121	3.974430009
0	0	0	0.609473308	1.934963062
0	0	0	1.925956395	3.219558406
1.765108089	0.054246266	0.052059476	0.028676547	4.162009572
0.101808428	0.192623097	0.182674616	0.089705027	0.171672005
1.005906831	2.331090085	3.009608707	0.001069143	3.179427491
0.162958427	0.342248548	0.320490045	0.141831331	0.297116684
2.842401162	0.009000186	0.008671351	1.026486428	2.363616718
1.70223653	0.305110194	0.286606966	0.129942898	1.87707288
0.00861391	0.014068589	0.013548597	1.63218375	1.642859987
1.397088796	0	0	0	2.079101805
0.05041451	1.288187699	0.084018263	3.453868949	0.079748355
2.914565361	0	0	0	2.703550914
0	0	0	1.435027437	2.377154311
1.133761385	0.045558717	1.153485177	0.02429472	0.041702898
0	0	0	1.980781992	2.781369212
0	0	0	0	0.868081745
1.190555151	0.066374316	0.06363184	0.034669447	0.060523925
0.114150198	0.220458466	1.672467346	2.583977593	1.663592882
0.079005678	0.144007752	0.137138557	0.069952441	1.453368449
0.700286414	0	0	0	2.50299941
0	0	0	0	3.591633456
2.118601654	0.50634827	0.503747207	0	1.8124443
1.089572271	0.029989855	0.028841468	1.086046081	2.130390346
0	0	0	0.971495162	0.967010281
1.590046144	0.001419191	0.001368242	2.005098105	3.006986359
1.80068133	0.065997123	0.063272295	0.034485194	1.218321985
1.46129754	0	0	1.874904049	1.846444887
1.344891043	0	0	1.755090697	3.246323981
1.549025781	0	0	0	0.965625267
1.193070507	0.067317995	0.064531265	0.035129831	2.612687404
0.16717977	0.353738832	2.012090257	0.145387825	2.695081951
0.867883757	0	0	0	2.1199655
1.496232276	0	0	0.926951363	3.198261406
0.873947399	0	0	0.878137308	1.81567156
0.439091204	0.417026307	0	0.450443104	2.174305628
0.015246765	0.025152169	0.024199192	0.013695487	2.109961221
1.014114132	0.00459232	0.004426231	1.600606769	3.607981798
1.081513912	1.696898099	0.02617199	0.014783145	2.445117283
0	2.281442912	1.330177854	1.771481247	2.503961765
0.660084688	5.320873747	5.735263635	0.66920413	3.215944616
0.060791435	0.107622122	0.102809108	0.054040705	0.097397785
0	0	0	0.3972041	2.201512487
0.804697806	0	0	0	2.276302274
1.596538745	0	0	0.69815446	2.484286575
2.218957605	0.910759435	0	0	3.189919423

0.035114774	0.059715562	0.057281181	1.165482367	2.251267849
0	0	0	0	1.741337749
1.309456837	1.366174301	2.01550663	1.295526525	1.363049502
0.489403687	0	0	1.620823476	2.417730596
2.255743747	6.126802179	5.609470825	0.039307672	3.14325559
0.021137446	0.035183255	0.033820754	0.018960622	1.118825111
0.014201127	0.023390162	0.022507389	0.012759378	0.021498776
1.965321822	0	0	1.739992998	3.463538966
0.194846748	0.433237685	2.197540344	0.168574748	0.370302449
0.057617707	0.101492007	0.09700429	0.051255567	3.015346214
1.075915622	0.025284663	0.024326385	1.668385224	2.436681674
0.053733061	0.094070786	0.089968687	1.250843588	2.381956283
1.66192352	0.022152839	0.021319056	1.064159962	4.502005464
0.117903554	0.229146797	0.216637895	0.103540246	1.686378324
0.010309789	3.084121744	3.253630673	1.049205063	4.476566847
0.973965219	1.54948031	0	0.974906128	1.962036536
1.842577114	0.080318042	0.076906781	0.041387566	2.663845437
1.167041913	1.488706149	2.860726501	1.181218697	0
0	0	0	0.984944549	2.29821152
1.262530285	0.094141558	0.090035825	0.047874142	1.943975618
0.079053937	0.144106959	0.137231866	1.36443741	0.12955641
1.051495718	0	0	0	3.225771978
1.237609419	0	0	0	3.58819229
0.600183861	0	0	0.61018657	1.406130737
0.089134489	0.165166229	0.157003281	0.078748863	2.966696742
0	0	0	0.903351118	2.169314011
0.063046924	0.112015723	0.106965803	0.056017728	4.188621466
1.826176041	0.074648384	0.071512909	0.038677595	1.87470426
1.653479765	0.019650883	0.01891538	1.65035609	2.086456109
0.006928757	0.011287706	0.010873129	1.62301371	3.054793976
1.260770914	0	0	0	4.919892675
0	0	0	1.913926238	1.48681258
0.968300692	0	0	0	2.273771229
2.430288684	1.099764991	0.028247218	1.084330933	2.454559026
0	0	0	0	0.978824555
0.064375903	0.114619073	0.109427299	1.298929407	1.367468336
0.054918705	5.042120382	4.692133613	0.048884029	2.995780095
0.006966582	0.011349971	0.010933048	1.033305713	1.038970873
1.755965396	0	0	0.600672788	3.271333002
0	4.062949412	5.147055953	0	3.203675896
2.118763086	4.629066177	4.973855466	1.094629253	4.248214395
0.992722265	4.089451995	3.288161227	0	2.485113577
1.228129573	0.080669945	0.077241393	1.218317779	2.332047282
0.494703862	0.47188293	0	0.971899104	0.903867338
0.050718839	0.088373841	0.084561667	0.045188193	2.360899441
1.144541079	1.784453062	1.166150575	0.026260964	0.045221869
1.015131452	8.353011307	7.059345912	0.002739431	2.608449135
0	0	0	0	2.665401315
1.217336171	2.090814185	1.548811733	1.230280573	0.672732184
0	0	0	0	2.218399654
1.562900566	0	0.870564741	1.835577486	2.379623057
0.032408409	3.261244214	3.582696416	0.028994973	1.801955768
0.952128455	0	0	1.943676116	1.930159325
0.980724405	0	0	0.981424748	1.558929566
0.904282156	6.133152861	5.78750403	1.477118592	3.165107865
1.245867321	0	0	1.651412032	3.600272302
1.115935464	6.108301487	6.054233208	0.021046139	3.995291866
0	3.770489943	3.391850425	0	1.736217547

hsa-miR-525-5p	hsa-miR-512-5p	hsa-miR-520d-3p	hsa-miR-760	hsa-miR-518f-3p
0.111651886	0.105945265	0.107128075	2.40368185	0.103837553
0.071887743	0.068480405	0.069188946	3.095470787	0.067214829
0.034402968	0.032893546	0.033208372	0.026993828	0.032329967
0.017836046	0.017081401	0.017239008	3.400630331	1.058757039
0.121010834	0.114720556	0.116023334	1.99571984	1.372439454
0.050218169	0.047940066	0.048414618	0.03910502	0.04709135
0.1638432	0.154676607	0.156568337	0.121048571	0.151316163
1.069166547	0.020464705	1.070028997	1.068435399	0.02012373
0	0	0	1.907530855	0
0.047239231	0.045109503	0.045553253	2.778715066	0.044315734
0	0	0	1.065277993	0
0.231922685	0.217497623	0.220457455	1.627993204	0.212261365
0.110996618	0.105330257	0.106504786	0.084001102	0.10323722
0	0	0	2.560758292	0
0.195739886	0.18421399	0.186586234	0.142576798	0.180008055
0.042358655	0.040468422	0.040862425	1.74156977	0.039763437
0	0	0	1.399318938	0
2.526898069	0.361901035	1.72629363	1.338539318	2.53109261
2.663156406	0.076910394	1.254613117	2.302913903	1.255118409
0.252780679	0.236577391	0.239896162	2.790374712	0.230713536
1.530021734	0.16987128	0.172005397	0.132181999	0.166084
0.301043874	0.280428077	0.284633076	0.209585931	0.273020069
1.09075457	0.026957301	1.696209253	0.02217531	0.026501502
3.19452264	2.570367973	3.421620338	1.432337314	2.90867323
1.445067374	0.140640549	0.142317398	0.110651169	1.45016961
0	0	0	2.361860801	0
1.972221558	0	1.559317208	3.778231507	0
1.195332237	0.05911739	0.059716986	0.048008332	0.058045782
0.085881815	0.081698726	0.082567589	1.260267631	0.080148073
0.039777451	1.128886492	1.128703918	1.125325126	0.037353419
0	0.938736587	0	1.294080705	0
0	0	0	1.584612205	0
1.355938217	1.350641275	0.543265986	1.654993474	0.541170798
0.763279343	0.759880206	0	2.246979405	0.759301443
0.097543457	0.092686148	1.304161502	0.074281531	0.09088853
0.285720858	1.803115075	1.803459727	0.200201564	0.259646873
0.128789633	0.122001976	0.12340686	1.376996206	0.119501102
1.35828089	0	0	1.362875822	0
0.084149239	0.080064167	0.080912792	0.064483527	0.078549479
0.02155982	0.02064003	0.02083207	2.087120073	0.020295995
0.101218691	0.096143679	0.09719648	2.370384116	0.094266471
0.066177621	0.063076351	0.063721545	1.823127691	0.061923533
0.12833004	0.121572079	0.12297087	1.375787686	0.119081983
2.579264685	1.813770946	1.19051009	0.046243277	1.813425236
0	0	0	1.91809521	0
0.925456327	1.898918675	1.492248005	0.925621946	0.924232629
0.165262673	0.155995018	0.157907376	0.122019596	1.495251971
2.257350956	0.184191139	0.186562996	1.544198233	0.179985883
0.115149603	0.109226741	0.110454017	0.086977309	0.107040249
0	0	0	2.398277367	0
0.002647654	1.008801225	0.002562067	2.333289561	0.002498859
0	0	0	0.658208625	0
0.054084976	0.051611799	0.052126826	1.782264365	0.050690903
0.021167707	0.02026543	0.02045382	2.085580495	0.019927928
0.014215886	0.013619279	0.013743916	1.045799264	0.013395902
10.59996743	8.072256272	9.013721722	1.307637364	7.708148687

0	0	0	0.595580282	0
0	0	0.864754932	0.866322347	0
0	0	0	2.729851543	0
0	0	0	4.483169443	0.667504455
1.112770495	1.722945588	1.114165949	1.111268999	0.03305301
0	0	0	2.356282332	0
0	0	0	0.685027788	0
0.009092352	0.008715179	0.008794005	1.619928569	0.008573861
0	0	0	3.134503328	0
0.066310969	0.063202621	0.06384928	2.252995419	0.062047194
1.193828041	0.058646498	0.059240716	2.236047363	0.05758447
1.980832146	0.985487876	0.985511694	0	0
4.432077845	2.297929113	3.433205697	1.978312775	2.298001611
0	0	0	2.511010004	0
0	0	0	2.230411985	0
0.056676252	0.054070784	0.054613247	2.814405475	0.05310098
0.203980859	0.191815422	0.194317549	2.223815777	0.187381413
4.180271518	3.179749141	3.7105298	2.816198209	3.469329225
0.367864965	0.340459012	0.346016449	0.249161182	0.330708035
0.009362515	0.008973896	0.009055113	2.361709752	0.008828295
0.326725677	0.303593322	0.308301092	0.225050976	0.295312491
1.643086404	0.014026999	0.014155491	0.011599358	0.013796717
0.82917178	0	0	0	0
2.36860915	1.288271601	1.928316332	0.070265141	0.08581731
0	0	0	4.067464739	0
0	0	0	0.856682981	0
1.151237412	0.045414082	0.04586113	1.759752427	0.04461443
0	0	0	3.144863066	0
0	0	0	1.830920321	0
0.069428815	0.066154023	0.066835137	0.053573118	0.064937258
2.369017108	0.219491613	0.222488202	1.632892402	0.214191024
1.453335564	0.143452374	0.145171577	0.112742855	0.14039619
0.683313734	0	0	2.122074962	0
0	0	0	2.436840592	0
1.300399455	0.506061237	1.295047993	0	0
1.100477705	0.029897885	0.030182089	2.450672535	0.02938903
0	0	0	0	0
2.328714361	0.00141512	0.001427695	1.590688947	1.004916802
0.069031749	0.065778255	0.066454961	1.832614725	0.064569336
0.885858873	0	0	1.44725343	0
0	0	0	2.023295432	0
0	0	0	0.966281124	0
0.070422341	1.223630013	0.067786298	0.054314249	0.065857689
2.018186895	0.351861057	0.357693646	1.932074006	0.341635254
0	0	0	1.411723071	0
0	0	0	1.89348398	0
0	0	0	0	0
0.424076528	0.416694015	0	1.66960719	0
1.084515901	0.025075878	0.025311612	1.083541753	0.024653682
0.004775179	0.004579046	0.00462005	2.019741199	0.004505517
0.028352367	1.092580006	0.027380804	2.439172284	0.0266658
1.722309909	1.720641987	1.720668045	1.337427886	1.720861208
8.615030709	6.343062933	6.830093652	1.136176169	6.531156785
0.11302545	0.107234179	0.108434399	0.085456466	1.349591932
0	0	0	2.481443671	0
0	0	0	0	0
0	0	0	0	0
3.32105157	0.910713855	0.910868624	1.479219795	0

0.062423481	0.059520124	0.060124328	1.810558402	0.058440309
0	0	0	2.003663249	0
2.464952719	0.112723047	1.365924102	0.089640288	0.11045173
0	0	0	2.304336563	0
7.758738926	6.126014019	6.523602792	1.242289438	5.758726643
0.036691855	0.03507409	0.035411452	2.145337256	0.034470251
1.078679324	0.023319502	1.681506883	0.019211056	0.022928432
1.006720571	0	1.001124561	3.891883555	0
0.469992278	0.430699064	0.43859495	0.30573273	0.416930833
0.106524155	0.10113047	0.10224889	0.080783223	0.099136888
2.112462052	0.025207947	0.025444995	0.020750938	0.024783401
0.098663935	0.09374052	0.094762086	0.075095664	0.091918726
0.023073787	0.022086107	0.022292297	2.682934203	0.021716756
0.243533357	0.228128062	0.231285889	0.173734184	0.222545452
5.339026979	2.666422711	4.480939092	2.660192652	4.409264048
1.549774438	0	0.971002998	2.960498353	2.281664209
0.08412756	0.080043711	0.080892083	1.88183009	0.078529471
3.285331983	1.780986738	2.232422588	1.802146353	2.23414353
0	0	0	0.98287787	0
0.098738841	0.093810998	1.307662867	1.296093801	0.091987585
0.151880586	0.1435511	0.145271795	0.112816215	0.14049228
0	0	0	2.598826382	0
0	0	0	1.586966495	0
0	0	0	1.419042879	0
0.174434574	0.164505065	0.166552418	0.128264433	0.16087049
0	0	0	0.891283896	0
0.117689858	0.111608535	0.112868382	3.025254615	0.109364357
0.078146116	0.07439635	0.0751757	0.060052334	0.073004812
0.020462899	0.019592028	0.019773871	0.01616468	0.019266242
0.011744704	0.011254554	0.011356971	2.371659155	0.011070974
0.714170373	0.71009136	0	2.386145036	0
0.920536945	0	0	0	0
0	0	0	2.275842262	0
0.030612595	0.02928042	1.705549786	1.097217914	0.028782761
0	0	0	0	0
0.120455553	0.114200355	0.115495926	1.354945085	0.111892921
8.295492156	5.780526324	5.414751634	0.076827797	5.945166067
0.011809561	0.011316632	0.011419628	1.038127821	0.011132012
0	0	0	1.695695991	0
7.784335698	5.523684289	6.207594863	1.49977004	5.528939687
7.723418303	4.924875939	6.321998887	3.317728112	6.298539257
6.344365036	4.407960918	4.540901016	2.369807314	3.4309923
1.262509247	0.080394216	0.081246917	0.064740961	0.078872281
0	0	0	0	0
0.092637672	0.088067063	0.089015893	2.673906277	0.086374373
3.042827852	3.041070513	2.815285297	0.040176692	2.545038605
9.400191173	7.573434021	7.565865161	1.016633418	6.596105055
0	0	0	0.902998606	0
2.793228307	1.548213721	1.54840252	0.676014658	2.486824126
0	0	0	1.281185482	0
0.45479207	0	1.203145034	2.541944815	0
5.235199078	3.065209682	4.360005104	0.044503601	2.837180245
0	0	0	0.947711085	0
0	0	0	2.293939689	0
8.549371465	6.806841299	7.296176552	0.895947504	6.33913763
1.585095579	0	0.698341827	2.553791919	0
8.818520213	6.17831839	6.487802286	1.128771701	7.221143935
5.69097406	3.592015241	5.134450801	2.312288899	5.138713866

hsa-miR-498-5p	hsa-miR-431-3p	hsa-miR-137-3p	hsa-miR-2114-3p	hsa-miR-1292-5p
0.105551769	6.162791436	2.425848972	0.094570921	2.393473881
0.068244421	0.072717224	5.903914384	0.06160509	1.836717516
0.03278858	3.976507437	3.47916804	6.667478526	2.13372237
0.017028829	3.409463846	0.015712873	1.058467917	2.070931003
0.114287266	7.018629493	1.368733096	2.790613618	0.086173493
0.047781917	3.036187799	0.043846374	4.982551074	0.037173093
0.154048214	3.85162449	0.138730293	1.483453697	0.114070711
0.020401224	4.632762079	0.018813208	0.01859659	1.067514599
0	4.526471798	0	0.927126331	1.500223071
0.044961606	3.391571134	4.538046787	0.040779457	1.145702141
0	1.945539416	6.773506428	0	1.0703754
0.216516369	5.809509605	0.192889094	0.189796506	0.155968855
0.104939509	4.307021473	0.095322661	3.027576611	1.957046005
0	6.099692418	3.295466688	0	3.297551435
0.183426707	4.261495864	0.164346056	0.161831042	2.963285557
0.040337087	4.457056025	0.037064155	1.746986922	1.13122909
0	8.179501584	0	0	1.699134466
1.318400934	5.452433285	0	2.443714216	0
1.889124782	4.628846032	0.069967079	1.252657326	0.058810822
0.235477809	3.763997962	0.209100231	0.205661463	0.168248194
0.169162707	5.229346362	0.151941451	4.939950845	0.124418395
0.279036834	3.668686501	0.245948745	0.241674631	0.195731287
0.026872425	1.090038487	0.024751823	6.341565635	2.700805009
3.418308194	3.959600264	1.448723107	0.125804754	2.068019341
0.140083291	3.621571537	2.54679818	0.124650271	1.420311674
0	4.792454896	0	0.693522626	1.902789552
0	3.427355571	5.326170211	0	2.557706709
1.198018263	4.824264917	0.053957364	0.053286158	1.80458993
1.270116469	4.571619115	0.074257636	0.073294434	0.062329987
0.03788938	3.186165193	0.034830348	0.034414587	0.029617879
0	5.906714532	1.795002198	1.282114005	0.498185771
0	3.584503545	0	0.999700567	0
0.542130651	4.125771673	0	0.539510793	0
0	3.604986871	0.758950154	0	1.297289205
0.092350692	5.88599006	0.084074419	0.082962339	1.287763906
1.802876502	3.338972277	0.234348582	1.780434589	0.187142436
0.12153483	8.882005685	0.110074876	3.97911057	1.369978266
0	9.813099398	0	0.81493393	2.076708173
0.079781626	3.177016164	0.072793847	6.887088148	1.875565125
0.020575979	3.807738389	0.018973762	3.098475657	2.085294936
0.095793331	3.773116094	0.087155413	3.881717496	2.692358376
0.06286143	4.639846124	0.057528628	0.056807608	1.200673216
0.121106953	4.147869195	0.109695597	3.511561239	0.091029752
1.813731052	7.426121384	3.585323294	1.189433772	1.796860923
0	11.45401486	1.506417586	0	1.509883347
0	4.338115129	4.207969449	1.900816508	1.497006884
0.155359798	4.155449146	1.488841519	0.137826851	1.460706666
0.183403983	2.253288909	0.164326344	1.565484031	1.532502035
0.108818493	3.968654236	0.098778539	0.097434148	1.9692352
0	10.12787712	4.433705371	0	1.827526383
0.002531889	5.058414115	0.002342361	1.008783011	1.008510291
0	4.049916608	2.91102375	1.153978447	2.496125804
0.05144018	5.221127032	0.047172431	3.560961729	1.165788688
0.020202596	5.968179935	0.018630708	1.667762117	1.066868747
0.013577701	3.77187325	0.01253625	4.31572169	0.010740154
7.506267817	9.185815913	1.301289705	2.65654277	1.704283204

0	5.175055504	0	0	1.084511703
0	2.794876245	0	2.392463654	0
0	2.141246135	0.702546421	0	1.226615851
0	8.843223851	6.309590654	0	2.113797661
0.033522655	3.165526323	0.030840351	0.03047546	0.026259922
0	4.679149371	1.778287904	0.833832012	0.837719666
0	4.209771699	0	0	2.137239729
1.030100185	4.368927353	0.008029507	4.043566657	0.006889587
0	4.81653243	0.974096852	0.974131073	1.968090038
0.062987213	3.895878788	3.750787719	0.056919828	1.818689483
0.058448532	3.991810043	0.053532239	0.052866918	2.230622395
0	4.976778741	0.985489954	2.786968656	0.985944344
2.974512342	3.558415231	2.298466844	0.98273116	0
0	4.931998689	0	0	2.359308128
0	5.134715211	0	0	1.913411043
0.053890036	3.726627557	0.049397389	5.208908808	1.173327577
0.190985233	5.611477608	1.588496554	5.085506787	1.551408506
3.817430574	3.710614404	1.592940676	0.001742969	0.001515473
0.338623914	4.638118742	0.295491472	0.28998933	0.231785158
0.008946798	6.476005109	0.008267468	2.363383503	2.624582846
0.302036908	3.740929145	0.265188637	3.643164801	0.209852049
0.013984135	2.653065903	0.012910561	0.012763922	2.05849131
0	2.331300293	0	0	2.350382735
0.087183738	4.230192853	0.079443212	1.285428313	2.332099173
0	3.3476751	6.060037166	0	3.223512071
0	4.804518733	0	2.786983249	1.413974685
1.769370783	5.190861931	1.15270409	1.152402802	1.146647977
0	5.362741878	0	0	1.563814536
0	5.758098247	0	0	1.430340343
0.065927158	3.487546564	0.060301306	0.059541153	1.209924746
2.367682706	3.706121775	1.662808768	0.191460142	2.722642893
0.142881092	4.353785464	1.452963707	3.392339972	1.427780031
0.678641606	3.168257232	0	1.858765712	0.682905672
0	2.414584654	1.264658159	5.565254826	1.275405627
0	0.962821948	0	5.281956557	2.05108818
0.02980312	3.472784757	0.027436775	6.637655834	2.712887616
0	4.591236399	0	1.957068383	1.546897277
0.001410924	5.007819533	0.001305572	1.004898359	1.590591394
0.065552856	4.415925518	0.059962945	0.059207588	1.827513585
0	2.159647592	1.846577078	0	2.430798898
0	5.604191278	4.461140798	0	2.028646656
0	3.946146094	0.965636666	1.955256587	1.545290587
0.066863589	4.505231412	0.061147622	2.279301115	0.051546207
2.021854801	6.147581856	0.304786244	0.299040451	0.238439283
0	3.096774983	0	0	0.858346458
0	4.114085494	0.91613417	2.207237496	1.487386999
0	5.384534183	0	0.861376733	1.824971179
0.416369319	5.388171588	2.773567322	0	1.681028226
0.024997264	3.124648145	0.023032413	0.022764647	2.428114912
0.004565364	4.417195828	0.004222061	0.004175078	1.015317955
0.027039165	6.316387564	0.024904657	2.118053873	1.689538878
1.330148238	4.700112361	0	0.794334849	0.798811285
5.201775031	4.942391223	1.497669994	0.635765707	0
0.106834907	5.327031233	0.097011905	0.095696014	2.998201797
0	6.654200364	0	2.332303448	1.777525948
0	4.998692273	0	0	2.03782801
0	4.422209574	0.666078262	1.16844811	1.180189133
1.475273801	4.761754023	2.458900056	1.476456477	0.9130769

0.059318837	4.386813437	1.819746205	2.249806817	2.233800561
0	6.934043923	0	0.603376079	1.469008288
2.802824722	2.46276125	1.362767573	3.282667071	1.980090358
0	5.442758793	0.896719932	0.462610884	0.909852314
6.347731428	3.158415253	0.068884878	0.068001041	1.238333341
0.034961618	5.887236206	1.726605625	0.031774291	0.027368032
0.023246688	4.583221474	0.021426192	3.92102707	1.675277556
0	3.876025836	0.99993317	1.877626697	1.905927791
0.428099642	3.819404404	0.368076833	0.360562129	0.282872093
0.100758341	4.810715982	0.091592274	3.60808826	0.076449587
0.025128895	3.938220122	2.110398728	0.022883905	1.08282174
0.09340054	4.778564632	0.085014378	1.304544565	1.920205886
0.022017339	4.641165103	0.020297612	0.020063106	1.072761264
0.227081522	5.896801839	0.201934715	1.682571406	2.30058559
3.545346806	2.400945646	0.015483421	0.015306505	0.013251901
0	3.415028062	0	1.962205547	1.551452782
0.079761254	3.971565948	0.072775523	1.262403583	0.061115443
1.122596892	5.391011891	0	0	1.136128086
0	4.779492087	0.982540587	0.982564158	0.983084852
0.093470715	2.391190107	0.085077196	0.083949628	1.290990103
0.142979323	4.463417497	0.129011239	3.589638334	1.428041667
0	6.900510725	0	0	1.376332372
0	4.46630564	2.125449628	1.198715498	0
0	4.001739106	0	0.574404395	2.360583546
0.163825184	5.335857811	0.147283968	4.996272998	2.90705522
0	5.545595998	0	0.889599698	0.892443995
0.111189483	3.506580299	0.100888592	2.003115528	2.411806037
0.074136829	1.876388823	0.06771136	2.63950564	1.856679266
0.019531377	3.802424501	0.018013894	0.01780686	1.661181403
0.011220386	5.419205172	0.010364161	1.038683137	2.047109767
0	4.456801425	4.43663475	0.708914784	2.178477405
0	3.574380871	0	2.692360626	0
0	3.268270135	0	0	1.544154017
0.029187742	2.724723009	2.127639814	3.841641581	1.697562012
0	3.289868729	0	2.777572716	2.294996215
0.113769455	5.638973844	0.103182631	1.366132081	1.984653929
6.587638987	3.616802739	0.087015564	4.23168364	1.927415105
0.01128227	1.631668374	0.010421207	2.37404825	1.629431348
0	5.919701331	0.564519031	0.564597613	1.704452788
4.816895166	4.000209734	1.119417434	0	0.634744744
5.257633183	2.474232972	3.479660905	2.960655099	1.711315273
4.408996433	7.67486988	6.136528434	0	0.956494813
0.08011032	5.308642009	0.073089485	2.329983076	1.876657803
0	5.336343818	0	0	1.544856375
0.087751235	5.607784633	0.079952281	1.921970157	0.066985786
2.814543579	3.410933713	1.165272816	2.20916808	2.196110831
7.618939999	4.19642878	3.024087241	0.004477386	1.01642345
0	3.843407862	0	1.870283181	1.469923488
1.175511275	4.717280716	0	1.851912087	1.188740045
0	5.592240074	1.274485529	0.749247661	0
0	1.902494983	0	3.713972753	1.503325485
3.852299228	3.066601956	0.049971529	1.800968024	0.042280253
0	5.641195217	0	1.520572937	0.948314409
0	1.559062558	2.555009865	0	1.973503488
6.885168425	5.285608247	0.89420918	0	2.184600693
0	3.49453167	0.696334921	0	1.60251756
7.058717943	4.52467175	0.035811993	1.131757983	0.030442663
2.866037932	5.550598723	1.34292008	1.343383669	2.31767429

hsa-miR-615-3p	hsa-miR-1276	hsa-miR-466	hsa-miR-517-5p	hsa-miR-522-3p
0.085547788	3.192754977	0.100560571	0.111331041	0.106653369
0.056071007	1.824225428	0.065239544	0.071696914	0.068904727
0.027298033	1.103457449	5.83802847	0.034318737	0.033082145
0.014261897	1.648680624	0.016355908	0.017794	0.01717583
0.092335526	0.075854444	0.108796319	0.120656847	0.115500418
0.039557922	1.756783668	3.932535606	0.050090849	0.048224312
2.120884563	0.099770748	0.146117992	0.163325148	0.155808603
0.01706452	1.06515519	0.019588939	0.021325488	0.020578737
0	1.503564551	0	0	0
1.148349887	1.140209908	0.043073876	0.047120238	0.045375306
4.36936429	0	0	0	0
0.168742769	1.580537246	0.204215087	0.23110183	0.219267706
0.085070444	1.937253973	0.099982846	0.110678057	0.106033408
4.497973315	2.29990277	0	0	0
0.14460226	0.116726381	0.173522212	0.195086408	0.185633125
0.033487528	2.154399781	0.038659962	0.042253093	0.040704436
3.294652107	0.574948357	0	1.38793117	0.564401208
0	1.103150927	0	2.804494764	1.536664008
0.062775044	1.231953715	0.073209806	3.164392401	0.077395383
0.182333461	1.621908247	0.221721119	0.25185667	0.238561767
0.13402542	0.10856703	5.47710894	2.670728688	0.171148136
0.212910366	0.168146939	0.261712698	0.299862387	0.282941277
1.089935422	0.018879082	6.56317225	0.028108984	0.027109784
0.113149092	1.403775097	0.134291851	5.616029953	3.189479211
1.430507784	2.037114816	0.133040432	0.148297729	0.141644093
0	0	0	0	0
0	2.973768328	0	0	0
1.192306192	0.040518024	0.056371633	0.061837305	0.059476499
1.888889124	0.055172844	0.077731132	0.085647221	0.082218997
0.031484329	1.722933076	0.036322206	0.039678886	0.038232549
0	0.96564562	0	0	0
0	1.584632605	0	0	0
0.542003092	1.028499986	0	0	0
0	1.701128738	0	2.441116309	0
0.075208764	0.062150541	0.08808983	0.097270741	0.093289639
0.203331159	0.161063947	0.249093112	0.284623967	0.268891262
0.097934938	1.353122003	3.102725224	0.128407362	0.122842903
2.903822504	0.823889412	0	0	0
0.06527233	0.05412638	7.210803232	0.08392018	0.080572327
2.412403495	1.065702023	1.670261712	0.021508554	0.020755085
0.077914541	0.064326155	0.091344901	0.100933649	0.096774008
0.051751142	1.806808557	2.600738069	0.066004029	0.063462755
1.377314987	0.080039222	4.62949299	0.12794946	0.122409362
0.046787339	1.174853217	0.054263061	3.600423173	0.057237589
0	2.504491091	0	0	0
0	0	0	0	1.492221552
1.472308674	3.533582868	0.147344539	0.164738835	1.495324695
0.144585494	0.116713491	2.702902295	0.195061764	0.185610044
0.088091115	1.319788531	0.103641941	0.114816486	0.109961444
0	0.870440911	0	0	0
0.002132223	0.001808598	1.008821389	0.002641633	0.002552999
0	1.863835424	0	0	0
0.042528506	0.035550456	0.049251107	0.053946702	0.051920279
0.016899648	1.660239335	0.019398575	0.021117419	0.020378299
0.011386424	0.009625553	5.714392582	0.014182657	0.013693956
0	0.780753801	4.98490751	10.59415263	7.621198356

0	0	0	0	0
0	0	5.27745895	0	0.864650795
0	0	0	0	0
0	2.34959079	0	0	0
0.027901484	2.129563813	0.03214902	2.148916396	0.033823293
5.003471531	1.797775127	0	0	0
0	1.211942492	0	1.56396797	0
1.029505386	0.00618043	4.745714735	0.009071355	0.008762411
1.554486554	0	0	0	0
0.051852278	2.827404666	0.060242852	0.066136979	0.063589902
0.048196969	0.040207807	0.055925209	0.061341917	0.059002388
0	0.986395467	2.301994272	0	0
0	1.979735888	0	3.295715517	1.977055011
0	1.443349854	0	0	0
1.911409692	0	0	0	0
1.176580092	1.166625466	4.722315085	0.056530547	0.05439569
0.150164866	0.120995582	3.34684094	0.203290547	0.193312149
0.001604555	2.329865501	0.001832144	6.076705404	3.817466626
0.253352685	1.827813976	0.315920116	0.366283366	0.343778548
0.007516114	0.006362761	0.008599669	0.00934088	0.00902256
0.228705547	0.179739169	4.833117876	0.325396431	0.306406371
0.011725455	0.009910928	0.013435306	0.014607823	0.014103986
0	0.834915081	0.826044688	0	0
0.071135042	1.261302245	0.083200553	0.091778215	0.088060962
0	3.229197377	1.903752688	0	0.927606142
0	0	0	0	0
3.005726501	1.14111521	0.043363406	0.047439808	0.04568186
0.981945063	0.982776751	0.981588355	1.975713034	0
3.395912521	0	0	0	0
0.054213495	0.045130921	0.063037704	0.069245453	0.066561929
0.170171435	0.13622747	0.206047618	2.836988435	1.675791851
0.114260099	1.407433763	2.118359383	2.577778664	1.458422161
0	0.689850653	0.677296244	0	0
0	0	5.765544895	0	0
0.505152361	0.979341438	6.803119826	0	4.848184305
0.024840386	0.020896655	6.105401246	0.031184152	0.030068144
0	0	0	0	0
0.001188714	1.590353953	0.001357127	2.00647275	1.004903594
0.053913158	1.200463428	0.062681957	0.068849587	0.066183522
0	2.659288847	0	1.845001411	0.884331078
0.782869975	1.729295464	0	0	0
0	0	0	0	0
0.054964517	2.840880311	0.063927603	0.07023597	0.067508652
0.260857273	0.203011908	0.326146299	0.378992329	0.355344546
0	1.42051656	0	1.805180104	0
0	0.920909715	3.679488674	0	0
0.863092338	1.428142622	0	0	0
4.90365392	1.709974205	0	0	0
0.020872572	0.017583985	0.023991812	0.026142379	0.025217106
0.003841724	1.602333091	0.004390009	0.004764264	0.004603616
0.022560161	0.018994084	0.025946696	0.028283892	0.027278088
0.796545795	0.803980934	0	0	0.795079841
0	2.301295432	0	6.875698074	6.513599855
0.086547495	1.314520653	0.101771122	0.112699801	0.107952697
2.230778201	0	0	0	0
0	1.340551217	0	0	0
0.668847593	1.192240226	0	0	0
0	1.893374816	0	3.560443557	1.475318284

1.19355591	1.182469076	0.056753407	0.062261027	0.059881991
0.605998017	1.112138704	0	0	0
0.090794255	0.07462768	6.954185805	3.312655402	0.113486398
0.903445354	0	0	0	0
0.061818199	0.051324125	0.072069983	9.100813422	6.730488322
0.029083431	1.712751275	0.033524577	0.036601558	0.035276186
0.019423688	0.01637197	5.118799259	0.024307134	0.023450306
7.070298455	1.931337064	0	0	0
0.311288419	0.238688849	2.192605082	0.467699978	2.215587341
0.08180499	0.067447308	4.266975058	0.106221057	0.101800057
0.020981441	0.017675004	0.024117855	2.112545988	1.086067793
0.076034618	1.902738057	0.089082695	0.098387477	0.094352162
0.01840506	0.015519112	0.021137556	0.02301873	0.022209638
0.176336809	0.140883395	0.213976634	0.242655684	0.23001636
1.056609873	0.011869333	0.016116711	7.615458417	4.673689131
0	0	0	3.277801497	0
0.065256151	0.05411327	0.076168884	0.08389857	0.08055172
0	1.527429667	0	4.167258829	2.72234289
0	0	0	0	0
0.076089799	0.062859389	0.089149054	0.098462132	0.094423186
0.114334655	0.093227899	5.757738264	0.151410405	0.144580857
0	1.689413696	0	0	0
0	0	0	0	0
2.789880507	2.202237916	0	0	0
0.130040621	0.105478638	6.432129123	0.173872811	1.52011532
0	1.460754059	0.889304009	1.852413452	0.889784107
0.089933327	1.326060825	0.105877254	0.117347743	0.112362719
3.11989137	2.270973575	0.070834246	0.077935982	0.074863052
0.016342319	2.077035195	0.018755231	0.020414366	0.019700975
0.009417983	1.03616046	1.038869785	0.01171741	0.011315919
1.229328348	2.769736849	0	1.602397365	0.710405895
0	2.222874938	4.045950984	0	0
2.275625527	0	0	0	0
0.024333118	0.020473671	4.539478088	0.030538281	0.029446928
0	1.561580687	0	0	0
0.091934356	0.075535257	0.108308495	2.470754471	0.114975906
0.077791799	1.28442729	6.653821099	7.683754285	6.188951385
0.009469703	1.036357668	5.228659525	0.011782112	0.011378344
0	0	0	0	0
0	1.523189586	0	7.420355671	6.281485481
0.027386526	0.023017424	0.031550059	6.365575965	4.755132983
3.412576186	0.510414893	0	5.312576314	3.899434227
1.257286833	0.054337858	0.076499785	0.084268858	0.080904816
0	0.48154523	0	0	0
0.071583215	2.644095212	0.083737778	0.092381176	0.088635185
0.040643113	0.033997242	0.047036997	4.249811338	2.546251932
0.004119655	0.003491833	0.004708056	8.68191975	7.945498314
0	2.200244264	0	0	0
0	0	0	1.551036922	0.674758776
0	0.760202969	0	0	0.750362099
0	1.973410836	0	2.364114001	0.448276637
0.045025207	2.531595344	0.052187534	5.490979587	3.726632563
0	1.525774565	0	0	0
4.554742462	0.979803511	0	0	0
0	0.900052786	0	8.788012235	6.947911177
3.125446081	0.708629817	0	1.584732071	0.698097608
0.032364868	2.149436284	1.132590688	8.784571267	7.421129096
0.805550885	1.758867392	0	5.599867506	4.26401032

hsa-miR-518c-5p	hsa-miR-20b-5p	hsa-miR-6514-5p	hsa-miR-548x-3p	hsa-miR-524-5p
0.111966889	5.874933023	1.928815184	0.069417198	0.095742228
1.224738873	7.655909479	2.57121263	0.045999869	0.062318276
1.110183149	6.731081915	1.101296569	0.022641571	0.030134378
1.057764606	6.601394323	2.065570801	0.011888978	0.015695433
0.121358414	5.250984673	0.070965505	0.074738946	0.103504282
0.050343082	3.40483606	1.145378405	0.032656392	0.043794515
0.164352133	3.240879212	1.42345123	2.071369343	0.138532444
1.669190461	4.43059651	1.65907263	0.014209804	0.018792176
0	6.128158381	1.505388571	0	0
0.047355972	5.628728948	2.980775646	0.030788202	0.041230655
0	4.609488959	1.09197128	1.457357686	0
0.232729756	4.238596234	0.125517068	0.132935996	0.192587509
0.111309377	5.265793072	1.301874649	0.069041971	0.09519729
0	5.853118294	1.565285766	0	0
0.196382108	4.619108217	2.132560054	1.502169606	0.164100974
0.042462214	4.795735803	0.026435551	0.027709304	1.13650699
0.570365235	5.062435614	1.065407217	1.059032579	0
1.54335629	3.415360807	1.115494267	0	0.749909264
2.326748794	4.968650407	1.843589962	1.230762672	0.069879613
0.253689406	5.973664762	0.13487094	0.142977105	0.208764749
0.180802623	5.77568604	1.458762253	3.371953919	0.151719653
0.302206521	1.828057402	0.155559979	0.165248735	0.245531376
0.02824373	5.502099776	1.083526782	0.018632683	0.024723771
3.42713951	5.91543996	2.459815734	0.090897856	1.448588365
0.149205463	7.381670237	0.085436497	0.090115596	1.444714883
0	3.398036881	0	0	0
3.138349085	6.977014179	1.975994141	0	0
0.062156687	5.949108432	1.788298783	0.03996342	0.053892138
0.08611207	5.362019967	1.239554701	0.054394178	0.074163987
0.039874143	5.816152494	2.141402984	0.026071776	0.034789964
0	4.521969414	0.511487628	0	0
0	6.845004303	1.58464041	0	0
2.251493381	6.856434396	1.036841029	0	0
2.227882353	4.058031087	0.773222084	0	0.758963186
0.097811164	5.263447739	0.058246068	0.061260909	0.083966268
2.510950986	5.194942792	1.663531686	4.257808806	0.233957714
0.129165019	5.22446169	0.075083052	0.079109597	0.109925945
1.750963932	4.736183582	1.377988037	2.926506311	0
0.084374055	4.982971759	2.280683947	0.053364118	0.072702318
0.0216101	7.505160895	0.013689995	0.014329769	0.018952542
0.101498509	4.159170347	0.060268277	0.063401364	0.08704261
0.066347963	2.604637955	0.040508829	0.042525097	0.057458555
0.128703765	5.163195075	0.074841105	0.078852677	0.109547285
4.991074142	6.635695356	0.036707176	1.173996736	1.189799098
0	5.594829568	0.940149109	0	0
0.925563429	5.046895969	0.929927845	0.928939927	0
0.165777299	5.361996289	1.426552668	3.529140706	1.488686075
0.196357247	5.215255893	1.491196977	2.58321365	0.164081299
0.11547667	6.147414766	0.067831039	1.946287851	0.098647749
0	3.781051815	1.838900244	0	0
0.002653557	5.769021595	1.008072722	0.001785913	0.002339842
0	6.90302778	2.127181187	0.668846486	0
0.054220642	3.421794013	1.155932061	2.519258024	0.047116233
0.021217028	5.357418093	1.063230869	0.014073415	0.018609889
0.014248474	5.028130855	1.042790863	0.009502574	0.012522439
9.407311031	5.224761119	0.784055714	0	7.356980982

0	4.522975029	1.105823255	0	0
0.866626619	5.167885386	0	0	0.864230824
0.70847534	6.363263451	1.244117173	1.239167036	0
0	3.349818008	2.361057265	2.352133178	0
0.035260683	5.177950713	1.103486389	1.105211024	0.030804912
0	3.32203515	0	0	0
0	5.277939731	0.698480975	0	0
0.009112943	5.906108372	1.617044171	0.006102103	0.008020755
0	3.866491465	0.976141501	0	0
1.208032592	5.128921542	0.040586109	0.042606576	0.057572218
0.061658405	4.883767575	2.211775707	0.039657813	0.053467585
0.985712678	6.085030806	0	0	0
2.78209365	7.247975231	1.566322237	0	2.782936653
0	5.512379429	1.081541116	0.588322968	0
0	5.278914641	1.509042739	0	0
0.056819215	4.078552106	0.034959814	0.036678018	0.049338256
0.204659349	6.974027213	1.508144754	0.119063652	0.170636925
6.03300542	3.179814725	1.006077519	1.00617004	2.008596042
0.369422668	5.670868759	0.18220874	0.19405987	0.294953503
1.62227146	6.093998831	2.034883277	0.006282089	0.008258451
0.32803415	6.415738079	0.166061959	0.176586459	0.264725857
0.014675682	6.121388209	0.009351817	0.009784219	0.012896325
0	3.806111015	0	3.857137284	0
1.926081603	6.831279004	0.055192364	1.259928886	0.079341965
0.928664132	4.527160698	0.932872299	1.914332196	1.496888561
0	6.114342007	0	0	0
0.047677308	4.481210626	0.029555065	2.986399583	0.041506667
0.981919804	5.014966244	0	0.982847336	0
0	5.639096647	1.43928812	0	0
0.069608752	4.329357528	1.196973755	0.044507111	0.060227424
3.188467813	5.764886454	0.126505063	3.270407116	0.194283327
0.152235986	5.716398267	1.396750133	0.091753756	0.128744177
0	3.910169653	0.694024458	0	0
0	5.643036083	0.756279562	3.232648818	0
1.808746997	9.040742864	1.348420037	0	0
0.031334694	6.182982572	0.019689082	0.020622676	0.027405488
0	4.2697227	0	0	0
2.814497049	5.59283902	1.590225909	0.000996016	0.001304172
0.069210508	5.86016185	1.195928175	0.044265556	0.05988953
1.443847956	6.14740176	1.864151732	0	0
0	4.334489149	1.337723027	0	0
0	5.192512344	1.96061035	0	0
1.220359939	5.897482246	0.042960747	0.045110905	0.06107257
0.382295483	4.816241828	0.187068676	0.199328843	0.30422432
0	4.619307838	1.423988851	0	0
0	5.033368654	0	2.480768094	0
0	4.980523369	2.153320898	0	0
0	4.370975028	2.275041891	0	0
0.026267133	8.393798575	0.016575105	0.017355164	0.023006412
0.004785881	7.753538845	1.601917357	0.003215603	0.004217501
2.120681583	6.05302737	1.68229813	0.01874612	0.024876422
2.029147632	5.459548326	0	0	0
7.437654833	5.084262559	0.653331856	0	5.227081111
0.113345174	6.16807272	0.066688217	0.070202624	0.096883892
0	4.076201252	0	0	0.359624137
0	5.704537987	2.057041756	0	0
0	6.694967354	0	0	0
2.865727844	8.868577974	1.896032549	4.585522117	0

0.062582888	5.979679074	0.038326418	0.040224639	0.054255181
0	3.950295811	2.616472362	0	0
3.686635683	4.338153224	0.069828623	3.991839457	0.10173962
0	4.247619706	1.286529883	0	0
7.574070525	7.893865991	1.223287431	3.457180912	5.048933612
0.036780433	5.139318606	0.023006285	0.024105849	0.032118802
2.431030643	3.287496057	1.669080047	2.414972456	0.021402093
0	6.008229746	0.557511479	0	0.538741352
0.472252978	5.78068726	0.219080144	0.234144257	0.367340722
0.106821716	5.300481834	0.063167442	1.296652957	0.091472686
0.026405856	4.834602343	0.016660701	3.273499651	0.023127004
0.098935319	4.778515393	0.058863796	0.061914663	0.084904814
0.023127788	5.791995586	0.014632709	0.015318113	0.020274842
0.244396416	5.687250372	0.130755996	0.138557642	0.201614378
5.448840773	4.259346252	1.052704496	2.876833673	2.887642485
3.277806201	2.543450674	0.973234764	0	0.970953701
0.084352309	8.295046217	0.050765961	0.053351213	0.07268402
2.721367039	5.943945775	1.536760808	0	2.242982356
0	6.685871125	1.566141326	0	0
0.099010471	4.84991972	0.058905054	0.06195833	0.084967536
0.152342425	5.312596103	1.396987337	0.091811169	0.128830447
0	5.600643355	1.949322355	0	0
0	6.897572963	0.705978187	0	0
0	5.954091601	1.454741946	0	0
2.192541514	6.618211913	0.09831682	2.096871008	0.147070713
0	5.545651313	0.897337182	0	0
0.118025769	4.926633224	0.069192918	0.072858529	0.100754462
0.078352345	5.661401394	2.262834127	0.049775058	0.067627108
0.0205105	5.391398769	1.061172774	0.013612238	0.017993793
0.01177147	3.643883012	1.62624442	0.007865849	0.010352802
0.714550196	5.98527762	0.724721595	0	0
0.920650952	4.776148433	0	0	0
0	5.33520707	1.546720557	0	0
2.458057951	5.857491468	2.435002734	0.020205494	0.026842628
0.979159866	5.010452297	1.562125632	0	0
1.365695244	7.29846867	0.070669738	0.074425139	0.103044845
7.256972168	5.170282646	0.060176648	2.933850185	4.677312151
0.011836479	5.616690306	2.630583999	0.007908886	0.010409783
0	5.210151542	1.069740733	0	0
6.613801434	4.557511488	0.646709445	0.643163575	5.082063859
6.424176018	4.758461008	1.702447838	1.70483887	4.861630974
5.233974548	10.26570612	1.630550257	3.929338243	3.303211275
0.084724943	5.688932129	1.236031007	0.053572286	0.072997529
0	5.282570596	0.486720066	0	0
0.092889443	6.371264072	1.256628893	0.058386226	0.07985028
2.214660259	7.027292706	0.031977524	1.151827405	0.045007443
10.11880238	5.312510843	0.003297673	3.190817562	6.468025432
0	5.160428996	1.47678207	0	0.901364562
3.83587609	6.70797975	0.689675151	0	0.672771188
0	5.614311007	1.300456776	0.760967088	0
1.704131018	5.757321603	1.542286919	3.584268462	0
4.980634223	5.838361035	0.035353457	1.778221507	2.231747547
0	6.252769124	0	0	0
0	8.557322987	1.975404521	0	0
8.514606202	4.151913543	0.901772325	0	5.769044381
0.702406372	6.667823313	1.236507473	0	0
8.213431282	4.904846336	0.02556272	3.326919722	5.944482199
5.397042155	5.423346824	2.078552584	0	3.159845936

hsa-miR-6844	hsa-miR-653-5p	hsa-miR-1229-3p	hsa-miR-509-3p	hsa-miR-146b-5p
0.065449497	3.814575621	1.334827443	0.111039425	10.98936609
1.8169562	3.127756515	1.845290004	3.919604357	12.49751948
2.447798331	4.170327769	1.714786469	4.25791879	12.67528064
1.646930004	6.115595425	2.397837822	2.89202163	12.19943979
0.070424544	5.007191222	1.360284928	3.089518726	12.46733996
0.030920835	3.232518217	1.771146859	6.535568723	12.55676364
2.491471479	4.279288655	1.472340785	0.16285457	15.65990827
0.013483972	5.75473278	1.666839415	0.021279263	12.37098298
1.915775959	4.467765	0	2.699736679	12.21452867
3.633963649	4.877588311	4.960658073	3.023924553	15.41132235
0	6.847029758	0	1.942166147	12.86109201
1.561759983	4.840923426	1.636828695	2.363856118	11.63116279
0.065098494	3.481487219	2.741349936	0.110388515	9.75163616
2.978021822	2.780709933	2.560417637	2.558830289	11.6679065
1.489650783	5.409665227	0.148272805	1.572381767	14.09441904
1.123346216	4.457001498	2.168813244	3.370076075	9.077844364
0.580421666	6.5636492	1.395178644	0	11.22364686
0	4.997249558	0.75391448	1.541226426	12.02849006
0.048534431	4.183316616	1.248275531	1.252267452	13.19340059
1.601348359	5.657902742	1.684220402	2.892131887	14.48983803
0.100341093	9.2944261	0.137363491	3.297401594	12.63705452
1.687946458	3.384189376	0.218964951	2.553655376	11.95216742
1.083323029	3.597048207	0.022866365	2.447758032	13.93916108
0.085495273	4.456996382	2.529165499	2.917035944	11.95622944
0.084766908	5.412274044	0.114816036	1.446216697	12.66500454
1.233634589	2.336256678	2.543556842	2.335164151	10.78547489
2.29733592	4.288744889	1.973316013	4.214867811	12.58573192
1.787658704	5.20933437	1.193389372	3.283366536	13.42967995
0.051373389	2.955169287	1.262801673	3.978439383	12.64376685
0.024705288	1.126662032	2.159040603	1.127463385	11.57301226
0.512105202	5.338879768	4.04899186	0.944616037	11.72137154
1.999660328	4.321456528	1.999620623	5.976799119	15.5810049
0	3.930451274	2.27983585	1.870731614	8.788505681
3.005812796	5.58845373	1.988906846	2.792044826	12.97118758
1.268123929	5.982784372	2.36397921	3.428960596	13.29647861
0.147825643	8.04784854	2.433708133	4.064904616	13.58501408
0.074506102	3.724219629	2.02493363	1.390727638	13.82054424
1.779558749	3.032255689	2.325936423	3.86860028	12.75360402
0.050406515	3.844202817	1.885764466	6.551270128	13.42583819
2.402458809	4.108541543	2.678091787	4.196112422	12.25128018
1.899648521	4.551673072	4.313696602	3.015178011	15.41474198
1.187886605	3.300949943	3.079276741	1.208487851	12.96576728
1.342096452	4.475923266	2.023561904	3.11634914	12.94097699
0.036446387	4.610624597	2.561983211	2.246586697	12.03021651
3.090484713	4.145028687	3.079119192	0.936085543	12.40990966
0	5.49111576	1.494785689	0.925270198	12.67027094
2.061301958	5.80871733	2.573237681	0.164263009	12.95924509
0.10775121	3.583239407	1.551302184	2.258325727	15.36860513
0.067317146	4.323403445	0.090101253	2.005167732	10.87558101
2.156319493	6.057656893	3.261802942	2.603403728	13.0705682
0.001697211	4.968184939	2.333441955	2.011608892	13.40478073
0.672769702	4.855871264	0	1.818742907	11.90184605
0.033195846	6.745553477	1.784631562	5.395410185	9.778538659
1.658156497	4.567396072	2.900027774	0.021071668	12.03124863
1.042690127	2.061330509	1.046138696	1.641524068	13.06138822
1.723060171	4.782550352	2.260008438	1.993203215	11.83606115

0.611259374	5.102186472	0	0.599435062	12.69826069
0	2.605327564	0.865569634	0	10.14518325
0.719155702	5.989951701	0.704507558	0	13.65829754
2.718338108	3.440327072	1.851099391	2.474020165	11.87520761
0.021931654	5.879574923	0.028460947	4.17367997	10.86493361
0	4.632200441	0	5.116408081	9.702098562
1.219380831	5.303273662	1.572504078	2.808738523	12.63510687
0.00579602	3.746468498	0.00743956	3.504939352	12.26280444
1.557066458	4.418346463	1.554299368	0.974399284	11.8243394
1.188238857	5.02118587	2.586519013	2.60625398	11.58686323
0.037519426	4.544379382	2.239398539	0.061196645	11.4710671
0	5.064229449	0	1.98080499	11.77509951
1.566373716	7.503269853	2.976195497	3.295717879	11.13906783
0	5.251665688	1.948685691	1.051232646	13.23996202
0.935693651	4.531896899	0	0	13.26136129
0.034712377	4.361093871	1.793605062	0.056398034	11.65027244
1.506476617	4.646671443	0.154014246	4.840191513	13.46582132
0.00127753	4.010298105	2.593817637	2.331094191	11.63330859
0.180532562	4.093003387	1.924055511	0.364850194	10.38877082
2.357613113	4.633629846	3.850378261	0.009321194	13.28039272
0.164570578	4.185269557	1.84223835	0.324191321	14.69542592
0.009289347	6.434569058	0.011951776	2.65334312	14.19851134
0.83787364	2.330736938	0	0.82875615	15.83365578
0.054784708	4.750750618	1.911252336	1.927048968	11.75281186
1.916271592	5.609442702	2.486202697	0	12.89915533
0	5.218300194	0.855888974	1.804758264	11.79131483
0.029348342	3.813065845	0.038278284	4.037128665	12.21860483
0	5.992664229	1.563245075	2.558667242	12.71471384
0	2.96790538	1.829149578	3.377790659	10.00804039
2.235566974	4.026441495	1.215268683	0.069078717	13.75507796
0.125438532	3.909689552	0.174686583	5.337788786	12.16928914
1.395531124	4.462815139	0.117003571	3.432559031	12.80980184
0	4.529922725	2.965724299	2.491897506	10.79835557
0.756706826	3.741240566	1.269233916	1.648178578	10.98529361
0.522225187	4.178653097	1.82697367	3.501251411	12.40956558
1.691527614	4.404439517	1.100009536	4.325787414	13.89498046
0	4.340021663	1.545888521	0	12.19217993
1.004493719	5.708316249	1.590746789	3.007256699	13.28107278
0.041856265	3.780473156	2.864364791	3.780181706	10.28128181
0	7.274677541	1.850176317	1.443408967	12.47408352
2.049174703	4.029313452	2.275242014	3.23342071	12.71016171
0	4.337810792	0	3.269684728	13.15197621
2.23860171	3.91374589	3.614520974	1.221269376	11.98296032
0.18533563	4.511870667	1.948686579	2.02042439	14.90433752
0	4.406323404	0.856100445	1.407274914	10.08998071
0	3.569250973	0.917066534	3.454496222	13.42477222
0	5.384783606	0.862614977	4.097618555	11.27277068
1.489945361	4.603014665	1.862295469	1.838463447	10.81884384
0.016462509	6.13536674	1.68477956	1.084732963	12.02940613
1.014491691	3.483473818	0.003914469	2.020888388	12.92929208
0.017778986	4.390422591	1.692692484	4.390112699	12.43201058
0	5.267863982	0.795915421	4.656919679	10.7081903
1.847071327	5.377151834	2.44791191	1.788774765	11.92891237
0.066184121	3.059339873	2.414479187	3.05984604	11.77536085
0.378159377	3.806608383	1.337475835	3.719891733	9.654933999
0	4.851606973	1.326484852	2.485267068	13.76759059
1.199936821	5.563385013	0	1.83593674	11.53482519
1.487365215	4.10505864	2.20250025	3.445726988	10.17635314

3.684404546	2.256491932	3.722482435	2.25734858	16.68177324
2.451865298	4.878474517	3.755215023	1.978231231	12.88416915
1.319863556	7.178665741	0.092877284	1.362737489	12.15173759
1.287912946	4.005122895	1.520690186	0	10.86364203
0.047815995	5.079108055	1.244599533	3.356514716	12.79002012
1.107501075	4.504686418	1.116856433	1.727957684	13.42621527
1.072353451	2.697234566	1.078403773	2.431201092	13.04866814
2.91700526	3.280477317	1.361040821	4.376312415	10.35362454
0.21695858	4.111072668	0.321478559	5.495452321	14.99329638
0.062693294	2.420315252	0.083648179	1.327412041	10.6338094
0.016547501	7.49424057	1.084628652	3.296867162	8.838164499
0.058425762	5.54943675	1.298887391	0.098136168	13.08180631
1.068615215	5.025268124	2.419417718	1.676010946	12.14996746
1.583967244	4.666380673	2.78302191	2.396756083	13.51889493
0.011121702	3.672490276	1.652574172	2.890805997	11.68561219
0	3.414984148	0.971310625	4.041895693	13.17473269
0.0503944	3.177490464	1.257792447	1.262094535	11.65637691
0	6.655655885	1.797463851	2.414486173	13.50727946
1.566193298	6.397966842	3.435014878	2.974078215	12.85037733
0.058466676	0.099370233	1.932614903	5.818723473	12.62492593
0.086345507	4.748322632	2.534159735	2.922833333	13.14257763
1.950941203	4.454962474	3.061694813	2.68415055	12.41884843
2.179294799	8.549815838	1.885788776	2.118254565	10.69414627
0.593422004	3.835174543	1.70636776	3.64737248	11.07912607
0.097527748	4.741108344	1.498980475	7.309329654	13.22143835
0	5.925886594	0.890645957	3.911146831	12.57869161
0.068667299	4.434535613	1.991463511	5.118588156	11.60323157
0.047035855	3.81914123	2.298211624	6.802181076	8.409703031
0.012917849	4.563900773	0.016661254	1.066421874	12.35685069
1.035366247	4.645809866	3.0526219	2.375362758	13.9545688
0	11.97593242	2.166469737	1.90113227	11.8681246
2.225848018	3.459494216	1.896435801	2.468346264	9.971749042
0.967487581	2.755620277	1.543075379	1.542053415	16.26955607
1.090284797	2.950269329	1.0979909	0.030470677	14.97985395
0	3.427088798	0.979098396	1.97197843	11.60052734
0.070131343	3.989433859	1.358784598	2.470951878	12.53063339
0.059727654	6.984031377	0.079530278	1.957851607	11.74265365
2.366755842	3.227864432	3.223174489	2.863833415	14.49534207
1.070700255	3.487190373	1.690565551	2.295822092	10.74123075
0.647249624	5.830981386	0.630845702	2.020448128	12.3900433
2.123268021	5.497247407	2.138925955	1.719743673	12.82417188
0.980804237	10.45350849	0	4.361140221	11.84161433
1.235381042	3.55054581	1.886904108	2.677687634	11.69433721
0.941819918	3.972184462	0.469359363	2.116992962	13.08269839
0.055118435	2.981590702	1.913191304	1.929128413	8.296506766
0.031752663	5.780510094	1.162412934	2.21510516	13.50798033
0.003275958	5.934132328	0.004197715	1.605516713	11.54136651
0	4.697741429	0.902418493	1.86835104	11.04287138
0	4.342929462	0.674734133	2.091523299	10.43318862
0	5.891391813	2.228201057	0.753044468	11.707677
0	5.652894719	2.763255863	1.476147591	10.53414806
0.035103021	7.494874978	2.553385582	1.181678806	14.0437274
0.950919135	4.558558049	1.52170214	3.08943699	15.29026085
1.975479961	4.053698517	0	4.489471329	13.86722736
1.469067916	7.091336797	0.895332279	1.858703152	11.7212116
0	8.164629015	1.896122352	1.209097465	12.22063131
0.0253855	3.899208107	1.738219388	3.19160143	8.485709798
2.079270065	4.918648174	2.309007706	3.490666361	10.37574845

hsa-miR-518e-3p	hsa-miR-518a-5p	hsa-miR-527	hsa-miR-5187-5p	hsa-miR-431-5p
0.109210457	0.104621245	0.10418388	1.976985763	4.675458997
0.070433431	0.067685845	0.067423044	2.878548213	1.856757637
0.033760128	0.032539904	0.032422798	2.732600095	3.334045075
1.058331381	1.058731094	1.058746808	3.666601243	2.402524454
0.1183182	0.113262868	0.112781493	1.363115129	2.024692432
0.049247067	0.047407383	0.047231078	1.772776237	1.159915523
0.159909212	0.152564079	0.15186741	2.57668368	0.162624928
0.020988355	0.020250784	0.020179917	1.667488908	3.426465297
0	0	0	1.49749771	2.699728328
1.151496174	0.044611328	0.044446428	2.189044333	2.197721369
0	0	0	2.658937017	0.588724796
0.225706246	0.214202671	0.213118442	1.643494167	3.463722778
0.108572508	0.104015468	0.103581143	1.335565369	0.110247158
0	0	0	1.563225507	5.140757621
0.190783793	0.181568743	0.18069729	0.152912141	2.258577389
1.136278289	0.040025998	0.039879531	2.764782296	0.042110171
1.385340614	0	0	2.621649401	5.832218111
3.175741052	2.182555641	2.182994016	1.746294012	4.357869119
2.327815785	1.255085082	1.255109668	0.065838337	2.327447217
0.245788972	0.23288642	0.231672695	0.193535882	1.718468102
0.175778062	0.167489866	0.166704939	1.518141627	3.731781376
0.292121618	0.275761934	0.274229923	1.798477811	1.836233567
1.696025418	0.026671315	0.026576595	1.090877882	2.1202485
3.624674945	2.568628471	2.567986219	2.093232028	2.572794547
2.115391088	0.138766685	0.138148425	1.437376687	1.446453305
0	0	0	1.888590411	4.794122502
0.979109093	0	0	3.429642253	1.559460493
0.06076951	0.058444701	0.05822214	0.050872317	2.255758622
1.268764625	0.080724994	0.080403076	1.894380747	3.184752727
0.039025369	0.037598741	0.037461893	0.032915797	2.991292553
0.499137679	0	0	0.493397936	4.555962268
0	0	0	2.321530932	1.999603778
1.004002244	1.000670975	1.000510418	0.540362738	3.437032679
0.761389476	0	0	3.22037569	1.287995596
0.095467127	0.091557149	0.091184037	2.368211026	0.096901786
0.277432212	0.262203154	0.260774976	1.765268948	1.799358933
0.125882737	0.120430597	0.119911806	2.029699615	7.075768258
0	0	0	2.06719546	8.875441818
0.082404397	0.079113043	0.078798582	2.322470016	1.262300551
0.021168385	0.020424189	0.020352686	0.017965802	3.274433926
0.099048827	0.094964631	0.094575025	3.578027093	1.958542915
0.064854388	0.062352641	0.062113232	1.828300344	3.301026713
0.125435942	0.120007482	0.119490923	2.468516423	2.840370794
1.813537986	2.246068894	2.24588788	2.564788631	3.077029249
0	0	0	1.507444913	8.761559954
0.924842191	0.924278992	0.92425166	1.90214879	3.688674972
0.161284972	0.153859592	0.153155405	1.480182416	3.246699863
0.190759865	0.18154632	0.180675007	1.556616746	3.783933685
0.112615133	0.107853165	0.107399481	0.092626688	3.497257625
0	0	0	3.797533334	8.527445956
0.002601651	0.002513987	0.00250555	2.333557774	3.472797474
0.658910902	0	0	2.922499452	2.616042483
0.053030481	0.051033782	0.050842496	2.543677507	3.958624697
0.020783734	0.020053688	0.019983543	1.068662802	4.692881176
0.013962096	0.013479151	0.013432719	1.640292422	1.641545806
8.138597932	6.941043263	6.867816698	1.304085253	8.262860049

0	0	0	1.074721555	3.929451707
0	0.864309419	0.864255676	0.865059003	2.794220221
1.591971124	0	0	2.36757804	2.138916406
0	0	0	3.179317469	8.968219659
1.113707578	0.033267993	0.033148072	1.112809804	2.149037347
0	0	0	2.094118516	2.558058636
0	0	0	1.191466574	1.859934704
0.008931954	0.008626534	0.008597157	1.620559294	3.214711147
0	0	0	2.287448199	4.12923147
0.064984705	0.062477272	0.062237321	3.083375852	0.065901489
0.060283769	0.057979828	0.057759255	3.064993117	0.061125695
0	0	0	1.567687686	3.678055653
3.433089161	2.297971446	2.297987844	1.977865366	1.977111841
0	0	0	3.391212921	2.132878045
0	0	0	3.222372043	4.887661447
0.055565184	0.053462048	0.053260611	0.04659898	2.837257275
0.198747141	0.189026404	0.18810783	0.158882515	1.594220218
5.654358815	3.331791891	3.179702281	0.001672391	2.816999821
0.355954497	0.334311231	0.33229711	3.4244675	0.36415201
0.009197247	0.008882564	0.008852296	0.00783862	2.040761788
0.316698296	0.298375495	0.296663773	0.243867489	1.897024919
0.014380426	0.013882538	0.013834672	1.04775063	1.047742108
0	0	0	1.374040666	0.828667244
1.28692951	0.086442209	0.086093506	0.074669507	1.92716554
0	0	0	3.355227332	2.22045925
0	0	0	3.246203487	3.766760247
1.769153814	0.044912213	0.044746092	2.190252822	4.137341455
0	0	0	2.297520029	3.142338806
0	0	0	2.620548953	4.245870346
0.068031278	0.065390134	0.065137459	1.216683091	2.284372476
1.674378446	0.216156077	0.21505857	2.32060002	3.470459337
0.14820556	0.141531447	0.14089772	0.120459931	2.123838131
0.680847666	0	0	3.579857103	2.098568048
0	0	0	1.267639865	2.412871571
0.509104086	2.341005361	2.341435038	2.763984504	0
0.030680087	0.029578595	0.029472855	2.127583542	2.728137734
0	0	0	0.967278978	1.956279516
1.004879459	0.001400975	0.001396286	3.707386795	4.865750278
0.067643333	0.065019296	0.064768248	2.599705578	1.217207112
0	0	0	0.884757199	0.885516438
0	0	0	2.489407082	4.491676438
0	0	0	1.955888277	0.966026255
0.069001923	0.066317877	0.06606112	1.842760442	4.504129416
0.368132031	0.345412735	0.343301023	1.961569403	2.756456788
0	0	0	0.85556354	0.8566592
0	0	0	2.469334003	1.482792528
0	0	0	2.969188537	3.586913802
0	0	0	2.887189854	3.704416954
0.025724558	1.085789288	1.085810424	2.433358056	1.084778795
0.004691791	0.004532926	0.00451764	1.015700572	2.607534493
1.696698492	0.02683673	0.026741387	3.296195269	5.570938834
2.027977011	0	0	2.037591856	3.833485167
7.541819253	6.946590242	6.917246487	0.636755963	5.003046513
0.110547603	0.105890755	0.105447	2.419461911	0.112259328
0	0	0	1.071646192	4.553271025
0	0	0	0.788015241	3.653152952
0	0	0	1.17117412	2.472980818
2.675116634	0.910585638	0.910552613	1.477568897	3.560490242

0.061184965	0.058842279	0.058618016	3.263330546	4.886576196
0	0	0	2.697822494	5.933580855
2.465803358	2.801630156	2.801028983	0.095495045	0.118064164
0	0	0	1.750615972	4.132758465
7.770874082	6.012136203	6.011218509	2.30638142	3.35647948
0.036002782	0.034695172	0.034569706	2.475787993	4.180759545
1.079333454	1.079867536	1.079887589	2.100309823	1.078923897
0	0.540871333	0.540637669	1.885033249	1.634260162
2.218305275	0.422005896	0.419167222	0.334590214	0.464615769
0.104217312	0.099878235	0.099464515	0.085962326	3.793868997
0.025860246	1.08623405	1.086255265	2.108183494	1.085218556
0.096559207	0.092596318	0.092218196	2.371977362	3.005383287
0.022653424	0.021854378	0.021777616	2.685553476	3.698970033
0.23688976	0.224614583	0.223458879	2.34425676	1.695342623
5.516705933	4.611455565	4.611286105	1.653103057	2.401365465
0.971126716	0.970916062	0.97090644	2.767193628	2.281520966
0.082383243	0.079092869	0.078778501	2.654277389	1.262236466
3.286293807	2.233607414	2.233897936	3.004949661	3.948650529
0	0	0	0	1.563779347
0.09663221	0.092665778	0.092287321	2.372228822	0.098087782
0.148308434	0.141628514	0.140994241	1.445556987	2.578420924
0	0	0	3.328849905	7.174274894
0	0	0	1.882688709	3.293537947
0	0	0	2.154185778	2.313270412
0.170170439	0.16221986	0.161466503	1.503542208	0.173113622
0.890347993	0	0	0.890210829	4.214516833
0.115087186	0.110198669	0.109733036	1.354023909	2.013727568
0.07654506	0.07352263	0.073233705	3.912690352	1.244491651
0.020092307	0.019387639	0.019319928	2.084662211	2.088037005
0.011536229	0.011139395	0.011101234	3.053264263	4.704588739
1.600840426	0.709602484	0.709466917	3.634355648	3.425255985
0	0	0	2.473779023	2.208919865
0	0	0	3.649861022	2.534225328
2.458371452	0.028968158	0.028864743	3.722368817	2.458286652
0	0	0	2.556173059	1.971970644
0.117778024	0.112750693	0.112271959	1.361598123	0.119627502
6.597378097	5.777101262	5.701838826	1.307798667	1.311775436
0.011599903	0.011200821	0.011162444	0.00987801	1.632075004
0	0	0	2.760581462	6.252960164
6.683775333	6.36727176	6.323026788	2.431727997	3.079685447
5.981183775	4.415762224	4.254490528	2.140302846	2.146340967
4.930481804	4.672103246	4.581048995	1.811592825	5.439793019
0.08274572	0.079438537	0.079122573	2.655595901	3.845141882
0	0	0	0.907042301	3.554002093
0.090684464	0.087004038	0.086652675	0.075143358	3.211582248
3.238325466	1.784295706	1.784226031	2.534272311	2.21515501
8.206901908	7.383905841	7.264697986	0.004294929	0.005093889
0.90210233	0	0	3.316735202	2.442000378
2.926064977	2.796344187	2.649922536	0.673909598	2.301151341
0	0	0	1.971823621	3.974752827
0.87607654	0	0	2.755497353	0.453452973
4.36206302	4.592671139	4.356623882	2.226717208	2.570130028
0	0	0	1.931420258	5.844405088
0	0	0	2.293296498	1.558836771
7.843658344	7.030345158	6.970593315	0.894912229	2.84054112
0.699677176	0	0	1.590772703	3.403079957
8.265487726	7.485042811	7.465926416	1.739520606	4.106790943
5.47906977	4.141387347	4.14187331	2.306530909	3.588941096

hsa-miR-518a-3p	hsa-miR-508-3p	hsa-miR-1295a	hsa-miR-523-3p	hsa-miR-18a-3p
0.107283606	2.437289698	0.105594355	0.105972545	3.274566122
0.069282024	4.989234283	2.626959569	0.06849676	4.416170319
0.033249693	6.263655701	0.032799945	0.032900818	2.473133458
0.017259685	5.046512857	2.40234104	0.017085043	3.787404177
0.116194679	4.237519985	0.114334156	0.114750597	2.023366268
0.048476926	8.570787617	1.16163685	0.047951025	0.04659607
0.156817404	4.008708764	0.154116198	0.15472019	0.149368512
1.669897998	1.068611263	3.27253556	1.669918693	0.019924407
0	4.46773778	2.700228177	0	1.902891842
0.045611512	2.527528441	0.044977619	0.045119751	7.058775576
0	3.661977767	1.414123545	0	2.635354265
0.220847806	2.358106764	2.361644489	0.217565714	0.209239022
0.106659226	1.988025743	4.88574665	1.343611282	2.770623179
0	3.672266748	2.780812868	0	5.586472074
0.186898812	3.582439243	0.183511863	0.184268606	4.777608803
0.040914147	4.013557302	0.040351307	0.040477522	4.107551105
0.564876457	0.572593235	0.563698001	0.563936149	1.907601949
1.536938705	3.713909488	2.306718392	2.306382544	1.31804328
1.889319523	3.16354711	2.661609328	1.254905823	2.3249362
0.240334078	3.526776843	2.419507265	0.236653705	1.719944055
0.17228649	5.161287029	1.53575779	0.16992043	2.207499694
0.285188598	0.306314295	4.083264563	2.548878388	1.835623761
0.027245227	5.429878354	0.026881615	0.026963181	1.092180957
2.914408097	3.426139263	1.454257735	4.637025254	2.565437813
0.142538092	4.224468791	4.210033223	1.450237326	2.901362739
0	2.963067032	1.202039059	0	0.693752472
0	4.666950454	2.292759965	0	5.325045449
0.059795734	4.102094629	0.058939261	0.059131234	2.254153851
0.082681768	4.796184693	4.296933802	0.081718776	0.079245101
0.03842844	1.738259471	3.648258841	0.038020575	3.772482182
0.497140086	2.592286297	0.495787998	0	2.927455562
0	8.266299076	2.999559328	0	2.999562833
1.002185398	2.25365505	1.632528159	0	2.074235286
0	3.772338374	0.759765522	0	2.630520189
0.093826583	4.977522531	1.304546595	0.092709402	0.089842482
0.270983233	5.279730488	0.265395025	1.80312945	0.255674874
0.123591669	2.839875402	0.121585381	0.122034366	0.118048565
0	5.047780787	1.356044782	0.815345391	5.126991738
0.081024305	8.988234837	1.900796487	0.080083751	1.89969473
0.020857268	5.281303596	1.670629323	0.020644467	3.425130154
0.09733488	3.617437205	3.905547233	0.096167966	1.957906592
0.06380629	3.628369376	4.396518362	0.063091246	2.270110495
0.123154876	4.897317274	2.049230384	0.121604329	2.047047467
0.057543337	5.246584436	1.190812411	2.246540911	3.858961702
0	1.914520835	1.505695647	0	4.543219546
2.88685533	2.696065071	2.696155669	0.924388496	2.216491114
0.158159168	3.680187861	2.962239037	0.156039074	3.231139702
0.186875523	1.565876304	1.576777738	0.184245746	0.177553297
0.11061541	4.770524693	3.490492937	0.109255045	2.004200849
0	4.420661182	2.961345868	0	4.991662788
1.008784869	3.183052237	1.595679726	0.002539963	2.819996245
0.657495459	2.617976642	0.656572879	0	3.672230861
0.052194454	6.88536187	3.051256699	0.051623692	1.792872879
0.020478538	3.913212537	4.427358592	0.020269783	3.270614623
0.013760266	3.530051026	1.04689112	0.01362216	2.650647037
7.497091121	2.646660765	1.299923779	7.956704473	1.99353985

0	2.348103473	2.165288467	0	2.167368954
0	2.13147052	2.387388443	0	1.417832923
0	0	0.70378177	0	4.207026867
0	4.84520779	0	0	3.766804311
0.033994882	5.177995891	0.033534294	0.033637596	1.722636231
0	6.59010502	1.379719231	0	2.342768371
0	3.17643082	0	0	1.561585092
0.008804345	5.135200323	5.046483819	1.030086652	2.627253183
0	1.553795657	2.547955015	0	3.420607558
0.063934218	2.270640384	2.87498639	0.063217549	4.211264879
0.059318757	3.453774508	6.922821275	0.058660218	3.740173602
1.980749349	3.437310572	1.567254739	0.985488348	0.985453356
3.433195688	4.779759747	1.977068078	2.974488365	0.982664926
0	2.867073741	4.458786067	0	3.659012071
0	1.500924776	0	0	3.065971252
0.054684483	1.177691641	0.053909604	0.05408331	2.233635473
0.194647308	7.114806305	0.191075026	0.191873018	1.59702797
4.595275318	1.592913043	3.469384159	3.594976115	3.59486327
0.346751873	1.980687893	0.338822187	0.340586488	0.325125931
0.009065766	2.628780841	2.040814684	0.008975773	4.13411157
0.308923435	5.627924184	6.355649738	0.303701404	1.894997747
0.014172347	3.070639716	0.013988777	0.014029968	3.069638111
0	2.076847515	2.075134834	0	3.428570299
0.088562522	2.704841671	3.401989149	0.08751889	1.926789048
0	3.060173754	4.34537215	0	3.696198744
0	4.618938685	0	0	1.804452337
1.153059324	5.366547979	2.198928232	2.199014738	0.044147709
0	3.431416732	0.981627865	0.981635795	3.972063957
0.868659443	5.104293431	1.42263937	0	2.612742796
0.066924607	0.070240526	0.065951717	0.066169746	3.112019772
1.675692569	6.693952643	0.218605658	1.675746434	2.361782524
0.145397862	4.832217158	4.338024014	0.143491992	2.570574562
0	3.626213227	7.918498802	0	2.659148892
0	3.891226616	0	0.74244274	2.767217966
0.507138888	3.688455116	1.804858533	0	3.218475071
0.030219388	6.746153294	1.101907464	0.02990445	2.459831531
0	2.276293096	0	0	2.276292153
0.001429344	4.708216658	1.004909952	1.00490789	1.004919208
0.066543851	5.505415797	2.615732199	0.065793876	3.110418639
0.884450088	2.635687638	4.768599242	0.884219386	3.521487303
0	4.915336471	0.781098753	0	2.261889305
0	6.133888369	0	0	2.757582323
0.067877223	2.892774014	2.891562675	0.067110107	1.223827801
0.358465729	4.126608823	3.218740469	0.351994807	3.551878587
0.855323518	2.117825371	4.22686894	0	2.781927547
0	6.071399078	1.887917481	0	1.482172826
0	5.856269869	2.125959697	0	1.414128267
0	2.651956839	1.835881039	0	3.250162738
0.025342546	2.930112294	6.65489729	0.025081325	2.438231058
0.004625428	2.830317061	0.004566846	0.004579994	2.343685616
1.696877029	4.844463515	1.696889199	0.027130519	4.459896289
2.496802951	5.296557384	2.685176566	0.794871936	2.282946378
7.292752609	3.895077553	3.20008801	6.107382502	3.778577541
0.108592224	4.315060601	0.106878118	0.10726186	1.997210134
0	4.739994137	1.731146102	0	2.73218521
0	4.550789849	2.486042635	0	2.990926615
0	3.682101025	2.916872474	0	2.476595976
1.47536364	3.765747464	4.181149556	1.47528587	4.182731962

0.060203682	3.878958913	0.059340628	0.059534074	2.856916784
0	3.232517177	3.236485929	0	2.807202057
1.365863023	2.014016475	2.463849047	1.366252947	2.016416274
0	1.242668139	0.897980987	0	0.896566768
6.461605898	4.963217486	2.92777281	7.458926275	4.447620518
0.035455733	3.344237492	1.119174674	0.035081883	3.340889468
1.079640008	5.295316792	0.023254573	0.023324547	0.022699867
0	7.207082562	0.541452945	0	4.73745871
0.43964252	8.013572564	0.42838031	0.430879778	2.202142209
0.102395937	2.419080187	4.053474836	0.101156268	1.330699151
1.689144819	4.941802123	4.522724767	0.025213424	2.704825308
0.094896372	2.390865865	0.093437337	0.093764087	2.725850548
0.022319352	1.073973764	2.691120022	0.022090871	3.27939874
0.231702472	3.938789017	0.227194687	0.22820069	1.697131264
4.410487069	4.337541146	2.075913404	4.410043735	4.731070076
0.971010412	5.123171565	6.976543229	1.961415582	3.656498281
0.081003564	1.897099195	0.079791828	0.080063289	2.337158359
3.191189991	3.461679332	2.415423271	2.024378908	2.980962574
0	5.615663537	2.297706657	0	2.974462833
0.094967883	8.664145542	0.093507545	0.093834586	2.390041093
0.145498278	4.65980579	2.124312355	0.143590752	2.121425802
0	4.808418217	2.686789195	0	4.320557455
0	2.120663291	0.690823414	0	4.007933683
0	4.001757122	0.576691747	0	5.161338899
0.166822045	10.51173798	0.163898734	0.164552222	2.190203277
0	5.545589839	3.733817724	0	0.889359953
0.113034067	6.611001289	0.111234833	0.111637588	2.456517617
0.075278096	8.550578129	2.651334771	0.074414337	4.260262192
0.01979773	1.065720676	2.679411088	0.01959623	3.420027201
0.011370405	3.380018635	0.011224086	0.011256921	1.632004376
0	2.151071713	1.900026011	0	2.362777226
0	3.334919169	4.572829419	0	2.209433422
0	3.530529552	0	0	2.27300377
1.099636983	2.457568417	1.70555296	1.09980387	0.028492005
0	2.292679111	5.873603483	0	0
0.115666322	3.848353408	2.023525226	0.11423023	0.110552061
5.783898263	4.46256207	4.257177214	6.43671044	0.093023053
0.011433139	3.867149217	1.632333997	0.011319012	1.03908615
0	3.247913935	0	0	2.855604289
5.873293004	3.536397092	1.118723159	4.815972951	3.087758713
5.125547089	3.334332246	3.332802621	6.800645825	3.622628648
4.507708982	5.45309975	3.355555863	3.426716613	5.318880742
0.081358968	3.973099186	1.265993738	0.080413893	1.900904684
0	3.492425196	3.734722143	0	1.943325054
0.0891406	2.707135277	0.087785419	0.088088955	3.727198879
3.237395343	3.83123175	2.814565901	2.546006734	2.213969767
7.945587768	3.347479969	3.726146393	9.193933538	2.345253323
0	3.170453372	3.752827117	0	3.172652712
1.176152657	1.849076416	3.267729055	1.844706845	2.303625763
0	2.92457607	3.060358463	0	1.96198261
1.472154362	3.236755304	2.719137564	0	3.009577107
3.853934946	2.568895507	2.838323091	3.852714506	1.183871928
0	4.374479404	2.247671915	0	1.929294361
0	6.623179056	0	0	5.096018651
6.936978474	3.295422673	0.894335592	7.058352426	2.173866337
0.698426226	1.884892256	3.407516831	1.20593495	2.527576119
8.252449561	4.524670369	6.486149729	7.523110013	2.171514702
5.556609297	4.992066318	2.041458067	4.003379128	3.856013443

hsa-miR-517c-3p	hsa-miR-1283	hsa-miR-942-3p	hsa-miR-3189-3p	hsa-miR-514a-3p
0.101247021	0.099269995	1.324568413	1.316710564	1.339927332
0.065654089	0.064459067	1.835956946	0.048854737	4.145247438
0.031632748	1.111931256	1.107132856	1.105010495	4.258247392
0.01644913	0.016180057	1.651440179	0.012569524	4.615075907
0.109550952	0.107378027	0.085497567	0.079677348	3.519728377
0.046042773	0.04523847	2.190485116	0.034621782	7.728096491
0.147204236	0.144079553	0.11312811	1.443679028	2.159007649
1.0702651	0.019376753	1.067378276	0.015027885	1.068910579
0	0	2.48938351	0	3.586556907
0.043334799	0.042582196	1.145381421	3.184697609	3.023691616
0	0	2.686625135	0	2.630857898
0.205891171	0.201077269	2.714416381	1.593224068	0.2338116
0.100664582	0.098701105	0.078852448	0.073549281	3.481485386
0	0	2.298796529	3.883238775	1.975938388
0.174875523	0.17098545	0.132911041	3.797717356	0.197242539
0.038891862	0.038222895	1.130944681	1.128287851	3.197921245
0	0	2.906842307	0	0
1.53758254	1.061301945	0	0	3.757620779
2.323824488	1.886139892	0.058374046	0.054597251	2.326324123
0.223592478	0.218220265	1.656082657	0.153757827	2.890456513
0.161456246	0.157944301	0.12337111	0.1144056	4.934526523
0.264060572	0.257327723	1.754109228	1.7294322	0.303766554
1.6958127	1.092089163	0.02097411	0.019717096	5.636449557
1.453139889	2.111550414	1.422663555	0.097107135	3.627337273
0.134006262	0.131226993	0.103538502	1.408162067	2.911125458
0	0	2.371503787	0	2.519984331
1.559363233	0.978937941	1.974211302	1.974878986	4.721378289
0.056723141	0.055709602	0.045263296	0.042409735	2.25484594
0.078238034	0.076777245	1.255308936	1.249486476	3.556197961
0.036538946	0.035913675	2.479448909	0.027616618	2.991080001
0	0	0.498613992	1.842521898	1.7919602
0	0	2.32154962	1.58462692	6.671940654
1.350673698	0	1.015074666	0	2.074922301
0	0	0	0.767427553	1.977595444
0.088676478	0.086986336	0.069810473	0.065193812	4.536632327
0.251286053	1.792978349	1.723713349	1.700451546	5.432832464
0.116431823	0.11409273	1.368965818	0.084384741	1.388205202
0	0	1.366388674	1.370598059	3.786734325
0.076683466	0.075256144	0.060674289	1.244850763	8.422274286
0.019869921	0.019542335	1.067945081	0.015155001	5.469047491
0.091957199	0.090193309	1.926816124	4.368807144	4.159155236
0.060501163	0.059411576	2.245839408	3.422201253	1.207157703
0.116025812	0.11369662	1.367791842	0.084107881	3.547239616
3.266371841	1.190628115	0.043608603	0.040868527	3.600471649
0	0	0	2.241239774	2.231766887
0	0.924167681	3.06794229	0.927771479	2.216334834
0.14844231	0.145284572	2.106510074	0.105864322	2.163394492
0.174854169	0.170964708	1.530835347	0.123087434	0.197217533
0.104353691	0.102303969	2.734456046	0.07610001	3.672288714
0	0	2.143515101	1.428062307	3.867768962
1.008822155	0.002409761	1.595024104	2.010444091	2.82014474
0	0	1.54482693	1.553515467	2.064308099
1.1737368	0.048681378	1.165415921	1.161944633	6.536385283
0.01950993	0.019188543	1.066733827	0.014883371	2.416948071
0.013119087	0.01290613	1.045110808	0.010043291	1.046052744
6.712642409	6.798793282	1.704972475	0	3.216394329

0	0	1.453964344	0	1.722742119
0	0.864035338	0.867906346	0.869900775	1.818742585
0	0	2.170128519	0.711990829	0
0	0	1.181612383	3.42367157	4.181352541
0.032339061	0.031790755	1.109462085	0.024498026	5.670672614
0	0	1.389307618	0	6.244250471
0	0	1.200925866	0	2.317283094
0.008398662	0.008263823	0.006843973	0.006446371	3.953290945
0	0	0	1.969000899	1.965822276
0.06062162	0.05952959	1.817992205	4.502529074	2.876642257
0.0562736	0.055269038	1.802313311	0.042083714	1.19309299
0	0	0	0	3.97733906
2.298124188	1.977279489	0	0	3.673730933
0	0	0	0.584006979	2.313566068
0	0.93021332	3.365042406	0	0
0.051903455	0.050985389	1.786404799	2.534152112	1.178493232
0.181974357	0.177878972	0.137917375	0.127656214	6.446351974
1.593009444	1.592988857	2.593461801	2.007864867	1.00650764
0.318981308	0.310215925	0.229474573	0.210006186	3.204958274
0.008647789	0.008508869	0.007046209	1.02930106	3.74781887
0.285319128	0.277824821	0.207825074	0.190691903	4.364282204
0.013511356	0.013291832	1.639756743	0.010341355	2.653206555
0	0	2.093144288	3.086962415	0.829759551
1.925935489	0.082168328	0.066067966	0.061729661	1.283509417
0	0	2.489945776	1.502728548	2.480510345
0	0	1.414342469	0	3.096142704
0.043626251	0.04286811	1.14632485	0.032847356	5.190607732
0	0	0	1.564345122	2.558724351
0	0	1.430677995	1.433820699	4.428936911
0.063436401	0.062286997	0.050475495	0.047260003	1.216889487
0.207743763	1.669148577	2.276098156	0.143937112	5.763368169
0.136652717	0.133805205	2.070593005	0.098032325	4.564263023
0	0	1.576482962	1.201476219	3.368686576
0	0	0.746939163	1.281458989	3.660070258
0	3.382393762	2.052800975	0	3.745259546
0.028759345	0.028275596	1.097629507	0.021828725	5.619132135
0	0	0	0	0.96761062
1.004919876	0.001343042	1.590577269	2.00582975	4.255618774
0.063078106	0.061936038	2.255503048	0.047002005	4.233518503
0	0	2.651607996	0	0.886259796
0	0	0.785535456	2.293321736	4.278439343
0	0	1.545386456	0	5.302104292
0.06433269	0.063164893	1.212239322	0.04790497	2.893391729
0.329350743	0.320177752	2.576977999	0.215845047	3.598921754
0	0	2.801427912	0	0.857581604
1.482271177	0	0.918620973	0.919929294	5.072908106
0	0	1.422460691	0.867023349	4.527876649
0	0	1.175116933	0.422230382	2.011477278
0.024131011	0.023729309	0.019528824	2.424581002	3.296179531
0.004414326	0.004344117	1.015288858	1.602636268	2.343769715
0.026097918	1.09265047	1.088787438	1.087061213	4.141471862
0	0.79400261	1.737164207	0	5.582842032
5.978368129	4.939828544	0	0.645795327	2.422154592
0.102467482	0.100461981	0.080204557	0.07479704	3.489290685
0	0	1.35431145	1.098722497	4.600756498
0	0	0.791961886	0	3.244492695
0	0	0.67219951	0	2.291391227
0.910434275	0.910442587	2.879799783	0	3.945315359

0.057107586	0.056086357	0.045562583	1.799399408	4.104027919
0	0	1.099583012	1.778165556	3.135636211
0.107661831	0.105533918	2.413857359	1.335001646	1.360397418
0	0	0.91066636	2.17748869	0.474762613
5.060383616	5.714224699	2.887648668	1.232418924	3.685053437
0.033723367	0.033149838	1.114019405	0.025527495	3.172591512
0.022444221	0.022072021	1.675035128	0.017094314	4.768073258
0	0.538999774	1.373712401	2.315802919	5.596057844
0.40051421	0.388341485	0.279852527	0.254594484	6.200751209
0.096685563	0.094813984	0.075861215	0.070787338	3.034857386
0.024257827	0.023853898	2.91675377	1.679702968	4.454931708
0.089677137	0.087964585	2.351903528	0.065895186	1.302720098
0.021259386	0.020907782	0.017226465	0.016202413	0.023199967
0.215760605	0.210638191	0.161372356	0.148922726	2.39373076
3.08287785	3.405434488	1.055592141	0.012387301	5.090975779
0	0	2.285014781	2.286044934	4.708489245
0.076664008	0.075237105	1.250480482	0.056717323	2.675485381
1.782682773	2.028816712	2.273506808	1.520771363	0.640122301
0	0.982500684	0.983115749	0.983408512	3.432773012
0.089744018	0.088029965	0.070619304	0.065942038	7.349677473
0.136745613	0.133895697	0.105537246	0.098094393	4.1073001
0	0	1.017395308	0.551616426	4.174242386
0	0	1.210708084	0	1.873325501
0	0	0.580581888	0.584741273	2.977949881
0.156427139	0.153053535	0.119769847	0.111117698	8.543108612
0	0	1.456125289	0.894291129	3.412128421
0.106607514	0.104504609	1.975340112	3.41551338	6.033439616
0.071289641	0.069977051	1.233904583	0.052890593	7.645811786
0.018862731	0.018552464	0.015300498	1.063330655	1.066007105
0.010843429	0.010668341	1.037356033	1.036668148	2.050802561
0	0.708671902	3.961867168	3.905273348	1.902533691
0	0	1.491335386	0	3.574284436
0.964570964	0	0	0	3.116715981
1.099964344	1.704728651	2.70991446	0.021385951	2.129819952
0	0	1.560633897	0	2.554705353
0.10905904	0.106897861	1.347535939	0.079339564	4.445377633
4.836366834	4.831362447	0.072181248	0.067386498	3.913119737
0.010903181	0.010727103	1.037560022	0.008357124	3.227864123
0	0	1.0485029	0	1.394886575
5.199325551	4.957233375	2.619905298	0	3.080108676
4.164572896	2.963597742	0.025598746	3.137692865	3.334536853
3.861275741	3.968557499	3.002812881	0.965128236	4.684105636
1.265901814	0.075563333	2.904400708	1.245787885	0.085027153
0	0.468364827	0.917964794	0.478078783	2.868184724
0.084290574	0.08269778	0.066479914	1.26742023	1.927563417
2.213326728	0.04649546	2.195471663	0.035561024	2.546156973
7.640567845	7.185451781	3.022169003	0.003641077	2.022312439
0	0	1.470183869	0	3.930321056
2.093565978	0.672726086	0.678849664	0	2.30242904
0	0	0	0	1.962954587
0	0.444289906	3.932342869	1.518205056	2.717065385
3.963827118	2.833450347	1.174871002	0.039348162	2.569506027
0	0	0.94840479	0.949264242	4.669397331
0	0	0	0	5.579975038
5.666511009	5.261682746	1.461727237	0.898843163	1.859246039
0	0	1.603370415	1.225521073	1.586100655
6.570921392	5.921559374	2.7494124	0.028382127	4.450230066
3.68891489	3.859929176	1.749247015	0.810705341	4.918646163

hsa-miR-525-3p	hsa-miR-92a-1-5p	hsa-miR-548f-3p	hsa-miR-519a-3p	hsa-miR-3691-5p
0.1006695	4.671562194	0.090034809	0.107007987	1.952789372
0.065305354	6.298286352	1.223987312	0.069117064	0.050662753
0.031476627	2.741861622	0.028556384	0.033176456	1.106195608
0.016370715	4.177290617	1.058026223	0.017223035	0.012997268
0.108916056	3.318860675	0.097246754	0.115891041	0.082824988
0.045808187	2.809883657	0.041434405	0.048366494	1.152714388
0.14629027	3.239772365	0.129634038	0.156376079	0.109409306
0.019606808	2.908264157	0.017832044	0.020635746	1.066813537
0	5.092209502	0	0	0.929748367
0.043115309	6.788373117	0.039020489	0.045508255	2.994500341
0	3.366631748	0.582614107	0	1.738084118
0.204480727	3.694168506	0.179115142	0.220156243	0.149135621
0.100091029	4.076710226	0.089528009	0.10638554	1.319367322
0	5.328549986	1.976627148	0	1.977876463
0.173736776	4.253324763	0.153113501	0.18634499	2.949517207
0.038696787	5.938468303	0.035053847	0.040822475	1.129770185
0	2.84224675	0	0	1.705712461
0	3.240347072	0	1.319183939	1.355915161
2.323316376	2.32780637	0.065911905	2.327442415	0.05664331
0.222017644	3.959397903	0.19380846	0.239558283	1.64695645
0.160428586	3.727941306	0.141760626	0.171788435	1.488768313
0.262084547	3.379349815	0.227009822	0.284204562	0.186622945
0.025810951	2.447845052	0.023444102	0.027186026	0.020399702
2.113653937	2.916067801	0.119423269	3.795156224	1.417711073
2.109207221	5.104579045	0.118339991	2.566726197	0.100192591
0	0.696438963	0.694501988	0	2.376417676
0	4.55185958	0	0.979015821	3.779580246
1.198014642	4.09994896	0.050927398	0.059656169	1.801302817
0.077811591	2.346724277	0.069918051	0.082479419	1.252721511
0.036356625	4.443142998	0.03295006	0.038342811	0.028590656
0	3.5472338	0	0.496896728	0.500454682
0	4.643385465	0	0	0
0.540008536	4.261222643	1.00412929	1.632678296	1.018398815
0.758858203	3.416351976	0	0	0
0.088182936	4.839341014	0.079068842	0.093591784	0.067693044
0.249440477	3.854510431	0.21658707	0.270067314	0.178542724
0.115748242	4.566003002	0.10320362	0.123264177	2.436508506
0	5.47382807	0	0	1.766924321
0.076266811	1.900854691	0.06855175	0.080826678	1.248012405
0.019774445	3.103163518	0.017983875	0.020812606	1.664071046
0.091442074	3.014347531	0.081936452	0.097089612	1.92235317
0.060183242	3.103738539	0.054274913	0.063656097	1.198280232
1.392255764	3.115028323	0.102852611	0.122828808	2.000031692
1.81239108	4.81032113	1.187653558	2.246801451	0.042354346
0	4.219537517	0	0	0.937706937
0	3.968146286	0	2.695901519	0
0.147518642	0.160574813	3.614963997	0.157713016	2.098938507
0.173715602	2.719442238	0.153095485	0.186321792	0.128376136
0.103754881	4.318543318	0.092735914	0.110329406	1.330105098
0	5.904633803	0	0	1.829772993
1.008821567	3.71389732	0.002224641	0.002559775	0.001948071
0	3.871113517	1.156655987	0	2.316142711
0.049299124	1.793312696	0.044559699	0.052074594	3.213380673
0.019416262	2.091184755	0.017659452	0.020434726	1.066174886
0.013057032	2.875065041	0.011891487	0.013731285	0.01038297
7.643371219	2.645432286	0	7.441477038	0

0	3.11562858	0	0	1.088442424
0	4.103639162	0	0	0.868777171
0	3.673785619	1.21807882	0	0.7099846
0	4.381924175	0	0.66891387	2.837446
0.032179201	2.47789289	0.029189493	0.033919878	1.108502098
0	4.483139134	0	0	2.364263533
0	3.712787372	0	0	1.203698211
0.008359375	2.039683924	0.0076207	0.008786018	0.006662478
0	4.207359732	5.132474931	0	2.289450211
0.060302985	5.017324365	0.054381554	0.063783683	1.815157693
0.055980522	3.280443297	0.050528717	0.059180445	0.043617894
0.985451174	3.884429925	0	0	0.986077293
2.974860071	2.297884389	0	1.97705254	0.983404952
0	3.836779801	0	0	1.971644398
0	3.592198101	0	0	1.914607546
0.051635645	3.432336024	1.178709567	0.05455823	0.040314484
0.180775263	3.372251629	0.159093885	0.194063056	1.5425581
1.006639634	7.267912249	1.592827208	2.008762821	3.008540879
0.316404613	0.353851783	0.271226917	0.345449362	1.865446549
0.008607313	5.440976635	0.007846319	0.009046883	0.006859264
0.283118255	3.732943817	0.244238301	0.307821099	0.199887282
0.013447386	4.322281266	0.012245991	0.014142469	1.63917013
0	4.416227597	0	0	1.382036805
1.288034233	4.592727198	0.074754467	0.088343204	0.064078765
0	3.587655632	3.066715736	0	3.487825406
0	4.891482257	0	0	0.859279637
1.153427623	1.152646736	0.039280429	0.045815798	1.144991459
0	4.141264674	0	0	1.977738243
0	4.246933858	0.868917241	0	0.872569039
0.063100999	3.313739002	0.056871915	0.066766041	1.207398746
0.206316431	4.377166188	0.180657862	0.222183236	1.607913803
0.135820074	4.101334657	0.120609662	1.458308055	0.102049368
0	2.097782935	0	0	1.197901394
0	2.199416209	0	0	0
0	3.568662906	1.822150146	4.899286608	2.404652971
0.02861832	3.610851759	0.025978773	0.030153279	1.096783983
0	4.118297955	0	0.96707754	0
1.004919571	2.591964186	0.001240115	0.001426421	1.004701219
0.062744848	5.790363224	0.056555084	0.066386313	2.580588339
0.883822562	5.133388646	1.848954197	0.884396107	1.856326036
0	1.701757345	0	0	1.72145356
0	3.754127067	0	0	0.966987927
0.063991912	4.418808561	0.057664267	0.067716079	2.256616373
0.326653405	3.233205935	0.279469726	0.35709833	0.226665261
0	4.971800319	0	0	2.134165459
0	4.325798706	0	0.91636495	0.919193685
0	4.861221143	0	0	0.865879069
0	4.345402771	0.412949369	0	1.455911622
0.02401392	1.085306942	0.021820067	0.025287718	0.018995724
0.004393872	5.451257838	0.004008957	0.004615896	2.019059287
0.025970714	4.140442756	0.023588426	0.027354833	1.088025744
0.794036369	3.257179331	0.795212872	0.795199466	1.739746904
5.550272833	2.72779107	1.131525356	6.746532478	0.643562843
0.101881623	3.288958003	0.091096367	0.10831254	0.077722469
0	2.049723699	0	0	0.769284563
0	4.172821414	0.787988496	0	0.793197984
0	3.349645181	0	0	1.564455316
0.910429994	3.76633152	4.453563156	0.910850999	2.689779203

0.056809639	3.611139041	0.051268285	0.060063042	1.187682894
0	4.623218017	0	0	0.611294556
0.10704012	4.52805167	1.357397614	0.113868736	1.973469169
0	3.604389808	0	0	1.2604589
4.881875762	5.80250023	0.064900798	6.180710864	1.235386242
0.033556147	3.497335581	1.117554147	0.03537725	2.139909934
0.022335735	3.820659111	0.020302334	0.023515703	0.017682423
0	4.920050907	1.357923105	0	1.913571001
0.39692727	6.218171552	0.335165306	0.437787598	2.033118408
0.096138915	2.421958996	0.086062375	0.102135351	0.073533027
0.024140086	3.937460877	0.021934127	0.025420967	1.081947202
0.089177037	2.731393923	0.079943922	0.094658394	1.287013259
0.021156906	3.814482115	0.019235577	0.022271398	2.414286094
0.214259339	4.107833685	0.187320435	0.230964454	0.155623584
3.405903396	5.412134575	0.014681834	4.548113548	1.055132206
0	1.549547252	2.283220437	0	1.964702465
0.076247476	1.263814031	0.068534643	0.080805995	1.247953389
1.783041409	3.08607538	0	3.087588927	3.152524285
0	2.974114939	0	0	0
0.089243479	3.234305996	0.080002396	0.094729692	1.915014976
0.135912265	4.456162728	1.4457137	0.14509696	0.102114466
0	4.756701987	0.542070515	0	2.311238095
0	5.166342481	0	0	1.59733127
0	7.079329521	1.702508528	0	0.582368053
0.155440103	3.508216451	2.162572954	0.1663443	0.115786748
0	3.00138764	0	0	1.45731327
0.105993129	4.219933344	0.094693285	0.112740457	1.336634696
0.070906536	6.043185931	0.06380408	0.075096625	1.852468006
0.018772305	3.559956193	0.017076069	0.019755441	2.07983237
0.010792412	2.863469681	0.009833703	0.011346592	2.046400272
0.708775454	2.54461231	3.013796097	0.710578723	2.755301928
0	2.688203363	0.919856465	0	2.219364497
0	3.944903278	0	0	0
0.028028961	2.72519531	0.025446909	1.099677544	1.695999687
0	1.971903061	0	0	1.56087871
0.108427585	3.086301211	0.096820235	0.115364367	0.082471702
5.573678569	1.959051673	1.307890623	5.548154422	0.069981748
0.010851875	2.375706949	0.009887761	0.011409191	2.046652098
0	3.06216761	0	0	1.051762757
5.596113648	3.614166746	0	5.873965129	1.136007007
5.210327142	3.625928313	0.028649221	4.871118806	0.024890456
4.232416058	5.810222961	10.02559192	4.508398396	3.012080722
0.076578771	2.949629148	1.260726534	0.08116039	1.248964322
0	2.657642082	0	0	0.475598589
0.083825516	3.876364668	0.07522894	0.088919596	2.658209216
1.783310114	4.642517758	0.04257589	3.041658547	2.192884501
7.02579074	6.176634828	0.004299112	8.561951658	1.604160919
0	4.013290515	0	0	1.471255152
2.094027084	4.393444024	1.179744872	2.927425043	0
0	2.775967492	0	0.750509777	1.988414633
0	4.093816315	0.444346954	1.472020255	2.292249499
1.804073491	1.182892452	0.047187918	3.970169572	1.173230885
0	4.237893497	0	0	0.94878156
0	4.872812416	0	0	0.97933463
6.973641216	4.415127834	1.862486695	6.894771314	0.897937413
0	1.881101923	0	0	2.380991313
6.365161401	2.502174987	1.739574487	6.78729701	0.029384949
4.492560506	4.201008569	0	4.58392084	0

hsa-miR-4664-5p	hsa-miR-518c-3p	hsa-miR-6716-3p	hsa-miR-154-3p	hsa-miR-141-3p
0.076222484	0.108221426	1.946041501	5.26464124	3.482850441
1.213706493	0.069842825	1.211305952	1.226103594	5.392087071
1.105949343	0.033498468	1.104874369	2.967436027	3.487123378
1.650535423	0.017384137	1.055277834	2.892024203	3.896875165
0.082151569	0.117228045	3.229796423	4.235648163	8.462746158
0.035598047	0.048852173	2.511352259	1.160139093	2.808871616
0.108474319	0.158320794	0.104565351	1.488289129	3.473269885
0.015432727	1.069896024	2.670509544	3.426426508	3.914147515
0.929882107	0	1.502360785	2.219815777	4.131820839
2.993756885	0.045962358	0.032503902	1.76757298	4.40113362
0	0	1.74529938	0.588212376	4.609614306
0.147770372	0.223207253	1.592090416	4.077239647	4.510381461
0.075804891	0.107590452	1.944192386	0.109857115	2.43402345
3.14618265	0	3.298495364	4.293211916	8.930159501
3.442655776	0.188786775	0.122519319	0.193405423	5.349037273
2.975744819	0.041225595	1.733932506	2.178264471	4.378511877
0	0	1.418313673	4.498752921	3.406214048
0	2.419866957	2.127825918	3.299922738	3.669012771
1.238258815	2.663103531	1.235523343	3.164286435	0.082016687
1.644611897	0.242982157	0.152992474	0.24948276	3.525415523
0.118205513	1.534939569	1.480369919	2.671135811	3.731199693
0.184809803	0.288551184	0.177278513	0.296830258	5.151732443
1.687182332	0.0274463	1.685891283	1.091164385	2.938843346
0.100228372	2.915527209	1.410898401	2.119232329	3.192080065
1.412944055	1.449362652	0.095828267	3.421470451	3.187027246
1.219070264	0	0.703183746	2.518899754	3.656037478
2.558251396	0	2.296069744	1.559435132	4.359083143
0.043626701	0.060270102	0.0422383	1.196218713	3.610087962
0.059550308	0.083369885	2.300845045	1.267788049	4.204382021
0.028382956	0.038719344	1.1206164	1.127710884	4.366438922
1.595256759	0	1.843459085	7.535487494	10.28171252
1.584623443	0	0.999715732	1.584598697	4.247457125
0	0.544224047	2.999913624	3.197451172	4.654803571
1.693414869	0.760836521	2.007101687	2.792001209	4.548125833
2.672354699	0.094625256	1.279876881	2.727520713	5.098874255
0.176829483	0.274111957	1.699022691	1.800294338	4.404112733
0.087030037	0.124706412	1.359118551	5.789061522	2.838914037
1.767539818	0	0	7.463479502	3.291412361
1.247352432	0.081696342	1.244489814	1.900146378	1.896377004
1.067225235	0.021008934	1.662863988	4.356902903	4.357231656
1.292959283	0.098169233	1.289433815	0.100198937	4.368594919
1.197776486	0.064316825	0.04495999	0.065556261	3.103200505
1.362603411	0.124264761	1.357985146	0.12696905	3.721462568
0.042037249	2.849161222	2.216392068	3.273269825	7.601158331
1.510971436	0	3.085923239	8.170602319	4.895324314
2.226148817	0	0	1.492701463	3.342873564
0.109324614	0.159679049	1.446200102	3.246484904	6.773379538
1.522327621	0.188763177	0.122505649	2.25891208	4.509086098
1.329160827	0.111588635	1.955866021	1.352798114	6.764480947
0	0	0.869057912	6.312624392	1.818309226
2.010568045	1.008769796	0.001878622	2.597513366	3.598390327
1.852010872	0	0	3.015425245	4.60813292
0.038246376	0.052601776	0.037042885	3.958432716	2.555200934
1.066027745	0.020627319	2.405332104	4.692763346	1.667730636
1.637473339	1.046711017	1.636832923	0.014096854	3.771810689
2.275306712	8.513779283	0	6.966709337	1.995740666

0	0	0.603012001	2.780407202	4.18735853
0.869008712	0	1.429452768	0	2.388326765
0	0	0.712225782	1.592809836	7.781665507
1.566253221	0	0	5.940020763	2.916920695
1.108249886	1.113955376	1.107149068	1.113280568	4.080193069
1.792187136	0	2.36794561	2.913186579	2.55947091
1.587517245	0	0	2.941621528	4.100561648
1.028698161	0.008866566	1.028429175	2.363446294	2.363124459
0	0	1.555883831	2.547817788	2.961648882
2.837062763	0.064445916	1.811271886	1.837082899	3.895613351
1.184605196	0.05978886	2.222538748	1.194707671	2.855870627
0	1.567277703	0	3.299953127	1.980974921
0.983438612	3.4331395	0.983586403	0.982873404	3.973006948
1.431633831	0	1.436665931	3.646277652	4.000656501
0	0	1.506146026	2.704640687	3.797440442
2.209575661	0.05511355	0.038750161	0.056154635	2.565721741
1.540726931	0.196639379	0.127048019	1.594984874	5.172518961
1.006330934	4.180187852	0.001413928	2.817000337	4.765389702
0.21819036	0.351209579	0.208867006	1.992942494	4.297358544
3.039499277	0.009129875	1.619347824	0.00928501	5.74995568
1.788229052	0.31269249	2.881434283	3.739248888	5.401162105
1.639017516	0.014273795	0.01030246	1.047808771	4.244676222
1.382499766	0	0.833226876	2.329956647	2.331755874
1.268590655	0.089308447	0.061469823	2.369251545	3.735745357
1.501929554	0	1.502844007	1.903369516	3.060313412
0	0	1.417988827	3.231731374	3.23229827
0.033769855	1.152819013	2.175425641	3.546967742	2.797635774
0.982411272	0	1.978097856	4.05874636	3.556793305
0	0	1.434021501	3.876225889	5.509171389
1.824317226	0.067463643	0.047066972	2.284513645	3.487162134
1.605767045	2.835168469	0.143232134	1.672389472	2.831440839
2.498845468	0.146763405	1.414640971	2.578225401	3.631642723
1.581113578	0	0.687229917	3.055258141	2.802928884
2.233385651	0.74346219	0	2.412752277	3.0459018
1.324436814	0	0.513379704	0	4.298648458
0.022426338	0.030443935	1.095591103	0.030987984	3.472611869
0	0	1.547893769	3.756112742	5.470186988
1.590504326	2.006494548	2.32792457	5.217248336	4.708215234
1.205759944	0.067079389	1.203465191	1.84681999	4.327438809
0.888228639	0.884658128	0	0.885359537	4.717496913
0	0	2.036905686	2.661765494	1.705459633
0.967053621	0	0	2.274076222	2.274458355
2.25569813	0.068425031	0.047709037	4.332442051	2.620641357
1.883953301	0.363146892	0.214664404	2.75658541	4.125350397
1.41650938	0	0.860814633	0	3.470693742
0.919345555	0	0.920014522	2.463658291	1.4839296
0	0	1.829782731	3.242629305	4.358861005
2.376445257	0	2.242224355	3.489608477	12.60653695
1.680269741	0.025528758	1.679071753	3.296176572	6.943673178
2.018999785	0.004657789	2.341235041	2.830396786	5.382876049
0.020377991	0.027616825	1.68649612	5.636387205	4.229238814
2.528102025	2.027873285	0.802052175	2.683826184	3.98380943
1.822884263	7.087502827	1.828215205	4.32026431	3.469219095
0.077096786	0.109543905	1.319702646	1.346723168	4.90807685
1.362019766	0	2.740893125	5.55620426	3.889854065
1.730827971	0	2.711692746	0	5.521454172
2.522602728	0	2.125775268	3.953368528	3.755000565
0	0.911051252	0	2.19651985	3.44599636

3.242216907	0.060681703	1.185158081	3.612143593	7.077803412
1.103546956	0	1.778881454	4.345887604	3.770321571
1.338701701	2.01887891	0.078038144	0.11764019	1.357595808
0.915017933	0	1.267316121	2.529128546	3.481632312
1.234767076	6.52586352	0.053556205	1.248955418	7.933965287
1.716642369	0.035722343	0.025428068	3.497864258	3.636424444
1.075766912	1.681445424	2.092894646	1.681120873	3.287289236
2.124962746	0.54352203	1.677274745	0	4.078324598
0.265172854	0.446005886	0.253126541	2.216851354	3.450440982
0.072945936	0.103282502	0.07048388	3.463848347	3.793678169
1.680756024	0.025663352	1.679551517	1.085337719	2.705610028
1.28622232	0.095705881	1.282797406	4.255827262	3.762460209
2.089391168	0.022482204	2.088136158	2.916188521	6.142401988
0.154183684	0.234221076	1.615927723	3.498893144	3.734348081
0.012718896	5.17728297	1.054481007	1.057268363	3.546267969
0	1.961401196	0	1.549657652	3.126353612
1.247293608	0.0816754	1.244431818	0.083308126	3.176366452
0.640285179	2.232212965	2.072948613	4.312992793	3.19031432
1.978902952	0	0	1.976874519	3.432828174
2.345762958	0.095778117	0.065662086	0.097748862	5.103837555
0.101253388	0.146865014	2.490526369	1.455647665	3.808188237
1.919295864	0	1.025514118	5.731469442	4.918221954
2.875816333	0	1.217325708	3.48206812	4.673749076
0.582851287	0	2.378302182	3.579059055	2.476232639
2.12031675	0.168449884	0.11060353	0.172427083	4.646425675
0.893544051	0	2.855290276	3.526763905	4.070662847
0.080055173	0.114033233	1.331482307	1.360044216	4.529057783
0.054443585	0.075895111	1.847845708	0.077394087	4.763030119
2.403871812	0.019941335	1.063251898	2.679890024	4.860042514
0.008531904	0.011451256	2.632700638	2.051034101	2.374979777
1.238411408	0.711256118	1.935939735	2.544584709	6.337425235
0.922406616	0	0.923052511	1.892402981	1.487765319
1.95712683	0	0.966409423	1.541993557	3.944640463
1.094620412	1.705478886	0.021303432	1.705083109	3.85090774
1.974400835	0	0.979901727	0	2.554823374
2.410732711	0.116693947	0.078993661	1.367910192	4.539932918
1.292519894	6.04979668	1.289001012	1.958280569	3.012515794
1.037175191	0.011514448	1.036823609	0.011711232	2.863563355
1.958227719	0	2.176459022	4.224045331	12.27568569
0.637309694	6.033669618	0.639365907	2.227568646	3.536891454
1.106286817	6.30248098	0.023955933	0.03421359	2.47392763
0	5.073938353	1.850464818	5.750600648	9.578198488
1.248301241	0.082034244	0.056716607	3.179130696	13.1777969
0	0.907454969	2.006856435	3.927416022	3.779021532
1.270207883	0.089892272	1.267018327	4.122928506	3.408443403
1.156415126	3.563912719	1.764413259	3.704098418	4.499806512
1.016228222	7.526704247	1.603880358	1.016832459	6.436788401
1.878638322	0	1.472754662	1.464549707	1.466009351
0	2.926572252	1.196262182	3.618148142	3.53781805
0	0	1.993290515	3.83234479	2.925063979
2.133769986	0.874802684	1.962206066	0.452823559	4.444357287
1.172801259	3.854727598	1.17093161	0.05681699	3.589889095
0	0	0	4.958865224	3.377766308
0.979376349	0	0.979559562	0	4.358267902
0	7.710310609	0	2.173201292	3.159454539
0.704249697	0	2.387704466	3.305881933	6.398824438
1.125210905	8.971784724	0.02827087	0.040547394	2.170801981
1.354790899	5.73856649	2.325866002	3.490809958	3.014284525

hsa-miR-515-3p	hsa-miR-373-3p	hsa-miR-526b-3p	hsa-miR-6715b-3p	hsa-miR-337-5p
0.10542835	0.110229269	0.083662003	1.343899778	1.294504526
0.068170378	0.07104094	0.054905426	1.857489045	0.040658398
0.032755634	1.110947567	0.026764451	5.126488568	0.020129211
0.017012326	1.058163378	0.013991215	2.891972275	0.010598581
0.114151378	2.025232155	0.090273612	3.088802967	1.316117254
0.047732286	0.049652948	0.038763712	3.558315501	0.028959958
0.153851216	0.161548618	0.11980655	1.489779336	0.085748792
1.070171034	0.021150615	0.016738561	2.418206823	2.399284984
0	0.927765166	0	4.054563792	0
0.044915191	0.046710944	0.036517823	0.046382153	0.02731597
0	0	0	0	0
0.21620895	0.228292074	0.164439167	0.226050191	2.1858457
0.104816951	0.10958411	0.083196901	3.480127091	0.060683722
0	0	0	1.975792755	1.979821179
0.183179973	0.19284738	0.141057648	3.067431605	0.099888443
0.040295868	0.04188995	0.032823981	5.164077883	0.024603259
0	1.386396535	0	0.566788375	2.212071088
1.318354956	0.367108173	0.35961489	1.894765725	1.84689268
0.076556112	0.079838697	0.061452083	1.889005302	0.045345462
0.235133388	0.248695628	0.177582367	0.246175455	0.12353654
0.168940608	0.1776311	0.130798723	5.005014765	0.09309878
0.278601256	1.837765157	0.207100173	2.554165392	0.142082925
1.696210753	1.091246634	0.021988919	5.871111461	0.016581934
1.45426844	2.119448771	2.077977125	1.452419142	0.079479639
0.13990857	0.146726717	0.109537047	0.145469317	0.078810141
0	0.697617474	0	2.962200538	0.714839801
0	0	0	1.972117754	0
0.058854954	0.061283028	0.047581209	1.196881729	0.035375581
0.081318711	0.084841094	0.065150748	2.346737464	0.047979148
0.037850872	0.039339792	0.030863206	1.128147589	0.023158802
0	0.500449068	0	1.281348809	0.985220244
0	0	0	2.321512083	0.999731004
0.542052588	0	0	0	0
0	0	0	1.28692664	0.777483133
0.092245463	0.096333899	0.07358389	1.946933361	0.053945874
1.802789949	0.280874915	0.197860489	0.277889715	0.136329505
0.121388335	0.127095246	0.095724859	4.017991358	2.9850293
0	4.280745222	0	2.058262163	1.383181619
0.079692985	0.08313304	0.063889773	3.177234754	0.047082411
0.020555873	0.021332107	0.016880717	2.909168591	0.012766526
0.095683434	0.099954559	0.076222247	3.244360645	0.055801818
0.062793993	0.065407221	0.050684954	2.272247071	0.037619333
0.120961091	0.126643127	0.095403887	3.545435572	0.069144261
0.056642258	1.189245414	0.045833402	2.24697412	1.166369977
0	0	0	4.219376783	1.516428658
1.492185254	0	0	0.924868694	0.931472741
0.155160663	2.968196216	0.120765433	2.167062432	0.086393095
0.18315729	1.573816577	0.141041392	2.259524406	0.099877737
0.108690452	0.113672675	0.086139783	4.420451461	0.062733076
0	0	0	0	3.29219501
0.002529518	0.002620901	0.002092885	3.472769826	1.007881728
0	0	0	2.761843359	0.677726402
0.051386323	0.053471174	0.041669205	3.0531097	0.031080797
0.020182872	0.020944336	0.01657696	3.101416957	0.012538954
0.013564648	0.014068265	0.011171746	4.320285836	0.008476346
7.8799187	9.921256049	6.085619257	5.040438159	3.293329862

0	0	0	2.344818958	2.261691631
0	0	0	2.794279909	0
0	0	0	2.844739943	0
0	2.782295357	0	2.290555744	0.689068578
0.033488915	0.034792938	0.027355377	0.034554381	0.020566842
0	0	0	0	1.808207444
0	0.686232915	0	3.554612507	0
0.008680622	0.008999062	0.007163823	3.044240599	1.026862183
0	0.974326932	0	6.900923809	0
0.062919622	0.065538803	0.050783781	1.21000049	0.037690654
0.058386412	0.060792653	0.047211368	4.380581296	0.035107716
0	0	0	0.985578272	1.983972876
2.782218499	1.97708721	1.978384658	4.496325408	0.984455376
0	0	0	3.775821317	1.091492742
0	0	0	0	1.511122038
0.053833316	0.05602949	0.043610121	5.133313678	0.032493817
0.190725073	2.745855889	0.146449464	3.093640359	0.103430354
4.332122972	1.592996426	1.006473751	1.593009443	0.001201567
0.338049738	0.360890132	0.246032737	7.286951989	0.165831594
0.008938291	0.009266393	0.007375678	3.954431887	0.005608272
0.301549737	0.320858424	0.222320296	5.295440903	0.151460446
0.013970679	0.014489885	0.011504211	0.014395036	0.008726947
0	0	0	3.18618983	0
0.087085414	0.090903775	0.069610515	0.090202448	0.051141155
0	10.00584791	0	2.700363357	0
0	0	0	5.826071604	0.867150951
1.153370892	0.047027453	0.036759742	0.046696207	0.027493249
0	1.975664872	0	4.141219985	0
0	0.869577975	0	4.179818125	0.879797519
0.065855974	0.068615107	0.053090846	2.889615457	0.039353447
0.218187025	0.230421501	0.165821472	0.228151174	0.116008995
0.142701978	0.14969334	0.111603316	4.457248096	1.384357419
0	0	0	4.203759602	0
0	0.744784156	0	2.910789844	0.760773249
0	0.95671928	0	2.939247394	0
0.029773375	0.030922645	0.024357493	6.065197873	1.090012946
0	1.544847924	0	0.967235981	1.550297444
1.004910788	0.001460388	0.001166833	1.590925527	0.000890391
0.065482131	0.068223358	0.052797423	0.067720689	0.039142194
0	0	0	5.0207525	0
0	0	0	1.313935766	0.797808336
0	0.965948505	0	1.543090383	0
0.066791253	0.069595296	0.053824545	0.069081057	0.03988139
0.349333524	0.373320147	0.253243795	2.755760045	0.170146243
0	0	0	4.891398482	0
0	0	0	2.204281555	0
0	0	0	3.778511455	0.873433772
0	0	0.415296165	0	0
0.024972588	0.025925635	0.020470413	4.300891591	0.015450016
0.004561068	1.01571225	0.003770561	0.004696449	0.002872956
0.027012347	0.028048252	0.022123831	2.715241617	1.081912422
2.281716854	0	1.732922302	3.752741582	1.35961526
6.443887214	2.859534509	5.475036508	2.2385903	1.169984971
0.106709678	1.347025222	0.08463601	4.902511949	0.061686658
0	0	0	0	1.851729402
0	0	0	1.322110066	1.351278653
0	0	0	1.834816527	0
0.910658515	4.24934995	0	2.865696023	0.918927794

0.059255675	0.061702455	0.047897405	1.198177629	0.035604509
0	1.088733112	0	1.977112566	0.627399514
0.112165892	6.032995671	0.08877267	5.178876044	1.311230375
0	0	0	0.469744261	1.591129144
6.300903959	5.331499603	3.316063217	2.930220793	0.044678654
0.034926317	0.036290885	0.028512699	0.036041225	1.105001285
1.079818614	0.024106446	0.01905067	4.028104055	0.014389974
0	0	0	1.351668512	0
0.427287116	2.217371426	0.301596631	0.45374582	0.198445483
0.100641618	1.328326613	0.080014686	4.48068317	0.058460701
1.086181983	1.085414726	0.020577085	2.112831533	0.015529594
0.093293893	0.097437809	0.074389231	0.096676327	0.05451293
0.021995753	0.022829238	0.01805242	2.916115608	1.662369328
0.226753686	0.239652396	0.171784943	1.698059364	0.119836004
3.994034771	1.057320068	3.076454088	0.017275783	0.010445631
1.961428956	0.971210431	0	0	0
0.079672639	0.083111667	0.063873982	9.136412457	0.047071173
1.488585217	0	1.132085263	1.781628636	1.165310863
0	0	0	2.297652136	0
0.093363973	0.097511605	0.074443039	0.096749436	0.0545508
0.142800054	0.149797525	0.111675782	2.922299268	1.384585508
0	0	0	4.177653857	1.717276019
0	0	0	3.391575782	1.236086143
0	0.580697232	0	1.405071789	1.467330662
0.163612067	0.171946926	0.126931367	4.428914578	0.090521795
0	0	0	1.449628601	0
0.111058056	0.116173114	0.087934183	0.115231899	0.063979621
0.074055405	0.077213761	0.059504393	1.245649897	0.043954429
0.019512338	1.066610708	0.01603067	11.51052769	1.059659137
0.01120966	0.011623447	0.009241228	2.640014483	1.034590588
0	0	0	4.894846617	0
0	1.486508243	0	4.870134759	0.926899056
0	0	0	0	0
0.029158652	0.030282561	1.096993575	4.908608328	0.01797449
0	0	0	0.978963957	1.976575946
0.113634315	0.118895104	0.089882958	4.707150977	1.314846433
5.04002832	0.099790074	4.095545878	3.77075444	0.055717746
0.011271483	0.011687615	0.009291956	3.86692549	0.007057641
0	0	0	2.295113557	1.079815702
5.780771084	0	4.372819272	2.570303824	0
5.169960226	2.146474931	4.73446397	2.475105644	0.020193407
4.25914827	6.968898828	3.41946079	0.947370192	0.522096911
0.080021254	1.26393366	0.064144523	2.677709599	2.598865121
0	0.476152844	0	3.49336283	0.493395329
0.087652159	0.091499942	0.070047717	5.89207792	0.051450338
2.214994421	1.1646364	1.160620739	1.784159595	0.02973557
7.494072763	4.274395017	5.667131744	1.016890615	1.015201539
0	0	0	2.183265553	0
1.175478202	2.091039423	0	0.676570954	2.166246567
0	0	0	3.674774522	2.015422362
0	4.539026463	0	1.20536411	1.271627917
2.569013695	3.26332414	2.815862111	10.161913	0.032857701
0	0	0	4.375010005	0
0	0	0	1.971435163	0
7.169844781	1.858495739	4.549779797	1.858340807	0
0	0	0	3.580995575	0
7.177872411	5.291551153	4.982772786	4.370442315	0.023794153
4.765636748	2.041549991	4.171043061	0.805160956	0.819297946

hsa-miR-3117-3p	hsa-miR-7705	hsa-miR-205-5p	hsa-miR-25-5p	hsa-miR-521
2.39504555	2.416479403	5.370833129	0.066209574	0.074752585
1.217445182	1.224356007	2.899633313	0.043969807	0.049356392
0.025890226	2.140275248	1.719006123	0.021690245	0.024205104
0.013547041	2.398877221	0.017852701	1.053540498	0.012688458
1.987723715	2.446889164	2.813709372	0.071250618	0.080549122
0.037464366	4.39159546	0.050268614	0.031254484	0.034966402
0.115114662	1.477786008	0.164048649	0.093452053	0.10625275
0.016203858	2.413709914	4.809852526	0.013623758	0.015170908
3.60026839	1.905500956	0	0	0
1.146050269	3.375490369	0.047286377	2.166369333	0.032956419
1.435913313	3.129313525	0	3.208393085	0
0.15750536	2.768298646	6.786004695	0.126074579	0.144533601
0.080148301	2.748480222	0.111122897	0.065853957	0.07434423
0	2.560078213	4.778272998	1.97913345	0
0.135330457	3.707686872	2.719377724	2.569009607	0.124562443
3.345002655	0.035283445	4.854494567	1.123821496	0.029646382
0.566570452	2.129878663	1.036362761	1.434980913	0.570943914
1.081041382	0.751780989	0	1.114728841	1.092808947
1.242529412	1.250473731	1.25146241	2.271285695	1.236723766
1.66082081	2.821939483	7.250191505	2.253071682	0.155681703
0.12557869	2.964783255	0.180459138	2.098704053	0.115742564
0.197787095	0.229123624	3.028213134	1.69174216	0.180519924
0.021278204	2.115780077	0.028203955	1.083633395	0.019906816
0.106265142	1.442259154	1.448743587	1.393844869	2.053516586
0.10532419	0.119258558	0.148937253	2.457120963	0.097349365
1.597433124	1.585812258	0.69927497	2.589448131	0
2.557608699	2.293900266	3.427316182	0.980631276	0
0.045956536	1.194981307	0.062062386	0.038221433	0.042839183
1.881977265	1.895274799	1.266418936	0.051952901	0.058439296
3.974137096	2.488608934	0.039816501	3.148798562	0.027887212
1.824570412	1.566735229	5.696080353	0.973792694	0
0	2.806941638	1.584600054	1.584639939	0
1.013427724	0.540156489	2.910205058	1.945633746	0
2.46958842	0.759871095	2.627418874	0	0.766923779
1.917968247	1.298533863	1.300460337	0.058474156	0.06588659
0.189081874	3.244303221	1.79659855	0.149819076	0.172774196
0.092134146	0.10398212	2.841652299	0.075387163	0.085316611
0	2.540737391	0	0	0
0.061634793	4.160203746	0.084240019	0.050973962	0.057323487
1.068231701	2.08932896	0.021580127	1.064455299	0.015299277
0.073466226	1.945124616	0.101331674	1.901254434	0.068211183
1.81901598	1.828963175	0.066246409	1.188649165	0.045603403
1.369899277	0.10362794	0.128480926	0.075144092	0.085036192
3.044942542	0.049374978	2.246209504	0.036844511	0.041280977
1.509691874	1.916281184	0.936281337	1.51439348	0
0.926319671	1.494011875	0.92549854	1.50224146	0
4.582372864	2.138442531	1.489669221	0.094164445	0.10708222
0.135315016	3.009224148	0.195974316	0.109107892	0.124548495
1.335588435	0.093421385	0.115281658	0.068101866	0.076926742
0.86648497	0.863913288	0	1.433514022	0
2.010793511	2.011240339	1.008673027	1.594406479	1.008366918
1.844019345	2.474389541	0	1.18480098	0
1.778960287	2.215332766	6.199255687	0.033555866	0.037563853
0.016047612	2.677809572	0.021187627	0.013493171	0.015024971
1.045296085	1.046444946	0.014229048	1.042843553	0.010137753
0	2.00207277	6.000832833	0	4.677119916

1.451820944	1.438212283	3.465887394	0	0
0.867474169	0.864917303	0	0	1.428736403
0	1.598905955	1.594076275	0	0
0	0	0	1.199421939	0
3.469456384	1.112993493	1.112703981	1.704772743	0.024736361
0	1.382950124	0	0	0
1.581094086	2.332974982	0.687945089	1.21819125	0
1.619414182	3.041553387	0.009100669	1.617092104	0.006506481
1.555017778	0	0	0	0
0.049038252	1.829420884	6.182015512	0.040739241	0.04569136
1.18774116	0.050871134	0.061564958	1.787134669	0.042509559
0	2.302599754	0	0	0
0	2.783431432	4.060374947	0	0.983521211
1.058908877	0.574368965	0	0.59181284	0
0	1.9098273	0	2.238858822	0
0.042133314	0.046962446	0.056733986	0.035090112	0.039297207
0.140450253	3.032415613	2.281576252	0.113055492	0.129183815
1.006419241	2.330711447	2.008724411	2.329654296	2.007898104
0.234354199	1.940130881	6.157185237	0.183094437	0.212874622
3.210700816	2.626724249	1.622297628	1.618033879	0.006698585
1.810038877	1.855967677	0.327253575	0.166849725	0.193222358
0.011141053	2.061202819	0.014655653	2.054190121	0.010438689
0	3.326412169	0	0	0
0.067125711	1.282841403	0.092133605	1.255482821	0.062380955
0	1.906053235	9.49367321	2.235351773	0
0.857900334	2.122954533	0	0.86420475	0
3.517616479	1.763801711	0.047607191	0.029663909	0.03317302
0	2.781943043	0	1.565134229	0
0	1.827445872	0	0.877096409	0
0.051257368	1.840087239	0.069501477	0.042548885	0.047743622
0.158814145	0.1822215	0.234428438	1.568892489	2.255616265
1.429123096	4.041309085	5.84027243	0.087336124	0.099143698
1.193704354	1.185060229	1.185741841	0.693765266	0
0	0.742303845	0.746207468	0	0
0	0.954085149	2.340172485	0.521291698	2.558368302
0.023566086	1.704609705	0.031290256	1.692013491	0.022039792
0.96796273	1.957567695	0	1.549244859	0
3.006473308	2.81410812	4.095114206	0.000956158	0.001061161
0.050975172	1.838732617	0.069103935	2.828674412	0.047482717
0	0	0.885922767	0	0
0	1.70819709	0	1.337419247	0
0.966615485	0.965860954	0	2.281360509	0
0.051962941	3.758520623	1.220564187	0.04312367	0.04839587
1.909868869	1.965505002	3.599018977	0.187984519	0.218818401
3.964706063	2.123254332	0	2.144718956	0
0	0.916623607	1.483077404	1.493153396	0
2.809452949	0.861952083	0.863594824	0	0
1.880546149	0.41281207	1.64665835	1.487353319	0
0.019811009	1.084786509	0.026230308	0.01663437	0.018538205
2.341976453	0.004033383	4.725455354	1.014542454	0.003427128
1.689848458	2.708084941	4.728414336	0.017965162	0.020028367
2.521945135	2.509534868	4.119811396	1.353628823	0.801420333
0.641046601	2.887200102	0.64283574	0.653053238	3.782227951
4.125932743	3.025699069	2.784637774	0.066953872	0.075607502
1.57807678	1.070838635	0.766002755	1.392656243	0
2.511278943	1.717191653	0	3.180833605	0
0.671404786	1.170655011	0	0.682883251	0
0.912920121	1.477365304	0.912028928	1.89587236	0

1.190399587	3.435918835	0.062487854	0.038470319	0.043120956
1.467909458	1.747901588	0	1.119365058	0
0.085485415	2.000732235	3.686598405	2.379310699	0.079234587
0.467539146	0.899232303	0	1.285807977	0
3.309956624	2.639028731	6.082472961	3.085600348	4.47101679
1.114526218	0.030627003	0.036727628	1.107909845	0.025776432
0.018438942	0.020430909	2.431074227	2.413000458	0.017257794
0.544043842	0.539442835	0	0.557199931	0
0.286234962	2.133174647	2.212470391	0.220202081	0.258295582
0.077100399	1.962026975	4.288769338	0.063417295	0.071548183
5.24245978	1.686341389	0.026368825	1.675797634	0.018634376
1.291594112	2.976315425	6.218421697	0.059094595	0.066596425
2.680926124	1.674081087	2.425437713	0.014684793	0.01635704
0.164457666	1.672625722	1.692870158	0.131341965	0.150771142
0.013350203	2.0731848	0.017590713	2.388347351	3.532603553
0	0.971142119	0	0	0
2.905758595	0.069017105	1.899008618	2.281141145	0.05730951
0	1.7930022	0.639168216	0.649366695	0.641422835
0.983051438	0.982658764	6.219712059	0	0
1.291796795	5.163123702	1.3039239	0.059136035	0.066643843
1.429385856	1.446681917	1.453351674	2.031851075	0.0992066
0	3.484320196	0	1.40547774	0
0	1.20087111	4.276185073	1.227686149	0
0.579716461	2.643895305	0	0.592541755	0
0.121898957	2.947822447	7.233627111	1.44714123	0.112408472
3.018410477	1.855939274	1.450468588	1.870562851	0
0.084683086	1.354826373	0.117825482	0.069469933	0.078499884
1.857589303	1.242717018	0.078229393	1.836350566	0.053438383
0.015519396	1.664227893	0.020482125	2.075046696	0.014531554
0.008951015	2.373021388	0.011755515	1.035492515	0.008389451
2.177304465	1.607755179	0.71432042	1.251330363	0
0	4.203440402	0	1.496679991	0
0	0.964887751	1.542155254	2.280193033	0
0.023086209	5.227157109	4.909776054	2.110530934	0.021592525
0	0.978974002	0.979141424	0	0
1.349491087	0.09754163	6.782158733	0.070953496	0.080207062
0.073351518	1.944589844	2.401345122	2.658746343	3.829099829
0.009000117	3.053750814	0.011820433	0.007587311	0.00843541
2.341696762	1.927754184	0.573291846	0	0
1.131465475	2.430291657	2.02122162	0	3.153645407
0.025973888	0.028834175	0.034549791	1.101749558	1.708279803
5.701896426	2.629252807	3.194386927	2.43269727	2.05527339
1.252795592	1.261177649	1.262351646	1.236371568	0.057548956
2.326505177	2.139108642	0.913169233	0	0
1.275202841	1.284570368	0.092739334	0.055745093	0.062766973
1.158995773	0.042859453	2.214769393	1.149440755	0.035915733
1.01645726	1.01685779	2.022355999	1.015591068	4.976097739
1.469640422	1.466115188	0	0	0
0.678062333	0.673690708	3.263450865	0.689413722	0.681966688
1.284416065	1.971030008	4.48380826	0	0
0	0.873243667	0.880774118	0	0
2.216981097	1.798366102	2.237217786	1.164989249	2.210067271
0.948216748	1.931249544	0	1.938559609	0
0	0	0	0.980157136	0
0	2.437957626	2.431656683	1.87625213	5.105935502
0.701526636	2.142627634	0.702168188	0.712397318	0.705279886
1.127209387	1.73990792	0.040951571	0.025656022	4.624023626
1.351339166	2.049988913	0	0	2.066759911

hsa-miR-6516-5p	hsa-miR-5589-5p	hsa-miR-493-5p	hsa-miR-92b-3p	hsa-miR-371a-5p
2.976188789	2.439395782	4.902525814	7.341462685	0.102750057
1.211101059	1.225035468	1.854725393	5.517902807	0.066560342
0.0238165	2.474362321	5.384177596	6.284594797	1.719731526
0.012490009	5.484890532	4.413032337	5.485146844	0.016652493
0.079095978	1.367752626	6.933684916	6.707626577	0.111203888
2.776890406	6.108366099	3.036306556	2.809479064	0.046651893
2.521123394	6.000720159	3.474493288	4.008930581	0.149587546
1.065958956	5.838629727	5.468240697	6.68147919	0.019946885
0	5.307641853	5.553186964	6.289535218	0
1.749880749	0.047255713	2.19659502	5.445343993	0.043904659
0	0	1.945120632	9.395773179	0
3.018755049	5.466453039	5.906332813	6.981344907	0.209578462
1.314214887	3.952366643	4.591375863	5.153776365	0.102157255
2.976789574	0	5.901199449	6.462223219	0
2.16218807	0.195830489	3.347774529	6.742764338	0.177848521
1.128002672	1.135128472	4.457060709	4.207630961	0.039398239
0.572112232	0	6.679846795	7.547981022	0.562308667
0	3.571729389	6.734069599	6.289105992	0
1.855601754	4.702800588	5.292658171	6.519278189	0.074717191
1.633763501	7.879812171	3.967756434	5.003621888	0.227712712
0.113515154	4.665499681	3.732296714	4.667022187	0.16413767
1.726899646	3.907318342	3.025493255	3.02591175	0.269239642
2.106703287	5.001217681	1.69482463	3.723355145	0.026265198
0.096374737	5.707832308	2.570070778	5.36336283	0.137411817
1.407012402	5.044610428	4.553377032	6.020154921	1.449867041
2.572036531	11.39760122	5.083099633	3.804702729	0
2.296101218	0	3.667972615	5.580777968	0
1.18379873	5.208787985	4.46809105	5.209565583	0.057491289
0.057430068	1.905185794	4.571613267	5.731481614	0.079346806
0.027435813	7.505755712	3.186249828	5.199998427	0.037012172
2.677708641	0.945924872	6.447530124	7.073194616	0
0.999715956	6.08698059	3.584503411	5.727437884	0
0	0	4.96650342	7.159906521	0
1.303594609	5.318002434	5.01691907	6.861509108	0
1.279589034	4.026606676	4.912742073	5.918976744	0.089960274
0.169122614	3.625688789	0.289944161	4.24590119	0.2561205
1.358724876	0.128842609	9.504413506	6.045660496	0.11821203
1.371050849	0.817995895	9.066900122	2.313815794	0
1.865663292	6.829402049	2.338097137	3.844129704	0.077766717
1.662780673	4.015042215	4.968809244	6.360312781	0.020117567
3.951744782	0.101258185	3.443116557	5.329604979	0.093297321
2.565564768	3.628639657	4.710745028	4.565241927	0.061327163
1.991676615	3.34788423	4.265842817	5.334638005	0.117798433
1.177245557	4.610281017	6.581204734	6.803769525	0.055335892
1.511862198	2.712064768	10.25632145	6.537609388	0
0.927901631	3.582214879	4.049605868	4.831598596	0
0.10505292	4.879824872	4.402816348	5.851782901	0.150851045
2.162129645	4.11881987	3.066627679	4.618983922	0.177826695
0.075548605	7.566221547	3.297905965	4.519194674	0.105912334
2.148599681	2.12916888	9.956612212	6.19810266	0
1.008326278	6.163870187	5.657977011	6.336114315	0.00247779
0.66536256	1.525875521	3.733732046	7.311736548	0
2.524520129	6.126302838	4.066952693	6.335779393	1.173803253
0.014788712	6.109848651	6.358363474	5.601630175	0.019752883
2.054950038	5.24390757	3.771890121	4.828562369	0.013280002
2.023102513	3.791838336	10.1907661	4.422478525	8.34400132

1.464663228	4.971653385	5.93613675	6.823462133	0.593286274
1.429592216	4.363966688	3.111850012	2.387772323	0
0.712385286	5.158742158	1.893914381	9.428399341	0
2.981835089	0	8.514941932	3.525948334	0
1.107054737	6.223490541	4.344816367	5.084570611	0.032753917
0	5.788057964	4.768911212	6.274938826	0
0.691910551	5.227832609	4.734429512	6.610301076	0
1.618333169	4.570731233	4.294833755	5.047659609	0.008500517
2.290053374	2.285959589	5.128270608	8.270588089	0
3.056904583	6.494334332	4.964524333	7.435361631	0.061449478
1.795861547	6.989704512	4.200503296	5.587305292	0.057034919
1.982728742	4.225106332	6.43588927	5.106021426	0
2.300696317	5.181746761	3.879965903	5.43157738	0
1.980363279	4.758075589	5.764652611	5.992753906	0
0	3.476870699	4.794305594	5.55868836	0
0.038645128	6.835636565	3.260263808	3.063970188	0.052599012
0.126639296	4.417493686	4.28938168	4.748969067	0.185105865
0.00141041	3.331922423	3.469491569	7.385760126	0.001864217
0.208102356	4.296525871	3.564564663	3.564875582	0.325751196
1.029242056	0.009365511	5.093490551	5.178433453	0.008752728
0.189011241	0.326910144	4.667481465	5.940161493	0.291092941
2.380135624	4.322820422	2.653108403	4.397042385	0.013677237
2.099475471	2.075738255	3.719580514	4.41404693	0
1.889092259	8.471058129	4.676251283	5.522095598	0.084949588
1.502922094	1.903618584	3.34763559	7.756862943	8.981604104
1.418137146	4.086105413	5.688248129	6.800494332	0
1.750931964	3.813012745	6.824787962	6.305241569	0.044200316
0.982581553	4.614685276	6.05770702	5.69825448	0
0	2.136203784	6.507817221	5.104354478	0
1.204360677	2.889682519	3.314337869	4.654146062	0.064307925
1.595818141	7.150641417	5.117599146	5.913130785	0.211475504
1.414166866	3.632999397	2.920658754	5.528312407	0.138822982
1.585666116	7.025315282	3.459866635	5.791380483	0
2.645027046	7.47887309	2.763970749	4.15083487	0
0.513578184	0	0.962306647	5.432395556	0.504211857
2.443050875	3.31966138	3.472836993	5.444948443	0.029125265
0	2.537678805	5.75386287	6.438138058	0
1.004644909	2.328713758	6.347771248	8.475561963	0.001380857
0.046681036	2.282426825	4.233546499	4.652401675	0.063944046
0	3.278962118	2.417087305	3.902022381	0
3.021313018	0	5.626930423	6.406560864	0
0	7.153565462	4.337802073	5.403878326	0
1.8238111	3.318897668	4.797070692	5.294727992	0.065218215
0.213871988	5.171858166	5.777707492	5.549883648	0.336440888
1.418387776	9.28445044	2.373686898	1.806114531	0
1.489912587	3.569241003	4.114069751	6.629839249	0
0.867246394	2.126341793	5.769043876	6.917514718	0
1.465071528	3.23656618	6.225511577	6.592698184	0
1.080469025	2.705605456	4.777194332	8.575170568	0.024434774
0.003374677	5.025132683	3.831770061	6.697971258	1.015884167
0.019709823	2.448391951	7.01719923	6.992256996	0.026427942
2.056081871	4.563930877	4.86423132	6.444371287	0
1.149306755	6.752378207	5.619720461	6.625768448	1.787793024
0.07425646	7.454886438	4.908039622	4.836817646	0.103992295
0.774627107	2.052585625	6.740406661	5.740055089	0
0	4.730684351	6.295281645	6.930940469	0
0.676386692	4.23234536	5.903517384	7.374540198	0
0	5.875732939	2.865965272	4.02740144	0.910473558

2.552971309	2.257010139	4.887915123	8.543218512	1.199473394
2.02826031	3.703980568	6.98365193	6.578843006	0
0.077808765	0.118926949	2.463143032	4.533912408	2.01652966
1.997850615	2.52994903	5.214373742	5.838879342	0
1.231850806	6.611708182	2.657139219	6.02707979	0.073550347
0.025361148	2.75186231	6.1412813	7.106697551	0.034157348
1.672581666	4.767913665	4.982756009	4.292784724	0.022725642
1.385138624	2.679847474	3.703451914	5.344129661	0
0.252141828	5.288773535	5.936608447	3.454773416	0.409973001
0.070279777	3.035262101	3.46383677	4.735797766	0.098107973
0.018338938	5.264027048	4.302413001	3.296749957	0.024563276
1.908469558	6.321607394	5.368343167	5.672545258	0.090978046
1.668078462	4.203632959	5.07275016	9.349453639	0.021525213
0.147693202	5.224076952	5.570259584	6.120576306	0.219687018
1.648732456	7.95626556	3.408239201	5.135487864	0.016408793
2.286152549	4.202106086	2.543455393	4.477375794	0
0.056322067	0.084159284	4.08889264	5.759708901	0.077746941
1.823010162	4.675389111	6.261761234	5.516031296	0
0.983440327	6.016216958	5.059469785	6.713096266	0
0.065473778	1.304043792	1.947430617	3.004773094	0.091046073
1.414417457	3.965664402	5.802377903	5.578046475	0.138917725
1.388818918	3.768155794	7.56526897	5.480502969	0
0	2.955227355	4.888656781	6.374562688	0
2.378946221	0	6.258361218	6.816090932	0
0.110257911	4.316165438	4.317062916	4.914501463	0.159002009
0	4.94870008	6.276178594	7.250809377	0
2.395440385	3.837867946	2.800371526	8.054239578	0.108206854
1.228114637	5.072482169	1.87668318	5.868547901	0.072285443
1.063198583	5.629506247	5.532438746	6.013495129	0.019097267
0.008259544	3.518027702	6.365895656	7.375192388	0.010975709
2.595719994	3.327744128	4.924816203	9.06030385	0
0.923108271	4.330068016	4.041520231	4.263064796	0
0	2.755563345	3.116764993	4.031884253	0
0.02124789	4.475464416	1.704056088	4.401360743	0.028524789
1.974765297	5.63832245	3.873906816	4.773552708	0
0.078761039	5.301993945	4.712479771	5.680722479	0.110702984
0.06690659	5.641131715	4.267139227	5.642740691	0.093145788
2.3690854	3.867161736	2.863684138	4.147871559	0.011036209
1.965526058	2.728813446	6.75747507	6.087748286	0
0	4.211100359	3.942301646	6.413450991	0
2.455010704	6.944535582	1.109954661	7.03516668	0.03214287
0.965995034	0	6.893710868	7.811653425	4.416883824
0.056556904	2.340741097	5.572963415	6.382965596	0.078085792
0.478573672	5.045518886	5.78771755	6.902015442	0
1.890872342	3.738853342	4.333669088	4.89226924	0.085500053
0.035324086	4.055787196	4.89806634	6.23539697	0.047952273
1.016065222	1.016754033	5.196993623	6.177062186	1.605616424
0.905888249	3.843374229	4.163642784	5.36168192	0
0.682993544	4.294957759	5.534760619	5.900522393	0
1.291530706	6.213447791	5.926444255	6.84335344	0
0.893427677	5.821828951	2.912195377	5.478436279	0.445598437
0.039083823	6.090584614	2.569098882	5.281954063	1.183874695
0.949357923	5.668426951	7.420353727	6.082947924	0
1.974334244	0	1.971666148	4.136072881	0
0.899019416	4.078363094	5.31799625	5.112021152	0
0	0.702066496	4.433765368	9.616422701	0
1.123805374	5.961244144	2.995855611	4.200900495	0.038060544
2.735506125	2.698517011	5.449715482	7.157303421	0

hsa-miR-3680-3p	hsa-miR-577	hsa-miR-34b-5p	hsa-miR-203b-3p	hsa-miR-6788-3p
1.303148648	0.096480901	0.07616539	1.344176598	0.08141396
0.043691241	0.062767426	0.050240987	3.651494444	1.838530593
0.021559368	4.620526232	1.708750926	0.033700291	0.026124681
0.011334009	0.015797387	0.01289771	0.017485051	1.652032689
0.070773442	4.409055553	0.082089312	4.787273298	0.087817261
1.145246234	0.044097796	0.035573551	1.16082454	0.037812601
0.092799254	0.13969094	1.448889195	0.159545317	0.116366509
1.658995841	0.018915136	0.015422581	0.020952222	2.897921275
1.915613843	1.496259119	0.929894604	2.699756815	0.928840256
2.756885478	4.386435443	0.033526086	0.046247096	0.03562613
0	4.53743119	0.590201567	0	2.369338745
0.125141787	0.194354697	0.147644307	0.225133188	1.618467264
1.301566	1.340967748	0.075748156	0.108347693	2.995788607
0.983216851	0	1.564269137	9.811586682	0.982242594
1.49068187	0.16553653	1.522220878	0.190326083	0.136856749
0.026370385	0.037273514	0.030155003	2.178459266	2.981217516
1.982375747	1.386141484	3.645230668	1.032406449	0.566053882
0.3752835	0.357287015	0	2.804771849	0.759808966
0.048764352	1.8840034	0.056165844	0.078988978	1.243279291
0.134461323	1.708998626	0.159096195	0.245145133	3.382360512
2.097058566	1.528767534	2.573552921	0.175366903	2.59367716
0.155071512	0.247978773	0.18464249	0.291302043	2.434515135
1.083454662	2.118039162	1.687154509	0.027609391	1.68958437
0.085931411	0.128503547	1.416307876	0.146347564	0.107401874
0.085198732	0.127318517	2.053763135	0.144954	2.068113925
1.233143374	0.693328566	0	0	1.596338904
1.562314072	1.972552602	0	0	0.979550112
2.805467537	0.054273642	0.043596151	0.060655261	1.189727786
1.857741933	0.074711879	1.251983213	0.083929004	3.922206136
0.024816382	0.035026107	1.121845593	0.038955382	2.481288358
0.511705714	0.937491883	2.388080765	3.781764623	0.497207498
0	0	0.999713181	5.882166481	4.087056197
0	1.000673688	4.035610861	0.544990587	2.972776717
1.312461825	0	2.666183798	0.761254205	1.687294097
0.058092393	0.084599081	1.910254418	0.095274439	0.071644887
1.662694388	4.158205314	2.373033947	1.802638706	0.191414125
0.074878204	2.483981993	0.086963456	0.125613382	2.011958071
1.378165513	0	1.368781861	0	3.90356457
2.608217021	0.073237796	1.869972654	0.082242367	1.252611501
0.013657216	0.019076599	0.015553156	9.079399505	2.410582788
2.329480376	0.087702673	0.069483266	0.098847495	1.930549752
2.226082015	0.057868428	0.046414105	0.064731403	3.588197943
2.403221045	0.110415385	0.086676716	0.125167766	3.796226266
0.036614617	3.851059655	0.042007912	3.445272548	1.182925146
0.940194383	0.93524418	1.51098904	0	3.081950719
0.929980315	2.698872843	0	0	1.49652486
3.683993422	3.639348538	1.452235329	1.493992651	2.112898282
1.490630822	0.165516634	0.127136723	0.190302231	0.136841092
0.067648593	1.352719444	0.078387032	3.297169602	1.336725303
0	0.863161326	0	2.96019702	0.866222961
1.008066206	0.002354563	0.001933534	0.002597363	1.595109716
1.185526498	0	0.663244657	0	1.164253667
0.033347983	2.549785612	0.038219903	0.052932417	3.025583715
2.077326983	1.069373325	1.066014015	0.020747971	1.664234815
0.009061372	0.012603174	0.010303922	0.01393845	0.010913939
1.325857705	3.229272393	0.777146572	2.244247069	0.774435267

1.106149841	0	1.756365443	0.597088814	0
1.84029163	0	0.869030362	0	2.143176692
0.718850949	0	0	0	0
1.200129976	2.088267335	0	3.149223898	0.67150489
0.022029656	3.970469161	0.025155997	0.034456363	2.137609556
1.400043133	0	0	4.428783769	3.087890238
0.698655102	0.681742133	0.689881833	1.562108569	1.883108885
1.027501737	0.008071914	1.02869243	0.008917006	1.02918005
0.976160191	0.974082959	0	1.553398497	0
2.22649455	0.057983001	1.198103376	1.210180958	1.202160462
0.037693016	0.053845738	0.043260157	0.06017055	4.421207741
0	2.786820846	0	1.567293847	0
0	5.465962726	3.147443038	0	2.299784425
0	2.141891586	0	0	2.355375848
1.509114223	0	0	0	0
3.666141276	1.180917848	0.039986179	0.055461872	1.17425398
0.112237507	0.172146436	1.54055681	2.745238304	1.555587896
1.006072622	1.592950433	1.006329701	0.00195404	0.00153945
0.181612987	1.966147587	0.217982783	1.995146051	1.891398596
0.005992534	0.008311156	0.006807474	2.852233087	3.848835762
0.165531967	0.267440242	1.787938481	0.315778438	2.487571233
1.044016269	1.048225068	0.010609914	0.014356048	3.38570975
1.391424517	3.639478949	0	0	0.829787945
1.254857198	1.286457538	0.063530337	0.089914653	2.334901914
0	5.747710142	0	1.496309418	1.909194513
0	1.406742721	0	0	1.413107184
0.029481701	0.041792656	0.033746711	0.046560142	1.757504526
0.983112212	1.976024193	0	0	1.563691617
0	1.423671057	1.836551974	0	3.138267316
0.042280132	0.060659588	0.048596331	0.067901408	1.211091102
0.126126194	2.798748357	0.148851004	0.227222585	1.623255788
1.396318151	0.129801787	0.101109425	1.457109644	2.515513967
0.69420017	0	0.685352476	0	2.341898809
1.685164841	2.206237933	0	0.743909933	0.745831601
2.72770799	0	8.061563499	2.181680443	0.962892095
1.092276157	0.027588416	0.022411354	0.030626085	1.700599183
0	0.967003941	0.968389765	3.534309008	1.546679244
0.000950662	1.004908232	1.004690537	3.007247262	2.328178442
1.809033087	0.060318961	0.048330286	0.067514308	2.587669296
0.892265782	6.576464894	0	0	0.886656608
0.793979916	0	1.722231519	1.701818402	0
0	0	0	0	2.277752921
0.042850954	1.222576728	1.209584068	0.068869933	0.052461451
0.186452683	0.307521993	0.224115237	0.366986378	0.244322105
0	0.854633912	0	0	0.857834397
1.493365084	5.515976874	1.488797189	0	1.486856889
0	0.861193269	1.827767233	0	2.139128851
1.207300086	0.827360843	1.181395673	0.419870822	1.171237259
1.675103914	0.02315843	3.277913796	0.025679787	0.019987913
1.601900395	1.01584683	0.003482395	7.913896157	2.019480247
0.017857676	1.092418188	0.02036444	7.578895367	2.43737394
1.354110827	1.33132176	0.800655769	1.331020822	1.735043155
0	1.127997598	1.145578561	0	1.513886662
0.066509251	0.09763305	0.077038936	2.784660598	2.401147392
0	0	1.362170558	1.069968615	1.08368922
0	0	0	0	0.790985629
1.199413825	1.167632534	1.184556209	1.168592113	0.670930537
0.917183845	5.255836998	0	0	1.887693455

0.038229438	0.054639555	1.187166888	7.493093046	2.56435922
0	0	1.103624664	0.607835125	1.762878821
4.095048444	6.330700178	0.080745668	0.115999915	0.086363993
0.93389214	7.015291772	0.470813752	1.23442807	0.907963958
2.266643455	4.046868318	3.471894268	1.249997549	1.863080039
0.022950009	0.032335503	1.112726097	6.769387538	1.719807357
1.072466678	1.680370023	0.017545501	0.023878793	0.018603068
0	0	0	1.63238732	1.011481006
0.218325835	0.371664329	0.264903848	0.451240504	2.067169017
0.062999114	0.092172493	0.072891651	0.104003326	0.077879672
0.016620532	3.12051269	0.018946039	1.689005992	1.083221768
0.058708296	1.943978402	1.9135691	0.096363894	1.292541015
2.084040734	1.075511336	0.016629152	5.872816406	2.091757085
0.130361586	2.369079322	0.15405074	1.698466732	2.307462756
0.011170167	1.057753691	0.012710587	0.017228675	2.394616836
0.973255673	0	0	0	1.963950827
2.608152156	0.073219343	0.058354254	0.082221261	0.062222976
0.649836919	2.031811071	0.640328115	0	2.056013341
0	2.298194815	0	2.297651171	0.983012493
0.058749432	1.305895011	1.286346579	0.096436722	0.072478092
0.086786695	2.896675093	1.420370944	0.147977812	1.430970014
0.560155322	0.541184239	0	0.546697061	2.740965811
1.228358461	1.575569433	0	0	0.694251149
0	0	0	0.579325655	1.717109856
0.098036592	6.006363659	2.120126017	3.884310276	1.486154035
0.897411555	1.450097061	0.893562198	1.449548627	0
1.944334092	0.101539462	1.335576285	1.361493494	1.97974342
0.047257972	0.068119984	2.609909444	0.076396339	1.858664134
0.01297438	1.06709922	0.014772255	1.06683987	1.661661786
1.626202492	1.0387679	0.008526385	8.826074578	2.370925507
2.206382415	0	0.71658127	0	2.175922261
0.925314151	0.919264935	0	0	2.890517296
0.967441348	0	0	1.953024712	1.543920828
2.699403344	0.027021568	0.021956168	0.029992558	0.023293928
0	0	0	0	4.058578881
1.951555847	2.014603341	0.081739665	0.117529821	0.087439038
0.060017255	0.087561793	0.069375641	0.098685184	1.930042242
0.007542999	1.038980053	0.00857311	0.011580367	1.037805697
0	0	0	0.569675881	2.155756482
1.152784111	0.628870954	1.136885011	0	0.633821133
1.101528723	0.030435801	0.024694535	0.033811135	0.026209144
0.515744091	4.025580015	3.11246491	5.071942009	0.501265129
1.235800813	0.073535508	0.058598597	8.98401776	2.908641934
0	0.903804271	0.476148224	4.187716027	0
0.055383166	0.08044708	0.063924399	2.708740441	1.276082189
1.14910338	1.781559012	0.036540694	0.050534652	1.15944582
0.003289969	2.608468277	4.022116285	1.016905964	2.020882686
0.908539793	0.901313197	1.878670629	0	0.903681241
1.207710043	0	0.680933488	2.090647844	0.677592322
0	0.749035229	1.68018628	0	1.981994694
2.336475639	6.709503281	1.231890325	0	0.448055001
0.035264575	3.841564729	0.040441441	1.182806761	2.217905296
0.950850453	0.946719253	1.524217973	0	1.522953783
0.98019575	5.177203679	0	0	1.560014546
0	0.894165205	0	0.894966077	2.183652536
1.23677439	2.534524512	2.886875904	1.207315498	2.156923337
0.025499829	1.742443007	0.029151312	0.040063718	1.12755697
0.815794134	2.51772863	1.75244204	0	1.350739106

hsa-miR-892a	hsa-miR-524-3p	hsa-miR-376b-5p	hsa-miR-517b-3p	hsa-miR-517a-3p
0.072719564	0.071481851	2.412001939	0.111861865	0.111861619
0.048080373	0.047301738	1.223044312	0.072012585	0.072012439
0.023612086	0.023249394	2.139032357	0.034458054	0.034457989
0.012385554	0.012200102	0.01467317	1.057794063	1.057794131
0.078334019	0.076986157	2.441989349	0.121242524	0.121242253
0.034089389	0.03355353	0.040768118	0.050301445	0.050301347
0.103189426	0.101329726	2.128641411	0.164182417	0.16418202
0.014806685	0.014583741	1.667098042	3.564752757	3.564752754
0	0	1.906194013	0	0
0.032133391	0.031630425	0.038396285	1.766870689	1.766870853
0	0	0	0.590159038	0.590158634
0.140086716	0.137396212	2.309296352	0.232460546	0.232459916
0.072323907	0.07109388	2.409879979	0.111205101	0.111204857
0	0	1.563318133	0	0
0.120855022	0.118607962	2.662150249	0.196167917	0.196167416
1.127623627	0.02846197	1.134589754	1.135022492	1.135022653
0	0	2.992576186	1.036523768	1.036523284
0	0.774483896	3.236985447	3.238483508	3.238483412
0.053721515	0.0528413	3.329870165	2.663039115	2.663039265
0.150805611	0.1478583	0.189693046	0.253386262	0.253385552
1.477905074	0.110291353	2.174065089	0.180610533	0.180610083
0.174646163	0.171103457	0.221942252	0.301818594	0.301817685
0.019423754	0.019128196	0.023081769	2.447593004	2.447593068
0.095415408	2.043885882	1.438897418	4.229401453	4.22940121
0.09458733	0.092909202	2.527543134	2.567141904	2.567142212
0	0	3.432334096	0.699379548	0.699379234
0	0	0.979210931	4.054517266	4.054517273
0.041746585	0.041079481	1.193885521	0.06210396	0.062103836
0.056900219	0.055961838	0.068725011	0.086035309	0.086035129
0.027198418	0.02677729	0.032429909	0.039841914	0.039841839
0	0	4.045388573	0.946288145	0.946287594
0	0	0	0	0
0	0	2.439350211	3.930579983	3.930580092
0	0	2.241928337	3.074389132	3.074389067
0.064125422	0.063052356	0.07769484	0.097721914	0.097721705
0.167217613	0.163863971	0.211825901	2.511230537	2.511231168
0.082949192	0.081509266	4.409582805	0.129039855	0.129039562
0	0	3.31135068	0	0
0.055817189	0.054898701	0.067385242	0.084299108	0.084298933
0.014931864	0.014706971	0.017709358	0.021593342	0.021593303
0.066379519	0.065263734	1.306916223	0.10140522	0.101405001
0.044433555	0.043719464	0.053378036	1.20774049	1.207740741
0.082677795	0.081243321	0.100981847	0.128579155	0.128578863
0.040231569	0.039590745	1.186900937	3.983677177	3.983677114
0	0	4.154551478	0	0
0	0	0.925031769	2.886572124	2.886572094
0.103990427	0.102113583	0.128190129	0.165605684	0.165605282
0.120841577	0.11859482	1.553412473	0.196143092	0.196142591
0.074825827	0.073547031	0.0910811	0.115367622	0.115367367
0	0	3.968533411	0	0
0.001858658	0.001831508	1.595359664	3.183076781	3.183076783
0	0	2.292879197	0	0
1.771446521	0.036037703	0.043838331	1.791988925	1.791989111
0.01466437	0.014443641	1.068445382	0.02120059	0.021200551
0.009897155	0.009749816	0.0117125	0.014237613	0.014237588
0	5.144182587	4.350355944	9.880409496	9.880409926

0	0	3.430709936	0	0
0	0	0	1.41949069	1.419490537
0	0	0	0	0
0	0	3.386214852	0	0
0.024129614	0.023758544	0.028732524	2.477606288	2.477606366
0	0	2.352601917	0	0
0	0	1.571480116	0.688053019	0.688052695
0.006353359	0.006259558	1.620398372	0.009106081	0.009106065
0	0	0	0	0
0.044519041	0.043803446	2.257721848	0.066424788	0.066424655
0.041426119	0.040764586	0.049700284	1.818877033	1.818877241
0	0	2.979946821	2.978476407	2.978476402
0	0.983717299	0	4.294656974	4.294656983
0	0	1.050982953	0	0
0	0	0.931020372	0.931458943	0.931458865
0.038302414	0.037694831	0.045890406	0.056771561	0.056771449
0.125308676	0.122960923	1.573433939	0.204433055	0.204432525
0.00139892	0.001378507	2.59385545	4.469857748	4.469857744
0.205618035	0.201253347	2.616803084	0.36890278	0.368901563
0.006540872	0.00644426	1.030574939	1.030514417	1.030514453
0.186816748	0.182957471	1.846387439	0.32759752	0.327596497
0.010190777	0.010038963	0.012061516	0.014664484	0.014664457
0	0	0	0	0
0.060725047	0.059715794	1.281058054	2.70619136	2.706191522
2.493326945	0	0.928163819	0	0
0	0	0	0	0
0.032344195	0.031837697	2.189081745	0.047638104	0.047638012
0	0	0	0	0
0	0	1.828627043	0	0
0.04651341	0.045762616	2.59916638	0.069548769	0.069548629
0.141217115	2.67981012	1.644507699	3.471011057	3.471011066
0.096321576	0.094607217	1.442995257	1.452877421	1.452877977
0	0	1.186640382	0	0
0	0	0	0.746297618	0.746297347
0.514237573	0	0	1.300841027	1.300840493
2.117378476	0.021173688	1.100235598	0.031309848	0.03130979
0	0	2.540278913	0	0
0.001036506	0.001021395	2.006222625	3.007253968	3.00725397
0.046259889	0.045513585	1.214670206	0.069150918	0.069150779
0	0	0	0	0
0	0	1.709982359	0	0
0	0	0.965995904	0	0
0.047147165	0.046385131	1.841325915	0.070544265	0.070544122
0.211297818	0.206776607	1.953749022	0.381748385	0.381747104
0	0	0.855880742	0	0
8.615742497	0	0	0	0
0	0	2.13395183	0	0
0	0	2.035724717	0.424497276	0.424496773
0.018089735	0.017815308	1.685100244	0.026246544	0.026246496
0.003347055	0.003297989	1.015654819	0.004782314	0.004782306
0.019542223	0.019244783	2.706803016	3.306214426	3.306214438
0	0.803332079	2.512074727	3.256466193	3.256466162
0	3.559333452	1.801935259	8.845898492	8.845899121
0.073547841	0.072294007	0.089475905	1.344816495	1.344816916
0	0	4.15827147	0	0
0	0	2.283877004	0	0
0	0	1.548891092	0.672700828	0.672700491
0	0	0	2.455745707	2.455745647

0.04202052	0.041348647	0.050426415	0.062529751	0.062529626
0	0	2.206685486	0	0
2.397005415	0.075738366	0.093892854	2.805927323	2.805927507
0	0	1.955997134	0	0
0.052917734	2.605607212	0.063804563	8.145779932	8.145779426
0.02514272	0.024755195	1.117126905	0.036750909	0.03675084
0.016841504	0.016586738	1.678264474	3.116391871	3.116391892
0	0	0	0.548215109	0.548214676
3.719682665	0.243343455	0.326524083	2.966378458	2.96637903
0.069614348	0.068436679	2.39531699	0.10672251	0.106722277
0.018183482	0.017907574	0.021596287	0.026385151	0.026385103
0.06481379	0.063727713	1.299761257	0.098844843	0.098844631
0.015963281	0.015722284	2.908566032	0.02310979	0.023109747
0.146085544	0.143252161	2.787616328	0.244108517	0.244107843
2.067329266	1.054013772	0.014459414	6.045891144	6.045891065
0	0	0	2.764835091	2.764835077
0.055803622	0.054885383	0.067368466	0.084277384	0.084277209
0	0.644195033	1.499194213	3.617713445	3.617713468
0	0	2.56138682	0	0
0.064859772	1.280014313	0.078609614	1.949107068	1.949107385
0.096382366	0.094666734	2.536881499	0.152188421	0.15218806
0	0	1.00769545	0	0
0	0	0.691114543	0	0
0	0	1.413771214	0	0
0.109132261	0.107144411	2.602609492	0.174802453	0.174802022
0	0	2.173107213	1.450509673	1.450509549
0.076349543	0.075040847	0.092997225	0.117913772	0.117913509
0.052045208	0.051195399	1.241263287	0.078283596	0.078283435
0.014183187	0.013969937	1.066205338	0.020494635	0.020494598
0.008191149	0.008069692	2.636650071	1.038215568	1.038215612
0	0	1.227125855	3.110823876	3.110823806
0	0	0.920068078	0.920611918	0.920611828
0	0	0.965026314	0	0
0.021066578	0.02074483	0.025051846	4.32291378	4.322913727
0	0	0	0.979149441	0.979149416
0.078002799	0.076661492	2.44022662	0.120685938	0.120685668
0.066277343	2.939188874	0.080377095	7.967641233	7.967640594
0.008236001	0.008113866	0.009739626	0.011827508	0.011827487
0	0	1.689069229	0	0
0	2.641073878	0.630494759	7.828575583	7.82857618
1.104811053	1.706205065	0.028201249	7.542991058	7.542990853
1.853175126	1.856934847	2.019045323	5.85470806	5.854708607
0.056036045	0.055113544	2.921951184	0.084649631	0.084649454
0	0	3.009996293	0	0
0.061099536	0.060083293	2.349190718	0.092805507	0.092805311
0.035013073	1.153289187	0.041889563	5.255132733	5.255132555
0.003588889	5.343262675	0.004235546	10.11746467	10.11614046
0	0	1.466791801	0	0
0	0.684574396	2.95619873	4.754135469	4.754135692
0	0	1.666588485	0	0
0	0	0.444789079	1.70390195	1.703901424
2.207532363	1.16929593	0.046419709	6.19046677	6.190466497
0.949482822	0	0.947281375	0	0
0	0	0	0	0
0	4.446049209	0.89516085	8.586240357	8.586240553
0	0	0	2.68758877	2.68758862
0.027951606	4.330486401	0.033338871	9.326663087	9.324065754
0	2.548025547	0	6.267262148	6.267262411

hsa-miR-5589-3p	hsa-miR-410-3p	hsa-miR-520d-5p	hsa-miR-301b-5p	hsa-miR-412-5p
2.779981186	6.769234751	0.079880877	0.077434625	5.559709596
0.07137001	2.626593225	0.05255896	1.833628766	3.127630151
2.145904456	4.928438368	0.025686067	0.024982285	2.741582377
5.340092693	4.615102279	0.013443189	2.39412741	4.090243722
2.024799636	5.68352276	0.086143149	0.083473584	3.850770933
4.243133727	3.558625061	0.037161265	1.153066451	3.698129186
6.495396974	5.031000126	0.114028377	0.110310609	2.962276694
5.502377164	6.853353705	0.016078872	1.066954319	5.064241092
4.740519392	6.183852271	0	1.501028749	4.881835032
0.046916317	3.927962594	0.035015357	5.919092726	2.527906998
0	1.708104373	0	1.441326981	1.064655061
4.235481218	6.241921004	0.155906595	1.605323018	5.189464893
4.964945217	5.211135992	0.079440077	0.077009376	3.811973785
0.981942602	7.54764594	0	0	5.901219221
3.348515269	3.346553074	0.134007071	0.129485161	1.56793304
2.508220367	5.019053616	0.031484062	0.030610408	3.523752822
0	7.855028257	0	3.841012585	5.993169346
3.714119025	7.428553024	0	0.363481337	6.453710146
4.904896282	5.709988288	0.058791222	1.23948804	4.550013145
6.054521687	4.303965252	0.168179667	1.649197902	3.527780535
1.531868495	6.304152044	0.124371352	0.120242753	3.296865935
4.444750691	3.906403764	0.195648045	0.188375103	3.383799833
4.389182776	4.389562623	0.021112155	1.087684518	1.694988018
4.345776749	4.100696236	0.105278405	3.100568766	2.115879768
5.353504883	5.241696527	0.104347885	0.101003891	4.820686016
9.416750928	6.048474777	0	2.375204192	2.12603291
0.979208525	4.054527589	0	0.979789484	3.55265791
4.690890794	6.188795939	0.045577855	0.044274582	2.85878395
1.905989558	5.657566369	0.062309062	1.253363206	5.618339219
7.486465124	4.657472751	0.029608612	1.122433376	4.783500737
2.319841697	9.474471139	0	0.499986356	5.183806576
5.584483381	5.284921641	0	0.999711969	4.321456472
0.547204149	5.222591533	0	0.546993386	4.622803635
5.018566611	5.926568979	0	1.299559072	4.839744099
5.09614846	4.845032288	0.07032132	1.912518885	4.912581616
3.861159313	3.861856154	0.187063894	0.180197916	4.245870814
0.127753097	9.014987658	0.091300954	2.43814696	8.082059132
0.817351123	10.90025301	0	2.079411091	8.994070384
5.304562436	4.088745768	0.061109995	0.059306134	3.844177326
2.909237427	6.282414189	0.016215214	0.01578023	5.155504417
0.100445579	4.860188026	0.072825557	1.923451589	4.267811858
2.605776872	6.279468162	0.048537317	0.047140845	3.474019662
3.347664286	5.335060557	0.090997395	3.263443987	3.115632069
5.04475685	5.579158371	0.043910575	1.180239668	4.192312157
2.90313793	10.6711348	0	0	11.26139656
3.582290646	5.124724658	0	0.926970978	4.338113172
5.356902353	4.284150428	0.114931397	2.100795875	4.014024139
1.572840494	5.687618609	0.133991816	1.525738766	4.618846656
8.985520217	4.845404104	0.082228585	0.079699138	2.002876898
0	11.04091812	0	2.815422027	8.495343479
4.657722416	5.921054557	0.002013112	2.332899212	5.223498611
2.616009938	5.275449656	0	0	1.527358356
6.198130508	6.825382627	0.039936127	0.038807657	4.261300186
5.809711296	7.144364846	0.015923875	1.066314225	4.966815276
5.243700605	4.320726742	0.010736937	1.637762288	4.320749694
2.458809209	10.03352738	5.83703524	2.273491158	8.194177723

4.797645537	6.132755872	0	1.087647574	2.347161543
2.130302805	3.690576986	1.425691022	0	0
4.775134696	5.100252857	0	1.614652133	2.351465194
0.671943862	10.3124723	0	3.413951995	9.149340744
5.850497069	4.699992592	0.026251767	0.025531589	4.568201488
4.972675835	6.095363323	0	0	5.394684069
3.553676386	5.001356225	0	0.689014561	3.63428664
3.74641949	6.2962339	0.006887542	1.618958065	4.691205796
1.965677165	6.243268626	0	0	3.131002425
4.639142203	5.533038846	0.048631523	1.815856755	3.896007002
6.108356263	4.382636844	0.045225098	1.185510069	3.608447363
4.43653176	6.499986094	0	0	2.564253491
4.72742682	4.972309341	1.565053903	0.983373127	5.101552171
4.820206896	6.534744515	0	1.062937478	4.847374231
2.704648861	6.114459086	0	3.228873001	3.215428953
5.487367258	4.597298008	0.041788971	1.784598457	3.064074289
5.012120358	4.647066265	0.139064699	0.134332299	3.375082098
2.594410826	6.332624962	0.001515029	0.001475745	5.595583535
1.99224831	4.477837014	0.231681202	0.222618576	1.984272032
1.030651326	5.093503246	0.007091088	0.006904805	2.628851732
0.323128899	4.797986477	0.209760882	0.201802524	4.523775896
3.881140142	4.244690599	0.011056124	1.046159115	3.658157709
1.371885306	5.136151106	0	1.781343785	3.887716666
7.966709821	5.479154126	0.066547735	0.064561968	4.597332788
0.928314981	5.307997509	0	3.487463075	3.347585577
3.76679335	6.114118859	0	0	4.347365561
4.231029043	6.74787497	0.035246548	0.034260511	5.559535856
3.778890704	6.840456701	0	0	5.140452632
0.869818191	7.566833982	0	0.872355885	4.737901252
4.791187975	4.134437523	0.050830238	1.82582118	1.846429119
7.44760762	5.117972233	0.157198632	2.267810937	5.11707573
3.632382772	3.964960737	0.106297009	0.102878597	4.831912958
6.981664057	5.022799216	0	3.431518432	0
4.731813725	4.26519983	0	1.277795255	1.64987439
0	0.516293446	0.508377811	0.51015746	2.009752183
2.728144728	5.288610472	0.023381225	0.022743853	4.913165109
4.035714086	5.618966406	0	0.968261046	4.854014931
3.329333484	7.374283889	0.001122453	1.004710461	4.007632903
1.217299261	5.033594772	0.050550645	1.206789371	3.312847234
3.815321242	3.403664109	0	0.887835106	3.815122531
0.783589233	6.578645164	0	3.775575851	6.133653803
7.153787748	5.336775075	0	1.545682279	4.193628861
3.785993232	6.667021952	0.051529288	2.853217247	5.866131141
4.93677931	5.377024419	0.238331333	0.228924303	5.374809513
7.158994405	4.286757981	0	0	3.674667136
1.888196758	5.148579688	0	0	4.770950775
1.415249253	6.886370405	0	1.826794283	4.900691521
2.541498175	6.953658998	0	1.890192152	5.591428206
1.68813881	4.991871284	0.019656927	0.019125512	3.587716497
4.417123531	5.347240053	0.003626278	0.003531784	5.069589712
0.028166511	8.113194584	0.021241425	1.688382885	5.502891451
5.298277818	5.917403688	0	1.739106603	6.418344291
5.764993423	6.415494402	4.57164974	3.91072185	2.728668875
9.41945445	3.819307946	0.080803874	2.723122747	4.761856673
0.370436701	7.750788478	0	0	6.863593388
4.551832061	7.293623793	0	0	3.35801203
3.439366109	6.316754543	0	0.673415157	4.330239194
6.120433935	5.80706303	0.913083348	1.481825069	5.497310664

2.257448472	6.062669773	0.045879427	0.044566704	4.623095556
3.231618076	7.374553166	0	1.101930905	3.63528006
0.117939718	1.357937083	1.344327999	1.340613323	3.080915463
0.904020043	6.655811514	0	0.913234588	2.756689037
7.123138556	4.901357808	3.896777882	0.056204207	4.768921791
2.155247408	7.254369394	0.027359512	0.026607114	5.471255505
3.821168025	5.334934963	0.018295981	1.076099119	1.078290199
2.071484082	4.485774193	0	0.546279654	3.820540447
5.677640774	4.901239219	0.282736138	0.27092037	4.352679957
2.421727108	5.889422791	0.076423178	1.308536676	5.888556795
5.178619821	4.71881509	0.019759166	1.68130042	4.302409797
6.161758006	6.138118073	0.071085845	1.91586141	5.63260806
2.916191326	5.847651079	0.017338861	0.016872553	4.438949777
6.340405567	5.571093647	0.16276931	0.15701368	3.216223891
6.676492317	4.549523365	2.394041835	1.055246901	3.54641951
4.538859061	4.760888397	0	0.972048792	4.202073538
0.083506371	3.971359668	0.061094977	0.059291614	1.897935781
4.779914811	7.294267863	0.637746322	0	4.964732341
6.346917534	4.431786279	0	0.983212178	4.972054155
1.305191043	1.300896648	0.071136922	1.287964476	1.302539604
4.90802329	5.058304909	0.106365354	2.065662139	0.15327349
3.11117534	9.039844987	0	0	7.371730094
2.118090525	5.435962881	0	0	4.275871772
1.051801641	6.894895489	0	0.581915241	5.359086025
4.31453321	4.045701972	0.120734599	2.563456814	1.511889249
5.34260755	6.445761145	0	0.893166695	6.192003467
4.105652732	3.978248995	0.083928153	0.081338086	2.011257424
3.945969801	4.366861344	0.056937825	3.298807193	2.316104037
5.427352981	6.566537624	0.015399878	0.01498753	5.427920827
2.375378649	6.830507684	0.008883131	1.628723784	5.148858955
2.544648888	5.797487081	0	2.182854316	5.052419589
1.892455911	5.294452614	0	0.922121465	3.779653533
0.965044361	3.645740572	0	0.965981525	4.265955219
4.400897248	4.322971847	0.022905309	0.02228157	1.097949704
4.358914105	4.824142805	0	0.979641676	2.554722888
6.432536356	4.937701514	0.085773337	0.083117083	4.540035274
4.711446571	5.934207877	3.843239327	1.294105582	5.053681894
4.230340872	4.308602568	0.008931852	0.008696209	1.03827394
1.91310722	7.424817327	0	1.050945055	6.825370887
3.612426885	5.642805868	4.016349858	1.810203804	4.305552043
5.66652093	2.741653028	3.466183924	1.106768158	1.718967958
1.28951446	8.481852638	3.27564134	2.905156567	6.648210486
3.972518831	6.187121944	0.061352259	2.303436889	5.95886301
4.862756204	6.543611165	0	0.475129186	3.35940286
1.929335064	6.418536299	0.066963083	0.06496328	5.481171594
4.644186793	5.749258294	1.158595716	0.037100527	4.574328698
0.005089668	5.071419924	5.514541085	1.016294855	3.025038133
3.651983018	5.538145666	0	1.470989099	3.843346846
4.681909318	5.816957381	1.188770174	0	5.555679686
5.301102392	6.685797157	0	0	5.779023746
3.963925805	3.501817	0	0.887175718	2.227086867
7.077442739	4.081367404	0.042266649	1.786792938	1.180293116
4.722058286	7.388167843	0	0	5.746160538
0.978700913	4.136090631	0	4.141284063	1.558999328
3.295203907	6.26757143	4.245294308	0	5.285715244
0	5.961323844	0	0.703443304	4.127146368
5.01113906	5.415568934	4.814719267	3.867860981	4.371839181
3.01319094	6.825118626	2.060691645	0	5.857227239

hsa-miR-6720-3p	hsa-miR-6516-3p	hsa-miR-6734-5p	hsa-miR-561-5p	hsa-miR-432-5p
2.345660915	1.296442545	0.05914965	2.386311449	5.726294399
0.04064452	1.199037754	0.039469486	2.261478702	2.291895622
0.020122644	1.099273053	1.697081965	2.131610696	4.757932379
0.010595199	1.0524469	0.010308295	0.012999418	3.896945263
1.940641593	0.066733859	0.063585264	1.979540697	6.862632574
0.028950321	0.029422349	0.028133795	0.035869042	3.941566787
0.085716767	0.087288668	1.404800049	0.109431403	3.240471328
1.062249005	1.062602468	0.012311655	0.015544925	5.016908416
0	0.933643549	0.934573722	0	4.881791546
0.027306914	1.739100298	1.132153149	0.033803302	2.527551305
6.055683694	0.604812537	1.105823303	3.750064376	1.94554321
0.115073603	2.190918691	2.17676286	0.149167908	6.383097803
0.060662131	1.294901783	1.289486126	1.319388567	3.952390707
0	2.563820362	1.980078072	1.564207531	6.50949947
0.09985016	2.116564787	0.096620771	0.128417435	4.119371062
0.024595152	1.724123566	0.02390798	0.030402465	4.602211983
0.586096233	0	0.588653401	2.161818845	7.760024349
2.465999439	0	0.801826559	0	6.059723083
0.04532983	1.836910487	0.044006644	0.056653629	4.703533242
0.123487215	0.125911408	0.119332302	4.780803314	4.145782473
0.093063534	1.447115667	1.438171278	0.119267171	5.906491669
0.142024451	1.670802922	0.137104373	0.18666585	3.382481202
0.016576568	1.679422302	1.080583248	0.020403135	2.447125472
0.079450304	0.080889869	1.376180461	0.101099191	3.19309081
1.377788551	1.380459814	0.076327224	1.41424341	2.564800076
0	1.239052238	0.716922908	0	4.928862461
0	1.562794007	0	0	3.290102159
0.035363643	1.173509386	0.034352509	0.043964725	5.109657877
0.047962516	0.048777762	1.845486081	0.060027618	4.488254391
0.023151195	0.023523724	0.022506408	0.028595558	3.35821205
0	1.343930621	1.64692344	3.004175556	6.124449218
0	1.584647641	1.584653302	2.806970173	3.45897558
1.047282068	0	1.720746015	1.670729747	3.198429838
0.777503043	0.776538848	0.779224147	0	3.92226491
1.261089292	1.882555985	1.258075118	0.06770566	5.502165661
0.136273914	0.139007213	0.131594808	0.178583259	3.862687874
1.33372323	1.336021477	2.712366713	0.087767307	9.246709972
0	0.82936436	1.385261355	0	9.800146989
0.04706612	2.272804788	1.225893539	0.058877782	2.337813182
0.012762431	2.400530343	1.656570961	0.015676574	4.357279782
0.055782159	3.138886014	1.266745156	1.293792787	3.617462342
0.037606576	0.038231659	1.181213117	0.046808906	4.315111389
0.069119244	1.334977765	1.328676943	0.087477469	5.101300193
1.166348938	0.034659652	2.196033243	0.042361832	6.804055579
1.516437518	3.244316463	0.942018375	0	11.59017227
0	0	0	0	3.967490758
0.08636079	0.08794644	1.407733198	1.453836835	4.402783257
0.099839459	0.101719187	1.468779436	0.128402958	3.784444029
0.062710665	1.304632174	0.060815754	3.416689236	3.496890292
0	0	1.845476427	0	10.35622568
0.001595699	1.007923599	1.594013046	0.001948385	5.47352937
0	0.676507407	0.679981829	1.169482076	4.21092091
0.031070406	1.152681774	0.030190089	1.16388854	4.788437129
0.012534935	0.0127317	2.073877551	0.01539526	6.768914632
0.008473655	2.374400629	2.050183986	0.010384677	2.651116706
0.788180982	0.787252322	0	0.776757612	9.847755078

1.493031171	1.113363177	0.619198126	0	5.532414354
0	1.437921259	0	0.86877176	1.420405149
0.723675581	0	1.646808163	2.895164069	1.596069292
0	1.900191673	0.691273718	0	8.522416696
0.020560127	2.122598845	0.019990764	0.025360106	4.080308013
0.84781759	0	0.849082426	0.838984159	5.149155359
0	0	0.705817344	0	4.359849433
0.005446125	1.027007224	0.005300393	1.028760996	4.570798207
0	1.557607122	2.292882638	0	5.167768735
0.037677871	1.795742992	1.181551214	0.046899422	4.905310528
1.780308976	0.035676008	1.777814148	0.043625624	3.280774584
0	0	1.984177301	0	5.62044143
0	1.566736054	1.567060414	0.983404165	3.144004972
0	1.763777112	2.023790781	0	5.206315577
0	0	0	1.505058685	5.586619976
0.03248292	1.767494205	2.187089696	0.040321577	3.587257547
0.103390463	0.105349653	1.484756309	0.133215306	4.84431286
1.005933316	0.001219538	1.005876414	1.592460504	3.331907529
1.770380601	0.169244705	0.159812806	0.220494454	5.431570366
0.005606504	1.027800986	0.005456424	0.006860382	5.604011465
0.151397189	0.154508989	0.146077544	0.199934171	5.028122915
0.008724175	1.043213552	0.008488948	1.639173712	3.070645105
0	1.39508883	0	0	3.887813098
0.051123311	0.051998113	1.244946959	0.064090619	4.889834542
2.500131427	0.934029909	0	0	4.836774612
2.410591522	0	1.429588394	0	5.45841412
2.751709688	0.027930771	1.737048577	0.034025888	6.020225007
1.565726785	1.56560209	2.564148449	0	5.828265805
0	0	0	0	7.008222466
1.191540573	0.039996432	0.038205809	0.049011641	3.487545444
0.115963262	1.552617917	0.112109163	0.15039019	4.746737556
0.080182245	2.0182477	0.077678293	0.1020697	3.965341711
1.609521715	0	1.613561332	0	2.310782854
0	0.759776621	1.300895836	1.278381331	2.59993119
0	0	1.367982566	3.9074576	0
0.018337716	2.434084713	1.089079403	0.022592053	4.404469748
1.550302019	0	0.970577234	0	5.156303458
1.59007374	1.004421022	0.000866548	0.001086439	5.914792652
0.039128877	1.803589416	1.18842777	0.048743087	4.577827535
0	1.867303211	0.895408327	0	2.635672189
0	1.342428177	0	0	5.735022855
0	0.968886524	0	0	4.26752039
0.0398678	1.195289263	0.038717078	0.049683046	5.385500398
0.170073149	0.173671104	0.163932795	0.226720545	5.630874738
0	0.866730194	0.868485207	0	2.78009582
0	0.923626739	0.924687658	3.058256362	3.7746346
0	0.872840141	0	0	5.834943596
1.22195031	0.436221777	1.754205382	0	5.768407666
3.266435843	0.015689149	0.015022172	1.081531896	4.380026859
1.014177859	0.002916101	0.002795664	0.003509243	3.931395054
1.081902702	0.016941361	0.016219155	0.02052786	7.316969695
1.759871573	2.072629627	0.812308762	0.800296316	4.700112324
0	1.168000136	1.173635748	0	5.462181178
0.061664666	2.352187133	1.294146446	0.077737248	4.599491247
1.135151093	0.382816084	0.805325965	1.787301018	6.956132123
0	0	0.805532817	0	5.545725274
2.156658392	1.592259254	1.598760698	1.564432232	4.629345189
1.489763753	0.918530399	1.490802704	0	4.10508058

0.035592487	1.784432814	0.034574318	0.044254608	4.947189401
1.129400289	1.127251558	0.62985317	0	6.596604028
0.064538173	1.313300297	1.307482348	0.08148299	3.080264536
0.488123295	0.942178063	1.306951443	4.21208618	4.719372878
0.044663274	0.045417108	1.828277752	1.235400763	4.544615319
1.7056295	0.021759336	1.704143723	0.026427974	6.316660678
0.014385342	1.071113324	1.665551895	0.01768538	4.768147115
1.046350645	0	1.42118029	0	2.075194344
0.198356743	0.202728725	0.190913952	0.2681613	4.901194662
0.058439995	2.334841886	0.056688761	0.073546894	5.076390585
0.015524581	0.015770003	1.075502228	0.019097577	4.454964422
0.054493776	0.055432861	0.052873462	0.068437819	5.319224132
1.0670605	0.013853902	0.013267342	1.072005123	5.163132242
0.119788453	0.122125134	0.115782135	0.15565764	6.386824286
0.010442299	1.051692811	0.010159634	0.012810775	2.890545901
0	0	1.554804898	0	3.954520202
0.047054887	0.047853162	0.04567651	0.058863379	3.176927532
0.655364114	0.654068974	0	0	6.375758348
0	1.566559112	0	0.98324351	4.431780246
1.26395529	0.055471442	1.879463408	4.369267693	1.301589514
0.080231333	2.018496466	0.077725622	0.102134813	4.986879182
0	1.047198983	1.424743256	0	8.005685485
0	0.70995627	0	2.724821279	4.374240022
0.599017048	0.597614752	0.601535924	0	5.138038814
0.090487687	0.092162102	0.087608796	0.115810405	4.741648256
1.46670114	1.873767909	1.467973442	0	5.545595647
1.932311545	0.065080512	0.062019487	0.080724132	4.106804787
1.213555184	1.214914629	2.842129046	2.281950268	1.241730392
0.012125598	0.012315753	1.059054732	0.014889261	5.315377817
1.034586637	0.007125796	0.006828637	0.008592927	5.789810613
1.651183764	0.728529871	1.965346232	1.624100678	4.502141697
0.926906653	1.498675076	1.500149853	0	3.459608163
0	1.960465019	1.548217592	0.966043741	3.405348869
0.017968655	1.088714546	1.087300017	0.022133011	1.703936871
0.980964249	0.98086339	1.97687371	0	2.776492437
0.065307358	0.066458034	1.934402433	0.082487531	5.00554588
0.055698121	0.056660368	0.05403804	0.06999485	4.368143319
0.007055407	2.042717745	1.034433526	0.008640023	3.057181617
0.589367896	1.450448004	0.591915608	0.572552397	6.333062492
1.1616227	0.651151971	1.165279516	0.636839502	3.280579383
0.020186818	1.699626088	0.019628256	0.024894688	1.718634421
3.519679091	1.350055795	0.995086765	3.655798531	6.404307658
0.047247311	0.048049179	0.045862756	0.059110115	5.04387139
0	0.49189623	0	0.475586906	5.168040533
1.249251665	1.250877578	2.290164537	3.516833106	5.294009823
1.145264295	0.030211174	1.143712944	0.036844871	4.055861644
0.003079316	0.003126563	0.002997371	2.606412406	4.550317331
0	1.887703089	0.911248993	0	4.364426929
0	0	0.697148002	0.680449946	5.18304298
0.768140187	0	0.769915918	0	5.52433212
0	0	1.56661796	1.747207116	2.367261228
0.032846674	1.161306916	0.031912498	1.173240955	4.525842605
0.95192367	0	0	0	6.81954065
0	0.980537284	0.980821587	0	3.289321884
0	0.90341616	2.461554963	0	4.685778499
0	0	0.719754205	4.981281906	4.647337927
0.023786326	0.024169633	0.023122928	0.029389996	4.006875253
0	0.818495952	0	1.751717949	5.161230403

hsa-miR-1224-5p	hsa-miR-485-3p	hsa-miR-371a-3p	hsa-miR-944	hsa-miR-3934-5p
1.97835149	5.763847484	0.102299839	1.994125081	0.066780838
1.848541733	2.627823684	0.066289089	0.067872162	0.04433202
1.110600397	4.696183658	0.031916711	4.753317403	2.448973289
1.654276046	3.085855709	0.016591686	2.667445136	0.011488464
2.007485913	5.409035519	0.110708682	2.47061637	0.071871615
1.773322054	0.050462557	0.046469651	3.936950729	0.031504884
0.130810734	0.164839531	0.148872945	2.160882662	0.0943022
1.667705163	4.107638049	0.01987349	1.669899539	0.013728588
0	3.971859497	0	1.495554653	0.932243214
5.702079015	1.149785203	0.043734173	0.044728198	1.137768013
7.828151136	1.063915451	0	1.414036878	3.100259235
1.645740487	4.840679712	0.208471458	1.66990848	0.127290592
0.090281141	2.436413721	1.343543346	4.660213318	0.066421745
2.297591585	4.970799415	0.981703469	2.296629444	0.983141789
3.282592307	4.119458762	0.176956572	0.182187615	0.110141387
0.035316772	3.197951665	0.039246759	0.040129798	0.026742553
0.561933344	5.226037365	0	3.488425097	1.730987031
0.357317731	5.426465568	0	1.894562386	0.374019694
0.066440402	4.549868285	0.074407532	1.255058115	2.272372252
0.195771575	3.244535573	0.226475245	4.543691664	0.136807283
0.143075737	4.066420304	1.534636919	5.442919871	0.102525722
0.229431474	3.027402294	1.835064295	1.838897606	0.157870517
0.023615362	2.939305328	0.026167143	2.120217861	1.682195781
1.442395546	2.116681188	0.136769493	1.454271628	1.395215558
2.090956937	3.421901036	0.135489289	2.11400916	2.025991189
0	3.87384973	0	3.881341802	0
2.293890147	1.972273842	0	3.138661436	0
3.068061314	3.877251164	0.057261416	4.196039132	0.038532395
1.265237965	3.184580462	1.270161825	0.080953267	1.859540485
0.033196038	2.76509945	0.036870616	0.03769572	0.025165379
2.006731407	8.958360263	0	3.734356946	1.865271022
0	4.321456571	0	1.584596938	1.999657513
3.322895083	3.281831872	0	0.541752913	1.9437991
0	4.452737083	0	3.998083148	0.772554861
1.298611051	4.844592684	0.089575831	1.304627392	0.058970783
1.768260529	1.794565478	0.254667632	3.322320815	1.667469577
1.385251904	8.32393672	0.117678616	4.008509954	1.34588695
0.815596474	8.16603178	0.814724575	0	0
0.06910444	1.898493714	0.077442411	6.814640577	0.051400177
1.668398441	5.065090822	0.020043514	4.10635209	0.013844359
0.08261536	2.740513612	0.092895982	4.91338546	1.279292147
1.829045026	2.271049573	0.061079958	3.101057696	1.801951997
1.384005806	2.839564851	0.117267296	4.554338442	0.075804088
0.049423077	5.462675559	0.055115649	1.190892657	0.037143368
0	9.459642524	0	0.935258614	0
0	3.342606842	0	4.524705027	0
0.131878887	3.680847792	0.150128801	3.997454415	2.499976081
1.55839737	2.718509887	0.176934888	3.767685684	1.493854774
1.347915578	3.298431124	0.105445429	1.355698484	1.313387511
0.863896098	8.923420647	0	0.863347374	0
0.002240094	4.871944978	0.002469041	1.595678875	1.59443653
0	3.584712616	0	5.710231474	2.125054594
0.044901044	6.398586197	0.050016503	3.418432971	0.033826082
0.017786731	5.810280407	0.019680234	2.091009929	1.063481443
0.011976035	2.386268479	0.013231891	6.991134674	2.052838546
0.771105871	8.775834709	7.967915688	1.299845061	0

0.593156515	4.856548865	0	2.652331227	0
0	1.41975992	0	2.795561413	0.873134982
0.703463585	3.212550112	0	0	2.601716282
0.667553913	7.275135909	0	3.040565963	0
2.143343262	2.745090158	0.032629832	5.787883109	0.022337486
0.834456637	4.853595781	0	0	0
1.190895226	3.783365708	0	0	1.217310755
1.620648143	3.853583263	0.008470067	3.745717867	0.005899241
0	4.280989302	0	2.547997409	0.976054291
1.207893396	3.474682903	0.061201716	1.837651055	0.041072509
0.050920849	3.60851082	0.056807092	0.058136186	1.176701565
0	5.468511167	0	2.564293691	0
0	2.974350721	0	2.782244839	0
0.574343884	4.627709061	0.574463561	1.046012061	0
1.909800181	3.476882645	0	1.907104111	0.935330157
1.179024747	1.178614486	0.052390883	4.437467427	1.163659006
0.160615116	4.536957279	1.596781823	3.084364076	0.114121411
0.001685622	5.534168194	0.001857645	2.008751032	1.006100377
0.274313015	3.205233815	0.323713579	3.183975207	0.185029002
2.03941472	4.440767872	0.008721356	0.008904005	1.02844249
0.246911588	3.455605721	0.289356446	4.750420729	0.168569707
0.012333133	2.063166541	0.013627641	3.070111496	2.377747381
0	1.766896529	0	0.826495756	0.837046948
1.282913039	2.979324229	0.084590161	1.92785759	0.055874737
8.621731776	3.79269851	8.558869456	2.700989201	2.235039257
2.380850625	4.667754134	0	2.373033259	0
0.039577847	4.935850192	0.04402857	3.543736661	1.744793177
0	4.363285494	0	3.779320232	0
0	4.178742623	0	0	0
0.057319321	2.618091924	0.064047079	0.065569267	1.197846436
0.18244904	2.835710527	0.21035511	4.814898107	0.128295595
1.446542231	2.57663122	0.138172408	3.191525079	1.398775932
0	4.962342372	0	1.181106734	0
2.427834554	1.267399221	0	4.379119449	0.755576793
0	0	0.950847909	3.039743947	0
1.100749948	3.148022834	0.029015825	4.475008844	0.019914225
0	5.304367997	0	1.956209267	0.969492141
0.001248708	6.434174004	0.001375994	2.32870593	2.005595456
2.270170164	1.845422984	0.063684873	2.885463261	1.196795515
0	2.159180796	0	6.428277474	4.375964208
2.488403322	4.334571387	0	0	0
0.965855683	4.033434327	0	2.949265994	0.968206951
1.22008249	4.729216662	0.064953174	1.223773227	0.043478266
0.282686404	3.599027996	0.334306377	0.346916173	0.189985169
0.855396866	1.408044289	0	1.405899503	0
2.208651248	3.454479715	0	5.510468809	0.922031045
0.861934717	4.417409836	0.8611206	1.414069195	0
1.656565291	4.82607833	0	1.641446417	1.485436367
0.021978866	3.449326202	0.024343932	0.02487315	0.016763271
2.342911207	3.023368946	0.004451496	1.015865506	0.003109415
0.023760797	6.979360553	0.026329243	1.696864291	1.084369728
0	3.983517609	0	3.373670577	2.065382359
0	5.137800344	0.636208626	2.863198379	0
0.09186507	3.664105359	0.103535543	1.998686886	1.308248669
1.07074082	6.909854809	0	0.755694276	2.003947139
0.787804022	5.546238341	0	1.320966419	1.742596525
1.546451025	4.01284719	0	2.080511313	1.197793334
0	4.027400697	1.880797839	0	1.486684493

0.051666792	4.54844362	1.199456717	0.059001255	0.038783475
1.992509438	5.763499366	0	0.60502696	1.494464432
0.096422234	1.360650152	1.366105044	2.01801685	0.070718191
4.842686438	3.71347379	0	1.503869062	3.087496077
0.06542009	2.657781243	0.07324625	7.176553267	0.048753381
0.030655631	4.991869624	0.034027539	1.728636705	0.023271379
1.078965627	1.078439501	0.022641485	4.368251176	0.015609865
1.643475868	2.073436248	0	1.867818679	0
0.339318786	5.403608537	0.407120761	4.827916171	0.222654818
0.086781387	3.464219106	0.097681948	4.866717281	0.063961382
1.085240876	2.930989586	2.112164527	2.43938381	0.016849876
1.30178716	5.461341779	0.09058847	3.894364538	1.272937266
0.019374727	4.364694398	0.021445721	1.075685172	1.06905479
0.189198032	4.775957204	0.218507968	2.855213538	0.132620218
1.057270657	1.654369533	0.01634891	2.401102789	0.011322329
0.971137619	2.543381489	0	0.970923806	0
2.323621051	3.549051062	0.07742273	2.673816753	0.051387797
0.632753379	4.264808357	0	2.024750731	0
0	4.43178908	0	0.98252081	0.983873393
0.0806627	1.948533235	0.090656153	3.229895296	0.059638968
0.121766954	4.107255635	0.138266596	3.422705726	0.08817628
0.541869578	6.467904001	0	1.003103253	1.404048792
2.131720361	4.325988021	0	1.196893402	2.749556384
2.776597637	4.448696928	0	1.403084564	1.080936847
2.61031444	2.998230355	0.158229817	5.307280622	0.099640016
0	4.819578479	0	0	1.462936516
2.436362833	2.460051622	0.107727735	0.110528992	1.319505176
2.302656836	3.15339712	0.071987352	1.24707854	1.220704338
1.066527679	5.234951033	0.019027136	3.558874704	1.656341839
3.053485269	5.06128124	0.010936161	2.863085022	0.007603287
0.709681577	3.110982357	0	4.511836428	1.948935892
0	1.487134809	0	4.45788967	0
2.536579219	0	0.964576348	2.755872569	0
0.025633658	1.70441811	0.028417751	5.360872525	0.019512014
1.972807945	2.554696537	0	2.776595934	0
0.097646454	4.539819393	0.110210476	2.46869545	0.071571538
0.082483987	2.400809212	0.09274522	3.903679234	0.060928373
0.009957727	1.038346077	0.010996437	4.451843488	1.03578013
2.880182829	5.987742037	0	0	1.068161419
0.629760204	2.968649312	0	1.776436476	0.645822638
0.028861018	1.110364133	0.032021266	5.755030449	0.021930293
5.263365489	5.151756601	4.306867854	2.171560215	0.97808329
3.519753964	5.401606468	0.077759943	2.340172495	2.611423607
0	5.128651796	0	2.770612063	1.292533292
0.075843854	4.67847453	0.085137898	3.870639728	1.257818555
0.042900619	4.776971275	0.047764421	2.814205536	1.14987592
1.605154761	3.610896598	2.022342369	2.831870099	2.605093158
0	3.019724596	0	2.662012925	0.908160048
0.67366468	5.261161325	0	2.301976501	0
0	4.744608639	0	2.426324042	0
2.752865543	1.90030355	0	2.916129809	1.539554689
0.047551478	2.569585857	0.053003806	3.432362722	0.035772311
0	5.890531939	0	3.618814406	0.950637462
0	0.978837987	0	5.175810323	0
0	4.528869916	1.455091384	0.894258806	0.90143908
1.209620994	3.089253163	0.696633652	2.525677808	2.910214103
0.034128115	2.996108937	0.037914639	4.519633066	0.025858997
0	5.129289965	0	0.803621491	0

hsa-miR-509-3-5p	hsa-miR-3922-3p	hsa-miR-7-5p	hsa-miR-496	hsa-miR-520g-5p
0.091914325	2.403495055	1.337899467	3.05302531	0.071695246
1.224999206	1.220342481	2.280024463	0.069714786	0.04743608
0.029078812	2.952667512	2.466955814	2.967019522	0.023312016
1.058228974	2.884584037	1.058074188	1.656039388	0.012232132
0.099306119	1.995548087	1.363691495	3.317937127	0.077218505
2.530655978	0.039068722	2.200670986	1.161144856	0.033646022
0.132554126	1.466512799	0.130290583	1.490879388	0.101650075
0.018150206	2.086367037	1.069400743	3.425969097	0.014622244
0	0	0.927504391	3.693649755	0
0.039751556	2.512182257	3.20403705	2.19802547	0.031717245
0	2.928765855	5.088590894	0	0
0.183516626	0.166085978	1.644876571	2.364448751	0.137859185
0.091395049	2.734115662	1.975770276	1.992679801	0.071305952
0	0	3.296114971	2.558839031	0
0.156711571	3.481769667	4.033515921	2.718746059	0.118994841
1.135628086	1.133127728	0.035200653	1.74995808	0.028539541
0	3.685959975	0.562021226	4.829709703	0
0.751157262	0.756630361	2.849805753	4.077306088	0
0.067221275	2.633397949	0.066206927	3.16295691	0.052993143
0.198688257	0.179399727	2.369342628	2.422892312	0.14836531
0.145025229	0.132034643	1.51912203	3.526099553	0.110645792
0.233034644	0.209320771	1.800453582	0.287781098	0.171712482
1.091233343	1.690838902	2.115676701	2.120482787	0.019179233
0.122062649	0.111529705	1.441918044	2.120041092	0.094012118
0.120950095	2.515893276	0.118927062	0.143565456	0.093198346
0	0	2.352066749	3.085027492	0
0	2.557085264	2.293925513	1.972100193	0
0.051907054	0.0479629	0.051148239	1.822265362	0.041194601
0.071318749	0.065693218	1.265008049	2.346608201	0.056123699
0.033558971	1.732415149	1.735683223	1.740268286	0.026849994
0	2.021071713	2.346451951	5.94436245	0
1.99961469	0.999706173	0.999702371	3.169477464	0
0	1.366463107	0.540217994	0.544023959	0
0	2.652607661	1.678584619	2.627363982	0
0.08068317	1.292705398	2.369099524	1.303797153	0.06323741
0.222242885	0.199951878	2.901660776	0.273395579	0.164440613
0.105414788	2.018485785	1.384863044	6.551062108	0.081757451
0	0.817400692	1.755540301	6.336255682	0
1.890889187	0.064420352	0.068860271	0.081542882	0.055057137
2.089718102	1.66661283	3.096604887	2.90900179	0.014745811
0.083619161	0.076871463	1.944790301	2.402704598	0.065456143
0.055326598	1.203814582	3.603324782	4.217673787	0.043842682
0.105054752	2.017140375	2.029298477	4.141548776	0.081490568
0.049983228	2.229382785	1.187842435	4.283062074	1.175877606
0	2.911206442	2.716931494	7.313603051	0
0	2.222382843	1.494065801	1.49231767	0
1.483361599	3.396544123	2.582463631	1.494738841	0.102436878
0.156693017	1.543987596	1.557713244	2.718669124	0.118981647
0.094682284	1.978027889	1.988754604	2.452370915	0.073767496
0	0	0.863956616	6.251569804	0
0.002262866	1.595221896	2.596876147	3.012968479	1.008271642
0	2.651617982	2.077253249	2.271050594	0
3.038475305	0.041999185	0.044750278	0.0525088	0.036137588
0.017974406	0.016700964	1.666834714	2.907201851	0.014481762
0.012100673	0.011254256	1.046424179	2.386461218	0.009775266
0.770849753	0.773162239	1.303788769	6.763035452	5.729437067

0.592886623	1.079548161	1.730852084	2.901735471	0
0	0.86634572	0.864960401	0	0
0	3.252305082	2.366792831	0	0
0.6672738	1.855627712	2.941639975	6.590756212	0
2.737126307	1.111241574	2.960511082	1.114001513	0.023822611
2.092669332	0	0.834525779	2.558154972	0
0	0.68506799	2.333343445	2.105082832	0
2.625743126	1.619917711	2.625493438	3.630476497	0.006275762
0	1.554604431	1.966736549	3.131086982	0
0.055435548	1.20420236	0.054619127	1.837817973	0.043926924
0.051499857	1.807375262	1.193385048	1.820466195	0.040878745
0.985560831	0.985774667	3.564305998	4.299273761	0
0	1.978320361	0.982831771	1.563878767	0
0	0.576805866	0	2.615244397	0
0	2.71257246	3.484368002	1.907027311	0
0.047537935	0.043956945	1.178887157	1.802913014	0.03779969
1.581063749	1.563492148	0.159942471	1.597866812	0.123365087
0.001702734	2.816194304	1.592836588	3.710554054	0.001382034
0.278913687	1.907675558	0.272946936	3.198332631	0.202003036
0.007983004	1.620944513	2.362753486	3.04545296	0.006460949
0.250892157	0.224759611	2.54185398	3.446485504	0.183620692
1.047924476	1.640876229	2.385312266	0.01425065	0.010065185
0	0.828737607	2.083101997	0	0
1.28372146	1.906851344	1.282661977	3.207485311	0.059889861
0.927772243	1.499396298	5.776105267	2.480258844	0
0	0	1.408738236	3.576919604	0
1.15166478	0.0370477	2.519022368	3.927654953	0.031925125
0.981725304	2.782685945	0.981767722	1.975626685	0
0	1.428073319	1.42552033	3.378640673	0
0.057980125	1.213244465	1.216967773	2.618440438	0.04589216
1.653716667	1.632628441	1.649984026	1.675392103	0.138967414
1.448112345	2.084255882	1.446057963	0.146451304	0.094902589
0	2.336262266	1.56285401	2.491863432	0
0	1.271545505	1.654008382	2.199432989	0
0	2.381547698	3.074541562	0	0
0.026451221	0.024543019	0.026085363	1.101576117	1.094824304
0	0	0.967249922	1.544712535	0
0.001261371	1.00480587	0.001244914	3.466855498	0.001024006
0.057656367	0.053225741	2.868664669	2.283152667	0.045642355
0	4.081868958	2.423554902	2.158449059	0
0.781046973	1.320413024	1.708378108	3.453553951	0
0	0	1.543897681	2.535654054	0
0.058789835	2.267214523	2.274781697	3.490201669	0.046516612
1.971618923	0.256149757	2.656788698	3.230283958	0.207553061
0	0	1.409000562	1.406238006	0
2.880293406	2.211436797	1.891253769	2.204274177	0
0.8617435	1.419739381	0.861995857	3.779176899	0
0	1.166343166	1.657172167	3.939975448	0
0.022213057	1.08352187	2.107848442	3.124498279	0.017862698
0.004078144	2.019734082	1.015715743	2.607518281	0.003306465
0.024015025	1.691475788	1.091530358	4.53362087	0.019296144
1.333146693	3.024312081	2.037120587	2.999091778	0
0	2.767161674	1.502480582	4.323900246	4.113601901
0.093002085	1.334932878	1.982126194	1.348737038	0.072510176
0.754614766	0.760108966	0.755377649	3.479022333	0
1.324062628	2.03270383	2.49875976	4.554488623	0
0	3.296466191	2.493442207	2.635693651	0
1.883454116	0.912127606	2.201178623	2.675239573	0

0.052255233	0.048281895	1.815834483	2.590489527	0.041464589
0	1.462247489	0.604180597	4.944259582	0
0.09762641	2.426099378	5.432141114	1.365520761	0.075966387
0	1.246964305	1.951658697	3.66295014	0
1.247485232	2.298453877	2.307089488	0.077089505	3.097333126
0.030988863	1.115882988	1.117660861	4.08576161	0.024822101
1.079131411	1.077782758	1.078913564	1.079541353	0.016630734
1.641914833	1.008174557	2.710966431	1.631844977	0
2.141322032	0.305290794	2.131558116	2.217783401	0.244304622
0.087844672	0.080701427	0.086463687	4.057949973	0.06863974
1.686707873	1.083952754	1.686267332	2.112863611	0.01795522
0.081579045	0.075020486	1.936599384	3.430965949	0.063915008
0.01957992	3.27042932	1.07480858	2.915935215	0.015763904
0.191986868	2.3207443	2.346395104	1.699223874	0.143739637
0.014941836	0.013890178	2.073121643	1.655013826	0.012054939
1.550138916	0	4.127999012	0	0
0.069903652	2.313967499	1.889207283	1.900900399	0.055043775
0.632475502	1.131374857	1.126326613	4.010099296	0.644004029
0	0.98288143	0.982665707	2.974142426	0
1.302884812	1.296003527	2.706310709	0.095593064	1.280314738
1.448391983	1.436462806	2.542551749	2.125354966	0.094962324
0.541617593	1.657985202	0	4.927032608	0
0	2.542909237	1.882052024	3.295226007	0
0	0.57754327	1.050421809	3.268252132	0
0.140631587	0.128122435	1.504480229	2.651495598	0.107486777
1.45125606	2.175451839	0.890126471	3.001561892	0
1.356040223	1.98564647	0.095142405	1.362120306	0.075266456
2.635804149	0.059994037	0.064088416	1.246323605	0.051342004
0.017380198	0.016150433	1.664171278	3.421643087	0.014006767
0.010005835	0.009309166	2.860243378	3.227359058	0.008090672
0	4.016442797	1.225769856	2.855970237	0
0	0	1.487999959	1.892155905	0
0	0	0	0.964781959	0
0.025909113	1.09719437	2.125294359	1.099515187	0.020800387
0	0	0	1.971895942	0
0.098868843	1.993897078	1.362171029	2.024091983	0.076892712
0.083485926	1.302366763	2.714257295	3.440270994	0.065355576
0.010060861	0.009360275	1.038642148	1.038884911	0.008134963
0	1.695850292	2.48050401	3.819742675	0
0	2.257632765	2.430735194	2.716094063	3.780847421
0.02917346	1.109217597	2.957777593	1.111912573	0.023386905
0.945032955	2.370050786	5.383358458	4.129420463	1.613712024
0.070203446	1.256294606	2.656453838	4.713435202	0.05527267
0	1.535470848	0.468470948	3.6745873	0
0.076752774	0.070636886	1.91711633	3.735627675	0.060258563
0.04337994	0.04013931	2.801987871	3.042130676	0.034556777
1.016892416	2.34381631	1.016850621	0.004987545	3.92830721
0	1.468170667	2.188450824	2.441977969	0
0	1.563248736	1.179459634	3.620363029	0.684395076
0	2.637333149	1.277107975	3.67586813	0
0.872507163	0.878565791	2.856735382	1.20387967	0
0.048088221	3.04172851	1.798192295	0.055662923	1.169461382
0.946983583	2.252239987	0.947098802	3.82396305	0
0.978575551	0.978889346	1.559274469	2.291983954	0
0	0.895966554	1.862308849	2.650575697	3.668538995
0.696972515	3.625455911	2.543388726	3.090230369	0
0.034502024	1.128739266	2.492931073	1.744530258	3.74545587
0	1.348695841	1.345191157	3.850796233	2.070660543

hsa-miR-409-5p	hsa-miR-147b-3p	hsa-miR-376c-5p	hsa-miR-382-3p	hsa-miR-539-5p
5.764380551	1.950496515	2.415621924	3.283739642	4.593713751
2.627543484	0.049972038	1.224111727	2.294118484	1.855499505
4.419500012	1.105752368	2.140038693	2.14614318	4.076692918
4.550833536	1.055717619	0.014930088	3.409280933	2.667793974
6.747384319	0.081620641	2.445950191	3.517376478	6.486957959
0.050591077	0.035389028	0.041526251	0.049060991	1.158886413
2.158463964	1.447891194	2.133295503	3.239730697	3.241593234
4.917134697	1.066545891	1.667571471	4.194588143	4.917085539
4.467746577	0.929989399	1.905632083	3.210475552	4.740148327
1.766303652	1.143433253	0.039106526	1.151659564	3.391835954
1.94448078	0	0	0.586291501	1.420036814
5.109986367	1.599483691	2.316340677	3.694083671	5.263982002
4.198301181	0.075321021	2.413476256	3.051220818	3.051794296
6.099726204	0.98254862	1.563206012	4.43076896	5.328210032
4.261570139	2.171047174	2.66895226	3.066642175	4.119482188
6.021326984	1.129215446	1.135159411	2.178466716	3.52380284
6.712238474	0	3.092030915	4.537995383	6.964888492
5.931034398	0	3.294989728	4.626529171	5.355951684
4.774400639	1.23775563	3.333276547	3.360523407	4.284054167
3.527792463	1.642751275	0.194379372	0.244463804	3.528087058
4.337337535	1.485920443	2.179278796	3.016539179	4.455531011
3.383817862	0.183384639	0.227713795	0.290435118	3.907884
3.597026846	0.020139307	0.023494012	1.091609769	1.090456898
3.19385875	0.099558021	1.44162632	2.916041644	2.916198082
4.095904915	0.09868732	2.532536719	3.187666102	3.622181165
6.150458742	2.378706362	3.330140271	2.334699638	4.560328715
1.972307946	0.979901413	0.979141974	2.554853443	2.968812598
4.102168901	2.226990902	1.194777581	3.08631332	4.824014427
5.869312666	0.059182426	0.070082704	2.682869115	3.711344664
4.283598676	0.02821894	0.033021737	1.128330689	2.496909683
8.155765052	2.389550949	4.038615083	6.306534723	6.333682082
4.754411247	0.999713601	0.999702477	2.321512082	2.321513658
6.350606166	1.019995899	2.580501492	2.680978371	4.125704639
4.182174591	2.666945488	2.455931241	3.311254703	2.940403878
5.545032648	1.282635128	0.079258541	1.947105606	4.619988321
4.404674142	0.175482531	0.217248192	0.275864124	3.625751846
9.094315833	0.086462265	4.59703125	6.160533934	8.225952776
9.261272317	0	3.529847459	7.182977629	8.962250353
1.898024237	1.246825723	0.068712732	2.676202601	3.549131441
4.633647904	0.015476039	0.018021681	4.35635202	4.75417497
4.041700256	0.069096308	1.308551819	3.24387632	3.443477354
4.315122052	0.046167648	0.054398612	2.605710966	4.221048177
3.878673127	1.361750951	0.10311115	2.049847544	4.019592346
5.994923047	1.179006419	1.806075604	4.371845027	5.848474665
10.43064906	0	4.490394861	7.802263971	10.25880403
5.920298586	2.900679621	0.924805636	1.898933267	4.127318489
2.620474638	1.451227333	0.131036253	2.622407358	2.163590205
3.961917364	1.520940119	1.557295905	3.578937138	4.509009843
4.214687634	2.993192622	0.092964513	1.354393139	2.451052243
8.812473186	0.868216057	4.044424479	5.841525015	9.407713975
4.537440228	3.011263413	1.595415376	3.01298259	4.183674444
4.444863537	2.318666372	2.475170869	2.894647449	4.159190977
4.909253991	0.038020505	0.044659152	4.165352677	4.167381992
5.467130613	1.065909978	1.068707772	5.014546536	5.753659503
4.465412332	0.010253454	0.01191613	2.875062827	3.771915033
8.360327636	0	4.600304254	7.312369686	8.577402451

4.942908084	0	3.425679942	3.54395647	5.12716214
1.42000404	0	0	2.130013205	2.605294314
3.10033588	0.710726383	0	2.534251405	2.84510504
6.777282777	0	3.382087925	5.630190667	8.217714285
4.173995142	1.711144127	0.029252454	2.970635715	3.16577769
5.394688349	0	2.351030051	2.91347301	4.187480263
4.970795758	1.205018162	1.569623534	3.064079551	2.663143959
4.21672576	0.006580094	1.620593232	2.627706625	3.853583385
4.129157496	0	0	2.285870797	5.243723818
4.964404296	1.813841116	2.259758054	1.210291284	3.301537606
3.281059446	0.043031991	0.050642959	2.856983712	3.608506049
5.783927489	0	3.301444156	2.78616417	6.368824332
5.016698197	0.98346559	0	1.977056695	3.673730218
5.43720546	0	1.049588131	4.413025429	4.986454995
5.096362239	2.495507117	0.930810046	2.704700758	5.410104269
2.233989524	3.027146231	0.046753561	1.802802566	2.566838895
5.376272892	0.131065908	1.577539418	2.284008227	4.289172422
5.098021199	3.178449869	3.009183075	3.009775239	5.13984214
2.720667503	0.21642289	2.628941029	2.723515041	4.092936567
6.509111328	0.006774407	1.030685352	3.954358418	5.541167624
4.364531835	0.196349641	1.854136244	1.900521859	0.329461064
1.642861109	1.045883768	0.01227139	2.653328975	2.877046643
3.186070941	0.832202187	0	1.765304303	0.829690937
3.581081779	0.063180759	1.914892673	3.579210742	4.230175471
3.347584319	0	0.927945906	2.48021534	3.972535697
5.402142386	0.859721945	0	3.470631952	4.155920596
6.019935375	0.033572365	2.190502789	4.03584748	6.096465458
6.310073357	1.564179085	0	3.878444947	5.725248134
6.0285326	0	2.142378793	4.541229223	5.847114625
3.117497457	1.206442829	2.601595594	1.848875694	2.283450048
4.636522777	0.14789313	1.649450756	1.674854773	4.087301478
3.432412163	0.100510963	1.445762122	1.457312452	3.432625366
3.844125415	1.199233453	1.185351652	2.801117646	4.091707151
2.414009141	0.749028307	0	0	1.95136063
0	0	0	0	0
4.155403778	0.02229847	1.100638915	2.461136812	3.472941763
5.877227818	0	2.539936178	3.27192912	3.948312869
5.562458876	0.001073009	2.006263182	4.329573678	5.095284892
4.855417345	4.79105377	1.215686109	2.887798553	4.132887236
3.517849714	0	0	0.884792043	2.635315679
5.272242674	0	1.708532928	1.701749356	4.800774783
3.532008688	0	0.965885638	1.543053653	2.274233986
4.65844988	0.04899829	1.843065117	4.725598671	5.834023043
5.065964252	0.222497478	1.963249852	0.365775406	4.945233705
4.286681776	0	0.855499102	0	3.232098218
4.324784183	0	0	0.916648796	3.454483034
6.322995117	0.866304212	2.610233126	4.235518533	4.941286745
5.948660875	0	2.187992822	4.245301756	4.75546532
3.937601869	1.680048702	1.685687767	2.705629432	4.131120541
5.112673934	1.015110549	1.01571	3.724637117	3.483475056
6.622212156	0.020262343	2.707839316	4.897378323	6.275379702
5.954782039	1.344226846	2.510019113	3.574625835	4.24410949
4.850314471	1.823863697	1.799442439	3.196791443	4.450304981
4.975647272	0.076603417	0.091320206	1.348294861	4.314982798
6.923587935	1.592102643	4.219607024	5.624164591	6.058814372
4.730232351	0	2.281937109	4.292273202	4.23148747
4.422233483	1.185091173	1.546984256	3.607277448	3.823415268
4.983659732	1.482528432	0.911168173	2.455514983	3.445715546

3.612437036	0.043651611	0.051384383	3.284329044	3.458070129
6.69369757	1.10421428	2.38699231	4.4325059	5.730793416
3.312270016	0.080285871	0.095845284	0.115741132	3.511676909
5.696328464	0	1.95207831	3.124016875	5.597951551
3.685006946	1.855168598	0.065052059	2.929968051	2.321631796
5.433697103	1.112539636	1.724369772	4.179739715	5.04266001
4.982711563	1.673582543	1.67880823	3.440246202	4.647463775
3.575654409	0	0	2.406716942	2.408591706
5.405746114	0.262883504	0.336371103	2.218287403	5.173454478
4.39025221	1.935737245	2.398752026	3.264248086	4.062911669
1.084537868	1.081613654	0.021980661	1.689034588	2.439528148
4.849470508	0.067457742	1.301344623	3.760572698	4.358308018
5.163059095	0.016546494	2.909461132	3.572385813	4.976250314
5.794666269	0.153050882	2.796309465	2.863638757	4.666244587
2.401086766	2.39241961	0.01471246	0.017197443	3.255523185
4.041833975	2.285691351	0	0.971094022	3.2778175
4.564123181	0.058036546	0.068695579	1.900785551	2.9483059
5.369502939	0.640654614	1.497192096	5.542949677	4.638704643
5.431316725	0.983305518	2.561203531	2.781844805	5.016442425
3.004974143	1.913004029	0.080194696	1.306892092	3.005223109
4.748539404	1.419456617	2.541993259	3.430381703	3.433001826
6.354335553	0.550083465	1.361409268	5.658268907	6.243065675
4.673765093	0.697804633	0.690543933	3.726888296	3.567410867
5.379420307	0	1.411652753	4.634599546	4.78962952
5.884653696	0.113997479	2.608583653	3.275510251	5.272449945
5.88340595	1.457872729	2.172133069	3.527242419	4.405985896
3.681989724	0.079539898	0.094927601	0.114590242	1.357206462
2.316114726	1.230489641	1.242446011	1.879811663	0.078576541
4.10317333	2.668040956	1.066458262	2.904058374	3.802480425
4.645823696	0.008484799	2.637071327	3.379914966	5.616277438
4.149979352	4.163217802	1.225898498	2.35987803	3.516390311
3.198296674	0	0.919829133	2.468281107	3.459487691
2.947454677	0	0.964913094	2.272669785	3.116710309
2.457824801	2.117023829	0.025501332	0.029936568	2.45794194
3.966899027	1.560993524	0	1.559167551	3.427086917
5.191429207	1.341923786	2.444167125	3.086275431	4.79107202
4.368136499	0.068989366	0.082003682	2.402272657	4.267052837
1.631785444	0.008531291	0.009908154	1.03882515	1.03833758
6.832679961	1.053326258	1.686349323	4.544827457	4.769416293
5.016086896	0.637680441	0.62990989	2.227129513	3.279947214
1.718973671	0.024569505	0.028710941	0.03374747	2.145831795
6.266425648	2.052246466	2.015219459	4.95609093	4.563025578
6.077057267	0.058279397	2.925194979	3.178258762	3.705652377
5.970066352	0.923050709	3.004131007	4.56732195	4.758440602
5.342873999	1.26962064	2.352131835	2.981378583	5.524121112
4.42134573	1.156109304	0.042670504	2.215489623	3.238687246
5.860132585	1.016200796	0.004307861	2.832031852	3.833535551
3.750788297	0	1.466241771	3.652513669	2.442330185
6.053859491	1.575630298	3.189216977	3.361825236	4.478911303
5.614509513	1.990059646	1.66499276	3.399379616	4.530460342
4.241633842	4.6450768	0.444295095	1.699901808	3.55989197
2.236598867	1.78506466	0.04729384	1.182903953	2.236846417
6.270524639	0	0.947115913	5.69582127	6.707310868
2.554206559	0	0	0.978574851	2.292096426
6.619078475	0	1.457531806	3.739989155	4.078285942
4.236761081	0	0.697398367	3.404315672	3.579807235
3.517198622	2.154150277	0.033948548	1.131890122	3.782780093
5.667876112	0.809752992	0	3.768575593	4.198939593

hsa-miR-3131	hsa-miR-31-5p	hsa-miR-499a-5p	hsa-miR-3660	hsa-miR-141-5p
0.079726654	1.342228579	1.345577337	0.058103111	1.339383183
0.052462987	0.062230886	1.857662392	0.038798597	1.855070573
0.025641839	0.030094974	3.973662208	0.01924743	1.718512281
1.056486003	1.058544265	2.66754386	0.010143993	2.667722328
0.085974785	4.08200699	2.025316007	0.062450416	7.202417312
0.037095621	0.043735486	2.808619465	0.027667053	3.036444296
0.113793519	1.48476523	0.154004869	0.081475878	1.483447134
0.01605179	0.018768232	0.020396841	0.012114513	3.102382873
1.500280656	0	1.495558238	0	3.792038476
1.755109596	0.041175393	1.152358643	0.026100708	3.21938619
0	6.621385784	0.583890867	0	3.190726207
0.15556126	2.789235071	0.216448721	0.109089894	4.510673824
0.079286839	0.095054628	3.277098585	0.057796043	0.111982692
2.298745642	0.981754683	0	0	6.27418995
4.747877479	0.163822202	0.183372416	0.094787521	3.78498823
0.031429141	1.136479512	0.04032802	0.023515009	2.507559295
0.567142751	7.063919118	0	1.456697239	2.593494254
0	0.357175715	2.421948201	0	3.469306659
0.058682459	0.06978007	1.889119016	0.043251518	1.886341997
0.167799591	0.208383251	1.722406115	0.116978876	2.41862212
0.124110383	1.527549118	0.169113838	0.088399558	2.668677171
0.195186414	0.245056861	3.647547259	0.134322275	3.026371615
3.288340246	0.024691837	1.092055813	0.015861171	1.090280394
0.105065028	0.12725598	1.454262835	2.001639027	2.115764058
0.10413676	2.546158358	2.565093547	0.074931409	3.42161402
0.699084969	0.693401053	2.124152044	0	2.683227318
0	0	0	0	3.874141456
0.045495858	0.053817898	2.857395033	0.033774895	4.296695507
0.06219295	0.074057409	3.180775142	0.045751896	2.34486588
2.153350745	0.034743995	1.128935614	0.022137622	4.001676315
0	4.989833628	0	2.109874289	8.401909159
0	0	3.169479945	0	2.584535432
0	1.638596167	1.001076145	0	3.642355703
0	1.981154038	0	0	3.197453894
0.070188118	1.301950817	1.304557366	0.051412017	1.299008498
0.186628309	4.307034952	0.26516562	0.128947583	1.79267357
3.27217522	0.109756486	5.849836201	0.066010027	1.387578784
0	4.710999827	1.356038296	0.832345978	1.359208433
1.250975875	0.072598152	0.079762126	0.044900689	3.177405065
0.016187893	0.018928386	1.070758272	0.012216285	3.8077849
0.072686915	0.086914243	1.315309784	0.0531713	1.30958855
1.200536527	0.0573788	0.062846595	0.035909217	3.103980851
0.090817869	0.109378532	1.39319679	0.065801048	2.496460045
3.408827633	1.189756562	1.190822192	0.032569439	6.04953637
0	1.506456303	0	0	1.914347544
1.905861194	5.792275895	0.924347796	0	3.342654617
0.11469429	0.13945215	1.495482095	0.082083168	4.512070852
0.133705864	0.163802569	0.183349702	0.094777455	3.583028772
0.082069095	2.437151113	1.355641048	0.0597362	5.778598971
4.331605456	0	2.793761833	0	2.386193154
0.002009832	0.002336975	0.002531368	0.001529128	3.33519233
0.660830635	1.823312881	2.062208884	0	3.328268929
0.039865151	3.237389663	4.959183668	3.355148359	2.556035897
0.015897064	0.018586187	1.069490199	0.011998796	1.068145333
0.010719077	0.012506716	4.531086365	0.008114522	1.641183672
2.014435412	0	7.916865528	0	1.303946048

0.59834149	1.433697068	1.718296435	0	3.810967923
0	0	1.81738295	0	0.867090243
0	5.272363056	0	2.215856258	7.38634184
2.830402389	0	0	0	3.824533443
0.026206508	0.03076457	3.869782303	0.019665032	3.165688504
2.102961937	0.833714331	2.747737252	0	3.732019127
0	0	1.561239331	0	3.063899607
1.029032089	0.008010791	7.712056424	0.005216893	2.627593766
3.135431002	0	1.965616827	0	4.351294304
4.265622633	1.209913327	1.837752234	0.035977044	3.301396494
0.045143796	1.195373673	0.058434867	0.033520031	2.252676837
0	0	0	0	1.567474072
0	1.564086754	1.977069218	1.981268293	2.560276398
0	1.694325007	1.046252295	0	2.976826318
0.932374822	0	0.93037455	1.923398214	3.591800802
1.173212812	0.04927095	0.053877559	0.03103232	3.064049892
1.550895647	0.170343828	1.598389674	0.09811691	3.375017965
0.001512563	0.00175842	1.006627232	0.001151126	6.549848043
0.231104794	1.962485959	0.338497536	0.156456602	3.85330035
1.029887127	0.008248186	2.364565707	0.005370434	3.954650623
0.209255338	0.264199774	0.301929687	0.143072053	3.45499643
0.01103772	0.012880119	2.06344978	0.008354215	1.642845995
0	0	2.075148449	1.802841983	1.373326551
0.066422642	0.079226742	2.368185748	0.048752212	1.925073271
6.293027284	0.927478828	4.743307601	0	3.06005969
0.858201692	0	1.804034117	0	2.946541894
1.146553586	0.041451002	3.390643557	0.026269638	2.198072763
0	0.98164114	1.562545949	0	2.97277556
1.430439004	0	0	0	4.485215309
0.05073776	0.060143336	0.065911499	0.037558126	3.487784033
0.15684969	0.193936375	3.689443605	0.109924596	2.366171377
0.106081161	1.452669571	0.142841683	0.07625418	0.153230108
0.683015488	0	0	0	2.655681577
0	0	1.646439673	0	3.045038586
0	0	5.072379187	0	1.810259571
1.699760528	1.101563541	0.029796577	0.017543242	3.147934077
0.968041422	1.95694947	2.537798434	0	4.591243664
1.590587263	2.328552438	0.001410634	0.00085304	3.814933542
0.050458731	0.059805973	2.282455941	0.037357282	4.415916863
0.887134812	1.443534144	0	0.895946527	2.159381214
0	1.314286129	2.260580342	0	3.4521379
2.762125613	0	2.274146295	0	1.543497014
0.0514354	0.06098715	1.852449057	0.03806002	1.219512141
0.237732795	2.690958396	0.349803255	0.160469369	3.598520027
0.858410516	0	1.405924043	0	4.156256841
2.474536031	0	0.916194238	0	2.874313397
0	2.387488765	1.414091161	0	3.242736489
2.925836606	0.41247568	0.829478369	0	11.88869145
0.019623543	1.687171246	1.085730085	0.014780131	5.492290446
2.019329096	0.004212309	2.343796532	0.002751882	5.06959137
0.02120522	0.024844279	0.027033266	0.015957116	2.714916182
1.736718613	1.331620821	0.794749809	0	3.573519097
0	2.596922369	0.637412708	0	2.422518031
0.080647585	0.096738225	0.106807356	0.05874563	4.088667877
0	0.359609342	0.756186516	0	3.114479254
2.038047439	1.713973645	1.321024936	0	4.771785421
0	1.542334196	1.166883673	0	2.914731743
0	0.910614075	1.475273221	0	3.034105261

1.18979592	0.054180383	0.059304942	0.033992699	6.190180688
0.609255918	0.603286933	1.446350789	0	1.092704779
0.084557015	0.101585211	10.24773933	3.690163544	0.119951256
0	1.509282734	0.466009701	0	3.415732684
1.860808561	2.313461201	0.075420902	0.042618263	7.327840054
0.027312225	0.032076615	0.034953853	0.020482004	3.344393952
0.018265006	0.021374659	2.430923625	0.013767516	3.821313767
6.197192135	1.000035539	0	0	4.520801726
0.281982477	0.36650443	3.425401153	0.186726117	0.477154399
0.076276637	0.091336601	6.524284619	0.055690643	0.107462264
1.082772056	0.023097241	3.448454223	1.075050829	2.705989594
1.919892018	0.08478013	1.307840528	0.051949604	2.730512217
1.072718113	4.916962475	1.07564798	0.01305443	5.163069661
1.638036615	0.201250085	1.699362854	0.113512096	1.689441429
1.055670708	1.65413859	1.655061932	0.009997758	2.075472459
1.551476582	0.970957639	1.961426343	0	3.277836289
0.060981412	0.072579884	1.264897718	0.044890021	2.675217848
0.637844868	1.49235166	0	0	2.577140455
0	0	0.982530878	0	1.977005927
1.290773441	2.38127495	0.093447232	0.051985504	3.607918219
1.427681274	1.452953735	1.458906312	0.076300515	2.121059211
2.612750803	0	1.635372924	0	3.645892489
0	0	0.690794315	0	3.077470105
0	0	1.403166311	0	1.409999587
0.1204829	0.146828119	0.163778292	0.085973059	1.511740285
0	2.169588167	0	0	2.643724281
0.083764824	2.004583047	3.673680688	0.060915964	4.529186902
3.303426352	1.245269139	1.247003772	0.041930389	4.957066933
1.661122201	0.017970909	2.41388758	0.011607442	3.42209512
0.00886842	0.01033987	3.379457621	0.006720675	2.863415492
2.749239877	2.156225592	0.709922633	0	4.821341921
0	0	1.486017924	0	0.920932415
0	0	0.96465039	0	3.530493068
0.022866118	0.026807789	6.106487257	0.0171908	3.469883608
0	0	3.668152594	0	1.972111286
0.085605816	0.102888062	2.469262144	0.062194469	2.469125605
0.072573601	0.086774848	4.701338328	0.05309162	2.400270715
0.008917058	0.010396778	2.640055308	0.006757345	3.380382838
0.570468586	1.6796228	0	0	10.50190139
1.132462409	3.561529296	1.484201403	0.656167245	2.410911254
2.94927363	5.16124996	1.112280599	0.01930869	1.718938534
5.371451839	3.587195019	1.284932846	3.679107445	8.793136271
0.061238155	4.17194678	1.901997735	0.045072757	10.6767429
0	1.952116686	0.905117128	0	1.520690068
2.664305283	0.0797342	2.706557761	0.049045489	2.36971749
0.038107622	1.165206993	1.784402964	1.142810104	1.162876846
1.016414333	2.831154988	1.017002597	0.002950408	4.274494646
0	0	1.463854176	0	2.184019391
0.678580282	1.552026306	1.175503865	0	2.92651301
0.754346987	0	0	0	2.61202812
2.12224485	2.239151831	2.224661013	5.050371503	3.560078078
1.789044042	0.049843462	1.805338168	0.031378713	4.081554374
0	1.52044318	1.519796041	0	3.089536573
1.973525466	0.978477573	1.55867751	0	2.9680682
0.897114301	3.302192596	2.173279534	0	2.173886509
0	5.754089719	1.582192809	5.421036188	5.756199917
0.030380156	0.035723012	2.172303037	0.02274352	2.501494372
0	2.87440664	0	0	2.864858436

hsa-miR-5683	hsa-miR-129-5p	hsa-miR-889-3p	hsa-miR-204-5p	hsa-miR-653-3p
1.992753204	5.489749251	6.94834622	6.965097591	0.082082038
1.228267808	1.85675779	4.036535422	5.25382307	1.839318571
0.032028187	2.739876859	5.930768652	11.14216843	1.108121509
3.787439413	0.016615119	5.926220399	4.735606137	2.07166376
2.468064277	2.467752013	7.099502413	8.438804105	1.990855317
2.208478003	1.778675439	4.049205262	10.70539111	0.038095748
1.49099874	0.149148123	4.876327843	7.217475045	1.46187483
2.417374462	2.091553499	6.587537301	5.944198143	2.085472226
1.902879255	2.220371644	7.245776423	6.464792783	0
0.043891008	1.152511708	4.318019842	5.791617913	0.035891611
5.642896555	8.802480914	3.522324874	9.180447819	1.727469597
1.668279124	2.814521505	6.742090969	6.916002705	0.160858282
3.93714892	1.34357842	5.907858131	5.601144331	0.081627131
0.981705528	1.562711349	7.803070182	4.363482272	0
0.177777021	2.252873285	4.508868462	5.21563616	1.538264372
1.749628362	1.137214973	5.737375462	5.705592079	0.032265629
1.383390618	7.653032911	8.093389885	0	2.148418673
3.314673049	2.426222973	7.298990796	7.724307726	1.343467535
3.680595878	1.888034119	6.390501351	7.133506595	1.243877409
0.227613473	0.226951557	3.762108129	7.074742033	0.173633559
3.004032233	3.003386077	6.396236029	8.614567424	3.202009694
0.269114742	0.268281875	3.379586414	4.289284848	0.202283186
2.119815177	0.026204927	4.534436595	11.31305423	1.689874395
3.609579924	1.4537206	4.65318558	4.347288191	0.108328569
2.560914154	3.405819799	6.188360996	6.473695187	1.42440523
2.967912398	2.686876386	6.063265426	5.839234123	0.697770143
2.292894575	0.978933597	4.666965024	6.340862323	0
0.05747288	0.05734998	5.51360657	5.391101227	1.806806307
4.291734987	2.949144702	6.244933489	11.35617741	0.063964524
0.037000838	1.129083735	5.486300208	6.450598583	0.030340465
1.278314452	2.166156369	9.46745003	4.23824444	2.207870623
5.087001759	1.584597404	5.882159284	6.845003347	0.999707932
3.207816858	2.918589143	5.919698452	3.82313703	0.543833083
3.422077971	2.796310545	6.012657332	5.695948987	2.823448717
0.089929479	0.089723921	5.50233226	6.015242864	0.07222135
4.355262867	0.255226762	4.244345855	7.420441177	2.405413695
2.494551057	3.106742681	9.979782598	8.83205529	0.0938751
2.314214723	0.814741384	11.51212371	8.077065955	0
4.378530226	0.077567344	4.717612822	11.52335908	0.062729471
3.912270235	1.670463345	7.25259665	6.48771513	0.016603637
1.957972705	1.95781492	4.714039623	7.94810672	1.300247623
0.061307366	4.392079428	5.492758867	8.799672468	0.049789154
2.04714165	2.046917132	5.832046164	6.654287533	1.372228159
2.245190435	3.975132846	7.129973205	7.251426868	1.798987083
0.935162813	3.70839813	11.09750682	5.47941548	0
1.899201485	1.899232503	5.086337983	6.805775924	0
2.162650652	2.95543212	4.708380229	3.477575769	1.465354717
0.177755206	0.177278249	4.989202903	5.961898902	0.138086572
3.658410795	4.498488065	3.967953263	5.924730244	0.084505248
2.38682147	0.863097307	10.47628787	4.904010675	1.826026122
0.00247709	0.002472413	6.522020312	6.834426682	1.008563921
1.818019339	1.522620247	6.018248119	7.268325371	0
2.554362296	2.223499269	6.555967403	5.553608141	2.207081024
1.069592212	2.090681506	7.611004209	6.797365494	2.084662331
1.641766358	2.874154483	4.881096603	11.89535868	1.045497807
0.77032453	0.770289945	10.67851784	8.378796914	0

1.71908382	1.960448641	6.53689363	6.50876584	1.44947978
0.864119829	0.864103747	3.783030642	8.492798927	0
0.702905572	1.21348939	5.265890633	7.790535413	2.16531026
1.167210976	1.167206812	10.61061663	6.205588041	0.671133647
5.215169951	1.722614513	5.307632637	6.795392926	2.464270857
1.775866584	1.775920829	6.042289523	5.394452359	1.787170439
0.6822392	0	5.799656317	6.19549385	1.19802931
2.850402208	2.627238259	6.440683353	10.09990377	0.007048855
0.974036862	3.545976097	5.765368779	5.481702833	0
1.211343493	1.836953053	5.36725763	6.574561628	1.202636367
3.079872591	1.196640947	4.465662956	5.471660643	0.046383032
2.786408191	0	7.126098334	7.523217487	0
3.97368951	0.982664257	5.726923899	6.038048854	4.620133892
3.787829325	0	6.885961742	5.583672831	1.714652322
2.224975247	0.930220818	6.599526864	3.977325746	0
0.052582346	1.181804831	4.360911934	5.449649678	0.042850793
3.354712028	1.596920668	6.148089307	9.162518928	1.557406255
4.817496443	1.593019037	7.323629819	4.654330663	0.001550046
5.905652973	2.701167238	5.03233084	5.032238002	0.239983954
1.031040981	4.570504033	6.19945316	5.297179297	0.007257251
4.132933941	3.068887495	5.130664919	8.609823068	2.491714078
2.652596997	0.013646753	4.776185063	5.120617172	2.059090607
1.370361305	0	4.880987654	11.16364149	0.829538718
1.92684657	1.926709223	5.758232606	6.022459037	1.903391842
1.903445182	3.888330221	6.128715277	9.3915648	1.908942373
1.804431362	3.771952699	6.182661176	5.430420896	0.857407596
3.021680207	2.527715115	6.96836608	6.561907399	1.147736084
1.562568698	1.562572685	7.189207496	6.509281679	0.982099591
3.70125954	0	7.943538442	6.721868657	0
0.064287035	0.064147572	4.234921737	5.090059307	1.83130155
3.445278	2.361454289	5.272889487	5.033440873	0.162202438
2.121218524	1.458050354	5.252950008	8.926116192	0.109382698
0.677690783	2.806427514	5.604132532	5.293982948	1.192183297
1.646850627	0.741531187	3.046151394	7.850151726	0
4.660845769	2.185250389	1.305983761	9.381368751	0.506974652
3.850085152	3.145360833	5.481664701	10.25105302	0.023950863
0.966948449	3.121095664	6.516023342	7.632452323	0.967833375
2.006467557	1.5909328	8.33441071	5.531438585	1.0047769
4.639968973	2.88329562	5.033530342	5.86054561	0.051860163
4.341093892	1.442430668	4.56613369	7.198689176	2.169497521
2.477372369	2.008747533	6.268761503	6.000588075	0
3.270615885	0.965558855	5.988478362	6.776707221	0
3.638459883	1.851572704	7.48595312	5.802395431	1.21443675
2.015450477	0.335127452	5.779734684	6.036518803	0.246956258
2.781872552	1.405965096	4.286870299	4.462712539	0.857617351
3.19538386	1.88823802	5.695181436	9.591981715	0
0.861148096	1.414138431	6.721665272	5.877173834	1.420917159
2.851992233	2.548416769	7.557653282	4.307452119	0.416153618
2.438258425	5.17311896	5.65870378	6.528410872	1.082965289
0.004466077	3.345318606	6.134036889	4.548504728	0.003710532
1.69667957	2.120484715	8.996298398	5.14476338	0.02175634
3.137964879	0.794184429	6.961521867	6.350004602	2.045512342
2.032137363	1.126921533	6.879395645	5.61936825	1.512912062
1.997287981	1.997102292	5.378180446	5.040634271	0.083034646
5.124744985	4.527463074	8.126204273	2.814646033	1.347959358
2.487608179	2.0183885	7.307616344	6.37792921	1.330172773
4.606141646	2.292641664	6.191462834	5.769337802	2.108826457
5.903329957	2.676561611	6.406803345	5.660404836	0

1.199472443	3.451282997	6.008159638	6.575788401	0.047055814
1.736540343	4.074950797	8.187406956	4.344556977	1.096335732
1.366198606	3.071536809	3.079002303	11.42777316	1.98459896
1.504199099	4.41261715	6.57620356	2.859987725	0
0.073525991	0.073363401	5.238061645	6.671011068	1.240283994
1.728375387	1.119325776	7.519468611	6.397122542	0.028032271
2.696130031	1.079926485	5.854151934	11.10997415	0.018736421
5.465049803	2.806253345	4.375403094	4.521155599	1.368960434
0.409743899	0.408217447	7.572125316	7.813098214	0.293624865
3.255774041	2.417477914	6.56606739	10.41198434	0.078514251
3.294409047	1.086295394	5.179364847	4.941768052	2.694883598
0.09094684	0.090738537	6.860377549	5.988778061	0.073009586
0.021518849	1.075758575	6.050702081	5.02535286	0.017755313
4.503947442	2.387310132	6.388243103	6.163768164	0.167999854
1.05792581	2.400719396	5.045181081	4.734291978	0.013563005
4.655611497	2.28176387	4.760901833	5.625371752	0
4.772849671	1.264966021	5.354715124	3.176015584	0.062714004
1.781906097	0.63252221	7.276394623	6.689635965	2.618075355
0.982497883	0.982496661	5.526413918	6.381011502	0.982981647
8.202345383	1.308043882	2.728975168	7.639662986	0.073062249
0.138865546	1.458340836	5.058168498	7.842093014	0.109453431
0	1.636105427	9.736526826	6.262108789	1.013977304
1.196684005	1.572732611	6.43204292	6.863170272	4.698899365
4.505523109	3.660760483	7.314049701	7.035578599	0
4.020692542	2.642538542	3.71082352	12.11320465	0.124335964
1.852170269	1.449225597	7.005715047	6.516704204	1.45486199
1.362806741	5.466628454	4.332965837	4.780362963	1.344359406
3.809018521	6.190998223	4.366660588	6.261165535	0.058433341
1.067308623	1.067310635	6.256697284	5.532502995	0.015768054
3.378939447	2.639382761	6.63215357	6.062072852	1.037673504
0	1.90108777	6.016393582	5.197146641	7.271339236
2.689424994	0.919172467	6.076619488	7.730444825	0
0	0	4.587466273	6.434155863	0
0.02851622	1.70528878	3.469518049	11.28123581	0.023462731
0.978781285	2.292695657	5.095292729	8.344737079	0
3.306881409	0.110400172	5.191867071	7.18770937	1.351649828
2.734734836	0.092899519	6.407527539	8.003414552	1.299794244
1.039085363	0.011011764	4.230425759	9.944110255	0.00914209
0	1.674249131	8.344039622	3.98268395	0
2.412663112	2.412906762	5.747803796	5.469313884	0.633434506
1.720155409	2.14568075	3.752838707	5.730675777	1.108466724
4.647689275	5.447731191	8.788665624	6.21558029	5.380490081
0.078059693	1.900834086	6.764982919	5.532398177	0.062978993
2.41691928	1.513344352	6.935784786	6.970277334	0
2.368229868	2.368009901	7.397709863	6.025029073	0.068759735
2.214014279	2.544287042	6.165316976	5.872476172	1.159804028
2.34525826	2.831715992	5.275033053	5.934195766	1.604579947
0.901232346	0	5.837227423	6.211804549	0
2.303542583	2.651646262	6.929061044	6.743546837	1.566249224
1.657782312	1.272987042	6.335582085	6.254086756	2.641071103
4.646794797	0.870800513	3.776701282	5.567325829	0
4.266910985	1.804696505	4.738648117	5.838670836	3.038585958
1.51984579	2.248025762	8.732375129	6.021467009	0.948013065
0.978420728	0	4.214030113	7.564726355	0.979012268
2.652010048	2.652098811	6.565752285	5.658308119	3.552451846
1.881986134	4.702027508	6.362917298	4.237212457	2.873740713
2.993242436	2.171468099	5.375926723	5.907703965	0.031186779
2.867947527	3.276873113	6.511037869	5.77567894	1.746583921

hsa-miR-766-5p	hsa-miR-551b-3p	hsa-miR-539-3p	hsa-miR-20b-3p	hsa-miR-1185-1-3p
1.29611109	5.094944452	2.776323059	2.413437809	1.316992773
0.041210131	5.902055703	0.067999736	2.278442574	0.048969168
0.020390102	1.719235259	1.719978702	1.715529988	1.105087865
1.052397822	3.896911239	0.016974277	2.073826424	0.01259667
1.317865376	1.36847492	2.470826799	2.00459763	1.340660998
0.02934293	0.050058869	0.047617885	0.04106588	0.034700412
0.08702374	2.963517618	0.153397518	0.128262935	0.105321079
0.012821278	4.632482738	2.683426339	0.017681599	1.06614402
1.507169805	5.373301896	2.219957673	3.217953029	1.502178667
1.738917273	9.603347486	1.76814701	1.149762154	0.032706823
0	5.985747046	0	1.423891054	0
1.547842995	1.665174051	1.670000584	0.177054974	0.143179146
0.061542604	1.339970414	1.343731608	1.334783572	0.073730537
0.983521624	2.296590192	3.142773382	1.563273163	0
0.101413145	6.472268448	2.256996145	1.555024144	1.51636719
2.145820459	0.042226576	1.137103815	1.134819945	0.02942349
1.446129481	1.035410625	2.594418683	1.682597131	1.051308719
0	0.761308993	2.721899954	0.357635986	0.769949746
0.045967035	3.690201623	2.326611508	1.249454639	1.236064619
2.23047251	8.990483389	0.234340689	0.191526555	0.154195888
0.094502223	1.530941332	0.168429161	0.140228942	0.114710227
0.144414597	0.299566187	1.8391105	0.22419845	0.178728429
0.016795086	1.09091155	1.696200391	1.090725697	0.019760395
2.445404618	5.749912906	0.140834032	1.439990352	0.097357607
0.07996646	6.360233691	0.139506129	2.865580027	1.408552869
0	4.47446561	1.577941058	1.206913571	1.221584251
1.976611642	8.048115171	0	2.778455956	0.98001202
0.035850119	0.061796818	1.822169588	1.813298143	0.042507706
0.048640517	0.085588311	1.906702766	0.069257873	0.057972078
0.023461057	6.929280767	1.740284664	0.032662403	0.027678365
0.516847555	1.988964929	5.498452098	0.941435839	3.592950527
0.999729827	0.999703714	2.321513349	0.999703081	0
1.045320226	2.68092579	1.632549373	1.887649942	0
2.026851668	4.297062506	1.671299819	1.987126489	1.302843564
0.054698868	5.312343235	1.946762492	0.078308413	0.065351796
1.644017111	0.284348943	0.263915365	0.213946129	0.171079933
0.070364377	0.1283114	4.728076141	0.10216364	3.028504892
0	4.088953114	6.368702988	0	4.137443062
0.047730185	1.261937515	1.265013742	0.067906268	0.05686624
0.012929168	2.684808363	3.102358831	3.266529945	0.015187931
0.056583723	0.10086208	0.095430216	1.307573577	1.290130381
0.03812646	1.208312007	0.062638566	0.053778745	1.196020501
0.070139709	0.127853921	2.495993978	0.101816853	0.084319507
1.167201457	2.579407697	1.813677139	1.805209146	0.040962623
2.508970946	0.936137962	6.792044386	0.935830657	4.721971284
0	6.19817319	0.924316709	0.924939586	0.927727441
0.087679191	5.236815173	0.154702047	1.478864639	0.106141861
0.101402246	0.194897829	1.576715717	2.664771783	1.516312594
0.063624672	0.114732854	2.450441948	1.98657601	0.076288505
0	4.421047331	6.624154267	0	3.139027275
2.331884531	2.820162852	2.011626	1.00870799	0.001889542
0	6.929594652	1.522289735	2.0792266	0
0.031493772	4.723152905	2.824553794	0.044160688	1.162070655
0.012698611	5.394486292	3.424178279	0.017510522	0.01491569
0.008583235	0.014174308	1.046910524	1.046304533	0.010064853
0.787407068	4.934516185	6.233023935	1.304538166	3.141295147

0.615592435	2.505522368	2.652113959	0	1.092258661
0	0	0.864382318	0.865212291	0.869826727
0	6.290545018	0.70360888	0	0
0	5.394371216	5.336070309	0.668042396	3.218303042
0.020833675	4.344493348	0.033411138	1.112614577	1.107367682
2.123285575	3.541463567	1.77547939	0	2.108696419
0	3.852847811	1.858784381	0	0
0.005516042	1.02971904	1.030111696	1.620476435	1.618448174
0	4.129207228	1.553326165	1.554149434	0
1.184517499	1.836627885	1.211251178	2.25853112	1.196390515
1.781494814	0.061301796	2.253423018	1.811588271	0.042180864
0	3.148140889	3.300187426	0	0
0	6.571987505	0	3.145758256	0.983556944
2.014603634	3.174622765	2.748812614	1.050414956	1.067645026
0	5.952508717	2.224619582	0.930934658	0
1.1593792	0.056493951	1.181702791	0.046229371	0.038995444
0.10501928	1.593435374	2.28110132	2.240062888	0.128004199
2.007313436	1.592975561	2.331076217	0.001660413	0.001422138
0.168656176	5.332367225	0.336729392	0.267650146	0.210658708
3.495340953	0.009335444	1.622606242	2.039182865	0.006650806
0.153983529	1.895967171	0.300429116	0.241136819	0.191267723
1.043173423	0.014599215	1.64352856	2.060857272	0.010363572
0.840231447	3.050555893	1.370346324	0	0.83297611
1.249127808	2.706497901	1.288395729	2.949961957	0.061878193
1.507626422	3.885498687	0.927469773	0.928075001	0
0.866637181	1.407124526	1.804065583	2.791507649	0.860391847
0.027855627	0.047409696	3.218493769	0.038932696	0.032921663
1.979576799	0	3.142603829	1.97655305	0
0.879326801	0	3.25563477	1.828243843	0.873593088
0.039885961	7.518312799	1.848839971	1.216248991	1.205015207
0.117831288	0.233058601	0.217470663	1.646478094	0.144340563
0.081391966	1.454127953	0.142289429	0.119354535	0.098285622
1.219680574	1.18503611	2.098393813	0	0.686843908
1.691793662	0.745669997	0	0.742735452	0
0.526937419	0.958458893	0	4.156189515	0
0.018580444	1.100651727	0.029704804	2.453510148	1.095783925
0.970171037	1.544953657	1.544664486	0	0.968605979
2.327487123	3.329335255	4.177451112	1.004856821	2.005835389
2.2283799	3.640726408	0.065319135	1.215079628	0.047111662
1.867452729	7.242586222	1.442356871	0	0
0	5.672690544	2.475986901	0	0.788331385
1.961615935	4.033486433	1.542968487	0	0.96728473
1.195085335	7.296922283	3.487405503	1.219171746	0.04801695
0.173063236	0.378575427	2.743346234	0.27574277	0.21652137
0	0	0	2.124293266	0
0	2.463739608	0.91615948	0.916824799	0
2.157651844	0	2.383076252	2.133475722	0
0.43648136	0.423318318	2.652636597	0.830783087	0.848559895
2.092509185	7.030016529	1.688612026	3.925715829	1.679308132
1.014242271	0.004761522	2.607423598	2.828952925	0.003402872
1.082296225	1.696231043	4.045240442	1.09127718	2.107775173
0	5.20885652	1.720688401	0	0.801766178
2.109487357	5.924046743	2.422236586	1.132171229	1.525801047
1.299329844	0.112618043	2.443002615	0.090199362	0.074981829
0.798356901	1.329343575	3.588912022	1.925264792	1.796701117
0	0	2.839922746	1.325509911	1.33691217
3.139353878	5.700130456	2.474460172	2.309893448	0.675822974
2.892649469	3.321057356	2.196664841	4.392855466	0

0.036082358	1.822661637	2.256980403	2.244498542	0.042787097
0	0	3.140341968	0	3.217387729
0.065482299	4.997070636	0.111859436	0.094658639	0.078574236
0	1.237859866	2.650031722	1.517935243	0.472578719
1.21816619	7.011865034	1.884556598	4.239625184	0.053906175
1.706334417	3.879504902	2.155155384	1.117322305	1.111827709
0.014574002	1.078815189	1.079837675	1.678482059	0.017131625
0.56269443	1.005974725	0.999946213	2.093316532	0
0.20198952	5.035286771	2.211589426	0.330362989	2.012735415
1.90585556	0.106144957	1.977098168	1.322387325	1.30385418
1.673409345	1.688538855	1.086202305	2.107859676	0.018497712
1.265222343	1.304423696	0.09304816	0.079173741	1.283474227
1.067378888	7.207069231	2.691022765	1.673653977	1.071275963
0.121730917	0.242435551	1.699199709	1.668266346	0.14934364
2.062901273	0.017522507	1.057857073	0.014558866	0.012414043
0.973736981	5.345619171	0	0	0
2.272374056	6.446067459	1.26494913	0.067889344	0.056852387
2.103889329	0	2.415737685	2.042057843	0.641920826
2.564767969	5.21997393	1.976853887	0.982705889	0
1.265402877	4.540521573	1.308108823	0.079231563	0.066102078
1.386801513	0.151292395	2.123975134	0.11943315	1.4159947
1.714494046	0	4.314050811	2.279206959	1.679971996
0	7.086675105	1.87062244	2.347818859	0
1.090610974	0.582060169	2.870651507	1.412917059	2.191502164
0.091879857	0.173731838	1.520224619	2.160146831	0.111411817
0	5.573314459	0.889569665	0	0
0.06489123	7.71165927	0.110755267	0.093754378	0.077846591
2.58223463	1.878606068	0.073867762	0.063209112	0.053015539
0.012283776	0.020402173	1.666348135	0.016932253	1.659191379
1.62524959	3.518016239	3.865854246	0.00975227	1.628062702
2.215303041	7.294798972	0.709780929	1.22662931	0
0.926599548	0	0	1.48832419	0
0.968024197	5.19112365	0	0.964980249	0
0.018206294	0.030519613	1.705535476	0.025228455	1.093859741
0.980880231	0	0.978813516	2.293862351	0
1.316586148	1.366902624	2.023306713	0.095856321	0.079537343
0.056498338	1.311344964	1.314870556	0.0810153	0.067550524
0.007146336	0.011775216	1.039035757	0.009805869	1.036893575
2.000322875	1.039746573	2.600621452	0	1.422231468
1.159911982	7.58624776	0.630545699	1.494004708	1.139640488
1.099490571	7.848614049	0.032786924	1.110561915	0.02410263
0.520873314	4.876007078	4.513340665	6.418100977	0.964835473
0.047914189	4.643423116	2.946798398	1.260100014	2.297462347
0	2.272373405	2.414009268	0.907615049	2.348660118
1.25060533	6.218689711	2.97820078	1.283360283	0.062260703
1.145997252	3.238797558	3.040577065	1.163102595	1.155079134
0.00311862	6.934129896	2.345359736	2.607794722	1.603925401
0	1.868412715	1.463846951	0.902141271	0
0	3.766955955	2.091214638	1.557760846	1.195608338
0.767311393	6.624947538	2.212198113	1.665948516	0.757643216
1.556426221	4.537174573	0.87206071	2.647186463	0
0.033296017	4.525172906	0.054332647	0.04676298	0.039438458
0	1.929303996	3.725386429	2.925457378	0.949232537
0	7.630474275	0	3.429031262	0
0	7.425174279	1.455003099	0.89503864	0
2.415520464	6.801588092	2.525529426	2.358822297	1.225295864
0.024105152	0.040784149	0.038833557	0.033578371	0.028445695
0	6.222678527	1.341511073	0	0

hsa-miR-483-3p	hsa-miR-135b-5p	hsa-miR-4739	hsa-miR-206	hsa-miR-199b-5p
1.335332556	2.778469911	1.327177491	1.336215604	6.247254303
13.91187476	1.227752958	1.217936394	1.223256735	3.324412044
3.486642899	3.624741975	1.711631562	2.139229475	4.564535244
11.62829695	2.402504241	4.247943685	0.014722652	6.11577765
5.724132783	4.857683142	2.425011886	1.361822717	9.661920456
6.994551727	0.048462791	2.519995041	3.538224177	1.158077256
2.957298298	3.23724125	0.116050334	2.129550123	2.614452445
4.107325807	1.070016108	0.016311155	2.087954365	8.639650946
3.347453867	3.587391481	0.928878161	2.705961388	8.079116024
2.526300962	0.045598296	0.035543762	0.038532967	0.047774582
1.42512145	7.423373671	0	0.583008445	9.393607842
2.82089835	3.455526525	0.15888443	0.176209275	7.404911729
3.478739663	0.106624188	0.080757606	0.088287413	2.775529795
11.83967159	7.758947949	2.298576129	2.56027755	5.614380912
3.061575903	5.560341759	1.535994921	0.150733586	6.767845526
14.07719746	3.660907345	0.031955405	0.034619667	5.118933719
0.575096846	3.556799004	0	0	2.843671859
6.652748844	3.474525995	0	0	7.099353565
7.334976471	2.662654416	1.866863528	0.065040397	7.035158222
4.906596781	0.240234694	3.38113154	1.688253752	1.709903243
2.662730395	2.212608952	1.499525766	3.654301163	1.525297221
1.818022668	0.28506251	1.76264326	0.22304596	1.826360214
3.457872206	1.091865587	0.021420775	2.114932887	3.596921575
2.110233005	0.143851722	0.107114813	0.11767323	3.42609347
4.735845882	2.115329292	1.422780402	1.435683677	6.59676897
1.882818368	0	3.54121808	0	2.830208426
13.65060004	0	0.979561761	0.979197043	3.552682225
7.14084516	0.05977787	0.046281948	2.24257454	3.455888902
4.649606193	0.082655865	2.91291968	0.068986022	3.85113496
5.956717097	0.038417481	0.030049992	0.032543826	1.126075954
3.269026598	7.889633874	0	1.28811552	8.867742591
10.03155617	0.999700557	2.806960553	2.584558487	2.806919178
2.256956893	1.002158502	0.544366992	2.276876852	9.396711877
2.630186049	2.941217065	0	0	8.006168611
3.227879562	0.093796524	1.28925949	0.077995363	10.05142307
1.781990318	0.270865881	2.400705275	0.212863184	2.506206013
13.69423634	1.39405408	1.372055926	0.101735799	6.1418305
9.221372529	0	2.551295878	0	2.060308603
4.297152015	0.080999007	1.252414745	3.514441383	1.25923329
5.755827682	0.020851552	1.068362341	3.266346667	5.635413604
1.305930238	4.361637305	3.709098557	1.307240653	1.955304713
4.314288857	0.063787065	2.577352257	0.05357434	1.20618192
4.146045905	2.049822387	2.446941393	2.026118407	4.374765233
13.08283077	3.271674186	1.79814211	1.187070693	10.06898721
11.07827069	4.220014528	1.919173361	0.935871163	9.786475206
0.926575323	1.898888908	0.926201733	1.494460226	2.476169218
2.614844629	2.16688378	3.904409003	0.12873617	3.479045929
3.57857443	0.186804605	0.136455534	0.150715922	2.253069911
6.821463454	3.06519976	1.336440487	0.091442902	3.496825407
9.963045379	0	0	0	4.027145649
2.011457013	1.008785374	1.008542827	1.595370676	7.002923721
5.479353335	1.817028245	0	2.924909642	6.796512826
2.221872962	4.164031686	1.166549035	1.170369961	0.054707202
2.682125712	0.020472931	2.408927333	1.06849797	3.913208708
6.341784884	0.013756557	1.638615214	3.649994749	2.651103636
1.306397739	5.637862925	1.310165019	0.771644059	5.695573718

4.79617404	3.30405736	1.450842742	0	6.18131729
5.796412838	0.86478295	0	0	0.86746031
2.538250058	8.041408684	0	0	3.591383711
12.91275752	1.539596067	0	0	1.545193198
6.520254275	1.114145701	0.026640112	0.028832614	3.490848687
3.064236972	0.834469075	0	0.834902506	6.708279207
8.944351411	0	2.134870979	1.571111968	5.277804807
5.405435078	1.030034409	0.006984879	5.290382527	2.363197594
3.660531831	7.521068207	1.967904729	0.974355258	6.066457618
2.603200779	1.837866272	0.049387804	1.827735644	6.303031431
1.815774096	0.059301053	0.045923202	0.049881626	3.28073425
6.261855739	1.567263844	0	3.439016713	4.436465192
2.560445622	0.982738141	0.983184946	0	3.144008625
1.935412637	1.047212599	0	1.05070145	8.570498455
3.065039653	2.896001899	0	0.930977883	3.698271394
3.258934398	0.054668323	2.810194654	0.046056473	0.057331979
7.038373885	2.743147319	0.141644725	2.238844179	3.374071553
1.592856679	3.009751704	0.001536161	0.001654804	6.160427645
7.96246722	0.346584927	0.236665795	0.266185736	3.852346835
14.48626485	6.370435793	1.620513071	1.621479439	7.901699814
4.180323928	0.308782172	1.813022582	0.239866027	3.45355377
2.387584759	1.643517498	0.01121395	1.641723366	2.876901781
1.769122625	1.765123568	0	0.827467657	1.373843726
9.020111561	2.977952586	1.27420221	0.073748234	5.522359569
11.99302323	8.434170694	0	0.92811979	8.693876439
7.286532395	1.80399618	0	0.855591444	8.26694814
3.812143308	1.769403195	3.000840997	0.038789381	5.664377823
2.296710601	2.296407214	0.982142211	0.981822338	7.264693205
11.44020103	0	0	0.869160062	4.737844122
1.843761906	0.066904311	0.051624481	0.056133823	1.845691289
6.551485403	1.675701959	0.160207723	0.177719709	4.245389207
3.428667539	0.145346518	1.430309035	2.873664614	2.574707308
2.936895144	1.554869922	1.193067701	1.564553272	7.262935208
3.169392555	1.646537273	3.083366513	0	1.650677465
4.050115045	3.960897139	3.3639035	0.50389116	5.989797144
5.651809916	2.133198645	2.448546748	5.047670115	4.742779525
8.870626262	2.537685744	1.95907991	0	5.853332366
5.815250983	1.004899178	0.001138094	3.006759424	9.002323243
1.212905295	1.219103062	3.862295275	2.59818832	5.141109618
0.887541506	1.844326453	0.886713564	0	1.444614924
11.52383166	2.007533874	2.027066491	0.781823983	7.684266823
6.403364313	0	0.966559124	0	2.274374312
2.28438318	1.223318548	1.213766666	0.056914657	1.218816617
2.739197542	2.023883822	3.876984715	0.274217229	7.184986281
2.374956777	0.855314551	3.495678057	0.855803236	2.117848567
2.464908088	2.204309088	3.055445496	0	5.148590988
13.01244336	0.861827814	0.864353523	0	7.738665795
11.6169343	5.09140051	0.416674313	3.363206566	8.715026381
4.13074392	1.085551589	0.0199433	1.084446353	6.864071356
5.234702068	1.015821251	1.015377013	1.01566591	4.272707418
10.13237053	2.121183033	0.021552029	1.693167151	8.207983802
2.686433982	2.281234378	1.339756582	0	4.65600066
0.647253576	0	1.514192857	0	7.168332277
6.047820052	0.108556417	1.965579988	0.089830213	3.289553017
8.681930643	8.374212455	0	2.772265125	8.241914199
5.660884125	2.98757823	0	1.7186572	7.89268166
3.350808829	2.079843822	0.671048376	0	8.567301336
0.913292591	0	0	0	1.476978814

6.458481969	4.465340569	2.235410306	2.57382485	1.194952326
9.396934652	1.086104879	0	0.604699322	7.968149797
2.010657687	0.114127876	1.982802358	0.094267878	5.064123838
6.263199885	2.647763233	0	0	6.485121676
0.081042123	1.884834612	1.239512621	1.245509501	2.929298925
3.878974457	1.728677515	0.027765274	0.030056687	9.06199572
3.115611303	0.023559983	0.018561678	1.078636888	2.696982264
13.63937021	1.001214873	1.660152603	0	6.582370262
3.44286085	0.439404659	0.28926585	0.328400366	2.202576291
1.320925967	1.330301372	0.077682971	3.225979328	2.759060739
3.124697768	0.025469045	1.0831679	2.433374984	1.084254301
6.71566251	2.393191414	0.072244967	2.702809079	1.301259817
3.814665011	1.075488615	1.671097405	0.019005626	7.051223134
5.062533538	0.231607932	2.306522315	0.184241472	7.442955096
2.400470716	2.07599483	0.013438012	0.014508151	1.654069948
3.954652232	0	0	0.971238286	0
6.898007086	0.080978273	0.062070719	0.06762361	1.897050249
3.288195817	0.63469288	2.05642726	1.127548463	7.573764842
3.558255009	1.976829116	0	0.982717713	8.838775333
4.849210621	1.951290592	3.839163135	2.370244982	4.77887789
5.477623261	3.196333145	1.430572793	0.118916642	6.888269086
11.06838193	1.353805177	1.664135088	1.363138308	6.620525696
8.856657926	1.197603961	0	0	6.702529477
11.44615081	1.40369697	0	0.575681823	8.192831818
3.708944693	2.195021034	0.122902107	2.159134847	2.189232797
5.104205498	2.833736817	0.892090741	0.890405526	7.854703498
5.445759605	1.362475183	1.979320702	0.093368024	4.333435495
13.40045226	0.075254867	1.858394374	0.062964071	0.079092437
2.903392802	0.019792318	1.66159669	1.066256035	4.563938419
6.570070087	1.038724449	2.8578113	2.048932701	7.07356215
0.718017955	1.222268549	1.233192646	0	4.891064664
1.893915497	0	0	2.213792057	2.688624207
3.405557765	0.964733387	0	0.965003491	1.542420476
1.096855422	0.029586563	1.096271137	0.025138386	1.097624736
7.391672429	0	1.560451966	0	0.97929594
7.245161142	1.370380873	2.423333515	2.00224517	5.681120006
4.157015972	0.09714419	0.07390159	1.941854093	3.013171041
4.060053941	0.011430075	1.037782524	1.630893064	1.038149567
12.69607967	2.729686159	1.046151936	0	8.303803504
0	1.484562514	0	1.123641216	8.091956243
1.108915379	0.033349537	1.108165221	0.02829937	1.718593506
10.85685865	3.558192947	0	2.866864143	7.753040657
6.747273319	3.374074732	0.06233264	0.067912264	5.356249394
5.066078433	4.477026514	0.915515385	0.468923325	8.660426308
3.407092431	1.289697947	0.068045731	0.07421536	2.707092773
3.237228717	2.215383825	0.038755385	4.312092814	8.39956278
3.610782071	6.699143258	1.604513555	1.605037108	6.312761796
2.852646064	1.463954772	0.903730808	0	3.170463453
6.92105092	2.647613674	0	1.180631475	7.021602333
3.295405527	2.4253334	0	0.750555746	8.191511458
0	2.717241731	4.994253945	0	4.535565644
2.567415438	0.055311067	0.042914859	1.180168336	3.85664072
7.160337475	0	1.523000561	0.947247985	6.795288594
12.16250842	0	0.979062039	2.555823093	2.968107413
5.954310699	0.894588135	0	0.895100235	7.291642545
2.970110671	1.880895432	0	0.697848744	7.029482894
1.741308429	0.039509168	0.030887643	1.130254315	0.041368452
1.347057026	2.69888648	0	0	7.972991133

hsa-miR-487a-3p	hsa-miR-499a-3p	hsa-miR-4784	hsa-miR-380-3p	hsa-miR-506-3p
3.268973949	0.098396896	0.068978446	0.090393448	0.076082406
0.06475726	1.227561941	0.045722726	0.059051786	1.831885786
1.719163678	4.161817906	1.704168627	1.110508772	0.02459118
1.058749846	0.016060654	1.648181268	1.058066961	2.069570802
1.371213234	0.106418874	0.074261589	1.363621897	0.081998826
0.045439284	2.206272404	8.032831229	0.041583586	3.004442649
0.144857346	0.142703292	1.431348743	0.130190463	0.108262362
2.682017889	0.019232699	0.01413001	1.667606045	0.015407831
0.926899736	0	0	1.905590887	0
0.042770117	1.152170441	0.030608704	0.039160233	1.143631262
1.056100692	0	1.086513236	0	0.590267316
2.348421227	0.198964266	0.131992615	0.179952388	0.147461125
1.342732015	1.342087002	0.068605915	0.089884274	0.075665694
3.673205651	0	1.564964492	2.782134295	0.982528317
0.171952946	0.16927484	2.581332739	0.153798549	0.126998701
1.137078103	1.136929224	1.125498158	0.035178283	1.12939132
2.601700816	0	0	3.597251676	0
2.732914654	0	0.779183325	2.210765179	0.364543178
1.886473454	2.321123614	1.230246419	1.250280127	0.056107085
0.219554385	1.713085013	0.141945438	0.194736209	0.158895042
2.653984032	0.156398757	1.467513928	1.519003432	2.134341306
0.258997647	1.826183368	0.164013295	1.800213929	0.184399407
0.0256121	1.695294464	0.018526981	0.023525164	0.020221179
0.133157396	0.131217943	0.09029909	0.119926384	0.100035486
1.448276131	0.130001929	0.089522852	0.118837545	0.099159834
0.693338356	1.880133222	0.706982893	0	0
0	0	0.980401614	0	0.979878101
0.055962577	1.197625617	1.180023437	1.194841784	0.043551741
1.9039138	2.339477773	0.054060664	0.070185512	0.059444495
0.036069833	1.738902878	0.025921361	1.127223447	0.028335794
5.871836285	0.49284941	0.507944627	4.338678647	0
0.99969936	0.999699547	0.999720876	0	3.906498452
1.351191629	1.874007325	1.936930377	0	0
1.285876263	0	0.770867085	0	0
0.087407788	1.303365571	0.060879982	0.079376997	0.067037638
0.246556216	0.242241482	1.67775459	0.21766149	0.176441637
3.700568641	5.825204154	1.350273558	4.59750563	1.363510255
6.511462071	0.814614592	0	4.47380282	0.820864777
0.075612187	0.074624918	0.053037616	0.068813247	2.629369743
1.670158705	1.669958954	0.014249279	1.069977251	0.015538277
0.090633107	1.314012199	1.282666734	1.308670308	0.069408565
1.210612409	0.058929265	1.191346637	0.054475839	0.046366538
1.391802268	1.39098401	0.078345007	0.103272624	0.086580319
2.243436885	0.053198545	1.17362512	1.187813928	1.179262248
6.955390301	0	0.939400301	4.712612708	0
3.34589373	0	0.929060376	0.9247897	0
1.492895571	0.143893852	0.098329975	0.131252355	0.109110663
0.171932053	0.169254365	0.114073449	1.557580549	0.126984419
1.354587551	2.778306314	0.070961108	0.093107275	1.328945026
5.850638168	0	1.43146545	4.43949383	0
1.008819461	1.008813724	0.001776175	1.595419434	0.001931743
1.819761875	0	0.66925724	0.655938594	0
1.791913117	2.551345161	0.034862625	1.171031194	2.20053349
3.686129742	0.01904595	0.013994412	1.666818768	0.015259533
0.012959352	3.87596484	0.009449796	0.01193151	0.010294185
4.673816032	4.026650039	1.322102028	4.815512365	0

3.225863892	0	1.100445858	0.593264827	0
0	2.132545502	0	0	0
1.213793421	0	1.628053252	0.703572044	0
5.67022997	0	1.578936284	4.374408014	1.185392209
1.1144195	2.14695756	0.023004106	0.029291753	2.133860893
0	0	1.799287938	0.834539339	0.839326663
1.861345746	0	0.695629592	0	0
1.621355991	6.437255125	1.027899757	0.00764609	1.028684084
1.553449738	0	2.290880798	0	0
0.059802125	1.210730579	2.231463485	0.05458292	0.046456113
0.055519776	1.817947524	1.178670855	0.050714279	1.184498932
2.564549451	0.985462357	1.569074422	0.985596169	0
0	0.98267522	0	0	0
1.931032136	0.573654664	1.076025022	0	0
0	0	0	0	0
0.051214567	1.181342977	0.036461407	1.178860357	0.039945764
0.178897311	0.176078828	0.118238501	0.159813034	0.131731365
3.469181993	0.001800175	1.006158725	0.001679507	0.001453863
0.312386255	0.306394135	0.192545038	1.938652831	0.217681239
2.040443438	1.031009463	0.006247462	1.030693207	0.006801095
1.88803337	1.88404389	1.750416123	0.245501081	0.197457921
0.013346694	1.643102887	1.044663254	1.047793452	0.010599881
0.826031785	0.826052409	0	0	0
1.28778098	1.287303799	0.057671867	0.075043273	0.063462856
1.903887752	0	0.932038738	0	0
1.406035148	0	0.862779632	0	0
1.153343029	0.042532194	0.030808823	1.76362245	1.144561651
2.29673406	1.975913955	1.564839593	1.976452728	1.564147487
2.137544776	0.867955115	0.875788143	0.868851869	0
0.062573826	0.061778293	1.20008985	0.057083621	0.048546295
0.204082795	0.200734529	2.228637019	0.181504458	0.148665953
0.134514232	1.455421309	0.091148386	0.121118889	0.10099386
0.677211725	1.556692944	0	0	0
1.263502589	1.952829802	1.287293122	0	0
0	4.331946733	0	0	0
1.101968515	0.028061079	0.020505152	0.026069123	1.096510801
2.276576902	1.956658117	2.544710914	0.967253407	0
2.328633354	0.001333473	0.000990595	2.328430631	1.004689209
0.06222104	0.061430571	1.199025113	1.215759319	1.205639102
0	0.883854942	0.890905678	0	0.888275139
2.479529595	0	0.791652164	0.781394945	0
1.54312725	1.543206464	0.967914033	0.965877837	1.545905958
1.850273863	0.062648075	0.04483963	1.219868232	0.049210658
2.716273992	0.316180711	0.197761044	0.280988694	0.223802489
0	1.406557821	0	0	0
0	0	0.921346005	0	0
1.414466619	1.414716735	0	0.862007838	0.866169009
2.308055352	0	0	1.657290141	0
1.688029569	1.687781136	0.017256997	0.021895232	0.01883002
1.01588117	2.343458947	0.003198008	2.020194934	0.003479157
3.593045427	1.092591596	0.018639746	2.116342867	0.02034474
0.794006881	0	0.804928121	0.795125535	1.740574944
2.737092697	0	1.535658401	1.131274047	0
0.100961864	0.09957637	0.069758217	1.341643342	0.076954855
3.553885837	0.753168841	0.782685621	2.855719795	0.367001802
2.489719363	0.78677746	0	0.787899531	0
0.666096361	0	0	1.546846123	0
0.910434562	0	0	0.911149831	0

0.056341249	1.198920543	0.039985256	0.051456862	1.801829709
2.811583539	1.084926126	0.618241146	2.549944275	0
0.10606423	8.941141461	2.716831208	0.095993296	0.080656897
1.505646485	0	1.278476925	1.51600805	0
0.071529656	1.881118046	1.844841244	0.065146501	0.055263481
1.119220529	0.032895578	0.023967388	1.11764477	0.026189061
1.680971571	1.079805021	0.016068358	0.020372026	0.017528638
1.350104123	0	1.689489751	0	1.018536212
2.188538335	3.364337499	1.973591612	3.28769693	0.264513131
1.97328441	4.455514678	0.066053934	0.08640251	1.306654675
0.023954813	2.111184985	0.017346256	0.022009705	0.018927771
3.221270271	0.08720778	0.061529161	0.080256037	0.067761582
1.075712448	1.07564646	1.069755629	1.673999319	0.016613203
1.693140454	0.208391075	0.13756511	0.188207822	0.153857569
1.05789882	0.015825935	0.011651478	1.0572271	0.012698508
0	0.970897711	0.972888482	0	0
0.075593043	0.074606062	0.053024799	0.068796065	0.058292928
2.237926657	0	0	1.126371288	0
1.56373725	0.982507705	0	1.564142668	0
0.088457366	1.306834132	0.061572522	0.080314776	2.943546694
0.13460532	2.115270825	0.091205355	1.446241534	0.101058157
4.328543725	0	0	2.437950548	0.549798734
0.689452357	1.197287662	0	0	0
1.40407325	1.046651476	0	1.05046755	1.06577503
1.517308375	0.151568436	1.454848474	1.504366776	4.756137186
3.005011503	0	0	0.89013657	0.893588634
0.105028705	1.361098625	0.072394891	0.095073936	1.968428878
1.246666316	0.069396385	1.223253203	0.06404508	3.883745151
0.018629992	1.06721642	0.013535906	1.066476307	0.014758148
1.631843161	0.010590621	1.03593627	1.630866227	0.008518362
1.221782619	0	1.247434812	0.709789499	0
0.919150272	0	0.924337661	0	0
0.964576817	0	1.958745539	0	0
0.027811936	1.099807452	0.020090457	0.025535301	0.021934851
0.978780685	0.978792475	0	0	0
1.369643448	2.459758719	0.073950077	0.097211047	1.342489129
1.955135481	3.227570624	0.062909096	0.082126916	1.292334057
1.039071903	0.010648944	0.007865124	1.038637491	0.008565042
3.348678891	0.564605962	1.064161721	1.396718766	0.573162391
1.77895059	0	0.643590834	0.629870468	0
0.031333161	0.030960681	1.103100989	0.028749466	0.024670409
3.368496689	0.496878005	1.864856927	3.537879209	0.96182169
1.265569714	1.898161443	0.05324437	1.260972841	1.248149576
2.551010357	0.468184491	0.934689407	1.247335354	0
1.92689348	2.363627122	0.05802529	0.07551986	0.063856445
1.782985099	0.046128241	0.033341661	2.205907098	1.156327516
0.004677638	0.004625342	0.003428984	0.004313321	0.003730622
0	0	1.475122045	0	0
1.175363139	0.6726863	0	0.673774853	0
0.748963553	0.74894553	0	1.66487666	0
1.20199627	0.444091437	0.901443894	0	0.45190837
0.051812194	1.183397168	0.036873323	0.047359964	1.172702913
2.509519603	0	0	1.931316478	0
0	1.558833118	0.979921963	0	2.779698158
0.894034178	0	1.466994667	0.894840493	0
1.205800555	2.346478688	0.709878996	0.697359593	0.704345404
1.74352675	0.036642746	1.725487697	0.03399464	0.029122544
1.341878827	0	0.813659013	1.739810834	1.752529956

hsa-miR-346	hsa-miR-483-5p	hsa-miR-668-3p	hsa-miR-708-5p	hsa-miR-508-5p
0.073296784	0.113799307	0.061568564	5.023657468	0.0898038
1.210983914	12.0565951	0.041016408	2.627703714	1.847602422
1.104729549	3.625550011	1.099012712	4.925084575	2.466547241
2.879694065	11.61012942	0.010685772	3.085461025	1.057999481
1.339232248	5.305913001	0.066209705	4.111675737	1.362976277
0.034338792	6.032893926	0.029208492	0.048335164	1.159072838
0.104058076	3.237672472	0.086575698	0.156250964	0.129275822
1.662089834	3.564349074	1.062443033	4.106257461	0.017792804
0	1.497412767	0	3.347604164	0
1.141994537	1.148021135	0.027549534	0.04547896	1.149992894
0.592611275	1.424503138	0	2.146120588	0
1.590889192	2.35303621	1.546646145	4.362429808	0.1785765
2.375761437	2.432625503	0.061240919	2.775801397	1.335441245
1.564535247	11.11984162	0	1.975779364	0
2.933344842	2.248923292	2.114559115	2.258821015	0.152672618
0.029120398	13.02942828	0.024812343	1.750022405	0.034973635
0	0	0	3.981886298	0
0	6.102711633	0	4.007668935	0.357493667
0.054131608	6.297141602	0.04574877	3.35889298	0.065750785
2.28915583	3.522950391	0.124811062	0.239338476	0.193211712
0.113311286	3.527777111	2.513916838	0.171647253	0.141360383
0.176306902	2.540642421	0.143594351	0.283925843	0.226274244
0.01956121	5.873121774	0.016720279	0.027169228	0.023391841
0.096207001	2.111096614	1.382378647	2.119760408	0.119099327
1.406747659	3.418715096	0.079560159	4.33122527	0.118019625
3.730765299	0.702779015	0	1.202461813	0
0	11.9944842	0.981047643	2.554903762	0
0.042057198	5.662183182	0.035683517	0.059616576	0.050806763
0.057337484	5.836703857	0.048408265	2.346282569	0.069745726
2.473975857	3.185493396	0.023354965	0.038318516	0.032875015
0	2.922540137	2.445179409	5.525820591	0
2.999620933	9.174436522	0	1.584597023	2.584556704
1.023738824	0	0.563811624	7.073038305	0
0.767850808	1.980028104	0	5.088937625	0
1.279424511	2.998011881	0.05443441	5.839408271	0.078870321
0.168789145	2.50021744	0.137766264	0.269807862	0.215896193
0.08362107	11.68490604	3.865499601	0.123171313	1.384086883
0	9.851571579	3.090709502	0	1.359896908
0.056245161	4.642559848	0.047502711	0.080770623	4.258178297
2.894264378	5.812517275	0.012872092	2.09281252	1.668147887
0.066899706	2.397926085	0.056309102	1.314978656	0.08172955
0.044766092	3.47278127	0.037948409	0.063613491	0.054145429
0.08334712	4.146568462	0.069789998	2.838056209	1.382846268
0.040529922	11.3860776	0.034404832	4.087919207	0.048927986
1.511902258	11.04511155	4.38194545	5.94085327	0
1.908674117	0.926473584	0.931289248	0.924491517	0
0.104867121	2.962937499	1.414571617	1.495242707	0.130328504
0.121891964	3.062547349	0.100866361	0.186164844	0.152654668
0.075422272	4.095671996	0.063311484	0.1102483	0.092496719
0	8.417301115	2.641716223	1.416659966	1.419747807
0.001871274	2.011471963	1.007904323	2.820121151	0.002219923
0.665462004	5.226285225	1.192853904	5.076238793	0
0.036885835	1.168706826	1.152264736	1.173400651	1.786312743
1.06529743	3.425055862	0.012642583	0.020422294	1.068645254
1.044161689	5.718470403	0.008545728	1.046815	0.01186568
0	0.777177271	4.661333751	0.771415976	0

0	4.279576334	0	4.493631822	0
0	5.796342278	0	0	0
0	1.598022935	0	0	0
0	11.34558013	2.373024734	0	1.172007107
0.024302231	5.177913899	0.020740022	1.114197862	1.112778944
0	3.732740944	0	4.430507493	1.383347862
1.208085649	7.896775217	0.703055048	3.787843804	0
0.006396954	4.632208302	0.005492112	0.008780817	1.620546118
2.290075489	2.961733104	0	3.282851999	0
0.044852279	2.874820276	0.038020405	4.310790756	0.054251786
0.041734132	2.250834132	0.03541314	2.586417284	0.050409129
0	6.316289437	0.986924057	2.564228465	0
0	1.564284832	0	0	0
0	2.138692276	0.597520921	4.156726024	0
0.933709379	2.22553052	0.936815191	5.098396364	0
0.038585262	1.176489591	0.032774879	0.054522413	0.046539481
1.531839629	8.255912612	0.104460714	3.367907603	2.689929479
0.001408404	1.592867665	1.005950961	4.097662669	1.006562694
0.207667214	7.351067568	0.167662114	1.994963111	0.270290459
0.006585774	12.7499645	0.005653863	4.047331706	0.007829453
0.188626977	2.607041534	0.153095798	1.900715686	0.243426609
2.056523264	1.642373188	0.008798423	1.048204322	0.012219392
0	2.332378771	0	0	0
0.06119543	7.311365107	0.051601598	1.288020611	0.074568401
0	10.66431383	0	1.903143971	0
0	7.230923294	0	5.377550926	0.855376787
1.750824243	2.797087203	0.027728422	2.529577059	1.763268361
0	3.142487179	0	3.878589497	0.981788353
0.873928361	11.49060953	0.879491141	1.422749523	0.868960389
1.204247947	0.070653744	0.03969899	2.284448983	0.056735488
0.14248878	5.86674503	0.117190537	2.83281823	0.180113215
0.097122057	4.234127854	0.080977307	1.458335562	0.12028179
1.890726409	0.687130471	0	4.357781951	0
0	0.749246376	0	2.199592172	0.742544968
4.579779077	4.220866248	0	1.571958587	0.954717408
1.696293596	6.518764488	0.018497346	1.101752737	1.704272678
0	7.934580205	0	4.270420083	0
2.327912914	6.197748954	0.000897547	5.293093852	1.004862807
0.046607459	2.885853338	0.039485725	1.219192384	0.056419508
0	0	0	3.816887654	0
0	10.29624554	2.055576612	4.282807235	0
3.125834415	5.725585271	1.548708169	1.95431354	0
0.047502077	1.847642318	1.806840279	0.067670368	0.057525709
0.213421069	4.122401621	0.172036574	3.225511077	0.278493828
0	0.858975719	0	0	0
0	1.889575186	0	2.204331008	1.484321819
0	11.61631084	0	4.361744206	2.39135731
3.289636886	11.54712555	0.874092937	3.659905284	0.413002178
1.678911815	3.587266032	0.015578578	3.124221694	0.021771607
0.003369855	4.609925749	0.002896157	2.343832144	0.004000419
2.432189061	9.364584616	0.016821622	4.785107753	1.091406208
0	2.284197627	0	2.684551854	0.795270683
2.079598534	0	0.658306854	4.180490293	0
4.210101505	5.881068184	0.062254216	2.444338255	1.34105268
1.599911649	7.100044261	1.84903421	5.946864243	1.333740721
0	4.232021116	0	3.563680091	0.788047405
2.126512309	2.784624073	0	4.980240861	0.667260673
0	1.882211602	1.489440695	1.47533628	0.911229687

1.798634901	5.211469018	0.035914593	1.199053463	2.245181582
0	9.051099068	2.78132111	5.999678	0
1.333904289	0.121197854	0.065158779	0.113784459	0.095360304
2.179813017	6.299374632	0	4.590052992	0
2.875955763	0.080963802	0.045075468	0.076365968	1.246187555
0.025323001	2.482675954	1.105327125	4.085108418	0.030360843
0.016959973	3.820964167	0.014509419	0.023501295	1.078808621
0	11.90729986	0	4.304790773	1.885254753
0.251581672	2.19218971	0.200741559	0.437262803	0.333906785
0.070163478	2.757034043	0.058995063	0.102061448	0.085843273
0.018311791	2.438656617	1.076516566	0.025405323	1.085069703
1.282339206	6.533357442	0.055007181	2.730289847	0.079742853
0.016075342	3.69868284	1.06727034	4.284802366	0.01919311
1.614659333	5.063264348	1.568086792	3.489473151	0.186749597
0.012291006	2.074930231	1.05156233	2.075984814	1.057160686
0	3.540393036	0	0.970993925	0.971185471
3.63339336	6.449504004	0.047491364	0.080749957	0.068366205
0	3.619472622	0	3.545669629	0
0.98344768	4.220481979	0.984260656	5.700335085	0
4.166437316	1.94476956	0.055045433	2.730569454	3.201407348
0.097183444	4.986312065	0.081026957	4.64610637	2.097838727
0	10.94523578	0.565527504	2.074840851	0.542136561
1.217754681	8.124480151	0	5.576587431	0.690675854
0.58532065	10.23852782	0	4.204903007	1.050896043
0.110061051	4.434065909	0.091402564	0.166208871	5.044377457
0	4.773948746	0.89927016	4.909705602	0.89023139
0.076959981	3.836256896	0.064571004	2.014850712	3.039159114
2.275956592	12.26690381	0.044344208	1.246698276	3.494327576
0.014282337	2.41319623	1.059837724	1.666350121	0.017038558
1.036576551	5.616386168	1.034692017	4.307439937	0.009812466
1.936540229	1.229626038	0.729029526	3.686930026	0
0	3.198805062	0	0.919508356	0
0.966449613	1.542653762	0.968071527	0.964715338	0.964939912
0.021216227	1.703221048	1.088483822	1.70555611	0.025389924
0	7.651884486	0	0	0.979006031
0.078628501	5.303707386	0.065936329	3.512582777	1.361460101
0.066796621	3.771145718	0.056224178	0.096861313	0.081599815
1.036776112	5.79016961	0.007115214	1.038955501	1.038593987
0	11.83152632	0.58858744	5.743919801	0
0	0	0	5.160044256	0
0.023857278	1.717827456	0.020363296	1.112105637	0.028584598
0	10.43102063	0	5.802403779	0.946442957
0.056465892	6.867175774	0.047685751	3.968127432	0.068658477
0	4.499928922	0	4.847606253	0
0.061573187	2.705310157	1.25014326	2.707664209	0.075041513
0.03526976	1.780687068	0.029991203	5.107806763	0.042476839
2.605964131	3.485106688	0.003105169	4.113569957	0.004289949
0	2.184888426	0	0	0
0	6.551315178	0	3.266232896	0
0	2.613906945	0.767593361	4.581542317	0
0.454384336	0.887544975	0	2.608912684	0
0.03902322	1.80127559	0.03314214	1.805415463	1.180624242
0	7.497279369	0.95178737	3.503220145	0.947155061
0	10.87534134	0	1.971418813	0
0	4.996896659	0	4.15395045	0
0	2.527517092	0.717079947	3.883874957	0
0.028153309	0.041589386	0.02399599	0.039407144	1.130561457
0.811069848	2.29788121	0	6.87320453	2.306654376

hsa-miR-380-5p	hsa-miR-3681-5p	hsa-miR-376a-3p	hsa-miR-802	hsa-miR-202-5p
1.345538957	0.082856021	2.439168465	0.105631328	2.725547866
0.066653624	0.054406307	0.068767749	3.321294981	0.052654591
1.112057961	3.315870949	2.474255821	1.111910008	1.107405133
1.058775386	0.013874993	0.017145361	0.017039464	0.013465606
2.023610876	0.089392741	2.471946491	0.114374866	1.350103403
1.161802652	0.038423257	0.04813257	1.161632028	0.037226668
1.491081832	0.118571626	1.491521293	0.154175227	0.114262513
2.091621462	0.01659863	3.10184038	1.669915639	0.016105849
2.892041411	0	0	0	0
0.043963273	1.147149043	1.152228184	0.04499152	0.035076691
0.582672506	2.676648796	0	0	0.587398788
2.356438205	0.16260966	1.670279685	0.216714627	0.156250983
3.043758588	3.884130728	0.105806343	0.105018513	0.079592803
3.432094453	0	4.829388319	0.981742181	0
0.178155671	0.139548455	0.185174511	7.243127079	0.13429223
1.137215053	1.132511059	2.507806335	1.749999274	1.131286296
3.564813373	0	3.409803693	0	0
3.538138209	1.567468095	3.67635661	1.726217892	2.374140149
2.659327548	0.060885818	1.889267338	0.076695253	0.058899603
1.720289077	0.175564385	0.237920162	0.235699947	0.168558738
2.207950492	2.156948082	2.668029956	1.535758327	0.124631541
1.836124812	0.204637158	0.28212834	0.279317815	0.196108554
1.092177913	1.690204695	1.091968928	0.026889594	0.021147993
0.137632897	0.109403187	0.142669557	0.141532092	0.105491112
1.449920035	0.108428837	0.141319917	2.565226062	2.064436617
0.693819505	0	0	0.694700401	0
1.559327357	0.979471512	0	0	0
0.057570332	0.047155303	2.588199135	0.058958034	0.045659556
1.270217129	0.064545837	1.269941793	0.081467954	0.062424771
0.037060835	2.155746181	0.038161396	7.206792735	1.729728098
4.543935089	0	7.351346292	2.462532534	0
2.584538176	1.584614224	1.9996029	4.321465281	0
1.350359535	0	1.350805672	0	0.545126892
1.976615906	0	2.22711057	0	2.253208368
1.30460232	0.072888956	0.09309478	0.092418522	1.287911112
1.799942147	0.195539987	0.268134335	0.265516691	1.726854297
5.509163315	0.094781031	4.884941672	0.121629271	0.091480598
5.372421186	0	5.739337045	0	0
0.077878259	0.063298096	0.080408268	0.07983876	0.061223196
1.070859396	0.016739551	1.670631919	1.670629857	1.665200392
1.315335245	0.075498606	0.096570477	0.09586417	1.297815576
0.06141217	0.05022827	1.837368651	0.062904895	1.200767377
1.393030239	0.094463796	0.122138942	0.121200986	0.091176337
1.190977273	0.045424668	2.246666414	0.056740995	0.043989004
6.202186528	0	7.086387748	0	0
2.696790371	0	2.475816801	0	0
1.494917975	0.119518482	0.156769637	3.843944925	0.115167776
2.253562463	0.139532415	0.185151503	0.183563057	0.134276935
0.106072976	4.247032287	0.109724182	0.108901033	0.082387545
6.562436982	0	5.7776714	2.385878038	0
1.008820331	0.002075982	3.012929705	0.002533417	0.002016378
0.655572888	0.659023727	2.454632186	5.427779735	2.0952444
2.82310501	0.041300918	2.556209772	0.05147489	0.040006843
2.681978922	0.016438431	2.682727796	0.020215306	2.896359386
1.046966577	0.011079553	0.013669861	0.013586112	0.010754727
5.730950886	0.773793214	6.091995277	0	0

2.654686549	0	3.21458807	1.070988559	0.598138761
0.864133776	0	0	0	0
1.213486409	0	1.890422057	1.591110089	1.226487483
6.421937915	0	6.867430084	1.835303263	0
2.47649867	5.195734772	1.11427122	0.033544398	0.026296856
0	0.836638871	1.379804175	1.379722133	2.579973321
0.682285858	5.186227402	2.316173883	5.626081361	1.20012528
1.030149681	2.846936449	3.214390002	0.0086942	0.00689885
1.965710987	0	1.965599449	0	0
0.061534677	0.050326111	1.211040293	4.632847898	3.586259867
2.25240458	0.046789105	0.058887511	0.05848857	1.187360043
3.148490975	1.568075079	4.784654276	0.985482691	3.680927539
0	1.978457572	0.982716846	0	0
2.475447602	2.167878871	1.92701208	0	0
2.705725218	0	1.907030901	0	0
1.181812844	0.043223063	0.054290819	1.181641389	0.041863268
0.185429471	2.217939633	1.598410504	0.191152996	2.984962217
1.006638638	0.001562285	4.010160081	0.001906006	0.001517484
0.326454173	4.388181444	0.342704147	0.338994394	0.23225637
2.040682121	0.007315353	1.030957776	0.00895228	2.0377044
1.895592327	0.219616218	0.305496234	1.899769932	0.210265253
0.013694283	0.011409201	0.014079145	0.013992806	0.011074457
1.765522614	0	1.765105012	0	0
1.288416463	0.068958327	2.705013876	1.288328337	1.90018829
1.4961237	0	1.903154257	0	0
2.781441089	2.128711344	1.405730814	5.933557313	2.609567168
3.388678221	1.75858443	4.134208138	0.045295224	2.181745322
2.97311286	0	2.558724239	5.015665027	0.982201506
3.120703818	0	3.379364487	0.868337571	1.430272383
1.220827516	0.052610055	1.22052012	0.065973038	0.050922385
2.362461723	0.163972426	0.220704325	0.21869894	1.61873849
1.458213511	0.110469918	0.144148875	0.14299657	1.428028347
1.554903557	0.681259838	1.181527095	0	0
1.26314161	0.745141271	1.263523656	0	0.746538543
0	0	0	0.505837158	0.508273708
2.459926211	0.024150294	1.708040168	0.029822289	2.712979524
2.75956364	0.967769022	1.544671838	3.272270816	0.968013195
2.006469388	2.006085124	3.177215019	0.001411773	1.004748914
1.21962256	0.052319583	0.066052699	0.065598439	0.050642231
1.442402625	7.944773303	1.44241685	9.226759513	0
1.312704808	0	2.662721318	1.31282026	3.270774879
1.954464848	1.544854613	2.757163579	0	0
0.06530936	0.053336358	1.852558241	0.066910212	0.051622842
2.733123087	0.250026106	2.023099624	0.350324475	0.238928613
0	3.961945097	0.855122432	0	0.858302413
1.888178518	4.130124844	0.916290772	0	0
2.383824566	0	2.125834962	0	1.422036718
3.023959199	0.836565855	2.160695276	0.829560807	0
1.085846438	2.429755086	1.085648424	2.929620439	7.11145838
1.015883474	5.802948222	2.020936819	3.608848825	1.603114576
3.303630853	0.021936584	4.784806596	5.047741333	0.021277494
1.33007021	0	3.372452326	0	1.340655553
2.987361138	0	3.095091154	5.322490025	0
0.10414944	1.332999286	0.107720666	0.106915633	0.080959641
4.025941092	0	5.557822964	0.364035161	0
2.675287127	0	3.123060079	0	0
1.538743747	0	2.079999541	6.76519051	2.679775294
0.910480052	0.912431671	0.910771669	5.965452221	0

1.823170267	0.04746838	1.823842382	1.823772817	1.80611756
3.574400538	0	4.118548863	0.605392465	0.609054801
0.109447137	0.087908941	0.113239876	0.112384662	1.98035579
2.762838123	0	0.898589327	0.466089224	0
1.251274892	0.059961347	0.076026429	0.075492724	1.238449518
2.482873544	0.028267877	3.496270258	2.15524378	0.027406621
0.022754568	0.01889056	0.023408098	0.023261417	0.018326837
0.999213855	1.367722229	1.631406632	1.000184832	1.662580258
2.931210867	0.297513423	2.952899536	0.428624117	0.283488471
1.975864696	0.079249328	2.420982801	0.100833582	3.203811938
0.024594673	2.105003593	0.025304135	1.08616611	0.019792603
1.307879705	0.073685562	1.950928911	1.307823873	1.29093732
1.676369267	0.017901047	2.099136335	4.113886248	2.091148863
1.697449436	2.313959968	2.395360981	0.227292956	0.163132961
1.654962872	1.651449564	0.016894162	0.016789878	0.013269988
0	3.130514194	0.970972648	0	0
0.07785845	2.642089771	0.08038771	0.079818372	1.251219843
2.417657472	2.265722155	3.08820185	3.832142549	0
0	0	0	5.830549148	1.56484871
0.091180204	2.690847801	0.094225516	1.308043202	0.07127143
0.139141839	0.110541498	0.144248204	1.458898634	1.42829019
4.146588799	0.545123975	4.311022764	1.003433203	0
2.119243303	0.693490723	2.957269589	3.297643389	0
2.985209777	0	3.179881593	0.576713712	1.965304221
1.519557166	0.125606293	0.165334266	0.163962596	0.120985539
3.154107271	0	2.643754052	0	1.861360469
1.362842975	0.087079994	0.11211916	4.840752487	5.024142003
0.072387962	4.014153515	0.074712385	0.07418931	1.234583796
2.087796839	0.015896875	2.414003397	0.019543646	0.015425676
2.050893487	2.047754517	2.37532345	4.146487746	0.008897785
2.860203965	3.649804094	0.710299982	2.710129059	1.619721326
0	0.9209853	0.919447203	0	0
0.964583313	0	0.964689335	0	0
0.028561583	2.121323287	0.029393196	0.029206489	0.022944351
1.559139809	0	0	0	0
2.466401194	1.352740756	0.114725441	0.113856572	1.348660089
2.39857425	4.200608033	1.959040631	0.095707678	0.072849919
2.051170722	0.009215613	0.011358433	1.039017944	0.008946588
3.069853194	0	3.251106487	0	0
1.118155413	0	0.631222596	1.484212061	0
0.032184671	0.026621412	1.720446625	0.03291751	0.025813241
5.279029404	2.032267462	4.912961839	0.943962922	0.956332923
1.901048561	0.063550176	3.700801068	0.080167728	1.252247298
1.943085408	0	2.768663782	0.471338686	0.473242896
1.29019875	0.069390854	1.930318105	0.087815098	1.274592691
2.544534806	1.160209517	1.165893403	0.049150149	1.158682687
1.605618935	0.004010484	1.016984891	1.017000731	2.020750495
1.463897275	0	0.901567287	0	0
2.487651676	0.676813726	3.699051758	0	0.67839384
1.657735347	0	2.211771152	0	1.284918123
1.471705849	0.880218201	0.448075995	0	0
0.053287145	2.219500162	1.183571893	0.054559594	0.042341873
3.504211545	0	3.503328941	0	0
0	0	1.558681645	0	0.979131291
1.455052009	1.865894372	0	0.89434026	0
1.582495819	0	1.582247224	1.582194578	1.218619316
0.038110702	1.128146037	1.744598209	0.038990341	0.030485885
0.803275793	0.80652351	3.493191092	2.041450693	0

hsa-miR-211-5p	hsa-miR-1911-5p	hsa-miR-200c-5p
1.343246957	0.100769154	0.084893479
0.063173074	0.06536555	0.055666939
0.030519399	0.031503584	0.027113215
1.0586411	0.016384257	0.014168177
2.017278488	0.109025601	0.091619962
1.161198056	0.045848687	0.03928273
1.486571524	0.146447907	0.121696849
3.098741	1.070257028	0.016951651
1.496162029	0	0.928250589
1.765926165	0.043153203	1.148069005
0.581616662	3.294252893	0
1.660059358	0.204723854	1.629264412
2.761510931	0.100189998	0.084420392
0.981730239	0	2.298173339
0.16683706	0.17393313	1.545282917
0.037501429	0.038730468	0.033257652
0	0	0
1.062001578	0.75012034	0.359051769
0.070853534	0.073353567	0.062316327
3.702459734	6.113890135	0.180680258
4.702884167	3.702918339	1.507910881
2.516715239	0.262424969	0.210886827
1.091925235	0.025832773	0.022272309
1.450016377	0.134588669	0.112237313
0.128253021	1.44892566	0.111232508
2.343063675	0	1.210244876
1.972507074	0	0
3.271278072	0.056478487	0.048230783
1.268757942	0.077885192	0.066074145
2.164724506	0.036388105	0.031269163
0.492696235	2.718165696	4.280489259
1.999608838	0.999699289	0.999705807
0	0.999948217	0.542313383
3.209742482	0	0
3.735079615	0.088268109	0.074645156
1.787731867	4.727641987	0.2014264
0.111586106	0.115866179	0.097167828
0.814676426	0	0
0.073721561	1.898646736	0.064792914
3.802514181	0.019790933	0.017095689
0.088299249	0.091530969	0.0773275
1.209999156	1.210770754	0.051381578
0.111200523	0.115462569	0.096841119
0.052582475	0.054365455	0.046456732
1.914793227	0	0
0	0	0
4.236424149	2.610046742	2.122962272
0.166816963	0.173911925	0.14335365
2.439798599	5.14086974	0.087414012
3.492601442	0	0
1.008805968	0.002439181	0.002118607
0	1.523168983	0
7.324402979	8.079564081	0.042230735
2.41502215	0.019432437	0.016787913
1.046866693	0.013067749	0.011312101
0	0	0

1.432596299	0	0
6.198483081	0	0
0	1.592093253	2.729046003
0	0	0
1.114164863	1.114477126	0.027712324
0	3.826536245	0
4.834551617	0	0
1.030092254	0.00836616	0.007252961
0	0	0
1.834252071	0.060357988	0.051481913
5.323289888	0.056031116	0.047855374
0.985474111	0	0
0	4.497240184	0
1.046003675	1.402564383	0
0	2.485724911	0
0.049996325	0.051681878	0.044200328
1.590567358	0.180982008	2.225330038
0.001781607	1.593005023	0.001594322
0.300981613	0.31684827	1.910404256
0.008358681	0.008614303	0.007467499
3.892968196	0.283497332	0.226480707
0.013054595	1.048359089	1.047206474
0	3.315872499	0
1.922577994	0.083367293	0.070606304
0.927396901	0.927315625	0
0	0	0
1.152960086	0.043443318	0.037249732
1.562726188	1.975797142	0
0	0	0.870061729
0.061049871	1.220635706	0.053824332
0.197692068	0.206562465	0.168658352
3.171431739	0.135963701	0.113337493
0	0	0
0	6.382551611	0
3.388041507	0.503480557	0
2.457670718	0.028642671	0.024673137
2.276891728	0	0
0.001319734	0.001359396	1.004811893
0.060706772	0.062802374	0.053526395
0	0.883823573	0
1.313761786	0	0
0.965601356	0	0
2.281828711	1.223641758	0.05456934
0.310522535	0.327117785	1.93443588
1.406839457	0	0
0	0	0
0	0	0.863265523
0	0	6.720533981
1.085618635	0.02403414	0.020733302
3.022584816	0.004397404	2.019775859
4.942123646	1.092719505	0.022409049
0.794072616	0	0
0.635592868	0	0
1.347145246	0.101982714	1.972561367
0.359832699	0	0
2.996394526	0	0.789584477
2.086609715	0	0.669142601
0	5.070085231	0

3.077717957	0.056861073	2.568964902
0	0	0
2.78879151	0.107147392	0.090092718
0.462711563	0	0
7.50107456	0.072211172	1.86753408
0.032531041	0.03358502	0.028885735
0.021670077	0.022354469	0.019294515
0	0	0.541598913
0.375606178	0.397544357	0.307903446
3.01712813	0.096233245	0.081183879
1.086061856	0.024160418	0.020841409
0.086125305	0.089263341	0.075463881
0.020528042	0.021174603	1.672355399
0.20519438	0.214518097	0.174753209
2.888086496	0.01614463	0.013962018
6.51080402	2.543875079	0
0.073702967	0.076319367	1.255682585
1.491265617	1.489375774	0
0	0	0
4.423254454	0.089329858	0.075518584
2.113091337	0.136056013	0.113411322
0.5412066	0	1.01093706
0	0	0
0.574313752	0.574768358	0
0.149450345	0.155610328	0.128960383
0.88939705	0	0
0.102249252	0.106099138	1.348106449
0.068565194	0.070972661	0.06033781
0.018217306	0.018787921	1.065637621
3.377706441	0.010801223	0.009356792
0	0.708786269	0.711756528
0.919230294	0	0
0.964621359	0	1.955450339
0.027183007	0.028052779	0.024169473
0	0.97877667	0
1.368109822	0.108536536	0.09122245
1.312920771	0.091382822	0.077205826
1.039004591	0.010860736	0.009408169
0	0	5.075195187
0.62885654	1.484972023	0
0.03061906	3.330099786	0.027201072
1.286550577	5.097071552	5.224658749
1.264686622	0.076651023	6.272074979
1.949723321	0	0
0.080986361	0.083905776	1.279592524
1.165557636	0.047124378	0.040359703
0.004577312	0.004715993	0.004093223
4.171598009	0	0
3.638121798	0	0
1.274033385	0.749024051	0.751940242
2.37774071	7.080607511	0
0.050578186	1.80411198	1.178214547
0	0	1.522207669
0.978449018	0	0
3.013653454	0	0.895855364
0	0	1.899648091
4.891217373	0.037417326	1.128929034
1.342517393	0.803092613	0

Table S5. GO annotation of the target genes

ONTOLOGY	ID	Description	GeneRatic
BP	GO:0009410	response to xenobiotic stimulus	9/115
BP	GO:0035690	cellular response to drug	10/115
BP	GO:0034116	positive regulation of heterotypic cell-cell adhesion	3/115
BP	GO:0042448	progesterone metabolic process	3/115
BP	GO:0014074	response to purine-containing compound	6/115
BP	GO:0051591	response to cAMP	5/115
BP	GO:0034114	regulation of heterotypic cell-cell adhesion	3/115
BP	GO:0003018	vascular process in circulatory system	6/115
BP	GO:0070371	ERK1 and ERK2 cascade	8/115
BP	GO:0048147	negative regulation of fibroblast proliferation	3/115
BP	GO:0071466	cellular response to xenobiotic stimulus	6/115
BP	GO:0007596	blood coagulation	8/115
BP	GO:0007599	hemostasis	8/115
BP	GO:0050817	coagulation	8/115
BP	GO:0042554	superoxide anion generation	3/115
BP	GO:0046683	response to organophosphorus	5/115
BP	GO:0042428	serotonin metabolic process	2/115
BP	GO:0050861	positive regulation of B cell receptor signaling pathway	2/115
BP	GO:0070472	regulation of uterine smooth muscle contraction	2/115
BP	GO:0071313	cellular response to caffeine	2/115
BP	GO:0072203	cell proliferation involved in metanephros development	2/115
BP	GO:0071867	response to monoamine	3/115
BP	GO:0071869	response to catecholamine	3/115
BP	GO:0008207	C21-steroid hormone metabolic process	3/115
BP	GO:0097756	negative regulation of blood vessel diameter	4/115
BP	GO:0035296	regulation of tube diameter	5/115
BP	GO:0050880	regulation of blood vessel size	5/115
BP	GO:0097746	regulation of blood vessel diameter	5/115
BP	GO:0042446	hormone biosynthetic process	4/115
BP	GO:0030534	adult behavior	5/115
BP	GO:0035150	regulation of tube size	5/115
BP	GO:0046886	positive regulation of hormone biosynthetic process	2/115
BP	GO:0070471	uterine smooth muscle contraction	2/115
BP	GO:0023061	signal release	9/115
BP	GO:0030168	platelet activation	5/115
BP	GO:0070372	regulation of ERK1 and ERK2 cascade	7/115
BP	GO:0010769	regulation of cell morphogenesis involved in differentiatic	7/115
BP	GO:0010770	positive regulation of cell morphogenesis involved in diffe	5/115
BP	GO:0071415	cellular response to purine-containing compound	2/115
BP	GO:1901160	primary amino compound metabolic process	2/115
BP	GO:0090277	positive regulation of peptide hormone secretion	4/115
BP	GO:0009743	response to carbohydrate	6/115
BP	GO:0006953	acute-phase response	3/115
BP	GO:0022604	regulation of cell morphogenesis	9/115
BP	GO:0010752	regulation of cGMP-mediated signaling	2/115
BP	GO:0072216	positive regulation of metanephros development	2/115
BP	GO:1901722	regulation of cell proliferation involved in kidney develop	2/115
BP	GO:0007631	feeding behavior	4/115
BP	GO:0048661	positive regulation of smooth muscle cell proliferation	4/115
BP	GO:0021549	cerebellum development	4/115
BP	GO:0010975	regulation of neuron projection development	9/115
BP	GO:0032352	positive regulation of hormone metabolic process	2/115
BP	GO:0033604	negative regulation of catecholamine secretion	2/115
BP	GO:0045073	regulation of chemokine biosynthetic process	2/115
BP	GO:0046321	positive regulation of fatty acid oxidation	2/115
BP	GO:1903358	regulation of Golgi organization	2/115

BP	GO:0060537	muscle tissue development	8/115
BP	GO:0007229	integrin-mediated signaling pathway	4/115
BP	GO:0018958	phenol-containing compound metabolic process	4/115
BP	GO:0045599	negative regulation of fat cell differentiation	3/115
BP	GO:0030195	negative regulation of blood coagulation	3/115
BP	GO:0071320	cellular response to cAMP	3/115
BP	GO:0010566	regulation of ketone biosynthetic process	2/115
BP	GO:0032354	response to follicle-stimulating hormone	2/115
BP	GO:0042033	chemokine biosynthetic process	2/115
BP	GO:0050755	chemokine metabolic process	2/115
BP	GO:0071871	response to epinephrine	2/115
BP	GO:0097237	cellular response to toxic substance	6/115
BP	GO:1900047	negative regulation of hemostasis	3/115
BP	GO:0031000	response to caffeine	2/115
BP	GO:0036270	response to diuretic	2/115
BP	GO:0022037	metencephalon development	4/115
BP	GO:0010332	response to gamma radiation	3/115
BP	GO:0048488	synaptic vesicle endocytosis	3/115
BP	GO:0140238	presynaptic endocytosis	3/115
BP	GO:0022409	positive regulation of cell-cell adhesion	6/115
BP	GO:1903034	regulation of response to wounding	5/115
BP	GO:0071496	cellular response to external stimulus	7/115
BP	GO:0043279	response to alkaloid	4/115
BP	GO:0050819	negative regulation of coagulation	3/115
BP	GO:0090257	regulation of muscle system process	6/115
BP	GO:0035994	response to muscle stretch	2/115
BP	GO:0050770	regulation of axonogenesis	5/115
BP	GO:2000756	regulation of peptidyl-lysine acetylation	3/115
BP	GO:0010888	negative regulation of lipid storage	2/115
BP	GO:0032930	positive regulation of superoxide anion generation	2/115
BP	GO:0060004	reflex	2/115
BP	GO:0030098	lymphocyte differentiation	7/115
BP	GO:0034113	heterotypic cell-cell adhesion	3/115
BP	GO:0001505	regulation of neurotransmitter levels	7/115
BP	GO:0050433	regulation of catecholamine secretion	3/115
BP	GO:0048148	behavioral response to cocaine	2/115
BP	GO:0050432	catecholamine secretion	3/115
BP	GO:0042135	neurotransmitter catabolic process	2/115
BP	GO:0072111	cell proliferation involved in kidney development	2/115
BP	GO:0010038	response to metal ion	7/115
BP	GO:0009749	response to glucose	5/115
BP	GO:0050854	regulation of antigen receptor-mediated signaling pathwa	3/115
BP	GO:0018107	peptidyl-threonine phosphorylation	4/115
BP	GO:0030198	extracellular matrix organization	7/115
BP	GO:0035270	endocrine system development	4/115
BP	GO:0010976	positive regulation of neuron projection development	6/115
BP	GO:0032928	regulation of superoxide anion generation	2/115
BP	GO:0045666	positive regulation of neuron differentiation	7/115
BP	GO:0009746	response to hexose	5/115
BP	GO:0034754	cellular hormone metabolic process	4/115
BP	GO:0007409	axonogenesis	8/115
BP	GO:0019915	lipid storage	3/115
BP	GO:0035914	skeletal muscle cell differentiation	3/115
BP	GO:0030539	male genitalia development	2/115
BP	GO:0072215	regulation of metanephros development	2/115
BP	GO:0046887	positive regulation of hormone secretion	4/115
BP	GO:0036465	synaptic vesicle recycling	3/115
BP	GO:0050769	positive regulation of neurogenesis	8/115

BP	GO:0034284	response to monosaccharide	5/115
BP	GO:0071456	cellular response to hypoxia	5/115
BP	GO:0030900	forebrain development	7/115
BP	GO:0018210	peptidyl-threonine modification	4/115
BP	GO:0014850	response to muscle activity	2/115
BP	GO:0042430	indole-containing compound metabolic process	2/115
BP	GO:0050857	positive regulation of antigen receptor-mediated signalin	2/115
BP	GO:0031346	positive regulation of cell projection organization	7/115
BP	GO:0009612	response to mechanical stimulus	5/115
BP	GO:0032024	positive regulation of insulin secretion	3/115
BP	GO:0014829	vascular smooth muscle contraction	2/115
BP	GO:0031639	plasminogen activation	2/115
BP	GO:0043951	negative regulation of cAMP-mediated signaling	2/115
BP	GO:0006801	superoxide metabolic process	3/115
BP	GO:1901983	regulation of protein acetylation	3/115
BP	GO:0014706	striated muscle tissue development	7/115
BP	GO:0070374	positive regulation of ERK1 and ERK2 cascade	5/115
BP	GO:0017157	regulation of exocytosis	5/115
BP	GO:0036294	cellular response to decreased oxygen levels	5/115
BP	GO:0006700	C21-steroid hormone biosynthetic process	2/115
BP	GO:1903203	regulation of oxidative stress-induced neuron death	2/115
BP	GO:0006814	sodium ion transport	5/115
BP	GO:0042310	vasoconstriction	3/115
BP	GO:0061045	negative regulation of wound healing	3/115
BP	GO:0035902	response to immobilization stress	2/115
BP	GO:0036475	neuron death in response to oxidative stress	2/115
BP	GO:0046885	regulation of hormone biosynthetic process	2/115
BP	GO:0051953	negative regulation of amine transport	2/115
BP	GO:0051937	catecholamine transport	3/115
BP	GO:0045444	fat cell differentiation	5/115
BP	GO:0022407	regulation of cell-cell adhesion	7/115
BP	GO:0046879	hormone secretion	6/115
BP	GO:0008209	androgen metabolic process	2/115
BP	GO:0034698	response to gonadotropin	2/115
BP	GO:0042730	fibrinolysis	2/115
BP	GO:0045577	regulation of B cell differentiation	2/115
BP	GO:0030193	regulation of blood coagulation	3/115
BP	GO:0071260	cellular response to mechanical stimulus	3/115
BP	GO:1901214	regulation of neuron death	6/115
BP	GO:1900046	regulation of hemostasis	3/115
BP	GO:0035107	appendage morphogenesis	4/115
BP	GO:0035108	limb morphogenesis	4/115
BP	GO:0051592	response to calcium ion	4/115
BP	GO:0061041	regulation of wound healing	4/115
BP	GO:0050773	regulation of dendrite development	4/115
BP	GO:0050855	regulation of B cell receptor signaling pathway	2/115
BP	GO:1900181	negative regulation of protein localization to nucleus	2/115
BP	GO:0021766	hippocampus development	3/115
BP	GO:0048708	astrocyte differentiation	3/115
BP	GO:1904018	positive regulation of vasculature development	5/115
BP	GO:0000302	response to reactive oxygen species	5/115
BP	GO:0042445	hormone metabolic process	5/115
BP	GO:0030902	hindbrain development	4/115
BP	GO:0046320	regulation of fatty acid oxidation	2/115
BP	GO:0048145	regulation of fibroblast proliferation	3/115
BP	GO:0071277	cellular response to calcium ion	3/115
BP	GO:0016358	dendrite development	5/115
BP	GO:0009914	hormone transport	6/115

BP	GO:0042133	neurotransmitter metabolic process	4/115
BP	GO:0071453	cellular response to oxygen levels	5/115
BP	GO:0014910	regulation of smooth muscle cell migration	3/115
BP	GO:0048144	fibroblast proliferation	3/115
BP	GO:0050818	regulation of coagulation	3/115
BP	GO:0060560	developmental growth involved in morphogenesis	5/115
BP	GO:0030431	sleep	2/115
BP	GO:0050772	positive regulation of axonogenesis	3/115
BP	GO:0043062	extracellular structure organization	7/115
BP	GO:0046677	response to antibiotic	6/115
BP	GO:0033002	muscle cell proliferation	5/115
BP	GO:0001655	urogenital system development	6/115
BP	GO:0051235	maintenance of location	6/115
BP	GO:0045987	positive regulation of smooth muscle contraction	2/115
BP	GO:0070570	regulation of neuron projection regeneration	2/115
BP	GO:0071214	cellular response to abiotic stimulus	6/115
BP	GO:0104004	cellular response to environmental stimulus	6/115
BP	GO:1904035	regulation of epithelial cell apoptotic process	3/115
BP	GO:0007519	skeletal muscle tissue development	4/115
BP	GO:1903305	regulation of regulated secretory pathway	4/115
BP	GO:0030336	negative regulation of cell migration	6/115
BP	GO:0051090	regulation of DNA-binding transcription factor activity	7/115
BP	GO:0019934	cGMP-mediated signaling	2/115
BP	GO:0061036	positive regulation of cartilage development	2/115
BP	GO:2000758	positive regulation of peptidyl-lysine acetylation	2/115
BP	GO:2001024	negative regulation of response to drug	2/115
BP	GO:0015844	monoamine transport	3/115
BP	GO:1903035	negative regulation of response to wounding	3/115
BP	GO:0071868	cellular response to monoamine stimulus	2/115
BP	GO:0071870	cellular response to catecholamine stimulus	2/115
BP	GO:0097009	energy homeostasis	2/115
BP	GO:0014909	smooth muscle cell migration	3/115
BP	GO:0071560	cellular response to transforming growth factor beta stimu	5/115
BP	GO:1904062	regulation of cation transmembrane transport	6/115
BP	GO:0030072	peptide hormone secretion	5/115
BP	GO:0071312	cellular response to alkaloid	2/115
BP	GO:0021537	telencephalon development	5/115
BP	GO:1990138	neuron projection extension	4/115
BP	GO:1901216	positive regulation of neuron death	3/115
BP	GO:0048660	regulation of smooth muscle cell proliferation	4/115
BP	GO:0060538	skeletal muscle organ development	4/115
BP	GO:0046627	negative regulation of insulin receptor signaling pathway	2/115
BP	GO:0060428	lung epithelium development	2/115
BP	GO:0071392	cellular response to estradiol stimulus	2/115
BP	GO:0090322	regulation of superoxide metabolic process	2/115
BP	GO:0070997	neuron death	6/115
BP	GO:0071559	response to transforming growth factor beta	5/115
BP	GO:0051952	regulation of amine transport	3/115
BP	GO:2000146	negative regulation of cell motility	6/115
BP	GO:0006937	regulation of muscle contraction	4/115
BP	GO:0048659	smooth muscle cell proliferation	4/115
BP	GO:0097327	response to antineoplastic agent	3/115
BP	GO:0006979	response to oxidative stress	7/115
BP	GO:0014912	negative regulation of smooth muscle cell migration	2/115
BP	GO:0045923	positive regulation of fatty acid metabolic process	2/115
BP	GO:0021543	pallium development	4/115
BP	GO:0010771	negative regulation of cell morphogenesis involved in diff	3/115
BP	GO:0016572	histone phosphorylation	2/115

BP	GO:0032350	regulation of hormone metabolic process	2/115
BP	GO:1900077	negative regulation of cellular response to insulin stimulus	2/115
BP	GO:1904037	positive regulation of epithelial cell apoptotic process	2/115
BP	GO:0001666	response to hypoxia	6/115
BP	GO:0060840	artery development	3/115
BP	GO:2001023	regulation of response to drug	3/115
BP	GO:0033574	response to testosterone	2/115
BP	GO:0050821	protein stabilization	4/115
BP	GO:0046883	regulation of hormone secretion	5/115
BP	GO:0048736	appendage development	4/115
BP	GO:0060173	limb development	4/115
BP	GO:0015837	amine transport	3/115
BP	GO:0031668	cellular response to extracellular stimulus	5/115
BP	GO:0050714	positive regulation of protein secretion	5/115
BP	GO:0014046	dopamine secretion	2/115
BP	GO:0014059	regulation of dopamine secretion	2/115
BP	GO:0071526	semaphorin-plexin signaling pathway	2/115
BP	GO:0090184	positive regulation of kidney development	2/115
BP	GO:0008217	regulation of blood pressure	4/115
BP	GO:0014812	muscle cell migration	3/115
BP	GO:0032868	response to insulin	5/115
BP	GO:0036293	response to decreased oxygen levels	6/115
BP	GO:0042181	ketone biosynthetic process	2/115
BP	GO:1903706	regulation of hemopoiesis	7/115
BP	GO:0034198	cellular response to amino acid starvation	2/115
BP	GO:1901985	positive regulation of protein acetylation	2/115
BP	GO:0015696	ammonium transport	3/115
BP	GO:0015711	organic anion transport	7/115
BP	GO:0021761	limbic system development	3/115
BP	GO:0034765	regulation of ion transmembrane transport	7/115
BP	GO:0071248	cellular response to metal ion	4/115
BP	GO:0032965	regulation of collagen biosynthetic process	2/115
BP	GO:0044331	cell-cell adhesion mediated by cadherin	2/115
BP	GO:0016049	cell growth	7/115
BP	GO:0042594	response to starvation	4/115
BP	GO:0006939	smooth muscle contraction	3/115
BP	GO:0006631	fatty acid metabolic process	6/115
BP	GO:1904019	epithelial cell apoptotic process	3/115
BP	GO:0031018	endocrine pancreas development	2/115
BP	GO:0051271	negative regulation of cellular component movement	6/115
BP	GO:0017158	regulation of calcium ion-dependent exocytosis	3/115
BP	GO:0099504	synaptic vesicle cycle	4/115
BP	GO:0010883	regulation of lipid storage	2/115
BP	GO:0048512	circadian behavior	2/115
BP	GO:0071354	cellular response to interleukin-6	2/115
BP	GO:1990928	response to amino acid starvation	2/115
BP	GO:0002793	positive regulation of peptide secretion	5/115
BP	GO:0007622	rhythmic behavior	2/115
BP	GO:0014911	positive regulation of smooth muscle cell migration	2/115
BP	GO:0048806	genitalia development	2/115
BP	GO:0032963	collagen metabolic process	3/115
BP	GO:0070482	response to oxygen levels	6/115
BP	GO:0045933	positive regulation of muscle contraction	2/115
BP	GO:0040013	negative regulation of locomotion	6/115
BP	GO:0010712	regulation of collagen metabolic process	2/115
BP	GO:0060042	retina morphogenesis in camera-type eye	2/115
BP	GO:1903532	positive regulation of secretion by cell	6/115
BP	GO:0016079	synaptic vesicle exocytosis	3/115

BP	GO:0045766	positive regulation of angiogenesis	4/115
BP	GO:0002931	response to ischemia	2/115
BP	GO:0009409	response to cold	2/115
BP	GO:0014009	glial cell proliferation	2/115
BP	GO:0070741	response to interleukin-6	2/115
BP	GO:0097164	ammonium ion metabolic process	4/115
BP	GO:0045785	positive regulation of cell adhesion	6/115
BP	GO:0010811	positive regulation of cell-substrate adhesion	3/115
BP	GO:0043618	regulation of transcription from RNA polymerase II promc	3/115
BP	GO:0048675	axon extension	3/115
BP	GO:0034599	cellular response to oxidative stress	5/115
BP	GO:0099003	vesicle-mediated transport in synapse	4/115
BP	GO:0019369	arachidonic acid metabolic process	2/115
BP	GO:0043949	regulation of cAMP-mediated signaling	2/115
BP	GO:0090276	regulation of peptide hormone secretion	4/115
BP	GO:0015872	dopamine transport	2/115
BP	GO:0043523	regulation of neuron apoptotic process	4/115
BP	GO:0046717	acid secretion	3/115
BP	GO:0007517	muscle organ development	6/115
BP	GO:0030326	embryonic limb morphogenesis	3/115
BP	GO:0035113	embryonic appendage morphogenesis	3/115
BP	GO:0007131	reciprocal meiotic recombination	2/115
BP	GO:0031638	zymogen activation	2/115
BP	GO:0032964	collagen biosynthetic process	2/115
BP	GO:0035065	regulation of histone acetylation	2/115
BP	GO:0042113	B cell activation	5/115
BP	GO:0015893	drug transport	4/115
BP	GO:0002720	positive regulation of cytokine production involved in imr	2/115
BP	GO:0006584	catecholamine metabolic process	2/115
BP	GO:0009712	catechol-containing compound metabolic process	2/115
BP	GO:0030199	collagen fibril organization	2/115
BP	GO:0035825	homologous recombination	2/115
BP	GO:0043620	regulation of DNA-templated transcription in response to	3/115
BP	GO:0001558	regulation of cell growth	6/115
BP	GO:0042220	response to cocaine	2/115
BP	GO:0071241	cellular response to inorganic substance	4/115
BP	GO:0070588	calcium ion transmembrane transport	5/115
BP	GO:0010001	glial cell differentiation	4/115
BP	GO:1901342	regulation of vasculature development	6/115
BP	GO:0090183	regulation of kidney development	2/115
BP	GO:0030183	B cell differentiation	3/115
BP	GO:0045598	regulation of fat cell differentiation	3/115
BP	GO:0072329	monocarboxylic acid catabolic process	3/115
BP	GO:0007568	aging	5/115
BP	GO:0051047	positive regulation of secretion	6/115
BP	GO:0001933	negative regulation of protein phosphorylation	6/115
BP	GO:0051052	regulation of DNA metabolic process	6/115
BP	GO:0019229	regulation of vasoconstriction	2/115
BP	GO:0032722	positive regulation of chemokine production	2/115
BP	GO:0035904	aorta development	2/115
BP	GO:1902041	regulation of extrinsic apoptotic signaling pathway via dea	2/115
CC	GO:0070821	tertiary granule membrane	3/121
CC	GO:0031252	cell leading edge	7/121
CC	GO:0150034	distal axon	5/121
CC	GO:0009897	external side of plasma membrane	6/121
CC	GO:0030667	secretory granule membrane	5/121
CC	GO:0043679	axon terminus	3/121
MF	GO:0005452	inorganic anion exchanger activity	2/115

MF	GO:0030553	cGMP binding	2/115
MF	GO:0015291	secondary active transmembrane transporter activity	6/115
MF	GO:0070402	NADPH binding	2/115
MF	GO:0001227	DNA-binding transcription repressor activity, RNA polyme	6/115
MF	GO:0035173	histone kinase activity	2/115
MF	GO:0047555	3',5'-cyclic-GMP phosphodiesterase activity	2/115
MF	GO:0001228	DNA-binding transcription activator activity, RNA polyme	8/115
MF	GO:0019865	immunoglobulin binding	2/115
MF	GO:0050662	coenzyme binding	6/115
MF	GO:0008514	organic anion transmembrane transporter activity	5/115
MF	GO:0004114	3',5'-cyclic-nucleotide phosphodiesterase activity	2/115
MF	GO:0015293	symporter activity	4/115
MF	GO:0004112	cyclic-nucleotide phosphodiesterase activity	2/115
MF	GO:0035035	histone acetyltransferase binding	2/115
MF	GO:0043178	alcohol binding	3/115
MF	GO:0031624	ubiquitin conjugating enzyme binding	2/115
MF	GO:0035258	steroid hormone receptor binding	3/115
MF	GO:0030551	cyclic nucleotide binding	2/115
MF	GO:0044390	ubiquitin-like protein conjugating enzyme binding	2/115
MF	GO:0046873	metal ion transmembrane transporter activity	7/115
MF	GO:0022804	active transmembrane transporter activity	6/115
MF	GO:0004712	protein serine/threonine/tyrosine kinase activity	2/115
MF	GO:0050681	androgen receptor binding	2/115
MF	GO:0042805	actinin binding	2/115
MF	GO:0005246	calcium channel regulator activity	2/115
MF	GO:1990841	promoter-specific chromatin binding	2/115
MF	GO:0099106	ion channel regulator activity	3/115
MF	GO:0051018	protein kinase A binding	2/115
MF	GO:0019001	guanyl nucleotide binding	6/115
MF	GO:0032561	guanyl ribonucleotide binding	6/115
MF	GO:0008146	sulfotransferase activity	2/115
MF	GO:0015459	potassium channel regulator activity	2/115
MF	GO:0050661	NADP binding	2/115
MF	GO:0019207	kinase regulator activity	4/115
MF	GO:0001047	core promoter binding	2/115

BgRatio	pvalue	p.adjust	qvalue
292/18670	8.18E-05	0.07431409	0.06749785
369/18670	9.53E-05	0.07431409	0.06749785
16/18670	0.000120249	0.07431409	0.06749785
16/18670	0.000120249	0.07431409	0.06749785
149/18670	0.000321638	0.13740977	0.124806264
97/18670	0.000333519	0.13740977	0.124806264
26/18670	0.000533791	0.14943735	0.135730658
173/18670	0.000709538	0.14943735	0.135730658
317/18670	0.000776255	0.14943735	0.135730658
30/18670	0.000818727	0.14943735	0.135730658
180/18670	0.000872473	0.14943735	0.135730658
336/18670	0.001128897	0.14943735	0.135730658
341/18670	0.001240283	0.14943735	0.135730658
342/18670	0.001263579	0.14943735	0.135730658
36/18670	0.001401687	0.14943735	0.135730658
134/18670	0.001443082	0.14943735	0.135730658
10/18670	0.001638793	0.14943735	0.135730658
10/18670	0.001638793	0.14943735	0.135730658
10/18670	0.001638793	0.14943735	0.135730658
10/18670	0.001638793	0.14943735	0.135730658
10/18670	0.001638793	0.14943735	0.135730658
38/18670	0.001641371	0.14943735	0.135730658
38/18670	0.001641371	0.14943735	0.135730658
39/18670	0.001770224	0.14943735	0.135730658
84/18670	0.001804192	0.14943735	0.135730658
143/18670	0.001921157	0.14943735	0.135730658
143/18670	0.001921157	0.14943735	0.135730658
143/18670	0.001921157	0.14943735	0.135730658
86/18670	0.001967044	0.14943735	0.135730658
144/18670	0.001980634	0.14943735	0.135730658
144/18670	0.001980634	0.14943735	0.135730658
11/18670	0.001994916	0.14943735	0.135730658
11/18670	0.001994916	0.14943735	0.135730658
462/18670	0.002236963	0.15558628	0.141315591
153/18670	0.002578182	0.15558628	0.141315591
300/18670	0.002578468	0.15558628	0.141315591
301/18670	0.002626679	0.15558628	0.141315591
154/18670	0.002651821	0.15558628	0.141315591
13/18670	0.002806467	0.15558628	0.141315591
13/18670	0.002806467	0.15558628	0.141315591
96/18670	0.002935836	0.15558628	0.141315591
230/18670	0.003020359	0.15558628	0.141315591
47/18670	0.003030691	0.15558628	0.141315591
484/18670	0.00305111	0.15558628	0.141315591
14/18670	0.003261067	0.15558628	0.141315591
14/18670	0.003261067	0.15558628	0.141315591
14/18670	0.003261067	0.15558628	0.141315591
101/18670	0.003524651	0.15558628	0.141315591
101/18670	0.003524651	0.15558628	0.141315591
102/18670	0.003651344	0.15558628	0.141315591
499/18670	0.003729317	0.15558628	0.141315591
15/18670	0.003747671	0.15558628	0.141315591
15/18670	0.003747671	0.15558628	0.141315591
15/18670	0.003747671	0.15558628	0.141315591
15/18670	0.003747671	0.15558628	0.141315591
15/18670	0.003747671	0.15558628	0.141315591

408/18670	0.003763876	0.15558628	0.141315591
103/18670	0.003781096	0.15558628	0.141315591
103/18670	0.003781096	0.15558628	0.141315591
51/18670	0.003823581	0.15558628	0.141315591
53/18670	0.004263015	0.15558628	0.141315591
53/18670	0.004263015	0.15558628	0.141315591
16/18670	0.004265874	0.15558628	0.141315591
16/18670	0.004265874	0.15558628	0.141315591
16/18670	0.004265874	0.15558628	0.141315591
16/18670	0.004265874	0.15558628	0.141315591
16/18670	0.004265874	0.15558628	0.141315591
247/18670	0.004279881	0.15558628	0.141315591
54/18670	0.004493741	0.15933958	0.144724634
17/18670	0.004815276	0.15933958	0.144724634
17/18670	0.004815276	0.15933958	0.144724634
111/18670	0.004933348	0.15933958	0.144724634
56/18670	0.004977557	0.15933958	0.144724634
56/18670	0.004977557	0.15933958	0.144724634
56/18670	0.004977557	0.15933958	0.144724634
255/18670	0.004991269	0.15933958	0.144724634
179/18670	0.005031621	0.15933958	0.144724634
339/18670	0.005032442	0.15933958	0.144724634
112/18670	0.005092163	0.15933958	0.144724634
57/18670	0.005230781	0.16163113	0.146805999
259/18670	0.005377912	0.16265394	0.147734993
18/18670	0.005395479	0.16265394	0.147734993
183/18670	0.005518888	0.16436977	0.149293446
59/18670	0.005760177	0.16951379	0.153965638
19/18670	0.006006089	0.17065578	0.155002885
19/18670	0.006006089	0.17065578	0.155002885
19/18670	0.006006089	0.17065578	0.155002885
353/18670	0.006245138	0.1741228	0.158151908
61/18670	0.00632057	0.1741228	0.158151908
354/18670	0.006339422	0.1741228	0.158151908
62/18670	0.006612533	0.17859442	0.162213375
20/18670	0.006646718	0.17859442	0.162213375
64/18670	0.00722026	0.18905022	0.171710151
21/18670	0.007316978	0.18905022	0.171710151
21/18670	0.007316978	0.18905022	0.171710151
364/18670	0.007341756	0.18905022	0.171710151
197/18670	0.007487297	0.19009489	0.172659002
65/18670	0.007536124	0.19009489	0.172659002
126/18670	0.007686306	0.1916475	0.174069203
368/18670	0.007773986	0.1916475	0.174069203
127/18670	0.007899294	0.1916475	0.174069203
281/18670	0.007907785	0.1916475	0.174069203
22/18670	0.008016486	0.19239567	0.174748748
371/18670	0.008110325	0.19277618	0.175094359
202/18670	0.008295605	0.19350305	0.175754562
129/18670	0.008336841	0.19350305	0.175754562
468/18670	0.008411866	0.19350305	0.175754562
68/18670	0.008532295	0.19350305	0.175754562
68/18670	0.008532295	0.19350305	0.175754562
23/18670	0.008744864	0.1937897	0.176014917
23/18670	0.008744864	0.1937897	0.176014917
131/18670	0.008789995	0.1937897	0.176014917
69/18670	0.008880692	0.1937897	0.176014917
474/18670	0.009044375	0.1937897	0.176014917

207/18670	0.009162634	0.1937897	0.176014917
207/18670	0.009162634	0.1937897	0.176014917
381/18670	0.009309414	0.1937897	0.176014917
134/18670	0.009499463	0.1937897	0.176014917
24/18670	0.009501735	0.1937897	0.176014917
24/18670	0.009501735	0.1937897	0.176014917
24/18670	0.009501735	0.1937897	0.176014917
383/18670	0.009564055	0.1937897	0.176014917
210/18670	0.009711869	0.19518487	0.17728212
72/18670	0.009975474	0.19847012	0.180266038
25/18670	0.010286728	0.19847012	0.180266038
25/18670	0.010286728	0.19847012	0.180266038
25/18670	0.010286728	0.19847012	0.180266038
73/18670	0.010357057	0.19847012	0.180266038
73/18670	0.010357057	0.19847012	0.180266038
390/18670	0.010495636	0.19957856	0.181272809
215/18670	0.010676974	0.20147695	0.182997079
217/18670	0.011080774	0.20324368	0.184601755
217/18670	0.011080774	0.20324368	0.184601755
26/18670	0.011099473	0.20324368	0.184601755
26/18670	0.011099473	0.20324368	0.184601755
218/18670	0.011286538	0.20514943	0.186332709
76/18670	0.011552251	0.20687989	0.187904448
76/18670	0.011552251	0.20687989	0.187904448
27/18670	0.011939604	0.20687989	0.187904448
27/18670	0.011939604	0.20687989	0.187904448
27/18670	0.011939604	0.20687989	0.187904448
27/18670	0.011939604	0.20687989	0.187904448
77/18670	0.011967566	0.20687989	0.187904448
223/18670	0.012354601	0.20798393	0.188907226
403/18670	0.01239893	0.20798393	0.188907226
312/18670	0.012794715	0.20798393	0.188907226
28/18670	0.01280676	0.20798393	0.188907226
28/18670	0.01280676	0.20798393	0.188907226
28/18670	0.01280676	0.20798393	0.188907226
28/18670	0.01280676	0.20798393	0.188907226
79/18670	0.012823744	0.20798393	0.188907226
79/18670	0.012823744	0.20798393	0.188907226
313/18670	0.012981041	0.20798393	0.188907226
80/18670	0.013264657	0.20798393	0.188907226
148/18670	0.013301019	0.20798393	0.188907226
148/18670	0.013301019	0.20798393	0.188907226
148/18670	0.013301019	0.20798393	0.188907226
148/18670	0.013301019	0.20798393	0.188907226
149/18670	0.01360454	0.20798393	0.188907226
29/18670	0.013700582	0.20798393	0.188907226
29/18670	0.013700582	0.20798393	0.188907226
81/18670	0.013714151	0.20798393	0.188907226
81/18670	0.013714151	0.20798393	0.188907226
230/18670	0.01396237	0.21045718	0.191153622
232/18670	0.014446421	0.21100153	0.191648039
232/18670	0.014446421	0.21100153	0.191648039
152/18670	0.014541463	0.21100153	0.191648039
30/18670	0.014620715	0.21100153	0.191648039
83/18670	0.014638968	0.21100153	0.191648039
83/18670	0.014638968	0.21100153	0.191648039
233/18670	0.014692629	0.21100153	0.191648039
322/18670	0.014744628	0.21100153	0.191648039

153/18670	0.014862625	0.21100153	0.191648039
234/18670	0.014941643	0.21100153	0.191648039
84/18670	0.01511433	0.21100153	0.191648039
84/18670	0.01511433	0.21100153	0.191648039
84/18670	0.01511433	0.21100153	0.191648039
235/18670	0.015193476	0.21100153	0.191648039
31/18670	0.015566806	0.21323632	0.193677857
85/18670	0.015598351	0.21323632	0.193677857
422/18670	0.015613177	0.21323632	0.193677857
327/18670	0.015793419	0.21451281	0.194837257
239/18670	0.016229257	0.21801898	0.19802184
330/18670	0.016446975	0.21801898	0.19802184
330/18670	0.016446975	0.21801898	0.19802184
32/18670	0.016538506	0.21801898	0.19802184
32/18670	0.016538506	0.21801898	0.19802184
331/18670	0.016668927	0.21801898	0.19802184
331/18670	0.016668927	0.21801898	0.19802184
88/18670	0.017102531	0.21892769	0.198847196
160/18670	0.017236662	0.21892769	0.198847196
160/18670	0.017236662	0.21892769	0.198847196
334/18670	0.017347207	0.21892769	0.198847196
432/18670	0.017524893	0.21892769	0.198847196
33/18670	0.01753547	0.21892769	0.198847196
33/18670	0.01753547	0.21892769	0.198847196
33/18670	0.01753547	0.21892769	0.198847196
33/18670	0.01753547	0.21892769	0.198847196
90/18670	0.018148878	0.22432013	0.203745038
90/18670	0.018148878	0.22432013	0.203745038
34/18670	0.018557355	0.22597923	0.205251954
34/18670	0.018557355	0.22597923	0.205251954
34/18670	0.018557355	0.22597923	0.205251954
91/18670	0.018685153	0.22642009	0.205652377
249/18670	0.019022201	0.22937991	0.208340717
342/18670	0.019248562	0.23069388	0.209534171
250/18670	0.019317813	0.23069388	0.209534171
35/18670	0.019603823	0.23201834	0.210737148
251/18670	0.019616437	0.23201834	0.210737148
168/18670	0.020225174	0.2350397	0.213481386
94/18670	0.020346478	0.2350397	0.213481386
169/18670	0.020619767	0.2350397	0.213481386
169/18670	0.020619767	0.2350397	0.213481386
36/18670	0.020674537	0.2350397	0.213481386
36/18670	0.020674537	0.2350397	0.213481386
36/18670	0.020674537	0.2350397	0.213481386
36/18670	0.020674537	0.2350397	0.213481386
348/18670	0.020764957	0.2350397	0.213481386
255/18670	0.020841281	0.2350397	0.213481386
95/18670	0.020917773	0.2350397	0.213481386
349/18670	0.021025362	0.23517962	0.213608466
171/18670	0.021423131	0.23670405	0.214993073
171/18670	0.021423131	0.23670405	0.214993073
96/18670	0.021497835	0.23670405	0.214993073
451/18670	0.021603568	0.23670405	0.214993073
37/18670	0.021769164	0.23670405	0.214993073
37/18670	0.021769164	0.23670405	0.214993073
172/18670	0.021831927	0.23670405	0.214993073
97/18670	0.022086667	0.23842027	0.216551881
38/18670	0.022887375	0.24282228	0.220550129

38/18670	0.022887375	0.24282228	0.220550129
38/18670	0.022887375	0.24282228	0.220550129
38/18670	0.022887375	0.24282228	0.220550129
359/18670	0.023752394	0.25040301	0.227435537
100/18670	0.023905789	0.25040301	0.227435537
100/18670	0.023905789	0.25040301	0.227435537
39/18670	0.024028843	0.25062995	0.227641668
178/18670	0.024385088	0.25111975	0.228086539
266/18670	0.024464545	0.25111975	0.228086539
179/18670	0.024827452	0.25111975	0.228086539
179/18670	0.024827452	0.25111975	0.228086539
102/18670	0.025162379	0.25111975	0.228086539
268/18670	0.025164219	0.25111975	0.228086539
268/18670	0.025164219	0.25111975	0.228086539
40/18670	0.025193244	0.25111975	0.228086539
40/18670	0.025193244	0.25111975	0.228086539
40/18670	0.025193244	0.25111975	0.228086539
40/18670	0.025193244	0.25111975	0.228086539
182/18670	0.026183624	0.25994345	0.236100912
104/18670	0.026454011	0.26157726	0.237584869
272/18670	0.02660194	0.26199201	0.237961573
370/18670	0.027016578	0.26501977	0.240711617
42/18670	0.027589566	0.26906105	0.244382226
475/18670	0.027646241	0.26906105	0.244382226
43/18670	0.028820855	0.27830138	0.252775015
43/18670	0.028820855	0.27830138	0.252775015
108/18670	0.029142209	0.2803095	0.254598943
482/18670	0.029605975	0.28262047	0.256697949
109/18670	0.02983607	0.28262047	0.256697949
483/18670	0.029893428	0.28262047	0.256697949
190/18670	0.030014911	0.28262047	0.256697949
44/18670	0.030073813	0.28262047	0.256697949
44/18670	0.030073813	0.28262047	0.256697949
484/18670	0.030182769	0.28262047	0.256697949
191/18670	0.030515936	0.28380266	0.257771709
110/18670	0.030538636	0.28380266	0.257771709
383/18670	0.031243391	0.28807651	0.261653552
111/18670	0.031249894	0.28807651	0.261653552
45/18670	0.031348132	0.28807651	0.261653552
384/18670	0.031585456	0.2891824	0.262658006
112/18670	0.031969832	0.29126578	0.264550294
194/18670	0.032048662	0.29126578	0.264550294
46/18670	0.032643505	0.29237226	0.265555287
46/18670	0.032643505	0.29237226	0.265555287
46/18670	0.032643505	0.29237226	0.265555287
46/18670	0.032643505	0.29237226	0.265555287
288/18670	0.03287393	0.29337312	0.266464345
47/18670	0.03395963	0.29981502	0.27231538
47/18670	0.03395963	0.29981502	0.27231538
47/18670	0.03395963	0.29981502	0.27231538
115/18670	0.03418158	0.30070059	0.273119727
394/18670	0.03514157	0.30804951	0.279794587
48/18670	0.035296207	0.30831175	0.280032768
396/18670	0.035882617	0.31233039	0.283682811
49/18670	0.036652939	0.31680442	0.287746479
49/18670	0.036652939	0.31680442	0.287746479
399/18670	0.037013011	0.31880196	0.289560799
119/18670	0.037251164	0.31973916	0.290412039

204/18670	0.037480456	0.31993935	0.290593869
50/18670	0.038029531	0.31993935	0.290593869
50/18670	0.038029531	0.31993935	0.290593869
50/18670	0.038029531	0.31993935	0.290593869
50/18670	0.038029531	0.31993935	0.290593869
205/18670	0.03805104	0.31993935	0.290593869
403/18670	0.03855553	0.32216722	0.292617393
121/18670	0.038837311	0.32216722	0.292617393
121/18670	0.038837311	0.32216722	0.292617393
121/18670	0.038837311	0.32216722	0.292617393
302/18670	0.039062118	0.32271626	0.293116073
207/18670	0.039207187	0.32271626	0.293116073
51/18670	0.039425692	0.32271626	0.293116073
51/18670	0.039425692	0.32271626	0.293116073
208/18670	0.039792754	0.32464584	0.294868664
52/18670	0.040841134	0.3321029	0.301641753
210/18670	0.040978881	0.33213047	0.30166679
124/18670	0.041280264	0.33297807	0.302436649
410/18670	0.041352859	0.33297807	0.302436649
125/18670	0.042111489	0.33388246	0.303258081
125/18670	0.042111489	0.33388246	0.303258081
53/18670	0.04227557	0.33388246	0.303258081
53/18670	0.04227557	0.33388246	0.303258081
53/18670	0.04227557	0.33388246	0.303258081
53/18670	0.04227557	0.33388246	0.303258081
310/18670	0.042897644	0.33664206	0.305764572
214/18670	0.043411112	0.33664206	0.305764572
54/18670	0.043728717	0.33664206	0.305764572
54/18670	0.043728717	0.33664206	0.305764572
54/18670	0.043728717	0.33664206	0.305764572
54/18670	0.043728717	0.33664206	0.305764572
54/18670	0.043728717	0.33664206	0.305764572
127/18670	0.043799156	0.33664206	0.305764572
416/18670	0.043850625	0.33664206	0.305764572
55/18670	0.045200295	0.34536937	0.313691395
217/18670	0.045287757	0.34536937	0.313691395
315/18670	0.045406572	0.34536937	0.313691395
218/18670	0.045923294	0.34822817	0.316287974
422/18670	0.046441545	0.35108103	0.318879172
56/18670	0.046690026	0.35188337	0.319607913
131/18670	0.04727465	0.35326829	0.320865804
132/18670	0.048164235	0.35326829	0.320865804
132/18670	0.048164235	0.35326829	0.320865804
321/18670	0.048531311	0.35326829	0.320865804
428/18670	0.049126274	0.35326829	0.320865804
429/18670	0.04958289	0.35326829	0.320865804
429/18670	0.04958289	0.35326829	0.320865804
58/18670	0.049722849	0.35326829	0.320865804
58/18670	0.049722849	0.35326829	0.320865804
58/18670	0.049722849	0.35326829	0.320865804
58/18670	0.049722849	0.35326829	0.320865804
73/19717	0.010263768	0.61608393	0.616083928
403/19717	0.012218935	0.61608393	0.616083928
285/19717	0.031264323	0.61608393	0.616083928
393/19717	0.034330355	0.61608393	0.616083928
298/19717	0.036796429	0.61608393	0.616083928
119/19717	0.036934401	0.61608393	0.616083928
13/17697	0.003115956	0.30346866	0.287046711

15/17697	0.004159119	0.30346866	0.287046711
237/17697	0.004531439	0.30346866	0.287046711
16/17697	0.004733173	0.30346866	0.287046711
242/17697	0.005011026	0.30346866	0.287046711
17/17697	0.005341584	0.30346866	0.287046711
18/17697	0.005983889	0.30346866	0.287046711
439/17697	0.007912752	0.35112839	0.332127369
24/17697	0.010524134	0.35874602	0.339332784
291/17697	0.011875897	0.35874602	0.339332784
211/17697	0.01225087	0.35874602	0.339332784
28/17697	0.014172438	0.35874602	0.339332784
144/17697	0.014496733	0.35874602	0.339332784
29/17697	0.015158283	0.35874602	0.339332784
29/17697	0.015158283	0.35874602	0.339332784
85/17697	0.017954195	0.37559078	0.355266003
32/17697	0.018286262	0.37559078	0.355266003
92/17697	0.022097957	0.37559078	0.355266003
36/17697	0.022839648	0.37559078	0.355266003
37/17697	0.024043731	0.37559078	0.355266003
438/17697	0.024244027	0.37559078	0.355266003
362/17697	0.030845976	0.37559078	0.355266003
43/17697	0.031791273	0.37559078	0.355266003
44/17697	0.033166278	0.37559078	0.355266003
46/17697	0.035984847	0.37559078	0.355266003
48/17697	0.038892509	0.37559078	0.355266003
48/17697	0.038892509	0.37559078	0.355266003
118/17697	0.041655975	0.37559078	0.355266003
50/17697	0.041886525	0.37559078	0.355266003
394/17697	0.043678784	0.37559078	0.355266003
394/17697	0.043678784	0.37559078	0.355266003
52/17697	0.044964214	0.37559078	0.355266003
52/17697	0.044964214	0.37559078	0.355266003
52/17697	0.044964214	0.37559078	0.355266003
207/17697	0.046137177	0.37559078	0.355266003
55/17697	0.049731894	0.37559078	0.355266003

geneID	Count
RGS17/PPARGC1A/PDE2A/ABAT/SDK1/EGR1/SLC8A1/CYP2C18/SRD5A1	9
IL6/MYC/SLC31A1/PPARGC1A/PDE2A/EGR1/NR4A3/CD69/SLC8A1/SRD5A1	10
THY1/ALOX15/FGA	3
PPARGC1A/EGR1/SRD5A1	3
DUOX2/PPARGC1A/PDE2A/FOS/SLC8A1/SRD5A1	6
DUOX2/PDE2A/FOS/SLC8A1/SRD5A1	5
THY1/ALOX15/FGA	3
SERPINF2/PDE2A/CRP/SLC8A1/ADRA2B/FGA	6
MYC/MAP2K1/ALOX15/ATF3/SERPINF2/BMPER/C1QL4/FGA	8
MYC/DACH1/C1QL4	3
PPARGC1A/PDE2A/EGR1/SLC8A1/CYP2C18/SRD5A1	6
IL6/SERPINF2/EHD3/ABAT/CD177/ADRA2B/PRKCB/FGA	8
IL6/SERPINF2/EHD3/ABAT/CD177/ADRA2B/PRKCB/FGA	8
IL6/SERPINF2/EHD3/ABAT/CD177/ADRA2B/PRKCB/FGA	8
DUOX2/CD177/CRP	3
DUOX2/PDE2A/FOS/SLC8A1/SRD5A1	5
SRD5A1/RNF180	2
NFAM1/PRKCB	2
ABAT/ADRA2B	2
PPARGC1A/SLC8A1	2
MYC/EGR1	2
PPARGC1A/NR4A3/SRD5A1	3
PPARGC1A/NR4A3/SRD5A1	3
PPARGC1A/EGR1/SRD5A1	3
CRP/SLC8A1/ADRA2B/FGA	4
SERPINF2/CRP/SLC8A1/ADRA2B/FGA	5
SERPINF2/CRP/SLC8A1/ADRA2B/FGA	5
SERPINF2/CRP/SLC8A1/ADRA2B/FGA	5
DUOX2/PPARGC1A/EGR1/SRD5A1	4
EN1/ABAT/SDK1/NR4A3/RNF180	5
SERPINF2/CRP/SLC8A1/ADRA2B/FGA	5
PPARGC1A/EGR1	2
ABAT/ADRA2B	2
IL6/SOX4/SYT2/ABAT/GLUD1/ADRA2B/PRKCB/FGA/SYP	9
IL6/ABAT/ADRA2B/PRKCB/FGA	5
MAP2K1/ALOX15/ATF3/SERPINF2/BMPER/C1QL4/FGA	7
MAP2K1/THY1/PLXNC1/PTPRD/FGA/TRPC5/SEMA6D	7
MAP2K1/PLXNC1/PTPRD/FGA/TRPC5	5
PPARGC1A/SLC8A1	2
SRD5A1/RNF180	2
SOX4/ABAT/GLUD1/FGA	4
KHK/SOX4/PPARGC1A/EGR1/SLC8A1/PRKCB	6
IL6/SERPINF2/CRP	3
MAP2K1/THY1/SYT2/PLXNC1/RND1/PTPRD/FGA/TRPC5/SEMA6D	9
PDE2A/PDE11A	2
MYC/EGR1	2
MYC/EGR1	2
EN1/DACH1/NR4A3/FOS	4
IL6/SERPINF2/PPARGC1A/NR4A3	4
MAP2K1/EN1/PPARGC1A/ABAT	4
MAP2K1/THY1/SYT2/PACSIN1/PLXNC1/PTPRD/SDK1/TRPC5/SEMA6D	9
PPARGC1A/EGR1	2
ABAT/ADRA2B	2
IL6/EGR1	2
PPARGC1A/NR4A3	2
MAP2K1/EHD3	2

SOX6/ATF3/PPARGC1A/VGLL2/EGR1/FOS/SLC8A1/PIM1	8
THY1/CD177/ITGA9/ANGPTL3	4
DUOX2/ABAT/SRD5A1/RNF180	4
IL6/MMP11/C1QL4	3
SERPINF2/ABAT/FGA	3
PDE2A/SLC8A1/SRD5A1	3
PPARGC1A/EGR1	2
PPARGC1A/SRD5A1	2
IL6/EGR1	2
IL6/EGR1	2
PPARGC1A/SRD5A1	2
IL6/DUOX2/SLC31A1/PPARGC1A/EGR1/NR4A3	6
SERPINF2/ABAT/FGA	3
PPARGC1A/SLC8A1	2
PPARGC1A/SLC8A1	2
MAP2K1/EN1/PPARGC1A/ABAT	4
MYC/EGR1/DCUN1D3	3
SYT2/PACSIN1/SYP	3
SYT2/PACSIN1/SYP	3
IL6/THY1/ALOX15/SERPINF2/NR4A3/FGA	6
MAP2K1/DUOX2/SERPINF2/ABAT/FGA	5
CHEK1/ATF3/PDE2A/SLC38A2/FOS/PIM1/SRD5A1	7
PPARGC1A/ABAT/SDK1/SLC8A1	4
SERPINF2/ABAT/FGA	3
PPARGC1A/EHD3/ABAT/NR4A3/SLC8A1/ADRA2B	6
FOS/SLC8A1	2
MAP2K1/THY1/PLXNC1/TRPC5/SEMA6D	5
CHEK1/SOX4/PPARGC1A	3
IL6/CRP	2
CD177/CRP	2
SATB1/NR4A3	2
IL6/SOX4/EGR1/SATB1/NFAM1/KLF6/ATP11C	7
THY1/ALOX15/FGA	3
SYT2/ABAT/PRIMA1/PRKCB/SRD5A1/SYP/RNF180	7
SYT2/ABAT/ADRA2B	3
ABAT/SDK1	2
SYT2/ABAT/ADRA2B	3
ABAT/PRIMA1	2
MYC/EGR1	2
KHK/ALOX15/SYT2/PPARGC1A/ABAT/FOS/FGA	7
KHK/SOX4/PPARGC1A/EGR1/SLC8A1	5
THY1/NFAM1/PRKCB	3
CHEK1/MAP2K1/PRKCB/TRPC5	4
IL6/ADAMTSL2/SERPINF2/OLFML2A/MMP11/ITGA9/FGA	7
IL6/MAP2K1/SOX4/SRD5A1	4
MAP2K1/SYT2/PACSIN1/PLXNC1/PTPRD/TRPC5	6
CD177/CRP	2
MAP2K1/SYT2/PACSIN1/PLXNC1/PTPRD/ADRA2B/TRPC5	7
KHK/SOX4/PPARGC1A/EGR1/SLC8A1	5
PPARGC1A/EGR1/CYP2C18/SRD5A1	4
MAP2K1/THY1/PLXNC1/SLITRK4/DCLK1/NR4A3/TRPC5/SEMA6D	8
IL6/CRP/ANGPTL3	3
ATF3/EGR1/FOS	3
HOXD13/SRD5A1	2
MYC/EGR1	2
SOX4/ABAT/GLUD1/FGA	4
SYT2/PACSIN1/SYP	3
IL6/MAP2K1/SYT2/PACSIN1/PLXNC1/PTPRD/ADRA2B/TRPC5	8

KHK/SOX4/PPARGC1A/EGR1/SLC8A1	5
ANGPT4/MYC/PPARGC1A/EGR1/SLC8A1	5
PPARGC1A/DCLK1/MFSD2A/SLC38A2/NR4A3/SLC8A1/SRD5A1	7
CHEK1/MAP2K1/PRKCB/TRPC5	4
PPARGC1A/SRD5A1	2
SRD5A1/RNF180	2
NFAM1/PRKCB	2
MAP2K1/TAPT1/SYT2/PACSIN1/PLXNC1/PTPRD/TRPC5	7
CHEK1/PDE2A/SLC38A2/FOS/SLC8A1	5
SOX4/ABAT/GLUD1	3
SLC8A1/ADRA2B	2
SERPINF2/FGA	2
PDE2A/PDE11A	2
DUOX2/CD177/CRP	3
CHEK1/SOX4/PPARGC1A	3
SOX6/ATF3/VGLL2/EGR1/FOS/SLC8A1/PIM1	7
MAP2K1/ALOX15/SERPINF2/BMPER/FGA	5
SYT2/CD177/PRKCB/FGA/SYP	5
ANGPT4/MYC/PPARGC1A/EGR1/SLC8A1	5
PPARGC1A/EGR1	2
NR4A3/MCL1	2
SLC38A4/SLC38A2/SLC4A4/SLC8A1/SGK1	5
SLC8A1/ADRA2B/FGA	3
SERPINF2/ABAT/FGA	3
FOS/SLC8A1	2
NR4A3/MCL1	2
PPARGC1A/EGR1	2
ABAT/ADRA2B	2
SYT2/ABAT/ADRA2B	3
IL6/PPARGC1A/MMP11/NR4A3/C1QL4	5
IL6/THY1/ALOX15/SERPINF2/ABAT/NR4A3/FGA	7
IL6/SOX4/ABAT/GLUD1/ADRA2B/FGA	6
PPARGC1A/SRD5A1	2
PPARGC1A/SRD5A1	2
SERPINF2/FGA	2
NFAM1/ATP11C	2
SERPINF2/ABAT/FGA	3
CHEK1/PDE2A/SLC38A2	3
EN1/PPARGC1A/EGR1/NR4A3/FOS/MCL1	6
SERPINF2/ABAT/FGA	3
EN1/FREM2/SOX4/HOXD13	4
EN1/FREM2/SOX4/HOXD13	4
ALOX15/SYT2/FOS/FGA	4
DUOX2/SERPINF2/ABAT/FGA	4
PACSIN1/PTPRD/SDK1/TRPC5	4
NFAM1/PRKCB	2
DCLK1/PKIA	2
MFSD2A/NR4A3/SRD5A1	3
IL6/SOX6/MAP2K1	3
ANGPT4/BMPER/EGR1/PRKCB/ANGPTL3	5
IL6/PPARGC1A/NR4A3/FOS/SLC8A1	5
DUOX2/PPARGC1A/EGR1/CYP2C18/SRD5A1	5
MAP2K1/EN1/PPARGC1A/ABAT	4
PPARGC1A/NR4A3	2
MYC/DACH1/C1QL4	3
ALOX15/SYT2/FOS	3
PACSIN1/PTPRD/DCLK1/SDK1/TRPC5	5
IL6/SOX4/ABAT/GLUD1/ADRA2B/FGA	6

ABAT/PRIMA1/SRD5A1/RNF180	4
ANGPT4/MYC/PPARGC1A/EGR1/SLC8A1	5
PPARGC1A/NR4A3/SEMA6D	3
MYC/DACH1/C1QL4	3
SERPINF2/ABAT/FGA	3
SYT2/HOXD13/DCLK1/TRPC5/SEMA6D	5
FOS/SRD5A1	2
MAP2K1/PLXNC1/TRPC5	3
IL6/ADAMTSL2/SERPINF2/OLFML2A/MMP11/ITGA9/FGA	7
IL6/ABAT/EGR1/NR4A3/SLC8A1/SRD5A1	6
IL6/SERPINF2/PPARGC1A/NR4A3/PIM1	5
MYC/SOX4/HOXD13/BMPER/EGR1/SRD5A1	6
IL6/THY1/TAPT1/CRP/SLC8A1/ANGPTL3	6
ABAT/ADRA2B	2
MAP2K1/THY1	2
MYC/CHEK1/PDE2A/EGR1/EFHD1/SLC38A2	6
MYC/CHEK1/PDE2A/EGR1/EFHD1/SLC38A2	6
IL6/PPARGC1A/FGA	3
ATF3/VGLL2/EGR1/FOS	4
SYT2/CD177/PRKCB/SYP	4
ANGPT4/THY1/DACH1/MCC/PPARGC1A/SEMA6D	6
IL6/PPARGC1A/NFAM1/FOS/SGK1/PIM1/PRKCB	7
PDE2A/PDE11A	2
SOX6/TAPT1	2
SOX4/PPARGC1A	2
ABAT/NR4A3	2
SYT2/ABAT/ADRA2B	3
SERPINF2/ABAT/FGA	3
NR4A3/SRD5A1	2
NR4A3/SRD5A1	2
PPARGC1A/NR4A3	2
PPARGC1A/NR4A3/SEMA6D	3
SOX6/ADAMTSL2/PPARGC1A/PDE2A/FOS	5
THY1/PPARGC1A/EHD3/DPP6/KCNS2/SLC8A1	6
IL6/SOX4/ABAT/GLUD1/FGA	5
PPARGC1A/SLC8A1	2
MFSD2A/SLC38A2/NR4A3/SLC8A1/SRD5A1	5
SYT2/DCLK1/TRPC5/SEMA6D	4
EGR1/FOS/MCL1	3
IL6/SERPINF2/PPARGC1A/NR4A3	4
ATF3/VGLL2/EGR1/FOS	4
PRKCB/CISH	2
MAP2K1/ADAMTSL2	2
PPARGC1A/SRD5A1	2
CD177/CRP	2
EN1/PPARGC1A/EGR1/NR4A3/FOS/MCL1	6
SOX6/ADAMTSL2/PPARGC1A/PDE2A/FOS	5
SYT2/ABAT/ADRA2B	3
ANGPT4/THY1/DACH1/MCC/PPARGC1A/SEMA6D	6
EHD3/ABAT/SLC8A1/ADRA2B	4
IL6/SERPINF2/PPARGC1A/NR4A3	4
SLC31A1/EGR1/SRD5A1	3
IL6/DUOX2/PPARGC1A/NR4A3/FOS/SLC8A1/MCL1	7
PPARGC1A/SEMA6D	2
PPARGC1A/NR4A3	2
MFSD2A/SLC38A2/NR4A3/SRD5A1	4
THY1/TRPC5/SEMA6D	3
CHEK1/PRKCB	2

PPARGC1A/EGR1	2
PRKCB/CISH	2
IL6/PPARGC1A	2
ANGPT4/MYC/PPARGC1A/ABAT/EGR1/SLC8A1	6
SOX4/PDE2A/ANGPTL3	3
SYT2/ABAT/NR4A3	3
HOXD13/SRD5A1	2
SOX4/PPARGC1A/A1CF/PIM1	4
SOX4/ABAT/GLUD1/ADRA2B/FGA	5
EN1/FREM2/SOX4/HOXD13	4
EN1/FREM2/SOX4/HOXD13	4
SYT2/ABAT/ADRA2B	3
ATF3/SLC38A2/FOS/PIM1/SRD5A1	5
IL6/SOX4/ABAT/GLUD1/FGA	5
SYT2/ABAT	2
SYT2/ABAT	2
PLXNC1/SEMA6D	2
MYC/EGR1	2
SERPINF2/ABAT/SGK1/ADRA2B	4
PPARGC1A/NR4A3/SEMA6D	3
KHK/EGR1/PRKCB/SRD5A1/CISH	5
ANGPT4/MYC/PPARGC1A/ABAT/EGR1/SLC8A1	6
PPARGC1A/EGR1	2
MYC/NFAM1/NR4A3/FOS/ATP11C/PIM1/PRKCB	7
ATF3/SLC38A2	2
SOX4/PPARGC1A	2
SYT2/ABAT/SLC38A2	3
ABAT/MFSD2A/SLC38A4/SLC22A11/SLC38A2/SLC4A4/ATP11C	7
MFSD2A/NR4A3/SRD5A1	3
THY1/PPARGC1A/EHD3/DPP6/KCNS2/SLC8A1/KCNK17	7
ALOX15/SYT2/PPARGC1A/FOS	4
IL6/SERPINF2	2
CDH10/SERPINF2	2
SYT2/DCLK1/DCUN1D3/SGK1/CISH/TRPC5/SEMA6D	7
ATF3/PPARGC1A/SLC38A2/SRD5A1	4
ABAT/SLC8A1/ADRA2B	3
ALOX15/PPARGC1A/NR4A3/MMAA/CYP2C18/ANGPTL3	6
IL6/PPARGC1A/FGA	3
IL6/SOX4	2
ANGPT4/THY1/DACH1/MCC/PPARGC1A/SEMA6D	6
SYT2/PRKCB/SYP	3
SYT2/PACSIN1/PRKCB/SYP	4
IL6/CRP	2
EGR1/SRD5A1	2
IL6/PPARGC1A	2
ATF3/SLC38A2	2
IL6/SOX4/ABAT/GLUD1/FGA	5
EGR1/SRD5A1	2
NR4A3/SEMA6D	2
HOXD13/SRD5A1	2
IL6/SERPINF2/MMP11	3
ANGPT4/MYC/PPARGC1A/ABAT/EGR1/SLC8A1	6
ABAT/ADRA2B	2
ANGPT4/THY1/DACH1/MCC/PPARGC1A/SEMA6D	6
IL6/SERPINF2	2
THY1/SDK1	2
IL6/SOX4/ABAT/GLUD1/CD177/FGA	6
SYT2/PRKCB/SYP	3

ANGPT4/BMPER/PRKCB/ANGPTL3	4
PPARGC1A/EGR1	2
PPARGC1A/FOS	2
IL6/SOX4	2
IL6/PPARGC1A	2
ABAT/MFSD2A/SRD5A1/RNF180	4
IL6/THY1/ALOX15/SERPINF2/NR4A3/FGA	6
THY1/ALOX15/FGA	3
CHEK1/ATF3/EGR1	3
DCLK1/TRPC5/SEMA6D	3
IL6/PPARGC1A/NR4A3/FOS/MCL1	5
SYT2/PACSIN1/PRKCB/SYP	4
ALOX15/CYP2C18	2
PDE2A/PDE11A	2
SOX4/ABAT/GLUD1/FGA	4
SYT2/ABAT	2
EN1/PPARGC1A/NR4A3/MCL1	4
ABAT/SLC38A2/SGK1	3
SOX6/ATF3/VGLL2/EGR1/FOS/PIM1	6
EN1/FREM2/HOXD13	3
EN1/FREM2/HOXD13	3
RAD54B/PRDM9	2
SERPINF2/FGA	2
IL6/SERPINF2	2
CHEK1/PPARGC1A	2
IL6/NFAM1/KLF6/ATP11C/PRKCB	5
MYC/SLC31A1/SYT2/ABAT	4
IL6/NR4A3	2
ABAT/RNF180	2
ABAT/RNF180	2
SERPINF2/MMP11	2
RAD54B/PRDM9	2
CHEK1/ATF3/EGR1	3
SYT2/DCUN1D3/SGK1/CISH/TRPC5/SEMA6D	6
ABAT/SDK1	2
ALOX15/SYT2/PPARGC1A/FOS	4
THY1/EHD3/PDE2A/SLC8A1/TRPC5	5
IL6/SOX6/MAP2K1/SOX4	4
IL6/ANGPT4/BMPER/EGR1/PRKCB/ANGPTL3	6
MYC/EGR1	2
NFAM1/KLF6/ATP11C	3
IL6/MMP11/C1QL4	3
ABAT/MMAA/LDHD	3
CHEK1/MAP2K1/PPARGC1A/ABAT/FOS	5
IL6/SOX4/ABAT/GLUD1/CD177/FGA	6
MYC/THY1/ATF3/PPARGC1A/C1QL4/PKIA	6
IL6/MYC/CHEK1/DACH1/PPARGC1A/PRDM9	6
ADRA2B/FGA	2
IL6/EGR1	2
SOX4/PDE2A	2
ATF3/FGA	2
FCAR/CD177/SIGLEC14	3
DUOX2/THY1/PACSIN1/MCC/FRMD4B/CD177/ANGPTL3	7
THY1/PACSIN1/PRKCB/TRPC5/SYP	5
THY1/LIFR/SLC22A11/FCGR3A/CD69/FGA	6
SYT2/FCAR/NFAM1/CD177/SIGLEC14	5
PACSIN1/PRKCB/SYP	3
SLC22A11/SLC4A4	2

PDE2A/PDE11A	2
MFSD2A/SLC38A4/SLC22A11/SLC38A2/SLC4A4/SLC8A1	6
GRHPR/SRD5A1	2
MYC/SOX6/EN1/DACH1/ATF3/SATB1	6
CHEK1/PRKCB	2
PDE2A/PDE11A	2
MYC/SOX4/ATF3/HOXD13/EGR1/KLF6/NR4A3/FOS	8
FCAR/FCGR3A	2
GRHPR/ABAT/GLUD1/PANK1/LDHD/SRD5A1	6
MFSD2A/SLC38A4/SLC22A11/SLC38A2/SLC4A4	5
PDE2A/PDE11A	2
MFSD2A/SLC38A4/SLC38A2/SLC4A4	4
PDE2A/PDE11A	2
EGR1/NR4A3	2
SYT2/TRPC5/SYP	3
DCUN1D3/RNF180	2
PPARGC1A/NR4A3/PRKCB	3
PDE2A/PDE11A	2
DCUN1D3/RNF180	2
SLC31A1/PDE2A/KCNS2/SLC4A4/SLC8A1/KCNK17/TRPC5	7
MFSD2A/SLC38A4/SLC22A11/SLC38A2/SLC4A4/SLC8A1	6
MAP2K1/SGK1	2
PPARGC1A/PRKCB	2
SYNPO2/TRPC5	2
SGK1/PRKCB	2
PPARGC1A/EGR1	2
DPP6/SGK1/PRKCB	3
AKAP3/PKIA	2
RND1/EHD3/PDE2A/GLUD1/PDE11A/MMAA	6
RND1/EHD3/PDE2A/GLUD1/PDE11A/MMAA	6
NDST3/SULT4A1	2
DPP6/SGK1	2
GRHPR/SRD5A1	2
ANGPT4/MAP2K1/CISH/PKIA	4
MYC/FOS	2

Table S6. KEGG annotation of the target genes

ID	Description	GeneRatio	BgRatio
hsa04630	JAK-STAT signaling pathway	6/58	162/8011
hsa05020	Prion diseases	3/58	35/8011
hsa01521	EGFR tyrosine kinase inhibitor resistance	4/58	79/8011
hsa04371	Apelin signaling pathway	5/58	137/8011
hsa04012	ErbB signaling pathway	4/58	85/8011
hsa05166	Human T-cell leukemia virus 1 infection	6/58	219/8011
hsa04933	AGE-RAGE signaling pathway in diabetic complications	4/58	100/8011
hsa05161	Hepatitis B	5/58	162/8011
hsa04928	Parathyroid hormone synthesis, secretion and action	4/58	106/8011
hsa04066	HIF-1 signaling pathway	4/58	109/8011
hsa04726	Serotonergic synapse	4/58	115/8011

pvalue	p.adjust	qvalue	geneID
0.001058035	0.132181031	0.110491883	IL6/MYC/LIFR/MCL1/PIM1/CISH
0.001999912	0.132181031	0.110491883	IL6/MAP2K1/EGR1
0.002482518	0.132181031	0.110491883	IL6/MAP2K1/NRG2/PRKCB
0.003012289	0.132181031	0.110491883	MAP2K1/PPARGC1A/EGR1/PLIN1/SLC8A1
0.003239731	0.132181031	0.110491883	MYC/MAP2K1/NRG2/PRKCB
0.004821175	0.156656443	0.130951206	IL6/MYC/CHEK1/MAP2K1/EGR1/FOS
0.005790819	0.156656443	0.130951206	IL6/EGR1/PIM1/PRKCB
0.00614339	0.156656443	0.130951206	IL6/MYC/MAP2K1/FOS/PRKCB
0.007105119	0.159746052	0.133533852	MAP2K1/EGR1/FOS/PRKCB
0.007830689	0.159746052	0.133533852	IL6/ANGPT4/MAP2K1/PRKCB
0.009424936	0.174789722	0.146109056	MAP2K1/ALOX15/CYP2C18/PRKCB

Count
6
3
4
5
4
6
4
5
4
4
4

Table S7.ValidatIon of the proportional hazard assumption of cox regression model

	chisq	df	p
age	2.003598	1	0.156926
stage	0.275101	1	0.599931
T	0.512572	1	0.474028
M	4.825195	1	0.028047
N	11.06468	1	0.00088
riskScore	1.209221	1	0.271486
GLOBAL	14.50385	6	0.124487
